# Comparative Genomic Analysis Reveals a Possible Novel Non-Tuberculous Mycobacterium Species with High Pathogenic Potential

**DOI:** 10.1371/journal.pone.0150413

**Published:** 2016-04-01

**Authors:** Siew Woh Choo, Avirup Dutta, Guat Jah Wong, Wei Yee Wee, Mia Yang Ang, Cheuk Chuen Siow

**Affiliations:** 1 Department of Oral Biology and Biomedical Sciences, Faculty of Dentistry, University of Malaya, 50603, Kuala Lumpur, Malaysia; 2 Genome Informatics Research Laboratory, High Impact Research (HIR) Building, University of Malaya, 50603, Kuala Lumpur, Malaysia; Cambridge University, UNITED KINGDOM

## Abstract

Mycobacteria have been reported to cause a wide range of human diseases. We present the first whole-genome study of a Non-Tuberculous *Mycobacterium*, *Mycobacterium* sp. UM_CSW (referred to hereafter as UM_CSW), isolated from a patient diagnosed with bronchiectasis. Our data suggest that this clinical isolate is likely a novel mycobacterial species, supported by clear evidence from molecular phylogenetic, comparative genomic, ANI and AAI analyses. UM_CSW is closely related to the *Mycobacterium avium* complex. While it has characteristic features of an environmental bacterium, it also shows a high pathogenic potential with the presence of a wide variety of putative genes related to bacterial virulence and shares very similar pathogenomic profiles with the known pathogenic mycobacterial species. Thus, we conclude that this possible novel *Mycobacterium* species should be tightly monitored for its possible causative role in human infections.

## Introduction

Mycobacteria show multiple modes of existence in nature, ranging from soil-dwelling saprophytes to animal and human pathogens [1]. The species associated with human infections have been increasing, particularly among the non-tuberculous mycobacteria (NTM) [2]. Although NTM species cause mostly opportunistic infections from an environmental source [3], potentially fatal infections [4] and human-to-human transmission have also been reported [5].

In our routine diagnostic service, a NTM was isolated from the sputum sample of a patient with a long history of bronchiectasis. As the results of our gene-based phylogenetic analysis suggested that it might be a novel species, we performed whole-genome sequencing to obtain more information on its taxonomic position, biology and pathogenicity. Here, we report the supporting evidence for its novelty and some interesting observations from our genomic analysis. The UM_CSW genome assembly has been deposited at GenBank under the accession number AUWQ00000000.

## Materials and Methods

### Library preparation and whole-genome sequencing

For DNA library preparation, the DNA samples were fragmented using Covaris S2 for 120 seconds at temperature of 5.5–6.0 degree Celsius. Agilent BioAnalyzer 2100 was used to examine the quantity and quality of the fragmented DNA. The sample was size selected using Invitrogen 2% agarose E-gels. Only the fragments having the adapter molecules at both ends underwent 10 cycles of PCR for the purpose of library construction. The validation of constructed genomic library was performed using Agilent BioAnalyzer 2100. The pool of 8pM was loaded onto 1 lane of Illumina HiSeq 2000 flow cell v3 for 100bp paired-end sequencing according to manufacturer’s recommended protocols.

### Read preprocessing, genome assembly and annotation

Exact duplicates and exact reverse complement duplicates reads resulted from shot-gun sequencing were filtered by using standalone PRINSEQ lite version 0.20 [6]. Sequencing of UM_CSW at approximately 800x coverage (assuming genome size of 6Mbp) enabled us to prepare reads for assembly at higher quality by further filtering on reads with ambiguous nucleotides. The raw Illumina reads were trimmed at a threshold of 0.01 (Phred score of 20) and the sequences obtained were *de novo* assembled using CLC Genomic Workbench version 5.1 (CLC bio, Denmark). The assembly of the reads was carried out at length faction of 0.7 and similarity fraction of 0.9, accepting minimal size of contig at 500bp.

The assembled genome was annotated using the RAST pipeline, which is a fully automated annotation engine for complete or draft archaeal and bacterial genomes [7]. Of the 6,076 RAST-predicted protein-coding genes, 1,944 (32%) genes were successfully assigned to 376 subsystems/functional categories, whereas the remaining genes were not assigned to any subsystem. The presence of plasmid replicons was predicted by PlasmidFinder-1.2 [8]. Clusters of regularly interspaced repeats (CRISPR) were identified by CRISPR Finder [9].

### Genome size estimation

To estimate the genome size of UM_CSW, k-mer was counted using the tool called Preqc from the SGA assembly program [10]. The tool counts and plots 31-mer histogram from 20,000 reads and estimates the genome size by identifying the peak of the Poisson distribution. The estimation is based on the principle as follows, where the mean number of times a unique genomic k-mer appears in the reads, λ_k is as follows:

λ_k=(n(l−k+1))/G

Where n is the number of reads, l is the read length and G being the genome size.

### Phylogenetic study

Three bacterial classification markers *hsp65*, *rpoB*, and 16S rRNA were selected for the single gene-based phylogenetic studies. To clarify the phylogenetic relationship between UM_CSW and members of the *M*. *avium* complex using whole-genome data, the core genome of *M*. *intracellulare* ATCC13950, *M*. *indicus pranii* MTCC9506, *M*. *colombiense* CECT3035, *M*. *avium* K10 along with *M*. *tuberculosis* CCDC5079, *M*. *afficanum* GM041182 and *M*. *marinum* M was identified using Panseq 2.0 [11]. *M*. *tuberculosis* CCDC5079, *M*. *africanum* GM041182, and *M*. *marinum* M were used as outgroups. The SNPs within each core genome were identified, extracted and concatenated into one large sequence.

Model testing was performed on each set of aligned sequence prior the phylogenetic reconstructions. Using the proposed substitution models based on corrected Akaike information criterion, maximum likelihood trees were inferred with 1,000 boostrapping replication. All the likelihood estimations were done in MEGA version 5.2 [12].

### Amino Acid Identity (AAI) Analysis

The average amino acid identity (AAI) was calculated using the method described by Konstantinidis & Tiedje [13]. Mycobacterial genomes from GenBank were downloaded and annotated using RAST. The protein-coding sequences of the UM_CSW genome was used as the reference for comparison against other mycobacterial genomes using the BLAST search to determine the conserved genes. The cut-off for the BLAST search was set at ≥30% sequence identity and ≥70% sequence coverage at the amino acid level. The average of the amino acid identity of all conserved genes between a pair of genomes was computed to measure the genetic relatedness between them.

### Whole-genome Average Nucleotide Identity (ANI) analysis

The ANI has been reported to be a reliable alternative to the use of classical housekeeping genes for the evaluation of bacterial genetic relatedness [13]. To categorize genomes into the same species, the genomes must show an ANI value > 95% which is equivalent to the 70% DNA-DNA Hybridization (DDH) level [14]. The ANI is calculated based on the average percentage sequence similarity between all conserved genes in a pair of different genomes. The ANI calculation were performed using JSpecies [15] using the Blast parameters of 150 as dropoff value for gapped alignment, -1 as the penalty for a nucleotide mismatch, F as the filter query sequence, 1e-15 as the expectation value (E) and 2 as the number of processors to use; ANI calculation parameters as, percentage of identity > = 30, percentage of alignment > = 70 and length as 1020.

### Genome Island prediction

Genomic islands in the genome of UM_CSW were predicted using IslandViewer, which is based on unique features in codon usage, dinucleotide sequence composition and presence of mobile element genes [16]. IslandViewer integrates few other genomic island prediction methods to predict the genomic islands. It includes the sequence composition base approaches SIGI-HMM [17] and IslandPath-DIMOB [18] that were shown to have highest specificity of around 86% to 92% and an accuracy of about 86%, as well as the comparative genomics approach IslandPick [19].

### Estimation of pathogenicity of the mycobacterial genomes

PathogenFinder 1.1, a web-server for the prediction of bacterial pathogenicity [20] was used to estimate the pathogenicity of UM_CSW along with 19 other mycobacterial genomes. The genome sequences of type strains of 19 mycobacterial species were downloaded from NCBI in the fasta format for the analysis. Each genome was then uploaded to the PathogenFinder 1.1 web-server individually, selecting Automatic Model Section as Phylum and Assembled Genome/Contigs* for the Sequencing Platform as the parameters for the estimation.

### Virulence gene and prophage prediction

The protein sequences predicted by RAST were BLAST searched against the virulence factor database (VFDB) [21]. Using our in-house Perl scripts, we selected the genes that were orthologous to virulence genes with at least 60% identity and at least 60% sequence coverage in query and subject.

Prophages in the genome of UM_CSW was identified using PHAST (PhAge Search Tool) web server [22]. Putative protein sequences in the genome of UM_CSW were BLAST searched against two prophage sequence databases to identify phage-related proteins. The two databases are the NCBI phage database and the prophage database that contains 159 prophage regions and 9,061 proteins that are not found in the NCBI phage database.

### Comparative Pathogenomics Analysis

All *Mycobacterium* genomes used in this analysis were downloaded from NCBI and re-annotated using the RAST pipeline. For each genome, all RAST-predicted protein-coding gene sequences were searched against the VFDB database [21] through the use of BLASTP [23]. The genes that were orthologous to virulence genes must have at least 60% identity and at least 60% sequence coverage in query and subject. The output results were processed and passed to our in-house R scripts for clustering and visualization using a heat map with dendrograms showing clustered mycobacterial species with closely related sets of virulence genes, sorted according to similarities across the strains and genes. This graphical representation is useful and to allow for the spontaneous comparison of pathogenomic profiles between genomes.

## Results

### General Genome Overview

**Fig 1:**
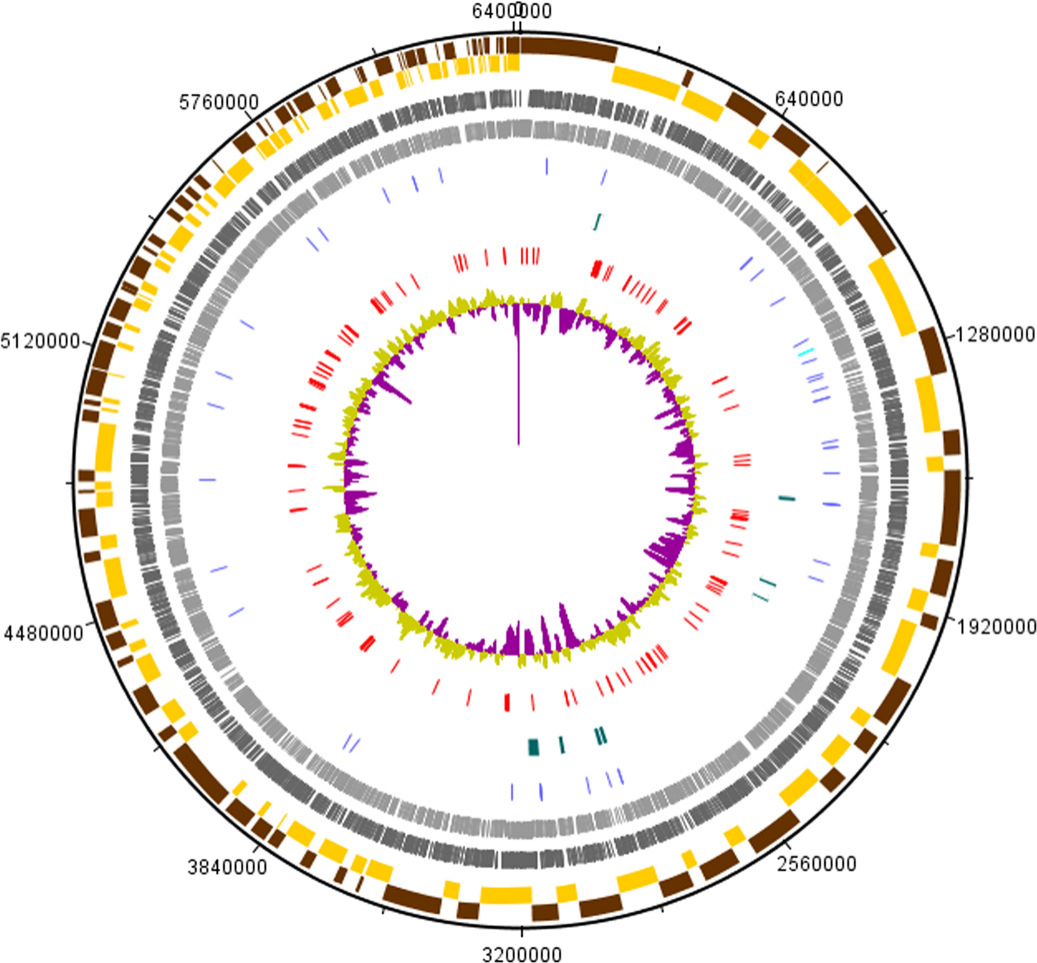
Circular representation of UM_CSW genome. The indication for each feature from the outermost layer: (a) The alternating brown and yellow coloured bars represent the odd and even numbered contigs respectively; (b) The dark and light grey coloured bars represent the protein-coding genes in the forward and the reverse strands respectively; (c) rRNAs (light blue) and tRNAs (dark blue); (d) genomic islands (green); (e) virulence genes (red); (f) GC plot (purple and light green).

We sequenced the genome of UM_CSW using the Illumina HiSeq technology platform at high sequencing depth (approximately 737x, assuming genome size of 6.51Mbp). This yielded 48,832,804 raw reads. The *de novo* assembly of the pre-processed 48,173,293 reads resulted in 186 contigs with a total genomic size of 6.4Mbp. With the contig sizes ranging from 521 bp to 231,836 bp, the assembly had N75, N50 and N25 values of 39,170 bp, 66,558 bp and 123,976 bp respectively. This assembly covered 98.3% of the predicted genome size of UM_CSW (6.51Mbp), suggesting that the assembled genome is close to complete. As with other known mycobacteria, the genome of UM_CSW has a high G+C composition of 67.8%. It contains 6,076 putative coding sequences (CDSs), 47 tRNAs and a single 16S-5S-23S ribosomal RNA operon (S1 Table). No plasmid replicons, prophages or clusters of regularly interspaced repeats (CRISPR) were found in the genome. Circular representation of UM_CSW genome is shown in Fig 1.

### Molecular and Phylogenetic Analyses

**Fig 2:**
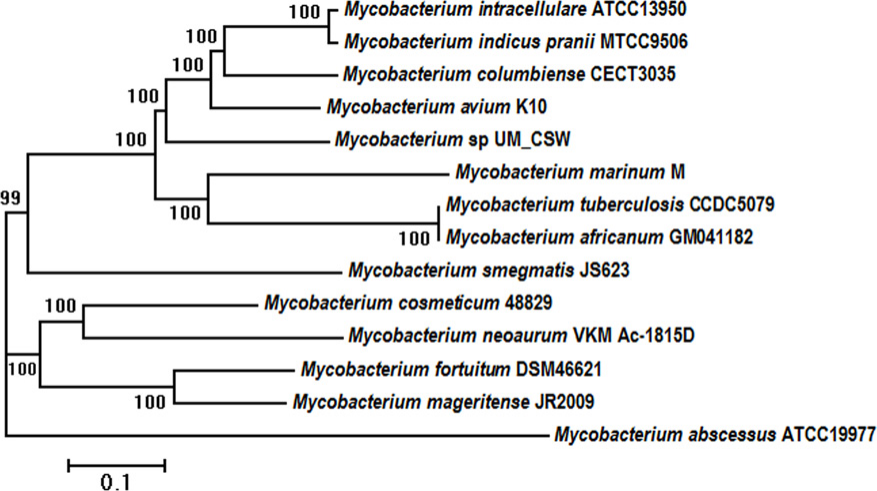
Phylogenetic relationship of UM_CSW with other mycobacterial species. The phylogenetic tree was generated using core genome SNPs and the maximum likelihood method. Bootstrap numbers were generated in 1,000 runs. Nodes with bootstrap support values are indicated.

The taxonomic position of UM_CSW was initially examined by phylogenetic inferences of three global bacterial identification markers, the 16S rRNA, *hsp65* and *rpoB*. Using type strains of recognized *Mycobacterium* species, the 16S rRNA-based tree showed the closest relatives of UM_CSW to be *M*. *interjectum* (99.5% sequence similarity; 100% sequence coverage) and *M*. *saskatchewanense* (99% sequence similarity; 100% sequence coverage) (S1 Fig), both of which are slow-growing schotochromogens. In the *hsp65*-based tree, however, the closest neighbors were two non-pigmented rapid growers, *M*. *cosmeticum* (95.7% sequence similarity) and *M*. *canariasense* (94.9% sequence similarity) (S2 Fig). Further incongruity was seen in the *rpoB* tree where another two slow-growing schotochomogens, *M*. *arosiense* and *M*. *colombiense* were seen as the closest relatives with 96.5% and 96.7% sequence similarities respectively (S3 Fig). The other species grouped along with UM_CSW in the *rpoB* tree were *M*. *intracellulare*, *M*. *chimaera* and *M*. *timonense* which, along with *M*. *arosiense* and *M*. *colombiense*, form a part of the *M*. *avium* complex (MAC) [24]. Interestingly, neither the 16S- nor the *hsp65*-based tree grouped UM_CSW with any of the members of the *M*. *avium* complex. Moreover, the closest relatives, *M*. *interjectum* and *M*. *saskatchewanense* in the *16S* gene-based tree, and *M*. *cosmeticum* and *M*. *canariasense* in the *hsp65*-based tree, had more distant relationships with UM_CSW in the *rpoB*-based tree, with sequence similarities of 95.7%, 95.4%, 91.6%, and 90.3% respectively. The close relationship between UM_CSW and the members of the *M*. *avium* complex is also supported by a core-genome SNP-derived phylogenetic tree (Fig 2).

### ANI and AAI Analyses

In an attempt to resolve the discrepancies from the single gene based phylogenetic inferences, we subjected our isolate to ANI and AAI analyses. Using UM_CSW as the reference genome, we calculated the ANI and AAI for 31 other mycobacterial species with genome sequences available. Two genomes with an ANI >95% and AAI >96% were considered to belong to the same species, while those with ANI and AAI below these thresholds were considered separate species [14].

**Fig 3:**
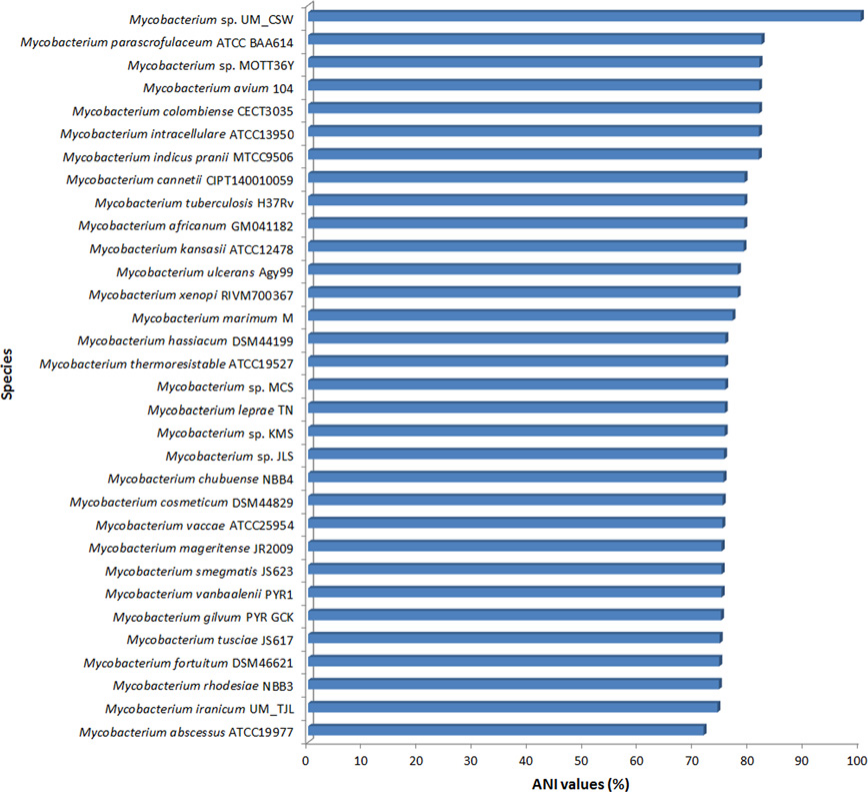
ANI analysis. The top six relatives of *Mycobacterium* sp. UM_CSW are all members of the *M*. *avium* complex.

Our analysis showed that the closest relatives of UM_CSW were all members of the *M*. *avium* complex such as *M*. *parascrofulaceum* ATCC25954, *Mycobacterium* sp. MOTT36Y, *M*. *intracellulare* ATCC13950, *M*. *colombiense* CECT3035, and *M*. *avium* 104 (Fig 3 and S4 Fig). The ANIs between UM_CSW and these species ranged between 81.6% and 82.1% (Fig 3), whereas the AAI values ranged between 81.7% and 82.2%. The ANI and AAI values between UM_CSW and *M*. *cosmeticum* which was the closest relative in the *hsp65*-based tree were only 75% and 68.2% respectively. These ANI (<95%) and AAI (<96%) values suggest that UM_CSW is genetically distinct from known members of the *M*. *avium* complex and other mycobacteria used in this analysis.

### Functional Analysis

**Fig 4:**
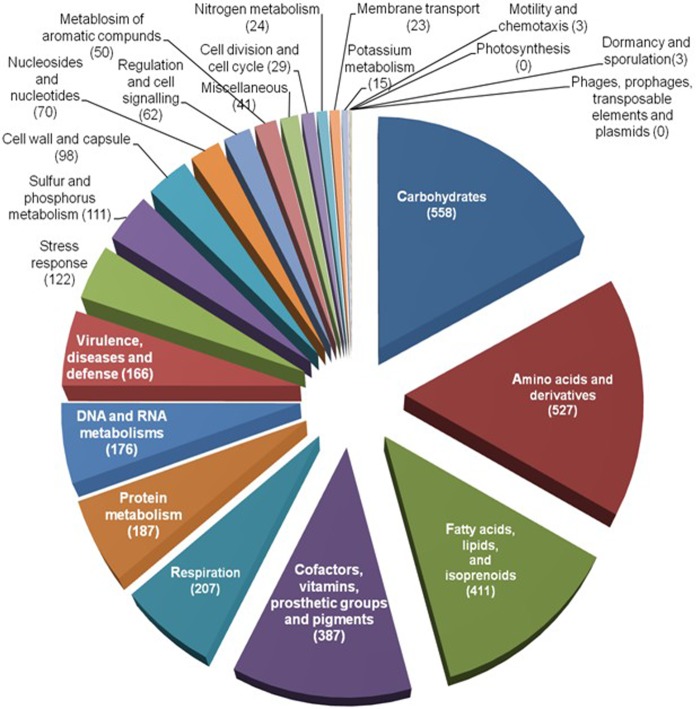
Functional classification of RAST-predicted protein-coding genes in the genome of UM_CSW.

The RAST subsystem annotation revealed that a large portion of the genome is comprised of genes related to basic structures and functions of the bacterial cell (Fig 4). In addition, there were a large number (122) of stress response genes that may indicate the ability of UM_CSW to adapt to different environments. Interestingly, no genes were involved in phages, prophages, transposable elements and plasmids, suggesting that UM_CSW may have a strong defence system to prevent the invasion of foreign DNA into its genome. Among the 166 genes involved in virulence, disease and defence, the majority (78%) were involved in invasion and intracellular resistance, while the rest (22%) were involved in the resistance to antibiotics and toxic compounds. In this latter category, there were genes involved in copper homeostasis (7 genes), resistance to beta-lactam antibiotics (7 genes), cobalt-zinc-cadmium resistance (5 genes) and resistance to fluoroquinolones (2 genes), as well as two genes encoding mercuric reductase. However, it was arsenic resistance genes which led this category with 13 genes. The presence of genes responsible for metal and arsenic resistance suggests that UM_CSW might have an environmental origin.

### Arsenic Resistance Potential

Studies have shown that arsenic may occur in four oxidation states, of which, arsenate [As(V)] and arsenite [As(III)] are readily soluble in water and abundantly present in nature. As(V), being an analogue of phosphate, can enter bacterial cells via phosphate transport systems (Pst and Pit) [25]. We found in the UM_CSW genome, putative *Pst* genes coding for the phosphate transport system by which arsenic may enter the cell. Microbes are known to reduce As(V) by two distinct mechanisms: the ArsC system that confers resistance by reducing As(V) and another mechanism that involves dissimilatory reduction of arsenate, as part of a respiratory pathway. The UM_CSW genome houses genes encoding arsenical resistance protein (Acr3), arsenate reductase (EC 1.20.4.1), arsenic efflux pump protein, and arsenical pump-driving ATPase (EC 3.6.3.16), which indicates that UM_CSW may have the AsrC system of arsenic resistance [25].

### Genomic Island Analysis

We predicted eight putative Genomic Islands (GIs) that are at least 10kb in size (Table 1; S2 Table). All GIs showed bias of G+C content (58%-62.8%) compared to the overall G+C content (67.8%) of the UM_CSW genome.

**Table 1:** Summary information of the GIs predicted in the genome of UM_CSW.

GIs	Genomic Length (bp)	Number of CDSs	G+C content (%)	Selected Genes
GI1	11,040	15	62.3	ThiJ/PfpI family protein
				putative transcriptional regulator, AraC family
				low molecular weight T-cell antigen TB8.4
GI2	14,745	13	61.7	Transcriptional regulator, TetR family
				putative cytochrome P450 hydroxylase
				Hydrolase
				Transcriptional regulator KorSA, GntR family
				Transfer protein traSA
GI3	10,251	10	62.8	Coenzyme F420-dependent oxidoreductase
				Transcriptional regulator, TetR family
				putative cytochrome P450 hydroxylase
				Long-chain-fatty-acid—CoA ligase (EC 6.2.1.3)
GI4	11,329	15	61.3	TesB-like acyl-CoA thioesterase 4
				Transcriptional regulator, TetR family
				PROBABLE CHOLESTEROL DEHYDROGENASE
				Transcriptional regulator, IclR family
GI5	14057	12	58	ATP-dependent DNA helicase UvrD/PcrA
				putative ATPase
GI6	12666	12	59.7	NADH oxidase (putative)
				dNTP triphosphohydrolase, broad substrate specificity, subgroup 2
				DNA methylase N-4/N-6
GI7	12727	5	59.9	Superfamily II DNA/RNA helicases, SNF2 family
				Adenine-specific DNA methylase containing a Zn-ribbon
GI8	37271	37	62.6	Integrase
				Death on curing protein, Doc toxin
				Long-chain-fatty-acid—CoA ligase (EC 6.2.1.3)
				Butyryl-CoA dehydrogenase (EC 1.3.99.2)
				Transcriptional regulator, GntR family
				L-carnitine dehydratase/bile acid-inducible protein F
				Alpha-methylacyl-CoA racemase (EC 5.1.99.4)
				MaoC family protein
				Alcohol dehydrogenase (EC 1.1.1.1)
				3-ketoacyl-CoA thiolase (EC 2.3.1.16) @ Acetyl-CoA acetyltransferase (EC 2.3.1.9)
				Enoyl-CoA hydratase (EC 4.2.1.17)
				3-ketoacyl-(acyl-carrier-protein) reductase (EC:1.-)
				putative acyl-CoA dehydrogenase (EC:1.3.99.-)
				acyl-CoA dehydrogenase domain protein
				Acyl-CoA thioesterase II (EC 3.1.2.-)
				probable oxidoreductase/Short-chain dehydrogenase
				putative cytochrome P450 hydroxylase

To identify the origin of these GIs, we performed simple BLAST searches using the sequences of the GIs against the National Center for Biotechnology Information (NCBI) NR database [26]. Interestingly, we found that the whole sequence of GI2 aligned perfectly with a sequence segment in the genomes of *M*. *sp*. MOTT36Y and *M*. *yongonense* 05–1390 (100% sequence identity; 100% sequence coverage; e-value of 0). This suggests that G12 might have originated from *M*. *sp*. MOTT36Y or *M*. *yongonense* 05–1390 and was inserted into the genome of UM_CSW through horizontal transfer. It is also possible that all these segments originated from the same source. The horizontal transfer of G12 is further supported by the presence of an integrase and tRNA genes which were found ahead of this GI. There are 13 putative genes in G12 comprising seven hypothetical proteins, several transcriptional regulator proteins and a transfer protein traSA flanking it. One of the transcriptional regulator proteins is from the KorSA, GntR family, which has been reported to be a transcriptional repressor usually binding to a conserved 17-nucleotide sequence upstream of the genes *korSA*, and *pra*, a gene reported to positively control the replication, integration, and excision of the mobile integrative genetic element of *Streptomyces* pSAM2 [27, 28]. The *traSA* gene has been reported to be essential for pSAM2 intermycelial transfer and pock formation [28]. However, in-spite of being smaller, TraSA has similarity to *Streptomyces* plasmid transfer protein and more generally to double‑stranded DNA translocases [28] which usually possess a transmembrane domain in their N‑terminal part, an FtsK/SpoIIIE domain in their central part, and a winged‑Helix‑Turn‑Helix (wHTH) fold region. Sequence analysis indicates that the transmembrane domain in the N‑terminal part might associate traSA with the membrane while the FtsK/SpoIIIE domain acts as a pump which actively transports double‑stranded DNA [29]. The GI also has PPE proteins and proteins from the TetR family which might be essential for the survival of the organism inside a host and the maintenance of pathogenicity.

GI4 is another interesting GI with 15 putative protein-coding genes, 11 of which encode hypothetical proteins. Of the remaining 4 proteins, one is from the TetR family, while the others are a TesB-like acyl-CoA thioesterase 4, an IclR family transcriptional regulator and a probable cholesterol dehydrogenase. Of the 11 hypothetical proteins, three showed strong homology with the Emopamil-binding protein (EBP) of *M*. *rhodesiae*. The EBP protein has emopamil binding domains, including the sterol acceptor site and the catalytic centre, which show Delta8-Delta7 sterol isomerase activity. Human sterol isomerase, a homologue of mouse EBP, is suggested not only to play a role in cholesterol biosynthesis, but also to affect lipoprotein internalisation. It has already been shown that the utilization of host cholesterol is essential for the persistence of *Mycobacterium* within the IFN-γ-activated macrophages [30]. Moreover, cholesterol plays a key role in the uptake of mycobacteria by macrophages and has also been reported to mediate the phagosomal association of a coat protein TACO, which prevents the degradation of mycobacteria in lysosomes [31]. Other reports suggested that cholesterol oxidase is essential for the pathogenesis conferred by *Mycobacterium*, by interfering with the TLR2-mediated signaling pathway and thus enabling intracellular growth and survival in human macrophages [32]. Thus the presence of the homolog of EBP and a probable cholesterol dehydrogenase in the same GI indicates that this region may be essential for cholesterol metabolism by the organism, which in turn, might play a role in its pathogenicity.

In GI1, we predicted a possible Stage II sporulation protein along with the presence of a putative heat shock protein 70 (partial), transcriptional regulator of AraC family, ThiJ/PfpI family protein and a response regulator receiver/ANTAR domain-containing protein. The presence of these proteins in close vicinity, points to the possibility of them being part of the stress response system of the organism. The predicted region also hosts a low molecular weight T-cell antigen TB8.4, a possible transmembrane protein or permease and a probable antibiotic biosynthesis monooxygenase.

The largest island, GI8, has a genomic length of 37,271 bp, a G+C content of 62.6% and 37 genes. This predicted region is flanked by a couple of hypothetical proteins followed by 4 tRNA genes and an integrase. One of the hypothetical proteins showed homology with a dehydratase MaoC which was preceded by another MaoC family protein. MaoC family proteins normally have an oxidoreductase activity and dehydratase MaoC has been reported to be an enoyl-CoA hydratase that is involved in linking the β-oxidation and polyhydroxyalkanoate (PHA) biosynthetic pathways [33]. The region also has the transcriptional regulator of the GntR family and a possible TetR family transcriptional regulator as well. Most interesting is the presence of Death on curing protein, Doc toxin, in this region. Doc toxin is a part of a two protein operon, the Phd-Doc toxin–antitoxin (TA) system (suicide or ‘‘addiction modules”) where Doc is the toxin and Phd (prevents-host-death) is the antitoxin. TA systems usually play an important role in cell survival during intervals of stress, by inducing a state of reversible growth arrest. TA systems have also been associated with biofilm formation, bacterial persistence during antibiotic treatment, and bacterial pathogenesis [34]. We could not find the antitoxin Phd, the counterpart of the Doc toxin in TA systems within the GI8. We found the presence of another Doc toxin which was immediately followed by a hypothetical protein showing strong homology with the antitoxin Phd in the genome; however, they were not a part of any of the predicted GIs.

The presence of these GIs suggests that UM_CSW is likely to have acquired, through horizontal gene transfer, new capabilities which are important for the survival and adaptation of this bacterium in its ecology and are potentially contributory to its virulence.

### UM_CSW Has a Pathogenic Potential

Although UM_CSW was isolated from a clinically derived sample, it could be just a colonizing bacterium. To examine its pathogenicity, we used the PathogenFinder, which is a web-server for the prediction of bacterial pathogenicity by analysing the genome, or raw reads provided by the user [20]. PathogenFinder uses an unbiased method which relies on groups of proteins, created without regard to their annotated function or known involvement in pathogenicity. The method has been tested with many groups of bacteria and had achieved an accuracy of 88.6%. In this analysis, we also compared the predicted pathogenicity of UM_CSW with 19 other well-studied mycobacterial species (comprising both pathogenic and the non-pathogenic mycobacteria).

Our results showed UM_CSW to be predicted as a human pathogen with a probability of 0.841. As anticipated, all the known pathogenic mycobacteria were classified as human pathogens with high probability of around 0.8 and above, while the known non-pathogenic mycobacteria much lower probability (S4 Table). These results further support our view that UM_CSW might be a human pathogen capable of causing disease in the source patient.

### Virulence Genes in the UM_CSW Genome

To examine the virulence gene profile of UM_CSW, we used our in-house analysis pipeline. We predicted 141 virulence genes which are orthologous to the experimentally verified virulence genes in the virulence factor database (VFDB) (S5 Table). This number constitutes 73.8% of the 191 genes in *M*. *tuberculosis* and includes genes important for mycobacterial cell wall structure, cell entry, protein and lipid metabolism, inhibition of antimicrobial factors, gene expression regulation, and metal transportation. For instance, UM_CSW appeared to have all three fbp-encoded proteins (fbpA, fbpB and fbpC) in the antigen 85 complex required for maintaining the integrity of the mycobacterial cell wall [35], the whole set of nine mammalian cell entry (mce) genes found widely among mycobacteria and five sigma factors (transcription initiation factors) associated with virulence in *M*. *tuberculosis* (sigA, sigE, sigF, sigH and sigM) [36]. The number of PE (proline-glutamate) and PPE (proline-proline-glutamate) protein families, however, is much less than in other mycobacterial pathogens, forming only approximately 1.8% of the coding genome (as predicted by the RAST server), compared to about 10% in *M*. *tuberculosis* [37]. These proteins, unique for mycobacteria are involved in host immune evasion and cell entry [38]. Molecular evolutionary studies have revealed that various orthologues of PE/PPE were found to be deleted in avirulent mycobacterial strains [39], indicating that this family of proteins, owing to their importance in disease pathogenesis, may be specific to the pathogenic mycobacteria [40] including NTM. The PE-PGRS (PE_polimorphic GC-rich sequences) genes that encode cell surface proteins are closely linked with 5 esx gene clusters (ESX1-5) [40]. In UM_CSW, we found only 3 ESX clusters, ESX 3, ESX 4 and ESX 5. The presence of ESX-5 supports the classification of UM_CSW as a slow-growing *Mycobacterium*, as most rapid-growing mycobacteria appear to lack this cluster of secretion factors [40]. Genes of the Lpr family (Lpr-BCDEGHKLOQ) were also predicted. LprG has been reported to play a vital role in the expression of surface-exposed lipoarabinomannan (LAM), through which *M*. *tuberculosis* interacts with the mannose receptors on macrophages. This interaction is critical for cell entry, inhibition of phagosome-lysosome fusion and intracellular survival of *M*. *tuberculosis* [41]. Studies have shown that genetic deletion of LprG resulted in reduced virulence of *M*. *tuberculosis* [42].

### Virulence genes that inhibit apoptosis and resistance to host toxic compounds

Bacteria are able to cause disease if they are able to counteract the antimicrobial responses of the host cell through increasing resistance to host toxic compounds, avoidance of the induction of apoptosis and inhibiting phagosome maturation [43]. We found virulence genes (*hspX*, *ahpC*, *sodC* and *katG*) in UM_CSW that have been reported to detoxify reactive oxygen species (ROS) and reactive nitrogen species (RNS) [44, 45]. UM_CSW also showed the presence of virulence genes like *secA2* and *nuoG* which are reported to be involved in inhibition of apoptosis. Hinchey and colleagues showed that the secA2 is able to prevent apoptosis in bone marrow derived macrophage, while a *M*. *tuberculosis* secA2 mutant induced higher macrophages apoptosis compared to its parental strain [46].

### Putative haemolysin

Haemolytic activity has been reported in *M*. *tuberculosis* [47], *M*. *haemophilum* [48] as well as in fast-growing mycobacteria [49]. The haemolysin-like genes we predicted in UM_CSW might play a role in altering the membrane encompassing the bacterium in macrophage phagosomes to allow molecules to pass through more readily and to facilitate interactions between the bacterium and the host cells’ killing mechanisms [50].

### Mycobactin genes

Mycobactins are a class of salicylate containing siderophores produced by both pathogenic and saprophytic mycobacteria for iron internalization. They also act as a temporary iron-holding molecule in order to prevent sudden influx of excess iron in case of sudden availability of the metal after a period of iron limitation [51, 52]. Recent studies have shown that mycobactins are very important for the growth and the virulence of *M*. *tuberculosis*, and the disruption of mycobactin biosynthesis may lead to attenuation of growth and virulence [53]. The genome of UM_CSW showed the presence of most of the mycobactin genes (*mbt)* except for *mbtJ*, *fadD33*, *fadD332* and *fadE14*. *mbtJ*, encoding for acetyl hydroxylase, has been reported to be involved in synthesis of the mycobactin core scaffold while *fadD33* and *fadE14*, encoding for acyl-AMP ligase/acyl-CoA synthase and acyl-ACP dehydrogenase respectively, are responsible for the assembly of mycobactin side chains [54]. Based on the reports suggesting the importance of *fadD33* in the growth of *M*. *tuberculosis* in the liver [55], it may be hypothesised that the absence of *fadD33* in the genome of UM_CSW may affect its growth in the liver.

### Other virulence genes

Two interesting genes found in UM_CSW, are the *papA2* associated with the biosynthesis of the cell surface lipid Sulfolipid-1 which is unique to pathogenic mycobacteria, and *sapM* which encodes a lipid phosphatase. *PapA2* is essential for the sequential acylation of trehalose-2-sulfate to form SL(1278), a diacylated intermediate in Sulfolipid-1 biosynthesis, which has been shown to activate the adaptive immune response in human patients [56]. The *sapM* gene has been reported to be essential for arresting phagosomal maturation and growth of the pathogen [57].

**Fig 5:**
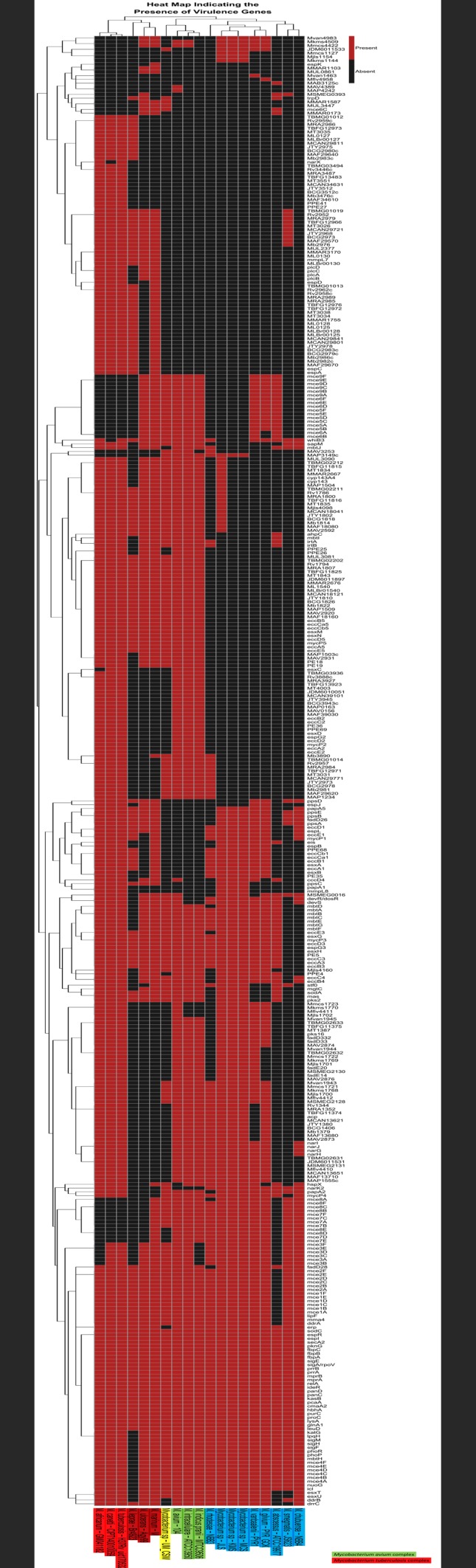
Comparison of virulence genes in UM_CSW and other mycobacterial species. *Mycobacterium* species were grouped according to their virulence gene profiles. From right to left: group 1, rapid growers *M*. *chelonae* to *M*. *rhodesiae*; group 2, members of the *M*. *avium* complex and UM_CSW; group 3, slow growers *M*. *marinum*, *M*. *ulcerans* and *M*. *leprae* and group 4, members of the *M*. *tuberculosis* complex.

Also present were a set of genes related to cellular metabolism such as TBMG01014, Rv2957, RA2984, TBFG12971, MT3031, MCAN29771, JTY2973, BCG2978, Mb2981, MAP1234, MAF29620, which are present only in UM_CSW, *M*. *africanum*, *M*. *canetti*, *M*. *tuberculosis* H37Rv, *M*. *avium* 104, *M*. *intracellulare* ATCC13950 and *M*. *indicus pranii* (Fig 5); the sufBDC-conserved protein, a DUF59-SUF system FeS assembly protein, which, through its implication in the bacterial resistance to iron limitation and oxidative stress, may play an essential role in cell survival [58], and the *mlsA2*, which is normally a part of a large circular virulence plasmid called pMUM present in *M*. *ulcerans*, harbouring three large genes (*mlsA1*, *mlsA2*, and *mlsB*) [59]. These genes are responsible for encoding polyketide synthases that are required for the synthesis of the lipid toxin mycolactone, which is the primary virulence factor for the pathogens [59].

In addition, there were transporter proteins, transmembrane proteins, permease, multidrug resistant proteins (e.g. protein B, Drug efflux membrane protein, EmrB/QacA family drug resistance transporter) and a number of metal transporter proteins such as Znu, czcD and MnTH which may have potential roles in the pathogenicity of UM_CSW.

### Comparative Pathogenomics Analysis

On the heat map (Fig 5), the putative gene *ahpC* which has been reported to be required for stress adaptation and drug resistance [60] is the only virulence gene that differentiated known pathogens (*M*. *tuberculosis* complex, *M*. *avium* complex, *M*. *leprae* Br4923, *M*. *ulcerans* Agy99, *M*. *marinum* M, and *M*. *abscessus* ATCC19977) from mycobacterial species that are not normally considered human pathogens (*M*. *smegmatis* JS623, *M*. *chubuense* NBB4, *M*. *rhodesiae* NBB3, *Mycobacterium* sp. JLS, *Mycobacterium* sp. KMS, *Mycobacterium* sp. MCS, *M*. *vanbaalenii* PYR1, and *M*. *gilvum* PYR_GCK). The gene profile of UM_CSW is most similar to that of the *M*. *avium* complex represented by *M*. *avium* 104, *M*. *intracellulare* ATCC13950, and *M*. *indicus pranii* MTCC9506. A number of mammalian cell entry (mce) operons, *mce5*, *mce6* and *mce9* were found in UM_CSW and *M*. *avium* complex but not in *M*. *tuberculosis* and some other pathogenic mycobacteria. On the other hand, the genes encoding phospholipses C (*plcA*, *plcB*, *plcC* and *plcD*) which enable intracellular pathogens to escape from phagosomal vacuoles by disrupting the host membrane [61] were all absent in UM_CSW and the *M*. *avium* complex but were in the *M*. *tuberculosis* complex and some other mycobacterial pathogens.

The genes found in both the *M*. *tuberculosis* complex and *M*. *avium* complex but not in UM_CSW included many related to the biosynthesis of PDIM (phthiocerol dimycocerosate) which is essential for the prevention of phagosomal acidification [62] and the genes for the biosynthesis and transport of PGL (phenolic glycolipid) which has been reported to reduce pro-inflammatory cytokine release in the host [63, 64]. Other important virulence genes found missing include *nark 2* which encodes the nitrate/nitrite transporter responsible for inducing nitrate reductase activity in response to decreasing oxygen levels by regulating both its transcription and activity [65]; *narG*, *narH*, *narI* and *narJ* that encode the anaerobic nitrate reductase enzyme required for nitrate respiration in the absence of oxygen [66] and *narX* for fused nitrate reductase, the first transcriptionally induced gene in anaerobic dormant mycobacteria [67].

## Discussion

We present here the genome sequence and analysis of a clinically derived isolate, *Mycobacterium sp*. UM_CSW. Using the three global bacterial identification markers, 16S rRNA, *hsp65* and *rpoB*, we failed to resolve the taxonomic position of this isolate which was shown to cluster variously with both rapid- and slow-growing mycobacterial species. After further analyses with nucleotide and protein sequences from whole-genome and whole-proteome data as well as comparative pathogenomic analysis against other mycobacterial species, it became more apparent that UM_CSW is likely to be a novel *Mycobacterium* species closely related to the *M*. *avium* complex.

A thorough *in-silico* functional analysis revealed some interesting features of UM_CSW. This genome has arsenic- and arsenic resistance-related genes suggesting an environmental origin and the potential for the development of resistance to this chemical element. On the other hand, there is evidence for a high pathogenic potential with the presence of genes associated with the maintenance of cell wall integrity; cell entry; evasion of host immune system; resistance against host toxic compounds, iron limitation and oxidative stress; inhibition of apoptosis; and other virulence factors. The results of PathogenFinder also indicated that UM_CSW might be a human pathogen. However, since UM_CSW was isolated from a patient with a long history of bronchiectasis, we cannot discount the possibility of it being an opportunistic pathogen like most NTMs [2]. With the presence of all the potential virulence factors, whether UM_CSW is a human pathogen or just an opportunistic pathogen still needs to be experimentally verified.

## Conclusion

We have successfully sequenced and analyzed the genome of a strain of *Mycobacterium* that might be a new mycobacterial species as we proposed in this study. Our analysis suggested an environmental origin but also a high potential for causing disease in human hosts. Future biological and epidemiological studies will determine its significance as a human pathogen.

## Supporting Information

S1 Fig Phylogenetic tree based on *16S rRNA* gene sequences using Hasegawa-Kishino-Yano with Gamma distribution having invariant site model. (TIF)

S2 Fig Phylogenetic tree based on *hsp65* gene sequences using Tamura-Nei with Gamma distribution model. (TIF)

S3 Fig Phylogenetic tree based on *rpoB* gene sequences using Hasegawa-Kishino-Yano with Gamma distribution having invariant site model. (TIF)

S4 Fig AAI analysis. (TIF)

S1 Table A List of RAST-predicted genes. (PDF)

S2 Table Full list of predicted GIs in UM_CSW genome. (PDF)

S3 Table A list of sporulation-related genes in UM_CSW genome. (PDF)

S4 Table Prediction of human pathogen using PathogenFinder. (PDF)

S5 Table A list of predicted virulence genes in the UM_CSW genome. (PDF)
