# Supplementary figures and images for: Comparative Genomic Analysis Reveals a Possible Novel Non-Tuberculous Mycobacterium Species with High Pathogenic Potential
Source: PLoS One. 2016 Apr 1;11(4):e0150413. doi: 10.1371/journal.pone.0150413 (PMC4818103; doi:10.1371/journal.pone.0150413)

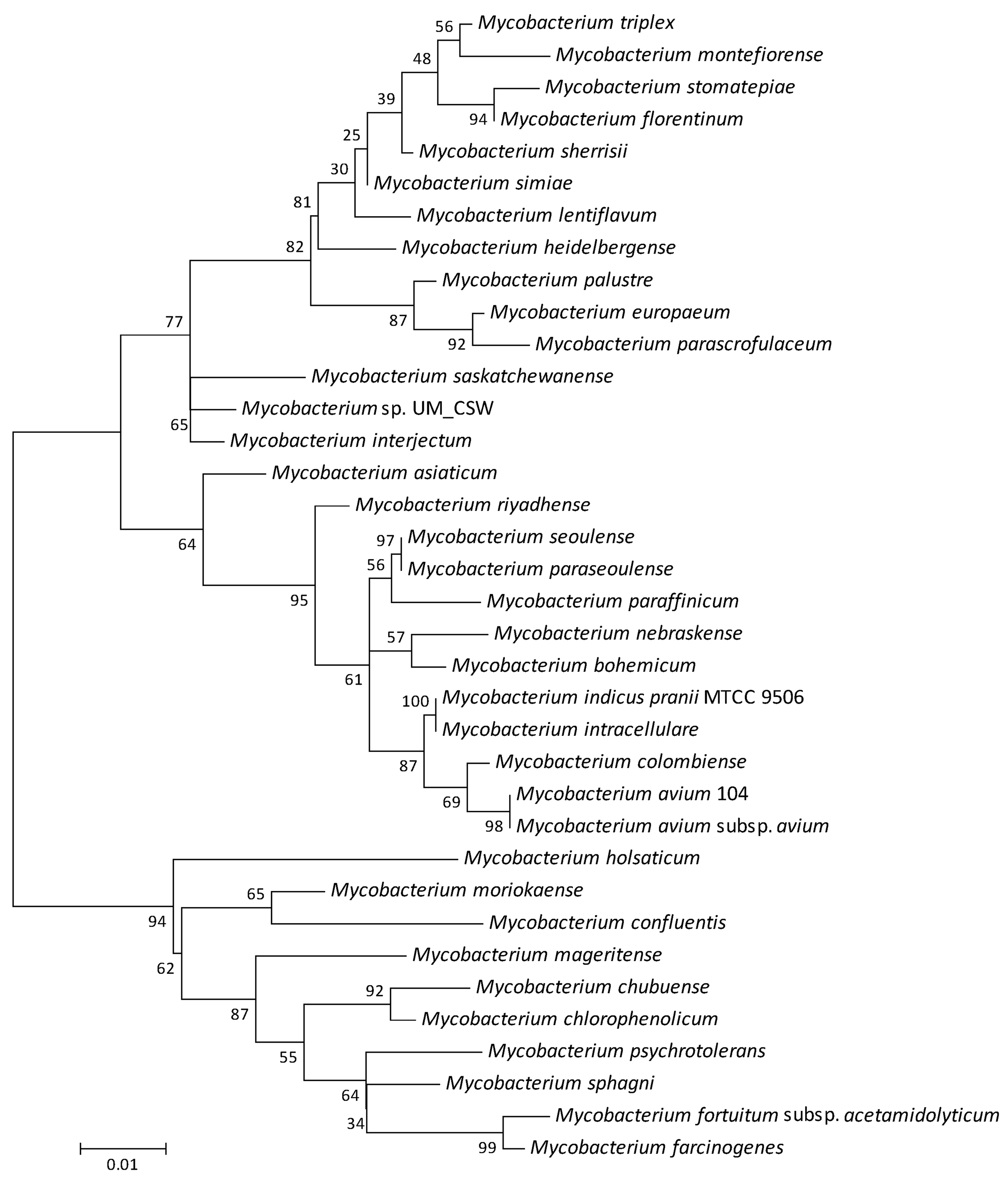

Supplement: S1 Fig — (TIF) [file pone.0150413.s001.tif]

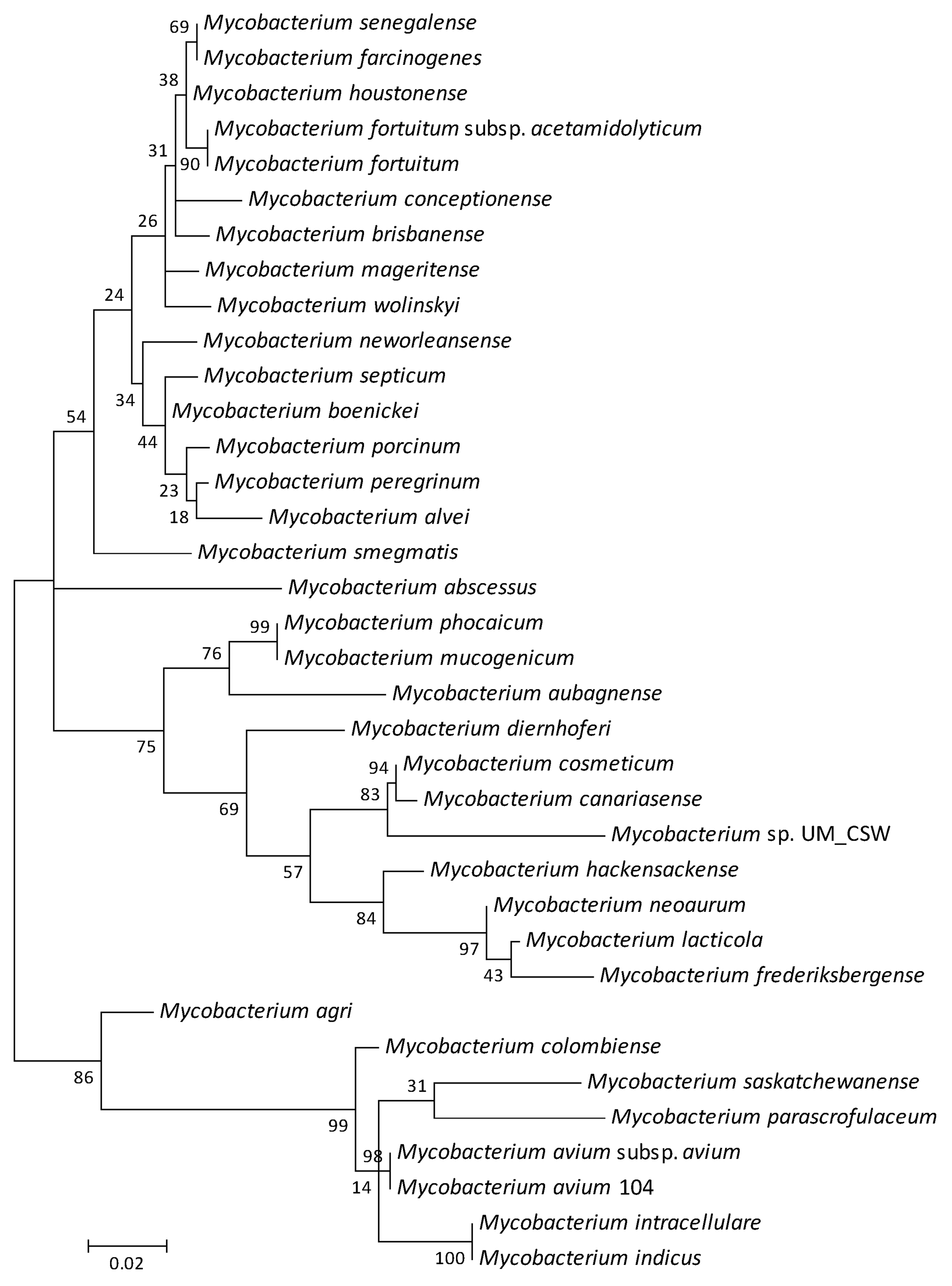

Supplement: S2 Fig — (TIF) [file pone.0150413.s002.tif]

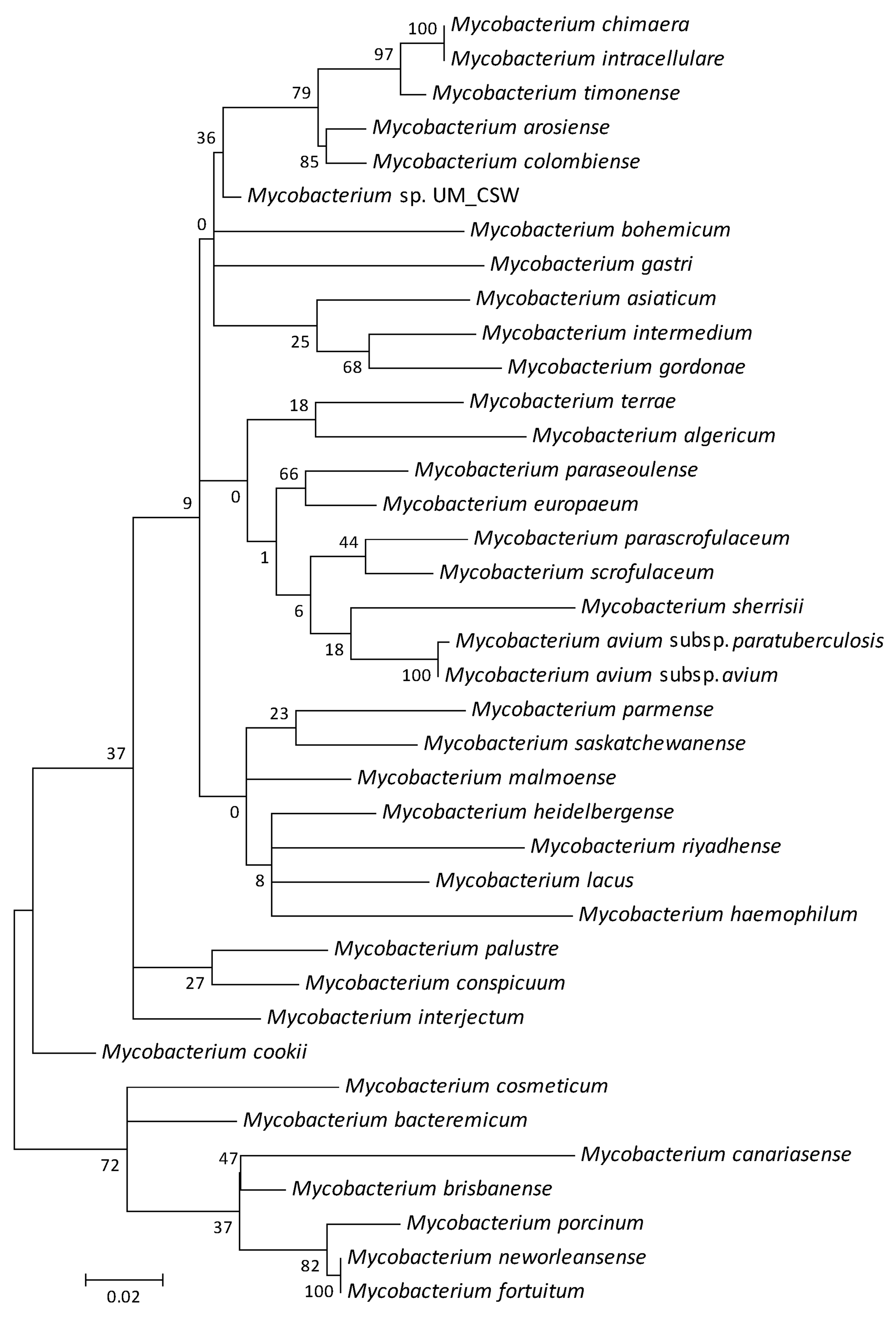

Supplement: S3 Fig — (TIF) [file pone.0150413.s003.tif]

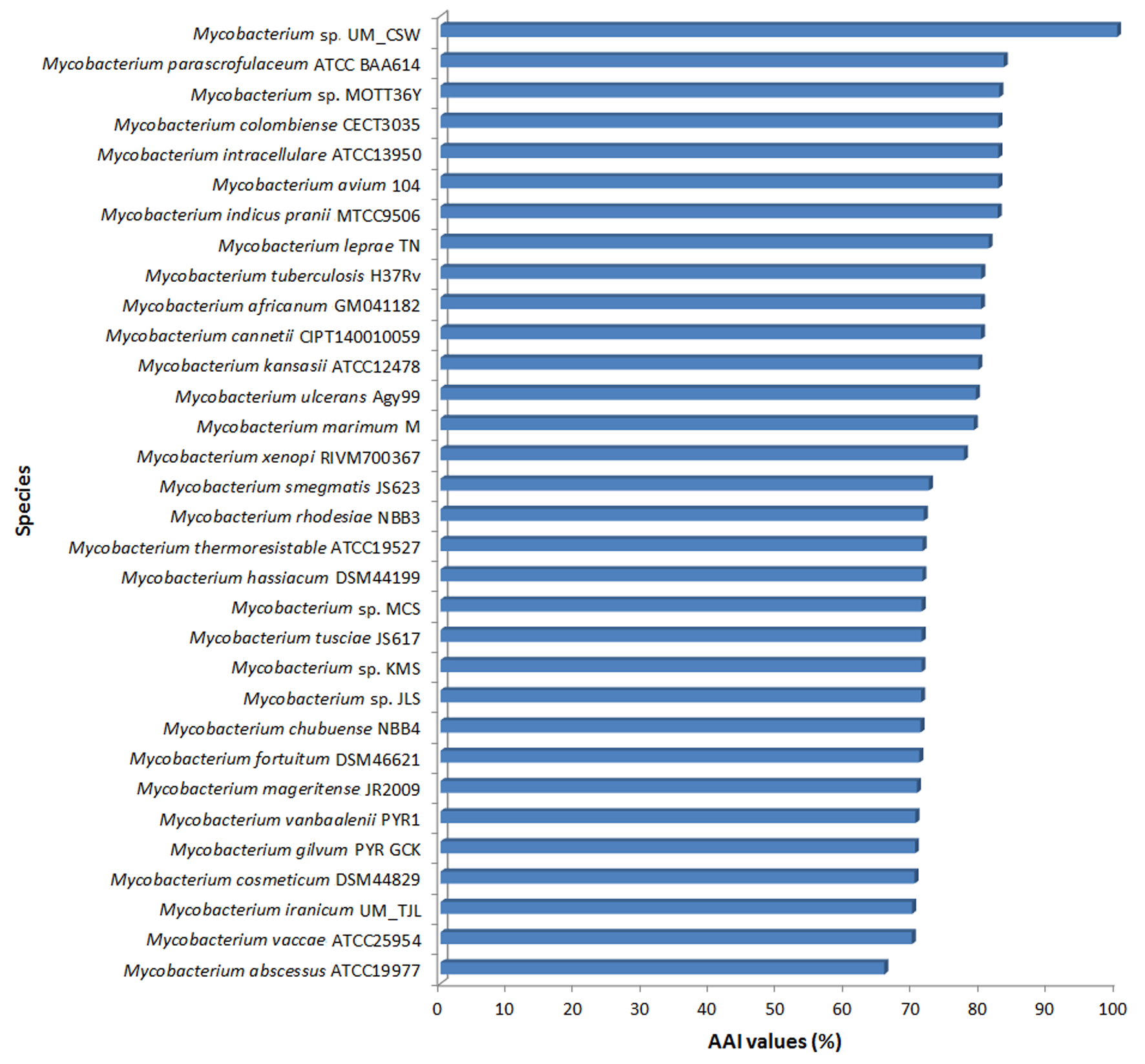

Supplement: S4 Fig — (TIF) [file pone.0150413.s004.tif]
