# Supplementary material for: Comparative Genomic Analysis Reveals a Possible Novel Non-Tuberculous Mycobacterium Species with High Pathogenic Potential
Source: PLoS One. 2016 Apr 1;11(4):e0150413. doi: 10.1371/journal.pone.0150413 (PMC4818103; doi:10.1371/journal.pone.0150413)
Supplement: S1 Table — (PDF) [file pone.0150413.s005.pdf]

Supplementary Table S1: A List of RAST-predicted genes

RAST_ORF_id	contig number	start	stop	strand	function	figfam	evidence_codes
fig|6666666.28482.peg.1	Contig_1	73	597	+	NAD-dependent glyceraldehyde-3-phosphate dehydrogenase (EC 1.2.1.12)	FIG00132586	isu;Redox-dependent_regulation_of_nucleus_processes isu;Pyridoxin_(Vitamin_B6)_Biosynthesis
fig|6666666.28482.peg.2	Contig_1	2128	608	-	NADH-ubiquinone oxidoreductase chain N (EC 1.6.5.3)	FIG00000512	icw(3);Respiratory_Complex_I icw(3);NADH_ubiquinone_oxidoreductase
fig|6666666.28482.peg.3	Contig_1	3630	2125	-	NADH-ubiquinone oxidoreductase chain M (EC 1.6.5.3)	FIG00138227	icw(6);Respiratory_Complex_I icw(6);NADH_ubiquinone_oxidoreductase
fig|6666666.28482.peg.4	Contig_1	5507	3627	-	NADH-ubiquinone oxidoreductase chain L (EC 1.6.5.3)	FIG00068727	icw(1);Respiratory_Complex_I icw(1);NADH_ubiquinone_oxidoreductase
fig|6666666.28482.peg.5	Contig_1	5850	5518	-	NADH-ubiquinone oxidoreductase chain K (EC 1.6.5.3)	FIG00000559	idu(1);Respiratory_Complex_I idu(1);NADH_ubiquinone_oxidoreductase
fig|6666666.28482.peg.6	Contig_1	6617	5814	-	NADH-ubiquinone oxidoreductase chain J (EC 1.6.5.3)	FIG00000569	icw(2);Respiratory_Complex_I icw(2);NADH_ubiquinone_oxidoreductase
fig|6666666.28482.peg.7	Contig_1	7042	6614	-	NADH-ubiquinone oxidoreductase chain I (EC 1.6.5.3)	FIG00138210	icw(4);Respiratory_Complex_I icw(4);NADH_ubiquinone_oxidoreductase
fig|6666666.28482.peg.8	Contig_1	8252	7035	-	NADH-ubiquinone oxidoreductase chain H (EC 1.6.5.3)	FIG00133041	icw(3);Respiratory_Complex_I icw(3);NADH_ubiquinone_oxidoreductase
fig|6666666.28482.peg.9	Contig_1	10549	8249	-	NADH-ubiquinone oxidoreductase chain G (EC 1.6.5.3)	FIG00010922	icw(7);Respiratory_Complex_I icw(7);NADH_ubiquinone_oxidoreductase

fig|6666666.28482.peg.10	Contig_1	11862	10546	-	NADH-ubiquinone oxidoreductase chain F (EC 1.6.5.3)	FIG00117890	icw(5);Respiratory_Complex_I icw(5);NADH_ubiquinone_oxidoreductase
fig|6666666.28482.peg.11	Contig_1	12586	11864	-	NADH-ubiquinone oxidoreductase chain E (EC 1.6.5.3)	FIG00000705	icw(1);Respiratory_Complex_I icw(1);NADH_ubiquinone_oxidoreductase
fig|6666666.28482.peg.12	Contig_1	13875	12583	-	NADH-ubiquinone oxidoreductase chain D (EC 1.6.5.3)	FIG00138449	icw(6);Respiratory_Complex_I icw(6);NADH_ubiquinone_oxidoreductase
fig|6666666.28482.peg.13	Contig_1	14528	13875	-	NADH-ubiquinone oxidoreductase chain C (EC 1.6.5.3)	FIG00000785	icw(4);Respiratory_Complex_I icw(4);NADH_ubiquinone_oxidoreductase
fig|6666666.28482.peg.14	Contig_1	15079	14525	-	NADH-ubiquinone oxidoreductase chain B (EC 1.6.5.3)		icw(1);Respiratory_Complex_I icw(1);NADH_ubiquinone_oxidoreductase
fig|6666666.28482.peg.15	Contig_1	15465	15088	-	NADH ubiquinone oxidoreductase chain A (EC 1.6.5.3)	FIG00000558	icw(4);Respiratory_Complex_I icw(4);NADH_ubiquinone_oxidoreductase
fig|6666666.28482.peg.16	Contig_1	16130	15729	-	Probable response regulator	FIG01378529	ff
fig|6666666.28482.peg.17	Contig_1	16696	16160	-	FIG00824964: hypothetical protein	FIG00824962	ff
fig|6666666.28482.peg.18	Contig_1	17132	16731	-	FIG00821985: hypothetical protein	FIG00821981	ff
fig|6666666.28482.peg.19	Contig_1	17542	17201	-	FIG00825230: hypothetical protein	FIG00825229	ff
fig|6666666.28482.peg.20	Contig_1	18537	17539	-	FIG00823333: hypothetical protein	FIG00823332	ff
fig|6666666.28482.peg.21	Contig_1	19772	18534	-	Isovaleryl-CoA dehydrogenase (EC 1.3.99.10)	FIG00138712	icw(1);HMG_CoA_Synthesis icw(1);Leucine_Degradation_and_HMG-CoA_Metabolism
fig|6666666.28482.peg.22	Contig_1	20392	19775	-	Transcriptional regulator, TetR family	FIG00554417	ff

fig|6666666.28482.peg.23	Contig_1	21288	20392	-	Hydroxymethylglutaryl-CoA lyase (EC 4.1.3.4)	FIG00000963	idu(2);HMG_CoA_Synthesis idu(2);Leucine_Degradation_and_HMG-CoA_Metabolism
fig|6666666.28482.peg.24	Contig_1	22484	21285	-	L-carnitine dehydratase/bile acid-inducible protein F (EC 2.8.3.16)	FIG00450481	ff
fig|6666666.28482.peg.25	Contig_1	23656	22481	-	FIG00822874: hypothetical protein	FIG00822872	ff
fig|6666666.28482.peg.26	Contig_1	24747	23656	-	Homogentisate 1,2-dioxygenase (EC 1.13.11.5)	FIG00001172	isu;Homogentisate_pathway_of_aromatic_compound_degradation isu;Pterin_carbinolamine_dehydratase
fig|6666666.28482.peg.27	Contig_1	25968	25012	-	Alcohol dehydrogenase (EC 1.1.1.1)	FIG01007502	idu(17);Glycerolipid_and_Glycerophospholipid_Metabolism_in_Bacteria idu(17);Fermentations:_Mixed_acid icw(1);Butanol_Biosynthesis
fig|6666666.28482.peg.28	Contig_1	26120	26641	+	GCN5-related N-acetyltransferase		
fig|6666666.28482.peg.29	Contig_1	27839	26634	-	Probable acyl-CoA dehydrogenase (EC 1.3.99.3)	FIG01304408	icw(1);Isoleucine_degradation icw(1);Valine_degradation
fig|6666666.28482.peg.30	Contig_1	29281	27872	-	Butyryl-CoA dehydrogenase (EC 1.3.99.2)	FIG00009149	idu(39);Isoleucine_degradation idu(39);Lysine_fermentation idu(39);Butanol_Biosynthesis idu(39);Acetyl-CoA_fermentation_to_Butyrate idu(39);Valine_degradation idu(39);Anaerobic_respiratory_reductases

fig|6666666.28482.peg.31	Contig_1	30150	29371	-	Histidinol-phosphatase [alternative form] (EC 3.1.3.15)	FIG00001748	idu(1);Histidine_Biosynthesis
fig|6666666.28482.peg.32	Contig_1	30173	30502	+	FIG00820561: hypothetical protein	FIG01314374	ff
fig|6666666.28482.peg.33	Contig_1	31678	30506	-	PPE family protein	FIG00013075	idu(57);CBSS-164757.7.peg.5020
fig|6666666.28482.peg.34	Contig_1	32079	32870	+	Universal stress protein family	FIG00017615	ff
fig|6666666.28482.peg.35	Contig_1	32893	33531	+	Two component transcriptional regulatory protein DevR	FIG01330944	ff
fig|6666666.28482.peg.36	Contig_1	34911	33541	-	Ferredoxin--NADP(+) reductase, actinobacterial (eukaryote-like) type (EC 1.18.1.2)	FIG00134403	isu;Inorganic_Sulfur_Assimilation
fig|6666666.28482.peg.37	Contig_1	35052	36170	+	Peptide chain release factor 2	FIG01255877	isu;Programmed_frameshift icw(1);Translation_termination_factors_bacterial
fig|6666666.28482.peg.38	Contig_1	36160	37155	+	Potassium efflux system KefA protein / Small-conductance mechanosensitive channel	FIG00021330	isu;Potassium_homeostasis
fig|6666666.28482.peg.39	Contig_1	37152	37631	+	FIG01258542: hypothetical protein	FIG01258536	ff
fig|6666666.28482.peg.40	Contig_1	37675	38364	+	Cell division transporter, ATP-binding protein FtsE (TC 3.A.5.1.1)	FIG00002914	isu;Bacterial_Cell_Division

fig|6666666.28482.peg.41	Contig_1	38365	39258	+	Cell division protein FtsX	FIG00000552	icw(1);Bacterial_Cell_Division
fig|6666666.28482.peg.42	Contig_1	39261	39770	+	tmRNA-binding protein SmpB	FIG00000194	isu;Staphylococcal_pathogenicity_islands_SaPI isu;Heat_shock_dnaK_gene_cluster_extended isu;Translation_termination_factors_bacterial
fig|6666666.28482.peg.43	Contig_1	39824	40612	+	FIG00827717: hypothetical protein	FIG00827706	ff
fig|6666666.28482.peg.44	Contig_1	40646	41491	+	FIG00822482: hypothetical protein	FIG00822481	ff
fig|6666666.28482.peg.45	Contig_1	41640	42602	+	hypothetical protein		
fig|6666666.28482.peg.46	Contig_1	44283	43282	-	putative oxidoreductase		
fig|6666666.28482.peg.47	Contig_1	44366	45667	+	Butyryl-CoA dehydrogenase (EC 1.3.99.2)	FIG00009149	idu(39);Isoleucine_degradation idu(39);Lysine_fermentation idu(39);Butanol_Biosynthesis idu(39);Acetyl-CoA_fermentation_to_Butyrate idu(39);Valine_degradation idu(39);Anaerobic_respiratory_reductases
fig|6666666.28482.peg.48	Contig_1	45651	46247	+	hypothetical protein		
fig|6666666.28482.peg.49	Contig_1	46244	47275	+	phosphotransferase		
fig|6666666.28482.peg.50	Contig_1	47272	48036	+	short-chain dehydrogenase/reductase SDR		
fig|6666666.28482.peg.51	Contig_1	48129	48001	-	hypothetical protein		
fig|6666666.28482.peg.52	Contig_1	48199	48666	+	hypothetical protein		

fig|6666666.28482.peg.53	Contig_1	48765	49559	+	Enoyl-CoA hydratase (EC 4.2.1.17)	FIG00033206	idu(51);n-Phenylalkanoic_acid_degradation idu(51);Polyhydroxybutyrate_metabolism icw(1);Isoleucine_degradation idu(51);Fatty_acid_metabolism_cluster icw(1);Lysine_fermentation icw(1);Butanol_Biosynthesis icw(1);Acetyl-CoA_fermentation_to_Butyrate icw(1);Valine_degradation idu(51);Butyrate_metabolism_cluster
fig|6666666.28482.peg.54	Contig_1	49585	50493	+	Membrane protein, putative	FIG01030897	ff
fig|6666666.28482.peg.55	Contig_1	52075	50480	-	hypothetical protein	FIG00638284	ff
fig|6666666.28482.peg.56	Contig_1	52389	52249	-	hypothetical protein		

fig|6666666.28482.peg.57	Contig_1	52280	53470	+	(MTCY164.08c), len: 436. Esterase/lipase, identical to TR:P77909 (EMBL:U76006) ESTERASE/LIPASE (EC 3.1.1.3) (TRIACYLGLYCEROL LIPASE) (437 aa); N-terminal similar to N-terminal of M. tuberculosis PE family members, eg Y03A_MYCTU Q10637 hypothetical glycine-rich 49.6 kd pro (603 aa), fasta scores, opt: 334 E(): 4.8e-13, (43.4% identity in 152 aa overlap). Other relatives include MTCY1A11.25c, 7.8e-14; MTCY21B4.13c, 2.4e-12; MTCY270.06, 5.4e-12; MTCY359.33, 1.2e-11; MTC1A11.04, 1.2ee-11; cY130.seq, 1.3e-11	FIG01316343	ff
fig|6666666.28482.peg.58	Contig_1	54095	53613	-	Transcriptional regulator, HxlR family	FIG00906075	ff
fig|6666666.28482.peg.59	Contig_1	54176	55300	+	Pigment protein	FIG00549183	ff
fig|6666666.28482.peg.60	Contig_1	55297	56262	+	putative oxidoreductase	FIG00945653	ff

fig|6666666.28482.peg.61	Contig_1	56266	57282	+	conserved hypothetical NADP-dependent oxidoreductase		
fig|6666666.28482.peg.62	Contig_1	57678	57496	-	hypothetical protein		
fig|6666666.28482.peg.63	Contig_1	57715	59424	+	N-acetylmuramic acid 6-phosphate etherase (EC 4.2.-.-)	FIG00001103	isu;Recycling_of_Peptidoglycan_Amino_Sugars isu;UDP-N-acetylmuramate_from_Fructose-6-phosphate_Biosynthesis
fig|6666666.28482.peg.64	Contig_1	59542	59405	-	hypothetical protein		
fig|6666666.28482.peg.65	Contig_1	59525	61012	+	Beta-lactamase class C and other penicillin binding proteins	FIG00000681	idu(2);Beta-lactamase
fig|6666666.28482.peg.66	Contig_1	61715	61020	-	hypothetical protein		
fig|6666666.28482.peg.67	Contig_1	61900	62256	+	hypothetical protein		
fig|6666666.28482.peg.68	Contig_1	64139	62283	-	Potassium channel protein	FIG00006661	idu(2);Potassium_homeostasis
fig|6666666.28482.peg.69	Contig_1	64345	65781	+	Glutamine synthetase type I (EC 6.3.1.2)	FIG00134889	idu(2);Glutamine,_Glutamate,_Aspartate_and_Asparagine_Biosynthesis idu(2);Ammonia_assimilation idu(2);Glutamine_synthetases idu(2);Peptidoglycan_Biosynthesis
fig|6666666.28482.peg.70	Contig_1	66471	65788	-	hypothetical protein		
fig|6666666.28482.peg.71	Contig_1	66861	66484	-	hypothetical protein		
fig|6666666.28482.peg.72	Contig_1	67741	67145	-	conserved hypothetical secreted protein		
fig|6666666.28482.peg.73	Contig_1	67869	68102	+	hypothetical protein		
fig|6666666.28482.peg.74	Contig_1	68768	68106	-	conserved hypothetical regulatory protein		

fig|6666666.28482.peg.75	Contig_1	69583	68891	-	conserved hypothetical regulatory protein	FIG00830769	ff
fig|6666666.28482.peg.76	Contig_1	69779	69892	+	hypothetical protein		
fig|6666666.28482.peg.77	Contig_1	69978	70373	+	hypothetical protein		
fig|6666666.28482.peg.78	Contig_1	70370	71197	+	Probable F420-dependent oxidoreductase family protein	FIG01361662	ff
fig|6666666.28482.peg.79	Contig_1	72690	71218	-	flavin-containing monooxygenase (putative secreted protein)		
fig|6666666.28482.peg.80	Contig_1	74175	72727	-	Aldehyde dehydrogenase (EC 1.2.1.3)	FIG01292786	idu(16);Pyruvate_metabolism_II:_acetyl-CoA,_acetogenesis_from_pyruvate idu(16);Glycerolipid_and_Glycerophospholipid_Metabolism_in_Bacteria idu(16);Methylglyoxal_Metabolism
fig|6666666.28482.peg.81	Contig_1	74981	74172	-	4-carboxymuconolactone decarboxylase (EC 4.1.1.44)	FIG01304850	idu(2);Protocatechuate_branch_of_beta-ketoadipate_pathway
fig|6666666.28482.peg.82	Contig_1	75756	75007	-	3-oxoacyl-[acyl-carrier protein] reductase (EC 1.1.1.100)	FIG00621114	idu(38);CBSS-246196.1.peg.364 idu(38);CBSS-164757.7.peg.5020 idu(38);Fatty_Acid_Biosynthesis_FASII
fig|6666666.28482.peg.83	Contig_1	76643	75813	-	oxidoreductase, short chain dehydrogenase-reductase family	FIG01348516	ff

fig|6666666.28482.peg.84	Contig_1	76688	77281	+	Transcriptional regulator, TetR family	FIG00554417	ff
fig|6666666.28482.peg.85	Contig_1	78746	77283	-	Selenoprotein O and cysteine-containing homologs	FIG00004562	isu;Selenoprotein_O
fig|6666666.28482.peg.86	Contig_1	79356	78775	-	hypothetical protein		
fig|6666666.28482.peg.87	Contig_1	79769	79488	-	hypothetical protein		
fig|6666666.28482.peg.88	Contig_1	79955	80764	+	hypothetical protein		
fig|6666666.28482.peg.89	Contig_1	80825	81760	+	Citrate lyase beta chain (EC 4.1.3.6)	FIG00010100	idu(2);Citrate_Metabolism,_Transport,_and_Regulation
fig|6666666.28482.peg.90	Contig_1	81772	82488	+	FIG00829307: hypothetical protein	FIG00829303	ff
fig|6666666.28482.peg.91	Contig_1	83706	82450	-	FIG029007: hypothetical protein	FIG01369934	ff
fig|6666666.28482.peg.92	Contig_1	83754	84110	+	FIG016027: protein of unknown function YeaO	FIG01310523	ff
fig|6666666.28482.peg.93	Contig_1	84112	84984	+	FIG00995258: hypothetical protein	FIG00995257	ff
fig|6666666.28482.peg.94	Contig_1	84999	85628	+	FIG00822523: hypothetical protein	FIG00822519	ff
fig|6666666.28482.peg.95	Contig_1	86005	85637	-	Protein CrcB homolog 2	FIG00820613	ff
fig|6666666.28482.peg.96	Contig_1	86400	86002	-	Protein CrcB homolog 1	FIG01322783	ff
fig|6666666.28482.peg.97	Contig_1	86482	88110	+	Phosphoglucomutase (EC 5.4.2.2)	FIG00000901	ff
fig|6666666.28482.peg.98	Contig_1	88203	89333	+	Multidrug resistance protein B	FIG01356213	ff
fig|6666666.28482.rna.1	Contig_1	89390	89462	+	tRNA-Ala-GGC		isu;tRNAs
fig|6666666.28482.peg.99	Contig_1	89990	91144	+	Putative uncharacterized protein BCG_3873	FIG00994577	ff

fig|6666666.28482.peg.100	Contig_1	92223	91165	-	putative cytochrome P450 hydroxylase	FIG00032437	idu(49);Nitric_oxide_synthase
fig|6666666.28482.peg.101	Contig_1	93210	92557	-	Putative secreted protein	FIG01353358	ff
fig|6666666.28482.peg.102	Contig_1	94905	93499	-	Adenylate cyclase (EC 4.6.1.1)	FIG00001294	idu(25);cAMP_signaling_in_bacteria
fig|6666666.28482.peg.103	Contig_1	95228	95398	+	hypothetical protein		
fig|6666666.28482.peg.104	Contig_1	95930	95445	-	Transcriptional regulator, MarR family		
fig|6666666.28482.peg.105	Contig_1	96026	96649	+	reductase		
fig|6666666.28482.peg.106	Contig_1	96715	97614	+	hypothetical protein	FIG00638284	ff
fig|6666666.28482.peg.107	Contig_1	97595	98335	+	3-oxoacyl-[acyl-carrier protein] reductase (EC 1.1.1.100)	FIG00621114	idu(38);CBSS-246196.1.peg.364 idu(38);CBSS-164757.7.peg.5020 idu(38);Fatty_Acid_Biosynthesis_FASI I
fig|6666666.28482.peg.108	Contig_1	99218	98397	-	5-valerolactone hydrolase	FIG00968451	ff
fig|6666666.28482.peg.109	Contig_1	100686	99277	-	Aldehyde dehydrogenase (EC 1.2.1.3)	FIG01292786	idu(16);Pyruvate_metabolism_II:_ace tyl- CoA,_acetogenesis_from_pyruvate idu(16);Glycerolipid_and_Glycerophospholipid_Metabolism_in_Bacteria idu(16);Methylglyoxal_Metabolism
fig|6666666.28482.peg.110	Contig_1	100782	102047	+	Phenylpropionate dioxygenase and related ring-hydroxylating dioxygenases, large terminal subunit	FIG01371907	ff

fig|6666666.28482.peg.111	Contig_1	102044	102382	+	FIG00821688: hypothetical protein	FIG00821687	ff
fig|6666666.28482.peg.112	Contig_1	102451	103626	+	amidohydrolase		
fig|6666666.28482.peg.113	Contig_1	103623	104099	+	Transcriptional regulator, MarR family	FIG00554610	ff
fig|6666666.28482.peg.114	Contig_1	104175	104474	+	Probable phenylacetic acid degradation NADH oxidoreductase paaE (EC 1.-.-.-)	FIG01355358	ff
fig|6666666.28482.peg.115	Contig_1	104510	105214	+	3-oxoacyl-[acyl-carrier protein] reductase (EC 1.1.1.100)	FIG00621114	idu(38);CBSS-246196.1.peg.364 idu(38);CBSS-164757.7.peg.5020 idu(38);Fatty_Acid_Biosynthesis_FASI I
fig|6666666.28482.peg.116	Contig_1	105257	106525	+	Phenylpropionate dioxygenase and related ring-hydroxylating dioxygenases, large terminal subunit	FIG01371907	ff
fig|6666666.28482.peg.117	Contig_1	106528	106863	+	FIG00822032: hypothetical protein	FIG00822031	ff
fig|6666666.28482.peg.118	Contig_1	106903	107748	+	Taurine catabolism dioxygenase TauD/TfdA	FIG00822811	ff
fig|6666666.28482.peg.119	Contig_1	107761	108072	+	conserved hypothetical protein		
fig|6666666.28482.peg.120	Contig_1	108995	108120	-	Transcriptional regulator, IclR family	FIG00022569	idu(12);Homogentisate_pathway_of_ aromatic_compound_degradation
fig|6666666.28482.peg.121	Contig_1	109082	110026	+	Short chain dehydrogenase	FIG01310822	ff
fig|6666666.28482.peg.122	Contig_1	110023	110826	+	FIG00820258: hypothetical protein	FIG01126297	ff

fig|6666666.28482.peg.123	Contig_1	110833	112047	+	FIG00830454: hypothetical protein	FIG01129856	ff
fig|6666666.28482.peg.124	Contig_1	112062	113342	+	FIG00829067: hypothetical protein	FIG01126475	ff
fig|6666666.28482.peg.125	Contig_1	116423	113355	-	hypothetical protein	FIG00638284	ff
fig|6666666.28482.peg.126	Contig_1	117750	116599	-	PPE family protein	FIG00013075	icw(1);CBSS-164757.7.peg.5020
fig|6666666.28482.peg.127	Contig_1	118339	118058	-	FIG00826766: hypothetical protein	FIG00826765	ff
fig|6666666.28482.peg.128	Contig_1	118441	119937	+	PE family protein		ff
fig|6666666.28482.peg.129	Contig_1	119948	120754	+	3-oxoacyl-[acyl-carrier protein] reductase (EC 1.1.1.100)	FIG00621114	idu(38);CBSS-246196.1.peg.364 idu(38);CBSS-164757.7.peg.5020 idu(38);Fatty_Acid_Biosynthesis_FASII
fig|6666666.28482.peg.130	Contig_1	123278	120759	-	Glycogen phosphorylase (EC 2.4.1.1)	FIG00000476	idu(1);Glycogen_metabolism idu(1);Maltose_and_Maltodextrin_Utilization
fig|6666666.28482.peg.131	Contig_1	124265	123426	-	hypothetical protein	FIG00638284	ff
fig|6666666.28482.peg.132	Contig_1	124435	125931	+	hypothetical protein	FIG00638284	ff
fig|6666666.28482.peg.133	Contig_1	128294	125928	-	Transmembrane transport protein MmpL13b	FIG01314334	ff
fig|6666666.28482.peg.134	Contig_1	128546	128980	+	Transcriptional regulator, TetR family		
fig|6666666.28482.peg.135	Contig_1	129732	129019	-	conserved hypothetical secreted protein		
fig|6666666.28482.peg.136	Contig_1	129789	130685	+	FIG00821214: hypothetical protein	FIG00821213	ff
fig|6666666.28482.peg.137	Contig_1	130727	131146	+	Possible membrane protein	FIG01121868	ff
fig|6666666.28482.peg.138	Contig_1	131348	131103	-	hypothetical protein		

fig|6666666.28482.peg.139	Contig_1	131492	132292	+	5-keto-D-gluconate 5-reductase (EC 1.1.1.69)	FIG00001584	isu;D-gluconate_and_ketogluconates_metabolism
fig|6666666.28482.peg.140	Contig_1	132293	133237	+	aminoglycoside phosphotransferase	FIG00824137	ff
fig|6666666.28482.peg.141	Contig_1	133439	133245	-	hypothetical protein		
fig|6666666.28482.peg.142	Contig_1	133699	133559	-	hypothetical protein		
fig|6666666.28482.peg.143	Contig_1	133656	134342	+	FIG00826132: hypothetical protein	FIG00826131	ff
fig|6666666.28482.peg.144	Contig_1	135637	134339	-	Butyryl-CoA dehydrogenase (EC 1.3.99.2)	FIG00009149	idu(39);Isoleucine_degradation idu(39);Lysine_fermentation idu(39);Butanol_Biosynthesis idu(39);Acetyl-CoA_fermentation_to_Butyrate idu(39);Valine_degradation idu(39);Anaerobic_respiratory_reductases
fig|6666666.28482.peg.145	Contig_1	136436	135660	-	Enoyl-CoA hydratase (EC 4.2.1.17)	FIG00033206	idu(51);n-Phenylalkanoic_acid_degradation idu(51);Polyhydroxybutyrate_metabolism icw(1);Isoleucine_degradation idu(51);Fatty_acid_metabolism_cluster icw(1);Lysine_fermentation icw(1);Butanol_Biosynthesis icw(1);Acetyl-CoA_fermentation_to_Butyrate icw(1);Valine_degradation idu(51);Butyrate_metabolism_cluster
fig|6666666.28482.peg.146	Contig_1	136753	137709	+	dehydrogenase	FIG01007645	ff

fig|6666666.28482.peg.147	Contig_1	138534	137713	-	Dehydrogenases with different specificities (related to short-chain alcohol dehydrogenases)	FIG00975245	ff
fig|6666666.28482.peg.148	Contig_1	139441	138563	-	Polyphosphate kinase 2 (EC 2.7.4.1)	FIG00507844	isu;Polyphosphate
fig|6666666.28482.peg.149	Contig_1	141001	139475	-	FIG00824604: hypothetical protein	FIG00824603	ff
fig|6666666.28482.peg.150	Contig_1	141176	141060	-	hypothetical protein		
fig|6666666.28482.peg.151	Contig_1	141472	141173	-	hypothetical protein		
fig|6666666.28482.peg.152	Contig_1	143833	141575	-	Carbon starvation protein A	FIG00001750	isu;Stress_related_cluster isu;Two-component_sensor_regulator_linked_to_Carbon_Starvation_Protein_A isu;Carbon_Starvation
fig|6666666.28482.peg.153	Contig_1	145456	143885	-	ATP-dependent DNA ligase (EC 6.5.1.1)	FIG00052980	idu(2);DNA_replication,_archaeal idu(2);DNA_Repair_Base_Excision idu(2);DNA_ligases
fig|6666666.28482.peg.154	Contig_1	145549	146307	+	3-hydroxyacyl-CoA dehydrogenase type II (EC 1.1.1.35)	FIG01073099	ff
fig|6666666.28482.peg.155	Contig_1	146417	148594	+	FadE22	FIG00824887	ff
fig|6666666.28482.peg.156	Contig_1	148898	148671	-	FIG00821891: hypothetical protein	FIG00821889	ff
fig|6666666.28482.peg.157	Contig_1	149067	150260	+	Acetyl-CoA acetyltransferase	FIG01005417	ff
fig|6666666.28482.peg.158	Contig_1	150269	151717	+	Transcriptional regulator, GntR family	FIG00621925	ff
fig|6666666.28482.peg.159	Contig_1	151819	152823	+	FIG00823661: hypothetical protein	FIG00823660	ff
fig|6666666.28482.peg.160	Contig_1	153045	153518	+	hypothetical protein		

fig|6666666.28482.peg.161	Contig_1	155027	153543	-	Cytochrome P450 136	FIG01314131	ff
fig|6666666.28482.peg.162	Contig_1	155214	155074	-	hypothetical protein		
fig|6666666.28482.peg.163	Contig_1	155227	155805	+	Transcriptional regulator, TetR family	FIG00554417	ff
fig|6666666.28482.peg.164	Contig_1	155824	156738	+	Short chain dehydrogenase	FIG01310822	ff
fig|6666666.28482.peg.165	Contig_1	157746	156700	-	DNA polymerase-like protein MT3142	FIG00149033	isu;DNA_repair,_bacterial
fig|6666666.28482.peg.166	Contig_1	158338	157736	-	Transcriptional regulator, TetR family	FIG00554417	ff
fig|6666666.28482.peg.167	Contig_1	158428	158985	+	NADPH:quinone oxidoreductase	FIG01132576	ff
fig|6666666.28482.peg.168	Contig_1	159525	159764	+	Glutaredoxin-like protein NrdH, required for reduction of Ribonucleotide reductase class Ib	FIG00003291	isu;Glutathione:_Redox_cycle isu;Ribonucleotide_reduction isu;Glutaredoxins
fig|6666666.28482.peg.169	Contig_1	159805	160314	+	Ribonucleotide reduction protein NrdI	FIG00001039	icw(2);Ribonucleotide_reduction
fig|6666666.28482.peg.170	Contig_1	160284	163676	+	Ribonucleotide reductase of class Ib (aerobic), alpha subunit (EC 1.17.4.1)	FIG00001069	icw(1);Ribonucleotide_reduction
fig|6666666.28482.peg.171	Contig_1	165258	163711	-	Choline dehydrogenase (EC 1.1.99.1)	FIG00057416	idu(4);Choline_uptake_and_conversion_to_betaine_clusters idu(4);Choline_and_Betaine_Uptake_and_Betaine_Biosynthesis
fig|6666666.28482.peg.172	Contig_1	165497	166399	+	Putative S-adenosyl-L-methionine-dependent methyltransferase		
fig|6666666.28482.peg.173	Contig_1	166771	166917	+	hypothetical protein		

fig|6666666.28482.peg.174	Contig_1	166914	169778	+	Putative membrane protein	FIG01056920	ff	
fig|6666666.28482.peg.175	Contig_1	169816	171219	+	FAD linked oxidase domain protein	FIG00498745	ff	
fig|6666666.28482.peg.176	Contig_1	172154	171333	-	alpha/beta hydrolase fold-1	FIG00823727	ff	
fig|6666666.28482.peg.177	Contig_1	172565	173353	+	putative transcriptional regulator, AraC family			
fig|6666666.28482.peg.178	Contig_1	173462	174148	+	Transcriptional regulator, TetR family	FIG00554417	ff	
fig|6666666.28482.peg.179	Contig_1	174264	175817	+	Monooxygenase, flavin-binding family	FIG01367379	ff	
fig|6666666.28482.peg.180	Contig_1	176687	175779	-	Short-chain dehydrogenase/reductase SDR		idu(13);CBSS-164757.7.peg.5020	idu(13);Transcription_repair_cluster
fig|6666666.28482.peg.181	Contig_1	176789	177361	+	Transcriptional regulator, TetR family	FIG00554417	ff	
fig|6666666.28482.peg.182	Contig_1	177881	177333	-	Transcriptional regulator, TetR family	FIG00554417	ff	
fig|6666666.28482.peg.183	Contig_1	178311	177865	-	FIG00821720: hypothetical protein	FIG00821718	ff	
fig|6666666.28482.peg.184	Contig_1	179925	178345	-	FAD dependent oxidoreductase			
fig|6666666.28482.peg.185	Contig_1	180019	180633	+	Transcriptional regulator, TetR family	FIG00554417	ff	
fig|6666666.28482.peg.186	Contig_1	180746	181720	+	Ribonucleotide reductase of class Ib (aerobic), beta subunit (EC 1.17.4.1)	FIG00000960	isu;Ribonucleotide_reduction	
fig|6666666.28482.peg.187	Contig_1	182017	183054	+	FOG: GGDEF domain			
fig|6666666.28482.peg.188	Contig_1	183051	183581	+	hypothetical protein			
fig|6666666.28482.peg.189	Contig_1	183885	183649	-	hypothetical protein			

fig|6666666.28482.peg.190	Contig_1	184037	184249	+	hypothetical protein		
fig|6666666.28482.peg.191	Contig_1	184465	184881	+	FIG00822200: hypothetical protein	FIG00822199	ff
fig|6666666.28482.peg.192	Contig_1	185910	184870	-	Cinnamyl alcohol dehydrogenase/reductase (EC 1.1.1.195) @ Alcohol dehydrogenase (EC 1.1.1.1)		idu(17);Glycerolipid_and_Glycerophospholipid_Metabolism_in_Bacteria idu(17);Fermentations:_Mixed_acid idu(17);Butanol_Biosynthesis
fig|6666666.28482.peg.193	Contig_1	187059	185989	-	Iron(III) dicitrate transport system, periplasmic iron-binding protein FecB (TC 3.A.1.14.1)	FIG00054285	ff
fig|6666666.28482.peg.194	Contig_1	187223	188983	+	Cytochrome c oxidase polypeptide I (EC 1.9.3.1)	FIG00132629	isu;Terminal_cytochrome_C_oxidases
fig|6666666.28482.peg.195	Contig_1	188999	190234	+	Phosphoserine phosphatase (EC 3.1.3.3)	FIG00000149	idu(3);CBSS-336982.3.peg.3874 idu(3);Serine_Biosynthesis
fig|6666666.28482.peg.196	Contig_1	190287	191162	+	ABC transporter ATP-binding protein	FIG01956052	ff
fig|6666666.28482.peg.197	Contig_1	191159	191992	+	FIG00996530: hypothetical protein	FIG00996529	ff

fig|6666666.28482.peg.198	Contig_1	191980	192672	+	Enoyl-CoA hydratase (EC 4.2.1.17)	FIG00033206	idu(51);n-Phenylalkanoic_acid_degradation idu(51);Polyhydroxybutyrate_metabolism idu(51);Isoleucine_degradation idu(51);Fatty_acid_metabolism_cluster idu(51);Lysine_fermentation idu(51);Butanol_Biosynthesis idu(51);Acetyl-CoA_fermentation_to_Butyrate idu(51);Valine_degradation idu(51);Butyrate_metabolism_cluster
fig|6666666.28482.peg.199	Contig_1	192683	193666	+	Methyltransferase domain	FIG00995955	ff
fig|6666666.28482.peg.200	Contig_1	193706	194902	+	Methyltransferase (EC 2.1.1.-)	FIG00515945	ff
fig|6666666.28482.peg.201	Contig_1	194960	195655	+	Possible membrane protein	FIG01121868	ff
fig|6666666.28482.peg.202	Contig_1	196077	195739	-	FIG00822582: hypothetical protein	FIG00822580	ff
fig|6666666.28482.peg.203	Contig_1	197396	196074	-	FIG00995884: hypothetical protein	FIG00820108	ff
fig|6666666.28482.peg.204	Contig_1	197486	198223	+	Galactoside O-acetyltransferase (EC 2.3.1.18)	FIG00139238	isu;Lactose_utilization
fig|6666666.28482.peg.205	Contig_1	198772	198230	-	FIG00820106: hypothetical protein	FIG00820103	ff
fig|6666666.28482.peg.206	Contig_1	200187	198856	-	Deoxyribodipyrimidine photolyase (EC 4.1.99.3)	FIG00000633	isu;DNA_repair,_bacterial_photolyase isu;EC699-706

fig|6666666.28482.peg.207	Contig_1	200670	200191	-	tryptophan-rich sensory protein	FIG01211525	ff
fig|6666666.28482.peg.208	Contig_1	200834	200667	-	FIG00821761: hypothetical protein	FIG00821760	ff
fig|6666666.28482.peg.209	Contig_1	200909	201382	+	Transcriptional regulator, MarR family	FIG00554610	ff
fig|6666666.28482.peg.210	Contig_1	203576	201333	-	Transmembrane transport protein MmpL13b	FIG01314334	ff
fig|6666666.28482.peg.211	Contig_1	203767	204357	+	hypothetical protein	FIG00638284	ff
fig|6666666.28482.peg.212	Contig_1	204360	204863	+	TspO and MBR like proteins		
fig|6666666.28482.peg.213	Contig_1	204860	205906	+	Isopentenyl-diphosphate delta-isomerase, FMN-dependent (EC 5.3.3.2)	FIG00001120	isu;Isoprenoid_Biosynthesis isu;Isoprenoid_Biosynthesis:_Interconversions
fig|6666666.28482.peg.214	Contig_1	205917	207179	+	glycosyltransferase, putative	FIG01227982	ff
fig|6666666.28482.peg.215	Contig_1	207686	207144	-	Transcriptional regulator, MarR family	FIG00554610	ff
fig|6666666.28482.peg.216	Contig_1	208170	207739	-	putative membrane protein	FIG00672847	ff
fig|6666666.28482.peg.217	Contig_1	209210	208218	-	CrtV-methyltransferase-like protein	FIG00121395	ff
fig|6666666.28482.peg.218	Contig_1	210715	209207	-	"phi-Carotenoid synthase" (EC 1.3.-.- and EC 2.1.1-)	FIG00039628	ff
fig|6666666.28482.peg.219	Contig_1	211437	210712	-	CrtT-methyltransferase-like protein	FIG00121578	ff
fig|6666666.28482.peg.220	Contig_1	211735	211424	-	Lycopene cyclase	FIG00117432	ff

fig|6666666.28482.peg.221	Contig_1	212055	211732	-	Lycopene cyclase	FIG00117432	ff
fig|6666666.28482.peg.222	Contig_1	212993	212052	-	Phytoene synthase (EC 2.5.1.32)	FIG00000928	isu;Protein_deglycation isu;Proteorhodopsin isu;CBSS-320388.3.peg.3759
fig|6666666.28482.peg.223	Contig_1	214519	212990	-	Phytoene dehydrogenase (EC 1.14.99.-)	FIG00132544	icw(1);Protein_deglycation icw(1);Proteorhodopsin
fig|6666666.28482.peg.224	Contig_1	215592	214516	-	IdsA2_2		
fig|6666666.28482.peg.225	Contig_1	216319	215840	-	hypothetical protein		
fig|6666666.28482.peg.226	Contig_1	216630	217304	+	hypothetical protein		
fig|6666666.28482.peg.227	Contig_1	218669	217314	-	Cell division inhibitor	FIG00002048	idu(1);CBSS-83333.1.peg.946 idu(1);Persister_Cells
fig|6666666.28482.peg.228	Contig_1	219924	218680	-	Glycosyl transferase, group 1	FIG00018328	ff
fig|6666666.28482.peg.229	Contig_1	221608	220022	-	Glycogen branching enzyme, GH-57-type, archaeal (EC 2.4.1.18)	FIG00002744	isu;Glycogen_metabolism
fig|6666666.28482.peg.230	Contig_1	222423	221605	-	Methyltransferase (EC 2.1.1.-)	FIG00515945	ff
fig|6666666.28482.peg.231	Contig_1	222652	223452	+	Electron transfer flavoprotein, beta subunit	FIG01304886	isu;Lysine_fermentation isu;Acetyl-CoA_fermentation_to_Butyrate isu;Heme,_hemin_uptake_and_utilization_systems_in_GramNegatives
fig|6666666.28482.peg.232	Contig_1	223509	224450	+	Electron transfer flavoprotein, alpha subunit	FIG00136866	icw(1);Lysine_fermentation icw(1);Acetyl-CoA_fermentation_to_Butyrate
fig|6666666.28482.peg.233	Contig_1	224688	225545	+	Putative hemolysin	FIG01004154	ff

fig|6666666.28482.peg.234	Contig_1	225542	226363	+	1-acyl-sn-glycerol-3-phosphate acyltransferase (EC 2.3.1.51)	FIG00005243	idu(6);Glycerolipid_and_Glycerophospholipid_Metabolism_in_Bacteria idu(6);Ribosome_post-transcriptional_modification_and_chromosomal_segregation_cluster
fig|6666666.28482.peg.235	Contig_1	226397	227575	+	Cysteine desulfurase (EC 2.8.1.7)	FIG00000001	idu(2);Iron-sulfur_cluster_assembly idu(2);CBSS-84588.1.peg.1247 idu(2);CBSS-393130.3.peg.794 idu(2);mnm5U34_biosynthesis_bacteria idu(2);Alanine_biosynthesis
fig|6666666.28482.peg.236	Contig_1	227576	228652	+	tRNA-specific 2-thiouridylase MnmA	FIG00000626	isu;RNA_methylation
fig|6666666.28482.peg.237	Contig_1	228798	229082	+	hypothetical protein		
fig|6666666.28482.peg.238	Contig_1	229683	229087	-	Lipoprotein LpqA	FIG01316065	ff
fig|6666666.28482.peg.239	Contig_1	229827	230846	+	vitamin-B12 independent methionine synthase family protein	FIG00543935	ff
fig|6666666.28482.peg.240	Contig_1	230911	231396	+	hypothetical protein		
fig|6666666.28482.peg.241	Contig_1	231393	231710	+	Transcriptional regulator, ArsR family	FIG00003845	idu(3);CBSS-1085.1.peg.1363
fig|6666666.28482.peg.242	Contig_10	672	19	-	Transcriptional regulator, TetR family	FIG00554417	ff
fig|6666666.28482.peg.243	Contig_10	788	1153	+	Possible membrane protein	FIG01121868	ff

fig|6666666.28482.peg.244	Contig_10	1188	1658	+	Cell division protein DivIC (FtsB), stabilizes FtsL against RasP cleavage	FIG00485194	idu(10);Possible_new_toxin-antitoxin_system_including_DivIC idu(10);Bacterial_Cell_Division idu(10);Cell_division-ribosomal_stress_proteins_cluster idu(10);Bacterial_Cytoskeleton idu(10);Stationary_phase_repair_cluster
fig|6666666.28482.peg.245	Contig_10	2436	1669	-	Enoyl-CoA hydratase (EC 4.2.1.17)	FIG00033206	idu(51);n-Phenylalkanoic_acid_degradation idu(51);Polyhydroxybutyrate_metabolism idu(51);Isoleucine_degradation idu(51);Fatty_acid_metabolism_cluster idu(51);Lysine_fermentation idu(51);Butanol_Biosynthesis idu(51);Acetyl-CoA_fermentation_to_Butyrate idu(51);Valine_degradation idu(51);Butyrate_metabolism_cluster
fig|6666666.28482.peg.246	Contig_10	3155	2433	-	Phenylacetic acid degradation operon negative regulatory protein PaaX	FIG00020123	ff
fig|6666666.28482.peg.247	Contig_10	4097	3159	-	Enoyl-CoA hydratase	FIG01356316	ff
fig|6666666.28482.peg.248	Contig_10	5769	4159	-	Acyl-CoA dehydrogenase (EC 1.3.99.3)	FIG00002266	ff
fig|6666666.28482.peg.249	Contig_10	6640	5834	-	Lipoprotein LpqP	FIG01387107	ff

fig|6666666.28482.peg.250	Contig_10	7437	6682	-	Endonuclease IV (EC 3.1.21.2)	FIG00000899	isu;DNA_repair,_bacterial
fig|6666666.28482.peg.251	Contig_10	11520	7573	-	DNA-directed RNA polymerase beta' subunit (EC 2.7.7.6)	FIG00000242	isu;Mycobacterium_virulence_operon_involved_in_DNA_transcription isu;RNA_polymerase_bacterial
fig|6666666.28482.peg.252	Contig_10	14867	11577	-	DNA-directed RNA polymerase beta subunit (EC 2.7.7.6)	FIG00000156	icw(1);Mycobacterium_virulence_operon_involved_in_DNA_transcription icw(1);RNA_polymerase_bacterial
fig|6666666.28482.peg.253	Contig_10	16504	15491	-	Methionine ABC transporter ATP-binding protein	FIG00006151	ff
fig|6666666.28482.peg.254	Contig_10	18146	16665	-	"Lignostilbene-alpha,beta-dioxygenase and related enzymes"	FIG01324754	ff
fig|6666666.28482.peg.255	Contig_10	18218	18901	+	Transcriptional regulator, TetR family	FIG00554417	ff
fig|6666666.28482.peg.256	Contig_10	19347	18952	-	LSU ribosomal protein L7/L12 (P1/P2)	FIG00000141	icw(1);Ribosome_LSU_bacterial isu;LSU_ribosomal_proteins_cluster
fig|6666666.28482.peg.257	Contig_10	20013	19390	-	LSU ribosomal protein L10p (P0)	FIG00000159	isu;Ribosome_LSU_bacterial icw(1);LSU_ribosomal_proteins_cluster
fig|6666666.28482.peg.258	Contig_10	21169	20255	-	Sugar kinase	FIG01338213	ff
fig|6666666.28482.peg.259	Contig_10	21771	21169	-	POSSIBLE MALONYL COA-ACYL CARRIER PROTEIN TRANSACYLASE FABD2 (MCT) (EC 2.3.1.39)	FIG00820219	ff

fig|6666666.28482.peg.260	Contig_10	25946	21783	-	Alpha-mannosidase (EC 3.2.1.24)	FIG00009194	isu;Mannose_Metabolism
fig|6666666.28482.peg.261	Contig_10	26005	26595	+	FIG00823430: hypothetical protein	FIG00823427	ff
fig|6666666.28482.peg.262	Contig_10	26571	27572	+	carboxyl esterase	FIG01381021	ff
fig|6666666.28482.peg.263	Contig_10	27625	28971	+	Ubiquinone biosynthesis monooxygenase UbiB	FIG00000320	idu(1);Ubiquinone_Biosynthesis
fig|6666666.28482.peg.264	Contig_10	28981	29886	+	carboxyl esterase	FIG01381021	ff
fig|6666666.28482.peg.265	Contig_10	29979	30839	+	Methoxy mycolic acid synthase 1 MmaA1 (EC 2.1.1.-)	FIG01353571	ff
fig|6666666.28482.peg.266	Contig_10	30876	31739	+	Cyclopropane-fatty-acyl-phospholipid synthase (EC 2.1.1.79)	FIG00001555	ff
fig|6666666.28482.peg.267	Contig_10	31857	32738	+	Cyclopropane-fatty-acyl-phospholipid synthase (EC 2.1.1.79)	FIG00001555	ff
fig|6666666.28482.peg.268	Contig_10	32826	33725	+	Cyclopropane-fatty-acyl-phospholipid synthase (EC 2.1.1.79)	FIG00001555	ff
fig|6666666.28482.peg.269	Contig_10	33822	34520	+	FIG00822465: hypothetical protein	FIG00822464	ff
fig|6666666.28482.peg.270	Contig_10	34529	35746	+	RNA polymerase sigma-70 factor, ECF subfamily	FIG00952839	ff
fig|6666666.28482.peg.271	Contig_10	36523	35816	-	LSU ribosomal protein L1p (L10Ae)	FIG00000264	icw(1);Ribosome_LSU_bacterial icw(3);LSU_ribosomal_proteins_cluster
fig|6666666.28482.peg.272	Contig_10	37074	36646	-	LSU ribosomal protein L11p (L12e)	FIG00000182	isu;Ribosome_LSU_bacterial icw(1);LSU_ribosomal_proteins_cluster

fig|6666666.28482.peg.273	Contig_10	37905	37120	-	Transcription antitermination protein NusG	FIG00000269	icw(2);LSU_ribosomal_proteins_cluster isu;Transcription_factors_bacterial
fig|6666666.28482.peg.274	Contig_10	38398	37934	-	Preprotein translocase subunit SecE (TC 3.A.5.1.1)	FIG00001219	isu;LSU_ribosomal_proteins_cluster
fig|6666666.28482.rna.2	Contig_10	38569	38497	-	tRNA-Trp-CCA		isu;tRNAs
fig|6666666.28482.peg.275	Contig_10	39214	38714	-	FIG00820537: hypothetical protein	FIG00820536	ff
fig|6666666.28482.peg.276	Contig_10	39646	39218	-	(3R)-hydroxyacyl-ACP dehydratase subunit HadB		
fig|6666666.28482.peg.277	Contig_10	40112	39633	-	FIG00995223: hypothetical protein	FIG00821179	ff
fig|6666666.28482.peg.278	Contig_10	40305	40156	-	LSU ribosomal protein L33p @ LSU ribosomal protein L33p, zinc-dependent		icw(2);Ribosome_LSU_bacterial icw(2);Ribosome_LSU_bacterial
fig|6666666.28482.rna.3	Contig_10	40447	40374	-	tRNA-Met-CAT		
fig|6666666.28482.rna.4	Contig_10	40573	40501	-	tRNA-Thr-GGT		
fig|6666666.28482.peg.279	Contig_10	40904	40701	-	FIG00822280: hypothetical protein	FIG00822279	ff
fig|6666666.28482.peg.280	Contig_10	40991	41689	+	FIG00822416: hypothetical protein	FIG00822414	ff
fig|6666666.28482.peg.281	Contig_10	41785	42618	+	Possible membrane protein	FIG01121868	ff
fig|6666666.28482.peg.282	Contig_10	42642	43337	+	Enoyl-CoA hydratase	FIG01422808	ff
fig|6666666.28482.peg.283	Contig_10	43955	43464	-	Cyanate hydratase (EC 4.2.1.104)	FIG00095254	isu;Cyanate_hydrolysis
fig|6666666.28482.peg.284	Contig_10	44796	44041	-	FIG00833504: hypothetical protein	FIG00833502	ff

fig|6666666.28482.peg.285	Contig_10	45351	44818	-	FIG00830631: hypothetical protein	FIG00830630	ff
fig|6666666.28482.peg.286	Contig_10	45406	48693	+	Exodeoxyribonuclease V gamma chain (EC 3.1.11.5)	FIG00493440	isu;DNA-replication icw(1);DNA_repair,_bacterial_RecBCD_pathway
fig|6666666.28482.peg.287	Contig_10	48694	52005	+	Exodeoxyribonuclease V beta chain (EC 3.1.11.5)	FIG00043030	icw(1);DNA-replication isu;DNA_repair,_bacterial_RecBCD_pathway
fig|6666666.28482.peg.288	Contig_10	52002	53723	+	Exodeoxyribonuclease V alpha chain (EC 3.1.11.5)	FIG00047674	isu;CBSS-269801.1.peg.2186 icw(1);DNA-replication icw(1);DNA_repair,_bacterial_RecBCD_pathway
fig|6666666.28482.peg.289	Contig_10	53839	54546	+	Possible membrane protein	FIG00825285	ff
fig|6666666.28482.peg.290	Contig_10	56102	54552	-	MCE-family protein Mce1F	FIG00821171	idu(7);Mycobacterium_virulence_operon_MCE_involved_in_cell_invasion
fig|6666666.28482.peg.291	Contig_10	57248	56106	-	MCE-family lipoprotein LprK (MCE-family lipoprotein Mce1e)	FIG00819998	icw(2);Mycobacterium_virulence_operon_MCE_involved_in_cell_invasion
fig|6666666.28482.peg.292	Contig_10	58768	57311	-	MCE-family protein Mce1D	FIG00820669	icw(1);Mycobacterium_virulence_operon_MCE_involved_in_cell_invasion
fig|6666666.28482.peg.293	Contig_10	60189	58765	-	MCE-family protein Mce1C	FIG00821293	icw(7);Mycobacterium_virulence_operon_MCE_involved_in_cell_invasion
fig|6666666.28482.peg.294	Contig_10	61217	60186	-	MCE-family protein Mce1B	FIG00763551	icw(5);Mycobacterium_virulence_operon_MCE_involved_in_cell_invasion
fig|6666666.28482.peg.295	Contig_10	62389	61214	-	MCE-family protein Mce1A	FIG00820494	icw(2);Mycobacterium_virulence_operon_MCE_involved_in_cell_invasion

fig|6666666.28482.peg.296	Contig_10	63297	62443	-	Conserved hypothetical integral membrane protein YrbE1B	FIG00691621	icw(3);Mycobacterium_virulence_operon_MCE_involved_in_cell_invasion
fig|6666666.28482.peg.297	Contig_10	64111	63314	-	Conserved hypothetical integral membrane protein YrbE1A	FIG00690583	idu(7);Mycobacterium_virulence_operon_MCE_involved_in_cell_invasion
fig|6666666.28482.peg.298	Contig_10	64830	64108	-	Transcriptional regulator, GntR family	FIG00621925	ff
fig|6666666.28482.peg.299	Contig_10	65863	64922	-	FIG00829409: hypothetical protein	FIG00829406	ff
fig|6666666.28482.peg.300	Contig_10	66816	65866	-	FIG00820461: hypothetical protein	FIG00820460	ff
fig|6666666.28482.peg.301	Contig_10	66947	69334	+	Possible membrane protein	FIG01121868	ff
fig|6666666.28482.peg.302	Contig_10	71971	69341	-	Alpha-1,2-mannosidase	FIG00004078	isu;Mannose_Metabolism
fig|6666666.28482.peg.303	Contig_10	72127	72801	+	Lipoprotein LpqN	FIG01321357	ff
fig|6666666.28482.peg.304	Contig_10	73052	73363	+	FIG030357: hypothetical protein	FIG01326564	ff
fig|6666666.28482.peg.305	Contig_10	74027	73347	-	FIG00827459: hypothetical protein	FIG00827456	ff

fig|6666666.28482.peg.306	Contig_10	75140	74379	-	Enoyl-CoA hydratase (EC 4.2.1.17)	FIG00033206	idu(51);n-Phenylalkanoic_acid_degradation idu(51);Polyhydroxybutyrate_metabolism idu(51);Isoleucine_degradation idu(51);Fatty_acid_metabolism_cluster idu(51);Lysine_fermentation idu(51);Butanol_Biosynthesis idu(51);Acetyl-CoA_fermentation_to_Butyrate idu(51);Valine_degradation idu(51);Butyrate_metabolism_cluster
fig|6666666.28482.peg.307	Contig_10	76234	75137	-	Galactokinase (EC 2.7.1.6)	FIG00024073	icw(1);Lactose_and_Galactose_Uptake_and_Utilization
fig|6666666.28482.peg.308	Contig_10	77325	76231	-	Galactose-1-phosphate uridylyltransferase (EC 2.7.7.10)	FIG00623809	isu;Lactose_and_Galactose_Uptake_and_Utilization
fig|6666666.28482.peg.309	Contig_10	77461	79602	+	FIG00825533: hypothetical protein	FIG00825532	ff
fig|6666666.28482.peg.310	Contig_10	79626	79964	+	hypothetical protein		
fig|6666666.28482.peg.311	Contig_10	80215	79994	-	hypothetical protein		
fig|6666666.28482.peg.312	Contig_10	80371	81351	+	FIG00821847: hypothetical protein	FIG00821846	ff
fig|6666666.28482.peg.313	Contig_10	81375	82577	+	Multidrug resistance protein	FIG01352351	ff
fig|6666666.28482.peg.314	Contig_10	82711	83940	+	putative cytochrome P450 hydroxylase	FIG00032437	idu(49);Nitric_oxide_synthase

fig|6666666.28482.peg.315	Contig_10	85458	84016	-	6-aminohexanoate-cyclic-dimer hydrolase (EC 3.5.2.12)	FIG00627545	ff
fig|6666666.28482.peg.316	Contig_10	85707	85537	-	hypothetical protein		
fig|6666666.28482.peg.317	Contig_10	85736	86911	+	FIG00823762: hypothetical protein	FIG00823758	ff
fig|6666666.28482.peg.318	Contig_10	87944	86913	-	MaoC domain protein dehydratase		isu;CBSS-216591.1.peg.168
fig|6666666.28482.peg.319	Contig_10	88123	89046	+	Cobalt-zinc-cadmium resistance protein CzcD	FIG00000503	idu(1);CBSS-36873.1.peg.4752 idu(1);Cobalt-zinc-cadmium_resistance
fig|6666666.28482.peg.320	Contig_10	90335	89043	-	Manganese transport protein MntH	FIG00554286	ff
fig|6666666.28482.peg.321	Contig_10	90441	91244	+	carveol-like dehydrogenase		
fig|6666666.28482.rna.5	Contig_10	91513	91431	-	tRNA-Tyr-GTA		
fig|6666666.28482.peg.322	Contig_10	91582	92073	+	hypothetical protein		
fig|6666666.28482.peg.323	Contig_10	92156	93667	+	monooxygenase, flavin-binding family	FIG01386881	ff
fig|6666666.28482.peg.324	Contig_10	95184	96212	+	Glycerol-3-phosphate dehydrogenase [NAD(P)+] (EC 1.1.1.94)	FIG00000309	idu(1);Glycerol_and_Glycerol-3-phosphate_Uptake_and_Utilization idu(1);Glycerolipid_and_Glycerophospholipid_Metabolism_in_Bacteria
fig|6666666.28482.peg.325	Contig_10	96445	97944	+	putative transmembrane transport protein	FIG01126523	ff
fig|6666666.28482.peg.326	Contig_10	99373	97949	-	PE-PGRS FAMILY PROTEIN		
fig|6666666.28482.peg.327	Contig_10	100337	99483	-	Probable protease htpX homolog (EC 3.4.24.-)	FIG01385468	ff

fig|6666666.28482.peg.328	Contig_10	101398	100409	-	Octaprenyl-diphosphate synthase (EC 2.5.1.-) / Dimethylallyltransferase (EC 2.5.1.1) / Geranyltranstransferase (farnesyldiphosphate synthase) (EC 2.5.1.10) / Geranylgeranyl pyrophosphate synthetase (EC 2.5.1.29)	FIG00000019	isu;Isoprenoid_Biosynthesis
fig|6666666.28482.peg.329	Contig_10	101438	102658	+	Possible oxidoreductase (EC 1.-.-.-)	FIG00832005	ff
fig|6666666.28482.peg.330	Contig_10	102800	103477	+	Possible benzoquinone methyltransferase	FIG01318683	ff
fig|6666666.28482.peg.331	Contig_10	103537	103839	+	POSSIBLE CONSERVED SECRETED PROTEIN	FIG00996000	ff
fig|6666666.28482.peg.332	Contig_10	104531	103836	-	Ubiquinone/menaquinone biosynthesis methyltransferase UbiE (EC 2.1.1.-)	FIG01303154	idu(2);Menaquinone_and_Phylloquinone_Biosynthesis idu(2);Menaquinone_Biosynthesis_via_Futalosine idu(2);Ubiquinone_Biosynthesis
fig|6666666.28482.peg.333	Contig_10	105866	104550	-	FIG00825991: hypothetical protein	FIG00825990	ff
fig|6666666.28482.peg.334	Contig_10	107007	105856	-	FIG00821700: hypothetical protein	FIG00821699	ff
fig|6666666.28482.peg.335	Contig_10	107094	107561	+	transcriptional regulator	FIG00629921	ff

fig|6666666.28482.peg.336	Contig_10	107607	108431	+	oxidoreductase, short-chain dehydrogenase-reductase family	FIG01625937	ff
fig|6666666.28482.peg.337	Contig_10	109587	108433	-	Glycosyltransferase	FIG00007960	ff
fig|6666666.28482.peg.338	Contig_10	110159	109617	-	Possible membrane protein	FIG01121868	ff
fig|6666666.28482.peg.339	Contig_10	111790	110156	-	2-succinyl-5-enolpyruvyl-6-hydroxy-3-cyclohexene-1-carboxylic-acid synthase (EC 2.2.1.9)	FIG00000866	icw(1);Menaquinone_and_Phylloquinone_Biosynthesis_--_gjo icw(1);Menaquinone_and_Phylloquinone_Biosynthesis
fig|6666666.28482.peg.340	Contig_10	112626	111838	-	2-succinyl-6-hydroxy-2,4-cyclohexadiene-1-carboxylate synthase (EC 4.2.99.20)	FIG00016466	isu;Menaquinone_and_Phylloquinone_Biosynthesis_--_gjo isu;Menaquinone_and_Phylloquinone_Biosynthesis
fig|6666666.28482.peg.341	Contig_10	113594	112623	-	O-succinylbenzoate synthase (EC 4.2.1.113)	FIG01306622	isu;Muconate_lactonizing_enzyme_family icw(3);Menaquinone_and_Phylloquinone_Biosynthesis_--_gjo icw(3);Menaquinone_and_Phylloquinone_Biosynthesis
fig|6666666.28482.peg.342	Contig_10	115195	113591	-	Amidohydrolase family protein	FIG01334632	ff
fig|6666666.28482.peg.343	Contig_10	115263	116867	+	O-succinylbenzoic acid--CoA ligase (EC 6.2.1.26)	FIG00000860	icw(2);Menaquinone_and_Phylloquinone_Biosynthesis_--_gjo icw(2);Menaquinone_and_Phylloquinone_Biosynthesis
fig|6666666.28482.peg.344	Contig_10	117485	116937	-	Transcriptional regulator, TetR family	FIG00554417	ff
fig|6666666.28482.peg.345	Contig_10	117568	118455	+	FIG00823672: hypothetical protein	FIG00823670	ff

fig|6666666.28482.peg.346	Contig_10	118466	118921	+	FIG00819980: hypothetical protein	FIG00819977	ff
fig|6666666.28482.peg.347	Contig_10	118927	119619	+	hypothetical protein		
fig|6666666.28482.peg.348	Contig_10	119616	120518	+	Naphthoate synthase (EC 4.1.3.36)	FIG00071623	idu(2);Menaquinone_and_Phylloquinone_Biosynthesis_--_gjo idu(2);Menaquinone_and_Phylloquinone_Biosynthesis
fig|6666666.28482.peg.349	Contig_10	120601	121440	+	"Oxidoreductase, short-chain dehydrogenase/reductase family"	FIG01320732	ff
fig|6666666.28482.peg.350	Contig_10	121430	122056	+	FIG00832411: hypothetical protein	FIG00832410	ff
fig|6666666.28482.peg.351	Contig_10	122070	122459	+	FIG020377: hypothetical protein	FIG01001139	ff
fig|6666666.28482.peg.352	Contig_10	122575	123792	+	Probable low-affinity inorganic phosphate transporter	FIG00009601	isu;Phosphate_metabolism
fig|6666666.28482.peg.353	Contig_10	123789	124088	+	hypothetical protein		
fig|6666666.28482.peg.354	Contig_10	124136	124441	+	FIG00995255: hypothetical protein	FIG00820338	ff
fig|6666666.28482.peg.355	Contig_10	124486	125595	+	O-succinylbenzoic acid--CoA ligase (EC 6.2.1.26)	FIG00000860	icw(1);Menaquinone_and_Phylloquinone_Biosynthesis_--_gjo icw(1);Menaquinone_and_Phylloquinone_Biosynthesis
fig|6666666.28482.peg.356	Contig_10	125610	126962	+	Putative ESX-1 secretion system component Rv3877	FIG00092051	ff
fig|6666666.28482.peg.357	Contig_10	126974	127159	+	hypothetical protein		
fig|6666666.28482.peg.358	Contig_10	127904	127242	-	FIG00821404: hypothetical protein	FIG00821403	ff
fig|6666666.28482.peg.359	Contig_10	128557	127901	-	Glycosyltransferase (EC 2.4.1.-)	FIG01193496	ff

fig|6666666.28482.peg.360	Contig_10	130956	128572	-	interspersed repeat antigen, putative		
fig|6666666.28482.peg.361	Contig_100	199	1362	+	FIG00830647: hypothetical monooxygenase	FIG00830644	ff
fig|6666666.28482.peg.362	Contig_100	1943	2095	+	hypothetical protein		
fig|6666666.28482.peg.363	Contig_100	2598	2092	-	hypothetical protein		
fig|6666666.28482.peg.364	Contig_100	2833	3162	+	hypothetical protein		
fig|6666666.28482.peg.365	Contig_100	3678	3211	-	FIG00823285: hypothetical protein	FIG00823280	ff
fig|6666666.28482.peg.366	Contig_100	3807	4889	+	D-alanyl-D-alanine carboxypeptidase (EC 3.4.16.4)	FIG00561498	idu(3);Murein_Hydrolases idu(3);CBSS-84588.1.peg.1247 idu(3);Metallocarboxypeptidases_(EC _3.4.17.-) idu(3);Peptidoglycan_Biosynthesis
fig|6666666.28482.peg.367	Contig_100	6453	4897	-	Allophanate hydrolase (EC 3.5.1.54)	FIG00229246	isu;Urea_carboxylase_and_Allophanate_hydrolase_cluster
fig|6666666.28482.peg.368	Contig_100	7873	6467	-	Short chain fatty acids transporter	FIG00013994	isu;Polyhydroxybutyrate_metabolism
fig|6666666.28482.peg.369	Contig_100	8140	8877	+	3-oxoacyl-[acyl-carrier protein] reductase (EC 1.1.1.100)	FIG00621114	idu(38);CBSS-246196.1.peg.364 idu(38);CBSS-164757.7.peg.5020 idu(38);Fatty_Acid_Biosynthesis_FASII
fig|6666666.28482.peg.370	Contig_100	8899	9645	+	putative short-chain dehydrogenase	FIG01059210	ff
fig|6666666.28482.peg.371	Contig_100	9680	10423	+	hypothetical protein		
fig|6666666.28482.peg.372	Contig_100	10437	11414	+	RNA polymerase sigma-70 factor, family protein		
fig|6666666.28482.peg.373	Contig_101	2066	840	-	FIG00823053: hypothetical protein	FIG00823051	ff
fig|6666666.28482.peg.374	Contig_101	2223	2086	-	hypothetical protein		

fig|6666666.28482.peg.375	Contig_101	2222	2710	+	Transcriptional regulator, TetR family	FIG00554417	ff
fig|6666666.28482.peg.376	Contig_101	2856	3332	+	Bile acid 7-alpha dehydratase BaiE (EC 4.2.1.106)	FIG00774380	ff
fig|6666666.28482.peg.377	Contig_101	3975	3322	-	FIG019540: hypothetical protein	FIG01325408	ff
fig|6666666.28482.peg.378	Contig_101	4676	4047	-	hypothetical protein	FIG00638284	ff
fig|6666666.28482.peg.379	Contig_101	4710	5192	+	FIG00820335: hypothetical protein	FIG00820334	ff
fig|6666666.28482.peg.380	Contig_101	5460	5176	-	FIG00826871: hypothetical protein	FIG00826870	ff
fig|6666666.28482.peg.381	Contig_101	5459	6157	+	Cobalamin biosynthesis protein BluB @ 5,6-dimethylbenzimidazole synthase, flavin destructase family	FIG00001607	isu;Cobalamin_synthesis isu;Coenzyme_B12_biosynthesis
fig|6666666.28482.peg.382	Contig_101	6230	7594	+	probable sulfatase atsG	FIG01736791	ff
fig|6666666.28482.peg.383	Contig_101	8383	7601	-	Trans-aconitate 2-methyltransferase (EC 2.1.1.144)	FIG00626701	ff
fig|6666666.28482.peg.384	Contig_101	8465	9679	+	FIG00821889: hypothetical protein	FIG00821888	ff
fig|6666666.28482.peg.385	Contig_101	10750	9752	-	ESX-3 secretion system protein EccE3	FIG01337146	ff
fig|6666666.28482.peg.386	Contig_101	12150	10747	-	Type VII secretion-associated serine protease mycosin MycP3	FIG01341840	ff
fig|6666666.28482.peg.387	Contig_101	13556	12132	-	ESX-3 secretion system protein EccD3	FIG01324346	ff

fig|6666666.28482.peg.388	Contig_101	14492	13611	-	RD1 region associated protein Rv3866	FIG00029355	ff
fig|6666666.28482.peg.389	Contig_101	14803	14513	-	10 kDa antigen CFP7	FIG01333717	ff
fig|6666666.28482.peg.390	Contig_101	15128	14835	-	PE family protein	FIG01311212	ff
fig|6666666.28482.peg.391	Contig_101	15374	15180	-	PPE family protein	FIG00013075	idu(57);CBSS-164757.7.peg.5020
fig|6666666.28482.peg.392	Contig_102	33	3659	+	Metal cation transporting P-type ATPase CtpH	FIG01340869	ff
fig|6666666.28482.peg.393	Contig_102	3721	3996	+	FIG00821936: hypothetical protein	FIG00821935	ff
fig|6666666.28482.peg.394	Contig_102	7792	4001	-	FIG00829714: hypothetical protein	FIG00829710	ff
fig|6666666.28482.peg.395	Contig_102	8172	7789	-	anti sigma factor antagonist	FIG01125797	ff
fig|6666666.28482.peg.396	Contig_102	8933	8253	-	FIG00828626: hypothetical protein	FIG00828625	ff
fig|6666666.28482.peg.397	Contig_102	9103	10734	+	Hydroxymethylpyrimidine phosphate synthase ThiC	FIG01958823	ff
fig|6666666.28482.peg.398	Contig_102	10731	11558	+	Hydroxymethylpyrimidine phosphate kinase ThiD (EC 2.7.4.7)	FIG01958950	ff
fig|6666666.28482.peg.399	Contig_102	11608	12231	+	FIG022825: hypothetical protein	FIG00828299	ff
fig|6666666.28482.peg.400	Contig_102	12262	12465	+	hypothetical protein		
fig|6666666.28482.peg.401	Contig_102	12521	12955	+	Transcriptional regulator, MarR family	FIG00554610	ff
fig|6666666.28482.peg.402	Contig_102	12975	13844	+	FIG00828275: hypothetical protein	FIG00828264	ff
fig|6666666.28482.peg.403	Contig_102	15304	13847	-	Lipoprotein peptidase LpqM	FIG01332312	ff
fig|6666666.28482.peg.404	Contig_102	15536	15652	+	hypothetical protein		

fig|6666666.28482.peg.405	Contig_103	716	393	-	Preprotein translocase subunit YajC (TC 3.A.5.1.1)	FIG00051656	ff
fig|6666666.28482.peg.406	Contig_103	893	2242	+	Gamma-aminobutyrate:alpha-ketoglutarate aminotransferase (EC 2.6.1.19)	FIG00006767	isu;Putrescine_utilization_pathways
fig|6666666.28482.peg.407	Contig_103	2375	5851	+	Long-chain-fatty-acid--CoA ligase (EC 6.2.1.3)	FIG00018699	idu(72);n-Phenylalkanoic_acid_degradation idu(72);Fatty_acid_metabolism_cluster idu(72);Biotin_synthesis_cluster idu(72);Biotin_biosynthesis
fig|6666666.28482.peg.408	Contig_103	6554	5916	-	FIG00825396: hypothetical protein	FIG00825394	ff
fig|6666666.28482.peg.409	Contig_103	7105	6719	-	FIG00822359: hypothetical protein	FIG00822353	ff
fig|6666666.28482.peg.410	Contig_103	7865	7215	-	FIG00825396: hypothetical protein	FIG00825394	ff
fig|6666666.28482.peg.411	Contig_103	9006	7957	-	Holliday junction DNA helicase RuvB	FIG00000352	icw(2);DNA-replication icw(2);RuvABC_plus_a_hypothetical
fig|6666666.28482.peg.412	Contig_103	9596	9003	-	Holliday junction DNA helicase RuvA	FIG00000314	isu;DNA-replication icw(1);RuvABC_plus_a_hypothetical
fig|6666666.28482.peg.413	Contig_103	10162	9593	-	Crossover junction endodeoxyribonuclease RuvC (EC 3.1.22.4)	FIG00095112	icw(1);DNA-replication isu;RuvABC_plus_a_hypothetical
fig|6666666.28482.peg.414	Contig_103	10305	10916	+	Possible membrane protein	FIG01121868	ff

fig|6666666.28482.peg.415	Contig_103	10963	11430	+	FIG00993990: hypothetical protein	FIG00820419	ff
fig|6666666.28482.peg.416	Contig_103	11427	11858	+	Possible membrane protein	FIG01121868	ff
fig|6666666.28482.peg.417	Contig_103	11898	12353	+	hypothetical protein		
fig|6666666.28482.peg.418	Contig_103	12350	13885	+	Spermidine synthase (EC 2.5.1.16)	FIG00000642	isu;Polyamine_Metabolism
fig|6666666.28482.peg.419	Contig_103	14496	13882	-	SAM-dependent methyltransferase (EC 2.1.1.-)	FIG00509796	ff
fig|6666666.28482.peg.420	Contig_103	15265	14510	-	FIG000859: hypothetical protein	FIG00000859	ff
fig|6666666.28482.peg.421	Contig_103	16093	15497	-	Pyridoxine biosynthesis glutamine amidotransferase, glutaminase subunit (EC 2.4.2.-)	FIG00001203	icw(1);Pyridoxin_(Vitamin_B6)_Biosynthesis
fig|6666666.28482.peg.422	Contig_103	16946	16101	-	Acyl-CoA thioesterase II (EC 3.1.2.-)	FIG00002217	idu(3);Acyl-CoA_thioesterase_II
fig|6666666.28482.peg.423	Contig_103	17870	16956	-	Pyridoxine biosynthesis glutamine amidotransferase, synthase subunit (EC 2.4.2.-)	FIG00021445	isu;Pyridoxin_(Vitamin_B6)_Biosynthesis
fig|6666666.28482.peg.424	Contig_104	61	540	+	hypothetical protein	FIG00638284	ff
fig|6666666.28482.peg.425	Contig_104	1132	590	-	FIG00820972: hypothetical protein	FIG00820970	ff
fig|6666666.28482.peg.426	Contig_104	1678	1145	-	hypothetical protein		
fig|6666666.28482.peg.427	Contig_104	2573	1692	-	Catechol 1,2-dioxygenase 1 (EC 1.13.11.1)	FIG00007171	isu;Catechol_branch_of_beta-ketoadipate_pathway

fig|6666666.28482.peg.428	Contig_104	3585	2614	-	Alcohol dehydrogenase, zinc-binding		
fig|6666666.28482.peg.429	Contig_104	4388	3723	-	FIG002283: Isochorismatase family protein	FIG00002283	ff
fig|6666666.28482.peg.430	Contig_104	6346	4451	-	Exoenzymes regulatory protein AepA precursor	FIG00002517	ff
fig|6666666.28482.peg.431	Contig_104	7697	6684	-	hypothetical protein	FIG00638284	ff
fig|6666666.28482.peg.432	Contig_104	8686	7700	-	lipase		
fig|6666666.28482.peg.433	Contig_104	10303	8711	-	3-polyprenyl-4-hydroxybenzoate carboxy-lyase (EC 4.1.1.-)	FIG00000548	icw(1);Ubiquinone_Biosynthesis
fig|6666666.28482.peg.434	Contig_104	10998	10363	-	3-polyprenyl-4-hydroxybenzoate carboxy-lyase UbiX (EC 4.1.1.-)	FIG00000573	isu;Ubiquinone_Biosynthesis
fig|6666666.28482.peg.435	Contig_104	12473	11988	-	cupin domain protein		
fig|6666666.28482.peg.436	Contig_104	13221	12517	-	putative oxidoreductase		
fig|6666666.28482.peg.437	Contig_104	13889	13239	-	hypothetical protein		
fig|6666666.28482.peg.438	Contig_104	14697	14008	-	putative esterase		idu(2);Alpha-Amylase_locus_in_Streptocococcus
fig|6666666.28482.peg.439	Contig_104	14968	14792	-	hypothetical protein		
fig|6666666.28482.peg.440	Contig_104	15770	15120	-	FIG00822234: hypothetical protein	FIG00822233	ff
fig|6666666.28482.peg.441	Contig_104	16176	15946	-	FIG00822234: hypothetical protein		
fig|6666666.28482.peg.442	Contig_104	16518	18152	+	hypothetical protein	FIG00638284	ff
fig|6666666.28482.peg.443	Contig_104	18156	18440	+	hypothetical protein		

fig|6666666.28482.peg.444	Contig_104	18986	18552	-	regulatory protein		
fig|6666666.28482.peg.445	Contig_104	19100	19609	+	FIG00820972: hypothetical protein	FIG00820970	ff
fig|6666666.28482.peg.446	Contig_104	19832	20806	+	Transcriptional regulator containing an amidase domain and an AraC-type DNA-binding HTH domain	FIG00003657	ff
fig|6666666.28482.peg.447	Contig_104	20831	21847	+	FIG00828378: hypothetical protein	FIG00828377	ff
fig|6666666.28482.peg.448	Contig_104	23051	21867	-	hypothetical protein	FIG00638284	ff
fig|6666666.28482.peg.449	Contig_104	24840	23206	-	acetolactate synthase large subunit		
fig|6666666.28482.peg.450	Contig_104	25639	24980	-	hypothetical protein	FIG00638284	ff
fig|6666666.28482.peg.451	Contig_104	28723	25718	-	INTEGRAL MEMBRANE PROTEIN (Rhomboid family)	FIG00525068	ff
fig|6666666.28482.peg.452	Contig_104	28822	29328	+	hypothetical protein	FIG00638284	ff
fig|6666666.28482.peg.453	Contig_104	30347	29325	-	Lon-like protease with PDZ domain	FIG00051666	ff
fig|6666666.28482.peg.454	Contig_104	30443	31807	+	Collagen alpha 1(I) chain precursor	FIG00672327	ff
fig|6666666.28482.peg.455	Contig_104	31904	32623	+	hypothetical protein		
fig|6666666.28482.peg.456	Contig_104	32842	33639	+	hypothetical protein	FIG00638284	ff
fig|6666666.28482.peg.457	Contig_104	33695	33958	+	hypothetical protein		
fig|6666666.28482.peg.458	Contig_104	34509	34003	-	PE family protein		
fig|6666666.28482.peg.459	Contig_104	34777	35637	+	FIG00822196: hypothetical protein	FIG00822195	ff
fig|6666666.28482.peg.460	Contig_104	35715	37043	+	Ubiquinone biosynthesis monooxygenase UbiB	FIG00000320	idu(1);Ubiquinone_Biosynthesis

fig|6666666.28482.peg.461	Contig_104	37356	37120	-	WhiB-family transcriptional regulator	FIG00027266	isu;WhiB_and_WhiB-type_regulatory_proteins_
fig|6666666.28482.peg.462	Contig_104	37684	37523	-	hypothetical protein		
fig|6666666.28482.peg.463	Contig_104	39927	37786	-	ATP-dependent DNA helicase UvrD/PcrA, actinomycete paralog	FIG00004012	isu;DNA_repair,_bacterial_UvrD_and_related_helicases
fig|6666666.28482.peg.464	Contig_104	40056	40301	+	POSSIBLE GLUTAREDOXIN PROTEIN	FIG00661781	ff
fig|6666666.28482.peg.465	Contig_104	41231	40308	-	NADH pyrophosphatase (EC 3.6.1.22)	FIG00000911	isu;Nudix_proteins_(nucleoside_triphosphate_hydrolases)
fig|6666666.28482.peg.466	Contig_104	42308	41238	-	Potassium channel protein	FIG00006661	idu(2);Potassium_homeostasis
fig|6666666.28482.peg.467	Contig_104	44257	42314	-	ATP-dependent DNA helicase SCO5184	FIG00137374	isu;DNA_repair,_bacterial_RecBCD_pathway
fig|6666666.28482.peg.468	Contig_105	609	887	+	FIG025441: hypothetical protein	FIG00824290	ff
fig|6666666.28482.peg.469	Contig_105	1016	1483	+	FIG00818182: hypothetical protein	FIG00672167	ff
fig|6666666.28482.peg.470	Contig_105	1480	2988	+	Serine/threonine phosphatase PPP (EC 3.1.3.16)	FIG00741411	ff
fig|6666666.28482.peg.471	Contig_105	2985	4394	+	Cell division protein FtsW	FIG00000025	icw(1);Bacterial_Cell_Division icw(1);Bacterial_Cytoskeleton

fig|6666666.28482.peg.472	Contig_105	4391	5869	+	Cell division protein FtsI [Peptidoglycan synthetase] (EC 2.4.1.129)	FIG00046929	idu(2);Bacterial_Cell_Division idu(2);16S_rRNA_modification_within_P_site_of_ribosome idu(2);Flagellum_in_Campylobacter idu(2);Bacterial_Cytoskeleton idu(2);Peptidoglycan_Biosynthesis
fig|6666666.28482.peg.473	Contig_106	2357	879	-	hypothetical protein		
fig|6666666.28482.peg.474	Contig_106	3901	2354	-	chain length determinant protein		
fig|6666666.28482.peg.475	Contig_106	5020	3905	-	FIG00824412: hypothetical protein	FIG00824410	ff
fig|6666666.28482.peg.476	Contig_106	6114	5017	-	methyltransferase, putative	FIG00883498	ff
fig|6666666.28482.peg.477	Contig_106	7352	6111	-	methyltransferase, putative	FIG00883498	ff
fig|6666666.28482.peg.478	Contig_106	8628	7333	-	hypothetical protein	FIG00638284	ff
fig|6666666.28482.peg.479	Contig_106	10799	8781	-	FIG00827174: hypothetical protein	FIG00827173	ff
fig|6666666.28482.peg.480	Contig_106	12274	10796	-	FIG00829077: hypothetical protein	FIG00829076	ff
fig|6666666.28482.peg.481	Contig_106	13779	12274	-	Undecaprenyl-phosphate galactosephosphotransferase (EC 2.7.8.6)	FIG00002968	isu;Exopolysaccharide_Biosynthesis
fig|6666666.28482.peg.482	Contig_106	14642	13797	-	teichoic acid biosynthesis protein	FIG00828655	ff

fig|6666666.28482.peg.483	Contig_106	15242	14685	-	dTDP-4-dehydrorhamnose 3,5-epimerase (EC 5.1.3.13)	FIG00000408	icw(1);dTDP-rhamnose_synthesis icw(1);Rhamnose_containing_glycans idu(1);Capsular_heptose_biosynthesis
fig|6666666.28482.peg.484	Contig_106	16285	15239	-	UDP-glucose 4-epimerase (EC 5.1.3.2)	FIG00022300	idu(4);Rhamnose_containing_glycans idu(4);Lactose_and_Galactose_Uptake_and_Utilization idu(4);N-linked_Glycosylation_in_Bacteria idu(4);Lacto-N-Biose_I_and_Galacto-N-Biose_Metabolic_Pathway
fig|6666666.28482.peg.485	Contig_106	16972	16310	-	FIG01131441: hypothetical protein	FIG01131440	ff
fig|6666666.28482.peg.486	Contig_106	17772	16969	-	Glucose-1-phosphate cytidylyltransferase (EC 2.7.7.33)	FIG00132976	isu;dTDP-rhamnose_synthesis
fig|6666666.28482.peg.487	Contig_106	19078	17849	-	PPE family protein	FIG00013075	idu(57);CBSS-164757.7.peg.5020
fig|6666666.28482.peg.488	Contig_106	19463	19606	+	hypothetical protein		
fig|6666666.28482.peg.489	Contig_106	19603	20280	+	Transcriptional regulator, TetR family	FIG00554417	ff
fig|6666666.28482.peg.490	Contig_106	20413	21141	+	Transcriptional regulator, TetR family	FIG00554417	ff
fig|6666666.28482.peg.491	Contig_106	21565	21230	-	FIG00821141: hypothetical protein	FIG00821140	ff
fig|6666666.28482.peg.492	Contig_106	21822	21646	-	hypothetical protein		
fig|6666666.28482.peg.493	Contig_106	23360	21819	-	Methionyl-tRNA synthetase (EC 6.1.1.10)	FIG01265301	isu;tRNA_aminoacylation,_Met

fig|6666666.28482.peg.494	Contig_106	23379	24242	+	Putative deoxyribonuclease YcfH	FIG00000184	isu;YcfH
fig|6666666.28482.peg.495	Contig_106	24412	25539	+	Cell wall-binding protein	FIG00615230	ff
fig|6666666.28482.peg.496	Contig_106	25512	26450	+	Dimethyladenosine transferase (EC 2.1.1.-)	FIG00000225	ff
fig|6666666.28482.peg.497	Contig_106	26466	27461	+	4-diphosphocytidyl-2-C-methyl-D-erythritol kinase (EC 2.7.1.148)	FIG00000377	isu;Nonmevalonate_Branch_of_Isoprenoid_Biosynthesis isu;Isoprenoid_Biosynthesis
fig|6666666.28482.peg.498	Contig_106	27718	29352	+	Long-chain-fatty-acid--CoA ligase (EC 6.2.1.3)	FIG00018699	idu(72);n-Phenylalkanoic_acid_degradation idu(72);Fatty_acid_metabolism_cluster idu(72);Biotin_synthesis_cluster idu(72);Biotin_biosynthesis
fig|6666666.28482.peg.499	Contig_106	30047	29439	-	Transcriptional regulator, TetR family	FIG00554417	ff
fig|6666666.28482.peg.500	Contig_106	30126	31745	+	Beta-carotene ketolase (EC 1.14.-.-)	FIG00071345	idu(3);Proteorhodopsin
fig|6666666.28482.peg.501	Contig_106	31742	32071	+	ferredoxin		
fig|6666666.28482.peg.502	Contig_106	32068	32979	+	3-oxoacyl-[acyl-carrier protein] reductase (EC 1.1.1.100)	FIG00621114	idu(38);CBSS-246196.1.peg.364 icw(1);CBSS-164757.7.peg.5020 idu(38);Fatty_Acid_Biosynthesis_FASII

fig|6666666.28482.peg.503	Contig_106	33574	32999	-	Peptidyl-tRNA hydrolase (EC 3.1.1.29)	FIG00000151	icw(2);Transcription_repair_cluster isu;Cell_division-ribosomal_stress_proteins_cluster isu;Sporulation-associated_proteins_with_broader_functions isu;Translation_termination_factors_bacterial
fig|6666666.28482.peg.504	Contig_106	34246	33587	-	LSU ribosomal protein L25p	FIG00001896	isu;Ribosome_LSU_bacterial icw(5);Transcription_repair_cluster
fig|6666666.28482.peg.505	Contig_106	35538	34657	-	Short-chain dehydrogenase/reductase SDR	FIG00546160	idu(13);CBSS-164757.7.peg.5020 idu(13);Transcription_repair_cluster
fig|6666666.28482.peg.506	Contig_106	36200	35571	-	lipoprotein, putative	FIG00947911	ff
fig|6666666.28482.peg.507	Contig_106	36625	36281	-	Arsenate reductase (EC 1.20.4.1)	FIG00000770	icw(4);Transcription_repair_cluster idu(5);CBSS-315749.4.peg.3658 idu(5);Arsenic_resistance idu(5);Anaerobic_respiratory_reductases
fig|6666666.28482.peg.508	Contig_106	37606	36626	-	Ribose-phosphate pyrophosphokinase (EC 2.7.6.1)	FIG00096830	icw(2);Transcription_repair_cluster isu;Pentose_phosphate_pathway isu;De_Novo_Purine_Biosynthesis

fig|6666666.28482.peg.509	Contig_106	39182	37704	-	N-acetylglucosamine-1-phosphate uridyltransferase (EC 2.7.7.23) / Glucosamine-1-phosphate N-acetyltransferase (EC 2.3.1.157)	FIG00000353	icw(1);Transcription_repair_cluster icw(4);Transcription_repair_cluster isu;Sialic_Acid_Metabolism isu;Sialic_Acid_Metabolism isu;UDP-N-acetylmuramate_from_Fructose-6-phosphate_Biosynthesis isu;UDP-N-acetylmuramate_from_Fructose-6-phosphate_Biosynthesis isu;Peptidoglycan_Biosynthesis isu;Peptidoglycan_Biosynthesis
fig|6666666.28482.rna.6	Contig_106	39355	39284	-	tRNA-Gln-TTG		
fig|6666666.28482.peg.510	Contig_106	39412	40092	+	Transcriptional regulator, TetR family	FIG00554417	ff
fig|6666666.28482.peg.511	Contig_106	40992	40099	-	putative oxidoreductase, short-chain dehydrogenase/reductase family		
fig|6666666.28482.peg.512	Contig_106	42041	40995	-	PROBABLE OXIDOREDUCTASE	FIG01416034	ff
fig|6666666.28482.peg.513	Contig_106	42646	42056	-	Transcriptional regulator, TetR family	FIG00554417	ff
fig|6666666.28482.peg.514	Contig_106	42782	44044	+	hypothetical protein	FIG00638284	ff
fig|6666666.28482.peg.515	Contig_106	44041	45201	+	hypothetical protein	FIG00638284	ff
fig|6666666.28482.peg.516	Contig_106	46548	45208	-	Diaminopimelate decarboxylase (EC 4.1.1.20)	FIG00138250	idu(2);CBSS-323850.3.peg.3269 idu(2);Lysine_Biosynthesis_DAP_Pathway,_GJO_scratch idu(2);Lysine_Biosynthesis_DAP_Pathway

fig|6666666.28482.peg.517	Contig_106	46656	50318	+	Transcription-repair coupling factor	FIG00067681	isu;Transcription_repair_cluster isu;DNA-replication isu;Cell_division-ribosomal_stress_proteins_cluster isu;Transcription_factors_bacterial
fig|6666666.28482.peg.518	Contig_106	50368	51414	+	Nucleoside triphosphate pyrophosphohydrolase MazG (EC 3.6.1.8)	FIG00002974	isu;Nucleoside_triphosphate_pyrophosphohydrolase_MazG
fig|6666666.28482.peg.519	Contig_106	51744	51586	-	hypothetical protein		
fig|6666666.28482.peg.520	Contig_106	51737	52327	+	FIG00997322: hypothetical protein	FIG00997321	ff
fig|6666666.28482.peg.521	Contig_106	52372	53661	+	Enolase (EC 4.2.1.11)	FIG00000118	isu;Serine-glyoxylate_cycle
fig|6666666.28482.peg.522	Contig_106	53787	54293	+	Cell division protein DivIC (FtsB), stabilizes FtsL against RasP cleavage	FIG00485194	icw(1);Possible_new_toxin-antitoxin_system_including_DivIC idu(10);Bacterial_Cell_Division idu(10);Cell_division-ribosomal_stress_proteins_cluster idu(10);Bacterial_Cytoskeleton idu(10);Stationary_phase_repair_cluster
fig|6666666.28482.peg.523	Contig_106	54286	54774	+	FIG004853: possible toxin to DivIC		isu;Possible_new_toxin-antitoxin_system_including_DivIC
fig|6666666.28482.peg.524	Contig_106	54768	55712	+	Exopolyphosphatase (EC 3.6.1.11)	FIG00066575	idu(2);Polyphosphate idu(2);Phosphate_metabolism
fig|6666666.28482.peg.525	Contig_107	538	71	-	Lipoprotein LppP	FIG00820809	ff
fig|6666666.28482.peg.526	Contig_107	831	5006	+	Assimilatory nitrate reductase large subunit (EC:1.7.99.4)	FIG00059898	idu(1);Nitrate_and_nitrite_ammonification

fig|6666666.28482.peg.527	Contig_107	5208	5384	+	hypothetical protein		
fig|6666666.28482.peg.528	Contig_107	5814	5374	-	protein of unknown function DUF417		
fig|6666666.28482.peg.529	Contig_107	6353	5871	-	hypothetical protein		
fig|6666666.28482.peg.530	Contig_107	6982	6416	-	hypothetical protein		
fig|6666666.28482.peg.531	Contig_107	7106	7681	+	Transcriptional regulator, TetR family		
fig|6666666.28482.peg.532	Contig_107	8254	7697	-	hypothetical protein	FIG00638284	ff
fig|6666666.28482.peg.533	Contig_107	8344	9279	+	protein of unknown function DUF6, transmembrane		
fig|6666666.28482.peg.534	Contig_107	9345	10991	+	NAD-dependent malic enzyme (EC 1.1.1.38)	FIG00000835	isu;Pyruvate_metabolism_I:_anaplerotic_reactions,_PEP
fig|6666666.28482.peg.535	Contig_107	11489	10995	-	FIG00822322: hypothetical protein	FIG00822320	ff
fig|6666666.28482.peg.536	Contig_107	12076	11486	-	MCE associated membrane protein	FIG00820195	ff
fig|6666666.28482.peg.537	Contig_107	12618	12073	-	FIG00821219: hypothetical protein	FIG00821218	ff
fig|6666666.28482.peg.538	Contig_107	14114	12636	-	MCE-family protein Mce1F	FIG00821171	idu(7);Mycobacterium_virulence_operon_MCE_involved_in_cell_invasion
fig|6666666.28482.peg.539	Contig_107	15223	14114	-	MCE-family lipoprotein LprK (MCE-family lipoprotein Mce1e)	FIG00819998	icw(3);Mycobacterium_virulence_operon_MCE_involved_in_cell_invasion
fig|6666666.28482.peg.540	Contig_107	16572	15232	-	MCE-family protein Mce1D	FIG00820669	icw(1);Mycobacterium_virulence_operon_MCE_involved_in_cell_invasion
fig|6666666.28482.peg.541	Contig_107	17741	16569	-	MCE-family protein Mce1C	FIG00821293	icw(7);Mycobacterium_virulence_operon_MCE_involved_in_cell_invasion

fig|6666666.28482.peg.542	Contig_107	18754	17738	-	MCE-family protein Mce1B	FIG00763551	icw(6);Mycobacterium_virulence_operon_MCE_involved_in_cell_invasion
fig|6666666.28482.peg.543	Contig_107	20034	18766	-	MCE-family protein Mce1A	FIG00820494	icw(2);Mycobacterium_virulence_operon_MCE_involved_in_cell_invasion
fig|6666666.28482.peg.544	Contig_107	20887	20039	-	Conserved hypothetical integral membrane protein YrbE1B	FIG00691621	icw(3);Mycobacterium_virulence_operon_MCE_involved_in_cell_invasion
fig|6666666.28482.peg.545	Contig_107	21651	20884	-	Conserved hypothetical integral membrane protein YrbE1A	FIG00690583	idu(7);Mycobacterium_virulence_operon_MCE_involved_in_cell_invasion
fig|6666666.28482.peg.546	Contig_107	23773	21884	-	Cysteine desulfurase (EC 2.8.1.7)	FIG00000001	idu(2);Iron-sulfur_cluster_assembly idu(2);CBSS-84588.1.peg.1247 idu(2);CBSS-393130.3.peg.794 idu(2);mnm5U34_biosynthesis_bacteria idu(2);Alanine_biosynthesis
fig|6666666.28482.peg.547	Contig_107	24692	23760	-	Major membrane protein I	FIG01153610	ff
fig|6666666.28482.peg.548	Contig_107	25082	26359	+	Unknown function. Previously sequenced as TR:O32977 (EMBL:Z98741).	FIG01328012	ff
fig|6666666.28482.peg.549	Contig_107	26369	27301	+	Cysteine synthase (EC 2.5.1.47)	FIG00064110	icw(1);Cysteine_Biosynthesis
fig|6666666.28482.peg.550	Contig_107	27304	27987	+	Serine acetyltransferase (EC 2.3.1.30)	FIG00000128	isu;Cysteine_Biosynthesis isu;Conserved_gene_cluster_possibly_involved_in_RNA_metabolism

fig|6666666.28482.peg.551	Contig_107	28233	28514	+	hypothetical protein		
fig|6666666.28482.peg.552	Contig_107	28801	28502	-	Muconolactone isomerase (EC 5.3.3.4)	FIG00002342	isu;Catechol_branch_of_beta-ketoadipate_pathway
fig|6666666.28482.rna.7	Contig_107	28956	28884	-	tRNA-Asn-GTT		
fig|6666666.28482.peg.553	Contig_107	29108	29386	+	FIG00820230: hypothetical protein	FIG00820227	ff
fig|6666666.28482.peg.554	Contig_107	31295	29361	-	DNA primase (EC 2.7.7.-)	FIG00019251	isu;CBSS-349161.4.peg.2417 isu;DNA-replication
fig|6666666.28482.peg.555	Contig_107	31922	31323	-	Superoxide dismutase [Cu-Zn] precursor (EC 1.15.1.1)	FIG00001181	idu(2);Oxidative_stress idu(2);Protection_from_Reactive_Oxygen_Species
fig|6666666.28482.peg.556	Contig_107	33201	31933	-	Deoxyguanosinetriphosphate triphosphohydrolase (EC 3.1.5.1)	FIG00023004	ff
fig|6666666.28482.peg.557	Contig_107	33242	35245	+	Possible membrane protein	FIG01121868	ff
fig|6666666.28482.peg.558	Contig_107	35285	35404	+	hypothetical protein		
fig|6666666.28482.peg.559	Contig_108	349	50	-	PE family protein	FIG01313150	ff
fig|6666666.28482.peg.560	Contig_109	1946	165	-	Long-chain fatty-acid-CoA ligase (EC 6.2.1.3), Mycobacterial subgroup FadD6	FIG01953996	ff
fig|6666666.28482.peg.561	Contig_109	2537	1995	-	Lysine decarboxylase family	FIG01349038	ff
fig|6666666.28482.peg.562	Contig_11	2947	1532	-	PE family protein		
fig|6666666.28482.peg.563	Contig_11	4288	3236	-	PE-PGRS family protein		idu(16);PPE_gene_cluster_in_Mycobacteria
fig|6666666.28482.peg.564	Contig_11	6149	4623	-	hypothetical protein	FIG00638284	ff
fig|6666666.28482.peg.565	Contig_11	8045	6495	-	hypothetical protein	FIG00638284	ff
fig|6666666.28482.peg.566	Contig_11	9747	8326	-	hypothetical protein	FIG00638284	ff

fig|6666666.28482.peg.567	Contig_11	12341	9855	-	Phenylalanyl-tRNA synthetase beta chain (EC 6.1.1.20)	FIG00000116	icw(1);tRNA_aminoacylation,_Phe
fig|6666666.28482.peg.568	Contig_11	13384	12341	-	Phenylalanyl-tRNA synthetase alpha chain (EC 6.1.1.20)	FIG00000098	isu;tRNA_aminoacylation,_Phe
fig|6666666.28482.peg.569	Contig_11	14315	13509	-	Possible membrane protein	FIG01121868	ff
fig|6666666.28482.peg.570	Contig_11	15257	14334	-	Purine cyclase-related protein	FIG00026664	isu;cAMP_signaling_in_bacteria
fig|6666666.28482.peg.571	Contig_11	16278	15376	-	PE FAMILY PROTEIN		
fig|6666666.28482.peg.572	Contig_11	16523	17581	+	FIG00820092: hypothetical protein	FIG00820090	ff
fig|6666666.28482.peg.573	Contig_11	18299	17595	-	PE-PGRS FAMILY PROTEIN [FIRST PART]		
fig|6666666.28482.peg.574	Contig_11	19241	18456	-	FIG011178: rRNA methylase	FIG01289107	isu;RNA_methylation
fig|6666666.28482.peg.575	Contig_11	19637	19251	-	LSU ribosomal protein L20p	FIG00000216	icw(1);Ribosome_LSU_bacterial icw(2);Mycobacterium_virulence_operon_involved_in_protein_synthesis_(LSU_ribosomal_proteins)
fig|6666666.28482.peg.576	Contig_11	19899	19705	-	LSU ribosomal protein L35p	FIG00000259	isu;Ribosome_LSU_bacterial isu;Mycobacterium_virulence_operon_involved_in_protein_synthesis_(LSU_ribosomal_proteins)
fig|6666666.28482.peg.577	Contig_11	20497	19973	-	Translation initiation factor 3	FIG00000206	isu;Translation_initiation_factors_bacterial icw(1);Mycobacterium_virulence_operon_involved_in_protein_synthesis_(LSU_ribosomal_proteins)

fig|6666666.28482.peg.578	Contig_11	20987	24322	+	Putative membrane protein found fused to lysyl-tRNA synthetase like protein / Lysyl-tRNA synthetase (class II) related protein found fused to membrane protein	FIG00009947	isu;tRNA_aminoacylation,_Lys isu;tRNA_aminoacylation,_Lys
fig|6666666.28482.peg.579	Contig_11	24471	24319	-	hypothetical protein		
fig|6666666.28482.peg.580	Contig_11	24466	25875	+	FIG00824695: hypothetical protein	FIG01366795	ff
fig|6666666.28482.peg.581	Contig_11	26560	25856	-	Dienelactone hydrolase family	FIG01069253	ff
fig|6666666.28482.peg.582	Contig_11	26705	27355	+	Transcriptional regulator, TetR family	FIG00554417	ff
fig|6666666.28482.peg.583	Contig_11	27384	29546	+	POSSIBLE MOLYBDOPTERIN BIOSYNTHESIS PROTEIN MOEY	FIG00824264	ff
fig|6666666.28482.peg.584	Contig_11	30421	29552	-	hypothetical protein	FIG00638284	ff
fig|6666666.28482.peg.585	Contig_11	30630	32000	+	putative cytochrome P450	FIG00866649	ff
fig|6666666.28482.peg.586	Contig_11	32515	34422	+	FIG00822590: hypothetical protein	FIG01753111	ff
fig|6666666.28482.peg.587	Contig_11	35152	34430	-	FIG00822093: hypothetical protein	FIG00822089	ff
fig|6666666.28482.peg.588	Contig_11	35967	35251	-	FIG00826287: hypothetical protein	FIG00826286	ff
fig|6666666.28482.peg.589	Contig_11	36731	37009	+	hypothetical protein	FIG00638284	ff
fig|6666666.28482.peg.590	Contig_11	37019	37258	+	FIG00820127: hypothetical protein	FIG00820126	ff
fig|6666666.28482.peg.591	Contig_11	37260	38450	+	hypothetical protein		

fig|6666666.28482.peg.592	Contig_11	41424	38512	-	Excinuclease ABC subunit A	FIG00000147	isu;DNA_repair,_UvrABC_system
fig|6666666.28482.peg.593	Contig_11	41655	42230	+	metallo-beta-lactamase superfamily protein	FIG01193879	ff
fig|6666666.28482.peg.594	Contig_11	42791	42351	-	Universal stress protein UspA and related nucleotide-binding proteins	FIG01023736	ff
fig|6666666.28482.peg.595	Contig_11	43099	44682	+	Possible membrane protein	FIG01121868	ff
fig|6666666.28482.peg.596	Contig_11	44722	45441	+	FIG00828037: hypothetical protein	FIG01336641	ff
fig|6666666.28482.peg.597	Contig_11	46844	45444	-	Drug efflux membrane protein	FIG01387845	ff
fig|6666666.28482.peg.598	Contig_11	49009	46841	-	Excinuclease ABC subunit B	FIG00000146	isu;DNA_repair,_UvrABC_system
fig|6666666.28482.peg.599	Contig_11	48989	49132	+	hypothetical protein		
fig|6666666.28482.peg.600	Contig_11	51254	49212	-	Serine/threonine protein kinase (EC 2.7.11.1)	FIG00933379	ff
fig|6666666.28482.peg.601	Contig_11	51431	54106	+	ABC transporter, ATP-binding protein	FIG00744535	ff
fig|6666666.28482.peg.602	Contig_11	54865	54119	-	3-oxoacyl-[acyl-carrier protein] reductase (EC 1.1.1.100)	FIG00621114	idu(38);CBSS-246196.1.peg.364 idu(38);CBSS-164757.7.peg.5020 idu(38);Fatty_Acid_Biosynthesis_FASII
fig|6666666.28482.peg.603	Contig_11	55007	55447	+	hypothetical protein		
fig|6666666.28482.peg.604	Contig_11	55485	55922	+	hypothetical protein		

fig|6666666.28482.peg.605	Contig_11	55919	56917	+	ATP-dependent DNA ligase (EC 6.5.1.1) clustered with Ku protein, LigD	FIG00002268	icw(1);DNA_Repair_Base_Excision icw(1);DNA_ligases icw(1);Nonhomologous_End-Joining_in_Bacteria icw(1);Bacillus_subtilis_scratch_-_gjo
fig|6666666.28482.peg.606	Contig_11	56914	57801	+	ATP-dependent DNA ligase (EC 6.5.1.1) clustered with Ku protein, LigD	FIG00002268	icw(1);DNA_Repair_Base_Excision icw(1);DNA_ligases icw(1);Nonhomologous_End-Joining_in_Bacteria icw(1);Bacillus_subtilis_scratch_-_gjo
fig|6666666.28482.peg.607	Contig_11	57794	58573	+	DNA-formamidopyrimidine glycosylase(EC:3.2.2.23)	FIG01524345	ff
fig|6666666.28482.peg.608	Contig_11	58585	59001	+	FIG00827185: hypothetical protein	FIG00827183	ff
fig|6666666.28482.peg.609	Contig_11	60367	59153	-	Dephospho-CoA kinase (EC 2.7.1.24)	FIG00000267	isu;Coenzyme_A_Biosynthesis
fig|6666666.28482.peg.610	Contig_11	61825	60380	-	SSU ribosomal protein S1p	FIG00000210	isu;Cell_division-ribosomal_stress_proteins_cluster
fig|6666666.28482.peg.611	Contig_11	61835	62032	+	hypothetical protein		
fig|6666666.28482.peg.612	Contig_11	64770	62029	-	DNA polymerase I (EC 2.7.7.7)	FIG00000404	idu(1);DNA_Repair_Base_Excision idu(1);DNA-replication
fig|6666666.28482.peg.613	Contig_11	65087	64800	-	Uncharacterized conserved protein	FIG01340511	ff
fig|6666666.28482.peg.614	Contig_11	65219	65662	+	FIG00823085: hypothetical protein	FIG00823077	ff
fig|6666666.28482.peg.615	Contig_11	65659	66858	+	Probable nonspecific lipid-transfer protein	FIG01418201	ff
fig|6666666.28482.peg.616	Contig_11	67702	67052	-	response regulator	FIG00826122	ff

fig|6666666.28482.rna.8	Contig_11	67776	67849	+	tRNA-Leu-CAA		isu;tRNAs
fig|6666666.28482.peg.617	Contig_11	67860	68372	+	hypothetical protein	FIG00638284	ff
fig|6666666.28482.peg.618	Contig_11	68398	69726	+	Adenylate cyclase (EC 4.6.1.1)	FIG00001294	idu(25);cAMP_signaling_in_bacteria
fig|6666666.28482.peg.619	Contig_11	69823	70368	+	Possible membrane protein	FIG01121868	ff
fig|6666666.28482.peg.620	Contig_11	70412	71878	+	Cytochrome d ubiquinol oxidase subunit I (EC 1.10.3.-)	FIG00096593	isu;Terminal_cytochrome_d_ubiquinol_oxidases isu;Terminal_cytochrome_oxidases
fig|6666666.28482.peg.621	Contig_11	71890	72930	+	Cytochrome d ubiquinol oxidase subunit II (EC 1.10.3.-)	FIG00000390	icw(1);Terminal_cytochrome_d_ubiquinol_oxidases icw(1);Terminal_cytochrome_oxidases
fig|6666666.28482.peg.622	Contig_11	72987	74600	+	Transport ATP-binding protein CydD	FIG01010650	icw(2);Terminal_cytochrome_d_ubiquinol_oxidases icw(2);Terminal_cytochrome_oxidases
fig|6666666.28482.peg.623	Contig_11	74587	76236	+	Transport ATP-binding protein CydC	FIG00046358	icw(3);Terminal_cytochrome_d_ubiquinol_oxidases icw(3);Terminal_cytochrome_oxidases
fig|6666666.28482.peg.624	Contig_11	77605	76211	-	Putative membrane protein MT1655	FIG01531367	ff
fig|6666666.28482.peg.625	Contig_11	78636	77755	-	Acyl-CoA thioesterase II (EC 3.1.2.-)	FIG00002217	idu(3);Acyl-CoA_thioesterase_II
fig|6666666.28482.peg.626	Contig_11	80009	78648	-	Pyruvate kinase (EC 2.7.1.40)	FIG00000043	isu;Glycerate_metabolism isu;Pyruvate_metabolism_I:_anaplerotic_reactions,_PEP
fig|6666666.28482.peg.627	Contig_11	80534	80130	-	Possible membrane protein	FIG01121868	ff
fig|6666666.28482.peg.628	Contig_11	80793	80548	-	FIG00822583: hypothetical protein	FIG00822582	ff

fig|6666666.28482.peg.629	Contig_11	82849	80999	-	Prolipoprotein diacylglyceryl transferase (EC 2.4.99.-)	FIG00000985	isu;Lipoprotein_Biosynthesis
fig|6666666.28482.peg.630	Contig_11	83661	82846	-	Tryptophan synthase alpha chain (EC 4.2.1.20)	FIG00105533	isu;Chorismate:_Intermediate_for_synthesis_of_Tryptophan,_PAPA_antibiotics,_PABA,_3-hydroxyanthranilate_and_more. isu;Tryptophan_synthesis
fig|6666666.28482.peg.631	Contig_11	84920	83658	-	Tryptophan synthase beta chain (EC 4.2.1.20)	FIG00116323	icw(1);Chorismate:_Intermediate_for_synthesis_of_Tryptophan,_PAPA_antibiotics,_PABA,_3-hydroxyanthranilate_and_more. icw(1);Tryptophan_synthesis
fig|6666666.28482.peg.632	Contig_11	85775	84957	-	Indole-3-glycerol phosphate synthase (EC 4.1.1.48)	FIG00135159	icw(2);Chorismate:_Intermediate_for_synthesis_of_Tryptophan,_PAPA_antibiotics,_PABA,_3-hydroxyanthranilate_and_more. icw(2);Tryptophan_synthesis
fig|6666666.28482.peg.633	Contig_11	86534	85875	-	FIG00997095: hypothetical protein	FIG00997093	ff
fig|6666666.28482.peg.634	Contig_11	88065	86527	-	Anthranilate synthase, aminase component (EC 4.1.3.27)	FIG00011663	icw(3);Chorismate:_Intermediate_for_synthesis_of_Tryptophan,_PAPA_antibiotics,_PABA,_3-hydroxyanthranilate_and_more. icw(3);Tryptophan_synthesis
fig|6666666.28482.peg.635	Contig_11	88169	88642	+	Alkyl hydroperoxide reductase subunit C-like protein	FIG01258694	idu(1);Oxidative_stress idu(1);Rubrerythrin idu(1);Thioredoxin-disulfide_reductase
fig|6666666.28482.peg.636	Contig_11	89828	88725	-	Calcium/proton antiporter	FIG00634903	ff

fig|6666666.28482.peg.637	Contig_11	90717	89893	-	O-Methyltransferase involved in polyketide biosynthesis		
fig|6666666.28482.peg.638	Contig_11	91582	90725	-	Transcriptional regulator, AraC family	FIG00510130	ff
fig|6666666.28482.peg.639	Contig_11	91639	93165	+	Serine/threonine protein kinase (EC 2.7.11.1)	FIG00933379	ff
fig|6666666.28482.peg.640	Contig_11	93509	93162	-	Phosphoribosyl-AMP cyclohydrolase (EC 3.5.4.19)	FIG01303889	icw(6);Histidine_Biosynthesis
fig|6666666.28482.peg.641	Contig_11	94291	93506	-	Imidazole glycerol phosphate synthase cyclase subunit (EC 4.1.3.-)	FIG00000258	icw(5);Histidine_Biosynthesis
fig|6666666.28482.peg.642	Contig_11	95105	94293	-	Histidinol-phosphatase [alternative form] (EC 3.1.3.15)	FIG00001748	icw(2);Histidine_Biosynthesis
fig|6666666.28482.peg.643	Contig_11	95841	95110	-	Phosphoribosylformimino-5-aminoimidazole carboxamide ribotide isomerase (EC 5.3.1.16) / Acting phosphoribosylanthranilate isomerase (EC 5.3.1.24)	FIG00137296	isu;Chorismate:_Intermediate_for_synthesis_of_Tryptophan,_PAPA_antibiotics,_PABA,_3-hydroxyanthranilate_and_more. isu;Histidine_Biosynthesis isu;Tryptophan_synthesis
fig|6666666.28482.peg.644	Contig_11	96541	95924	-	Imidazole glycerol phosphate synthase amidotransferase subunit (EC 2.4.2.-)	FIG00010738	icw(1);Histidine_Biosynthesis

fig|6666666.28482.peg.645	Contig_11	97155	96538	-	Imidazoleglycerol-phosphate dehydratase (EC 4.2.1.19)	FIG00000601	icw(4);Histidine_Biosynthesis
fig|6666666.28482.peg.646	Contig_11	98288	97152	-	Histidinol-phosphate aminotransferase (EC 2.6.1.9)	FIG00000474	icw(3);Histidine_Biosynthesis
fig|6666666.28482.peg.647	Contig_11	99643	98285	-	Histidinol dehydrogenase (EC 1.1.1.23)	FIG00000303	icw(5);Histidine_Biosynthesis
fig|6666666.28482.peg.648	Contig_11	100317	99703	-	hypothetical protein		
fig|6666666.28482.peg.649	Contig_11	100577	100990	+	FIG00820654: hypothetical protein	FIG00820653	ff
fig|6666666.28482.peg.650	Contig_11	102133	101105	-	putative dehydrogenase	FIG01127283	ff
fig|6666666.28482.peg.651	Contig_11	102174	102740	+	Transcriptional regulator, TetR family		ff
fig|6666666.28482.peg.652	Contig_11	103139	102726	-	hypothetical protein		
fig|6666666.28482.peg.653	Contig_11	103222	104025	+	possible transcriptional regulator, AraC family		
fig|6666666.28482.peg.654	Contig_11	104330	104758	+	hypothetical protein		
fig|6666666.28482.peg.655	Contig_11	105633	104779	-	Quinolinate phosphoribosyltransferase [decarboxylating] (EC 2.4.2.19)	FIG00000413	icw(1);Mycobacterium_virulence_operon_possibly_involved_in_quinolinate_biosynthesis icw(2);NAD_and_NADP_cofactor_biosynthesis_global
fig|6666666.28482.peg.656	Contig_11	107210	105636	-	L-aspartate oxidase (EC 1.4.3.16)	FIG00000575	icw(2);Mycobacterium_virulence_operon_possibly_involved_in_quinolinate_biosynthesis icw(3);NAD_and_NADP_cofactor_biosynthesis_global

fig|6666666.28482.peg.657	Contig_11	108256	107207	-	Quinolinate synthetase (EC 2.5.1.72)	FIG00000463	isu;Mycobacterium_virulence_operon_possibly_involved_in_quinolinate_biosynthesis isu;NAD_and_NADP_cofactor_biosynthesis_global
fig|6666666.28482.peg.658	Contig_11	108393	109010	+	Nudix-related transcriptional regulator NrtR	FIG00017495	icw(1);NAD_and_NADP_cofactor_biosynthesis_global
fig|6666666.28482.peg.659	Contig_11	109167	110507	+	Lipase 1 (EC 3.1.1.3)	FIG01327998	ff
fig|6666666.28482.peg.660	Contig_11	111311	110652	-	Possible membrane protein	FIG01121868	ff
fig|6666666.28482.peg.661	Contig_11	111535	111308	-	FIG00820826: hypothetical protein	FIG00820825	ff
fig|6666666.28482.peg.662	Contig_11	112589	111549	-	Biotin synthase (EC 2.8.1.6)	FIG00000467	isu;Biotin_biosynthesis_Experimental isu;Biotin_synthesis_cluster isu;Biotin_biosynthesis
fig|6666666.28482.peg.663	Contig_11	112731	112922	+	hypothetical protein		
fig|6666666.28482.peg.664	Contig_11	113001	114839	+	FIG00830155: hypothetical protein	FIG00830153	ff
fig|6666666.28482.peg.665	Contig_11	114844	115206	+	FIG00827828: hypothetical protein	FIG00827827	ff
fig|6666666.28482.peg.666	Contig_11	115203	116567	+	FIG00829768: hypothetical protein	FIG00829766	ff
fig|6666666.28482.peg.667	Contig_11	117177	116671	-	FIG00820026: hypothetical protein	FIG00820025	ff
fig|6666666.28482.peg.668	Contig_11	117851	117177	-	Dethiobiotin synthetase (EC 6.3.3.3)	FIG00000423	isu;Biotin_biosynthesis_Experimental isu;Biotin_synthesis_cluster isu;Biotin_biosynthesis

fig|6666666.28482.peg.669	Contig_11	119008	117848	-	8-amino-7-oxononanoate synthase (EC 2.3.1.47)	FIG00051001	icw(1);Biotin_biosynthesis_Experimental icw(1);Biotin_synthesis_cluster icw(1);Biotin_biosynthesis
fig|6666666.28482.peg.670	Contig_11	119802	119047	-	Collagen triple helix repeat		
fig|6666666.28482.peg.671	Contig_110	35	376	+	transcriptional regulator, TetR family		
fig|6666666.28482.peg.672	Contig_110	1850	381	-	Aldehyde dehydrogenase (EC 1.2.1.3)	FIG01292786	idu(16);Pyruvate_metabolism_II:_acetyl-CoA,_acetogenesis_from_pyruvate idu(16);Glycerolipid_and_Glycerophospholipid_Metabolism_in_Bacteria idu(16);Methylglyoxal_Metabolism
fig|6666666.28482.peg.673	Contig_110	2712	1945	-	POSSIBLE METHYLTRANSFERASE (METHYLASE)	FIG01121659	ff
fig|6666666.28482.peg.674	Contig_110	3913	2738	-	hypothetical protein	FIG00638284	ff
fig|6666666.28482.peg.675	Contig_110	4253	5407	+	Glycosyltransferase (EC 2.4.1.-)	FIG01193496	ff
fig|6666666.28482.peg.676	Contig_110	7076	5391	-	Possible membrane protein	FIG01121868	ff
fig|6666666.28482.peg.677	Contig_110	8384	7086	-	PROBABLE CONSERVED MEMBRANE PROTEIN	FIG00662487	ff
fig|6666666.28482.peg.678	Contig_110	8531	9700	+	Lysophospholipid acyltransferase	FIG00149539	ff
fig|6666666.28482.peg.679	Contig_110	10175	9702	-	hypothetical protein		

fig|6666666.28482.peg.680	Contig_110	11721	10249	-	Long-chain-fatty-acid--CoA ligase (EC 6.2.1.3)	FIG00018699	idu(72);n-Phenylalkanoic_acid_degradation idu(72);Fatty_acid_metabolism_cluster idu(72);Biotin_synthesis_cluster idu(72);Biotin_biosynthesis
fig|6666666.28482.peg.681	Contig_110	12698	11718	-	Phosphotriesterase homology protein	FIG01324117	ff
fig|6666666.28482.peg.682	Contig_110	14176	13466	-	hypothetical protein		
fig|6666666.28482.peg.683	Contig_110	15439	14189	-	Argininosuccinate lyase (EC 4.3.2.1)	FIG00000333	idu(1);Arginine_Biosynthesis_--_gjo idu(1);Arginine_Biosynthesis_extended
fig|6666666.28482.peg.684	Contig_110	15980	15456	-	Putative transcriptional regulator		
fig|6666666.28482.peg.685	Contig_110	16871	15996	-	Citrate lyase beta chain (EC 4.1.3.6)	FIG00010100	idu(2);Citrate_Metabolism,_Transport,_and_Regulation
fig|6666666.28482.peg.686	Contig_110	18157	16898	-	2-amino-3-ketobutyrate coenzyme A ligase (EC 2.3.1.29)	FIG00000741	isu;Glycine_Biosynthesis
fig|6666666.28482.peg.687	Contig_110	18324	19211	+	hypothetical protein	FIG00638284	ff
fig|6666666.28482.peg.688	Contig_110	19281	20990	+	Acyl-CoA dehydrogenase (EC 1.3.99.3)	FIG00002266	ff
fig|6666666.28482.peg.689	Contig_110	21168	21830	+	Transcriptional regulator, TetR family	FIG00554417	ff
fig|6666666.28482.peg.690	Contig_110	21827	22768	+	Ribonucleotide-diphosphate reductase subunit beta (EC 1.17.4.1)	FIG01313305	ff

fig|6666666.28482.peg.691	Contig_110	24202	22784	-	Succinate-semialdehyde dehydrogenase [NAD] (EC 1.2.1.24); Succinate-semialdehyde dehydrogenase [NADP+] (EC 1.2.1.16)	FIG01959002	ff
fig|6666666.28482.peg.692	Contig_110	24283	25929	+	COG0028: Thiamine pyrophosphate-requiring enzymes	FIG00452446	ff
fig|6666666.28482.peg.693	Contig_111	66	920	+	MCE-family lipoprotein LprK (MCE-family lipoprotein Mce1e)	FIG00819998	icw(1);Mycobacterium_virulence_operon_MCE_involved_in_cell_invasion
fig|6666666.28482.peg.694	Contig_111	921	2600	+	MCE-family protein Mce1F	FIG00821171	idu(7);Mycobacterium_virulence_operon_MCE_involved_in_cell_invasion
fig|6666666.28482.peg.695	Contig_111	3149	2634	-	FIG00822490: hypothetical protein	FIG00822486	ff
fig|6666666.28482.peg.696	Contig_111	3812	3237	-	FIG00820816: hypothetical protein	FIG00820815	ff
fig|6666666.28482.peg.697	Contig_111	4549	3809	-	hypothetical protein		
fig|6666666.28482.peg.698	Contig_111	4764	5117	+	FIG00828565: hypothetical protein	FIG00828564	ff
fig|6666666.28482.peg.699	Contig_111	5409	5134	-	SECRETED PROTEIN ANTIGEN	FIG01357523	ff
fig|6666666.28482.peg.700	Contig_111	6051	5470	-	Transcriptional regulator, TetR family	FIG00554417	ff
fig|6666666.28482.peg.701	Contig_111	6157	6942	+	FIG00820623: hypothetical protein	FIG00820622	ff
fig|6666666.28482.peg.702	Contig_111	7017	7745	+	FIG00822891: hypothetical protein	FIG00822890	ff

fig|6666666.28482.peg.703	Contig_111	7742	8314	+	Cytochrome c oxidase polypeptide III (EC 1.9.3.1)	FIG00000640	idu(1);Terminal_cytochrome_C_oxidases idu(1);CBSS-316057.3.peg.563
fig|6666666.28482.peg.704	Contig_111	8311	8601	+	FIG00825808: hypothetical protein	FIG00825806	ff
fig|6666666.28482.peg.705	Contig_112	81	1745	+	hypothetical protein	FIG00638284	ff
fig|6666666.28482.peg.706	Contig_112	1849	2778	+	DrrA		
fig|6666666.28482.peg.707	Contig_112	2775	3536	+	ABC-2 type transporter		
fig|6666666.28482.peg.708	Contig_112	3533	4321	+	ABC-2 type transporter		
fig|6666666.28482.peg.709	Contig_112	4346	5620	+	GtfA	FIG01525338	ff
fig|6666666.28482.peg.710	Contig_112	5644	5757	+	hypothetical protein		
fig|6666666.28482.peg.711	Contig_112	5754	12287	+	Long-chain-fatty-acid--CoA ligase (EC 6.2.1.3)	FIG00018699	idu(72);n-Phenylalkanoic_acid_degradation idu(72);Fatty_acid_metabolism_cluster idu(72);Biotin_synthesis_cluster idu(72);Biotin_biosynthesis
fig|6666666.28482.peg.712	Contig_112	12338	14521	+	O-antigen acetylase	FIG01370327	ff
fig|6666666.28482.peg.713	Contig_112	15983	14526	-	Glucose-methanol-choline (GMC) oxidoreductase:NAD binding site	FIG00001629	idu(3);Respiratory_dehydrogenases_1
fig|6666666.28482.peg.714	Contig_113	1766	219	-	PPE family protein	FIG00013075	idu(57);CBSS-164757.7.peg.5020
fig|6666666.28482.peg.715	Contig_113	1803	1916	+	hypothetical protein		

fig|6666666.28482.peg.716	Contig_113	2130	5360	+	Adenylyl cyclase class-3/4/guanylyl cyclase / Disease resistance domain-containing protein / Tetratricopeptide repeat-containing protein / Transcriptional regulator, LuxR family	FIG01386132	ff
fig|6666666.28482.peg.717	Contig_113	5672	5454	-	hypothetical protein		
fig|6666666.28482.peg.718	Contig_114	348	479	+	hypothetical protein		
fig|6666666.28482.peg.719	Contig_114	553	1509	+	Prephenate dehydratase (EC 4.2.1.51)	FIG00341289	isu;Chorismate_Synthesis isu;Phenylalanine_and_Tyrosine_Branches_from_Chorismate
fig|6666666.28482.peg.720	Contig_114	1521	2192	+	putative phosphoglycerate mutase	FIG00997142	ff
fig|6666666.28482.peg.721	Contig_114	2768	2418	-	FIG00820873: hypothetical protein	FIG00820872	ff
fig|6666666.28482.peg.722	Contig_114	4149	2773	-	PROBABLE CONSERVED MEMBRANE PROTEIN	FIG00662487	ff
fig|6666666.28482.peg.723	Contig_114	4262	5515	+	Seryl-tRNA synthetase (EC 6.1.1.11)	FIG00000121	isu;tRNA_aminoacylation,_Ser isu;CBSS-326442.4.peg.1852
fig|6666666.28482.peg.724	Contig_114	6030	5512	-	FIG00445285: hypothetical protein	FIG00445283	ff
fig|6666666.28482.peg.725	Contig_114	6152	6454	+	putative transcriptional regulatory protein, related to MarR family		
fig|6666666.28482.peg.726	Contig_114	6935	6456	-	FIG00821485: hypothetical protein	FIG00821484	ff

fig|6666666.28482.peg.727	Contig_114	7007	7615	+	Beta-carotene ketolase (EC 1.14.-.-)	FIG00071345	icw(1);Proteorhodopsin
fig|6666666.28482.peg.728	Contig_114	7683	9245	+	Beta-carotene ketolase (EC 1.14.-.-)	FIG00071345	icw(1);Proteorhodopsin
fig|6666666.28482.peg.729	Contig_114	9586	9242	-	Putative uncharacterized protein BCG_3881	FIG01318042	ff
fig|6666666.28482.peg.730	Contig_114	11133	9583	-	Rieske (2Fe-2S) domain protein	FIG00999265	ff
fig|6666666.28482.peg.731	Contig_114	11273	12046	+	1-acyl-sn-glycerol-3-phosphate acyltransferase (EC 2.3.1.51)	FIG00005243	icw(2);Glycerolipid_and_Glycerophospholipid_Metabolism_in_Bacteria icw(2);Ribosome_post-transcriptional_modification_and_chromosomal_segregation_cluster
fig|6666666.28482.peg.732	Contig_114	12046	12813	+	1-acyl-sn-glycerol-3-phosphate acyltransferase (EC 2.3.1.51)	FIG00005243	icw(2);Glycerolipid_and_Glycerophospholipid_Metabolism_in_Bacteria icw(2);Ribosome_post-transcriptional_modification_and_chromosomal_segregation_cluster
fig|6666666.28482.peg.733	Contig_114	12830	13615	+	1-acyl-sn-glycerol-3-phosphate acyltransferase (EC 2.3.1.51)	FIG00005243	icw(2);Glycerolipid_and_Glycerophospholipid_Metabolism_in_Bacteria icw(2);Ribosome_post-transcriptional_modification_and_chromosomal_segregation_cluster
fig|6666666.28482.peg.734	Contig_114	13635	14450	+	Cof family hydrolase	FIG01362030	ff
fig|6666666.28482.peg.735	Contig_114	15982	14447	-	PE_PGRS family protein		
fig|6666666.28482.peg.736	Contig_114	17693	16086	-	Putative uncharacterized protein BCG_3873	FIG00994577	ff

fig|6666666.28482.peg.737	Contig_115	709	1437	+	hypothetical protein	FIG00638284	ff
fig|6666666.28482.peg.738	Contig_115	2314	1505	-	COG1376: Uncharacterized protein conserved in bacteria		
fig|6666666.28482.peg.739	Contig_115	2519	4357	+	Pyruvate decarboxylase (EC 4.1.1.1); Alpha-keto-acid decarboxylase (EC 4.1.1.-)	FIG00001998	idu(2);Pyruvate_metabolism_II:_acetyl-CoA,_acetogenesis_from_pyruvate
fig|6666666.28482.peg.740	Contig_115	6094	4370	-	Possible membrane protein	FIG01121868	ff
fig|6666666.28482.peg.741	Contig_115	6621	6166	-	hypothetical protein		
fig|6666666.28482.peg.742	Contig_115	6776	7585	+	Long-chain-fatty-acid--CoA ligase (EC 6.2.1.3)	FIG00018699	icw(1);n-Phenylalkanoic_acid_degradation icw(1);Fatty_acid_metabolism_cluster icw(1);Biotin_synthesis_cluster icw(1);Biotin_biosynthesis
fig|6666666.28482.peg.743	Contig_115	7589	9232	+	Long-chain-fatty-acid--CoA ligase (EC 6.2.1.3)	FIG00018699	icw(1);n-Phenylalkanoic_acid_degradation icw(1);Fatty_acid_metabolism_cluster icw(1);Biotin_synthesis_cluster icw(1);Biotin_biosynthesis
fig|6666666.28482.peg.744	Contig_115	9232	10500	+	Lipase (EC 3.1.1.3)	FIG01320296	ff
fig|6666666.28482.peg.745	Contig_115	10639	11484	+	FIG00826634: hypothetical protein	FIG00826633	ff

fig|6666666.28482.peg.746	Contig_115	12886	11549	-	Wax ester synthase/acyl-CoA:diacylglycerol acyltransferase	FIG01309728	ff
fig|6666666.28482.peg.747	Contig_115	12881	13000	+	hypothetical protein		
fig|6666666.28482.peg.748	Contig_115	13980	12997	-	hypothetical protein		
fig|6666666.28482.peg.749	Contig_115	14996	13971	-	FIG002813: LPPG:FO 2-phospho-L-lactate transferase like, CofD-like		
fig|6666666.28482.peg.750	Contig_115	15868	14993	-	FIG000506: Predicted P-loop-containing kinase		
fig|6666666.28482.peg.751	Contig_115	17808	15865	-	Excinuclease ABC subunit C	FIG00000185	isu;DNA_repair,_UvrABC_system
fig|6666666.28482.peg.752	Contig_115	18972	19136	+	Enoyl-CoA hydratase (EC 4.2.1.17)		idu(51);n-Phenylalkanoic_acid_degradation idu(51);Polyhydroxybutyrate_metabolism idu(51);Isoleucine_degradation idu(51);Fatty_acid_metabolism_cluster idu(51);Lysine_fermentation idu(51);Butanol_Biosynthesis idu(51);Acetyl-CoA_fermentation_to_Butyrate idu(51);Valine_degradation idu(51);Butyrate_metabolism_cluster
fig|6666666.28482.peg.753	Contig_115	19878	19210	-	Lipoprotein LprH	FIG01346249	ff

fig|6666666.28482.peg.754	Contig_115	20307	19897	-	Cell division protein DivIC (FtsB), stabilizes FtsL against RasP cleavage	FIG00485194	idu(10);Possible_new_toxin-antitoxin_system_including_DivIC idu(10);Bacterial_Cell_Division idu(10);Cell_division-ribosomal_stress_proteins_cluster idu(10);Bacterial_Cytoskeleton idu(10);Stationary_phase_repair_cluster
fig|6666666.28482.peg.755	Contig_115	20813	20358	-	6,7-dimethyl-8-ribityllumazine synthase (EC 2.5.1.78)	FIG00000231	isu;Riboflavin_synthesis_cluster icw(1);Riboflavin,_FMN_and_FAD_metabolism
fig|6666666.28482.peg.756	Contig_115	22141	20837	-	3,4-dihydroxy-2-butanone 4-phosphate synthase (EC 4.1.99.12) / GTP cyclohydrolase II (EC 3.5.4.25)		icw(2);Riboflavin_synthesis_cluster icw(3);Riboflavin_synthesis_cluster icw(3);Riboflavin,_FMN_and_FAD_metabolism icw(3);Riboflavin,_FMN_and_FAD_metabolism icw(1);riboflavin_to_FAD isu;Molybdenum_cofactor_biosynthesis
fig|6666666.28482.peg.757	Contig_115	22307	22678	+	secreted protein antigen		
fig|6666666.28482.peg.758	Contig_115	23343	22738	-	Riboflavin synthase eubacterial/eukaryotic (EC 2.5.1.9)	FIG00000273	icw(4);Riboflavin_synthesis_cluster isu;Riboflavin,_FMN_and_FAD_metabolism isu;riboflavin_to_FAD
fig|6666666.28482.peg.759	Contig_115	23515	24135	+	Lipoprotein LprG	FIG01327396	ff

fig|6666666.28482.peg.760	Contig_115	24132	25694	+	EmrB/QacA family drug resistance transporter	FIG00997405	ff
fig|6666666.28482.peg.761	Contig_115	26726	25698	-	Diaminohydroxyphosphoribosylaminopyrimidine deaminase (EC 3.5.4.26) / 5-amino-6-(5-phosphoribosylamino)uracil reductase (EC 1.1.1.193)	FIG00000384	isu;Riboflavin_synthesis_cluster icw(3);Riboflavin_synthesis_cluster icw(1);Riboflavin,_FMN_and_FAD_metabolism icw(1);Riboflavin,_FMN_and_FAD_metabolism
fig|6666666.28482.peg.762	Contig_115	27421	26723	-	Ribulose-phosphate 3-epimerase (EC 5.1.3.1)	FIG00000047	icw(1);Riboflavin_synthesis_cluster isu;Pentose_phosphate_pathway
fig|6666666.28482.peg.763	Contig_115	28559	27450	-	Ribosomal RNA small subunit methyltransferase B (EC 2.1.1.-)	FIG00138175	ff
fig|6666666.28482.peg.764	Contig_116	25	150	+	hypothetical protein		
fig|6666666.28482.peg.765	Contig_116	255	1103	+	hypothetical protein		
fig|6666666.28482.peg.766	Contig_116	2816	1158	-	Ubiquinol--cytochrome c reductase, cytochrome B subunit (EC 1.10.2.2)	FIG00000754	icw(2);Ubiquinone_Menaquinone-cytochrome_c_reductase_complexes
fig|6666666.28482.peg.767	Contig_116	4015	2813	-	Ubiquinol-cytochrome C reductase iron-sulfur subunit (EC 1.10.2.2)	FIG00000797	icw(1);Ubiquinone_Menaquinone-cytochrome_c_reductase_complexes

fig|6666666.28482.peg.768	Contig_116	4752	3991	-	ubiquinol cytochrome C oxidoreductase, cytochrome C1 subunit	FIG00001102	isu;Ubiquinone_Menaquinone-cytochrome_c_reductase_complexes
fig|6666666.28482.peg.769	Contig_116	5492	4893	-	Cytochrome c oxidase polypeptide III (EC 1.9.3.1)	FIG00000640	idu(1);Terminal_cytochrome_C_oxidases idu(1);CBSS-316057.3.peg.563
fig|6666666.28482.peg.770	Contig_116	5643	6725	+	Anthranilate phosphoribosyltransferase (EC 2.4.2.18)	FIG00062957	isu;Chorismate:_Intermediate_for_synthesis_of_Tryptophan,_PAPA_antibiotics,_PABA,_3-hydroxyanthranilate_and_more. isu;Tryptophan_synthesis
fig|6666666.28482.peg.771	Contig_117	1604	1314	-	FIG00820970: hypothetical protein	FIG00820967	ff
fig|6666666.28482.peg.772	Contig_117	1680	3383	+	Dihydroxy-acid dehydratase (EC 4.2.1.9)	FIG00138258	isu;Branched-Chain_Amino_Acid_Biosynthesis
fig|6666666.28482.peg.773	Contig_117	3873	3433	-	PROBABLE CONSERVED TRANSMEMBRANE PROTEIN	FIG00662132	ff
fig|6666666.28482.peg.774	Contig_117	3983	4972	+	FIG00831487: hypothetical protein	FIG00831485	ff
fig|6666666.28482.peg.775	Contig_117	5636	4977	-	O-methyltransferase	FIG00821228	ff
fig|6666666.28482.peg.776	Contig_117	5725	5871	+	FIG00821308: hypothetical protein	FIG00821306	ff
fig|6666666.28482.peg.777	Contig_117	7702	5903	-	Beta-glucosidase (EC 3.2.1.21)	FIG00001469	isu;Beta-Glucoside_Metabolism
fig|6666666.28482.peg.778	Contig_118	3374	1509	-	FIG00824757: hypothetical protein	FIG00824754	ff
fig|6666666.28482.peg.779	Contig_118	3627	4421	+	FIG00826773: hypothetical protein	FIG00826770	ff

fig|6666666.28482.peg.780	Contig_118	4509	5696	+	NADH dehydrogenase (EC 1.6.99.3)	FIG00008708	idu(6);Respiratory_dehydrogenases_1 idu(6);PPE_gene_cluster_in_Mycobacteria idu(6);Riboflavin_synthesis_cluster
fig|6666666.28482.peg.781	Contig_118	5693	6604	+	FIG00826795: hypothetical protein	FIG00826794	ff
fig|6666666.28482.peg.782	Contig_118	6621	7874	+	Para-aminobenzoate synthase, aminase component (EC 2.6.1.85)	FIG00341164	isu;Chorismate:_Intermediate_for_synthesis_of_Tryptophan,_PAPA_antibiotics,_PABA,_3-hydroxyanthranilate_and_more. isu;Folate_Biosynthesis isu;Tryptophan_synthesis
fig|6666666.28482.peg.783	Contig_119	994	1581	+	hypothetical protein	FIG00638284	ff
fig|6666666.28482.peg.784	Contig_119	3074	1590	-	Acetyl-CoA acetyltransferase	FIG00832423	ff
fig|6666666.28482.peg.785	Contig_119	5617	3209	-	Acyl-CoA transferases/carnitine dehydratase	FIG01313105	ff
fig|6666666.28482.peg.786	Contig_119	5772	6674	+	Undecaprenyl-diphosphatase (EC 3.6.1.27)	FIG00000555	ff
fig|6666666.28482.peg.787	Contig_119	7216	6671	-	GCN5-related N-acetyltransferase	FIG01272034	ff
fig|6666666.28482.peg.788	Contig_119	7246	8094	+	Oxidoreductase, short-chain dehydrogenase/reductase family	FIG00993242	ff

fig|6666666.28482.peg.789	Contig_119	8310	10697	+	Xylulose-5-phosphate phosphoketolase (EC 4.1.2.9); Fructose-6-phosphate phosphoketolase (EC 4.1.2.22)	FIG00137542	isu;Fermentations:_Lactate isu;Fermentations:_Lactate isu;Pentose_phosphate_pathway isu;Pentose_phosphate_pathway
fig|6666666.28482.peg.790	Contig_119	10933	12153	+	PPE family protein	FIG00013075	idu(57);CBSS-164757.7.peg.5020
fig|6666666.28482.peg.791	Contig_119	12408	12241	-	hypothetical protein		
fig|6666666.28482.peg.792	Contig_119	12490	13071	+	hypothetical protein		
fig|6666666.28482.peg.793	Contig_119	13628	14371	+	Possible membrane protein	FIG01121868	ff
fig|6666666.28482.peg.794	Contig_119	15404	14376	-	Alcohol dehydrogenase (EC 1.1.1.1)	FIG01007502	idu(17);Glycerolipid_and_Glycerophospholipid_Metabolism_in_Bacteria idu(17);Fermentations:_Mixed_acid idu(17);Butanol_Biosynthesis
fig|6666666.28482.peg.795	Contig_119	15752	15453	-	hypothetical protein	FIG00638284	ff
fig|6666666.28482.peg.796	Contig_119	16989	15961	-	Alanine and proline-rich secreted protein Apa, fibronectin attachment protein	FIG00822366	ff
fig|6666666.28482.peg.797	Contig_119	18184	17087	-	Molybdenum transport ATP-binding protein ModC (TC 3.A.1.8.1)	FIG00002742	isu;Molybdenum_cofactor_biosynthesis
fig|6666666.28482.peg.798	Contig_119	18942	18181	-	Molybdenum transport system permease protein ModB (TC 3.A.1.8.1)	FIG00002718	icw(1);Molybdenum_cofactor_biosynthesis

fig|6666666.28482.peg.799	Contig_119	19075	21696	+	Disease resistance domain-containing protein / Transcriptional regulator, LuxR family		
fig|6666666.28482.peg.800	Contig_119	22494	21709	-	Molybdenum ABC transporter, periplasmic molybdenum-binding protein ModA (TC 3.A.1.8.1)	FIG00021644	icw(2);Molybdenum_cofactor_biosynthesis
fig|6666666.28482.peg.801	Contig_119	22714	23385	+	Oxidoreductase, short-chain dehydrogenase/reductase family	FIG00993242	ff
fig|6666666.28482.peg.802	Contig_119	23431	24357	+	Oxidoreductase	FIG01321859	ff
fig|6666666.28482.peg.803	Contig_119	24533	25915	+	NADH dehydrogenase (EC 1.6.99.3)	FIG00008708	idu(6);Respiratory_dehydrogenases_1 idu(6);PPE_gene_cluster_in_Mycobacteria idu(6);Riboflavin_synthesis_cluster
fig|6666666.28482.peg.804	Contig_119	26569	26153	-	Putative esterase	FIG00998593	ff
fig|6666666.28482.peg.805	Contig_119	26865	27284	+	Transcriptional regulator, BlaI family	FIG00996263	ff
fig|6666666.28482.peg.806	Contig_119	27299	28249	+	Peptidase M48, Ste24p precursor	FIG00996554	ff
fig|6666666.28482.peg.807	Contig_119	28353	29810	+	6-phosphogluconate dehydrogenase, decarboxylating (EC 1.1.1.44)	FIG00000405	idu(1);Pentose_phosphate_pathway idu(1);D-gluconate_and_ketogluconates_metabolism

fig|6666666.28482.peg.808	Contig_119	29854	31290	+	Inosine-5'-monophosphate dehydrogenase (EC 1.1.1.205)	FIG00020613	idu(4);Purine_conversions idu(4);Purine_salvage_cluster
fig|6666666.28482.peg.809	Contig_119	31459	32829	+	Magnesium and cobalt efflux protein CorC	FIG00042627	idu(1);CBSS-56780.10.peg.1536
fig|6666666.28482.peg.810	Contig_119	32826	33866	+	hypothetical protein		
fig|6666666.28482.peg.811	Contig_119	34095	36302	+	Malate synthase G (EC 2.3.3.9)	FIG00001433	isu;Glyoxylate_bypass isu;Photorespiration_(oxidative_C2_cycle) isu;Glyoxylate_bypass_cluster
fig|6666666.28482.peg.812	Contig_119	36419	38452	+	FIG00996932: hypothetical protein	FIG00820280	ff
fig|6666666.28482.peg.813	Contig_119	38585	39445	+	Haloalkane dehalogenase 2 (EC 3.8.1.5)	FIG01311780	ff
fig|6666666.28482.peg.814	Contig_12	56	754	+	Phenylpropionate dioxygenase and related ring-hydroxylating dioxygenases, large terminal subunit	FIG01371907	ff
fig|6666666.28482.peg.815	Contig_12	2159	813	-	FIG00821743: hypothetical protein	FIG00821742	ff
fig|6666666.28482.peg.816	Contig_12	2257	3723	+	Aldehyde dehydrogenase (EC 1.2.1.3)	FIG01292786	idu(16);Pyruvate_metabolism_II:_acetyl-CoA,_acetogenesis_from_pyruvate idu(16);Glycerolipid_and_Glycerophospholipid_Metabolism_in_Bacteria idu(16);Methylglyoxal_Metabolism

fig|6666666.28482.peg.817	Contig_12	4251	3718	-	hypothetical protein	FIG00638284	ff
fig|6666666.28482.peg.818	Contig_12	4681	4244	-	FIG00822385: hypothetical protein	FIG00822384	ff
fig|6666666.28482.peg.819	Contig_12	5214	4678	-	FIG00821462: hypothetical protein	FIG00821460	ff
fig|6666666.28482.peg.820	Contig_12	5636	5211	-	FIG00823763: hypothetical protein	FIG00823762	ff
fig|6666666.28482.peg.821	Contig_12	7293	5728	-	FIG00825609: hypothetical protein	FIG00825607	ff
fig|6666666.28482.peg.822	Contig_12	8420	7293	-	FIG00821359: hypothetical protein	FIG00821357	ff
fig|6666666.28482.peg.823	Contig_12	8544	9779	+	putative cytochrome P450 hydroxylase	FIG00032437	idu(49);Nitric_oxide_synthase
fig|6666666.28482.peg.824	Contig_12	10221	9781	-	alternate gene name: yeeL		
fig|6666666.28482.peg.825	Contig_12	10453	10262	-	FIG00822039: hypothetical protein	FIG00822036	ff
fig|6666666.28482.peg.826	Contig_12	12862	10472	-	FIG00828044: hypothetical protein	FIG00828040	ff
fig|6666666.28482.peg.827	Contig_12	12925	13758	+	Transcriptional regulator, GntR family	FIG00621925	ff
fig|6666666.28482.peg.828	Contig_12	13764	14483	+	acetoacetyl-CoA reductase(EC:1.1.1.36)	FIG01667335	ff
fig|6666666.28482.peg.829	Contig_12	15312	14446	-	FIG00825280: hypothetical protein	FIG00825278	ff

fig|6666666.28482.peg.830	Contig_12	15383	16441	+	Isovaleryl-CoA dehydrogenase (EC 1.3.99.10); Butyryl-CoA dehydrogenase (EC 1.3.99.2)	FIG01304407	icw(1);Isoleucine_degradation idu(5);HMG_CoA_Synthesis icw(1);Lysine_fermentation icw(1);Butanol_Biosynthesis icw(1);Acetyl-CoA_fermentation_to_Butyrate idu(5);Leucine_Degradation_and_HMG-CoA_Metabolism icw(1);Valine_degradation icw(1);Anaerobic_respiratory_reductases
fig|6666666.28482.peg.831	Contig_12	16438	17544	+	Butyryl-CoA dehydrogenase (EC 1.3.99.2)	FIG00009149	icw(1);Isoleucine_degradation icw(1);Lysine_fermentation icw(1);Butanol_Biosynthesis icw(1);Acetyl-CoA_fermentation_to_Butyrate icw(1);Valine_degradation icw(1);Anaerobic_respiratory_reductases
fig|6666666.28482.peg.832	Contig_12	17550	18698	+	Acyl-CoA dehydrogenase (EC 1.3.99.-)	FIG00820935	ff
fig|6666666.28482.peg.833	Contig_12	18695	19630	+	FIG00823450: hypothetical protein	FIG01416347	ff
fig|6666666.28482.peg.834	Contig_12	19627	21126	+	Long-chain-fatty-acid--CoA ligase (EC 6.2.1.3)	FIG00018699	idu(72);n-Phenylalkanoic_acid_degradation idu(72);Fatty_acid_metabolism_cluster idu(72);Biotin_synthesis_cluster idu(72);Biotin_biosynthesis
fig|6666666.28482.peg.835	Contig_12	21362	21132	-	hypothetical protein		
fig|6666666.28482.peg.836	Contig_12	21541	21939	+	hypothetical protein		

fig|6666666.28482.peg.837	Contig_12	21981	23006	+	Transcriptional regulator, TetR family		
fig|6666666.28482.peg.838	Contig_12	23088	24293	+	BarH	FIG00668014	ff
fig|6666666.28482.peg.839	Contig_12	24779	24285	-	hypothetical protein		
fig|6666666.28482.peg.840	Contig_12	24997	26952	+	Serine phosphatase RsbU, regulator of sigma subunit	FIG00017431	idu(4);SigmaB_stress_responce_regulation idu(4);Sigma-B_stress_response_cluster_1
fig|6666666.28482.peg.841	Contig_12	28218	26983	-	FIG00822014: hypothetical protein	FIG01547276	ff
fig|6666666.28482.peg.842	Contig_12	29366	28215	-	FIG00832621: hypothetical protein	FIG00832619	ff
fig|6666666.28482.peg.843	Contig_12	29521	30309	+	POSSIBLE MEMBRANE PROTEIN		
fig|6666666.28482.peg.844	Contig_12	30306	31007	+	hypothetical protein		
fig|6666666.28482.peg.845	Contig_12	31976	31017	-	FIG00821956: hypothetical protein	FIG00821954	ff
fig|6666666.28482.peg.846	Contig_12	32410	31982	-	hypothetical protein		
fig|6666666.28482.peg.847	Contig_12	33563	32415	-	Amidohydrolase 2		
fig|6666666.28482.peg.848	Contig_12	34600	33563	-	FIG00821228: hypothetical protein	FIG01354128	ff
fig|6666666.28482.peg.849	Contig_12	34965	34606	-	Rieske [2Fe-2S] domain, putative	FIG00825996	ff
fig|6666666.28482.peg.850	Contig_12	35112	36338	+	putative cytochrome P450 hydroxylase	FIG00032437	idu(49);Nitric_oxide_synthase
fig|6666666.28482.peg.851	Contig_12	36335	37492	+	Alcohol dehydrogenase (EC 1.1.1.1)	FIG01007502	idu(17);Glycerolipid_and_Glycerophospholipid_Metabolism_in_Bacteria idu(17);Fermentations:_Mixed_acid idu(17);Butanol_Biosynthesis
fig|6666666.28482.peg.852	Contig_12	38367	37633	-	3-oxoacyl-[acyl-carrier protein] reductase (EC 1.1.1.100)	FIG00621114	idu(38);CBSS-246196.1.peg.364 idu(38);CBSS-164757.7.peg.5020 idu(38);Fatty_Acid_Biosynthesis_FASII

fig|6666666.28482.peg.853	Contig_12	38804	38364	-	hypothetical protein		
fig|6666666.28482.peg.854	Contig_12	39459	38911	-	Protein yceI precursor	FIG00948541	ff
fig|6666666.28482.peg.855	Contig_12	39788	39558	-	hypothetical protein		
fig|6666666.28482.peg.856	Contig_12	40838	40011	-	FIG00827278: hypothetical protein	FIG00827275	ff
fig|6666666.28482.peg.857	Contig_12	42364	41123	-	putative cytochrome P450 hydroxylase	FIG00032437	idu(49);Nitric_oxide_synthase
fig|6666666.28482.peg.858	Contig_12	43383	42361	-	PROBABLE OXIDOREDUCTASE	FIG01416034	ff
fig|6666666.28482.peg.859	Contig_12	43524	45020	+	Propionyl-CoA carboxylase beta chain (EC 6.4.1.3)	FIG01304918	idu(2);Mycobacterium_virulence_operon_involved_in_fatty_acids_biosynthesis idu(2);Serine-glyoxylate_cycle
fig|6666666.28482.peg.860	Contig_12	46136	45090	-	N5,N10-methylenetetrahydromethanopterin reductase-related protein	FIG01460162	ff
fig|6666666.28482.peg.861	Contig_12	46507	46127	-	FIG00822683: hypothetical protein	FIG00822682	ff
fig|6666666.28482.peg.862	Contig_12	47625	46507	-	FIG00823551: hypothetical protein	FIG00823549	ff
fig|6666666.28482.peg.863	Contig_12	49264	47645	-	Cyclohexanone monooxygenase (EC 1.14.13.22)	FIG00975774	ff
fig|6666666.28482.peg.864	Contig_12	49706	49254	-	bile acid 7a-dehydratase	FIG01672580	ff
fig|6666666.28482.peg.865	Contig_12	49790	50782	+	Terminal oxygenase KshA	FIG01318144	ff
fig|6666666.28482.peg.866	Contig_12	50855	51472	+	Isochorismatase (EC 3.3.2.1)	FIG00009017	idu(3);Chorismate:_Intermediate_for_synthesis_of_Tryptophan,_PAPA_antibiotics,_PABA,_3-hydroxyanthranilate_and_more.

fig|6666666.28482.peg.867	Contig_12	51469	52083	+	FIG00825422: hypothetical protein	FIG00825421	ff
fig|6666666.28482.peg.868	Contig_12	52092	53744	+	Long-chain-fatty-acid--CoA ligase (EC 6.2.1.3)	FIG00018699	idu(72);n-Phenylalkanoic_acid_degradation idu(72);Fatty_acid_metabolism_cluster idu(72);Biotin_synthesis_cluster idu(72);Biotin_biosynthesis
fig|6666666.28482.peg.869	Contig_12	54578	53748	-	hypothetical protein	FIG00638284	ff
fig|6666666.28482.peg.870	Contig_12	54839	56047	+	putative cytochrome P450 hydroxylase	FIG00032437	idu(49);Nitric_oxide_synthase
fig|6666666.28482.peg.871	Contig_12	56057	57280	+	L-carnitine dehydratase/bile acid-inducible protein F	FIG01318850	ff
fig|6666666.28482.peg.872	Contig_12	57277	59247	+	3-ketoacyl-CoA thiolase (acaB-12)	FIG00822791	ff
fig|6666666.28482.peg.873	Contig_12	59314	60027	+	NAD-dependent epimerase/dehydratase		
fig|6666666.28482.peg.874	Contig_12	60238	60528	+	protein of unknown function DUF485	FIG00994736	ff
fig|6666666.28482.peg.875	Contig_12	60525	62159	+	sodium:solute symporter protein	FIG00999154	ff
fig|6666666.28482.peg.876	Contig_12	64867	62192	-	regulatory protein, LuxR		
fig|6666666.28482.peg.877	Contig_12	66418	65228	-	FIG00824968: hypothetical protein	FIG00824966	ff

fig|6666666.28482.peg.878	Contig_12	67504	66446	-	Butyryl-CoA dehydrogenase (EC 1.3.99.2)	FIG00009149	idu(39);Isoleucine_degradation idu(39);Lysine_fermentation idu(39);Butanol_Biosynthesis idu(39);Acetyl-CoA_fermentation_to_Butyrate idu(39);Valine_degradation idu(39);Anaerobic_respiratory_reductases
fig|6666666.28482.peg.879	Contig_12	68336	67506	-	PROBABLE ANTIBIOTIC-RESISTANCE PROTEIN	FIG01366849	ff
fig|6666666.28482.peg.880	Contig_12	69952	68333	-	Long-chain-fatty-acid--CoA ligase (EC 6.2.1.3)	FIG00018699	idu(72);n-Phenylalkanoic_acid_degradation idu(72);Fatty_acid_metabolism_cluster idu(72);Biotin_synthesis_cluster idu(72);Biotin_biosynthesis
fig|6666666.28482.peg.881	Contig_12	71177	69975	-	BarH	FIG00668014	ff
fig|6666666.28482.peg.882	Contig_12	72063	71209	-	Enoyl-CoA hydratase (EC 4.2.1.17)	FIG00033206	icw(1);n-Phenylalkanoic_acid_degradation idu(51);Polyhydroxybutyrate_metabolism icw(1);Isoleucine_degradation icw(1);Fatty_acid_metabolism_cluster icw(1);Lysine_fermentation icw(1);Butanol_Biosynthesis icw(1);Acetyl-CoA_fermentation_to_Butyrate icw(1);Valine_degradation idu(51);Butyrate_metabolism_cluster

fig|6666666.28482.peg.883	Contig_12	72541	72269	-	FIG00545001: hypothetical protein	FIG00544999	ff
fig|6666666.28482.peg.884	Contig_12	72684	72842	+	hypothetical protein		
fig|6666666.28482.peg.885	Contig_12	72937	73176	+	hypothetical protein		
fig|6666666.28482.peg.886	Contig_12	74265	73258	-	dihydrodipicolinate synthetase	FIG01465979	ff
fig|6666666.28482.peg.887	Contig_12	75097	74300	-	3-oxoacyl-[acyl-carrier protein] reductase (EC 1.1.1.100)	FIG00621114	idu(38);CBSS-246196.1.peg.364 idu(38);CBSS-164757.7.peg.5020 idu(38);Fatty_Acid_Biosynthesis_FASI I
fig|6666666.28482.peg.888	Contig_12	75252	76043	+	Transcriptional regulator, TetR family	FIG00554417	ff
fig|6666666.28482.peg.889	Contig_12	77130	76225	-	Enoyl-CoA hydratase (EC 4.2.1.17)	FIG00033206	icw(4);n-Phenylalkanoic_acid_degradation icw(3);Polyhydroxybutyrate_metaboli sm icw(3);Isoleucine_degradation icw(4);Fatty_acid_metabolism_cluste r icw(3);Lysine_fermentation icw(3);Butanol_Biosynthesis icw(3);Acetyl-CoA_fermentation_to_Butyrate icw(3);Valine_degradation icw(3);Butyrate_metabolism_cluster
fig|6666666.28482.peg.890	Contig_12	77810	77127	-	FIG00822114: hypothetical protein	FIG00822113	ff

fig|6666666.28482.peg.891	Contig_12	77942	78763	+	Enoyl-CoA hydratase (EC 4.2.1.17)	FIG00033206	icw(5);n-Phenylalkanoic_acid_degradation icw(3);Polyhydroxybutyrate_metabolism icw(3);Isoleucine_degradation icw(5);Fatty_acid_metabolism_cluster icw(3);Lysine_fermentation icw(3);Butanol_Biosynthesis icw(3);Acetyl-CoA_fermentation_to_Butyrate icw(3);Valine_degradation icw(3);Butyrate_metabolism_cluster
fig|6666666.28482.peg.892	Contig_12	78774	79586	+	Enoyl-CoA hydratase (EC 4.2.1.17)	FIG00821959	icw(6);n-Phenylalkanoic_acid_degradation icw(3);Polyhydroxybutyrate_metabolism icw(3);Isoleucine_degradation icw(6);Fatty_acid_metabolism_cluster icw(3);Lysine_fermentation icw(3);Butanol_Biosynthesis icw(3);Acetyl-CoA_fermentation_to_Butyrate icw(3);Valine_degradation icw(3);Butyrate_metabolism_cluster

fig|6666666.28482.peg.893	Contig_12	79577	80377	+	Enoyl-CoA hydratase (EC 4.2.1.17)	FIG00033206	icw(6);n-Phenylalkanoic_acid_degradation icw(3);Polyhydroxybutyrate_metabolism icw(3);Isoleucine_degradation icw(6);Fatty_acid_metabolism_cluster icw(3);Lysine_fermentation icw(3);Butanol_Biosynthesis icw(3);Acetyl-CoA_fermentation_to_Butyrate icw(3);Valine_degradation icw(3);Butyrate_metabolism_cluster
fig|6666666.28482.peg.894	Contig_12	80374	81837	+	Long-chain-fatty-acid--CoA ligase (EC 6.2.1.3)	FIG00018699	icw(2);n-Phenylalkanoic_acid_degradation icw(2);Fatty_acid_metabolism_cluster icw(2);Biotin_synthesis_cluster icw(2);Biotin_biosynthesis
fig|6666666.28482.peg.895	Contig_12	81902	83368	+	Long-chain-fatty-acid--CoA ligase (EC 6.2.1.3)	FIG00018699	icw(2);n-Phenylalkanoic_acid_degradation icw(2);Fatty_acid_metabolism_cluster icw(2);Biotin_synthesis_cluster icw(2);Biotin_biosynthesis

fig|6666666.28482.peg.896	Contig_12	83361	84893	+	Long-chain-fatty-acid--CoA ligase (EC 6.2.1.3)	FIG00018699	icw(2);n-Phenylalkanoic_acid_degradation icw(2);Fatty_acid_metabolism_cluster icw(2);Biotin_synthesis_cluster icw(2);Biotin_biosynthesis
fig|6666666.28482.peg.897	Contig_12	85210	84890	-	hypothetical protein		
fig|6666666.28482.peg.898	Contig_12	86024	85332	-	Predicted regulator PutR for proline utilization, GntR family	FIG00004161	idu(2);Proline,_4-hydroxyproline_uptake_and_utilization
fig|6666666.28482.peg.899	Contig_12	87222	86029	-	Butyryl-CoA dehydrogenase (EC 1.3.99.2)	FIG00009149	icw(1);Isoleucine_degradation icw(1);Lysine_fermentation icw(1);Butanol_Biosynthesis icw(1);Acetyl-CoA_fermentation_to_Butyrate icw(1);Valine_degradation icw(1);Anaerobic_respiratory_reductases
fig|6666666.28482.peg.900	Contig_12	88421	87219	-	FIG00827952: hypothetical protein	FIG00827950	ff
fig|6666666.28482.peg.901	Contig_12	89635	88418	-	beta-ketothiolase(EC:2.3.1.9)		
fig|6666666.28482.peg.902	Contig_12	90390	89650	-	3-oxoacyl-[acyl-carrier protein] reductase (EC 1.1.1.100)	FIG00621114	idu(38);CBSS-246196.1.peg.364 idu(38);CBSS-164757.7.peg.5020 idu(38);Fatty_Acid_Biosynthesis_FASII

fig|6666666.28482.peg.903	Contig_12	90525	91697	+	Butyryl-CoA dehydrogenase (EC 1.3.99.2)	FIG00009149	icw(1);Isoleucine_degradation icw(1);Lysine_fermentation icw(1);Butanol_Biosynthesis icw(1);Acetyl-CoA_fermentation_to_Butyrate icw(1);Valine_degradation icw(1);Anaerobic_respiratory_reductases
fig|6666666.28482.peg.904	Contig_12	91694	92515	+	FIG00825648: hypothetical protein	FIG00825647	ff
fig|6666666.28482.peg.905	Contig_12	92546	92989	+	FIG00825123: hypothetical protein	FIG00825122	ff
fig|6666666.28482.peg.906	Contig_12	94062	92986	-	FIG00828143: hypothetical protein	FIG00828140	ff
fig|6666666.28482.peg.907	Contig_12	95879	94131	-	ABC transporter ATP-binding protein	FIG01956052	ff
fig|6666666.28482.peg.908	Contig_12	98446	95876	-	ABC transporter ATP-binding protein	FIG01956052	ff
fig|6666666.28482.peg.909	Contig_12	99939	98707	-	POSSIBLE LINOLEOYL-CoA DESATURASE (DELTA(6)-DESATURASE)	FIG00539310	ff
fig|6666666.28482.peg.910	Contig_12	101075	99948	-	Flavodoxin reductases (ferredoxin-NADPH reductases) family 1	FIG01954108	idu(3);Anaerobic_respiratory_reductases
fig|6666666.28482.peg.911	Contig_12	101350	102261	+	hypothetical protein	FIG00638284	ff
fig|6666666.28482.peg.912	Contig_12	102561	102262	-	FIG00823680: hypothetical protein	FIG00823679	ff
fig|6666666.28482.peg.913	Contig_12	103090	102662	-	FIG00672531: hypothetical protein	FIG01121505	ff
fig|6666666.28482.rna.9	Contig_12	103383	103300	-	tRNA-Leu-TAG		
fig|6666666.28482.peg.914	Contig_12	103455	103835	+	Lipoprotein LprD	FIG00861686	ff

fig|6666666.28482.peg.915	Contig_12	103832	104173	+	CONSERVED MEMBRANE PROTEIN	FIG00818075	ff
fig|6666666.28482.peg.916	Contig_12	104931	104326	-	Nucleoside 5-triphosphatase RdgB (dHAPTP, dITP, XTP-specific) (EC 3.6.1.15)	FIG00000584	isu;Heat_shock_dnaK_gene_cluster_extended isu;Housecleaning_nucleoside_triphosphate_pyrophosphatases
fig|6666666.28482.peg.917	Contig_12	105720	104941	-	Ribonuclease PH (EC 2.7.7.56)	FIG00135600	isu;tRNA_processing icw(1);Heat_shock_dnaK_gene_cluster_extended
fig|6666666.28482.peg.918	Contig_12	106530	105748	-	Metal-dependent hydrolases of the beta-lactamase superfamily III	FIG00113090	isu;Beta-lactamase
fig|6666666.28482.peg.919	Contig_12	107417	106602	-	Glutamate racemase (EC 5.1.1.3)	FIG00000397	isu;Glutamine,_Glutamate,_Aspartate_and_Asparagine_Biosynthesis isu;Poly-gamma-glutamate_biosynthesis isu;Peptidoglycan_Biosynthesis
fig|6666666.28482.peg.920	Contig_12	108076	107414	-	Rhomboid membrane family protein	FIG00824237	ff
fig|6666666.28482.peg.921	Contig_12	109289	108249	-	possible hydrolase	FIG01037871	ff
fig|6666666.28482.peg.922	Contig_12	109890	109306	-	Transcriptional regulatory protein	FIG00447785	ff
fig|6666666.28482.peg.923	Contig_12	110161	109916	-	ATP-dependent Clp protease adaptor protein ClpS	FIG00018741	isu;Proteolysis_in_bacteria,_ATP-dependent isu;ClpAS_cluster
fig|6666666.28482.peg.924	Contig_12	110277	111575	+	Nicotinate phosphoribosyltransferase (EC 2.4.2.11)	FIG00000387	isu;NAD_and_NADP_cofactor_biosynthesis_global isu;Redox-dependent_regulation_of_nucleus_processes
fig|6666666.28482.peg.925	Contig_12	111587	113590	+	DinG family ATP-dependent helicase YoaA	FIG00000680	isu;DNA_repair,_bacterial_DinG_and_relatives

fig|6666666.28482.peg.926	Contig_12	116201	113574	-	Glycogen phosphorylase (EC 2.4.1.1)	FIG00000476	icw(1);Glycogen_metabolism idu(1);Maltose_and_Maltodextrin_Utilization
fig|6666666.28482.peg.927	Contig_12	116424	118517	+	Putative glucanase glgE (EC 3.2.1.-)	FIG00143078	isu;Trehalose_Biosynthesis
fig|6666666.28482.peg.928	Contig_12	118524	120719	+	1,4-alpha-glucan (glycogen) branching enzyme, GH-13-type (EC 2.4.1.18)	FIG00024930	icw(1);Trehalose_Biosynthesis isu;Glycogen_metabolism
fig|6666666.28482.peg.929	Contig_12	121649	120747	-	COG3118: Thioredoxin domain-containing protein EC-YbbN	FIG00000875	ff

fig|6666666.28482.peg.930	Contig_12	122987	121785	-	3-ketoacyl-CoA thiolase (EC 2.3.1.16) @ Acetyl-CoA acetyltransferase (EC 2.3.1.9)	FIG00000004	idu(11);CBSS-246196.1.peg.364 idu(9);Serine-glyoxylate_cycle idu(11);Serine-glyoxylate_cycle idu(11);n-Phenylalkanoic_acid_degradation idu(9);Ethylmalonyl-CoA_pathway_of_C2_assimilation,_GJO idu(9);Polyhydroxybutyrate_metabolism idu(11);Polyhydroxybutyrate_metabolism idu(11);Isoleucine_degradation idu(11);Fatty_acid_metabolism_cluster idu(11);Biotin_synthesis_cluster idu(11);Biotin_biosynthesis idu(9);Lysine_fermentation idu(9);Isoprenoid_Biosynthesis idu(9);Butanol_Biosynthesis idu(9);Acetyl-CoA_fermentation_to_Butyrate idu(9);Butyrate_metabolism_cluster
fig|6666666.28482.peg.931	Contig_12	123096	123503	+	Methylmalonyl-CoA epimerase (EC 5.1.99.1); Ethylmalonyl-CoA epimerase	FIG00019870	icw(1);Ethylmalonyl-CoA_pathway_of_C2_assimilation,_GJO
fig|6666666.28482.peg.932	Contig_12	123821	123591	-	FIG00997641: hypothetical protein	FIG00997640	ff
fig|6666666.28482.peg.933	Contig_12	124555	123884	-	FIG00996186: hypothetical protein	FIG00945501	ff

fig|6666666.28482.peg.934	Contig_12	124601	126214	+	Adenylate cyclase (EC 4.6.1.1)	FIG00001294	idu(25);cAMP_signaling_in_bacteria
fig|6666666.28482.peg.935	Contig_12	126433	127764	+	Oxalate/formate antiporter	FIG01351990	ff
fig|6666666.28482.peg.936	Contig_12	129032	127743	-	Major facilitator superfamily precursor	FIG01057154	ff
fig|6666666.28482.peg.937	Contig_12	129189	130076	+	transcriptional regulator, LysR family protein		
fig|6666666.28482.peg.938	Contig_12	130632	130033	-	Transcriptional regulator, TetR family	FIG00554417	ff
fig|6666666.28482.peg.939	Contig_12	130879	131433	+	pyridoxamine 5'-phosphate oxidase-related FMN-binding	FIG01780640	ff
fig|6666666.28482.peg.940	Contig_12	131446	132750	+	hypothetical protein		
fig|6666666.28482.peg.941	Contig_12	132819	134306	+	Methylated-DNA--protein-cysteine methyltransferase (EC 2.1.1.63)	FIG00000328	icw(1);DNA_repair,_bacterial
fig|6666666.28482.peg.942	Contig_12	134303	134794	+	Methylated-DNA--protein-cysteine methyltransferase (EC 2.1.1.63)	FIG00000328	icw(1);DNA_repair,_bacterial
fig|6666666.28482.rna.10	Contig_12	134955	134840	-	5S RNA		
fig|6666666.28482.rna.11	Contig_12	138134	134998	-	Large Subunit Ribosomal RNA; lsuRNA; LSU rRNA		
fig|6666666.28482.rna.12	Contig_12	139942	138419	-	Small Subunit Ribosomal RNA; ssuRNA; SSU rRNA		

fig|6666666.28482.peg.943	Contig_12	141572	140319	-	UDP-N-acetylglucosamine 1-carboxyvinyltransferase (EC 2.5.1.7)	FIG00034392	isu;UDP-N-acetylmuramate_from_Fructose-6-phosphate_Biosynthesis isu;Peptidoglycan_Biosynthesis
fig|6666666.28482.peg.944	Contig_12	141645	142226	+	Cob(I)alamin adenosyltransferase PduO (EC 2.5.1.17)	FIG00001045	isu;Glycerol_fermenation_to_1,3-propanediol isu;Coenzyme_B12_biosynthesis isu;G3E_family_of_P-loop_GTPases_(metallocenter_biosynthesis)
fig|6666666.28482.peg.945	Contig_12	142735	142304	-	Possible membrane protein	FIG01121868	ff
fig|6666666.28482.peg.946	Contig_12	143119	142754	-	ATP synthase epsilon chain (EC 3.6.3.14)	FIG00000249	icw(3);F0F1-type_ATP_synthase
fig|6666666.28482.peg.947	Contig_12	144612	143146	-	ATP synthase beta chain (EC 3.6.3.14)	FIG00040241	icw(2);F0F1-type_ATP_synthase
fig|6666666.28482.peg.948	Contig_12	145549	144632	-	ATP synthase gamma chain (EC 3.6.3.14)	FIG00023994	icw(4);F0F1-type_ATP_synthase
fig|6666666.28482.peg.949	Contig_12	147229	145556	-	ATP synthase alpha chain (EC 3.6.3.14)	FIG00000082	icw(5);F0F1-type_ATP_synthase
fig|6666666.28482.peg.950	Contig_12	148623	147283	-	ATP synthase B' chain (EC 3.6.3.14) / ATP synthase delta chain (EC 3.6.3.14)	FIG01305175	icw(1);F0F1-type_ATP_synthase icw(4);F0F1-type_ATP_synthase
fig|6666666.28482.peg.951	Contig_12	149129	148629	-	ATP synthase B chain (EC 3.6.3.14)	FIG00000186	icw(2);F0F1-type_ATP_synthase
fig|6666666.28482.peg.952	Contig_12	149402	149157	-	ATP synthase C chain (EC 3.6.3.14)	FIG00017607	isu;F0F1-type_ATP_synthase
fig|6666666.28482.peg.953	Contig_12	150204	149449	-	ATP synthase A chain (EC 3.6.3.14)	FIG00000138	icw(5);F0F1-type_ATP_synthase
fig|6666666.28482.peg.954	Contig_12	150664	150197	-	hypothetical protein		

fig|6666666.28482.peg.955	Contig_12	152094	150913	-	Undecaprenyl-phosphate N-acetylglucosaminyl 1-phosphate transferase (EC 2.7.8.-)	FIG01955782	isu;Methicillin_resistance_in_Staphylococci isu;Teichoic_and_lipoteichoic_acids_biosynthesis
fig|6666666.28482.peg.956	Contig_12	152794	152141	-	TsaC protein (YrdC domain) required for threonylcarbamoyladenosine t(6)A37 modification in tRNA		
fig|6666666.28482.peg.957	Contig_12	153669	152791	-	Methylase of polypeptide chain release factors	FIG00003217	ff
fig|6666666.28482.peg.958	Contig_12	154756	153683	-	Peptide chain release factor 1	FIG00000188	isu;Translation_termination_factors_bacterial
fig|6666666.28482.peg.959	Contig_12	155077	154835	-	LSU ribosomal protein L31p	FIG00000092	isu;Ribosome_LSU_bacterial
fig|6666666.28482.peg.960	Contig_12	155060	155245	+	hypothetical protein		
fig|6666666.28482.peg.961	Contig_12	156801	155221	-	Long-chain-fatty-acid--CoA ligase (EC 6.2.1.3)	FIG00018699	idu(72);n-Phenylalkanoic_acid_degradation idu(72);Fatty_acid_metabolism_cluster idu(72);Biotin_synthesis_cluster idu(72);Biotin_biosynthesis
fig|6666666.28482.peg.962	Contig_12	158589	156814	-	Transcription termination factor Rho	FIG00000392	isu;Transcription_factors_bacterial
fig|6666666.28482.peg.963	Contig_12	159860	158913	-	Homoserine kinase (EC 2.7.1.39)	FIG00000582	isu;CBSS-269482.1.peg.1294 icw(2);Threonine_and_Homoserine_Biosynthesis
fig|6666666.28482.peg.964	Contig_12	160904	159864	-	Threonine synthase (EC 4.2.3.1)	FIG00000134	icw(1);Threonine_and_Homoserine_Biosynthesis

fig|6666666.28482.peg.965	Contig_12	162268	160943	-	Homoserine dehydrogenase (EC 1.1.1.3)	FIG00000434	isu;Threonine_and_Homoserine_Biosynthesis
fig|6666666.28482.peg.966	Contig_12	163690	162272	-	Diaminopimelate decarboxylase (EC 4.1.1.20)	FIG00138250	idu(2);CBSS-323850.3.peg.3269 idu(2);Lysine_Biosynthesis_DAP_Pathway,_GJO_scratch idu(2);Lysine_Biosynthesis_DAP_Pathway
fig|6666666.28482.peg.967	Contig_12	165339	163687	-	Arginyl-tRNA synthetase (EC 6.1.1.19)	FIG00000088	isu;tRNA_aminoacylation,_Arg
fig|6666666.28482.peg.968	Contig_12	166575	165433	-	low-specificity D-threonine aldolase	FIG00140689	isu;Serine-glyoxylate_cycle isu;Threonine_degradation
fig|6666666.28482.peg.969	Contig_12	167018	166572	-	tautomerase, putative		
fig|6666666.28482.peg.970	Contig_12	167126	168094	+	transcriptional regulator, AraC family		
fig|6666666.28482.rna.13	Contig_12	168169	168241	+	tRNA-Arg-CCG		isu;tRNAs
fig|6666666.28482.peg.971	Contig_12	168490	169260	+	hypothetical protein	FIG00638284	ff
fig|6666666.28482.peg.972	Contig_12	169650	169405	-	site-specific recombinase, phage integrase family protein		
fig|6666666.28482.peg.973	Contig_12	170178	169873	-	hypothetical protein		
fig|6666666.28482.peg.974	Contig_12	170409	171065	+	cutinase Cut1	FIG01392700	ff
fig|6666666.28482.peg.975	Contig_12	171304	171495	+	hypothetical protein		
fig|6666666.28482.peg.976	Contig_12	171944	171528	-	FIG00825572: hypothetical protein	FIG00825568	ff
fig|6666666.28482.peg.977	Contig_12	171943	172065	+	hypothetical protein		
fig|6666666.28482.peg.978	Contig_12	172763	172062	-	hypothetical protein		
fig|6666666.28482.peg.979	Contig_12	173019	172822	-	hypothetical protein		
fig|6666666.28482.peg.980	Contig_12	173180	173034	-	hypothetical protein		
fig|6666666.28482.peg.981	Contig_12	173376	173825	+	Transcriptional regulator, MarR family		

fig|6666666.28482.peg.982	Contig_12	173901	174278	+	putative integral membrane protein		
fig|6666666.28482.peg.983	Contig_12	174820	174275	-	Putative carboxymuconolactone decarboxylase		
fig|6666666.28482.peg.984	Contig_12	174904	175692	+	3-oxoacyl-[acyl-carrier protein] reductase (EC 1.1.1.100)	FIG00621114	icw(1);CBSS-246196.1.peg.364 icw(1);CBSS-164757.7.peg.5020 icw(1);Fatty_Acid_Biosynthesis_FASII
fig|6666666.28482.peg.985	Contig_12	176751	175939	-	3-oxoacyl-[acyl-carrier protein] reductase (EC 1.1.1.100)	FIG00621114	icw(1);CBSS-246196.1.peg.364 icw(1);CBSS-164757.7.peg.5020 icw(1);Fatty_Acid_Biosynthesis_FASII
fig|6666666.28482.peg.986	Contig_12	176836	177825	+	dTDP-glucose 4,6-dehydratase (EC 4.2.1.46)	FIG00030688	idu(4);dTDP-rhamnose_synthesis idu(4);Rhamnose_containing_glycans
fig|6666666.28482.peg.987	Contig_12	177918	178376	+	Transcriptional regulator, TetR family		
fig|6666666.28482.peg.988	Contig_12	178894	178364	-	hypothetical protein	FIG00638284	ff
fig|6666666.28482.peg.989	Contig_12	179476	178925	-	ADP-ribose pyrophosphatase (EC 3.6.1.13)	FIG00000367	idu(1);NAD_and_NADP_cofactor_biosynthesis_global idu(1);CBSS-216591.1.peg.168 idu(1);Nudix_proteins_(nucleoside_triphosphate_hydrolases) idu(1);Ribosome_post-transcriptional_modification_and_chromosomal_segregation_cluster idu(1);CBSS-342610.3.peg.1794
fig|6666666.28482.peg.990	Contig_12	179499	179870	+	FIG00830738: hypothetical protein	FIG00830734	ff
fig|6666666.28482.peg.991	Contig_12	179969	179844	-	hypothetical protein		

fig|6666666.28482.peg.992	Contig_12	179958	181673	+	Potassium channel protein	FIG00006661	idu(2);Potassium_homeostasis
fig|6666666.28482.peg.993	Contig_12	182560	181670	-	FIG00824659: hypothetical protein	FIG00824655	ff
fig|6666666.28482.peg.994	Contig_12	182579	183001	+	glyoxalase/bleomycin resistance protein/dioxygenase	FIG00821958	ff
fig|6666666.28482.peg.995	Contig_12	183107	183496	+	FIG00820839: hypothetical protein	FIG00820835	ff
fig|6666666.28482.peg.996	Contig_12	183978	183493	-	Predicted transcriptional regulator of sulfate adenylyltransferase, Rrf2 family	FIG00011844	isu;Rrf2_family_transcriptional_regulators
fig|6666666.28482.peg.997	Contig_12	185852	184011	-	Sulfate adenylyltransferase subunit 1 (EC 2.7.7.4) / Adenylylsulfate kinase (EC 2.7.1.25)	FIG00133521	idu(1);Cysteine_Biosynthesis icw(1);Cysteine_Biosynthesis idu(1);O-Methyl_Phosphoramidate_Capsule_Modification_in_Campylobacter idu(1);Inorganic_Sulfur_Assimilation icw(1);Inorganic_Sulfur_Assimilation
fig|6666666.28482.peg.998	Contig_12	186781	185852	-	Sulfate adenylyltransferase subunit 2 (EC 2.7.7.4)	FIG00000728	icw(1);Cysteine_Biosynthesis icw(1);Inorganic_Sulfur_Assimilation
fig|6666666.28482.peg.999	Contig_12	186949	187386	+	FIG00820608: hypothetical protein	FIG00820606	ff
fig|6666666.28482.peg.1000	Contig_12	187947	187456	-	Carbonic anhydrase (EC 4.2.1.1)	FIG00023019	idu(1);Cyanate_hydrolysis

fig|6666666.28482.peg.1001	Contig_12	188084	189061	+	Oligopeptide transport system permease protein OppB (TC 3.A.1.5.1)	FIG00945384	icw(2);ABC_transporter_oligopeptide_(TC_3.A.1.5.1)
fig|6666666.28482.peg.1002	Contig_12	189058	189972	+	Oligopeptide transport system permease protein OppC (TC 3.A.1.5.1)	FIG00455853	isu;ABC_transporter_oligopeptide_(TC_3.A.1.5.1)
fig|6666666.28482.peg.1003	Contig_12	189969	191810	+	Oligopeptide transport system permease protein OppB (TC 3.A.1.5.1)	FIG00945384	icw(2);ABC_transporter_oligopeptide_(TC_3.A.1.5.1)
fig|6666666.28482.peg.1004	Contig_12	191896	193539	+	"Oligopeptide ABC transporter, periplasmic oligopeptide-binding protein OppA (TC 3.A.1.5.1)"	FIG01307132	ff
fig|6666666.28482.peg.1005	Contig_12	194773	193526	-	FIG00824587: hypothetical protein	FIG00824586	ff
fig|6666666.28482.peg.1006	Contig_12	197439	194818	-	DNA double-strand break repair Rad50 ATPase	FIG00944792	isu;Rad50-Mre11_DNA_repair_cluster
fig|6666666.28482.peg.1007	Contig_12	198572	197436	-	DNA double-strand break repair protein Mre11	FIG00614964	icw(1);Rad50-Mre11_DNA_repair_cluster
fig|6666666.28482.peg.1008	Contig_120	38	391	+	Possible membrane protein	FIG01121868	ff
fig|6666666.28482.peg.1009	Contig_120	399	1295	+	FIG00821563: hypothetical protein	FIG00821562	ff
fig|6666666.28482.peg.1010	Contig_120	1407	1997	+	Immunogenic protein Mpt70	FIG01320678	ff

fig|6666666.28482.peg.1011	Contig_120	2052	2543	+	FIG00824620: hypothetical protein	FIG00824619	ff
fig|6666666.28482.peg.1012	Contig_120	2540	3637	+	FIG00822822: hypothetical protein	FIG00822820	ff
fig|6666666.28482.peg.1013	Contig_120	3634	4755	+	hypothetical protein		
fig|6666666.28482.peg.1014	Contig_120	4752	6101	+	FIG01124584: hypothetical protein	FIG00863066	ff
fig|6666666.28482.peg.1015	Contig_120	6563	6105	-	hypothetical protein		
fig|6666666.28482.peg.1016	Contig_120	7315	6560	-	Beta-lactamase-like protein	FIG01478403	ff
fig|6666666.28482.peg.1017	Contig_120	7357	8367	+	F420-dependent glucose-6-phosphate dehydrogenase	FIG01001620	ff
fig|6666666.28482.peg.1018	Contig_120	8360	10444	+	Phosphate acetyltransferase (EC 2.3.1.8)	FIG00000522	icw(1);Pyruvate_metabolism_II:_acetyl-CoA,_acetogenesis_from_pyruvate isu;Ethanolamine_utilization isu;Fermentations:_Lactate isu;Fermentations:_Mixed_acid isu;Propanediol_utilization isu;Threonine_anaerobic_catabolism_gene_cluster
fig|6666666.28482.peg.1019	Contig_120	10458	11594	+	Acetate kinase (EC 2.7.2.1)	FIG00067248	isu;Pyruvate_metabolism_II:_acetyl-CoA,_acetogenesis_from_pyruvate icw(1);Ethanolamine_utilization icw(1);Fermentations:_Lactate icw(1);Fermentations:_Mixed_acid isu;CBSS-257314.1.peg.752 icw(1);Threonine_anaerobic_catabolism_gene_cluster

fig|6666666.28482.peg.1020	Contig_120	12776	11583	-	FIG00828616: hypothetical protein	FIG00828614	ff
fig|6666666.28482.peg.1021	Contig_120	14101	12779	-	FIG00825805: hypothetical protein	FIG00825804	ff
fig|6666666.28482.peg.1022	Contig_120	14283	15134	+	FIG00821524: hypothetical protein	FIG00821522	ff
fig|6666666.28482.peg.1023	Contig_120	15304	15188	-	hypothetical protein		
fig|6666666.28482.peg.1024	Contig_120	17587	15329	-	serine/threonine protein kinase	FIG00723707	ff
fig|6666666.28482.peg.1025	Contig_120	18549	17587	-	"Glutamine ABC transporter, periplasmic glutamine-binding protein (TC 3.A.1.3.2)"	FIG00545941	ff
fig|6666666.28482.peg.1026	Contig_120	19865	18546	-	Possible membrane protein	FIG01121868	ff
fig|6666666.28482.peg.1027	Contig_120	19989	20621	+	"Possible mutator protein MutT3 (7,8-dihydro-8-oxoguanine-triphosphatase) "	FIG00994542	ff
fig|6666666.28482.peg.1028	Contig_120	21261	20593	-	Thiamin-phosphate pyrophosphorylase (EC 2.5.1.3)	FIG00000407	icw(2);Thiamin_biosynthesis
fig|6666666.28482.peg.1029	Contig_120	21391	22398	+	Glycine oxidase ThiO (EC 1.4.3.19)	FIG00001017	isu;Thiamin_biosynthesis
fig|6666666.28482.peg.1030	Contig_120	22395	22592	+	Sulfur carrier protein ThiS	FIG00058747	icw(3);Thiamin_biosynthesis
fig|6666666.28482.peg.1031	Contig_120	22696	23400	+	Thiazole biosynthesis protein ThiG	FIG00000563	icw(1);Thiamin_biosynthesis
fig|6666666.28482.peg.1032	Contig_120	23409	24164	+	putative secreted hydrolase	FIG01121198	ff
fig|6666666.28482.peg.1033	Contig_120	24693	24169	-	FIG00825681: hypothetical protein	FIG00825678	ff

fig|6666666.28482.peg.1034	Contig_120	25274	24747	-	FIG00825681: hypothetical protein	FIG00825678	ff
fig|6666666.28482.peg.1035	Contig_120	25694	25945	+	FIG00828114: hypothetical protein	FIG00828109	ff
fig|6666666.28482.peg.1036	Contig_120	25994	26386	+	FIG00828332: hypothetical protein	FIG00828331	ff
fig|6666666.28482.peg.1037	Contig_120	26406	27860	+	Aminopeptidase Y (Arg, Lys, Leu preference) (EC 3.4.11.15)	FIG00004058	isu;Aminopeptidases_(EC_3.4.11.-)
fig|6666666.28482.peg.1038	Contig_120	27857	29329	+	Aminopeptidase Y precursor (EC 3.4.11.-)	FIG00824150	ff
fig|6666666.28482.peg.1039	Contig_121	1018	1467	+	FIG00825618: hypothetical protein	FIG00825617	ff
fig|6666666.28482.peg.1040	Contig_121	1580	2710	+	2-nitropropane dioxygenase (EC 1.13.11.32)	FIG01304356	idu(5);CBSS-164757.7.peg.5020
fig|6666666.28482.peg.1041	Contig_121	2959	2717	-	FIG00822024: hypothetical protein	FIG00822023	ff
fig|6666666.28482.peg.1042	Contig_121	3314	3024	-	Possible membrane protein	FIG01121868	ff
fig|6666666.28482.peg.1043	Contig_121	3737	3327	-	Possible membrane protein	FIG01121868	ff
fig|6666666.28482.peg.1044	Contig_121	4362	3775	-	Dihydrofolate reductase (EC 1.5.1.3)	FIG00000048	idu(3);Folate_Biosynthesis
fig|6666666.28482.peg.1045	Contig_121	4550	5827	+	Cytochrome P450 124	FIG00821294	ff
fig|6666666.28482.peg.1046	Contig_122	608	769	+	hypothetical protein		
fig|6666666.28482.peg.1047	Contig_122	915	2669	+	drug transporter		
fig|6666666.28482.peg.1048	Contig_122	6225	2833	-	Superfamily I DNA and RNA helicases and helicase subunits	FIG01310944	ff
fig|6666666.28482.peg.1049	Contig_122	6393	6752	+	hypothetical protein		

fig|6666666.28482.peg.1050	Contig_123	228	632	+	FIG00822480: hypothetical protein	FIG00822479	ff
fig|6666666.28482.peg.1051	Contig_123	1365	646	-	Cell division protein DivIC (FtsB), stabilizes FtsL against RasP cleavage	FIG00485194	idu(10);Possible_new_toxin-antitoxin_system_including_DivIC idu(10);Bacterial_Cell_Division idu(10);Cell_division-ribosomal_stress_proteins_cluster idu(10);Bacterial_Cytoskeleton idu(10);Stationary_phase_repair_cluster
fig|6666666.28482.peg.1052	Contig_123	1413	2438	+	Quinone oxidoreductase (EC 1.6.5.5)	FIG00002982	idu(7);Quinone_oxidoreductase_family
fig|6666666.28482.peg.1053	Contig_123	3055	2435	-	hypothetical protein		
fig|6666666.28482.peg.1054	Contig_123	3174	3626	+	Transcriptional regulator, MarR family		
fig|6666666.28482.peg.1055	Contig_123	3784	4617	+	FIG00820744: hypothetical protein	FIG00820743	ff
fig|6666666.28482.peg.1056	Contig_123	6147	4618	-	FIG00827250: hypothetical protein	FIG00827249	ff
fig|6666666.28482.peg.1057	Contig_123	6412	6182	-	hypothetical protein		
fig|6666666.28482.peg.1058	Contig_123	6519	6824	+	FIG00820008: hypothetical protein	FIG00820007	ff
fig|6666666.28482.peg.1059	Contig_123	6845	7516	+	FIG00821508: hypothetical protein	FIG00821507	ff
fig|6666666.28482.peg.1060	Contig_123	7543	8223	+	FIG00823582: hypothetical protein	FIG00823581	ff
fig|6666666.28482.peg.1061	Contig_123	8939	8214	-	FIG00822574: hypothetical protein	FIG00822573	ff
fig|6666666.28482.peg.1062	Contig_123	8964	10460	+	INTEGRAL MEMBRANE PROTEIN (Rhomboid family)	FIG00525068	ff

fig|6666666.28482.peg.1063	Contig_123	10521	11303	+	3-oxoacyl-[acyl-carrier protein] reductase (EC 1.1.1.100)	FIG00621114	idu(38);CBSS-246196.1.peg.364 idu(38);CBSS-164757.7.peg.5020 idu(38);Fatty_Acid_Biosynthesis_FASII
fig|6666666.28482.peg.1064	Contig_123	11480	12661	+	PE-PGRS FAMILY PROTEIN		
fig|6666666.28482.peg.1065	Contig_123	13177	12674	-	PE_PGRS family protein		
fig|6666666.28482.peg.1066	Contig_123	14295	13312	-	Ketol-acid reductoisomerase (EC 1.1.1.86)		idu(1);Branched-Chain_Amino_Acid_Biosynthesis idu(1);Coenzyme_A_Biosynthesis
fig|6666666.28482.peg.1067	Contig_123	14444	15889	+	PE-PGRS family protein		idu(16);PPE_gene_cluster_in_Mycobacteria
fig|6666666.28482.peg.1068	Contig_123	15930	16526	+	probable cutinase(EC:3.1.1.74)		
fig|6666666.28482.peg.1069	Contig_123	18736	16532	-	hypothetical protein		
fig|6666666.28482.peg.1070	Contig_123	18837	19814	+	Methylthioribose-1-phosphate isomerase (EC 5.3.1.23)	FIG00001494	isu;Methionine_Salvage
fig|6666666.28482.rna.14	Contig_123	19905	19835	-	tRNA-Gly-CCC		isu;tRNAs
fig|6666666.28482.peg.1071	Contig_123	20403	20098	-	hypothetical protein		
fig|6666666.28482.peg.1072	Contig_123	20851	21378	+	Possible conserved exported protein	FIG01343981	ff
fig|6666666.28482.peg.1073	Contig_123	21420	21992	+	Deoxycytidine triphosphate deaminase (EC 3.5.4.13)	FIG00102132	isu;pyrimidine_conversions
fig|6666666.28482.peg.1074	Contig_123	22100	24046	+	FIG00825295: hypothetical protein	FIG00825293	ff
fig|6666666.28482.peg.1075	Contig_123	24318	25757	+	FIG00827027: hypothetical protein	FIG00827021	ff

fig|6666666.28482.peg.1076	Contig_123	25860	26972	+	FIG00830582: hypothetical protein	FIG00830581	ff
fig|6666666.28482.peg.1077	Contig_124	809	2368	+	CONSERVED MEMBRANE PROTEIN	FIG00818075	ff
fig|6666666.28482.peg.1078	Contig_124	2476	3324	+	FIG00821758: hypothetical protein	FIG00821757	ff
fig|6666666.28482.peg.1079	Contig_124	4590	3379	-	Arginine deiminase (EC 3.5.3.6)	FIG00001104	isu;Arginine_and_Ornithine_Degradation isu;Arginine_Deiminase_Pathway
fig|6666666.28482.peg.1080	Contig_124	4652	5257	+	Alkylated DNA repair protein AlkB	FIG00001574	isu;DNA_repair,_bacterial
fig|6666666.28482.peg.1081	Contig_124	6039	5383	-	FIG00820856: hypothetical protein	FIG00820854	ff
fig|6666666.28482.peg.1082	Contig_124	6741	6067	-	FIG00821923: hypothetical protein	FIG00821922	ff
fig|6666666.28482.peg.1083	Contig_124	7800	6748	-	cyclic nucleotide-binding protein	FIG01328426	ff
fig|6666666.28482.peg.1084	Contig_124	8427	7804	-	hypothetical protein	FIG00638284	ff
fig|6666666.28482.peg.1085	Contig_124	8565	9770	+	Type VII secretion protein EccE	FIG00822757	idu(1);PPE_gene_cluster_in_Mycobacteria
fig|6666666.28482.peg.1086	Contig_124	10679	9783	-	NAD-dependent epimerase/dehydratase		
fig|6666666.28482.peg.1087	Contig_124	12286	10691	-	4Fe-4S ferredoxin, iron-sulfur binding		idu(1);Inorganic_Sulfur_Assimilation
fig|6666666.28482.peg.1088	Contig_124	12602	13474	+	hypothetical protein	FIG00638284	ff
fig|6666666.28482.peg.1089	Contig_124	13679	13515	-	hypothetical protein		
fig|6666666.28482.peg.1090	Contig_124	13690	14550	+	acyltransferase 3		

fig|6666666.28482.peg.1091	Contig_124	14560	15615	+	Putative N-acetylgalactosaminyl-diphosphoundecaprenol glucuronosyltransferase		isu;Teichuronic_acid_biosynthesis
fig|6666666.28482.peg.1092	Contig_124	16227	15616	-	D-glycero-D-manno-heptose 1,7-bisphosphate phosphatase (EC 3.1.1.-)	FIG00001235	icw(1);LOS_core_oligosaccharide_biosynthesis icw(2);Capsular_heptose_biosynthesis
fig|6666666.28482.peg.1093	Contig_124	17438	16263	-	Glycosyl transferase, group 1		
fig|6666666.28482.peg.1094	Contig_124	18145	17507	-	Phosphoheptose isomerase 1 (EC 5.3.1.-)	FIG00002799	idu(2);LOS_core_oligosaccharide_biosynthesis idu(2);Capsular_heptose_biosynthesis
fig|6666666.28482.peg.1095	Contig_124	19101	18130	-	D,D-heptose 7-phosphate kinase	FIG00009038	icw(1);Capsular_heptose_biosynthesis
fig|6666666.28482.peg.1096	Contig_124	19650	19447	-	hypothetical protein		
fig|6666666.28482.peg.1097	Contig_124	19633	20478	+	hypothetical protein	FIG00638284	ff
fig|6666666.28482.peg.1098	Contig_124	20518	21546	+	hypothetical protein		
fig|6666666.28482.peg.1099	Contig_124	21715	22425	+	hypothetical protein		
fig|6666666.28482.peg.1100	Contig_124	22741	24909	+	O-antigen acetylase	FIG01370327	ff
fig|6666666.28482.peg.1101	Contig_124	24931	26205	+	hypothetical protein	FIG00638284	ff
fig|6666666.28482.peg.1102	Contig_124	26275	27126	+	Macrocin-O-methyltransferase		
fig|6666666.28482.peg.1103	Contig_124	27723	27154	-	hypothetical protein		
fig|6666666.28482.peg.1104	Contig_124	29915	27756	-	hypothetical protein	FIG00638284	ff
fig|6666666.28482.peg.1105	Contig_124	31333	29912	-	hypothetical protein	FIG00638284	ff

fig|6666666.28482.peg.1106	Contig_124	31523	32746	+	PROBABLE CONSERVED TRANSMEMBRANE PROTEIN	FIG00662132	ff
fig|6666666.28482.peg.1107	Contig_124	32748	33263	+	PROBABLE CONSERVED TRANSMEMBRANE PROTEIN		
fig|6666666.28482.peg.1108	Contig_124	33351	35126	+	hypothetical protein		
fig|6666666.28482.peg.1109	Contig_124	35152	35568	+	hypothetical protein		
fig|6666666.28482.peg.1110	Contig_124	36025	35609	-	Holo-[acyl-carrier protein] synthase (EC 2.7.8.7)		idu(1);Fatty_Acid_Biosynthesis_FASII
fig|6666666.28482.peg.1111	Contig_124	36283	36038	-	Acyl carrier protein	FIG00002328	icw(1);Fatty_Acid_Biosynthesis_FASII idu(2);Glycerolipid_and_Glycerophos pholipid_Metabolism_in_Bacteria
fig|6666666.28482.peg.1112	Contig_124	36505	37125	+	Transcriptional regulator	FIG01312420	ff
fig|6666666.28482.peg.1113	Contig_124	37153	37908	+	hypothetical protein		
fig|6666666.28482.peg.1114	Contig_124	38605	37925	-	serine esterase, cutinase family	FIG01349249	ff
fig|6666666.28482.peg.1115	Contig_124	38749	40257	+	Long-chain-fatty-acid--CoA ligase (EC 6.2.1.3)	FIG00018699	idu(72);n-Phenylalkanoic_acid_degradation idu(72);Fatty_acid_metabolism_clust er idu(72);Biotin_synthesis_cluster idu(72);Biotin_biosynthesis
fig|6666666.28482.peg.1116	Contig_124	40335	41723	+	coenzyme F390 synthetase-like protein		

fig|6666666.28482.peg.1117	Contig_124	43002	41725	-	O-acetylhomoserine sulfhydrylase (EC 2.5.1.49) / O-succinylhomoserine sulfhydrylase (EC 2.5.1.48)	FIG00000417	ff
fig|6666666.28482.peg.1118	Contig_124	43969	43022	-	Homoserine O-succinyltransferase(EC:2.3.1.46)		
fig|6666666.28482.peg.1119	Contig_124	45249	43975	-	hypothetical protein	FIG00638284	ff
fig|6666666.28482.peg.1120	Contig_124	47071	45254	-	hypothetical protein	FIG00638284	ff
fig|6666666.28482.peg.1121	Contig_124	48210	47068	-	Low-specificity L-threonine aldolase (EC 4.1.2.5)	FIG00000679	isu;Threonine_degradation isu;Glycine_Biosynthesis
fig|6666666.28482.peg.1122	Contig_125	1036	617	-	FIG00821456: hypothetical protein	FIG00821455	ff
fig|6666666.28482.peg.1123	Contig_126	3807	1186	-	hypothetical protein	FIG00638284	ff
fig|6666666.28482.peg.1124	Contig_126	4703	3897	-	Probable peptidyl-prolyl cis-trans isomerase B (EC 5.2.1.8) (PPIase B) (Rotamase B)	FIG01123881	ff
fig|6666666.28482.peg.1125	Contig_127	1006	86	-	tRNA delta(2)-isopentenylpyrophosphate transferase (EC 2.5.1.8)	FIG00000196	ff
fig|6666666.28482.peg.1126	Contig_127	1686	1003	-	FIG01121091: hypothetical protein	FIG00820084	ff
fig|6666666.28482.peg.1127	Contig_127	2660	1707	-	hypothetical protein	FIG00638284	ff
fig|6666666.28482.peg.1128	Contig_127	3286	4512	+	ATPase involved in DNA repair	FIG00945813	ff
fig|6666666.28482.peg.1129	Contig_127	5102	4509	-	Possible membrane protein	FIG01121868	ff

fig|6666666.28482.peg.1130	Contig_127	6544	5099	-	tRNA-i(6)A37 methylthiotransferase	FIG00108342	isu;tRNA_processing isu;Methylthiotransferases
fig|6666666.28482.peg.1131	Contig_127	7177	6659	-	Regulatory protein RecX	FIG00004099	icw(1);DNA_repair_system_including_RecA,_MutS_and_a_hypothetical_protein icw(1);RecA_and_RecX
fig|6666666.28482.peg.1132	Contig_127	8183	7143	-	RecA protein	FIG00000234	isu;DNA_repair,_bacterial_UmuCD_system isu;DNA-replication isu;DNA_repair_system_including_RecA,_MutS_and_a_hypothetical_protein isu;RecA_and_RecX isu;DNA_repair,_bacterial
fig|6666666.28482.peg.1133	Contig_127	8521	9192	+	FIG00826822: hypothetical protein	FIG00826821	ff
fig|6666666.28482.peg.1134	Contig_127	9487	9281	-	FIG00820724: hypothetical protein	FIG00820722	ff
fig|6666666.28482.peg.1135	Contig_127	10664	9498	-	Alanine rich transferase	FIG00821690	ff
fig|6666666.28482.peg.1136	Contig_127	10728	11159	+	FIG00820841: hypothetical protein	FIG00820839	ff
fig|6666666.28482.peg.1137	Contig_128	101	2179	+	DNA ligase (EC 6.5.1.2)	FIG00051439	isu;DNA_Repair_Base_Excision isu;CBSS-393121.3.peg.1913
fig|6666666.28482.peg.1138	Contig_128	2248	3300	+	Cystathionine gamma-lyase (EC 4.4.1.1)	FIG00001346	idu(1);Cysteine_Biosynthesis
fig|6666666.28482.peg.1139	Contig_128	3297	3971	+	hypothetical protein		
fig|6666666.28482.peg.1140	Contig_128	4642	3977	-	FIG00820558: hypothetical protein	FIG00820556	ff

fig|6666666.28482.peg.1141	Contig_128	4785	5084	+	Aspartyl-tRNA(Asn) amidotransferase subunit C (EC 6.3.5.6) @ Glutamyl-tRNA(Gln) amidotransferase subunit C (EC 6.3.5.7)	FIG00000432	isu;tRNA_aminoacylation,_Asp_and_Asn icw(2);tRNA_aminoacylation,_Glu_and_Gln
fig|6666666.28482.peg.1142	Contig_128	5081	6568	+	Aspartyl-tRNA(Asn) amidotransferase subunit A (EC 6.3.5.6) @ Glutamyl-tRNA(Gln) amidotransferase subunit A (EC 6.3.5.7)	FIG00000359	icw(2);tRNA_aminoacylation,_Asp_and_Asn isu;tRNA_aminoacylation,_Glu_and_Gln
fig|6666666.28482.peg.1143	Contig_128	6623	7654	+	6-phosphofructokinase (EC 2.7.1.11)	FIG00058830	isu;N-Acetyl-Galactosamine_and_Galactosamine_Utilization isu;D-Tagatose_and_Galactitol_Utilization
fig|6666666.28482.peg.1144	Contig_128	7783	8931	+	FIG00828598: hypothetical protein	FIG00828596	ff
fig|6666666.28482.peg.1145	Contig_128	9002	10513	+	Aspartyl-tRNA(Asn) amidotransferase subunit B (EC 6.3.5.6) @ Glutamyl-tRNA(Gln) amidotransferase subunit B (EC 6.3.5.7)	FIG00000399	icw(1);tRNA_aminoacylation,_Asp_and_Asn icw(1);tRNA_aminoacylation,_Glu_and_Gln
fig|6666666.28482.peg.1146	Contig_128	10531	11085	+	Oxidoreductase	FIG01321859	ff
fig|6666666.28482.peg.1147	Contig_128	12203	11082	-	Glucose/sorbosone dehydrogenase, lipoprotein LppZ	FIG01038209	ff

fig|6666666.28482.peg.1148	Contig_128	12508	13284	+	Membrane protein 2, distant similarity to thiosulphate:quinone oxidoreductase DoxD	FIG00006938	ff
fig|6666666.28482.peg.1149	Contig_128	13647	13288	-	Low molecular weight protein antigen 6	FIG01393835	ff
fig|6666666.28482.peg.1150	Contig_128	14044	13901	-	hypothetical protein		
fig|6666666.28482.peg.1151	Contig_128	14016	15884	+	Acetolactate synthase large subunit (EC 2.2.1.6)	FIG00136692	isu;Acetolactate_synthase_subunits icw(1);Branched-Chain_Amino_Acid_Biosynthesis isu;Acetoin,_butanediol_metabolism
fig|6666666.28482.peg.1152	Contig_128	15884	16384	+	Acetolactate synthase small subunit (EC 2.2.1.6)	FIG00000433	icw(1);Acetolactate_synthase_subunits icw(2);Branched-Chain_Amino_Acid_Biosynthesis icw(1);Acetoin,_butanediol_metabolism
fig|6666666.28482.peg.1153	Contig_128	16406	17419	+	Ketol-acid reductoisomerase (EC 1.1.1.86)	FIG00000244	idu(1);Branched-Chain_Amino_Acid_Biosynthesis idu(1);Coenzyme_A_Biosynthesis
fig|6666666.28482.peg.1154	Contig_128	18076	17459	-	Trp repressor binding protein	FIG01227591	ff
fig|6666666.28482.peg.1155	Contig_128	18225	18854	+	putative integral membrane protein (DUF1211)		
fig|6666666.28482.peg.1156	Contig_128	20277	18832	-	Phytoene dehydrogenase and related proteins	FIG00139162	ff
fig|6666666.28482.peg.1157	Contig_128	20307	21893	+	D-3-phosphoglycerate dehydrogenase (EC 1.1.1.95)	FIG00031250	idu(4);Serine_Biosynthesis idu(4);Pyridoxin_(Vitamin_B6)_Biosynthesis

fig|6666666.28482.peg.1158	Contig_128	24169	22805	-	Integral membrane transport protein	FIG00820204	ff
fig|6666666.28482.peg.1159	Contig_128	24221	25021	+	2-hydroxyhepta-2,4-diene-1,7-dioate isomerase (EC 5.3.3.-) / 5-carboxymethyl-2-oxo-hex-3- ene-1,7-dioate decarboxylase (EC 4.1.1.68)	FIG00137538	isu;Aromatic_amino_acid_degradation isu;Aromatic_amino_acid_degradation isu;4-Hydroxyphenylacetic_acid_catabolic_pathway isu;4-Hydroxyphenylacetic_acid_catabolic_pathway
fig|6666666.28482.peg.1160	Contig_128	25018	26472	+	Glutamyl-tRNA synthetase (EC 6.1.1.17) @ Glutamyl-tRNA(Gln) synthetase (EC 6.1.1.24)	FIG00000686	isu;Heme_and_Siroheme_Biosynthesis isu;tRNA_aminoacylation,_Glu_and_Gln isu;tRNA_aminoacylation,_Glu_and_Gln
fig|6666666.28482.rna.15	Contig_128	26576	26647	+	tRNA-Gln-CTG		
fig|6666666.28482.rna.16	Contig_128	26720	26792	+	tRNA-Glu-CTC		
fig|6666666.28482.peg.1161	Contig_128	27312	26800	-	FIG00820161: hypothetical protein	FIG00820156	ff
fig|6666666.28482.peg.1162	Contig_129	1183	65	-	Aminopeptidase YpdF (MP-, MA-, MS-, AP-, NP-specific)	FIG00135469	idu(1);Protein_degradation
fig|6666666.28482.peg.1163	Contig_129	1229	1882	+	Possible membrane protein	FIG01121868	ff
fig|6666666.28482.peg.1164	Contig_129	2768	1893	-	hypothetical protein		
fig|6666666.28482.peg.1165	Contig_129	2904	3080	+	hypothetical protein		
fig|6666666.28482.peg.1166	Contig_129	4113	3610	-	dienelactone hydrolase(EC:3.1.1.45)		

fig|6666666.28482.peg.1167	Contig_129	4628	4188	-	3-dehydroquinate dehydratase II (EC 4.2.1.10)	FIG00000430	isu;Chorismate_Synthesis icw(2);Common_Pathway_For_Synthesis_of_Aromatic_Compounds_(DAHP_synthase_to_chorismate) isu;Quinate_degradation
fig|6666666.28482.peg.1168	Contig_129	5707	4625	-	3-dehydroquinate synthase (EC 4.2.3.4)	FIG00000224	icw(1);Chorismate_Synthesis icw(3);Common_Pathway_For_Synthesis_of_Aromatic_Compounds_(DAHP_synthase_to_chorismate)
fig|6666666.28482.peg.1169	Contig_129	6228	5704	-	Shikimate kinase I (EC 2.7.1.71)	FIG00000256	icw(3);Chorismate_Synthesis isu;Common_Pathway_For_Synthesis_of_Aromatic_Compounds_(DAHP_synthase_to_chorismate) isu;Benzoate_transport_and_degradation_cluster
fig|6666666.28482.peg.1170	Contig_129	7433	6228	-	Chorismate synthase (EC 4.2.3.5)	FIG00000212	icw(2);Chorismate_Synthesis icw(4);Common_Pathway_For_Synthesis_of_Aromatic_Compounds_(DAHP_synthase_to_chorismate)
fig|6666666.28482.peg.1171	Contig_129	7882	7454	-	hypothetical protein	FIG00638284	ff
fig|6666666.28482.peg.1172	Contig_129	8766	7993	-	Shikimate 5-dehydrogenase I alpha (EC 1.1.1.25)	FIG00000022	icw(4);Chorismate_Synthesis icw(1);Common_Pathway_For_Synthesis_of_Aromatic_Compounds_(DAHP_synthase_to_chorismate) isu;CBSS-281090.3.peg.464
fig|6666666.28482.peg.1173	Contig_129	10024	8777	-	FIG004453: protein YceG like	FIG00004453	icw(1);CBSS-281090.3.peg.464
fig|6666666.28482.peg.1174	Contig_129	10514	10017	-	Putative Holliday junction resolvase YggF		icw(2);CBSS-281090.3.peg.464 isu;CBSS-257314.1.peg.488

fig|6666666.28482.peg.1175	Contig_129	13214	10521	-	Alanyl-tRNA synthetase (EC 6.1.1.7)	FIG00000139	isu;tRNA_aminoacylation,_Ala icw(3);CBSS-281090.3.peg.464 icw(1);CBSS-257314.1.peg.488
fig|6666666.28482.peg.1176	Contig_129	13678	13277	-	FIG027887: hypothetical protein	FIG01341564	ff
fig|6666666.28482.peg.1177	Contig_129	13838	14458	+	hypothetical protein Rv2558	FIG01312331	ff
fig|6666666.28482.peg.1178	Contig_129	14571	15194	+	FIG030001: hypothetical protein	FIG01331854	ff
fig|6666666.28482.peg.1179	Contig_129	15655	15320	-	FIG00825855: hypothetical protein	FIG00825851	ff
fig|6666666.28482.peg.1180	Contig_129	16989	15652	-	ATPase, AAA family	FIG01360707	ff
fig|6666666.28482.peg.1181	Contig_129	17141	18130	+	PROBABLE PROLINE AND GLYCINE RICH TRANSMEMBRANE PROTEIN	FIG01320781	ff
fig|6666666.28482.peg.1182	Contig_129	18194	21532	+	Large protein containing transglutaminase-like domain	FIG00687328	ff
fig|6666666.28482.peg.1183	Contig_129	21533	24163	+	Protein containing domains DUF404, DUF407, DUF403	FIG00998060	ff
fig|6666666.28482.peg.1184	Contig_129	25225	24170	-	FIG00821164: hypothetical protein	FIG00821160	ff
fig|6666666.28482.peg.1185	Contig_129	26150	25218	-	Protein containing transglutaminase-like domain, putative cysteine protease	FIG00453060	ff
fig|6666666.28482.peg.1186	Contig_129	26223	26612	+	FIG00822845: hypothetical protein	FIG00822842	ff
fig|6666666.28482.peg.1187	Contig_129	27638	26607	-	hypothetical protein		
fig|6666666.28482.peg.1188	Contig_129	28080	27649	-	AclJ	FIG00862767	ff

fig|6666666.28482.peg.1189	Contig_129	29852	28077	-	Aspartyl-tRNA synthetase (EC 6.1.1.12) @ Aspartyl-tRNA(Asn) synthetase (EC 6.1.1.23)	FIG00000656	isu;tRNA_aminoacylation,_Asp_and_Asn isu;tRNA_aminoacylation,_Asp_and_Asn
fig|6666666.28482.peg.1190	Contig_129	29932	30879	+	hypothetical protein	FIG00638284	ff
fig|6666666.28482.peg.1191	Contig_129	30974	31780	+	2-dehydropantoate 2-reductase (EC 1.1.1.169)	FIG00034973	isu;Coenzyme_A_Biosynthesis
fig|6666666.28482.peg.1192	Contig_129	31790	32293	+	FIG00820672: hypothetical protein	FIG00820671	ff
fig|6666666.28482.peg.1193	Contig_129	32303	33181	+	YpfJ protein, zinc metalloprotease superfamily	FIG00003100	isu;Broadly_distributed_proteins_not_in_subsystems
fig|6666666.28482.peg.1194	Contig_129	33188	34801	+	protein of unknown function DUF894, DitE		
fig|6666666.28482.peg.1195	Contig_129	35217	34816	-	Cell division protein DivIC (FtsB), stabilizes FtsL against RasP cleavage	FIG00485194	idu(10);Possible_new_toxin-antitoxin_system_including_DivIC idu(10);Bacterial_Cell_Division idu(10);Cell_division-ribosomal_stress_proteins_cluster idu(10);Bacterial_Cytoskeleton idu(10);Stationary_phase_repair_cluster
fig|6666666.28482.peg.1196	Contig_129	35284	36849	+	Acid phosphatase (EC 3.1.3.2)	FIG01328760	ff
fig|6666666.28482.peg.1197	Contig_129	37870	36851	-	Radical SAM domain protein	FIG00741867	ff
fig|6666666.28482.peg.1198	Contig_129	37923	38801	+	Haloalkane dehalogenase (EC 3.8.1.5)	FIG01364259	ff
fig|6666666.28482.peg.1199	Contig_129	39025	38798	-	hypothetical protein		

fig|6666666.28482.peg.1200	Contig_129	39660	39118	-	EBNA-1		
fig|6666666.28482.peg.1201	Contig_129	39655	39876	+	hypothetical protein		
fig|6666666.28482.peg.1202	Contig_13	468	1340	+	FIG00825062: hypothetical protein	FIG00825059	ff
fig|6666666.28482.peg.1203	Contig_13	1410	2129	+	Putative secreted protein	FIG01353358	ff
fig|6666666.28482.peg.1204	Contig_13	2624	11851	+	[Acyl-carrier-protein] acetyl transferase of FASI (EC 2.3.1.38) / Enoyl-[acyl-carrier-protein] reductase of FASI (EC 1.3.1.9) / 3-hydroxypalmitoyl-[acyl-carrier-protein] dehydratase of FASI (EC 4.2.1.61) / [Acyl-carrier-protein] malonyl transferase of FASI (EC 2.3.1.39) / [Acyl-carrier-protein] palmitoyl transferase of FASI (EC 2.3.1.-) / Acyl carrier protein of FASI / 3-oxoacyl-[acyl-carrier-protein] reductase of FASI (EC 1.1.1.100) / 3-oxoacyl-[acyl-carrier-protein] synthase of FASI (EC 2.3.1.41)	FIG01318712	ff
fig|6666666.28482.peg.1205	Contig_13	11880	12272	+	Holo-[acyl-carrier protein] synthase (EC 2.7.8.7)	FIG00000540	idu(1);Fatty_Acid_Biosynthesis_FASII

fig|6666666.28482.peg.1206	Contig_13	12305	13609	+	Catalyzes the cleavage of p-aminobenzoyl-glutamate to p-aminobenzoate and glutamate, subunit A	FIG00001384	ff
fig|6666666.28482.peg.1207	Contig_13	13994	13614	-	POSSIBLE CONSERVED MEMBRANE PROTEIN		
fig|6666666.28482.peg.1208	Contig_13	14304	14663	+	hypothetical protein		
fig|6666666.28482.peg.1209	Contig_13	15170	14691	-	Thiol peroxidase, Bcp-type (EC 1.11.1.15)	FIG00132548	isu;CBSS-316057.3.peg.3521 isu;Thioredoxin-disulfide_reductase
fig|6666666.28482.peg.1210	Contig_13	15275	15502	+	hypothetical protein		
fig|6666666.28482.peg.1211	Contig_13	16243	16668	+	membrane protein, MmpS family	FIG01309473	ff
fig|6666666.28482.peg.1212	Contig_13	16665	19622	+	Putative membrane protein	FIG01056920	ff
fig|6666666.28482.rna.17	Contig_13	19723	19651	-	tRNA-Lys-CTT		
fig|6666666.28482.peg.1213	Contig_13	21108	19870	-	FIG00826393: hypothetical protein	FIG01312095	ff
fig|6666666.28482.peg.1214	Contig_13	21283	23643	+	Transmembrane transport protein MmpL13b	FIG01314334	ff
fig|6666666.28482.peg.1215	Contig_13	23636	24529	+	FIG00820849: hypothetical protein	FIG00820848	ff
fig|6666666.28482.peg.1216	Contig_13	24648	25871	+	ErfK/YbiS/YcfS/YnhG family protein	FIG01228918	ff
fig|6666666.28482.peg.1217	Contig_13	25959	26249	+	hypothetical protein		
fig|6666666.28482.peg.1218	Contig_13	26688	28253	+	DNA polymerase III, subunit epsilon (dnaQ)		
fig|6666666.28482.peg.1219	Contig_13	29148	28528	-	hypothetical protein	FIG00638284	ff
fig|6666666.28482.rna.18	Contig_13	30242	30170	-	tRNA-His-GTG		

fig|6666666.28482.peg.1220	Contig_13	30943	30296	-	3'-to-5' oligoribonuclease (orn)	FIG00001097	isu;RNA_processing_and_degradation,_bacterial
fig|6666666.28482.peg.1221	Contig_13	31049	32605	+	ATPase	FIG01006075	ff
fig|6666666.28482.peg.1222	Contig_13	33565	32609	-	Cyclopropane-fatty-acyl-phospholipid synthase (EC 2.1.1.79)	FIG00001555	ff
fig|6666666.28482.peg.1223	Contig_13	34612	33797	-	Short-chain dehydrogenase/reductase SDR	FIG00546160	idu(13);CBSS-164757.7.peg.5020 idu(13);Transcription_repair_cluster
fig|6666666.28482.peg.1224	Contig_13	34680	35966	+	Permease of the major facilitator transporter superfamily	FIG01351855	ff
fig|6666666.28482.peg.1225	Contig_13	36592	35969	-	POSSIBLE CONSERVED PROLINE RICH MEMBRANE PROTEIN	FIG01329840	ff
fig|6666666.28482.peg.1226	Contig_13	37400	36825	-	Transcriptional regulator, TetR family	FIG00554417	ff
fig|6666666.28482.peg.1227	Contig_13	37581	39200	+	Acetoacetyl-CoA synthetase (EC 6.2.1.16) / Long-chain-fatty-acid--CoA ligase (EC 6.2.1.3)	FIG00137255	idu(72);n-Phenylalkanoic_acid_degradation idu(1);Polyhydroxybutyrate_metabolism idu(1);HMG_CoA_Synthesis idu(72);Fatty_acid_metabolism_cluster idu(72);Biotin_synthesis_cluster idu(72);Biotin_biosynthesis icw(3);Leucine_Degradation_and_HMG-CoA_Metabolism

fig|6666666.28482.peg.1228	Contig_13	39284	40039	+	Succinyl-CoA:3-ketoacid-coenzyme A transferase subunit A (EC 2.8.3.5)	FIG00133228	isu;Protocatechuate_branch_of_beta-ketoadipate_pathway isu;Catechol_branch_of_beta-ketoadipate_pathway icw(1);Serine-glyoxylate_cycle icw(4);Leucine_Degradation_and_HMG-CoA_Metabolism
fig|6666666.28482.peg.1229	Contig_13	40036	40686	+	Succinyl-CoA:3-ketoacid-coenzyme A transferase subunit B (EC 2.8.3.5)	FIG00133276	icw(1);Protocatechuate_branch_of_beta-ketoadipate_pathway icw(1);Catechol_branch_of_beta-ketoadipate_pathway isu;Serine-glyoxylate_cycle icw(2);Leucine_Degradation_and_HMG-CoA_Metabolism
fig|6666666.28482.peg.1230	Contig_13	40683	42269	+	Methylcrotonyl-CoA carboxylase carboxyl transferase subunit (EC 6.4.1.4)	FIG00138249	icw(2);Serine-glyoxylate_cycle icw(2);HMG_CoA_Synthesis icw(1);Leucine_Degradation_and_HMG-CoA_Metabolism
fig|6666666.28482.peg.1231	Contig_13	42274	44244	+	Methylcrotonyl-CoA carboxylase biotin-containing subunit (EC 6.4.1.4)	FIG00183535	icw(3);Serine-glyoxylate_cycle icw(4);HMG_CoA_Synthesis icw(3);Leucine_Degradation_and_HMG-CoA_Metabolism
fig|6666666.28482.peg.1232	Contig_13	44241	45416	+	Isovaleryl-CoA dehydrogenase (EC 1.3.99.10)	FIG00138712	icw(2);HMG_CoA_Synthesis icw(6);Leucine_Degradation_and_HMG-CoA_Metabolism
fig|6666666.28482.peg.1233	Contig_13	45499	45993	+	Oxidase regulatory-related protein	FIG00820637	ff
fig|6666666.28482.peg.1234	Contig_13	45993	46814	+	Hydroxymethylglutaryl-CoA lyase (EC 4.1.3.4)	FIG00000963	idu(2);HMG_CoA_Synthesis idu(2);Leucine_Degradation_and_HMG-CoA_Metabolism

fig|6666666.28482.peg.1235	Contig_13	46943	48034	+	Branched-chain alpha-keto acid dehydrogenase, E1 component, alpha subunit (EC 1.2.4.4)	FIG00000964	isu;Isoleucine_degradation icw(1);Leucine_Degradation_and_HMG-CoA_Metabolism isu;Valine_degradation isu;Dehydrogenase_complexes
fig|6666666.28482.peg.1236	Contig_13	48031	49065	+	Branched-chain alpha-keto acid dehydrogenase, E1 component, beta subunit (EC 1.2.4.4)	FIG00055130	icw(3);Isoleucine_degradation icw(4);Leucine_Degradation_and_HMG-CoA_Metabolism icw(3);Valine_degradation icw(2);Dehydrogenase_complexes
fig|6666666.28482.peg.1237	Contig_13	49062	50219	+	Dihydrolipoamide acyltransferase component of branched-chain alpha-keto acid dehydrogenase complex (EC 2.3.1.168)	FIG01304883	icw(2);Isoleucine_degradation icw(2);Leucine_Degradation_and_HMG-CoA_Metabolism icw(2);Valine_degradation icw(1);Dehydrogenase_complexes
fig|6666666.28482.peg.1238	Contig_13	50624	50223	-	hypothetical protein		
fig|6666666.28482.peg.1239	Contig_13	51128	50949	-	hypothetical protein		
fig|6666666.28482.peg.1240	Contig_13	51127	51264	+	hypothetical protein		

fig|6666666.28482.peg.1241	Contig_13	52133	51336	-	Enoyl-CoA hydratase (EC 4.2.1.17)	FIG00033206	idu(51);n-Phenylalkanoic_acid_degradation idu(51);Polyhydroxybutyrate_metabolism icw(1);Isoleucine_degradation idu(51);Fatty_acid_metabolism_cluster idu(51);Lysine_fermentation idu(51);Butanol_Biosynthesis idu(51);Acetyl-CoA_fermentation_to_Butyrate icw(1);Valine_degradation idu(51);Butyrate_metabolism_cluster
fig|6666666.28482.peg.1242	Contig_13	52239	52487	+	hypothetical protein		
fig|6666666.28482.peg.1243	Contig_13	52916	52491	-	FIG00830246: hypothetical protein	FIG00830243	ff
fig|6666666.28482.peg.1244	Contig_13	52982	53545	+	Transcriptional regulator, TetR family	FIG00554417	ff
fig|6666666.28482.peg.1245	Contig_13	53653	63327	+	Long-chain-fatty-acid--CoA ligase (EC 6.2.1.3)	FIG00018699	idu(72);n-Phenylalkanoic_acid_degradation idu(72);Fatty_acid_metabolism_cluster idu(72);Biotin_synthesis_cluster idu(72);Biotin_biosynthesis
fig|6666666.28482.peg.1246	Contig_13	63636	64442	+	Hydroxyproline-rich glycoprotein DZ-HRGP precursor		
fig|6666666.28482.peg.1247	Contig_13	65045	64479	-	serine esterase, cutinase family	FIG01349249	ff

fig|6666666.28482.peg.1248	Contig_13	65691	65260	-	14 kDa antigen (16 kDa antigen) (HSP 16.3)	FIG01320402	ff
fig|6666666.28482.peg.1249	Contig_13	66170	65868	-	hypothetical protein		
fig|6666666.28482.peg.1250	Contig_13	66546	66373	-	hypothetical protein		
fig|6666666.28482.peg.1251	Contig_13	66635	66751	+	hypothetical protein		
fig|6666666.28482.peg.1252	Contig_13	66847	67155	+	hypothetical protein		
fig|6666666.28482.rna.19	Contig_13	67252	67179	-	tRNA-Arg-TCT		
fig|6666666.28482.peg.1253	Contig_13	67438	68685	+	Carboxylesterase LipQ	FIG00833775	ff
fig|6666666.28482.peg.1254	Contig_13	68927	70294	+	Acyltransferase, ws/dgat/mgat subfamily protein	FIG01331760	ff
fig|6666666.28482.peg.1255	Contig_13	70291	72066	+	Phosphoserine phosphatase (EC 3.1.3.3) / 1-acyl-sn-glycerol-3-phosphate acyltransferase (EC 2.3.1.51)	FIG00135282	idu(6);Glycerolipid_and_Glycerophospholipid_Metabolism_in_Bacteria idu(3);CBSS-336982.3.peg.3874 idu(6);Ribosome_post-transcriptional_modification_and_chromosomal_segregation_cluster idu(3);Serine_Biosynthesis
fig|6666666.28482.peg.1256	Contig_13	72063	74432	+	Glycerol-3-phosphate acyltransferase (EC 2.3.1.15)	FIG00001327	icw(1);Glycerolipid_and_Glycerophospholipid_Metabolism_in_Bacteria
fig|6666666.28482.peg.1257	Contig_13	74459	76492	+	Copper resistance protein D	FIG00001556	isu;Copper_homeostasis
fig|6666666.28482.peg.1258	Contig_13	76690	76574	-	YoeB toxin protein	FIG00006169	ff
fig|6666666.28482.peg.1259	Contig_13	78777	76891	-	hypothetical protein	FIG00638284	ff
fig|6666666.28482.peg.1260	Contig_13	78784	78993	+	hypothetical protein		
fig|6666666.28482.peg.1261	Contig_13	79513	79995	+	Single-strand DNA binding protein	FIG00820266	ff
fig|6666666.28482.peg.1262	Contig_13	80076	81752	+	ABC transporter ATP-binding protein	FIG01956052	ff

fig|6666666.28482.peg.1263	Contig_13	81828	86699	+	NAD-specific glutamate dehydrogenase (EC 1.4.1.2), large form	FIG00003209	isu;Glutamate_dehydrogenases isu;Glutamine,_Glutamate,_Aspartate_and_Asparagine_Biosynthesis
fig|6666666.28482.peg.1264	Contig_13	86747	87112	+	FIG00995656: hypothetical protein	FIG00823158	ff
fig|6666666.28482.peg.1265	Contig_13	87133	87771	+	FIG01001213: hypothetical protein	FIG00820498	ff
fig|6666666.28482.peg.1266	Contig_13	89354	87786	-	Alpha-glucosidase (EC 3.2.1.20)	FIG00745599	isu;Maltose_and_Maltodextrin_Utilization isu;D-Galacturonate_and_D-Glucuronate_Utilization
fig|6666666.28482.peg.1267	Contig_13	89776	89378	-	Hemoglobin-like protein HbO	FIG00001245	isu;Bacterial_hemoglobins
fig|6666666.28482.peg.1268	Contig_13	90057	90557	+	HNH endonuclease family protein	FIG00017582	ff
fig|6666666.28482.peg.1269	Contig_13	90613	90849	+	FIG00821364: hypothetical protein	FIG00821363	ff
fig|6666666.28482.peg.1270	Contig_13	90836	91303	+	FIG00997368: hypothetical protein	FIG00820011	ff
fig|6666666.28482.peg.1271	Contig_13	93973	91391	-	Membrane alanine aminopeptidase N (EC 3.4.11.2)	FIG00000714	isu;Aminopeptidases_(EC_3.4.11.-)
fig|6666666.28482.peg.1272	Contig_13	94102	94725	+	FIG00820885: hypothetical protein	FIG00820884	ff
fig|6666666.28482.peg.1273	Contig_13	94763	95251	+	Ribose 5-phosphate isomerase B (EC 5.3.1.6) / Galactose 6-phosphate isomerase	FIG00135914	isu;D-ribose_utilization isu;Pentose_phosphate_pathway
fig|6666666.28482.peg.1274	Contig_13	95255	96061	+	Formamidopyrimidine-DNA glycosylase (EC 3.2.2.23)	FIG00035634	idu(3);DNA_Repair_Base_Excision

fig|6666666.28482.peg.1275	Contig_13	96150	96425	+	conserved hypothetical secreted protein		
fig|6666666.28482.peg.1276	Contig_13	97672	96479	-	Esterase A	FIG01314929	ff
fig|6666666.28482.rna.20	Contig_13	97774	97704	-	tRNA-Gly-TCC		
fig|6666666.28482.rna.21	Contig_13	97931	98004	+	tRNA-Pro-TGG		
fig|6666666.28482.peg.1277	Contig_13	98046	99488	+	Cell division trigger factor (EC 5.2.1.8)	FIG00000286	isu;Bacterial_Cell_Division
fig|6666666.28482.peg.1278	Contig_13	99607	100206	+	ATP-dependent Clp protease proteolytic subunit (EC 3.4.21.92)	FIG00000028	icw(2);Proteolysis_in_bacteria,_ATP-dependent icw(1);cAMP_signaling_in_bacteria
fig|6666666.28482.peg.1279	Contig_13	100203	100838	+	ATP-dependent Clp protease proteolytic subunit (EC 3.4.21.92)	FIG00000028	icw(2);Proteolysis_in_bacteria,_ATP-dependent icw(1);cAMP_signaling_in_bacteria
fig|6666666.28482.peg.1280	Contig_13	101753	100893	-	Homocysteine S-methyltransferase (EC 2.1.1.10)	FIG00002804	isu;S-methylmethionine
fig|6666666.28482.peg.1281	Contig_13	101926	101747	-	hypothetical protein		
fig|6666666.28482.peg.1282	Contig_13	102047	103327	+	ATP-dependent Clp protease ATP-binding subunit ClpX	FIG00000241	isu;Proteolysis_in_bacteria,_ATP-dependent
fig|6666666.28482.peg.1283	Contig_13	103358	104596	+	Integral membrane transport protein	FIG00820204	ff
fig|6666666.28482.peg.1284	Contig_13	104950	106791	+	2-oxoglutarate oxidoreductase, alpha subunit (EC 1.2.7.3)	FIG00937303	ff
fig|6666666.28482.peg.1285	Contig_13	106788	107888	+	2-oxoglutarate oxidoreductase, beta subunit (EC 1.2.7.3)	FIG00049580	ff

fig|6666666.28482.peg.1286	Contig_13	107959	108495	+	Molybdopterin-guanine dinucleotide biosynthesis protein MobA	FIG00000902	isu;ar-431-EC_Molybdopterin-guanine_dinucleotide_biosynthesis isu;Molybdenum_cofactor_biosynthesis
fig|6666666.28482.peg.1287	Contig_130	1555	2427	+	Non functional Dihydropteroate synthase 2	FIG00018087	ff
fig|6666666.28482.peg.1288	Contig_130	2424	3380	+	Glycosyltransferases involved in cell wall biogenesis	FIG01313034	ff
fig|6666666.28482.peg.1289	Contig_130	3419	3760	+	FIG01000442: hypothetical protein	FIG00820927	ff
fig|6666666.28482.peg.1290	Contig_130	3757	4323	+	DNA-3-methyladenine glycosylase (EC 3.2.2.20)	FIG00000673	isu;DNA_Repair_Base_Excision isu;CBSS-326442.4.peg.1852
fig|6666666.28482.peg.1291	Contig_130	4510	4677	+	hypothetical protein	FIG00638284	ff
fig|6666666.28482.peg.1292	Contig_130	5848	4685	-	Predicted glycogen synthase, ADP-glucose transglucosylase (EC 2.4.1.21), Actinobacterial type	FIG00139522	isu;Glycogen_metabolism
fig|6666666.28482.peg.1293	Contig_130	6022	7218	+	Glucose-1-phosphate adenylyltransferase (EC 2.7.7.27)	FIG00000717	icw(1);Glycogen_metabolism
fig|6666666.28482.peg.1294	Contig_130	8981	7299	-	Cocaine esterase (EC 3.1.1.-)	FIG01350540	ff
fig|6666666.28482.peg.1295	Contig_130	9674	9000	-	isoprenylcysteine carboxyl methyltransferase (icmt) family protein		

fig|6666666.28482.peg.1296	Contig_130	11331	9688	-	PROBABLE TETRONASIN-TRANSPORT INTEGRAL MEMBRANE PROTEIN ABC TRANSPORTER	FIG00996565	ff
fig|6666666.28482.peg.1297	Contig_130	12248	11328	-	ABC-type multidrug transport system, ATPase component	FIG00001005	idu(1);CBSS-196164.1.peg.1690
fig|6666666.28482.peg.1298	Contig_130	12876	12238	-	Transcriptional regulator, TetR family	FIG00554417	ff
fig|6666666.28482.peg.1299	Contig_130	13625	12957	-	O-methyltransferase	FIG00821228	ff
fig|6666666.28482.peg.1300	Contig_130	13828	14610	+	RNA polymerase sigma-70 factor, ECF subfamily	FIG00952839	ff
fig|6666666.28482.peg.1301	Contig_130	15458	14676	-	FIG025093: Probable membrane protein	FIG01306444	idu(2);Mycobacterium_virulence_operon_involved_in_protein_synthesis_(SSU_ribosomal_proteins)
fig|6666666.28482.peg.1302	Contig_130	18373	15731	-	hypothetical protein	FIG00638284	ff
fig|6666666.28482.peg.1303	Contig_130	18945	18775	-	hypothetical protein		
fig|6666666.28482.peg.1304	Contig_131	170	1018	+	Signal recognition particle receptor protein FtsY (=alpha subunit) (TC 3.A.5.1.1)	FIG00000095	isu;Bacterial_signal_recognition_particle_(SRP) isu;Bacterial_Cell_Division
fig|6666666.28482.peg.1305	Contig_131	1158	2588	+	Ammonium transporter	FIG00004764	icw(1);Ammonia_assimilation
fig|6666666.28482.peg.1306	Contig_131	2585	2923	+	Nitrogen regulatory protein P-II	FIG00005111	idu(1);Ammonia_assimilation
fig|6666666.28482.peg.1307	Contig_131	3083	5413	+	[Protein-PII] uridylyltransferase (EC 2.7.7.59)	FIG00002864	icw(2);Ammonia_assimilation isu;CBSS-312309.3.peg.1965
fig|6666666.28482.peg.1308	Contig_131	5838	5644	-	hypothetical protein		

fig|6666666.28482.peg.1309	Contig_132	36	602	+	Protoporphyrinogen IX oxidase, aerobic, HemY (EC 1.3.3.4)	FIG00068302	icw(1);Heme_and_Siroheme_Biosynthesis
fig|6666666.28482.peg.1310	Contig_132	608	1303	+	Hemoprotein HemQ, essential component of heme biosynthetic pathway in Gram-positive bacteria	FIG00003629	isu;Heme_and_Siroheme_Biosynthesis
fig|6666666.28482.peg.1311	Contig_132	1325	2074	+	Methyltransferase (EC 2.1.1.-) colocalized with Q	FIG00026000	ff
fig|6666666.28482.peg.1312	Contig_132	3420	2071	-	FIG00821608: hypothetical protein	FIG00821607	ff
fig|6666666.28482.peg.1313	Contig_133	134	15	-	hypothetical protein		
fig|6666666.28482.peg.1314	Contig_133	342	1550	+	PPE family protein	FIG00013075	idu(57);CBSS-164757.7.peg.5020
fig|6666666.28482.peg.1315	Contig_133	2082	2342	+	hypothetical protein		
fig|6666666.28482.peg.1316	Contig_133	2450	2746	+	FIG00824639: hypothetical protein	FIG00823510	ff
fig|6666666.28482.peg.1317	Contig_134	4	1023	+	PPE family protein	FIG00013075	idu(57);CBSS-164757.7.peg.5020
fig|6666666.28482.peg.1318	Contig_134	1123	1404	+	FIG00824639: hypothetical protein	FIG00823510	ff
fig|6666666.28482.peg.1319	Contig_135	3187	875	-	ATP-dependent helicase	FIG00995938	ff
fig|6666666.28482.peg.1320	Contig_135	3460	3663	+	Cold shock protein CspA	FIG00004100	idu(1);Cold_shock,_CspA_family_of_proteins
fig|6666666.28482.peg.1321	Contig_135	3789	4388	+	FIG00995018: hypothetical protein	FIG00820764	ff
fig|6666666.28482.peg.1322	Contig_135	4584	7373	+	DNA topoisomerase I (EC 5.99.1.2)	FIG00000204	isu;CBSS-272943.3.peg.1367 isu;DNA_topoisomerases,_Type_I,_ATP-independent
fig|6666666.28482.peg.1323	Contig_135	8974	7370	-	Adenylate cyclase (EC 4.6.1.1)	FIG00001294	idu(25);cAMP_signaling_in_bacteria
fig|6666666.28482.peg.1324	Contig_135	9059	9211	+	hypothetical protein		

fig|6666666.28482.peg.1325	Contig_135	9171	10346	+	DNA polymerase III delta prime subunit (EC 2.7.7.7)	FIG00057709	isu;DNA-replication
fig|6666666.28482.rna.22	Contig_135	10407	10479	+	tRNA-Thr-CGT		
fig|6666666.28482.peg.1326	Contig_135	10891	11133	+	hypothetical protein		
fig|6666666.28482.peg.1327	Contig_135	13015	11246	-	PROBABLE CONSERVED TRANSMEMBRANE PROTEIN	FIG00662132	ff
fig|6666666.28482.peg.1328	Contig_135	13053	13997	+	UDP-glucose 4-epimerase (EC 5.1.3.2)	FIG00022300	idu(4);Rhamnose_containing_glycans idu(4);Lactose_and_Galactose_Uptake_and_Utilization idu(4);N-linked_Glycosylation_in_Bacteria idu(4);Lacto-N-Biose_I_and_Galacto-N-Biose_Metabolic_Pathway
fig|6666666.28482.peg.1329	Contig_135	14563	14213	-	Cell division protein DivIC (FtsB), stabilizes FtsL against RasP cleavage	FIG00485194	idu(10);Possible_new_toxin-antitoxin_system_including_DivIC idu(10);Bacterial_Cell_Division idu(10);Cell_division-ribosomal_stress_proteins_cluster idu(10);Bacterial_Cytoskeleton idu(10);Stationary_phase_repair_cluster
fig|6666666.28482.peg.1330	Contig_135	15255	14560	-	Dolichol-phosphate mannosyltransferase (EC 2.4.1.83) in lipid-linked oligosaccharide synthesis cluster	FIG00543846	idu(1);Lipid-linked_oligosaccharide_synthesis_related_cluster
fig|6666666.28482.peg.1331	Contig_135	16560	15280	-	hypothetical protein	FIG00638284	ff

fig|6666666.28482.peg.1332	Contig_135	16645	17733	+	Integral membrane protein	FIG01956050	ff
fig|6666666.28482.peg.1333	Contig_135	18431	17730	-	Putative uncharacterized protein BCG_1787	FIG00831112	ff
fig|6666666.28482.peg.1334	Contig_135	18976	18479	-	Inorganic pyrophosphatase (EC 3.6.1.1)	FIG00000429	isu;Phosphate_metabolism
fig|6666666.28482.peg.1335	Contig_135	19125	20477	+	D-alanyl-D-alanine carboxypeptidase (EC 3.4.16.4)	FIG00561498	idu(3);Murein_Hydrolases idu(3);CBSS-84588.1.peg.1247 idu(3);Metallocarboxypeptidases_(EC _3.4.17.-) idu(3);Peptidoglycan_Biosynthesis
fig|6666666.28482.peg.1336	Contig_135	20468	21535	+	hypothetical protein	FIG00821505	ff
fig|6666666.28482.peg.1337	Contig_135	21514	22473	+	tRNA(Ile)-lysidine synthetase	FIG00000257	isu;tRNA_processing isu;Cell_division-ribosomal_stress_proteins_cluster
fig|6666666.28482.peg.1338	Contig_135	22517	23128	+	Hypoxanthine-guanine phosphoribosyltransferase (EC 2.4.2.8)	FIG00000355	isu;Purine_conversions icw(1);Cell_division-ribosomal_stress_proteins_cluster
fig|6666666.28482.peg.1339	Contig_135	23927	23217	-	Lipoprotein LpqG	FIG01334576	ff
fig|6666666.28482.peg.1340	Contig_135	24223	24522	+	PE family protein	FIG01311212	ff

fig|6666666.28482.peg.1341	Contig_136	3504	160	-	Dihydrolipoamide succinyltransferase component (E2) of 2-oxoglutarate dehydrogenase complex (EC 2.3.1.61) / 2-oxoglutarate dehydrogenase E1 component (EC 1.2.4.2)	FIG00004392	isu;TCA_Cycle isu;TCA_Cycle isu;Dehydrogenase_complexes isu;Dehydrogenase_complexes
fig|6666666.28482.peg.1342	Contig_137	1149	436	-	hypothetical protein		
fig|6666666.28482.peg.1343	Contig_137	2598	1165	-	Long-chain-fatty-acid--CoA ligase (EC 6.2.1.3)	FIG00018699	idu(72);n-Phenylalkanoic_acid_degradation idu(72);Fatty_acid_metabolism_cluster idu(72);Biotin_synthesis_cluster idu(72);Biotin_biosynthesis
fig|6666666.28482.peg.1344	Contig_137	3306	2608	-	hypothetical protein	FIG00638284	ff
fig|6666666.28482.peg.1345	Contig_137	3497	3979	+	hypothetical protein		
fig|6666666.28482.peg.1346	Contig_137	3999	4628	+	nitroreductase	FIG01123116	ff
fig|6666666.28482.peg.1347	Contig_137	4628	5122	+	FIG00822669: hypothetical protein	FIG00822668	ff
fig|6666666.28482.peg.1348	Contig_137	5812	6495	+	Transcriptional regulator, FUR family	FIG00002441	idu(2);Oxidative_stress
fig|6666666.28482.peg.1349	Contig_137	6548	7042	+	FIG00823749: hypothetical protein	FIG00823745	ff
fig|6666666.28482.peg.1350	Contig_137	7059	8039	+	D-amino-acid oxidase (EC 1.4.3.3)	FIG00002178	isu;Proline,_4-hydroxyproline_uptake_and_utilization

fig|6666666.28482.peg.1351	Contig_137	8036	8818	+	Enoyl-CoA hydratase (EC 4.2.1.17)	FIG00033206	idu(51);n-Phenylalkanoic_acid_degradation idu(51);Polyhydroxybutyrate_metabolism idu(51);Isoleucine_degradation idu(51);Fatty_acid_metabolism_cluster idu(51);Lysine_fermentation idu(51);Butanol_Biosynthesis idu(51);Acetyl-CoA_fermentation_to_Butyrate idu(51);Valine_degradation idu(51);Butyrate_metabolism_cluster
fig|6666666.28482.peg.1352	Contig_137	9225	8833	-	Possible membrane protein	FIG01121868	ff
fig|6666666.28482.peg.1353	Contig_137	10322	9255	-	hypothetical protein	FIG00638284	ff
fig|6666666.28482.peg.1354	Contig_137	10376	11650	+	Sialic acid-transport integral membrane protein NanT	FIG01308258	ff
fig|6666666.28482.peg.1355	Contig_137	12942	11653	-	Molybdopterin binding motif, CinA N-terminal domain / C-terminal domain of CinA type S	FIG01304044	isu;NAD_and_NADP_cofactor_biosynthesis_global isu;Riboflavin_synthesis_cluster
fig|6666666.28482.peg.1356	Contig_137	12997	14373	+	lignin peroxidase LipJ		
fig|6666666.28482.peg.1357	Contig_137	14391	14957	+	Lipoprotein LppD	FIG01314811	ff
fig|6666666.28482.peg.1358	Contig_137	15259	14954	-	ExtraCellular Mutant; Ecm15p	FIG01312625	ff
fig|6666666.28482.peg.1359	Contig_137	15311	15742	+	D-tyrosyl-tRNA(Tyr) deacylase	FIG00000473	isu;D-tyrosyl-tRNA(Tyr)_deacylase isu;CBSS-342610.3.peg.283

fig|6666666.28482.peg.1360	Contig_137	15790	16707	+	Putative S-adenosyl-L-methionine-dependent methyltransferase	FIG01342021	ff
fig|6666666.28482.peg.1361	Contig_137	17740	16715	-	Threonine dehydrogenase and related Zn-dependent dehydrogenases	FIG01292486	idu(2);Threonine_degradation
fig|6666666.28482.peg.1362	Contig_137	19709	17769	-	Probable monooxygenase Y4ID (EC 1.14.13.-)	FIG01394124	ff
fig|6666666.28482.peg.1363	Contig_138	2559	34	-	putative transporter permease	FIG01600014	ff
fig|6666666.28482.peg.1364	Contig_138	3269	2556	-	putative ABC transporter ATP-binding protein	FIG00995347	ff
fig|6666666.28482.peg.1365	Contig_138	3439	4224	+	FIG00825647: hypothetical protein	FIG00825646	ff
fig|6666666.28482.peg.1366	Contig_138	4224	4595	+	FIG00820067: hypothetical protein	FIG00820065	ff
fig|6666666.28482.peg.1367	Contig_138	4666	5313	+	FIG00815605: hypothetical protein	FIG00815604	ff
fig|6666666.28482.peg.1368	Contig_138	5776	5321	-	hypothetical protein		
fig|6666666.28482.peg.1369	Contig_138	5909	7657	+	Amidohydrolase 3		
fig|6666666.28482.peg.1370	Contig_138	8385	7654	-	Cutinase Cut1	FIG00826187	ff
fig|6666666.28482.peg.1371	Contig_138	8981	8505	-	FIG00832781: hypothetical protein	FIG00821740	ff
fig|6666666.28482.peg.1372	Contig_138	9728	9063	-	Transcriptional regulator, TetR family	FIG00554417	ff
fig|6666666.28482.peg.1373	Contig_138	10369	9782	-	FIG00824313: hypothetical protein	FIG00824312	ff
fig|6666666.28482.peg.1374	Contig_138	10412	10957	+	FIG00821471: hypothetical protein	FIG00821470	ff

fig|6666666.28482.peg.1375	Contig_138	11066	11917	+	Enoyl-CoA hydratase (EC 4.2.1.17)	FIG00033206	icw(1);n-Phenylalkanoic_acid_degradation idu(51);Polyhydroxybutyrate_metabolism idu(51);Isoleucine_degradation icw(1);Fatty_acid_metabolism_cluster idu(51);Lysine_fermentation idu(51);Butanol_Biosynthesis idu(51);Acetyl-CoA_fermentation_to_Butyrate idu(51);Valine_degradation idu(51);Butyrate_metabolism_cluster
fig|6666666.28482.peg.1376	Contig_138	13669	11969	-	FIG00824882: hypothetical protein	FIG00824878	ff
fig|6666666.28482.peg.1377	Contig_138	15039	13666	-	FIG00822601: hypothetical protein	FIG00822600	ff
fig|6666666.28482.peg.1378	Contig_138	16589	15054	-	Long-chain-fatty-acid--CoA ligase (EC 6.2.1.3)	FIG00018699	idu(72);n-Phenylalkanoic_acid_degradation idu(72);Fatty_acid_metabolism_cluster idu(72);Biotin_synthesis_cluster idu(72);Biotin_biosynthesis

fig|6666666.28482.peg.1379	Contig_138	17469	16597	-	Enoyl-CoA hydratase (EC 4.2.1.17)	FIG00033206	icw(1);n-Phenylalkanoic_acid_degradation idu(51);Polyhydroxybutyrate_metabolism idu(51);Isoleucine_degradation icw(1);Fatty_acid_metabolism_cluster idu(51);Lysine_fermentation idu(51);Butanol_Biosynthesis idu(51);Acetyl-CoA_fermentation_to_Butyrate idu(51);Valine_degradation idu(51);Butyrate_metabolism_cluster
fig|6666666.28482.peg.1380	Contig_138	17720	17484	-	hypothetical	FIG01337044	ff
fig|6666666.28482.peg.1381	Contig_138	18711	17713	-	Acetoin dehydrogenase E1 component beta-subunit (EC 1.2.4.-)	FIG01270039	idu(1);Acetoin,_butanediol_metabolism idu(1);Dehydrogenase_complexes
fig|6666666.28482.peg.1382	Contig_138	19705	18716	-	Acetoin dehydrogenase E1 component alpha-subunit (EC 1.2.4.-)	FIG00138523	icw(1);Acetoin,_butanediol_metabolism icw(1);Dehydrogenase_complexes
fig|6666666.28482.peg.1383	Contig_138	20606	19740	-	FIG00820090: hypothetical protein	FIG00820089	ff
fig|6666666.28482.peg.1384	Contig_138	20854	22047	+	FIG00824352: hypothetical protein	FIG00824349	ff
fig|6666666.28482.peg.1385	Contig_138	22209	22096	-	hypothetical protein		
fig|6666666.28482.peg.1386	Contig_138	22307	22576	+	hypothetical protein		
fig|6666666.28482.peg.1387	Contig_138	22608	23027	+	protein of unknown function DUF302		

fig|6666666.28482.peg.1388	Contig_138	23034	23393	+	Possible carboxymuconolactone decarboxylase family protein (EC 4.1.1.44)	FIG01384312	ff
fig|6666666.28482.peg.1389	Contig_138	23512	24876	+	Diacyglycerol O-acyltransferase (EC 2.3.1.20)	FIG01394190	ff
fig|6666666.28482.peg.1390	Contig_138	25071	25337	+	FIG00828553: hypothetical protein	FIG00828551	ff
fig|6666666.28482.peg.1391	Contig_138	26134	25409	-	Transcriptional regulator, IclR family	FIG00022569	idu(12);Homogentisate_pathway_of_aromatic_compound_degradation
fig|6666666.28482.peg.1392	Contig_138	26232	27572	+	oxidoreductase, FAD-binding	FIG01339311	ff
fig|6666666.28482.peg.1393	Contig_138	27572	28366	+	FIG00820194: hypothetical protein	FIG00820191	ff
fig|6666666.28482.peg.1394	Contig_138	28598	28446	-	FIG00821218: hypothetical protein	FIG00821215	ff
fig|6666666.28482.peg.1395	Contig_139	1613	528	-	Uroporphyrinogen III decarboxylase (EC 4.1.1.37)	FIG00000428	isu;Heme_and_Siroheme_Biosynthesis

fig|6666666.28482.peg.1396	Contig_139	1671	2474	+	Enoyl-CoA hydratase (EC 4.2.1.17)	FIG00033206	idu(51);n-Phenylalkanoic_acid_degradation idu(51);Polyhydroxybutyrate_metabolism idu(51);Isoleucine_degradation idu(51);Fatty_acid_metabolism_cluster idu(51);Lysine_fermentation idu(51);Butanol_Biosynthesis idu(51);Acetyl-CoA_fermentation_to_Butyrate idu(51);Valine_degradation idu(51);Butyrate_metabolism_cluster
fig|6666666.28482.peg.1397	Contig_139	2648	3229	+	Uncharacterized protein Q1 colocalized with Q	FIG00004682	ff
fig|6666666.28482.peg.1398	Contig_139	3333	4514	+	Ribonuclease D (EC 3.1.26.3)	FIG00000804	isu;tRNA_processing
fig|6666666.28482.peg.1399	Contig_14	93	1049	+	DNA polymerase I (EC 2.7.7.7)	FIG00000404	idu(1);DNA_Repair_Base_Excision idu(1);DNA-replication
fig|6666666.28482.peg.1400	Contig_14	1751	1056	-	Possible membrane protein	FIG01121868	ff
fig|6666666.28482.peg.1401	Contig_14	4690	1901	-	FIG005666: putative helicase	FIG00005666	icw(4);Cluster-based_Subsystem_Grouping_Hypotheticals_-_perhaps_Proteosome_Related

fig|6666666.28482.peg.1402	Contig_14	5659	4691	-	Twin-arginine translocation protein TatC	FIG00000335	icw(1);Cluster-based_Subsystem_Grouping_Hypotheticals_-_perhaps_Proteosome_Related isu;Twin-arginine_translocation_system
fig|6666666.28482.peg.1403	Contig_14	5930	5709	-	Twin-arginine translocation protein TatA	FIG00138313	icw(4);Cluster-based_Subsystem_Grouping_Hypotheticals_-_perhaps_Proteosome_Related icw(1);Twin-arginine_translocation_system
fig|6666666.28482.peg.1404	Contig_14	7003	6026	-	FIG019733: possible DNA-binding protein	FIG00019733	icw(2);Cluster-based_Subsystem_Grouping_Hypotheticals_-_perhaps_Proteosome_Related
fig|6666666.28482.peg.1405	Contig_14	7956	7000	-	FIG005453: Putative DeoR-family transcriptional regulator	FIG00005453	isu;Cluster-based_Subsystem_Grouping_Hypotheticals_-_perhaps_Proteosome_Related
fig|6666666.28482.peg.1406	Contig_14	9585	8203	-	Pup ligase PafA, possible component of postulated heterodimer PafA-PafA'	FIG00137619	icw(8);Cluster-based_Subsystem_Grouping_Hypotheticals_-_perhaps_Proteosome_Related icw(3);Proteasome_archaeal
fig|6666666.28482.peg.1407	Contig_14	10414	9632	-	Proteasome subunit alpha (EC 3.4.25.1), bacterial	FIG00145504	icw(6);Cluster-based_Subsystem_Grouping_Hypotheticals_-_perhaps_Proteosome_Related icw(1);Proteasome_archaeal

fig|6666666.28482.peg.1408	Contig_14	11190	10411	-	Proteasome subunit beta (EC 3.4.25.1), bacterial	FIG00145554	icw(2);Cluster-based_Subsystem_Grouping_Hypotheticals_-_perhaps_Proteosome_Related icw(4);Proteasome_archaeal
fig|6666666.28482.peg.1409	Contig_14	11492	11298	-	Prokaryotic ubiquitin-like protein Pup	FIG00016412	icw(5);Cluster-based_Subsystem_Grouping_Hypotheticals_-_perhaps_Proteosome_Related icw(2);Proteasome_archaeal
fig|6666666.28482.peg.1410	Contig_14	13104	11596	-	Pup ligase PafA' paralog, possible component of postulated heterodimer PafA-PafA'	FIG00143645	icw(2);Cluster-based_Subsystem_Grouping_Hypotheticals_-_perhaps_Proteosome_Related isu;Proteasome_archaeal
fig|6666666.28482.peg.1411	Contig_14	13251	14432	+	Possible membrane protein	FIG01121868	ff
fig|6666666.28482.peg.1412	Contig_14	14442	15083	+	FIG00821521: hypothetical protein	FIG00821520	ff
fig|6666666.28482.peg.1413	Contig_14	16955	15111	-	hypothetical protein	FIG00638284	ff
fig|6666666.28482.peg.1414	Contig_14	18819	16990	-	Bacterial proteasome-activating AAA-ATPase (PAN)	FIG00138585	isu;Cluster-based_Subsystem_Grouping_Hypotheticals_-_perhaps_Proteosome_Related isu;Proteasome_archaeal
fig|6666666.28482.peg.1415	Contig_14	19144	19692	+	Probable conserved lipoprotein lppK	FIG01324282	ff
fig|6666666.28482.peg.1416	Contig_14	19698	20000	+	YlxP-like protein	FIG00001863	isu;NusA-TFII_Cluster isu;CBSS-350688.3.peg.1509
fig|6666666.28482.peg.1417	Contig_14	20845	20003	-	RNA methyltransferase	FIG01124700	ff

fig|6666666.28482.peg.1418	Contig_14	20995	21786	+	RecB family exonuclease	FIG00815647	ff
fig|6666666.28482.peg.1419	Contig_14	22034	23293	+	ATP-dependent RNA helicase	FIG01231651	ff
fig|6666666.28482.peg.1420	Contig_14	23301	23516	+	hypothetical protein		
fig|6666666.28482.peg.1421	Contig_14	24970	23603	-	PF00070 family, FAD-dependent NAD(P)-disulphide oxidoreductase	FIG00001597	idu(1);Mercuric_reductase
fig|6666666.28482.peg.1422	Contig_14	25476	24967	-	Hypothetical membrane protein Rv2120c	FIG01326130	ff
fig|6666666.28482.peg.1423	Contig_14	26459	25602	-	ATP phosphoribosyltransferase (EC 2.4.2.17)	FIG00000811	icw(1);Riboflavin_synthesis_cluster icw(1);Histidine_Biosynthesis
fig|6666666.28482.peg.1424	Contig_14	26725	26462	-	Phosphoribosyl-ATP pyrophosphatase (EC 3.6.1.31)	FIG00000871	isu;Riboflavin_synthesis_cluster isu;Histidine_Biosynthesis
fig|6666666.28482.peg.1425	Contig_14	26848	27273	+	JadX-like protein		
fig|6666666.28482.peg.1426	Contig_14	31033	27284	-	5-methyltetrahydrofolate--homocysteine methyltransferase (EC 2.1.1.13)	FIG00000672	ff
fig|6666666.28482.peg.1427	Contig_14	31388	32263	+	FIG00823557: hypothetical protein	FIG00823551	ff
fig|6666666.28482.peg.1428	Contig_14	33143	32268	-	Oxidoreductase, short-chain dehydrogenase/reductase family	FIG00993242	ff

fig|6666666.28482.peg.1429	Contig_14	34787	33324	-	Long-chain-fatty-acid--CoA ligase (EC 6.2.1.3)	FIG00018699	idu(72);n-Phenylalkanoic_acid_degradation idu(72);Fatty_acid_metabolism_cluster idu(72);Biotin_synthesis_cluster idu(72);Biotin_biosynthesis
fig|6666666.28482.peg.1430	Contig_14	35837	34899	-	hypothetical protein	FIG00638284	ff
fig|6666666.28482.peg.1431	Contig_14	36493	35834	-	Nitroreductase		
fig|6666666.28482.peg.1432	Contig_14	37602	36490	-	hypothetical protein		
fig|6666666.28482.peg.1433	Contig_14	37704	38612	+	Enoyl-CoA hydratase (EC 4.2.1.17)	FIG00033206	icw(1);n-Phenylalkanoic_acid_degradation idu(51);Polyhydroxybutyrate_metabolism icw(1);Isoleucine_degradation icw(1);Fatty_acid_metabolism_cluster icw(1);Lysine_fermentation icw(1);Butanol_Biosynthesis icw(1);Acetyl-CoA_fermentation_to_Butyrate icw(1);Valine_degradation idu(51);Butyrate_metabolism_cluster
fig|6666666.28482.peg.1434	Contig_14	38651	39061	+	Uncharacterized protein, possibly involved in aromatic compounds catabolism	FIG00955696	ff
fig|6666666.28482.peg.1435	Contig_14	40077	39040	-	acyl-CoA dehydrogenase domain protein		

fig|6666666.28482.peg.1436	Contig_14	40164	41324	+	Butyryl-CoA dehydrogenase (EC 1.3.99.2)	FIG00009149	idu(39);Isoleucine_degradation idu(39);Lysine_fermentation idu(39);Butanol_Biosynthesis idu(39);Acetyl-CoA_fermentation_to_Butyrate idu(39);Valine_degradation idu(39);Anaerobic_respiratory_reductases
fig|6666666.28482.peg.1437	Contig_14	42059	41313	-	transcriptional regulator, TetR family		
fig|6666666.28482.peg.1438	Contig_14	42469	43614	+	BarH	FIG00668014	ff
fig|6666666.28482.peg.1439	Contig_14	44406	43633	-	Enoyl-CoA hydratase (EC 4.2.1.17)	FIG00033206	idu(51);n-Phenylalkanoic_acid_degradation idu(51);Polyhydroxybutyrate_metabolism icw(1);Isoleucine_degradation idu(51);Fatty_acid_metabolism_cluster icw(1);Lysine_fermentation icw(1);Butanol_Biosynthesis icw(1);Acetyl-CoA_fermentation_to_Butyrate icw(1);Valine_degradation idu(51);Butyrate_metabolism_cluster
fig|6666666.28482.peg.1440	Contig_14	45539	44454	-	D-3-phosphoglycerate dehydrogenase (EC 1.1.1.95)	FIG00031250	idu(4);Serine_Biosynthesis idu(4);Pyridoxin_(Vitamin_B6)_Biosynthesis
fig|6666666.28482.peg.1441	Contig_14	46654	45536	-	conserved hypothetical protein		

fig|6666666.28482.peg.1442	Contig_14	47497	46703	-	2,4-dihydroxyhept-2-ene-1,7-dioic acid aldolase (EC 4.1.2.-)	FIG00110223	idu(1);Aromatic_amino_acid_degradation idu(1);4-Hydroxyphenylacetic_acid_catabolic_pathway
fig|6666666.28482.peg.1443	Contig_14	47586	48731	+	Acetyl-CoA acetyltransferase	FIG01005417	ff
fig|6666666.28482.peg.1444	Contig_14	49016	49135	+	hypothetical protein		
fig|6666666.28482.peg.1445	Contig_14	50549	49125	-	Long-chain-fatty-acid--CoA ligase (EC 6.2.1.3)	FIG00018699	idu(72);n-Phenylalkanoic_acid_degradation idu(72);Fatty_acid_metabolism_cluster idu(72);Biotin_synthesis_cluster idu(72);Biotin_biosynthesis
fig|6666666.28482.peg.1446	Contig_14	50752	51627	+	Oxidoreductase, short chain dehydrogenase/reductase family	FIG00445780	ff
fig|6666666.28482.peg.1447	Contig_14	52458	51631	-	Enoyl-CoA hydratase (EC 4.2.1.17)	FIG00033206	icw(4);n-Phenylalkanoic_acid_degradation icw(3);Polyhydroxybutyrate_metabolism icw(3);Isoleucine_degradation icw(4);Fatty_acid_metabolism_cluster icw(3);Lysine_fermentation icw(3);Butanol_Biosynthesis icw(3);Acetyl-CoA_fermentation_to_Butyrate icw(3);Valine_degradation icw(3);Butyrate_metabolism_cluster

fig|6666666.28482.peg.1448	Contig_14	52512	53762	+	hypothetical protein	FIG00638284	ff
fig|6666666.28482.peg.1449	Contig_14	53767	54573	+	Enoyl-CoA hydratase (EC 4.2.1.17)	FIG00033206	icw(5);n-Phenylalkanoic_acid_degradation icw(3);Polyhydroxybutyrate_metabolism icw(3);Isoleucine_degradation icw(5);Fatty_acid_metabolism_cluster icw(3);Lysine_fermentation icw(3);Butanol_Biosynthesis icw(3);Acetyl-CoA_fermentation_to_Butyrate icw(3);Valine_degradation icw(3);Butyrate_metabolism_cluster
fig|6666666.28482.peg.1450	Contig_14	54580	55827	+	putative acyl-CoA dehydrogenase	FIG00476656	ff
fig|6666666.28482.peg.1451	Contig_14	55824	56639	+	Enoyl-CoA hydratase (EC 4.2.1.17)	FIG00033206	icw(4);n-Phenylalkanoic_acid_degradation icw(3);Polyhydroxybutyrate_metabolism icw(3);Isoleucine_degradation icw(4);Fatty_acid_metabolism_cluster icw(3);Lysine_fermentation icw(3);Butanol_Biosynthesis icw(3);Acetyl-CoA_fermentation_to_Butyrate icw(3);Valine_degradation icw(3);Butyrate_metabolism_cluster

fig|6666666.28482.peg.1452	Contig_14	56636	57436	+	Enoyl-CoA hydratase (EC 4.2.1.17)	FIG00033206	icw(4);n-Phenylalkanoic_acid_degradation icw(3);Polyhydroxybutyrate_metabolism icw(3);Isoleucine_degradation icw(4);Fatty_acid_metabolism_cluster icw(3);Lysine_fermentation icw(3);Butanol_Biosynthesis icw(3);Acetyl-CoA_fermentation_to_Butyrate icw(3);Valine_degradation icw(3);Butyrate_metabolism_cluster
fig|6666666.28482.peg.1453	Contig_14	57433	58884	+	Long-chain-fatty-acid--CoA ligase (EC 6.2.1.3)	FIG00018699	idu(72);n-Phenylalkanoic_acid_degradation idu(72);Fatty_acid_metabolism_cluster idu(72);Biotin_synthesis_cluster idu(72);Biotin_biosynthesis
fig|6666666.28482.peg.1454	Contig_14	59227	59346	+	hypothetical protein		
fig|6666666.28482.peg.1455	Contig_14	59366	60073	+	FIG00822652: hypothetical protein	FIG00822651	ff
fig|6666666.28482.peg.1456	Contig_14	60169	60531	+	hypothetical protein		
fig|6666666.28482.peg.1457	Contig_14	60544	61146	+	hypothetical protein		
fig|6666666.28482.peg.1458	Contig_14	61143	61901	+	3-oxoacyl-[acyl-carrier protein] reductase (EC 1.1.1.100)	FIG00621114	idu(38);CBSS-246196.1.peg.364 idu(38);CBSS-164757.7.peg.5020 idu(38);Fatty_Acid_Biosynthesis_FASII
fig|6666666.28482.peg.1459	Contig_14	61913	62800	+	luciferase-like protein		
fig|6666666.28482.peg.1460	Contig_14	63027	64205	+	BarH	FIG00668014	ff

fig|6666666.28482.peg.1461	Contig_14	65762	64278	-	hypothetical protein		
fig|6666666.28482.peg.1462	Contig_14	67036	65831	-	Butyryl-CoA dehydrogenase (EC 1.3.99.2)	FIG00009149	icw(2);Isoleucine_degradation icw(2);Lysine_fermentation icw(2);Butanol_Biosynthesis icw(2);Acetyl-CoA_fermentation_to_Butyrate icw(2);Valine_degradation icw(2);Anaerobic_respiratory_reductases
fig|6666666.28482.peg.1463	Contig_14	67998	67030	-	Butyryl-CoA dehydrogenase (EC 1.3.99.2)	FIG00009149	icw(2);Isoleucine_degradation icw(2);Lysine_fermentation icw(2);Butanol_Biosynthesis icw(2);Acetyl-CoA_fermentation_to_Butyrate icw(2);Valine_degradation icw(2);Anaerobic_respiratory_reductases
fig|6666666.28482.peg.1464	Contig_14	69202	68006	-	amidohydrolase 2	FIG00501263	ff
fig|6666666.28482.peg.1465	Contig_14	69450	70565	+	Butyryl-CoA dehydrogenase (EC 1.3.99.2)	FIG00009149	icw(2);Isoleucine_degradation icw(2);Lysine_fermentation icw(2);Butanol_Biosynthesis icw(2);Acetyl-CoA_fermentation_to_Butyrate icw(2);Valine_degradation icw(2);Anaerobic_respiratory_reductases
fig|6666666.28482.peg.1466	Contig_14	70562	71464	+	luciferase		
fig|6666666.28482.peg.1467	Contig_14	71908	71471	-	hypothetical protein	FIG00638284	ff
fig|6666666.28482.peg.1468	Contig_14	71959	72588	+	Isochorismatase (EC 3.3.2.1)	FIG00009017	idu(3);Chorismate:_Intermediate_for_synthesis_of_Tryptophan,_PAPA_antibiotics,_PABA,_3-hydroxyanthranilate_and_more.
fig|6666666.28482.peg.1469	Contig_14	72691	73884	+	BarH	FIG00668014	ff

fig|6666666.28482.peg.1470	Contig_14	73881	74360	+	Acyl dehydratase	FIG01303815	idu(2);CBSS-246196.1.peg.364
fig|6666666.28482.peg.1471	Contig_14	76759	74357	-	FIG00820650: hypothetical protein	FIG00820648	ff
fig|6666666.28482.peg.1472	Contig_14	78219	76756	-	Betaine aldehyde dehydrogenase (EC 1.2.1.8)	FIG01304892	idu(1);Choline_uptake_and_conversion_to_betaine_clusters idu(1);Choline_and_Betaine_Uptake_and_Betaine_Biosynthesis
fig|6666666.28482.peg.1473	Contig_14	78451	79644	+	putative cytochrome P450 hydroxylase	FIG00032437	idu(49);Nitric_oxide_synthase
fig|6666666.28482.peg.1474	Contig_14	79644	80831	+	FadE26_2	FIG01708305	ff
fig|6666666.28482.peg.1475	Contig_14	80849	81421	+	FIG00820262: hypothetical protein	FIG00820260	ff
fig|6666666.28482.peg.1476	Contig_14	81421	81834	+	hypothetical protein		
fig|6666666.28482.peg.1477	Contig_14	81857	82966	+	FIG00821501: hypothetical protein	FIG00821499	ff
fig|6666666.28482.peg.1478	Contig_14	82963	83415	+	involved in the lipid metabolism	FIG01582522	ff
fig|6666666.28482.peg.1479	Contig_14	83412	84581	+	putative thiolase	FIG01122915	ff
fig|6666666.28482.peg.1480	Contig_14	84605	85201	+	Transcriptional regulator, TetR family	FIG00554417	ff
fig|6666666.28482.peg.1481	Contig_14	86083	85211	-	Transcriptional regulator, IclR family	FIG00022569	idu(12);Homogentisate_pathway_of_aromatic_compound_degradation
fig|6666666.28482.peg.1482	Contig_14	86178	87227	+	oxidoreductase, zinc-binding dehydrogenase family protein	FIG00824870	ff
fig|6666666.28482.peg.1483	Contig_14	87227	87634	+	hypothetical protein		
fig|6666666.28482.peg.1484	Contig_14	87627	88460	+	Short-chain dehydrogenase/reductase SDR precursor	FIG00917812	ff
fig|6666666.28482.peg.1485	Contig_14	88469	89422	+	hypothetical protein	FIG00638284	ff

fig|6666666.28482.peg.1486	Contig_14	89645	90871	+	putative cytochrome P450 hydroxylase	FIG00032437	icw(1);Nitric_oxide_synthase
fig|6666666.28482.peg.1487	Contig_14	91052	92059	+	hypothetical protein		
fig|6666666.28482.peg.1488	Contig_14	93387	92194	-	putative cytochrome P450 hydroxylase	FIG00032437	icw(1);Nitric_oxide_synthase
fig|6666666.28482.peg.1489	Contig_14	94655	93423	-	L-carnitine dehydratase/bile acid-inducible protein F	FIG01318850	ff
fig|6666666.28482.peg.1490	Contig_14	95310	94780	-	hypothetical protein		
fig|6666666.28482.peg.1491	Contig_14	95426	97036	+	Long-chain-fatty-acid--CoA ligase (EC 6.2.1.3)	FIG00018699	idu(72);n-Phenylalkanoic_acid_degradation idu(72);Fatty_acid_metabolism_cluster idu(72);Biotin_synthesis_cluster idu(72);Biotin_biosynthesis
fig|6666666.28482.peg.1492	Contig_14	97473	97033	-	hypothetical protein		
fig|6666666.28482.peg.1493	Contig_14	97586	98374	+	Oxidoreductase, short-chain dehydrogenase/reductase family	FIG00824296	ff
fig|6666666.28482.peg.1494	Contig_14	98451	99485	+	FIG00826901: hypothetical protein	FIG00826898	ff
fig|6666666.28482.peg.1495	Contig_14	100264	99482	-	Short-chain dehydrogenase/reductase SDR(EC:1.1.1.100)		
fig|6666666.28482.peg.1496	Contig_14	100350	101162	+	hypothetical protein		
fig|6666666.28482.peg.1497	Contig_14	102161	101199	-	hypothetical protein		
fig|6666666.28482.peg.1498	Contig_14	103214	102372	-	3-oxoacyl-[acyl-carrier protein] reductase (EC 1.1.1.100)	FIG00621114	idu(38);CBSS-246196.1.peg.364 idu(38);CBSS-164757.7.peg.5020 idu(38);Fatty_Acid_Biosynthesis_FASII

fig|6666666.28482.peg.1499	Contig_14	103364	104086	+	short chain dehydrogenase		
fig|6666666.28482.peg.1500	Contig_14	104175	104924	+	oxidoreductase, short chain dehydrogenase/reductase family protein	FIG01225860	ff
fig|6666666.28482.peg.1501	Contig_14	105085	106131	+	hypothetical protein		
fig|6666666.28482.peg.1502	Contig_14	107246	106182	-	conserved hypothetical protein		
fig|6666666.28482.peg.1503	Contig_14	108509	107259	-	putative cytochrome P450 hydroxylase	FIG00032437	idu(49);Nitric_oxide_synthase
fig|6666666.28482.peg.1504	Contig_14	108626	109699	+	hypothetical protein	FIG00638284	ff
fig|6666666.28482.peg.1505	Contig_14	110878	109691	-	hypothetical protein		
fig|6666666.28482.peg.1506	Contig_14	110975	112099	+	FIG00825548: hypothetical protein	FIG00825545	ff
fig|6666666.28482.peg.1507	Contig_14	112248	112790	+	Putative TetR-type transcriptional regulator		
fig|6666666.28482.peg.1508	Contig_14	112911	114191	+	FIG00821608: hypothetical protein	FIG00821607	ff
fig|6666666.28482.peg.1509	Contig_14	114184	114978	+	Short-chain dehydrogenase/reductase SDR	FIG00546160	idu(13);CBSS-164757.7.peg.5020 idu(13);Transcription_repair_cluster
fig|6666666.28482.peg.1510	Contig_14	114975	116444	+	Aldehyde dehydrogenase (EC 1.2.1.3)	FIG01292786	idu(16);Pyruvate_metabolism_II:_acetyl-CoA,_acetogenesis_from_pyruvate icw(1);Glycerolipid_and_Glycerophospholipid_Metabolism_in_Bacteria idu(16);Methylglyoxal_Metabolism

fig|6666666.28482.peg.1511	Contig_14	116484	117629	+	Alcohol dehydrogenase (EC 1.1.1.1)	FIG01007502	idu(17);Glycerolipid_and_Glycerophospholipid_Metabolism_in_Bacteria idu(17);Fermentations:_Mixed_acid idu(17);Butanol_Biosynthesis
fig|6666666.28482.peg.1512	Contig_14	117650	118048	+	hypothetical protein		
fig|6666666.28482.peg.1513	Contig_14	119220	118045	-	L-cysteine:1D-myo-inositol 2-amino-2-deoxy-alpha-D-glucopyranoside ligase MshC	FIG00146048	isu;Glutathione_analogs:_mycothiol
fig|6666666.28482.peg.1514	Contig_14	120095	119307	-	Protein cysQ homolog	FIG01309573	ff
fig|6666666.28482.peg.1515	Contig_14	120938	120150	-	Phosphatidylinositol 3- and 4-kinase family protein	FIG01122160	ff
fig|6666666.28482.peg.1516	Contig_14	121545	120958	-	FIG00820819: hypothetical protein	FIG00820818	ff
fig|6666666.28482.peg.1517	Contig_14	122376	121609	-	Phosphoglycerate mutase (EC 5.4.2.1)	FIG00005840	idu(2);Phosphoglycerate_mutase_protein_family
fig|6666666.28482.peg.1518	Contig_14	122720	122418	-	FIG00821698: hypothetical protein	FIG00821697	ff
fig|6666666.28482.peg.1519	Contig_14	122885	123898	+	Lipoprotein LppL	FIG01001528	ff
fig|6666666.28482.peg.1520	Contig_14	123952	124158	+	FIG00821060: hypothetical protein	FIG00821059	ff
fig|6666666.28482.peg.1521	Contig_14	124158	125276	+	Dihydroorotate dehydrogenase (EC 1.3.3.1)	FIG00000475	ff
fig|6666666.28482.peg.1522	Contig_14	125461	127524	+	hypothetical protein	FIG00638284	ff
fig|6666666.28482.peg.1523	Contig_14	128133	127603	-	Phospholipid-binding protein	FIG01351068	ff
fig|6666666.28482.peg.1524	Contig_14	129505	128159	-	FIG016551: Putative peptidase	FIG01398458	ff
fig|6666666.28482.rna.23	Contig_14	129629	129714	+	tRNA-Leu-GAG		isu;tRNAs

fig|6666666.28482.peg.1525	Contig_14	130098	129898	-	hypothetical protein		
fig|6666666.28482.peg.1526	Contig_14	130254	130931	+	Phosphoribosyl transferase domain protein	FIG01348412	ff
fig|6666666.28482.peg.1527	Contig_14	131297	130935	-	Probable transmembrane protein	FIG01276430	ff
fig|6666666.28482.peg.1528	Contig_14	132182	131379	-	FIG055075: Possibly a cell division protein, antigen 84 in Mycobacteria	FIG00340323	ff
fig|6666666.28482.peg.1529	Contig_14	132724	132422	-	FIG021764: Possible membrane protein	FIG00021764	ff
fig|6666666.28482.peg.1530	Contig_14	133473	132844	-	FIG021292: hypothetical protein	FIG00340333	ff
fig|6666666.28482.peg.1531	Contig_14	134315	133539	-	Hypothetical protein YggS, proline synthase co-transcribed bacterial homolog PROSC	FIG00003955	isu;A_Hypothetical_Protein_Related_to_Proline_Metabolism
fig|6666666.28482.peg.1532	Contig_14	134521	134321	-	COG1496: Uncharacterized conserved protein	FIG00003370	ff
fig|6666666.28482.peg.1533	Contig_140	2017	104	-	1-deoxy-D-xylulose 5-phosphate synthase (EC 2.2.1.7)	FIG00000343	isu;Thiamin_biosynthesis isu;Nonmevalonate_Branch_of_Isoprenoid_Biosynthesis isu;Pyridoxin_(Vitamin_B6)_Biosynthesis isu;Isoprenoid_Biosynthesis
fig|6666666.28482.peg.1534	Contig_140	2071	2514	+	FIG00822000: hypothetical protein	FIG00821999	ff
fig|6666666.28482.peg.1535	Contig_140	2540	3829	+	Na+/H+ antiporter NhaD type	FIG01261528	ff

fig|6666666.28482.peg.1536	Contig_140	5121	3913	-	23S rRNA (Uracil-5-) -methyltransferase RumA (EC 2.1.1.-)	FIG01304282	isu;RNA_methylation
fig|6666666.28482.peg.1537	Contig_140	7124	5130	-	PROBABLE CONSERVED INTEGRAL MEMBRANE ALANINE AND VALINE AND LEUCINE RICH PROTEIN	FIG00945276	ff
fig|6666666.28482.peg.1538	Contig_140	7214	7897	+	Trk system potassium uptake protein TrkA	FIG00000838	icw(1);Potassium_homeostasis
fig|6666666.28482.peg.1539	Contig_140	7894	8568	+	Trk system potassium uptake protein TrkA	FIG00000838	icw(1);Potassium_homeostasis
fig|6666666.28482.peg.1540	Contig_140	9382	8708	-	PROBABLE CONSERVED INTEGRAL MEMBRANE ALANINE AND LEUCINE RICH PROTEIN	FIG00672006	ff
fig|6666666.28482.peg.1541	Contig_140	9747	9379	-	FIG00945047: hypothetical protein	FIG00945046	ff
fig|6666666.28482.peg.1542	Contig_141	36	278	+	Ornithine carbamoyltransferase (EC 2.1.3.3)	FIG00000084	icw(1);Arginine_and_Ornithine_Degradation icw(1);Arginine_Deiminase_Pathway icw(3);Arginine_Biosynthesis_--_gjo icw(3);Arginine_Biosynthesis_extended

fig|6666666.28482.peg.1543	Contig_141	275	769	+	Arginine pathway regulatory protein ArgR, repressor of arg regulon	FIG00003270	isu;Arginine_and_Ornithine_Degradation isu;Arginine_Deiminase_Pathway isu;Arginine_Biosynthesis_--_gjo isu;Arginine_Biosynthesis_extended
fig|6666666.28482.peg.1544	Contig_141	782	1993	+	Argininosuccinate synthase (EC 6.3.4.5)	FIG00000301	icw(2);Arginine_Biosynthesis_--_gjo icw(2);Arginine_Biosynthesis_extended
fig|6666666.28482.peg.1545	Contig_141	2073	3491	+	Argininosuccinate lyase (EC 4.3.2.1)	FIG00000333	icw(1);Arginine_Biosynthesis_--_gjo icw(1);Arginine_Biosynthesis_extended
fig|6666666.28482.peg.1546	Contig_141	3986	5047	+	Chalcone synthase (EC 2.3.1.74)	FIG00443156	ff
fig|6666666.28482.peg.1547	Contig_141	5181	11513	+	Malonyl CoA-acyl carrier protein transacylase (EC 2.3.1.39)	FIG00000319	idu(7);Fatty_Acid_Biosynthesis_FASII
fig|6666666.28482.peg.1548	Contig_141	11529	17858	+	Malonyl CoA-acyl carrier protein transacylase (EC 2.3.1.39)	FIG00000319	icw(1);Fatty_Acid_Biosynthesis_FASII
fig|6666666.28482.peg.1549	Contig_141	17897	20986	+	Malonyl CoA-acyl carrier protein transacylase (EC 2.3.1.39)	FIG00000319	icw(1);Fatty_Acid_Biosynthesis_FASII
fig|6666666.28482.peg.1550	Contig_141	21168	22229	+	Chalcone synthase (EC 2.3.1.74)	FIG00443156	ff
fig|6666666.28482.peg.1551	Contig_142	155	292	+	hypothetical protein		

fig|6666666.28482.peg.1552	Contig_143	1347	52	-	Integrase, catalytic region		
fig|6666666.28482.peg.1553	Contig_144	824	123	-	Transcriptional regulator, IclR family	FIG00022569	idu(12);Homogentisate_pathway_of_aromatic_compound_degradation
fig|6666666.28482.peg.1554	Contig_144	890	2326	+	3-isopropylmalate dehydratase large subunit (EC 4.2.1.33)	FIG00000280	icw(1);Branched-Chain_Amino_Acid_Biosynthesis icw(1);Leucine_Biosynthesis
fig|6666666.28482.peg.1555	Contig_144	2344	2940	+	3-isopropylmalate dehydratase small subunit (EC 4.2.1.33)	FIG00000282	idu(1);Branched-Chain_Amino_Acid_Biosynthesis idu(1);Leucine_Biosynthesis
fig|6666666.28482.peg.1556	Contig_144	3155	3796	+	DNA-binding protein HU / low-complexity, AKP-rich domain	FIG01296719	isu;DNA_structural_proteins,_bacterial
fig|6666666.28482.peg.1557	Contig_144	4761	3862	-	Possible hydrolase mutT1 (EC 3.-.-.-)	FIG00544763	ff
fig|6666666.28482.peg.1558	Contig_144	7044	4873	-	Polyphosphate kinase (EC 2.7.4.1)	FIG00000504	isu;Purine_conversions isu;High_affinity_phosphate_transporter_and_control_of_PHO_regulon isu;Polyphosphate isu;Phosphate_metabolism
fig|6666666.28482.peg.1559	Contig_144	7824	7168	-	2-phospho-L-lactate guanylyltransferase (EC 2.7.7.68)	FIG01121259	ff
fig|6666666.28482.peg.1560	Contig_144	7915	8934	+	Glycerol-3-phosphate dehydrogenase [NAD(P)+] (EC 1.1.1.94)	FIG00000309	idu(1);Glycerol_and_Glycerol-3-phosphate_Uptake_and_Utilization idu(1);Glycerolipid_and_Glycerophospholipid_Metabolism_in_Bacteria
fig|6666666.28482.peg.1561	Contig_144	9014	10123	+	D-alanine--D-alanine ligase (EC 6.3.2.4)	FIG00000350	isu;Peptidoglycan_biosynthesis--gjo isu;Peptidoglycan_Biosynthesis

fig|6666666.28482.peg.1562	Contig_144	10696	10124	-	Possible membrane protein	FIG01121868	ff
fig|6666666.28482.peg.1563	Contig_144	10812	11774	+	Thiamine-monophosphate kinase (EC 2.7.4.16)	FIG00000570	isu;Riboflavin_synthesis_cluster isu;Thiamin_biosynthesis
fig|6666666.28482.peg.1564	Contig_144	11784	12074	+	FIG00821587: hypothetical protein	FIG00821585	ff
fig|6666666.28482.peg.1565	Contig_144	12071	12754	+	Uracil-DNA glycosylase, family 1	FIG00000538	isu;DNA_Repair_Base_Excision isu;Uracil-DNA_glycosylase
fig|6666666.28482.peg.1566	Contig_144	12751	13692	+	Quinone oxidoreductase (EC 1.6.5.5)	FIG00002982	idu(7);Quinone_oxidoreductase_family
fig|6666666.28482.peg.1567	Contig_144	13968	13774	-	LSU ribosomal protein L28p	FIG00000266	isu;Ribosome_LSU_bacterial
fig|6666666.28482.peg.1568	Contig_144	14216	15889	+	Dihydroxyacetone kinase family protein	FIG00042616	isu;Glycerolipid_and_Glycerophospholipid_Metabolism_in_Bacteria
fig|6666666.28482.peg.1569	Contig_144	15892	18132	+	ATP-dependent DNA helicase RecG (EC 3.6.1.-)	FIG00021289	isu;DNA-replication
fig|6666666.28482.peg.1570	Contig_144	18129	18839	+	Possible membrane protein	FIG01121868	ff
fig|6666666.28482.peg.1571	Contig_144	19700	18849	-	2,5-diketo-D-gluconic acid reductase (EC 1.1.1.-)	FIG01068018	ff
fig|6666666.28482.peg.1572	Contig_144	19954	21066	+	lipase-esterase (lipN)	FIG01338277	ff
fig|6666666.28482.peg.1573	Contig_144	21142	21900	+	POSSIBLE CONSERVED MEMBRANE OR SECRETED PROTEIN	FIG01328650	ff
fig|6666666.28482.peg.1574	Contig_144	21897	22532	+	Putative ESX-1 secretion system component Rv3877	FIG00092051	ff

fig|6666666.28482.peg.1575	Contig_144	22537	23091	+	Ribosomal RNA small subunit methyltransferase D (EC 2.1.1.-)	FIG00005547	ff
fig|6666666.28482.peg.1576	Contig_144	23169	23651	+	Phosphopantetheine adenylyltransferase (EC 2.7.7.3)	FIG00000261	isu;CBSS-266117.6.peg.1260 isu;Coenzyme_A_Biosynthesis
fig|6666666.28482.peg.1577	Contig_144	24852	23671	-	PROBABLE INTEGRAL MEMBRANE PROTEIN	FIG01315135	ff
fig|6666666.28482.peg.1578	Contig_144	25205	25942	+	Cell division initiation protein	FIG00534428	ff
fig|6666666.28482.peg.1579	Contig_144	26103	26699	+	COG1399 protein, clustered with ribosomal protein L32p	FIG01269488	ff
fig|6666666.28482.peg.1580	Contig_144	26696	27409	+	Ribonuclease III (EC 3.1.26.3)	FIG00000208	isu;RNA_processing_and_degradation,_bacterial
fig|6666666.28482.peg.1581	Contig_144	27426	28298	+	Formamidopyrimidine-DNA glycosylase (EC 3.2.2.23)	FIG00035634	idu(3);DNA_Repair_Base_Excision
fig|6666666.28482.peg.1582	Contig_144	29107	28331	-	FIG00823221: hypothetical protein	FIG01311952	ff
fig|6666666.28482.peg.1583	Contig_144	29160	29582	+	FIG00997866: hypothetical protein	FIG00820325	ff
fig|6666666.28482.peg.1584	Contig_144	31038	29542	-	hypothetical protein	FIG00638284	ff
fig|6666666.28482.peg.1585	Contig_146	43	1278	+	PPE family protein	FIG00013075	idu(57);CBSS-164757.7.peg.5020
fig|6666666.28482.peg.1586	Contig_146	2324	1284	-	FIG019327: Probable conserved membrane protein	FIG00019327	ff
fig|6666666.28482.peg.1587	Contig_146	3448	2324	-	Phosphatidylinositol alpha-mannosyltransferase (EC 2.4.1.57)	FIG00005657	ff

fig|6666666.28482.peg.1588	Contig_146	4422	3448	-	Lauroyl/myristoyl acyltransferase involved in lipid A biosynthesis (Lauroyl/myristoyl acyltransferase)	FIG00006567	ff
fig|6666666.28482.peg.1589	Contig_146	5042	4419	-	CDP-diacylglycerol--glycerol-3-phosphate 3-phosphatidyltransferase (EC 2.7.8.5)	FIG00001550	idu(2);Glycerolipid_and_Glycerophospholipid_Metabolism_in_Bacteria
fig|6666666.28482.peg.1590	Contig_146	5605	5048	-	FIG049476: HIT family protein	FIG00049476	ff
fig|6666666.28482.peg.1591	Contig_146	7652	5598	-	Threonyl-tRNA synthetase (EC 6.1.1.3)	FIG00000101	isu;tRNA_aminoacylation,_Thr
fig|6666666.28482.peg.1592	Contig_146	7692	7910	+	hypothetical protein		
fig|6666666.28482.peg.1593	Contig_146	8685	7918	-	hypothetical protein	FIG00638284	ff
fig|6666666.28482.peg.1594	Contig_146	10132	8867	-	N-acetyl-L,L-diaminopimelate deacetylase (EC 3.5.1.47)	FIG00016846	isu;Lysine_Biosynthesis_DAP_Pathway,_GJO_scratch isu;Lysine_Biosynthesis_DAP_Pathway
fig|6666666.28482.peg.1595	Contig_146	10838	10125	-	putative integral membrane protein		
fig|6666666.28482.peg.1596	Contig_146	11499	10876	-	FIG00823977: hypothetical protein	FIG00823976	ff
fig|6666666.28482.peg.1597	Contig_147	180	1121	+	LpqP protein	FIG00825448	ff
fig|6666666.28482.peg.1598	Contig_147	1203	3806	+	hypothetical protein	FIG00638284	ff
fig|6666666.28482.peg.1599	Contig_147	7121	3873	-	Adenylate cyclase (EC 4.6.1.1) / Guanylate cyclase (EC 4.6.1.2)	FIG00004786	idu(25);cAMP_signaling_in_bacteria
fig|6666666.28482.peg.1600	Contig_147	7787	7179	-	Transcriptional regulator, TetR family	FIG00554417	ff
fig|6666666.28482.peg.1601	Contig_147	7856	8686	+	Oxidoreductase	FIG01321859	ff

fig|6666666.28482.peg.1602	Contig_147	8723	9484	+	FIG00825820: hypothetical protein	FIG00825815	ff
fig|6666666.28482.peg.1603	Contig_147	11035	9581	-	hypothetical protein	FIG00638284	ff
fig|6666666.28482.peg.1604	Contig_148	103	1308	+	DNA polymerase IV (EC 2.7.7.7)	FIG00023943	idu(1);DNA_repair,_bacterial
fig|6666666.28482.peg.1605	Contig_148	1703	3625	+	hypothetical protein	FIG00638284	ff
fig|6666666.28482.peg.1606	Contig_15	268	2142	+	Serine/threonine protein kinase PrkC, regulator of stationary phase	FIG00030191	ff
fig|6666666.28482.peg.1607	Contig_15	2818	2120	-	Anthranilate synthase, amidotransferase component (EC 4.1.3.27) @ Para-aminobenzoate synthase, amidotransferase component (EC 2.6.1.85)	FIG00132685	isu;Chorismate:_Intermediate_for_synthesis_of_Tryptophan,_PAPA_antibiotics,_PABA,_3-hydroxyanthranilate_and_more. isu;Chorismate:_Intermediate_for_synthesis_of_Tryptophan,_PAPA_antibiotics,_PABA,_3-hydroxyanthranilate_and_more. isu;Folate_Biosynthesis isu;Tryptophan_synthesis isu;Tryptophan_synthesis
fig|6666666.28482.peg.1608	Contig_15	3601	2858	-	FIG215594: Membrane spanning protein	FIG00480581	ff
fig|6666666.28482.peg.1609	Contig_15	3722	4003	+	FIG018426: putative septation inhibitor protein	FIG01124317	ff
fig|6666666.28482.peg.1610	Contig_15	4240	4584	+	FIG020554: membrane protein	FIG00666500	icw(1);Peptidyl-prolyl_cis-trans_isomerase_containing_cluster
fig|6666666.28482.peg.1611	Contig_15	5127	4597	-	Peptidyl-prolyl cis-trans isomerase (EC 5.2.1.8)	FIG01955868	idu(1);Peptidyl-prolyl_cis-trans_isomerase_containing_cluster

fig|6666666.28482.peg.1612	Contig_15	5261	5692	+	FIG024317: hypothetical protein	FIG01310638	icw(2);Peptidyl-prolyl_cis-trans_isomerase_containing_cluster
fig|6666666.28482.peg.1613	Contig_15	6910	5696	-	putative cytochrome P450 hydroxylase	FIG00032437	idu(49);Nitric_oxide_synthase
fig|6666666.28482.peg.1614	Contig_15	7107	7673	+	Transcriptional regulator, TetR family	FIG00628138	ff
fig|6666666.28482.peg.1615	Contig_15	7670	7888	+	hypothetical protein		
fig|6666666.28482.peg.1616	Contig_15	9164	7899	-	PE-PGRS family protein		idu(16);PPE_gene_cluster_in_Mycobacteria
fig|6666666.28482.peg.1617	Contig_15	9778	10077	+	FIG018298: PE family protein		icw(1);PPE_gene_cluster_in_Mycobacteria
fig|6666666.28482.peg.1618	Contig_15	10084	11247	+	PPE family protein	FIG00013075	idu(57);CBSS-164757.7.peg.5020
fig|6666666.28482.peg.1619	Contig_15	11485	11279	-	putative long chain fatty acid-CoA synthetase Faa4p		
fig|6666666.28482.peg.1620	Contig_15	12055	11942	-	hypothetical protein		
fig|6666666.28482.rna.24	Contig_15	12209	12137	-	tRNA-Ala-TGC		
fig|6666666.28482.rna.25	Contig_15	12434	12361	-	tRNA-Ile-GAT		
fig|6666666.28482.peg.1621	Contig_15	13474	12584	-	FIG187021: hypothetical protein	FIG00735262	icw(2);DNA_replication_cluster_1
fig|6666666.28482.peg.1622	Contig_15	16107	13591	-	DNA gyrase subunit A (EC 5.99.1.3)	FIG00000080	icw(1);DNA_gyrase_subunits icw(1);DNA_topoisomerases,_Type_II,_ATP-dependent icw(1);DNA_replication_cluster_1 icw(1);Resistance_to_fluoroquinolones

fig|6666666.28482.peg.1623	Contig_15	18180	16147	-	DNA gyrase subunit B (EC 5.99.1.3)	FIG00028203	isu;DNA_gyrase_subunits isu;DNA_topoisomerases,_Type_II,_ATP-dependent isu;DNA_replication_cluster_1 isu;Resistance_to_fluoroquinolones
fig|6666666.28482.peg.1624	Contig_15	18992	18435	-	Zn-ribbon-containing, possibly RNA-binding protein and truncated derivatives	FIG00403940	icw(3);DNA_replication_cluster_1
fig|6666666.28482.peg.1625	Contig_15	20131	18989	-	DNA recombination and repair protein RecF	FIG00000412	icw(5);DNA_replication_cluster_1
fig|6666666.28482.peg.1626	Contig_15	21331	20132	-	DNA polymerase III beta subunit (EC 2.7.7.7)	FIG00066425	icw(1);DNA-replication icw(1);DNA_replication_cluster_1
fig|6666666.28482.peg.1627	Contig_15	23369	21813	-	Chromosomal replication initiator protein DnaA	FIG00000441	isu;DNA-replication icw(2);DNA_replication_cluster_1
fig|6666666.28482.peg.1628	Contig_15	23960	24103	+	LSU ribosomal protein L34p	FIG00000471	isu;Ribosome_LSU_bacterial icw(3);RNA_modification_cluster
fig|6666666.28482.peg.1629	Contig_15	24177	24482	+	Ribonuclease P protein component (EC 3.1.26.5)	FIG00092649	isu;tRNA_processing icw(1);RNA_modification_cluster
fig|6666666.28482.peg.1630	Contig_15	24506	24835	+	Protein YidD	FIG00004843	icw(2);RNA_modification_cluster icw(2);Mycobacterium_virulence_operon_involved_in_an_unknown_function_with_a_Jag_Protein_and_YidC_and_YidD

fig|6666666.28482.peg.1631	Contig_15	24825	25949	+	Inner membrane protein translocase component YidC, long form	FIG00002818	isu;CTP_synthase_(EC_6.3.4.2)_cluster isu;RNA_modification_cluster isu;Mycobacterium_virulence_operon_involved_in_an_unknown_function_with_a_Jag_Protein_and_YidC_and_YidD
fig|6666666.28482.peg.1632	Contig_15	26031	26618	+	RNA-binding protein Jag	FIG00001656	icw(1);Mycobacterium_virulence_operon_involved_in_an_unknown_function_with_a_Jag_Protein_and_YidC_and_YidD
fig|6666666.28482.peg.1633	Contig_15	26676	27422	+	rRNA small subunit 7-methylguanosine (m7G) methyltransferase GidB	FIG01089612	isu;RNA_modification_and_chromosome_partitioning_cluster isu;RNA_methylation
fig|6666666.28482.peg.1634	Contig_15	27419	28399	+	Chromosome (plasmid) partitioning protein ParA / Sporulation initiation inhibitor protein Soj	FIG00006461	icw(2);RNA_modification_and_chromosome_partitioning_cluster idu(2);Bacterial_Cell_Division idu(2);Ribosome_post-transcriptional_modification_and_chromosomal_segregation_cluster icw(1);Bacterial_Cytoskeleton icw(1);Bacterial_Cytoskeleton icw(1);Plasmid_replication
fig|6666666.28482.peg.1635	Contig_15	28423	29406	+	Chromosome (plasmid) partitioning protein ParB / Stage 0 sporulation protein J	FIG00021843	icw(1);RNA_modification_and_chromosome_partitioning_cluster isu;Bacterial_Cytoskeleton isu;Bacterial_Cytoskeleton isu;Plasmid_replication
fig|6666666.28482.peg.1636	Contig_15	29682	30434	+	FIG007808: hypothetical protein	FIG00545451	ff

fig|6666666.28482.peg.1637	Contig_15	31651	30431	-	N-acetylmuramoyl-L-alanine amidase (EC 3.5.1.28)	FIG00001385	isu;Murein_Hydrolases isu;Recycling_of_Peptidoglycan_Amino_Acids
fig|6666666.28482.peg.1638	Contig_15	32137	31784	-	Thioredoxin reductase (EC 1.8.1.9)	FIG00018396	icw(1);pyrimidine_conversions icw(1);Thioredoxin-disulfide_reductase icw(1);Glycine_reductase,_sarcosine_reductase_and_betaine_reductase
fig|6666666.28482.peg.1639	Contig_15	33141	32134	-	Thioredoxin reductase (EC 1.8.1.9)	FIG00018396	icw(1);pyrimidine_conversions icw(1);Thioredoxin-disulfide_reductase icw(1);Glycine_reductase,_sarcosine_reductase_and_betaine_reductase
fig|6666666.28482.peg.1640	Contig_15	34051	33320	-	FIG00999975: hypothetical protein	FIG00822882	ff
fig|6666666.28482.peg.1641	Contig_15	34655	34044	-	RNA polymerase sigma-70 factor, ECF subfamily	FIG00952839	ff
fig|6666666.28482.peg.1642	Contig_15	38232	34729	-	Proposed peptidoglycan lipid II flippase MurJ	FIG00001204	isu;Peptidoglycan_lipid_II_flippase
fig|6666666.28482.peg.1643	Contig_15	38323	38595	+	hypothetical protein		
fig|6666666.28482.peg.1644	Contig_15	38592	39620	+	Mobile element protein	FIG01306568	ff
fig|6666666.28482.peg.1645	Contig_15	41998	39671	-	hypothetical protein		
fig|6666666.28482.peg.1646	Contig_15	42810	42055	-	MutT/nudix family protein	FIG01323436	ff
fig|6666666.28482.peg.1647	Contig_15	42901	44343	+	tRNA nucleotidyltransferase (EC 2.7.7.21) (EC 2.7.7.25)	FIG00000289	isu;Polyadenylation_bacterial isu;tRNA_nucleotidyltransferase

fig|6666666.28482.peg.1648	Contig_15	44894	44403	-	hypothetical protein		
fig|6666666.28482.peg.1649	Contig_15	44919	45863	+	Antiactivator of flagellar biosynthesis FleN, an ATPase	FIG00825705	ff
fig|6666666.28482.peg.1650	Contig_15	45975	46622	+	hypothetical protein		
fig|6666666.28482.peg.1651	Contig_15	46619	48325	+	hypothetical protein	FIG00638284	ff
fig|6666666.28482.peg.1652	Contig_15	48458	48589	+	hypothetical protein		
fig|6666666.28482.peg.1653	Contig_15	48723	49397	+	PROBABLE CONSERVED MEMBRANE PROTEIN	FIG00662487	ff
fig|6666666.28482.peg.1654	Contig_15	49763	49413	-	WhiB-type transcription regulator	FIG00019991	idu(2);WhiB_and_WhiB-type_regulatory_proteins_
fig|6666666.28482.peg.1655	Contig_15	50894	50232	-	Phosphate transport system regulatory protein PhoU	FIG00104848	idu(3);High_affinity_phosphate_transporter_and_control_of_PHO_regulon idu(3);Phosphate_metabolism
fig|6666666.28482.peg.1656	Contig_15	51510	51046	-	FIG00822575: hypothetical protein	FIG00822574	ff
fig|6666666.28482.peg.1657	Contig_15	52360	51512	-	FIG00822859: hypothetical protein	FIG00822857	ff
fig|6666666.28482.peg.1658	Contig_15	52471	53346	+	RNA polymerase sigma-70 factor	FIG01303902	idu(4);CBSS-313593.3.peg.2729 idu(4);Transcription_initiation,_bacterial_sigma_factors
fig|6666666.28482.peg.1659	Contig_15	54089	53343	-	Antiactivator of flagellar biosynthesis FleN, an ATPase	FIG00825705	ff
fig|6666666.28482.peg.1660	Contig_15	56101	54902	-	NADH:flavin oxidoreductases, Old Yellow Enzyme family	FIG01338127	ff

fig|6666666.28482.peg.1661	Contig_15	56159	57007	+	Methylenetetrahydrofolate dehydrogenase (NADP+) (EC 1.5.1.5) / Methenyltetrahydrofolate cyclohydrolase (EC 3.5.4.9)	FIG00000105	isu;One-carbon_metabolism_by_tetrahydropterines isu;One-carbon_metabolism_by_tetrahydropterines isu;Serine-glyoxylate_cycle isu;Serine-glyoxylate_cycle
fig|6666666.28482.peg.1662	Contig_15	57011	57286	+	hypothetical protein	FIG00638284	ff
fig|6666666.28482.peg.1663	Contig_15	57899	57459	-	hypothetical protein	FIG00638284	ff
fig|6666666.28482.peg.1664	Contig_15	58160	59629	+	probable oxidoreductase	FIG01124638	ff
fig|6666666.28482.peg.1665	Contig_15	59629	60009	+	Glyoxalase family protein	FIG00007915	ff
fig|6666666.28482.peg.1666	Contig_150	152	814	+	PROBABLE CONSERVED TRANSMEMBRANE PROTEIN	FIG00662132	ff
fig|6666666.28482.peg.1667	Contig_150	819	1643	+	Glycerophosphoryl diester phosphodiesterase (EC 3.1.4.46)	FIG00002239	idu(1);Glycerol_and_Glycerol-3-phosphate_Uptake_and_Utilization idu(1);CBSS-176299.4.peg.1996A
fig|6666666.28482.peg.1668	Contig_150	2507	1644	-	Cell envelope-associated transcriptional attenuator LytR-CpsA-Psr, subfamily A1 (as in PMID19099556)	FIG00025213	idu(3);Cell_envelope-associated_LytR-CpsA-Psr_transcriptional_attenuators
fig|6666666.28482.peg.1669	Contig_151	1303	131	-	Acetylornithine aminotransferase (EC 2.6.1.11)	FIG00001168	icw(3);Arginine_Biosynthesis_--_gjo icw(3);Arginine_Biosynthesis_extended

fig|6666666.28482.peg.1670	Contig_151	2205	1318	-	Acetylglutamate kinase (EC 2.7.2.8)	FIG00000383	icw(1);Arginine_Biosynthesis_--_gjo icw(1);Arginine_Biosynthesis_extended
fig|6666666.28482.peg.1671	Contig_151	3416	2202	-	Glutamate N-acetyltransferase (EC 2.3.1.35) / N-acetylglutamate synthase (EC 2.3.1.1)	FIG00000630	isu;Arginine_Biosynthesis_--_gjo icw(1);Arginine_Biosynthesis_--_gjo isu;Arginine_Biosynthesis_extended icw(1);Arginine_Biosynthesis_extended
fig|6666666.28482.peg.1672	Contig_151	4456	3413	-	N-acetyl-gamma-glutamyl-phosphate reductase (EC 1.2.1.38)	FIG00000358	icw(2);Arginine_Biosynthesis_--_gjo icw(2);Arginine_Biosynthesis_extended
fig|6666666.28482.peg.1673	Contig_151	5307	4606	-	hypothetical protein		
fig|6666666.28482.peg.1674	Contig_153	982	47	-	PPE family protein	FIG00013075	idu(57);CBSS-164757.7.peg.5020
fig|6666666.28482.peg.1675	Contig_153	2636	1041	-	hypothetical protein	FIG00638284	ff
fig|6666666.28482.peg.1676	Contig_153	3389	2880	-	Ubiquinone/menaquinone biosynthesis methyltransferase UbiE (EC 2.1.1.-) @ 2-heptaprenyl-1,4-naphthoquinone methyltransferase (EC 2.1.1.163)	FIG00006851	isu;Menaquinone_and_Phylloquinone_Biosynthesis_--_gjo isu;Menaquinone_and_Phylloquinone_Biosynthesis isu;Menaquinone_and_Phylloquinone_Biosynthesis isu;Menaquinone_Biosynthesis_via_Futalosine_--_gjo idu(2);Menaquinone_Biosynthesis_via_Futalosine idu(2);Ubiquinone_Biosynthesis
fig|6666666.28482.peg.1677	Contig_153	4120	3506	-	FIG00820129: hypothetical protein	FIG01359873	ff
fig|6666666.28482.peg.1678	Contig_153	5322	4117	-	FIG00826834: hypothetical protein	FIG00826832	ff

fig|6666666.28482.peg.1679	Contig_153	6252	5368	-	Short-chain dehydrogenase/reductase SDR	FIG00546160	idu(13);CBSS-164757.7.peg.5020 idu(13);Transcription_repair_cluster
fig|6666666.28482.peg.1680	Contig_153	6810	7619	+	RNA polymerase sigma factor SigB	FIG00132885	idu(2);SigmaB_stress_responce_regulation idu(2);Biofilm_formation_in_Staphylococcus idu(2);Sigma-B_stress_response_cluster_1 idu(2);Transcription_initiation,_bacterial_sigma_factors idu(2);Methicillin_resistance_in_Staphylococci
fig|6666666.28482.peg.1681	Contig_153	7736	8362	+	FIG00825727: hypothetical protein	FIG00825726	ff
fig|6666666.28482.peg.1682	Contig_153	9985	8699	-	Nitrate reductase [NADH] (EC 1.7.1.1)	FIG01357562	ff
fig|6666666.28482.peg.1683	Contig_155	1232	21	-	Mobile element protein	FIG01306568	ff
fig|6666666.28482.peg.1684	Contig_156	43	1050	+	MCE-family lipoprotein LprK (MCE-family lipoprotein Mce1e)	FIG00819998	icw(3);Mycobacterium_virulence_operon_MCE_involved_in_cell_invasion
fig|6666666.28482.peg.1685	Contig_156	1044	2594	+	MCE-family protein Mce1F	FIG00821171	icw(1);Mycobacterium_virulence_operon_MCE_involved_in_cell_invasion
fig|6666666.28482.peg.1686	Contig_156	2606	3373	+	FIG033430: Probable conserved MCE associated membrane protein	FIG01306442	icw(2);Mycobacterium_virulence_operon_MCE_involved_in_cell_invasion
fig|6666666.28482.peg.1687	Contig_156	3370	4356	+	FIG033285: Conserved MCE associated transmembrane protein	FIG00821527	icw(4);Mycobacterium_virulence_operon_MCE_involved_in_cell_invasion

fig|6666666.28482.peg.1688	Contig_156	4353	4907	+	FIG034772: Probable conserved MCE associated protein	FIG00821709	icw(5);Mycobacterium_virulence_operon_MCE_involved_in_cell_invasion
fig|6666666.28482.peg.1689	Contig_156	4874	5608	+	FIG030769: Probable conserved MCE associated membrane protein	FIG01306441	isu;Mycobacterium_virulence_operon_MCE_involved_in_cell_invasion
fig|6666666.28482.peg.1690	Contig_156	5632	6321	+	membrane protein, putative	FIG01288841	ff
fig|6666666.28482.peg.1691	Contig_156	7396	6314	-	Possible lipoprotein LprO	FIG00823768	ff
fig|6666666.28482.peg.1692	Contig_156	8855	7503	-	Possible membrane protein	FIG01121868	ff
fig|6666666.28482.peg.1693	Contig_156	9694	8969	-	Pirin	FIG01319925	ff
fig|6666666.28482.peg.1694	Contig_156	10559	9705	-	Transcriptional regulator, IclR family	FIG00022569	idu(12);Homogentisate_pathway_of_aromatic_compound_degradation
fig|6666666.28482.peg.1695	Contig_156	10656	11765	+	FIG00824416: hypothetical protein	FIG00824413	ff
fig|6666666.28482.peg.1696	Contig_156	12677	11742	-	Permease of the drug/metabolite transporter (DMT) superfamily	FIG00846773	ff
fig|6666666.28482.peg.1697	Contig_156	12836	13687	+	LysR	FIG01414472	ff
fig|6666666.28482.peg.1698	Contig_156	15103	13697	-	FIG00825281: hypothetical protein	FIG00825280	ff
fig|6666666.28482.peg.1699	Contig_156	16101	15100	-	RNA polymerase factor sigma-70	FIG00820006	ff
fig|6666666.28482.peg.1700	Contig_156	16197	17036	+	Monoglyceride lipase (EC 3.1.1.23)	FIG01303922	isu;Triacylglycerol_metabolism
fig|6666666.28482.peg.1701	Contig_157	308	736	+	Chromosome partition protein smc	FIG00026918	isu;DNA_structural_proteins,_bacterial

fig|6666666.28482.peg.1702	Contig_159	69	311	+	PE-PGRS family protein		idu(16);PPE_gene_cluster_in_Mycobacteria
fig|6666666.28482.peg.1703	Contig_159	910	398	-	Membrane protein 2, distant similarity to thiosulphate:quinone oxidoreductase DoxD	FIG00006938	ff
fig|6666666.28482.peg.1704	Contig_159	1185	2681	+	Pyruvate,phosphate dikinase (EC 2.7.9.1)	FIG00001076	isu;Pyruvate_metabolism_I:_anaplerotic_reactions,_PEP
fig|6666666.28482.peg.1705	Contig_159	2678	3292	+	FIG00823918: hypothetical protein	FIG00823917	ff
fig|6666666.28482.peg.1706	Contig_159	4540	3296	-	FIG028963: hypothetical protein	FIG00823859	ff
fig|6666666.28482.peg.1707	Contig_159	5458	4553	-	Probable epoxide hydrolase ephC (EC 3.3.2.9)	FIG01350511	ff
fig|6666666.28482.peg.1708	Contig_159	6086	5604	-	3-demethylubiquinone-9 3-methyltransferase	FIG01311947	ff
fig|6666666.28482.peg.1709	Contig_159	6223	7092	+	Possible peroxidase bpoB (Non-haem peroxidase) (EC 1.11.1.-)	FIG01316813	ff
fig|6666666.28482.peg.1710	Contig_159	8111	7089	-	6-phosphogluconate dehydrogenase, decarboxylating (EC 1.1.1.44)	FIG00000405	icw(1);Pentose_phosphate_pathway idu(1);D-gluconate_and_ketogluconates_metabolism
fig|6666666.28482.peg.1711	Contig_159	9533	8124	-	Glucose-6-phosphate 1-dehydrogenase (EC 1.1.1.49)	FIG00000332	idu(1);Pentose_phosphate_pathway
fig|6666666.28482.peg.1712	Contig_159	9646	10470	+	Putative adenylate/guanylate cyclase	FIG01409435	ff

fig|6666666.28482.peg.1713	Contig_159	10631	10422	-	FIG00820811: hypothetical protein	FIG00820810	ff
fig|6666666.28482.peg.1714	Contig_159	10727	11407	+	FIG022780: hypothetical protein	FIG00827818	ff
fig|6666666.28482.peg.1715	Contig_159	11763	11404	-	FIG00822251: hypothetical protein	FIG00822249	ff
fig|6666666.28482.peg.1716	Contig_16	816	526	-	SSU ribosomal protein S6p	FIG00000170	ff
fig|6666666.28482.peg.1717	Contig_16	1585	944	-	ThiJ/PfpI family protein	FIG00015923	idu(8);CBSS-176280.1.peg.1561
fig|6666666.28482.peg.1718	Contig_16	3308	1662	-	Multimodular transpeptidase-transglycosylase (EC 2.4.1.129) (EC 3.4.-.-)	FIG00000011	icw(1);Peptidoglycan_Biosynthesis
fig|6666666.28482.peg.1719	Contig_16	5860	3305	-	Multimodular transpeptidase-transglycosylase (EC 2.4.1.129) (EC 3.4.-.-)	FIG00000011	icw(1);Peptidoglycan_Biosynthesis
fig|6666666.28482.peg.1720	Contig_16	6323	5916	-	FIG00822203: hypothetical protein	FIG00822202	ff
fig|6666666.28482.peg.1721	Contig_16	6356	7369	+	Possible membrane protein	FIG01121868	ff
fig|6666666.28482.peg.1722	Contig_16	7542	8084	+	Transcriptional regulator, PadR family	FIG00002474	idu(4);CBSS-1352.1.peg.856
fig|6666666.28482.peg.1723	Contig_16	8151	9257	+	Inositol-1-phosphate synthase (EC 5.5.1.4)	FIG00066508	isu;Di-Inositol-Phosphate_biosynthesis
fig|6666666.28482.peg.1724	Contig_16	9449	10375	+	Hydrolase	FIG00801965	ff
fig|6666666.28482.peg.1725	Contig_16	10444	11238	+	Possible oxidoreductase	FIG00553614	ff
fig|6666666.28482.peg.1726	Contig_16	11370	12110	+	Transcriptional regulator, GntR family	FIG00621925	ff

fig|6666666.28482.peg.1727	Contig_16	12160	13965	+	Amino acid ABC transporter, permease protein, 3-TM region, His/Glu/Gln/Arg/opine family protein	FIG01373972	ff
fig|6666666.28482.peg.1728	Contig_16	13962	14711	+	Amino acid ABC transporter, ATP-binding protein	FIG00519964	ff
fig|6666666.28482.peg.1729	Contig_16	14778	15275	+	Transcriptional regulator, MarR family	FIG00554610	ff
fig|6666666.28482.peg.1730	Contig_16	15317	15997	+	Short chain dehydrogenase	FIG01310822	ff
fig|6666666.28482.peg.1731	Contig_16	18887	15981	-	Leucyl-tRNA synthetase (EC 6.1.1.4)	FIG00000114	isu;tRNA_aminoacylation,_Leu
fig|6666666.28482.peg.1732	Contig_16	19073	20167	+	Proline-rich 28 kDa antigen	FIG01257277	ff
fig|6666666.28482.peg.1733	Contig_16	20236	20586	+	hypothetical protein		
fig|6666666.28482.peg.1734	Contig_16	20609	21631	+	hypothetical protein		
fig|6666666.28482.peg.1735	Contig_16	22230	21628	-	FIG00996838: hypothetical protein	FIG00821672	ff
fig|6666666.28482.peg.1736	Contig_16	22339	23646	+	Possible membrane protein	FIG01121868	ff
fig|6666666.28482.peg.1737	Contig_16	23656	24429	+	FIG00822256: hypothetical protein	FIG00822255	ff
fig|6666666.28482.peg.1738	Contig_16	25801	24542	-	FIG00821436: hypothetical protein	FIG00821434	ff
fig|6666666.28482.peg.1739	Contig_16	25884	26027	+	FIG00820102: hypothetical protein	FIG00820096	ff
fig|6666666.28482.peg.1740	Contig_16	26274	26041	-	FIG00820005: hypothetical protein	FIG00820004	ff
fig|6666666.28482.peg.1741	Contig_16	26666	26349	-	FIG00822329: hypothetical protein	FIG00822322	ff

fig|6666666.28482.peg.1742	Contig_16	28095	26719	-	FIG00821131: hypothetical protein	FIG00821130	ff
fig|6666666.28482.peg.1743	Contig_16	28537	28199	-	hypothetical protein		
fig|6666666.28482.peg.1744	Contig_16	29365	28580	-	NlpC/P60 family, cell wall-associated hydrolase, invasion-associated protein	FIG01316122	ff
fig|6666666.28482.peg.1745	Contig_16	30126	29362	-	Transcriptional regulatory protein	FIG00447785	ff
fig|6666666.28482.peg.1746	Contig_16	30273	30689	+	WhiB family transcriptional regulator	FIG01310014	ff
fig|6666666.28482.peg.1747	Contig_16	30761	32143	+	Acyl-CoA dehydrogenase	FIG01471012	ff
fig|6666666.28482.peg.1748	Contig_16	32172	33143	+	FIG00821582: hypothetical protein	FIG00821581	ff
fig|6666666.28482.peg.1749	Contig_16	33140	33457	+	FIG00825846: hypothetical protein	FIG00825845	ff
fig|6666666.28482.peg.1750	Contig_16	33693	33454	-	FIG00314463: hypothetical protein	FIG01845904	ff
fig|6666666.28482.peg.1751	Contig_16	33763	34116	+	FIG00828522: hypothetical protein	FIG00828517	ff
fig|6666666.28482.peg.1752	Contig_16	34139	34627	+	glutathione peroxidase family protein(EC:1.11.1.12,EC:1.11.1.9)		
fig|6666666.28482.rna.26	Contig_16	34781	34699	-	tRNA-Leu-CAG		isu;tRNAs
fig|6666666.28482.peg.1753	Contig_160	1820	708	-	Trehalose-6-phosphate phosphatase (EC 3.1.3.12)	FIG00001349	idu(1);Trehalose_Biosynthesis

fig|6666666.28482.peg.1754	Contig_160	3178	1862	-	Wax ester synthase/acyl-CoA:diacylglycerol acyltransferase	FIG01309728	ff
fig|6666666.28482.peg.1755	Contig_160	3383	6628	+	DNA polymerase III alpha subunit (EC 2.7.7.7)	FIG00041038	idu(1);DNA-replication idu(1);CBSS-350688.3.peg.1509
fig|6666666.28482.peg.1756	Contig_160	6775	7113	+	conserved hypothetical secreted protein		
fig|6666666.28482.peg.1757	Contig_160	7533	7123	-	FIG00820233: hypothetical protein	FIG00820230	ff
fig|6666666.28482.peg.1758	Contig_161	3294	1954	-	Glutamine synthetase type I (EC 6.3.1.2)	FIG00134889	idu(2);Glutamine,_Glutamate,_Aspartate_and_Asparagine_Biosynthesis idu(2);Ammonia_assimilation idu(2);Glutamine_synthetases idu(2);Peptidoglycan_Biosynthesis
fig|6666666.28482.peg.1759	Contig_162	275	1486	+	Mobile element protein	FIG01306568	ff
fig|6666666.28482.peg.1760	Contig_163	575	90	-	FIG00821384: hypothetical protein	FIG00821383	ff
fig|6666666.28482.peg.1761	Contig_164	26	958	+	Mobile element protein	FIG01306568	ff
fig|6666666.28482.peg.1762	Contig_165	194	1333	+	Mobile element protein	FIG01306568	ff
fig|6666666.28482.peg.1763	Contig_166	246	1328	+	Mobile element protein	FIG01306568	ff
fig|6666666.28482.peg.1764	Contig_167	1361	1047	-	Proline and threonine rich protein	FIG00825745	ff

fig|6666666.28482.peg.1765	Contig_169	1278	709	-	Phosphoheptose isomerase (EC 5.3.1.-)	FIG00000620	isu;LOS_core_oligosaccharide_biosynthesis isu;Capsular_heptose_biosynthesis
fig|6666666.28482.peg.1766	Contig_169	2270	1275	-	hypothetical protein		
fig|6666666.28482.peg.1767	Contig_169	2794	2279	-	D-glycero-D-manno-heptose 1,7-bisphosphate phosphatase (EC 3.1.1.-); possible Histidinol-phosphatase (EC 3.1.3.15)	FIG01407672	icw(1);LOS_core_oligosaccharide_biosynthesis icw(1);Capsular_heptose_biosynthesis
fig|6666666.28482.peg.1768	Contig_17	48	7805	+	FIG00820660: hypothetical protein	FIG00820658	ff
fig|6666666.28482.peg.1769	Contig_17	7890	9545	+	3-ketoacyl-CoA thiolase	FIG00825273	ff
fig|6666666.28482.peg.1770	Contig_17	9542	10429	+	FIG00824394: hypothetical protein	FIG00824393	ff
fig|6666666.28482.peg.1771	Contig_17	10429	11244	+	FIG00446426: hypothetical protein	FIG00446425	ff
fig|6666666.28482.peg.1772	Contig_17	11283	12041	+	2-hydroxy-3-oxopropionate reductase (EC 1.1.1.60)	FIG00133229	idu(1);Glycerate_metabolism idu(1);Allantoin_Utilization idu(1);Photorespiration_(oxidative_C2_cycle) idu(1);D-galactarate,_D-glucarate_and_D-glycerate_catabolism idu(1);D-galactarate,_D-glucarate_and_D-glycerate_catabolism_-_gjo
fig|6666666.28482.peg.1773	Contig_17	12147	13352	+	putative cytochrome P450 hydroxylase	FIG00032437	idu(49);Nitric_oxide_synthase
fig|6666666.28482.peg.1774	Contig_17	13364	13564	+	Probable Ferredoxin FdxD	FIG01446880	ff

fig|6666666.28482.peg.1775	Contig_17	13857	13561	-	FIG00820944: hypothetical protein	FIG00820941	ff
fig|6666666.28482.peg.1776	Contig_17	14056	13850	-	fdxD-related protein	FIG01419919	ff
fig|6666666.28482.peg.1777	Contig_17	15333	14053	-	cytochrome p450 107b1	FIG00825630	ff
fig|6666666.28482.peg.1778	Contig_17	15975	15448	-	FIG00824107: hypothetical protein	FIG00824106	ff
fig|6666666.28482.peg.1779	Contig_17	16586	15972	-	Transcriptional regulator, TetR family	FIG00554417	ff
fig|6666666.28482.peg.1780	Contig_17	17836	16691	-	3-ketoacyl-CoA thiolase (EC 2.3.1.16) @ Acetyl-CoA acetyltransferase (EC 2.3.1.9)	FIG00000004	idu(11);CBSS-246196.1.peg.364 idu(9);Serine-glyoxylate_cycle idu(11);Serine-glyoxylate_cycle icw(1);n-Phenylalkanoic_acid_degradation idu(9);Ethylmalonyl-CoA_pathway_of_C2_assimilation,_GJO idu(9);Polyhydroxybutyrate_metabolism icw(1);Polyhydroxybutyrate_metabolism icw(2);Isoleucine_degradation icw(1);Fatty_acid_metabolism_cluster idu(11);Biotin_synthesis_cluster idu(11);Biotin_biosynthesis icw(2);Lysine_fermentation idu(9);Isoprenoid_Biosynthesis icw(2);Butanol_Biosynthesis icw(2);Acetyl-CoA_fermentation_to_Butyrate icw(1);Butyrate_metabolism_cluster

fig|6666666.28482.peg.1781	Contig_17	18644	17829	-	Enoyl-CoA hydratase (EC 4.2.1.17)	FIG00033206	idu(51);n-Phenylalkanoic_acid_degradation icw(1);Polyhydroxybutyrate_metabolism icw(1);Isoleucine_degradation idu(51);Fatty_acid_metabolism_cluster icw(1);Lysine_fermentation icw(1);Butanol_Biosynthesis icw(1);Acetyl-CoA_fermentation_to_Butyrate icw(1);Valine_degradation idu(51);Butyrate_metabolism_cluster
fig|6666666.28482.peg.1782	Contig_17	19822	18641	-	Butyryl-CoA dehydrogenase (EC 1.3.99.2)	FIG00009149	idu(39);Isoleucine_degradation idu(39);Lysine_fermentation idu(39);Butanol_Biosynthesis idu(39);Acetyl-CoA_fermentation_to_Butyrate idu(39);Valine_degradation idu(39);Anaerobic_respiratory_reductases
fig|6666666.28482.peg.1783	Contig_17	21220	19922	-	FIG00821608: hypothetical protein	FIG00821607	ff
fig|6666666.28482.peg.1784	Contig_17	21502	22035	+	FIG00823110: hypothetical protein	FIG00823105	ff
fig|6666666.28482.peg.1785	Contig_17	22040	23317	+	Phenylpropionate dioxygenase and related ring-hydroxylating dioxygenases, large terminal subunit	FIG01371907	ff

fig|6666666.28482.peg.1786	Contig_17	23301	23624	+	FIG00820992: hypothetical protein	FIG00820991	ff
fig|6666666.28482.peg.1787	Contig_17	23628	24842	+	Prolidase (EC 3.4.13.9)	FIG01313749	ff
fig|6666666.28482.peg.1788	Contig_17	24826	25248	+	FIG00822989: hypothetical protein	FIG00822988	ff
fig|6666666.28482.peg.1789	Contig_17	25245	26342	+	Acyl-CoA dehydrogenase (EC 1.3.99.-)	FIG00820935	ff
fig|6666666.28482.peg.1790	Contig_17	26346	27140	+	Butyryl-CoA dehydrogenase (EC 1.3.99.2)	FIG00009149	idu(39);Isoleucine_degradation idu(39);Lysine_fermentation idu(39);Butanol_Biosynthesis idu(39);Acetyl-CoA_fermentation_to_Butyrate idu(39);Valine_degradation idu(39);Anaerobic_respiratory_reductases
fig|6666666.28482.peg.1791	Contig_17	27245	27457	+	FIG00827257: hypothetical protein	FIG00827256	ff
fig|6666666.28482.peg.1792	Contig_17	28629	27454	-	Acyl-CoA dehydrogenase (EC 1.3.99.-)	FIG01333225	ff
fig|6666666.28482.peg.1793	Contig_17	28849	29499	+	Transcriptional regulator, TetR family	FIG00554417	ff
fig|6666666.28482.peg.1794	Contig_17	29672	30523	+	FIG00820419: hypothetical protein	FIG01323985	ff
fig|6666666.28482.peg.1795	Contig_17	30562	31368	+	short chain dehydrogenase		
fig|6666666.28482.peg.1796	Contig_17	34215	31831	-	CAIB-BAIF family family	FIG01503811	ff
fig|6666666.28482.peg.1797	Contig_17	35375	34257	-	Xaa-Pro dipeptidase (EC 3.4.13.9)		
fig|6666666.28482.peg.1798	Contig_17	36606	35365	-	FIG00826794: hypothetical protein	FIG00826792	ff

fig|6666666.28482.peg.1799	Contig_17	37789	36617	-	BarH	FIG00668014	ff
fig|6666666.28482.peg.1800	Contig_17	39092	37839	-	putative cytochrome P450 hydroxylase	FIG00032437	idu(49);Nitric_oxide_synthase
fig|6666666.28482.peg.1801	Contig_17	39288	39094	-	Ferredoxin	FIG00001676	idu(4);Soluble_cytochromes_and_functionally_related_electron_carriers idu(4);Inorganic_Sulfur_Assimilation
fig|6666666.28482.peg.1802	Contig_17	39979	39299	-	Transcriptional regulator, TetR family	FIG00554417	ff
fig|6666666.28482.peg.1803	Contig_17	40854	40045	-	Short chain dehydrogenase	FIG01310822	ff
fig|6666666.28482.peg.1804	Contig_17	40995	41762	+	putative secreted protein	FIG00905474	ff
fig|6666666.28482.peg.1805	Contig_17	41786	42529	+	3-oxoacyl-[acyl-carrier protein] reductase (EC 1.1.1.100)	FIG00621114	idu(38);CBSS-246196.1.peg.364 idu(38);CBSS-164757.7.peg.5020 idu(38);Fatty_Acid_Biosynthesis_FASII
fig|6666666.28482.peg.1806	Contig_17	42537	43403	+	hypothetical protein	FIG00638284	ff
fig|6666666.28482.peg.1807	Contig_17	43486	45057	+	Multicopper oxidase	FIG00060246	idu(2);Copper_homeostasis
fig|6666666.28482.peg.1808	Contig_17	45882	45061	-	2-hydroxymuconic semialdehyde hydrolase (EC 3.7.1.9)	FIG00042074	idu(1);Central_meta-cleavage_pathway_of_aromatic_compound_degradation
fig|6666666.28482.peg.1809	Contig_17	46888	45896	-	FIG00823018: hypothetical protein	FIG00823016	ff
fig|6666666.28482.peg.1810	Contig_17	47703	46876	-	3-alpha-hydroxysteroid dehydrogenase	FIG01322334	ff

fig|6666666.28482.peg.1811	Contig_17	49028	47724	-	F420-dependent N(5),N(10)-methylenetetrahydromethanopterin reductase (EC 1.5.99.11)	FIG00820831	isu;Methanogenesis
fig|6666666.28482.peg.1812	Contig_17	50784	49048	-	3-oxosteroid 1-dehydrogenase (EC 1.3.99.4)	FIG01318047	ff
fig|6666666.28482.peg.1813	Contig_17	51608	50781	-	2,3-dihydroxyphenylpropionate 1,2-dioxygenase (EC 1.13.11.-)	FIG01955093	ff
fig|6666666.28482.peg.1814	Contig_17	53346	51646	-	3-(3-hydroxy-phenyl)propionate hydroxylase (EC 1.14.13.-)	FIG00138127	idu(3);Cinnamic_Acid_Degradation
fig|6666666.28482.peg.1815	Contig_17	54218	53355	-	2-hydroxy-6-oxo-6-phenylhexa-2,4-dienoate hydrolase (EC 3.7.1.-)	FIG00003780	idu(2);carbazol_degradation_cluster idu(2);Central_meta-cleavage_pathway_of_aromatic_compound_degradation idu(2);Biphenyl_Degradation
fig|6666666.28482.peg.1816	Contig_17	54347	55012	+	Transcriptional regulator, IclR family	FIG00022569	idu(12);Homogentisate_pathway_of_aromatic_compound_degradation
fig|6666666.28482.peg.1817	Contig_17	55039	56670	+	3-oxosteroid 1-dehydrogenase (EC 1.3.99.4)	FIG01318047	ff
fig|6666666.28482.peg.1818	Contig_17	57896	56679	-	POSSIBLE TWO COMPONENT SENSOR KINASE	FIG01324569	ff

fig|6666666.28482.peg.1819	Contig_17	57965	58618	+	DNA-binding response regulator, LuxR family	FIG01307606	ff
fig|6666666.28482.peg.1820	Contig_17	58621	59577	+	Microsomal dipeptidase (EC 3.4.13.19)	FIG00030415	isu;Pyrroloquinoline_Quinone_biosynthesis
fig|6666666.28482.peg.1821	Contig_17	59968	59594	-	Limonene-1,2-epoxide hydrolase		
fig|6666666.28482.peg.1822	Contig_17	60903	60016	-	putative periplasmic protein kinase ArgK and related GTPases of G3E family	FIG00007966	idu(1);G3E_family_of_P-loop_GTPases_(metallocenter_biosynthesis)

fig|6666666.28482.peg.1823	Contig_17	62092	60911	-	3-ketoacyl-CoA thiolase (EC 2.3.1.16) @ Acetyl-CoA acetyltransferase (EC 2.3.1.9)	FIG00000004	idu(11);CBSS-246196.1.peg.364 idu(9);Serine-glyoxylate_cycle idu(11);Serine-glyoxylate_cycle icw(1);n-Phenylalkanoic_acid_degradation idu(9);Ethylmalonyl-CoA_pathway_of_C2_assimilation,_GJO idu(9);Polyhydroxybutyrate_metabolism icw(1);Polyhydroxybutyrate_metabolism icw(2);Isoleucine_degradation icw(1);Fatty_acid_metabolism_cluster idu(11);Biotin_synthesis_cluster idu(11);Biotin_biosynthesis icw(2);Lysine_fermentation idu(9);Isoprenoid_Biosynthesis icw(2);Butanol_Biosynthesis icw(2);Acetyl-CoA_fermentation_to_Butyrate icw(1);Butyrate_metabolism_cluster
fig|6666666.28482.peg.1824	Contig_17	62673	62155	-	Phenolic acid decarboxylase (EC 4.1.1.-)	FIG00023936	isu;CBSS-1352.1.peg.856
fig|6666666.28482.peg.1825	Contig_17	62803	63036	+	hypothetical protein		
fig|6666666.28482.peg.1826	Contig_17	63122	64012	+	FIG00824413: hypothetical protein	FIG00824412	ff

fig|6666666.28482.peg.1827	Contig_17	64839	64072	-	Enoyl-CoA hydratase (EC 4.2.1.17)	FIG00033206	idu(51);n-Phenylalkanoic_acid_degradation icw(1);Polyhydroxybutyrate_metabolism icw(1);Isoleucine_degradation idu(51);Fatty_acid_metabolism_cluster icw(1);Lysine_fermentation icw(1);Butanol_Biosynthesis icw(1);Acetyl-CoA_fermentation_to_Butyrate icw(1);Valine_degradation idu(51);Butyrate_metabolism_cluster
fig|6666666.28482.peg.1828	Contig_17	65937	64840	-	Butyryl-CoA dehydrogenase (EC 1.3.99.2)	FIG00009149	idu(39);Isoleucine_degradation idu(39);Lysine_fermentation idu(39);Butanol_Biosynthesis idu(39);Acetyl-CoA_fermentation_to_Butyrate idu(39);Valine_degradation idu(39);Anaerobic_respiratory_reductases
fig|6666666.28482.peg.1829	Contig_17	66829	65924	-	FIG00823074: hypothetical protein	FIG00823067	ff
fig|6666666.28482.peg.1830	Contig_17	68210	66807	-	4-coumarate--CoA ligase 1 (EC 6.2.1.12)	FIG00820129	ff
fig|6666666.28482.peg.1831	Contig_17	68311	68655	+	Probable phenylacetic acid degradation NADH oxidoreductase paaE (EC 1.-.-.-)	FIG01355358	ff
fig|6666666.28482.peg.1832	Contig_17	68675	68989	+	FIG00829186: hypothetical protein	FIG00829181	ff

fig|6666666.28482.peg.1833	Contig_17	69003	70265	+	FIG00823137: hypothetical protein	FIG00823136	ff
fig|6666666.28482.peg.1834	Contig_17	71277	70354	-	conserved hypothetical protein SCE20.23		
fig|6666666.28482.peg.1835	Contig_17	72167	71274	-	NAD-dependent epimerase/dehydratase	FIG00988521	ff
fig|6666666.28482.peg.1836	Contig_17	72332	73246	+	Transcriptional regulators, LysR family		
fig|6666666.28482.peg.1837	Contig_17	74269	73232	-	L-carnitine dehydratase/bile acid-inducible protein F (EC 2.8.3.16)	FIG00450481	ff
fig|6666666.28482.peg.1838	Contig_17	75222	74266	-	putative oxidoreductase	FIG00945653	ff
fig|6666666.28482.peg.1839	Contig_17	75635	75207	-	B12 binding domain of Methylmalonyl-CoA mutase (EC 5.4.99.2)	FIG00004321	isu;G3E_family_of_P-loop_GTPases_(metallocenter_biosynthesis)
fig|6666666.28482.peg.1840	Contig_17	77213	75636	-	Methylmalonyl-CoA mutase (EC 5.4.99.2)	FIG00000927	idu(1);Serine-glyoxylate_cycle icw(1);G3E_family_of_P-loop_GTPases_(metallocenter_biosynthesis)
fig|6666666.28482.peg.1841	Contig_17	78103	77291	-	Methyltransferase (EC 2.1.1.-)	FIG00515945	ff
fig|6666666.28482.peg.1842	Contig_17	78870	78163	-	D-alanyl-D-alanine dipeptidase	FIG01369240	ff
fig|6666666.28482.peg.1843	Contig_17	79491	78886	-	Transcriptional regulator, TetR family	FIG00554417	ff
fig|6666666.28482.peg.1844	Contig_17	79554	81266	+	FIG00828524: hypothetical protein	FIG00828522	ff
fig|6666666.28482.peg.1845	Contig_17	81352	82494	+	luciferase family protein		

fig|6666666.28482.peg.1846	Contig_17	82507	82959	+	hypothetical protein	FIG00638284	ff
fig|6666666.28482.peg.1847	Contig_17	83013	84665	+	PPE family protein	FIG00013075	idu(57);CBSS-164757.7.peg.5020
fig|6666666.28482.peg.1848	Contig_17	84910	86412	+	hypothetical protein		
fig|6666666.28482.peg.1849	Contig_17	86693	86574	-	hypothetical protein		
fig|6666666.28482.peg.1850	Contig_17	87422	86841	-	Transcriptional regulator, TetR family	FIG00554417	ff
fig|6666666.28482.peg.1851	Contig_17	87506	88699	+	putative cytochrome P450 hydroxylase	FIG00032437	idu(49);Nitric_oxide_synthase
fig|6666666.28482.peg.1852	Contig_17	89393	88758	-	Transcriptional regulator, TetR family	FIG00554417	ff
fig|6666666.28482.peg.1853	Contig_17	90190	93276	+	hypothetical protein	FIG00638284	ff
fig|6666666.28482.peg.1854	Contig_17	93321	94169	+	Hydrolase	FIG00801965	ff
fig|6666666.28482.peg.1855	Contig_17	94677	94198	-	FIG00825226: hypothetical protein	FIG00825223	ff
fig|6666666.28482.peg.1856	Contig_17	94909	96477	+	hypothetical protein		
fig|6666666.28482.peg.1857	Contig_17	96659	96793	+	hypothetical protein		
fig|6666666.28482.peg.1858	Contig_17	97011	97790	+	Transcriptional regulator KorSA, GntR family	FIG01122925	ff
fig|6666666.28482.peg.1859	Contig_17	97890	98312	+	FIG00831412: hypothetical protein	FIG00831411	ff
fig|6666666.28482.peg.1860	Contig_17	98309	99655	+	Transfer protein traSA		
fig|6666666.28482.peg.1861	Contig_17	99900	101057	+	Integrase	FIG01116662	ff
fig|6666666.28482.rna.27	Contig_17	101166	101093	-	tRNA-Phe-GAA		isu;tRNAs
fig|6666666.28482.rna.28	Contig_17	101269	101196	-	tRNA-Asp-GTC		
fig|6666666.28482.rna.29	Contig_17	101371	101299	-	tRNA-Glu-TTC		
fig|6666666.28482.rna.30	Contig_17	101507	101579	+	tRNA-Lys-TTT		
fig|6666666.28482.peg.1862	Contig_17	101708	102880	+	Mobile element protein	FIG01306568	ff
fig|6666666.28482.peg.1863	Contig_17	103064	103873	+	FIG00821728: hypothetical protein	FIG00821727	ff

fig|6666666.28482.peg.1864	Contig_17	104762	103863	-	O-Methyltransferase involved in polyketide biosynthesis	FIG01313020	ff
fig|6666666.28482.peg.1865	Contig_17	104856	105356	+	Deaminase	FIG01956300	ff
fig|6666666.28482.peg.1866	Contig_17	105402	105791	+	transcriptional regulator, ArsR family	FIG01319550	ff
fig|6666666.28482.peg.1867	Contig_17	105788	105991	+	zinc transporter Slc39a7	FIG01491207	ff
fig|6666666.28482.peg.1868	Contig_17	107011	105998	-	FIG00820092: hypothetical protein	FIG00820090	ff
fig|6666666.28482.peg.1869	Contig_17	107079	107693	+	Transcriptional regulator, TetR family	FIG00554417	ff
fig|6666666.28482.peg.1870	Contig_17	107826	108842	+	Fatty acid desaturase occurring in virulence cluster	FIG00996037	icw(1);Mycobacterium_virulence_operon_involved_in_lipid_metabolism
fig|6666666.28482.peg.1871	Contig_17	108859	109992	+	tRNA dihydrouridine synthase B (EC 1.-.-.-)	FIG00000580	isu;Mycobacterium_virulence_operon_involved_in_lipid_metabolism
fig|6666666.28482.peg.1872	Contig_17	110197	112287	+	Cell envelope-associated transcriptional attenuator LytR-CpsA-Psr, subfamily A1 (as in PMID19099556)	FIG00025213	idu(3);Cell_envelope-associated_LytR-CpsA-Psr_transcriptional_attenuators
fig|6666666.28482.peg.1873	Contig_17	112424	113014	+	Phosphate transport system regulatory protein PhoU	FIG00104848	icw(3);High_affinity_phosphate_transporter_and_control_of_PHO_regulon icw(2);Phosphate_metabolism
fig|6666666.28482.peg.1874	Contig_17	113787	113011	-	Phosphate transport ATP-binding protein PstB (TC 3.A.1.7.1)	FIG00011114	icw(2);High_affinity_phosphate_transporter_and_control_of_PHO_regulon icw(4);Phosphate_metabolism

fig|6666666.28482.peg.1875	Contig_17	114714	113800	-	Phosphate transport system permease protein PstA (TC 3.A.1.7.1)	FIG00083946	icw(3);High_affinity_phosphate_transporter_and_control_of_PHO_regulon icw(1);Phosphate_metabolism
fig|6666666.28482.peg.1876	Contig_17	115676	114711	-	Phosphate transport system permease protein PstC (TC 3.A.1.7.1)	FIG00018561	icw(1);High_affinity_phosphate_transporter_and_control_of_PHO_regulon idu(1);Phosphate_metabolism
fig|6666666.28482.peg.1877	Contig_17	116761	115802	-	Phosphate ABC transporter, periplasmic phosphate-binding protein PstS (TC 3.A.1.7.1)	FIG00010508	icw(5);High_affinity_phosphate_transporter_and_control_of_PHO_regulon icw(5);Phosphate_metabolism icw(1);PhoR-PhoB_two-component_regulatory_system
fig|6666666.28482.peg.1878	Contig_17	116808	116942	+	hypothetical protein		
fig|6666666.28482.peg.1879	Contig_17	117957	117085	-	Acetyl-CoA:Cys-GlcN-Ins acetyltransferase, mycothiol synthase MshD	FIG00011306	isu;Glutathione_analogs:_mycothiol
fig|6666666.28482.peg.1880	Contig_17	118781	118005	-	Phosphate regulon transcriptional regulatory protein PhoB (SphR)	FIG01290653	idu(1);High_affinity_phosphate_transporter_and_control_of_PHO_regulon icw(2);Phosphate_metabolism idu(1);PhoR-PhoB_two-component_regulatory_system
fig|6666666.28482.peg.1881	Contig_17	118959	119771	+	FIG00821015: hypothetical protein	FIG00821014	ff
fig|6666666.28482.peg.1882	Contig_17	119848	120276	+	Thioredoxin	FIG01318274	isu;CBSS-315749.4.peg.3658 isu;Glycine_reductase,_sarcosine_reductase_and_betaine_reductase
fig|6666666.28482.peg.1883	Contig_17	120501	120965	+	Possible membrane protein	FIG00822196	ff

fig|6666666.28482.peg.1884	Contig_17	121002	121835	+	Thiosulfate sulfurtransferase, rhodanese (EC 2.8.1.1)	FIG00004487	ff
fig|6666666.28482.peg.1885	Contig_17	121867	122139	+	probable sseC protein		
fig|6666666.28482.peg.1886	Contig_17	122336	123004	+	DUF1794	FIG00020072	idu(1);Iron-sulfur_cluster_assembly
fig|6666666.28482.peg.1887	Contig_17	124120	123254	-	Aminodeoxychorismate lyase (EC 4.1.3.38)	FIG00000708	isu;Chorismate:_Intermediate_for_synthesis_of_Tryptophan,_PAPA_antibiotics,_PABA,_3-hydroxyanthranilate_and_more. isu;Folate_Biosynthesis isu;Tryptophan_synthesis
fig|6666666.28482.peg.1888	Contig_17	124180	125274	+	Folate-dependent protein for Fe/S cluster synthesis/repair in oxidative stress	FIG00043717	icw(1);Iron-sulfur_cluster_assembly
fig|6666666.28482.peg.1889	Contig_17	125422	125598	+	FIG00821405: hypothetical protein	FIG00821404	ff
fig|6666666.28482.peg.1890	Contig_17	126742	125648	-	Phosphoribosylformylglycinamidine cyclo-ligase (EC 6.3.3.1)	FIG00000222	icw(1);De_Novo_Purine_Biosynthesis
fig|6666666.28482.peg.1891	Contig_17	128338	126803	-	Amidophosphoribosyltransferase (EC 2.4.2.14)	FIG00000179	isu;De_Novo_Purine_Biosynthesis
fig|6666666.28482.peg.1892	Contig_17	128865	128476	-	hypothetical protein		
fig|6666666.28482.peg.1893	Contig_17	129450	128869	-	3',5'-cyclic-nucleotide phosphodiesterase (EC 3.1.4.17)	FIG00001540	isu;cAMP_signaling_in_bacteria isu;CBSS-342610.3.peg.1794
fig|6666666.28482.peg.1894	Contig_17	131756	129471	-	Phosphoribosylformylglycinamidine synthase, synthetase subunit (EC 6.3.5.3)	FIG01303876	icw(2);De_Novo_Purine_Biosynthesis

fig|6666666.28482.peg.1895	Contig_17	132562	131774	-	helix-turn-helix domain protein		
fig|6666666.28482.peg.1896	Contig_17	132690	133085	+	hypothetical protein		
fig|6666666.28482.peg.1897	Contig_17	133668	133339	-	glyoxalase family protein superfamily	FIG01000012	ff
fig|6666666.28482.peg.1898	Contig_17	134969	133701	-	Aspartyl aminopeptidase	FIG00015518	ff
fig|6666666.28482.peg.1899	Contig_17	135069	136028	+	Predicted dye-decolorizing peroxidase (DyP), encapsulated subgroup	FIG00014133	isu;Encapsulating_protein_for_DyP-type_peroxidase_and_ferritin-like_protein_oligomers
fig|6666666.28482.peg.1900	Contig_17	136025	136822	+	Encapsulating protein for a DyP-type peroxidase or ferritin-like protein oligomers	FIG00014104	icw(1);Encapsulating_protein_for_DyP-type_peroxidase_and_ferritin-like_protein_oligomers
fig|6666666.28482.peg.1901	Contig_17	137038	137817	+	hypothetical protein		
fig|6666666.28482.peg.1902	Contig_17	139611	137911	-	Flavohemoprotein (Hemoglobin-like protein) (Flavohemoglobin) (Nitric oxide dioxygenase) (EC 1.14.12.17)	FIG00000983	isu;Glutaredoxins isu;Flavohaemoglobin isu;Bacterial_hemoglobins
fig|6666666.28482.peg.1903	Contig_17	139940	139800	-	hypothetical protein		
fig|6666666.28482.peg.1904	Contig_17	139971	140633	+	putative hydrolase	FIG00662601	ff
fig|6666666.28482.peg.1905	Contig_17	140709	141401	+	Transcriptional regulator, TetR family	FIG00554417	ff
fig|6666666.28482.peg.1906	Contig_17	141398	142048	+	Transcriptional regulator, TetR family	FIG00554417	ff
fig|6666666.28482.peg.1907	Contig_17	142268	143242	+	Quinone oxidoreductase (EC 1.6.5.5)	FIG00002982	idu(7);Quinone_oxidoreductase_family

fig|6666666.28482.peg.1908	Contig_17	143304	143975	+	haloacid dehalogenase, type II(EC:3.8.1.2)		
fig|6666666.28482.peg.1909	Contig_17	143979	145433	+	PF00070 family, FAD-dependent NAD(P)-disulphide oxidoreductase	FIG00001597	idu(1);Mercuric_reductase
fig|6666666.28482.peg.1910	Contig_17	145437	145904	+	FIG00820938: hypothetical protein	FIG00820937	ff
fig|6666666.28482.peg.1911	Contig_17	146296	145898	-	biphenyl-2,3-diol 1,2-dioxygenase III-related protein	FIG00105129	isu;Biphenyl_Degradation
fig|6666666.28482.peg.1912	Contig_17	146886	146299	-	Transcriptional regulator, TetR family	FIG00628138	ff
fig|6666666.28482.peg.1913	Contig_17	146944	147345	+	hypothetical protein		
fig|6666666.28482.peg.1914	Contig_17	147437	148645	+	putative cytochrome P450 hydroxylase	FIG00032437	idu(49);Nitric_oxide_synthase
fig|6666666.28482.peg.1915	Contig_17	148743	149222	+	FIG00820357: hypothetical protein	FIG00820356	ff
fig|6666666.28482.peg.1916	Contig_17	150319	149240	-	UDP-glucose 4-epimerase	FIG00763453	ff
fig|6666666.28482.peg.1917	Contig_17	150441	151277	+	FIG00824813: hypothetical protein	FIG00824812	ff
fig|6666666.28482.peg.1918	Contig_17	151299	152684	+	Diacyglycerol O-acyltransferase (EC 2.3.1.20)	FIG01460290	ff
fig|6666666.28482.peg.1919	Contig_17	153355	152681	-	Phosphoribosylformylglycinamidine synthase, glutamine amidotransferase subunit (EC 6.3.5.3)	FIG00025475	icw(1);De_Novo_Purine_Biosynthesis

fig|6666666.28482.peg.1920	Contig_17	153591	153352	-	Phosphoribosylformylglycinamidine synthase, PurS subunit (EC 6.3.5.3)	FIG00000869	isu;De_Novo_Purine_Biosynthesis
fig|6666666.28482.peg.1921	Contig_17	154330	153656	-	FIG00820729: hypothetical protein	FIG00820728	ff
fig|6666666.28482.peg.1922	Contig_17	154364	155002	+	FIG00998332: hypothetical protein	FIG00820472	ff
fig|6666666.28482.peg.1923	Contig_17	156743	155076	-	Fumarate/succinate/L-aspartate dehydrogenases	FIG01355113	ff
fig|6666666.28482.peg.1924	Contig_17	157496	156792	-	FIG00998196: hypothetical protein	FIG00820963	ff
fig|6666666.28482.peg.1925	Contig_17	157695	159302	+	Inner membrane component of tripartite multidrug resistance system	FIG00005204	ff
fig|6666666.28482.peg.1926	Contig_17	159862	159299	-	Transcriptional regulator, TetR family	FIG00554417	ff
fig|6666666.28482.peg.1927	Contig_17	162491	160329	-	Protease II (EC 3.4.21.83)	FIG00614142	ff
fig|6666666.28482.peg.1928	Contig_17	163381	162488	-	Phosphoribosylaminoimidazole-succinocarboxamide synthase (EC 6.3.2.6)	FIG00000195	isu;De_Novo_Purine_Biosynthesis
fig|6666666.28482.peg.1929	Contig_17	163433	164062	+	POSSIBLE CONSERVED TRANSMEMBRANE PROTEIN	FIG01417063	ff
fig|6666666.28482.peg.1930	Contig_17	164967	164068	-	putative esterase		idu(2);Alpha-Amylase_locus_in_Streptocococcus

fig|6666666.28482.peg.1931	Contig_17	166072	165080	-	putative C4-dicarboxylate transporter	FIG01387533	ff
fig|6666666.28482.peg.1932	Contig_17	167316	166069	-	Cytochrome P450 126A3	FIG01369642	ff
fig|6666666.28482.peg.1933	Contig_17	168731	167313	-	Adenylosuccinate lyase (EC 4.3.2.2)	FIG00000117	isu;Purine_conversions isu;De_Novo_Purine_Biosynthesis
fig|6666666.28482.peg.1934	Contig_17	168764	169498	+	FIG00820278: hypothetical protein	FIG00820277	ff
fig|6666666.28482.peg.1935	Contig_17	170072	169503	-	Transcriptional regulator, TetR family	FIG00554417	ff
fig|6666666.28482.peg.1936	Contig_17	170182	171081	+	Gamma-glutamyltranspeptidase (EC 2.3.2.2)	FIG00005992	icw(1);Glutathione:_Biosynthesis_and_gamma-glutamyl_cycle icw(1);Utilization_of_glutathione_as_a_sulphur_source icw(1);Poly-gamma-glutamate_biosynthesis
fig|6666666.28482.peg.1937	Contig_17	171225	172808	+	Gamma-glutamyltranspeptidase (EC 2.3.2.2)	FIG00005992	icw(1);Glutathione:_Biosynthesis_and_gamma-glutamyl_cycle icw(1);Utilization_of_glutathione_as_a_sulphur_source icw(1);Poly-gamma-glutamate_biosynthesis
fig|6666666.28482.peg.1938	Contig_17	174073	172805	-	Phosphoribosylamine--glycine ligase (EC 6.3.4.13)	FIG00000235	isu;De_Novo_Purine_Biosynthesis
fig|6666666.28482.peg.1939	Contig_17	174505	174098	-	Transcriptional regulator, MarR family		
fig|6666666.28482.peg.1940	Contig_17	174587	175684	+	aldo/keto reductase	FIG01122489	ff

fig|6666666.28482.peg.1941	Contig_170	548	306	-	PROBABLE CONSERVED TRANSMEMBRANE PROTEIN	FIG00662132	ff
fig|6666666.28482.peg.1942	Contig_175	34	201	+	Cyclohexanone monooxygenase (EC 1.14.13.22)	FIG00975774	ff
fig|6666666.28482.peg.1943	Contig_175	685	206	-	cholesterol oxidase(EC:1.1.3.6)		
fig|6666666.28482.peg.1944	Contig_177	1463	810	-	L-seryl-tRNA(Sec) selenium transferase (EC 2.9.1.1)	FIG00009265	isu;Selenocysteine_metabolism
fig|6666666.28482.peg.1945	Contig_18	2130	688	-	hypothetical protein	FIG00638284	ff
fig|6666666.28482.peg.1946	Contig_18	3092	2253	-	short-chain dehydrogenase/reductase SDR		
fig|6666666.28482.peg.1947	Contig_18	3709	3089	-	dTDP-4-dehydrorhamnose 3,5-epimerase (EC 5.1.3.13)	FIG00000408	icw(1);dTDP-rhamnose_synthesis icw(1);Rhamnose_containing_glycans idu(1);Capsular_heptose_biosynthesis
fig|6666666.28482.peg.1948	Contig_18	4712	3711	-	dTDP-glucose 4,6-dehydratase (EC 4.2.1.46)	FIG00030688	idu(4);dTDP-rhamnose_synthesis idu(4);Rhamnose_containing_glycans

fig|6666666.28482.peg.1949	Contig_18	5601	4735	-	Coenzyme F420-dependent N5,N10-methylene tetrahydromethanopterin reductase and related flavin-dependent oxidoreductases	FIG00465245	ff
fig|6666666.28482.peg.1950	Contig_18	6452	5622	-	Coenzyme F420-dependent N5,N10-methylene tetrahydromethanopterin reductase and related flavin-dependent oxidoreductases	FIG00465245	ff
fig|6666666.28482.peg.1951	Contig_18	7975	6482	-	sensor histidine kinase	FIG01107919	ff
fig|6666666.28482.peg.1952	Contig_18	9660	7984	-	Thioredoxin reductase (EC 1.8.1.9)	FIG00018396	idu(3);pyrimidine_conversions idu(3);Thioredoxin-disulfide_reductase idu(3);Glycine_reductase,_sarcosine_reductase_and_betaine_reductase
fig|6666666.28482.peg.1953	Contig_18	9912	10133	+	Translation initiation factor 1	FIG00000143	isu;Translation_initiation_factors_bacterial
fig|6666666.28482.peg.1954	Contig_18	10166	10279	+	LSU ribosomal protein L36p	FIG00000229	isu;Ribosome_LSU_bacterial
fig|6666666.28482.peg.1955	Contig_18	10443	10817	+	SSU ribosomal protein S13p (S18e)	FIG00000160	ff
fig|6666666.28482.peg.1956	Contig_18	10824	11243	+	SSU ribosomal protein S11p (S14e)	FIG00055673	ff

fig|6666666.28482.peg.1957	Contig_18	11247	11852	+	SSU ribosomal protein S4p (S9e)	FIG00000148	isu;CBSS-393130.3.peg.794
fig|6666666.28482.peg.1958	Contig_18	11984	13027	+	DNA-directed RNA polymerase alpha subunit (EC 2.7.7.6)	FIG00000140	isu;RNA_polymerase_bacterial
fig|6666666.28482.peg.1959	Contig_18	13144	13788	+	LSU ribosomal protein L17p	FIG00000154	icw(1);Ribosome_LSU_bacterial
fig|6666666.28482.peg.1960	Contig_18	13919	14629	+	tRNA pseudouridine synthase A (EC 4.2.1.70)	FIG00000093	isu;tRNA_processing isu;RNA_pseudouridine_syntheses
fig|6666666.28482.peg.1961	Contig_18	15246	14626	-	serine esterase, cutinase family	FIG01349249	ff
fig|6666666.28482.peg.1962	Contig_18	16079	15303	-	serine esterase, cutinase family	FIG01349249	ff
fig|6666666.28482.peg.1963	Contig_18	16255	17643	+	PROBABLE CONSERVED MEMBRANE PROTEIN	FIG00662487	ff
fig|6666666.28482.peg.1964	Contig_18	18876	17608	-	subtilase family protein	FIG00546145	ff
fig|6666666.28482.peg.1965	Contig_18	20213	18873	-	PROBABLE CONSERVED INTEGRAL MEMBRANE PROTEIN		
fig|6666666.28482.peg.1966	Contig_18	20480	23926	+	FtsK/SpoIIIE family protein	FIG01309712	ff
fig|6666666.28482.peg.1967	Contig_18	23923	25179	+	Type VII secretion-associated protein	FIG00820120	ff
fig|6666666.28482.peg.1968	Contig_18	25251	25529	+	ESAT-6 like protein EsxU	FIG00822192	ff
fig|6666666.28482.peg.1969	Contig_18	25544	25840	+	probable secreted protein	FIG00999229	ff
fig|6666666.28482.peg.1970	Contig_18	27033	25894	-	hypothetical protein		
fig|6666666.28482.peg.1971	Contig_18	27318	28265	+	hypothetical protein		

fig|6666666.28482.peg.1972	Contig_18	31439	28266	-	Adenylate cyclase (EC 4.6.1.1)	FIG00001294	idu(25);cAMP_signaling_in_bacteria
fig|6666666.28482.peg.1973	Contig_18	31709	32152	+	LSU ribosomal protein L13p (L13Ae)	FIG00000211	isu;Ribosome_LSU_bacterial
fig|6666666.28482.peg.1974	Contig_18	32149	32640	+	SSU ribosomal protein S9p (S16e)	FIG00001110	ff
fig|6666666.28482.peg.1975	Contig_18	32767	34104	+	Phosphoglucosamine mutase (EC 5.4.2.10)	FIG00009682	isu;Sialic_Acid_Metabolism isu;UDP-N-acetylmuramate_from_Fructose-6-phosphate_Biosynthesis
fig|6666666.28482.peg.1976	Contig_18	34172	34438	+	FIG00820542: hypothetical protein	FIG00820541	ff
fig|6666666.28482.peg.1977	Contig_180	1086	943	-	hypothetical protein		
fig|6666666.28482.peg.1978	Contig_180	1463	1092	-	PE-PGRS FAMILY PROTEIN		
fig|6666666.28482.peg.1979	Contig_184	859	740	-	Mobile element protein	FIG01306568	ff
fig|6666666.28482.peg.1980	Contig_19	452	15	-	FIG00820003: hypothetical protein	FIG00820002	ff
fig|6666666.28482.peg.1981	Contig_19	1042	452	-	FIG00822063: hypothetical protein	FIG00822060	ff
fig|6666666.28482.peg.1982	Contig_19	1194	1598	+	PUTATIVE LIPOPROTEIN LPQV	FIG01316007	ff
fig|6666666.28482.peg.1983	Contig_19	1688	2743	+	UPF0028 protein YchK	FIG01304453	isu;Broadly_distributed_proteins_not_in_subsystems
fig|6666666.28482.peg.1984	Contig_19	3610	2744	-	probably phosphoesterase, evidenced by COGnitor	FIG01311108	ff
fig|6666666.28482.peg.1985	Contig_19	4476	3607	-	glutamine amidotransferases, class-II	FIG01443783	ff

fig|6666666.28482.peg.1986	Contig_19	5009	4518	-	FIG00820309: hypothetical protein	FIG00820308	ff
fig|6666666.28482.peg.1987	Contig_19	6068	5010	-	FIG00821199: hypothetical protein	FIG00821197	ff
fig|6666666.28482.peg.1988	Contig_19	7794	6166	-	Long-chain-fatty-acid--CoA ligase (EC 6.2.1.3)	FIG00018699	idu(72);n-Phenylalkanoic_acid_degradation idu(72);Fatty_acid_metabolism_cluster idu(72);Biotin_synthesis_cluster idu(72);Biotin_biosynthesis
fig|6666666.28482.peg.1989	Contig_19	8991	7912	-	hypothetical protein		
fig|6666666.28482.peg.1990	Contig_19	8965	9078	+	hypothetical protein		
fig|6666666.28482.peg.1991	Contig_19	9481	10641	+	hypothetical protein	FIG00638284	ff
fig|6666666.28482.peg.1992	Contig_19	10988	10650	-	Nitrogen regulatory protein P-II	FIG00005111	idu(1);Ammonia_assimilation
fig|6666666.28482.peg.1993	Contig_19	12411	11005	-	Ammonium transporter	FIG00004764	icw(1);Ammonia_assimilation
fig|6666666.28482.peg.1994	Contig_19	12799	14013	+	FAD dependent oxidoreductase		
fig|6666666.28482.peg.1995	Contig_19	14122	15513	+	Cytochrome P450	FIG01320789	ff
fig|6666666.28482.peg.1996	Contig_19	16231	15464	-	hypothetical protein		
fig|6666666.28482.peg.1997	Contig_19	16503	16228	-	hypothetical protein		
fig|6666666.28482.peg.1998	Contig_19	17270	16515	-	putative carboxymethylenebutenolidase(EC:3.1.1.45)	FIG00827058	ff
fig|6666666.28482.peg.1999	Contig_19	17444	18154	+	two-component system response regulator	FIG00517054	ff
fig|6666666.28482.peg.2000	Contig_19	18765	18157	-	sigma factor, sigma 70 type, group 4 (ECF)		

fig|6666666.28482.peg.2001	Contig_19	19045	19398	+	nitrate/nitrite response regulator protein		
fig|6666666.28482.peg.2002	Contig_19	19520	19395	-	hypothetical protein		
fig|6666666.28482.peg.2003	Contig_19	19643	20749	+	Heat shock protein HtrA		
fig|6666666.28482.peg.2004	Contig_19	22398	20788	-	hypothetical protein	FIG00638284	ff
fig|6666666.28482.peg.2005	Contig_19	23226	22633	-	hypothetical protein		
fig|6666666.28482.peg.2006	Contig_19	23557	23856	+	PE family protein		
fig|6666666.28482.peg.2007	Contig_19	23872	25002	+	PPE family protein	FIG00013075	idu(57);CBSS-164757.7.peg.5020
fig|6666666.28482.peg.2008	Contig_19	26239	25649	-	hypothetical protein		
fig|6666666.28482.peg.2009	Contig_19	27165	26269	-	hypothetical protein	FIG00638284	ff
fig|6666666.28482.peg.2010	Contig_19	28745	27165	-	3-(3-hydroxy-phenyl)propionate hydroxylase (EC 1.14.13.-)	FIG00138127	idu(3);Cinnamic_Acid_Degradation
fig|6666666.28482.peg.2011	Contig_19	29659	28742	-	Fumarylacetoacetate hydrolase family protein	FIG00007658	idu(1);Gentisare_degradation idu(1);Pterin_carbinolamine_dehydratase idu(1);Salicylate_and_gentisate_catabolism
fig|6666666.28482.peg.2012	Contig_19	30843	29698	-	2,3-dihydroxybiphenyl 1,2-dioxygenase	FIG00004009	ff
fig|6666666.28482.peg.2013	Contig_19	31306	31695	+	membrane protein, MmpS family		
fig|6666666.28482.peg.2014	Contig_19	31692	34625	+	Putative membrane protein		
fig|6666666.28482.peg.2015	Contig_19	34806	34672	-	hypothetical protein		

fig|6666666.28482.peg.2016	Contig_19	35271	36599	+	NADH dehydrogenase (EC 1.6.99.3)	FIG00008708	idu(6);Respiratory_dehydrogenases_1 idu(6);PPE_gene_cluster_in_Mycobacteria idu(6);Riboflavin_synthesis_cluster
fig|6666666.28482.peg.2017	Contig_19	40490	36657	-	signal transduction histidine kinase		
fig|6666666.28482.peg.2018	Contig_19	40840	42351	+	Adenylate cyclase (EC 4.6.1.1)	FIG00001294	idu(25);cAMP_signaling_in_bacteria
fig|6666666.28482.peg.2019	Contig_19	42643	42413	-	hypothetical protein		
fig|6666666.28482.peg.2020	Contig_19	42978	42847	-	hypothetical protein		
fig|6666666.28482.peg.2021	Contig_19	43021	44409	+	Succinate-semialdehyde dehydrogenase [NAD] (EC 1.2.1.24); Succinate-semialdehyde dehydrogenase [NADP+] (EC 1.2.1.16)	FIG01959002	ff
fig|6666666.28482.peg.2022	Contig_19	44449	46149	+	Pyruvate decarboxylase (EC 4.1.1.1); Alpha-keto-acid decarboxylase (EC 4.1.1.-)	FIG00001998	idu(2);Pyruvate_metabolism_II:_acetyl-CoA,_acetogenesis_from_pyruvate
fig|6666666.28482.peg.2023	Contig_19	46595	46263	-	hypothetical protein		
fig|6666666.28482.peg.2024	Contig_19	46786	46655	-	hypothetical protein		
fig|6666666.28482.peg.2025	Contig_19	48249	47002	-	glycosyltransferase, putative	FIG01227982	ff
fig|6666666.28482.peg.2026	Contig_19	48595	49275	+	hypothetical protein		
fig|6666666.28482.peg.2027	Contig_19	49576	49319	-	hypothetical protein		

fig|6666666.28482.peg.2028	Contig_19	52213	49973	-	Transmembrane transport protein MmpL13b	FIG01314334	ff
fig|6666666.28482.peg.2029	Contig_19	53322	52210	-	Chalcone synthase (EC 2.3.1.74)	FIG00443156	ff
fig|6666666.28482.peg.2030	Contig_19	55113	53344	-	Long-chain-fatty-acid--CoA ligase (EC 6.2.1.3)	FIG00018699	idu(72);n-Phenylalkanoic_acid_degradation idu(72);Fatty_acid_metabolism_cluster idu(72);Biotin_synthesis_cluster idu(72);Biotin_biosynthesis
fig|6666666.28482.peg.2031	Contig_19	55376	55110	-	hypothetical protein		
fig|6666666.28482.peg.2032	Contig_19	55747	55598	-	hypothetical protein		
fig|6666666.28482.peg.2033	Contig_19	55910	55770	-	hypothetical protein		
fig|6666666.28482.peg.2034	Contig_19	56399	56034	-	hypothetical protein		
fig|6666666.28482.peg.2035	Contig_19	56694	57530	+	secreted lipase		
fig|6666666.28482.peg.2036	Contig_19	57842	58897	+	putative surface layer protein		
fig|6666666.28482.peg.2037	Contig_19	59810	59055	-	hypothetical protein	FIG00638284	ff
fig|6666666.28482.peg.2038	Contig_19	59921	60280	+	hypothetical protein		
fig|6666666.28482.peg.2039	Contig_19	60363	60638	+	FIG00822349: hypothetical protein	FIG00822348	ff
fig|6666666.28482.peg.2040	Contig_19	60721	63078	+	Lead, cadmium, zinc and mercury transporting ATPase (EC 3.6.3.3) (EC 3.6.3.5); Copper-translocating P-type ATPase (EC 3.6.3.4)	FIG00658111	idu(1);CBSS-196620.1.peg.2477 idu(1);Copper_homeostasis
fig|6666666.28482.peg.2041	Contig_19	63090	63332	+	hypothetical protein		
fig|6666666.28482.peg.2042	Contig_19	63403	63981	+	FIG00826235: hypothetical protein	FIG00826234	ff

fig|6666666.28482.peg.2043	Contig_19	65553	63991	-	3-phytase precursor (EC 3.1.3.8)	FIG01041340	ff
fig|6666666.28482.peg.2044	Contig_19	66397	65624	-	FIG00823602: hypothetical protein	FIG00823600	ff
fig|6666666.28482.peg.2045	Contig_19	67672	66446	-	3-demethylubiquinone-9 3-methyltransferase (EC 2.1.1.64)	FIG00030029	ff
fig|6666666.28482.peg.2046	Contig_19	67791	68153	+	FIG00828335: hypothetical protein	FIG00828334	ff
fig|6666666.28482.peg.2047	Contig_19	68684	68280	-	hypothetical protein		
fig|6666666.28482.peg.2048	Contig_19	69363	68878	-	Lactoylglutathione lyase (EC 4.4.1.5)	FIG00004743	idu(1);Glutathione:_Non-redox_reactions idu(1);Methylglyoxal_Metabolism
fig|6666666.28482.peg.2049	Contig_19	69747	69388	-	Arsenate reductase (EC 1.20.4.1)	FIG00000770	icw(1);Transcription_repair_cluster icw(1);CBSS-315749.4.peg.3658 icw(2);Arsenic_resistance icw(1);Anaerobic_respiratory_reductases
fig|6666666.28482.peg.2050	Contig_19	70900	69794	-	Arsenate reductase (EC 1.20.4.1)	FIG00000770	icw(1);Transcription_repair_cluster icw(1);CBSS-315749.4.peg.3658 icw(2);Arsenic_resistance icw(1);Anaerobic_respiratory_reductases
fig|6666666.28482.peg.2051	Contig_19	71223	70897	-	Arsenical resistance operon repressor		idu(1);Arsenic_resistance
fig|6666666.28482.peg.2052	Contig_19	72016	72483	+	hypothetical protein		
fig|6666666.28482.peg.2053	Contig_19	72596	72781	+	hypothetical protein		
fig|6666666.28482.peg.2054	Contig_19	74306	73023	-	phage-related major capsid protein		

fig|6666666.28482.peg.2055	Contig_19	74292	74519	+	hypothetical protein		
fig|6666666.28482.peg.2056	Contig_19	74922	74590	-	hypothetical protein		
fig|6666666.28482.peg.2057	Contig_19	75158	74919	-	hypothetical protein		
fig|6666666.28482.peg.2058	Contig_19	75388	75155	-	hypothetical protein		
fig|6666666.28482.peg.2059	Contig_19	76010	75720	-	hypothetical protein		
fig|6666666.28482.peg.2060	Contig_19	76273	76007	-	hypothetical protein		
fig|6666666.28482.peg.2061	Contig_19	76560	76270	-	hypothetical protein		
fig|6666666.28482.peg.2062	Contig_19	77969	76557	-	hypothetical protein	FIG00638284	ff
fig|6666666.28482.peg.2063	Contig_19	78493	78335	-	hypothetical protein		
fig|6666666.28482.peg.2064	Contig_19	78643	79257	+	hypothetical protein		
fig|6666666.28482.peg.2065	Contig_19	79254	80480	+	site-specific recombinase, phage integrase family protein		
fig|6666666.28482.rna.31	Contig_19	80593	80520	-	tRNA-Leu-TAA		
fig|6666666.28482.peg.2066	Contig_19	80953	81801	+	PPE family protein		icw(1);CBSS-164757.7.peg.5020
fig|6666666.28482.peg.2067	Contig_19	81910	83046	+	PPE family protein	FIG00013075	icw(1);CBSS-164757.7.peg.5020
fig|6666666.28482.peg.2068	Contig_2	2017	77	-	Acyl-coenzyme A oxidase 3, peroxisomal (EC 1.3.3.6)	FIG00826267	ff
fig|6666666.28482.peg.2069	Contig_2	2159	2725	+	Putative protein-S-isoprenylcysteine methyltransferase		
fig|6666666.28482.peg.2070	Contig_2	4994	2709	-	FIG00820139: hypothetical protein	FIG00820138	ff
fig|6666666.28482.peg.2071	Contig_2	5660	5163	-	Transcriptional regulator, MarR family	FIG00554610	ff

fig|6666666.28482.peg.2072	Contig_2	5749	7836	+	Candidate substrate-specific domain of ECF transporters in Mycobacteria / Duplicated ATPase component of energizing module of predicted ECF transporter in Mycobacteria	FIG00108957	isu;ECF_class_transporters icw(1);ECF_class_transporters
fig|6666666.28482.peg.2073	Contig_2	7890	8684	+	Transmembrane component of energizing module of ECF transporters in Mycobacteria	FIG00143223	icw(1);ECF_class_transporters
fig|6666666.28482.peg.2074	Contig_2	8744	9139	+	hypothetical protein		
fig|6666666.28482.peg.2075	Contig_2	9132	10952	+	Choline-sulfatase (EC 3.1.6.6)	FIG00001198	isu;Sulfatases_and_sulfatase_modifying_factor_1 isu;Choline_and_Betaine_Uptake_and_Betaine_Biosynthesis
fig|6666666.28482.peg.2076	Contig_2	11587	10991	-	transcriptional regulator, TetR family		
fig|6666666.28482.peg.2077	Contig_2	11872	11747	-	hypothetical protein		
fig|6666666.28482.peg.2078	Contig_2	11937	12710	+	integral membrane protein		
fig|6666666.28482.peg.2079	Contig_2	12707	13654	+	Hydrolase, alpha/beta fold family protein		
fig|6666666.28482.peg.2080	Contig_2	14078	13632	-	Transcriptional regulator, AsnC family	FIG01956023	ff
fig|6666666.28482.peg.2081	Contig_2	14259	15032	+	NG,NG-dimethylarginine dimethylaminohydrolase 1 (EC 3.5.3.18)	FIG00002156	icw(1);Dimethylarginine_metabolism

fig|6666666.28482.peg.2082	Contig_2	15029	16264	+	Ornithine aminotransferase (EC 2.6.1.13)	FIG00035201	isu;Arginine_and_Ornithine_Degradation isu;Dimethylarginine_metabolism
fig|6666666.28482.peg.2083	Contig_2	16294	17724	+	Cationic amino acid transport integral membrane protein RocE	FIG01357586	ff
fig|6666666.28482.peg.2084	Contig_2	17721	18599	+	Universal stress protein family	FIG00017615	ff
fig|6666666.28482.peg.2085	Contig_2	19649	18684	-	FIG00821598: hypothetical membrane protein		
fig|6666666.28482.peg.2086	Contig_2	21020	19728	-	Periplasmic sugar-binding lipoprotein UspC	FIG01316674	ff
fig|6666666.28482.peg.2087	Contig_2	21889	21065	-	Inositol transport system permease protein	FIG00008562	ff
fig|6666666.28482.peg.2088	Contig_2	22760	21876	-	Multiple sugar ABC transporter, membrane-spanning permease protein MsmF	FIG00007586	ff
fig|6666666.28482.peg.2089	Contig_2	23677	22943	-	hypothetical protein	FIG00638284	ff
fig|6666666.28482.peg.2090	Contig_2	24714	23722	-	Glutamine-transport ATP-binding protein ABC transporter GlnQ		
fig|6666666.28482.peg.2091	Contig_2	25778	24717	-	Glutamine-transport transmembrane protein ABC transporter		
fig|6666666.28482.peg.2092	Contig_2	27090	26260	-	DNA-binding protein		

fig|6666666.28482.peg.2093	Contig_2	27933	27100	-	DNA-binding protein		
fig|6666666.28482.peg.2094	Contig_2	29542	28016	-	hypothetical protein		
fig|6666666.28482.peg.2095	Contig_2	30317	29553	-	3-oxoacyl-[acyl-carrier protein] reductase (EC 1.1.1.100)	FIG00621114	idu(38);CBSS-246196.1.peg.364 idu(38);CBSS-164757.7.peg.5020 idu(38);Fatty_Acid_Biosynthesis_FASII
fig|6666666.28482.peg.2096	Contig_2	30617	31204	+	hypothetical protein		
fig|6666666.28482.peg.2097	Contig_2	31461	32681	+	Glutaryl-CoA dehydrogenase (EC 1.3.99.7)	FIG01332484	ff
fig|6666666.28482.peg.2098	Contig_2	32695	33936	+	Hydroxyproline dehydratase putative	FIG01390869	ff
fig|6666666.28482.peg.2099	Contig_2	33933	34715	+	Enoyl-CoA hydratase (EC 4.2.1.17)	FIG00033206	idu(51);n-Phenylalkanoic_acid_degradation idu(51);Polyhydroxybutyrate_metabolism idu(51);Isoleucine_degradation idu(51);Fatty_acid_metabolism_cluster idu(51);Lysine_fermentation idu(51);Butanol_Biosynthesis idu(51);Acetyl-CoA_fermentation_to_Butyrate idu(51);Valine_degradation idu(51);Butyrate_metabolism_cluster

fig|6666666.28482.peg.2100	Contig_2	34765	35706	+	3-hydroxyacyl-CoA dehydrogenase (EC 1.1.1.35)	FIG00001558	icw(2);n-Phenylalkanoic_acid_degradation icw(2);Polyhydroxybutyrate_metabolism icw(2);Isoleucine_degradation icw(2);Fatty_acid_metabolism_cluster icw(2);Acetyl-CoA_fermentation_to_Butyrate icw(2);Valine_degradation icw(2);Butyrate_metabolism_cluster
fig|6666666.28482.peg.2101	Contig_2	35703	35816	+	hypothetical protein		
fig|6666666.28482.peg.2102	Contig_2	35896	36795	+	hypothetical protein	FIG00638284	ff
fig|6666666.28482.peg.2103	Contig_2	36792	38183	+	NADH dehydrogenase (EC 1.6.99.3)	FIG00008708	idu(6);Respiratory_dehydrogenases_1 idu(6);PPE_gene_cluster_in_Mycobacteria idu(6);Riboflavin_synthesis_cluster

fig|6666666.28482.peg.2104	Contig_2	38202	39089	+	3-hydroxybutyryl-CoA dehydrogenase (EC 1.1.1.157); 3-hydroxyacyl-CoA dehydrogenase (EC 1.1.1.35)	FIG00000819	icw(2);n-Phenylalkanoic_acid_degradation icw(2);Polyhydroxybutyrate_metabolism icw(2);Polyhydroxybutyrate_metabolism icw(2);Isoleucine_degradation icw(2);Fatty_acid_metabolism_cluster icw(1);Lysine_fermentation icw(1);Butanol_Biosynthesis icw(1);Acetyl-CoA_fermentation_to_Butyrate icw(2);Acetyl-CoA_fermentation_to_Butyrate icw(2);Valine_degradation icw(2);Butyrate_metabolism_cluster
fig|6666666.28482.peg.2105	Contig_2	39106	39855	+	hypothetical protein		
fig|6666666.28482.peg.2106	Contig_2	40153	40965	+	hypothetical protein	FIG00638284	ff
fig|6666666.28482.peg.2107	Contig_2	40978	41484	+	hypothetical protein		
fig|6666666.28482.peg.2108	Contig_2	41645	43162	+	TldD family protein, Actinobacterial subgroup	FIG00018909	idu(1);CBSS-316273.3.peg.448 idu(1);Putative_TldE-TldD_proteolytic_complex
fig|6666666.28482.peg.2109	Contig_2	43159	44532	+	TldE/PmbA family protein, Actinobacterial subgroup	FIG00020189	icw(1);CBSS-316273.3.peg.448 icw(1);Putative_TldE-TldD_proteolytic_complex
fig|6666666.28482.peg.2110	Contig_2	44597	45370	+	FIG00821236: hypothetical protein	FIG00821235	ff
fig|6666666.28482.peg.2111	Contig_2	45562	45431	-	hypothetical protein		
fig|6666666.28482.rna.32	Contig_2	46468	46541	+	tRNA-Met-CAT		

fig|6666666.28482.peg.2112	Contig_2	49360	48761	-	hypothetical protein		
fig|6666666.28482.peg.2113	Contig_2	53094	51814	-	Predicted protein kinase		
fig|6666666.28482.peg.2114	Contig_2	53119	53253	+	hypothetical protein		
fig|6666666.28482.peg.2115	Contig_2	53495	54646	+	putative cytochrome P450 hydroxylase	FIG00032437	idu(49);Nitric_oxide_synthase
fig|6666666.28482.peg.2116	Contig_2	54984	56297	+	Nitrilotriacetate monooxygenase component A (EC 1.14.13.-)	FIG01346536	ff
fig|6666666.28482.peg.2117	Contig_2	56568	56401	-	hypothetical protein		
fig|6666666.28482.peg.2118	Contig_2	56725	57639	+	PE-PGRS FAMILY PROTEIN		
fig|6666666.28482.peg.2119	Contig_2	59008	58373	-	hypothetical protein	FIG00638284	ff
fig|6666666.28482.peg.2120	Contig_2	59368	59156	-	hypothetical protein		
fig|6666666.28482.peg.2121	Contig_2	59990	60244	+	putative major facilitator superfamily protein	FIG01331046	ff
fig|6666666.28482.peg.2122	Contig_2	60451	62106	+	hypothetical protein		
fig|6666666.28482.peg.2123	Contig_2	62279	63427	+	Threonine dehydrogenase and related Zn-dependent dehydrogenases	FIG00053044	idu(2);Threonine_degradation
fig|6666666.28482.peg.2124	Contig_2	63446	64057	+	hypothetical protein	FIG00638284	ff
fig|6666666.28482.peg.2125	Contig_2	64408	64115	-	low molecular weight T-cell antigen TB8.4		
fig|6666666.28482.peg.2126	Contig_2	64776	64405	-	hypothetical protein		
fig|6666666.28482.peg.2127	Contig_2	66621	65581	-	protein of unknown function UPF0118	FIG00628027	ff
fig|6666666.28482.peg.2128	Contig_2	67477	66755	-	hypothetical protein		
fig|6666666.28482.peg.2129	Contig_2	68101	67775	-	hypothetical protein		
fig|6666666.28482.peg.2130	Contig_2	70060	68333	-	hypothetical protein	FIG00638284	ff

fig|6666666.28482.peg.2131	Contig_2	71473	70460	-	putative transcriptional regulator, AraC family		
fig|6666666.28482.peg.2132	Contig_2	71721	71939	+	hypothetical protein		
fig|6666666.28482.peg.2133	Contig_2	72145	72008	-	hypothetical protein		
fig|6666666.28482.peg.2134	Contig_2	72300	72860	+	ThiJ/PfpI family protein	FIG00015923	icw(1);CBSS-176280.1.peg.1561
fig|6666666.28482.peg.2135	Contig_2	73188	73445	+	hypothetical protein		
fig|6666666.28482.peg.2136	Contig_2	73469	74026	+	ThiJ/PfpI family protein	FIG00015923	icw(1);CBSS-176280.1.peg.1561
fig|6666666.28482.peg.2137	Contig_2	74190	74486	+	hypothetical protein	FIG00638284	ff
fig|6666666.28482.peg.2138	Contig_2	75952	74573	-	Glutamate decarboxylase (EC 4.1.1.15)	FIG01376814	ff
fig|6666666.28482.peg.2139	Contig_2	76565	77086	+	Possible membrane protein	FIG01121868	ff
fig|6666666.28482.peg.2140	Contig_2	77226	77666	+	hypothetical protein		
fig|6666666.28482.peg.2141	Contig_2	78286	79023	+	Oxidoreductase, aldo/keto reductase family	FIG00003513	ff
fig|6666666.28482.peg.2142	Contig_2	81032	79290	-	Thioredoxin reductase (EC 1.8.1.9)	FIG00018396	idu(3);pyrimidine_conversions idu(3);Thioredoxin-disulfide_reductase idu(3);Glycine_reductase,_sarcosine_reductase_and_betaine_reductase
fig|6666666.28482.peg.2143	Contig_2	81771	81322	-	thioredoxin	FIG00848322	ff
fig|6666666.28482.peg.2144	Contig_2	84409	81785	-	ClpB protein	FIG00025216	idu(1);Proteolysis_in_bacteria,_ATP-dependent idu(1);USS-DB-7 icw(3);Protein_chaperones idu(1);Type_VI_secretion_systems

fig|6666666.28482.peg.2145	Contig_2	84708	84391	-	FIG01123039: hypothetical protein	FIG01123038	ff
fig|6666666.28482.peg.2146	Contig_2	85619	84705	-	DnaJ-class molecular chaperone CbpA	FIG00027226	icw(2);Protein_chaperones
fig|6666666.28482.peg.2147	Contig_2	86150	85626	-	Heat shock protein GrpE	FIG00000103	icw(1);Heat_shock_dnaK_gene_cluster_extended idu(1);Protein_chaperones
fig|6666666.28482.peg.2148	Contig_2	88040	86157	-	Chaperone protein DnaK	FIG00023369	idu(1);Heat_shock_dnaK_gene_cluster_extended icw(1);Protein_chaperones
fig|6666666.28482.peg.2149	Contig_2	90013	88529	-	Succinate-semialdehyde dehydrogenase [NAD] (EC 1.2.1.24); Succinate-semialdehyde dehydrogenase [NADP+] (EC 1.2.1.16)	FIG01959002	ff
fig|6666666.28482.peg.2150	Contig_2	90601	91758	+	hypothetical protein	FIG00638284	ff
fig|6666666.28482.peg.2151	Contig_2	91869	92594	+	ThiJ/PfpI family protein	FIG00015923	idu(8);CBSS-176280.1.peg.1561
fig|6666666.28482.peg.2152	Contig_2	92729	95266	+	Excinuclease ABC subunit A paralog of unknown function	FIG00133034	isu;DNA_repair,_UvrABC_system
fig|6666666.28482.peg.2153	Contig_2	96862	95339	-	PE-PGRS FAMILY PROTEIN		
fig|6666666.28482.peg.2154	Contig_2	98358	97441	-	hypothetical protein	FIG00638284	ff
fig|6666666.28482.peg.2155	Contig_2	99612	98557	-	Transcriptional regulator, AraC family	FIG00510130	ff
fig|6666666.28482.peg.2156	Contig_2	99714	101510	+	FIG00828677: hypothetical protein	FIG00828675	ff
fig|6666666.28482.peg.2157	Contig_2	102059	102304	+	hypothetical protein		

fig|6666666.28482.peg.2158	Contig_2	103041	102421	-	Adenylate cyclase (EC 4.6.1.1)	FIG00001294	idu(25);cAMP_signaling_in_bacteria
fig|6666666.28482.peg.2159	Contig_2	103922	103365	-	D-alanyl-D-alanine carboxypeptidase family	FIG01875983	ff
fig|6666666.28482.peg.2160	Contig_2	104868	103984	-	hypothetical protein		
fig|6666666.28482.peg.2161	Contig_2	107738	104988	-	hypothetical protein	FIG00638284	ff
fig|6666666.28482.peg.2162	Contig_2	108470	107937	-	Metal-dependent hydrolase YbeY, involved in rRNA and/or ribosome maturation and assembly		icw(1);CBSS-56780.10.peg.1536
fig|6666666.28482.peg.2163	Contig_2	109513	108467	-	Phosphate starvation-inducible protein PhoH, predicted ATPase	FIG00045341	isu;CBSS-56780.10.peg.1536 isu;Phosphate_metabolism
fig|6666666.28482.peg.2164	Contig_2	110374	109571	-	Ribosomal RNA small subunit methyltransferase E (EC 2.1.1.-)	FIG00000248	icw(2);Heat_shock_dnaK_gene_cluster_extended isu;RNA_methylation
fig|6666666.28482.peg.2165	Contig_2	111626	110481	-	Chaperone protein DnaJ	FIG00000070	icw(1);Heat_shock_dnaK_gene_cluster_extended idu(1);Protein_chaperones
fig|6666666.28482.peg.2166	Contig_2	112740	111697	-	Heat-inducible transcription repressor HrcA	FIG00000544	isu;Heat_shock_dnaK_gene_cluster_extended
fig|6666666.28482.peg.2167	Contig_2	112905	113219	+	FIG00996129: hypothetical protein	FIG00820392	ff
fig|6666666.28482.peg.2168	Contig_2	113283	113879	+	FIG00821010: hypothetical protein	FIG01396054	ff

fig|6666666.28482.peg.2169	Contig_2	113876	114757	+	putative RNA polymerase sigma factor	FIG01121797	ff
fig|6666666.28482.peg.2170	Contig_2	115254	114751	-	Low molecular weight antigen MTB12 precursor	FIG01312173	ff
fig|6666666.28482.peg.2171	Contig_2	115884	115405	-	FIG00827776: hypothetical protein	FIG00827775	ff
fig|6666666.28482.peg.2172	Contig_2	116201	115986	-	Protein MbtH		
fig|6666666.28482.peg.2173	Contig_2	117486	116182	-	L-lysine 6-monooxygenase MbtG (EC 1.14.13.59)	FIG00821968	ff
fig|6666666.28482.peg.2174	Contig_2	121826	117483	-	Long-chain-fatty-acid--CoA ligase (EC 6.2.1.3)	FIG00018699	icw(1);n-Phenylalkanoic_acid_degradation icw(1);Fatty_acid_metabolism_cluster icw(1);Biotin_synthesis_cluster icw(1);Biotin_biosynthesis
fig|6666666.28482.peg.2175	Contig_2	126985	121823	-	Long-chain-fatty-acid--CoA ligase (EC 6.2.1.3)	FIG00018699	icw(1);n-Phenylalkanoic_acid_degradation icw(1);Fatty_acid_metabolism_cluster icw(1);Biotin_synthesis_cluster icw(1);Biotin_biosynthesis
fig|6666666.28482.peg.2176	Contig_2	129942	126982	-	Polyketide synthetase MbtD	FIG00821155	ff
fig|6666666.28482.peg.2177	Contig_2	131281	129968	-	Malonyl CoA-acyl carrier protein transacylase (EC 2.3.1.39)	FIG00000319	idu(7);Fatty_Acid_Biosynthesis_FASII

fig|6666666.28482.peg.2178	Contig_2	135494	131271	-	Phenyloxazoline synthase MbtB (EC 6.3.2.-)	FIG01192827	ff
fig|6666666.28482.peg.2179	Contig_2	135597	137240	+	Bifunctional salicyl-AMP ligase/salicyl-S-arcp synthetase MbtA	FIG01343285	ff
fig|6666666.28482.peg.2180	Contig_2	138253	137237	-	Fatty acid desaturase occurring in virulence cluster	FIG00996037	idu(2);Mycobacterium_virulence_operon_involved_in_lipid_metabolism
fig|6666666.28482.peg.2181	Contig_2	139776	138424	-	isochorismate synthase MbtI	FIG01363036	ff
fig|6666666.28482.peg.2182	Contig_2	140154	141392	+	putative sodium-dependent bicarbonate transporter	FIG00102673	icw(1);CO2_uptake,_carboxysome
fig|6666666.28482.peg.2183	Contig_2	141625	142869	+	putative sodium-dependent bicarbonate transporter	FIG00102673	icw(1);CO2_uptake,_carboxysome
fig|6666666.28482.peg.2184	Contig_2	143402	145141	+	Sulfate transporter		
fig|6666666.28482.peg.2185	Contig_2	146324	145152	-	Putative coproporphyrinogen III oxidase of BS HemN-type, oxygen-independent (EC 1.3.99.22), in heat shock gene cluster	FIG01303216	ff
fig|6666666.28482.peg.2186	Contig_2	146778	146299	-	hypothetical protein		
fig|6666666.28482.peg.2187	Contig_2	146749	148428	+	Ferredoxin--sulfite reductase, actinobacterial type (EC 1.8.7.1)	FIG00005903	icw(1);Inorganic_Sulfur_Assimilation

fig|6666666.28482.peg.2188	Contig_2	148425	149168	+	Phosphoadenylyl-sulfate reductase [thioredoxin] (EC 1.8.4.8) / Adenylyl-sulfate reductase [thioredoxin] (EC 1.8.4.10)	FIG01303877	icw(1);Cysteine_Biosynthesis icw(1);Inorganic_Sulfur_Assimilation icw(2);Inorganic_Sulfur_Assimilation
fig|6666666.28482.peg.2189	Contig_2	149252	149878	+	Sirohydrochlorin ferrochelatase (EC 4.99.1.4)	FIG00132864	idu(2);Heme_and_Siroheme_Biosynthesis
fig|6666666.28482.peg.2190	Contig_2	150054	149890	-	hypothetical protein		
fig|6666666.28482.peg.2191	Contig_2	150265	152211	+	Gamma-glutamyltranspeptidase (EC 2.3.2.2)	FIG00005992	idu(2);Glutathione:_Biosynthesis_and_gamma-glutamyl_cycle idu(2);Utilization_of_glutathione_as_a_sulphur_source idu(2);Poly-gamma-glutamate_biosynthesis
fig|6666666.28482.peg.2192	Contig_2	153309	152218	-	Sulfate and thiosulfate import ATP-binding protein CysA (EC 3.6.3.25)	FIG00000959	idu(1);Uptake_of_selenate_and_selenite icw(4);Cysteine_Biosynthesis icw(4);Inorganic_Sulfur_Assimilation
fig|6666666.28482.peg.2193	Contig_2	154146	153331	-	Sulfate transport system permease protein CysW	FIG01272192	icw(1);Cysteine_Biosynthesis icw(1);Inorganic_Sulfur_Assimilation
fig|6666666.28482.peg.2194	Contig_2	154991	154146	-	Sulfate transport system permease protein CysT	FIG00018897	icw(2);Cysteine_Biosynthesis icw(2);Inorganic_Sulfur_Assimilation
fig|6666666.28482.peg.2195	Contig_2	156034	154988	-	Sulfate and thiosulfate binding protein CysP	FIG00001000	idu(2);Cysteine_Biosynthesis idu(2);Inorganic_Sulfur_Assimilation
fig|6666666.28482.peg.2196	Contig_2	156541	156347	-	hypothetical protein		

fig|6666666.28482.peg.2197	Contig_2	156635	158686	+	Glucoamylase (EC 3.2.1.3)	FIG00012589	isu;Trehalose_Biosynthesis isu;Maltose_and_Maltodextrin_Utilization
fig|6666666.28482.peg.2198	Contig_2	159446	158709	-	PROBABLE CONSERVED LIPOPROTEIN LPPR	FIG01415001	ff
fig|6666666.28482.peg.2199	Contig_2	160401	159634	-	PROBABLE CONSERVED LIPOPROTEIN LPPR	FIG01415001	ff
fig|6666666.28482.peg.2200	Contig_2	162341	160404	-	Translation elongation factor LepA	FIG00000165	isu;Heat_shock_dnaK_gene_cluster_extended isu;Translation_elongation_factors_bacterial
fig|6666666.28482.peg.2201	Contig_2	162369	162947	+	RNA 3'-terminal phosphate cyclase (EC 6.5.1.4)	FIG00002119	isu;RNA_3'-terminal_phosphate_cyclase isu;tRNA_splicing
fig|6666666.28482.peg.2202	Contig_2	163402	162974	-	Inosine-5'-monophosphate dehydrogenase (EC 1.1.1.205)	FIG00020613	idu(4);Purine_conversions idu(4);Purine_salvage_cluster
fig|6666666.28482.peg.2203	Contig_2	163576	164418	+	metallo-beta-lactamase family protein	FIG00882049	ff
fig|6666666.28482.peg.2204	Contig_2	165058	164402	-	hypothetical protein		
fig|6666666.28482.peg.2205	Contig_2	165216	165710	+	Transcriptional regulator, MarR family		
fig|6666666.28482.peg.2206	Contig_20	130	1626	+	hypothetical protein	FIG00638284	ff

fig|6666666.28482.peg.2207	Contig_20	2773	1709	-	N-succinyl-L,L-diaminopimelate desuccinylase (EC 3.5.1.18)	FIG00133108	isu;Lysine_Biosynthesis_DAP_Pathway,_GJO_scratch isu;Arginine_Biosynthesis_--_gjo isu;A_Glutathione-dependent_Thiol_Reductase_Associated_with_a_Step_in_Lysine_Biosynthesis isu;Lysine_Biosynthesis_DAP_Pathway isu;Arginine_Biosynthesis_extended
fig|6666666.28482.peg.2208	Contig_20	2818	3771	+	2,3,4,5-tetrahydropyridine-2,6-dicarboxylate N-succinyltransferase (EC 2.3.1.117)	FIG00000908	icw(1);Lysine_Biosynthesis_DAP_Pathway,_GJO_scratch icw(1);Lysine_Biosynthesis_DAP_Pathway
fig|6666666.28482.peg.2209	Contig_20	5074	3773	-	Permeases of the major facilitator superfamily	FIG00613966	ff
fig|6666666.28482.peg.2210	Contig_20	6687	5257	-	Long-chain-fatty-acid--CoA ligase (EC 6.2.1.3)	FIG00018699	idu(72);n-Phenylalkanoic_acid_degradation idu(72);Fatty_acid_metabolism_cluster idu(72);Biotin_synthesis_cluster idu(72);Biotin_biosynthesis
fig|6666666.28482.peg.2211	Contig_20	7608	6697	-	FIG00825538: hypothetical protein	FIG00825535	ff
fig|6666666.28482.peg.2212	Contig_20	9179	7710	-	Betaine aldehyde dehydrogenase (EC 1.2.1.8)	FIG01304892	idu(1);Choline_uptake_and_conversion_to_betaine_clusters idu(1);Choline_and_Betaine_Uptake_and_Betaine_Biosynthesis

fig|6666666.28482.peg.2213	Contig_20	9426	9974	+	FIG00820262: hypothetical protein	FIG00820260	ff
fig|6666666.28482.peg.2214	Contig_20	9974	10384	+	Acetyl-CoA acetyltransferase	FIG01005417	ff
fig|6666666.28482.peg.2215	Contig_20	10381	10821	+	involved in the lipid metabolism	FIG01582522	ff
fig|6666666.28482.peg.2216	Contig_20	10818	11987	+	putative thiolase	FIG01122915	ff
fig|6666666.28482.peg.2217	Contig_20	11984	12586	+	Transcriptional regulator, TetR family	FIG00554417	ff
fig|6666666.28482.peg.2218	Contig_20	13336	12590	-	metallo-beta-lactamase family protein		
fig|6666666.28482.peg.2219	Contig_20	14420	13452	-	Proline dehydrogenase (EC 1.5.99.8) (Proline oxidase)	FIG00002570	isu;Respiratory_dehydrogenases_1 isu;Proline,_4-hydroxyproline_uptake_and_utilization
fig|6666666.28482.peg.2220	Contig_20	16051	14420	-	Delta-1-pyrroline-5-carboxylate dehydrogenase (EC 1.5.1.12)	FIG00043720	isu;Arginine_and_Ornithine_Degradation isu;CBSS-196620.1.peg.2477 icw(1);Proline,_4-hydroxyproline_uptake_and_utilization
fig|6666666.28482.peg.2221	Contig_20	16136	17716	+	Regulator of polyketide synthase expression	FIG00056421	ff
fig|6666666.28482.peg.2222	Contig_20	18002	18490	+	PE-PGRS FAMILY PROTEIN		
fig|6666666.28482.peg.2223	Contig_20	19347	18595	-	FIG025093: Probable membrane protein		idu(2);Mycobacterium_virulence_operon_involved_in_protein_synthesis_(SSU_ribosomal_proteins)
fig|6666666.28482.peg.2224	Contig_20	19811	19482	-	FIG00826766: hypothetical protein		

fig|6666666.28482.peg.2225	Contig_20	20497	19928	-	hypothetical protein		
fig|6666666.28482.peg.2226	Contig_20	20567	21370	+	Sulfotransferase	FIG00995709	ff
fig|6666666.28482.peg.2227	Contig_20	21367	22284	+	Sulfate adenylyltransferase subunit 2 (EC 2.7.7.4)	FIG00000728	icw(1);Cysteine_Biosynthesis icw(1);Inorganic_Sulfur_Assimilation
fig|6666666.28482.peg.2228	Contig_20	22284	24194	+	Sulfate adenylyltransferase subunit 1 (EC 2.7.7.4) / Adenylylsulfate kinase (EC 2.7.1.25)	FIG00133521	idu(1);Cysteine_Biosynthesis icw(1);Cysteine_Biosynthesis idu(1);O-Methyl_Phosphoramidate_Capsule_Modification_in_Campylobacter idu(1);Inorganic_Sulfur_Assimilation icw(1);Inorganic_Sulfur_Assimilation
fig|6666666.28482.peg.2229	Contig_20	24250	24387	+	hypothetical protein		
fig|6666666.28482.peg.2230	Contig_20	24392	25519	+	FIG00823877: hypothetical protein	FIG00823876	ff
fig|6666666.28482.peg.2231	Contig_20	25549	26193	+	Lepb1170_F2_64	FIG00821835	ff
fig|6666666.28482.peg.2232	Contig_20	28565	26229	-	FIG00821312: hypothetical protein	FIG00821311	ff
fig|6666666.28482.peg.2233	Contig_20	29697	28612	-	N-succinyl-L,L-diaminopimelate aminotransferase alternative (EC 2.6.1.17)	FIG00001368	isu;Lysine_Biosynthesis_DAP_Pathway,_GJO_scratch isu;Lysine_Biosynthesis_DAP_Pathway
fig|6666666.28482.peg.2234	Contig_20	30032	29706	-	4Fe-4S ferredoxin, iron-sulfur binding	FIG00551095	idu(1);Inorganic_Sulfur_Assimilation
fig|6666666.28482.peg.2235	Contig_20	30402	30737	+	LOW MOLECULAR WEIGHT T-CELL ANTIGEN TB8.4	FIG00821016	ff
fig|6666666.28482.peg.2236	Contig_20	31571	30750	-	FIG00827386: hypothetical protein	FIG00827382	ff

fig|6666666.28482.peg.2237	Contig_20	34218	31636	-	7,8-didemethyl-8-hydroxy-5-deazariboflavin synthase subunit 1 / 7,8-didemethyl-8-hydroxy-5-deazariboflavin synthase subunit 2	FIG00073737	isu;Coenzyme_F420_synthesis isu;Coenzyme_F420_synthesis
fig|6666666.28482.peg.2238	Contig_20	34446	35534	+	PE-PGRS FAMILY PROTEIN		
fig|6666666.28482.peg.2239	Contig_20	36005	35544	-	FIG00820431: hypothetical protein	FIG00820429	ff
fig|6666666.28482.peg.2240	Contig_20	37000	36098	-	N-acetyl-1-D-myo-inosityl-2-amino-2-deoxy-alpha-D-glucopyranoside deacetylase MshB	FIG00023524	isu;Glutathione_analogs:_mycothiol
fig|6666666.28482.peg.2241	Contig_20	37121	37672	+	Transcriptional regulator, TetR family	FIG00554417	ff
fig|6666666.28482.peg.2242	Contig_20	39951	37669	-	hypothetical protein	FIG00638284	ff
fig|6666666.28482.peg.2243	Contig_20	42110	40185	-	LpqW	FIG00544125	ff
fig|6666666.28482.peg.2244	Contig_20	44028	42139	-	GTP-binding protein TypA/BipA	FIG00000268	ff
fig|6666666.28482.peg.2245	Contig_20	44574	44182	-	Mutator mutT protein (7,8-dihydro-8-oxoguanine-triphosphatase) (EC 3.6.1.-)	FIG00000892	isu;Nudix_proteins_(nucleoside_triphosphate_hydrolases)
fig|6666666.28482.peg.2246	Contig_20	44775	45551	+	FIG00828295: hypothetical protein	FIG00828293	ff

fig|6666666.28482.peg.2247	Contig_20	45593	45877	+	Pterin-4-alpha-carbinolamine dehydratase (EC 4.2.1.96)	FIG00004933	isu;Pterin_carbinolamine_dehydratase
fig|6666666.28482.peg.2248	Contig_20	47136	45874	-	probable conserved membrane protein	FIG01001774	ff
fig|6666666.28482.peg.2249	Contig_20	47240	47911	+	Conserved alanine and proline rich protein	FIG00821598	ff
fig|6666666.28482.peg.2250	Contig_21	992	513	-	FIG00821872: hypothetical protein	FIG00821871	ff
fig|6666666.28482.peg.2251	Contig_21	1757	1008	-	Dehydrogenases with different specificities (related to short-chain alcohol dehydrogenases)	FIG00975245	ff
fig|6666666.28482.peg.2252	Contig_21	2992	1754	-	alkyl sulfatase (EC 3.1.6.-)	FIG01326491	ff
fig|6666666.28482.peg.2253	Contig_21	4275	3007	-	FIG00826152: hypothetical protein	FIG00826150	ff
fig|6666666.28482.peg.2254	Contig_21	4669	4283	-	FIG00832292: hypothetical protein	FIG00828234	ff
fig|6666666.28482.peg.2255	Contig_21	4793	5179	+	hypothetical protein		
fig|6666666.28482.peg.2256	Contig_21	5416	6546	+	conserved hypothetical secreted protein		
fig|6666666.28482.peg.2257	Contig_21	6984	6577	-	Arsenate reductase (EC 1.20.4.1)	FIG00000770	idu(5);Transcription_repair_cluster idu(5);CBSS-315749.4.peg.3658 idu(5);Arsenic_resistance idu(5);Anaerobic_respiratory_reductases
fig|6666666.28482.rna.33	Contig_21	7218	7132	-	tRNA-Ser-CGA		isu;tRNAs

fig|6666666.28482.peg.2258	Contig_21	7813	7352	-	tRNA-specific adenosine-34 deaminase (EC 3.5.4.-)	FIG00000291	isu;tRNA_processing
fig|6666666.28482.peg.2259	Contig_21	8286	7867	-	FIG00994171: hypothetical protein	FIG00821149	ff
fig|6666666.28482.peg.2260	Contig_21	8503	9441	+	Arogenate dehydrogenase (EC 1.3.1.43)	FIG00132820	isu;Chorismate_Synthesis isu;Ribosome_post-transcriptional_modification_and_chromosomal_segregation_cluster isu;Phenylalanine_and_Tyrosine_Branches_from_Chorismate
fig|6666666.28482.peg.2261	Contig_21	10003	9404	-	FIG00998044: hypothetical protein	FIG00822636	ff
fig|6666666.28482.peg.2262	Contig_21	10754	10008	-	Glycine betaine ABC transport system permease protein	FIG01954415	icw(1);Choline_and_Betaine_Uptake_and_Betaine_Biosynthesis
fig|6666666.28482.peg.2263	Contig_21	11437	10751	-	L-proline glycine betaine ABC transport system permease protein ProW (TC 3.A.1.12.1)	FIG00005559	icw(3);Choline_and_Betaine_Uptake_and_Betaine_Biosynthesis
fig|6666666.28482.peg.2264	Contig_21	12531	11440	-	L-proline glycine betaine ABC transport system permease protein ProV (TC 3.A.1.12.1)	FIG00008556	isu;Choline_and_Betaine_Uptake_and_Betaine_Biosynthesis
fig|6666666.28482.peg.2265	Contig_21	13475	12528	-	L-proline glycine betaine binding ABC transporter protein ProX (TC 3.A.1.12.1)	FIG00002823	icw(2);Choline_and_Betaine_Uptake_and_Betaine_Biosynthesis

fig|6666666.28482.peg.2266	Contig_21	13584	13934	+	Cell division protein DivIC (FtsB), stabilizes FtsL against RasP cleavage	FIG00485194	idu(10);Possible_new_toxin-antitoxin_system_including_DivIC idu(10);Bacterial_Cell_Division idu(10);Cell_division-ribosomal_stress_proteins_cluster idu(10);Bacterial_Cytoskeleton idu(10);Stationary_phase_repair_cluster
fig|6666666.28482.peg.2267	Contig_21	15146	14037	-	Acyl-CoA dehydrogenase FadE36 (EC 1.3.99.-)	FIG01332120	ff
fig|6666666.28482.peg.2268	Contig_21	15780	15148	-	FIG00824627: hypothetical protein	FIG00824624	ff
fig|6666666.28482.peg.2269	Contig_21	15864	16535	+	FIG00823067: hypothetical protein	FIG00823066	ff
fig|6666666.28482.peg.2270	Contig_21	16603	17634	+	probable phosphotransferase	FIG01454510	ff
fig|6666666.28482.peg.2271	Contig_21	17636	18400	+	Oxidoreductase, short-chain dehydrogenase/reductase family	FIG00993242	ff
fig|6666666.28482.peg.2272	Contig_21	18421	19704	+	Butyryl-CoA dehydrogenase (EC 1.3.99.2)	FIG00009149	idu(39);Isoleucine_degradation idu(39);Lysine_fermentation idu(39);Butanol_Biosynthesis idu(39);Acetyl-CoA_fermentation_to_Butyrate idu(39);Valine_degradation idu(39);Anaerobic_respiratory_reductases
fig|6666666.28482.peg.2273	Contig_21	19694	20305	+	Transcriptional regulator, TetR family	FIG00554417	ff

fig|6666666.28482.peg.2274	Contig_21	20305	21276	+	Quinone oxidoreductase (EC 1.6.5.5)	FIG00002982	idu(7);Quinone_oxidoreductase_family
fig|6666666.28482.peg.2275	Contig_21	23223	21349	-	alkyl sulfatase (EC 3.1.6.-)	FIG01326491	ff
fig|6666666.28482.peg.2276	Contig_21	23549	23869	+	19 kDa lipoprotein antigen precursor LpqH	FIG01316369	ff
fig|6666666.28482.peg.2277	Contig_21	25439	23922	-	sensor histidine kinase	FIG01107919	ff
fig|6666666.28482.peg.2278	Contig_21	26121	25417	-	putative two-component system response regulator	FIG01124340	ff
fig|6666666.28482.peg.2279	Contig_21	26424	26269	-	hypothetical protein		
fig|6666666.28482.peg.2280	Contig_21	26704	26471	-	hypothetical protein		
fig|6666666.28482.peg.2281	Contig_21	27217	26807	-	FIG172205: hypothetical protein	FIG01517835	ff
fig|6666666.28482.peg.2282	Contig_21	27317	28153	+	FIG00821932: hypothetical protein	FIG00821929	ff
fig|6666666.28482.peg.2283	Contig_21	28398	28267	-	hypothetical protein		
fig|6666666.28482.peg.2284	Contig_21	28409	28645	+	protein of unknown function DUF1271		
fig|6666666.28482.peg.2285	Contig_21	29631	28687	-	O-Methyltransferase involved in polyketide biosynthesis	FIG01313020	ff
fig|6666666.28482.peg.2286	Contig_21	29735	30082	+	hypothetical protein		
fig|6666666.28482.peg.2287	Contig_21	30085	31119	+	probable phosphotransferase	FIG01454510	ff
fig|6666666.28482.peg.2288	Contig_21	31139	31639	+	hypothetical protein	FIG00638284	ff
fig|6666666.28482.peg.2289	Contig_21	31678	32298	+	hypothetical protein		
fig|6666666.28482.peg.2290	Contig_21	32341	32883	+	GCN5-related N-acetyltransferase		

fig|6666666.28482.peg.2291	Contig_22	11	1141	+	360aa long hypothetical malate dehydrogenase(EC:1.1.1.37)		
fig|6666666.28482.peg.2292	Contig_22	1179	2300	+	hypothetical protein		
fig|6666666.28482.peg.2293	Contig_22	3101	3349	+	hypothetical protein		
fig|6666666.28482.peg.2294	Contig_22	3357	3512	+	hypothetical protein		
fig|6666666.28482.peg.2295	Contig_22	4341	3589	-	transcriptional regulator, LuxR family		
fig|6666666.28482.peg.2296	Contig_22	4524	5318	+	3-oxoacyl-[acyl-carrier protein] reductase (EC 1.1.1.100)	FIG00621114	idu(38);CBSS-246196.1.peg.364 idu(38);CBSS-164757.7.peg.5020 idu(38);Fatty_Acid_Biosynthesis_FASI I
fig|6666666.28482.peg.2297	Contig_22	5645	6085	+	hypothetical protein		
fig|6666666.28482.peg.2298	Contig_22	6757	6122	-	Mobile element protein	FIG01306568	ff
fig|6666666.28482.peg.2299	Contig_22	9258	7627	-	Cyclohexanone monooxygenase (EC 1.14.13.22)	FIG00975774	ff
fig|6666666.28482.peg.2300	Contig_22	9977	9357	-	transcriptional regulator, TetR family		
fig|6666666.28482.peg.2301	Contig_22	10129	11247	+	Alcohol dehydrogenase (EC 1.1.1.1)	FIG01007502	idu(17);Glycerolipid_and_Glycerophospholipid_Metabolism_in_Bacteria idu(17);Fermentations:_Mixed_acid idu(17);Butanol_Biosynthesis
fig|6666666.28482.peg.2302	Contig_22	11416	11234	-	hypothetical protein		
fig|6666666.28482.peg.2303	Contig_22	11704	12255	+	regulatory protein, TetR		
fig|6666666.28482.peg.2304	Contig_22	13476	12292	-	Esterase A	FIG01314929	ff
fig|6666666.28482.peg.2305	Contig_22	13716	14885	+	luciferase		

fig|6666666.28482.peg.2306	Contig_22	15785	15051	-	20-beta-hydroxysteroid dehydrogenase FabG3(EC:1.1.1.53)		
fig|6666666.28482.peg.2307	Contig_22	16925	15879	-	Alcohol dehydrogenase (EC 1.1.1.1)	FIG01007502	idu(17);Glycerolipid_and_Glycerophospholipid_Metabolism_in_Bacteria idu(17);Fermentations:_Mixed_acid idu(17);Butanol_Biosynthesis
fig|6666666.28482.peg.2308	Contig_22	17496	17320	-	hypothetical protein		
fig|6666666.28482.peg.2309	Contig_22	18600	17551	-	probable phosphotransferase	FIG01454510	ff
fig|6666666.28482.peg.2310	Contig_22	19337	18591	-	Oxidoreductase, short-chain dehydrogenase/reductase family	FIG00993242	ff
fig|6666666.28482.peg.2311	Contig_22	20631	19372	-	putative acyl-CoA dehydrogenase(EC:1.3.99.3)		
fig|6666666.28482.peg.2312	Contig_22	20803	21519	+	Transcriptional regulator, TetR family	FIG00554417	ff
fig|6666666.28482.peg.2313	Contig_22	21516	21965	+	hypothetical protein		
fig|6666666.28482.peg.2314	Contig_22	23590	22451	-	acyl-CoA dehydrogenase-like		
fig|6666666.28482.peg.2315	Contig_22	24546	23587	-	acyl-CoA dehydrogenase domain protein		
fig|6666666.28482.peg.2316	Contig_22	25225	24956	-	hypothetical protein		

fig|6666666.28482.peg.2317	Contig_22	26824	25265	-	Long-chain-fatty-acid--CoA ligase (EC 6.2.1.3)	FIG00018699	idu(72);n-Phenylalkanoic_acid_degradation idu(72);Fatty_acid_metabolism_cluster idu(72);Biotin_synthesis_cluster idu(72);Biotin_biosynthesis
fig|6666666.28482.peg.2318	Contig_22	28251	27001	-	conserved hypothetical protein		
fig|6666666.28482.peg.2319	Contig_22	29312	28320	-	FIG00823018: hypothetical protein	FIG00823016	ff
fig|6666666.28482.peg.2320	Contig_22	30253	29396	-	amidohydrolase family protein		
fig|6666666.28482.peg.2321	Contig_22	30424	30675	+	acetyl-CoA acetyltransferase		

fig|6666666.28482.peg.2322	Contig_22	30633	30800	+	3-ketoacyl-CoA thiolase (EC 2.3.1.16) @ Acetyl-CoA acetyltransferase (EC 2.3.1.9)	FIG00000004	icw(2);CBSS-246196.1.peg.364 idu(9);Serine-glyoxylate_cycle idu(11);Serine-glyoxylate_cycle icw(1);n-Phenylalkanoic_acid_degradation idu(9);Ethylmalonyl-CoA_pathway_of_C2_assimilation,_GJO idu(9);Polyhydroxybutyrate_metabolism idu(11);Polyhydroxybutyrate_metabolism idu(11);Isoleucine_degradation icw(1);Fatty_acid_metabolism_cluster icw(1);Biotin_synthesis_cluster icw(1);Biotin_biosynthesis idu(9);Lysine_fermentation idu(9);Isoprenoid_Biosynthesis idu(9);Butanol_Biosynthesis idu(9);Acetyl-CoA_fermentation_to_Butyrate idu(9);Butyrate_metabolism_cluster
fig|6666666.28482.peg.2323	Contig_22	30800	31537	+	3-oxoacyl-[acyl-carrier protein] reductase (EC 1.1.1.100)	FIG00621114	icw(1);CBSS-246196.1.peg.364 icw(1);CBSS-164757.7.peg.5020 icw(1);Fatty_Acid_Biosynthesis_FASII
fig|6666666.28482.peg.2324	Contig_22	31547	32314	+	3-oxoacyl-[acyl-carrier protein] reductase (EC 1.1.1.100)	FIG00621114	icw(1);CBSS-246196.1.peg.364 icw(1);CBSS-164757.7.peg.5020 icw(1);Fatty_Acid_Biosynthesis_FASII

fig|6666666.28482.peg.2325	Contig_22	32333	34138	+	3-methylmercaptopropionyl-CoA dehydrogenase (DmdC)	FIG00802415	ff
fig|6666666.28482.peg.2326	Contig_22	34470	34285	-	hypothetical protein		
fig|6666666.28482.peg.2327	Contig_22	34451	34765	+	hypothetical protein		
fig|6666666.28482.peg.2328	Contig_22	36107	35022	-	Alpha-methylacyl-CoA racemase (EC 5.1.99.4)	FIG01312689	ff
fig|6666666.28482.peg.2329	Contig_22	36998	36852	-	hypothetical protein		
fig|6666666.28482.peg.2330	Contig_22	37264	37028	-	hypothetical protein		
fig|6666666.28482.peg.2331	Contig_22	37364	38230	+	Coenzyme F420-dependent oxidoreductase	FIG00015014	idu(3);Anaerobic_respiratory_reductases
fig|6666666.28482.peg.2332	Contig_22	38227	38964	+	hypothetical protein		
fig|6666666.28482.peg.2333	Contig_22	40076	39459	-	Transcriptional regulator, TetR family	FIG00554417	ff
fig|6666666.28482.peg.2334	Contig_22	41362	40157	-	putative cytochrome P450 hydroxylase	FIG00032437	idu(49);Nitric_oxide_synthase
fig|6666666.28482.peg.2335	Contig_22	42144	41587	-	Transcriptional regulator, TetR family	FIG00628138	ff
fig|6666666.28482.peg.2336	Contig_22	42381	43235	+	hypothetical protein		
fig|6666666.28482.peg.2337	Contig_22	44597	43371	-	L-carnitine dehydratase/bile acid-inducible protein F	FIG01318850	ff
fig|6666666.28482.peg.2338	Contig_22	45474	44848	-	hypothetical protein		
fig|6666666.28482.peg.2339	Contig_22	47325	45787	-	Long-chain-fatty-acid--CoA ligase (EC 6.2.1.3)	FIG00018699	idu(72);n-Phenylalkanoic_acid_degradation idu(72);Fatty_acid_metabolism_cluster idu(72);Biotin_synthesis_cluster idu(72);Biotin_biosynthesis

fig|6666666.28482.peg.2340	Contig_22	48478	47879	-	hypothetical protein		
fig|6666666.28482.peg.2341	Contig_22	49933	48653	-	FIG00821608: hypothetical protein	FIG00821607	ff
fig|6666666.28482.peg.2342	Contig_22	50312	51091	+	short-chain dehydrogenase/reductase SDR		
fig|6666666.28482.peg.2343	Contig_22	51789	51478	-	fdxD-related protein	FIG01419919	ff
fig|6666666.28482.peg.2344	Contig_22	53084	51786	-	NADH-quinone oxidoreductase chain F 2 (EC 1.6.99.5); Hypothetical FeS oxidoreductase	FIG00087063	idu(1);Respiratory_Complex_I
fig|6666666.28482.peg.2345	Contig_22	53473	53081	-	Rieske (2Fe-2S) domain-containing protein	FIG01358726	ff
fig|6666666.28482.peg.2346	Contig_22	54616	53543	-	Amidohydrolase 2	FIG00821472	ff
fig|6666666.28482.peg.2347	Contig_22	55817	54711	-	FIG00821228: hypothetical protein	FIG01354128	ff
fig|6666666.28482.peg.2348	Contig_22	56165	57535	+	putative cytochrome P450 hydroxylase	FIG00032437	idu(49);Nitric_oxide_synthase
fig|6666666.28482.peg.2349	Contig_22	58874	57909	-	Rieske (2Fe-2S) domain protein		
fig|6666666.28482.peg.2350	Contig_22	59521	59955	+	XoxI		
fig|6666666.28482.peg.2351	Contig_22	61379	60126	-	FIG00822014: hypothetical protein	FIG01547276	ff
fig|6666666.28482.peg.2352	Contig_22	62527	61376	-	Acyl-CoA dehydrogenase, short-chain specific (EC 1.3.99.2)	FIG00009849	idu(2);Isoleucine_degradation
fig|6666666.28482.peg.2353	Contig_22	63707	62814	-	hypothetical protein		
fig|6666666.28482.peg.2354	Contig_22	63812	64303	+	hypothetical protein	FIG00638284	ff

fig|6666666.28482.peg.2355	Contig_22	64325	65752	+	Succinate-semialdehyde dehydrogenase [NADP+] (EC 1.2.1.16)	FIG01958943	ff
fig|6666666.28482.peg.2356	Contig_22	65749	66186	+	hypothetical protein		
fig|6666666.28482.peg.2357	Contig_22	67200	66499	-	Short-chain dehydrogenase/reductase SDR		idu(13);CBSS-164757.7.peg.5020 idu(13);Transcription_repair_cluster
fig|6666666.28482.peg.2358	Contig_22	68623	67421	-	putative cytochrome P450 hydroxylase	FIG00032437	idu(49);Nitric_oxide_synthase
fig|6666666.28482.peg.2359	Contig_22	68727	69539	+	Enoyl-CoA hydratase (EC 4.2.1.17)	FIG00033206	idu(51);n-Phenylalkanoic_acid_degradation idu(51);Polyhydroxybutyrate_metabolism idu(51);Isoleucine_degradation idu(51);Fatty_acid_metabolism_cluster idu(51);Lysine_fermentation idu(51);Butanol_Biosynthesis idu(51);Acetyl-CoA_fermentation_to_Butyrate idu(51);Valine_degradation idu(51);Butyrate_metabolism_cluster
fig|6666666.28482.peg.2360	Contig_22	70242	71624	+	aldehyde dehydrogenase		
fig|6666666.28482.peg.2361	Contig_22	71889	72320	+	hypothetical protein		
fig|6666666.28482.peg.2362	Contig_22	73695	72412	-	hypothetical protein		
fig|6666666.28482.peg.2363	Contig_22	73849	73712	-	hypothetical protein		

fig|6666666.28482.peg.2364	Contig_22	75478	73973	-	Long-chain-fatty-acid--CoA ligase (EC 6.2.1.3)	FIG00018699	idu(72);n-Phenylalkanoic_acid_degradation idu(72);Fatty_acid_metabolism_cluster idu(72);Biotin_synthesis_cluster idu(72);Biotin_biosynthesis
fig|6666666.28482.peg.2365	Contig_22	76230	75475	-	hypothetical protein		
fig|6666666.28482.peg.2366	Contig_22	77696	76230	-	Aldehyde dehydrogenase (EC 1.2.1.3)	FIG01292786	idu(16);Pyruvate_metabolism_II:_acetyl-CoA,_acetogenesis_from_pyruvate idu(16);Glycerolipid_and_Glycerophospholipid_Metabolism_in_Bacteria idu(16);Methylglyoxal_Metabolism
fig|6666666.28482.peg.2367	Contig_22	78408	77800	-	Transcriptional regulator, TetR family		
fig|6666666.28482.peg.2368	Contig_22	79243	78650	-	Transcriptional regulator, TetR family		
fig|6666666.28482.peg.2369	Contig_22	79576	81594	+	Acetoin dehydrogenase E1 component alpha-subunit (EC 1.2.4.-)	FIG00138523	idu(2);Acetoin,_butanediol_metabolism idu(2);Dehydrogenase_complexes
fig|6666666.28482.peg.2370	Contig_22	81591	81818	+	2-oxoglutarate dehydrogenase, E2 component, dihydrolipoamide succinyltransferase		
fig|6666666.28482.peg.2371	Contig_22	81815	82669	+	Enoyl-CoA hydratase/isomerase		

fig|6666666.28482.peg.2372	Contig_22	82633	83646	+	Enoyl-[acyl-carrier-protein] reductase [FMN] (EC 1.3.1.9)	FIG00007641	idu(1);Fatty_Acid_Biosynthesis_FASII
fig|6666666.28482.peg.2373	Contig_22	83751	84839	+	Formaldehyde dehydrogenase MscR, NAD/mycothiol-dependent (EC 1.2.1.66) / S-nitrosomycothiol reductase MscR	FIG00013253	idu(1);Glutathione_analogs:_mycothiol icw(2);Glutathione_analogs:_mycothiol
fig|6666666.28482.peg.2374	Contig_22	84839	85117	+	Putative hydrolase in cluster with formaldehyde/S-nitrosomycothiol reductase MscR	FIG00146043	icw(2);Glutathione_analogs:_mycothiol
fig|6666666.28482.peg.2375	Contig_22	85166	85462	+	Putative hydrolase in cluster with formaldehyde/S-nitrosomycothiol reductase MscR	FIG00146043	icw(2);Glutathione_analogs:_mycothiol
fig|6666666.28482.peg.2376	Contig_22	85466	85603	+	hypothetical protein		
fig|6666666.28482.peg.2377	Contig_22	85698	87191	+	Methylmalonate-semialdehyde dehydrogenase (EC 1.2.1.27)	FIG00137372	idu(1);Valine_degradation
fig|6666666.28482.peg.2378	Contig_22	87338	87844	+	hypothetical protein		
fig|6666666.28482.peg.2379	Contig_22	87919	88635	+	3-oxoacyl-[acyl-carrier protein] reductase (EC 1.1.1.100)	FIG00621114	idu(38);CBSS-246196.1.peg.364 idu(38);CBSS-164757.7.peg.5020 idu(38);Fatty_Acid_Biosynthesis_FASII

fig|6666666.28482.peg.2380	Contig_22	90381	88753	-	Long-chain-fatty-acid--CoA ligase (EC 6.2.1.3)	FIG00018699	idu(72);n-Phenylalkanoic_acid_degradation idu(72);Fatty_acid_metabolism_cluster idu(72);Biotin_synthesis_cluster idu(72);Biotin_biosynthesis
fig|6666666.28482.peg.2381	Contig_22	91730	90882	-	hypothetical protein		
fig|6666666.28482.peg.2382	Contig_22	91860	91723	-	hypothetical protein		
fig|6666666.28482.peg.2383	Contig_22	93157	91916	-	putative cytochrome P450 hydroxylase	FIG00032437	idu(49);Nitric_oxide_synthase
fig|6666666.28482.peg.2384	Contig_22	94329	93154	-	Butyryl-CoA dehydrogenase (EC 1.3.99.2)	FIG00009149	idu(39);Isoleucine_degradation idu(39);Lysine_fermentation idu(39);Butanol_Biosynthesis idu(39);Acetyl-CoA_fermentation_to_Butyrate idu(39);Valine_degradation idu(39);Anaerobic_respiratory_reductases
fig|6666666.28482.peg.2385	Contig_22	95351	94326	-	hypothetical protein	FIG00638284	ff
fig|6666666.28482.peg.2386	Contig_22	96456	95353	-	hypothetical protein	FIG00638284	ff
fig|6666666.28482.peg.2387	Contig_22	97730	96459	-	hypothetical protein	FIG00638284	ff
fig|6666666.28482.peg.2388	Contig_22	98447	97848	-	Transcriptional regulator, TetR family		
fig|6666666.28482.peg.2389	Contig_22	99104	98655	-	hypothetical protein		
fig|6666666.28482.peg.2390	Contig_22	100327	99101	-	L-carnitine dehydratase/bile acid-inducible protein F	FIG01318850	ff
fig|6666666.28482.peg.2391	Contig_22	101956	100358	-	Choline dehydrogenase (EC 1.1.99.1)	FIG00057416	idu(4);Choline_uptake_and_conversion_to_betaine_clusters idu(4);Choline_and_Betaine_Uptake_and_Betaine_Biosynthesis
fig|6666666.28482.peg.2392	Contig_22	102509	102354	-	hypothetical protein		

fig|6666666.28482.peg.2393	Contig_22	102541	102822	+	hypothetical protein		
fig|6666666.28482.peg.2394	Contig_22	103160	103924	+	hypothetical protein		
fig|6666666.28482.peg.2395	Contig_22	104052	103921	-	hypothetical protein		
fig|6666666.28482.peg.2396	Contig_22	104157	104600	+	Protein of unknown function DUF1486		
fig|6666666.28482.peg.2397	Contig_22	104815	104687	-	hypothetical protein		
fig|6666666.28482.peg.2398	Contig_22	104951	105118	+	hypothetical protein		
fig|6666666.28482.peg.2399	Contig_22	105942	105142	-	TesB-like acyl-CoA thioesterase 4		idu(2);Acyl-CoA_thioesterase_II
fig|6666666.28482.peg.2400	Contig_22	106985	106479	-	Transcriptional regulator, TetR family		
fig|6666666.28482.peg.2401	Contig_22	108343	107339	-	PROBABLE CHOLESTEROL DEHYDROGENASE	FIG00820427	ff
fig|6666666.28482.peg.2402	Contig_22	109228	108434	-	hypothetical protein		
fig|6666666.28482.peg.2403	Contig_22	110587	109928	-	hypothetical protein		
fig|6666666.28482.peg.2404	Contig_22	111107	111781	+	hypothetical protein		
fig|6666666.28482.peg.2405	Contig_22	112030	112821	+	hypothetical protein		
fig|6666666.28482.peg.2406	Contig_22	113683	112847	-	Transcriptional regulator, IclR family		idu(12);Homogentisate_pathway_of_aromatic_compound_degradation
fig|6666666.28482.peg.2407	Contig_22	113853	115094	+	hypothetical protein	FIG00638284	ff
fig|6666666.28482.peg.2408	Contig_22	116266	115997	-	RelB/StbD replicon stabilization protein (antitoxin to RelE/StbE)	FIG01304488	ff
fig|6666666.28482.peg.2409	Contig_22	117767	116973	-	FIG00820444: hypothetical protein	FIG00820442	ff
fig|6666666.28482.peg.2410	Contig_22	118501	117764	-	FIG00822942: hypothetical protein	FIG00822941	ff
fig|6666666.28482.peg.2411	Contig_22	119779	118529	-	MCE-family protein Mce1F	FIG00821171	idu(7);Mycobacterium_virulence_operon_MCE_involved_in_cell_invasion

fig|6666666.28482.peg.2412	Contig_22	120984	119776	-	MCE-family lipoprotein LprK (MCE-family lipoprotein Mce1e)	FIG00819998	icw(3);Mycobacterium_virulence_operon_MCE_involved_in_cell_invasion
fig|6666666.28482.peg.2413	Contig_22	122102	120981	-	MCE-family protein Mce1D	FIG00820669	icw(1);Mycobacterium_virulence_operon_MCE_involved_in_cell_invasion
fig|6666666.28482.peg.2414	Contig_22	123154	122099	-	MCE-family protein Mce1C	FIG00821293	icw(7);Mycobacterium_virulence_operon_MCE_involved_in_cell_invasion
fig|6666666.28482.peg.2415	Contig_22	124208	123147	-	MCE-family protein Mce1B	FIG00763551	icw(6);Mycobacterium_virulence_operon_MCE_involved_in_cell_invasion
fig|6666666.28482.peg.2416	Contig_22	125790	124210	-	MCE-family protein Mce1A	FIG00820494	icw(2);Mycobacterium_virulence_operon_MCE_involved_in_cell_invasion
fig|6666666.28482.peg.2417	Contig_22	126656	125793	-	Conserved hypothetical integral membrane protein YrbE1B	FIG00691621	icw(3);Mycobacterium_virulence_operon_MCE_involved_in_cell_invasion
fig|6666666.28482.peg.2418	Contig_22	127497	126658	-	Conserved hypothetical integral membrane protein YrbE1A	FIG00690583	idu(7);Mycobacterium_virulence_operon_MCE_involved_in_cell_invasion
fig|6666666.28482.peg.2419	Contig_22	127801	128406	+	Transcriptional regulator, TetR family	FIG00554417	ff
fig|6666666.28482.peg.2420	Contig_22	129979	130164	+	hypothetical protein		
fig|6666666.28482.peg.2421	Contig_22	130869	130985	+	hypothetical protein		
fig|6666666.28482.peg.2422	Contig_22	132075	132797	+	site-specific recombinase, phage integrase family protein		
fig|6666666.28482.peg.2423	Contig_22	135482	132891	-	hypothetical protein	FIG00638284	ff

fig|6666666.28482.peg.2424	Contig_22	135725	135594	-	hypothetical protein		
fig|6666666.28482.peg.2425	Contig_23	1077	214	-	FIG00822559: hypothetical protein	FIG00822557	ff
fig|6666666.28482.peg.2426	Contig_23	4296	1171	-	Isoleucyl-tRNA synthetase (EC 6.1.1.5)	FIG00000085	isu;tRNA_aminoacylation,_Ile
fig|6666666.28482.peg.2427	Contig_23	4815	4585	-	Putative uncharacterized protein BCG_1587		
fig|6666666.28482.peg.2428	Contig_23	6008	5364	-	Transcriptional regulator, TetR family	FIG00554417	ff
fig|6666666.28482.peg.2429	Contig_23	7129	6005	-	2-nitropropane dioxygenase (EC 1.13.11.32)	FIG01304356	idu(5);CBSS-164757.7.peg.5020
fig|6666666.28482.peg.2430	Contig_23	7744	7169	-	Carboxymuconolactone decarboxylase	FIG00825342	ff
fig|6666666.28482.peg.2431	Contig_23	7941	7828	-	hypothetical protein		
fig|6666666.28482.peg.2432	Contig_23	8747	15013	+	Polyketide synthase	FIG00823157	ff
fig|6666666.28482.peg.2433	Contig_23	15111	16517	+	POSSIBLE CONSERVED POLYKETIDE SYNTHASE ASSOCIATED PROTEIN PAPA2	FIG01352858	ff
fig|6666666.28482.peg.2434	Contig_23	16567	17817	+	Uncharacterized protein Rv1526c/MT1577		ff
fig|6666666.28482.peg.2435	Contig_23	18616	17831	-	POSSIBLE RHAMNOSYL TRANSFERASE WBBL2	FIG01317637	ff
fig|6666666.28482.peg.2436	Contig_23	19911	18664	-	Uncharacterized glycosyltransferase Rv1524/MT1575 (EC 2.-.-.-)		ff
fig|6666666.28482.peg.2437	Contig_23	20713	19928	-	Probable methyltransferase (EC 2.1.1.-)		

fig|6666666.28482.peg.2438	Contig_23	20883	24611	+	Transmembrane transport protein MmpL12	FIG01343169	ff
fig|6666666.28482.peg.2439	Contig_23	26462	24708	-	Long-chain-fatty-acid--CoA ligase (EC 6.2.1.3)	FIG00018699	idu(72);n-Phenylalkanoic_acid_degradation idu(72);Fatty_acid_metabolism_cluster idu(72);Biotin_synthesis_cluster idu(72);Biotin_biosynthesis
fig|6666666.28482.peg.2440	Contig_23	26593	27939	+	hypothetical protein		
fig|6666666.28482.peg.2441	Contig_23	28842	27892	-	Glycosyltransferase PglI (EC 2.4.1.-)	FIG00015097	isu;N-linked_Glycosylation_in_Bacteria
fig|6666666.28482.peg.2442	Contig_23	29656	28892	-	hypothetical protein	FIG00638284	ff
fig|6666666.28482.peg.2443	Contig_23	29999	30964	+	GDP-mannose 4,6 dehydratase (EC 4.2.1.47)	FIG01318417	ff
fig|6666666.28482.peg.2444	Contig_23	30961	32058	+	mannosyltransferase		
fig|6666666.28482.peg.2445	Contig_23	32846	32055	-	hypothetical protein		
fig|6666666.28482.peg.2446	Contig_23	33113	34129	+	"GDP-mannose 4,6-dehydratase (EC 4.2.1.47)"	FIG01463199	ff
fig|6666666.28482.peg.2447	Contig_23	34987	35106	+	hypothetical protein		
fig|6666666.28482.peg.2448	Contig_23	36072	35128	-	dTDP-glucose 4,6-dehydratase (EC 4.2.1.46)	FIG00030688	idu(4);dTDP-rhamnose_synthesis idu(4);Rhamnose_containing_glycans
fig|6666666.28482.peg.2449	Contig_23	37405	36089	-	UDP-glucose dehydrogenase (EC 1.1.1.22)	FIG00000532	isu;Lipid_A-Ara4N_pathway_(_Polymyxin_resistance_)
fig|6666666.28482.peg.2450	Contig_23	37568	37771	+	hypothetical protein		
fig|6666666.28482.peg.2451	Contig_23	38259	38795	+	hypothetical protein	FIG00638284	ff

fig|6666666.28482.peg.2452	Contig_23	38917	39840	+	GDP-fucose synthetase	FIG01321732	ff
fig|6666666.28482.peg.2453	Contig_23	40121	40894	+	PGL/p-HBAD biosynthesis glycosyltransferase	FIG01358340	ff
fig|6666666.28482.peg.2454	Contig_23	42767	41148	-	hypothetical protein	FIG00638284	ff
fig|6666666.28482.peg.2455	Contig_23	44298	42796	-	Methyltransferase type 11		
fig|6666666.28482.peg.2456	Contig_23	45348	44329	-	UDP-glucose 4-epimerase (EC 5.1.3.2)	FIG00022300	idu(4);Rhamnose_containing_glycans idu(4);Lactose_and_Galactose_Uptake_and_Utilization idu(4);N-linked_Glycosylation_in_Bacteria idu(4);Lacto-N-Biose_I_and_Galacto-N-Biose_Metabolic_Pathway
fig|6666666.28482.peg.2457	Contig_23	46604	45345	-	UDP-glucose 6-dehydrogenase, UdgL	FIG00830476	ff
fig|6666666.28482.peg.2458	Contig_23	47086	47430	+	FIG00831004: hypothetical protein	FIG00831003	ff
fig|6666666.28482.peg.2459	Contig_23	48961	47516	-	Probable transmembrane protein		
fig|6666666.28482.peg.2460	Contig_23	49395	49622	+	Dodecin (COG3360) Flavin-binding		
fig|6666666.28482.peg.2461	Contig_23	50836	49619	-	Esterase LipL	FIG01388262	ff
fig|6666666.28482.peg.2462	Contig_23	50813	50989	+	hypothetical protein		
fig|6666666.28482.peg.2463	Contig_23	51944	50970	-	putative periplasmic protein kinase ArgK and related GTPases of G3E family	FIG00007966	idu(1);G3E_family_of_P-loop_GTPases_(metallocenter_biosynthesis)
fig|6666666.28482.peg.2464	Contig_23	53273	51972	-	Possible membrane protein	FIG01121868	ff

fig|6666666.28482.peg.2465	Contig_23	55728	53512	-	Methylmalonyl-CoA mutase (EC 5.4.99.2)	FIG00000927	icw(1);Serine-glyoxylate_cycle icw(2);G3E_family_of_P-loop_GTPases_(metallocenter_biosynthesis)
fig|6666666.28482.peg.2466	Contig_23	57616	55733	-	Methylmalonyl-CoA mutase, small subunit (EC 5.4.99.2)		isu;Serine-glyoxylate_cycle isu;G3E_family_of_P-loop_GTPases_(metallocenter_biosynthesis)
fig|6666666.28482.peg.2467	Contig_23	57901	58557	+	DedA family protein paralog	FIG00027618	isu;DedA_family_of_inner_membrane_proteins
fig|6666666.28482.peg.2468	Contig_23	58746	59024	+	hypothetical protein		
fig|6666666.28482.peg.2469	Contig_23	59047	59298	+	PROBABLE PROLINE AND GLYCINE RICH TRANSMEMBRANE PROTEIN		
fig|6666666.28482.peg.2470	Contig_23	60834	59368	-	Signal transduction histidine kinase	FIG01225284	ff
fig|6666666.28482.peg.2471	Contig_23	61118	60831	-	Two component system response regulator	FIG01309596	ff
fig|6666666.28482.peg.2472	Contig_23	61489	61106	-	Two component system response regulator	FIG01309596	ff
fig|6666666.28482.peg.2473	Contig_23	63003	61642	-	L-Proline/Glycine betaine transporter ProP	FIG00061305	isu;Proline,_4-hydroxyproline_uptake_and_utilization
fig|6666666.28482.peg.2474	Contig_23	63455	63099	-	FIG00822897: hypothetical protein	FIG00822896	ff
fig|6666666.28482.peg.2475	Contig_23	64608	63466	-	Putative stomatin/prohibitin-family membrane protease subunit YbbK	FIG00000531	ff

fig|6666666.28482.peg.2476	Contig_23	65071	64637	-	Putative activity regulator of membrane protease YbbK	FIG00019415	ff
fig|6666666.28482.peg.2477	Contig_23	65129	66049	+	FIG00821059: hypothetical protein	FIG00821055	ff
fig|6666666.28482.peg.2478	Contig_23	67185	66181	-	Ferrochelatase, protoheme ferro-lyase (EC 4.99.1.1)	FIG00000345	isu;Heme_and_Siroheme_Biosynthesis
fig|6666666.28482.peg.2479	Contig_23	68001	67192	-	Enoyl-[acyl-carrier-protein] reductase [NADH] (EC 1.3.1.9)	FIG00000524	icw(1);Fatty_Acid_Biosynthesis_FASII
fig|6666666.28482.peg.2480	Contig_23	68722	68036	-	3-oxoacyl-[acyl-carrier protein] reductase (EC 1.1.1.100)	FIG00621114	idu(38);CBSS-246196.1.peg.364 idu(38);CBSS-164757.7.peg.5020 idu(38);Fatty_Acid_Biosynthesis_FASII
fig|6666666.28482.peg.2481	Contig_23	69926	68919	-	Possible membrane protein	FIG01121868	ff
fig|6666666.28482.peg.2482	Contig_23	70873	69923	-	Possible membrane protein	FIG01121868	ff
fig|6666666.28482.peg.2483	Contig_23	72000	70870	-	COG0714: MoxR-like ATPases	FIG00821266	ff
fig|6666666.28482.peg.2484	Contig_23	72856	72122	-	Invasion protein	FIG01316782	ff
fig|6666666.28482.peg.2485	Contig_23	74245	72881	-	NLP/P60 family protein	FIG00012683	ff
fig|6666666.28482.peg.2486	Contig_23	75104	74565	-	Possible membrane protein	FIG01121868	ff
fig|6666666.28482.peg.2487	Contig_23	75270	78068	+	Aconitate hydratase (EC 4.2.1.3) @ 2-methylisocitrate dehydratase (EC 4.2.1.99)	FIG00138200	isu;Serine-glyoxylate_cycle isu;TCA_Cycle isu;Glyoxylate_bypass isu;Methylcitrate_cycle

fig|6666666.28482.peg.2488	Contig_23	78081	78662	+	Transcriptional regulator, TetR family	FIG00554417	ff
fig|6666666.28482.peg.2489	Contig_23	78907	78701	-	POSSIBLE TRANSCRIPTIONAL REGULATORY PROTEIN	FIG01346806	ff
fig|6666666.28482.peg.2490	Contig_23	80628	79000	-	ABC transporter ATP-binding protein	FIG01956052	ff
fig|6666666.28482.peg.2491	Contig_23	81381	80650	-	Enoyl-CoA hydratase (EC 4.2.1.17)	FIG00033206	idu(51);n-Phenylalkanoic_acid_degradation idu(51);Polyhydroxybutyrate_metabolism idu(51);Isoleucine_degradation idu(51);Fatty_acid_metabolism_cluster idu(51);Lysine_fermentation idu(51);Butanol_Biosynthesis idu(51);Acetyl-CoA_fermentation_to_Butyrate idu(51);Valine_degradation idu(51);Butyrate_metabolism_cluster
fig|6666666.28482.peg.2492	Contig_23	82197	81484	-	hypothetical protein	FIG00638284	ff
fig|6666666.28482.peg.2493	Contig_23	82538	83980	+	POSSIBLE CONSERVED POLYKETIDE SYNTHASE ASSOCIATED PROTEIN PAPA2	FIG01352858	ff
fig|6666666.28482.peg.2494	Contig_23	84336	83977	-	Thioredoxin TrxB1	FIG01340204	ff
fig|6666666.28482.peg.2495	Contig_23	84544	85623	+	hypothetical protein	FIG00638284	ff

fig|6666666.28482.peg.2496	Contig_23	85709	87538	+	3-methylmercaptopropionyl-CoA dehydrogenase (DmdC)	FIG00802415	ff
fig|6666666.28482.peg.2497	Contig_23	87610	88386	+	FIG00826949: hypothetical protein	FIG00826946	ff
fig|6666666.28482.peg.2498	Contig_23	88465	89211	+	FIG00828798: hypothetical protein	FIG00828796	ff
fig|6666666.28482.peg.2499	Contig_23	89574	89227	-	PaaD-like protein (DUF59) involved in Fe-S cluster assembly	FIG00009637	isu;Iron-sulfur_cluster_assembly
fig|6666666.28482.peg.2500	Contig_23	90032	89571	-	Putative iron-sulfur cluster assembly scaffold protein for SUF system, SufE2	FIG00000306	icw(1);Iron-sulfur_cluster_assembly
fig|6666666.28482.peg.2501	Contig_23	91270	90035	-	Cysteine desulfurase (EC 2.8.1.7), SufS subfamily	FIG00229199	icw(5);Iron-sulfur_cluster_assembly isu;mnm5U34_biosynthesis_bacteria isu;Alanine_biosynthesis
fig|6666666.28482.peg.2502	Contig_23	92054	91272	-	Iron-sulfur cluster assembly ATPase protein SufC	FIG00018727	icw(4);Iron-sulfur_cluster_assembly icw(3);CBSS-196164.1.peg.1690
fig|6666666.28482.peg.2503	Contig_23	93208	92051	-	Iron-sulfur cluster assembly protein SufD	FIG00072797	icw(3);Iron-sulfur_cluster_assembly icw(2);CBSS-196164.1.peg.1690
fig|6666666.28482.peg.2504	Contig_23	94647	93205	-	Iron-sulfur cluster assembly protein SufB	FIG00000451	icw(6);Iron-sulfur_cluster_assembly icw(2);CBSS-196164.1.peg.1690 isu;Staphylococcal_phi-Mu50B-like_prophages
fig|6666666.28482.peg.2505	Contig_23	95363	94644	-	Iron-sulfur cluster regulator SufR	FIG00003245	isu;Iron-sulfur_cluster_assembly icw(3);CBSS-196164.1.peg.1690

fig|6666666.28482.peg.2506	Contig_23	95492	97192	+	POSSIBLE CONSERVED INTEGRAL MEMBRANE PROTEIN	FIG01037873	ff
fig|6666666.28482.peg.2507	Contig_23	97222	98142	+	ABC-type multidrug transport system, ATPase component	FIG00001005	icw(1);CBSS-196164.1.peg.1690
fig|6666666.28482.peg.2508	Contig_23	98139	98930	+	ABC-type multidrug transport system, permease component		isu;CBSS-196164.1.peg.1690
fig|6666666.28482.peg.2509	Contig_23	99061	100014	+	Cytochrome oxidase assembly protein	FIG01121947	ff
fig|6666666.28482.peg.2510	Contig_23	100568	101599	+	Quinone oxidoreductase (EC 1.6.5.5)	FIG00002982	idu(7);Quinone_oxidoreductase_family
fig|6666666.28482.peg.2511	Contig_23	102629	101748	-	Heme O synthase, protoheme IX farnesyltransferase (EC 2.5.1.-) COX10-CtaB	FIG00000497	isu;CBSS-316057.3.peg.563 icw(2);CBSS-196164.1.peg.1690 isu;Biogenesis_of_cytochrome_c_oxidases
fig|6666666.28482.peg.2512	Contig_23	102931	105021	+	Transketolase (EC 2.2.1.1)	FIG00020289	isu;Pentose_phosphate_pathway
fig|6666666.28482.peg.2513	Contig_23	105037	106158	+	Transaldolase (EC 2.2.1.2)	FIG00000023	isu;Fructose_utilization icw(2);Pentose_phosphate_pathway
fig|6666666.28482.peg.2514	Contig_23	106155	107717	+	Glucose-6-phosphate 1-dehydrogenase (EC 1.1.1.49)	FIG00000332	icw(1);Pentose_phosphate_pathway
fig|6666666.28482.peg.2515	Contig_23	107767	108678	+	OpcA, an allosteric effector of glucose-6-phosphate dehydrogenase, actinobacterial	FIG00018823	ff
fig|6666666.28482.peg.2516	Contig_24	22	1884	+	hypothetical protein	FIG00638284	ff

fig|6666666.28482.peg.2517	Contig_24	3274	3101	-	SMALL SECRETED PROTEIN	FIG01361580	ff
fig|6666666.28482.peg.2518	Contig_24	3548	3348	-	conserved hypothetical small secreted protein		
fig|6666666.28482.peg.2519	Contig_24	3648	4793	+	Beta-hexosaminidase (EC 3.2.1.52)	FIG00001088	isu;N-Acetyl-Galactosamine_and_Galactosamine_Utilization isu;Chitin_and_N-acetylglucosamine_utilization
fig|6666666.28482.peg.2520	Contig_24	4849	5469	+	Transcriptional regulator, TetR family	FIG00554417	ff
fig|6666666.28482.peg.2521	Contig_24	5495	6739	+	Chloride channel protein	FIG00731882	ff
fig|6666666.28482.peg.2522	Contig_24	7584	6736	-	Acyl dehydratase	FIG01303815	idu(2);CBSS-246196.1.peg.364
fig|6666666.28482.peg.2523	Contig_24	8945	7581	-	3-oxoacyl-[acyl-carrier protein] reductase (EC 1.1.1.100)	FIG00621114	icw(1);CBSS-246196.1.peg.364 idu(38);CBSS-164757.7.peg.5020 idu(38);Fatty_Acid_Biosynthesis_FASII

fig|6666666.28482.peg.2524	Contig_24	9096	10409	+	3-ketoacyl-CoA thiolase (EC 2.3.1.16) @ Acetyl-CoA acetyltransferase (EC 2.3.1.9)	FIG00000004	icw(2);CBSS-246196.1.peg.364 icw(1);Serine-glyoxylate_cycle icw(1);Serine-glyoxylate_cycle idu(11);n-Phenylalkanoic_acid_degradation idu(9);Ethylmalonyl-CoA_pathway_of_C2_assimilation,_GJO idu(9);Polyhydroxybutyrate_metabolism idu(11);Polyhydroxybutyrate_metabolism idu(11);Isoleucine_degradation idu(11);Fatty_acid_metabolism_cluster idu(11);Biotin_synthesis_cluster idu(11);Biotin_biosynthesis idu(9);Lysine_fermentation idu(9);Isoprenoid_Biosynthesis idu(9);Butanol_Biosynthesis idu(9);Acetyl-CoA_fermentation_to_Butyrate idu(9);Butyrate_metabolism_cluster
fig|6666666.28482.peg.2525	Contig_24	12545	10710	-	3-methylmercaptopropionyl-CoA dehydrogenase (DmdC)	FIG00802415	ff
fig|6666666.28482.peg.2526	Contig_24	12736	13779	+	2-Oxobutyrate oxidase, putative	FIG00003889	ff

fig|6666666.28482.peg.2527	Contig_24	13776	14273	+	Nitrilotriacetate monooxygenase component B (EC 1.14.13.-)	FIG00039067	idu(1);Aromatic_Amin_Catabolism
fig|6666666.28482.peg.2528	Contig_24	15102	14353	-	Succinate dehydrogenase iron-sulfur protein (EC 1.3.99.1)	FIG00026006	icw(1);Serine-glyoxylate_cycle icw(1);TCA_Cycle idu(1);Succinate_dehydrogenase
fig|6666666.28482.peg.2529	Contig_24	17030	15102	-	Succinate dehydrogenase flavoprotein subunit (EC 1.3.99.1)	FIG00136922	idu(1);Serine-glyoxylate_cycle idu(1);TCA_Cycle icw(2);Succinate_dehydrogenase
fig|6666666.28482.peg.2530	Contig_24	17804	17073	-	putative succinate dehydrogenase [membrane anchor subunit] (succinic dehydrogenase)	FIG00010624	icw(1);Succinate_dehydrogenase
fig|6666666.28482.peg.2531	Contig_24	18286	17990	-	FIG00820825: hypothetical protein	FIG00820824	ff
fig|6666666.28482.peg.2532	Contig_24	18364	19134	+	Probable carboxyvinyl-carboxyphosphonate phosphorylmutase (EC 2.7.8.23)	FIG00012387	ff
fig|6666666.28482.peg.2533	Contig_24	19641	19201	-	Heat shock protein hsp	FIG01344888	ff
fig|6666666.28482.peg.2534	Contig_24	19856	22420	+	Nitrite reductase [NAD(P)H] large subunit (EC 1.7.1.4)	FIG00000873	icw(2);Nitrate_and_nitrite_ammonification
fig|6666666.28482.peg.2535	Contig_24	22434	22799	+	Nitrite reductase [NAD(P)H] small subunit (EC 1.7.1.4)	FIG00138942	icw(1);Nitrate_and_nitrite_ammonification
fig|6666666.28482.peg.2536	Contig_24	23194	22796	-	FIG00825710: hypothetical protein	FIG00825709	ff

fig|6666666.28482.peg.2537	Contig_24	24005	23277	-	Sirohydrochlorin ferrochelatase (EC 4.99.1.4)	FIG00132864	idu(2);Heme_and_Siroheme_Biosynthesis
fig|6666666.28482.peg.2538	Contig_24	25147	23996	-	Bifunctional uroporphyrinogen-III synthetase/response regulator domain protein	FIG00816213	ff
fig|6666666.28482.peg.2539	Contig_24	26607	25225	-	Nitrate/nitrite transporter	FIG00005529	idu(2);Nitrate_and_nitrite_ammonification
fig|6666666.28482.peg.2540	Contig_24	27388	26843	-	Aminoglycoside 2'-N-acetyltransferase AAC (AAC(2')-IC)	FIG01346537	ff
fig|6666666.28482.peg.2541	Contig_24	28274	27390	-	Allophanate hydrolase 2 subunit 2 (EC 3.5.1.54)	FIG00010262	icw(1);Urea_carboxylase_and_Allophanate_hydrolase_cluster icw(1);EC699-706
fig|6666666.28482.peg.2542	Contig_24	28963	28289	-	Allophanate hydrolase 2 subunit 1 (EC 3.5.1.54)	FIG00000847	isu;Urea_carboxylase_and_Allophanate_hydrolase_cluster isu;EC699-706
fig|6666666.28482.peg.2543	Contig_24	30006	29065	-	ABC transporter, periplasmic iron-transport lipoprotein	FIG01312725	ff
fig|6666666.28482.peg.2544	Contig_24	31359	30109	-	ATP-dependent DNA ligase (EC 6.5.1.1)	FIG00052980	idu(2);DNA_replication,_archaeal idu(2);DNA_Repair_Base_Excision idu(2);DNA_ligases
fig|6666666.28482.peg.2545	Contig_24	31410	33098	+	Long-chain-fatty-acid--CoA ligase (EC 6.2.1.3)	FIG00018699	idu(72);n-Phenylalkanoic_acid_degradation idu(72);Fatty_acid_metabolism_cluster idu(72);Biotin_synthesis_cluster idu(72);Biotin_biosynthesis

fig|6666666.28482.peg.2546	Contig_24	33444	33109	-	FIG00825611: hypothetical protein	FIG00825609	ff
fig|6666666.28482.peg.2547	Contig_25	1532	360	-	FIG00822682: hypothetical protein	FIG00822681	ff
fig|6666666.28482.peg.2548	Contig_25	1602	2210	+	Transcriptional regulator, TetR family	FIG00554417	ff
fig|6666666.28482.peg.2549	Contig_25	2634	2221	-	FIG00822364: hypothetical protein	FIG00822363	ff
fig|6666666.28482.peg.2550	Contig_25	3099	2710	-	secreted protein antigen		
fig|6666666.28482.peg.2551	Contig_25	3653	4132	+	hypothetical protein		
fig|6666666.28482.peg.2552	Contig_25	4561	4133	-	hypothetical protein		
fig|6666666.28482.peg.2553	Contig_25	4938	4651	-	Histone acetyltransferase HPA2 and related acetyltransferases		idu(1);CBSS-216591.1.peg.168
fig|6666666.28482.peg.2554	Contig_25	6205	5132	-	GTP-binding and nucleic acid-binding protein YchF	FIG00049915	ff
fig|6666666.28482.peg.2555	Contig_25	6335	6493	+	hypothetical protein		
fig|6666666.28482.peg.2556	Contig_25	7279	6686	-	hypothetical protein		
fig|6666666.28482.peg.2557	Contig_25	8355	7465	-	4-hydroxy-3-methylbut-2-enyl diphosphate reductase (EC 1.17.1.2)	FIG00000381	isu;Nonmevalonate_Branch_of_Isoprenoid_Biosynthesis isu;Isoprenoid_Biosynthesis
fig|6666666.28482.peg.2558	Contig_25	8538	9128	+	FIG00997309: hypothetical protein	FIG00821685	ff
fig|6666666.28482.peg.2559	Contig_25	9176	10378	+	Exodeoxyribonuclease VII large subunit (EC 3.1.11.6)	FIG00000323	isu;Purine_salvage_cluster isu;DNA_repair,_bacterial
fig|6666666.28482.peg.2560	Contig_25	10452	10610	+	Exodeoxyribonuclease VII small subunit (EC 3.1.11.6)	FIG00000435	icw(1);Purine_salvage_cluster icw(1);DNA_repair,_bacterial

fig|6666666.28482.peg.2561	Contig_25	10685	11770	+	PROBABLE CHOLESTEROL DEHYDROGENASE	FIG00820427	ff
fig|6666666.28482.peg.2562	Contig_25	12365	11802	-	Transcriptional regulator, TetR family	FIG00554417	ff
fig|6666666.28482.peg.2563	Contig_25	14028	12493	-	Carboxylesterase, type B	FIG01336027	ff
fig|6666666.28482.peg.2564	Contig_25	14805	14044	-	Dienelactone hydrolase family protein	FIG00824265	ff
fig|6666666.28482.peg.2565	Contig_25	15539	14856	-	FIG00820022: hypothetical protein	FIG00820021	ff
fig|6666666.28482.peg.2566	Contig_25	15596	16630	+	Fructose-1,6-bisphosphatase, GlpX type (EC 3.1.3.11)	FIG00138413	ff
fig|6666666.28482.peg.2567	Contig_25	16667	18082	+	Fumarate hydratase class II (EC 4.2.1.2)	FIG00000340	isu;TCA_Cycle
fig|6666666.28482.peg.2568	Contig_25	18100	19089	+	PROBABLE MEMBRANE GLYCINE AND PROLINE RICH PROTEIN	FIG01270577	ff
fig|6666666.28482.peg.2569	Contig_25	20533	19079	-	Adenylate cyclase (EC 4.6.1.1)	FIG00001294	idu(25);cAMP_signaling_in_bacteria
fig|6666666.28482.peg.2570	Contig_25	21519	20644	-	Possible glycosyl hydrolase (EC 3.-.-.-)	FIG01314096	ff
fig|6666666.28482.peg.2571	Contig_25	22954	21653	-	Predicted ATPase related to phosphate starvation-inducible protein PhoH	FIG00075426	isu;Phosphate_metabolism
fig|6666666.28482.peg.2572	Contig_25	24043	23216	-	Fatty acid desaturase occurring in virulence cluster	FIG00996037	idu(2);Mycobacterium_virulence_operon_involved_in_lipid_metabolism

fig|6666666.28482.peg.2573	Contig_25	25448	24129	-	Serine hydroxymethyltransferase 1 (EC 2.1.2.1)	FIG00147090	isu;Serine_Biosynthesis
fig|6666666.28482.peg.2574	Contig_25	25592	26464	+	Pantothenate kinase (EC 2.7.1.33)	FIG00000936	isu;Coenzyme_A_Biosynthesis
fig|6666666.28482.peg.2575	Contig_25	26902	26486	-	FIG00820427: hypothetical protein	FIG00820425	ff
fig|6666666.28482.peg.2576	Contig_25	28485	27106	-	FIG00821548: hypothetical protein	FIG00821546	ff
fig|6666666.28482.peg.2577	Contig_25	29316	28528	-	Undecaprenyl pyrophosphate synthetase (EC 2.5.1.31)	FIG00000110	ff
fig|6666666.28482.peg.2578	Contig_25	29453	30205	+	COG1272: Predicted membrane protein hemolysin III homolog	FIG01323667	ff
fig|6666666.28482.peg.2579	Contig_25	30597	30214	-	Steroid delta-isomerase (EC 5.3.3.1)	FIG01310988	ff
fig|6666666.28482.peg.2580	Contig_25	32635	30620	-	Thymidylate kinase (EC 2.7.4.9)	FIG00000175	idu(1);pyrimidine_conversions idu(1);CBSS-393133.3.peg.2787
fig|6666666.28482.peg.2581	Contig_25	32888	32622	-	probable secreted protein	FIG00999229	ff
fig|6666666.28482.peg.2582	Contig_25	33757	32885	-	Mycothiol S-conjugate amidase Mca	FIG00006621	isu;Glutathione_analogs:_mycothiol
fig|6666666.28482.peg.2583	Contig_25	33758	33898	+	hypothetical protein		
fig|6666666.28482.peg.2584	Contig_25	33895	34329	+	FIG00821098: hypothetical protein	FIG00821096	ff
fig|6666666.28482.peg.2585	Contig_25	34497	35045	+	Transcription elongation factor GreA	FIG00000288	isu;Transcription_factors_bacterial
fig|6666666.28482.peg.2586	Contig_25	36284	35118	-	Cystathionine gamma-lyase (EC 4.4.1.1)	FIG00001346	idu(1);Cysteine_Biosynthesis

fig|6666666.28482.peg.2587	Contig_25	36383	36823	+	putative membrane protein	FIG00672847	ff
fig|6666666.28482.peg.2588	Contig_25	37439	36786	-	Proline-rich antigen homolog	FIG01375448	ff
fig|6666666.28482.peg.2589	Contig_25	38927	37521	-	Cystathionine beta-synthase (EC 4.2.1.22)	FIG00022427	icw(1);Cysteine_Biosynthesis
fig|6666666.28482.peg.2590	Contig_25	39969	39082	-	esterase	FIG01380254	ff
fig|6666666.28482.peg.2591	Contig_25	40393	41370	+	CONSERVED EXPORTED PROTEIN	FIG00815422	ff
fig|6666666.28482.peg.2592	Contig_25	41446	42663	+	3-ketoacyl-CoA thiolase (EC 2.3.1.16) @ Acetyl-CoA acetyltransferase (EC 2.3.1.9)	FIG00000004	idu(11);CBSS-246196.1.peg.364 idu(9);Serine-glyoxylate_cycle idu(11);Serine-glyoxylate_cycle idu(11);n-Phenylalkanoic_acid_degradation idu(9);Ethylmalonyl-CoA_pathway_of_C2_assimilation,_GJO idu(9);Polyhydroxybutyrate_metabolism idu(11);Polyhydroxybutyrate_metabolism idu(11);Isoleucine_degradation idu(11);Fatty_acid_metabolism_cluster idu(11);Biotin_synthesis_cluster idu(11);Biotin_biosynthesis idu(9);Lysine_fermentation idu(9);Isoprenoid_Biosynthesis idu(9);Butanol_Biosynthesis idu(9);Acetyl-CoA_fermentation_to_Butyrate idu(9);Butyrate_metabolism_cluster

fig|6666666.28482.peg.2593	Contig_25	43674	42781	-	putative sigma factor		
fig|6666666.28482.peg.2594	Contig_25	44831	43671	-	NADH dehydrogenase (EC 1.6.99.3)	FIG00008708	idu(6);Respiratory_dehydrogenases_1 idu(6);PPE_gene_cluster_in_Mycobacteria idu(6);Riboflavin_synthesis_cluster
fig|6666666.28482.peg.2595	Contig_25	45803	44967	-	PROBABLE CONSERVED TRANSMEMBRANE PROTEIN	FIG00662132	ff
fig|6666666.28482.peg.2596	Contig_25	46037	47083	+	3-hydroxyisobutyryl-CoA hydrolase (EC 3.1.2.4)	FIG00133111	isu;Valine_degradation
fig|6666666.28482.peg.2597	Contig_25	47097	47876	+	Enoyl-CoA hydratase [valine degradation] (EC 4.2.1.17)	FIG00014562	icw(1);Valine_degradation
fig|6666666.28482.peg.2598	Contig_25	47886	49625	+	Predicted membrane protein (DUF2319)	FIG01329746	ff
fig|6666666.28482.peg.2599	Contig_26	1241	198	-	N5,N10-methylenetetrahydromethanopterin reductase-related protein	FIG01460162	ff
fig|6666666.28482.peg.2600	Contig_26	1637	1287	-	FIG00824417: hypothetical protein	FIG00824416	ff
fig|6666666.28482.peg.2601	Contig_26	2119	1691	-	Transcriptional regulator, MarR family	FIG00554610	ff
fig|6666666.28482.peg.2602	Contig_26	2215	2880	+	Methyltransferase type 11		
fig|6666666.28482.peg.2603	Contig_26	3639	2893	-	FIG00996653: hypothetical protein	FIG00821869	ff

fig|6666666.28482.peg.2604	Contig_26	3964	3758	-	Putative uncharacterized protein BCG_1587		
fig|6666666.28482.peg.2605	Contig_26	4459	6336	+	Glucosamine--fructose-6-phosphate aminotransferase [isomerizing] (EC 2.6.1.16)	FIG00000038	isu;Sialic_Acid_Metabolism isu;UDP-N-acetylmuramate_from_Fructose-6-phosphate_Biosynthesis
fig|6666666.28482.peg.2606	Contig_26	6427	7257	+	PROBABLE CONSERVED TRANSMEMBRANE PROTEIN	FIG00662132	ff
fig|6666666.28482.peg.2607	Contig_26	7293	8009	+	POSSIBLE CONSERVED TRANSMEMBRANE PROTEIN	FIG01417063	ff
fig|6666666.28482.peg.2608	Contig_26	8013	9863	+	Acylamino-acid-releasing enzyme	FIG01330763	ff
fig|6666666.28482.peg.2609	Contig_26	9881	11299	+	NAD(P)HX epimerase / NAD(P)HX dehydratase	FIG00000592	isu;YjeE isu;YjeE
fig|6666666.28482.peg.2610	Contig_26	11320	12705	+	Glutamate decarboxylase (EC 4.1.1.15)	FIG01376814	ff
fig|6666666.28482.peg.2611	Contig_26	12869	14056	+	Alanine racemase (EC 5.1.1.1)	FIG00001642	isu;Pyruvate_Alanine_Serine_Interconversions isu;Alanine_biosynthesis
fig|6666666.28482.peg.2612	Contig_26	14109	15161	+	Hydrolase (HAD superfamily)	FIG00510207	ff
fig|6666666.28482.peg.2613	Contig_26	15154	15681	+	ATPase YjeE, predicted to have essential role in cell wall biosynthesis	FIG00000228	ff

fig|6666666.28482.peg.2614	Contig_26	15678	16298	+	Inactive homolog of metal-dependent proteases, putative molecular chaperone	FIG00000247	ff
fig|6666666.28482.peg.2615	Contig_26	16295	16762	+	Ribosomal-protein-S18p-alanine acetyltransferase (EC 2.3.1.-)	FIG00122145	ff
fig|6666666.28482.peg.2616	Contig_26	16759	17781	+	YgjD/Kae1/Qri7 family, required for threonylcarbamoyladenosine (t(6)A) formation in tRNA	FIG00134348	ff
fig|6666666.28482.peg.2617	Contig_26	18033	18335	+	Heat shock protein 60 family co-chaperone GroES	FIG00009229	isu;GroEL_GroES
fig|6666666.28482.peg.2618	Contig_26	18413	20032	+	Heat shock protein 60 family chaperone GroEL	FIG00000056	idu(1);Staphylococcal_pathogenicity_islands_SaPI icw(1);GroEL_GroES
fig|6666666.28482.peg.2619	Contig_26	21108	20092	-	Alcohol dehydrogenase (EC 1.1.1.1)	FIG01007502	idu(17);Glycerolipid_and_Glycerophospholipid_Metabolism_in_Bacteria idu(17);Fermentations:_Mixed_acid idu(17);Butanol_Biosynthesis
fig|6666666.28482.peg.2620	Contig_26	21210	21779	+	Transcriptional regulator, TetR family		
fig|6666666.28482.peg.2621	Contig_26	21982	23367	+	Adenylate cyclase (EC 4.6.1.1)	FIG00001294	icw(1);cAMP_signaling_in_bacteria
fig|6666666.28482.peg.2622	Contig_26	23455	24957	+	Adenylate cyclase (EC 4.6.1.1)	FIG00001294	icw(1);cAMP_signaling_in_bacteria
fig|6666666.28482.peg.2623	Contig_26	25052	26152	+	FIG01123205: hypothetical protein	FIG01123204	ff

fig|6666666.28482.peg.2624	Contig_26	26886	26158	-	Superoxide dismutase [Cu-Zn] precursor (EC 1.15.1.1)	FIG00001181	idu(2);Oxidative_stress idu(2);Protection_from_Reactive_Oxygen_Species
fig|6666666.28482.peg.2625	Contig_26	26992	27270	+	hypothetical protein		
fig|6666666.28482.peg.2626	Contig_26	27457	27275	-	hypothetical protein		
fig|6666666.28482.peg.2627	Contig_26	27544	27669	+	hypothetical protein		
fig|6666666.28482.peg.2628	Contig_26	28033	27725	-	Sporulation regulatory protein WhiD	FIG00036805	isu;WhiB_and_WhiB-type_regulatory_proteins_
fig|6666666.28482.peg.2629	Contig_26	28399	29199	+	FIG00996157: hypothetical protein	FIG00822850	ff
fig|6666666.28482.peg.2630	Contig_26	29281	29856	+	RNA polymerase sigma-70 factor	FIG01303902	idu(4);CBSS-313593.3.peg.2729 idu(4);Transcription_initiation,_bacterial_sigma_factors
fig|6666666.28482.peg.2631	Contig_26	29849	30607	+	FIG00997706: hypothetical protein	FIG00821000	ff
fig|6666666.28482.peg.2632	Contig_26	31019	30609	-	FIG00820793: hypothetical protein	FIG00820792	ff
fig|6666666.28482.peg.2633	Contig_26	31160	32773	+	Inosine-5'-monophosphate dehydrogenase (EC 1.1.1.205)	FIG00020613	icw(1);Purine_conversions icw(1);Purine_salvage_cluster
fig|6666666.28482.peg.2634	Contig_26	32788	33915	+	Inosine-5'-monophosphate dehydrogenase (EC 1.1.1.205)	FIG00020613	icw(1);Purine_conversions icw(1);Purine_salvage_cluster
fig|6666666.28482.peg.2635	Contig_26	33967	35703	+	Cholesterol oxidase (EC 1.1.3.6)	FIG00817191	ff
fig|6666666.28482.peg.2636	Contig_26	36618	35713	-	Alpha-ketoglutarate-dependent taurine dioxygenase (EC 1.14.11.17)	FIG00001391	idu(1);Alkanesulfonate_assimilation
fig|6666666.28482.peg.2637	Contig_26	36753	37262	+	Transcriptional regulator, TetR family	FIG00554417	ff

fig|6666666.28482.peg.2638	Contig_26	39622	37256	-	Maltose phosphorylase (EC 2.4.1.8) / Trehalose phosphorylase (EC 2.4.1.64)	FIG00043765	isu;Trehalose_Uptake_and_Utilization isu;Trehalose_Biosynthesis isu;Maltose_and_Maltodextrin_Utilization
fig|6666666.28482.peg.2639	Contig_26	40359	39619	-	Beta-phosphoglucomutase (EC 5.4.2.6)	FIG00796613	isu;N-Acetyl-Galactosamine_and_Galactosamine_Utilization icw(1);Trehalose_Uptake_and_Utilization icw(1);Maltose_and_Maltodextrin_Utilization
fig|6666666.28482.peg.2640	Contig_26	41073	40462	-	Transcriptional regulator, TetR family	FIG00554417	ff
fig|6666666.28482.peg.2641	Contig_26	41183	42304	+	hypothetical protein	FIG00638284	ff
fig|6666666.28482.peg.2642	Contig_26	42781	42275	-	hypothetical protein	FIG00638284	ff
fig|6666666.28482.peg.2643	Contig_26	43180	42785	-	hypothetical protein		
fig|6666666.28482.peg.2644	Contig_26	44061	43177	-	hydrolase, alpha/beta fold family		
fig|6666666.28482.peg.2645	Contig_26	44870	44067	-	hypothetical protein	FIG00638284	ff
fig|6666666.28482.peg.2646	Contig_26	45691	44867	-	hypothetical protein	FIG00638284	ff
fig|6666666.28482.peg.2647	Contig_26	47267	45723	-	FIG00824483: hypothetical protein	FIG00824482	ff
fig|6666666.28482.peg.2648	Contig_26	47362	47997	+	Transcriptional regulator, HxlR family	FIG00906075	ff
fig|6666666.28482.peg.2649	Contig_26	48102	48980	+	PPE family protein	FIG00013075	idu(57);CBSS-164757.7.peg.5020
fig|6666666.28482.peg.2650	Contig_26	49497	48985	-	hypothetical protein		
fig|6666666.28482.peg.2651	Contig_26	50597	49494	-	putative cytochrome P450 hydroxylase	FIG00032437	idu(49);Nitric_oxide_synthase
fig|6666666.28482.peg.2652	Contig_26	50669	51277	+	Transcriptional regulator, TetR family	FIG00554417	ff
fig|6666666.28482.peg.2653	Contig_26	51803	51240	-	Transcriptional regulator, TetR family		

fig|6666666.28482.peg.2654	Contig_26	51945	53141	+	Acyl-CoA dehydrogenase, type 2, C-terminal domain		
fig|6666666.28482.peg.2655	Contig_26	53709	53149	-	Transcriptional regulator, TetR family		
fig|6666666.28482.peg.2656	Contig_26	54591	53755	-	oxidoreductase, short chain dehydrogenase/reductase family		
fig|6666666.28482.peg.2657	Contig_26	55402	54605	-	short-chain dehydrogenase/reductase SDR		
fig|6666666.28482.peg.2658	Contig_26	55597	56025	+	membrane protein, MmpS family	FIG01309473	ff
fig|6666666.28482.peg.2659	Contig_26	56022	58862	+	Putative membrane protein	FIG01056920	ff
fig|6666666.28482.peg.2660	Contig_26	58859	59236	+	hypothetical protein		
fig|6666666.28482.peg.2661	Contig_26	59310	60845	+	GMP synthase [glutamine-hydrolyzing] (EC 6.3.5.2)	FIG00042267	isu;Purine_conversions isu;Staphylococcal_pathogenicity_islands_SaPI
fig|6666666.28482.peg.2662	Contig_26	61484	60855	-	Transcriptional regulator, TetR family	FIG00554417	ff
fig|6666666.28482.peg.2663	Contig_26	61604	62332	+	Short-chain dehydrogenase/reductase SDR		idu(13);CBSS-164757.7.peg.5020 idu(13);Transcription_repair_cluster
fig|6666666.28482.peg.2664	Contig_26	62397	63278	+	NmrA family protein		
fig|6666666.28482.peg.2665	Contig_26	63308	63721	+	hypothetical protein		
fig|6666666.28482.peg.2666	Contig_26	64309	63722	-	PROBABLE MEMBRANE PROTEIN	FIG00816212	ff
fig|6666666.28482.peg.2667	Contig_26	64399	65055	+	FIG00821108: hypothetical protein	FIG00821107	ff

fig|6666666.28482.peg.2668	Contig_26	65061	66620	+	DNA polymerase-like protein PA0670	FIG00027620	ff
fig|6666666.28482.peg.2669	Contig_26	66770	66639	-	hypothetical protein		
fig|6666666.28482.peg.2670	Contig_26	67896	66949	-	Inosine-uridine preferring nucleoside hydrolase (EC 3.2.2.1)	FIG00000527	isu;Purine_conversions
fig|6666666.28482.peg.2671	Contig_26	68653	67907	-	FIG00826842: hypothetical protein	FIG00826839	ff
fig|6666666.28482.peg.2672	Contig_26	70648	68696	-	Oxidoreductase, short-chain dehydrogenase/reductase family	FIG00822305	ff
fig|6666666.28482.peg.2673	Contig_26	71369	70665	-	Lipoprotein LpqD	FIG00820321	ff
fig|6666666.28482.peg.2674	Contig_27	22	150	+	hypothetical protein		
fig|6666666.28482.peg.2675	Contig_27	192	461	+	conserved hypothetical protein		
fig|6666666.28482.peg.2676	Contig_27	3368	468	-	putative LuxR-family transcriptional regulator		
fig|6666666.28482.peg.2677	Contig_27	3526	4917	+	oxidoreductase, FAD-dependent	FIG01896183	ff
fig|6666666.28482.peg.2678	Contig_27	4971	5228	+	hypothetical protein		
fig|6666666.28482.peg.2679	Contig_27	5980	5234	-	FIG00828461: hypothetical protein	FIG00828458	ff
fig|6666666.28482.peg.2680	Contig_27	6108	7289	+	FIG00828381: hypothetical protein	FIG00828378	ff
fig|6666666.28482.peg.2681	Contig_27	8053	7286	-	hypothetical protein		
fig|6666666.28482.peg.2682	Contig_27	8908	8054	-	FIG00825125: hypothetical protein	FIG00825124	ff
fig|6666666.28482.peg.2683	Contig_27	10487	9096	-	FIG00825560: hypothetical protein	FIG00825558	ff

fig|6666666.28482.peg.2684	Contig_27	12764	10569	-	Lead, cadmium, zinc and mercury transporting ATPase (EC 3.6.3.3) (EC 3.6.3.5); Copper-translocating P-type ATPase (EC 3.6.3.4)	FIG00658111	idu(1);CBSS-196620.1.peg.2477 idu(1);Copper_homeostasis
fig|6666666.28482.peg.2685	Contig_27	12973	12761	-	Copper chaperone	FIG00005595	icw(1);Copper_homeostasis
fig|6666666.28482.peg.2686	Contig_27	14130	13084	-	hypothetical protein		
fig|6666666.28482.peg.2687	Contig_27	15003	14182	-	FIG00832108: hypothetical protein	FIG00832106	ff
fig|6666666.28482.peg.2688	Contig_27	15114	15587	+	FIG00827934: hypothetical protein	FIG00827933	ff
fig|6666666.28482.peg.2689	Contig_27	16385	17392	+	FIG00829144: hypothetical protein	FIG00829140	ff
fig|6666666.28482.peg.2690	Contig_27	17446	19233	+	Cyclohexanone monooxygenase (EC 1.14.13.22)	FIG00975774	ff
fig|6666666.28482.peg.2691	Contig_27	19230	20846	+	Cyclohexanone monooxygenase (EC 1.14.13.22)	FIG00975774	ff
fig|6666666.28482.peg.2692	Contig_27	22258	20858	-	hypothetical protein	FIG00638284	ff
fig|6666666.28482.peg.2693	Contig_27	22404	23483	+	Sulfate and thiosulfate binding protein CysP	FIG00001000	idu(2);Cysteine_Biosynthesis idu(2);Inorganic_Sulfur_Assimilation
fig|6666666.28482.peg.2694	Contig_27	23503	25242	+	Sulfate permease	FIG00005216	icw(1);Cysteine_Biosynthesis
fig|6666666.28482.peg.2695	Contig_27	26085	25246	-	Short chain dehydrogenase	FIG01310822	ff
fig|6666666.28482.peg.2696	Contig_27	27438	26140	-	FIG00821664: hypothetical protein	FIG00821663	ff
fig|6666666.28482.peg.2697	Contig_27	28689	27871	-	Short-chain dehydrogenase/reductase SDR precursor	FIG00917812	ff

fig|6666666.28482.peg.2698	Contig_27	29190	28690	-	FIG00828325: hypothetical protein	FIG00828324	ff
fig|6666666.28482.peg.2699	Contig_27	29311	30171	+	Transcriptional regulator, IclR family	FIG00022569	idu(12);Homogentisate_pathway_of_aromatic_compound_degradation
fig|6666666.28482.peg.2700	Contig_27	30851	30279	-	hypothetical protein		
fig|6666666.28482.peg.2701	Contig_27	30982	31938	+	transcriptional regulator, LysR family		
fig|6666666.28482.peg.2702	Contig_27	32031	33437	+	2-methylcitrate dehydratase (EC 4.2.1.79)	FIG00001287	idu(1);Methylcitrate_cycle
fig|6666666.28482.peg.2703	Contig_27	33493	34257	+	POSSIBLE MEMBRANE PROTEIN	FIG00692835	ff
fig|6666666.28482.peg.2704	Contig_27	34258	35103	+	3-alpha-hydroxysteroid dehydrogenase (EC 1.1.1.50)	FIG00830506	ff
fig|6666666.28482.peg.2705	Contig_27	36025	35435	-	hypothetical protein		
fig|6666666.28482.peg.2706	Contig_27	36111	36803	+	short chain dehydrogenase		
fig|6666666.28482.peg.2707	Contig_27	37045	37497	+	FIG00825553: hypothetical protein	FIG00825552	ff
fig|6666666.28482.peg.2708	Contig_27	37499	38305	+	short-chain dehydrogenase/reductase SDR	FIG00440067	ff
fig|6666666.28482.peg.2709	Contig_27	39055	38309	-	methyltransferase, UbiE/COQ5 family	FIG01504808	ff
fig|6666666.28482.peg.2710	Contig_27	40546	39170	-	Adenylate cyclase (EC 4.6.1.1)	FIG00001294	idu(25);cAMP_signaling_in_bacteria
fig|6666666.28482.peg.2711	Contig_27	41203	40610	-	Transcriptional regulator, TetR family	FIG00554417	ff
fig|6666666.28482.peg.2712	Contig_27	41309	42244	+	FIG00828462: hypothetical protein	FIG00828461	ff

fig|6666666.28482.peg.2713	Contig_27	43218	42241	-	2-amino-3-carboxymuconate-6-semialdehyde decarboxylase (EC 4.1.1.45)	FIG00010796	ff
fig|6666666.28482.peg.2714	Contig_27	43357	44142	+	DltE protein	FIG00823437	ff
fig|6666666.28482.peg.2715	Contig_27	44258	45346	+	FIG00824533: hypothetical protein	FIG00824532	ff
fig|6666666.28482.peg.2716	Contig_28	140	922	+	Alpha-acetolactate decarboxylase (EC 4.1.1.5)	FIG00001396	isu;Alpha-acetolactate_operon isu;Acetoin,_butanediol_metabolism
fig|6666666.28482.peg.2717	Contig_28	1774	1109	-	hypothetical protein		
fig|6666666.28482.peg.2718	Contig_28	2721	1771	-	hypothetical protein	FIG00638284	ff
fig|6666666.28482.peg.2719	Contig_28	2806	3588	+	Putative HTH-type transcriptional regulator		
fig|6666666.28482.peg.2720	Contig_28	3666	4790	+	glycosyl transferase		
fig|6666666.28482.peg.2721	Contig_28	5170	4838	-	hypothetical protein		
fig|6666666.28482.peg.2722	Contig_28	5438	6289	+	Thiosulfate sulfurtransferase, rhodanese (EC 2.8.1.1)	FIG00004487	ff
fig|6666666.28482.peg.2723	Contig_28	7301	6291	-	FIG00821330: hypothetical protein	FIG00821329	ff
fig|6666666.28482.peg.2724	Contig_28	7475	8272	+	alpha/beta hydrolase fold		
fig|6666666.28482.peg.2725	Contig_28	8269	9051	+	3-oxoacyl-[acyl-carrier protein] reductase (EC 1.1.1.100)	FIG00621114	idu(38);CBSS-246196.1.peg.364 idu(38);CBSS-164757.7.peg.5020 idu(38);Fatty_Acid_Biosynthesis_FASI I
fig|6666666.28482.peg.2726	Contig_28	9048	9911	+	Coenzyme F420-dependent oxidoreductase	FIG00015014	idu(3);Anaerobic_respiratory_reducta ses

fig|6666666.28482.peg.2727	Contig_28	9924	10337	+	FIG00830078: hypothetical protein	FIG00830076	ff
fig|6666666.28482.peg.2728	Contig_28	10334	11062	+	FIG00824101: hypothetical protein	FIG00824100	ff
fig|6666666.28482.peg.2729	Contig_28	11162	11665	+	LppO	FIG01682981	ff
fig|6666666.28482.peg.2730	Contig_28	11892	12881	+	FIG00823423: hypothetical protein	FIG00823422	ff
fig|6666666.28482.peg.2731	Contig_28	14162	12963	-	putative cytochrome P450 hydroxylase	FIG00032437	icw(1);Nitric_oxide_synthase
fig|6666666.28482.peg.2732	Contig_28	14809	14171	-	Transcriptional regulator, TetR family	FIG00554417	ff
fig|6666666.28482.peg.2733	Contig_28	14870	14986	+	hypothetical protein		
fig|6666666.28482.peg.2734	Contig_28	15007	15768	+	FIG00820932: hypothetical protein	FIG00820930	ff
fig|6666666.28482.peg.2735	Contig_28	16972	15776	-	putative cytochrome P450 hydroxylase	FIG00032437	icw(1);Nitric_oxide_synthase
fig|6666666.28482.peg.2736	Contig_28	17961	16969	-	FIG00825406: hypothetical protein	FIG00825404	ff
fig|6666666.28482.peg.2737	Contig_28	19092	18031	-	FIG00826901: hypothetical protein	FIG00826898	ff
fig|6666666.28482.peg.2738	Contig_28	19282	20376	+	hypothetical protein	FIG00638284	ff
fig|6666666.28482.peg.2739	Contig_28	20386	21579	+	FIG00828083: hypothetical protein	FIG00828082	ff
fig|6666666.28482.peg.2740	Contig_28	22452	21724	-	FIG00826011: hypothetical protein	FIG00826010	ff
fig|6666666.28482.peg.2741	Contig_28	22563	23468	+	Haloalkane dehalogenase (EC 3.8.1.5)	FIG01364259	ff
fig|6666666.28482.peg.2742	Contig_28	24507	23476	-	FIG00833085: hypothetical protein	FIG00833081	ff
fig|6666666.28482.peg.2743	Contig_28	24737	25168	+	FIG00821774: hypothetical protein	FIG00821772	ff
fig|6666666.28482.peg.2744	Contig_28	25923	25183	-	CysQ protein	FIG01352967	ff

fig|6666666.28482.peg.2745	Contig_28	26086	26640	+	FIG00830662: hypothetical protein	FIG00830661	ff
fig|6666666.28482.peg.2746	Contig_28	27808	26645	-	FIG00822698: hypothetical protein	FIG00822697	ff
fig|6666666.28482.peg.2747	Contig_28	28037	29092	+	Oxalate decarboxylase (EC 4.1.1.2)	FIG01323605	ff
fig|6666666.28482.peg.2748	Contig_28	29112	29510	+	FIG00829888: hypothetical protein	FIG00829886	ff
fig|6666666.28482.peg.2749	Contig_28	29577	30455	+	FIG00822009: hypothetical protein	FIG00822008	ff
fig|6666666.28482.peg.2750	Contig_28	30833	30426	-	FIG00825195: hypothetical protein	FIG00825190	ff
fig|6666666.28482.peg.2751	Contig_28	30917	31663	+	PknM	FIG01539262	ff
fig|6666666.28482.peg.2752	Contig_28	31885	33213	+	Mg/Co/Ni transporter MgtE / CBS domain	FIG00437800	idu(1);Magnesium_transport
fig|6666666.28482.peg.2753	Contig_28	33210	34847	+	Manganese transport protein MntH	FIG00554286	ff
fig|6666666.28482.peg.2754	Contig_28	34942	35184	+	Excalibur		
fig|6666666.28482.peg.2755	Contig_28	36390	35197	-	Permeases of the major facilitator superfamily	FIG00613966	ff
fig|6666666.28482.peg.2756	Contig_28	36629	37348	+	Predicted regulator PutR for proline utilization, GntR family	FIG00004161	idu(2);Proline,_4-hydroxyproline_uptake_and_utilization
fig|6666666.28482.peg.2757	Contig_28	37565	37350	-	FIG00827338: hypothetical protein	FIG00827337	ff
fig|6666666.28482.peg.2758	Contig_28	39555	37633	-	Chaperone protein HtpG	FIG00000594	isu;Protein_chaperones
fig|6666666.28482.peg.2759	Contig_28	39648	40268	+	FIG00825159: hypothetical protein	FIG00825157	ff

fig|6666666.28482.peg.2760	Contig_28	41836	40277	-	FIG00827108: hypothetical protein	FIG00827107	ff
fig|6666666.28482.peg.2761	Contig_28	42672	41833	-	TesB-like acyl-CoA thioesterase 4	FIG00085764	idu(2);Acyl-CoA_thioesterase_II
fig|6666666.28482.peg.2762	Contig_28	43468	42743	-	FIG00828734: hypothetical protein	FIG00828733	ff
fig|6666666.28482.peg.2763	Contig_28	43666	44334	+	Xanthine dehydrogenase, molybdenum binding subunit (EC 1.17.1.4)	FIG00000962	ff
fig|6666666.28482.peg.2764	Contig_28	45321	44401	-	FIG00822145: hypothetical protein	FIG00822143	ff
fig|6666666.28482.peg.2765	Contig_28	45416	45736	+	hypothetical protein		
fig|6666666.28482.peg.2766	Contig_28	45759	46226	+	hypothetical protein		
fig|6666666.28482.peg.2767	Contig_28	46858	46223	-	FIG00820691: hypothetical protein	FIG00820690	ff
fig|6666666.28482.peg.2768	Contig_28	48309	47371	-	Transcriptional regulator, AraC family	FIG00510130	ff
fig|6666666.28482.peg.2769	Contig_28	48392	49648	+	probable multidrug resistance transporter, MFS superfamily		
fig|6666666.28482.peg.2770	Contig_28	50363	49671	-	ThiJ/PfpI family protein	FIG00015923	idu(8);CBSS-176280.1.peg.1561
fig|6666666.28482.peg.2771	Contig_28	50676	51683	+	FIG00826852: hypothetical protein	FIG00826845	ff
fig|6666666.28482.peg.2772	Contig_28	51895	51680	-	hypothetical protein	FIG00638284	ff
fig|6666666.28482.peg.2773	Contig_28	53060	51984	-	Vanillate O-demethylase oxidoreductase (EC 1.14.13.-)	FIG01303955	idu(2);Anaerobic_respiratory_reductases
fig|6666666.28482.peg.2774	Contig_28	53976	53071	-	FIG00825795: hypothetical protein	FIG00825791	ff

fig|6666666.28482.peg.2775	Contig_28	54904	53996	-	FF domain protein	FIG01341765	ff
fig|6666666.28482.peg.2776	Contig_28	58187	54924	-	Long-chain-fatty-acid--CoA ligase (EC 6.2.1.3)	FIG00018699	idu(72);n-Phenylalkanoic_acid_degradation idu(72);Fatty_acid_metabolism_cluster idu(72);Biotin_synthesis_cluster idu(72);Biotin_biosynthesis
fig|6666666.28482.peg.2777	Contig_28	58764	59006	+	FIG00825611: hypothetical protein	FIG00825609	ff
fig|6666666.28482.peg.2778	Contig_28	60089	59016	-	FIG00826800: hypothetical protein	FIG00826799	ff
fig|6666666.28482.peg.2779	Contig_28	60266	61135	+	Short-chain dehydrogenase/reductase SDR	FIG00546160	idu(13);CBSS-164757.7.peg.5020 idu(13);Transcription_repair_cluster
fig|6666666.28482.peg.2780	Contig_28	61274	62191	+	hypothetical protein	FIG01687736	ff
fig|6666666.28482.peg.2781	Contig_28	63316	62198	-	EmbR		
fig|6666666.28482.peg.2782	Contig_28	64746	63334	-	Probable serine/threonine-protein kinase pknH (EC 2.7.11.1)		
fig|6666666.28482.peg.2783	Contig_28	64936	66123	+	hypothetical protein		
fig|6666666.28482.peg.2784	Contig_28	66304	67104	+	hypothetical protein		
fig|6666666.28482.peg.2785	Contig_28	67101	67763	+	hypothetical protein		
fig|6666666.28482.peg.2786	Contig_28	68001	68552	+	immunogenic protein MPB64/MPT64 precursor		
fig|6666666.28482.peg.2787	Contig_28	68570	69829	+	hypothetical protein		
fig|6666666.28482.peg.2788	Contig_28	70012	70755	+	Lipoprotein LppH		
fig|6666666.28482.peg.2789	Contig_28	71104	86379	+	FIG00820660: hypothetical protein	FIG00820658	ff
fig|6666666.28482.peg.2790	Contig_28	89303	86376	-	hypothetical protein		

fig|6666666.28482.peg.2791	Contig_28	89929	89300	-	conserved hypothetical secreted protein	FIG00536634	ff
fig|6666666.28482.peg.2792	Contig_28	90064	91053	+	PE family protein		
fig|6666666.28482.peg.2793	Contig_28	92533	91088	-	Nitrate/nitrite transporter	FIG00005529	idu(2);Nitrate_and_nitrite_ammonification
fig|6666666.28482.peg.2794	Contig_28	93066	92743	-	hypothetical protein	FIG00638284	ff
fig|6666666.28482.peg.2795	Contig_28	93308	93826	+	conserved hypothetical protein		
fig|6666666.28482.peg.2796	Contig_28	94740	93838	-	FIG00832991: hypothetical protein	FIG00832989	ff
fig|6666666.28482.peg.2797	Contig_28	94958	95845	+	Endoglucanase A precursor (EC 3.2.1.4)	FIG01365504	ff
fig|6666666.28482.peg.2798	Contig_29	933	487	-	hypothetical protein	FIG00638284	ff
fig|6666666.28482.peg.2799	Contig_29	1925	1104	-	Exodeoxyribonuclease III (EC 3.1.11.2)	FIG00000065	isu;DNA_repair,_bacterial
fig|6666666.28482.peg.2800	Contig_29	2650	1928	-	Histone acetyltransferase HPA2 and related acetyltransferases	FIG00001650	idu(1);CBSS-216591.1.peg.168
fig|6666666.28482.peg.2801	Contig_29	3273	2680	-	Peptide deformylase (EC 3.5.1.88)	FIG00000017	isu;Translation_termination_factors_bacterial
fig|6666666.28482.peg.2802	Contig_29	3478	3786	+	FIG01000274: hypothetical protein	FIG00822393	ff
fig|6666666.28482.peg.2803	Contig_29	3851	4279	+	Tuberculin related peptide	FIG01317574	ff
fig|6666666.28482.peg.2804	Contig_29	4326	5033	+	Superoxide dismutase [Cu-Zn] precursor (EC 1.15.1.1)	FIG00001181	idu(2);Oxidative_stress idu(2);Protection_from_Reactive_Oxygen_Species
fig|6666666.28482.peg.2805	Contig_29	5086	6153	+	Carboxylate-amine ligase	FIG01194400	ff

fig|6666666.28482.peg.2806	Contig_29	6158	6808	+	Uncharacterized protein, similar to the N-terminal domain of Lon protease	FIG01309879	ff
fig|6666666.28482.peg.2807	Contig_29	6820	7638	+	Enoyl-CoA hydratase (EC 4.2.1.17)	FIG00033206	idu(51);n-Phenylalkanoic_acid_degradation idu(51);Polyhydroxybutyrate_metabolism idu(51);Isoleucine_degradation idu(51);Fatty_acid_metabolism_cluster idu(51);Lysine_fermentation idu(51);Butanol_Biosynthesis idu(51);Acetyl-CoA_fermentation_to_Butyrate idu(51);Valine_degradation idu(51);Butyrate_metabolism_cluster
fig|6666666.28482.peg.2808	Contig_29	8186	7644	-	hypothetical protein		
fig|6666666.28482.peg.2809	Contig_29	9124	8213	-	FIG00831858: hypothetical protein	FIG00831853	ff
fig|6666666.28482.peg.2810	Contig_29	10593	9142	-	LuxC, acyl-CoA reductase		
fig|6666666.28482.peg.2811	Contig_29	12026	10590	-	hypothetical protein	FIG00638284	ff
fig|6666666.28482.peg.2812	Contig_29	13607	12084	-	transmembrane efflux pump		
fig|6666666.28482.peg.2813	Contig_29	14331	13591	-	FIG00826918: hypothetical protein	FIG00826916	ff
fig|6666666.28482.peg.2814	Contig_29	14422	14988	+	Transcriptional regulator, TetR family	FIG00554417	ff

fig|6666666.28482.peg.2815	Contig_29	17127	14962	-	Cell division protein FtsH (EC 3.4.24.-)	FIG00018369	idu(1);Bacterial_Cell_Division idu(1);Cell_division-ribosomal_stress_proteins_cluster
fig|6666666.28482.peg.2816	Contig_29	17987	17124	-	CDP-diacylglycerol--serine O-phosphatidyltransferase (EC 2.7.8.8)	FIG00000775	isu;Glycerolipid_and_Glycerophospholipid_Metabolism_in_Bacteria
fig|6666666.28482.peg.2817	Contig_29	18715	17984	-	Phosphatidylserine decarboxylase (EC 4.1.1.65)	FIG00001264	icw(1);Glycerolipid_and_Glycerophospholipid_Metabolism_in_Bacteria
fig|6666666.28482.peg.2818	Contig_29	19929	18724	-	Molybdopterin biosynthesis protein MoeA	FIG00018218	idu(1);Molybdenum_cofactor_biosynthesis
fig|6666666.28482.peg.2819	Contig_29	19984	20460	+	hypothetical protein		
fig|6666666.28482.peg.2820	Contig_29	20457	21902	+	Cyclohexanone monooxygenase (EC 1.14.13.22)	FIG00975774	ff
fig|6666666.28482.peg.2821	Contig_29	22037	22204	+	hypothetical protein		
fig|6666666.28482.peg.2822	Contig_29	22728	22210	-	FIG00826887: hypothetical protein	FIG00826886	ff
fig|6666666.28482.peg.2823	Contig_29	22736	23437	+	FIG00830052: hypothetical protein	FIG00830045	ff
fig|6666666.28482.peg.2824	Contig_29	24370	23456	-	probable oxidoreductase/Short-chain dehydrogenase	FIG01319309	ff
fig|6666666.28482.peg.2825	Contig_29	24474	25040	+	Transcriptional regulator, TetR family	FIG00554417	ff
fig|6666666.28482.peg.2826	Contig_29	25205	26830	+	Heat shock protein 60 family chaperone GroEL	FIG00000056	idu(1);Staphylococcal_pathogenicity_islands_SaPI idu(1);GroEL_GroES
fig|6666666.28482.peg.2827	Contig_29	26981	27814	+	FIG00827009: hypothetical protein	FIG00827007	ff

fig|6666666.28482.peg.2828	Contig_29	28261	27863	-	FIG00825576: hypothetical protein	FIG00825575	ff
fig|6666666.28482.peg.2829	Contig_29	29671	28280	-	PPE family protein	FIG00013075	idu(57);CBSS-164757.7.peg.5020
fig|6666666.28482.peg.2830	Contig_29	31461	29710	-	D-aminoacylase (EC 3.5.1.81)	FIG01332753	ff
fig|6666666.28482.peg.2831	Contig_29	31509	31877	+	PROBABLE LACTOYLGLUTATHIONE LYASE (EC 4.4.1.5)	FIG00884358	ff
fig|6666666.28482.peg.2832	Contig_29	31888	32403	+	FIG00820188: hypothetical protein	FIG00820187	ff
fig|6666666.28482.peg.2833	Contig_29	32414	33259	+	FIG00827740: hypothetical protein	FIG00827739	ff
fig|6666666.28482.peg.2834	Contig_29	33953	33252	-	FIG111991: hypothetical protein	FIG00002457	isu;CBSS-313593.3.peg.2729
fig|6666666.28482.peg.2835	Contig_29	34507	33950	-	RNA polymerase sigma-70 factor	FIG01303902	icw(1);CBSS-313593.3.peg.2729 idu(4);Transcription_initiation,_bacterial_sigma_factors
fig|6666666.28482.peg.2836	Contig_29	36334	34652	-	DipZ protein	FIG01311174	ff
fig|6666666.28482.peg.2837	Contig_29	39254	36366	-	Putative membrane protein	FIG01056920	ff
fig|6666666.28482.peg.2838	Contig_29	39742	39251	-	membrane protein, MmpS family	FIG01309473	ff
fig|6666666.28482.peg.2839	Contig_29	40323	39994	-	FIG00820218: hypothetical protein	FIG00820217	ff
fig|6666666.28482.peg.2840	Contig_29	40917	40519	-	Possible membrane protein	FIG01121868	ff

fig|6666666.28482.peg.2841	Contig_29	41952	41044	-	Enoyl-CoA hydratase (EC 4.2.1.17)	FIG00033206	idu(51);n-Phenylalkanoic_acid_degradation idu(51);Polyhydroxybutyrate_metabolism idu(51);Isoleucine_degradation idu(51);Fatty_acid_metabolism_cluster idu(51);Lysine_fermentation idu(51);Butanol_Biosynthesis idu(51);Acetyl-CoA_fermentation_to_Butyrate idu(51);Valine_degradation idu(51);Butyrate_metabolism_cluster
fig|6666666.28482.peg.2842	Contig_29	42272	41961	-	FIG00823266: hypothetical protein	FIG00823265	ff
fig|6666666.28482.peg.2843	Contig_29	42352	43608	+	FIG00832352: hypothetical protein	FIG00821880	ff
fig|6666666.28482.peg.2844	Contig_29	43837	43595	-	hypothetical protein		
fig|6666666.28482.peg.2845	Contig_29	45927	43882	-	Prolyl endopeptidase (EC 3.4.21.26)	FIG00011272	isu;Serine_endopeptidase_(EC_3.4.21.-)
fig|6666666.28482.peg.2846	Contig_29	45985	47508	+	Aldehyde dehydrogenase (EC 1.2.1.3)	FIG01292786	idu(16);Pyruvate_metabolism_II:_acetyl-CoA,_acetogenesis_from_pyruvate idu(16);Glycerolipid_and_Glycerophospholipid_Metabolism_in_Bacteria idu(16);Methylglyoxal_Metabolism
fig|6666666.28482.peg.2847	Contig_29	47508	48335	+	FIG00821442: hypothetical protein	FIG00821441	ff

fig|6666666.28482.peg.2848	Contig_29	48328	48861	+	Possible membrane protein	FIG01121868	ff
fig|6666666.28482.peg.2849	Contig_29	50949	48880	-	hypothetical protein	FIG00638284	ff
fig|6666666.28482.peg.2850	Contig_29	50969	51352	+	Long-chain-fatty-acid--CoA ligase (EC 6.2.1.3)	FIG00018699	idu(72);n-Phenylalkanoic_acid_degradation idu(72);Fatty_acid_metabolism_cluster idu(72);Biotin_synthesis_cluster idu(72);Biotin_biosynthesis
fig|6666666.28482.peg.2851	Contig_29	51590	51459	-	hypothetical protein		
fig|6666666.28482.peg.2852	Contig_29	51752	53146	+	Dihydrolipoamide dehydrogenase (EC 1.8.1.4)	FIG00136193	idu(1);TCA_Cycle idu(1);Photorespiration_(oxidative_C2_cycle) idu(1);Leucine_Degradation_and_HMG-CoA_Metabolism idu(1);Glycine_cleavage_system
fig|6666666.28482.peg.2853	Contig_29	53154	53438	+	hypothetical protein		
fig|6666666.28482.peg.2854	Contig_29	53992	53435	-	FIG00821074: hypothetical protein	FIG00821073	ff
fig|6666666.28482.peg.2855	Contig_29	55413	53989	-	"Transcriptional regulator, XRE family"	FIG00822523	ff
fig|6666666.28482.peg.2856	Contig_29	55576	56370	+	Acyl-ACP thioesterase	FIG01319281	ff
fig|6666666.28482.peg.2857	Contig_29	56657	57943	+	Isocitrate lyase (EC 4.1.3.1) / Methylisocitrate lyase (EC 4.1.3.30)	FIG00148624	idu(1);Serine-glyoxylate_cycle idu(1);Glyoxylate_bypass isu;Methylcitrate_cycle idu(1);Glyoxylate_bypass_cluster

fig|6666666.28482.peg.2858	Contig_29	58099	58962	+	3-hydroxybutyryl-CoA dehydrogenase (EC 1.1.1.157); 3-hydroxyacyl-CoA dehydrogenase (EC 1.1.1.35)	FIG00000819	idu(4);n-Phenylalkanoic_acid_degradation idu(4);Polyhydroxybutyrate_metabolism idu(2);Polyhydroxybutyrate_metabolism idu(4);Isoleucine_degradation idu(4);Fatty_acid_metabolism_cluster idu(2);Lysine_fermentation idu(2);Butanol_Biosynthesis idu(2);Acetyl-CoA_fermentation_to_Butyrate idu(4);Acetyl-CoA_fermentation_to_Butyrate idu(4);Valine_degradation idu(4);Butyrate_metabolism_cluster
fig|6666666.28482.peg.2859	Contig_29	59046	59906	+	POSSIBLE MYCOLIC ACID SYNTHASE UMAA1	FIG01316311	ff
fig|6666666.28482.peg.2860	Contig_29	60794	59931	-	Cyclopropane-fatty-acyl-phospholipid synthase (EC 2.1.1.79)	FIG00001555	ff
fig|6666666.28482.peg.2861	Contig_29	61951	60938	-	FIG00820166: hypothetical protein	FIG00820164	ff
fig|6666666.28482.peg.2862	Contig_29	62665	61964	-	"Transcriptional regulator, TetR family"	FIG01310990	ff
fig|6666666.28482.peg.2863	Contig_29	62810	62685	-	hypothetical protein		
fig|6666666.28482.peg.2864	Contig_29	62931	64142	+	Possible membrane protein	FIG01121868	ff

fig|6666666.28482.peg.2865	Contig_29	64227	64646	+	"Transcriptional regulator, XRE family"	FIG00822523	ff
fig|6666666.28482.peg.2866	Contig_29	64793	65386	+	Iron-regulated heparin binding hemagglutinin HbhA (Adhesin)	FIG00819985	ff
fig|6666666.28482.peg.2867	Contig_29	65475	65750	+	FIG00820770: hypothetical membrane protein	FIG00820769	ff
fig|6666666.28482.peg.2868	Contig_29	65755	66174	+	POSSIBLE CONSERVED SECRETED PROTEIN	FIG00996000	ff
fig|6666666.28482.peg.2869	Contig_29	66175	66864	+	Deoxyribose-phosphate aldolase (EC 4.1.2.4)	FIG00000450	isu;Deoxyribose_and_Deoxynucleoside_Catabolism
fig|6666666.28482.peg.2870	Contig_29	67661	66861	-	Possible membrane protein	FIG01121868	ff
fig|6666666.28482.peg.2871	Contig_29	68734	67898	-	Carbon-nitrogen hydrolase	FIG00554419	ff
fig|6666666.28482.peg.2872	Contig_29	69350	68826	-	hypothetical protein		
fig|6666666.28482.peg.2873	Contig_29	69377	70474	+	UDP-N-acetylenolpyruvoylglucosamine reductase (EC 1.1.1.158)	FIG00000163	isu;UDP-N-acetylmuramate_from_Fructose-6-phosphate_Biosynthesis isu;Peptidoglycan_Biosynthesis
fig|6666666.28482.peg.2874	Contig_29	70596	71885	+	Possible lipoprotein LprQ	FIG00864239	ff
fig|6666666.28482.peg.2875	Contig_29	72585	71869	-	Serine 3-dehydrogenase	FIG00814703	ff
fig|6666666.28482.peg.2876	Contig_29	72849	74102	+	Transcriptional regulatory protein	FIG00447785	ff

fig|6666666.28482.peg.2877	Contig_29	74155	75486	+	Glycosyltransferase MshA involved in mycothiol biosynthesis (EC 2.4.1.-)	FIG01295925	isu;Glutathione_analogs:_mycothiol
fig|6666666.28482.peg.2878	Contig_29	75483	76001	+	Uncharacterized protein Rv0487/MT0505 clustered with mycothiol biosynthesis gene	FIG00020314	icw(1);Glutathione_analogs:_mycothiol
fig|6666666.28482.peg.2879	Contig_29	76025	76774	+	Phosphoglycerate mutase (EC 5.4.2.1)	FIG00005840	idu(2);Phosphoglycerate_mutase_protein_family
fig|6666666.28482.peg.2880	Contig_29	76930	78144	+	Phosphate regulon sensor protein PhoR (SphS) (EC 2.7.13.3); Sensor-like histidine kinase senX3 (EC 2.7.13.3)	FIG00138089	icw(1);High_affinity_phosphate_transporter_and_control_of_PHO_regulon isu;Phosphate_metabolism icw(1);PhoR-PhoB_two-component_regulatory_system
fig|6666666.28482.peg.2881	Contig_29	78141	78824	+	Phosphate regulon transcriptional regulatory protein PhoB (SphR); Sensory transduction protein regX3	FIG00137819	idu(1);High_affinity_phosphate_transporter_and_control_of_PHO_regulon icw(1);Phosphate_metabolism idu(1);PhoR-PhoB_two-component_regulatory_system
fig|6666666.28482.peg.2882	Contig_29	80781	78928	-	GMC-type oxidoreductase	FIG01438966	ff
fig|6666666.28482.peg.2883	Contig_29	81065	80778	-	FIG00823091: hypothetical protein	FIG00823088	ff
fig|6666666.28482.peg.2884	Contig_29	82060	81104	-	FIG00824158: hypothetical protein	FIG00824157	ff

fig|6666666.28482.peg.2885	Contig_29	82137	82859	+	Lactate-responsive regulator LldR in Enterobacteria, GntR family	FIG00004506	isu;Lactate_utilization
fig|6666666.28482.peg.2886	Contig_29	83658	82873	-	FIG00820527: hypothetical protein	FIG00820526	ff
fig|6666666.28482.peg.2887	Contig_29	83754	84770	+	Exopolyphosphatase (EC 3.6.1.11)	FIG00066575	idu(2);Polyphosphate idu(2);Phosphate_metabolism
fig|6666666.28482.peg.2888	Contig_29	84818	85705	+	hypothetical protein		
fig|6666666.28482.peg.2889	Contig_29	85722	86564	+	"AP endonuclease, family protein 2"	FIG00820391	ff
fig|6666666.28482.peg.2890	Contig_29	86590	87429	+	TesB-like acyl-CoA thioesterase 3	FIG00020817	isu;Acyl-CoA_thioesterase_II
fig|6666666.28482.peg.2891	Contig_29	87457	88329	+	Pyrroline-5-carboxylate reductase (EC 1.5.1.2)	FIG00133325	isu;Proline_Synthesis isu;A_Hypothetical_Protein_Related_to_Proline_Metabolism
fig|6666666.28482.peg.2892	Contig_29	88590	88727	+	hypothetical protein		
fig|6666666.28482.peg.2893	Contig_29	89018	90133	+	UDP-glucose 4-epimerase (EC 5.1.3.2)	FIG00022300	idu(4);Rhamnose_containing_glycans idu(4);Lactose_and_Galactose_Uptake_and_Utilization idu(4);N-linked_Glycosylation_in_Bacteria idu(4);Lacto-N-Biose_I_and_Galacto-N-Biose_Metabolic_Pathway
fig|6666666.28482.peg.2894	Contig_29	90250	91206	+	Phospholipid/glycerol acyltransferase	FIG00815019	ff
fig|6666666.28482.peg.2895	Contig_29	92140	91220	-	Cyclopropane-fatty-acyl-phospholipid synthase 2, CmaA2 (EC 2.1.1.79)	FIG01314782	ff

fig|6666666.28482.peg.2896	Contig_29	92667	92152	-	(3R)-hydroxyacyl-ACP dehydratase subunit HadA	FIG00821897	ff
fig|6666666.28482.peg.2897	Contig_29	93621	92710	-	Phosphoserine phosphatase (EC 3.1.3.3)	FIG00000149	idu(3);CBSS-336982.3.peg.3874 idu(3);Serine_Biosynthesis
fig|6666666.28482.peg.2898	Contig_29	94012	93740	-	FIG00830030: hypothetical protein	FIG00830027	ff
fig|6666666.28482.peg.2899	Contig_29	94301	94023	-	putative ESAT-6 like protein 13 esxF		
fig|6666666.28482.peg.2900	Contig_29	94418	94675	+	FIG00822170: hypothetical protein	FIG00822168	ff
fig|6666666.28482.peg.2901	Contig_29	94806	94627	-	hypothetical protein		
fig|6666666.28482.peg.2902	Contig_29	94777	96147	+	Glutamyl-tRNA reductase (EC 1.2.1.70)	FIG00004795	icw(2);Heme_and_Siroheme_Biosynthesis
fig|6666666.28482.peg.2903	Contig_29	96152	97105	+	Porphobilinogen deaminase (EC 2.5.1.61)	FIG00000363	icw(3);Heme_and_Siroheme_Biosynthesis
fig|6666666.28482.peg.2904	Contig_29	97173	98795	+	Uroporphyrinogen-III methyltransferase (EC 2.1.1.107) / Uroporphyrinogen-III synthase (EC 4.2.1.75)	FIG00137145	idu(1);Dissimilatory_nitrite_reductase isu;Heme_and_Siroheme_Biosynthesis icw(3);Heme_and_Siroheme_Biosynthesis idu(1);Coenzyme_B12_biosynthesis
fig|6666666.28482.peg.2905	Contig_29	98995	99882	+	Porphobilinogen synthase (EC 4.2.1.24)	FIG00000348	icw(1);Heme_and_Siroheme_Biosynthesis
fig|6666666.28482.peg.2906	Contig_29	99896	100450	+	Transmembrane transport protein MmpL2	FIG00999337	ff

fig|6666666.28482.peg.2907	Contig_29	100447	100692	+	putative transmembrane protein		
fig|6666666.28482.peg.2908	Contig_29	101004	100696	-	FIG00829481: hypothetical protein	FIG00829479	ff
fig|6666666.28482.peg.2909	Contig_29	101661	101005	-	ThiJ/PfpI family protein	FIG00015923	idu(8);CBSS-176280.1.peg.1561
fig|6666666.28482.peg.2910	Contig_29	101736	102689	+	Transcriptional regulator, AraC family	FIG00510130	ff
fig|6666666.28482.peg.2911	Contig_29	103117	102686	-	Anti-sigma F factor antagonist (spoIIAA-2); Anti-sigma B factor antagonist RsbV	FIG00507769	idu(5);SigmaB_stress_responce_regulation idu(5);Sigma-B_stress_response_cluster_1
fig|6666666.28482.peg.2912	Contig_29	103434	104684	+	Membrane acyltransferase	FIG01371329	ff
fig|6666666.28482.peg.2913	Contig_29	104854	105549	+	Possible membrane protein	FIG01121868	ff
fig|6666666.28482.peg.2914	Contig_29	105710	105594	-	hypothetical protein		
fig|6666666.28482.peg.2915	Contig_29	107893	105776	-	O-antigen acetylase	FIG01370327	ff
fig|6666666.28482.peg.2916	Contig_29	108835	107945	-	Possible membrane protein	FIG01121868	ff
fig|6666666.28482.peg.2917	Contig_29	108912	110585	+	Phospholipase C precursor (EC 3.1.4.3)	FIG01339894	ff
fig|6666666.28482.peg.2918	Contig_29	110677	111822	+	Alkanesulfonate monooxygenase (EC 1.14.14.5)	FIG00113730	idu(1);Alkanesulfonates_Utilization idu(1);Alkanesulfonate_assimilation
fig|6666666.28482.peg.2919	Contig_29	111826	113166	+	major facilitator family transporter	FIG01313819	ff
fig|6666666.28482.peg.2920	Contig_29	113304	114659	+	Aromatic amino acid transport protein AroP	FIG00116138	isu;Aromatic_amino_acid_degradation
fig|6666666.28482.peg.2921	Contig_29	115047	114643	-	FIG00820204: hypothetical protein	FIG00820202	ff

fig|6666666.28482.peg.2922	Contig_29	115947	115072	-	hypothetical protein	FIG00638284	ff
fig|6666666.28482.peg.2923	Contig_29	115971	116312	+	hypothetical protein		
fig|6666666.28482.peg.2924	Contig_29	116374	117717	+	Glutamate-1-semialdehyde aminotransferase (EC 5.4.3.8)	FIG00000338	icw(1);CBSS-196164.1.peg.461 isu;Heme_and_Siroheme_Biosynthesis
fig|6666666.28482.peg.2925	Contig_29	117717	118325	+	Hypothetical, related to broad specificity phosphatases COG0406	FIG00004688	isu;CBSS-196164.1.peg.461
fig|6666666.28482.peg.2926	Contig_29	118328	118912	+	Thiol:disulfide oxidoreductase related to ResA	FIG00618007	icw(1);Biogenesis_of_c-type_cytochromes icw(3);CBSS-196164.1.peg.461
fig|6666666.28482.peg.2927	Contig_29	118909	119688	+	Cytochrome c-type biogenesis protein CcdA (DsbD analog)	FIG00133238	icw(2);Biogenesis_of_c-type_cytochromes isu;Sulfur_oxidation icw(4);CBSS-196164.1.peg.461 isu;Periplasmic_disulfide_interchange
fig|6666666.28482.peg.2928	Contig_29	119688	121370	+	Ccs1/ResB-related putative cytochrome C-type biogenesis protein	FIG00003692	icw(3);Biogenesis_of_c-type_cytochromes icw(5);CBSS-196164.1.peg.461
fig|6666666.28482.peg.2929	Contig_29	121367	122353	+	Cytochrome c-type biogenesis protein CcsA/ResC	FIG00001246	isu;Biogenesis_of_c-type_cytochromes icw(2);CBSS-196164.1.peg.461
fig|6666666.28482.peg.2930	Contig_29	122398	123684	+	RD1 region associated protein Rv3876	FIG00044407	ff
fig|6666666.28482.peg.2931	Contig_29	123846	123691	-	hypothetical protein		
fig|6666666.28482.peg.2932	Contig_29	123893	124237	+	Possible membrane protein	FIG01121868	ff

fig|6666666.28482.peg.2933	Contig_29	125215	124343	-	1,4-dihydroxy-2-naphthoate octaprenyltransferase (EC 2.5.1.74)	FIG00085608	isu;Menaquinone_and_Phylloquinone_Biosynthesis_--_gjo isu;Menaquinone_and_Phylloquinone_Biosynthesis
fig|6666666.28482.peg.2934	Contig_29	125250	126026	+	5'-methylthioadenosine phosphorylase (EC 2.4.2.28)	FIG00049258	isu;Methionine_Salvage
fig|6666666.28482.peg.2935	Contig_29	126029	127054	+	UDP-glucose 4-epimerase (EC 5.1.3.2)	FIG00022300	idu(4);Rhamnose_containing_glycans idu(4);Lactose_and_Galactose_Uptake_and_Utilization idu(4);N-linked_Glycosylation_in_Bacteria idu(4);Lacto-N-Biose_I_and_Galacto-N-Biose_Metabolic_Pathway
fig|6666666.28482.peg.2936	Contig_3	2960	135	-	Glycine dehydrogenase [decarboxylating] (glycine cleavage system P protein) (EC 1.4.4.2)	FIG00000700	icw(1);Photorespiration_(oxidative_C2_cycle) icw(1);Glycine_cleavage_system
fig|6666666.28482.peg.2937	Contig_3	3867	3238	-	FIG00820710: hypothetical protein	FIG00820709	ff
fig|6666666.28482.peg.2938	Contig_3	4621	4127	-	hypothetical protein		
fig|6666666.28482.peg.2939	Contig_3	5453	4686	-	Transcriptional regulator, MerR family	FIG00003460	idu(1);Cobalt-zinc-cadmium_resistance
fig|6666666.28482.peg.2940	Contig_3	5936	5466	-	FIG00672531: hypothetical protein	FIG01121505	ff
fig|6666666.28482.peg.2941	Contig_3	6601	6203	-	Glycine cleavage system H protein	FIG00000296	isu;Photorespiration_(oxidative_C2_cycle) isu;CBSS-315749.4.peg.3658 isu;Glycine_cleavage_system

fig|6666666.28482.peg.2942	Contig_3	7343	6639	-	Division initiation protein	FIG00008602	icw(2);CBSS-227882.1.peg.6980
fig|6666666.28482.peg.2943	Contig_3	7842	7510	-	FIG025307: hypothetical protein	FIG00025307	isu;CBSS-227882.1.peg.6980
fig|6666666.28482.peg.2944	Contig_3	8794	7868	-	FIG030330: hypothetical protein	FIG00030330	icw(1);CBSS-227882.1.peg.6980
fig|6666666.28482.peg.2945	Contig_3	9416	8787	-	CDP-diacylglycerol--glycerol-3-phosphate 3-phosphatidyltransferase (EC 2.7.8.5)	FIG00001550	idu(2);Glycerolipid_and_Glycerophospholipid_Metabolism_in_Bacteria
fig|6666666.28482.peg.2946	Contig_3	11800	9476	-	Protein export cytoplasm protein SecA ATPase RNA helicase (TC 3.A.5.1.1)	FIG00034293	ff
fig|6666666.28482.peg.2947	Contig_3	13470	11827	-	COG0028: Thiamine pyrophosphate-requiring enzymes	FIG00452446	ff
fig|6666666.28482.peg.2948	Contig_3	13552	15471	+	Drugs-transport transmembrane ATP-binding protein ABC transporter	FIG00820139	ff
fig|6666666.28482.peg.2949	Contig_3	17051	15579	-	fumarate reductase/succinate dehydrogenase flavoprotein, N-terminal:FAD dependent oxidoreductase	FIG00141996	idu(1);Succinate_dehydrogenase
fig|6666666.28482.peg.2950	Contig_30	4	684	+	L-threonine 3-O-phosphate decarboxylase (EC 4.1.1.81)	FIG00000863	isu;Cobalamin_synthesis isu;Coenzyme_B12_biosynthesis

fig|6666666.28482.peg.2951	Contig_30	681	1850	+	FIG042796: Hypothetical protein	FIG01264307	icw(2);CBSS-100226.1.peg.2266 isu;CBSS-349161.4.peg.2417
fig|6666666.28482.peg.2952	Contig_30	1840	2577	+	FIG137478: Hypothetical protein	FIG01303938	isu;CBSS-100226.1.peg.2266
fig|6666666.28482.peg.2953	Contig_30	2574	3689	+	FIG006762: Phosphoglycerate mutase family	FIG00006762	icw(1);CBSS-100226.1.peg.2266
fig|6666666.28482.peg.2954	Contig_30	3699	4283	+	Transcriptional regulator, TetR family		
fig|6666666.28482.peg.2955	Contig_30	5478	4270	-	putative cytochrome P450 hydroxylase	FIG00032437	idu(49);Nitric_oxide_synthase
fig|6666666.28482.peg.2956	Contig_30	7196	5475	-	putative D-aminoacylase	FIG01131709	ff
fig|6666666.28482.peg.2957	Contig_30	8398	7208	-	Amidohydrolase 2		
fig|6666666.28482.peg.2958	Contig_30	8861	8466	-	FIG00821467: hypothetical protein	FIG00821466	ff
fig|6666666.28482.peg.2959	Contig_30	9121	8858	-	FIG034581: hypothetical protein		ff
fig|6666666.28482.peg.2960	Contig_30	9205	12393	+	Adenylate cyclase (EC 4.6.1.1)	FIG00001294	idu(25);cAMP_signaling_in_bacteria
fig|6666666.28482.peg.2961	Contig_30	12431	12679	+	hypothetical protein		
fig|6666666.28482.peg.2962	Contig_30	13415	12723	-	PE_PGRS family protein		
fig|6666666.28482.peg.2963	Contig_30	14753	13515	-	putative cytochrome P450 hydroxylase	FIG00032437	idu(49);Nitric_oxide_synthase
fig|6666666.28482.peg.2964	Contig_30	14925	19526	+	ABC-1 domain protein		
fig|6666666.28482.peg.2965	Contig_30	19513	22734	+	Adenylate cyclase (EC 4.6.1.1)	FIG00001294	idu(25);cAMP_signaling_in_bacteria
fig|6666666.28482.peg.2966	Contig_30	22758	22958	+	hypothetical protein	FIG00638284	ff
fig|6666666.28482.peg.2967	Contig_30	24495	22960	-	probable sulfite oxidase	FIG00815108	ff
fig|6666666.28482.peg.2968	Contig_30	24648	24505	-	hypothetical protein		

fig|6666666.28482.peg.2969	Contig_30	24825	28295	+	High-affnity carbon uptake protein Hat/HatR	FIG00451198	isu;CO2_uptake,_carboxysome
fig|6666666.28482.peg.2970	Contig_30	29776	28292	-	Aldehyde dehydrogenase (EC 1.2.1.3)	FIG01292786	idu(16);Pyruvate_metabolism_II:_acetyl-CoA,_acetogenesis_from_pyruvate icw(1);Glycerolipid_and_Glycerophospholipid_Metabolism_in_Bacteria idu(16);Methylglyoxal_Metabolism
fig|6666666.28482.peg.2971	Contig_30	29864	30934	+	Alcohol dehydrogenase (EC 1.1.1.1)	FIG01007502	idu(17);Glycerolipid_and_Glycerophospholipid_Metabolism_in_Bacteria idu(17);Fermentations:_Mixed_acid idu(17);Butanol_Biosynthesis
fig|6666666.28482.peg.2972	Contig_30	31132	30938	-	hypothetical protein	FIG01841460	ff
fig|6666666.28482.peg.2973	Contig_30	32538	31129	-	FIG00822502: hypothetical protein	FIG00822499	ff
fig|6666666.28482.peg.2974	Contig_30	34328	32826	-	Chaperonin GroEL (HSP60 family)	FIG01358020	ff
fig|6666666.28482.peg.2975	Contig_30	35593	34385	-	hypothetical protein		
fig|6666666.28482.peg.2976	Contig_30	36575	35727	-	3-methyl-2-oxobutanoate hydroxymethyltransferase (EC 2.1.2.11)	FIG00000364	isu;Coenzyme_A_Biosynthesis_cluster isu;Coenzyme_A_Biosynthesis
fig|6666666.28482.peg.2977	Contig_30	36704	38086	+	Wax ester synthase/acyl-CoA:diacylglycerol acyltransferase	FIG01309728	ff
fig|6666666.28482.peg.2978	Contig_30	38144	39700	+	Probable exported protease [EC:3.4.-.-]	FIG01357694	ff

fig|6666666.28482.peg.2979	Contig_30	39872	41485	+	Putative hydrolase	FIG01331319	ff
fig|6666666.28482.peg.2980	Contig_31	4013	888	-	ATP-dependent DNA helicase SCO5183	FIG00030006	isu;DNA_repair,_bacterial_RecBCD_pathway
fig|6666666.28482.peg.2981	Contig_31	4090	4287	+	FIG00823358: hypothetical protein	FIG00823348	ff
fig|6666666.28482.peg.2982	Contig_31	4284	5081	+	possible lipase	FIG00994937	ff
fig|6666666.28482.peg.2983	Contig_31	5084	5392	+	Methylated DNA-protein cysteine methyltransferase	FIG00820289	ff
fig|6666666.28482.peg.2984	Contig_31	6373	5519	-	FIG00994019: hypothetical protein	FIG00820742	ff
fig|6666666.28482.peg.2985	Contig_31	7591	6410	-	Sulfur carrier protein adenylyltransferase ThiF	FIG00001002	isu;Thiamin_biosynthesis
fig|6666666.28482.peg.2986	Contig_31	8755	7748	-	FIG00820929: hypothetical protein	FIG00820928	ff
fig|6666666.28482.peg.2987	Contig_31	8945	9631	+	Transcriptional regulator, TetR family	FIG00554417	ff
fig|6666666.28482.peg.2988	Contig_31	9890	9618	-	putative ATP-binding protein	FIG00945249	ff
fig|6666666.28482.peg.2989	Contig_31	10110	10805	+	putative membrane protein	FIG00672847	ff
fig|6666666.28482.peg.2990	Contig_31	11526	10807	-	FIG00821439: hypothetical protein	FIG00821438	ff
fig|6666666.28482.peg.2991	Contig_31	11774	13291	+	putative ATP-dependent RNA helicase	FIG01121467	ff

fig|6666666.28482.peg.2992	Contig_31	13307	14521	+	Cell division protein DivIC (FtsB), stabilizes FtsL against RasP cleavage	FIG00485194	idu(10);Possible_new_toxin-antitoxin_system_including_DivIC icw(1);Bacterial_Cell_Division idu(10);Cell_division-ribosomal_stress_proteins_cluster idu(10);Bacterial_Cytoskeleton idu(10);Stationary_phase_repair_cluster
fig|6666666.28482.peg.2993	Contig_31	15415	14615	-	Chromosome (plasmid) partitioning protein ParA / Sporulation initiation inhibitor protein Soj	FIG00006461	idu(2);RNA_modification_and_chromosome_partitioning_cluster idu(2);Bacterial_Cell_Division idu(2);Ribosome_post-transcriptional_modification_and_chromosomal_segregation_cluster icw(1);Bacterial_Cytoskeleton icw(1);Bacterial_Cytoskeleton idu(2);Plasmid_replication
fig|6666666.28482.peg.2994	Contig_31	15613	16224	+	putative phosphoglycerate mutase family protein	FIG01308292	ff
fig|6666666.28482.peg.2995	Contig_31	16221	17318	+	Isochorismate synthase (EC 5.4.4.2)	FIG01304982	isu;Chorismate:_Intermediate_for_synthesis_of_Tryptophan,_PAPA_antibiotics,_PABA,_3-hydroxyanthranilate_and_more.
fig|6666666.28482.peg.2996	Contig_31	17315	17797	+	Diamine acetyltransferase (EC 2.3.1.57)	FIG01342327	ff
fig|6666666.28482.peg.2997	Contig_31	18180	17749	-	PROBABLE CONSERVED INTEGRAL MEMBRANE PROTEIN		

fig|6666666.28482.peg.2998	Contig_31	18259	19215	+	Transcription regulator [contains diacylglycerol kinase catalytic domain]	FIG00001646	ff
fig|6666666.28482.peg.2999	Contig_31	19485	19739	+	WhiB-like transcription regulator	FIG00018409	isu;WhiB_and_WhiB-type_regulatory_proteins_
fig|6666666.28482.peg.3000	Contig_31	21308	19803	-	Signal transduction histidine kinase, subgroup 2		
fig|6666666.28482.peg.3001	Contig_31	21579	21358	-	Biotin carboxyl carrier protein	FIG00005415	idu(1);Urea_carboxylase_and_Allophanate_hydrolase_cluster idu(1);Fatty_Acid_Biosynthesis_FASII
fig|6666666.28482.peg.3002	Contig_31	22125	21823	-	Anti-sigma factor	FIG00997799	ff
fig|6666666.28482.peg.3003	Contig_31	22817	22122	-	RNA polymerase sigma-54 factor RpoN	FIG00006092	idu(2);Flagellar_motility idu(2);Flagellum idu(2);Transcription_initiation,_bacterial_sigma_factors
fig|6666666.28482.peg.3004	Contig_31	23065	23913	+	Short-chain dehydrogenase/reductase SDR	FIG00546160	idu(13);CBSS-164757.7.peg.5020 idu(13);Transcription_repair_cluster
fig|6666666.28482.peg.3005	Contig_31	23916	24455	+	Cys-tRNA(Pro) deacylase YbaK	FIG00003050	ff
fig|6666666.28482.peg.3006	Contig_31	25370	24549	-	Poly(3-hydroxyalkanoate) depolymerase	FIG01304243	icw(1);Polyhydroxybutyrate_metabolism
fig|6666666.28482.peg.3007	Contig_31	27095	25404	-	Polyhydroxyalkanoic acid synthase	FIG00000970	idu(1);Polyhydroxybutyrate_metabolism

fig|6666666.28482.peg.3008	Contig_31	28609	27098	-	Long-chain-fatty-acid--CoA ligase (EC 6.2.1.3)	FIG00018699	idu(72);n-Phenylalkanoic_acid_degradation idu(72);Fatty_acid_metabolism_cluster idu(72);Biotin_synthesis_cluster idu(72);Biotin_biosynthesis
fig|6666666.28482.peg.3009	Contig_31	28752	29975	+	hypothetical protein	FIG00638284	ff
fig|6666666.28482.peg.3010	Contig_31	30553	29972	-	Probable conserved membrane protein		
fig|6666666.28482.peg.3011	Contig_31	30814	31008	+	hypothetical protein		
fig|6666666.28482.peg.3012	Contig_31	32322	31012	-	FIG00828711: hypothetical protein	FIG00828709	ff
fig|6666666.28482.peg.3013	Contig_31	32461	34269	+	CONSERVED MEMBRANE PROTEIN	FIG00818075	ff
fig|6666666.28482.peg.3014	Contig_31	34266	35687	+	Phytoene dehydrogenase and related proteins	FIG00139162	ff
fig|6666666.28482.peg.3015	Contig_31	35827	36993	+	Butyryl-CoA dehydrogenase (EC 1.3.99.2)	FIG00009149	idu(39);Isoleucine_degradation idu(39);Lysine_fermentation idu(39);Butanol_Biosynthesis idu(39);Acetyl-CoA_fermentation_to_Butyrate idu(39);Valine_degradation idu(39);Anaerobic_respiratory_reductases
fig|6666666.28482.peg.3016	Contig_31	37083	37334	+	FIG00830431: hypothetical protein	FIG00830430	ff
fig|6666666.28482.peg.3017	Contig_31	37376	37927	+	hypothetical protein	FIG00638284	ff

fig|6666666.28482.peg.3018	Contig_31	38762	37893	-	3-hydroxybutyryl-CoA dehydrogenase (EC 1.1.1.157); 3-hydroxyacyl-CoA dehydrogenase (EC 1.1.1.35)	FIG00000819	idu(4);n-Phenylalkanoic_acid_degradation idu(4);Polyhydroxybutyrate_metabolism idu(2);Polyhydroxybutyrate_metabolism icw(1);Isoleucine_degradation idu(4);Fatty_acid_metabolism_cluster icw(1);Lysine_fermentation icw(1);Butanol_Biosynthesis icw(1);Acetyl-CoA_fermentation_to_Butyrate icw(1);Acetyl-CoA_fermentation_to_Butyrate icw(1);Valine_degradation idu(4);Butyrate_metabolism_cluster
fig|6666666.28482.peg.3019	Contig_31	40250	38853	-	PE family protein		
fig|6666666.28482.peg.3020	Contig_31	41048	40305	-	FIG00996837: hypothetical protein	FIG00996836	ff
fig|6666666.28482.peg.3021	Contig_31	41125	42411	+	5-Enolpyruvylshikimate-3-phosphate synthase (EC 2.5.1.19)	FIG00075970	isu;Chorismate_Synthesis isu;Common_Pathway_For_Synthesis_of_Aromatic_Compounds_(DAHP_synthase_to_chorismate)
fig|6666666.28482.peg.3022	Contig_31	42408	43388	+	Ribosome small subunit-stimulated GTPase EngC	FIG00000464	ff
fig|6666666.28482.peg.3023	Contig_31	43941	43468	-	Transcriptional regulator, HxlR family	FIG00906075	ff

fig|6666666.28482.peg.3024	Contig_31	44038	45219	+	3-ketoacyl-CoA thiolase (EC 2.3.1.16) @ Acetyl-CoA acetyltransferase (EC 2.3.1.9)	FIG00000004	idu(11);CBSS-246196.1.peg.364 idu(9);Serine-glyoxylate_cycle idu(11);Serine-glyoxylate_cycle idu(11);n-Phenylalkanoic_acid_degradation idu(9);Ethylmalonyl-CoA_pathway_of_C2_assimilation,_GJO idu(9);Polyhydroxybutyrate_metabolism idu(11);Polyhydroxybutyrate_metabolism idu(11);Isoleucine_degradation idu(11);Fatty_acid_metabolism_cluster idu(11);Biotin_synthesis_cluster idu(11);Biotin_biosynthesis idu(9);Lysine_fermentation idu(9);Isoprenoid_Biosynthesis idu(9);Butanol_Biosynthesis idu(9);Acetyl-CoA_fermentation_to_Butyrate idu(9);Butyrate_metabolism_cluster
fig|6666666.28482.peg.3025	Contig_31	45228	46121	+	hypothetical protein		
fig|6666666.28482.peg.3026	Contig_31	46114	46614	+	FIG00824368: hypothetical protein	FIG00824365	ff
fig|6666666.28482.peg.3027	Contig_31	47610	46639	-	Nitrilase/cyanide hydratase and apolipoprotein N-acyltransferase	FIG01355120	ff
fig|6666666.28482.peg.3028	Contig_31	47868	48395	+	hypothetical protein		

fig|6666666.28482.peg.3029	Contig_31	49526	48429	-	Carboxylesterase, type B	FIG01336027	ff
fig|6666666.28482.peg.3030	Contig_31	49968	50468	+	hypothetical protein	FIG00638284	ff
fig|6666666.28482.peg.3031	Contig_31	50917	50516	-	putative transmembrane protein		
fig|6666666.28482.peg.3032	Contig_31	52145	51759	-	hypothetical protein		
fig|6666666.28482.peg.3033	Contig_31	52526	52194	-	protein of unknown function DUF732		
fig|6666666.28482.peg.3034	Contig_31	53134	52598	-	hypothetical protein	FIG00638284	ff
fig|6666666.28482.peg.3035	Contig_31	54457	53177	-	POSSIBLE LINOLEOYL-CoA DESATURASE (DELTA(6)-DESATURASE)	FIG00539310	ff
fig|6666666.28482.peg.3036	Contig_31	55735	54545	-	Flavodoxin reductases (ferredoxin-NADPH reductases) family 1	FIG01954108	idu(3);Anaerobic_respiratory_reductases
fig|6666666.28482.peg.3037	Contig_31	56334	55822	-	FIG00996471: hypothetical protein	FIG00820145	ff
fig|6666666.28482.peg.3038	Contig_31	57200	56334	-	UDP-galactose-lipid carrier transferase (EC 2.-.-.-)	FIG01236906	ff
fig|6666666.28482.peg.3039	Contig_31	58663	57245	-	Wax ester synthase/acyl-CoA:diacylglycerol acyltransferase	FIG01309728	ff
fig|6666666.28482.peg.3040	Contig_31	58991	59332	+	FIG00830285: hypothetical protein	FIG00830283	ff
fig|6666666.28482.peg.3041	Contig_31	60479	59340	-	Kef-type K+ transport system, membrane component	FIG01956314	ff
fig|6666666.28482.peg.3042	Contig_31	60966	60484	-	TrkA, Potassium channel	FIG01334851	ff

fig|6666666.28482.peg.3043	Contig_31	62949	61009	-	Kup system potassium uptake protein	FIG00000843	isu;Potassium_homeostasis
fig|6666666.28482.peg.3044	Contig_31	63471	62998	-	GtrA family protein		
fig|6666666.28482.peg.3045	Contig_31	63555	64019	+	hypothetical protein		
fig|6666666.28482.peg.3046	Contig_31	64780	64016	-	hypothetical protein	FIG00638284	ff
fig|6666666.28482.peg.3047	Contig_31	64976	66712	+	FIG00996672: hypothetical protein	FIG00996670	ff
fig|6666666.28482.peg.3048	Contig_31	67529	66735	-	Adenylate cyclase (EC 4.6.1.1)	FIG00001294	idu(25);cAMP_signaling_in_bacteria
fig|6666666.28482.peg.3049	Contig_31	69155	67605	-	transcriptional regulator, LuxR family protein	FIG01428339	ff
fig|6666666.28482.peg.3050	Contig_31	72167	69318	-	Protein export cytoplasm protein SecA ATPase RNA helicase (TC 3.A.5.1.1)	FIG00034293	ff
fig|6666666.28482.peg.3051	Contig_31	72971	72264	-	Ribosomal subunit interface protein	FIG00001306	isu;Ribosome_activity_modulation
fig|6666666.28482.peg.3052	Contig_31	73928	73287	-	Competence protein F homolog, phosphoribosyltransferase domain; protein YhgH required for utilization of DNA as sole source of carbon and energy	FIG00004440	isu;Biotin_biosynthesis_Experimental isu;CBSS-216591.1.peg.168
fig|6666666.28482.peg.3053	Contig_31	75269	73974	-	FIG00467522: hypothetical protein	FIG00467521	ff
fig|6666666.28482.peg.3054	Contig_31	75310	75429	+	hypothetical protein		
fig|6666666.28482.peg.3055	Contig_31	77544	75787	-	LpqB	FIG00863150	ff
fig|6666666.28482.peg.3056	Contig_31	79124	77544	-	Sensor histidine kinase MtrB (EC 2.7.3.-)	FIG00868343	ff

fig|6666666.28482.peg.3057	Contig_31	79911	79225	-	DNA-binding response regulator mtrA	FIG00545527	ff
fig|6666666.28482.peg.3058	Contig_31	80624	79992	-	Thymidylate kinase (EC 2.7.4.9)	FIG00000175	idu(1);pyrimidine_conversions idu(1);CBSS-393133.3.peg.2787
fig|6666666.28482.peg.3059	Contig_31	82188	80716	-	Adenosylhomocysteinase (EC 3.3.1.1)	FIG00000858	ff
fig|6666666.28482.peg.3060	Contig_31	82894	82247	-	Transcriptional regulator, TetR family	FIG00554417	ff
fig|6666666.28482.peg.3061	Contig_31	83103	82921	-	Rubredoxin	FIG01955902	icw(1);Rubrerythrin
fig|6666666.28482.peg.3062	Contig_31	83273	83100	-	Rubredoxin	FIG01955902	icw(1);Rubrerythrin
fig|6666666.28482.peg.3063	Contig_31	84493	83270	-	Alkane-1 monooxygenase (EC 1.14.15.3)	FIG01363988	ff
fig|6666666.28482.peg.3064	Contig_31	86176	84668	-	Amino acid permease	FIG01345058	ff
fig|6666666.28482.peg.3065	Contig_31	86270	87649	+	putative secreted protein	FIG00905474	ff
fig|6666666.28482.peg.3066	Contig_31	88853	87627	-	Mannose-6-phosphate isomerase (EC 5.3.1.8)	FIG00598409	isu;Mannose_Metabolism
fig|6666666.28482.peg.3067	Contig_31	89401	88862	-	FIG00692620: hypothetical protein	FIG00692617	ff
fig|6666666.28482.peg.3068	Contig_32	1509	16	-	FIG00826601: hypothetical protein	FIG00826600	ff
fig|6666666.28482.peg.3069	Contig_32	1878	3209	+	metabolite-proton symporter	FIG00994071	ff

fig|6666666.28482.peg.3070	Contig_32	3435	4661	+	3-ketoacyl-CoA thiolase (EC 2.3.1.16)	FIG01279374	idu(11);CBSS-246196.1.peg.364 idu(11);Serine-glyoxylate_cycle idu(11);n-Phenylalkanoic_acid_degradation idu(11);Polyhydroxybutyrate_metabolism idu(11);Isoleucine_degradation idu(11);Fatty_acid_metabolism_cluster idu(11);Biotin_synthesis_cluster idu(11);Biotin_biosynthesis
fig|6666666.28482.peg.3071	Contig_32	4664	6178	+	Lignostilbene-alpha,beta-dioxygenase and related enzymes	FIG01358959	ff
fig|6666666.28482.peg.3072	Contig_32	6175	6663	+	FIG00826613: hypothetical protein	FIG00826612	ff
fig|6666666.28482.peg.3073	Contig_32	7092	6667	-	PROBABLE CONSERVED TRANSMEMBRANE PROTEIN	FIG00662132	ff
fig|6666666.28482.peg.3074	Contig_32	7960	7142	-	putative hydroxylase	FIG01122344	ff
fig|6666666.28482.peg.3075	Contig_32	8476	8003	-	FIG00820677: hypothetical protein	FIG00820675	ff
fig|6666666.28482.peg.3076	Contig_32	8667	8482	-	FIG00821394: hypothetical protein	FIG00821392	ff
fig|6666666.28482.peg.3077	Contig_32	11090	8703	-	Cation-transporting ATPase, E1-E2 family	FIG01322067	ff
fig|6666666.28482.peg.3078	Contig_32	12676	11087	-	Beta-lactamase (EC 3.5.2.6)	FIG00000747	idu(1);Beta-lactamase idu(1);Tn552
fig|6666666.28482.peg.3079	Contig_32	12982	13506	+	unknown		

fig|6666666.28482.peg.3080	Contig_32	14629	13511	-	Outer membrane protein romA	FIG01371060	ff
fig|6666666.28482.peg.3081	Contig_32	15369	14635	-	Enoyl-CoA hydratase (EC 4.2.1.17)	FIG00033206	idu(51);n-Phenylalkanoic_acid_degradation idu(51);Polyhydroxybutyrate_metabolism idu(51);Isoleucine_degradation idu(51);Fatty_acid_metabolism_cluster idu(51);Lysine_fermentation idu(51);Butanol_Biosynthesis idu(51);Acetyl-CoA_fermentation_to_Butyrate idu(51);Valine_degradation idu(51);Butyrate_metabolism_cluster
fig|6666666.28482.peg.3082	Contig_32	15432	16892	+	Acetyl-coenzyme A carboxyl transferase alpha chain (EC 6.4.1.2) / Acetyl-coenzyme A carboxyl transferase beta chain (EC 6.4.1.2)	FIG01303802	isu;Fatty_Acid_Biosynthesis_FASII isu;Fatty_Acid_Biosynthesis_FASII
fig|6666666.28482.peg.3083	Contig_32	17499	16930	-	Transcriptional regulator, TetR family	FIG00554417	ff
fig|6666666.28482.peg.3084	Contig_32	17602	18741	+	pentachlorophenol 4-monooxygenase		
fig|6666666.28482.peg.3085	Contig_32	18800	19510	+	DNA-binding response regulator	FIG01031438	ff
fig|6666666.28482.peg.3086	Contig_32	19588	20877	+	Sensor-type histidine kinase prrB (EC 2.7.13.3)	FIG01405604	ff

fig|6666666.28482.peg.3087	Contig_32	22158	20887	-	POSSIBLE CONSERVED EXPORTED OR MEMBRANE PROTEIN	FIG01334926	ff
fig|6666666.28482.peg.3088	Contig_32	22304	22155	-	hypothetical protein		
fig|6666666.28482.peg.3089	Contig_32	23296	22316	-	Outer membrane protein	FIG01111221	ff
fig|6666666.28482.peg.3090	Contig_32	23404	23667	+	FIG00821893: hypothetical protein	FIG00821892	ff
fig|6666666.28482.peg.3091	Contig_32	23670	25253	+	Beta-carotene ketolase (EC 1.14.-.-)	FIG00071345	idu(3);Proteorhodopsin
fig|6666666.28482.peg.3092	Contig_32	26590	25295	-	Citrate synthase (si) (EC 2.3.3.1)	FIG00130344	icw(1);Serine-glyoxylate_cycle icw(1);TCA_Cycle icw(1);Glyoxylate_bypass
fig|6666666.28482.peg.3093	Contig_32	27394	26768	-	Pyridoxamine 5'-phosphate oxidase (EC 1.4.3.5)	FIG00133002	isu;Pyridoxin_(Vitamin_B6)_Biosynthesis
fig|6666666.28482.peg.3094	Contig_32	27545	28621	+	Citrate synthase (si) (EC 2.3.3.1)	FIG00130344	icw(1);Serine-glyoxylate_cycle icw(1);TCA_Cycle icw(1);Glyoxylate_bypass
fig|6666666.28482.peg.3095	Contig_33	14	1762	+	Long-chain-fatty-acid--CoA ligase (EC 6.2.1.3)	FIG00018699	idu(72);n-Phenylalkanoic_acid_degradation idu(72);Fatty_acid_metabolism_cluster idu(72);Biotin_synthesis_cluster idu(72);Biotin_biosynthesis
fig|6666666.28482.peg.3096	Contig_33	1785	2453	+	FIG00830296: hypothetical protein	FIG00830295	ff
fig|6666666.28482.peg.3097	Contig_33	2480	3247	+	Putative uncharacterized protein BCG_1966	FIG01319018	ff

fig|6666666.28482.peg.3098	Contig_33	4028	3267	-	Probable short-chain type dehydrogenase/reductase (EC 1.-.-.-)	FIG00026991	idu(2);CBSS-164757.7.peg.5020
fig|6666666.28482.peg.3099	Contig_33	4092	4568	+	FIG00825523: hypothetical protein	FIG00825522	ff
fig|6666666.28482.peg.3100	Contig_33	4657	5142	+	hypothetical protein		
fig|6666666.28482.peg.3101	Contig_33	5891	5139	-	ThiJ/PfpI family protein	FIG00015923	idu(8);CBSS-176280.1.peg.1561
fig|6666666.28482.peg.3102	Contig_33	6860	5910	-	Transcriptional regulator, AraC family	FIG00510130	ff
fig|6666666.28482.peg.3103	Contig_33	6971	7465	+	Thiol peroxidase, Tpx-type (EC 1.11.1.15)	FIG00002857	isu;CBSS-257314.1.peg.752 isu;Thioredoxin-disulfide_reductase
fig|6666666.28482.peg.3104	Contig_33	7591	7896	+	hypothetical protein		
fig|6666666.28482.peg.3105	Contig_33	8845	7877	-	Enoyl-[acyl-carrier-protein] reductase [FMN] (EC 1.3.1.9)	FIG00007641	idu(1);Fatty_Acid_Biosynthesis_FASII
fig|6666666.28482.peg.3106	Contig_33	9222	8845	-	hypothetical protein		
fig|6666666.28482.peg.3107	Contig_33	9431	10615	+	FIG00830011: hypothetical protein	FIG00830008	ff
fig|6666666.28482.peg.3108	Contig_33	10837	10610	-	hypothetical protein		
fig|6666666.28482.peg.3109	Contig_33	10853	12310	+	FIG00825509: hypothetical protein	FIG00825507	ff
fig|6666666.28482.peg.3110	Contig_33	12330	13688	+	FIG00825270: hypothetical protein	FIG00825268	ff
fig|6666666.28482.peg.3111	Contig_33	13711	15096	+	hypothetical protein		
fig|6666666.28482.peg.3112	Contig_33	15351	18377	+	Putative membrane protein		
fig|6666666.28482.peg.3113	Contig_33	18983	19975	+	Saccharopine dehydrogenase		
fig|6666666.28482.peg.3114	Contig_33	19972	20721	+	hypothetical protein		

fig|6666666.28482.peg.3115	Contig_33	21488	20805	-	Response regulator CitB of citrate metabolism	FIG00229361	ff
fig|6666666.28482.peg.3116	Contig_33	23155	21470	-	hypothetical protein	FIG00638284	ff
fig|6666666.28482.peg.3117	Contig_33	23217	24143	+	FIG00821999: hypothetical protein	FIG00821997	ff
fig|6666666.28482.peg.3118	Contig_33	24981	24160	-	alpha/beta hydrolase fold		
fig|6666666.28482.peg.3119	Contig_33	25095	26456	+	FIG00822747: hypothetical protein	FIG00822745	ff
fig|6666666.28482.peg.3120	Contig_33	26449	27303	+	PROBABLE MEMBRANE PROTEIN	FIG00816212	ff
fig|6666666.28482.peg.3121	Contig_33	27333	27779	+	Transcriptional regulator, AraC family	FIG00510130	ff
fig|6666666.28482.peg.3122	Contig_33	27784	28194	+	putative lyase	FIG00999432	ff
fig|6666666.28482.peg.3123	Contig_33	28519	28265	-	hypothetical protein		
fig|6666666.28482.peg.3124	Contig_33	30058	28616	-	D-serine/D-alanine/glycine transporter	FIG00003984	isu;Pyruvate_Alanine_Serine_Interconversions
fig|6666666.28482.peg.3125	Contig_33	30384	30115	-	iron-regulated elongation factor tu Tuf-like	FIG00823055	ff
fig|6666666.28482.peg.3126	Contig_33	31402	30389	-	Quinone oxidoreductase (EC 1.6.5.5)	FIG00002982	idu(7);Quinone_oxidoreductase_family
fig|6666666.28482.peg.3127	Contig_33	31481	32140	+	FIG00827117: hypothetical protein	FIG00827116	ff
fig|6666666.28482.peg.3128	Contig_33	32222	32893	+	FIG00827117: hypothetical protein	FIG00827116	ff
fig|6666666.28482.peg.3129	Contig_33	32923	33768	+	putative methyltransferase	FIG01125506	ff
fig|6666666.28482.peg.3130	Contig_33	34582	33770	-	FIG00827721: hypothetical protein	FIG00827719	ff

fig|6666666.28482.peg.3131	Contig_33	34837	35034	+	hypothetical protein		
fig|6666666.28482.peg.3132	Contig_33	35167	35367	+	hypothetical protein		
fig|6666666.28482.peg.3133	Contig_33	36018	35377	-	hypothetical protein	FIG00829159	ff
fig|6666666.28482.peg.3134	Contig_33	36122	36796	+	putative secreted protein	FIG00905474	ff
fig|6666666.28482.peg.3135	Contig_33	37853	36783	-	FIG00822799: hypothetical protein	FIG00822798	ff
fig|6666666.28482.peg.3136	Contig_33	38610	37840	-	putative methyltransferase	FIG01125506	ff
fig|6666666.28482.peg.3137	Contig_33	39833	38604	-	Glycosyltransferase	FIG00007960	ff
fig|6666666.28482.peg.3138	Contig_33	39795	39962	+	hypothetical protein		
fig|6666666.28482.peg.3139	Contig_33	40411	40031	-	Chitinase C precursor (EC 3.2.1.14)	FIG01355520	ff
fig|6666666.28482.peg.3140	Contig_33	40560	41375	+	FIG00824527: hypothetical protein	FIG00824526	ff
fig|6666666.28482.peg.3141	Contig_33	42281	41394	-	Alpha/beta hydrolase fold (EC 3.8.1.5)		
fig|6666666.28482.peg.3142	Contig_33	44690	42456	-	Catalase (EC 1.11.1.6) / Peroxidase (EC 1.11.1.7)	FIG00000907	icw(1);Oxidative_stress icw(1);Oxidative_stress idu(3);Photorespiration_(oxidative_C2_cycle) idu(1);Protection_from_Reactive_Oxygen_Species idu(3);Protection_from_Reactive_Oxygen_Species idu(1);Auxin_degradation
fig|6666666.28482.peg.3143	Contig_33	45179	44739	-	Transcriptional regulator, FUR family	FIG00002441	idu(2);Oxidative_stress
fig|6666666.28482.peg.3144	Contig_33	46592	45327	-	ErfK/YbiS/YcfS/YnhG family protein	FIG01228918	ff
fig|6666666.28482.peg.3145	Contig_33	46663	47424	+	FIG00830083: hypothetical protein	FIG00830082	ff

fig|6666666.28482.peg.3146	Contig_33	47484	49013	+	FAD dependent oxidoreductase	FIG00453665	ff
fig|6666666.28482.peg.3147	Contig_33	50061	49018	-	FIG00823150: hypothetical protein	FIG00823149	ff
fig|6666666.28482.peg.3148	Contig_33	50282	51631	+	PPE family protein	FIG00013075	idu(57);CBSS-164757.7.peg.5020
fig|6666666.28482.peg.3149	Contig_33	51945	52229	+	hypothetical protein		
fig|6666666.28482.peg.3150	Contig_33	52308	53111	+	Mlr4090 protein	FIG01358600	ff
fig|6666666.28482.peg.3151	Contig_33	53399	53112	-	hypothetical protein	FIG00638284	ff
fig|6666666.28482.peg.3152	Contig_33	53957	53403	-	Alkyl hydroperoxide reductase and/or thiol-specific antioxidant family (AhpC/TSA) protein		
fig|6666666.28482.peg.3153	Contig_33	54572	53979	-	FIG00822146: hypothetical protein	FIG00822145	ff
fig|6666666.28482.peg.3154	Contig_33	55259	54630	-	FIG00827381: hypothetical protein	FIG00827380	ff
fig|6666666.28482.peg.3155	Contig_33	56118	55348	-	FIG00823638: hypothetical protein	FIG00823636	ff
fig|6666666.28482.peg.3156	Contig_33	56291	57895	+	PPE family protein	FIG00013075	idu(57);CBSS-164757.7.peg.5020
fig|6666666.28482.peg.3157	Contig_33	58076	59014	+	hypothetical protein	FIG00638284	ff
fig|6666666.28482.peg.3158	Contig_33	59077	59976	+	FIG00823859: hypothetical protein	FIG00823854	ff
fig|6666666.28482.peg.3159	Contig_33	63609	60175	-	Adenylyl cyclase class-3/4/guanylyl cyclase / Disease resistance domain-containing protein / Tetratricopeptide repeat-containing protein / Transcriptional regulator, LuxR family	FIG01386132	ff

fig|6666666.28482.peg.3160	Contig_33	63734	64972	+	putative cytochrome P450 hydroxylase	FIG00032437	idu(49);Nitric_oxide_synthase
fig|6666666.28482.peg.3161	Contig_33	66386	65364	-	FIG00822630: hypothetical protein	FIG00822629	ff
fig|6666666.28482.peg.3162	Contig_33	66718	66509	-	hypothetical protein		
fig|6666666.28482.peg.3163	Contig_33	66707	68176	+	2-polyprenyl-6-methoxyphenol hydroxylase and related FAD-dependent oxidoreductases	FIG00453279	ff
fig|6666666.28482.peg.3164	Contig_33	68358	68185	-	hypothetical protein		
fig|6666666.28482.peg.3165	Contig_33	68454	69953	+	FIG00832063: hypothetical protein	FIG00832061	ff
fig|6666666.28482.peg.3166	Contig_33	71182	69950	-	FIG00831158: hypothetical protein	FIG00831154	ff
fig|6666666.28482.peg.3167	Contig_33	71273	72151	+	Citrate lyase beta chain (EC 4.1.3.6)	FIG00010100	idu(2);Citrate_Metabolism,_Transport,_and_Regulation
fig|6666666.28482.peg.3168	Contig_33	72267	72653	+	hypothetical protein		
fig|6666666.28482.peg.3169	Contig_33	73259	72669	-	cAMP-binding proteins - catabolite gene activator and regulatory subunit of cAMP-dependent protein kinases	FIG00003090	idu(1);cAMP_signaling_in_bacteria idu(1);CBSS-479431.5.peg.3955
fig|6666666.28482.peg.3170	Contig_33	74141	73362	-	Ribulosamine/erythrulosamine 3-kinase potentially involved in protein deglycation	FIG00002860	isu;Protein_deglycation
fig|6666666.28482.peg.3171	Contig_33	74258	75493	+	FIG00824986: hypothetical protein	FIG00824984	ff
fig|6666666.28482.peg.3172	Contig_34	453	1202	+	hypothetical protein		
fig|6666666.28482.peg.3173	Contig_34	1199	2041	+	hypothetical protein	FIG00638284	ff

fig|6666666.28482.peg.3174	Contig_34	2069	3658	+	hypothetical protein	FIG00638284	ff
fig|6666666.28482.rna.34	Contig_34	3811	3739	-	tRNA-Arg-ACG		isu;tRNAs
fig|6666666.28482.rna.35	Contig_34	3935	3847	-	tRNA-Ser-GCT		
fig|6666666.28482.peg.3175	Contig_34	4025	5101	+	Biosynthetic Aromatic amino acid aminotransferase beta (EC 2.6.1.57)	FIG00032670	idu(1);Phenylalanine_and_Tyrosine_Branches_from_Chorismate
fig|6666666.28482.peg.3176	Contig_34	5098	5559	+	FIG00820948: hypothetical protein	FIG00820947	ff
fig|6666666.28482.peg.3177	Contig_34	6198	5614	-	Transcriptional regulator, ArsR family	FIG00003845	idu(3);CBSS-1085.1.peg.1363
fig|6666666.28482.peg.3178	Contig_34	6222	7046	+	Enoyl-CoA hydratase	FIG00820022	ff
fig|6666666.28482.peg.3179	Contig_34	7072	8310	+	Beta-lactamase	FIG00021206	isu;Beta-lactamase
fig|6666666.28482.peg.3180	Contig_34	8341	8532	+	FIG00825779: hypothetical protein	FIG00825778	ff
fig|6666666.28482.peg.3181	Contig_34	8568	9674	+	FIG00820291: hypothetical protein	FIG00820290	ff
fig|6666666.28482.peg.3182	Contig_34	10416	10279	-	hypothetical protein		
fig|6666666.28482.peg.3183	Contig_34	11403	11519	+	hypothetical protein		
fig|6666666.28482.peg.3184	Contig_34	12993	12826	-	hypothetical protein		
fig|6666666.28482.peg.3185	Contig_34	13698	14291	+	hypothetical protein		
fig|6666666.28482.peg.3186	Contig_34	14783	14902	+	hypothetical protein		
fig|6666666.28482.peg.3187	Contig_34	15348	15091	-	hypothetical protein		
fig|6666666.28482.peg.3188	Contig_34	17494	15857	-	hypothetical protein		
fig|6666666.28482.peg.3189	Contig_34	19488	17779	-	ATP-dependent DNA helicase UvrD/PcrA	FIG00000372	idu(1);CBSS-393121.3.peg.1913 idu(1);DNA_repair,_bacterial_UvrD_and_related_helicases
fig|6666666.28482.peg.3190	Contig_34	21326	19485	-	hypothetical protein	FIG00638284	ff
fig|6666666.28482.peg.3191	Contig_34	21959	21735	-	hypothetical protein		
fig|6666666.28482.peg.3192	Contig_34	22593	22868	+	hypothetical protein		
fig|6666666.28482.peg.3193	Contig_34	24336	23227	-	putative ATPase	FIG01449448	ff
fig|6666666.28482.peg.3194	Contig_34	26572	25460	-	Arsenical-resistance protein ACR3	FIG00008889	isu;Arsenic_resistance

fig|6666666.28482.peg.3195	Contig_34	26770	27006	+	hypothetical protein		
fig|6666666.28482.peg.3196	Contig_34	27584	27111	-	Lactoylglutathione lyase (EC 4.4.1.5)	FIG00004743	idu(1);Glutathione:_Non-redox_reactions idu(1);Methylglyoxal_Metabolism
fig|6666666.28482.peg.3197	Contig_34	27689	28048	+	regulatory protein, ArsR		
fig|6666666.28482.peg.3198	Contig_34	28233	28901	+	Arsenical resistance operon repressor / Arsenate reductase (EC 1.20.4.1)		idu(5);Transcription_repair_cluster idu(5);CBSS-315749.4.peg.3658 icw(1);Arsenic_resistance icw(1);Arsenic_resistance idu(5);Anaerobic_respiratory_reductases
fig|6666666.28482.peg.3199	Contig_34	29074	30033	+	Phosphate ABC transporter, periplasmic phosphate-binding protein PstS (TC 3.A.1.7.1)	FIG00010508	idu(4);High_affinity_phosphate_transporter_and_control_of_PHO_regulon idu(4);Phosphate_metabolism idu(4);PhoR-PhoB_two-component_regulatory_system
fig|6666666.28482.peg.3200	Contig_34	30105	31559	+	NADH oxidase (putative)	FIG01704807	ff
fig|6666666.28482.peg.3201	Contig_34	33192	34709	+	dNTP triphosphohydrolase, broad substrate specificity, subgroup 2		isu;Purine_conversions isu;dNTP_triphosphohydrolase_protein_family
fig|6666666.28482.peg.3202	Contig_34	36030	35512	-	hypothetical protein	FIG00638284	ff
fig|6666666.28482.peg.3203	Contig_34	36240	36524	+	hypothetical protein		
fig|6666666.28482.peg.3204	Contig_34	36524	36775	+	hypothetical protein		
fig|6666666.28482.peg.3205	Contig_34	36831	37040	+	hypothetical protein		
fig|6666666.28482.peg.3206	Contig_34	37349	37468	+	hypothetical protein		
fig|6666666.28482.peg.3207	Contig_34	37461	39662	+	hypothetical protein	FIG00638284	ff
fig|6666666.28482.peg.3208	Contig_34	39659	40636	+	hypothetical protein	FIG00638284	ff

fig|6666666.28482.peg.3209	Contig_34	40678	41661	+	DNA methylase N-4/N-6		
fig|6666666.28482.peg.3210	Contig_34	41748	42101	+	hypothetical protein	FIG00638284	ff
fig|6666666.28482.peg.3211	Contig_34	42520	42771	+	hypothetical protein		
fig|6666666.28482.rna.36	Contig_34	45390	45304	-	tRNA-Ser-TGA		
fig|6666666.28482.peg.3212	Contig_34	45428	46396	+	Quinone oxidoreductase (EC 1.6.5.5)	FIG00002982	idu(7);Quinone_oxidoreductase_family
fig|6666666.28482.peg.3213	Contig_34	47716	46511	-	Cysteine desulfurase (EC 2.8.1.7)	FIG00000001	idu(2);Iron-sulfur_cluster_assembly idu(2);CBSS-84588.1.peg.1247 idu(2);CBSS-393130.3.peg.794 idu(2);mnm5U34_biosynthesis_bacteria idu(2);Alanine_biosynthesis
fig|6666666.28482.peg.3214	Contig_34	47830	49800	+	FIG00820726: hypothetical protein	FIG00820725	ff
fig|6666666.28482.peg.3215	Contig_34	49813	50358	+	FIG00822472: hypothetical protein	FIG00822465	ff
fig|6666666.28482.peg.3216	Contig_34	50363	51172	+	O-antigen export system, ATP-binding protein	FIG00548123	ff
fig|6666666.28482.peg.3217	Contig_34	51169	52095	+	Putative glycosyl transferase	FIG01195418	ff
fig|6666666.28482.peg.3218	Contig_34	52092	52922	+	O-antigen export system permease protein RfbD	FIG00521220	ff
fig|6666666.28482.peg.3219	Contig_34	52928	53296	+	FIG00827542: hypothetical protein		
fig|6666666.28482.peg.3220	Contig_34	54442	53462	-	hypothetical protein	FIG00638284	ff
fig|6666666.28482.peg.3221	Contig_34	54913	55452	+	Queuosine biosynthesis QueD, PTPS-I		

fig|6666666.28482.peg.3222	Contig_34	55624	56103	+	Nucleoside diphosphate kinase regulator	FIG01320511	ff
fig|6666666.28482.peg.3223	Contig_34	56185	56571	+	Possible membrane protein	FIG01121868	ff
fig|6666666.28482.peg.3224	Contig_34	56654	58024	+	Oxidoreductase, FAD-binding protein	FIG00546255	ff
fig|6666666.28482.peg.3225	Contig_34	58051	58818	+	3-oxoacyl-[acyl-carrier protein] reductase paralog (EC 1.1.1.100)	FIG00763697	ff
fig|6666666.28482.peg.3226	Contig_34	58821	60767	+	putative membrane protein	FIG00672847	ff
fig|6666666.28482.peg.3227	Contig_34	60767	64033	+	INTEGRAL MEMBRANE INDOLYLACETYLINOSITOL ARABINOSYLTRANSFERASE EMBC (ARABINOSYLINDOLYLACETYLINOSITOL SYNTHASE)	FIG01954080	ff
fig|6666666.28482.peg.3228	Contig_34	64157	64417	+	COG0640: Predicted transcriptional regulators		
fig|6666666.28482.peg.3229	Contig_34	64423	64896	+	Putative uncharacterized protein BCG_2054	FIG00826548	ff
fig|6666666.28482.peg.3230	Contig_34	64893	65516	+	FIG00821723: hypothetical protein	FIG00821722	ff
fig|6666666.28482.peg.3231	Contig_34	66663	65524	-	EmbR protein	FIG01423950	ff
fig|6666666.28482.peg.3232	Contig_34	66856	70080	+	Probable arabinosyltransferase A (EC 2.4.2.-)	FIG01331801	ff

fig|6666666.28482.peg.3233	Contig_34	70077	73325	+	Probable arabinosyltransferase B (EC 2.4.2.-)	FIG00820183	ff
fig|6666666.28482.peg.3234	Contig_34	73531	73998	+	hypothetical protein		
fig|6666666.28482.peg.3235	Contig_34	74180	73968	-	hypothetical protein		
fig|6666666.28482.peg.3236	Contig_34	74372	74839	+	hypothetical protein	FIG00638284	ff
fig|6666666.28482.peg.3237	Contig_34	74836	75252	+	hypothetical protein	FIG00638284	ff
fig|6666666.28482.peg.3238	Contig_34	75265	77049	+	Probable acyl-CoA dehydrogenase fadE35 (EC 1.3.99.-)		ff
fig|6666666.28482.peg.3239	Contig_34	78698	77118	-	Propionyl-CoA carboxylase beta chain (EC 6.4.1.3)	FIG01304918	idu(2);Mycobacterium_virulence_operon_involved_in_fatty_acids_biosynthesis idu(2);Serine-glyoxylate_cycle
fig|6666666.28482.peg.3240	Contig_34	84039	78706	-	Probable polyketide synthase, similar to many. e.g. gp|M63676|SERERYAA_1 S.erythraea first ORF of eryA gene, involved in complex polyketide formation in erythromycin biosynthesis.		ff
fig|6666666.28482.peg.3241	Contig_34	85948	84050	-	Long-chain-fatty-acid--CoA ligase (EC 6.2.1.3)	FIG00018699	idu(72);n-Phenylalkanoic_acid_degradation idu(72);Fatty_acid_metabolism_cluster idu(72);Biotin_synthesis_cluster idu(72);Biotin_biosynthesis
fig|6666666.28482.peg.3242	Contig_34	87223	86273	-	hypothetical protein Rv3802c	FIG00018712	ff

fig|6666666.28482.peg.3243	Contig_34	87254	87409	+	hypothetical protein		
fig|6666666.28482.peg.3244	Contig_34	88290	87406	-	Antigen 85-C precursor (85C) (Antigen 85 complex C) (Ag85C) (Mycolyl transferase 85C) (EC 2.3.1.-)	FIG00027986	ff
fig|6666666.28482.peg.3245	Contig_34	89430	88480	-	Esterase, secreted fibronectin-binding protein antigen 85-B FbpB	FIG00820332	ff
fig|6666666.28482.peg.3246	Contig_34	91798	89861	-	hypothetical protein Rv3805c	FIG00010871	ff
fig|6666666.28482.peg.3247	Contig_34	92738	91830	-	POSSIBLE CONSERVED INTEGRAL MEMBRANE PROTEIN	FIG01037873	ff
fig|6666666.28482.peg.3248	Contig_34	93258	92731	-	FIG008913: Membrane-associated phospholipid phosphatase	FIG00485540	ff
fig|6666666.28482.peg.3249	Contig_34	95143	93251	-	Galactofuranosyl transferase (EC 2.-.-.-)	FIG00511876	ff
fig|6666666.28482.peg.3250	Contig_34	96422	95223	-	UDP-galactopyranose mutase (EC 5.4.99.9)	FIG00003472	isu;LOS_core_oligosaccharide_biosynthesis
fig|6666666.28482.peg.3251	Contig_35	35	373	+	Phosphoglycolate phosphatase (EC 3.1.3.18)	FIG00003555	isu;Glycolate,_glyoxylate_interconversions isu;Photorespiration_(oxidative_C2_cycle) isu;2-phosphoglycolate_salvage
fig|6666666.28482.peg.3252	Contig_35	366	869	+	Low molecular weight protein tyrosine phosphatase (EC 3.1.3.48)	FIG00059124	isu;Protein_deglycation isu;LMPTP_YfkJ_cluster isu;CBSS-176280.1.peg.1561

fig|6666666.28482.peg.3253	Contig_35	866	1678	+	Cytochrome oxidase biogenesis protein Surf1, facilitates heme A insertion	FIG00018846	isu;CBSS-316057.3.peg.563 isu;Biogenesis_of_cytochrome_c_oxidases
fig|6666666.28482.peg.3254	Contig_35	2691	1759	-	Adenosylcobinamide-phosphate synthase	FIG00000689	isu;Cobalamin_synthesis isu;Coenzyme_B12_biosynthesis
fig|6666666.28482.peg.3255	Contig_35	2872	2732	-	hypothetical protein		
fig|6666666.28482.peg.3256	Contig_35	2949	3764	+	hypothetical protein Rv2237	FIG01319570	ff
fig|6666666.28482.peg.3257	Contig_35	4981	3848	-	Epoxide hydrolase (EC 3.3.2.9)	FIG00009455	idu(3);CBSS-410289.13.peg.3174
fig|6666666.28482.peg.3258	Contig_35	5900	5007	-	Sulfatase modifying factor 1 precursor (C-alpha-formyglycine-generating enzyme 1)	FIG00003489	idu(1);Sulfatases_and_sulfatase_modifying_factor_1
fig|6666666.28482.peg.3259	Contig_35	6822	5893	-	FIG068086: hypothetical protein	FIG00068086	icw(1);Sulfatases_and_sulfatase_modifying_factor_1
fig|6666666.28482.peg.3260	Contig_35	8366	6819	-	Arylsulfatase (EC 3.1.6.1)	FIG00016566	icw(2);Sulfatases_and_sulfatase_modifying_factor_1 idu(3);Galactosylceramide_and_Sulfatide_metabolism idu(3);Steroid_sulfates idu(3);Alkanesulfonate_assimilation
fig|6666666.28482.peg.3261	Contig_35	8813	8505	-	hypothetical protein		
fig|6666666.28482.peg.3262	Contig_35	10029	9127	-	hypothetical protein	FIG00638284	ff
fig|6666666.28482.peg.3263	Contig_35	10499	10191	-	hypothetical protein		
fig|6666666.28482.peg.3264	Contig_35	10498	10626	+	hypothetical protein		
fig|6666666.28482.peg.3265	Contig_35	13718	10788	-	Putative membrane protein	FIG01056920	ff
fig|6666666.28482.peg.3266	Contig_35	14305	13715	-	membrane protein, MmpS family	FIG01309473	ff

fig|6666666.28482.peg.3267	Contig_35	14931	14329	-	Transcriptional regulator, TetR family	FIG00554417	ff
fig|6666666.28482.rna.37	Contig_35	15344	15273	-	tRNA-Val-TAC		
fig|6666666.28482.peg.3268	Contig_35	15871	15410	-	Alkyl hydroperoxide reductase subunit C-like protein	FIG01258694	idu(1);Oxidative_stress idu(1);Rubrerythrin idu(1);Thioredoxin-disulfide_reductase
fig|6666666.28482.peg.3269	Contig_35	16293	15871	-	FIG00819990: hypothetical protein	FIG00819989	ff
fig|6666666.28482.peg.3270	Contig_35	16890	16387	-	FIG00820323: hypothetical protein	FIG00820322	ff
fig|6666666.28482.peg.3271	Contig_35	17094	19883	+	Pyruvate dehydrogenase E1 component (EC 1.2.4.1)	FIG00137886	isu;Pyruvate_metabolism_II:_acetyl-CoA,_acetogenesis_from_pyruvate isu;Dehydrogenase_complexes
fig|6666666.28482.peg.3272	Contig_35	20083	21408	+	Regulator of polyketide synthase expression	FIG00056421	ff
fig|6666666.28482.peg.3273	Contig_35	21549	22457	+	Malonyl CoA-acyl carrier protein transacylase (EC 2.3.1.39)	FIG00000319	icw(3);Fatty_Acid_Biosynthesis_FASII
fig|6666666.28482.peg.3274	Contig_35	22538	22885	+	Acyl carrier protein	FIG00002328	icw(2);Fatty_Acid_Biosynthesis_FASII idu(2);Glycerolipid_and_Glycerophospholipid_Metabolism_in_Bacteria
fig|6666666.28482.peg.3275	Contig_35	22882	24132	+	3-oxoacyl-[acyl-carrier-protein] synthase, KASII (EC 2.3.1.41)	FIG00002246	icw(1);Mycobacterium_virulence_operon_involved_in_fatty_acids_biosynthesis icw(1);Fatty_Acid_Biosynthesis_FASII

fig|6666666.28482.peg.3276	Contig_35	24253	25467	+	3-oxoacyl-[acyl-carrier-protein] synthase, KASII (EC 2.3.1.41)	FIG00002246	icw(1);Mycobacterium_virulence_operon_involved_in_fatty_acids_biosynthesis icw(1);Fatty_Acid_Biosynthesis_FASII
fig|6666666.28482.peg.3277	Contig_35	25512	26933	+	Propionyl-CoA carboxylase beta chain (EC 6.4.1.3)	FIG01304918	icw(2);Mycobacterium_virulence_operon_involved_in_fatty_acids_biosynthesis idu(2);Serine-glyoxylate_cycle
fig|6666666.28482.peg.3278	Contig_35	27635	27952	+	FIG00821976: hypothetical protein	FIG00821975	ff
fig|6666666.28482.peg.3279	Contig_35	28771	28028	-	hypothetical protein	FIG00638284	ff
fig|6666666.28482.peg.3280	Contig_35	30378	28768	-	FAD-binding monooxygenase, PheA/TfdB family, similarity to 2,4-dichlorophenol 6-monooxygenase	FIG01103239	ff
fig|6666666.28482.peg.3281	Contig_35	31086	30457	-	Transcriptional regulator, TetR family	FIG00554417	ff
fig|6666666.28482.peg.3282	Contig_35	32657	31128	-	Glycerol-3-phosphate dehydrogenase (EC 1.1.5.3)	FIG00229386	idu(1);Respiratory_dehydrogenases_1 idu(1);Glycerol_and_Glycerol-3-phosphate_Uptake_and_Utilization idu(1);Glycerolipid_and_Glycerophospholipid_Metabolism_in_Bacteria
fig|6666666.28482.peg.3283	Contig_35	33223	32654	-	Transcriptional regulator, TetR family	FIG00554417	ff
fig|6666666.28482.peg.3284	Contig_35	33241	34851	+	Flavoprotein	FIG00661484	ff
fig|6666666.28482.peg.3285	Contig_35	34848	35762	+	Diacylglycerol kinase-related protein	FIG01037700	ff

fig|6666666.28482.peg.3286	Contig_35	36773	35763	-	hypothetical protein	FIG00638284	ff
fig|6666666.28482.peg.3287	Contig_35	36888	37754	+	hypothetical protein	FIG00638284	ff
fig|6666666.28482.peg.3288	Contig_35	38297	37764	-	FIG00820627: hypothetical protein	FIG00820624	ff
fig|6666666.28482.peg.3289	Contig_35	39224	38400	-	Beta-lactamase class C and other penicillin binding proteins	FIG00000681	idu(2);Beta-lactamase
fig|6666666.28482.peg.3290	Contig_35	40240	39221	-	Possible transcriptional regulatory protein	FIG01311821	ff
fig|6666666.28482.peg.3291	Contig_35	40433	41506	+	Formaldehyde dehydrogenase MscR, NAD/mycothiol-dependent (EC 1.2.1.66) / S-nitrosomycothiol reductase MscR	FIG00013253	idu(1);Glutathione_analogs:_mycothiol icw(1);Glutathione_analogs:_mycothiol
fig|6666666.28482.peg.3292	Contig_35	41506	42129	+	Putative hydrolase in cluster with formaldehyde/S-nitrosomycothiol reductase MscR	FIG00146043	icw(1);Glutathione_analogs:_mycothiol
fig|6666666.28482.peg.3293	Contig_35	42223	42861	+	Flavin reductase (EC 1.5.1.30)		isu;Heme,_hemin_uptake_and_utilization_systems_in_GramNegatives
fig|6666666.28482.peg.3294	Contig_35	42906	43382	+	Transcriptional regulator, MarR family		
fig|6666666.28482.peg.3295	Contig_35	43511	43879	+	hypothetical protein	FIG00638284	ff
fig|6666666.28482.peg.3296	Contig_35	43882	44289	+	hypothetical protein		
fig|6666666.28482.peg.3297	Contig_35	44286	45827	+	Multicopper oxidase	FIG00060246	idu(2);Copper_homeostasis
fig|6666666.28482.peg.3298	Contig_35	45878	46153	+	hypothetical protein		
fig|6666666.28482.peg.3299	Contig_35	47390	46236	-	hypothetical protein	FIG00638284	ff

fig|6666666.28482.peg.3300	Contig_35	48197	47472	-	FIG00820673: hypothetical protein	FIG00820672	ff
fig|6666666.28482.peg.3301	Contig_35	49642	48194	-	Apolipoprotein N-acyltransferase (EC 2.3.1.-)	FIG01303912	isu;Lipoprotein_Biosynthesis
fig|6666666.28482.peg.3302	Contig_35	49729	50685	+	Oxidoreductase, short-chain dehydrogenase/reductase family	FIG00993242	ff
fig|6666666.28482.peg.3303	Contig_35	50699	51805	+	S-adenosyl-L-methionine:salicylic acid carboxyl methyltransferase-like protein	FIG00822093	ff
fig|6666666.28482.peg.3304	Contig_35	51878	52297	+	FIG00824532: hypothetical protein	FIG00824529	ff
fig|6666666.28482.peg.3305	Contig_35	53987	52299	-	hypothetical protein		
fig|6666666.28482.peg.3306	Contig_35	55177	54092	-	FIG00821845: hypothetical protein	FIG00821844	ff
fig|6666666.28482.peg.3307	Contig_35	55273	55620	+	FIG00830175: hypothetical protein	FIG00830173	ff
fig|6666666.28482.peg.3308	Contig_35	55634	56929	+	putative cytochrome P450 hydroxylase	FIG00032437	idu(49);Nitric_oxide_synthase
fig|6666666.28482.peg.3309	Contig_35	57027	57566	+	FIG00821908: hypothetical protein	FIG00821906	ff
fig|6666666.28482.peg.3310	Contig_35	58282	57575	-	haloacid dehalogenase, type II	FIG00823920	ff
fig|6666666.28482.peg.3311	Contig_35	58341	58694	+	Possible membrane protein	FIG01121868	ff
fig|6666666.28482.peg.3312	Contig_35	58691	59020	+	Possible membrane protein		
fig|6666666.28482.peg.3313	Contig_35	59020	59901	+	alpha/beta hydrolase fold		

fig|6666666.28482.peg.3314	Contig_35	59987	60715	+	cutinase Cut1	FIG01392700	ff
fig|6666666.28482.peg.3315	Contig_35	61448	60720	-	N-acetylmuramoyl-L-alanine amidase	FIG01231886	ff
fig|6666666.28482.peg.3316	Contig_35	61697	62560	+	epoxide hydrolase(EC:3.3.2.3)		
fig|6666666.28482.peg.3317	Contig_35	62659	63024	+	FIG00831733: hypothetical protein	FIG00831731	ff
fig|6666666.28482.peg.3318	Contig_35	63684	63028	-	Enoyl-CoA hydratase (EC 4.2.1.17)	FIG00033206	idu(51);n-Phenylalkanoic_acid_degradation idu(51);Polyhydroxybutyrate_metabolism idu(51);Isoleucine_degradation idu(51);Fatty_acid_metabolism_cluster idu(51);Lysine_fermentation idu(51);Butanol_Biosynthesis idu(51);Acetyl-CoA_fermentation_to_Butyrate idu(51);Valine_degradation idu(51);Butyrate_metabolism_cluster
fig|6666666.28482.peg.3319	Contig_35	64313	63681	-	Transcriptional regulator, TetR family	FIG00554417	ff
fig|6666666.28482.peg.3320	Contig_35	64754	64347	-	FIG00826350: hypothetical protein	FIG00826348	ff
fig|6666666.28482.peg.3321	Contig_35	65905	65081	-	Possible membrane protein		
fig|6666666.28482.peg.3322	Contig_35	66119	67381	+	hypothetical protein		
fig|6666666.28482.peg.3323	Contig_35	67518	68297	+	hypothetical protein	FIG00638284	ff

fig|6666666.28482.peg.3324	Contig_35	68422	69270	+	3-oxoacyl-[acyl-carrier protein] reductase (EC 1.1.1.100)	FIG00621114	idu(38);CBSS-246196.1.peg.364 idu(38);CBSS-164757.7.peg.5020 idu(38);Fatty_Acid_Biosynthesis_FASI I
fig|6666666.28482.peg.3325	Contig_35	70145	69267	-	hypothetical protein	FIG00638284	ff
fig|6666666.28482.peg.3326	Contig_35	72303	72085	-	hypothetical protein		
fig|6666666.28482.peg.3327	Contig_35	75225	72586	-	COG0553: Superfamily II DNA/RNA helicases, SNF2 family	FIG00628295	ff
fig|6666666.28482.peg.3328	Contig_35	75351	78761	+	Superfamily II DNA/RNA helicases, SNF2 family	FIG01255181	isu;CBSS-316273.3.peg.2709
fig|6666666.28482.peg.3329	Contig_35	78761	81562	+	Adenine-specific DNA methylase containing a Zn-ribbon	FIG00778850	ff
fig|6666666.28482.peg.3330	Contig_35	81564	84887	+	FIG00863870: hypothetical protein	FIG01495633	ff
fig|6666666.28482.peg.3331	Contig_35	87597	87469	-	hypothetical protein		
fig|6666666.28482.peg.3332	Contig_35	88078	87626	-	FIG00828583: hypothetical protein	FIG00828580	ff
fig|6666666.28482.peg.3333	Contig_35	88166	89437	+	cytochrome P450 superfamily protein	FIG01486212	ff
fig|6666666.28482.peg.3334	Contig_35	89445	90284	+	hypothetical protein		
fig|6666666.28482.peg.3335	Contig_35	91677	90268	-	secreted protein		
fig|6666666.28482.peg.3336	Contig_35	91803	92657	+	FIG00826817: hypothetical protein	FIG00826813	ff
fig|6666666.28482.peg.3337	Contig_35	93265	92729	-	Glyoxalase family protein		
fig|6666666.28482.peg.3338	Contig_35	93967	93290	-	short-chain dehydrogenase/reductase SDR		
fig|6666666.28482.peg.3339	Contig_35	94976	94083	-	hypothetical protein	FIG00638284	ff

fig|6666666.28482.peg.3340	Contig_35	95268	95858	+	transcriptional regulator, XRE family with cupin sensor		
fig|6666666.28482.peg.3341	Contig_35	96093	96335	+	hypothetical protein		
fig|6666666.28482.peg.3342	Contig_35	96577	97956	+	Probable dehydrogenase	FIG00821376	ff
fig|6666666.28482.peg.3343	Contig_35	99317	97953	-	serine/threonine protein kinase		
fig|6666666.28482.peg.3344	Contig_36	900	139	-	cutinase Cut1	FIG01392700	ff
fig|6666666.28482.peg.3345	Contig_36	1140	2060	+	Enoyl-[acyl-carrier-protein] reductase [NADPH] (EC 1.3.1.10)	FIG00032108	idu(2);Fatty_Acid_Biosynthesis_FASII
fig|6666666.28482.peg.3346	Contig_36	2057	3637	+	Long-chain-fatty-acid--CoA ligase (EC 6.2.1.3)	FIG00018699	idu(72);n-Phenylalkanoic_acid_degradation idu(72);Fatty_acid_metabolism_cluster idu(72);Biotin_synthesis_cluster idu(72);Biotin_biosynthesis
fig|6666666.28482.peg.3347	Contig_36	8477	3645	-	Probable cation-transporting ATPase I (EC 3.6.3.-)	FIG00826871	ff
fig|6666666.28482.peg.3348	Contig_36	8553	8915	+	hypothetical protein		
fig|6666666.28482.peg.3349	Contig_36	9495	9121	-	FIG00824993: hypothetical protein	FIG00824992	ff
fig|6666666.28482.peg.3350	Contig_36	9563	10420	+	FIG056164: rhomboid family serine protease	FIG00447744	isu;Peptidyl-prolyl_cis-trans_isomerase_containing_cluster
fig|6666666.28482.peg.3351	Contig_36	10417	10878	+	FIG00823811: hypothetical protein	FIG00823809	ff

fig|6666666.28482.peg.3352	Contig_36	12051	10891	-	Alcohol dehydrogenase (EC 1.1.1.1)	FIG01007502	icw(1);Glycerolipid_and_Glycerophospholipid_Metabolism_in_Bacteria icw(1);Fermentations:_Mixed_acid icw(1);Butanol_Biosynthesis
fig|6666666.28482.peg.3353	Contig_36	13727	12048	-	Long-chain-fatty-acid--CoA ligase (EC 6.2.1.3)	FIG00018699	idu(72);n-Phenylalkanoic_acid_degradation idu(72);Fatty_acid_metabolism_cluster idu(72);Biotin_synthesis_cluster idu(72);Biotin_biosynthesis
fig|6666666.28482.peg.3354	Contig_36	14926	13730	-	hypothetical protein	FIG00638284	ff
fig|6666666.28482.peg.3355	Contig_36	15921	14938	-	Alcohol dehydrogenase (EC 1.1.1.1)	FIG01007502	icw(1);Glycerolipid_and_Glycerophospholipid_Metabolism_in_Bacteria icw(1);Fermentations:_Mixed_acid icw(1);Butanol_Biosynthesis
fig|6666666.28482.peg.3356	Contig_36	17359	15929	-	Aldehyde dehydrogenase (EC 1.2.1.3)	FIG01292786	idu(16);Pyruvate_metabolism_II:_acetyl-CoA,_acetogenesis_from_pyruvate icw(1);Glycerolipid_and_Glycerophospholipid_Metabolism_in_Bacteria idu(16);Methylglyoxal_Metabolism
fig|6666666.28482.peg.3357	Contig_36	19094	17376	-	COG0028: Thiamine pyrophosphate-requiring enzymes	FIG00452446	ff

fig|6666666.28482.peg.3358	Contig_36	20317	19097	-	L-carnitine dehydratase/bile acid-inducible protein F (EC 2.8.3.16)	FIG00450481	ff
fig|6666666.28482.peg.3359	Contig_36	21245	20310	-	Short-chain dehydrogenase/reductase SDR	FIG00546160	idu(13);CBSS-164757.7.peg.5020 idu(13);Transcription_repair_cluster
fig|6666666.28482.peg.3360	Contig_36	23102	21246	-	Cyclohexanone monooxygenase (EC 1.14.13.22)	FIG00975774	ff
fig|6666666.28482.peg.3361	Contig_36	24466	23099	-	Phenylpropionate dioxygenase and related ring-hydroxylating dioxygenases, large terminal subunit	FIG01371907	ff
fig|6666666.28482.peg.3362	Contig_36	25311	24463	-	3-oxoacyl-[acyl-carrier protein] reductase (EC 1.1.1.100)	FIG00621114	idu(38);CBSS-246196.1.peg.364 icw(1);CBSS-164757.7.peg.5020 idu(38);Fatty_Acid_Biosynthesis_FASII
fig|6666666.28482.peg.3363	Contig_36	25497	27107	+	FIG00821196: hypothetical protein	FIG00821195	ff
fig|6666666.28482.peg.3364	Contig_36	27107	27847	+	Transcriptional regulator, GntR family	FIG00621925	ff
fig|6666666.28482.peg.3365	Contig_36	27858	29477	+	Long-chain-fatty-acid--CoA ligase (EC 6.2.1.3)	FIG00018699	idu(72);n-Phenylalkanoic_acid_degradation idu(72);Fatty_acid_metabolism_cluster idu(72);Biotin_synthesis_cluster idu(72);Biotin_biosynthesis

fig|6666666.28482.peg.3366	Contig_36	29479	30252	+	Oxidoreductase, short-chain dehydrogenase/reductase family	FIG00824710	ff
fig|6666666.28482.peg.3367	Contig_36	30249	31376	+	POSSIBLE OXIDOREDUCTASE	FIG00820017	ff
fig|6666666.28482.peg.3368	Contig_36	31373	32542	+	FIG00824222: hypothetical protein	FIG00824221	ff
fig|6666666.28482.peg.3369	Contig_36	32550	33311	+	FIG00827468: hypothetical protein	FIG00827465	ff
fig|6666666.28482.peg.3370	Contig_36	33336	34544	+	FIG00828474: hypothetical protein	FIG00828473	ff
fig|6666666.28482.peg.3371	Contig_36	34732	36729	+	FIG00822403: hypothetical protein	FIG00822402	ff
fig|6666666.28482.peg.3372	Contig_36	37381	36734	-	O-methyltransferase (EC 2.1.1.-)	FIG01366127	ff
fig|6666666.28482.peg.3373	Contig_36	37938	38147	+	FIG00821503: hypothetical protein	FIG00821502	ff
fig|6666666.28482.peg.3374	Contig_36	38193	39959	+	Manganese transport protein MntH	FIG00554286	ff
fig|6666666.28482.peg.3375	Contig_36	40875	39961	-	FIG00823373: hypothetical protein	FIG00823372	ff
fig|6666666.28482.peg.3376	Contig_36	41020	41628	+	hypothetical protein		
fig|6666666.28482.peg.3377	Contig_36	41809	41639	-	Long-chain-fatty-acid--CoA ligase (EC 6.2.1.3)	FIG00018699	idu(72);n-Phenylalkanoic_acid_degradation idu(72);Fatty_acid_metabolism_cluster idu(72);Biotin_synthesis_cluster idu(72);Biotin_biosynthesis
fig|6666666.28482.peg.3378	Contig_36	42617	41862	-	Possible membrane protein	FIG01121868	ff
fig|6666666.28482.peg.3379	Contig_36	42887	43006	+	hypothetical protein		

fig|6666666.28482.peg.3380	Contig_36	42990	43337	+	FIG00829031: hypothetical protein	FIG00829027	ff
fig|6666666.28482.peg.3381	Contig_36	43399	44340	+	Hydrogen peroxide-inducible genes activator	FIG00031825	idu(1);Oxidative_stress idu(1);LysR-family_proteins_in_Salmonella_enterica_Typhimurium idu(1);LysR-family_proteins_in_Escherichia_coli idu(1);Thioredoxin-disulfide_reductase
fig|6666666.28482.peg.3382	Contig_36	46045	44324	-	Oxalyl-CoA decarboxylase (EC 4.1.1.8)	FIG00340853	isu;Alkaloid_biosynthesis_from_L-lysine
fig|6666666.28482.peg.3383	Contig_36	46252	47805	+	Long-chain-fatty-acid--CoA ligase (EC 6.2.1.3)	FIG00018699	idu(72);n-Phenylalkanoic_acid_degradation idu(72);Fatty_acid_metabolism_cluster idu(72);Biotin_synthesis_cluster idu(72);Biotin_biosynthesis
fig|6666666.28482.peg.3384	Contig_36	50063	47904	-	Translation elongation factor G-related protein	FIG00001635	isu;Translation_elongation_factor_G_family isu;Translation_elongation_factors_bacterial
fig|6666666.28482.peg.3385	Contig_36	50657	50232	-	PPOX class F420-dependent enzyme	FIG00822235	ff
fig|6666666.28482.peg.3386	Contig_37	161	286	+	hypothetical protein		
fig|6666666.28482.peg.3387	Contig_37	888	478	-	Peptide methionine sulfoxide reductase MsrB (EC 1.8.4.12)	FIG00380575	isu;Peptide_methionine_sulfoxide_reductase isu;Unknown_carbohydrate_utilization_(_cluster_Ydj_)

fig|6666666.28482.peg.3388	Contig_37	2235	898	-	Putative membrane protein Q2 colocalized with Q	FIG00010003	ff
fig|6666666.28482.peg.3389	Contig_37	3817	2249	-	FIG00995896: hypothetical protein	FIG00863727	ff
fig|6666666.28482.peg.3390	Contig_37	4588	3833	-	5-amino-6-(5-phosphoribosylamino)uracil reductase (EC 1.1.1.193) homolog	FIG00995439	ff
fig|6666666.28482.peg.3391	Contig_37	4638	5663	+	putative ATP/GTP-binding integral membrane protein	FIG00822128	ff
fig|6666666.28482.peg.3392	Contig_37	6156	5620	-	FIG00820775: hypothetical protein	FIG00820774	ff
fig|6666666.28482.peg.3393	Contig_37	6653	6144	-	Putative alanine and valine rich exported protein	FIG01368946	ff
fig|6666666.28482.peg.3394	Contig_37	7412	6675	-	ATP-dependent Clp protease ATP-binding subunit ClpA	FIG00014000	isu;Proteolysis_in_bacteria,_ATP-dependent isu;CBSS-176299.4.peg.1996B isu;ClpAS_cluster
fig|6666666.28482.peg.3395	Contig_37	7512	7844	+	hypothetical protein		
fig|6666666.28482.peg.3396	Contig_37	7947	9437	+	sodium/alanine symporter family protein		
fig|6666666.28482.peg.3397	Contig_37	9660	11294	+	Long-chain-fatty-acid--CoA ligase (EC 6.2.1.3)	FIG00018699	idu(72);n-Phenylalkanoic_acid_degradation idu(72);Fatty_acid_metabolism_cluster idu(72);Biotin_synthesis_cluster idu(72);Biotin_biosynthesis
fig|6666666.28482.peg.3398	Contig_37	11512	11375	-	hypothetical protein		

fig|6666666.28482.peg.3399	Contig_37	11670	12596	+	FIG00828625: hypothetical protein	FIG00828620	ff
fig|6666666.28482.peg.3400	Contig_37	12673	13023	+	hypothetical protein		
fig|6666666.28482.peg.3401	Contig_37	13233	13030	-	O-demethylpuromycin-O-methyltransferase(EC:2.1.1.38)		
fig|6666666.28482.peg.3402	Contig_37	13383	14012	+	hypothetical protein		
fig|6666666.28482.peg.3403	Contig_37	14021	14962	+	Non-heme chloroperoxidase (EC 1.11.1.10)	FIG01225742	ff
fig|6666666.28482.peg.3404	Contig_37	15873	14959	-	Cobalt-zinc-cadmium resistance protein	FIG00000207	isu;Cobalt-zinc-cadmium_resistance
fig|6666666.28482.peg.3405	Contig_37	15981	17099	+	FIG00829989: hypothetical protein	FIG00829988	ff
fig|6666666.28482.peg.3406	Contig_37	17212	17358	+	hypothetical protein		
fig|6666666.28482.peg.3407	Contig_37	17369	17659	+	hypothetical protein		
fig|6666666.28482.peg.3408	Contig_37	17783	18085	+	hypothetical protein		
fig|6666666.28482.peg.3409	Contig_37	18195	18542	+	hypothetical protein		
fig|6666666.28482.peg.3410	Contig_37	18636	19136	+	hypothetical protein		
fig|6666666.28482.peg.3411	Contig_37	19212	19496	+	hypothetical protein		
fig|6666666.28482.peg.3412	Contig_37	19907	19518	-	hypothetical protein	FIG00638284	ff
fig|6666666.28482.peg.3413	Contig_37	20076	20420	+	hypothetical protein		
fig|6666666.28482.rna.38	Contig_37	22038	21967	-	tRNA-Val-GAC		isu;tRNAs
fig|6666666.28482.rna.39	Contig_37	22126	22056	-	tRNA-Cys-GCA		isu;tRNAs
fig|6666666.28482.rna.40	Contig_37	22228	22156	-	tRNA-Gly-GCC		isu;tRNAs
fig|6666666.28482.rna.41	Contig_37	22406	22477	+	tRNA-Val-CAC		isu;tRNAs
fig|6666666.28482.peg.3414	Contig_37	23675	22575	-	Integrase	FIG01116662	ff
fig|6666666.28482.peg.3415	Contig_37	24762	24920	+	hypothetical protein		
fig|6666666.28482.peg.3416	Contig_37	25610	26212	+	hypothetical protein		
fig|6666666.28482.peg.3417	Contig_37	26669	26307	-	Death on curing protein, Doc toxin	FIG00021347	ff

fig|6666666.28482.peg.3418	Contig_37	28578	27076	-	Long-chain-fatty-acid--CoA ligase (EC 6.2.1.3)	FIG00018699	idu(72);n-Phenylalkanoic_acid_degradation idu(72);Fatty_acid_metabolism_cluster idu(72);Biotin_synthesis_cluster idu(72);Biotin_biosynthesis
fig|6666666.28482.peg.3419	Contig_37	28906	28766	-	hypothetical protein		
fig|6666666.28482.peg.3420	Contig_37	29554	30726	+	Butyryl-CoA dehydrogenase (EC 1.3.99.2)	FIG00009149	idu(39);Isoleucine_degradation idu(39);Lysine_fermentation idu(39);Butanol_Biosynthesis idu(39);Acetyl-CoA_fermentation_to_Butyrate idu(39);Valine_degradation idu(39);Anaerobic_respiratory_reductases
fig|6666666.28482.peg.3421	Contig_37	30927	31619	+	Transcriptional regulator, GntR family	FIG00621925	ff
fig|6666666.28482.peg.3422	Contig_37	31663	31812	+	hypothetical protein		
fig|6666666.28482.peg.3423	Contig_37	33084	32026	-	putative cytochrome P450 hydroxylase	FIG00032437	idu(49);Nitric_oxide_synthase
fig|6666666.28482.peg.3424	Contig_37	34148	33516	-	Putative GntR-family transcriptional regulator		
fig|6666666.28482.peg.3425	Contig_37	35043	36311	+	L-carnitine dehydratase/bile acid-inducible protein F	FIG01318850	ff

fig|6666666.28482.peg.3426	Contig_37	38109	36589	-	Long-chain-fatty-acid--CoA ligase (EC 6.2.1.3)	FIG00018699	idu(72);n-Phenylalkanoic_acid_degradation idu(72);Fatty_acid_metabolism_cluster idu(72);Biotin_synthesis_cluster idu(72);Biotin_biosynthesis
fig|6666666.28482.peg.3427	Contig_37	39281	38118	-	Alpha-methylacyl-CoA racemase (EC 5.1.99.4)	FIG01312689	ff
fig|6666666.28482.peg.3428	Contig_37	39941	39480	-	MaoC family protein	FIG01332817	ff
fig|6666666.28482.peg.3429	Contig_37	40354	39938	-	hypothetical protein		
fig|6666666.28482.peg.3430	Contig_37	41466	40378	-	Alcohol dehydrogenase (EC 1.1.1.1)	FIG01007502	idu(17);Glycerolipid_and_Glycerophospholipid_Metabolism_in_Bacteria idu(17);Fermentations:_Mixed_acid idu(17);Butanol_Biosynthesis
fig|6666666.28482.peg.3431	Contig_37	41894	41463	-	Putative uncharacterized protein BCG_1584c		

fig|6666666.28482.peg.3432	Contig_37	43220	42072	-	3-ketoacyl-CoA thiolase (EC 2.3.1.16) @ Acetyl-CoA acetyltransferase (EC 2.3.1.9)	FIG00000004	idu(11);CBSS-246196.1.peg.364 idu(9);Serine-glyoxylate_cycle idu(11);Serine-glyoxylate_cycle icw(1);n-Phenylalkanoic_acid_degradation idu(9);Ethylmalonyl-CoA_pathway_of_C2_assimilation,_GJO idu(9);Polyhydroxybutyrate_metabolism icw(1);Polyhydroxybutyrate_metabolism icw(1);Isoleucine_degradation icw(1);Fatty_acid_metabolism_cluster idu(11);Biotin_synthesis_cluster idu(11);Biotin_biosynthesis icw(1);Lysine_fermentation idu(9);Isoprenoid_Biosynthesis icw(2);Butanol_Biosynthesis icw(1);Acetyl-CoA_fermentation_to_Butyrate icw(1);Butyrate_metabolism_cluster

fig|6666666.28482.peg.3433	Contig_37	44117	43254	-	Enoyl-CoA hydratase (EC 4.2.1.17)	FIG00033206	idu(51);n-Phenylalkanoic_acid_degradation icw(1);Polyhydroxybutyrate_metabolism idu(51);Isoleucine_degradation idu(51);Fatty_acid_metabolism_cluster idu(51);Lysine_fermentation icw(1);Butanol_Biosynthesis idu(51);Acetyl-CoA_fermentation_to_Butyrate idu(51);Valine_degradation idu(51);Butyrate_metabolism_cluster
fig|6666666.28482.peg.3434	Contig_37	44445	46127	+	3-ketoacyl-(acyl-carrier-protein) reductase(EC:1.-)		
fig|6666666.28482.peg.3435	Contig_37	46156	47262	+	putative acyl-CoA dehydrogenase(EC:1.3.99.-)		
fig|6666666.28482.peg.3436	Contig_37	47450	47325	-	hypothetical protein		
fig|6666666.28482.peg.3437	Contig_37	47583	48266	+	acyl-CoA dehydrogenase domain protein		
fig|6666666.28482.peg.3438	Contig_37	48233	49864	+	Long-chain-fatty-acid--CoA ligase (EC 6.2.1.3)	FIG00018699	idu(72);n-Phenylalkanoic_acid_degradation idu(72);Fatty_acid_metabolism_cluster idu(72);Biotin_synthesis_cluster idu(72);Biotin_biosynthesis
fig|6666666.28482.peg.3439	Contig_37	49864	50043	+	hypothetical protein		

fig|6666666.28482.peg.3440	Contig_37	50046	51197	+	Butyryl-CoA dehydrogenase (EC 1.3.99.2)	FIG00009149	idu(39);Isoleucine_degradation idu(39);Lysine_fermentation idu(39);Butanol_Biosynthesis idu(39);Acetyl-CoA_fermentation_to_Butyrate idu(39);Valine_degradation idu(39);Anaerobic_respiratory_reductases
fig|6666666.28482.peg.3441	Contig_37	51234	52013	+	Enoyl-CoA hydratase (EC 4.2.1.17)	FIG00033206	icw(1);n-Phenylalkanoic_acid_degradation idu(51);Polyhydroxybutyrate_metabolism icw(1);Isoleucine_degradation icw(1);Fatty_acid_metabolism_cluster icw(1);Lysine_fermentation icw(1);Butanol_Biosynthesis icw(1);Acetyl-CoA_fermentation_to_Butyrate icw(1);Valine_degradation idu(51);Butyrate_metabolism_cluster
fig|6666666.28482.peg.3442	Contig_37	52355	52035	-	hypothetical protein		
fig|6666666.28482.peg.3443	Contig_37	52614	53447	+	Acyl-CoA thioesterase II (EC 3.1.2.-)	FIG00002217	idu(3);Acyl-CoA_thioesterase_II
fig|6666666.28482.peg.3444	Contig_37	54308	53478	-	probable oxidoreductase/Short-chain dehydrogenase	FIG01319309	ff
fig|6666666.28482.peg.3445	Contig_37	55534	54305	-	putative cytochrome P450 hydroxylase	FIG00032437	idu(49);Nitric_oxide_synthase
fig|6666666.28482.peg.3446	Contig_37	55715	55539	-	hypothetical protein		
fig|6666666.28482.peg.3447	Contig_37	55998	55873	-	hypothetical protein		

fig|6666666.28482.peg.3448	Contig_37	57647	56499	-	Butyryl-CoA dehydrogenase (EC 1.3.99.2)	FIG00009149	icw(2);Isoleucine_degradation icw(2);Lysine_fermentation icw(2);Butanol_Biosynthesis icw(2);Acetyl-CoA_fermentation_to_Butyrate icw(2);Valine_degradation icw(2);Anaerobic_respiratory_reductases
fig|6666666.28482.peg.3449	Contig_37	57731	57862	+	hypothetical protein		
fig|6666666.28482.peg.3450	Contig_37	58726	57947	-	3-hydroxyacyl-CoA dehydrogenase	FIG01342845	ff
fig|6666666.28482.peg.3451	Contig_37	59860	58760	-	Butyryl-CoA dehydrogenase (EC 1.3.99.2)	FIG00009149	icw(2);Isoleucine_degradation icw(2);Lysine_fermentation icw(2);Butanol_Biosynthesis icw(2);Acetyl-CoA_fermentation_to_Butyrate icw(2);Valine_degradation icw(2);Anaerobic_respiratory_reductases
fig|6666666.28482.peg.3452	Contig_37	61005	59857	-	Butyryl-CoA dehydrogenase (EC 1.3.99.2)	FIG00009149	icw(2);Isoleucine_degradation icw(2);Lysine_fermentation icw(2);Butanol_Biosynthesis icw(2);Acetyl-CoA_fermentation_to_Butyrate icw(2);Valine_degradation icw(2);Anaerobic_respiratory_reductases
fig|6666666.28482.peg.3453	Contig_37	61211	61002	-	hypothetical protein		
fig|6666666.28482.peg.3454	Contig_38	1533	658	-	putative oxidoreductase YdbC(EC:1.-)		
fig|6666666.28482.peg.3455	Contig_38	1557	2057	+	FIG037376: hypothetical protein	FIG00821243	ff

fig|6666666.28482.peg.3456	Contig_38	3002	2061	-	2-nitropropane dioxygenase (EC 1.13.11.32)	FIG01304356	idu(5);CBSS-164757.7.peg.5020
fig|6666666.28482.peg.3457	Contig_38	3351	3007	-	FIG00821167: hypothetical protein	FIG00821164	ff
fig|6666666.28482.peg.3458	Contig_38	3807	3382	-	Possible membrane protein	FIG01121868	ff
fig|6666666.28482.peg.3459	Contig_38	4460	3804	-	Putative transcriptional regulatory protein	FIG01331484	ff
fig|6666666.28482.peg.3460	Contig_38	4591	4475	-	hypothetical protein		
fig|6666666.28482.peg.3461	Contig_38	4748	5638	+	Universal stress protein family	FIG00017615	ff
fig|6666666.28482.peg.3462	Contig_38	6432	5635	-	hypothetical protein	FIG00638284	ff
fig|6666666.28482.peg.3463	Contig_38	7242	6451	-	Universal stress protein family	FIG00017615	ff
fig|6666666.28482.peg.3464	Contig_38	8418	7267	-	Probable conserved transmembrane alanine and leucine rich protein		
fig|6666666.28482.peg.3465	Contig_38	9062	8454	-	hypothetical protein		
fig|6666666.28482.peg.3466	Contig_38	9250	9480	+	hypothetical protein		
fig|6666666.28482.peg.3467	Contig_38	10874	9477	-	Diacyglycerol O-acyltransferase (EC 2.3.1.20)	FIG00831328	ff
fig|6666666.28482.peg.3468	Contig_38	12143	10893	-	FIG00821576: hypothetical protein	FIG00821571	ff
fig|6666666.28482.peg.3469	Contig_38	12319	12591	+	hypothetical protein		
fig|6666666.28482.peg.3470	Contig_38	13089	12607	-	Flavodoxin	FIG00010844	isu;Flavodoxin

fig|6666666.28482.peg.3471	Contig_38	13204	15042	+	Long-chain-fatty-acid--CoA ligase (EC 6.2.1.3)	FIG00018699	idu(72);n-Phenylalkanoic_acid_degradation idu(72);Fatty_acid_metabolism_cluster idu(72);Biotin_synthesis_cluster idu(72);Biotin_biosynthesis
fig|6666666.28482.peg.3472	Contig_38	15352	15071	-	hypothetical protein		
fig|6666666.28482.peg.3473	Contig_38	15528	15388	-	hypothetical protein		
fig|6666666.28482.peg.3474	Contig_38	15617	16264	+	Transcriptional repressor EthR, TetR family	FIG01323805	ff
fig|6666666.28482.peg.3475	Contig_38	17477	16251	-	3-carboxy-cis,cis-muconate cycloisomerase (EC 5.5.1.2)	FIG00001777	icw(2);Protocatechuate_branch_of_beta-ketoadipate_pathway
fig|6666666.28482.peg.3476	Contig_38	17992	17474	-	Protocatechuate 3,4-dioxygenase alpha chain (EC 1.13.11.3)	FIG00138292	icw(1);Protocatechuate_branch_of_beta-ketoadipate_pathway
fig|6666666.28482.peg.3477	Contig_38	18740	17985	-	Protocatechuate 3,4-dioxygenase beta chain (EC 1.13.11.3)	FIG00001681	isu;Protocatechuate_branch_of_beta-ketoadipate_pathway
fig|6666666.28482.peg.3478	Contig_38	18947	20272	+	Phthalate 3,4-dioxygenase alpha subunit	FIG00171655	isu;Dioxygenases_(EC_1.14.12.-)
fig|6666666.28482.peg.3479	Contig_38	20269	20787	+	Aromatic-ring-hydroxylating dioxygenase, beta subunit	FIG00143547	isu;Aromatic_dioxygenase_mess icw(1);Dioxygenases_(EC_1.14.12.-)
fig|6666666.28482.peg.3480	Contig_38	20784	21011	+	dihydrodiol dehydrogenase		

fig|6666666.28482.peg.3481	Contig_38	21008	21802	+	Dihydrodiol dehydrogenase (EC 1.3.1.56)	FIG00136718	isu;Biphenyl_Degradation
fig|6666666.28482.peg.3482	Contig_38	21799	22524	+	COG1720: Uncharacterized conserved protein	FIG00001872	ff
fig|6666666.28482.peg.3483	Contig_38	22580	23428	+	FIG00825125: hypothetical protein	FIG00825124	ff
fig|6666666.28482.peg.3484	Contig_38	24236	23436	-	Transcriptional regulator, IclR family	FIG00022569	idu(12);Homogentisate_pathway_of_aromatic_compound_degradation
fig|6666666.28482.peg.3485	Contig_38	25330	24263	-	hypothetical protein	FIG00638284	ff
fig|6666666.28482.peg.3486	Contig_38	25447	26277	+	FIG00824233: hypothetical protein	FIG00824231	ff
fig|6666666.28482.peg.3487	Contig_38	26274	26933	+	DedA protein	FIG00002761	idu(1);Uptake_of_selenate_and_selenite idu(1);DedA_family_of_inner_membrane_proteins
fig|6666666.28482.peg.3488	Contig_38	27706	27086	-	Transcriptional regulator, TetR family	FIG00554417	ff
fig|6666666.28482.peg.3489	Contig_38	27862	28299	+	Anti-sigma F factor antagonist (spoIIAA-2); Anti-sigma B factor antagonist RsbV	FIG00722824	idu(5);SigmaB_stress_responce_regulation idu(5);Sigma-B_stress_response_cluster_1
fig|6666666.28482.peg.3490	Contig_38	28334	29419	+	Putative cytoplasmic protein	FIG00904518	ff
fig|6666666.28482.peg.3491	Contig_38	30176	29406	-	FIG00823196: hypothetical protein	FIG00823195	ff
fig|6666666.28482.peg.3492	Contig_38	31136	30213	-	Catalase (EC 1.11.1.6)	FIG00000313	idu(3);Oxidative_stress idu(3);Photorespiration_(oxidative_C2_cycle) idu(3);Protection_from_Reactive_Oxygen_Species

fig|6666666.28482.peg.3493	Contig_38	31942	31154	-	Short chain membrane-associated dehydrogenase	FIG00821400	ff
fig|6666666.28482.peg.3494	Contig_38	32687	32085	-	protein of unknown function DUF1470		
fig|6666666.28482.peg.3495	Contig_38	32753	33682	+	hypothetical protein	FIG00638284	ff
fig|6666666.28482.peg.3496	Contig_38	33699	35231	+	Long-chain-fatty-acid--CoA ligase (EC 6.2.1.3)	FIG00018699	idu(72);n-Phenylalkanoic_acid_degradation idu(72);Fatty_acid_metabolism_cluster idu(72);Biotin_synthesis_cluster idu(72);Biotin_biosynthesis
fig|6666666.28482.peg.3497	Contig_38	35224	36012	+	hypothetical protein	FIG00638284	ff
fig|6666666.28482.peg.3498	Contig_38	36834	36007	-	Naphthoate synthase (EC 4.1.3.36)	FIG00071623	idu(2);Menaquinone_and_Phylloquinone_Biosynthesis_--_gjo idu(2);Menaquinone_and_Phylloquinone_Biosynthesis
fig|6666666.28482.peg.3499	Contig_38	37183	36890	-	FIG00828916: hypothetical protein	FIG00828915	ff
fig|6666666.28482.peg.3500	Contig_38	37573	37854	+	WhiB-type transcription regulator	FIG00019991	idu(2);WhiB_and_WhiB-type_regulatory_proteins_
fig|6666666.28482.peg.3501	Contig_38	38511	37915	-	Transcriptional regulator, TetR family	FIG00554417	ff
fig|6666666.28482.peg.3502	Contig_38	41380	38516	-	membrane protein, MmpL family (mmpL4)	FIG01318281	ff
fig|6666666.28482.peg.3503	Contig_38	41814	41377	-	membrane protein, MmpS family	FIG01309473	ff
fig|6666666.28482.peg.3504	Contig_38	42025	42225	+	hypothetical protein		
fig|6666666.28482.peg.3505	Contig_38	43374	43853	+	hypothetical protein		
fig|6666666.28482.peg.3506	Contig_38	43910	44659	+	hypothetical protein		
fig|6666666.28482.peg.3507	Contig_38	45052	44936	-	hypothetical protein		
fig|6666666.28482.peg.3508	Contig_38	45428	45541	+	hypothetical protein		

fig|6666666.28482.peg.3509	Contig_38	46541	46128	-	hypothetical protein		
fig|6666666.28482.peg.3510	Contig_38	47516	47391	-	hypothetical protein		
fig|6666666.28482.peg.3511	Contig_38	47877	48956	+	hypothetical protein	FIG00638284	ff
fig|6666666.28482.peg.3512	Contig_38	48958	50613	+	Serine/threonine-protein kinase pknD (EC 2.7.11.1)		
fig|6666666.28482.peg.3513	Contig_38	50748	51779	+	PE_PGRS family protein		
fig|6666666.28482.rna.42	Contig_38	51974	51904	-	tRNA-Pseudo-CAC		
fig|6666666.28482.peg.3514	Contig_38	52913	52113	-	Enoyl-CoA hydratase (EC 4.2.1.17)	FIG00033206	icw(1);n-Phenylalkanoic_acid_degradation icw(1);Polyhydroxybutyrate_metabolism idu(51);Isoleucine_degradation icw(1);Fatty_acid_metabolism_cluster idu(51);Lysine_fermentation idu(51);Butanol_Biosynthesis idu(51);Acetyl-CoA_fermentation_to_Butyrate idu(51);Valine_degradation idu(51);Butyrate_metabolism_cluster
fig|6666666.28482.peg.3515	Contig_38	53200	52910	-	FIG00820985: hypothetical protein	FIG01577081	ff
fig|6666666.28482.peg.3516	Contig_38	53526	53329	-	hypothetical protein		
fig|6666666.28482.peg.3517	Contig_38	53863	54603	+	regulatory protein GntR, HTH	FIG01451455	ff

fig|6666666.28482.peg.3518	Contig_38	54719	55909	+	3-ketoacyl-CoA thiolase (EC 2.3.1.16) @ Acetyl-CoA acetyltransferase (EC 2.3.1.9)	FIG00000004	idu(11);CBSS-246196.1.peg.364 idu(9);Serine-glyoxylate_cycle idu(11);Serine-glyoxylate_cycle icw(2);n-Phenylalkanoic_acid_degradation idu(9);Ethylmalonyl-CoA_pathway_of_C2_assimilation,_GJO idu(9);Polyhydroxybutyrate_metabolism icw(1);Polyhydroxybutyrate_metabolism icw(1);Isoleucine_degradation icw(2);Fatty_acid_metabolism_cluster icw(1);Biotin_synthesis_cluster icw(1);Biotin_biosynthesis icw(1);Lysine_fermentation idu(9);Isoprenoid_Biosynthesis icw(1);Butanol_Biosynthesis icw(1);Acetyl-CoA_fermentation_to_Butyrate icw(1);Butyrate_metabolism_cluster
fig|6666666.28482.peg.3519	Contig_38	55911	56330	+	hypothetical protein	FIG00638284	ff
fig|6666666.28482.peg.3520	Contig_38	56437	58029	+	Long-chain-fatty-acid--CoA ligase (EC 6.2.1.3)	FIG00018699	idu(72);n-Phenylalkanoic_acid_degradation idu(72);Fatty_acid_metabolism_cluster idu(72);Biotin_synthesis_cluster idu(72);Biotin_biosynthesis
fig|6666666.28482.peg.3521	Contig_38	59169	58231	-	hypothetical protein	FIG00638284	ff

fig|6666666.28482.peg.3522	Contig_38	60401	59172	-	Hydroxyproline dehydratase putative	FIG01390869	ff
fig|6666666.28482.peg.3523	Contig_38	61921	60425	-	Aldehyde dehydrogenase (EC 1.2.1.3)	FIG01292786	idu(16);Pyruvate_metabolism_II:_acetyl-CoA,_acetogenesis_from_pyruvate idu(16);Glycerolipid_and_Glycerophospholipid_Metabolism_in_Bacteria idu(16);Methylglyoxal_Metabolism
fig|6666666.28482.peg.3524	Contig_38	63261	62092	-	amidohydrolase 2	FIG00501263	ff
fig|6666666.28482.peg.3525	Contig_38	63378	63668	+	hypothetical protein	FIG00638284	ff
fig|6666666.28482.peg.3526	Contig_38	63755	64969	+	Transcriptional regulator, TetR family	FIG00554417	ff
fig|6666666.28482.peg.3527	Contig_38	65369	66127	+	PROBABLE DEHYDROGENASE (EC 1.-.-.-)	FIG01877414	ff
fig|6666666.28482.peg.3528	Contig_38	67441	66752	-	Transcriptional regulator, TetR family	FIG00554417	ff
fig|6666666.28482.peg.3529	Contig_38	67554	68783	+	Butyryl-CoA dehydrogenase (EC 1.3.99.2)	FIG00009149	idu(39);Isoleucine_degradation idu(39);Lysine_fermentation idu(39);Butanol_Biosynthesis idu(39);Acetyl-CoA_fermentation_to_Butyrate idu(39);Valine_degradation idu(39);Anaerobic_respiratory_reductases
fig|6666666.28482.peg.3530	Contig_38	68802	69926	+	Putative acyl-CoA dehydrogenase(EC:1.3.99.3)		
fig|6666666.28482.peg.3531	Contig_38	70274	70131	-	hypothetical protein		
fig|6666666.28482.peg.3532	Contig_38	70900	70712	-	hypothetical protein		

fig|6666666.28482.peg.3533	Contig_38	71691	75197	+	Long-chain-fatty-acid--CoA ligase (EC 6.2.1.3)	FIG00018699	idu(72);n-Phenylalkanoic_acid_degradation idu(72);Fatty_acid_metabolism_cluster idu(72);Biotin_synthesis_cluster idu(72);Biotin_biosynthesis
fig|6666666.28482.peg.3534	Contig_38	75247	76146	+	probable oxidoreductase/Short-chain dehydrogenase	FIG01319309	ff
fig|6666666.28482.peg.3535	Contig_38	76797	76940	+	hypothetical protein		
fig|6666666.28482.peg.3536	Contig_38	77772	76984	-	hypothetical protein	FIG00638284	ff
fig|6666666.28482.peg.3537	Contig_38	78563	77835	-	FIG00821219: hypothetical protein	FIG00821218	ff
fig|6666666.28482.peg.3538	Contig_38	80005	78527	-	MCE-family protein Mce1F	FIG00821171	idu(7);Mycobacterium_virulence_operon_MCE_involved_in_cell_invasion
fig|6666666.28482.peg.3539	Contig_38	81167	80010	-	MCE-family lipoprotein LprK (MCE-family lipoprotein Mce1e)	FIG00819998	icw(2);Mycobacterium_virulence_operon_MCE_involved_in_cell_invasion
fig|6666666.28482.peg.3540	Contig_38	82474	81164	-	MCE-family protein Mce1D	FIG00820669	icw(1);Mycobacterium_virulence_operon_MCE_involved_in_cell_invasion
fig|6666666.28482.peg.3541	Contig_38	83907	82498	-	MCE-family protein Mce1C	FIG00821293	icw(7);Mycobacterium_virulence_operon_MCE_involved_in_cell_invasion
fig|6666666.28482.peg.3542	Contig_38	84932	83904	-	MCE-family protein Mce1B	FIG00763551	icw(5);Mycobacterium_virulence_operon_MCE_involved_in_cell_invasion
fig|6666666.28482.peg.3543	Contig_38	86454	84973	-	MCE-family protein Mce1A	FIG00820494	icw(2);Mycobacterium_virulence_operon_MCE_involved_in_cell_invasion

fig|6666666.28482.peg.3544	Contig_38	87278	86460	-	Conserved hypothetical integral membrane protein YrbE1B	FIG00691621	icw(2);Mycobacterium_virulence_operon_MCE_involved_in_cell_invasion
fig|6666666.28482.peg.3545	Contig_38	88105	87311	-	Conserved hypothetical integral membrane protein YrbE1A	FIG00690583	idu(7);Mycobacterium_virulence_operon_MCE_involved_in_cell_invasion
fig|6666666.28482.peg.3546	Contig_38	89458	90147	+	Transcriptional regulator, TetR family	FIG00554417	ff
fig|6666666.28482.peg.3547	Contig_38	90518	90838	+	hypothetical protein		
fig|6666666.28482.peg.3548	Contig_38	91208	90942	-	FIG00820120: hypothetical protein	FIG00820119	ff
fig|6666666.28482.peg.3549	Contig_38	91434	92078	+	hypothetical protein	FIG00638284	ff
fig|6666666.28482.peg.3550	Contig_38	92369	92566	+	Putative ferredoxin	FIG00820674	ff
fig|6666666.28482.peg.3551	Contig_38	94333	92660	-	Long-chain-fatty-acid--CoA ligase (EC 6.2.1.3)	FIG00018699	idu(72);n-Phenylalkanoic_acid_degradation idu(72);Fatty_acid_metabolism_cluster idu(72);Biotin_synthesis_cluster idu(72);Biotin_biosynthesis
fig|6666666.28482.peg.3552	Contig_38	95164	94340	-	phospholipid/glycerol acyltransferase		
fig|6666666.28482.peg.3553	Contig_38	96596	95319	-	protein of unknown function UPF0089		
fig|6666666.28482.peg.3554	Contig_38	97827	96958	-	hypothetical protein		
fig|6666666.28482.peg.3555	Contig_38	98392	98207	-	hypothetical protein		
fig|6666666.28482.peg.3556	Contig_38	98771	98583	-	L-carnitine dehydratase/bile acid-inducible protein F		
fig|6666666.28482.peg.3557	Contig_38	98860	99012	+	hypothetical protein		

fig|6666666.28482.peg.3558	Contig_38	100276	99182	-	Acyl-CoA dehydrogenase, C-terminal:Acyl-CoA dehydrogenase, central region:Acyl-CoA dehydrogenase, N-terminal		
fig|6666666.28482.peg.3559	Contig_38	101244	100639	-	Transcriptional regulator, TetR family	FIG00554417	ff
fig|6666666.28482.peg.3560	Contig_38	101404	102639	+	BarH	FIG00668014	ff
fig|6666666.28482.peg.3561	Contig_38	103012	103509	+	hydrolase, alpha/beta fold family protein, putative		
fig|6666666.28482.peg.3562	Contig_38	103613	103873	+	BarH	FIG00668014	ff
fig|6666666.28482.peg.3563	Contig_38	103953	104792	+	short-chain dehydrogenase/reductase SDR		
fig|6666666.28482.peg.3564	Contig_38	105002	105739	+	regulatory protein GntR, HTH	FIG01451455	ff
fig|6666666.28482.peg.3565	Contig_38	105793	106572	+	hypothetical protein		
fig|6666666.28482.peg.3566	Contig_38	107045	106587	-	Acyl dehydratase	FIG01303815	idu(2);CBSS-246196.1.peg.364
fig|6666666.28482.peg.3567	Contig_38	107893	107168	-	Putative GntR-family transcriptional regulator		
fig|6666666.28482.peg.3568	Contig_38	108043	109080	+	PROBABLE OXIDOREDUCTASE	FIG01416034	ff
fig|6666666.28482.peg.3569	Contig_38	109164	109601	+	hypothetical protein		
fig|6666666.28482.peg.3570	Contig_38	109794	109955	+	hypothetical protein		
fig|6666666.28482.peg.3571	Contig_38	110098	110853	+	3-oxoacyl-[acyl-carrier protein] reductase (EC 1.1.1.100)	FIG00621114	icw(2);CBSS-246196.1.peg.364 icw(1);CBSS-164757.7.peg.5020 icw(1);Fatty_Acid_Biosynthesis_FASII

fig|6666666.28482.peg.3572	Contig_38	111873	110950	-	3-oxoacyl-[acyl-carrier protein] reductase (EC 1.1.1.100)	FIG00621114	icw(2);CBSS-246196.1.peg.364 icw(1);CBSS-164757.7.peg.5020 icw(1);Fatty_Acid_Biosynthesis_FASII
fig|6666666.28482.peg.3573	Contig_38	112270	113088	+	Enoyl-CoA hydratase (EC 4.2.1.17)	FIG00033206	icw(1);n-Phenylalkanoic_acid_degradation icw(1);Polyhydroxybutyrate_metabolism icw(2);Isoleucine_degradation icw(1);Fatty_acid_metabolism_cluster icw(2);Lysine_fermentation icw(2);Butanol_Biosynthesis icw(2);Acetyl-CoA_fermentation_to_Butyrate icw(2);Valine_degradation icw(1);Butyrate_metabolism_cluster

fig|6666666.28482.peg.3574	Contig_38	113133	113867	+	Enoyl-CoA hydratase (EC 4.2.1.17)	FIG00033206	icw(1);n-Phenylalkanoic_acid_degradation icw(1);Polyhydroxybutyrate_metabolism icw(2);Isoleucine_degradation icw(1);Fatty_acid_metabolism_cluster icw(2);Lysine_fermentation icw(2);Butanol_Biosynthesis icw(2);Acetyl-CoA_fermentation_to_Butyrate icw(2);Valine_degradation icw(1);Butyrate_metabolism_cluster
fig|6666666.28482.peg.3575	Contig_38	114209	115363	+	Nonspecific lipid-transfer protein	FIG00828344	ff
fig|6666666.28482.peg.3576	Contig_38	115847	116869	+	Butyryl-CoA dehydrogenase (EC 1.3.99.2)	FIG00009149	idu(39);Isoleucine_degradation idu(39);Lysine_fermentation idu(39);Butanol_Biosynthesis idu(39);Acetyl-CoA_fermentation_to_Butyrate idu(39);Valine_degradation idu(39);Anaerobic_respiratory_reductases
fig|6666666.28482.peg.3577	Contig_38	116866	117999	+	putative acyl-CoA dehydrogenase		
fig|6666666.28482.peg.3578	Contig_38	118029	119183	+	4-hydroxybutyrate coenzyme A transferase	FIG00945012	ff
fig|6666666.28482.peg.3579	Contig_38	119216	119461	+	short-chain dehydrogenase/reductase SDR		

fig|6666666.28482.peg.3580	Contig_38	119647	120831	+	putative acyl-CoA dehydrogenase	FIG00476656	ff
fig|6666666.28482.peg.3581	Contig_38	120828	121820	+	Isovaleryl-CoA dehydrogenase (EC 1.3.99.10); Acyl-CoA dehydrogenase, short-chain specific (EC 1.3.99.2)	FIG00340338	icw(1);Isoleucine_degradation idu(5);HMG_CoA_Synthesis idu(5);Leucine_Degradation_and_HMG-CoA_Metabolism
fig|6666666.28482.peg.3582	Contig_38	122774	121959	-	Enoyl-CoA hydratase (EC 4.2.1.17)	FIG00033206	idu(51);n-Phenylalkanoic_acid_degradation idu(51);Polyhydroxybutyrate_metabolism icw(1);Isoleucine_degradation idu(51);Fatty_acid_metabolism_cluster idu(51);Lysine_fermentation idu(51);Butanol_Biosynthesis idu(51);Acetyl-CoA_fermentation_to_Butyrate idu(51);Valine_degradation idu(51);Butyrate_metabolism_cluster
fig|6666666.28482.peg.3583	Contig_38	123951	122791	-	Acetyl-CoA acetyltransferase	FIG01005417	ff
fig|6666666.28482.peg.3584	Contig_39	47	760	+	NADH-ubiquinone oxidoreductase chain G (EC 1.6.5.3)	FIG00010922	icw(5);Respiratory_Complex_I icw(5);NADH_ubiquinone_oxidoreductase
fig|6666666.28482.peg.3585	Contig_39	757	2028	+	NADH-ubiquinone oxidoreductase chain H (EC 1.6.5.3)	FIG00133041	icw(3);Respiratory_Complex_I icw(3);NADH_ubiquinone_oxidoreductase

fig|6666666.28482.peg.3586	Contig_39	2021	2566	+	NADH-ubiquinone oxidoreductase chain I (EC 1.6.5.3)	FIG00138210	icw(4);Respiratory_Complex_I icw(4);NADH_ubiquinone_oxidoreductase
fig|6666666.28482.peg.3587	Contig_39	2563	3363	+	NADH-ubiquinone oxidoreductase chain J (EC 1.6.5.3)	FIG00000569	icw(2);Respiratory_Complex_I icw(2);NADH_ubiquinone_oxidoreductase
fig|6666666.28482.peg.3588	Contig_39	3360	3659	+	NADH-ubiquinone oxidoreductase chain K (EC 1.6.5.3)	FIG00000559	idu(1);Respiratory_Complex_I idu(1);NADH_ubiquinone_oxidoreductase
fig|6666666.28482.peg.3589	Contig_39	3672	5558	+	NADH-ubiquinone oxidoreductase chain L (EC 1.6.5.3)	FIG00068727	icw(1);Respiratory_Complex_I icw(1);NADH_ubiquinone_oxidoreductase
fig|6666666.28482.peg.3590	Contig_39	5564	7126	+	NADH-ubiquinone oxidoreductase chain M (EC 1.6.5.3)	FIG00138227	icw(6);Respiratory_Complex_I icw(6);NADH_ubiquinone_oxidoreductase
fig|6666666.28482.peg.3591	Contig_39	7123	8703	+	NADH-ubiquinone oxidoreductase chain N (EC 1.6.5.3)	FIG00000512	icw(3);Respiratory_Complex_I icw(3);NADH_ubiquinone_oxidoreductase
fig|6666666.28482.peg.3592	Contig_39	9162	8716	-	Possible membrane protein	FIG01315779	ff
fig|6666666.28482.peg.3593	Contig_39	10418	9159	-	POSSIBLE CONSERVED SECRETED PROTEIN	FIG00996000	ff
fig|6666666.28482.peg.3594	Contig_39	11392	10424	-	Probable methanol dehydrogenase transcriptional regulatory protein MoxR3	FIG01001992	ff
fig|6666666.28482.peg.3595	Contig_39	11871	11389	-	FIG00997183: hypothetical protein	FIG00997177	ff
fig|6666666.28482.peg.3596	Contig_39	12794	11868	-	FIG00995849: hypothetical protein	FIG00995847	ff
fig|6666666.28482.peg.3597	Contig_39	13160	14170	+	putative YrbE family protein	FIG01497886	ff

fig|6666666.28482.peg.3598	Contig_39	14180	15040	+	ABC-type transport system involved in resistance to organic solvents, permease component	FIG00450905	ff
fig|6666666.28482.peg.3599	Contig_39	15044	16096	+	putative Mce family protein		
fig|6666666.28482.peg.3600	Contig_39	16093	17103	+	MCE-family protein Mce1B		icw(1);Mycobacterium_virulence_operon_MCE_involved_in_cell_invasion
fig|6666666.28482.peg.3601	Contig_39	17103	18125	+	putative Mce family protein		
fig|6666666.28482.peg.3602	Contig_39	18127	19284	+	MCE-family protein Mce1D		idu(6);Mycobacterium_virulence_operon_MCE_involved_in_cell_invasion
fig|6666666.28482.peg.3603	Contig_39	19296	20354	+	putative Mce family protein		
fig|6666666.28482.peg.3604	Contig_39	20351	21316	+	secreted protein		
fig|6666666.28482.peg.3605	Contig_39	21333	22088	+	hypothetical protein		
fig|6666666.28482.peg.3606	Contig_39	22099	22872	+	hypothetical protein		
fig|6666666.28482.peg.3607	Contig_39	23242	22877	-	hypothetical protein		
fig|6666666.28482.peg.3608	Contig_39	23344	24726	+	FIG00822613: hypothetical protein	FIG00822610	ff
fig|6666666.28482.peg.3609	Contig_39	24756	25361	+	Methyltransferase type 11		
fig|6666666.28482.peg.3610	Contig_39	26220	25348	-	7-alpha-hydroxysteroid dehydrogenase (EC 1.1.1.159)	FIG01308941	ff
fig|6666666.28482.peg.3611	Contig_39	26717	26217	-	Transcriptional regulator, TetR family	FIG00554417	ff
fig|6666666.28482.peg.3612	Contig_39	26879	28036	+	Phosphotransferase	FIG01421608	ff

fig|6666666.28482.peg.3613	Contig_39	28036	29151	+	Putative uncharacterized protein BCG_3193	FIG01325639	ff
fig|6666666.28482.peg.3614	Contig_39	30143	29148	-	PROBABLE OXIDOREDUCTASE	FIG01416034	ff
fig|6666666.28482.peg.3615	Contig_39	30225	31571	+	Amine oxidase [flavin-containing] A (EC 1.4.3.4)	FIG01366019	ff
fig|6666666.28482.peg.3616	Contig_39	32516	31566	-	Non-heme haloperoxidase Hpx	FIG01312018	ff
fig|6666666.28482.peg.3617	Contig_39	32645	34000	+	Monoamine oxidase	FIG00827338	ff
fig|6666666.28482.peg.3618	Contig_39	34453	34007	-	hypothetical protein	FIG00638284	ff
fig|6666666.28482.peg.3619	Contig_39	35562	34492	-	transcriptional regulator, AraC family	FIG00628273	ff
fig|6666666.28482.peg.3620	Contig_39	36716	35559	-	Glycosyl transferase, group 1	FIG01560076	ff
fig|6666666.28482.peg.3621	Contig_39	36879	37100	+	protein-glutamate methylesterase CheB(EC:3.1.1.61)		
fig|6666666.28482.peg.3622	Contig_39	37145	37735	+	CheB methylesterase(EC:3.1.1.61)	FIG00908064	ff
fig|6666666.28482.peg.3623	Contig_39	39596	37749	-	Chemotaxis protein methyltransferase CheR (EC 2.1.1.80)	FIG00000452	isu;Bacterial_Chemotaxis
fig|6666666.28482.peg.3624	Contig_39	40407	39649	-	FIG00824935: hypothetical protein	FIG00824933	ff
fig|6666666.28482.peg.3625	Contig_39	41419	40400	-	Protein-glutamate methylesterase	FIG01745458	ff
fig|6666666.28482.peg.3626	Contig_39	41517	43646	+	Catalase (EC 1.11.1.6)	FIG00000313	idu(3);Oxidative_stress idu(3);Photorespiration_(oxidative_C2_cycle) idu(3);Protection_from_Reactive_Oxygen_Species

fig|6666666.28482.peg.3627	Contig_39	43666	43923	+	FIG00823837: hypothetical protein	FIG00823834	ff
fig|6666666.28482.peg.3628	Contig_39	44642	44058	-	FIG00826083: hypothetical protein	FIG00826080	ff
fig|6666666.28482.peg.3629	Contig_39	44810	45754	+	Acyl-CoA dehydrogenase/oxidase domain protein	FIG00841842	ff
fig|6666666.28482.peg.3630	Contig_39	45751	46524	+	LmbE-like protein	FIG00998828	ff
fig|6666666.28482.peg.3631	Contig_39	46521	47078	+	probable methyltransferase		
fig|6666666.28482.peg.3632	Contig_39	47092	47874	+	endonuclease/exonuclease/phosphatase	FIG01502470	ff
fig|6666666.28482.peg.3633	Contig_39	48154	47879	-	hypothetical protein		
fig|6666666.28482.peg.3634	Contig_39	48503	48267	-	FIG00824078: hypothetical protein	FIG01339979	ff
fig|6666666.28482.peg.3635	Contig_39	48673	49515	+	hypothetical protein		
fig|6666666.28482.peg.3636	Contig_39	49786	50184	+	FIG00820286: hypothetical protein	FIG00820285	ff
fig|6666666.28482.peg.3637	Contig_39	50296	51351	+	Hypothetical protein PA2244 (similar to DNA topoisomerase IB, but possibly involved in glycosyl-transfer)	FIG00002100	isu;DNA_topoisomerases,_Type_I,_ATP-independent
fig|6666666.28482.peg.3638	Contig_4	75	539	+	FIG00823304: hypothetical protein	FIG00823303	ff
fig|6666666.28482.peg.3639	Contig_4	651	1700	+	Serine protease PepA	FIG01322276	ff
fig|6666666.28482.peg.3640	Contig_4	1812	3593	+	Trehalose synthase (EC 5.4.99.16)	FIG00005112	isu;Trehalose_Biosynthesis
fig|6666666.28482.peg.3641	Contig_4	3590	4933	+	uncharacterized protein probably involved in trehalose biosynthesis	FIG01418635	ff

fig|6666666.28482.peg.3642	Contig_4	4998	5711	+	Possible membrane protein	FIG01121868	ff
fig|6666666.28482.peg.3643	Contig_4	6787	5747	-	Antigen 85-C precursor (85C) (Antigen 85 complex C) (Ag85C) (Mycolyl transferase 85C) (EC 2.3.1.-)	FIG00027986	ff
fig|6666666.28482.peg.3644	Contig_4	7825	6998	-	ABC-type drug export system, membrane protein	FIG00822310	ff
fig|6666666.28482.peg.3645	Contig_4	8636	7815	-	ABC transporter, ATP-binding component(EC:3.6.3.-)	FIG00826428	ff
fig|6666666.28482.peg.3646	Contig_4	9079	8633	-	Transcriptional regulator, MarR family	FIG00554610	ff
fig|6666666.28482.peg.3647	Contig_4	9642	9145	-	Phosphoesterase	FIG01238380	ff
fig|6666666.28482.peg.3648	Contig_4	9833	9657	-	FIG00827455: hypothetical protein	FIG00827452	ff
fig|6666666.28482.peg.3649	Contig_4	9928	10383	+	Probable enoyl-CoA hydratase 1 (EC 4.2.1.17)	FIG00825052	ff
fig|6666666.28482.peg.3650	Contig_4	11746	10451	-	Butyryl-CoA dehydrogenase (EC 1.3.99.2)	FIG00009149	idu(39);Isoleucine_degradation idu(39);Lysine_fermentation idu(39);Butanol_Biosynthesis idu(39);Acetyl-CoA_fermentation_to_Butyrate idu(39);Valine_degradation idu(39);Anaerobic_respiratory_reductases
fig|6666666.28482.peg.3651	Contig_4	12526	11828	-	FIG00821964: hypothetical protein	FIG00821963	ff

fig|6666666.28482.peg.3652	Contig_4	12660	13556	+	Mn-containing catalase	FIG00459436	ff
fig|6666666.28482.peg.3653	Contig_4	14194	13616	-	hemerythrin HHE cation binding domain subfamily protein, putative	FIG01926271	ff
fig|6666666.28482.peg.3654	Contig_4	15339	14242	-	F420-dependent glucose-6-phosphate dehydrogenase Fgd3	FIG01311596	ff
fig|6666666.28482.peg.3655	Contig_4	15391	15996	+	puromycin N-acetyltransferase, putative	FIG00827377	ff
fig|6666666.28482.peg.3656	Contig_4	16328	16017	-	FIG00826668: hypothetical protein		
fig|6666666.28482.peg.3657	Contig_4	16500	16856	+	FIG00825777: hypothetical protein	FIG00825775	ff
fig|6666666.28482.peg.3658	Contig_4	17568	16894	-	Transcriptional regulator, TetR family	FIG00554417	ff
fig|6666666.28482.peg.3659	Contig_4	17639	18733	+	Flavodoxin reductases (ferredoxin-NADPH reductases) family 1	FIG01954108	idu(3);Anaerobic_respiratory_reductases
fig|6666666.28482.peg.3660	Contig_4	18759	19865	+	POSSIBLE LINOLEOYL-CoA DESATURASE (DELTA(6)-DESATURASE)	FIG00539310	ff
fig|6666666.28482.peg.3661	Contig_4	19968	20444	+	FIG00822225: hypothetical protein	FIG00822224	ff
fig|6666666.28482.peg.3662	Contig_4	20444	21487	+	FIG00823171: hypothetical protein	FIG00823170	ff
fig|6666666.28482.peg.3663	Contig_4	22666	21563	-	FIG00821861: hypothetical protein	FIG00821860	ff
fig|6666666.28482.peg.3664	Contig_4	23238	22663	-	Transcriptional regulator, TetR family	FIG00554417	ff

fig|6666666.28482.peg.3665	Contig_4	23267	24511	+	FIG00831221: hypothetical protein	FIG00831214	ff
fig|6666666.28482.peg.3666	Contig_4	24538	25428	+	Epoxide hydrolase (EC 3.3.2.9)	FIG00009455	idu(3);CBSS-410289.13.peg.3174
fig|6666666.28482.peg.3667	Contig_4	25653	25441	-	Molybdate-binding domain of ModE	FIG01304698	isu;Molybdenum_cofactor_biosynthesis
fig|6666666.28482.peg.3668	Contig_4	26328	25744	-	Transcriptional regulator, TetR family	FIG00554417	ff
fig|6666666.28482.peg.3669	Contig_4	26483	27823	+	Cytochrome P450 138	FIG01333220	ff
fig|6666666.28482.peg.3670	Contig_4	28340	27825	-	Peptide methionine sulfoxide reductase MsrA (EC 1.8.4.11)	FIG00002199	isu;Peptide_methionine_sulfoxide_reductase isu;Putative_sugar_ABC_transporter_(ytf_cluster)
fig|6666666.28482.peg.3671	Contig_4	28480	28986	+	FIG00821695: hypothetical protein	FIG00821694	ff
fig|6666666.28482.peg.3672	Contig_4	28983	30026	+	Dihydroflavonol-4-reductase (EC 1.1.1.219)	FIG00042188	idu(2);Biflavanoid_biosynthesis idu(2);Tannin_biosynthesis
fig|6666666.28482.peg.3673	Contig_4	31070	30045	-	Exopolyphosphatase (EC 3.6.1.11)	FIG00066575	idu(2);Polyphosphate idu(2);Phosphate_metabolism
fig|6666666.28482.peg.3674	Contig_4	31172	31555	+	FIG00822464: hypothetical protein	FIG00822461	ff
fig|6666666.28482.peg.3675	Contig_4	31900	31535	-	FIG00820093: hypothetical protein	FIG00820092	ff
fig|6666666.28482.peg.3676	Contig_4	31930	32940	+	FIG00822261: hypothetical protein	FIG00822260	ff
fig|6666666.28482.peg.3677	Contig_4	33028	33576	+	Non-specific DNA-binding protein Dps / Iron-binding ferritin-like antioxidant protein / Ferroxidase (EC 1.16.3.1)	FIG00109595	isu;Oxidative_stress isu;Oxidative_stress isu;Oxidative_stress

fig|6666666.28482.peg.3678	Contig_4	35003	33573	-	Chloride channel protein	FIG00731882	ff
fig|6666666.28482.peg.3679	Contig_4	35130	36092	+	Pirin	FIG01319925	ff
fig|6666666.28482.peg.3680	Contig_4	36167	36730	+	Transcriptional regulator, TetR family	FIG00554417	ff
fig|6666666.28482.peg.3681	Contig_4	36803	37744	+	FIG00823034: hypothetical protein	FIG00823032	ff
fig|6666666.28482.peg.3682	Contig_4	37764	38705	+	FIG00823034: hypothetical protein	FIG00823032	ff
fig|6666666.28482.peg.3683	Contig_4	38706	39632	+	FIG00829840: hypothetical protein	FIG00829836	ff
fig|6666666.28482.peg.3684	Contig_4	39746	41182	+	Aldehyde dehydrogenase (EC 1.2.1.3)	FIG01292786	idu(16);Pyruvate_metabolism_II:_acetyl-CoA,_acetogenesis_from_pyruvate idu(16);Glycerolipid_and_Glycerophospholipid_Metabolism_in_Bacteria idu(16);Methylglyoxal_Metabolism
fig|6666666.28482.peg.3685	Contig_4	41250	42101	+	Short-chain dehydrogenase/reductase SDR	FIG00546160	idu(13);CBSS-164757.7.peg.5020 idu(13);Transcription_repair_cluster
fig|6666666.28482.peg.3686	Contig_4	42118	43074	+	Quinone oxidoreductase (EC 1.6.5.5)	FIG00002982	idu(7);Quinone_oxidoreductase_family
fig|6666666.28482.peg.3687	Contig_4	44818	43097	-	PPE family protein	FIG00013075	icw(2);CBSS-164757.7.peg.5020
fig|6666666.28482.peg.3688	Contig_4	46658	45000	-	PPE family protein	FIG00013075	icw(2);CBSS-164757.7.peg.5020
fig|6666666.28482.peg.3689	Contig_4	47590	46769	-	Protein tyrosine phosphatase (EC 3.1.3.48)	FIG00819984	ff

fig|6666666.28482.peg.3690	Contig_4	48824	47583	-	Butyryl-CoA dehydrogenase (EC 1.3.99.2)	FIG00009149	idu(39);Isoleucine_degradation idu(39);Lysine_fermentation idu(39);Butanol_Biosynthesis idu(39);Acetyl-CoA_fermentation_to_Butyrate idu(39);Valine_degradation idu(39);Anaerobic_respiratory_reductases
fig|6666666.28482.peg.3691	Contig_4	48777	48899	+	hypothetical protein		
fig|6666666.28482.peg.3692	Contig_4	48934	49404	+	FIG00823327: hypothetical protein	FIG00823326	ff
fig|6666666.28482.peg.3693	Contig_4	49418	50560	+	NAD(P) transhydrogenase alpha subunit (EC 1.6.1.2)	FIG00001143	icw(2);Phosphate_metabolism
fig|6666666.28482.peg.3694	Contig_4	50561	50896	+	NAD(P) transhydrogenase alpha subunit (EC 1.6.1.2)	FIG00001143	icw(2);Phosphate_metabolism
fig|6666666.28482.peg.3695	Contig_4	50893	52317	+	NAD(P) transhydrogenase subunit beta (EC 1.6.1.2)	FIG00000629	isu;Phosphate_metabolism
fig|6666666.28482.peg.3696	Contig_4	52439	52314	-	FIG00821071: hypothetical protein	FIG00821070	ff
fig|6666666.28482.peg.3697	Contig_4	52531	52412	-	hypothetical protein		
fig|6666666.28482.peg.3698	Contig_4	52577	53200	+	Transcriptional regulator, TetR family	FIG00554417	ff
fig|6666666.28482.peg.3699	Contig_4	54663	53209	-	PE family protein	FIG01311212	ff
fig|6666666.28482.peg.3700	Contig_4	55682	54786	-	FIG00826838: hypothetical protein	FIG00826837	ff
fig|6666666.28482.peg.3701	Contig_4	55777	56877	+	FIG00827540: hypothetical protein	FIG00827537	ff

fig|6666666.28482.peg.3702	Contig_4	56896	57636	+	3-oxoacyl-[acyl-carrier protein] reductase (EC 1.1.1.100)	FIG00621114	idu(38);CBSS-246196.1.peg.364 idu(38);CBSS-164757.7.peg.5020 idu(38);Fatty_Acid_Biosynthesis_FASII
fig|6666666.28482.peg.3703	Contig_4	58246	58410	+	hypothetical protein		
fig|6666666.28482.peg.3704	Contig_4	59043	58459	-	hypothetical protein	FIG00638284	ff
fig|6666666.28482.peg.3705	Contig_4	59069	59248	+	hypothetical protein		
fig|6666666.28482.peg.3706	Contig_4	59399	60916	+	TldD family protein, Actinobacterial subgroup	FIG00018909	idu(1);CBSS-316273.3.peg.448 idu(1);Putative_TldE-TldD_proteolytic_complex
fig|6666666.28482.peg.3707	Contig_4	60913	62286	+	TldE/PmbA family protein, Actinobacterial subgroup	FIG00020189	icw(1);CBSS-316273.3.peg.448 icw(1);Putative_TldE-TldD_proteolytic_complex
fig|6666666.28482.peg.3708	Contig_4	62441	64120	+	Lipid A export ATP-binding/permease protein MsbA	FIG00017059	ff
fig|6666666.28482.peg.3709	Contig_4	64207	66039	+	Lipid A export ATP-binding/permease protein MsbA	FIG00017059	ff
fig|6666666.28482.peg.3710	Contig_4	67108	66068	-	hypothetical protein		
fig|6666666.28482.peg.3711	Contig_4	67597	67166	-	hypothetical protein		
fig|6666666.28482.peg.3712	Contig_4	68551	69480	+	Alpha-ketoglutarate-dependent taurine dioxygenase (EC 1.14.11.17)	FIG00001391	idu(1);Alkanesulfonate_assimilation
fig|6666666.28482.peg.3713	Contig_4	69573	70922	+	(S)-2-hydroxy-acid oxidase chain D (EC 1.1.3.15)	FIG00827136	ff
fig|6666666.28482.peg.3714	Contig_4	70957	71307	+	hypothetical protein		
fig|6666666.28482.peg.3715	Contig_4	71929	71390	-	Transcriptional regulator, TetR family	FIG00554417	ff

fig|6666666.28482.peg.3716	Contig_4	72794	71961	-	TesB-like acyl-CoA thioesterase 5	FIG00057597	isu;Acyl-CoA_thioesterase_II
fig|6666666.28482.peg.3717	Contig_4	73615	72812	-	FIG00822547: hypothetical protein	FIG00822546	ff
fig|6666666.28482.peg.3718	Contig_4	74155	73664	-	FIG00822620: hypothetical protein	FIG00822615	ff
fig|6666666.28482.peg.3719	Contig_4	74251	75141	+	Acyl-CoA thioesterase II (EC 3.1.2.-)	FIG00002217	icw(1);Acyl-CoA_thioesterase_II
fig|6666666.28482.peg.3720	Contig_4	75147	76061	+	FIG00827292: hypothetical protein	FIG00827279	ff
fig|6666666.28482.peg.3721	Contig_4	76062	77216	+	FIG00824968: hypothetical protein	FIG00824966	ff
fig|6666666.28482.peg.3722	Contig_4	77874	77197	-	FIG00821430: hypothetical protein	FIG00821427	ff
fig|6666666.28482.peg.3723	Contig_4	78365	77871	-	transcriptional regulatory protein		
fig|6666666.28482.peg.3724	Contig_4	78454	79374	+	NmrA-like		
fig|6666666.28482.peg.3725	Contig_4	79482	80669	+	acyl-CoA transferase	FIG01333498	ff
fig|6666666.28482.peg.3726	Contig_4	80760	81317	+	FIG00824032: hypothetical protein	FIG00824027	ff
fig|6666666.28482.peg.3727	Contig_4	81321	82025	+	FIG00822996: hypothetical protein	FIG00822995	ff
fig|6666666.28482.peg.3728	Contig_4	82022	83233	+	Nikkomycin biosynthesis protein SanQ	FIG01396881	ff
fig|6666666.28482.peg.3729	Contig_4	83235	84212	+	alcohol dehydrogenase	FIG00945718	ff
fig|6666666.28482.peg.3730	Contig_4	84209	84946	+	2,4-dihydroxyhept-2-ene-1,7-dioic acid aldolase (EC 4.1.2.-)	FIG00110223	idu(1);Aromatic_amino_acid_degradation idu(1);4-Hydroxyphenylacetic_acid_catabolic_pathway
fig|6666666.28482.peg.3731	Contig_4	84943	85335	+	conserved hypothetical protein		

fig|6666666.28482.peg.3732	Contig_4	85332	86060	+	3-oxoacyl-[acyl-carrier protein] reductase (EC 1.1.1.100)	FIG00621114	idu(38);CBSS-246196.1.peg.364 idu(38);CBSS-164757.7.peg.5020 idu(38);Fatty_Acid_Biosynthesis_FASI I
fig|6666666.28482.peg.3733	Contig_4	86057	87523	+	Cyclohexanone monooxygenase (EC 1.14.13.22)	FIG00975774	ff
fig|6666666.28482.peg.3734	Contig_4	87520	88470	+	Probable lipase/esterase	FIG01377204	ff
fig|6666666.28482.peg.3735	Contig_4	88490	89797	+	Alcohol dehydrogenase (EC 1.1.1.1)	FIG01007502	idu(17);Glycerolipid_and_Glycerophospholipid_Metabolism_in_Bacteria idu(17);Fermentations:_Mixed_acid idu(17);Butanol_Biosynthesis
fig|6666666.28482.peg.3736	Contig_4	89801	90256	+	putative 4-hydroxybenzoyl-CoA thioesterase	FIG00138976	isu;Gentisare_degradation
fig|6666666.28482.peg.3737	Contig_4	90286	90720	+	FIG00820956: hypothetical protein	FIG00820954	ff
fig|6666666.28482.peg.3738	Contig_4	91415	90723	-	Predicted regulator PutR for proline utilization, GntR family	FIG00004161	idu(2);Proline,_4-hydroxyproline_uptake_and_utilization
fig|6666666.28482.peg.3739	Contig_4	91575	93212	+	Long-chain-fatty-acid--CoA ligase (EC 6.2.1.3)	FIG00018699	idu(72);n-Phenylalkanoic_acid_degradation idu(72);Fatty_acid_metabolism_cluster idu(72);Biotin_synthesis_cluster idu(72);Biotin_biosynthesis

fig|6666666.28482.peg.3740	Contig_4	93461	94267	+	Conserved hypothetical integral membrane protein YrbE1A	FIG00690583	idu(7);Mycobacterium_virulence_operon_MCE_involved_in_cell_invasion
fig|6666666.28482.peg.3741	Contig_4	94269	95138	+	Conserved hypothetical integral membrane protein YrbE1B	FIG00691621	icw(2);Mycobacterium_virulence_operon_MCE_involved_in_cell_invasion
fig|6666666.28482.peg.3742	Contig_4	95141	96487	+	MCE-family protein Mce1A	FIG00820494	icw(1);Mycobacterium_virulence_operon_MCE_involved_in_cell_invasion
fig|6666666.28482.peg.3743	Contig_4	96484	97524	+	MCE-family protein Mce1B	FIG00763551	icw(3);Mycobacterium_virulence_operon_MCE_involved_in_cell_invasion
fig|6666666.28482.peg.3744	Contig_4	97548	99110	+	MCE-family protein Mce1C	FIG00821293	icw(4);Mycobacterium_virulence_operon_MCE_involved_in_cell_invasion
fig|6666666.28482.peg.3745	Contig_40	1996	770	-	Manganese transport protein MntH	FIG00554286	ff
fig|6666666.28482.peg.3746	Contig_40	2779	2087	-	3-oxoacyl-[acyl-carrier protein] reductase (EC 1.1.1.100)	FIG00621114	idu(38);CBSS-246196.1.peg.364 idu(38);CBSS-164757.7.peg.5020 idu(38);Fatty_Acid_Biosynthesis_FASII
fig|6666666.28482.peg.3747	Contig_40	3954	2878	-	FIG00820002: hypothetical protein	FIG00820001	ff
fig|6666666.28482.peg.3748	Contig_40	4903	4079	-	7-alpha-hydroxysteroid dehydrogenase (EC 1.1.1.159)	FIG01308941	ff

fig|6666666.28482.peg.3749	Contig_40	5289	6275	+	Phosphate ABC transporter, periplasmic phosphate-binding protein PstS (TC 3.A.1.7.1)	FIG00010508	icw(2);High_affinity_phosphate_transporter_and_control_of_PHO_regulon icw(2);Phosphate_metabolism idu(4);PhoR-PhoB_two-component_regulatory_system
fig|6666666.28482.peg.3750	Contig_40	6302	7279	+	Phosphate transport system permease protein PstC (TC 3.A.1.7.1)	FIG00018561	idu(1);High_affinity_phosphate_transporter_and_control_of_PHO_regulon idu(1);Phosphate_metabolism
fig|6666666.28482.peg.3751	Contig_40	7353	8255	+	Phosphate transport system permease protein PstA (TC 3.A.1.7.1)	FIG00083946	icw(1);High_affinity_phosphate_transporter_and_control_of_PHO_regulon icw(1);Phosphate_metabolism
fig|6666666.28482.peg.3752	Contig_40	9177	8269	-	Ku domain protein	FIG00001754	isu;DNA_Repair_Base_Excision isu;DNA_ligases isu;Nonhomologous_End-Joining_in_Bacteria isu;Bacillus_subtilis_scratch_-_gjo
fig|6666666.28482.peg.3753	Contig_40	10158	9250	-	Fructokinase (EC 2.7.1.4)	FIG00001653	isu;Fructose_utilization isu;Sucrose_utilization icw(1);Mannitol_Utilization
fig|6666666.28482.peg.3754	Contig_40	11567	10155	-	Multiple polyol-specific dehydrogenase (EC 1.1.1.-)	FIG00005739	isu;Mannitol_Utilization
fig|6666666.28482.peg.3755	Contig_40	12544	11687	-	FIG00829040: hypothetical protein	FIG00829038	ff
fig|6666666.28482.peg.3756	Contig_40	14076	12541	-	FIG00821215: hypothetical protein	FIG00821214	ff

fig|6666666.28482.peg.3757	Contig_40	14211	16469	+	ATP-dependent DNA ligase (EC 6.5.1.1) clustered with Ku protein, LigD	FIG00002268	idu(2);DNA_Repair_Base_Excision idu(2);DNA_ligases idu(2);Nonhomologous_End-Joining_in_Bacteria idu(2);Bacillus_subtilis_scratch_-_gjo
fig|6666666.28482.peg.3758	Contig_40	16466	18406	+	Fumarylacetoacetase (EC 3.7.1.2)	FIG00001596	idu(1);Homogentisate_pathway_of_aromatic_compound_degradation idu(1);Pterin_carbinolamine_dehydratase idu(1);Salicylate_and_gentisate_catabolism
fig|6666666.28482.peg.3759	Contig_40	19387	18473	-	O-Methyltransferase involved in polyketide biosynthesis	FIG01313020	ff
fig|6666666.28482.peg.3760	Contig_40	19471	20268	+	Enoyl-CoA hydratase (EC 4.2.1.17)	FIG00033206	idu(51);n-Phenylalkanoic_acid_degradation idu(51);Polyhydroxybutyrate_metabolism idu(51);Isoleucine_degradation idu(51);Fatty_acid_metabolism_cluster idu(51);Lysine_fermentation idu(51);Butanol_Biosynthesis idu(51);Acetyl-CoA_fermentation_to_Butyrate idu(51);Valine_degradation idu(51);Butyrate_metabolism_cluster
fig|6666666.28482.peg.3761	Contig_40	21138	20272	-	Hydride transferase 1 (Fragment)	FIG01353725	ff

fig|6666666.28482.peg.3762	Contig_40	21994	21230	-	FIG00823384: hypothetical protein	FIG00823383	ff
fig|6666666.28482.peg.3763	Contig_40	23452	22310	-	Cyclohexanone monooxygenase (EC 1.14.13.22)	FIG00975774	ff
fig|6666666.28482.peg.3764	Contig_40	24384	23479	-	FIG00822913: hypothetical protein	FIG00822912	ff
fig|6666666.28482.peg.3765	Contig_40	24484	25239	+	Precorrin-6A synthase (EC 2.1.1.152)	FIG00003681	isu;Coenzyme_B12_biosynthesis
fig|6666666.28482.peg.3766	Contig_40	25245	26108	+	Formamidopyrimidine-DNA glycosylase (EC 3.2.2.23)	FIG00035634	idu(3);DNA_Repair_Base_Excision
fig|6666666.28482.peg.3767	Contig_40	26138	26887	+	Oxidoreductase, short-chain dehydrogenase/reductase family (EC 1.1.1.-)	FIG01955289	idu(2);CBSS-291331.3.peg.3674 idu(2);Capsular_Polysaccharides_Biosynthesis_and_Assembly
fig|6666666.28482.peg.3768	Contig_40	28555	26891	-	Glucose-6-phosphate isomerase (EC 5.3.1.9)	FIG00000245	ff
fig|6666666.28482.peg.3769	Contig_40	28880	28608	-	Chorismate mutase I (EC 5.4.99.5)	FIG00001498	isu;Chorismate_Synthesis isu;Phenylalanine_and_Tyrosine_Branches_from_Chorismate
fig|6666666.28482.peg.3770	Contig_40	29269	32664	+	Pyruvate carboxyl transferase (EC 6.4.1.1)	FIG00001308	isu;Pyruvate_metabolism_I:_anaplerotic_reactions,_PEP
fig|6666666.28482.peg.3771	Contig_40	32908	32762	-	hypothetical protein		
fig|6666666.28482.peg.3772	Contig_40	32903	35224	+	ATP-dependent DNA helicase UvrD/PcrA	FIG00000372	idu(1);CBSS-393121.3.peg.1913 idu(1);DNA_repair,_bacterial_UvrD_and_related_helicases
fig|6666666.28482.peg.3773	Contig_40	35523	35221	-	FIG01258277: hypothetical protein	FIG01258275	ff
fig|6666666.28482.peg.3774	Contig_40	36832	35774	-	Phage peptidoglycan binding endopeptidase	FIG00821878	ff

fig|6666666.28482.peg.3775	Contig_40	37179	36862	-	hypothetical protein		
fig|6666666.28482.peg.3776	Contig_40	37228	38322	+	Succinyl-CoA ligase [ADP-forming] beta chain (EC 6.2.1.5)	FIG00000337	icw(1);Serine-glyoxylate_cycle icw(1);TCA_Cycle
fig|6666666.28482.peg.3777	Contig_40	38337	39239	+	Succinyl-CoA ligase [ADP-forming] alpha chain (EC 6.2.1.5)	FIG00038611	isu;Serine-glyoxylate_cycle isu;TCA_Cycle
fig|6666666.28482.peg.3778	Contig_40	39632	39327	-	PE_PGRS family protein		
fig|6666666.28482.peg.3779	Contig_41	236	2533	+	Transmembrane transport protein MmpL13b	FIG01314334	ff
fig|6666666.28482.peg.3780	Contig_41	2785	2534	-	hypothetical protein		
fig|6666666.28482.peg.3781	Contig_41	2889	3920	+	Iron-sulfur cluster-binding protein	FIG00000517	ff
fig|6666666.28482.peg.3782	Contig_41	4497	5543	+	Sulfate and thiosulfate binding protein CysP	FIG00001000	idu(2);Cysteine_Biosynthesis idu(2);Inorganic_Sulfur_Assimilation
fig|6666666.28482.peg.3783	Contig_41	5540	6430	+	Sulfate transport system permease protein CysT	FIG00018897	icw(2);Cysteine_Biosynthesis icw(2);Inorganic_Sulfur_Assimilation
fig|6666666.28482.peg.3784	Contig_41	6427	7236	+	Sulfate transport system permease protein CysW	FIG01272192	icw(1);Cysteine_Biosynthesis icw(1);Inorganic_Sulfur_Assimilation
fig|6666666.28482.peg.3785	Contig_41	7253	8314	+	Sulfate and thiosulfate import ATP-binding protein CysA (EC 3.6.3.25)	FIG00000959	idu(1);Uptake_of_selenate_and_selenite icw(3);Cysteine_Biosynthesis icw(3);Inorganic_Sulfur_Assimilation
fig|6666666.28482.peg.3786	Contig_41	8622	8329	-	protein of unknown function DUF732		
fig|6666666.28482.peg.3787	Contig_41	9102	8728	-	hypothetical protein		

fig|6666666.28482.peg.3788	Contig_41	9442	10308	+	CDP-alcohol phosphatidyltransferase		
fig|6666666.28482.peg.3789	Contig_41	10305	10943	+	1-acyl-sn-glycerol-3-phosphate acyltransferase (EC 2.3.1.51)	FIG00005243	idu(6);Glycerolipid_and_Glycerophospholipid_Metabolism_in_Bacteria idu(6);Ribosome_post-transcriptional_modification_and_chromosomal_segregation_cluster
fig|6666666.28482.peg.3790	Contig_41	11855	10950	-	Phosphatidate cytidylyltransferase (EC 2.7.7.41)	FIG00000097	icw(1);Glycerolipid_and_Glycerophospholipid_Metabolism_in_Bacteria
fig|6666666.28482.peg.3791	Contig_41	12021	13826	+	Asparagine synthetase [glutamine-hydrolyzing] (EC 6.3.5.4)	FIG00035175	idu(1);Glutamine,_Glutamate,_Aspartate_and_Asparagine_Biosynthesis idu(1);Glutamate_and_Aspartate_uptake_in_Bacteria icw(1);Cyanophycin_Metabolism
fig|6666666.28482.peg.3792	Contig_41	13823	15643	+	Cyanophycin synthase (EC 6.3.2.29)(EC 6.3.2.30)	FIG00003542	isu;Cyanophycin_Metabolism
fig|6666666.28482.peg.3793	Contig_41	15697	17181	+	sugar transporter	FIG00515631	ff
fig|6666666.28482.peg.3794	Contig_41	17376	17819	+	hypothetical protein		
fig|6666666.28482.peg.3795	Contig_41	17971	20148	+	Glutamine synthetase type III, GlnN (EC 6.3.1.2)	FIG00132839	isu;Glutamine,_Glutamate,_Aspartate_and_Asparagine_Biosynthesis isu;Ammonia_assimilation isu;Glutamine_synthetases isu;Peptidoglycan_Biosynthesis
fig|6666666.28482.peg.3796	Contig_41	20326	22293	+	Serine/threonine protein kinase (EC 2.7.11.1)	FIG00933379	ff
fig|6666666.28482.peg.3797	Contig_41	22474	22809	+	hypothetical protein		

fig|6666666.28482.peg.3798	Contig_41	24658	22817	-	Probable serine/threonine-protein kinase pknH (EC 2.7.11.1)	FIG00824811	ff
fig|6666666.28482.peg.3799	Contig_41	25134	24796	-	FIG00824585: hypothetical protein	FIG00824584	ff
fig|6666666.28482.peg.3800	Contig_41	25375	25863	+	FIG00820526: hypothetical protein	FIG00820525	ff
fig|6666666.28482.peg.3801	Contig_41	26528	25869	-	5-amino-6-(5-phosphoribosylamino)uracil reductase (EC 1.1.1.193) homolog	FIG00995439	ff
fig|6666666.28482.peg.3802	Contig_41	27039	26542	-	FIG00830308: hypothetical protein	FIG00830305	ff
fig|6666666.28482.peg.3803	Contig_41	28282	27044	-	amino acid permease family protein	FIG01308903	ff
fig|6666666.28482.peg.3804	Contig_41	28360	29070	+	Arsenate reductase (EC 1.20.4.1)	FIG00000770	idu(5);Transcription_repair_cluster idu(5);CBSS-315749.4.peg.3658 idu(5);Arsenic_resistance idu(5);Anaerobic_respiratory_reductases
fig|6666666.28482.peg.3805	Contig_41	29067	30545	+	Multicopper oxidase	FIG00060246	idu(2);Copper_homeostasis
fig|6666666.28482.peg.3806	Contig_41	30575	32410	+	Serine/threonine protein kinase (EC 2.7.11.1)	FIG00933379	ff
fig|6666666.28482.peg.3807	Contig_41	32507	34408	+	serine/threonine-protein kinase PknE(EC:2.7.11.1)		
fig|6666666.28482.peg.3808	Contig_41	34528	35274	+	hypothetical protein		
fig|6666666.28482.peg.3809	Contig_41	36400	35264	-	FIG00827535: hypothetical protein	FIG00827531	ff
fig|6666666.28482.peg.3810	Contig_41	36615	37061	+	hypothetical protein	FIG00638284	ff

fig|6666666.28482.peg.3811	Contig_41	37128	37994	+	FIG00820086: hypothetical protein	FIG00820085	ff
fig|6666666.28482.peg.3812	Contig_41	37961	38881	+	putative esterase	FIG00016132	idu(2);Alpha-Amylase_locus_in_Streptocococcus
fig|6666666.28482.peg.3813	Contig_41	39120	39359	+	hypothetical protein		
fig|6666666.28482.peg.3814	Contig_41	39892	40320	+	hypothetical protein		
fig|6666666.28482.peg.3815	Contig_41	40400	41074	+	Transcriptional regulator, PadR family	FIG00002474	idu(4);CBSS-1352.1.peg.856
fig|6666666.28482.peg.3816	Contig_41	42774	41071	-	3-oxosteroid 1-dehydrogenase (EC 1.3.99.4)	FIG01318047	ff
fig|6666666.28482.peg.3817	Contig_41	42875	43999	+	Terminal oxygenase KshA	FIG01318144	ff
fig|6666666.28482.peg.3818	Contig_41	43996	44304	+	FIG00824565: hypothetical protein	FIG00824562	ff
fig|6666666.28482.peg.3819	Contig_41	45188	44313	-	Cyclopropane-fatty-acyl-phospholipid synthase (EC 2.1.1.79)	FIG00001555	ff
fig|6666666.28482.peg.3820	Contig_41	46148	45432	-	FIG00831914: hypothetical protein	FIG00831905	ff
fig|6666666.28482.peg.3821	Contig_41	47308	46232	-	FIG00820875: hypothetical protein	FIG00820874	ff
fig|6666666.28482.peg.3822	Contig_41	48271	47354	-	LipH	FIG01375311	ff
fig|6666666.28482.peg.3823	Contig_41	49205	48339	-	Short-chain dehydrogenase/reductase SDR:Glucose/ribitol dehydrogenase	FIG00862012	ff
fig|6666666.28482.peg.3824	Contig_41	49681	49202	-	FIG00821271: hypothetical protein	FIG00821270	ff

fig|6666666.28482.peg.3825	Contig_41	51383	49782	-	Long-chain-fatty-acid--CoA ligase (EC 6.2.1.3)	FIG00018699	idu(72);n-Phenylalkanoic_acid_degradation idu(72);Fatty_acid_metabolism_cluster idu(72);Biotin_synthesis_cluster idu(72);Biotin_biosynthesis
fig|6666666.28482.peg.3826	Contig_41	51477	53408	+	Cyclohexanone monooxygenase (EC 1.14.13.22)	FIG00975774	ff
fig|6666666.28482.peg.3827	Contig_41	53438	54292	+	Enoyl-[acyl-carrier-protein] reductase [NADPH] (EC 1.3.1.10)	FIG00032108	idu(2);Fatty_Acid_Biosynthesis_FASII
fig|6666666.28482.peg.3828	Contig_41	54941	54294	-	FIG00822739: hypothetical protein	FIG00822737	ff
fig|6666666.28482.peg.3829	Contig_41	55281	54934	-	FIG00820833: hypothetical protein	FIG00820832	ff
fig|6666666.28482.peg.3830	Contig_41	56276	55350	-	Esterase/lipase	FIG00932446	ff
fig|6666666.28482.peg.3831	Contig_41	57082	56273	-	Transcriptional regulator, IclR family	FIG00022569	idu(12);Homogentisate_pathway_of_aromatic_compound_degradation
fig|6666666.28482.peg.3832	Contig_41	57671	57228	-	hypothetical protein		
fig|6666666.28482.peg.3833	Contig_41	59151	57685	-	fumarate reductase/succinate dehydrogenase flavoprotein, N-terminal:FAD dependent oxidoreductase	FIG00141996	idu(1);Succinate_dehydrogenase
fig|6666666.28482.peg.3834	Contig_41	60056	59148	-	2,3-dihydroxybiphenyl 1,2-dioxygenase (EC 1.13.11.39)	FIG01272995	isu;Aromatic_dioxygenase_mess isu;Biphenyl_Degradation isu;Dioxygenases_(EC_1.13.11.-)

fig|6666666.28482.peg.3835	Contig_41	61261	60077	-	hydroxylase	FIG01000257	ff
fig|6666666.28482.peg.3836	Contig_41	62207	61416	-	FIG00821553: hypothetical protein	FIG00821552	ff
fig|6666666.28482.peg.3837	Contig_41	63829	62228	-	Cyclohexanone monooxygenase (EC 1.14.13.22)	FIG00975774	ff
fig|6666666.28482.peg.3838	Contig_41	64015	64302	+	oxidoreductase, NAD/FAD-binding		
fig|6666666.28482.peg.3839	Contig_41	64309	65142	+	FIG00825001: hypothetical protein	FIG00824997	ff
fig|6666666.28482.peg.3840	Contig_41	65151	65942	+	Short chain oxidoreductase	FIG00830516	ff
fig|6666666.28482.peg.3841	Contig_41	65939	66811	+	luciferase family protein		
fig|6666666.28482.peg.3842	Contig_41	66808	67311	+	hypothetical protein		
fig|6666666.28482.peg.3843	Contig_41	67390	67977	+	hypothetical protein		
fig|6666666.28482.peg.3844	Contig_41	68107	69378	+	FIG00827177: hypothetical protein	FIG00827174	ff
fig|6666666.28482.peg.3845	Contig_41	70299	69430	-	2-hydroxymuconic semialdehyde hydrolase (EC 3.7.1.9)	FIG00042074	idu(1);Central_meta-cleavage_pathway_of_aromatic_compound_degradation
fig|6666666.28482.peg.3846	Contig_41	70374	70811	+	hypothetical protein		
fig|6666666.28482.peg.3847	Contig_41	72298	70910	-	FIG00828809: hypothetical protein	FIG00828807	ff
fig|6666666.28482.peg.3848	Contig_41	72937	72503	-	FIG00828690: hypothetical protein	FIG00828686	ff
fig|6666666.28482.peg.3849	Contig_41	73161	72979	-	hypothetical protein		
fig|6666666.28482.peg.3850	Contig_41	73282	73707	+	pyridoxamine 5'-phosphate oxidase-related, FMN-binding		
fig|6666666.28482.peg.3851	Contig_41	74758	73832	-	FIG00830838: hypothetical protein	FIG00830837	ff

fig|6666666.28482.peg.3852	Contig_41	74831	77065	+	Formate dehydrogenase-O, major subunit (EC 1.2.1.2)	FIG00011659	idu(1);Formate_hydrogenase
fig|6666666.28482.peg.3853	Contig_41	77167	77466	+	hypothetical protein		
fig|6666666.28482.peg.3854	Contig_41	78346	77495	-	short chain dehydrogenase		
fig|6666666.28482.peg.3855	Contig_41	78623	79711	+	esterase	FIG01380254	ff
fig|6666666.28482.peg.3856	Contig_41	79876	79712	-	hypothetical protein		
fig|6666666.28482.peg.3857	Contig_41	79904	82441	+	Probable conserved transmembrane ATP-binding protein ABC transporter	FIG01323737	ff
fig|6666666.28482.peg.3858	Contig_41	82721	82557	-	FIG00825214: hypothetical protein	FIG00825212	ff
fig|6666666.28482.peg.3859	Contig_41	83808	82822	-	hypothetical protein	FIG00638284	ff
fig|6666666.28482.peg.3860	Contig_41	84380	83811	-	Carboxymuconolactone decarboxylase		
fig|6666666.28482.peg.3861	Contig_41	85259	84435	-	hypothetical protein		
fig|6666666.28482.peg.3862	Contig_41	85864	85256	-	Transcriptional regulator, TetR family	FIG00554417	ff
fig|6666666.28482.peg.3863	Contig_41	86026	87204	+	FIG00824975: hypothetical protein	FIG00824974	ff
fig|6666666.28482.peg.3864	Contig_41	87194	88303	+	FIG00825789: hypothetical protein	FIG00825788	ff
fig|6666666.28482.peg.3865	Contig_41	88296	88796	+	FIG00823936: hypothetical protein	FIG00823935	ff
fig|6666666.28482.peg.3866	Contig_41	88804	89247	+	FIG00831340: hypothetical protein	FIG00831338	ff
fig|6666666.28482.peg.3867	Contig_41	89244	90446	+	FIG00827145: hypothetical protein	FIG00827143	ff
fig|6666666.28482.peg.3868	Contig_41	90448	90855	+	FIG00821070: hypothetical protein	FIG00821069	ff

fig|6666666.28482.peg.3869	Contig_41	90879	91838	+	Esterase/lipase	FIG00932446	ff
fig|6666666.28482.peg.3870	Contig_41	91835	93433	+	FIG00820978: hypothetical protein	FIG00820973	ff
fig|6666666.28482.peg.3871	Contig_41	93430	95007	+	Long-chain-fatty-acid--CoA ligase (EC 6.2.1.3)	FIG00018699	idu(72);n-Phenylalkanoic_acid_degradation idu(72);Fatty_acid_metabolism_cluster idu(72);Biotin_synthesis_cluster idu(72);Biotin_biosynthesis
fig|6666666.28482.peg.3872	Contig_41	96172	95009	-	hypothetical protein		
fig|6666666.28482.peg.3873	Contig_41	96708	96205	-	hypothetical protein		
fig|6666666.28482.peg.3874	Contig_41	97146	96709	-	hypothetical protein		
fig|6666666.28482.peg.3875	Contig_41	97278	99539	+	Transmembrane transport protein MmpL13b	FIG01314334	ff
fig|6666666.28482.peg.3876	Contig_41	100582	99731	-	integral membrane protein		
fig|6666666.28482.peg.3877	Contig_41	101022	100675	-	hypothetical protein		
fig|6666666.28482.peg.3878	Contig_41	101522	101049	-	hypothetical protein		
fig|6666666.28482.peg.3879	Contig_41	101622	101915	+	hypothetical protein		
fig|6666666.28482.peg.3880	Contig_41	101965	103578	+	Long-chain-fatty-acid--CoA ligase (EC 6.2.1.3)	FIG00018699	idu(72);n-Phenylalkanoic_acid_degradation idu(72);Fatty_acid_metabolism_cluster idu(72);Biotin_synthesis_cluster idu(72);Biotin_biosynthesis
fig|6666666.28482.peg.3881	Contig_41	103565	104338	+	Ribulose-5-phosphate 4-epimerase and related epimerases and aldolases	FIG00004579	ff
fig|6666666.28482.peg.3882	Contig_41	104464	105732	+	amidohydrolase	FIG01338948	ff

fig|6666666.28482.peg.3883	Contig_41	106025	105729	-	hypothetical protein		
fig|6666666.28482.peg.3884	Contig_41	106232	106080	-	hypothetical protein		
fig|6666666.28482.peg.3885	Contig_41	106270	107424	+	serine/threonine protein kinase		
fig|6666666.28482.peg.3886	Contig_41	107579	108574	+	putative hydrolase		
fig|6666666.28482.peg.3887	Contig_41	108717	108941	+	hypothetical protein		
fig|6666666.28482.peg.3888	Contig_41	109986	108967	-	2-nitropropane dioxygenase (EC 1.13.11.32)	FIG01304356	idu(5);CBSS-164757.7.peg.5020
fig|6666666.28482.peg.3889	Contig_41	110085	111686	+	Arylsulfatase (EC 3.1.6.1)	FIG00016566	idu(3);Sulfatases_and_sulfatase_modifying_factor_1 idu(3);Galactosylceramide_and_Sulfatide_metabolism idu(3);Steroid_sulfates idu(3);Alkanesulfonate_assimilation
fig|6666666.28482.peg.3890	Contig_41	111743	112741	+	NAD binding oxidoreductase	FIG01609121	ff
fig|6666666.28482.peg.3891	Contig_41	112749	113816	+	hypothetical protein		
fig|6666666.28482.peg.3892	Contig_41	114710	113898	-	conserved hypothetical protein		
fig|6666666.28482.peg.3893	Contig_41	115252	114710	-	protein export protein SecD, putative		
fig|6666666.28482.peg.3894	Contig_41	115425	116117	+	transcriptional regulator, TetR family protein	FIG00869404	ff
fig|6666666.28482.peg.3895	Contig_41	116241	117443	+	putative cytochrome P450 hydroxylase	FIG00032437	idu(49);Nitric_oxide_synthase
fig|6666666.28482.peg.3896	Contig_41	117443	118279	+	FIG00828457: hypothetical protein	FIG00828455	ff

fig|6666666.28482.peg.3897	Contig_41	118295	119263	+	Naphthoate synthase (EC 4.1.3.36)	FIG00071623	idu(2);Menaquinone_and_Phylloquinone_Biosynthesis_--_gjo idu(2);Menaquinone_and_Phylloquinone_Biosynthesis
fig|6666666.28482.peg.3898	Contig_41	119283	121619	+	FIG00824362: hypothetical protein	FIG00824361	ff
fig|6666666.28482.peg.3899	Contig_41	121616	122377	+	clavaldehyde dehydrogenase	FIG00144627	idu(1);Clavulanic_acid_biosynthesis
fig|6666666.28482.peg.3900	Contig_41	122374	123627	+	FIG00828983: hypothetical protein	FIG00828976	ff
fig|6666666.28482.peg.3901	Contig_41	123690	125054	+	Partial REP13E12 repeat protein	FIG00820827	ff
fig|6666666.28482.peg.3902	Contig_41	126483	125365	-	hypothetical protein		
fig|6666666.28482.peg.3903	Contig_41	127717	126473	-	FIG00826794: hypothetical protein	FIG00826792	ff
fig|6666666.28482.peg.3904	Contig_41	128881	127718	-	BarH	FIG00668014	ff
fig|6666666.28482.peg.3905	Contig_41	129845	128889	-	short chain dehydrogenase	FIG01311525	ff
fig|6666666.28482.peg.3906	Contig_41	131077	129842	-	putative cytochrome P450 hydroxylase	FIG00032437	idu(49);Nitric_oxide_synthase
fig|6666666.28482.peg.3907	Contig_41	131751	131074	-	Transcriptional regulator, TetR family	FIG00554417	ff
fig|6666666.28482.peg.3908	Contig_41	131980	132456	+	FIG00828429: hypothetical protein	FIG00828428	ff
fig|6666666.28482.peg.3909	Contig_41	132580	132464	-	hypothetical protein		
fig|6666666.28482.peg.3910	Contig_41	132736	132909	+	hypothetical protein		
fig|6666666.28482.peg.3911	Contig_41	134214	133258	-	Beta-lactamase class C and other penicillin binding proteins	FIG00000681	idu(2);Beta-lactamase
fig|6666666.28482.peg.3912	Contig_42	200	865	+	YkuD domain-containing hypothetical lipoprotein	FIG01375022	ff

fig|6666666.28482.peg.3913	Contig_42	972	1559	+	Transcriptional regulator, TetR family	FIG00554417	ff
fig|6666666.28482.peg.3914	Contig_42	1556	3805	+	Formate dehydrogenase-O, major subunit (EC 1.2.1.2)	FIG00011659	idu(1);Formate_hydrogenase
fig|6666666.28482.peg.3915	Contig_42	5839	3854	-	Metallopeptidase	FIG01213213	ff
fig|6666666.28482.peg.3916	Contig_42	5882	6529	+	Possible membrane protein	FIG01121868	ff
fig|6666666.28482.peg.3917	Contig_42	6526	7215	+	Possible membrane protein	FIG01121868	ff
fig|6666666.28482.peg.3918	Contig_42	7744	7220	-	FIG00821048: hypothetical protein	FIG00821047	ff
fig|6666666.28482.peg.3919	Contig_42	10758	7741	-	Transmembrane transport protein MmpL13	FIG00821827	ff
fig|6666666.28482.peg.3920	Contig_42	11080	11601	+	POSSIBLE EXPORTED PROTEIN	FIG01386146	ff
fig|6666666.28482.peg.3921	Contig_42	12806	11634	-	Possible membrane protein	FIG01121868	ff
fig|6666666.28482.peg.3922	Contig_42	12955	14112	+	Possible membrane protein	FIG01121868	ff
fig|6666666.28482.peg.3923	Contig_42	16918	14120	-	Transmembrane transport protein MmpL5	FIG01001691	ff
fig|6666666.28482.peg.3924	Contig_42	17702	16992	-	FIG01001556: hypothetical protein	FIG00820731	ff
fig|6666666.28482.peg.3925	Contig_42	18445	17720	-	tRNA (guanine46-N7-)-methyltransferase (EC 2.1.1.33)	FIG00002733	isu;RNA_methylation
fig|6666666.28482.peg.3926	Contig_42	18699	19784	+	FIG032248: hypothetical protein	FIG00032248	icw(1);CBSS-336982.3.peg.219

fig|6666666.28482.peg.3927	Contig_42	19742	21292	+	FIG033897: hypothetical protein	FIG00033897	isu;CBSS-336982.3.peg.219
fig|6666666.28482.peg.3928	Contig_42	21476	23302	+	Phosphoenolpyruvate carboxykinase [GTP] (EC 4.1.1.32)	FIG00001643	icw(2);CBSS-336982.3.peg.219 isu;Pyruvate_metabolism_I:_anaplerotic_reactions,_PEP
fig|6666666.28482.peg.3929	Contig_42	23855	23379	-	Redox-sensitive transcriptional activator SoxR	FIG00004255	isu;Oxidative_stress
fig|6666666.28482.peg.3930	Contig_42	23917	24630	+	possible short chain dehydrogenase	FIG00995056	ff
fig|6666666.28482.peg.3931	Contig_42	24627	25658	+	S-adenosylmethionine:tRNA ribosyltransferase-isomerase (EC 5.-.-.-)	FIG00000334	ff
fig|6666666.28482.peg.3932	Contig_42	25692	27218	+	Long-chain-fatty-acid--CoA ligase (EC 6.2.1.3)	FIG00018699	idu(72);n-Phenylalkanoic_acid_degradation idu(72);Fatty_acid_metabolism_cluster idu(72);Biotin_synthesis_cluster idu(72);Biotin_biosynthesis
fig|6666666.28482.peg.3933	Contig_42	27215	28177	+	Enoyl-CoA hydratase [valine degradation] (EC 4.2.1.17)	FIG00014562	icw(1);Valine_degradation
fig|6666666.28482.peg.3934	Contig_42	28625	28197	-	FIG00824252: hypothetical protein	FIG00824247	ff

fig|6666666.28482.peg.3935	Contig_42	29830	28703	-	Butyryl-CoA dehydrogenase (EC 1.3.99.2)	FIG00009149	idu(39);Isoleucine_degradation idu(39);Lysine_fermentation idu(39);Butanol_Biosynthesis idu(39);Acetyl-CoA_fermentation_to_Butyrate idu(39);Valine_degradation idu(39);Anaerobic_respiratory_reductases
fig|6666666.28482.peg.3936	Contig_42	29879	30874	+	FIG024080: hypothetical protein	FIG00823305	ff
fig|6666666.28482.peg.3937	Contig_42	30900	32099	+	FIG00820062: hypothetical protein	FIG00820061	ff
fig|6666666.28482.peg.3938	Contig_42	32093	33397	+	FIG00821918: hypothetical protein	FIG00821917	ff
fig|6666666.28482.peg.3939	Contig_42	34307	33405	-	Esterase LipW	FIG00821483	ff
fig|6666666.28482.peg.3940	Contig_42	34512	36626	+	Assimilatory nitrate reductase large subunit (EC:1.7.99.4)	FIG00059898	idu(1);Nitrate_and_nitrite_ammonification
fig|6666666.28482.peg.3941	Contig_42	36623	36976	+	hypothetical protein		
fig|6666666.28482.peg.3942	Contig_42	36995	39514	+	Nitrite reductase [NAD(P)H] large subunit (EC 1.7.1.4)	FIG00000873	icw(1);Nitrate_and_nitrite_ammonification
fig|6666666.28482.peg.3943	Contig_42	39511	39870	+	Rieske (2Fe-2S) domain protein		
fig|6666666.28482.peg.3944	Contig_42	40085	40738	+	Methyltransferase type 12		
fig|6666666.28482.peg.3945	Contig_42	40735	42024	+	PROBABLE CONSERVED TRANSMEMBRANE PROTEIN	FIG00662132	ff

fig|6666666.28482.peg.3946	Contig_42	42026	42523	+	PROBABLE CONSERVED TRANSMEMBRANE PROTEIN	FIG00662132	ff
fig|6666666.28482.peg.3947	Contig_42	42666	43838	+	Esterase LipC	FIG00824527	ff
fig|6666666.28482.peg.3948	Contig_42	43883	45292	+	Diacyglycerol O-acyltransferase (EC 2.3.1.20)	FIG01344808	ff
fig|6666666.28482.peg.3949	Contig_42	45401	46183	+	Enoyl-CoA hydratase (EC 4.2.1.17)	FIG00033206	icw(1);n-Phenylalkanoic_acid_degradation idu(51);Polyhydroxybutyrate_metabolism idu(51);Isoleucine_degradation icw(1);Fatty_acid_metabolism_cluster idu(51);Lysine_fermentation idu(51);Butanol_Biosynthesis idu(51);Acetyl-CoA_fermentation_to_Butyrate idu(51);Valine_degradation idu(51);Butyrate_metabolism_cluster
fig|6666666.28482.peg.3950	Contig_42	46770	46180	-	Transcriptional regulator, TetR family	FIG00554417	ff
fig|6666666.28482.peg.3951	Contig_42	46873	47412	+	hypothetical protein		
fig|6666666.28482.peg.3952	Contig_42	47479	48978	+	Long-chain-fatty-acid--CoA ligase (EC 6.2.1.3)	FIG00018699	idu(72);n-Phenylalkanoic_acid_degradation idu(72);Fatty_acid_metabolism_cluster idu(72);Biotin_synthesis_cluster idu(72);Biotin_biosynthesis

fig|6666666.28482.peg.3953	Contig_42	48975	49271	+	FIG00826455: hypothetical protein	FIG00826454	ff
fig|6666666.28482.peg.3954	Contig_42	50731	49289	-	hypothetical protein	FIG00638284	ff
fig|6666666.28482.peg.3955	Contig_42	52353	50956	-	PE-PGRS family protein		idu(16);PPE_gene_cluster_in_Mycobacteria
fig|6666666.28482.peg.3956	Contig_42	53914	52487	-	PE-PGRS FAMILY PROTEIN		
fig|6666666.28482.peg.3957	Contig_42	55453	54002	-	PE-PGRS FAMILY PROTEIN		
fig|6666666.28482.peg.3958	Contig_42	56219	55587	-	Transcriptional regulator, TetR family	FIG00554417	ff
fig|6666666.28482.peg.3959	Contig_42	56374	56243	-	FIG00823468: hypothetical protein	FIG00823464	ff
fig|6666666.28482.peg.3960	Contig_43	2114	1218	-	FIG00820338: hypothetical protein	FIG00820337	ff
fig|6666666.28482.peg.3961	Contig_43	2186	2890	+	Transcriptional regulator, TetR family	FIG00554417	ff
fig|6666666.28482.peg.3962	Contig_43	3990	3409	-	Glyoxalase family protein	FIG01955890	ff
fig|6666666.28482.peg.3963	Contig_43	4083	4727	+	Transcriptional regulator, TetR family	FIG00554417	ff
fig|6666666.28482.peg.3964	Contig_43	4751	5857	+	FIG00820646: hypothetical protein	FIG00820644	ff
fig|6666666.28482.peg.3965	Contig_43	5848	6804	+	D-3-phosphoglycerate dehydrogenase (EC 1.1.1.95)	FIG00031250	idu(4);Serine_Biosynthesis idu(4);Pyridoxin_(Vitamin_B6)_Biosynthesis
fig|6666666.28482.peg.3966	Contig_43	6860	9061	+	putative acyl-CoA dehydrogenase		
fig|6666666.28482.peg.3967	Contig_44	151	1104	+	Glutamate-ammonia-ligase adenylyltransferase (EC 2.7.7.42)	FIG00006278	isu;Ammonia_assimilation isu;CBSS-316057.3.peg.3521

fig|6666666.28482.peg.3968	Contig_44	1122	1748	+	FIG00822716: hypothetical protein	FIG00822713	ff
fig|6666666.28482.peg.3969	Contig_44	1745	2605	+	FIG00820321: hypothetical protein	FIG00820319	ff
fig|6666666.28482.peg.3970	Contig_44	2615	2833	+	hypothetical protein		
fig|6666666.28482.peg.3971	Contig_44	3511	3888	+	hypothetical protein		
fig|6666666.28482.peg.3972	Contig_44	5471	4035	-	Glutamine synthetase type I (EC 6.3.1.2)	FIG00134889	idu(2);Glutamine,_Glutamate,_Aspartate_and_Asparagine_Biosynthesis icw(1);Ammonia_assimilation idu(2);Glutamine_synthetases idu(2);Peptidoglycan_Biosynthesis
fig|6666666.28482.peg.3973	Contig_44	5721	6152	+	PROBABLE MEMBRANE PROTEIN	FIG00816212	ff
fig|6666666.28482.peg.3974	Contig_44	6911	6159	-	Transmembrane protein MT2276, clustered with lipoate gene	FIG00006368	ff
fig|6666666.28482.peg.3975	Contig_44	7884	6952	-	Lipoate synthase	FIG00000362	icw(1);Lipoic_acid_synthesis_cluster icw(1);Lipoic_acid_metabolism
fig|6666666.28482.peg.3976	Contig_44	8579	7881	-	Octanoate-[acyl-carrier-protein]-protein-N-octanoyltransferase	FIG00000528	isu;Lipoic_acid_synthesis_cluster isu;Lipoic_acid_metabolism
fig|6666666.28482.peg.3977	Contig_44	9537	8626	-	Cell division inhibitor	FIG00002048	idu(1);CBSS-83333.1.peg.946 idu(1);Persister_Cells

fig|6666666.28482.peg.3978	Contig_44	11326	9542	-	Dihydrolipoamide acyltransferase component of branched-chain alpha-keto acid dehydrogenase complex (EC 2.3.1.168)	FIG01304883	idu(1);Isoleucine_degradation idu(1);Leucine_Degradation_and_HMG-CoA_Metabolism idu(1);Valine_degradation idu(1);Dehydrogenase_complexes
fig|6666666.28482.peg.3979	Contig_44	11504	13276	+	Oxidoreductase, short-chain dehydrogenase/reductase family (EC 1.1.1.-)	FIG01955289	idu(2);CBSS-291331.3.peg.3674 idu(2);Capsular_Polysaccharides_Biosynthesis_and_Assembly
fig|6666666.28482.peg.3980	Contig_44	13728	13384	-	FIG00825986: hypothetical protein	FIG00825983	ff
fig|6666666.28482.peg.3981	Contig_44	13853	14308	+	hypothetical protein		
fig|6666666.28482.peg.3982	Contig_44	14313	15905	+	Phytoene dehydrogenase and related proteins	FIG00139162	ff
fig|6666666.28482.peg.3983	Contig_44	17536	15989	-	Cytosol aminopeptidase PepA (EC 3.4.11.1)	FIG00000336	isu;Aminopeptidases_(EC_3.4.11.-) isu;Dehydrogenase_complexes
fig|6666666.28482.peg.3984	Contig_44	18672	17548	-	Adenylate cyclase (EC 4.6.1.1)	FIG00001294	idu(25);cAMP_signaling_in_bacteria
fig|6666666.28482.peg.3985	Contig_44	18766	19869	+	Aminomethyltransferase (glycine cleavage system T protein) (EC 2.1.2.10)	FIG00138538	isu;Photorespiration_(oxidative_C2_cycle) isu;CBSS-87626.3.peg.3639 isu;Glycine_cleavage_system

fig|6666666.28482.peg.3986	Contig_44	19921	21027	+	Branched-chain amino acid aminotransferase (EC 2.6.1.42)	FIG00050603	isu;Branched-Chain_Amino_Acid_Biosynthesis isu;Pyruvate_Alanine_Serine_Interconversions isu;Isoleucine_degradation isu;Alanine_biosynthesis isu;Leucine_Degradation_and_HMG-CoA_Metabolism isu;Valine_degradation isu;Leucine_Biosynthesis
fig|6666666.28482.peg.3987	Contig_44	21836	21099	-	Cobalamin synthase	FIG00006032	icw(1);Cobalamin_synthesis icw(2);Coenzyme_B12_biosynthesis
fig|6666666.28482.peg.3988	Contig_44	22891	21836	-	Nicotinate-nucleotide--dimethylbenzimidazole phosphoribosyltransferase (EC 2.4.2.21)	FIG00000824	icw(2);Cobalamin_synthesis icw(3);Coenzyme_B12_biosynthesis
fig|6666666.28482.peg.3989	Contig_44	23427	22891	-	Adenosylcobinamide-phosphate guanylyltransferase (EC 2.7.7.62)	FIG00000764	isu;Cobalamin_synthesis icw(1);Coenzyme_B12_biosynthesis
fig|6666666.28482.peg.3990	Contig_44	24114	23455	-	CblZ, a non-orthologous displasment for Alpha-ribazole-5'-phosphate phosphatase	FIG00006903	isu;Coenzyme_B12_biosynthesis

fig|6666666.28482.peg.3991	Contig_44	24241	25392	+	Glycerate kinase (EC 2.7.1.31)	FIG00000456	idu(1);Glycerate_metabolism idu(1);Serine-glyoxylate_cycle idu(1);Allantoin_Utilization idu(1);Glycerolipid_and_Glycerophospholipid_Metabolism_in_Bacteria idu(1);Photorespiration_(oxidative_C2_cycle) idu(1);D-galactarate,_D-glucarate_and_D-glycerate_catabolism idu(1);D-galactarate,_D-glucarate_and_D-glycerate_catabolism_-_gjo
fig|6666666.28482.peg.3992	Contig_44	25475	25831	+	probable iron binding protein from the HesB_IscA_SufA family	FIG00000502	isu;Iron-sulfur_cluster_assembly
fig|6666666.28482.peg.3993	Contig_44	26315	25845	-	Possible membrane protein	FIG01121868	ff
fig|6666666.28482.peg.3994	Contig_44	26651	27625	+	Adenosine kinase (EC 2.7.1.20)	FIG00020335	isu;Purine_conversions
fig|6666666.28482.peg.3995	Contig_44	29696	27726	-	Asparagine synthetase [glutamine-hydrolyzing] (EC 6.3.5.4)	FIG00035175	idu(1);Glutamine,_Glutamate,_Aspartate_and_Asparagine_Biosynthesis idu(1);Glutamate_and_Aspartate_uptake_in_Bacteria idu(1);Cyanophycin_Metabolism
fig|6666666.28482.peg.3996	Contig_44	29826	30899	+	Cytochrome c oxidase polypeptide II (EC 1.9.3.1)	FIG00041887	isu;Terminal_cytochrome_C_oxidases isu;CBSS-316057.3.peg.563
fig|6666666.28482.peg.3997	Contig_44	30908	31327	+	Probable cytochrome c oxidase polypeptide 4 (EC 1.9.3.1)	FIG00484860	ff

fig|6666666.28482.peg.3998	Contig_44	31510	32469	+	Putative membrane protein MmpS3	FIG01258930	ff
fig|6666666.28482.peg.3999	Contig_45	1631	192	-	hypothetical protein	FIG00638284	ff
fig|6666666.28482.peg.4000	Contig_45	2730	3104	+	FIG00820548: hypothetical protein	FIG00820545	ff
fig|6666666.28482.peg.4001	Contig_45	3376	4749	+	serine protease	FIG01109635	ff
fig|6666666.28482.peg.4002	Contig_45	4757	5158	+	Twin-arginine translocation protein TatB	FIG00000670	isu;Twin-arginine_translocation_system
fig|6666666.28482.peg.4003	Contig_45	6375	5239	-	Mrp protein homolog	FIG00448421	ff
fig|6666666.28482.peg.4004	Contig_45	7477	6407	-	POSSIBLE MEMBRANE PROTEIN	FIG00692835	ff
fig|6666666.28482.peg.4005	Contig_45	7487	7774	+	hypothetical protein		
fig|6666666.28482.peg.4006	Contig_45	8300	7758	-	PROBABLE MEMBRANE PROTEIN	FIG00816212	ff
fig|6666666.28482.peg.4007	Contig_45	9610	8297	-	Mg/Co/Ni transporter MgtE / CBS domain	FIG00437800	idu(1);Magnesium_transport
fig|6666666.28482.peg.4008	Contig_45	10221	9676	-	Basic proline-rich protein	FIG00822000	ff
fig|6666666.28482.peg.4009	Contig_45	10288	10815	+	FIG00945083: hypothetical protein	FIG00945082	ff
fig|6666666.28482.peg.4010	Contig_45	10815	12194	+	Maltose/maltodextrin ABC transporter, substrate binding periplasmic protein MalE	FIG00002265	icw(1);Maltose_and_Maltodextrin_Utilization isu;Alpha-Amylase_locus_in_Streptocococcus isu;Bacterial_Chemotaxis
fig|6666666.28482.peg.4011	Contig_45	12191	13063	+	Maltose/maltodextrin ABC transporter, permease protein MalF	FIG00019868	isu;Maltose_and_Maltodextrin_Utilization
fig|6666666.28482.peg.4012	Contig_45	13092	13886	+	Inositol transport system permease protein	FIG00008562	ff

fig|6666666.28482.peg.4013	Contig_45	13891	15063	+	Glycerol-3-phosphate ABC transporter, ATP-binding protein UgpC (TC 3.A.1.1.3)	FIG00027233	idu(1);Glycerol_and_Glycerol-3-phosphate_Uptake_and_Utilization
fig|6666666.28482.peg.4014	Contig_45	15060	15644	+	FIG00820926: hypothetical protein	FIG00820925	ff
fig|6666666.28482.peg.4015	Contig_45	16849	15746	-	Magnesium and cobalt transport protein CorA	FIG00004180	isu;Magnesium_transport isu;Campylobacter_Iron_Metabolism
fig|6666666.28482.peg.4016	Contig_45	17018	18007	+	Malate dehydrogenase (EC 1.1.1.37)	FIG00000455	isu;Serine-glyoxylate_cycle isu;TCA_Cycle isu;Glyoxylate_bypass
fig|6666666.28482.peg.4017	Contig_45	18033	19229	+	NADP-dependent malic enzyme (EC 1.1.1.40)	FIG00000777	isu;Pyruvate_metabolism_I:_anaplerotic_reactions,_PEP
fig|6666666.28482.peg.4018	Contig_45	19226	20077	+	Substrate-binding region of ABC-type glycine betaine transport system	FIG00862992	ff
fig|6666666.28482.peg.4019	Contig_45	20935	20108	-	Short-chain type dehydrogenase/reductase	FIG01343100	ff
fig|6666666.28482.peg.4020	Contig_46	966	484	-	integral membrane protein		
fig|6666666.28482.peg.4021	Contig_46	1535	966	-	FIG00827194: hypothetical protein	FIG00827190	ff
fig|6666666.28482.peg.4022	Contig_46	1585	2973	+	DNA polymerase IV (EC 2.7.7.7)	FIG00023943	idu(1);DNA_repair,_bacterial
fig|6666666.28482.peg.4023	Contig_46	3879	2938	-	L-asparaginase (EC 3.5.1.1)	FIG00000376	isu;Glutamine,_Glutamate,_Aspartate_and_Asparagine_Biosynthesis

fig|6666666.28482.peg.4024	Contig_46	3881	4513	+	Lipoprotein signal peptidase (EC 3.4.23.36)	FIG00000081	isu;Signal_peptidase isu;Sex_pheromones_in_Enterococcus_faecalis_and_other_Firmicutes isu;CBSS-36873.1.peg.4752 isu;Lipoprotein_Biosynthesis
fig|6666666.28482.peg.4025	Contig_46	4513	5439	+	Ribosomal large subunit pseudouridine synthase D (EC 4.2.1.70)	FIG00000124	isu;RNA_pseudouridine_syntheses
fig|6666666.28482.peg.4026	Contig_46	6278	5520	-	FIG00825360: hypothetical protein	FIG00825359	ff
fig|6666666.28482.peg.4027	Contig_46	6728	6330	-	Cyanoglobin; Hemoglobin-like protein HbN	FIG00002685	isu;Bacterial_hemoglobins
fig|6666666.28482.peg.4028	Contig_46	6957	7976	+	"Oxidoreductase, short-chain dehydrogenase/reductase family"	FIG01320732	ff
fig|6666666.28482.peg.4029	Contig_46	7945	8784	+	Ketoacyl reductase	FIG01339396	ff
fig|6666666.28482.peg.4030	Contig_46	12188	9252	-	Putative membrane protein	FIG01056920	ff
fig|6666666.28482.peg.4031	Contig_46	12631	12185	-	membrane protein, MmpS family	FIG01309473	ff
fig|6666666.28482.peg.4032	Contig_46	13358	12750	-	Transcriptional regulator, TetR family	FIG00554417	ff
fig|6666666.28482.peg.4033	Contig_46	13459	17004	+	DNA polymerase III alpha subunit (EC 2.7.7.7)	FIG00041038	idu(1);DNA-replication idu(1);CBSS-350688.3.peg.1509
fig|6666666.28482.peg.4034	Contig_46	17302	20364	+	Adenylate cyclase (EC 4.6.1.1)	FIG00001294	idu(25);cAMP_signaling_in_bacteria
fig|6666666.28482.peg.4035	Contig_46	21341	20394	-	putative hydrolase		

fig|6666666.28482.peg.4036	Contig_46	21784	21338	-	FIG00827074: hypothetical protein	FIG00827072	ff
fig|6666666.28482.peg.4037	Contig_46	21844	22245	+	Transcriptional regulator, MerR family	FIG00003460	idu(1);Cobalt-zinc-cadmium_resistance
fig|6666666.28482.peg.4038	Contig_46	22262	22741	+	Glyoxalase/bleomycin resistance protein/dioxygenase	FIG00830431	ff
fig|6666666.28482.peg.4039	Contig_46	22815	24641	+	Long-chain-fatty-acid--CoA ligase (EC 6.2.1.3)	FIG00018699	idu(72);n-Phenylalkanoic_acid_degradation idu(72);Fatty_acid_metabolism_cluster idu(72);Biotin_synthesis_cluster idu(72);Biotin_biosynthesis
fig|6666666.28482.peg.4040	Contig_46	24686	26545	+	Glycerol-3-phosphate acyltransferase (EC 2.3.1.15)	FIG00001327	idu(1);Glycerolipid_and_Glycerophospholipid_Metabolism_in_Bacteria
fig|6666666.28482.peg.4041	Contig_46	28036	26597	-	2-polyprenyl-6-methoxyphenol hydroxylase and related FAD-dependent oxidoreductases	FIG00453279	ff
fig|6666666.28482.peg.4042	Contig_46	28440	28033	-	FIG235764: hypothetical protein	FIG01578613	ff
fig|6666666.28482.peg.4043	Contig_46	28534	29091	+	Transcriptional regulator, TetR family	FIG00628138	ff
fig|6666666.28482.peg.4044	Contig_46	29119	29556	+	AclJ	FIG00862767	ff
fig|6666666.28482.peg.4045	Contig_46	29664	30860	+	Threonine dehydratase (EC 4.3.1.19)	FIG00022899	isu;Threonine_degradation isu;Branched-Chain_Amino_Acid_Biosynthesis
fig|6666666.28482.peg.4046	Contig_46	30870	31748	+	FIG00828971: hypothetical protein	FIG00828970	ff

fig|6666666.28482.peg.4047	Contig_46	32390	31740	-	Pyridoxamine 5'-phosphate oxidase-related, FMN-binding	FIG01045148	ff
fig|6666666.28482.peg.4048	Contig_46	32431	33744	+	Predicted transcriptional regulator of pyridoxine metabolism	FIG00008736	isu;Pyridoxin_(Vitamin_B6)_Biosynthesis
fig|6666666.28482.peg.4049	Contig_46	34327	33722	-	G:T/U mismatch-specific uracil/thymine DNA-glycosylase	FIG00001253	isu;DNA_repair,_bacterial isu;Uracil-DNA_glycosylase
fig|6666666.28482.peg.4050	Contig_46	34917	34384	-	hypothetical protein	FIG00638284	ff
fig|6666666.28482.peg.4051	Contig_46	35489	34914	-	putative secreted protein		
fig|6666666.28482.peg.4052	Contig_46	35732	35577	-	hypothetical protein	FIG00638284	ff
fig|6666666.28482.peg.4053	Contig_46	35836	36486	+	putative two-component system response regulator		
fig|6666666.28482.peg.4054	Contig_46	37385	36483	-	O-Methyltransferase involved in polyketide biosynthesis	FIG01313020	ff
fig|6666666.28482.peg.4055	Contig_46	37473	37682	+	FIG00821924: hypothetical protein	FIG00821923	ff
fig|6666666.28482.peg.4056	Contig_46	37682	38086	+	FIG00820947: hypothetical protein	FIG00820946	ff
fig|6666666.28482.peg.4057	Contig_46	39826	38093	-	Malto-oligosyltrehalose trehalohydrolase (EC 3.2.1.141)	FIG00001338	icw(2);Trehalose_Biosynthesis

fig|6666666.28482.peg.4058	Contig_46	42119	39819	-	Malto-oligosyltrehalose synthase (EC 5.4.99.15)	FIG00001606	isu;Trehalose_Biosynthesis isu;Maltose_and_Maltodextrin_Utilization
fig|6666666.28482.peg.4059	Contig_46	44336	42123	-	Glycogen debranching enzyme (EC 3.2.1.-)	FIG00109688	icw(1);Trehalose_Biosynthesis idu(3);Glycogen_metabolism
fig|6666666.28482.peg.4060	Contig_46	46532	44370	-	O-antigen acetylase	FIG01370327	ff
fig|6666666.28482.peg.4061	Contig_46	47392	46646	-	NLP/P60 family protein	FIG00830690	ff
fig|6666666.28482.peg.4062	Contig_46	48246	47785	-	FIG00821756: hypothetical protein	FIG00821754	ff
fig|6666666.28482.peg.4063	Contig_46	48304	49608	+	Adenosylmethionine-8-amino-7-oxononanoate aminotransferase (EC 2.6.1.62)	FIG00012547	isu;Biotin_biosynthesis_Experimental isu;Biotin_synthesis_cluster isu;Biotin_biosynthesis
fig|6666666.28482.peg.4064	Contig_46	49707	50177	+	hypothetical protein		
fig|6666666.28482.peg.4065	Contig_46	50535	51071	+	PE-PGRS family protein		idu(16);PPE_gene_cluster_in_Mycobacteria
fig|6666666.28482.peg.4066	Contig_46	54011	51096	-	Putative membrane protein	FIG01056920	ff
fig|6666666.28482.peg.4067	Contig_46	54012	54149	+	hypothetical protein		
fig|6666666.28482.peg.4068	Contig_46	57023	54156	-	Putative membrane protein	FIG01056920	ff
fig|6666666.28482.peg.4069	Contig_46	57400	57020	-	membrane protein, MmpS family	FIG01309473	ff
fig|6666666.28482.peg.4070	Contig_46	59127	57715	-	hypothetical protein		
fig|6666666.28482.peg.4071	Contig_46	60717	59326	-	FIG01292463: hypothetical protein	FIG01292461	ff
fig|6666666.28482.peg.4072	Contig_46	61141	62409	+	hypothetical glycosyl transferase(EC:2.-)		

fig|6666666.28482.peg.4073	Contig_46	63910	62444	-	Glucose-methanol-choline (GMC) oxidoreductase:NAD binding site	FIG00001629	idu(3);Respiratory_dehydrogenases_1
fig|6666666.28482.peg.4074	Contig_47	3369	202	-	Adenylate cyclase (EC 4.6.1.1)	FIG00001294	idu(25);cAMP_signaling_in_bacteria
fig|6666666.28482.peg.4075	Contig_47	3636	3770	+	hypothetical protein		
fig|6666666.28482.peg.4076	Contig_47	4251	3793	-	Universal stress protein UspA and related nucleotide-binding proteins	FIG01023736	ff
fig|6666666.28482.peg.4077	Contig_47	5395	4358	-	ATP-dependent DNA ligase (EC 6.5.1.1)	FIG00052980	idu(2);DNA_replication,_archaeal icw(1);DNA_Repair_Base_Excision icw(1);DNA_ligases
fig|6666666.28482.peg.4078	Contig_47	5422	6477	+	ATP-dependent DNA ligase (EC 6.5.1.1) LigC	FIG00111848	isu;DNA_Repair_Base_Excision isu;DNA_ligases isu;Nonhomologous_End-Joining_in_Bacteria
fig|6666666.28482.peg.4079	Contig_47	6904	6491	-	conserved hypothetical secreted protein		
fig|6666666.28482.peg.4080	Contig_47	7400	7071	-	hypothetical protein		
fig|6666666.28482.peg.4081	Contig_47	7519	7971	+	FIG00830839: hypothetical protein	FIG00830838	ff
fig|6666666.28482.peg.4082	Contig_47	8012	8380	+	FIG00824363: hypothetical protein	FIG00824362	ff
fig|6666666.28482.peg.4083	Contig_47	10051	8822	-	putative cytochrome P450 hydroxylase	FIG00032437	idu(49);Nitric_oxide_synthase
fig|6666666.28482.peg.4084	Contig_47	10988	10083	-	haloalkane dehalogenase(EC:3.8.1.5)	FIG01630420	ff
fig|6666666.28482.peg.4085	Contig_47	11475	10990	-	MutT-like protein	FIG01324438	ff

fig|6666666.28482.peg.4086	Contig_47	12837	11482	-	Wax ester synthase/acyl-CoA:diacylglycerol acyltransferase	FIG01309728	ff
fig|6666666.28482.peg.4087	Contig_47	12981	13772	+	Acyltransferase family protein	FIG00832346	ff
fig|6666666.28482.peg.4088	Contig_47	14179	13769	-	hypothetical protein		
fig|6666666.28482.peg.4089	Contig_47	16706	15234	-	monooxygenase, flavin-binding family	FIG01386881	ff
fig|6666666.28482.peg.4090	Contig_47	16805	16954	+	hypothetical protein		
fig|6666666.28482.peg.4091	Contig_47	18193	17336	-	FIG00822389: hypothetical protein	FIG00822386	ff
fig|6666666.28482.peg.4092	Contig_47	18287	19357	+	FIG00827644: hypothetical protein	FIG00827643	ff
fig|6666666.28482.peg.4093	Contig_47	19354	19848	+	Possible lactoylglutathione lyase	FIG01331051	ff
fig|6666666.28482.peg.4094	Contig_47	19845	20618	+	FIG00822257: hypothetical protein	FIG00822256	ff
fig|6666666.28482.peg.4095	Contig_47	20615	21220	+	Transcriptional regulator, TetR family	FIG00554417	ff
fig|6666666.28482.peg.4096	Contig_47	21217	22326	+	hypothetical protein		
fig|6666666.28482.peg.4097	Contig_47	23858	22329	-	FIG00825594: hypothetical protein	FIG00825593	ff
fig|6666666.28482.peg.4098	Contig_48	373	1410	+	FIG00821239: hypothetical protein	FIG00821236	ff
fig|6666666.28482.peg.4099	Contig_48	1703	1407	-	FIG00995606: hypothetical protein	FIG00823054	ff
fig|6666666.28482.peg.4100	Contig_48	2025	1735	-	FIG00999563: hypothetical protein	FIG00822585	ff
fig|6666666.28482.peg.4101	Contig_48	2261	2061	-	FIG043778: hypothetical protein	FIG00043778	icw(5);CBSS-336982.3.peg.3874

fig|6666666.28482.peg.4102	Contig_48	2858	2280	-	FIG054221: Possible conserved alanine rich membrane protein		isu;CBSS-336982.3.peg.3874
fig|6666666.28482.peg.4103	Contig_48	3652	2855	-	FIG016317: Probable conserved transmembrane protein	FIG00016317	icw(1);CBSS-336982.3.peg.3874
fig|6666666.28482.peg.4104	Contig_48	4812	3649	-	Flp pilus assembly protein, ATPase CpaF	FIG00014856	icw(3);CBSS-336982.3.peg.3874
fig|6666666.28482.peg.4105	Contig_48	5900	4809	-	Septum site-determining protein MinD @ possible CpaE	FIG00029816	isu;Septum_site-determining_cluster_Min isu;Bacterial_Cell_Division icw(4);CBSS-336982.3.peg.3874 isu;Bacterial_Cytoskeleton
fig|6666666.28482.peg.4106	Contig_48	6341	7198	+	Phosphoserine phosphatase (EC 3.1.3.3)	FIG00000149	icw(2);CBSS-336982.3.peg.3874 idu(3);Serine_Biosynthesis
fig|6666666.28482.peg.4107	Contig_48	8386	7628	-	probable oxidoreductase	FIG01124638	ff
fig|6666666.28482.peg.4108	Contig_48	9999	8398	-	Dipeptide transport ATP-binding protein DppD (TC 3.A.1.5.2)	FIG00014907	icw(1);ABC_transporter_dipeptide_(TC_3.A.1.5.2)
fig|6666666.28482.peg.4109	Contig_48	10859	9996	-	Dipeptide transport system permease protein DppC (TC 3.A.1.5.2)	FIG00006691	icw(2);ABC_transporter_dipeptide_(TC_3.A.1.5.2)
fig|6666666.28482.peg.4110	Contig_48	11778	10852	-	Dipeptide transport system permease protein DppB (TC 3.A.1.5.2)	FIG01954097	isu;ABC_transporter_dipeptide_(TC_3.A.1.5.2)

fig|6666666.28482.peg.4111	Contig_48	13387	11780	-	Dipeptide-binding ABC transporter, periplasmic substrate-binding component (TC 3.A.1.5.2)	FIG00006128	icw(3);ABC_transporter_dipeptide_(TC_3.A.1.5.2) isu;Bacterial_Chemotaxis
fig|6666666.28482.peg.4112	Contig_48	13494	14183	+	hypothetical protein		
fig|6666666.28482.peg.4113	Contig_48	14726	14190	-	FIG00825127: hypothetical protein	FIG00825126	ff
fig|6666666.28482.peg.4114	Contig_48	14841	16790	+	Acetyl-coenzyme A synthetase (EC 6.2.1.1)	FIG00022631	idu(2);Pyruvate_metabolism_II:_acetyl-CoA,_acetogenesis_from_pyruvate idu(2);Ketoisovalerate_oxidoreductase
fig|6666666.28482.peg.4115	Contig_48	17533	16886	-	Protease	FIG01317673	ff
fig|6666666.28482.peg.4116	Contig_48	17805	17524	-	hypothetical protein		
fig|6666666.28482.peg.4117	Contig_48	17851	18381	+	FIG00820727: hypothetical protein	FIG01123008	ff
fig|6666666.28482.peg.4118	Contig_48	18382	19359	+	Epoxide hydrolase (EC 3.3.2.9)	FIG00009455	idu(3);CBSS-410289.13.peg.3174
fig|6666666.28482.peg.4119	Contig_48	20560	19352	-	putative serine protease	FIG00814692	ff
fig|6666666.28482.peg.4120	Contig_48	21333	20557	-	Hypothetical nudix hydrolase YeaB	FIG00136447	idu(1);Nudix_proteins_(nucleoside_triphosphate_hydrolases)
fig|6666666.28482.peg.4121	Contig_48	22001	21363	-	Possible membrane-anchored thioredoxin-like protein	FIG00544296	ff
fig|6666666.28482.peg.4122	Contig_48	22726	22019	-	Endonuclease III (EC 4.2.99.18)	FIG00000290	isu;DNA_Repair_Base_Excision isu;Control_of_cell_elongation_-_division_cycle_in_Bacilli
fig|6666666.28482.peg.4123	Contig_48	22861	23256	+	POSSIBLE MEMBRANE PROTEIN	FIG00692835	ff

fig|6666666.28482.peg.4124	Contig_48	23353	24027	+	cAMP-binding proteins - catabolite gene activator and regulatory subunit of cAMP-dependent protein kinases	FIG00003090	idu(1);cAMP_signaling_in_bacteria idu(1);CBSS-479431.5.peg.3955
fig|6666666.28482.peg.4125	Contig_48	24892	24098	-	FIG146518: Zn-dependent hydrolases, including glyoxylases	FIG00013561	icw(2);CBSS-479431.5.peg.3955
fig|6666666.28482.peg.4126	Contig_48	25353	24898	-	FIG137598: hypothetical protein	FIG00148911	icw(1);CBSS-479431.5.peg.3955
fig|6666666.28482.peg.4127	Contig_48	25517	25356	-	FIG011121: hypothetical protein	FIG00011121	icw(3);CBSS-479431.5.peg.3955
fig|6666666.28482.peg.4128	Contig_48	25595	26617	+	Arsenical pump-driving ATPase (EC 3.6.3.16)	FIG00003088	icw(1);Stress_related_cluster icw(1);Arsenic_resistance
fig|6666666.28482.peg.4129	Contig_48	26614	27768	+	Arsenical pump-driving ATPase (EC 3.6.3.16)	FIG00003088	icw(1);Stress_related_cluster icw(1);Arsenic_resistance
fig|6666666.28482.peg.4130	Contig_48	28410	28060	-	WhiB-type transcription regulator	FIG00019991	idu(2);WhiB_and_WhiB-type_regulatory_proteins_
fig|6666666.28482.peg.4131	Contig_48	28829	31198	+	Multimodular transpeptidase-transglycosylase (EC 2.4.1.129) (EC 3.4.-.-)	FIG00000011	idu(2);Peptidoglycan_Biosynthesis
fig|6666666.28482.peg.4132	Contig_48	31268	32236	+	putative secreted protein	FIG00905474	ff
fig|6666666.28482.peg.4133	Contig_48	32233	33345	+	Cysteine synthase B (EC 2.5.1.47)	FIG00470735	isu;Cysteine_Biosynthesis
fig|6666666.28482.rna.43	Contig_48	33399	33472	+	tRNA-Pro-CGG		isu;tRNAs
fig|6666666.28482.peg.4134	Contig_48	33827	34249	+	hypothetical protein		
fig|6666666.28482.peg.4135	Contig_48	34460	35524	+	hypothetical protein		

fig|6666666.28482.peg.4136	Contig_48	36154	35564	-	FIG00820655: hypothetical protein	FIG00820654	ff
fig|6666666.28482.peg.4137	Contig_48	36875	36231	-	hypothetical protein	FIG00638284	ff
fig|6666666.28482.peg.4138	Contig_48	38441	37092	-	Probable cytochrome P450 137 cyp137 (EC 1.14.-.-)	FIG01307566	ff
fig|6666666.28482.peg.4139	Contig_48	38792	38496	-	FIG00822896: hypothetical protein	FIG00822895	ff
fig|6666666.28482.peg.4140	Contig_48	39144	38947	-	hypothetical protein		
fig|6666666.28482.peg.4141	Contig_48	39529	39284	-	hypothetical protein		
fig|6666666.28482.peg.4142	Contig_48	39738	40439	+	FIG00825085: hypothetical protein	FIG00825084	ff
fig|6666666.28482.peg.4143	Contig_48	40913	40407	-	FIG00829970: hypothetical protein	FIG00829968	ff
fig|6666666.28482.peg.4144	Contig_48	40893	41579	+	FIG00820289: hypothetical protein	FIG00820286	ff
fig|6666666.28482.peg.4145	Contig_48	41580	42896	+	Peroxidase (EC 1.11.1.7)	FIG00067081	idu(1);Oxidative_stress idu(1);Protection_from_Reactive_Oxygen_Species idu(1);Auxin_degradation
fig|6666666.28482.peg.4146	Contig_48	42893	43897	+	FIG00829308: hypothetical protein	FIG00829307	ff
fig|6666666.28482.peg.4147	Contig_48	44227	43913	-	Anti-sigma F factor antagonist (spoIIAA-2); Anti-sigma B factor antagonist RsbV	FIG00722824	icw(4);SigmaB_stress_responce_regulation icw(5);Sigma-B_stress_response_cluster_1
fig|6666666.28482.peg.4148	Contig_48	44438	45331	+	RsbR, positive regulator of sigma-B	FIG00001285	icw(3);SigmaB_stress_responce_regulation icw(4);Sigma-B_stress_response_cluster_1
fig|6666666.28482.peg.4149	Contig_48	45354	45743	+	RsbS, negative regulator of sigma-B	FIG00002556	isu;SigmaB_stress_responce_regulation icw(1);Sigma-B_stress_response_cluster_1

fig|6666666.28482.peg.4150	Contig_48	45733	46143	+	Anti-sigma B factor RsbT		icw(1);SigmaB_stress_responce_regulation isu;Sigma-B_stress_response_cluster_1
fig|6666666.28482.peg.4151	Contig_48	46190	46762	+	Phosphoserine phosphatase RsbX (EC 3.1.3.3)	FIG01305469	icw(2);Sigma-B_stress_response_cluster_1
fig|6666666.28482.peg.4152	Contig_48	46759	48975	+	Serine phosphatase RsbU, regulator of sigma subunit	FIG00017431	icw(2);SigmaB_stress_responce_regulation icw(3);Sigma-B_stress_response_cluster_1
fig|6666666.28482.peg.4153	Contig_48	49019	50038	+	FIG00825121: hypothetical protein	FIG00825120	ff
fig|6666666.28482.peg.4154	Contig_48	50196	50035	-	hypothetical protein		
fig|6666666.28482.peg.4155	Contig_48	50876	50232	-	putative methyltransferase	FIG01125506	ff
fig|6666666.28482.peg.4156	Contig_48	51575	51021	-	ThiJ/PfpI family protein	FIG00015923	idu(8);CBSS-176280.1.peg.1561
fig|6666666.28482.peg.4157	Contig_48	52758	51589	-	Threonine dehydrogenase and related Zn-dependent dehydrogenases	FIG00053044	idu(2);Threonine_degradation
fig|6666666.28482.peg.4158	Contig_48	53699	52755	-	hypothetical protein	FIG00638284	ff
fig|6666666.28482.peg.4159	Contig_48	54864	53878	-	F420-dependent glucose-6-phosphate dehydrogenase	FIG01001620	ff
fig|6666666.28482.peg.4160	Contig_48	56076	54991	-	Formate dehydrogenase O putative subunit	FIG00069510	isu;Formate_hydrogenase
fig|6666666.28482.peg.4161	Contig_48	57035	56073	-	Formate dehydrogenase O beta subunit (EC 1.2.1.2)	FIG00001405	icw(1);Formate_hydrogenase

fig|6666666.28482.peg.4163	Contig_48	60309	57010	-	Formate dehydrogenase O alpha subunit (EC 1.2.1.2) @ selenocysteine-containing		icw(4);Formate_hydrogenase
fig|6666666.28482.peg.4162	Contig_48	59676	57010	-	Formate dehydrogenase O alpha subunit (EC 1.2.1.2) @ selenocysteine-containing	FIG00002195	icw(1);Selenocysteine_metabolism icw(4);Formate_hydrogenase icw(1);Hypothetical_protein_HNE_2485
fig|6666666.28482.peg.4164	Contig_48	60327	59746	-	Formate dehydrogenase O alpha subunit (EC 1.2.1.2) @ selenocysteine-containing	FIG00002195	icw(1);Selenocysteine_metabolism icw(4);Formate_hydrogenase icw(1);Hypothetical_protein_HNE_2485
fig|6666666.28482.rna.44	Contig_48	61388	61478	+	tRNA-SeC(p)-TCA		
fig|6666666.28482.peg.4166	Contig_49	44	1228	+	Glutaryl-CoA dehydrogenase (EC 1.3.99.7)	FIG01332484	ff
fig|6666666.28482.peg.4167	Contig_49	1242	1856	+	Possible membrane protein	FIG01121868	ff
fig|6666666.28482.peg.4168	Contig_49	2401	3792	+	NADH dehydrogenase (EC 1.6.99.3)	FIG00008708	idu(6);Respiratory_dehydrogenases_1 idu(6);PPE_gene_cluster_in_Mycobacteria idu(6);Riboflavin_synthesis_cluster
fig|6666666.28482.peg.4169	Contig_49	5009	3789	-	O-acetylhomoserine sulfhydrylase (EC 2.5.1.49)	FIG00963163	ff

fig|6666666.28482.peg.4170	Contig_49	5422	5006	-	Rhodanese-related sulfurtransferase	FIG01304277	ff
fig|6666666.28482.peg.4171	Contig_49	6639	5419	-	Phosphoribosylglycinamide formyltransferase 2 (EC 2.1.2.-)	FIG00000909	isu;De_Novo_Purine_Biosynthesis
fig|6666666.28482.peg.4172	Contig_49	7375	6737	-	FIG00822066: hypothetical protein	FIG00822064	ff
fig|6666666.28482.peg.4173	Contig_49	8670	7372	-	Adenylosuccinate synthetase (EC 6.3.4.4)	FIG00000107	isu;Purine_conversions
fig|6666666.28482.peg.4174	Contig_49	8674	8826	+	hypothetical protein		
fig|6666666.28482.peg.4175	Contig_49	8787	9389	+	FIG00820475: hypothetical protein	FIG00820474	ff
fig|6666666.28482.peg.4176	Contig_49	10309	9386	-	hypothetical protein	FIG00638284	ff
fig|6666666.28482.peg.4177	Contig_49	10569	10949	+	hypothetical protein		
fig|6666666.28482.peg.4178	Contig_49	10960	11736	+	Possible membrane protein	FIG01121868	ff
fig|6666666.28482.peg.4179	Contig_49	11777	12676	+	Cobalt-zinc-cadmium resistance protein CzcD	FIG00000503	idu(1);CBSS-36873.1.peg.4752 idu(1);Cobalt-zinc-cadmium_resistance
fig|6666666.28482.peg.4180	Contig_49	13086	12673	-	FIG00820939: hypothetical protein	FIG00820938	ff
fig|6666666.28482.peg.4181	Contig_49	13134	13910	+	PROBABLE CONSERVED MEMBRANE PROTEIN	FIG00662487	ff
fig|6666666.28482.peg.4182	Contig_49	15020	13986	-	Fructose-bisphosphate aldolase class II (EC 4.1.2.13)	FIG00000370	ff
fig|6666666.28482.peg.4183	Contig_49	15079	15786	+	DedA protein	FIG00002761	idu(1);Uptake_of_selenate_and_selenite idu(1);DedA_family_of_inner_membrane_proteins

fig|6666666.28482.peg.4184	Contig_49	17697	15745	-	Acetoacetyl-CoA synthetase (EC 6.2.1.16)	FIG00139370	idu(1);Polyhydroxybutyrate_metabolism idu(1);HMG_CoA_Synthesis idu(1);Leucine_Degradation_and_HMG-CoA_Metabolism
fig|6666666.28482.peg.4185	Contig_49	18824	17694	-	fructose-bisphosphate aldolase family protein	FIG00861911	ff
fig|6666666.28482.peg.4186	Contig_49	18871	19344	+	Isoquinoline 1-oxidoreductase alpha subunit (EC 1.3.99.16)	FIG00000941	icw(1);N-heterocyclic_aromatic_compound_degradation icw(1);CBSS-314267.3.peg.390
fig|6666666.28482.peg.4187	Contig_49	19337	21454	+	Isoquinoline 1-oxidoreductase beta subunit (EC 1.3.99.16)	FIG00000940	isu;N-heterocyclic_aromatic_compound_degradation isu;CBSS-314267.3.peg.390
fig|6666666.28482.peg.4188	Contig_49	22097	22699	+	PROBABLE SIGNAL PEPTIDE PROTEIN	FIG01373654	ff
fig|6666666.28482.peg.4189	Contig_49	22961	23614	+	Oxalate decarboxylase oxdD (EC 4.1.1.2)		
fig|6666666.28482.peg.4190	Contig_49	23631	23780	+	hypothetical protein		
fig|6666666.28482.peg.4191	Contig_49	23815	24675	+	hypothetical protein		
fig|6666666.28482.peg.4192	Contig_49	24677	25156	+	hypothetical protein		
fig|6666666.28482.peg.4193	Contig_49	25958	25215	-	Hydrolase, alpha/beta fold family		
fig|6666666.28482.peg.4194	Contig_49	26882	26124	-	hypothetical protein		
fig|6666666.28482.peg.4195	Contig_5	671	18	-	Transcriptional regulator, PadR family	FIG00002474	idu(4);CBSS-1352.1.peg.856
fig|6666666.28482.peg.4196	Contig_5	1090	668	-	FIG00823396: hypothetical protein	FIG00823395	ff
fig|6666666.28482.peg.4197	Contig_5	2262	1087	-	FIG00827839: hypothetical protein	FIG00827830	ff

fig|6666666.28482.peg.4198	Contig_5	2581	2252	-	FIG00823970: hypothetical protein	FIG00823964	ff
fig|6666666.28482.peg.4199	Contig_5	4916	2583	-	FIG00821320: hypothetical protein	FIG00821319	ff
fig|6666666.28482.peg.4200	Contig_5	6272	4917	-	FIG00830141: hypothetical protein	FIG00830140	ff
fig|6666666.28482.peg.4201	Contig_5	6542	7633	+	FIG00820829: hypothetical protein	FIG00820828	ff
fig|6666666.28482.peg.4202	Contig_5	7630	8802	+	FIG00821633: hypothetical protein	FIG00821630	ff
fig|6666666.28482.peg.4203	Contig_5	9295	8888	-	FIG00824333: hypothetical protein	FIG00824329	ff
fig|6666666.28482.peg.4204	Contig_5	10506	9298	-	FIG00824408: hypothetical protein	FIG00824406	ff
fig|6666666.28482.peg.4205	Contig_5	12771	10516	-	PROBABLE ACYL-CoA DEHYDROGENASE FADE6 (EC 1.3.99.-)	FIG00824752	ff
fig|6666666.28482.peg.4206	Contig_5	15354	13432	-	Pyruvate kinase family protein	FIG01460628	ff
fig|6666666.28482.peg.4207	Contig_5	15505	16584	+	FIG00827021: hypothetical protein	FIG00827019	ff
fig|6666666.28482.peg.4208	Contig_5	17487	16594	-	Fructose-bisphosphate aldolase class I (EC 4.1.2.13)	FIG01262685	idu(1);Unknown_carbohydrate_utilization_(_cluster_Yeg_)
fig|6666666.28482.peg.4209	Contig_5	18559	17561	-	Glutamine-transport ATP-binding protein ABC transporter	FIG00825489	ff
fig|6666666.28482.peg.4210	Contig_5	19612	18563	-	Glutamine-transport transmembrane protein ABC transporter	FIG01409078	ff

fig|6666666.28482.peg.4211	Contig_5	19867	19631	-	Isochorismatase (EC 3.3.2.1)	FIG00009017	idu(3);Chorismate:_Intermediate_for_synthesis_of_Tryptophan,_PAPA_antibiotics,_PABA,_3-hydroxyanthranilate_and_more.
fig|6666666.28482.peg.4212	Contig_5	20466	19873	-	Cytochrome B561	FIG01221566	ff
fig|6666666.28482.peg.4213	Contig_5	21593	20463	-	Catalase (EC 1.11.1.6)	FIG00000313	idu(3);Oxidative_stress idu(3);Photorespiration_(oxidative_C2_cycle) idu(3);Protection_from_Reactive_Oxygen_Species
fig|6666666.28482.peg.4214	Contig_5	22088	21900	-	hypothetical protein		
fig|6666666.28482.peg.4215	Contig_5	22087	22383	+	Anti-sigma F factor antagonist (spoIIAA-2); Anti-sigma B factor antagonist RsbV	FIG00722824	idu(5);SigmaB_stress_responce_regulation idu(5);Sigma-B_stress_response_cluster_1
fig|6666666.28482.peg.4216	Contig_5	23473	22388	-	aminoglycoside phosphotransferase	FIG00824137	ff
fig|6666666.28482.peg.4217	Contig_5	24609	23470	-	FIG00823690: hypothetical protein	FIG00823687	ff
fig|6666666.28482.peg.4218	Contig_5	24682	24858	+	hypothetical protein		
fig|6666666.28482.peg.4219	Contig_5	24851	25351	+	Transcriptional regulator, TetR family	FIG00554417	ff
fig|6666666.28482.peg.4220	Contig_5	25463	26599	+	FIG00822253: hypothetical protein	FIG00822252	ff
fig|6666666.28482.peg.4221	Contig_5	26697	28457	+	D-aminoacylase (EC 3.5.1.81)	FIG01332753	ff
fig|6666666.28482.peg.4222	Contig_5	28480	29826	+	Phenylpropionate dioxygenase and related ring-hydroxylating dioxygenases, large terminal subunit	FIG01371907	ff
fig|6666666.28482.peg.4223	Contig_5	30036	30248	+	hypothetical protein		

fig|6666666.28482.peg.4224	Contig_5	30998	30333	-	Response regulator containing a CheY-like receiver domain and an HTH DNA-binding domain	FIG01327243	ff
fig|6666666.28482.peg.4225	Contig_5	32203	30995	-	FIG00826666: hypothetical protein	FIG00826664	ff
fig|6666666.28482.peg.4226	Contig_5	32373	32714	+	hypothetical protein		
fig|6666666.28482.peg.4227	Contig_5	32707	35559	+	FIG00830249: hypothetical protein	FIG00830246	ff
fig|6666666.28482.peg.4228	Contig_5	36009	35563	-	FIG00819980: hypothetical protein	FIG00819977	ff
fig|6666666.28482.peg.4229	Contig_5	36501	36103	-	FIG00830071: hypothetical protein	FIG00830070	ff
fig|6666666.28482.peg.4230	Contig_5	37837	36506	-	FIG00823316: hypothetical protein	FIG00823312	ff
fig|6666666.28482.peg.4231	Contig_5	39623	38028	-	putative transmembrane transport protein	FIG01126523	ff
fig|6666666.28482.peg.4232	Contig_5	39928	40545	+	transcriptional regulatory protein (probably TetR/AcrR-family)	FIG00828629	ff
fig|6666666.28482.peg.4233	Contig_5	40576	41787	+	FadE25_2	FIG00826652	ff
fig|6666666.28482.peg.4234	Contig_5	42919	41798	-	putative LuxAB-like Oxygenase	FIG00477912	ff
fig|6666666.28482.peg.4235	Contig_5	44319	42925	-	FIG01124584: hypothetical protein	FIG00863066	ff
fig|6666666.28482.peg.4236	Contig_5	45555	44347	-	FMNH2-utilizing oxygenase	FIG01129775	ff

fig|6666666.28482.peg.4237	Contig_5	47083	46004	-	Alkanesulfonate monooxygenase (EC 1.14.14.5)	FIG00113730	icw(2);Alkanesulfonates_Utilization icw(3);Alkanesulfonate_assimilation
fig|6666666.28482.peg.4238	Contig_5	47201	48418	+	FIG00823043: hypothetical protein	FIG00823042	ff
fig|6666666.28482.peg.4239	Contig_5	48421	49368	+	FIG00822545: hypothetical protein	FIG00822544	ff
fig|6666666.28482.peg.4240	Contig_5	50096	49365	-	Alkanesulfonates transport system permease protein	FIG00138354	icw(1);Alkanesulfonates_Utilization icw(2);Alkanesulfonate_assimilation
fig|6666666.28482.peg.4241	Contig_5	51079	50114	-	Alkanesulfonates-binding protein	FIG00001221	isu;Alkanesulfonates_Utilization icw(1);Alkanesulfonate_assimilation
fig|6666666.28482.peg.4242	Contig_5	51888	51076	-	ABC-type nitrate/sulfonate/bicarbonate transport system, ATPase component	FIG00024999	isu;Alkanesulfonate_assimilation
fig|6666666.28482.peg.4243	Contig_5	52249	51968	-	FIG00825296: hypothetical protein	FIG00825295	ff
fig|6666666.28482.peg.4244	Contig_5	52417	53634	+	FIG00823721: hypothetical protein	FIG00823720	ff
fig|6666666.28482.peg.4245	Contig_5	54281	53652	-	hypothetical protein		
fig|6666666.28482.peg.4246	Contig_5	55478	54441	-	N5,N10-methylenetetrahydromethanopterin reductase-related protein, MSMEG1563 family	FIG00823643	ff
fig|6666666.28482.peg.4247	Contig_5	55921	55490	-	hypothetical protein		

fig|6666666.28482.peg.4248	Contig_5	56041	56607	+	Transcriptional regulator, PadR family	FIG00002474	idu(4);CBSS-1352.1.peg.856
fig|6666666.28482.peg.4249	Contig_5	59552	56604	-	Lysine decarboxylase (EC 4.1.1.18)	FIG01285094	idu(1);Lysine_degradation
fig|6666666.28482.peg.4250	Contig_5	59859	60074	+	FIG00829281: hypothetical protein	FIG00829279	ff
fig|6666666.28482.peg.4251	Contig_5	60129	60380	+	hypothetical protein		
fig|6666666.28482.peg.4252	Contig_5	60588	61658	+	luciferase family protein	FIG01400673	ff
fig|6666666.28482.peg.4253	Contig_5	61862	61674	-	hypothetical protein		
fig|6666666.28482.peg.4254	Contig_5	62662	61943	-	Hypothetical nudix hydrolase YeaB	FIG00136447	idu(1);Nudix_proteins_(nucleoside_triphosphate_hydrolases)
fig|6666666.28482.peg.4255	Contig_5	62754	63329	+	FIG00825627: hypothetical protein	FIG00825625	ff
fig|6666666.28482.peg.4256	Contig_5	63404	64309	+	FIG00822955: hypothetical protein	FIG00822953	ff
fig|6666666.28482.peg.4257	Contig_5	64746	64312	-	hypothetical protein		
fig|6666666.28482.peg.4258	Contig_5	65861	64977	-	Cyclopropane-fatty-acyl-phospholipid synthase (EC 2.1.1.79)	FIG00001555	ff
fig|6666666.28482.peg.4259	Contig_5	66083	67441	+	FIG00824750: hypothetical protein	FIG00824747	ff
fig|6666666.28482.peg.4260	Contig_5	67793	67443	-	Serine phosphatase RsbU, regulator of sigma subunit		idu(4);SigmaB_stress_responce_regulation idu(4);Sigma-B_stress_response_cluster_1
fig|6666666.28482.peg.4261	Contig_5	68393	67971	-	FIG00821490: hypothetical protein	FIG01359303	ff
fig|6666666.28482.peg.4262	Contig_5	69676	68462	-	Ferredoxin reductase	FIG00011423	idu(1);Anaerobic_respiratory_reductases
fig|6666666.28482.peg.4263	Contig_5	69869	69681	-	ferredoxin (3Fe-4S)		
fig|6666666.28482.peg.4264	Contig_5	70831	69866	-	FF domain protein	FIG01341765	ff
fig|6666666.28482.peg.4265	Contig_5	71096	71953	+	FIG00826463: hypothetical protein	FIG00826461	ff

fig|6666666.28482.peg.4266	Contig_5	72676	71978	-	COG1272: Predicted membrane protein hemolysin III homolog	FIG01323667	ff
fig|6666666.28482.peg.4267	Contig_5	73879	72809	-	hypothetical protein	FIG00638284	ff
fig|6666666.28482.peg.4268	Contig_5	74538	74705	+	hypothetical protein		
fig|6666666.28482.peg.4269	Contig_5	75154	74798	-	hypothetical protein	FIG00638284	ff
fig|6666666.28482.peg.4270	Contig_5	75226	76701	+	FIG00820761: hypothetical protein	FIG00820760	ff
fig|6666666.28482.peg.4271	Contig_5	77331	76903	-	hypothetical protein		
fig|6666666.28482.peg.4272	Contig_5	77615	78490	+	FIG00823100: hypothetical protein	FIG01363915	ff
fig|6666666.28482.peg.4273	Contig_5	79379	78555	-	Beta-lactamase related protein	FIG01424176	ff
fig|6666666.28482.peg.4274	Contig_5	81210	79657	-	Choline dehydrogenase (EC 1.1.99.1)	FIG00057416	idu(4);Choline_uptake_and_conversion_to_betaine_clusters idu(4);Choline_and_Betaine_Uptake_and_Betaine_Biosynthesis
fig|6666666.28482.peg.4275	Contig_5	81458	81225	-	hypothetical protein		
fig|6666666.28482.peg.4276	Contig_5	82387	81464	-	Alpha/beta hydrolase fold-3		
fig|6666666.28482.peg.4277	Contig_5	82924	82499	-	hypothetical protein		
fig|6666666.28482.peg.4278	Contig_5	83526	83005	-	Transcriptional regulator, TetR family		
fig|6666666.28482.peg.4279	Contig_5	84756	83677	-	alkylhydroperoxidase like protein, AhpD family		
fig|6666666.28482.peg.4280	Contig_5	85758	84904	-	Oxidoreductase, short-chain dehydrogenase/reductase family (EC 1.1.1.-)		idu(2);CBSS-291331.3.peg.3674 idu(2);Capsular_Polysaccharides_Biosynthesis_and_Assembly
fig|6666666.28482.peg.4281	Contig_5	85932	85795	-	hypothetical protein		
fig|6666666.28482.peg.4282	Contig_5	86067	86711	+	Transcriptional regulator, HxlR family	FIG00906075	ff

fig|6666666.28482.peg.4283	Contig_5	86772	86915	+	hypothetical protein		
fig|6666666.28482.peg.4284	Contig_5	90180	87097	-	Replicative DNA helicase (EC 3.6.1.-) @ intein-containing	FIG00018436	isu;Inteins isu;DNA-replication
fig|6666666.28482.peg.4285	Contig_5	91171	90713	-	LSU ribosomal protein L9p	FIG00000205	isu;Ribosome_LSU_bacterial
fig|6666666.28482.peg.4286	Contig_5	91456	91202	-	SSU ribosomal protein S18p @ SSU ribosomal protein S18p, zinc-dependent	FIG01306972	isu;Staphylococcal_pathogenicity_islands_SaPI
fig|6666666.28482.peg.4287	Contig_50	1590	697	-	Peptidyl-prolyl cis-trans isomerase (EC 5.2.1.8)	FIG01955868	idu(1);Peptidyl-prolyl_cis-trans_isomerase_containing_cluster
fig|6666666.28482.peg.4288	Contig_50	1698	2360	+	Hydroxyacylglutathione hydrolase (EC 3.1.2.6)	FIG00000013	isu;CBSS-342610.3.peg.1536 isu;Glutathione:_Non-redox_reactions isu;Methylglyoxal_Metabolism
fig|6666666.28482.peg.4289	Contig_50	2384	3619	+	Histidyl-tRNA synthetase (EC 6.1.1.21)	FIG00000087	isu;tRNA_aminoacylation,_His
fig|6666666.28482.peg.4290	Contig_50	3668	3976	+	FIG00827542: hypothetical protein	FIG00827541	ff
fig|6666666.28482.peg.4291	Contig_50	4027	4500	+	hypothetical protein		
fig|6666666.28482.peg.4292	Contig_50	4889	5008	+	hypothetical protein		
fig|6666666.28482.peg.4293	Contig_50	5313	4993	-	hypothetical protein		
fig|6666666.28482.peg.4294	Contig_50	5387	5506	+	hypothetical protein		
fig|6666666.28482.peg.4295	Contig_51	113	1204	+	FIG00824215: hypothetical protein	FIG00824211	ff
fig|6666666.28482.peg.4296	Contig_51	1478	2572	+	Probable endopeptidase	FIG00994167	ff
fig|6666666.28482.peg.4297	Contig_51	2535	3449	+	FIG00821136: hypothetical protein	FIG00821135	ff

fig|6666666.28482.peg.4298	Contig_51	3459	4601	+	Poly(glycerol-phosphate) alpha-glucosyltransferase (EC 2.4.1.52)	FIG00139262	isu;Teichoic_and_lipoteichoic_acids_biosynthesis
fig|6666666.28482.peg.4299	Contig_51	6511	4706	-	Long-chain-fatty-acid--CoA ligase (EC 6.2.1.3)	FIG00018699	idu(72);n-Phenylalkanoic_acid_degradation idu(72);Fatty_acid_metabolism_cluster idu(72);Biotin_synthesis_cluster idu(72);Biotin_biosynthesis
fig|6666666.28482.peg.4300	Contig_51	6692	7081	+	FIG00995034: hypothetical protein	FIG00820471	ff
fig|6666666.28482.peg.4301	Contig_51	7182	7619	+	FIG00821549: hypothetical protein	FIG00821548	ff
fig|6666666.28482.peg.4302	Contig_51	7729	8871	+	Arsenical pump-driving ATPase (EC 3.6.3.16)	FIG00003088	idu(2);Stress_related_cluster idu(2);Arsenic_resistance
fig|6666666.28482.peg.4303	Contig_51	8868	9257	+	FIG00821592: hypothetical protein	FIG00821590	ff
fig|6666666.28482.peg.4304	Contig_51	9345	10073	+	1-acyl-sn-glycerol-3-phosphate acyltransferase (EC 2.3.1.51)	FIG00005243	idu(6);Glycerolipid_and_Glycerophospholipid_Metabolism_in_Bacteria idu(6);Ribosome_post-transcriptional_modification_and_chromosomal_segregation_cluster
fig|6666666.28482.peg.4305	Contig_51	11305	10070	-	Possible membrane protein	FIG01121868	ff
fig|6666666.28482.peg.4306	Contig_51	11455	12339	+	Possible membrane protein	FIG01121868	ff
fig|6666666.28482.peg.4307	Contig_51	12372	12875	+	FIG00994909: hypothetical protein	FIG00994907	ff

fig|6666666.28482.peg.4308	Contig_51	12910	14298	+	2-keto-3-deoxy-D-arabino-heptulosonate-7-phosphate synthase II (EC 2.5.1.54)	FIG00133297	isu;Chorismate_Synthesis isu;Common_Pathway_For_Synthesis_of_Aromatic_Compounds_(DAHP_synthase_to_chorismate)
fig|6666666.28482.peg.4309	Contig_51	15580	14381	-	Probable serine/threonine-protein kinase pknL (EC 2.7.11.1)	FIG00547803	ff
fig|6666666.28482.peg.4310	Contig_51	15649	16053	+	Conserved hypothetical regulatory protein	FIG00996715	ff
fig|6666666.28482.peg.4311	Contig_51	17551	16040	-	Carotene biosynthesis associated membrane protein	FIG00861900	ff
fig|6666666.28482.peg.4312	Contig_51	18605	17556	-	Geranylgeranyl pyrophosphate synthetase (EC 2.5.1.29)	FIG00051800	ff
fig|6666666.28482.peg.4313	Contig_51	18770	19678	+	FIG00821173: hypothetical protein	FIG00821172	ff
fig|6666666.28482.peg.4314	Contig_51	20394	19675	-	Lipoprotein LppM	FIG00997195	ff
fig|6666666.28482.peg.4315	Contig_51	21056	20448	-	Acetyltransferase (EC 2.3.1.-)	FIG01195044	ff
fig|6666666.28482.peg.4316	Contig_51	21373	21774	+	hypothetical protein		
fig|6666666.28482.peg.4317	Contig_51	22165	22596	+	Cell division protein MraZ	FIG00000547	icw(2);Bacterial_Cell_Division icw(1);16S_rRNA_modification_within_P_site_of_ribosome icw(1);Bacterial_Cytoskeleton
fig|6666666.28482.peg.4318	Contig_51	22748	23770	+	rRNA small subunit methyltransferase H	FIG00000172	icw(1);Bacterial_Cell_Division icw(2);16S_rRNA_modification_within_P_site_of_ribosome

fig|6666666.28482.peg.4319	Contig_51	23767	24939	+	FIG034299: proline rich membrane protein	FIG00862611	ff
fig|6666666.28482.peg.4320	Contig_51	24936	26948	+	Cell division protein FtsI [Peptidoglycan synthetase] (EC 2.4.1.129)	FIG00046929	idu(2);Bacterial_Cell_Division idu(2);16S_rRNA_modification_within_P_site_of_ribosome idu(2);Flagellum_in_Campylobacter idu(2);Bacterial_Cytoskeleton icw(1);Peptidoglycan_Biosynthesis
fig|6666666.28482.peg.4321	Contig_51	27140	28660	+	UDP-N-acetylmuramoylalanyl-D-glutamate--2,6-diaminopimelate ligase (EC 6.3.2.13)	FIG00000243	icw(1);Peptidoglycan_biosynthesis--gjo isu;Methicillin_resistance_in_Staphylococci isu;Peptidoglycan_Biosynthesis
fig|6666666.28482.peg.4322	Contig_51	28657	30186	+	UDP-N-acetylmuramoylalanyl-D-glutamyl-2,6-diaminopimelate--D-alanyl-D-alanine ligase (EC 6.3.2.10)	FIG00024401	icw(2);Peptidoglycan_biosynthesis--gjo icw(1);Methicillin_resistance_in_Staphylococci icw(3);Peptidoglycan_Biosynthesis
fig|6666666.28482.peg.4323	Contig_51	30183	31262	+	Phospho-N-acetylmuramoyl-pentapeptide-transferase (EC 2.7.8.13)	FIG00000221	icw(5);Peptidoglycan_Biosynthesis
fig|6666666.28482.peg.4324	Contig_51	31264	32715	+	UDP-N-acetylmuramoylalanine--D-glutamate ligase (EC 6.3.2.9)	FIG00085033	icw(1);Peptidoglycan_biosynthesis--gjo icw(1);Peptidoglycan_Biosynthesis
fig|6666666.28482.peg.4325	Contig_51	32721	34406	+	Cell division protein FtsW	FIG00000025	idu(1);Bacterial_Cell_Division idu(1);Bacterial_Cytoskeleton

fig|6666666.28482.peg.4326	Contig_51	34403	35593	+	UDP-N-acetylglucosamine--N-acetylmuramyl-(pentapeptide) pyrophosphoryl-undecaprenol N-acetylglucosamine transferase (EC 2.4.1.227)	FIG00047056	icw(1);Peptidoglycan_Biosynthesis
fig|6666666.28482.peg.4327	Contig_51	35590	37059	+	UDP-N-acetylmuramate--alanine ligase (EC 6.3.2.8)	FIG00135554	isu;Peptidoglycan_biosynthesis--gjo icw(2);Peptidoglycan_Biosynthesis
fig|6666666.28482.peg.4328	Contig_51	37056	37988	+	Cell division protein FtsQ	FIG00021517	icw(1);Bacterial_Cell_Division icw(1);Bacterial_Cytoskeleton isu;CBSS-227882.1.peg.6980
fig|6666666.28482.peg.4329	Contig_52	323	553	+	hypothetical protein		
fig|6666666.28482.peg.4330	Contig_52	1315	584	-	FIG00996117: hypothetical protein	FIG00820216	ff
fig|6666666.28482.peg.4331	Contig_52	2739	1303	-	Cell envelope-associated transcriptional attenuator LytR-CpsA-Psr, subfamily A1 (as in PMID19099556)	FIG00025213	idu(3);Cell_envelope-associated_LytR-CpsA-Psr_transcriptional_attenuators
fig|6666666.28482.peg.4332	Contig_52	2868	3755	+	dTDP-4-dehydrorhamnose reductase (EC 1.1.1.133)	FIG00000368	isu;dTDP-rhamnose_synthesis isu;Rhamnose_containing_glycans

fig|6666666.28482.peg.4333	Contig_52	3769	4641	+	dTDP-Rha:A-D-GlcNAc-diphosphoryl polyprenol, A-3-L-rhamnosyl transferase WbbL	FIG01030833	icw(1);dTDP-rhamnose_synthesis
fig|6666666.28482.peg.4334	Contig_52	4643	5722	+	D-glycero-D-manno-heptose 1-phosphate guanosyltransferase	FIG00003212	isu;Capsular_heptose_biosynthesis isu;Capsular_Polysaccharides_Biosynthesis_and_Assembly
fig|6666666.28482.peg.4335	Contig_52	6313	5780	-	NTP pyrophosphohydrolases including oxidative damage repair enzymes	FIG00004782	idu(1);CBSS-216591.1.peg.168
fig|6666666.28482.peg.4336	Contig_52	7884	6310	-	probable DNA methylase	FIG01326804	ff
fig|6666666.28482.peg.4337	Contig_52	9247	7895	-	Coenzyme F420-0:L-glutamate ligase @ Coenzyme F420-1:L-glutamate ligase / domain of unknown function	FIG00009859	isu;Coenzyme_F420_synthesis isu;Coenzyme_F420_synthesis
fig|6666666.28482.peg.4338	Contig_52	10245	9253	-	Lactyl (2) diphospho-(5')guanosine:7,8-didemethyl-8-hydroxy-5-deazariboflavin 2-phospho-L-lactate transferase	FIG00044377	icw(1);Coenzyme_F420_synthesis
fig|6666666.28482.peg.4339	Contig_52	10539	10904	+	Sporulation regulatory protein WhiB	FIG00019146	isu;WhiB_and_WhiB-type_regulatory_proteins_
fig|6666666.28482.peg.4340	Contig_52	11340	10918	-	FIG00822333: hypothetical protein	FIG00822329	ff
fig|6666666.28482.peg.4341	Contig_52	11563	11964	+	hypothetical protein		

fig|6666666.28482.peg.4342	Contig_52	13064	11961	-	hypothetical protein	FIG00638284	ff
fig|6666666.28482.peg.4343	Contig_53	71	1381	+	Translation initiation factor 2	FIG00000102	icw(1);CBSS-138119.3.peg.2719 isu;NusA-TFII_Cluster isu;Translation_initiation_factors_bacterial icw(1);CBSS-350688.3.peg.1509
fig|6666666.28482.peg.4344	Contig_53	1433	1915	+	Ribosome-binding factor A	FIG00000164	icw(2);CBSS-138119.3.peg.2719 icw(1);NusA-TFII_Cluster icw(1);Translation_initiation_factors_bacterial isu;CBSS-350688.3.peg.1509
fig|6666666.28482.peg.4345	Contig_53	1890	2900	+	FIG146085: 3'-to-5' oligoribonuclease A, Bacillus type	FIG00002194	isu;CBSS-138119.3.peg.2719 isu;RNA_processing_and_degradation,_bacterial
fig|6666666.28482.peg.4346	Contig_53	3483	2908	-	FIG00829495: hypothetical protein	FIG00829492	ff
fig|6666666.28482.peg.4347	Contig_53	4239	3493	-	Enoyl-CoA hydratase (EC 4.2.1.17)	FIG00033206	idu(51);n-Phenylalkanoic_acid_degradation idu(51);Polyhydroxybutyrate_metabolism idu(51);Isoleucine_degradation idu(51);Fatty_acid_metabolism_cluster idu(51);Lysine_fermentation idu(51);Butanol_Biosynthesis idu(51);Acetyl-CoA_fermentation_to_Butyrate idu(51);Valine_degradation idu(51);Butyrate_metabolism_cluster
fig|6666666.28482.peg.4348	Contig_53	4380	4649	+	COGs COG0840	FIG01320521	ff

fig|6666666.28482.peg.4349	Contig_53	4646	5194	+	FIG00820705: hypothetical protein	FIG00820704	ff
fig|6666666.28482.peg.4350	Contig_53	6889	5153	-	Possible hydrolase	FIG01381897	ff
fig|6666666.28482.peg.4351	Contig_53	7504	6923	-	Possible membrane protein	FIG01121868	ff
fig|6666666.28482.peg.4352	Contig_53	7627	8592	+	putative SimX4 homolog	FIG01127638	ff
fig|6666666.28482.peg.4353	Contig_53	8589	9272	+	4'-phosphopantetheinyl transferase EntD (EC 2.7.8.-)	FIG01124387	ff
fig|6666666.28482.peg.4354	Contig_53	9269	10150	+	tRNA pseudouridine synthase B (EC 4.2.1.70)	FIG01016270	isu;CBSS-138119.3.peg.2719 isu;tRNA_processing isu;RNA_pseudouridine_syntheses isu;CBSS-350688.3.peg.1509
fig|6666666.28482.peg.4355	Contig_53	10982	10155	-	hypothetical protein	FIG00638284	ff
fig|6666666.28482.peg.4356	Contig_53	11149	12339	+	3-ketoacyl-CoA thiolase (EC 2.3.1.16)	FIG01279374	idu(11);CBSS-246196.1.peg.364 idu(11);Serine-glyoxylate_cycle idu(11);n-Phenylalkanoic_acid_degradation idu(11);Polyhydroxybutyrate_metabolism icw(1);Isoleucine_degradation idu(11);Fatty_acid_metabolism_cluster idu(11);Biotin_synthesis_cluster idu(11);Biotin_biosynthesis

fig|6666666.28482.peg.4357	Contig_53	12347	13582	+	Butyryl-CoA dehydrogenase (EC 1.3.99.2)	FIG00009149	idu(39);Isoleucine_degradation idu(39);Lysine_fermentation idu(39);Butanol_Biosynthesis idu(39);Acetyl-CoA_fermentation_to_Butyrate idu(39);Valine_degradation idu(39);Anaerobic_respiratory_reductases
fig|6666666.28482.peg.4358	Contig_53	14283	13579	-	Mn-dependent transcriptional regulator MntR	FIG00634996	ff
fig|6666666.28482.peg.4359	Contig_53	14429	15352	+	Riboflavin kinase (EC 2.7.1.26) / FMN adenylyltransferase (EC 2.7.7.2)	FIG00000220	isu;Riboflavin,_FMN_and_FAD_metabolism isu;Riboflavin,_FMN_and_FAD_metabolism isu;CBSS-350688.3.peg.1509 icw(1);CBSS-350688.3.peg.1509 isu;riboflavin_to_FAD isu;riboflavin_to_FAD
fig|6666666.28482.peg.4360	Contig_53	15616	15927	+	SSU ribosomal protein S15p (S13e)	FIG00000144	icw(1);CBSS-350688.3.peg.1509
fig|6666666.28482.peg.4361	Contig_53	16276	18522	+	Polyribonucleotide nucleotidyltransferase (EC 2.7.7.8)	FIG00000391	isu;Polyadenylation_bacterial icw(3);CBSS-350688.3.peg.1509 icw(1);CBSS-1806.1.peg.3045
fig|6666666.28482.peg.4362	Contig_53	18503	19864	+	FIG007959: peptidase, M16 family	FIG00007959	icw(2);CBSS-350688.3.peg.1509 isu;CBSS-1806.1.peg.3045
fig|6666666.28482.peg.4363	Contig_53	19925	20947	+	2-nitropropane dioxygenase (EC 1.13.11.32)	FIG01304356	idu(5);CBSS-164757.7.peg.5020
fig|6666666.28482.peg.4364	Contig_53	20985	21170	+	hypothetical protein		
fig|6666666.28482.peg.4365	Contig_53	22061	21366	-	hypothetical protein	FIG00638284	ff
fig|6666666.28482.peg.4366	Contig_53	22122	22637	+	Transcriptional regulator, AsnC family	FIG01956023	ff

fig|6666666.28482.peg.4367	Contig_53	22653	24113	+	Cyclohexanone monooxygenase (EC 1.14.13.22)	FIG00975774	ff
fig|6666666.28482.peg.4368	Contig_53	24167	25075	+	FF domain protein	FIG01341765	ff
fig|6666666.28482.peg.4369	Contig_53	25079	26839	+	FIG00824361: hypothetical protein	FIG00824358	ff
fig|6666666.28482.peg.4370	Contig_53	26850	27926	+	Vanillate O-demethylase oxidoreductase (EC 1.14.13.-)	FIG01303955	idu(2);Anaerobic_respiratory_reductases
fig|6666666.28482.peg.4371	Contig_53	28425	27913	-	Acetyltransferase, GNAT family		
fig|6666666.28482.peg.4372	Contig_53	29083	28442	-	Transcriptional regulator, TetR family	FIG00554417	ff
fig|6666666.28482.peg.4373	Contig_53	29142	30122	+	FIG00826122: hypothetical protein	FIG00826121	ff
fig|6666666.28482.peg.4374	Contig_53	30129	31208	+	FIG00823153: hypothetical protein	FIG00823152	ff
fig|6666666.28482.peg.4375	Contig_53	32585	31218	-	Partial REP13E12 repeat protein	FIG00820827	ff
fig|6666666.28482.peg.4376	Contig_53	32990	32706	-	hypothetical protein		
fig|6666666.28482.peg.4377	Contig_53	33179	33916	+	Dihydrodipicolinate reductase (EC 1.3.1.26)	FIG00000347	isu;Lysine_Biosynthesis_DAP_Pathway,_GJO_scratch isu;Lysine_Biosynthesis_DAP_Pathway
fig|6666666.28482.peg.4378	Contig_53	33913	34371	+	FIG00826342: hypothetical protein	FIG00826340	ff
fig|6666666.28482.peg.4379	Contig_53	34368	34823	+	Multimeric flavodoxin WrbA	FIG01193004	ff
fig|6666666.28482.peg.4380	Contig_53	35656	34808	-	TesB-like acyl-CoA thioesterase 4	FIG00085764	idu(2);Acyl-CoA_thioesterase_II

fig|6666666.28482.peg.4381	Contig_53	35706	37232	+	Long-chain-fatty-acid--CoA ligase (EC 6.2.1.3)	FIG00018699	idu(72);n-Phenylalkanoic_acid_degradation idu(72);Fatty_acid_metabolism_cluster idu(72);Biotin_synthesis_cluster idu(72);Biotin_biosynthesis
fig|6666666.28482.peg.4382	Contig_53	37229	40432	+	FIG00828084: hypothetical protein	FIG00828083	ff
fig|6666666.28482.peg.4383	Contig_53	40758	40444	-	PE-PGRS family protein	FIG01371720	idu(16);PPE_gene_cluster_in_Mycobacteria
fig|6666666.28482.peg.4384	Contig_53	40870	41637	+	3-oxoacyl-[acyl-carrier protein] reductase (EC 1.1.1.100)	FIG00621114	idu(38);CBSS-246196.1.peg.364 idu(38);CBSS-164757.7.peg.5020 idu(38);Fatty_Acid_Biosynthesis_FASII
fig|6666666.28482.peg.4385	Contig_53	41634	42827	+	putative acyl-CoA dehydrogenase	FIG00476656	ff
fig|6666666.28482.peg.4386	Contig_53	42824	43915	+	Acyl-CoA dehydrogenase (EC 1.3.99.-)	FIG00820935	ff
fig|6666666.28482.peg.4387	Contig_53	44917	46161	+	possible glycosyltransferase	FIG01399847	ff
fig|6666666.28482.peg.4388	Contig_53	46890	46150	-	Dienelactone hydrolase family	FIG01069253	ff
fig|6666666.28482.peg.4389	Contig_53	46933	47733	+	Thymidylate synthase (EC 2.1.1.45)	FIG00000398	icw(1);Folate_Biosynthesis isu;pyrimidine_conversions
fig|6666666.28482.peg.4390	Contig_53	47823	48266	+	Dihydrofolate reductase (EC 1.5.1.3)	FIG00000048	idu(3);Folate_Biosynthesis
fig|6666666.28482.peg.4391	Contig_53	48378	49079	+	FIG00820787: hypothetical protein	FIG00820786	ff
fig|6666666.28482.peg.4392	Contig_53	49133	50365	+	FIG00822893: hypothetical protein	FIG00815749	ff

fig|6666666.28482.peg.4393	Contig_53	50365	51705	+	Type I restriction-modification system, DNA-methyltransferase subunit M (EC 2.1.1.72)	FIG00000477	icw(1);Restriction-Modification_System icw(1);Type_I_Restriction-Modification
fig|6666666.28482.peg.4394	Contig_53	51702	52760	+	Type I restriction-modification system, specificity subunit S (EC 3.1.21.3)	FIG00000494	isu;Restriction-Modification_System isu;Type_I_Restriction-Modification
fig|6666666.28482.peg.4395	Contig_53	52858	53610	+	Thymidylate synthase thyX (EC 2.1.1.-)	FIG00001348	isu;Folate_Biosynthesis
fig|6666666.28482.peg.4396	Contig_53	53718	54620	+	Dihydrodipicolinate synthase (EC 4.2.1.52)	FIG00019600	isu;Lysine_Biosynthesis_DAP_Pathway,_GJO_scratch isu;Lysine_Biosynthesis_DAP_Pathway
fig|6666666.28482.peg.4397	Contig_53	54628	56304	+	Ribonuclease J2 (endoribonuclease in RNA processing)	FIG01303963	isu;Ribonucleases_in_Bacillus
fig|6666666.28482.peg.4398	Contig_53	57173	56301	-	O-Methyltransferase involved in polyketide biosynthesis	FIG01313020	ff
fig|6666666.28482.peg.4399	Contig_53	58004	57180	-	Dehydrogenase	FIG01323940	ff
fig|6666666.28482.peg.4400	Contig_53	58315	58001	-	Antibiotic biosynthesis monooxygenase	FIG00027695	idu(1);CBSS-1085.1.peg.1363
fig|6666666.28482.peg.4401	Contig_53	58648	60966	+	Cell division protein FtsK	FIG00000287	isu;Unspecified_monosaccharide_transport_cluster isu;Bacterial_Cell_Division isu;Bacterial_Cytoskeleton
fig|6666666.28482.peg.4402	Contig_53	61521	61033	-	Acetyltransferase, GNAT family	FIG00989082	ff

fig|6666666.28482.peg.4403	Contig_53	61635	62207	+	CDP-diacylglycerol--glycerol-3-phosphate 3-phosphatidyltransferase (EC 2.7.8.5)	FIG00001550	idu(2);Glycerolipid_and_Glycerophospholipid_Metabolism_in_Bacteria
fig|6666666.28482.peg.4404	Contig_53	62311	62646	+	Transcriptional regulator, XRE family	FIG01123280	ff
fig|6666666.28482.peg.4405	Contig_53	62760	63578	+	Phage shock protein A (IM30) , suppresses sigma54-dependent transcription	FIG00815031	ff
fig|6666666.28482.peg.4406	Contig_54	58	276	+	PPE family protein	FIG00013075	icw(2);CBSS-164757.7.peg.5020
fig|6666666.28482.peg.4407	Contig_54	840	2078	+	PPE family protein	FIG00013075	icw(3);CBSS-164757.7.peg.5020
fig|6666666.28482.peg.4408	Contig_54	2130	3359	+	PPE family protein	FIG00013075	icw(3);CBSS-164757.7.peg.5020
fig|6666666.28482.peg.4409	Contig_54	3715	4014	+	PE family protein	FIG01313150	ff
fig|6666666.28482.peg.4410	Contig_54	4241	4537	+	FIG00824639: hypothetical protein	FIG00823510	ff
fig|6666666.28482.peg.4411	Contig_54	4588	4872	+	ESAT-6-like protein EsxL	FIG00820465	ff
fig|6666666.28482.peg.4412	Contig_54	5817	4936	-	PPE family protein	FIG00013075	icw(2);CBSS-164757.7.peg.5020
fig|6666666.28482.peg.4413	Contig_54	6529	7302	+	DNA-binding response regulator TrcR	FIG01310367	ff
fig|6666666.28482.peg.4414	Contig_54	7299	8837	+	Sensor protein basS/pmrB (EC 2.7.3.-)	FIG00000853	isu;Lipid_A_modifications
fig|6666666.28482.peg.4415	Contig_54	9232	10902	+	Potassium-transporting ATPase A chain (EC 3.6.3.12) (TC 3.A.3.7.1)	FIG00002960	isu;Potassium_homeostasis
fig|6666666.28482.peg.4416	Contig_54	10902	13052	+	Potassium-transporting ATPase B chain (EC 3.6.3.12) (TC 3.A.3.7.1)	FIG00046742	icw(3);Potassium_homeostasis

fig|6666666.28482.peg.4417	Contig_54	13054	13977	+	Potassium-transporting ATPase C chain (EC 3.6.3.12) (TC 3.A.3.7.1)	FIG00007507	icw(1);Potassium_homeostasis
fig|6666666.28482.peg.4418	Contig_54	14001	16559	+	Osmosensitive K+ channel histidine kinase KdpD (EC 2.7.3.-)	FIG00000846	icw(1);Potassium_homeostasis
fig|6666666.28482.peg.4419	Contig_54	16609	17289	+	DNA-binding response regulator KdpE		
fig|6666666.28482.peg.4420	Contig_55	301	879	+	FIG00820113: hypothetical protein	FIG00820111	ff
fig|6666666.28482.peg.4421	Contig_55	1449	967	-	MCE-associated transmembrane protein	FIG00820242	ff
fig|6666666.28482.peg.4422	Contig_55	2105	1449	-	MCE-associated alanine and valine rich protein	FIG00820054	ff
fig|6666666.28482.peg.4423	Contig_55	3862	2141	-	MCE-family protein Mce1F	FIG00821171	idu(7);Mycobacterium_virulence_operon_MCE_involved_in_cell_invasion
fig|6666666.28482.peg.4424	Contig_55	5039	3873	-	MCE-family lipoprotein LprK (MCE-family lipoprotein Mce1e)	FIG00819998	icw(3);Mycobacterium_virulence_operon_MCE_involved_in_cell_invasion
fig|6666666.28482.peg.4425	Contig_55	6433	5036	-	MCE-family protein Mce1D	FIG00820669	icw(1);Mycobacterium_virulence_operon_MCE_involved_in_cell_invasion
fig|6666666.28482.peg.4426	Contig_55	7521	6457	-	MCE-family protein Mce1C	FIG00821293	icw(7);Mycobacterium_virulence_operon_MCE_involved_in_cell_invasion

fig|6666666.28482.peg.4427	Contig_55	8563	7511	-	MCE-family protein Mce1B	FIG00763551	icw(5);Mycobacterium_virulence_operon_MCE_involved_in_cell_invasion
fig|6666666.28482.peg.4428	Contig_55	9765	8563	-	MCE-family protein Mce1A	FIG00820494	icw(2);Mycobacterium_virulence_operon_MCE_involved_in_cell_invasion
fig|6666666.28482.peg.4429	Contig_55	10627	9785	-	Conserved hypothetical integral membrane protein YrbE1B	FIG00691621	icw(3);Mycobacterium_virulence_operon_MCE_involved_in_cell_invasion
fig|6666666.28482.peg.4430	Contig_55	11366	10635	-	Conserved hypothetical integral membrane protein YrbE1A	FIG00690583	idu(7);Mycobacterium_virulence_operon_MCE_involved_in_cell_invasion
fig|6666666.28482.peg.4431	Contig_55	12506	11637	-	Probable short-chain type dehydrogenase/reductase (EC 1.-.-.-)	FIG00026991	idu(2);CBSS-164757.7.peg.5020
fig|6666666.28482.peg.4432	Contig_55	12767	12576	-	Ferredoxin	FIG00001676	idu(4);Soluble_cytochromes_and_functionally_related_electron_carriers idu(4);Inorganic_Sulfur_Assimilation
fig|6666666.28482.peg.4433	Contig_55	12957	14138	+	FadE30	FIG00457540	icw(1);CBSS-164757.7.peg.5020
fig|6666666.28482.peg.4434	Contig_55	14172	15269	+	Butyryl-CoA dehydrogenase (EC 1.3.99.2)	FIG00009149	idu(39);Isoleucine_degradation idu(39);Lysine_fermentation idu(39);Butanol_Biosynthesis idu(39);Acetyl-CoA_fermentation_to_Butyrate idu(39);Valine_degradation idu(39);Anaerobic_respiratory_reductases

fig|6666666.28482.peg.4435	Contig_55	15301	16818	+	Long-chain-fatty-acid--CoA ligase (EC 6.2.1.3)	FIG00018699	icw(1);n-Phenylalkanoic_acid_degradation icw(1);Fatty_acid_metabolism_cluster icw(1);Biotin_synthesis_cluster icw(1);Biotin_biosynthesis
fig|6666666.28482.peg.4436	Contig_55	16800	17513	+	FIG00824473: hypothetical protein	FIG00824470	ff
fig|6666666.28482.peg.4437	Contig_55	19086	17536	-	COG0028: Thiamine pyrophosphate-requiring enzymes	FIG00452446	ff
fig|6666666.28482.peg.4438	Contig_55	19907	19083	-	PROBABLE ANTIBIOTIC-RESISTANCE PROTEIN	FIG01366849	ff
fig|6666666.28482.peg.4439	Contig_55	21609	19960	-	Long-chain-fatty-acid--CoA ligase (EC 6.2.1.3)	FIG00018699	icw(1);n-Phenylalkanoic_acid_degradation icw(1);Fatty_acid_metabolism_cluster icw(1);Biotin_synthesis_cluster icw(1);Biotin_biosynthesis

fig|6666666.28482.peg.4440	Contig_55	21674	22486	+	Enoyl-CoA hydratase (EC 4.2.1.17)	FIG00033206	icw(1);n-Phenylalkanoic_acid_degradation idu(51);Polyhydroxybutyrate_metabolism idu(51);Isoleucine_degradation icw(1);Fatty_acid_metabolism_cluster idu(51);Lysine_fermentation idu(51);Butanol_Biosynthesis idu(51);Acetyl-CoA_fermentation_to_Butyrate idu(51);Valine_degradation idu(51);Butyrate_metabolism_cluster
fig|6666666.28482.peg.4441	Contig_55	23889	22687	-	putative cytochrome P450 hydroxylase	FIG00032437	idu(49);Nitric_oxide_synthase
fig|6666666.28482.peg.4442	Contig_55	24602	23886	-	Acetoacetate decarboxylase family protein	FIG00822635	ff
fig|6666666.28482.peg.4443	Contig_55	25698	24646	-	Coenzyme F420-dependent oxidoreductase	FIG00015014	idu(3);Anaerobic_respiratory_reductases
fig|6666666.28482.peg.4444	Contig_55	25790	26755	+	FIG00996312: hypothetical protein	FIG00820395	ff
fig|6666666.28482.peg.4445	Contig_55	26771	27832	+	Lipid-transfer protein	FIG01125818	ff
fig|6666666.28482.peg.4446	Contig_55	27832	29025	+	3-ketoacyl-CoA thiolase	FIG00664812	ff
fig|6666666.28482.peg.4447	Contig_55	29022	29429	+	FIG00826355: hypothetical protein	FIG00826353	ff
fig|6666666.28482.peg.4448	Contig_55	29950	29426	-	carbonic anhydrase, family 3	FIG00553719	ff

fig|6666666.28482.peg.4449	Contig_55	30062	31231	+	Terminal oxygenase KshA	FIG01318144	ff
fig|6666666.28482.peg.4450	Contig_55	31236	31685	+	FIG00820011: hypothetical protein	FIG00820010	ff
fig|6666666.28482.peg.4451	Contig_55	33008	31863	-	FIG00994930: hypothetical protein	FIG00820936	ff
fig|6666666.28482.peg.4452	Contig_55	33781	33008	-	short chain dehydrogenase		
fig|6666666.28482.peg.4453	Contig_55	34920	33793	-	FIG00994788: hypothetical protein	FIG00822710	ff
fig|6666666.28482.peg.4454	Contig_55	35223	35008	-	hypothetical protein	FIG00638284	ff
fig|6666666.28482.peg.4455	Contig_55	36594	35371	-	PPE family protein	FIG00013075	icw(1);CBSS-164757.7.peg.5020
fig|6666666.28482.peg.4456	Contig_55	38517	36757	-	PPE family protein	FIG00013075	icw(1);CBSS-164757.7.peg.5020
fig|6666666.28482.peg.4457	Contig_55	39710	38652	-	4-hydroxy-2-oxovalerate aldolase (EC 4.1.3.-)	FIG00001725	ff
fig|6666666.28482.peg.4458	Contig_55	40624	39707	-	Acetaldehyde dehydrogenase, acetylating, (EC 1.2.1.10) in gene cluster for degradation of phenols, cresols, catechol	FIG00002495	idu(1);Central_meta-cleavage_pathway_of_aromatic_compound_degradation idu(1);Biphenyl_Degradation
fig|6666666.28482.peg.4459	Contig_55	41452	40667	-	2-keto-4-pentenoate hydratase (EC 4.2.1.-)	FIG01304357	ff
fig|6666666.28482.peg.4460	Contig_55	41516	43207	+	3-oxosteroid 1-dehydrogenase (EC 1.3.99.4)	FIG01318047	ff
fig|6666666.28482.peg.4461	Contig_55	43209	44069	+	Enoyl-CoA hydratase	FIG00999917	ff
fig|6666666.28482.peg.4462	Contig_55	44125	45879	+	Uncharacterized NTE family protein	FIG01324713	ff
fig|6666666.28482.peg.4463	Contig_55	46028	46975	+	adenylate cyclase		

fig|6666666.28482.peg.4464	Contig_55	47802	47002	-	FIG00824875: hypothetical protein	FIG00824874	ff
fig|6666666.28482.peg.4465	Contig_55	49129	47960	-	Lipid carrier protein IgrF	FIG00010128	icw(1);Cholesterol_catabolic_operon_in_Mycobacteria
fig|6666666.28482.peg.4466	Contig_55	49530	49129	-	Enoyl coenzyme A hydratase IgrE	FIG00018965	isu;Cholesterol_catabolic_operon_in_Mycobacteria
fig|6666666.28482.peg.4467	Contig_55	50489	49527	-	Conserved protein IgrD	FIG00026271	icw(5);Cholesterol_catabolic_operon_in_Mycobacteria
fig|6666666.28482.peg.4468	Contig_55	51649	50486	-	Probable acyl-CoA dehydrogenase FadE29 (EC 1.3.99.-); Acyl-CoA dehydrogenase IgrC	FIG00118764	isu;CBSS-164757.7.peg.5020 icw(2);Cholesterol_catabolic_operon_in_Mycobacteria
fig|6666666.28482.peg.4469	Contig_55	52662	51640	-	Probable acyl-CoA dehydrogenase FadE28 (EC 1.3.99.-); Acyl-CoA dehydrogenase IgrB	FIG00145528	icw(4);CBSS-164757.7.peg.5020 icw(4);Cholesterol_catabolic_operon_in_Mycobacteria
fig|6666666.28482.peg.4470	Contig_55	53918	52665	-	Putative cytochrome P450 125 (EC 1.14.-.-); Putative cytochrome P450 IgrA	FIG00146831	icw(7);CBSS-164757.7.peg.5020 icw(3);Cholesterol_catabolic_operon_in_Mycobacteria
fig|6666666.28482.peg.4471	Contig_55	54113	55276	+	Probable acetyl-CoA acetyltransferase FadA5 (EC 2.3.1.9)	FIG00142837	icw(6);CBSS-164757.7.peg.5020
fig|6666666.28482.peg.4472	Contig_55	55276	55650	+	CysQ, putative	FIG00109461	icw(9);CBSS-164757.7.peg.5020
fig|6666666.28482.peg.4473	Contig_55	55673	56128	+	Putative uncharacterized protein BCG_3611	FIG00022045	icw(2);CBSS-164757.7.peg.5020

fig|6666666.28482.peg.4474	Contig_55	57184	56279	-	Putative uncharacterized protein (Hypothetical short-chain type dehydrogenase/reductase)	FIG00146907	isu;CBSS-164757.7.peg.5020
fig|6666666.28482.peg.4475	Contig_55	58006	57209	-	Probable short-chain type dehydrogenase/reductase (EC 1.-.-.-)	FIG00026991	icw(4);CBSS-164757.7.peg.5020
fig|6666666.28482.peg.4476	Contig_55	58066	58824	+	Probable enoyl-CoA hydratase EchA20 (EC 4.2.1.17)	FIG00145538	icw(3);CBSS-164757.7.peg.5020
fig|6666666.28482.peg.4477	Contig_55	58821	59705	+	Putative CoA-transferase subunit alpha Rv3551/MT3655 (EC 2.8.3.-)	FIG00065948	icw(5);CBSS-164757.7.peg.5020
fig|6666666.28482.peg.4478	Contig_55	59702	60442	+	Putative CoA-transferase subunit beta Rv3552/MT3656 (EC 2.8.3.-)	FIG00145596	icw(2);CBSS-164757.7.peg.5020
fig|6666666.28482.peg.4479	Contig_55	60442	61527	+	2-nitropropane dioxygenase (EC 1.13.11.32)	FIG01304356	icw(6);CBSS-164757.7.peg.5020
fig|6666666.28482.peg.4480	Contig_55	61572	61991	+	FIG00822806: hypothetical protein	FIG00822805	ff
fig|6666666.28482.peg.4481	Contig_55	62051	62698	+	Putative uncharacterized protein	FIG01341280	ff
fig|6666666.28482.peg.4482	Contig_55	62695	63435	+	FIG00824227: hypothetical protein	FIG00824226	ff
fig|6666666.28482.peg.4483	Contig_55	63455	63931	+	Transcriptional regulator, AraC family	FIG00510130	ff

fig|6666666.28482.peg.4484	Contig_55	63924	64355	+	putative lyase	FIG00999432	ff
fig|6666666.28482.peg.4485	Contig_55	65540	64380	-	Probable acetyl-CoA acetyltransferase FadA6 (EC 2.3.1.9)	FIG00145469	icw(5);CBSS-164757.7.peg.5020
fig|6666666.28482.peg.4486	Contig_55	66189	65596	-	Transcriptional factor in putative operon for degradation of branched-chain alkanes, nitroalkanes and may be also cyclic ketones, alkenoic acids	FIG00020572	isu;CBSS-164757.7.peg.5020
fig|6666666.28482.peg.4487	Contig_55	66729	68384	+	PPE family protein	FIG00013075	icw(3);CBSS-164757.7.peg.5020
fig|6666666.28482.peg.4488	Contig_55	68522	68358	-	hypothetical protein		
fig|6666666.28482.peg.4489	Contig_55	68859	70532	+	PPE family protein	FIG00013075	icw(3);CBSS-164757.7.peg.5020
fig|6666666.28482.peg.4490	Contig_55	71330	70542	-	Short-chain dehydrogenase/reductas e SDR	FIG00546160	idu(13);CBSS-164757.7.peg.5020 idu(13);Transcription_repair_cluster
fig|6666666.28482.peg.4491	Contig_55	72487	71327	-	FadE30	FIG00457540	icw(3);CBSS-164757.7.peg.5020
fig|6666666.28482.peg.4492	Contig_55	72535	74091	+	Long-chain-fatty-acid--CoA ligase (EC 6.2.1.3)	FIG00018699	idu(72);n-Phenylalkanoic_acid_degradation idu(72);Fatty_acid_metabolism_clust er idu(72);Biotin_synthesis_cluster idu(72);Biotin_biosynthesis
fig|6666666.28482.peg.4493	Contig_55	74093	75226	+	Acyl-CoA dehydrogenase, short-chain specific (EC 1.3.99.2)	FIG00009849	icw(1);Isoleucine_degradation

fig|6666666.28482.peg.4494	Contig_55	75226	76170	+	Butyryl-CoA dehydrogenase (EC 1.3.99.2)	FIG00009149	idu(39);Isoleucine_degradation idu(39);Lysine_fermentation idu(39);Butanol_Biosynthesis idu(39);Acetyl-CoA_fermentation_to_Butyrate idu(39);Valine_degradation idu(39);Anaerobic_respiratory_reductases
fig|6666666.28482.peg.4495	Contig_55	76167	77120	+	Acyl-CoA dehydrogenase FadE33	FIG01289712	ff
fig|6666666.28482.peg.4496	Contig_55	77117	78286	+	Valine--pyruvate aminotransferase (EC 2.6.1.66)	FIG00132736	isu;Branched-Chain_Amino_Acid_Biosynthesis isu;Pyruvate_Alanine_Serine_Interconversions isu;Alanine_biosynthesis
fig|6666666.28482.peg.4497	Contig_55	80051	78276	-	Long-chain-fatty-acid--CoA ligase (EC 6.2.1.3)	FIG00018699	idu(72);n-Phenylalkanoic_acid_degradation idu(72);Fatty_acid_metabolism_cluster idu(72);Biotin_synthesis_cluster idu(72);Biotin_biosynthesis
fig|6666666.28482.peg.4498	Contig_55	81065	80241	-	Arylamine N-acetyltransferase (EC 2.3.1.5)	FIG01382398	ff
fig|6666666.28482.peg.4499	Contig_55	81250	84330	+	Probable serine/threonine-protein kinase pknK (EC 2.7.11.1)	FIG00761809	ff
fig|6666666.28482.peg.4500	Contig_55	84444	84872	+	hypothetical protein	FIG00638284	ff
fig|6666666.28482.peg.4501	Contig_55	85742	84936	-	hypothetical protein		

fig|6666666.28482.peg.4502	Contig_55	86388	85825	-	Nitrilotriacetate monooxygenase component B (EC 1.14.13.-)	FIG00039067	idu(1);Aromatic_Amin_Catabolism
fig|6666666.28482.peg.4503	Contig_55	87303	86401	-	2,3-dihydroxybiphenyl 1,2-dioxygenase	FIG00004009	ff
fig|6666666.28482.peg.4504	Contig_55	88178	87300	-	2-hydroxy-6-oxo-6-phenylhexa-2,4-dienoate hydrolase (EC 3.7.1.-)	FIG00003780	idu(2);carbazol_degradation_cluster idu(2);Central_meta-cleavage_pathway_of_aromatic_compound_degradation idu(2);Biphenyl_Degradation
fig|6666666.28482.peg.4505	Contig_55	89362	88178	-	POSSIBLE OXIDOREDUCTASE	FIG00820017	ff
fig|6666666.28482.peg.4506	Contig_55	89511	90593	+	Phenylacetate-CoA oxygenase/reductase, PaaK subunit	FIG00019269	ff
fig|6666666.28482.peg.4507	Contig_55	90622	91155	+	FIG00821138: hypothetical protein	FIG00821137	ff
fig|6666666.28482.peg.4508	Contig_55	92101	91214	-	FIG00821247: hypothetical protein	FIG00821246	ff
fig|6666666.28482.peg.4509	Contig_55	94311	92188	-	Acyl-CoA dehydrogenase (EC 1.3.99.-)	FIG00820935	ff
fig|6666666.28482.peg.4510	Contig_55	94528	95190	+	Transcriptional regulator kstR (Rv3574), TetR family	FIG00995019	ff
fig|6666666.28482.peg.4511	Contig_55	96227	95187	-	Transcriptional regulator, LacI family	FIG00781023	ff
fig|6666666.28482.peg.4512	Contig_55	96384	97286	+	Zinc ABC transporter, periplasmic-binding protein ZnuA	FIG00021555	ff

fig|6666666.28482.peg.4513	Contig_55	97295	98119	+	Zinc ABC transporter, ATP-binding protein ZnuC	FIG00059280	ff
fig|6666666.28482.peg.4514	Contig_55	98107	98976	+	Zinc ABC transporter, inner membrane permease protein ZnuB	FIG00005113	ff
fig|6666666.28482.peg.4515	Contig_55	98998	99756	+	Lactam utilization protein LamB	FIG00003076	isu;Urea_carboxylase_and_Allophanate_hydrolase_cluster isu;EC699-706
fig|6666666.28482.peg.4516	Contig_55	99756	100676	+	Zn-dependent hydrolases of the beta-lactamase fold	FIG00822592	ff
fig|6666666.28482.peg.4517	Contig_55	100690	101931	+	Arsenic efflux pump protein	FIG00081580	isu;Arsenic_resistance
fig|6666666.28482.peg.4518	Contig_55	102254	101943	-	hypothetical protein		
fig|6666666.28482.peg.4519	Contig_55	104979	102367	-	hypothetical protein	FIG00638284	ff
fig|6666666.28482.peg.4520	Contig_55	105192	106568	+	FIG00826217: hypothetical protein	FIG00826215	ff
fig|6666666.28482.peg.4521	Contig_55	106600	109755	+	Adenylate cyclase (EC 4.6.1.1)	FIG00001294	idu(25);cAMP_signaling_in_bacteria
fig|6666666.28482.peg.4522	Contig_55	110720	109761	-	Glycerophosphoryl diester phosphodiesterase (EC 3.1.4.46)	FIG00002239	idu(1);Glycerol_and_Glycerol-3-phosphate_Uptake_and_Utilization idu(1);CBSS-176299.4.peg.1996A
fig|6666666.28482.peg.4523	Contig_55	111745	110816	-	23S rRNA (guanosine-2'-O-) -methyltransferase rlmB (EC 2.1.1.-)	FIG00001049	isu;RNA_methylation
fig|6666666.28482.peg.4524	Contig_55	113152	111746	-	Cysteinyl-tRNA synthetase (EC 6.1.1.16)	FIG00000086	isu;tRNA_aminoacylation,_Cys isu;Conserved_gene_cluster_possibly_involved_in_RNA_metabolism

fig|6666666.28482.peg.4525	Contig_55	113693	113202	-	2-C-methyl-D-erythritol 2,4-cyclodiphosphate synthase (EC 4.6.1.12)	FIG00084105	isu;Nonmevalonate_Branch_of_Isoprenoid_Biosynthesis isu;Isoprenoid_Biosynthesis isu;Stationary_phase_repair_cluster
fig|6666666.28482.peg.4526	Contig_55	114376	113690	-	2-C-methyl-D-erythritol 4-phosphate cytidylyltransferase (EC 2.7.7.60)	FIG00074225	icw(1);Nonmevalonate_Branch_of_Isoprenoid_Biosynthesis icw(1);Isoprenoid_Biosynthesis isu;Teichoic_and_lipoteichoic_acids_biosynthesis icw(1);Stationary_phase_repair_cluster
fig|6666666.28482.peg.4527	Contig_55	114881	114393	-	CarD-like transcriptional regulator	FIG00648631	ff
fig|6666666.28482.peg.4528	Contig_55	115172	115690	+	Lipoprotein LpqE	FIG01039405	ff
fig|6666666.28482.peg.4529	Contig_55	115741	117168	+	DNA repair protein RadA	FIG00000283	isu;Proteolysis_in_bacteria,_ATP-dependent isu;DNA_repair,_bacterial
fig|6666666.28482.peg.4530	Contig_55	117165	118238	+	DNA integrity scanning protein disA	FIG00012395	ff
fig|6666666.28482.peg.4531	Contig_55	119084	118245	-	FIG00820636: hypothetical protein	FIG00820635	ff
fig|6666666.28482.peg.4532	Contig_55	119896	119279	-	Carbonic anhydrase (EC 4.2.1.1)	FIG00023019	idu(1);Cyanate_hydrolysis
fig|6666666.28482.peg.4533	Contig_55	119895	120794	+	A/G-specific adenine glycosylase (EC 3.2.2.-)	FIG00000395	icw(1);DNA_repair,_bacterial isu;CBSS-176280.1.peg.1561
fig|6666666.28482.peg.4534	Contig_55	122288	120810	-	Formate hydrogenlyase subunit 5	FIG00139433	isu;Formate_hydrogenase
fig|6666666.28482.peg.4535	Contig_55	123754	122285	-	Hydrogenase-4 component F (EC 1.-.-.-)	FIG00003207	icw(1);Formate_hydrogenase

fig|6666666.28482.peg.4536	Contig_55	124416	123754	-	Hydrogenase-4 component E (EC 1.-.-.-)	FIG00138953	icw(4);Formate_hydrogenase
fig|6666666.28482.peg.4537	Contig_55	125372	124413	-	Formate hydrogenlyase subunit 4	FIG00133502	icw(2);Formate_hydrogenase
fig|6666666.28482.peg.4538	Contig_55	127345	125369	-	Hydrogenase-4 component B (EC 1.-.-.-) / Formate hydrogenlyase subunit 3	FIG01955952	icw(3);Formate_hydrogenase icw(4);Formate_hydrogenase
fig|6666666.28482.peg.4539	Contig_55	127821	127342	-	Formate hydrogenlyase subunit 11	FIG01307050	ff
fig|6666666.28482.peg.4540	Contig_55	128160	127825	-	transcriptional regulator, ArsR family		
fig|6666666.28482.peg.4541	Contig_55	128429	129175	+	hypothetical protein		
fig|6666666.28482.peg.4542	Contig_55	129168	130865	+	Glucose-methanol-choline (GMC) oxidoreductase:NAD binding site	FIG00001629	idu(3);Respiratory_dehydrogenases_1
fig|6666666.28482.peg.4543	Contig_55	131568	130849	-	PUTATIVE HYDROLASE	FIG00995931	ff
fig|6666666.28482.peg.4544	Contig_55	131692	132000	+	Antibiotic biosynthesis monooxygenase	FIG00999654	idu(1);CBSS-1085.1.peg.1363
fig|6666666.28482.peg.4545	Contig_55	132032	133339	+	Beta-lactamase (EC 3.5.2.6)	FIG00000747	idu(1);Beta-lactamase idu(1);Tn552
fig|6666666.28482.peg.4546	Contig_55	133344	134057	+	hypothetical protein		
fig|6666666.28482.peg.4547	Contig_55	134122	134715	+	bifunctional deaminase-reductase-like protein		

fig|6666666.28482.peg.4548	Contig_55	135271	134720	-	Probable phosphoglycerate mutase	FIG01123304	ff
fig|6666666.28482.peg.4549	Contig_55	136059	135268	-	Predicted cobalt transporter CbtA	FIG00016867	isu;Transport_of_Nickel_and_Cobalt isu;Coenzyme_B12_biosynthesis
fig|6666666.28482.peg.4550	Contig_55	136295	136080	-	FIG00823300: hypothetical protein	FIG00823295	ff
fig|6666666.28482.peg.4551	Contig_55	136813	136433	-	FIG00827377: hypothetical protein	FIG00827374	ff
fig|6666666.28482.peg.4552	Contig_55	139423	136889	-	ATP-dependent Clp protease, ATP-binding subunit ClpC / Negative regulator of genetic competence clcC/mecB	FIG00011902	isu;Proteolysis_in_bacteria,_ATP-dependent
fig|6666666.28482.peg.4553	Contig_55	139466	139750	+	hypothetical protein		
fig|6666666.28482.peg.4554	Contig_55	140048	139722	-	Histone protein Lsr2	FIG00448295	ff
fig|6666666.28482.peg.4555	Contig_55	141670	140174	-	Lysyl-tRNA synthetase (class II) (EC 6.1.1.6)	FIG00000300	isu;tRNA_aminoacylation,_Lys
fig|6666666.28482.peg.4556	Contig_55	142663	141848	-	Pantothenate kinase type III, CoaX-like (EC 2.7.1.33)	FIG00000695	icw(1);Coenzyme_A_Biosynthesis_cluster isu;Coenzyme_A_Biosynthesis
fig|6666666.28482.peg.4557	Contig_55	143085	142666	-	Aspartate 1-decarboxylase (EC 4.1.1.11)	FIG00008304	icw(3);Coenzyme_A_Biosynthesis_cluster icw(1);Coenzyme_A_Biosynthesis
fig|6666666.28482.peg.4558	Contig_55	144014	143085	-	Pantoate--beta-alanine ligase (EC 6.3.2.1)	FIG00000440	isu;Coenzyme_A_Biosynthesis_cluster icw(2);Coenzyme_A_Biosynthesis

fig|6666666.28482.peg.4559	Contig_55	144922	144011	-	FIG173306: hypothetical protein	FIG00506717	icw(2);Coenzyme_A_Biosynthesis_cluster icw(3);Coenzyme_A_Biosynthesis
fig|6666666.28482.peg.4560	Contig_55	146284	145037	-	Possible membrane protein	FIG00819990	ff
fig|6666666.28482.peg.4561	Contig_55	146849	146373	-	FIG027937: secreted protein	FIG00996926	ff
fig|6666666.28482.peg.4562	Contig_55	147379	146849	-	2-amino-4-hydroxy-6-hydroxymethyldihydropteridine pyrophosphokinase (EC 2.7.6.3)	FIG00000171	icw(2);Folate_Biosynthesis
fig|6666666.28482.peg.4563	Contig_55	147789	147388	-	Dihydroneopterin aldolase (EC 4.1.2.25)	FIG00000373	icw(3);Folate_Biosynthesis
fig|6666666.28482.peg.4564	Contig_55	148591	147782	-	Dihydropteroate synthase (EC 2.5.1.15)	FIG00000307	isu;Folate_Biosynthesis
fig|6666666.28482.peg.4565	Contig_55	149235	148603	-	GTP cyclohydrolase I (EC 3.5.4.16) type 1	FIG00000226	icw(1);Folate_Biosynthesis isu;Molybdenum_cofactor_biosynthesis
fig|6666666.28482.peg.4566	Contig_55	151612	149252	-	Cell division protein FtsH (EC 3.4.24.-)	FIG00018369	idu(1);Bacterial_Cell_Division idu(1);Cell_division-ribosomal_stress_proteins_cluster
fig|6666666.28482.peg.4567	Contig_55	152182	151847	-	dioxygenase	FIG00825631	ff
fig|6666666.28482.peg.4568	Contig_55	152366	153292	+	Epoxide hydrolase (EC 3.3.2.9)	FIG00009455	idu(3);CBSS-410289.13.peg.3174
fig|6666666.28482.peg.4569	Contig_55	153292	154482	+	Limonene 1,2-monooxygenase	FIG01372910	ff
fig|6666666.28482.peg.4570	Contig_55	154482	155495	+	Sorbitol dehydrogenase (EC 1.1.1.14)	FIG00453259	ff

fig|6666666.28482.peg.4571	Contig_55	155831	155547	-	ESAT-6-like protein EsxL	FIG00820465	ff
fig|6666666.28482.peg.4572	Contig_55	156157	155861	-	FIG00824639: hypothetical protein	FIG00823510	ff
fig|6666666.28482.peg.4573	Contig_55	157354	156236	-	PPE family protein	FIG00013075	icw(1);CBSS-164757.7.peg.5020
fig|6666666.28482.peg.4574	Contig_55	158181	157384	-	PE family protein		
fig|6666666.28482.peg.4575	Contig_55	158364	158732	+	hypothetical protein		
fig|6666666.28482.peg.4576	Contig_55	158806	159096	+	FIG00825031: hypothetical protein	FIG00825029	ff
fig|6666666.28482.peg.4577	Contig_55	159609	159112	-	PPE family protein	FIG00013075	icw(1);CBSS-164757.7.peg.5020
fig|6666666.28482.peg.4578	Contig_56	1954	203	-	DNA repair protein RecN	FIG00000317	isu;DNA-replication isu;DNA_repair,_bacterial icw(2);CBSS-446462.5.peg.2987
fig|6666666.28482.peg.4579	Contig_56	2882	1962	-	NAD kinase (EC 2.7.1.23)	FIG00000075	isu;NAD_and_NADP_cofactor_biosynthesis_global isu;CBSS-446462.5.peg.2987
fig|6666666.28482.peg.4580	Contig_56	3688	2879	-	RNA binding methyltransferase FtsJ like	FIG00001125	icw(1);CBSS-446462.5.peg.2987
fig|6666666.28482.peg.4581	Contig_56	3882	3697	-	FIG00820006: hypothetical protein	FIG00820005	ff
fig|6666666.28482.peg.4582	Contig_56	4893	3895	-	4-nitrophenylphosphatase (EC 3.1.3.41)	FIG00672032	ff
fig|6666666.28482.peg.4583	Contig_56	5530	4895	-	TPR-repeat-containing protein	FIG01336235	ff
fig|6666666.28482.peg.4584	Contig_56	6161	5802	-	Lipoprotein LprJ	FIG01330511	ff
fig|6666666.28482.peg.4585	Contig_56	7479	6205	-	Tyrosyl-tRNA synthetase (EC 6.1.1.1)	FIG00346039	isu;tRNA_aminoacylation,_Tyr
fig|6666666.28482.peg.4586	Contig_56	7608	8483	+	transporter, bile acid/Na+ symporter family		

fig|6666666.28482.peg.4587	Contig_56	9070	8480	-	DNA-3-methyladenine glycosylase II (EC 3.2.2.21)	FIG00001134	isu;DNA_Repair_Base_Excision
fig|6666666.28482.peg.4588	Contig_56	9160	10422	+	Ammonium transporter	FIG00004764	idu(2);Ammonia_assimilation
fig|6666666.28482.peg.4589	Contig_56	11297	10431	-	Cyclopropane-fatty-acyl-phospholipid synthase (EC 2.1.1.79)	FIG00001555	ff
fig|6666666.28482.peg.4590	Contig_56	12110	11379	-	dienelactone hydrolase	FIG00814168	ff
fig|6666666.28482.peg.4591	Contig_56	14444	12180	-	hypothetical protein	FIG00638284	ff
fig|6666666.28482.peg.4592	Contig_56	14697	15461	+	ABC transporter ATP-binding protein	FIG01956052	ff
fig|6666666.28482.peg.4593	Contig_56	15545	16213	+	"ABC drug efflux pump, inner membrane subunit, DrrB family"	FIG01121530	ff
fig|6666666.28482.peg.4594	Contig_56	16215	16838	+	Transcriptional regulator, TetR family	FIG00554417	ff
fig|6666666.28482.peg.4595	Contig_56	17019	16804	-	FIG002473: Protein YcaR in KDO2-Lipid A biosynthesis cluster		isu;Broadly_distributed_proteins_not_in_subsystems
fig|6666666.28482.peg.4596	Contig_56	19988	17019	-	Polyhydroxyalkanoic acid synthase	FIG00000970	idu(1);Polyhydroxybutyrate_metabolism
fig|6666666.28482.peg.4597	Contig_56	20478	20104	-	hypothetical protein		
fig|6666666.28482.peg.4598	Contig_56	20964	21446	+	FIG00823740: hypothetical protein	FIG00823739	ff
fig|6666666.28482.peg.4599	Contig_56	21472	22215	+	FIG00455834: hypothetical protein	FIG00455833	ff

fig|6666666.28482.peg.4600	Contig_56	22215	23924	+	Glucose-methanol-choline (GMC) oxidoreductase:NAD binding site	FIG00001629	idu(3);Respiratory_dehydrogenases_1
fig|6666666.28482.peg.4601	Contig_56	23917	25707	+	FIG00830353: hypothetical protein	FIG00830350	ff
fig|6666666.28482.peg.4602	Contig_56	25727	26737	+	FIG00825543: hypothetical protein	FIG00825540	ff
fig|6666666.28482.peg.4603	Contig_56	26734	27186	+	hypothetical protein		
fig|6666666.28482.peg.4604	Contig_56	27196	28299	+	FIG00823456: hypothetical protein	FIG00823455	ff
fig|6666666.28482.peg.4605	Contig_56	28393	29103	+	FIG00831850: hypothetical protein	FIG00831847	ff
fig|6666666.28482.peg.4606	Contig_56	29165	30883	+	PROBABLE FIRST PART OF MACROLIDE-TRANSPORT ATP-BINDING PROTEIN ABC TRANSPORTER		
fig|6666666.28482.peg.4607	Contig_56	30891	32198	+	Cytochrome P450 139	FIG01346688	ff
fig|6666666.28482.peg.4608	Contig_57	91	1857	+	hypothetical protein	FIG00638284	ff
fig|6666666.28482.peg.4609	Contig_57	4447	1880	-	hypothetical protein	FIG00638284	ff
fig|6666666.28482.peg.4610	Contig_57	4677	6635	+	PE-PGRS FAMILY PROTEIN		
fig|6666666.28482.peg.4611	Contig_57	7684	6671	-	Alcohol dehydrogenase (EC 1.1.1.1)	FIG01007502	idu(17);Glycerolipid_and_Glycerophospholipid_Metabolism_in_Bacteria idu(17);Fermentations:_Mixed_acid idu(17);Butanol_Biosynthesis
fig|6666666.28482.peg.4612	Contig_57	7803	8237	+	hypothetical protein		
fig|6666666.28482.peg.4613	Contig_57	8234	9052	+	hypothetical protein		
fig|6666666.28482.peg.4614	Contig_57	9201	9464	+	hypothetical protein		

fig|6666666.28482.peg.4615	Contig_57	9489	10361	+	D-alanyl-D-alanine carboxypeptidase (EC 3.4.16.4)	FIG00561498	idu(3);Murein_Hydrolases idu(3);CBSS-84588.1.peg.1247 idu(3);Metallocarboxypeptidases_(EC _3.4.17.-) idu(3);Peptidoglycan_Biosynthesis
fig|6666666.28482.peg.4616	Contig_57	10981	10379	-	Transcriptional regulator, TetR family	FIG00554417	ff
fig|6666666.28482.peg.4617	Contig_57	12776	10983	-	N-acyl-D-glutamate amidohydrolase	FIG00820355	ff
fig|6666666.28482.peg.4618	Contig_57	14423	12882	-	hypothetical protein	FIG00638284	ff
fig|6666666.28482.peg.4619	Contig_57	15543	14461	-	Aminopeptidase YpdF (MP-, MA-, MS-, AP-, NP-specific)	FIG00135469	idu(1);Protein_degradation
fig|6666666.28482.peg.4620	Contig_57	17107	15551	-	Signal recognition particle, subunit Ffh SRP54 (TC 3.A.5.1.1)	FIG00000113	isu;Bacterial_signal_recognition_particle_(SRP)
fig|6666666.28482.peg.4621	Contig_57	17120	17992	+	FIG00825520: hypothetical protein	FIG00825518	ff
fig|6666666.28482.peg.4622	Contig_57	18186	19883	+	DNA or RNA helicase of superfamily protein II	FIG00821286	ff
fig|6666666.28482.peg.4623	Contig_58	295	1296	+	NAD binding oxidoreductase	FIG01609121	ff
fig|6666666.28482.peg.4624	Contig_58	2096	1302	-	putative hydrolase		
fig|6666666.28482.peg.4625	Contig_58	2304	3686	+	UDP-glucose 6-dehydrogenase(EC:1.1.1.22)		
fig|6666666.28482.peg.4626	Contig_58	3736	4731	+	dTDP-glucose 4,6-dehydratase (EC 4.2.1.46)	FIG00030688	idu(4);dTDP-rhamnose_synthesis idu(4);Rhamnose_containing_glycans
fig|6666666.28482.peg.4627	Contig_58	6923	4728	-	O-antigen acetylase	FIG01370327	ff

fig|6666666.28482.peg.4628	Contig_58	7080	8348	+	GtfA	FIG01525338	ff
fig|6666666.28482.peg.4629	Contig_58	10463	8358	-	O-antigen acetylase	FIG01370327	ff
fig|6666666.28482.peg.4630	Contig_58	11743	10670	-	NAD-dependent epimerase/dehydratase family protein	FIG00143216	ff
fig|6666666.28482.peg.4631	Contig_58	14112	11893	-	O-antigen acetylase	FIG01370327	ff
fig|6666666.28482.peg.4632	Contig_58	14262	15395	+	MtfA protein	FIG01419689	ff
fig|6666666.28482.peg.4633	Contig_58	15414	16682	+	GtfA	FIG01525338	ff
fig|6666666.28482.peg.4634	Contig_58	16740	17975	+	glycosyltransferase B		
fig|6666666.28482.peg.4635	Contig_58	18073	18204	+	hypothetical protein		
fig|6666666.28482.peg.4636	Contig_58	18261	29198	+	Malonyl CoA-acyl carrier protein transacylase (EC 2.3.1.39)	FIG00000319	idu(7);Fatty_Acid_Biosynthesis_FASII
fig|6666666.28482.peg.4637	Contig_58	29673	30263	+	hypothetical protein		
fig|6666666.28482.peg.4638	Contig_58	31084	30278	-	Probable methyltransferase (EC 2.1.1.-)		
fig|6666666.28482.peg.4639	Contig_58	31877	32107	+	MbtH protein	FIG01223139	ff
fig|6666666.28482.peg.4640	Contig_58	32113	36678	+	Long-chain-fatty-acid--CoA ligase (EC 6.2.1.3)	FIG00018699	idu(72);n-Phenylalkanoic_acid_degradation idu(72);Fatty_acid_metabolism_cluster idu(72);Biotin_synthesis_cluster idu(72);Biotin_biosynthesis
fig|6666666.28482.peg.4641	Contig_58	36683	49300	+	Long-chain-fatty-acid--CoA ligase (EC 6.2.1.3)	FIG00018699	idu(72);n-Phenylalkanoic_acid_degradation idu(72);Fatty_acid_metabolism_cluster idu(72);Biotin_synthesis_cluster idu(72);Biotin_biosynthesis

fig|6666666.28482.peg.4642	Contig_58	49999	49325	-	isoprenylcysteine carboxyl methyltransferase (icmt) family protein		
fig|6666666.28482.peg.4643	Contig_58	50455	50177	-	hypothetical protein		
fig|6666666.28482.peg.4644	Contig_59	4	576	+	PE family protein		
fig|6666666.28482.peg.4645	Contig_59	694	2568	+	PE-PGRS family protein		idu(16);PPE_gene_cluster_in_Mycobacteria
fig|6666666.28482.peg.4646	Contig_59	2999	2580	-	hypothetical membrane protein		
fig|6666666.28482.peg.4647	Contig_59	3922	2996	-	FIG00828022: hypothetical protein	FIG00828021	ff
fig|6666666.28482.peg.4648	Contig_59	4389	3919	-	FIG00826545: hypothetical protein	FIG00826544	ff
fig|6666666.28482.peg.4649	Contig_59	5100	4411	-	FIG00823447: hypothetical protein	FIG00823443	ff
fig|6666666.28482.peg.4650	Contig_59	5654	5097	-	RNA polymerase sigma factor	FIG00014368	ff
fig|6666666.28482.peg.4651	Contig_59	7102	5672	-	FIG00825309: hypothetical protein	FIG00825308	ff
fig|6666666.28482.peg.4652	Contig_59	7707	7135	-	Transcriptional regulator, TetR family	FIG00554417	ff
fig|6666666.28482.peg.4653	Contig_59	7792	8670	+	hypothetical protein		
fig|6666666.28482.peg.4654	Contig_59	8748	10268	+	Methylmalonate-semialdehyde dehydrogenase (EC 1.2.1.27)	FIG00137372	icw(2);Valine_degradation

fig|6666666.28482.peg.4655	Contig_59	10278	11438	+	Butyryl-CoA dehydrogenase (EC 1.3.99.2)	FIG00009149	idu(39);Isoleucine_degradation idu(39);Lysine_fermentation idu(39);Butanol_Biosynthesis idu(39);Acetyl-CoA_fermentation_to_Butyrate idu(39);Valine_degradation idu(39);Anaerobic_respiratory_reductases
fig|6666666.28482.peg.4656	Contig_59	11451	12326	+	3-hydroxyisobutyrate dehydrogenase (EC 1.1.1.31)	FIG00021583	icw(1);Valine_degradation
fig|6666666.28482.peg.4657	Contig_59	12901	12353	-	FIG00822571: hypothetical protein	FIG00822570	ff
fig|6666666.28482.peg.4658	Contig_59	13427	12924	-	Transcriptional regulator, MarR family	FIG00554610	ff
fig|6666666.28482.peg.4659	Contig_59	14488	13436	-	Biosynthetic Aromatic amino acid aminotransferase beta (EC 2.6.1.57)	FIG00032670	idu(1);Phenylalanine_and_Tyrosine_Branches_from_Chorismate
fig|6666666.28482.peg.4660	Contig_59	15507	14488	-	3-oxoacyl-[acyl-carrier-protein] synthase, KASIII (EC 2.3.1.41)	FIG00015100	idu(2);Fatty_Acid_Biosynthesis_FASII
fig|6666666.28482.peg.4661	Contig_59	16158	15511	-	FIG00820173: hypothetical protein	FIG00820172	ff
fig|6666666.28482.peg.4662	Contig_59	17839	16136	-	COG0028: Thiamine pyrophosphate-requiring enzymes	FIG00452446	ff
fig|6666666.28482.peg.4663	Contig_59	18687	17917	-	Possible membrane protein	FIG01121868	ff
fig|6666666.28482.peg.4664	Contig_59	19225	18734	-	RNA polymerase sigma-70 factor	FIG01303902	idu(4);CBSS-313593.3.peg.2729 idu(4);Transcription_initiation,_bacterial_sigma_factors

fig|6666666.28482.peg.4665	Contig_59	20128	19328	-	Methionine aminopeptidase (EC 3.4.11.18)	FIG00000036	idu(1);CBSS-312309.3.peg.1965 idu(1);Translation_termination_factors_bacterial
fig|6666666.28482.peg.4666	Contig_59	20670	20131	-	Adenylate kinase (EC 2.7.4.3)	FIG00000089	isu;Purine_conversions
fig|6666666.28482.peg.4667	Contig_59	21998	20673	-	Preprotein translocase secY subunit (TC 3.A.5.1.1)	FIG00937056	ff
fig|6666666.28482.peg.4668	Contig_59	22175	23080	+	O-Methyltransferase involved in polyketide biosynthesis	FIG01313020	ff
fig|6666666.28482.peg.4669	Contig_59	23759	23088	-	FIG00820922: hypothetical protein	FIG00820921	ff
fig|6666666.28482.peg.4670	Contig_59	25087	23750	-	Putative xylulose kinase (EC 2.7.1.17)	FIG00229240	isu;Xylose_utilization
fig|6666666.28482.peg.4671	Contig_59	25142	26098	+	D-3-phosphoglycerate dehydrogenase (EC 1.1.1.95)	FIG00031250	idu(4);Serine_Biosynthesis idu(4);Pyridoxin_(Vitamin_B6)_Biosynthesis
fig|6666666.28482.peg.4672	Contig_59	26095	26748	+	L-fuculose phosphate aldolase (EC 4.1.2.17)	FIG00002486	isu;L-fucose_utilization_temp isu;L-fucose_utilization
fig|6666666.28482.peg.4673	Contig_59	26749	27633	+	putative beta-lactamase	FIG01548503	ff
fig|6666666.28482.peg.4674	Contig_59	27637	28671	+	hypothetical protein		
fig|6666666.28482.peg.4675	Contig_59	29517	28660	-	Transcriptional regulator, AraC family	FIG00510130	ff
fig|6666666.28482.peg.4676	Contig_59	29583	30239	+	Methyltransferase Sare_0198	FIG01598253	ff
fig|6666666.28482.peg.4677	Contig_59	31450	30236	-	Adh	FIG01491610	ff
fig|6666666.28482.peg.4678	Contig_59	32235	31447	-	short-chain dehydrogenase Adh_1	FIG00825109	ff
fig|6666666.28482.peg.4679	Contig_59	32333	33277	+	FIG00824343: hypothetical protein	FIG00824342	ff

fig|6666666.28482.peg.4680	Contig_59	33318	34178	+	O-Methyltransferase involved in polyketide biosynthesis	FIG01313020	ff
fig|6666666.28482.peg.4681	Contig_59	34231	35100	+	FIG00824343: hypothetical protein	FIG00824342	ff
fig|6666666.28482.peg.4682	Contig_59	36872	35097	-	Possible protease IV SppA (endopeptidase IV) (signal peptide peptidase)	FIG01320210	ff
fig|6666666.28482.peg.4683	Contig_59	36911	37837	+	Luciferase-like monooxygenase (EC 1.14.-.-)	FIG00554275	ff
fig|6666666.28482.peg.4684	Contig_59	38278	37838	-	LSU ribosomal protein L15p (L27Ae)	FIG00000166	icw(6);Ribosome_LSU_bacterial
fig|6666666.28482.peg.4685	Contig_59	38457	38275	-	LSU ribosomal protein L30p (L7e)	FIG00000409	icw(5);Ribosome_LSU_bacterial
fig|6666666.28482.peg.4686	Contig_59	39149	38454	-	SSU ribosomal protein S5p (S2e)	FIG00000155	isu;Ribosomal_protein_S5p_acylation
fig|6666666.28482.peg.4687	Contig_59	39597	39190	-	LSU ribosomal protein L18p (L5e)	FIG00000200	isu;Ribosome_LSU_bacterial
fig|6666666.28482.peg.4688	Contig_59	40139	39600	-	LSU ribosomal protein L6p (L9e)	FIG00001577	icw(3);Ribosome_LSU_bacterial
fig|6666666.28482.peg.4689	Contig_59	40549	40151	-	SSU ribosomal protein S8p (S15Ae)	FIG00000153	ff
fig|6666666.28482.peg.4690	Contig_59	40754	40569	-	SSU ribosomal protein S14p (S29e) @ SSU ribosomal protein S14p (S29e), zinc-dependent		
fig|6666666.28482.peg.4691	Contig_59	41322	40759	-	LSU ribosomal protein L5p (L11e)	FIG00000174	icw(1);Ribosome_LSU_bacterial
fig|6666666.28482.peg.4692	Contig_59	41639	41322	-	LSU ribosomal protein L24p (L26e)	FIG00000198	icw(4);Ribosome_LSU_bacterial

fig|6666666.28482.peg.4693	Contig_59	42008	41640	-	LSU ribosomal protein L14p (L23e)	FIG00090781	icw(2);Ribosome_LSU_bacterial
fig|6666666.28482.peg.4694	Contig_59	43722	42205	-	13E12 repeat family protein	FIG00831556	ff
fig|6666666.28482.peg.4695	Contig_59	44848	43886	-	Possible membrane protein	FIG01121868	ff
fig|6666666.28482.peg.4696	Contig_59	45967	44966	-	Possible membrane protein	FIG01121868	ff
fig|6666666.28482.peg.4697	Contig_59	47010	46120	-	Sulfatase modifying factor 1 precursor (C-alpha-formyglycine-generating enzyme 1)	FIG00003489	idu(1);Sulfatases_and_sulfatase_modifying_factor_1
fig|6666666.28482.peg.4698	Contig_59	47985	47044	-	Possible membrane protein	FIG01121868	ff
fig|6666666.28482.peg.4699	Contig_59	48283	49410	+	Phosphate ABC transporter, periplasmic phosphate-binding protein PstS (TC 3.A.1.7.1)	FIG00010508	idu(4);High_affinity_phosphate_transporter_and_control_of_PHO_regulon idu(4);Phosphate_metabolism idu(4);PhoR-PhoB_two-component_regulatory_system
fig|6666666.28482.peg.4700	Contig_59	50341	49415	-	hypothetical protein		
fig|6666666.28482.peg.4701	Contig_59	52840	50480	-	Arylsulfatase (EC 3.1.6.1)	FIG00016566	idu(3);Sulfatases_and_sulfatase_modifying_factor_1 idu(3);Galactosylceramide_and_Sulfatide_metabolism idu(3);Steroid_sulfates idu(3);Alkanesulfonate_assimilation
fig|6666666.28482.peg.4702	Contig_59	53515	52886	-	membrane protein, putative		
fig|6666666.28482.peg.4703	Contig_59	53594	54301	+	Transcriptional regulator, TetR family	FIG00554417	ff

fig|6666666.28482.peg.4704	Contig_59	54670	54308	-	SSU ribosomal protein S17p (S11e)	FIG00000181	ff
fig|6666666.28482.peg.4705	Contig_59	54912	54667	-	LSU ribosomal protein L29p (L35e)	FIG00015729	icw(2);Ribosome_LSU_bacterial
fig|6666666.28482.peg.4706	Contig_59	55328	54912	-	LSU ribosomal protein L16p (L10e)	FIG00001132	icw(3);Ribosome_LSU_bacterial
fig|6666666.28482.peg.4707	Contig_59	56168	55332	-	SSU ribosomal protein S3p (S3e)	FIG00000218	ff
fig|6666666.28482.peg.4708	Contig_59	56725	56168	-	LSU ribosomal protein L22p (L17e)	FIG00000219	icw(5);Ribosome_LSU_bacterial
fig|6666666.28482.peg.4709	Contig_59	57003	56722	-	SSU ribosomal protein S19p (S15e)	FIG00000199	ff
fig|6666666.28482.peg.4710	Contig_59	57861	57019	-	LSU ribosomal protein L2p (L8e)	FIG00000223	icw(1);Ribosome_LSU_bacterial
fig|6666666.28482.peg.4711	Contig_59	58180	57878	-	LSU ribosomal protein L23p (L23Ae)	FIG00000271	isu;Ribosome_LSU_bacterial
fig|6666666.28482.peg.4712	Contig_59	58845	58180	-	LSU ribosomal protein L4p (L1e)	FIG00000263	icw(6);Ribosome_LSU_bacterial
fig|6666666.28482.peg.4713	Contig_59	59519	58845	-	LSU ribosomal protein L3p (L3e)	FIG00001818	icw(4);Ribosome_LSU_bacterial
fig|6666666.28482.peg.4714	Contig_59	59839	59534	-	SSU ribosomal protein S10p (S20e)	FIG00001107	ff
fig|6666666.28482.peg.4715	Contig_59	60655	60170	-	Transcriptional regulator, TetR family	FIG00554417	ff
fig|6666666.28482.peg.4716	Contig_59	61024	62541	+	Integral membrane protein	FIG01956050	ff
fig|6666666.28482.peg.4717	Contig_59	62667	62819	+	hypothetical protein		
fig|6666666.28482.peg.4718	Contig_6	47	268	+	Phosphoenolpyruvate synthase (EC 2.7.9.2)	FIG00000789	idu(2);Pyruvate_metabolism_I:_anaplerotic_reactions,_PEP
fig|6666666.28482.peg.4719	Contig_6	1000	275	-	FIG003003: hypothetical protein	FIG00634249	ff
fig|6666666.28482.peg.4720	Contig_6	1643	1008	-	FIG00820353: hypothetical protein	FIG00820351	ff

fig|6666666.28482.peg.4721	Contig_6	2019	1687	-	FIG00825063: hypothetical protein	FIG00825062	ff
fig|6666666.28482.peg.4722	Contig_6	2442	2131	-	FIG00820081: hypothetical protein	FIG00820079	ff
fig|6666666.28482.peg.4723	Contig_6	3109	2618	-	hypothetical protein		
fig|6666666.28482.peg.4724	Contig_6	5639	3327	-	ATP-dependent protease La (EC 3.4.21.53) Type I	FIG00132617	isu;Proteolysis_in_bacteria,_ATP-dependent
fig|6666666.28482.peg.4725	Contig_6	6981	5755	-	L-lactate dehydrogenase (EC 1.1.2.3)	FIG00121995	isu;Respiratory_dehydrogenases_1 isu;Lactate_utilization isu;L-rhamnose_utilization
fig|6666666.28482.peg.4726	Contig_6	7016	7441	+	NTP pyrophosphohydrolases including oxidative damage repair enzymes	FIG00004782	idu(1);CBSS-216591.1.peg.168
fig|6666666.28482.peg.4727	Contig_6	8713	7427	-	Neopullulanase (EC 3.2.1.135)	FIG00066643	isu;Maltose_and_Maltodextrin_Utilization
fig|6666666.28482.peg.4728	Contig_6	9253	8717	-	Alkylhydroperoxidase protein D	FIG00003720	icw(1);Thioredoxin-disulfide_reductase
fig|6666666.28482.peg.4729	Contig_6	9851	9264	-	Alkyl hydroperoxide reductase protein C (EC 1.6.4.-)	FIG00000710	icw(2);Thioredoxin-disulfide_reductase
fig|6666666.28482.peg.4730	Contig_6	9926	10897	+	Hydrogen peroxide-inducible genes activator	FIG00031825	idu(1);Oxidative_stress idu(1);LysR-family_proteins_in_Salmonella_enterica_Typhimurium idu(1);LysR-family_proteins_in_Escherichia_coli idu(1);Thioredoxin-disulfide_reductase
fig|6666666.28482.peg.4731	Contig_6	10924	11625	+	FIG00820973: hypothetical protein	FIG00820972	ff

fig|6666666.28482.peg.4732	Contig_6	11634	12077	+	Pyridoxine 5'-phosphate oxidase	FIG01381952	ff
fig|6666666.28482.peg.4733	Contig_6	12100	12849	+	Enoyl-CoA hydratase (EC 4.2.1.17)	FIG00033206	idu(51);n-Phenylalkanoic_acid_degradation idu(51);Polyhydroxybutyrate_metabolism idu(51);Isoleucine_degradation idu(51);Fatty_acid_metabolism_cluster idu(51);Lysine_fermentation idu(51);Butanol_Biosynthesis idu(51);Acetyl-CoA_fermentation_to_Butyrate idu(51);Valine_degradation idu(51);Butyrate_metabolism_cluster
fig|6666666.28482.peg.4734	Contig_6	13033	12854	-	FIG00820183: hypothetical protein	FIG00820182	ff
fig|6666666.28482.peg.4735	Contig_6	13273	13752	+	Bacterioferritin (BFR)	FIG01312040	ff
fig|6666666.28482.peg.4736	Contig_6	13830	15926	+	Multidrug resistance protein B	FIG01356213	ff
fig|6666666.28482.peg.4737	Contig_6	15923	16417	+	FIG00824327: hypothetical protein	FIG00824325	ff
fig|6666666.28482.peg.4738	Contig_6	16518	17399	+	FIG00828228: hypothetical protein	FIG00828224	ff
fig|6666666.28482.peg.4739	Contig_6	17413	18774	+	Glutamine synthetase (EC 6.3.1.2), putative	FIG01314524	ff
fig|6666666.28482.peg.4740	Contig_6	18771	19871	+	Amidohydrolase	FIG01311586	ff
fig|6666666.28482.peg.4741	Contig_6	19902	20735	+	acyltransferase family protein	FIG01250595	ff
fig|6666666.28482.peg.4742	Contig_6	20735	21169	+	cyclase/dehydrase		
fig|6666666.28482.peg.4743	Contig_6	21409	21531	+	hypothetical protein		

fig|6666666.28482.peg.4744	Contig_6	22902	21562	-	FIG00995345: hypothetical protein	FIG00995340	ff
fig|6666666.28482.peg.4745	Contig_6	23020	24408	+	PPE family protein	FIG00013075	icw(2);CBSS-164757.7.peg.5020
fig|6666666.28482.peg.4746	Contig_6	24405	25781	+	PPE family protein	FIG00013075	icw(2);CBSS-164757.7.peg.5020
fig|6666666.28482.peg.4747	Contig_6	26539	25778	-	Ubiquinone/menaquinone biosynthesis methyltransferase UbiE (EC 2.1.1.-)		idu(2);Menaquinone_and_Phylloquinone_Biosynthesis idu(2);Menaquinone_Biosynthesis_via_Futalosine idu(2);Ubiquinone_Biosynthesis
fig|6666666.28482.peg.4748	Contig_6	26682	26885	+	hypothetical protein		
fig|6666666.28482.peg.4749	Contig_6	27127	28605	+	PPE family protein	FIG00013075	icw(2);CBSS-164757.7.peg.5020
fig|6666666.28482.peg.4750	Contig_6	29082	28624	-	FIG00823935: hypothetical protein	FIG00823934	ff
fig|6666666.28482.peg.4751	Contig_6	29146	30054	+	Dienelactone hydrolase family	FIG01069253	ff
fig|6666666.28482.peg.4752	Contig_6	30051	30413	+	hypothetical protein		
fig|6666666.28482.peg.4753	Contig_6	30475	30657	+	hypothetical protein		
fig|6666666.28482.peg.4754	Contig_6	32042	30717	-	Cytochrome P450 140	FIG01337186	ff
fig|6666666.28482.peg.4755	Contig_6	32520	32101	-	Lipoprotein LppE	FIG01328245	ff
fig|6666666.28482.peg.4756	Contig_6	33367	32534	-	Probable short-chain type dehydrogenase/reductase (EC 1.1.-.-)	FIG00831186	ff
fig|6666666.28482.peg.4757	Contig_6	33805	33374	-	FIG00820653: hypothetical protein	FIG00820652	ff
fig|6666666.28482.peg.4758	Contig_6	34298	33879	-	Resuscitation-promoting factor RpfC	FIG01310076	ff
fig|6666666.28482.peg.4759	Contig_6	35014	34421	-	Periplasmic chorismate mutase I precursor (EC 5.4.99.5)	FIG00132814	isu;Chorismate_Synthesis isu;Phenylalanine_and_Tyrosine_Branches_from_Chorismate
fig|6666666.28482.peg.4760	Contig_6	35060	35632	+	FIG00822912: hypothetical protein	FIG00822909	ff

fig|6666666.28482.peg.4761	Contig_6	36674	35694	-	Esterase, secreted fibronectin-binding protein antigen 85-B FbpB	FIG00820332	ff
fig|6666666.28482.peg.4762	Contig_6	36815	36943	+	hypothetical protein		
fig|6666666.28482.peg.4763	Contig_6	37399	38526	+	Possible membrane protein	FIG01121868	ff
fig|6666666.28482.peg.4764	Contig_6	39089	38541	-	FIG00820863: hypothetical protein	FIG00820861	ff
fig|6666666.28482.peg.4765	Contig_6	40116	39103	-	Alcohol dehydrogenase (EC 1.1.1.1)	FIG01007502	idu(17);Glycerolipid_and_Glycerophospholipid_Metabolism_in_Bacteria idu(17);Fermentations:_Mixed_acid idu(17);Butanol_Biosynthesis
fig|6666666.28482.peg.4766	Contig_60	121	504	+	hypothetical protein		
fig|6666666.28482.peg.4767	Contig_60	610	1551	+	Alcohol dehydrogenase GroES domain protein	FIG00615601	ff
fig|6666666.28482.peg.4768	Contig_60	1626	2402	+	putative short-chain dehydrogenase		
fig|6666666.28482.peg.4769	Contig_60	2456	2971	+	Transcriptional regulator, MarR family	FIG00554610	ff
fig|6666666.28482.peg.4770	Contig_60	3305	3679	+	FIG00826668: hypothetical protein		
fig|6666666.28482.peg.4771	Contig_60	3777	5996	+	O-antigen acetylase	FIG01370327	ff
fig|6666666.28482.peg.4772	Contig_60	6301	6011	-	hypothetical protein		
fig|6666666.28482.peg.4773	Contig_60	6705	6316	-	exported protein		
fig|6666666.28482.peg.4774	Contig_60	6838	7275	+	hypothetical protein		
fig|6666666.28482.peg.4775	Contig_60	7378	8316	+	Phosphate transport system regulatory protein PhoU	FIG00104848	idu(3);High_affinity_phosphate_transporter_and_control_of_PHO_regulon idu(3);Phosphate_metabolism

fig|6666666.28482.peg.4776	Contig_60	8420	9574	+	FIG00830848: hypothetical protein	FIG00830846	ff
fig|6666666.28482.peg.4777	Contig_60	9686	9898	+	hypothetical protein		
fig|6666666.28482.peg.4778	Contig_60	10026	11003	+	hypothetical protein	FIG00638284	ff
fig|6666666.28482.peg.4779	Contig_60	11006	12145	+	Ferredoxin	FIG00459778	idu(4);Soluble_cytochromes_and_functionally_related_electron_carriers idu(4);Inorganic_Sulfur_Assimilation
fig|6666666.28482.peg.4780	Contig_60	12168	12983	+	2-polyprenylphenol hydroxylase and related flavodoxin oxidoreductases	FIG00460593	isu;Central_meta-cleavage_pathway_of_aromatic_compound_degradation
fig|6666666.28482.peg.4781	Contig_60	12984	13742	+	NAD-reducing hydrogenase subunit HoxY (EC 1.12.1.2)	FIG00006962	icw(1);Hydrogenases
fig|6666666.28482.peg.4782	Contig_60	13739	15031	+	Cytochrome-C3 hydrogenase alpha chain	FIG00995412	ff
fig|6666666.28482.peg.4783	Contig_60	15028	15507	+	Ni,Fe-hydrogenase maturation factor	FIG00054664	isu;Hydrogenases
fig|6666666.28482.peg.4784	Contig_60	15512	16921	+	Putative membrane protein MT1655	FIG01531367	ff
fig|6666666.28482.peg.4785	Contig_60	17357	16923	-	pyridoxamine 5'-phosphate oxidase family protein		
fig|6666666.28482.peg.4786	Contig_60	18432	17419	-	Oxygen-insensitive NADPH nitroreductase (EC 1.-.-.-)	FIG00904859	ff

fig|6666666.28482.peg.4787	Contig_60	18763	18509	-	Acyl carrier protein	FIG00002328	idu(2);Fatty_Acid_Biosynthesis_FASII idu(2);Glycerolipid_and_Glycerophospholipid_Metabolism_in_Bacteria
fig|6666666.28482.peg.4788	Contig_60	20008	18779	-	Dihydrolipoamide acetyltransferase component (E2) of acetoin dehydrogenase complex (EC 2.3.1.-)	FIG00001753	isu;Acetoin,_butanediol_metabolism icw(2);Dehydrogenase_complexes
fig|6666666.28482.peg.4789	Contig_60	20994	20005	-	Acetoin dehydrogenase E1 component beta-subunit (EC 1.2.4.-)	FIG01270039	icw(1);Acetoin,_butanediol_metabolism idu(1);Dehydrogenase_complexes
fig|6666666.28482.peg.4790	Contig_60	22022	20991	-	Acetoin dehydrogenase E1 component alpha-subunit (EC 1.2.4.-)	FIG00138523	icw(2);Acetoin,_butanediol_metabolism icw(1);Dehydrogenase_complexes
fig|6666666.28482.peg.4791	Contig_60	23830	22019	-	Acetyl-coenzyme A synthetase (EC 6.2.1.1)	FIG00022631	idu(2);Pyruvate_metabolism_II:_acetyl-CoA,_acetogenesis_from_pyruvate idu(2);Ketoisovalerate_oxidoreductase
fig|6666666.28482.peg.4792	Contig_60	24017	25003	+	hypothetical protein		
fig|6666666.28482.peg.4793	Contig_60	25013	25294	+	hypothetical protein		
fig|6666666.28482.peg.4794	Contig_60	25296	25538	+	hypothetical protein		
fig|6666666.28482.peg.4795	Contig_60	25681	26673	+	Tagatose-6-phosphate kinase (EC 2.7.1.144) / 1-phosphofructokinase (EC 2.7.1.56)	FIG00460883	isu;Fructose_utilization isu;Lactose_and_Galactose_Uptake_and_Utilization isu;D-Tagatose_and_Galactitol_Utilization

fig|6666666.28482.peg.4796	Contig_60	26741	27529	+	Universal stress protein family	FIG00017615	ff
fig|6666666.28482.peg.4797	Contig_60	27540	28376	+	Universal stress protein family		
fig|6666666.28482.peg.4798	Contig_60	28436	29107	+	Two component transcriptional regulatory protein DevR	FIG01330944	ff
fig|6666666.28482.peg.4799	Contig_60	29278	30996	+	Two-component sensor histidine kinase	FIG00821297	ff
fig|6666666.28482.peg.4800	Contig_60	31055	31603	+	GCN5-related N-acetyltransferase		
fig|6666666.28482.peg.4801	Contig_60	31591	32478	+	Universal stress protein family	FIG00017615	ff
fig|6666666.28482.peg.4802	Contig_60	32475	32849	+	hypothetical protein		
fig|6666666.28482.peg.4803	Contig_60	32900	34288	+	Partial REP13E12 repeat protein	FIG00820827	ff
fig|6666666.28482.peg.4804	Contig_60	35097	34291	-	Universal stress protein family	FIG00017615	ff
fig|6666666.28482.peg.4805	Contig_60	38873	35202	-	Trehalose-6-phosphate phosphatase (EC 3.1.3.12)	FIG00001349	idu(1);Trehalose_Biosynthesis
fig|6666666.28482.peg.4806	Contig_60	39142	38936	-	hypothetical protein		
fig|6666666.28482.peg.4807	Contig_60	39006	39893	+	Universal stress protein family	FIG00017615	ff
fig|6666666.28482.peg.4808	Contig_60	39932	41395	+	FIG00828840: hypothetical protein	FIG00828836	ff
fig|6666666.28482.peg.4809	Contig_60	41506	42510	+	Oxygen-insensitive NADPH nitroreductase (EC 1.-.-.-)	FIG00904859	ff

fig|6666666.28482.peg.4810	Contig_60	42526	43629	+	3-oxoacyl-[acyl-carrier-protein] synthase, KASIII (EC 2.3.1.41)	FIG00015100	icw(1);Fatty_Acid_Biosynthesis_FASII
fig|6666666.28482.peg.4811	Contig_60	43613	44494	+	3-oxoacyl-[acyl-carrier-protein] synthase, KASIII (EC 2.3.1.41)	FIG00015100	icw(1);Fatty_Acid_Biosynthesis_FASII
fig|6666666.28482.peg.4812	Contig_60	46984	44588	-	Phosphoenolpyruvate synthase (EC 2.7.9.2)	FIG00000789	idu(2);Pyruvate_metabolism_I:_anaplerotic_reactions,_PEP
fig|6666666.28482.peg.4813	Contig_60	47093	48472	+	Wax ester synthase/acyl-CoA:diacylglycerol acyltransferase	FIG01309728	ff
fig|6666666.28482.peg.4814	Contig_60	49145	48462	-	FIG00827884: hypothetical protein	FIG00827881	ff
fig|6666666.28482.peg.4815	Contig_60	49678	49220	-	18 kDa antigen	FIG01612281	ff
fig|6666666.28482.peg.4816	Contig_60	49825	50202	+	HspR	FIG01507885	ff
fig|6666666.28482.peg.4817	Contig_60	50374	51090	+	FIG00824452: hypothetical protein	FIG00824450	ff
fig|6666666.28482.peg.4818	Contig_60	51136	52323	+	FIG00826673: hypothetical protein	FIG00826669	ff
fig|6666666.28482.peg.4819	Contig_60	52393	54543	+	Glycogen debranching enzyme (EC 3.2.1.-)	FIG00109688	idu(3);Trehalose_Biosynthesis idu(3);Glycogen_metabolism
fig|6666666.28482.peg.4820	Contig_60	54636	55406	+	Short chain dehydrogenase	FIG01310822	ff
fig|6666666.28482.peg.4821	Contig_60	56380	55403	-	aldo/keto reductase	FIG01122489	ff
fig|6666666.28482.peg.4822	Contig_60	57389	56394	-	PknJ		
fig|6666666.28482.peg.4823	Contig_60	57489	58037	+	hypothetical protein		
fig|6666666.28482.peg.4824	Contig_60	58492	59052	+	FIG00823781: hypothetical protein	FIG00823780	ff
fig|6666666.28482.peg.4825	Contig_60	59067	59720	+	Uracil-DNA glycosylase, putative family 6	FIG00025538	isu;Uracil-DNA_glycosylase

fig|6666666.28482.peg.4826	Contig_60	61528	59735	-	pyruvate decarboxylase	FIG00957120	ff
fig|6666666.28482.peg.4827	Contig_60	61664	61521	-	hypothetical protein		
fig|6666666.28482.peg.4828	Contig_60	62716	61739	-	ADP-heptose--lipooligosaccharide heptosyltransferase II (EC 2.4.1.-)	FIG00013366	icw(1);LOS_core_oligosaccharide_biosynthesis
fig|6666666.28482.peg.4829	Contig_60	63411	62707	-	FIG00825699: hypothetical protein	FIG00825697	ff
fig|6666666.28482.peg.4830	Contig_60	63920	63423	-	ADP-heptose synthase (EC 2.7.-.-) / D-glycero-beta-D-manno-heptose 7-phosphate kinase		isu;LOS_core_oligosaccharide_biosynthesis icw(1);LOS_core_oligosaccharide_biosynthesis
fig|6666666.28482.rna.45	Contig_61	582	509	-	tRNA-Pro-GGG		isu;tRNAs
fig|6666666.28482.peg.4831	Contig_61	1389	643	-	Transcriptional regulator, IclR family	FIG00022569	idu(12);Homogentisate_pathway_of_aromatic_compound_degradation
fig|6666666.28482.peg.4832	Contig_61	2216	1386	-	Kynurenine formamidase, bacterial (EC 3.5.1.9)	FIG00001524	isu;NAD_and_NADP_cofactor_biosynthesis_global isu;Aromatic_amino_acid_degradation
fig|6666666.28482.peg.4833	Contig_61	2988	2230	-	Probable oxidoreductase (EC 1.-.-.-)		
fig|6666666.28482.peg.4834	Contig_61	4717	3317	-	GTP-binding protein EngA	FIG00129616	isu;Ribosome_post-transcriptional_modification_and_chromosomal_segregation_cluster isu;CBSS-290633.1.peg.1906
fig|6666666.28482.peg.4835	Contig_61	5388	4714	-	Cytidylate kinase (EC 2.7.4.14)	FIG00041008	icw(2);Ribosome_post-transcriptional_modification_and_chromosomal_segregation_cluster isu;pyrimidine_conversions

fig|6666666.28482.peg.4836	Contig_61	6131	5385	-	Ribosomal large subunit pseudouridine synthase B (EC 4.2.1.70)	FIG01956038	icw(2);CBSS-314276.3.peg.1499 icw(2);Ribosome_post-transcriptional_modification_and_chromosomal_segregation_cluster isu;RNA_pseudouridine_syntheses
fig|6666666.28482.peg.4837	Contig_61	6826	6128	-	Segregation and condensation protein B	FIG00133569	isu;CBSS-314276.3.peg.1499 icw(4);Ribosome_post-transcriptional_modification_and_chromosomal_segregation_cluster
fig|6666666.28482.peg.4838	Contig_61	7623	6823	-	Segregation and condensation protein A	FIG00019652	icw(1);CBSS-314276.3.peg.1499 icw(5);Ribosome_post-transcriptional_modification_and_chromosomal_segregation_cluster
fig|6666666.28482.peg.4839	Contig_61	8540	7620	-	Chromosome (plasmid) partitioning protein ParA / Sporulation initiation inhibitor protein Soj	FIG00006461	idu(2);RNA_modification_and_chromosome_partitioning_cluster idu(2);Bacterial_Cell_Division icw(7);Ribosome_post-transcriptional_modification_and_chromosomal_segregation_cluster idu(2);Bacterial_Cytoskeleton idu(2);Bacterial_Cytoskeleton idu(2);Plasmid_replication
fig|6666666.28482.peg.4840	Contig_61	8671	9423	+	Probable catechol-o-methyltransferase (EC 2.1.1.6)	FIG01323336	ff
fig|6666666.28482.peg.4841	Contig_61	10437	9496	-	Tyrosine recombinase XerD	FIG01227142	ff

fig|6666666.28482.peg.4842	Contig_61	11057	10434	-	ADP-ribose pyrophosphatase (EC 3.6.1.13)	FIG00000367	idu(1);NAD_and_NADP_cofactor_biosynthesis_global idu(1);CBSS-216591.1.peg.168 idu(1);Nudix_proteins_(nucleoside_triphosphate_hydrolases) idu(1);Ribosome_post-transcriptional_modification_and_chromosomal_segregation_cluster idu(1);CBSS-342610.3.peg.1794
fig|6666666.28482.peg.4843	Contig_61	12801	11050	-	CTP synthase (EC 6.3.4.2)	FIG00000176	isu;CTP_synthase_(EC_6.3.4.2)_cluster icw(2);Ribosome_post-transcriptional_modification_and_chromosomal_segregation_cluster isu;pyrimidine_conversions
fig|6666666.28482.peg.4844	Contig_61	13874	12930	-	FIG007481: hypothetical protein	FIG00007481	isu;CBSS-446462.5.peg.2987
fig|6666666.28482.peg.4845	Contig_61	15079	13898	-	FIG005773: conserved membrane protein ML1361	FIG00005773	icw(1);CBSS-446462.5.peg.2987
fig|6666666.28482.peg.4846	Contig_61	15457	15161	-	hypothetical protein		
fig|6666666.28482.peg.4847	Contig_62	590	183	-	Methyltransferase type 11		
fig|6666666.28482.peg.4848	Contig_62	1512	811	-	putative DNA-binding protein		
fig|6666666.28482.peg.4849	Contig_62	2157	1555	-	FIG00824105: hypothetical protein	FIG00824104	ff
fig|6666666.28482.peg.4850	Contig_62	2250	3320	+	diguanylate cyclase		
fig|6666666.28482.peg.4851	Contig_62	3439	3912	+	hypothetical protein	FIG00638284	ff
fig|6666666.28482.peg.4852	Contig_62	4724	3933	-	Succinate dehydrogenase iron-sulfur protein (EC 1.3.99.1)	FIG00026006	icw(1);Serine-glyoxylate_cycle icw(1);TCA_Cycle idu(1);Succinate_dehydrogenase

fig|6666666.28482.peg.4853	Contig_62	6478	4724	-	Succinate dehydrogenase flavoprotein subunit (EC 1.3.99.1)	FIG00136922	idu(1);Serine-glyoxylate_cycle idu(1);TCA_Cycle icw(3);Succinate_dehydrogenase
fig|6666666.28482.peg.4854	Contig_62	6965	6498	-	Succinate dehydrogenase hydrophobic membrane anchor protein	FIG00000807	icw(1);Succinate_dehydrogenase
fig|6666666.28482.peg.4855	Contig_62	7099	6962	-	hypothetical protein		
fig|6666666.28482.peg.4856	Contig_62	7118	8227	+	Mobile element protein	FIG01306568	ff
fig|6666666.28482.peg.4857	Contig_62	8613	8275	-	Succinate dehydrogenase cytochrome b-556 subunit	FIG00000800	icw(2);Succinate_dehydrogenase
fig|6666666.28482.peg.4858	Contig_62	8821	9225	+	Cytidine deaminase (EC 3.5.4.5)	FIG00000720	isu;pyrimidine_conversions
fig|6666666.28482.peg.4859	Contig_62	9222	10520	+	Thymidine phosphorylase (EC 2.4.2.4)	FIG00001131	isu;Deoxyribose_and_Deoxynucleoside_Catabolism icw(1);pyrimidine_conversions
fig|6666666.28482.peg.4860	Contig_62	10517	11596	+	Adenosine deaminase (EC 3.5.4.4)	FIG00000687	isu;Purine_conversions
fig|6666666.28482.peg.4861	Contig_62	11887	11603	-	Possible membrane protein		
fig|6666666.28482.peg.4862	Contig_62	11996	12679	+	hypothetical protein		
fig|6666666.28482.peg.4863	Contig_62	13359	12682	-	GntR-family transcriptional regulator		
fig|6666666.28482.peg.4864	Contig_62	13518	14552	+	Gentisate 1,2-dioxygenase (EC 1.13.11.4)	FIG00015188	isu;Gentisare_degradation isu;Salicylate_and_gentisate_catabolism

fig|6666666.28482.peg.4865	Contig_62	14606	15613	+	FIG00827792: hypothetical protein	FIG00827785	ff
fig|6666666.28482.peg.4866	Contig_62	16601	15618	-	PROBABLE MEMBRANE PROTEIN	FIG00816212	ff
fig|6666666.28482.peg.4867	Contig_62	17975	16668	-	FIG026501: hypothetical protein	FIG00544282	ff
fig|6666666.28482.peg.4868	Contig_62	18879	18028	-	Possible acid phosphatase (EC 3.1.3.2)	FIG01343968	ff
fig|6666666.28482.peg.4869	Contig_62	18972	19595	+	phosphoribosyltransferase		icw(1);pyrimidine_conversions
fig|6666666.28482.peg.4870	Contig_62	21164	19596	-	Phosphomannomutase (EC 5.4.2.8)	FIG00001022	isu;Mannose_Metabolism
fig|6666666.28482.peg.4871	Contig_62	21975	21169	-	Purine nucleoside phosphorylase (EC 2.4.2.1)	FIG00000659	isu;Purine_conversions isu;Adenosyl_nucleosidases isu;Deoxyribose_and_Deoxynucleoside_Catabolism isu;pyrimidine_conversions
fig|6666666.28482.peg.4872	Contig_62	22016	22681	+	cutinase	FIG01407927	ff
fig|6666666.28482.peg.4873	Contig_62	24148	22685	-	PPE family protein	FIG00013075	idu(57);CBSS-164757.7.peg.5020
fig|6666666.28482.peg.4874	Contig_63	70	573	+	Phosphohistidine phosphatase SixA	FIG00001992	ff
fig|6666666.28482.peg.4875	Contig_63	1079	570	-	Putative lipoprotein LprC precursor	FIG01333765	ff
fig|6666666.28482.peg.4876	Contig_63	1651	1124	-	Lipoprotein LprB	FIG01320481	ff
fig|6666666.28482.peg.4877	Contig_63	1801	3546	+	ABC transporter ATP-binding protein	FIG01956052	ff
fig|6666666.28482.peg.4878	Contig_63	3543	5420	+	Lipid A export ATP-binding/permease protein MsbA	FIG00017059	ff
fig|6666666.28482.peg.4879	Contig_63	5473	5796	+	hypothetical protein		
fig|6666666.28482.peg.4880	Contig_63	5820	6506	+	FIG00820366: hypothetical protein	FIG00820364	ff

fig|6666666.28482.peg.4881	Contig_63	6699	7727	+	Transcriptional regulatory protein EmbR	FIG01308039	ff
fig|6666666.28482.peg.4882	Contig_63	7821	8177	+	FIG00820785: hypothetical protein	FIG00820783	ff
fig|6666666.28482.peg.4883	Contig_63	8355	9053	+	FIG00820366: hypothetical protein	FIG00820364	ff
fig|6666666.28482.peg.4884	Contig_63	9092	10846	+	Serine/threonine protein kinase (EC 2.7.11.1)	FIG00933379	ff
fig|6666666.28482.peg.4885	Contig_63	11009	12895	+	Serine/threonine protein kinase (EC 2.7.11.1)	FIG00933379	ff
fig|6666666.28482.peg.4886	Contig_63	13497	12913	-	FIG00820425: hypothetical protein	FIG00820423	ff
fig|6666666.28482.peg.4887	Contig_63	14865	13738	-	Adenylate cyclase (EC 4.6.1.1)	FIG00001294	idu(25);cAMP_signaling_in_bacteria
fig|6666666.28482.peg.4888	Contig_63	16252	14876	-	Amidase (EC 3.5.1.4)	FIG00454158	ff
fig|6666666.28482.peg.4889	Contig_63	16296	16748	+	Bis(5'-nucleosyl)-tetraphosphatase (asymmetrical) (EC 3.6.1.17)	FIG00008590	isu;CBSS-176299.4.peg.1996A isu;pyrimidine_conversions
fig|6666666.28482.peg.4890	Contig_63	16753	17202	+	FIG029194: hypothetical protein	FIG01351665	ff
fig|6666666.28482.peg.4891	Contig_63	17288	21232	+	FIG00826180: hypothetical protein	FIG00826177	ff
fig|6666666.28482.peg.4892	Contig_63	22260	21229	-	bacterial luciferase family protein	FIG01390817	ff
fig|6666666.28482.peg.4893	Contig_63	22279	22413	+	hypothetical protein		
fig|6666666.28482.peg.4894	Contig_63	23525	22407	-	Oxidoreductase	FIG01321859	ff
fig|6666666.28482.peg.4895	Contig_63	24433	23522	-	Uracil-DNA glycosylase, family 5	FIG00018955	isu;Uracil-DNA_glycosylase

fig|6666666.28482.peg.4896	Contig_63	24412	25653	+	H+ antiporter integral membrane transport protein	FIG01357208	ff
fig|6666666.28482.peg.4897	Contig_63	25674	27041	+	"Glycolate oxidase, subunit GlcD"	FIG00821456	ff
fig|6666666.28482.peg.4898	Contig_63	27874	27053	-	O-methyltransferase-like protein	FIG01479193	ff
fig|6666666.28482.peg.4899	Contig_63	28341	27871	-	hypothetical protein	FIG00638284	ff
fig|6666666.28482.peg.4900	Contig_63	28426	29646	+	Cytochrome P450 130	FIG01370899	ff
fig|6666666.28482.peg.4901	Contig_63	29643	30215	+	Possible oxidoreductase subunit	FIG01327895	ff
fig|6666666.28482.peg.4902	Contig_63	31768	30512	-	putative peptide monooxygenase		
fig|6666666.28482.peg.4903	Contig_63	32561	31755	-	Putative hemolysin	FIG01004154	ff
fig|6666666.28482.peg.4904	Contig_63	34153	32573	-	hypothetical protein	FIG00638284	ff
fig|6666666.28482.peg.4905	Contig_63	35296	34154	-	Acyltransferase	FIG01336741	ff
fig|6666666.28482.peg.4906	Contig_63	36960	35293	-	Cold-shock DEAD-box protein A	FIG00001381	isu;ATP-dependent_RNA_helicases,_bacterial
fig|6666666.28482.peg.4907	Contig_63	37308	36973	-	hypothetical protein		
fig|6666666.28482.peg.4908	Contig_63	37319	37657	+	Putative lipoprotein lprE precursor	FIG01347963	ff
fig|6666666.28482.peg.4909	Contig_63	37976	37686	-	PE-PGRS FAMILY PROTEIN		
fig|6666666.28482.peg.4910	Contig_64	53	373	+	hypothetical protein		
fig|6666666.28482.peg.4911	Contig_64	1962	577	-	Glycyl-tRNA synthetase (EC 6.1.1.14)	FIG00001189	isu;tRNA_aminoacylation,_Gly
fig|6666666.28482.peg.4912	Contig_64	2162	2602	+	Transcriptional regulator, ArsR family	FIG00003845	idu(3);CBSS-1085.1.peg.1363
fig|6666666.28482.peg.4913	Contig_64	2649	3041	+	Zinc uptake regulation protein ZUR	FIG00000479	isu;Oxidative_stress

fig|6666666.28482.peg.4914	Contig_64	3188	4186	+	Hydrolase, alpha/beta fold family	FIG01359650	ff
fig|6666666.28482.peg.4915	Contig_64	4611	4183	-	FIG00820411: hypothetical protein	FIG00820410	ff
fig|6666666.28482.peg.4916	Contig_64	5495	4611	-	Undecaprenyl pyrophosphate synthetase (EC 2.5.1.31)	FIG00000110	ff
fig|6666666.28482.peg.4917	Contig_64	6258	5488	-	DNA recombination and repair protein RecO	FIG00000315	ff
fig|6666666.28482.peg.4918	Contig_64	6347	7852	+	Asp-tRNAAsn/Glu-tRNAGln amidotransferase A subunit and related amidases	FIG00634208	ff
fig|6666666.28482.peg.4919	Contig_64	7914	9371	+	Arginine decarboxylase (EC 4.1.1.19); Ornithine decarboxylase (EC 4.1.1.17); Lysine decarboxylase (EC 4.1.1.18)	FIG01304207	idu(1);Arginine_and_Ornithine_Degradation isu;Arginine_and_Ornithine_Degradation idu(1);Lysine_degradation idu(1);Polyamine_Metabolism isu;Polyamine_Metabolism
fig|6666666.28482.peg.4920	Contig_64	10164	9373	-	dehydrogenase DhgA		
fig|6666666.28482.peg.4921	Contig_64	11117	10206	-	GTP-binding protein Era	FIG01304818	isu;Bacterial_Cell_Division
fig|6666666.28482.peg.4922	Contig_64	11434	11114	-	hypothetical protein		
fig|6666666.28482.peg.4923	Contig_64	12738	11431	-	Magnesium and cobalt efflux protein CorC	FIG00042627	idu(1);CBSS-56780.10.peg.1536
fig|6666666.28482.peg.4924	Contig_64	13364	12840	-	PE-PGRS family protein		idu(16);PPE_gene_cluster_in_Mycobacteria
fig|6666666.28482.peg.4925	Contig_65	145	891	+	hypothetical protein		

fig|6666666.28482.peg.4926	Contig_65	2531	1152	-	3-phenylpropionate dioxygenase alpha subunit (digoxigenin alpha subunit)(EC:1.14.12.18)		
fig|6666666.28482.peg.4927	Contig_65	3456	2551	-	3-oxoacyl-[acyl-carrier-protein] reductase(EC:1.1.1.100)		
fig|6666666.28482.peg.4928	Contig_65	4435	3539	-	probable enoyl-CoA hydratase(EC:4.2.1.17)		
fig|6666666.28482.peg.4929	Contig_65	4511	4942	+	hypothetical protein		
fig|6666666.28482.peg.4930	Contig_65	4985	6295	+	amidohydrolase 2		
fig|6666666.28482.peg.4931	Contig_65	6292	7485	+	Butyryl-CoA dehydrogenase (EC 1.3.99.2)	FIG00009149	idu(39);Isoleucine_degradation idu(39);Lysine_fermentation idu(39);Butanol_Biosynthesis idu(39);Acetyl-CoA_fermentation_to_Butyrate idu(39);Valine_degradation idu(39);Anaerobic_respiratory_reductases
fig|6666666.28482.peg.4932	Contig_65	7475	8560	+	acyl-CoA dehydrogenase-like protein		
fig|6666666.28482.peg.4933	Contig_65	8557	10695	+	hypothetical protein	FIG00638284	ff
fig|6666666.28482.peg.4934	Contig_65	10709	10915	+	hypothetical protein		
fig|6666666.28482.peg.4935	Contig_65	10912	12030	+	Thiolase		
fig|6666666.28482.peg.4936	Contig_65	12093	13307	+	putative cytochrome P450 hydroxylase	FIG00032437	icw(1);Nitric_oxide_synthase
fig|6666666.28482.peg.4937	Contig_65	14808	13468	-	hypothetical protein	FIG00638284	ff
fig|6666666.28482.peg.4938	Contig_65	16179	14956	-	hypothetical protein	FIG00638284	ff
fig|6666666.28482.peg.4939	Contig_65	17393	16188	-	putative cytochrome P450 hydroxylase	FIG00032437	icw(2);Nitric_oxide_synthase

fig|6666666.28482.peg.4940	Contig_65	17530	18738	+	putative subunit of succinyl-CoA:benzylsuccinate CoA-transferase		
fig|6666666.28482.peg.4941	Contig_65	18930	19937	+	L-carnitine dehydratase/bile acid-inducible protein F		
fig|6666666.28482.peg.4942	Contig_65	19934	20383	+	Glyoxalase/bleomycin resistance protein/dioxygenase		
fig|6666666.28482.peg.4943	Contig_65	20430	21629	+	putative cytochrome P450 hydroxylase	FIG00032437	icw(1);Nitric_oxide_synthase
fig|6666666.28482.peg.4944	Contig_65	21632	22675	+	acyl-CoA dehydrogenase		
fig|6666666.28482.peg.4945	Contig_65	22678	23817	+	acyl-CoA dehydrogenase, middle domain protein		
fig|6666666.28482.peg.4946	Contig_65	24042	23824	-	hypothetical protein		
fig|6666666.28482.peg.4947	Contig_65	24160	25455	+	BarH	FIG00668014	ff
fig|6666666.28482.peg.4948	Contig_65	26871	25477	-	Adenylate cyclase (EC 4.6.1.1)	FIG00001294	idu(25);cAMP_signaling_in_bacteria
fig|6666666.28482.peg.4949	Contig_65	27914	26967	-	Gluconolactonase (EC 3.1.1.17)	FIG00127296	ff
fig|6666666.28482.peg.4950	Contig_65	28459	27911	-	hypothetical protein		
fig|6666666.28482.peg.4951	Contig_65	29280	28465	-	3-oxoacyl-[acyl-carrier protein] reductase (EC 1.1.1.100)	FIG00621114	idu(38);CBSS-246196.1.peg.364 idu(38);CBSS-164757.7.peg.5020 idu(38);Fatty_Acid_Biosynthesis_FASI I
fig|6666666.28482.peg.4952	Contig_65	29405	30292	+	hypothetical protein		

fig|6666666.28482.peg.4953	Contig_65	31111	30314	-	Enoyl-CoA hydratase (EC 4.2.1.17)	FIG00033206	icw(3);n-Phenylalkanoic_acid_degradation icw(3);Polyhydroxybutyrate_metabolism icw(3);Isoleucine_degradation icw(3);Fatty_acid_metabolism_cluster icw(3);Lysine_fermentation icw(3);Butanol_Biosynthesis icw(3);Acetyl-CoA_fermentation_to_Butyrate icw(3);Valine_degradation icw(3);Butyrate_metabolism_cluster
fig|6666666.28482.peg.4954	Contig_65	32310	31108	-	putative cytochrome P450 hydroxylase	FIG00032437	idu(49);Nitric_oxide_synthase
fig|6666666.28482.peg.4955	Contig_65	32406	33218	+	Enoyl-CoA hydratase (EC 4.2.1.17)	FIG00033206	icw(4);n-Phenylalkanoic_acid_degradation icw(4);Polyhydroxybutyrate_metabolism icw(4);Isoleucine_degradation icw(4);Fatty_acid_metabolism_cluster icw(4);Lysine_fermentation icw(4);Butanol_Biosynthesis icw(4);Acetyl-CoA_fermentation_to_Butyrate icw(4);Valine_degradation icw(4);Butyrate_metabolism_cluster

fig|6666666.28482.peg.4956	Contig_65	33221	34423	+	acyl-CoA dehydrogenase domain protein		
fig|6666666.28482.peg.4957	Contig_65	35184	34426	-	Enoyl-CoA hydratase (EC 4.2.1.17)	FIG00033206	icw(4);n-Phenylalkanoic_acid_degradation icw(4);Polyhydroxybutyrate_metabolism icw(4);Isoleucine_degradation icw(4);Fatty_acid_metabolism_cluster icw(4);Lysine_fermentation icw(4);Butanol_Biosynthesis icw(4);Acetyl-CoA_fermentation_to_Butyrate icw(4);Valine_degradation icw(4);Butyrate_metabolism_cluster
fig|6666666.28482.peg.4958	Contig_65	35981	35181	-	Enoyl-CoA hydratase (EC 4.2.1.17)	FIG00033206	icw(4);n-Phenylalkanoic_acid_degradation icw(4);Polyhydroxybutyrate_metabolism icw(4);Isoleucine_degradation icw(4);Fatty_acid_metabolism_cluster icw(4);Lysine_fermentation icw(4);Butanol_Biosynthesis icw(4);Acetyl-CoA_fermentation_to_Butyrate icw(4);Valine_degradation icw(4);Butyrate_metabolism_cluster

fig|6666666.28482.peg.4959	Contig_65	36839	35988	-	Enoyl-CoA hydratase (EC 4.2.1.17)	FIG00033206	icw(3);n-Phenylalkanoic_acid_degradation icw(3);Polyhydroxybutyrate_metabolism icw(3);Isoleucine_degradation icw(3);Fatty_acid_metabolism_cluster icw(3);Lysine_fermentation icw(3);Butanol_Biosynthesis icw(3);Acetyl-CoA_fermentation_to_Butyrate icw(3);Valine_degradation icw(3);Butyrate_metabolism_cluster
fig|6666666.28482.peg.4960	Contig_65	37054	36836	-	hypothetical		
fig|6666666.28482.peg.4961	Contig_65	39090	37066	-	acetoin:2,6-dichlorophenolindophenol oxidoreductase beta subunit(EC:1.1.1.-,EC:1.2.4.1)		
fig|6666666.28482.peg.4962	Contig_65	39200	40474	+	Lipase LipD	FIG01326298	ff
fig|6666666.28482.peg.4963	Contig_65	40532	40975	+	hypothetical protein		
fig|6666666.28482.peg.4964	Contig_65	41316	40972	-	hypothetical protein		
fig|6666666.28482.peg.4965	Contig_65	41762	41367	-	protein of unknown function DUF35		
fig|6666666.28482.peg.4966	Contig_65	42910	41759	-	Acetyl-CoA acetyltransferase	FIG01005417	ff

fig|6666666.28482.peg.4967	Contig_65	44412	42922	-	Long-chain-fatty-acid--CoA ligase (EC 6.2.1.3)	FIG00018699	idu(72);n-Phenylalkanoic_acid_degradation idu(72);Fatty_acid_metabolism_cluster idu(72);Biotin_synthesis_cluster idu(72);Biotin_biosynthesis
fig|6666666.28482.peg.4968	Contig_65	45482	44409	-	luciferase		
fig|6666666.28482.peg.4969	Contig_65	45583	46410	+	hydroxysteroid (17-beta) dehydrogenase 4		
fig|6666666.28482.peg.4970	Contig_65	46413	47051	+	Transcriptional regulator, TetR family	FIG00554417	ff
fig|6666666.28482.peg.4971	Contig_65	47041	47790	+	3-oxoacyl-[acyl-carrier protein] reductase (EC 1.1.1.100)	FIG00621114	idu(38);CBSS-246196.1.peg.364 idu(38);CBSS-164757.7.peg.5020 idu(38);Fatty_Acid_Biosynthesis_FASII
fig|6666666.28482.peg.4972	Contig_65	47861	48628	+	Enoyl-CoA hydratase (EC 4.2.1.17)	FIG00033206	icw(2);n-Phenylalkanoic_acid_degradation idu(51);Polyhydroxybutyrate_metabolism icw(1);Isoleucine_degradation icw(2);Fatty_acid_metabolism_cluster icw(1);Lysine_fermentation icw(1);Butanol_Biosynthesis icw(1);Acetyl-CoA_fermentation_to_Butyrate icw(1);Valine_degradation idu(51);Butyrate_metabolism_cluster

fig|6666666.28482.peg.4973	Contig_65	48655	50295	+	Long-chain-fatty-acid--CoA ligase (EC 6.2.1.3)	FIG00018699	idu(72);n-Phenylalkanoic_acid_degradation idu(72);Fatty_acid_metabolism_cluster idu(72);Biotin_synthesis_cluster idu(72);Biotin_biosynthesis
fig|6666666.28482.peg.4974	Contig_65	50292	50933	+	hypothetical protein		
fig|6666666.28482.peg.4975	Contig_65	50935	52062	+	Butyryl-CoA dehydrogenase (EC 1.3.99.2)	FIG00009149	idu(39);Isoleucine_degradation idu(39);Lysine_fermentation idu(39);Butanol_Biosynthesis idu(39);Acetyl-CoA_fermentation_to_Butyrate idu(39);Valine_degradation idu(39);Anaerobic_respiratory_reductases
fig|6666666.28482.peg.4976	Contig_65	52865	52068	-	3-oxoacyl-[acyl-carrier protein] reductase (EC 1.1.1.100)	FIG00621114	idu(38);CBSS-246196.1.peg.364 idu(38);CBSS-164757.7.peg.5020 idu(38);Fatty_Acid_Biosynthesis_FASII
fig|6666666.28482.peg.4977	Contig_65	53033	53644	+	Transcriptional regulator, TetR family		
fig|6666666.28482.peg.4978	Contig_65	53744	54511	+	clavaldehyde dehydrogenase		idu(1);Clavulanic_acid_biosynthesis
fig|6666666.28482.peg.4979	Contig_65	54529	55812	+	hypothetical protein		
fig|6666666.28482.peg.4980	Contig_65	56450	55836	-	Transcriptional regulator, TetR family		
fig|6666666.28482.peg.4981	Contig_65	56732	57478	+	short-chain dehydrogenase/reductase SDR		
fig|6666666.28482.peg.4982	Contig_65	57594	57926	+	Protein of unknown function DUF35		

fig|6666666.28482.peg.4983	Contig_65	57926	59101	+	diterpenoid dioxygenase		
fig|6666666.28482.peg.4984	Contig_65	59148	60020	+	3-hydroxyisobutyrate dehydrogenase (EC 1.1.1.31)	FIG00021583	idu(3);Valine_degradation
fig|6666666.28482.rna.46	Contig_65	60385	60313	-	tRNA-Arg-CCT		
fig|6666666.28482.peg.4985	Contig_65	61244	60426	-	FIG00824358: hypothetical protein	FIG00824357	ff
fig|6666666.28482.peg.4986	Contig_65	62332	61277	-	FIG00823130: hypothetical protein	FIG00823129	ff
fig|6666666.28482.peg.4987	Contig_65	63985	62450	-	hypothetical protein	FIG00638284	ff
fig|6666666.28482.peg.4988	Contig_65	64301	64146	-	hypothetical protein		
fig|6666666.28482.peg.4989	Contig_66	1229	186	-	Transcription termination protein NusA	FIG00000168	isu;NusA-TFII_Cluster isu;Transcription_factors_bacterial isu;CBSS-350688.3.peg.1509
fig|6666666.28482.peg.4990	Contig_66	1777	1226	-	COG0779: clustered with transcription termination protein NusA	FIG00000325	ff
fig|6666666.28482.peg.4991	Contig_66	1996	2493	+	PROBABLE CONSERVED TRANSMEMBRANE ALANINE RICH PROTEIN	FIG01333321	ff
fig|6666666.28482.peg.4992	Contig_66	2507	2953	+	FIG00999285: hypothetical protein	FIG00820360	ff
fig|6666666.28482.peg.4993	Contig_66	4708	2960	-	Prolyl-tRNA synthetase (EC 6.1.1.15)	FIG00000069	ff
fig|6666666.28482.peg.4994	Contig_66	4810	6048	+	hypothetical protein		
fig|6666666.28482.peg.4995	Contig_66	7568	6138	-	EmrB/QacA family drug resistance transporter	FIG00997405	ff

fig|6666666.28482.peg.4996	Contig_66	8960	7764	-	Siroheme synthase / Precorrin-2 oxidase (EC 1.3.1.76) / Sirohydrochlorin ferrochelatase (EC 4.99.1.4) / Uroporphyrinogen-III methyltransferase (EC 2.1.1.107)	FIG00090522	idu(1);Dissimilatory_nitrite_reductase isu;Heme_and_Siroheme_Biosynthesis isu;Heme_and_Siroheme_Biosynthesis isu;Heme_and_Siroheme_Biosynthesis idu(1);Coenzyme_B12_biosynthesis
fig|6666666.28482.peg.4997	Contig_66	10406	8970	-	Cobyrinic acid A,C-diamide synthase	FIG00138468	icw(1);Cobalamin_synthesis icw(3);Coenzyme_B12_biosynthesis
fig|6666666.28482.peg.4998	Contig_66	11025	10435	-	Cob(I)alamin adenosyltransferase (EC 2.5.1.17)	FIG00000641	isu;Cobalamin_synthesis icw(2);Coenzyme_B12_biosynthesis
fig|6666666.28482.peg.4999	Contig_66	12892	11066	-	ChlI component of cobalt chelatase involved in B12 biosynthesis / ChlD component of cobalt chelatase involved in B12 biosynthesis	FIG00003696	icw(1);Coenzyme_B12_biosynthesis icw(1);Coenzyme_B12_biosynthesis
fig|6666666.28482.peg.5000	Contig_66	13359	12889	-	ElaA protein	FIG00139273	isu;cAMP_signaling_in_bacteria
fig|6666666.28482.peg.5001	Contig_66	14843	13356	-	Malate:quinone oxidoreductase (EC 1.1.5.4)	FIG00000978	isu;TCA_Cycle
fig|6666666.28482.peg.5002	Contig_66	14956	15957	+	Lysophospholipase (EC 3.1.1.5)	FIG00004249	isu;Triacylglycerol_metabolism
fig|6666666.28482.peg.5003	Contig_66	15968	17362	+	NADPH-dependent mycothiol reductase Mtr	FIG00023452	isu;Glutathione_analogs:_mycothiol

fig|6666666.28482.peg.5004	Contig_66	17826	17350	-	Transcriptional regulator, FUR family	FIG00002441	idu(2);Oxidative_stress
fig|6666666.28482.peg.5005	Contig_66	17922	19022	+	HoxN/HupN/NixA family nickel/cobalt transporter	FIG00016929	idu(1);Transport_of_Nickel_and_Cobalt
fig|6666666.28482.peg.5006	Contig_66	19095	19805	+	Adenosylcobinamide amidohydrolase (EC 3.5.1.90)	FIG00058905	isu;Coenzyme_B12_biosynthesis
fig|6666666.28482.peg.5007	Contig_66	21565	19844	-	PPE family protein		idu(57);CBSS-164757.7.peg.5020
fig|6666666.28482.peg.5008	Contig_66	21926	21804	-	hypothetical protein		
fig|6666666.28482.peg.5009	Contig_66	21925	22260	+	[NiFe] hydrogenase nickel incorporation protein HypA	FIG00001182	idu(1);NiFe_hydrogenase_maturation icw(1);G3E_family_of_P-loop_GTPases_(metallocenter_biosynthesis)
fig|6666666.28482.peg.5010	Contig_66	22289	23059	+	[NiFe] hydrogenase nickel incorporation-associated protein HypB	FIG00001210	icw(1);NiFe_hydrogenase_maturation idu(1);G3E_family_of_P-loop_GTPases_(metallocenter_biosynthesis)
fig|6666666.28482.peg.5011	Contig_66	23373	24428	+	Uptake hydrogenase small subunit precursor (EC 1.12.99.6)	FIG00133578	icw(1);Hydrogenases
fig|6666666.28482.peg.5012	Contig_66	24475	26274	+	Uptake hydrogenase large subunit (EC 1.12.99.6)	FIG00138205	idu(1);Hydrogenases
fig|6666666.28482.peg.5013	Contig_66	26278	27186	+	FIG00821991: hypothetical protein	FIG00821990	ff
fig|6666666.28482.peg.5014	Contig_66	27236	27883	+	FIG00823144: hypothetical protein	FIG00823142	ff
fig|6666666.28482.peg.5015	Contig_66	27880	28533	+	FIG00821078: hypothetical protein	FIG00821076	ff
fig|6666666.28482.peg.5016	Contig_66	28530	29852	+	FIG00822393: hypothetical protein	FIG00822392	ff

fig|6666666.28482.peg.5017	Contig_66	29865	29984	+	hypothetical protein	FIG00638284	ff
fig|6666666.28482.peg.5018	Contig_66	30001	30489	+	Hydrogenase maturation protease (EC 3.4.24.-)	FIG00621884	idu(1);Coenzyme_F420_hydrogenase idu(1);Hydrogen-sensing_regulatory_system
fig|6666666.28482.peg.5019	Contig_66	30517	30759	+	Hydrogenase assembly chaperone HypC/HupF	FIG00821018	ff
fig|6666666.28482.peg.5020	Contig_66	30779	33085	+	[NiFe] hydrogenase metallocenter assembly protein HypF	FIG00001209	idu(1);NiFe_hydrogenase_maturation icw(2);G3E_family_of_P-loop_GTPases_(metallocenter_biosynthesis)
fig|6666666.28482.peg.5021	Contig_66	33087	33797	+	FIG00823788: hypothetical protein	FIG00823787	ff
fig|6666666.28482.peg.5022	Contig_66	33797	34465	+	Phosphoheptose isomerase 1 (EC 5.3.1.-)	FIG00002799	idu(2);LOS_core_oligosaccharide_biosynthesis idu(2);Capsular_heptose_biosynthesis
fig|6666666.28482.peg.5023	Contig_66	34462	35574	+	[NiFe] hydrogenase metallocenter assembly protein HypE	FIG00003378	icw(3);NiFe_hydrogenase_maturation icw(1);G3E_family_of_P-loop_GTPases_(metallocenter_biosynthesis)
fig|6666666.28482.peg.5024	Contig_66	35590	35862	+	[NiFe] hydrogenase metallocenter assembly protein HypC	FIG00003908	icw(1);NiFe_hydrogenase_maturation icw(3);G3E_family_of_P-loop_GTPases_(metallocenter_biosynthesis)
fig|6666666.28482.peg.5025	Contig_66	35874	37013	+	[NiFe] hydrogenase metallocenter assembly protein HypD	FIG00001208	icw(2);NiFe_hydrogenase_maturation idu(1);G3E_family_of_P-loop_GTPases_(metallocenter_biosynthesis)
fig|6666666.28482.peg.5026	Contig_66	37010	37753	+	FIG00826825: hypothetical protein	FIG00826822	ff
fig|6666666.28482.peg.5027	Contig_66	37803	38447	+	hypothetical protein		

fig|6666666.28482.peg.5028	Contig_66	38475	39587	+	FIG00825760: hypothetical protein	FIG01569761	ff
fig|6666666.28482.peg.5029	Contig_66	40183	41025	+	PPE family protein	FIG00013075	idu(57);CBSS-164757.7.peg.5020
fig|6666666.28482.peg.5030	Contig_66	41785	41012	-	Short-chain dehydrogenase/reductase in hypothetical Actinobacterial gene cluster	FIG00023196	isu;CBSS-262316.1.peg.2929
fig|6666666.28482.peg.5031	Contig_66	43152	41785	-	Aldehyde dehydrogenase in hypothetical Actinobacterial gene cluster	FIG00146356	icw(3);CBSS-262316.1.peg.2929
fig|6666666.28482.peg.5032	Contig_66	43871	43149	-	COG2071: predicted glutamine amidotransferases in hypothetical Actinobacterial gene cluster	FIG00146363	icw(1);CBSS-262316.1.peg.2929
fig|6666666.28482.peg.5033	Contig_66	45210	43858	-	Glutamine synthetase family protein in hypothetical Actinobacterial gene cluster	FIG00143561	isu;Glutamine,_Glutamate,_Aspartate_and_Asparagine_Biosynthesis icw(2);CBSS-262316.1.peg.2929
fig|6666666.28482.peg.5034	Contig_66	45782	45222	-	FIG00822818: hypothetical protein	FIG00822817	ff
fig|6666666.28482.peg.5035	Contig_66	47329	45833	-	Cobyric acid synthase	FIG00000530	isu;Cobalamin_synthesis isu;Coenzyme_B12_biosynthesis
fig|6666666.28482.peg.5036	Contig_66	48206	47400	-	Methionine aminopeptidase (EC 3.4.11.18)	FIG00000036	idu(1);CBSS-312309.3.peg.1965 idu(1);Translation_termination_factors_bacterial

fig|6666666.28482.peg.5037	Contig_66	48449	49516	+	nuclear receptor binding factor related protein	FIG01002080	ff
fig|6666666.28482.peg.5038	Contig_66	50185	49511	-	DNA-binding protein		
fig|6666666.28482.peg.5039	Contig_66	50451	50909	+	hypothetical protein		
fig|6666666.28482.peg.5040	Contig_66	50951	51919	+	dehydrogenase		
fig|6666666.28482.peg.5041	Contig_66	52623	52039	-	FIG020268: hypothetical protein	FIG01349205	ff
fig|6666666.28482.peg.5042	Contig_66	54521	52668	-	Cell division protein FtsI [Peptidoglycan synthetase] (EC 2.4.1.129)	FIG00046929	idu(2);Bacterial_Cell_Division idu(2);16S_rRNA_modification_within_P_site_of_ribosome idu(2);Flagellum_in_Campylobacter idu(2);Bacterial_Cytoskeleton idu(2);Peptidoglycan_Biosynthesis
fig|6666666.28482.peg.5043	Contig_66	55493	54639	-	GCN5-related N-acetyltransferase, FIGfam019367	FIG00019367	ff
fig|6666666.28482.peg.5044	Contig_66	56713	55556	-	1-hydroxy-2-methyl-2-(E)-butenyl 4-diphosphate synthase (EC 1.17.7.1)	FIG00000449	icw(2);Nonmevalonate_Branch_of_Isoprenoid_Biosynthesis icw(2);Isoprenoid_Biosynthesis
fig|6666666.28482.peg.5045	Contig_66	57933	56710	-	Membrane-associated zinc metalloprotease	FIG00002803	ff
fig|6666666.28482.peg.5046	Contig_66	59063	57930	-	1-deoxy-D-xylulose 5-phosphate reductoisomerase (EC 1.1.1.267)	FIG00000401	icw(1);Nonmevalonate_Branch_of_Isoprenoid_Biosynthesis icw(1);Isoprenoid_Biosynthesis
fig|6666666.28482.peg.5047	Contig_66	60291	59164	-	1-deoxy-D-xylulose 5-phosphate reductoisomerase (EC 1.1.1.267)	FIG00000401	icw(1);Nonmevalonate_Branch_of_Isoprenoid_Biosynthesis icw(1);Isoprenoid_Biosynthesis

fig|6666666.28482.peg.5048	Contig_66	60799	60296	-	Soluble secreted antigen MPT53 precursor	FIG01347939	ff
fig|6666666.28482.peg.5049	Contig_66	62063	60969	-	Ribosomal RNA large subunit methyltransferase N (EC 2.1.1.-)	FIG00000293	isu;RNA_methylation
fig|6666666.28482.peg.5050	Contig_66	63070	62141	-	Phosphatidate cytidylyltransferase (EC 2.7.7.41)	FIG00000097	idu(1);Glycerolipid_and_Glycerophospholipid_Metabolism_in_Bacteria
fig|6666666.28482.peg.5051	Contig_66	63644	63087	-	Ribosome recycling factor	FIG00000202	isu;CBSS-176299.4.peg.1996B isu;Translation_termination_factors_bacterial
fig|6666666.28482.peg.5052	Contig_66	64410	63661	-	Uridylate kinase (EC 2.7.4.-)	FIG00001819	icw(1);CBSS-176299.4.peg.1996B isu;CBSS-312309.3.peg.1965
fig|6666666.28482.peg.5053	Contig_66	64713	64949	+	FIG00826147: hypothetical protein	FIG01384975	ff
fig|6666666.28482.peg.5054	Contig_66	65116	65895	+	Transcriptional regulatory protein glnR	FIG01335353	ff
fig|6666666.28482.peg.5055	Contig_66	66795	65929	-	FIG00824661: hypothetical protein	FIG00824660	ff
fig|6666666.28482.peg.5056	Contig_66	67543	66959	-	FIG00826478: hypothetical protein	FIG00826477	ff
fig|6666666.28482.peg.5057	Contig_66	67736	67912	+	FIG00828846: hypothetical protein	FIG00828845	ff
fig|6666666.28482.peg.5058	Contig_66	67945	68784	+	Enoyl-[acyl-carrier-protein] reductase [NADPH] (EC 1.3.1.10)	FIG00032108	idu(2);Fatty_Acid_Biosynthesis_FASII
fig|6666666.28482.peg.5059	Contig_66	69024	68797	-	hypothetical protein		
fig|6666666.28482.peg.5060	Contig_66	69530	69117	-	FIG00822516: hypothetical protein	FIG00822515	ff

fig|6666666.28482.peg.5061	Contig_66	70081	69545	-	FIG00823334: hypothetical protein	FIG00823333	ff
fig|6666666.28482.peg.5062	Contig_66	70080	70601	+	Transcriptional regulator, MarR family	FIG00554610	ff
fig|6666666.28482.peg.5063	Contig_66	72016	70604	-	Putative amidase amiC (EC 3.5.1.4)	FIG01313765	ff
fig|6666666.28482.peg.5064	Contig_66	72865	72047	-	Translation elongation factor Ts	FIG00000191	icw(1);CBSS-176299.4.peg.1996B isu;Translation_elongation_factors_bacterial icw(1);CBSS-312309.3.peg.1965
fig|6666666.28482.peg.5065	Contig_66	73721	72876	-	SSU ribosomal protein S2p (SAe)	FIG00037273	isu;CBSS-176299.4.peg.1996B isu;CBSS-312309.3.peg.1965
fig|6666666.28482.peg.5066	Contig_66	73963	74445	+	Membrane proteins related to metalloendopeptidases	FIG00011382	ff
fig|6666666.28482.peg.5067	Contig_66	75635	74733	-	Tyrosine recombinase XerC	FIG00600726	isu;CBSS-323850.3.peg.3269
fig|6666666.28482.peg.5068	Contig_66	76831	75668	-	Lactate 2-monooxygenase (EC 1.13.12.4)	FIG00007876	isu;Pyruvate_metabolism_II:_acetyl-CoA,_acetogenesis_from_pyruvate
fig|6666666.28482.peg.5069	Contig_66	77684	76836	-	Iron utilization protein	FIG00997350	ff
fig|6666666.28482.peg.5070	Contig_66	77757	78608	+	Transcriptional regulator, IclR family	FIG00022569	idu(12);Homogentisate_pathway_of_aromatic_compound_degradation
fig|6666666.28482.peg.5071	Contig_66	79832	78624	-	PPE family protein	FIG00013075	icw(1);CBSS-164757.7.peg.5020
fig|6666666.28482.peg.5072	Contig_66	80051	79839	-	PE family protein	FIG01311212	ff
fig|6666666.28482.peg.5073	Contig_66	81290	80202	-	PPE family protein	FIG00013075	icw(1);CBSS-164757.7.peg.5020
fig|6666666.28482.peg.5074	Contig_66	82632	81325	-	Major facilitator superfamily (MFS_1) transporter		

fig|6666666.28482.peg.5075	Contig_66	82859	84079	+	L-carnitine dehydratase/bile acid-inducible protein F (EC 2.8.3.16)	FIG00450481	ff
fig|6666666.28482.peg.5076	Contig_66	84076	85008	+	Hydroxymethylglutaryl-CoA lyase (EC 4.1.3.4)	FIG00000963	idu(2);HMG_CoA_Synthesis idu(2);Leucine_Degradation_and_HMG-CoA_Metabolism
fig|6666666.28482.peg.5077	Contig_66	86142	85015	-	Rossmann fold nucleotide-binding protein Smf possibly involved in DNA uptake	FIG00035268	isu;CBSS-272943.3.peg.1367
fig|6666666.28482.peg.5078	Contig_66	87650	86139	-	MG(2+) CHELATASE FAMILY PROTEIN / ComM-related protein	FIG00000635	ff
fig|6666666.28482.peg.5079	Contig_66	88027	87650	-	Predicted endonuclease distantly related to archaeal Holliday junction resolvase	FIG00009496	ff
fig|6666666.28482.peg.5080	Contig_66	88315	88142	-	hypothetical protein		
fig|6666666.28482.peg.5081	Contig_66	89135	88320	-	Formate dehydrogenase chain D (EC 1.2.1.2)	FIG00000529	isu;Formate_hydrogenase
fig|6666666.28482.peg.5082	Contig_66	90222	89161	-	FIG00823170: hypothetical protein	FIG00823168	ff
fig|6666666.28482.peg.5083	Contig_66	90243	90983	+	FIG00827258: hypothetical protein	FIG00827257	ff
fig|6666666.28482.peg.5084	Contig_66	91295	90990	-	Protein often found in Actinomycetes clustered with signal peptidase and/or RNaseHII	FIG00004032	icw(1);Ribonuclease_H

fig|6666666.28482.peg.5085	Contig_66	92073	91345	-	Ribonuclease HII (EC 3.1.26.4)	FIG00000126	isu;Ribonucleases_in_Bacillus isu;DNA_replication,_archaeal isu;Ribonuclease_H
fig|6666666.28482.peg.5086	Contig_66	92936	92076	-	Signal peptidase I (EC 3.4.21.89)	FIG00000015	isu;Signal_peptidase
fig|6666666.28482.peg.5087	Contig_66	93335	92994	-	LSU ribosomal protein L19p	FIG00000214	isu;Ribosome_LSU_bacterial
fig|6666666.28482.peg.5088	Contig_66	93645	94601	+	Putative lipoprotein lppW precursor	FIG01328011	ff
fig|6666666.28482.peg.5089	Contig_66	95373	94687	-	tRNA (Guanine37-N1) -methyltransferase (EC 2.1.1.31)	FIG00000230	isu;RNA_methylation
fig|6666666.28482.peg.5090	Contig_66	95885	95391	-	16S rRNA processing protein RimM	FIG00000312	isu;KH_domain_RNA_binding_protein_YlqC
fig|6666666.28482.peg.5091	Contig_66	96161	95919	-	KH domain RNA binding protein YlqC	FIG00005628	icw(1);KH_domain_RNA_binding_protein_YlqC
fig|6666666.28482.peg.5092	Contig_67	6	815	+	hypothetical protein		
fig|6666666.28482.peg.5093	Contig_67	998	1564	+	Dihydrofolate reductase (EC 1.5.1.3)	FIG00000048	idu(3);Folate_Biosynthesis
fig|6666666.28482.peg.5094	Contig_67	1677	2495	+	Non-heme chloroperoxidase (EC 1.11.1.10)	FIG01225742	ff
fig|6666666.28482.peg.5095	Contig_67	2754	2518	-	hypothetical protein		
fig|6666666.28482.peg.5096	Contig_67	3603	3746	+	hypothetical protein		
fig|6666666.28482.peg.5097	Contig_67	5212	4067	-	diguanylate cyclase		
fig|6666666.28482.peg.5098	Contig_67	5957	5493	-	hypothetical protein	FIG00638284	ff
fig|6666666.28482.peg.5099	Contig_67	7065	6121	-	hypothetical protein	FIG00638284	ff
fig|6666666.28482.peg.5100	Contig_67	7259	7726	+	hypothetical protein		
fig|6666666.28482.peg.5101	Contig_67	7878	8288	+	hypothetical protein		
fig|6666666.28482.peg.5102	Contig_67	8504	9985	+	POSSIBLE CARBOXYLASE	FIG00841937	ff
fig|6666666.28482.peg.5103	Contig_67	9982	11175	+	6-aminohexanoate-dimer hydrolase	FIG01513469	ff

fig|6666666.28482.peg.5104	Contig_67	11305	11159	-	hypothetical protein		
fig|6666666.28482.peg.5105	Contig_67	11586	11831	+	hypothetical protein		
fig|6666666.28482.peg.5106	Contig_67	11786	12439	+	FIG00831057: hypothetical protein	FIG00831056	ff
fig|6666666.28482.peg.5107	Contig_67	12550	12753	+	Cold shock protein CspA	FIG00004100	idu(1);Cold_shock,_CspA_family_of_proteins
fig|6666666.28482.peg.5108	Contig_67	13063	12833	-	FIG00827556: hypothetical protein	FIG00827551	ff
fig|6666666.28482.peg.5109	Contig_67	13093	14019	+	Probable carboxyvinyl-carboxyphosphonate phosphorylmutase (EC 2.7.8.23)	FIG00012387	ff
fig|6666666.28482.peg.5110	Contig_67	14510	14367	-	hypothetical protein		
fig|6666666.28482.peg.5111	Contig_67	15152	14760	-	FIG00829547: hypothetical protein	FIG00829546	ff
fig|6666666.28482.peg.5112	Contig_67	15255	15704	+	hypothetical protein		
fig|6666666.28482.peg.5113	Contig_67	15813	16658	+	oxidoreductase		
fig|6666666.28482.peg.5114	Contig_67	16786	17484	+	hypothetical protein	FIG00638284	ff
fig|6666666.28482.peg.5115	Contig_67	17481	17957	+	hypothetical protein		
fig|6666666.28482.peg.5116	Contig_67	18213	19301	+	Sensory box/GGDEF family protein	FIG01325709	ff
fig|6666666.28482.peg.5117	Contig_67	19623	22181	+	regulatory protein, LuxR		
fig|6666666.28482.peg.5118	Contig_67	22216	22539	+	hypothetical protein		
fig|6666666.28482.peg.5119	Contig_67	22710	23816	+	FIG00827948: hypothetical protein	FIG00827946	ff
fig|6666666.28482.peg.5120	Contig_67	24275	23835	-	hypothetical protein		
fig|6666666.28482.peg.5121	Contig_68	245	394	+	hypothetical protein		
fig|6666666.28482.peg.5122	Contig_68	3740	3856	+	hypothetical protein		
fig|6666666.28482.peg.5123	Contig_68	3887	4087	+	hypothetical protein		
fig|6666666.28482.peg.5124	Contig_68	7329	7574	+	FIG00828124: hypothetical protein	FIG00828123	ff
fig|6666666.28482.peg.5125	Contig_68	7963	7628	-	hypothetical protein		

fig|6666666.28482.peg.5126	Contig_68	8329	8021	-	fdxD-related protein	FIG01419919	ff
fig|6666666.28482.peg.5127	Contig_68	9606	8326	-	NADH-quinone oxidoreductase chain F 2 (EC 1.6.99.5); Hypothetical FeS oxidoreductase	FIG00087063	idu(1);Respiratory_Complex_I
fig|6666666.28482.peg.5128	Contig_68	9665	10111	+	FIG00824538: hypothetical protein	FIG00824535	ff
fig|6666666.28482.peg.5129	Contig_68	10983	10117	-	FIG00820694: hypothetical protein	FIG00820693	ff
fig|6666666.28482.peg.5130	Contig_68	11327	10995	-	Rieske (2Fe-2S) domain-containing protein	FIG01358726	ff
fig|6666666.28482.peg.5131	Contig_68	12555	11410	-	Amidohydrolase 2	FIG00821472	ff
fig|6666666.28482.peg.5132	Contig_68	13684	12593	-	FIG00821228: hypothetical protein	FIG01354128	ff
fig|6666666.28482.peg.5133	Contig_68	13964	14605	+	hypothetical protein		
fig|6666666.28482.peg.5134	Contig_68	15653	14751	-	FIG00821205: hypothetical protein	FIG00821204	ff
fig|6666666.28482.peg.5135	Contig_68	16043	15663	-	4-carboxymuconolactone decarboxylase (EC 4.1.1.44)	FIG01304850	idu(2);Protocatechuate_branch_of_beta-ketoadipate_pathway
fig|6666666.28482.peg.5136	Contig_68	16126	16776	+	nitroreductase family protein	FIG01312078	ff
fig|6666666.28482.peg.5137	Contig_68	16873	17871	+	FIG00821611: hypothetical protein	FIG00821610	ff
fig|6666666.28482.peg.5138	Contig_68	17855	19030	+	Acyl-CoA dehydrogenase (EC 1.3.99.-)	FIG01333225	ff
fig|6666666.28482.peg.5139	Contig_68	20534	19116	-	FIG00824695: hypothetical protein	FIG01366795	ff

fig|6666666.28482.peg.5140	Contig_68	22097	20721	-	putative cytochrome P450 hydroxylase	FIG00032437	idu(49);Nitric_oxide_synthase
fig|6666666.28482.peg.5141	Contig_68	22312	22112	-	fdxD-related protein	FIG01419919	ff
fig|6666666.28482.peg.5142	Contig_68	23209	22334	-	Oxidoreductase, short-chain dehydrogenase/reductase family	FIG00822532	ff
fig|6666666.28482.peg.5143	Contig_68	23934	23233	-	3-oxoacyl-[acyl-carrier protein] reductase (EC 1.1.1.100)	FIG00621114	idu(38);CBSS-246196.1.peg.364 idu(38);CBSS-164757.7.peg.5020 idu(38);Fatty_Acid_Biosynthesis_FASII
fig|6666666.28482.peg.5144	Contig_68	24222	25715	+	Aldehyde dehydrogenase (EC 1.2.1.3)	FIG01292786	idu(16);Pyruvate_metabolism_II:_acetyl-CoA,_acetogenesis_from_pyruvate idu(16);Glycerolipid_and_Glycerophospholipid_Metabolism_in_Bacteria idu(16);Methylglyoxal_Metabolism
fig|6666666.28482.peg.5145	Contig_68	25712	26917	+	L-carnitine dehydratase/bile acid-inducible protein F	FIG01318850	ff
fig|6666666.28482.peg.5146	Contig_68	27150	27275	+	hypothetical protein		
fig|6666666.28482.peg.5147	Contig_68	27350	28099	+	Conserved hypothetical integral membrane protein YrbE1A	FIG00690583	idu(7);Mycobacterium_virulence_operon_MCE_involved_in_cell_invasion
fig|6666666.28482.peg.5148	Contig_68	28119	28967	+	Conserved hypothetical integral membrane protein YrbE1B	FIG00691621	icw(2);Mycobacterium_virulence_operon_MCE_involved_in_cell_invasion

fig|6666666.28482.peg.5149	Contig_68	28987	30264	+	MCE-family protein Mce1A	FIG00820494	icw(1);Mycobacterium_virulence_operon_MCE_involved_in_cell_invasion
fig|6666666.28482.peg.5150	Contig_68	30261	31301	+	MCE-family protein Mce1B	FIG00763551	icw(3);Mycobacterium_virulence_operon_MCE_involved_in_cell_invasion
fig|6666666.28482.peg.5151	Contig_68	31294	32376	+	MCE-family protein Mce1C	FIG00821293	icw(4);Mycobacterium_virulence_operon_MCE_involved_in_cell_invasion
fig|6666666.28482.peg.5152	Contig_69	121	1044	+	hypothetical protein		
fig|6666666.28482.peg.5153	Contig_69	1037	1906	+	similar to nucleoside-diphosphate-sugar epimerases		
fig|6666666.28482.peg.5154	Contig_69	1912	2217	+	conserved hypothetical protein		
fig|6666666.28482.peg.5155	Contig_69	2267	3007	+	hypothetical protein	FIG00638284	ff
fig|6666666.28482.peg.5156	Contig_69	2991	3959	+	sigma factor, sigma 70 type, group 4 (ECF)		
fig|6666666.28482.peg.5157	Contig_69	3956	4390	+	hypothetical protein		
fig|6666666.28482.peg.5158	Contig_69	4673	6256	+	L-lactate permease	FIG00016061	idu(1);Lactate_utilization
fig|6666666.28482.peg.5159	Contig_69	6348	7034	+	FIG00829569: hypothetical protein	FIG00829556	ff
fig|6666666.28482.peg.5160	Contig_69	8099	7059	-	hypothetical protein	FIG00638284	ff
fig|6666666.28482.peg.5161	Contig_69	9084	8302	-	D-beta-hydroxybutyrate dehydrogenase (EC 1.1.1.30)	FIG01954109	isu;Polyhydroxybutyrate_metabolism
fig|6666666.28482.peg.5162	Contig_69	9424	9089	-	Anti-sigma F factor antagonist (spoIIAA-2); Anti-sigma B factor antagonist RsbV	FIG00828677	icw(1);SigmaB_stress_responce_regulation icw(1);Sigma-B_stress_response_cluster_1

fig|6666666.28482.peg.5163	Contig_69	11937	9406	-	Serine phosphatase RsbU, regulator of sigma subunit	FIG00017431	idu(4);SigmaB_stress_responce_regulation idu(4);Sigma-B_stress_response_cluster_1
fig|6666666.28482.peg.5164	Contig_69	12874	11948	-	probable iron-sulfur binding protein YPO1417	FIG01478501	ff
fig|6666666.28482.peg.5165	Contig_69	13812	12871	-	Aldehyde reductase(EC:1.1.1.21)		
fig|6666666.28482.peg.5166	Contig_69	14549	13791	-	Isochorismatase (EC 3.3.2.1)	FIG00009017	idu(3);Chorismate:_Intermediate_for_synthesis_of_Tryptophan,_PAPA_antibiotics,_PABA,_3-hydroxyanthranilate_and_more.
fig|6666666.28482.peg.5167	Contig_69	15228	14656	-	Transcriptional regulator, TetR family		
fig|6666666.28482.peg.5168	Contig_69	15325	15771	+	FIG00823608: hypothetical protein	FIG00823607	ff
fig|6666666.28482.peg.5169	Contig_69	15974	16402	+	membrane protein, MmpS family	FIG01309473	ff
fig|6666666.28482.peg.5170	Contig_69	16450	19242	+	Putative membrane protein	FIG01056920	ff
fig|6666666.28482.peg.5171	Contig_69	20707	19274	-	FIG00827739: hypothetical protein	FIG00827738	ff
fig|6666666.28482.peg.5172	Contig_69	20684	20833	+	hypothetical protein		
fig|6666666.28482.peg.5173	Contig_69	20876	22432	+	Aldehyde dehydrogenase (EC 1.2.1.3)	FIG01292786	idu(16);Pyruvate_metabolism_II:_acetyl-CoA,_acetogenesis_from_pyruvate idu(16);Glycerolipid_and_Glycerophospholipid_Metabolism_in_Bacteria idu(16);Methylglyoxal_Metabolism

fig|6666666.28482.peg.5174	Contig_69	22604	23038	+	FIG00823693: hypothetical protein	FIG00823691	ff
fig|6666666.28482.peg.5175	Contig_69	23121	23501	+	hypothetical protein		
fig|6666666.28482.peg.5176	Contig_69	24214	23486	-	Sulfotransferase		
fig|6666666.28482.peg.5177	Contig_69	24377	26521	+	putative serine/threonine protein kinase		
fig|6666666.28482.peg.5178	Contig_69	27314	26541	-	FIG00828236: hypothetical protein	FIG00828235	ff
fig|6666666.28482.peg.5179	Contig_69	27606	27331	-	hypothetical protein		
fig|6666666.28482.peg.5180	Contig_69	28497	27646	-	POSSIBLE MEMBRANE PROTEIN		
fig|6666666.28482.peg.5181	Contig_69	29343	28519	-	hypothetical protein		
fig|6666666.28482.peg.5182	Contig_69	29467	30660	+	Aldose 1-epimerase (EC 5.1.3.3)	FIG00135025	isu;Maltose_and_Maltodextrin_Utilization isu;Lactose_and_Galactose_Uptake_and_Utilization
fig|6666666.28482.peg.5183	Contig_69	31158	30676	-	hypothetical protein		
fig|6666666.28482.peg.5184	Contig_69	31339	32652	+	putative cytochrome P450 hydroxylase	FIG00032437	idu(49);Nitric_oxide_synthase
fig|6666666.28482.peg.5185	Contig_69	34536	33589	-	hypothetical protein	FIG00638284	ff
fig|6666666.28482.peg.5186	Contig_69	34648	34983	+	hypothetical protein	FIG00638284	ff
fig|6666666.28482.peg.5187	Contig_69	36431	35025	-	Nitrate/nitrite transporter	FIG00005529	idu(2);Nitrate_and_nitrite_ammonification
fig|6666666.28482.peg.5188	Contig_69	39379	39059	-	Ferredoxin	FIG00001676	idu(4);Soluble_cytochromes_and_functionally_related_electron_carriers idu(4);Inorganic_Sulfur_Assimilation
fig|6666666.28482.peg.5189	Contig_69	41026	39431	-	FAD dependent oxidoreductase		
fig|6666666.28482.peg.5190	Contig_69	41249	41746	+	Transcriptional regulator, TetR family		

fig|6666666.28482.peg.5191	Contig_69	41905	43041	+	Phenylpropionate dioxygenase and related ring-hydroxylating dioxygenases, large terminal subunit		
fig|6666666.28482.peg.5192	Contig_69	43213	43091	-	hypothetical protein		
fig|6666666.28482.peg.5193	Contig_69	43956	43441	-	hypothetical protein		
fig|6666666.28482.peg.5194	Contig_69	45582	44302	-	Amidohydrolase 2		
fig|6666666.28482.peg.5195	Contig_69	47631	45850	-	D-aminoacylase (EC 3.5.1.81)	FIG01332753	ff
fig|6666666.28482.peg.5196	Contig_69	48233	49891	+	Long-chain-fatty-acid--CoA ligase (EC 6.2.1.3)	FIG00018699	idu(72);n-Phenylalkanoic_acid_degradation idu(72);Fatty_acid_metabolism_cluster idu(72);Biotin_synthesis_cluster idu(72);Biotin_biosynthesis
fig|6666666.28482.peg.5197	Contig_69	51858	50392	-	Cyclohexanone monooxygenase (EC 1.14.13.22)	FIG00975774	ff
fig|6666666.28482.peg.5198	Contig_69	52425	52033	-	alpha/beta hydrolase fold domain protein		
fig|6666666.28482.peg.5199	Contig_69	53510	52443	-	hypothetical protein		
fig|6666666.28482.peg.5200	Contig_69	53733	53867	+	hypothetical protein		
fig|6666666.28482.peg.5201	Contig_69	55105	54059	-	N5,N10-methylenetetrahydromethanopterin reductase-related protein	FIG01460162	ff
fig|6666666.28482.peg.5202	Contig_69	55476	55102	-	FIG00822683: hypothetical protein	FIG00822682	ff

fig|6666666.28482.peg.5203	Contig_69	55841	55473	-	protein of unknown function DUF227		
fig|6666666.28482.peg.5204	Contig_69	57002	55887	-	Alcohol dehydrogenase (EC 1.1.1.1)	FIG01007502	idu(17);Glycerolipid_and_Glycerophospholipid_Metabolism_in_Bacteria idu(17);Fermentations:_Mixed_acid idu(17);Butanol_Biosynthesis
fig|6666666.28482.peg.5205	Contig_69	58527	57037	-	Aldehyde dehydrogenase (EC 1.2.1.3)	FIG01292786	idu(16);Pyruvate_metabolism_II:_acetyl-CoA,_acetogenesis_from_pyruvate icw(1);Glycerolipid_and_Glycerophospholipid_Metabolism_in_Bacteria idu(16);Methylglyoxal_Metabolism
fig|6666666.28482.peg.5206	Contig_69	59100	58747	-	rieske [2Fe-2S] domain protein		
fig|6666666.28482.peg.5207	Contig_69	59450	59097	-	hypothetical protein		
fig|6666666.28482.peg.5208	Contig_69	60837	60325	-	hypothetical protein		
fig|6666666.28482.peg.5209	Contig_69	61643	60834	-	hypothetical protein	FIG00638284	ff
fig|6666666.28482.peg.5210	Contig_69	62425	61721	-	possible transcriptional regulator, GntR family		
fig|6666666.28482.peg.5211	Contig_69	62561	62704	+	hypothetical protein		
fig|6666666.28482.peg.5212	Contig_69	62727	63980	+	hypothetical protein	FIG00638284	ff
fig|6666666.28482.peg.5213	Contig_69	64082	64360	+	Rieske [2Fe-2S] domain, putative	FIG00825996	ff
fig|6666666.28482.peg.5214	Contig_69	64363	65487	+	amidohydrolase family protein	FIG00604942	ff

fig|6666666.28482.peg.5215	Contig_69	65484	66464	+	Flavodoxin reductases (ferredoxin-NADPH reductases) family 1; Vanillate O-demethylase oxidoreductase (EC 1.14.13.-)	FIG00001276	idu(2);Anaerobic_respiratory_reductases idu(3);Anaerobic_respiratory_reductases
fig|6666666.28482.peg.5216	Contig_7	52	276	+	hypothetical protein		
fig|6666666.28482.peg.5217	Contig_7	369	1628	+	Possible membrane protein	FIG01121868	ff
fig|6666666.28482.peg.5218	Contig_7	1739	4390	+	Valyl-tRNA synthetase (EC 6.1.1.9)	FIG00000129	isu;tRNA_aminoacylation,_Val
fig|6666666.28482.peg.5219	Contig_7	4387	5823	+	Dihydrofolate synthase (EC 6.3.2.12) / Folylpolyglutamate synthase (EC 6.3.2.17)	FIG00000106	isu;Folate_Biosynthesis isu;Folate_Biosynthesis
fig|6666666.28482.peg.5220	Contig_7	5862	6176	+	Possible membrane protein	FIG01121868	ff
fig|6666666.28482.peg.5221	Contig_7	6206	6616	+	Nucleoside diphosphate kinase (EC 2.7.4.6)	FIG00000251	isu;Purine_conversions isu;pyrimidine_conversions
fig|6666666.28482.peg.5222	Contig_7	7074	9689	+	Ribonuclease E (EC 3.1.26.12)	FIG00000913	isu;RNA_processing_and_degradation,_bacterial
fig|6666666.28482.peg.5223	Contig_7	9894	10205	+	LSU ribosomal protein L21p	FIG00000276	isu;Ribosome_LSU_bacterial isu;CBSS-176279.3.peg.868
fig|6666666.28482.peg.5224	Contig_7	10216	10482	+	LSU ribosomal protein L27p	FIG00000189	icw(1);Ribosome_LSU_bacterial icw(1);CBSS-176279.3.peg.868
fig|6666666.28482.peg.5225	Contig_7	10571	12013	+	COG0536: GTP-binding protein Obg	FIG00000169	ff
fig|6666666.28482.peg.5226	Contig_7	12010	13113	+	Glutamate 5-kinase (EC 2.7.2.11) / RNA-binding C-terminal domain PUA	FIG01303783	isu;Proline_Synthesis isu;Proline_Synthesis

fig|6666666.28482.peg.5227	Contig_7	13537	13118	-	FIG00823955: hypothetical protein	FIG00823954	ff
fig|6666666.28482.peg.5228	Contig_7	14040	13555	-	Organic hydroperoxide resistance transcriptional regulator	FIG00025887	isu;Oxidative_stress isu;CBSS-269482.1.peg.1294
fig|6666666.28482.peg.5229	Contig_7	15788	14145	-	PPE family protein	FIG00013075	idu(57);CBSS-164757.7.peg.5020
fig|6666666.28482.peg.5230	Contig_7	18614	15987	-	hypothetical protein	FIG00638284	ff
fig|6666666.28482.peg.5231	Contig_7	18651	19256	+	hypothetical protein		
fig|6666666.28482.peg.5232	Contig_7	20740	22623	+	Acetyl-coenzyme A synthetase (EC 6.2.1.1)	FIG00022631	idu(2);Pyruvate_metabolism_II:_acetyl-CoA,_acetogenesis_from_pyruvate idu(2);Ketoisovalerate_oxidoreductase
fig|6666666.28482.peg.5233	Contig_7	23278	22628	-	Transcriptional regulator, TetR family	FIG00554417	ff
fig|6666666.28482.peg.5234	Contig_7	23379	24644	+	putative cytochrome P450 hydroxylase	FIG00032437	idu(49);Nitric_oxide_synthase
fig|6666666.28482.peg.5235	Contig_7	25489	24641	-	NAD-dependent protein deacetylase of SIR2 family	FIG00008490	icw(1);Pyruvate_metabolism_II:_acetyl-CoA,_acetogenesis_from_pyruvate icw(1);Redox-dependent_regulation_of_nucleus_processes
fig|6666666.28482.peg.5236	Contig_7	26056	25511	-	Nicotinamidase (EC 3.5.1.19)	FIG00067300	icw(1);NAD_and_NADP_cofactor_biosynthesis_global idu(1);Redox-dependent_regulation_of_nucleus_processes

fig|6666666.28482.peg.5237	Contig_7	26116	28158	+	NAD synthetase (EC 6.3.1.5) / Glutamine amidotransferase chain of NAD synthetase	FIG00000914	isu;NAD_and_NADP_cofactor_biosynthesis_global isu;NAD_and_NADP_cofactor_biosynthesis_global
fig|6666666.28482.peg.5238	Contig_7	30106	28334	-	PE-PGRS family protein		idu(16);PPE_gene_cluster_in_Mycobacteria
fig|6666666.28482.peg.5239	Contig_7	31685	31140	-	hypothetical protein	FIG00638284	ff
fig|6666666.28482.peg.5240	Contig_7	32245	31685	-	3-isopropylmalate dehydratase small subunit (EC 4.2.1.33)	FIG00000282	idu(1);Branched-Chain_Amino_Acid_Biosynthesis idu(1);Leucine_Biosynthesis
fig|6666666.28482.peg.5241	Contig_7	33489	32242	-	3-isopropylmalate dehydratase large subunit (EC 4.2.1.33)	FIG00000280	icw(1);Branched-Chain_Amino_Acid_Biosynthesis icw(1);Leucine_Biosynthesis
fig|6666666.28482.peg.5242	Contig_7	34367	33480	-	Isocitrate lyase (EC 4.1.3.1)	FIG00000818	idu(1);Serine-glyoxylate_cycle idu(1);Glyoxylate_bypass idu(1);Glyoxylate_bypass_cluster
fig|6666666.28482.peg.5243	Contig_7	35808	34378	-	FIG00828468: hypothetical protein	FIG01457983	ff
fig|6666666.28482.peg.5244	Contig_7	35927	36703	+	Transcriptional regulator, GntR family	FIG00621925	ff
fig|6666666.28482.peg.5245	Contig_7	37367	36693	-	Putative protein-S-isoprenylcysteine methyltransferase	FIG01318516	ff
fig|6666666.28482.peg.5246	Contig_7	38260	37364	-	Ribokinase (EC 2.7.1.15)	FIG00028476	isu;D-ribose_utilization isu;Deoxyribose_and_Deoxynucleoside_Catabolism
fig|6666666.28482.peg.5247	Contig_7	38416	40635	+	Adenylate cyclase (EC 4.6.1.1)	FIG00001294	idu(25);cAMP_signaling_in_bacteria

fig|6666666.28482.peg.5248	Contig_7	40635	42038	+	Small-conductance mechanosensitive channel membrane protein	FIG00821874	ff
fig|6666666.28482.peg.5249	Contig_7	42085	43332	+	Gamma-glutamyl phosphate reductase (EC 1.2.1.41)	FIG00000365	isu;Proline_Synthesis
fig|6666666.28482.peg.5250	Contig_7	43361	44236	+	MoxR-like ATPases	FIG01225784	ff
fig|6666666.28482.peg.5251	Contig_7	44236	45681	+	VWA containing CoxE family protein		ff
fig|6666666.28482.peg.5252	Contig_7	45820	46479	+	Nicotinate-nucleotide adenylyltransferase (EC 2.7.7.18)	FIG01956071	isu;NAD_and_NADP_cofactor_biosynthesis_global
fig|6666666.28482.peg.5253	Contig_7	46476	46871	+	Iojap protein	FIG00135315	ff
fig|6666666.28482.peg.5254	Contig_7	46868	47542	+	Phosphoglycerate mutase (EC 5.4.2.1)	FIG00005840	idu(2);Phosphoglycerate_mutase_protein_family
fig|6666666.28482.peg.5255	Contig_7	47532	48275	+	FIG00999365: hypothetical protein	FIG00821116	ff
fig|6666666.28482.peg.5256	Contig_7	48334	49170	+	Hypothetical protein DUF194, DegV family	FIG00005590	ff
fig|6666666.28482.peg.5257	Contig_7	52074	49153	-	Transmembrane transport protein MmpL4	FIG01448110	ff
fig|6666666.28482.peg.5258	Contig_7	52463	52071	-	membrane protein, MmpS family	FIG01309473	ff
fig|6666666.28482.peg.5259	Contig_7	53711	52653	-	FIG00830683: hypothetical protein	FIG00830679	ff

fig|6666666.28482.peg.5260	Contig_7	55574	53826	-	Long-chain-fatty-acid--CoA ligase (EC 6.2.1.3)	FIG00018699	idu(72);n-Phenylalkanoic_acid_degradation idu(72);Fatty_acid_metabolism_cluster idu(72);Biotin_synthesis_cluster idu(72);Biotin_biosynthesis
fig|6666666.28482.peg.5261	Contig_7	56698	55619	-	POSSIBLE EXPORTED PROTEIN	FIG01386146	ff
fig|6666666.28482.peg.5262	Contig_7	59814	56803	-	membrane protein, MmpL family, putative	FIG00822041	ff
fig|6666666.28482.peg.5263	Contig_7	61332	59890	-	POSSIBLE CONSERVED POLYKETIDE SYNTHASE ASSOCIATED PROTEIN PAPA2	FIG01352858	ff
fig|6666666.28482.peg.5264	Contig_7	61765	72948	+	Malonyl CoA-acyl carrier protein transacylase (EC 2.3.1.39)	FIG00000319	idu(7);Fatty_Acid_Biosynthesis_FASII
fig|6666666.28482.peg.5265	Contig_7	73326	72961	-	FIG00821649: hypothetical protein	FIG00821646	ff
fig|6666666.28482.peg.5266	Contig_7	74513	73407	-	Acyl-CoA dehydrogenase family protein	FIG01288640	ff
fig|6666666.28482.peg.5267	Contig_7	75681	74521	-	Putative acyl-CoA dehydrogenase oxidoreductase (EC 1.3.99.3), may be related to NAD-dependent histone acetylation	FIG00001345	isu;CBSS-216591.1.peg.168
fig|6666666.28482.peg.5268	Contig_7	76054	75821	-	hypothetical protein		

fig|6666666.28482.peg.5269	Contig_7	76451	76041	-	FIG00824120: hypothetical protein	FIG01319003	ff
fig|6666666.28482.peg.5270	Contig_7	76588	77439	+	Putative competence protein ComEA	FIG00996743	ff
fig|6666666.28482.peg.5271	Contig_7	77445	79001	+	DNA internalization-related competence protein ComEC/Rec2	FIG01259681	ff
fig|6666666.28482.peg.5272	Contig_7	79028	79996	+	DNA polymerase III delta subunit (EC 2.7.7.7)	FIG00106663	isu;DNA-replication
fig|6666666.28482.peg.5273	Contig_7	80280	80020	-	SSU ribosomal protein S20p	FIG00000213	ff
fig|6666666.28482.peg.5274	Contig_7	80487	82133	+	Protein containing domains DUF404, DUF407	FIG00537781	ff
fig|6666666.28482.peg.5275	Contig_7	82133	83110	+	Protein containing domains DUF403	FIG00537369	ff
fig|6666666.28482.peg.5276	Contig_7	83110	83949	+	Protein containing transglutaminase-like domain, putative cysteine protease	FIG00453060	ff
fig|6666666.28482.peg.5277	Contig_70	165	1079	+	methyltransferase	FIG01346564	ff
fig|6666666.28482.peg.5278	Contig_70	1093	2280	+	L-carnitine dehydratase/bile acid-inducible protein F (EC 2.8.3.16)	FIG00450481	ff
fig|6666666.28482.peg.5279	Contig_70	2726	2277	-	Serine phosphatase RsbU, regulator of sigma subunit	FIG00017431	idu(4);SigmaB_stress_responce_regulation idu(4);Sigma-B_stress_response_cluster_1

fig|6666666.28482.peg.5280	Contig_70	4287	2701	-	Phytochrome, two-component sensor histidine kinase (EC 2.7.3.-); cyanobacterial phytochrome 1	FIG00144104	isu;Oxidative_stress isu;Oxygen_and_light_sensor_PpaA-PpsR
fig|6666666.28482.peg.5281	Contig_70	5450	4284	-	FIG00830295: hypothetical protein	FIG00830288	ff
fig|6666666.28482.peg.5282	Contig_70	6836	5667	-	Butyryl-CoA dehydrogenase (EC 1.3.99.2)	FIG00009149	idu(39);Isoleucine_degradation idu(39);Lysine_fermentation idu(39);Butanol_Biosynthesis idu(39);Acetyl-CoA_fermentation_to_Butyrate idu(39);Valine_degradation idu(39);Anaerobic_respiratory_reductases
fig|6666666.28482.peg.5283	Contig_70	7428	6862	-	Phosphoribosylaminoimidazole carboxylase catalytic subunit (EC 4.1.1.21)	FIG00000227	isu;De_Novo_Purine_Biosynthesis
fig|6666666.28482.peg.5284	Contig_70	8695	7415	-	Phosphoribosylaminoimidazole carboxylase ATPase subunit (EC 4.1.1.21)	FIG00000375	icw(1);De_Novo_Purine_Biosynthesis
fig|6666666.28482.peg.5285	Contig_70	8705	9364	+	FIG00821069: hypothetical protein	FIG00821068	ff
fig|6666666.28482.peg.5286	Contig_70	9837	9319	-	FIG00995613: hypothetical protein	FIG00820371	ff
fig|6666666.28482.peg.5287	Contig_70	10672	9848	-	Biotin-protein ligase (EC 6.3.4.15)	FIG00000972	isu;Biotin_synthesis_cluster isu;Biotin_biosynthesis

fig|6666666.28482.peg.5288	Contig_70	10723	12363	+	Methylcrotonyl-CoA carboxylase carboxyl transferase subunit (EC 6.4.1.4)	FIG00138249	idu(2);Serine-glyoxylate_cycle idu(2);HMG_CoA_Synthesis idu(2);Leucine_Degradation_and_HMG-CoA_Metabolism
fig|6666666.28482.peg.5289	Contig_70	12360	12578	+	FIG00820143: hypothetical protein	FIG00820141	ff
fig|6666666.28482.peg.5290	Contig_70	12575	13219	+	Septum formation protein Maf	FIG00021845	isu;Bacterial_Cell_Division isu;Bacterial_Cytoskeleton
fig|6666666.28482.peg.5291	Contig_70	13321	14211	+	Thiosulfate sulfurtransferase, rhodanese (EC 2.8.1.1)	FIG00004487	ff
fig|6666666.28482.peg.5292	Contig_70	14211	14630	+	Sulfur acceptor protein SufE for iron-sulfur cluster assembly	FIG00073306	isu;Iron-sulfur_cluster_assembly
fig|6666666.28482.peg.5293	Contig_70	14787	16547	+	Biotin carboxylase of acetyl-CoA carboxylase (EC 6.3.4.14) / Biotin carboxyl carrier protein of acetyl-CoA carboxylase	FIG00002877	isu;Fatty_Acid_Biosynthesis_FASII isu;Fatty_Acid_Biosynthesis_FASII
fig|6666666.28482.peg.5294	Contig_70	16744	17157	+	Anti-sigma F factor antagonist (spoIIAA-2); Anti-sigma B factor antagonist RsbV	FIG00507769	icw(2);SigmaB_stress_responce_regulation icw(2);Sigma-B_stress_response_cluster_1

fig|6666666.28482.peg.5295	Contig_70	17916	17128	-	RNA polymerase sigma factor SigB	FIG00132885	icw(1);SigmaB_stress_responce_regulation idu(2);Biofilm_formation_in_Staphylococcus idu(2);Sigma-B_stress_response_cluster_1 idu(2);Transcription_initiation,_bacterial_sigma_factors idu(2);Methicillin_resistance_in_Staphylococci
fig|6666666.28482.peg.5296	Contig_70	18350	17913	-	Serine-protein kinase RsbW (EC 2.7.11.1)	FIG00002196	isu;SigmaB_stress_responce_regulation icw(1);Sigma-B_stress_response_cluster_1
fig|6666666.28482.peg.5297	Contig_70	18626	18982	+	hypothetical protein		
fig|6666666.28482.peg.5298	Contig_70	19002	19313	+	UsfY protein		
fig|6666666.28482.peg.5299	Contig_70	19650	19324	-	Possible transmembrane protein	FIG01322657	ff
fig|6666666.28482.peg.5300	Contig_70	21138	19825	-	Probable L-lysine-epsilon aminotransferase (EC 2.6.1.36) (L-lysine aminotransferase) (Lysine 6-aminotransferase)	FIG00598383	ff
fig|6666666.28482.peg.5301	Contig_70	21644	21192	-	Leucine-responsive regulatory protein, regulator for leucine (or lrp) regulon and high-affinity branched-chain amino acid transport system	FIG00003800	isu;Glutamine,_Glutamate,_Aspartate_and_Asparagine_Biosynthesis isu;Branched-Chain_Amino_Acid_Biosynthesis

fig|6666666.28482.peg.5302	Contig_70	21687	22925	+	Uncharacterized protein Rv3292/MT3391	FIG00546464	ff
fig|6666666.28482.peg.5303	Contig_70	22922	24451	+	Aldehyde dehydrogenase B (EC 1.2.1.22)	FIG00138741	isu;Glycerolipid_and_Glycerophospholipid_Metabolism_in_Bacteria isu;Methylglyoxal_Metabolism
fig|6666666.28482.peg.5304	Contig_70	24626	25162	+	Transcriptional regulator, TetR family	FIG00554417	ff
fig|6666666.28482.peg.5305	Contig_70	25212	29804	+	Probable ATP-dependent helicase lhr (EC 3.6.1.-)	FIG01319231	ff
fig|6666666.28482.peg.5306	Contig_70	29808	30572	+	Formamidopyrimidine-DNA glycosylase (EC 3.2.2.23)	FIG00035634	idu(3);DNA_Repair_Base_Excision
fig|6666666.28482.peg.5307	Contig_70	30630	30950	+	hypothetical protein		
fig|6666666.28482.peg.5308	Contig_70	32084	30945	-	Phosphate ABC transporter, periplasmic phosphate-binding protein PstS (TC 3.A.1.7.1)	FIG00010508	idu(4);High_affinity_phosphate_transporter_and_control_of_PHO_regulon idu(4);Phosphate_metabolism idu(4);PhoR-PhoB_two-component_regulatory_system
fig|6666666.28482.peg.5309	Contig_70	32995	32099	-	POSSIBLE ESTERASE LIPOPROTEIN LPQC	FIG01332291	ff
fig|6666666.28482.peg.5310	Contig_70	33160	33513	+	hypothetical protein		
fig|6666666.28482.peg.5311	Contig_70	33665	33976	+	FIG00826668: hypothetical protein	FIG00826666	ff
fig|6666666.28482.peg.5312	Contig_70	34133	34303	+	hypothetical protein		

fig|6666666.28482.peg.5313	Contig_70	36493	34310	-	Arylsulfatase (EC 3.1.6.1)	FIG00016566	idu(3);Sulfatases_and_sulfatase_modifying_factor_1 idu(3);Galactosylceramide_and_Sulfatide_metabolism idu(3);Steroid_sulfates idu(3);Alkanesulfonate_assimilation
fig|6666666.28482.peg.5314	Contig_71	269	499	+	Iron-sulphur-binding reductase	FIG01326448	ff
fig|6666666.28482.peg.5315	Contig_71	836	513	-	transcriptional modulator of MazE/toxin, MazF		
fig|6666666.28482.peg.5316	Contig_71	982	833	-	hypothetical protein		
fig|6666666.28482.peg.5317	Contig_71	1071	2417	+	Aspartate aminotransferase (EC 2.6.1.1)	FIG00719736	isu;Coenzyme_M_biosynthesis_--_gjo isu;Glutamine,_Glutamate,_Aspartate_and_Asparagine_Biosynthesis isu;CBSS-216591.1.peg.168 isu;Threonine_and_Homoserine_Biosynthesis
fig|6666666.28482.peg.5318	Contig_71	2676	3254	+	hypothetical protein		
fig|6666666.28482.peg.5319	Contig_71	3321	4640	+	FIG00821472: hypothetical protein	FIG00821471	ff
fig|6666666.28482.peg.5320	Contig_71	5854	5477	-	FIG00821425: hypothetical protein	FIG00821424	ff
fig|6666666.28482.peg.5321	Contig_71	6629	5874	-	FIG00822836: hypothetical protein	FIG00822835	ff

fig|6666666.28482.peg.5322	Contig_72	2167	23	-	Enoyl-CoA hydratase [isoleucine degradation] (EC 4.2.1.17) / 3-hydroxyacyl-CoA dehydrogenase (EC 1.1.1.35) / 3-hydroxybutyryl-CoA epimerase (EC 5.1.2.3)	FIG01303838	isu;n-Phenylalkanoic_acid_degradation isu;n-Phenylalkanoic_acid_degradation isu;Polyhydroxybutyrate_metabolism isu;Polyhydroxybutyrate_metabolism isu;Isoleucine_degradation isu;Isoleucine_degradation isu;Fatty_acid_metabolism_cluster isu;Fatty_acid_metabolism_cluster isu;Acetyl-CoA_fermentation_to_Butyrate isu;Acetyl-CoA_fermentation_to_Butyrate idu(4);Valine_degradation idu(4);Butyrate_metabolism_cluster
fig|6666666.28482.peg.5323	Contig_72	3383	2172	-	3-ketoacyl-CoA thiolase [isoleucine degradation] (EC 2.3.1.16)	FIG01304912	icw(1);Isoleucine_degradation
fig|6666666.28482.peg.5324	Contig_72	3743	4915	+	putative aminotransferase	FIG00830125	ff
fig|6666666.28482.peg.5325	Contig_72	5336	4884	-	FIG00822577: hypothetical protein	FIG00822575	ff
fig|6666666.28482.peg.5326	Contig_72	5795	5340	-	FIG00822577: hypothetical protein	FIG00822575	ff
fig|6666666.28482.peg.5327	Contig_72	6987	5854	-	Alpha-methylacyl-CoA racemase (EC 5.1.99.4)	FIG01312689	ff

fig|6666666.28482.peg.5328	Contig_72	7436	6993	-	FIG00823411: hypothetical protein	FIG00823410	ff
fig|6666666.28482.peg.5329	Contig_72	7496	9187	+	Pyruvate decarboxylase (EC 4.1.1.1); Alpha-keto-acid decarboxylase (EC 4.1.1.-)	FIG00001998	idu(2);Pyruvate_metabolism_II:_acetyl-CoA,_acetogenesis_from_pyruvate
fig|6666666.28482.peg.5330	Contig_72	9369	9184	-	FIG00823260: hypothetical protein	FIG00823257	ff
fig|6666666.28482.peg.5331	Contig_72	9981	9379	-	Long-chain fatty-acid-CoA ligase (EC 6.2.1.3), Mycobacterial subgroup FadD16	FIG00820609	ff
fig|6666666.28482.peg.5332	Contig_72	10669	10016	-	Uncharacterized protein Rv1362c/MT1407	FIG00825912	ff
fig|6666666.28482.peg.5333	Contig_72	11607	10666	-	POSSIBLE MEMBRANE PROTEIN	FIG00692835	ff
fig|6666666.28482.peg.5334	Contig_72	13325	11913	-	Long-chain-fatty-acid--CoA ligase (EC 6.2.1.3)	FIG00018699	idu(72);n-Phenylalkanoic_acid_degradation idu(72);Fatty_acid_metabolism_cluster idu(72);Biotin_synthesis_cluster idu(72);Biotin_biosynthesis
fig|6666666.28482.peg.5335	Contig_72	14552	13362	-	BarH	FIG00668014	ff
fig|6666666.28482.peg.5336	Contig_72	15052	14642	-	hypothetical protein		
fig|6666666.28482.peg.5337	Contig_72	15181	15927	+	FIG00660553: hypothetical protein	FIG00824217	ff
fig|6666666.28482.peg.5338	Contig_72	16969	16268	-	Transcriptional regulator, TetR family	FIG00554417	ff
fig|6666666.28482.peg.5339	Contig_72	17160	18437	+	putative cytochrome P450 hydroxylase	FIG00032437	idu(49);Nitric_oxide_synthase

fig|6666666.28482.peg.5340	Contig_72	19475	18534	-	putative hydrolase		
fig|6666666.28482.peg.5341	Contig_73	246	836	+	Transcriptional regulator, TetR family	FIG00554417	ff
fig|6666666.28482.peg.5342	Contig_73	1531	2715	+	transposase		
fig|6666666.28482.rna.47	Contig_73	2837	2764	-	tRNA-Met-CAT		
fig|6666666.28482.peg.5343	Contig_73	3514	2903	-	hypothetical protein		
fig|6666666.28482.peg.5344	Contig_73	4652	3507	-	Glycerate kinase (EC 2.7.1.31)	FIG00000456	icw(1);Glycerate_metabolism idu(1);Serine-glyoxylate_cycle idu(1);Allantoin_Utilization idu(1);Glycerolipid_and_Glycerophospholipid_Metabolism_in_Bacteria idu(1);Photorespiration_(oxidative_C2_cycle) idu(1);D-galactarate,_D-glucarate_and_D-glycerate_catabolism idu(1);D-galactarate,_D-glucarate_and_D-glycerate_catabolism_-_gjo
fig|6666666.28482.peg.5345	Contig_73	6340	4652	-	L-lactate permease	FIG00016061	idu(1);Lactate_utilization
fig|6666666.28482.peg.5346	Contig_73	7711	6341	-	oxidoreductase, FAD-binding	FIG01339311	ff
fig|6666666.28482.peg.5347	Contig_73	7963	8823	+	Hydroxypyruvate isomerase (EC 5.3.1.22)	FIG00137389	isu;Glycerate_metabolism
fig|6666666.28482.peg.5348	Contig_73	8820	9704	+	2-hydroxy-3-oxopropionate reductase (EC 1.1.1.60)	FIG00133229	icw(1);Glycerate_metabolism icw(1);Allantoin_Utilization idu(1);Photorespiration_(oxidative_C2_cycle) idu(1);D-galactarate,_D-glucarate_and_D-glycerate_catabolism idu(1);D-galactarate,_D-glucarate_and_D-glycerate_catabolism_-_gjo

fig|6666666.28482.peg.5349	Contig_73	9709	11475	+	Glyoxylate carboligase (EC 4.1.1.47)	FIG00002065	icw(2);Glycerate_metabolism icw(2);Allantoin_Utilization icw(1);Photorespiration_(oxidative_C2_cycle)
fig|6666666.28482.peg.5350	Contig_73	11605	12336	+	Negative regulator of allantoin and glyoxylate utilization operons	FIG00005199	isu;Allantoin_Utilization
fig|6666666.28482.peg.5351	Contig_73	12594	12343	-	hypothetical protein		
fig|6666666.28482.peg.5352	Contig_73	12625	13365	+	Short-chain dehydrogenase/reductase SDR	FIG00546160	idu(13);CBSS-164757.7.peg.5020 idu(13);Transcription_repair_cluster
fig|6666666.28482.peg.5353	Contig_73	14132	16879	+	hypothetical protein	FIG00638284	ff
fig|6666666.28482.peg.5354	Contig_73	16987	19731	+	regulatory protein		
fig|6666666.28482.peg.5355	Contig_73	20020	22743	+	putative regulatory protein	FIG01332075	ff
fig|6666666.28482.peg.5356	Contig_73	22915	23511	+	metal-dependent phosphohydrolase		
fig|6666666.28482.peg.5357	Contig_73	23525	24196	+	nitroreductase family protein		
fig|6666666.28482.peg.5358	Contig_73	24973	24296	-	RNA polymerase sigma factor RpoE		isu;Transcription_initiation,_bacterial_sigma_factors
fig|6666666.28482.peg.5359	Contig_74	249	722	+	Transcriptional regulator, HxlR family	FIG00906075	ff
fig|6666666.28482.peg.5360	Contig_74	727	879	+	hypothetical protein		
fig|6666666.28482.peg.5361	Contig_74	1157	1041	-	hypothetical protein		
fig|6666666.28482.peg.5362	Contig_74	1278	1481	+	hypothetical protein		
fig|6666666.28482.peg.5363	Contig_74	2626	2492	-	hypothetical protein		
fig|6666666.28482.peg.5364	Contig_74	2975	2823	-	hypothetical protein		
fig|6666666.28482.peg.5365	Contig_74	2988	4478	+	Amidase(EC:3.5.1.4)		
fig|6666666.28482.peg.5366	Contig_74	5936	5556	-	Death on curing protein, Doc toxin	FIG00021347	ff
fig|6666666.28482.peg.5367	Contig_74	6121	5933	-	hypothetical protein		

fig|6666666.28482.peg.5368	Contig_74	6433	6161	-	hypothetical protein		
fig|6666666.28482.peg.5369	Contig_74	6539	7222	+	Methyltransferase (EC 2.1.1.-)	FIG00515945	ff
fig|6666666.28482.peg.5370	Contig_74	7844	7284	-	transcriptional regulator, TetR family		
fig|6666666.28482.peg.5371	Contig_74	7908	8951	+	Aldo-keto reductase	FIG00639501	ff
fig|6666666.28482.peg.5372	Contig_74	9750	9172	-	hypothetical protein		
fig|6666666.28482.peg.5373	Contig_74	10428	9766	-	Transcriptional regulator, TetR family	FIG00628138	ff
fig|6666666.28482.peg.5374	Contig_74	10579	10866	+	hypothetical protein		
fig|6666666.28482.peg.5375	Contig_74	11539	10874	-	nitroreductase family protein	FIG01312078	ff
fig|6666666.28482.peg.5376	Contig_74	12252	11563	-	hypothetical protein Rv2227	FIG01321447	ff
fig|6666666.28482.peg.5377	Contig_74	12788	12276	-	Methylated-DNA--protein-cysteine methyltransferase (EC 2.1.1.63)	FIG00000328	icw(1);DNA_repair,_bacterial
fig|6666666.28482.peg.5378	Contig_74	12923	13429	+	RNA polymerase sigma-54 factor RpoN	FIG00006092	idu(2);Flagellar_motility idu(2);Flagellum idu(2);Transcription_initiation,_bacterial_sigma_factors
fig|6666666.28482.peg.5379	Contig_74	13426	14031	+	Methylated-DNA--protein-cysteine methyltransferase (EC 2.1.1.63)	FIG00000328	icw(1);DNA_repair,_bacterial
fig|6666666.28482.peg.5380	Contig_74	15460	14099	-	Aldehyde dehydrogenase	FIG00991844	ff
fig|6666666.28482.peg.5381	Contig_74	15546	16472	+	FIG00821997: hypothetical protein	FIG00821996	ff
fig|6666666.28482.peg.5382	Contig_74	16533	17129	+	Transcriptional regulator, TetR family	FIG00554417	ff
fig|6666666.28482.peg.5383	Contig_74	17318	17190	-	hypothetical protein		

fig|6666666.28482.peg.5384	Contig_74	17368	17802	+	membrane protein, MmpS family	FIG01309473	ff
fig|6666666.28482.peg.5385	Contig_74	17799	20693	+	Putative membrane protein	FIG01056920	ff
fig|6666666.28482.peg.5386	Contig_74	21434	20706	-	3-alpha-(or 20-beta)-hydroxysteroid dehydrogenase(EC:1.1.1.53)	FIG01550593	ff
fig|6666666.28482.peg.5387	Contig_74	21882	21601	-	hypothetical protein		
fig|6666666.28482.peg.5388	Contig_74	23040	22165	-	FIG00824485: hypothetical protein	FIG00824483	ff
fig|6666666.28482.peg.5389	Contig_74	24284	23037	-	FIG00822014: hypothetical protein	FIG01547276	ff
fig|6666666.28482.peg.5390	Contig_74	24737	24372	-	FIG00986611: hypothetical protein	FIG00986610	ff
fig|6666666.28482.peg.5391	Contig_74	25777	24734	-	Alcohol dehydrogenase (EC 1.1.1.1)	FIG01007502	idu(17);Glycerolipid_and_Glycerophospholipid_Metabolism_in_Bacteria idu(17);Fermentations:_Mixed_acid idu(17);Butanol_Biosynthesis
fig|6666666.28482.peg.5392	Contig_74	25918	26724	+	TesB-like acyl-CoA thioesterase 2	FIG00149128	isu;Acyl-CoA_thioesterase_II
fig|6666666.28482.peg.5393	Contig_74	26747	27283	+	FIG00822959: hypothetical protein	FIG01321492	ff
fig|6666666.28482.peg.5394	Contig_74	27291	28385	+	FIG00831889: hypothetical protein	FIG00831883	ff
fig|6666666.28482.peg.5395	Contig_74	29372	28389	-	Dihydroflavonol-4-reductase (EC 1.1.1.219)	FIG00042188	idu(2);Biflavanoid_biosynthesis idu(2);Tannin_biosynthesis
fig|6666666.28482.peg.5396	Contig_74	30352	29372	-	Aldo-keto reductase	FIG00639501	ff
fig|6666666.28482.peg.5397	Contig_74	30399	31742	+	FIG00821779: hypothetical protein	FIG00821778	ff
fig|6666666.28482.peg.5398	Contig_74	31934	32182	+	hypothetical protein		

fig|6666666.28482.peg.5399	Contig_74	32294	32932	+	Transcriptional regulator, TetR family	FIG00554417	ff
fig|6666666.28482.peg.5400	Contig_74	33503	34297	+	Conserved hypothetical integral membrane protein YrbE1A	FIG00690583	idu(7);Mycobacterium_virulence_operon_MCE_involved_in_cell_invasion
fig|6666666.28482.peg.5401	Contig_74	34297	35169	+	Conserved hypothetical integral membrane protein YrbE1B	FIG00691621	icw(2);Mycobacterium_virulence_operon_MCE_involved_in_cell_invasion
fig|6666666.28482.peg.5402	Contig_74	35176	36501	+	MCE-family protein Mce1A	FIG00820494	icw(2);Mycobacterium_virulence_operon_MCE_involved_in_cell_invasion
fig|6666666.28482.peg.5403	Contig_74	36498	37538	+	MCE-family protein Mce1B	FIG00763551	icw(5);Mycobacterium_virulence_operon_MCE_involved_in_cell_invasion
fig|6666666.28482.peg.5404	Contig_74	37535	39094	+	MCE-family protein Mce1C	FIG00821293	icw(7);Mycobacterium_virulence_operon_MCE_involved_in_cell_invasion
fig|6666666.28482.peg.5405	Contig_74	39142	40659	+	MCE-family protein Mce1D	FIG00820669	icw(1);Mycobacterium_virulence_operon_MCE_involved_in_cell_invasion
fig|6666666.28482.peg.5406	Contig_74	40656	41834	+	MCE-family lipoprotein LprK (MCE-family lipoprotein Mce1e)	FIG00819998	icw(2);Mycobacterium_virulence_operon_MCE_involved_in_cell_invasion
fig|6666666.28482.peg.5407	Contig_74	41828	43378	+	MCE-family protein Mce1F	FIG00821171	idu(7);Mycobacterium_virulence_operon_MCE_involved_in_cell_invasion
fig|6666666.28482.peg.5408	Contig_74	43634	44131	+	hypothetical protein		
fig|6666666.28482.peg.5409	Contig_74	44244	44465	+	hypothetical protein	FIG00638284	ff
fig|6666666.28482.peg.5410	Contig_74	45115	44462	-	Transcriptional regulator, TetR family	FIG00554417	ff

fig|6666666.28482.peg.5411	Contig_74	45181	46344	+	2,3-dihydroxybiphenyl 1,2-dioxygenase	FIG00004009	ff
fig|6666666.28482.peg.5412	Contig_74	46341	47285	+	Fumarylacetoacetate hydrolase family protein	FIG00007658	idu(1);Gentisare_degradation idu(1);Pterin_carbinolamine_dehydratase idu(1);Salicylate_and_gentisate_catabolism
fig|6666666.28482.peg.5413	Contig_74	47282	48808	+	3-(3-hydroxy-phenyl)propionate hydroxylase (EC 1.14.13.-)	FIG00138127	idu(3);Cinnamic_Acid_Degradation
fig|6666666.28482.peg.5414	Contig_74	49770	48895	-	Universal stress protein family	FIG00017615	ff
fig|6666666.28482.peg.5415	Contig_74	50874	49951	-	FIG00822668: hypothetical protein	FIG00822666	ff
fig|6666666.28482.peg.5416	Contig_74	51140	50871	-	FIG00829490: hypothetical protein	FIG00829489	ff
fig|6666666.28482.peg.5417	Contig_74	51766	51137	-	RNA polymerase sigma-54 factor RpoN	FIG00006092	idu(2);Flagellar_motility idu(2);Flagellum idu(2);Transcription_initiation,_bacterial_sigma_factors
fig|6666666.28482.peg.5418	Contig_74	52853	51777	-	Butyryl-CoA dehydrogenase (EC 1.3.99.2)	FIG00009149	idu(39);Isoleucine_degradation idu(39);Lysine_fermentation idu(39);Butanol_Biosynthesis idu(39);Acetyl-CoA_fermentation_to_Butyrate idu(39);Valine_degradation idu(39);Anaerobic_respiratory_reductases
fig|6666666.28482.peg.5419	Contig_74	53420	52896	-	FIG00824308: hypothetical protein	FIG00824306	ff

fig|6666666.28482.peg.5420	Contig_74	54580	53747	-	FIG00824549: hypothetical protein	FIG00824548	ff
fig|6666666.28482.peg.5421	Contig_74	55767	54616	-	Glycogen debranching enzyme (EC 3.2.1.-)	FIG00109688	icw(1);Trehalose_Biosynthesis icw(1);Glycogen_metabolism
fig|6666666.28482.peg.5422	Contig_74	56713	55838	-	Glycogen debranching enzyme (EC 3.2.1.-)	FIG00109688	icw(1);Trehalose_Biosynthesis icw(1);Glycogen_metabolism
fig|6666666.28482.peg.5423	Contig_74	57408	56713	-	rhomboid family protein		
fig|6666666.28482.peg.5424	Contig_74	57681	57968	+	hypothetical protein		
fig|6666666.28482.peg.5425	Contig_74	59052	57973	-	Glycerol-3-phosphate ABC transporter, ATP-binding protein UgpC (TC 3.A.1.1.3)	FIG00027233	idu(1);Glycerol_and_Glycerol-3-phosphate_Uptake_and_Utilization
fig|6666666.28482.peg.5426	Contig_74	59897	59055	-	Probable sugar-transport integral membrane protein ABC transporter	FIG01319682	ff
fig|6666666.28482.peg.5427	Contig_74	60795	59884	-	Probable sugar-transport integral membrane protein ABC transporter	FIG01319682	ff
fig|6666666.28482.peg.5428	Contig_74	62114	60792	-	Probable sugar-binding lipoprotein	FIG01402923	ff
fig|6666666.28482.peg.5429	Contig_74	63099	62104	-	putative sigma factor	FIG01124738	ff
fig|6666666.28482.peg.5430	Contig_74	63260	63628	+	FIG00824060: hypothetical protein	FIG00824059	ff
fig|6666666.28482.peg.5431	Contig_74	64267	63635	-	hypothetical protein	FIG00638284	ff
fig|6666666.28482.peg.5432	Contig_74	65203	64379	-	Putative uncharacterized protein BCG_2061c	FIG01350741	ff

fig|6666666.28482.peg.5433	Contig_74	65772	65203	-	Nicotinamidase (EC 3.5.1.19)	FIG00067300	idu(1);NAD_and_NADP_cofactor_biosynthesis_global idu(1);Redox-dependent_regulation_of_nucleus_processes
fig|6666666.28482.peg.5434	Contig_74	65871	66851	+	FIG00824845: hypothetical protein	FIG00824843	ff
fig|6666666.28482.peg.5435	Contig_74	67436	66858	-	FIG027960: hypothetical protein	FIG01314328	ff
fig|6666666.28482.peg.5436	Contig_74	67838	67449	-	FIG00824223: hypothetical protein	FIG01310830	ff
fig|6666666.28482.peg.5437	Contig_74	68397	67927	-	Gluconokinase (EC 2.7.1.12)	FIG00000861	isu;D-gluconate_and_ketogluconates_metabolism
fig|6666666.28482.peg.5438	Contig_74	70412	68415	-	FIG00822903: hypothetical protein	FIG00822898	ff
fig|6666666.28482.peg.5439	Contig_74	71937	70432	-	Probable carboxylesterase LipT (EC 3.1.1.-)	FIG00825422	ff
fig|6666666.28482.peg.5440	Contig_74	72018	72695	+	Probable lipoprotein lppI	FIG01340654	ff
fig|6666666.28482.peg.5441	Contig_74	72736	73590	+	hypothetical protein		
fig|6666666.28482.peg.5442	Contig_74	74571	73594	-	Short-chain dehydrogenase/reductase SDR	FIG00546160	idu(13);CBSS-164757.7.peg.5020 idu(13);Transcription_repair_cluster
fig|6666666.28482.peg.5443	Contig_74	75650	74568	-	putative cytochrome P450 hydroxylase	FIG00032437	idu(49);Nitric_oxide_synthase
fig|6666666.28482.peg.5444	Contig_74	75876	76505	+	Transcriptional regulator, TetR family	FIG00554417	ff
fig|6666666.28482.peg.5445	Contig_74	77264	76512	-	short-chain dehydrogenase/reductase SDR(EC:1.1.1.275)		

fig|6666666.28482.peg.5446	Contig_74	78400	77327	-	FIG00821560: hypothetical protein	FIG00821559	ff
fig|6666666.28482.peg.5447	Contig_74	79217	78429	-	Oxidoreductase, short-chain dehydrogenase/reductase family	FIG00824296	ff
fig|6666666.28482.peg.5448	Contig_74	79434	80027	+	Transcriptional regulator, TetR family	FIG00554417	ff
fig|6666666.28482.peg.5449	Contig_74	81147	80044	-	FIG00825548: hypothetical protein	FIG00825545	ff
fig|6666666.28482.peg.5450	Contig_74	81422	82132	+	FIG00820766: hypothetical protein	FIG00820765	ff
fig|6666666.28482.peg.5451	Contig_74	82275	83804	+	MorD	FIG01424540	ff
fig|6666666.28482.peg.5452	Contig_74	83801	84583	+	ATPase	FIG01499921	ff
fig|6666666.28482.peg.5453	Contig_74	84599	84727	+	FIG00820854: hypothetical protein	FIG00820853	ff
fig|6666666.28482.peg.5454	Contig_74	84724	85431	+	FIG00822652: hypothetical protein	FIG00822651	ff
fig|6666666.28482.peg.5455	Contig_74	85445	85873	+	FIG00825075: hypothetical protein	FIG00825071	ff
fig|6666666.28482.peg.5456	Contig_74	85921	86925	+	FIG00832047: hypothetical protein	FIG00832041	ff
fig|6666666.28482.peg.5457	Contig_74	86946	87362	+	FIG00826460: hypothetical protein	FIG00826459	ff
fig|6666666.28482.peg.5458	Contig_74	87359	88600	+	putative cytochrome P450 hydroxylase	FIG00032437	icw(2);Nitric_oxide_synthase
fig|6666666.28482.peg.5459	Contig_74	88780	88577	-	hypothetical protein		
fig|6666666.28482.peg.5460	Contig_74	90198	88891	-	putative cytochrome P450 hydroxylase	FIG00032437	icw(2);Nitric_oxide_synthase
fig|6666666.28482.peg.5461	Contig_74	91652	90453	-	putative cytochrome P450 hydroxylase	FIG00032437	icw(3);Nitric_oxide_synthase
fig|6666666.28482.peg.5462	Contig_74	92032	92634	+	Transcriptional regulator, TetR family		

fig|6666666.28482.peg.5463	Contig_74	93301	92744	-	transcriptional regulator, TetR family		
fig|6666666.28482.peg.5464	Contig_74	93428	94324	+	Short chain dehydrogenase	FIG01310822	ff
fig|6666666.28482.peg.5465	Contig_74	94350	94658	+	hypothetical protein		
fig|6666666.28482.peg.5466	Contig_74	94667	95965	+	putative cytochrome P450 hydroxylase	FIG00032437	icw(1);Nitric_oxide_synthase
fig|6666666.28482.peg.5467	Contig_74	98551	96020	-	Phosphoenolpyruvate synthase (EC 2.7.9.2)	FIG00000789	idu(2);Pyruvate_metabolism_I:_anaplerotic_reactions,_PEP
fig|6666666.28482.peg.5468	Contig_74	111073	98591	-	Malonyl CoA-acyl carrier protein transacylase (EC 2.3.1.39)	FIG00000319	idu(7);Fatty_Acid_Biosynthesis_FASII
fig|6666666.28482.peg.5469	Contig_74	111698	111396	-	FIG00822025: hypothetical protein	FIG00822024	ff
fig|6666666.28482.peg.5470	Contig_74	111967	111758	-	hypothetical protein		
fig|6666666.28482.peg.5471	Contig_74	111941	112237	+	FIG00820327: hypothetical protein	FIG00485890	icw(3);Lipid-linked_oligosaccharide_synthesis_related_cluster
fig|6666666.28482.peg.5472	Contig_74	113028	112234	-	Dolichol-phosphate mannosyltransferase (EC 2.4.1.83) in lipid-linked oligosaccharide synthesis cluster	FIG00543846	idu(1);Lipid-linked_oligosaccharide_synthesis_related_cluster
fig|6666666.28482.peg.5473	Contig_74	115016	113025	-	Apolipoprotein N-acyltransferase (EC 2.3.1.-) in lipid-linked oligosaccharide synthesis cluster	FIG01122388	icw(2);Lipid-linked_oligosaccharide_synthesis_related_cluster

fig|6666666.28482.peg.5474	Contig_74	116610	115009	-	Exoenzymes regulatory protein AepA in lipid-linked oligosaccharide synthesis cluster	FIG01310319	icw(1);Lipid-linked_oligosaccharide_synthesis_related_cluster
fig|6666666.28482.peg.5475	Contig_75	34	420	+	Selenocysteine-specific translation elongation factor	FIG00001280	isu;Selenocysteine_metabolism
fig|6666666.28482.peg.5476	Contig_75	2595	421	-	membrane protein-like protein		
fig|6666666.28482.peg.5477	Contig_75	3139	2675	-	Transamidase GatB domain protein	FIG00012764	ff
fig|6666666.28482.peg.5478	Contig_75	3261	4472	+	putative integral membrane protein	FIG00761799	ff
fig|6666666.28482.peg.5479	Contig_75	4485	5138	+	PROBABLE CONSERVED MEMBRANE PROTEIN	FIG00662487	ff
fig|6666666.28482.peg.5480	Contig_75	5162	6277	+	FIG00818282: hypothetical protein	FIG00512629	ff
fig|6666666.28482.peg.5481	Contig_75	6274	7242	+	MoxR-like ATPase	FIG00820883	ff
fig|6666666.28482.peg.5482	Contig_75	7242	8564	+	Cell division protein DivIC (FtsB), stabilizes FtsL against RasP cleavage	FIG00485194	icw(1);Possible_new_toxin-antitoxin_system_including_DivIC icw(1);Bacterial_Cell_Division icw(1);Cell_division-ribosomal_stress_proteins_cluster icw(1);Bacterial_Cytoskeleton icw(1);Stationary_phase_repair_cluster
fig|6666666.28482.peg.5483	Contig_75	9628	8636	-	POSSIBLE CONSERVED TRANSMEMBRANE PROTEIN	FIG01417063	ff

fig|6666666.28482.peg.5484	Contig_75	9674	10573	+	Cell division protein DivIC (FtsB), stabilizes FtsL against RasP cleavage	FIG00485194	icw(1);Possible_new_toxin-antitoxin_system_including_DivIC icw(1);Bacterial_Cell_Division icw(1);Cell_division-ribosomal_stress_proteins_cluster icw(1);Bacterial_Cytoskeleton icw(1);Stationary_phase_repair_cluster
fig|6666666.28482.peg.5485	Contig_75	11093	10704	-	FIG00823559: hypothetical protein	FIG00823558	ff
fig|6666666.28482.peg.5486	Contig_75	11305	12006	+	Transcriptional regulator, PadR family	FIG00002474	idu(4);CBSS-1352.1.peg.856
fig|6666666.28482.peg.5487	Contig_75	13557	12031	-	Glycerol kinase (EC 2.7.1.30)	FIG00075702	isu;Glycerol_and_Glycerol-3-phosphate_Uptake_and_Utilization isu;Glycerolipid_and_Glycerophospholipid_Metabolism_in_Bacteria
fig|6666666.28482.peg.5488	Contig_75	13682	15154	+	FIG00820887: hypothetical protein	FIG00820886	ff
fig|6666666.28482.peg.5489	Contig_75	15162	15851	+	Methyltransferase (EC 2.1.1.-)	FIG00515945	ff
fig|6666666.28482.peg.5490	Contig_75	16994	15876	-	PE-PGRS family protein		idu(16);PPE_gene_cluster_in_Mycobacteria
fig|6666666.28482.peg.5491	Contig_75	18679	17273	-	PPE family protein	FIG00013075	idu(57);CBSS-164757.7.peg.5020
fig|6666666.28482.peg.5492	Contig_75	18808	18692	-	hypothetical protein		
fig|6666666.28482.peg.5493	Contig_75	20118	18976	-	Selenocysteine lyase, CsdB	FIG01319544	ff
fig|6666666.28482.peg.5494	Contig_75	21080	20115	-	ABC transporter ATP-binding protein	FIG01956052	ff
fig|6666666.28482.peg.5495	Contig_75	21775	21077	-	Glutamine amidotransferases class-II	FIG00995765	ff

fig|6666666.28482.peg.5496	Contig_75	23040	21775	-	Serine/threonine kinase	FIG00659720	ff
fig|6666666.28482.peg.5497	Contig_75	24335	23067	-	Similar to Glutamate--cysteine ligase (EC 6.3.2.2), function unknown	FIG00816555	ff
fig|6666666.28482.peg.5498	Contig_75	24306	24509	+	hypothetical protein		
fig|6666666.28482.peg.5499	Contig_75	25156	24503	-	FIG00820305: hypothetical protein	FIG00820304	ff
fig|6666666.28482.peg.5500	Contig_75	25751	25536	-	hypothetical protein		
fig|6666666.28482.peg.5501	Contig_75	26236	25880	-	FIG00820599: hypothetical protein	FIG00820598	ff
fig|6666666.28482.peg.5502	Contig_75	26735	26490	-	hypothetical protein		
fig|6666666.28482.peg.5503	Contig_76	81	242	+	hypothetical protein		
fig|6666666.28482.peg.5504	Contig_76	485	5068	+	Glutamate synthase [NADPH] large chain (EC 1.4.1.13)	FIG00000539	isu;Glutamine,_Glutamate,_Aspartate_and_Asparagine_Biosynthesis isu;Ammonia_assimilation
fig|6666666.28482.peg.5505	Contig_76	5061	6527	+	Glutamate synthase [NADPH] small chain (EC 1.4.1.13)	FIG00007957	icw(1);Glutamine,_Glutamate,_Aspartate_and_Asparagine_Biosynthesis icw(1);Ammonia_assimilation
fig|6666666.28482.peg.5506	Contig_76	6682	6822	+	FIG00820244: hypothetical protein	FIG00820243	ff
fig|6666666.28482.peg.5507	Contig_76	7750	6899	-	Coenzyme F420-dependent oxidoreductase	FIG00015014	idu(3);Anaerobic_respiratory_reductases
fig|6666666.28482.peg.5508	Contig_76	7802	8233	+	FIG00821842: hypothetical protein	FIG00821841	ff
fig|6666666.28482.peg.5509	Contig_76	8325	9266	+	DNA-dependent DNA polymerase beta chain	FIG00826878	ff
fig|6666666.28482.peg.5510	Contig_77	817	425	-	Probable tautomerase yrdN (EC 5.3.2.-)	FIG01659308	ff

fig|6666666.28482.peg.5511	Contig_77	911	1804	+	LysR family transcriptional regulator	FIG01330236	ff
fig|6666666.28482.peg.5512	Contig_77	2644	1871	-	alpha/beta hydrolase fold		
fig|6666666.28482.peg.5513	Contig_77	2762	3094	+	hypothetical protein		
fig|6666666.28482.peg.5514	Contig_77	3091	3639	+	transcriptional regulator, TetR family		
fig|6666666.28482.peg.5515	Contig_77	4493	3636	-	hypothetical protein	FIG00638284	ff
fig|6666666.28482.peg.5516	Contig_77	5112	4513	-	Phosphatidylethanolamine-binding protein	FIG00822182	ff
fig|6666666.28482.peg.5517	Contig_77	5608	5165	-	FIG00826419: hypothetical protein	FIG00826417	ff
fig|6666666.28482.peg.5518	Contig_77	6666	5644	-	Oxidoreductase FadB5	FIG01332653	ff
fig|6666666.28482.peg.5519	Contig_77	7084	6680	-	FIG00821341: hypothetical protein	FIG00821340	ff
fig|6666666.28482.peg.5520	Contig_77	7395	7081	-	Transcriptional regulator, ArsR family	FIG00003845	idu(3);CBSS-1085.1.peg.1363
fig|6666666.28482.peg.5521	Contig_77	7485	8207	+	Fumarylacetoacetase (EC 3.7.1.2)	FIG00001596	idu(1);Homogentisate_pathway_of_aromatic_compound_degradation idu(1);Pterin_carbinolamine_dehydratase idu(1);Salicylate_and_gentisate_catabolism
fig|6666666.28482.peg.5522	Contig_77	8578	8204	-	FIG00820307: hypothetical protein	FIG00820306	ff
fig|6666666.28482.peg.5523	Contig_77	8742	11030	+	Isocitrate lyase (EC 4.1.3.1), group III, Mycobacterial type ICL2	FIG00113839	isu;Glyoxylate_bypass
fig|6666666.28482.peg.5524	Contig_77	11063	11239	+	hypothetical protein		
fig|6666666.28482.peg.5525	Contig_78	3	161	+	hypothetical protein		

fig|6666666.28482.peg.5526	Contig_78	661	236	-	Molybdenum cofactor biosynthesis protein MoaE	FIG00018405	icw(1);Molybdenum_cofactor_biosynthesis
fig|6666666.28482.peg.5527	Contig_78	1140	658	-	Molybdenum cofactor biosynthesis protein MoaB	FIG00032229	icw(2);Molybdenum_cofactor_biosynthesis
fig|6666666.28482.peg.5528	Contig_78	1565	1140	-	Molybdenum cofactor biosynthesis protein MoaC	FIG00053487	isu;Molybdenum_cofactor_biosynthesis
fig|6666666.28482.peg.5529	Contig_78	1853	1644	-	FIG057355: hypothetical protein	FIG01311512	ff
fig|6666666.28482.peg.5530	Contig_78	1950	4202	+	probable DNA-binding protein	FIG00666011	ff
fig|6666666.28482.peg.5531	Contig_78	4264	5913	+	DNA repair helicase	FIG00544450	ff
fig|6666666.28482.peg.5532	Contig_78	7153	6008	-	Nonspecific lipid-transfer protein	FIG01353572	ff
fig|6666666.28482.peg.5533	Contig_78	7633	7220	-	FIG00826442: hypothetical protein	FIG00826438	ff
fig|6666666.28482.peg.5534	Contig_78	7951	8244	+	FIG00821729: hypothetical protein	FIG00821728	ff
fig|6666666.28482.peg.5535	Contig_78	9230	8241	-	Luciferase family protein	FIG00823277	ff
fig|6666666.28482.peg.5536	Contig_78	9325	9567	+	hypothetical protein		
fig|6666666.28482.peg.5537	Contig_78	9609	10817	+	Oxidoreductase	FIG01321859	ff
fig|6666666.28482.peg.5538	Contig_78	10838	11695	+	FIG00825180: hypothetical protein	FIG00825179	ff
fig|6666666.28482.peg.5539	Contig_79	19	243	+	Protein-export membrane protein SecD (TC 3.A.5.1.1)	FIG00001617	ff
fig|6666666.28482.peg.5540	Contig_79	247	1497	+	Protein-export membrane protein SecF (TC 3.A.5.1.1)	FIG00000468	ff

fig|6666666.28482.peg.5541	Contig_79	1503	3161	+	Oligopeptide ABC transporter, periplasmic oligopeptide-binding protein OppA (TC 3.A.1.5.1)	FIG00451095	isu;ABC_transporter_oligopeptide_(TC_3.A.1.5.1) isu;Sex_pheromones_in_Enterococcus_faecalis_and_other_Firmicutes
fig|6666666.28482.peg.5542	Contig_79	3193	3699	+	Adenine phosphoribosyltransferase (EC 2.4.2.7)	FIG00000277	isu;Purine_conversions icw(1);cAMP_signaling_in_bacteria
fig|6666666.28482.peg.5543	Contig_79	3729	6122	+	GTP pyrophosphokinase (EC 2.7.6.5), (p)ppGpp synthetase I	FIG00047414	isu;Stringent_Response,_(p)ppGpp_metabolism
fig|6666666.28482.peg.5544	Contig_79	6487	6119	-	hypothetical protein		
fig|6666666.28482.peg.5545	Contig_79	6634	9810	+	Adenylate cyclase (EC 4.6.1.1)	FIG00001294	idu(25);cAMP_signaling_in_bacteria
fig|6666666.28482.peg.5546	Contig_79	11216	9807	-	PE-PGRS family protein		idu(16);PPE_gene_cluster_in_Mycobacteria
fig|6666666.28482.peg.5547	Contig_79	13602	12142	-	Alpha,alpha-trehalose-phosphate synthase [UDP-forming] (EC 2.4.1.15)	FIG00001100	isu;Trehalose_Biosynthesis
fig|6666666.28482.peg.5548	Contig_79	13731	13603	-	FIG00820754: hypothetical protein	FIG00820753	ff
fig|6666666.28482.peg.5549	Contig_79	15315	13825	-	Amine oxidase [flavin-containing] A (EC 1.4.3.4)		

fig|6666666.28482.peg.5550	Contig_79	15561	16478	+	Enoyl-CoA hydratase (EC 4.2.1.17)	FIG00033206	idu(51);n-Phenylalkanoic_acid_degradation idu(51);Polyhydroxybutyrate_metabolism idu(51);Isoleucine_degradation idu(51);Fatty_acid_metabolism_cluster idu(51);Lysine_fermentation idu(51);Butanol_Biosynthesis idu(51);Acetyl-CoA_fermentation_to_Butyrate icw(1);Valine_degradation idu(51);Butyrate_metabolism_cluster
fig|6666666.28482.peg.5551	Contig_79	16552	17382	+	short chain dehydrogenase	FIG01311525	ff
fig|6666666.28482.peg.5552	Contig_79	18255	17365	-	3-hydroxyisobutyrate dehydrogenase (EC 1.1.1.31)	FIG00021583	idu(3);Valine_degradation
fig|6666666.28482.peg.5553	Contig_79	18321	19112	+	Dehydrogenases with different specificities (related to short-chain alcohol dehydrogenases)	FIG00975245	ff
fig|6666666.28482.peg.5554	Contig_79	20687	19131	-	Cell envelope-associated transcriptional attenuator LytR-CpsA-Psr, subfamily A1 (as in PMID19099556)	FIG00025213	idu(3);Cell_envelope-associated_LytR-CpsA-Psr_transcriptional_attenuators
fig|6666666.28482.peg.5555	Contig_79	21326	20976	-	hypothetical protein		

fig|6666666.28482.peg.5556	Contig_79	21628	21368	-	FIG00823724: hypothetical protein	FIG01487565	ff
fig|6666666.28482.peg.5557	Contig_79	21710	22456	+	FIG00825022: hypothetical protein	FIG00825021	ff
fig|6666666.28482.peg.5558	Contig_79	22636	22779	+	hypothetical protein		
fig|6666666.28482.peg.5559	Contig_79	22808	22984	+	hypothetical protein		
fig|6666666.28482.peg.5560	Contig_79	23046	23918	+	Peroxidase BpoA	FIG01321097	ff
fig|6666666.28482.peg.5561	Contig_79	25422	23896	-	INTEGRAL MEMBRANE PROTEIN (Rhomboid family)	FIG00525068	ff
fig|6666666.28482.peg.5562	Contig_79	26504	25491	-	2,3-dihydroxybiphenyl 1,2-dioxygenase	FIG00004009	ff
fig|6666666.28482.peg.5563	Contig_79	27413	26532	-	2-hydroxy-6-oxo-6-phenylhexa-2,4-dienoate hydrolase (EC 3.7.1.-)	FIG00003780	idu(2);carbazol_degradation_cluster idu(2);Central_meta-cleavage_pathway_of_aromatic_compound_degradation idu(2);Biphenyl_Degradation
fig|6666666.28482.peg.5564	Contig_79	28971	27424	-	3-(3-hydroxy-phenyl)propionate hydroxylase (EC 1.14.13.-)	FIG00138127	idu(3);Cinnamic_Acid_Degradation
fig|6666666.28482.peg.5565	Contig_79	29060	29716	+	Transcriptional regulator, TetR family	FIG00554417	ff
fig|6666666.28482.peg.5566	Contig_79	29713	30510	+	2-oxopent-4-dienoate hydratase	FIG00821141	ff

fig|6666666.28482.peg.5567	Contig_79	30522	31469	+	Acetaldehyde dehydrogenase, acetylating, (EC 1.2.1.10) in gene cluster for degradation of phenols, cresols, catechol	FIG00002495	icw(1);Central_meta-cleavage_pathway_of_aromatic_compound_degradation icw(1);Biphenyl_Degradation
fig|6666666.28482.peg.5568	Contig_79	31472	32488	+	4-hydroxy-2-oxovalerate aldolase (EC 4.1.3.-)	FIG00001725	ff
fig|6666666.28482.peg.5569	Contig_79	33703	32516	-	hypothetical protein		
fig|6666666.28482.peg.5570	Contig_79	33975	34571	+	integral membrane protein		
fig|6666666.28482.peg.5571	Contig_79	34579	34830	+	hypothetical protein		
fig|6666666.28482.peg.5572	Contig_79	34988	35611	+	FIG00826434: hypothetical protein	FIG00826431	ff
fig|6666666.28482.peg.5573	Contig_79	35743	36210	+	FIG00828898: hypothetical protein	FIG00828897	ff
fig|6666666.28482.peg.5574	Contig_79	36255	37124	+	hypothetical protein	FIG00638284	ff
fig|6666666.28482.peg.5575	Contig_79	37227	38006	+	enoyl-CoA dehydratase, EchA8_3		
fig|6666666.28482.peg.5576	Contig_79	38115	39422	+	PE-PGRS family protein		idu(16);PPE_gene_cluster_in_Mycobacteria
fig|6666666.28482.peg.5577	Contig_79	40195	39419	-	hypothetical protein	FIG00638284	ff
fig|6666666.28482.peg.5578	Contig_79	40357	40587	+	hypothetical protein	FIG00638284	ff
fig|6666666.28482.peg.5579	Contig_79	40731	40588	-	hypothetical protein		
fig|6666666.28482.peg.5580	Contig_79	41003	41791	+	serine esterase, cutinase family	FIG01335798	ff
fig|6666666.28482.rna.48	Contig_79	42873	42945	+	tRNA-Thr-TGT		
fig|6666666.28482.peg.5581	Contig_79	43811	43068	-	FIG00820010: hypothetical protein	FIG00820008	ff

fig|6666666.28482.peg.5582	Contig_79	44006	44677	+	DNA-binding response regulator	FIG01031438	ff
fig|6666666.28482.peg.5583	Contig_79	44706	46148	+	PhoR	FIG00822385	ff
fig|6666666.28482.peg.5584	Contig_79	46552	46136	-	HIT family protein	FIG00994683	ff
fig|6666666.28482.peg.5585	Contig_79	46633	47280	+	Dihydrofolate reductase (EC 1.5.1.3)	FIG00000048	idu(3);Folate_Biosynthesis
fig|6666666.28482.peg.5586	Contig_79	47994	47572	-	steroid isomerase, putative	FIG01310317	ff
fig|6666666.28482.peg.5587	Contig_79	49977	48010	-	2,4-dienoyl-CoA reductase [NADPH] (EC 1.3.1.34)	FIG00517072	ff
fig|6666666.28482.peg.5588	Contig_79	51165	50038	-	Alcohol dehydrogenase (EC 1.1.1.1)	FIG01007502	idu(17);Glycerolipid_and_Glycerophospholipid_Metabolism_in_Bacteria idu(17);Fermentations:_Mixed_acid idu(17);Butanol_Biosynthesis
fig|6666666.28482.peg.5589	Contig_79	51873	51319	-	FIG00994119: hypothetical protein	FIG00820531	ff
fig|6666666.28482.peg.5590	Contig_79	52085	51876	-	Ferredoxin	FIG00001676	idu(4);Soluble_cytochromes_and_functionally_related_electron_carriers idu(4);Inorganic_Sulfur_Assimilation
fig|6666666.28482.peg.5591	Contig_79	53453	52098	-	Cytochrome P450 51	FIG01316881	ff
fig|6666666.28482.peg.5592	Contig_79	54274	53450	-	oxidoreductase, short-chain dehydrogenase/reductase family	FIG01312329	ff
fig|6666666.28482.peg.5593	Contig_79	55486	54275	-	putative cytochrome P450 hydroxylase	FIG00032437	idu(49);Nitric_oxide_synthase
fig|6666666.28482.peg.5594	Contig_79	56103	55483	-	Transcriptional regulator, TetR family	FIG00554417	ff

fig|6666666.28482.peg.5595	Contig_79	56288	57757	+	Aldehyde dehydrogenase (EC 1.2.1.3)	FIG01292786	idu(16);Pyruvate_metabolism_II:_acetyl-CoA,_acetogenesis_from_pyruvate idu(16);Glycerolipid_and_Glycerophospholipid_Metabolism_in_Bacteria idu(16);Methylglyoxal_Metabolism
fig|6666666.28482.peg.5596	Contig_79	57767	58516	+	3-oxoacyl-[acyl-carrier protein] reductase (EC 1.1.1.100)	FIG00621114	idu(38);CBSS-246196.1.peg.364 idu(38);CBSS-164757.7.peg.5020 idu(38);Fatty_Acid_Biosynthesis_FASII
fig|6666666.28482.peg.5597	Contig_79	58565	59419	+	3-hydroxyisobutyrate dehydrogenase (EC 1.1.1.31)	FIG00021583	idu(3);Valine_degradation
fig|6666666.28482.peg.5598	Contig_79	59431	59868	+	4-carboxymuconolactone decarboxylase (EC 4.1.1.44)	FIG01304850	idu(2);Protocatechuate_branch_of_beta-ketoadipate_pathway
fig|6666666.28482.peg.5599	Contig_8	114	1079	+	Transcriptional regulator, AraC family		
fig|6666666.28482.peg.5600	Contig_8	1643	1104	-	hypothetical protein		
fig|6666666.28482.peg.5601	Contig_8	2381	1719	-	conserved hypothetical protein		
fig|6666666.28482.peg.5602	Contig_8	2577	2708	+	hypothetical protein		
fig|6666666.28482.peg.5603	Contig_8	3992	2778	-	Carbon monoxide oxidation accessory protein CoxE		isu;Carbon_monoxide_dehydrogenase_maturation_factors
fig|6666666.28482.peg.5604	Contig_8	4661	3999	-	carbon monoxide dehydrogenase G protein	FIG00001673	ff

fig|6666666.28482.peg.5605	Contig_8	5541	4675	-	carbon monoxide dehydrogenase D protein	FIG00029116	ff
fig|6666666.28482.peg.5606	Contig_8	6125	5541	-	Uncharacterized MobA-related protein	FIG00029967	ff
fig|6666666.28482.peg.5607	Contig_8	7021	6122	-	Carbon monoxide dehydrogenase F protein	FIG00006127	ff
fig|6666666.28482.peg.5608	Contig_8	9448	7055	-	Carbon monoxide dehydrogenase form I, large chain(EC:1.2.99.2)	FIG01304852	ff
fig|6666666.28482.peg.5609	Contig_8	9921	9445	-	Carbon monoxide dehydrogenase small chain (EC 1.2.99.2)	FIG00525841	ff
fig|6666666.28482.peg.5610	Contig_8	10805	9924	-	Carbon monoxide dehydrogenase medium chain (EC 1.2.99.2)	FIG00513859	ff
fig|6666666.28482.peg.5611	Contig_8	12006	10837	-	CO dehydrogenases maturation factor, CoxF family	FIG00024587	ff
fig|6666666.28482.peg.5612	Contig_8	12029	12994	+	Transcription regulator in CO-DH cluster	FIG00028984	ff
fig|6666666.28482.peg.5613	Contig_8	13688	13044	-	probable RNA methyltransferase	FIG00945552	ff
fig|6666666.28482.peg.5614	Contig_8	14608	13685	-	hypothetical protein Rv0381c	FIG01349032	ff
fig|6666666.28482.peg.5615	Contig_8	15327	14785	-	Orotate phosphoribosyltransferase (EC 2.4.2.10)	FIG00025759	ff

fig|6666666.28482.peg.5616	Contig_8	16084	15314	-	Probable short-chain dehydrogenase	FIG01324048	ff
fig|6666666.28482.peg.5617	Contig_8	16967	16119	-	Possible membrane protein	FIG01121868	ff
fig|6666666.28482.peg.5618	Contig_8	19692	17146	-	ClpB protein	FIG00025216	idu(1);Proteolysis_in_bacteria,_ATP-dependent idu(1);USS-DB-7 icw(2);Protein_chaperones idu(1);Type_VI_secretion_systems
fig|6666666.28482.peg.5619	Contig_8	19818	20969	+	oxidoreductase, putative	FIG01339212	ff
fig|6666666.28482.peg.5620	Contig_8	20983	21873	+	Aldose 1-epimerase	FIG01334418	ff
fig|6666666.28482.peg.5621	Contig_8	22421	21885	-	hypothetical protein	FIG00638284	ff
fig|6666666.28482.peg.5622	Contig_8	22822	22430	-	HspR, transcriptional repressor of DnaK operon	FIG00002442	isu;Heat_shock_dnaK_gene_cluster_extended icw(1);Protein_chaperones
fig|6666666.28482.peg.5623	Contig_8	24000	22822	-	Chaperone protein DnaJ	FIG00000070	icw(2);Heat_shock_dnaK_gene_cluster_extended icw(3);Protein_chaperones
fig|6666666.28482.peg.5624	Contig_8	24693	24055	-	Heat shock protein GrpE	FIG00000103	icw(3);Heat_shock_dnaK_gene_cluster_extended idu(1);Protein_chaperones
fig|6666666.28482.peg.5625	Contig_8	26558	24690	-	Chaperone protein DnaK	FIG00023369	icw(1);Heat_shock_dnaK_gene_cluster_extended icw(2);Protein_chaperones
fig|6666666.28482.peg.5626	Contig_8	27869	26955	-	LysR-family transcriptional regulator		

fig|6666666.28482.peg.5627	Contig_8	28000	29490	+	Aldehyde dehydrogenase (EC 1.2.1.3)	FIG01292786	idu(16);Pyruvate_metabolism_II:_acetyl-CoA,_acetogenesis_from_pyruvate idu(16);Glycerolipid_and_Glycerophospholipid_Metabolism_in_Bacteria idu(16);Methylglyoxal_Metabolism
fig|6666666.28482.peg.5628	Contig_8	29490	30446	+	amidohydrolase 2		
fig|6666666.28482.peg.5629	Contig_8	30443	31642	+	cytochrome P450		
fig|6666666.28482.peg.5630	Contig_8	31726	33174	+	L-asparagine permease	FIG00554902	ff
fig|6666666.28482.peg.5631	Contig_8	33638	33153	-	CTP:molybdopterin cytidylyltransferase	FIG00007638	isu;Molybdopterin_cytosine_dinucleotide
fig|6666666.28482.peg.5632	Contig_8	33766	36102	+	Carbon monoxide dehydrogenase large chain (EC 1.2.99.2) parolog without usual motifs	FIG01304853	ff
fig|6666666.28482.peg.5633	Contig_8	36099	36998	+	Carbon monoxide dehydrogenase medium chain (EC 1.2.99.2)	FIG00513859	ff
fig|6666666.28482.peg.5634	Contig_8	36991	37476	+	Carbon monoxide dehydrogenase small chain (EC 1.2.99.2)	FIG00525841	ff
fig|6666666.28482.peg.5635	Contig_8	37581	38183	+	Lipoprotein LpqJ	FIG01320124	ff
fig|6666666.28482.peg.5636	Contig_8	41386	38339	-	Adenylate cyclase (EC 4.6.1.1)	FIG00001294	idu(25);cAMP_signaling_in_bacteria
fig|6666666.28482.peg.5637	Contig_8	44259	41623	-	hypothetical protein	FIG00638284	ff
fig|6666666.28482.peg.5638	Contig_8	44659	45156	+	hypothetical protein		

fig|6666666.28482.peg.5639	Contig_8	48365	45171	-	Adenylate cyclase (EC 4.6.1.1)	FIG00001294	idu(25);cAMP_signaling_in_bacteria
fig|6666666.28482.peg.5640	Contig_8	49748	48531	-	Transcriptional regulator, IclR family	FIG00022569	idu(12);Homogentisate_pathway_of_aromatic_compound_degradation
fig|6666666.28482.peg.5641	Contig_8	50020	51615	+	PPE family protein	FIG00013075	idu(57);CBSS-164757.7.peg.5020
fig|6666666.28482.peg.5642	Contig_8	51668	52180	+	hypothetical protein	FIG00638284	ff
fig|6666666.28482.peg.5643	Contig_8	52191	53834	+	FIG00825842: hypothetical protein	FIG00825840	ff
fig|6666666.28482.peg.5644	Contig_80	27	1247	+	Glycosyl transferase, group 1	FIG00018328	ff
fig|6666666.28482.peg.5645	Contig_80	1252	2301	+	dTDP-glucose 4,6-dehydratase (EC 4.2.1.46)		idu(4);dTDP-rhamnose_synthesis idu(4);Rhamnose_containing_glycans
fig|6666666.28482.peg.5646	Contig_80	2310	3950	+	Carbamoyltransferase family protein	FIG01030283	ff
fig|6666666.28482.peg.5647	Contig_81	42	1103	+	PPE family protein	FIG00013075	icw(1);CBSS-164757.7.peg.5020
fig|6666666.28482.peg.5648	Contig_81	1204	2313	+	PPE family protein	FIG00013075	icw(1);CBSS-164757.7.peg.5020
fig|6666666.28482.peg.5649	Contig_81	2421	2702	+	putative esat-6 like protein		
fig|6666666.28482.peg.5650	Contig_81	2714	2998	+	ESAT-6-like protein EsxL	FIG00820465	ff
fig|6666666.28482.peg.5651	Contig_81	4426	3056	-	Amine oxidase [flavin-containing] A (EC 1.4.3.4)	FIG01366019	ff
fig|6666666.28482.peg.5652	Contig_81	5658	4597	-	response regulator receiver protein		
fig|6666666.28482.peg.5653	Contig_81	8358	5740	-	hypothetical protein	FIG00638284	ff
fig|6666666.28482.peg.5654	Contig_81	10809	8368	-	hypothetical protein	FIG00638284	ff
fig|6666666.28482.peg.5655	Contig_81	11668	11111	-	hypothetical protein	FIG00638284	ff
fig|6666666.28482.peg.5656	Contig_81	12138	11701	-	protein of unknown function DUF1486		

fig|6666666.28482.peg.5657	Contig_81	13561	12149	-	hypothetical protein		
fig|6666666.28482.peg.5658	Contig_81	13781	13545	-	hypothetical protein		
fig|6666666.28482.peg.5659	Contig_81	13941	13786	-	hypothetical protein		
fig|6666666.28482.peg.5660	Contig_81	13996	15369	+	2-methylcitrate dehydratase (EC 4.2.1.79)	FIG00001287	idu(1);Methylcitrate_cycle
fig|6666666.28482.peg.5661	Contig_81	15668	15450	-	hypothetical protein		
fig|6666666.28482.peg.5662	Contig_81	15779	17527	+	CONSERVED MEMBRANE PROTEIN	FIG00818075	ff
fig|6666666.28482.peg.5663	Contig_81	19850	17580	-	5-methyltetrahydropteroyl triglutamate--homocysteine methyltransferase (EC 2.1.1.14)	FIG00000683	ff
fig|6666666.28482.peg.5664	Contig_81	19839	20063	+	hypothetical protein		
fig|6666666.28482.peg.5665	Contig_81	21039	20233	-	Cytochrome P450 143		
fig|6666666.28482.peg.5666	Contig_81	21869	21213	-	FIG00824852: hypothetical protein	FIG00824851	ff
fig|6666666.28482.peg.5667	Contig_81	22458	21898	-	Transcriptional regulator, TetR family		
fig|6666666.28482.peg.5668	Contig_81	23572	22523	-	putative oxidoreductase		
fig|6666666.28482.peg.5669	Contig_81	23750	24724	+	putative transcriptional regulator, AraC family		
fig|6666666.28482.peg.5670	Contig_81	24868	26496	+	Choline dehydrogenase (EC 1.1.99.1)	FIG00057416	idu(4);Choline_uptake_and_conversion_to_betaine_clusters idu(4);Choline_and_Betaine_Uptake_and_Betaine_Biosynthesis
fig|6666666.28482.peg.5671	Contig_81	26532	27719	+	putative cytochrome P450 hydroxylase		idu(49);Nitric_oxide_synthase

fig|6666666.28482.peg.5672	Contig_81	28579	27716	-	3-alpha-hydroxysteroid dehydrogenase	FIG01322334	ff
fig|6666666.28482.peg.5673	Contig_81	29926	28637	-	Transcriptional regulator, TetR family	FIG00554417	ff
fig|6666666.28482.peg.5674	Contig_81	30664	30017	-	FIG00822384: hypothetical protein	FIG00822379	ff
fig|6666666.28482.peg.5675	Contig_81	31776	30664	-	Butyryl-CoA dehydrogenase (EC 1.3.99.2)	FIG00009149	icw(1);Isoleucine_degradation icw(1);Lysine_fermentation icw(1);Butanol_Biosynthesis icw(1);Acetyl-CoA_fermentation_to_Butyrate icw(1);Valine_degradation icw(1);Anaerobic_respiratory_reductases
fig|6666666.28482.peg.5676	Contig_81	32918	31773	-	Butyryl-CoA dehydrogenase (EC 1.3.99.2)	FIG00009149	icw(1);Isoleucine_degradation icw(1);Lysine_fermentation icw(1);Butanol_Biosynthesis icw(1);Acetyl-CoA_fermentation_to_Butyrate icw(1);Valine_degradation icw(1);Anaerobic_respiratory_reductases
fig|6666666.28482.peg.5677	Contig_81	33188	34903	+	FIG01284085: hypothetical protein	FIG01284078	ff
fig|6666666.28482.peg.5678	Contig_81	34929	35780	+	Nitric oxide reductase activation protein NorQ	FIG00133744	isu;Denitrification
fig|6666666.28482.peg.5679	Contig_81	35863	36981	+	FIG00820247: hypothetical protein	FIG00820244	ff
fig|6666666.28482.peg.5680	Contig_81	36981	38117	+	FIG00826509: hypothetical protein	FIG00826508	ff

fig|6666666.28482.peg.5681	Contig_81	38110	39090	+	Oxidoreductase, short-chain dehydrogenase/reductase family	FIG00823252	ff
fig|6666666.28482.peg.5682	Contig_81	39090	40307	+	putative cytochrome P450 hydroxylase	FIG00032437	idu(49);Nitric_oxide_synthase
fig|6666666.28482.peg.5683	Contig_81	40399	41883	+	Aldehyde dehydrogenase (EC 1.2.1.3)	FIG01292786	idu(16);Pyruvate_metabolism_II:_acetyl-CoA,_acetogenesis_from_pyruvate idu(16);Glycerolipid_and_Glycerophospholipid_Metabolism_in_Bacteria idu(16);Methylglyoxal_Metabolism
fig|6666666.28482.peg.5684	Contig_81	43426	41957	-	Tectonin-2 (Tectonin II)		

fig|6666666.28482.peg.5685	Contig_81	43776	44930	+	3-ketoacyl-CoA thiolase (EC 2.3.1.16) @ Acetyl-CoA acetyltransferase (EC 2.3.1.9)	FIG00000004	idu(11);CBSS-246196.1.peg.364 idu(9);Serine-glyoxylate_cycle idu(11);Serine-glyoxylate_cycle icw(1);n-Phenylalkanoic_acid_degradation idu(9);Ethylmalonyl-CoA_pathway_of_C2_assimilation,_GJO idu(9);Polyhydroxybutyrate_metabolism icw(1);Polyhydroxybutyrate_metabolism icw(1);Isoleucine_degradation icw(1);Fatty_acid_metabolism_cluster idu(11);Biotin_synthesis_cluster idu(11);Biotin_biosynthesis icw(1);Lysine_fermentation idu(9);Isoprenoid_Biosynthesis icw(1);Butanol_Biosynthesis icw(1);Acetyl-CoA_fermentation_to_Butyrate icw(1);Butyrate_metabolism_cluster

fig|6666666.28482.peg.5686	Contig_81	44927	45730	+	Enoyl-CoA hydratase (EC 4.2.1.17)	FIG00033206	idu(51);n-Phenylalkanoic_acid_degradation icw(1);Polyhydroxybutyrate_metabolism idu(51);Isoleucine_degradation idu(51);Fatty_acid_metabolism_cluster idu(51);Lysine_fermentation idu(51);Butanol_Biosynthesis idu(51);Acetyl-CoA_fermentation_to_Butyrate idu(51);Valine_degradation idu(51);Butyrate_metabolism_cluster
fig|6666666.28482.peg.5687	Contig_81	46798	45782	-	PUTATIVE OXIDOREDUCTASE	FIG01325211	ff
fig|6666666.28482.peg.5688	Contig_81	47295	46795	-	FIG00823635: hypothetical protein	FIG00823634	ff
fig|6666666.28482.peg.5689	Contig_81	48449	47310	-	Chalcone synthase (EC 2.3.1.74)	FIG00443156	ff
fig|6666666.28482.peg.5690	Contig_81	48496	48663	+	hypothetical protein		
fig|6666666.28482.peg.5691	Contig_81	48841	49989	+	FIG00822654: hypothetical protein	FIG00822653	ff
fig|6666666.28482.peg.5692	Contig_81	49986	50708	+	PROBABLE INTEGRAL MEMBRANE PROTEIN	FIG01315135	ff
fig|6666666.28482.peg.5693	Contig_81	52035	50671	-	Nucleoside-diphosphate-sugar epimerase	FIG01366027	ff

fig|6666666.28482.peg.5694	Contig_81	52924	52112	-	Enoyl-CoA hydratase (EC 4.2.1.17)	FIG00033206	idu(51);n-Phenylalkanoic_acid_degradation idu(51);Polyhydroxybutyrate_metabolism idu(51);Isoleucine_degradation idu(51);Fatty_acid_metabolism_cluster idu(51);Lysine_fermentation idu(51);Butanol_Biosynthesis idu(51);Acetyl-CoA_fermentation_to_Butyrate idu(51);Valine_degradation idu(51);Butyrate_metabolism_cluster
fig|6666666.28482.peg.5695	Contig_81	53018	54100	+	Alpha-methylacyl-CoA racemase (EC 5.1.99.4)	FIG01312689	ff
fig|6666666.28482.peg.5696	Contig_81	54144	54896	+	3-hydroxyacyl-CoA dehydrogenase [isoleucine degradation] (EC 1.1.1.35)	FIG01388763	ff
fig|6666666.28482.peg.5697	Contig_81	55158	55027	-	FIG00821293: hypothetical protein	FIG00821291	ff
fig|6666666.28482.peg.5698	Contig_81	55490	57814	+	Transmembrane transport protein MmpL13b	FIG01314334	ff
fig|6666666.28482.peg.5699	Contig_81	57807	58514	+	Transcriptional regulator, TetR family	FIG00554417	ff
fig|6666666.28482.peg.5700	Contig_81	58511	59152	+	Methyltransferase (EC 2.1.1.-)	FIG00515945	ff

fig|6666666.28482.peg.5701	Contig_81	59894	59223	-	NAD-dependent protein deacetylase of SIR2 family	FIG00008490	idu(1);Pyruvate_metabolism_II:_acetyl-CoA,_acetogenesis_from_pyruvate idu(1);Redox-dependent_regulation_of_nucleus_processes
fig|6666666.28482.peg.5702	Contig_81	59980	60345	+	Transcriptional regulator, GntR family	FIG00621925	ff
fig|6666666.28482.peg.5703	Contig_81	60879	60364	-	FIG00820224: hypothetical protein	FIG00820223	ff
fig|6666666.28482.peg.5704	Contig_81	60913	61356	+	Pyridoxine 5'-phosphate oxidase, Rv1155	FIG01337117	ff
fig|6666666.28482.peg.5705	Contig_81	61399	61575	+	FIG00821392: hypothetical protein	FIG00821390	ff
fig|6666666.28482.peg.5706	Contig_81	62651	61572	-	hypothetical protein		
fig|6666666.28482.peg.5707	Contig_81	63200	62673	-	hypothetical protein		
fig|6666666.28482.peg.5708	Contig_81	63240	63821	+	FIG00822543: hypothetical protein	FIG00822538	ff
fig|6666666.28482.peg.5709	Contig_81	63818	63964	+	FIG00822951: hypothetical protein	FIG00822949	ff
fig|6666666.28482.peg.5710	Contig_81	64360	63965	-	Transcriptional regulator, MarR family	FIG00554610	ff
fig|6666666.28482.peg.5711	Contig_82	99	335	+	PPE family protein	FIG00013075	icw(1);CBSS-164757.7.peg.5020
fig|6666666.28482.peg.5712	Contig_82	346	1248	+	O-Methyltransferase	FIG01333010	ff
fig|6666666.28482.peg.5713	Contig_82	1356	2867	+	PPE family protein	FIG00013075	icw(1);CBSS-164757.7.peg.5020
fig|6666666.28482.peg.5714	Contig_82	3038	4891	+	AAA family ATPase, ESX-1 secretion system component Rv3868	FIG00022220	ff
fig|6666666.28482.peg.5715	Contig_82	4888	6486	+	Type VII secretion protein EccB	FIG01323771	ff

fig|6666666.28482.peg.5716	Contig_82	6483	10463	+	Type VII secretion protein EccCa	FIG01342226	ff
fig|6666666.28482.peg.5717	Contig_82	10466	10774	+	PE family protein	FIG00017916	ff
fig|6666666.28482.peg.5718	Contig_83	466	1806	+	FAD/FMN-containing dehydrogenases	FIG00453123	ff
fig|6666666.28482.peg.5719	Contig_83	1803	3113	+	Cyclopropane-fatty-acyl-phospholipid synthase (EC 2.1.1.79)	FIG00001555	ff
fig|6666666.28482.peg.5720	Contig_83	4995	3199	-	DNA polymerase III subunits gamma and tau (EC 2.7.7.7)	FIG00000414	isu;DNA-replication isu;DNA_processing_cluster
fig|6666666.28482.peg.5721	Contig_83	6415	5123	-	Aspartate transaminase (EC 2.6.1.1)	FIG01340135	ff
fig|6666666.28482.rna.49	Contig_83	6631	6716	+	tRNA-Ser-GGA		isu;tRNAs
fig|6666666.28482.peg.5722	Contig_83	6836	7618	+	PROBABLE CONSERVED TRANSMEMBRANE PROTEIN	FIG00662132	ff

fig|6666666.28482.peg.5723	Contig_83	8360	7620	-	Enoyl-CoA hydratase (EC 4.2.1.17)	FIG00033206	idu(51);n-Phenylalkanoic_acid_degradation idu(51);Polyhydroxybutyrate_metabolism idu(51);Isoleucine_degradation idu(51);Fatty_acid_metabolism_cluster idu(51);Lysine_fermentation idu(51);Butanol_Biosynthesis idu(51);Acetyl-CoA_fermentation_to_Butyrate idu(51);Valine_degradation idu(51);Butyrate_metabolism_cluster
fig|6666666.28482.peg.5724	Contig_83	8731	8357	-	FIG00826013: hypothetical protein	FIG00826011	ff
fig|6666666.28482.peg.5725	Contig_83	8870	9358	+	Lipoprotein LpqH precursor (19 kDa lipoprotein antigen)	FIG01439634	ff
fig|6666666.28482.peg.5726	Contig_83	9454	11190	+	FIG00823916: hypothetical protein	FIG00823913	ff
fig|6666666.28482.peg.5727	Contig_83	11187	12533	+	FIG00821624: hypothetical protein	FIG00821620	ff
fig|6666666.28482.peg.5728	Contig_83	12838	12539	-	FIG00825109: hypothetical protein	FIG00825106	ff
fig|6666666.28482.peg.5729	Contig_83	12937	14262	+	FIG00820860: hypothetical protein	FIG00820859	ff
fig|6666666.28482.peg.5730	Contig_83	14259	15395	+	FIG00822703: hypothetical protein	FIG00822701	ff
fig|6666666.28482.peg.5731	Contig_83	15433	16488	+	FIG00825114: hypothetical protein	FIG00825110	ff
fig|6666666.28482.peg.5732	Contig_83	16656	18803	+	PPE family protein	FIG00013075	idu(57);CBSS-164757.7.peg.5020

fig|6666666.28482.peg.5733	Contig_83	20106	18808	-	Lipase LipD	FIG01326298	ff
fig|6666666.28482.peg.5734	Contig_83	20310	20146	-	hypothetical protein		
fig|6666666.28482.peg.5735	Contig_83	20368	20541	+	hypothetical protein		
fig|6666666.28482.peg.5736	Contig_83	20631	21386	+	serine esterase, cutinase family	FIG01349249	ff
fig|6666666.28482.peg.5737	Contig_83	21391	22386	+	Dihydroflavonol-4-reductase (EC 1.1.1.219)	FIG00042188	idu(2);Biflavanoid_biosynthesis idu(2);Tannin_biosynthesis
fig|6666666.28482.peg.5738	Contig_83	22505	23674	+	2,3-butanediol dehydrogenase, R-alcohol forming, (R)- and (S)-acetoin-specific (EC 1.1.1.4)	FIG00035092	isu;Acetoin,_butanediol_metabolism
fig|6666666.28482.peg.5739	Contig_83	24255	23869	-	FIG00828395: hypothetical protein	FIG00828394	ff
fig|6666666.28482.peg.5740	Contig_83	24972	24268	-	3-oxoacyl-[acyl-carrier protein] reductase (EC 1.1.1.100)	FIG00621114	idu(38);CBSS-246196.1.peg.364 idu(38);CBSS-164757.7.peg.5020 idu(38);Fatty_Acid_Biosynthesis_FASI I
fig|6666666.28482.peg.5741	Contig_83	25143	25697	+	Transcriptional regulator, TetR family	FIG00554417	ff
fig|6666666.28482.peg.5742	Contig_83	26901	25699	-	putative cytochrome P450 hydroxylase	FIG00032437	idu(49);Nitric_oxide_synthase
fig|6666666.28482.peg.5743	Contig_83	27745	26912	-	FIG00820419: hypothetical protein	FIG01323985	ff
fig|6666666.28482.peg.5744	Contig_83	28411	27824	-	Transcriptional regulator, TetR family	FIG00554417	ff
fig|6666666.28482.peg.5745	Contig_83	28518	29060	+	hypothetical protein		
fig|6666666.28482.peg.5746	Contig_83	29148	30014	+	NmrA family protein		
fig|6666666.28482.peg.5747	Contig_83	30044	30808	+	oxidoreductase, short chain dehydrogenase/reductase family		

fig|6666666.28482.peg.5748	Contig_83	30921	32258	+	Diaminopimelate decarboxylase (EC 4.1.1.20)	FIG00138250	idu(2);CBSS-323850.3.peg.3269 idu(2);Lysine_Biosynthesis_DAP_Pathway,_GJO_scratch idu(2);Lysine_Biosynthesis_DAP_Pathway
fig|6666666.28482.peg.5749	Contig_83	32255	33061	+	hypothetical protein		
fig|6666666.28482.peg.5750	Contig_83	34081	33074	-	Methionine ABC transporter ATP-binding protein	FIG00006151	ff
fig|6666666.28482.peg.5751	Contig_84	30	2087	+	ABC transporter, ATP-binding protein	FIG00744535	ff
fig|6666666.28482.peg.5752	Contig_84	2108	4534	+	ABC transporter, ATP-binding protein	FIG00744535	ff
fig|6666666.28482.peg.5753	Contig_84	5085	4531	-	pentapeptide repeat family protein	FIG00630298	ff
fig|6666666.28482.peg.5754	Contig_84	5671	5087	-	Putative ATP/GTP-binding protein	FIG01352018	ff
fig|6666666.28482.peg.5755	Contig_84	6026	5652	-	FIG00995222: hypothetical protein	FIG00821263	ff
fig|6666666.28482.peg.5756	Contig_84	6437	6033	-	FIG01121836: hypothetical protein	FIG01121835	ff
fig|6666666.28482.peg.5757	Contig_84	9220	6434	-	putative sensor-like histidine kinase	FIG01318892	ff
fig|6666666.28482.peg.5758	Contig_84	9475	9939	+	tRNA (cytidine(34)-2'-O)-methyltransferase (EC 2.1.1.207) ## TrmL		isu;RNA_methylation
fig|6666666.28482.peg.5759	Contig_85	378	584	+	hypothetical protein		
fig|6666666.28482.peg.5760	Contig_85	2009	780	-	PE-PGRS family protein		icw(1);PPE_gene_cluster_in_Mycobacteria
fig|6666666.28482.peg.5761	Contig_85	2184	2696	+	PE-PGRS family protein		icw(1);PPE_gene_cluster_in_Mycobacteria

fig|6666666.28482.peg.5762	Contig_85	2822	3385	+	Translation elongation factor P	FIG00000177	isu;Translation_elongation_factor_P_lysylation isu;Translation_elongation_factors_bacterial
fig|6666666.28482.peg.5763	Contig_85	3395	3853	+	Transcription termination protein NusB	FIG00016836	isu;Riboflavin_synthesis_cluster isu;Transcription_factors_bacterial
fig|6666666.28482.peg.5764	Contig_85	3850	4236	+	FIG00822125: hypothetical protein	FIG00822118	ff
fig|6666666.28482.peg.5765	Contig_85	4251	5771	+	Regulator of polyketide synthase expression	FIG00056421	ff
fig|6666666.28482.peg.5766	Contig_85	5901	6503	+	probable conserved transmembrane protein		
fig|6666666.28482.peg.5767	Contig_85	6623	7078	+	transcription elongation factor GreA		
fig|6666666.28482.peg.5768	Contig_85	7251	10058	+	Arginine decarboxylase (EC 4.1.1.19)	FIG00016145	idu(1);Arginine_and_Ornithine_Degradation idu(1);Polyamine_Metabolism
fig|6666666.28482.peg.5769	Contig_85	11138	10344	-	FIG00828282: hypothetical protein	FIG00828281	ff
fig|6666666.28482.peg.5770	Contig_85	11343	12560	+	Integral membrane transport protein	FIG00820204	ff
fig|6666666.28482.peg.5771	Contig_85	12800	13486	+	Putative alkanesulfonate metabolism utilization regulator	FIG00452675	ff
fig|6666666.28482.peg.5772	Contig_85	13483	13710	+	Adenylylsulfate reductase beta-subunit (EC 1.8.99.2)		isu;Inorganic_Sulfur_Assimilation isu;Anaerobic_respiratory_reductases

fig|6666666.28482.peg.5773	Contig_85	13710	15098	+	Organosulfonate ABC transporter substrate-binding protein	FIG01160591	ff
fig|6666666.28482.peg.5774	Contig_85	15095	15958	+	Organosulfonate ABC transporter permease protein	FIG01000979	ff
fig|6666666.28482.peg.5775	Contig_85	15955	16704	+	Organosulfonate ABC transporter ATP-binding protein	FIG00998479	ff
fig|6666666.28482.peg.5776	Contig_85	16704	19385	+	Putative reductase (alkanesulfonate metabolism)	FIG01266661	ff
fig|6666666.28482.peg.5777	Contig_85	21285	20455	-	FIG00829376: hypothetical protein	FIG00829373	ff
fig|6666666.28482.peg.5778	Contig_85	22034	21282	-	hypothetical protein		
fig|6666666.28482.peg.5779	Contig_85	23176	22031	-	[NiFe] hydrogenase metallocenter assembly protein HypD	FIG00001208	icw(3);NiFe_hydrogenase_maturation idu(1);G3E_family_of_P-loop_GTPases_(metallocenter_biosynthesis)
fig|6666666.28482.peg.5780	Contig_85	23463	23191	-	[NiFe] hydrogenase metallocenter assembly protein HypC	FIG00003908	icw(2);NiFe_hydrogenase_maturation icw(4);G3E_family_of_P-loop_GTPases_(metallocenter_biosynthesis)
fig|6666666.28482.peg.5781	Contig_85	24591	23479	-	[NiFe] hydrogenase metallocenter assembly protein HypE	FIG00003378	icw(4);NiFe_hydrogenase_maturation icw(1);G3E_family_of_P-loop_GTPases_(metallocenter_biosynthesis)
fig|6666666.28482.peg.5782	Contig_85	25250	24588	-	Phosphoheptose isomerase 1 (EC 5.3.1.-)	FIG00002799	idu(2);LOS_core_oligosaccharide_biosynthesis idu(2);Capsular_heptose_biosynthesis

fig|6666666.28482.peg.5783	Contig_85	25961	25257	-	possible sedoheptulose 7-phosphate isomerase		
fig|6666666.28482.peg.5784	Contig_85	26221	25958	-	[NiFe] hydrogenase metallocenter assembly protein HypC	FIG00003908	icw(2);NiFe_hydrogenase_maturation icw(4);G3E_family_of_P-loop_GTPases_(metallocenter_biosynthesis)
fig|6666666.28482.peg.5785	Contig_85	28605	26212	-	[NiFe] hydrogenase metallocenter assembly protein HypF	FIG00001209	idu(1);NiFe_hydrogenase_maturation icw(2);G3E_family_of_P-loop_GTPases_(metallocenter_biosynthesis)
fig|6666666.28482.peg.5786	Contig_85	29783	28590	-	Transcriptional regulator	FIG01312420	ff
fig|6666666.28482.peg.5787	Contig_85	30694	29780	-	Nitrogen-fixing NifU, C-terminal:Rieske [2Fe-2S] region	FIG00674168	ff
fig|6666666.28482.peg.5788	Contig_85	31947	30694	-	FOG: TPR repeat	FIG00555909	ff
fig|6666666.28482.peg.5789	Contig_85	32212	31940	-	hypothetical protein		
fig|6666666.28482.peg.5790	Contig_85	33066	32212	-	Hydrogenase maturation protease (EC 3.4.24.-)	FIG00621884	idu(1);Coenzyme_F420_hydrogenase idu(1);Hydrogen-sensing_regulatory_system
fig|6666666.28482.peg.5791	Contig_85	34670	33063	-	Uptake hydrogenase large subunit (EC 1.12.99.6)	FIG00138205	idu(1);Hydrogenases
fig|6666666.28482.peg.5792	Contig_85	35690	34728	-	Uptake hydrogenase small subunit precursor (EC 1.12.99.6)	FIG00133578	icw(1);Hydrogenases
fig|6666666.28482.peg.5793	Contig_85	36350	35748	-	hypothetical protein		
fig|6666666.28482.peg.5794	Contig_85	36464	37612	+	benABC operon transcriptional activator BenR	FIG00007036	isu;Benzoate_degradation

fig|6666666.28482.peg.5795	Contig_85	37658	37996	+	[NiFe] hydrogenase nickel incorporation protein HypA		idu(1);NiFe_hydrogenase_maturation icw(1);G3E_family_of_P-loop_GTPases_(metallocenter_biosynthesis)
fig|6666666.28482.peg.5796	Contig_85	38000	38818	+	[NiFe] hydrogenase nickel incorporation-associated protein HypB	FIG00001210	icw(1);NiFe_hydrogenase_maturation idu(1);G3E_family_of_P-loop_GTPases_(metallocenter_biosynthesis)
fig|6666666.28482.peg.5797	Contig_85	38861	39934	+	Fructose-1,6-bisphosphatase, type I (EC 3.1.3.11)	FIG00000732	isu;Putative_sugar_ABC_transporter_(ytf_cluster)
fig|6666666.28482.peg.5798	Contig_85	39940	40839	+	Phosphoribulokinase (EC 2.7.1.19)	FIG00070494	ff
fig|6666666.28482.peg.5799	Contig_85	40873	41928	+	HoxN/HupN/NixA family nickel/cobalt transporter	FIG00016929	idu(1);Transport_of_Nickel_and_Cobalt
fig|6666666.28482.peg.5800	Contig_85	42536	41940	-	hypothetical protein		
fig|6666666.28482.peg.5801	Contig_85	42958	42536	-	Ribulose bisphosphate carboxylase small chain (EC 4.1.1.39)	FIG00071133	isu;CO2_uptake,_carboxysome isu;Photorespiration_(oxidative_C2_cycle)
fig|6666666.28482.peg.5802	Contig_85	44407	42977	-	Ribulose bisphosphate carboxylase large chain (EC 4.1.1.39)	FIG00133533	icw(1);CO2_uptake,_carboxysome icw(1);Photorespiration_(oxidative_C2_cycle)
fig|6666666.28482.peg.5803	Contig_85	44579	45577	+	Fructose-bisphosphate aldolase class I (EC 4.1.2.13)	FIG01262685	idu(1);Unknown_carbohydrate_utilization_(_cluster_Yeg_)
fig|6666666.28482.peg.5804	Contig_85	46054	45605	-	probable transcriptional regulator, TetR family		
fig|6666666.28482.peg.5805	Contig_85	46454	46317	-	hypothetical protein		

fig|6666666.28482.peg.5806	Contig_86	521	21	-	FIG00824419: hypothetical protein	FIG00824418	ff
fig|6666666.28482.peg.5807	Contig_86	1319	636	-	Transcriptional regulator, TetR family	FIG00554417	ff
fig|6666666.28482.peg.5808	Contig_86	1431	1877	+	FIG00820982: hypothetical protein	FIG00820978	ff
fig|6666666.28482.peg.5809	Contig_86	2556	1879	-	FIG00820470: hypothetical protein	FIG00820469	ff
fig|6666666.28482.peg.5810	Contig_86	3587	2655	-	Membrane-bound C-5 sterol desaturase Erg3	FIG01324519	ff
fig|6666666.28482.peg.5811	Contig_86	3651	4850	+	NADH dehydrogenase (EC 1.6.99.3)	FIG00008708	idu(6);Respiratory_dehydrogenases_1 icw(2);PPE_gene_cluster_in_Mycobacteria idu(6);Riboflavin_synthesis_cluster
fig|6666666.28482.peg.5812	Contig_86	5573	4866	-	Mg(2+) transport ATPase protein C	FIG00436510	isu;PPE_gene_cluster_in_Mycobacteria isu;Magnesium_transport
fig|6666666.28482.peg.5813	Contig_86	6024	5698	-	FIG00819992: hypothetical protein	FIG00819991	icw(1);PPE_gene_cluster_in_Mycobacteria
fig|6666666.28482.peg.5814	Contig_86	7467	6052	-	PPE family protein	FIG00013075	icw(2);CBSS-164757.7.peg.5020
fig|6666666.28482.peg.5815	Contig_86	8816	7503	-	PPE family protein	FIG00013075	icw(3);CBSS-164757.7.peg.5020
fig|6666666.28482.peg.5816	Contig_86	10968	9775	-	PPE family protein	FIG00013075	icw(3);CBSS-164757.7.peg.5020
fig|6666666.28482.peg.5817	Contig_86	10987	11316	+	hypothetical protein		
fig|6666666.28482.peg.5818	Contig_86	12891	11614	-	PPE family protein	FIG00013075	icw(2);CBSS-164757.7.peg.5020
fig|6666666.28482.peg.5819	Contig_86	15287	13500	-	Type VII secretion AAA-ATPase EccA	FIG01399524	isu;PPE_gene_cluster_in_Mycobacteria
fig|6666666.28482.peg.5820	Contig_86	16531	15326	-	Type VII secretion protein EccE	FIG00822757	icw(2);PPE_gene_cluster_in_Mycobacteria
fig|6666666.28482.peg.5821	Contig_86	18395	16551	-	Type VII secretion-associated serine protease mycosin	FIG01428662	icw(1);PPE_gene_cluster_in_Mycobacteria

fig|6666666.28482.peg.5822	Contig_86	19809	18373	-	Type VII secretion integral membrane protein EccD	FIG01352284	icw(3);PPE_gene_cluster_in_Mycobacteria
fig|6666666.28482.peg.5823	Contig_86	21062	20160	-	FIG00820957: hypothetical protein	FIG00820956	ff
fig|6666666.28482.peg.5824	Contig_86	21296	21153	-	ESAT-6-like protein EsxL	FIG00820465	ff
fig|6666666.28482.peg.5825	Contig_87	160	315	+	hypothetical protein	FIG00638284	ff
fig|6666666.28482.peg.5826	Contig_87	403	1077	+	Superoxide dismutase [Mn/Fe] (EC 1.15.1.1)	FIG00008667	isu;Oxidative_stress isu;Mycobacterium_virulence_operon_involved_in_an_unknown_function_with_Superoxide_dismutase
fig|6666666.28482.peg.5827	Contig_87	1342	1806	+	FIG017534: hypothetical protein	FIG00821692	icw(2);Mycobacterium_virulence_operon_involved_in_an_unknown_function_with_Superoxide_dismutase
fig|6666666.28482.peg.5828	Contig_87	2040	2735	+	FIG037441: Conserved transmembrane protein	FIG01316480	icw(1);Mycobacterium_virulence_operon_involved_in_an_unknown_function_with_Superoxide_dismutase
fig|6666666.28482.peg.5829	Contig_87	3106	3588	+	ESX-1 secreted protein regulator EspR	FIG00821305	ff
fig|6666666.28482.peg.5830	Contig_87	3730	4386	+	FIG022958: hypothetical protein	FIG00995251	ff
fig|6666666.28482.peg.5831	Contig_87	4383	5015	+	FIG00820208: hypothetical protein	FIG00820207	ff
fig|6666666.28482.peg.5832	Contig_87	5012	5524	+	Copper resistance protein CopC precursor	FIG01498667	ff
fig|6666666.28482.peg.5833	Contig_87	5627	6049	+	hypothetical protein		
fig|6666666.28482.peg.5834	Contig_87	6051	6536	+	Ribonuclease E inhibitor RraA	FIG00000884	isu;RNA_processing_and_degradation,_bacterial

fig|6666666.28482.peg.5835	Contig_87	8002	6533	-	monooxygenase, flavin-binding family	FIG01386881	ff
fig|6666666.28482.peg.5836	Contig_87	8070	8720	+	Transcriptional repressor EthR, TetR family	FIG01323805	ff
fig|6666666.28482.peg.5837	Contig_88	1120	215	-	PPE family protein	FIG00013075	idu(57);CBSS-164757.7.peg.5020
fig|6666666.28482.peg.5838	Contig_89	3048	2389	-	Phosphate transport system regulatory protein PhoU	FIG00104848	idu(3);High_affinity_phosphate_transporter_and_control_of_PHO_regulon idu(3);Phosphate_metabolism
fig|6666666.28482.peg.5839	Contig_89	3219	3818	+	FIG00832151: hypothetical protein	FIG01483472	ff
fig|6666666.28482.peg.5840	Contig_89	4210	3815	-	Inosine-5'-monophosphate dehydrogenase (EC 1.1.1.205)	FIG00020613	idu(4);Purine_conversions idu(4);Purine_salvage_cluster
fig|6666666.28482.peg.5841	Contig_89	4367	6640	+	FUPA25 P-type ATPase	FIG01426022	ff
fig|6666666.28482.peg.5842	Contig_89	7765	6629	-	PPE family protein	FIG00013075	icw(1);CBSS-164757.7.peg.5020
fig|6666666.28482.peg.5843	Contig_89	8900	7767	-	PPE family protein	FIG00013075	icw(1);CBSS-164757.7.peg.5020
fig|6666666.28482.peg.5844	Contig_89	9887	9084	-	hypothetical protein	FIG00638284	ff
fig|6666666.28482.peg.5845	Contig_89	10589	10029	-	hypothetical protein		
fig|6666666.28482.peg.5846	Contig_89	10964	10800	-	13E12 repeat family protein		
fig|6666666.28482.peg.5847	Contig_9	488	315	-	hypothetical protein		
fig|6666666.28482.peg.5848	Contig_9	1436	522	-	Transcriptional regulator, AraC family		
fig|6666666.28482.peg.5849	Contig_90	2251	839	-	Mycofactocin system glycosyltransferase	FIG00600157	isu;Mycofactocin_system_cluster
fig|6666666.28482.peg.5850	Contig_90	2928	2248	-	Cyclic amid hydrolase in mycofactocin cluster		icw(6);Mycofactocin_system_cluster

fig|6666666.28482.peg.5851	Contig_90	4378	3197	-	Mycofactocin system heme/flavin dehydrogenase	FIG01307171	icw(4);Mycofactocin_system_cluster
fig|6666666.28482.peg.5852	Contig_90	5547	4381	-	Mycofactocin radical SAM maturase	FIG00660086	icw(1);Mycofactocin_system_cluster
fig|6666666.28482.peg.5853	Contig_90	5816	5544	-	Mycofactocin system small protein	FIG00661223	icw(5);Mycofactocin_system_cluster
fig|6666666.28482.peg.5854	Contig_90	5941	5822	-	Mycofactocin precursor	FIG00820162	icw(2);Mycofactocin_system_cluster
fig|6666666.28482.peg.5855	Contig_90	6002	6595	+	Mycofactocin system transcriptional regulator	FIG01311936	icw(3);Mycofactocin_system_cluster
fig|6666666.28482.peg.5856	Contig_90	6592	7656	+	FIG00821437: hypothetical protein	FIG00821436	ff
fig|6666666.28482.peg.5857	Contig_90	7709	8230	+	RNA polymerase, sigma-24 subunit, ECF subfamily		
fig|6666666.28482.peg.5858	Contig_90	8227	9462	+	Glyoxalase family protein	FIG00007915	ff
fig|6666666.28482.peg.5859	Contig_90	10400	9444	-	D-3-phosphoglycerate dehydrogenase (EC 1.1.1.95)	FIG00031250	idu(4);Serine_Biosynthesis idu(4);Pyridoxin_(Vitamin_B6)_Biosynthesis
fig|6666666.28482.peg.5860	Contig_90	11611	10439	-	Ferredoxin reductase	FIG00011423	idu(1);Anaerobic_respiratory_reductases
fig|6666666.28482.peg.5861	Contig_90	12383	11637	-	3-oxoacyl-[acyl-carrier protein] reductase (EC 1.1.1.100)	FIG00621114	idu(38);CBSS-246196.1.peg.364 idu(38);CBSS-164757.7.peg.5020 idu(38);Fatty_Acid_Biosynthesis_FASII
fig|6666666.28482.peg.5862	Contig_90	13344	12514	-	FIG025093: Probable membrane protein	FIG01306444	icw(1);Mycobacterium_virulence_operon_involved_in_protein_synthesis_(SSU_ribosomal_proteins)
fig|6666666.28482.peg.5863	Contig_90	14230	13430	-	hypothetical protein	FIG00638284	ff

fig|6666666.28482.peg.5864	Contig_90	15908	14364	-	PE-PGRS family protein		idu(16);PPE_gene_cluster_in_Mycobacteria
fig|6666666.28482.peg.5865	Contig_90	17254	16064	-	Translation elongation factor Tu	FIG00000039	icw(1);Translation_elongation_factors_bacterial icw(2);Mycobacterium_virulence_operon_involved_in_protein_synthesis_(SSU_ribosomal_proteins)
fig|6666666.28482.peg.5866	Contig_90	19515	17410	-	Translation elongation factor G	FIG00063189	isu;Tetracycline_resistance,_ribosome_protection_type,_too isu;Translation_elongation_factor_G_family isu;Translation_elongation_factors_bacterial isu;Tetracycline_resistance,_ribosome_protection_type icw(1);Mycobacterium_virulence_operon_involved_in_protein_synthesis_(SSU_ribosomal_proteins)
fig|6666666.28482.peg.5867	Contig_90	20056	19586	-	SSU ribosomal protein S7p (S5e)	FIG00000209	isu;Mycobacterium_virulence_operon_involved_in_protein_synthesis_(SSU_ribosomal_proteins)
fig|6666666.28482.peg.5868	Contig_90	20430	20056	-	SSU ribosomal protein S12p (S23e)	FIG00000203	isu;Ribosomal_protein_S12p_Asp_methylthiotransferase icw(3);Mycobacterium_virulence_operon_involved_in_protein_synthesis_(SSU_ribosomal_proteins)
fig|6666666.28482.peg.5869	Contig_91	297	533	+	hypothetical protein		
fig|6666666.28482.peg.5870	Contig_91	1363	530	-	Hydride transferase 1 (Fragment)	FIG01353725	ff
fig|6666666.28482.peg.5871	Contig_91	1575	1459	-	hypothetical protein		

fig|6666666.28482.peg.5872	Contig_91	1596	2501	+	antigen 34 kDa	FIG00820205	ff
fig|6666666.28482.peg.5873	Contig_91	2610	3878	+	FIG021574: Possible membrane protein related to de Novo purine biosynthesis	FIG00021574	icw(2);A_hypothetical_coupled_to_de_Novo_Purine_Biosynthesis
fig|6666666.28482.peg.5874	Contig_91	4025	4654	+	Phosphoribosylglycinamide formyltransferase (EC 2.1.2.2)	FIG00000253	icw(1);De_Novo_Purine_Biosynthesis isu;A_hypothetical_coupled_to_de_Novo_Purine_Biosynthesis
fig|6666666.28482.peg.5875	Contig_91	4651	6219	+	IMP cyclohydrolase (EC 3.5.4.10) / Phosphoribosylaminoimidazolecarboxamide formyltransferase (EC 2.1.2.3)	FIG00000275	isu;De_Novo_Purine_Biosynthesis icw(1);De_Novo_Purine_Biosynthesis icw(1);A_hypothetical_coupled_to_de_Novo_Purine_Biosynthesis icw(1);A_hypothetical_coupled_to_de_Novo_Purine_Biosynthesis
fig|6666666.28482.peg.5876	Contig_91	6262	7647	+	Magnesium chelatase, subunit ChlI (EC 6.6.1.1)	FIG00019368	icw(1);CBSS-336982.3.peg.1011
fig|6666666.28482.peg.5877	Contig_91	7640	9640	+	FIG019045: long form Mg-chelase associated protein with vWA domain	FIG00019045	isu;CBSS-336982.3.peg.1011
fig|6666666.28482.peg.5878	Contig_91	9713	10561	+	morphine 6-dehydrogenase(EC:1.1.1.218,EC:1.1.1.274)		
fig|6666666.28482.peg.5879	Contig_91	11237	10593	-	FIG00821093: hypothetical protein	FIG00821091	ff

fig|6666666.28482.peg.5880	Contig_91	12087	11317	-	Methylglutaconyl-CoA hydratase (EC 4.2.1.18)	FIG00138403	isu;HMG_CoA_Synthesis isu;Benzoate_transport_and_degradation_cluster isu;Leucine_Degradation_and_HMG-CoA_Metabolism
fig|6666666.28482.peg.5881	Contig_91	13253	12087	-	Isovaleryl-CoA dehydrogenase (EC 1.3.99.10)	FIG00138712	icw(3);HMG_CoA_Synthesis icw(4);Leucine_Degradation_and_HMG-CoA_Metabolism
fig|6666666.28482.peg.5882	Contig_91	15247	13250	-	Methylcrotonyl-CoA carboxylase biotin-containing subunit (EC 6.4.1.4)	FIG00183535	icw(1);Serine-glyoxylate_cycle icw(4);HMG_CoA_Synthesis icw(2);Leucine_Degradation_and_HMG-CoA_Metabolism
fig|6666666.28482.peg.5883	Contig_91	16848	15253	-	Methylcrotonyl-CoA carboxylase carboxyl transferase subunit (EC 6.4.1.4)	FIG00138249	idu(2);Serine-glyoxylate_cycle icw(1);HMG_CoA_Synthesis icw(1);Leucine_Degradation_and_HMG-CoA_Metabolism
fig|6666666.28482.peg.5884	Contig_91	18070	16922	-	Isovaleryl-CoA dehydrogenase (EC 1.3.99.10)	FIG00138712	icw(2);HMG_CoA_Synthesis icw(3);Leucine_Degradation_and_HMG-CoA_Metabolism
fig|6666666.28482.peg.5885	Contig_91	19770	18067	-	expressed protein	FIG00839682	ff
fig|6666666.28482.peg.5886	Contig_91	19999	20172	+	LSU ribosomal protein L32p	FIG00006632	isu;Ribosome_LSU_bacterial
fig|6666666.28482.peg.5887	Contig_91	20230	20922	+	Two-component response regulator	FIG01956054	ff
fig|6666666.28482.peg.5888	Contig_91	20939	22513	+	Osmosensitive K+ channel histidine kinase KdpD (EC 2.7.3.-)	FIG00000846	idu(1);Potassium_homeostasis
fig|6666666.28482.peg.5889	Contig_91	22672	24111	+	Heat shock protein HtrA	FIG00820933	ff
fig|6666666.28482.peg.5890	Contig_91	24195	24671	+	Molybdenum cofactor biosynthesis protein MoaB	FIG00032229	icw(1);Molybdenum_cofactor_biosynthesis

fig|6666666.28482.peg.5891	Contig_91	25146	24679	-	Large-conductance mechanosensitive channel	FIG00000518	icw(1);Potassium_homeostasis
fig|6666666.28482.peg.5892	Contig_91	25865	25212	-	Heat shock protein 22.5 (Hsp22.5)	FIG00821617	ff
fig|6666666.28482.peg.5893	Contig_91	26272	25946	-	Type I antifreeze protein	FIG00661702	ff
fig|6666666.28482.peg.5894	Contig_91	26940	26347	-	5-formyltetrahydrofolate cyclo-ligase (EC 6.3.3.2)	FIG00000252	isu;One-carbon_metabolism_by_tetrahydropterines isu;Serine-glyoxylate_cycle isu;Folate_Biosynthesis
fig|6666666.28482.peg.5895	Contig_91	27105	27929	+	UTP--glucose-1-phosphate uridylyltransferase (EC 2.7.7.9)	FIG00000108	isu;Ribosome_post-transcriptional_modification_and_chromosomal_segregation_cluster
fig|6666666.28482.peg.5896	Contig_91	27978	29258	+	Molybdopterin biosynthesis protein MoeA	FIG00018218	idu(1);Molybdenum_cofactor_biosynthesis
fig|6666666.28482.peg.5897	Contig_91	29274	29933	+	Ribosomal-protein-S5p-alanine acetyltransferase	FIG00000756	isu;Ribosomal_protein_S5p_acylation
fig|6666666.28482.peg.5898	Contig_91	30077	31090	+	PROBABLE CONSERVED TRANSMEMBRANE PROTEIN	FIG00662132	ff
fig|6666666.28482.rna.50	Contig_91	31141	31213	+	tRNA-Ala-CGC		isu;tRNAs
fig|6666666.28482.peg.5899	Contig_91	31301	31519	+	hypothetical protein		
fig|6666666.28482.peg.5900	Contig_91	31702	33630	+	FIG033455: hypothetical protein	FIG00822234	ff
fig|6666666.28482.peg.5901	Contig_91	33710	34186	+	Lipoprotein LppJ	FIG01346333	ff
fig|6666666.28482.peg.5902	Contig_91	34443	34210	-	hypothetical protein		

fig|6666666.28482.peg.5903	Contig_91	34586	35707	+	FIG00829482: hypothetical protein	FIG00829481	ff
fig|6666666.28482.peg.5904	Contig_91	35981	36703	+	Glycosyl transferase, family 2	FIG00454917	ff
fig|6666666.28482.peg.5905	Contig_91	36700	37839	+	hypothetical protein		
fig|6666666.28482.peg.5906	Contig_91	38088	38924	+	esterase	FIG01380254	ff
fig|6666666.28482.peg.5907	Contig_91	39631	38921	-	Transcriptional regulator, TetR family	FIG00554417	ff
fig|6666666.28482.peg.5908	Contig_91	40664	40023	-	Transcriptional regulator, TetR family	FIG00554417	ff
fig|6666666.28482.peg.5909	Contig_91	41203	41565	+	putative PAS/PAC sensor protein		
fig|6666666.28482.peg.5910	Contig_91	41933	42232	+	PE family protein		
fig|6666666.28482.peg.5911	Contig_91	42252	43667	+	PPE family protein	FIG00013075	idu(57);CBSS-164757.7.peg.5020
fig|6666666.28482.peg.5912	Contig_91	43763	44059	+	FIG00824639: hypothetical protein	FIG00823510	ff
fig|6666666.28482.peg.5913	Contig_92	1489	566	-	RNA polymerase sigma-70 factor	FIG01303902	idu(4);CBSS-313593.3.peg.2729 idu(4);Transcription_initiation,_bacterial_sigma_factors
fig|6666666.28482.peg.5914	Contig_92	1516	2859	+	Omega-amino acid--pyruvate aminotransferase (EC 2.6.1.18)	FIG00001350	isu;Pyruvate_Alanine_Serine_Interconversions
fig|6666666.28482.peg.5915	Contig_92	3214	2834	-	FIG00821204: hypothetical protein	FIG00821203	ff
fig|6666666.28482.peg.5916	Contig_92	3310	4494	+	D-alanyl-D-alanine carboxypeptidase (EC 3.4.16.4)	FIG00561498	idu(3);Murein_Hydrolases idu(3);CBSS-84588.1.peg.1247 idu(3);Metallocarboxypeptidases_(EC_3.4.17.-) idu(3);Peptidoglycan_Biosynthesis

fig|6666666.28482.peg.5917	Contig_92	4491	5834	+	Sugar-transport integral membrane proteinSugI	FIG01373312	ff
fig|6666666.28482.peg.5918	Contig_92	5825	6967	+	N-acetylglucosamine-6-phosphate deacetylase (EC 3.5.1.25)	FIG00076542	isu;Chitin_and_N-acetylglucosamine_utilization isu;Sialic_Acid_Metabolism
fig|6666666.28482.peg.5919	Contig_92	8448	6964	-	Cyclohexanone monooxygenase (EC 1.14.13.22)	FIG00975774	ff
fig|6666666.28482.peg.5920	Contig_92	9565	8525	-	putative membrane protein	FIG00672847	ff
fig|6666666.28482.peg.5921	Contig_92	10593	9574	-	Tryptophanyl-tRNA synthetase (EC 6.1.1.2)	FIG00000055	isu;tRNA_aminoacylation,_Trp
fig|6666666.28482.peg.5922	Contig_92	10680	11522	+	Alpha/beta hydrolase family	FIG01383046	ff
fig|6666666.28482.peg.5923	Contig_92	12740	11511	-	Isocitrate dehydrogenase [NADP] (EC 1.1.1.42)	FIG00000600	icw(1);TCA_Cycle
fig|6666666.28482.peg.5924	Contig_92	15015	12775	-	Isocitrate dehydrogenase [NADP] (EC 1.1.1.42); Monomeric isocitrate dehydrogenase [NADP] (EC 1.1.1.42)	FIG00001163	icw(1);TCA_Cycle
fig|6666666.28482.peg.5925	Contig_92	15402	15070	-	hypothetical protein		
fig|6666666.28482.peg.5926	Contig_92	15451	16815	+	O-acetylhomoserine sulfhydrylase (EC 2.5.1.49) / O-succinylhomoserine sulfhydrylase (EC 2.5.1.48)	FIG00000417	ff

fig|6666666.28482.peg.5927	Contig_92	16827	17954	+	Homoserine O-acetyltransferase (EC 2.3.1.31)	FIG00000716	ff
fig|6666666.28482.peg.5928	Contig_92	17951	18685	+	Methyltransferase (EC 2.1.1.-)	FIG00515945	ff
fig|6666666.28482.peg.5929	Contig_92	19949	18696	-	PPE family protein	FIG00013075	idu(57);CBSS-164757.7.peg.5020
fig|6666666.28482.peg.5930	Contig_93	8	208	+	Catalyzes the cleavage of p-aminobenzoyl-glutamate to p-aminobenzoate and glutamate, subunit A	FIG00001384	ff
fig|6666666.28482.peg.5931	Contig_93	205	1371	+	Possible N-acyl-L-amino acid amidohydrolase amiA1 (EC 3.5.1.-)	FIG00692008	ff
fig|6666666.28482.peg.5932	Contig_93	1427	2308	+	FIG00823482: hypothetical protein	FIG00823480	ff
fig|6666666.28482.peg.5933	Contig_93	2868	2386	-	FIG01122412: hypothetical protein	FIG01122410	ff
fig|6666666.28482.peg.5934	Contig_93	2953	4368	+	Dihydrolipoamide dehydrogenase (EC 1.8.1.4)	FIG00136193	idu(1);TCA_Cycle idu(1);Photorespiration_(oxidative_C2_cycle) idu(1);Leucine_Degradation_and_HMG-CoA_Metabolism idu(1);Glycine_cleavage_system
fig|6666666.28482.peg.5935	Contig_93	4425	6149	+	Glycerol-3-phosphate dehydrogenase (EC 1.1.5.3)	FIG00229386	idu(1);Respiratory_dehydrogenases_1 idu(1);Glycerol_and_Glycerol-3-phosphate_Uptake_and_Utilization idu(1);Glycerolipid_and_Glycerophospholipid_Metabolism_in_Bacteria

fig|6666666.28482.peg.5936	Contig_93	6189	7007	+	Ribosomal large subunit pseudouridine synthase A (EC 4.2.1.70)	FIG00001028	isu;RNA_pseudouridine_syntheses
fig|6666666.28482.peg.5937	Contig_93	7048	7386	+	hypothetical protein		
fig|6666666.28482.peg.5938	Contig_93	7469	7852	+	13E12 repeat family protein	FIG00831556	ff
fig|6666666.28482.peg.5939	Contig_94	539	874	+	FIG000557: hypothetical protein co-occurring with RecR	FIG00000557	isu;DNA_processing_cluster
fig|6666666.28482.peg.5940	Contig_94	888	1499	+	Recombination protein RecR	FIG00000190	isu;DNA-replication icw(1);DNA_processing_cluster
fig|6666666.28482.peg.5941	Contig_94	2252	1599	-	conserved hypothetical secreted protein		
fig|6666666.28482.peg.5942	Contig_94	3036	2344	-	Putative amidotransferase similar to cobyric acid synthase	FIG00628704	ff
fig|6666666.28482.peg.5943	Contig_94	4273	3044	-	proposed amino acid ligase found clustered with an amidotransferase	FIG00629105	ff
fig|6666666.28482.peg.5944	Contig_94	4405	5397	+	DNA polymerase III epsilon subunit DnaQ (EC 2.7.7.7)	FIG00544340	ff
fig|6666666.28482.peg.5945	Contig_94	5528	5893	+	FIG00824314: hypothetical protein	FIG00824313	ff
fig|6666666.28482.peg.5946	Contig_94	5893	6459	+	Transcriptional regulator, TetR family	FIG00554417	ff

fig|6666666.28482.peg.5947	Contig_94	8339	6528	-	2-isopropylmalate synthase (EC 2.3.3.13)	FIG00000157	isu;Branched-Chain_Amino_Acid_Biosynthesis isu;Leucine_Biosynthesis
fig|6666666.28482.peg.5948	Contig_94	9755	8520	-	possible secreted protein		
fig|6666666.28482.peg.5949	Contig_94	9940	10782	+	Cysteine protease		
fig|6666666.28482.peg.5950	Contig_94	10941	12206	+	Aspartokinase (EC 2.7.2.4)	FIG00000132	icw(1);Lysine_Biosynthesis_DAP_Pathway,_GJO_scratch isu;CBSS-216591.1.peg.168 icw(1);Threonine_and_Homoserine_Biosynthesis icw(1);Lysine_Biosynthesis_DAP_Pathway
fig|6666666.28482.peg.5951	Contig_94	12224	13255	+	Aspartate-semialdehyde dehydrogenase (EC 1.2.1.11)	FIG00026137	isu;Lysine_Biosynthesis_DAP_Pathway,_GJO_scratch isu;Threonine_and_Homoserine_Biosynthesis isu;Lysine_Biosynthesis_DAP_Pathway
fig|6666666.28482.peg.5952	Contig_94	13395	14390	+	FIG00821722: hypothetical protein	FIG00821721	ff
fig|6666666.28482.peg.5953	Contig_95	56	1810	+	hypothetical protein	FIG00638284	ff
fig|6666666.28482.peg.5954	Contig_95	1965	2522	+	FIG00820556: hypothetical protein	FIG00820555	ff
fig|6666666.28482.peg.5955	Contig_95	2606	3034	+	FIG00820898: hypothetical protein	FIG00820897	ff
fig|6666666.28482.peg.5956	Contig_95	3288	3043	-	hypothetical protein		
fig|6666666.28482.peg.5957	Contig_95	3864	3394	-	FIG00824719: hypothetical protein	FIG00824717	ff
fig|6666666.28482.peg.5958	Contig_95	4760	3861	-	FIG00826304: hypothetical protein	FIG00826302	ff

fig|6666666.28482.peg.5959	Contig_95	6954	4780	-	4-alpha-glucanotransferase (amylomaltase) (EC 2.4.1.25)	FIG00028461	isu;Glycogen_metabolism isu;Maltose_and_Maltodextrin_Utilization
fig|6666666.28482.peg.5960	Contig_95	7195	7040	-	hypothetical protein		
fig|6666666.28482.peg.5961	Contig_95	7184	7297	+	hypothetical protein		
fig|6666666.28482.peg.5962	Contig_95	7319	8839	+	Possible membrane protein	FIG01121868	ff
fig|6666666.28482.peg.5963	Contig_95	8836	13005	+	Type VII secretion protein EccCa, FtsK/SpoIIIE family	FIG01319961	ff
fig|6666666.28482.peg.5964	Contig_95	14224	13049	-	Cytochrome P450 143	FIG01309785	ff
fig|6666666.28482.peg.5965	Contig_95	14520	14660	+	Putative ferredoxin	FIG00820674	ff
fig|6666666.28482.peg.5966	Contig_95	15084	16355	+	PPE family protein	FIG00013075	idu(57);CBSS-164757.7.peg.5020
fig|6666666.28482.peg.5967	Contig_95	16572	16871	+	PE family protein	FIG01313150	ff
fig|6666666.28482.peg.5968	Contig_96	556	35	-	Cytoplasmic membrane protein FsxA	FIG00544483	isu;Lipid-linked_oligosaccharide_synthesis_related_cluster
fig|6666666.28482.peg.5969	Contig_96	630	1328	+	Dienelactone hydrolase family protein	FIG01379580	ff
fig|6666666.28482.peg.5970	Contig_96	2194	1340	-	ThiJ/PfpI family protein	FIG00015923	idu(8);CBSS-176280.1.peg.1561
fig|6666666.28482.peg.5971	Contig_96	2595	2200	-	FIG00995176: hypothetical protein	FIG00820000	ff
fig|6666666.28482.peg.5972	Contig_96	6279	2704	-	CobN component of cobalt chelatase involved in B12 biosynthesis	FIG00001395	isu;Coenzyme_B12_biosynthesis
fig|6666666.28482.peg.5973	Contig_96	6952	6272	-	FIG00826239: hypothetical protein	FIG00826238	ff
fig|6666666.28482.peg.5974	Contig_96	7998	7393	-	hypothetical protein	FIG00638284	ff
fig|6666666.28482.peg.5975	Contig_96	8775	8149	-	hypothetical protein		

fig|6666666.28482.peg.5976	Contig_96	8917	8756	-	hypothetical protein		
fig|6666666.28482.peg.5977	Contig_96	10202	9279	-	Alpha/beta hydrolase fold-3		
fig|6666666.28482.peg.5978	Contig_96	10428	10255	-	hypothetical protein		
fig|6666666.28482.peg.5979	Contig_96	10483	11298	+	hypothetical protein	FIG00638284	ff
fig|6666666.28482.peg.5980	Contig_96	12516	11620	-	LysR-family protein trancsriptional regulator		
fig|6666666.28482.peg.5981	Contig_96	12647	13303	+	short-chain dehydrogenase/reductase SDR		
fig|6666666.28482.peg.5982	Contig_96	13701	15257	+	Choline dehydrogenase (EC 1.1.99.1)	FIG00057416	idu(4);Choline_uptake_and_conversion_to_betaine_clusters idu(4);Choline_and_Betaine_Uptake_and_Betaine_Biosynthesis
fig|6666666.28482.peg.5983	Contig_96	15790	15521	-	hypothetical protein		
fig|6666666.28482.peg.5984	Contig_96	16094	16315	+	hypothetical protein		
fig|6666666.28482.peg.5985	Contig_96	16916	17155	+	alpha/beta hydrolase fold		
fig|6666666.28482.peg.5986	Contig_96	17195	18778	+	Long-chain-fatty-acid--CoA ligase (EC 6.2.1.3)	FIG00018699	idu(72);n-Phenylalkanoic_acid_degradation idu(72);Fatty_acid_metabolism_cluster idu(72);Biotin_synthesis_cluster idu(72);Biotin_biosynthesis
fig|6666666.28482.peg.5987	Contig_96	19515	19856	+	Adenylate cyclase (EC 4.6.1.1)	FIG00001294	idu(25);cAMP_signaling_in_bacteria
fig|6666666.28482.peg.5988	Contig_96	19946	20188	+	putative adenylate/guanylate cyclase		

fig|6666666.28482.peg.5989	Contig_96	20383	21492	+	Cobalamin biosynthesis protein CobG	FIG00002104	icw(2);Coenzyme_B12_biosynthesis
fig|6666666.28482.peg.5990	Contig_96	21497	22123	+	Cobalt-precorrin-8x methylmutase (EC 5.4.1.2)	FIG00051882	isu;Cobalamin_synthesis isu;Coenzyme_B12_biosynthesis
fig|6666666.28482.peg.5991	Contig_96	22120	23598	+	Cobalt-precorrin-2 C20-methyltransferase (EC 2.1.1.130) / Cobalt-precorrin-3b C17-methyltransferase	FIG00021888	icw(1);Cobalamin_synthesis icw(1);Cobalamin_synthesis icw(1);Coenzyme_B12_biosynthesis icw(1);Coenzyme_B12_biosynthesis
fig|6666666.28482.peg.5992	Contig_96	24968	23751	-	3-demethylubiquinone-9 3-methyltransferase (EC 2.1.1.64)	FIG00030029	ff
fig|6666666.28482.peg.5993	Contig_96	27484	25496	-	Biotin carboxyl carrier protein	FIG00005415	idu(1);Urea_carboxylase_and_Allophanate_hydrolase_cluster idu(1);Fatty_Acid_Biosynthesis_FASII
fig|6666666.28482.peg.5994	Contig_96	28495	27761	-	Putative O-methyltransferase		
fig|6666666.28482.peg.5995	Contig_96	31720	30011	-	hypothetical protein	FIG00638284	ff
fig|6666666.28482.peg.5996	Contig_96	33088	33447	+	NAD-dependent epimerase/dehydratase		
fig|6666666.28482.peg.5997	Contig_96	33501	34745	+	hypothetical protein		
fig|6666666.28482.peg.5998	Contig_96	34824	35564	+	hypothetical protein		
fig|6666666.28482.peg.5999	Contig_96	35741	35866	+	hypothetical protein		
fig|6666666.28482.peg.6000	Contig_96	38184	35938	-	hypothetical protein		
fig|6666666.28482.peg.6001	Contig_96	40483	38246	-	Glycosyl transferase, group 2 family protein	FIG00003788	isu;Exopolysaccharide_Biosynthesis

fig|6666666.28482.peg.6002	Contig_96	41851	40850	-	PE-PGRS family protein		idu(16);PPE_gene_cluster_in_Mycobacteria
fig|6666666.28482.peg.6003	Contig_96	41897	42013	+	hypothetical protein		
fig|6666666.28482.peg.6004	Contig_96	43827	42313	-	PPE family protein	FIG00013075	idu(57);CBSS-164757.7.peg.5020
fig|6666666.28482.peg.6005	Contig_96	44045	45322	+	RNA polymerase sigma-70 factor, ECF subfamily	FIG00952839	ff
fig|6666666.28482.peg.6006	Contig_96	46044	45319	-	Cobalt-precorrin-6x reductase (EC 1.3.1.54)	FIG00019212	isu;Cobalamin_synthesis icw(1);Coenzyme_B12_biosynthesis
fig|6666666.28482.peg.6007	Contig_96	46691	46044	-	Cobalt-precorrin-4 C11-methyltransferase (EC 2.1.1.133)	FIG00000933	icw(1);Cobalamin_synthesis icw(2);Coenzyme_B12_biosynthesis
fig|6666666.28482.peg.6008	Contig_96	47962	46796	-	Cobalt-precorrin-6y C5-methyltransferase (EC 2.1.1.-) / Cobalt-precorrin-6y C15-methyltransferase [decarboxylating] (EC 2.1.1.-)	FIG00001256	isu;Coenzyme_B12_biosynthesis isu;Coenzyme_B12_biosynthesis
fig|6666666.28482.peg.6009	Contig_96	48708	47959	-	Oxidoreductase, short-chain dehydrogenase/reductase family	FIG00993242	ff
fig|6666666.28482.peg.6010	Contig_96	48741	49154	+	FIG01000472: hypothetical protein	FIG00821178	ff
fig|6666666.28482.peg.6011	Contig_96	52642	49769	-	FIG00823413: hypothetical protein	FIG00823411	ff
fig|6666666.28482.peg.6012	Contig_96	54961	52706	-	amino-acid metabolism	FIG01368229	ff
fig|6666666.28482.peg.6013	Contig_96	56025	54988	-	Probable dipeptidase PepE (EC 3.4.13.-)	FIG00820965	ff

fig|6666666.28482.peg.6014	Contig_97	988	746	-	Molybdenum cofactor biosynthesis protein MoaD	FIG00003839	isu;Molybdenum_cofactor_biosynthesis
fig|6666666.28482.peg.6015	Contig_97	2108	1029	-	Molybdenum cofactor biosynthesis protein MoaA	FIG00002018	icw(1);Molybdenum_cofactor_biosynthesis
fig|6666666.28482.peg.6016	Contig_97	2518	2105	-	POSSIBLE CONSERVED INTEGRAL MEMBRANE PROTEIN	FIG01037873	ff
fig|6666666.28482.peg.6017	Contig_97	2681	3091	+	Cold shock protein CspC	FIG00001928	isu;Cold_shock,_CspA_family_of_proteins
fig|6666666.28482.peg.6018	Contig_97	3226	5187	+	Butyryl-CoA dehydrogenase (EC 1.3.99.2)	FIG00009149	idu(39);Isoleucine_degradation idu(39);Lysine_fermentation idu(39);Butanol_Biosynthesis idu(39);Acetyl-CoA_fermentation_to_Butyrate idu(39);Valine_degradation idu(39);Anaerobic_respiratory_reductases
fig|6666666.28482.peg.6019	Contig_97	5220	6224	+	FIG00820534: hypothetical protein	FIG00820533	ff
fig|6666666.28482.peg.6020	Contig_97	6531	6229	-	hypothetical protein		
fig|6666666.28482.peg.6021	Contig_97	7042	6551	-	POSSIBLE CONSERVED EXPORTED PROTEIN	FIG01351395	ff
fig|6666666.28482.peg.6022	Contig_97	8715	7051	-	possible membrane protein	FIG00821477	ff
fig|6666666.28482.peg.6023	Contig_97	8871	9776	+	FIG00821400: hypothetical protein	FIG00821399	ff
fig|6666666.28482.peg.6024	Contig_97	10228	9773	-	FIG00820282: hypothetical protein	FIG00820281	ff
fig|6666666.28482.peg.6025	Contig_97	10538	10251	-	POSSIBLE CONSERVED TRANSMEMBRANE PROTEIN	FIG01417063	ff

fig|6666666.28482.peg.6026	Contig_97	10678	11109	+	Transcriptional regulator, MarR family	FIG00554610	ff
fig|6666666.28482.peg.6027	Contig_97	11106	11930	+	putative rRNA methylase	FIG00544877	ff
fig|6666666.28482.peg.6028	Contig_97	11927	12199	+	PROBABLE TRANSMEMBRANE PROTEIN	FIG01329827	ff
fig|6666666.28482.peg.6029	Contig_97	13095	12340	-	FIG00996668: hypothetical protein	FIG00820358	ff
fig|6666666.28482.peg.6030	Contig_97	14396	13278	-	Phosphoserine aminotransferase (EC 2.6.1.52)	FIG00000445	isu;Serine_Biosynthesis isu;Pyridoxin_(Vitamin_B6)_Biosynthesis
fig|6666666.28482.peg.6031	Contig_97	14606	15631	+	possible transmembrane protein	FIG01327232	ff
fig|6666666.28482.peg.6032	Contig_98	389	886	+	hypothetical protein		
fig|6666666.28482.peg.6033	Contig_98	1091	1723	+	hypothetical protein		
fig|6666666.28482.peg.6034	Contig_98	1725	2720	+	conserved hypothetical protein		
fig|6666666.28482.peg.6035	Contig_98	3702	3532	-	hypothetical protein		
fig|6666666.28482.peg.6036	Contig_98	3865	3704	-	hypothetical protein		
fig|6666666.28482.peg.6037	Contig_98	5108	3870	-	putative phage head		
fig|6666666.28482.peg.6038	Contig_98	5873	5352	-	hypothetical protein		
fig|6666666.28482.peg.6039	Contig_98	6219	5866	-	hypothetical protein		
fig|6666666.28482.peg.6040	Contig_98	6539	6297	-	hypothetical protein		
fig|6666666.28482.peg.6041	Contig_98	6706	6536	-	hypothetical protein		
fig|6666666.28482.peg.6042	Contig_98	6891	6754	-	hypothetical protein		
fig|6666666.28482.peg.6043	Contig_98	8035	7736	-	hypothetical protein		
fig|6666666.28482.peg.6044	Contig_98	9258	8407	-	phage integrase family protein		

fig|6666666.28482.peg.6045	Contig_98	10936	9413	-	RNA polymerase sigma factor RpoD		isu;CBSS-349161.4.peg.2417 isu;Sigma-B_stress_response_cluster_1 isu;Flagellum isu;Transcription_initiation,_bacterial_sigma_factors
fig|6666666.28482.peg.6046	Contig_98	11960	11154	-	Polyphosphate glucokinase (EC 2.7.1.63)	FIG00002564	isu;Polyphosphate
fig|6666666.28482.peg.6047	Contig_98	12062	12928	+	Inositol-1-monophosphatase (EC 3.1.3.25)	FIG00002332	isu;Di-Inositol-Phosphate_biosynthesis
fig|6666666.28482.peg.6048	Contig_98	13496	12933	-	Possible secreted alanine rich protein	FIG00862420	ff
fig|6666666.28482.peg.6049	Contig_98	13764	14066	+	FIG00820574: hypothetical protein	FIG00820573	ff
fig|6666666.28482.peg.6050	Contig_98	14526	14071	-	PROBABLE CONSERVED ALANINE RICH TRANSMEMBRANE PROTEIN	FIG01105974	ff
fig|6666666.28482.peg.6051	Contig_98	14614	15057	+	Deoxyuridine 5'-triphosphate nucleotidohydrolase (EC 3.6.1.23)	FIG00000388	isu;Nudix_proteins_(nucleoside_triphosphate_hydrolases) isu;Housecleaning_nucleoside_triphosphate_pyrophosphatases
fig|6666666.28482.peg.6052	Contig_98	15054	15788	+	FIG00822685: hypothetical protein	FIG00822683	ff
fig|6666666.28482.peg.6053	Contig_98	16182	15850	-	FIG00994821: hypothetical protein	FIG00821556	ff

fig|6666666.28482.peg.6054	Contig_99	112	540	+	Diaminopimelate epimerase (EC 5.1.1.7)	FIG00000421	isu;CBSS-323850.3.peg.3269 isu;CBSS-84588.1.peg.1247 isu;Lysine_Biosynthesis_DAP_Pathway,_GJO_scratch isu;Lysine_Biosynthesis_DAP_Pathway
fig|6666666.28482.peg.6055	Contig_99	587	2014	+	GTP-binding protein HflX	FIG00000304	isu;Hfl_operon
fig|6666666.28482.peg.6056	Contig_99	2119	3279	+	Butyryl-CoA dehydrogenase (EC 1.3.99.2)	FIG00009149	idu(39);Isoleucine_degradation idu(39);Lysine_fermentation idu(39);Butanol_Biosynthesis idu(39);Acetyl-CoA_fermentation_to_Butyrate idu(39);Valine_degradation idu(39);Anaerobic_respiratory_reductases
fig|6666666.28482.peg.6057	Contig_99	3684	5858	+	Possible conserved transmembrane alanine and glycine rich protein	FIG01391152	ff
fig|6666666.28482.peg.6058	Contig_99	6553	5867	-	SOS-response repressor and protease LexA (EC 3.4.21.88)	FIG00000510	isu;DNA_repair,_bacterial_UmuCD_system isu;DNA_repair,_bacterial
fig|6666666.28482.peg.6059	Contig_99	6854	7348	+	Cell division protein DivIC (FtsB), stabilizes FtsL against RasP cleavage	FIG00485194	idu(10);Possible_new_toxin-antitoxin_system_including_DivIC idu(10);Bacterial_Cell_Division idu(10);Cell_division-ribosomal_stress_proteins_cluster idu(10);Bacterial_Cytoskeleton idu(10);Stationary_phase_repair_cluster

fig|6666666.28482.peg.6060	Contig_99	7418	7897	+	Ribonucleotide reductase transcriptional regulator NrdR	FIG00000382	isu;Ribonucleotide_reduction
fig|6666666.28482.peg.6061	Contig_99	7908	8393	+	DUF1794	FIG00020072	idu(1);Iron-sulfur_cluster_assembly
fig|6666666.28482.peg.6062	Contig_99	9082	8396	-	Phenazine biosynthesis protein PhzF like	FIG01247796	ff
fig|6666666.28482.peg.6063	Contig_99	10156	9116	-	Hydrolase, alpha/beta fold family	FIG01359650	ff
fig|6666666.28482.peg.6064	Contig_99	11165	10194	-	FIG00994994: hypothetical protein	FIG00821756	ff
fig|6666666.28482.peg.6065	Contig_99	11383	12210	+	FIG00821740: hypothetical protein	FIG00821738	ff
fig|6666666.28482.peg.6066	Contig_99	13627	12212	-	Soluble pyridine nucleotide transhydrogenase (EC 1.6.1.1)	FIG00001482	isu;Phosphate_metabolism
fig|6666666.28482.peg.6067	Contig_99	13726	14790	+	FIG00999418: hypothetical protein	FIG00820527	ff
fig|6666666.28482.peg.6068	Contig_99	15509	14814	-	Iron-dependent repressor IdeR/DtxR	FIG00040371	isu;Heme,_hemin_uptake_and_utilization_systems_in_GramPositives
fig|6666666.28482.peg.6069	Contig_99	16607	15648	-	RNA polymerase sigma factor SigB	FIG00132885	idu(2);SigmaB_stress_responce_regulation idu(2);Biofilm_formation_in_Staphylococcus idu(2);Sigma-B_stress_response_cluster_1 idu(2);Transcription_initiation,_bacterial_sigma_factors idu(2);Methicillin_resistance_in_Staphylococci

fig|6666666.28482.peg.6070	Contig_99	17139	16756	-	Possible membrane protein	FIG01121868	ff
fig|6666666.28482.peg.6071	Contig_99	17203	17442	+	FIG00998432: hypothetical protein	FIG00822566	ff
fig|6666666.28482.peg.6072	Contig_99	18429	17443	-	Ribonuclease BN (EC 3.1.-.-)	FIG00136522	ff
fig|6666666.28482.peg.6073	Contig_99	18701	18495	-	FIG00820633: hypothetical protein	FIG00820632	ff
fig|6666666.28482.peg.6074	Contig_99	18789	19154	+	Glutathione S-transferase domain protein		isu;Single-Rhodanese-domain_proteins
fig|6666666.28482.peg.6075	Contig_99	19516	19139	-	Putative translation initiation inhibitor, YjgF family		
fig|6666666.28482.peg.6076	Contig_99	19695	20120	+	hypothetical protein		
fig|6666666.28482.peg.6077	Contig_99	20149	22794	+	hypothetical protein		
