# Supplementary material for: Comparative Genomic Analysis Reveals a Possible Novel Non-Tuberculous Mycobacterium Species with High Pathogenic Potential
Source: PLoS One. 2016 Apr 1;11(4):e0150413. doi: 10.1371/journal.pone.0150413 (PMC4818103; doi:10.1371/journal.pone.0150413)
Supplement: S2 Table — (PDF) [file pone.0150413.s006.pdf]

Supplementary Table S2: Full list of predicted GIs in UM_CSW genome.

Genomic islands	Genomic size (bp)	GC content (%)	Contig number	Start	End	Gene description	Gene-sequences
GI1	11040	62.3	Contig 2	63446	74485	hypothetical protein	MTQTERLPAYTEAHPPTPSSDELRAQIPGWGADLDPKDRPSNPKLRQDLPTETHWDFPERQPEKWPRERSVEHRFLTPVFGTAQPPSGVSGMLRRYAYKFSEGRAAHWLILLYADRVDTAEHHVRSFLTTRPDNPITETGIKSELTHHGIQSRLGRKRSDLVHIWMDPFIVAGPWILGGQGIASLARKAVKAVGNNGGRGGGR
						low molecular weight T-cell antigen TB8.4	MTGADGAVSTNPEPQPDQDWIVNTKCSFPQFVAALNAEDIRAAQQFAITPAAQSWVGSYLASSPDQRRQMLRRAQSIPDARQYIDTMLRVASTCKNF
						hypothetical protein	MRYEEFISSVAERSGLFQGDAVALTRATLTTLAERISGGEARDLAAQLPSPLQDALLPTEEEAEAFSFDEFVNRVAERSGRDTEAAEAAVDAVLATIRDAVTPGEFDDVLSQLPSEFQRLGTR
						protein of unknown function UPF0118	MGPPGRRFDWHSPFFVGLTASAGVAVTYGAVRGLAAVSSVLLLIGVAFFIALGLEPAVSWLVGKKVPRWAAVAVVLFAVIAVVAGSLAAAIPPLVQQAREFTDQAPHYIQHIGDHSSWIGRLNDRFHLQQRITETVHGSGGAVVGQALKAGEAVFGAVADVVVVVVLTVYFLVDFPRIRATLYRFVPHSRRPRAILIGDQMFAKVSAYVFGNIVISAIAGVATFVWLVIFDVPYPLLLGIFVALLDLIPFGSSVAGVIVAAVALTVSIPVAIATLLFYTLFRFVEDYLLVPKIIGRAVNVPAVATLLAVLVGGALLGIVGALVAIPIAAAIQLAARELLFPTLDKL

hypothetical protein	MVVIGHIADPEEPRHGRRGKAVQSTIARLAAAMVGTAGIE<br>DALSKLTSASLALIPGADCAKISTIENGHLRSITATSQLTSSLDS<br>AQQAAGQGPCLETISAKKAIRCDDLRTDTRWPRFTPRATTA<br>GVYSVLSTPIDIPGETGATLTLFGFRAEAFGPDAEAVGAMLA<br>NHAAIALMHVKQERQFKAALATRNVIGQAKGMIMERFGID<br>AARAFAMLRAISQQTNTPLRELATRLVDGAKR
hypothetical protein	MQRLRAGTSIIYASGSLDGETRAVLCRAVADELTQGSAQLV<br>LELSSATSIDDTAVEALVDATGLAAESDTSICLVTSPTGPIARA<br>LAAADLLERFEIFATVDEAQRHP
hypothetical protein	MGIAHRAEPPSLHSVFNLSPLLFDGRRADAVLELAAEAVRSL<br>GKCSSEGAYRVVDGSLVDNSDPDRPLDSGLDAAVTASVGV<br>DHEIVLPDSNWRYAMTLRTTNTVTGVLVVRADNPASCHEL<br>VLLKMLAQQAATAMASADTIERERRQHVQLRELTDRHEETI<br>HRLSRSVAELERREHIHKALTNLSGSADAASIADALHDLTSLT<br>VSVEDTFGNLRAWSPAPIPTAYRAVGGGNREDVIRNAGSR<br>GHHSDCGNRIFSLIRARAELLGVVVLHDPQRRADRLDIFALE<br>YAAAVLAVEFSHQRSIAETESRLSRDLVDDLLTGTDNATAYA<br>RGEALGYNLHRPHRVTVLQWSAEIDGALIARSATRWATSA<br>GLHPLCARRPSMTILLTEDLPQPQSLYRAISAAVGHGRGWI<br>AIGSVATSPSELPRSFAEARRTLRMQKASVGRHGFRRFDDL<br>GVCRILDPSDNGPEVGEFLAEWLGPLMAYDQEKNAELVNT<br>LARYLDSGGNYDQTARALNIHRSTLRYRLGRIRDVSGRDLQ<br>NVETRLNLHLATKVSEMIGETQPIMRAASTGERRPSDEGM<br>PK

putative transcriptional regulator, AraC family	MTELPFDLAPEVRTTDIDEAASALGRVYVTAELVPNKTNSV<br>GMHMNAAQLPLITAGYLGFGADVTVRANDVTAYYIDAPLS<br>GSAVNRWRDGELVKTTTGSVAVFTPGTPCVLDWSGDCGQI<br>CLKVSEPQMRRQLEAMLNRPVRKRITFARQFDLSTTAAHD<br>WYHLVSLLAREVGRTDGLLNHRLAAANLQLLLIQGLLQIQP<br>HNYTEALAESEGAASANVAKRAIDLLHAHPETMWSTAQLA<br>RQTGVSARALQRAFERSDQPSPMAYLRRLRLHRVYTELAAS<br>SPDSVTVTMVAGRWGFVHLGRFAGQYQQLFGETPAETLR<br>HRVADALPTQPQPPL
hypothetical protein	MFISDRTTPQEPDMTTADEDSERDFLAKCVADAYQMLEIIP<br>GVNPNGAALVWLADQFMQVRRRTESHLASTA
hypothetical protein	MKRPNSRFLLAISRTGWLGRTVEILLRGLLASDTGTVKVSMP<br>WSI
hypothetical protein	MWSEYYDVATECAVTLRMLLASMSNVTSIWGSPRGIGRM<br>LPNFETAQ
ThiJ/PfpI family protein	MPNMLQGKTIAILAADGVERVELVHPSAAVHRAGGQTELL<br>SLRNGWIQMRNNDLDPAGTFTVDRAVAGASIDDYGGLLLP<br>GGTVNPDKLRTDERAVAFVRGFVESGKPVGVICRGPLTLIEA<br>GVVAGRTLTSYPSVRTDLRNAGANVVDEEVVVDGNLISSRS<br>PDDLPAFCVAIVKAFAKTPAGTS
hypothetical protein	MAQDTENQHTTGTQTGTENQGTTENDSAGSQAPGGNPD<br>NRETVQNMDAPELRTAVIELLTIIDDSDEMDVVVTISDDGT<br>VVVEAA
ThiJ/PfpI family protein	MTHELRGQRVGILAADGVERIELEQPRGALHGAGAMTEVI<br>SLHPGEIQARQFDMEPAGSFPVDRLVGDVSADDYDALLMP<br>GGTVNPDKLRMNADAVAFVKAFADTGKPIAVICHGPWTLA<br>EADVIQGVRMTSWPSIRTDLRRAGADVVDEEVVVDRQFISS<br>RSPNDLPAFCGAIVVQFAKAKQPV

						hypothetical protein	MATKALLVRLEAKPGKEDAVEKFLLSALPLVEQEPGTKPWF<br>AVRFSPSIFGIIDAFPDEAALEAHLGGAVGKALAEKADELFAS<br>PPDISKLDVLANKL
GI2	14745	61.7	Contig 17	84910	99654	hypothetical protein	MPGITRSSAHSTSSIAERLTLRPDTTRTASRNTDGAADAGRP<br>RVPEAGKAAGRESGSGRSDGGSGVDRVPAGTNGLVICAKS<br>VRDTVDGIVDAELSASDALGLRFPRPNMVASAAALFEPAEP<br>WAHGRDRLDVEPECGRFRITIGPGVVRLGWTNPVRAEKAS<br>ERTVGQHMQDVAGEVDRIKAGRDVPDPSGRAITEWSRKS<br>RAAMCRTFAELDYTPMVESGRVPAMVTLTYPGDWESVAP<br>DGASVKRHMMLWRKRFQREWGEPARYIWKMEFQRRGA<br>PHIHLWMAPPYTVGRSGRRFSEWLSQEWAEVVAHPDPEQ<br>RTRHLRAGTAIDSLNGLRACDPKRLAIYFTKHSSPNRLGDKE<br>YQHIVPEAWRRPGRGPGRFWGVHGLERATAVVEIAQDAY<br>LTARRIIRRWSRSQAIHSDCTHCFPTALVPRMTRVAVQRTN<br>SKTGKVRHRNVCRRRLLCRQGGFAGGYALVNDGPAFASQL<br>AAALSADRAKAVRDEQLAGRK
						hypothetical protein	MHRQPQLLNDCVHLTSLFSVGKSEFIKLPTIFMILQPQE
						Transcriptional regulator, TetR family	MREVPRKIADRLPPAAALFADRGLNDTKIEDVAATTGIAKAT<br>LYYYFAGKEEILAFLLEDVLQQVAHEVAAVVEAEGSAAQRLH<br>AVISAQLRVMAQRPAVCRALIGELGRAARMPVIADMISAAY<br>FAPVETLLRAGAGDGSLVTLDNPAVAAIALFGAVTTSALTYL<br>VLDDALDEDRIARTIHDLVFVGLRPR

putative cytochrome P450 hydroxylase	MTTVQLRYDPFDATIQDDLYPIYRQLRDKAPLYRAADSNTW<br>VFSRHEDVVSALLDHHTYSSVDGIFPTPPGSDFHASFLPMMI<br>LMDPPRHDQLRALVSKAFTPRRIAALSSGIEDLAEDLSDCLD<br>RGAGSVDFAADFAAVLPAMVIADLLGIPREDRAQFRQWST<br>TMVQSNPARGEMGEGLAAAAAVYAYFTDFLAERRREPRG<br>DLMSALVSAEIDGKRLTDEDLLGFCLLLLIAGHETTSNLLSNA<br>AVVLASDPDIRRRLVADDNLLGPAVEELLRYDSPVQGLSRTL<br>TRDVTLHGVTMSVGDSVLLLFGSANRDERVFADPDVFDIGR<br>KPEHQVAFGRGIHFCLGASLARMEARIALRALLARVPNWEV<br>DLENAQRLRSGLIRGYMSLPISWSAN
Transcriptional regulator, TetR family	MERDGFDMPVDDDVDPRLVRSRTRLLDAATKLLSAGGIEA<br>VTIDAVTKASKVARTTLYRHFSSSTQLLAATFERLLPQVHLPP<br>ATGSLRDQLIELLSRQATLFQEAPLHVTTLAWVALGPTSNST<br>QETYDRHALRTRIIEQYRQPFDLLLQSPEACADLDEFDPELIL<br>CQLVGPVAFARLTGLRAIDRQDCERIVDDFLAAHRRKVDEP<br>AS

hypothetical protein	MADNGWVSSGVATLSRPVREQLRKVAAGEGAYSDRLARL<br>AGFSIRHKVLVIGAWVVTAGVLAMLFPQLETVVKQQSVNL<br>VPPDAPSLQTVDRMGTAFGEQGSKTTVFVAFEDPTGLTAP<br>VRQRYKTMVSRLRADSQHVRLVQDLLADPVTAGQAMSQD<br>GKAWYVPVGVAGTLGDPRAAESVRAVRAIVAESFSGTSTIV<br>RVTGPPATFSDLIDSAEQDLIGISIVTAGLIAVILLVIYRSLVTAL<br>LPLLVIGVSLAVGRGVLSALGESGMPVSQFTIAFMTAILLGA<br>GTDYSVFLISRYHEQRRQMISADLAVINATATIGRVIMASAA<br>TVAFAFLSLVFAKLSVFSALGPACAIAVFVGVAATVTLFPPVL<br>ALAAKHGIGEPKADRTRRYWNWIAVAVVRRPAPLLAASLAL<br>VLGLAAVALTMHISYDDRQGQPATTTSNEGYRLLDRHFRKD<br>IVITQFMVVESPTDMRTSKGLADLDEMASRVSQLPGVTKVS<br>GVTRPTGARLDQAQLSWQNGQIGNKMAGAVAKGDAHKD<br>DLAKLTSGADQLAGGLAQLDTTLRTALTPLTAVLTQAQSSG<br>SQFQHFRPLLQQLSATTPTIDQAIRTGPGLRQEAQQAQNAI<br>ATIDPLVGALNTSPWCATTPECAQIRDQVQILVTLRDGGFFS<br>QLANLGDMYQPGSDNAAGTVADLQSTVTSLNKAFGALGD<br>PADMAGNIRRLQNGISQLASGAQALATGVHTLADSNIEML<br>SGMSQVATQLQNSARSSAGSDNASGFYLPANTFENRQFAD<br>VAKHFMSADGKTARFAIESSYNPYSSQAMDLAQKITEVAGA<br>ARPNTSLANATISMAGFPAVNSDIQRLLSADFHLLAIATLVIV<br>GVILVLLLRALLAPLYLLGTVVLNYGAALGIGTLVFQYGLGKEI<br>AWPVPLVAFIILVAVGADYNMLLISRLREESAHNIRVGVLRT<br>VANTGSVITSAGLIFAASMFGLMVGSVGIMIQVGFIVGCGLL<br>LDTFVVRTLTVPAIATLLREASWWPQRKSSTHNGRPHRTT

Hydrolase	MADCYRHAATHPVVLLLHGGGQNRHAWATTARRLYSHGY<br>TVVAYDTRGHGDSDWDPSGQYDIERFVSDLISVRGHVSAD<br>SPPAVVGASLGGLIILATHLLAPPDLWAAVVLVDITPRMEFH<br>GARRILSFMAGHPDGFGTLNDAADVIAEYNPRRARPENLD<br>GLHKVLRQRSDGRWIWRWDPAFISSNFDVLQGNLMTGSE<br>EFDAISGFLAEGARRITAPTLLVRGALSDVVSQETVSEFRQLV<br>RHAETTDVTGTGHMVAGDNNDAFTAAVTDFLDRAMRTLT
FIG00825226:<br>hypothetical protein	MGLTDASKGAVMHTQWIEQCTVQRVSLHEGLILDLDGYNE<br>LVISRPLRLTLPPAGAWPSDEVLIDPINLSPEERPLLDLAGAIC<br>TRAWCDDDGALHLCFSPGHRIDVDPDVAATSWELYGKCH<br>GYVACLPRGRVRAIRHDLPVDENDSDATQPKAMAHQ
hypothetical protein	MAITIPSGRPNWRFMRYVRPPTRDSKLEPLYPLRPATRPVR<br>LGIDVGTIAEPPEEGYITGFLTRDEIEVHLLIPAATEAPSGWTE<br>LLDEPPCHTVNFTNVADAGKFCDAAEFSVSTARGESYRAWS<br>KARFFAAYQQLDEHDAPDGLPPLTLDQRHRAAAYAAAAGA<br>VGIDAIVTTAPTAGRTDVADNDVVVSVTPDAAVPLIGHYLR<br>VTSNPVVTVERGMLVGGGSWETTESTATIVNLYDWGTVSG<br>LPYFDAASMFAAAAKGGPEAAEAFTSVRIRLRRAARAFDDL<br>LAALSNPLDGKRNEDVAEATAEAFDRELLYLAAVFDIFGRAY<br>QAMVDPSVDRKKARGSLDSRTFIDKEVRTQYDQSLLGDVT<br>RLRVYAWLCKQLRNHIHDGVLAVDTHPGRSYGNTMNVAL<br>NLSVIPELALGADNEMTQHHYDALGVWQTEPVSPFTGSSM<br>VADLATTGFTLIRAALEYIEAFTKLIVRNKPANAPSSSAFLGCV<br>QARPGEVEPAPPKRAVFYQALFGLHPDSV
hypothetical protein	MAAVDQSDNPMGQVLVNAGETFYLHRQSGFLKDFAPNTV<br>LKRFA

Transcriptional regulator KorSA, GntR family	MLQLGQIDRADDKPPYRQIAGMLREAIRSNRLAPGERLPSE<br>TELIEHFGVARMTVRQAVQELRSEGLVVSQHGRGVFIRPTP<br>PIRRLASDRFARQHRAAGKAAFTVEAEKSGYSPQVDNIAVS<br>REKPNSFVAERLRLSPDDDIVVRSRRYLANGRPVETAVSFIPA<br>AFAEGTRIEQVDTGPGGIYARLEENGHVLGHFTEEVAARMP<br>TPEERRQLELEPGVPVLTVLRTAYDANDVAVEVCDTVKVAS<br>AYLLEYEFPAR
FIG00831412: hypothetical protein	MSVPKWLKVSHQQVFPRKAFVVSDVTAVIDYERSTKDNKV<br>QATDRESGAPLWQVEVLDGDPTASKRARTLTVKFATPKQP<br>VCPPNTAGMPFTPVIFEELYILPYVDRSSESGRIAWSFRASG<br>MTADTGKAGSSAGDRVSA
Transfer protein traSA	MSPYDPVVPSDPYSIPDPDAGIDFFDLTHLLAVGAMWLIAV<br>IGVVAVSLVVWRVRSPATFGRYVATPACRARWLIWASISW<br>PRVAKACGLSTPEHVTRTDAQGKSRTRTVWTHPRLLGVSM<br>FGDCLRMTVRTRTGQTVDDLENAVPAIRDAVGAHSARSTLI<br>GPGTVRVEFVMKQCLSEAETAAFPTSVEPTGVEVGRRENGS<br>AWTLRVAGLHTLTVGCSGAGKGSVFWGIAGGLGPAIKAGT<br>VRLFAVDLKYGIEVSVGSALFSGIATTEANAARLLTNLEELLDS<br>RGRRMAGRARSHTPSTAEPLVVLLIDEFAGLTAYMTDAALR<br>KQVAGSLSRILTKGRAVGIVVAAFMQDPRKEILPMRGLFTQ<br>TVALRLRSRDEVAMVLGDGLADAAPAHRINPNEPGTGYVIA<br>EDGSTMRVRADFWPDSLIRSVAQEYGPTSSSRAGSAEN

GI3	10251	62.8	Contig 22	38227	48477	Coenzyme F420-dependent oxidoreductase	MAKLSVATPVVMLPAANGSWEKDASIEDLAQIAEAADRLGYHHLTCAEHIGLPAAERERRGTRYWDPLATFGYLAARTQRIRLTTYVLVLGYHHPLEIAKRYGTLDKVSNGRLILGVGVGSLKEEFELLGASFDDRGARGDDALRALRASLSVPEPTYHGEFYSFSGMVVDPCAVQDRVPIWIGGRTLRSLRRAVTLADGWAPFNVSPQQAREWLGRFDIQPGFEVLLPPLAPLNPIDEPDRTRHIIADTVAHGATIVSTMFRHRSLQNYLENLHALAEIYPPDGGAA
						hypothetical protein	MNRVKAGVFSLTPPAPADDDGSYLRWHLLDHMPEQYQLPGIVHGLRWIADGDYADHRLAANGPLSQIGNAVHYLVSDPVEETLDDFVALGSALRENGRFPISRPYLQVAGLRLLQWHSAPQALISGEVVPFRPHRGILLIVEEPADGRSNEWLQWLHAEHYPALLATPGTVGAWTFGTSTGWIHLPAGWRTDQQYITVVYLDADPLATTDAVAALVEDRWRSAAVRPVFAGPLRTMISWDAWPTS
						Transcriptional regulator, TetR family	MTDQEPRLRQRTDGRLNRSRDPAILNAALAALAEHGYHATNMDDIAARAGVGKAAIYRRWSSKAALMTDALVYWRPDLLSADVPDTGSLAGDLDALVERIKRSDSDLVSNDLVLRVAMEAAHEPELASALDDLMLGKGKRTMTAILTHAAKRGEISGDRDWSLVADVLVAMGLLQVARGQTVDANFVRQVIDTLVLPAVRTTNNA
						putative cytochrome P450 hydroxylase	MDPAMFFGTDAIQNPYPLYEQMRAASPVHRIGDSDFYAVCGWDAVVEAVARVDDFSSNLTATVVFEGDTGSVEPYSMMPLGDPMHSLATADDPAHAVHRKILLPHLSAKRVRVIEEFAQETARRLLSESLVDGRIEWMSTVANRLPMMVVARLLGLPHDDVDTLIRLGFATTTLLDGLVTPAQLEAAGTAVMELSAYVLDHFDKAATDAQPGLMSDLAARCASGQLDQPVALSMMLTLFSAAGESTASLLGSAAWILVSRPDIQEQVRAHPDLLGPFIEETLRYEPPFRAHYRHVRRHTALAGVELPADAHLLLMWGAANRDAAQFESPDEFRLDRGGAKGHVTFGKGAHFCIGAALARLEARVVLGILLERTDWIEAAEIGEWLPSILVRRLERLHLATR

Transcriptional regulator, TetR family	MAGVDWLVGQDRGSEATARIHAAAADLVSERGWEGFTIGALAAKVHCSPATIYRHAGGRTAIRNAVVNIHAARVIESMRDAIEGLTGAERVVSATAIALQRIRSDPLTQMIRSTRPPIDDDWMPSSEVVVQCAQEVLGQHDPDPLAQQWLIHVFLALWIWPMKDAEAELQMLQRFLGPPYDAAGS
hypothetical protein	MMQGDLEIAYEIIGEGEPYILTGGGRFSKDAGGVRELAEAIADRGKKVVIYDRLNCGASAVCFEGSSESVMQAESLARLIVDLDLGPTVLLGGSGGARISLLAAARHPEIARAVVMWWPSGGAFGSMSLAEVYNFPSIRAAWNGTMADVADDPTWAEVITRNPRNRDRFLAQDKQTFIETMERWAHVYCACGNPLVAGLTDEEAAAITLPVLVFRSGKSDYFHTRATSERLAANLQNAEIIDPVWGDREFLDRYEDSRDNGVSVWINWKQLAPQIVDWTEKVLD
L-carnitine dehydratase/bile acid-inducible protein F	MIKVMEGVRVLELAQYTFVPAAGAILADWGADVIKVEHPVRGDTQRGFLYMGGIKLEADRHPLIEHPNRGKRSVGVDVSTPGGQEVLYELAKTSDVFLTNYMPQARQKNKFDVEHIRAANPNIIYARGSAYGDKGPERLVGGFDGTAFWTRSGVGHALTPEALGGALPQGIPAFGDSIGGMNIAGGISAALFHREKTGEAVEIDVSLLSTAWWAAGASVTQGMETGETMRSTMPDETPPIFNPFLGNFLTSDGGTINLCIVSPTGYIRDTFEHLGLPELADDPRFSDVMPLIRNAAEAAELVAKAIRGKSFDYWREHLKTMKGQWAPFQSLLELASDEQAIANDMIVEYEASAGDKPFRVVRGPVQFNHEPLETTRAPQASEHTEIVLMELGMEWDRIEALKDAGAIA
hypothetical protein	MDDPVTDVEDTVESFPGIESGGKSTEVGHFMAAMRRLQDVVVSTDPDNELWGSAAREVNDLCERFELHRVPAGFGLRGRGPHLPGLGHPLMPPWMMTEYGPDGVTMQGHFSQYHVGSNNAVNGGVIPLLYDWQFGMIVSAVDRRDSRTAYLHIEYRKVTPINQPLTSRGRIETIDGRKVFVTATMTDSDGSVLSEASGLMIQLKSNQP

						Long-chain-fatty-acid--CoA ligase (EC 6.2.1.3)	MYPGHWGITKPDHPAVIMAGTRETITYGELDRRSRCLAGFLWSRGLRRGDHVAILMHNDVRYFEMLWAALRSGMYVTPVNWHLKPDEVAYVIGDCEAKLLFTSARQSDLVEDIDWRTLPALTTRVMADGTTTGFEPYESVVAFEGEPAEESSGAAMLYTSGTTGRPKGVLRPLPDTKPGDLDAASHRLAALFGIDEQSTYLSPAPQYHAAPLTFTLAAHRAGATVVMMEKFDARDALKAIDMHGVTHSQWVPTMFVRMLALSPEDKQAADLSTHSVALHAGAPCPPEIKRQMIDWWGPILVEYYGGSDGGGFTVIDSLDWLAHPGSVGRSRTGAVYIVDEDADAVVGPGQTGTVYFSGDPGFVYKGDPEKTERSRRAGGELSTMGDIGYLDADGYLYLTDRKEFTIISGGVNIYPREAEDVLILHSDVQDVAVFGIPHHEFGEEVKAAVQLKPGVTATPELAGQLIAFTRARLASFKCPRSVDFLEQLPRTPTGKLRKADLRSAYAQGNSTP
						hypothetical protein	MANKMFRKYGYDAVTLEQIADACVMSVRTILRYFNTKEALALAGEHELLESFRAQLGVRETDAVTCWRNFRNETLPLMETPESRERMRAIYETPPLLAEFLRIGEGYQQCLTAAIAEDWGEVASLEATVFASVLVSTTSAAFRRWYVSEEPFDLVALQDIVDRVCVAFDKGRQSADGRRVGRVSAANTAPTRRAPTGRR
GI4	11329	61.3	Contig 22	102354	113682	hypothetical protein	MKMYAARILSIMRLAGGISSPALGRTNFTSSSKRSSFNSFQIWILPPGKVL
						hypothetical protein	MREGFSWATATDSGRGSQRRSWAAAVDIFVDMAAADGGAATSAHFSATRIARLAISIVQVWLYEWWHGPRDSAIEEDAERLVSFLSLWLRDED

hypothetical protein	MYRWVAAVMTAGYIVTAAGTGLKIFPTTPITGLVACIIFTNAIFVAFWACWFLEGPGKKRTTLEKADEFTMIWACVAAVGSEMTWELPWLVADALGRAHITPHDRWVYIWYYYARVDERYLISDGALWGMEAVVILAAVLLLVQWFRLRSAPTHDPKRISALWWSLLAMVVMLTIFVVYYAAEVRHGFANVQRGFWDITVIFVYENLPWLAAPIISIPLTAKQLAYLYREQGRRTALAEEGALADDRADVRSLV
hypothetical protein	MHASAPSRKVADLAERLQTIHFGREQSAEGGTIVSPSARIGDC
Protein of unknown function DUF1486	MTIFKQEIKAAMTPDMTTDSALRRSREELVLRHVAAENAHDIDAAMATFTHPRYEIMPTGMVFDGAESVRTMLLQQWAQLPMLQYSAEAIYHGEHGLVVETRTTAPGRSIDMLSMNLFGFRGADLVLERCYFDQSLLAAQLEALAPD
hypothetical protein	MDGPSALRRRRFKFTETPVYGTDDRSLGLRPVGVPAEASMQQ
hypothetical protein	MTRLTRNGSMISIEIDPTRVSMRSKGGATPSEHRRWGVRMLGPLAHSLSRRRIHE
TesB-like acyl-CoA thioesterase 4	MASPQEPYFTENGKTFVPSDVARGGWGPTVGGHAIGGLLARAVESERADETLIPARFTVDILRRVALAPVRVEASVVRSGRRMQAVDAVMTQDGELVARAATLFLRQSEQPQEIPWTPTRSMPPLPEELTALPKGVPMQIVPYNDGVEMSGQLPWQQDGLRQAWIREIRSLVGDEELTPFIRAAMAVDVTASMTGFTRTGLGFINADYTLTLCRLPKGSFVGLAGLTHHSAAGVGVGAATLFDAEGPIGTGVTTAAANTTFRGGAL
Transcriptional regulator, TetR family	MRRSNLNAVARRANVSRSTLYRRFPTKEELLYAVVGRLTESIFAELQHHTTGMTAKQLVVEVFCIAVRETTSNPVLHQLMVAEPDTLAALLGFFGPGMSAVLDRAVELAVAQAINAGAKMPPADLRIVVELMIRLTTSLMNSPSAILDADDPAAARQFAQKFFAPMIW

PROBABLE CHOLESTEROL DEHYDROGENASE	MGRNLILELLAQGHQVRSLDLTRNPDADSRIEVAIGDICDPR<br>VVAEVVDGIDTVFHTAAVICLLGGRAATSQYREHSFAVNVE<br>ATQSLVSHARDAGVSRLVYTSSNTVVMGDEPIVGGNESLPY<br>TTRFADLYTETKVVAERLVLQHNGSGGLLTCAVRPSGIWGP<br>GDQTMFRKLLEAVASGQVRALIGSRKTVMDYTYIGNLVHA<br>EILAANQLGPNGRASGQAYFITDGEPVNVFEFARPVVEACG<br>QHWPRLRVPGGLVRAVMTWWQWLHFRLGVTAPPLEPQI<br>VTRLCVDNYYSIDKARRDLGYEPMFTREQGLASSLLYYTDM<br>FKTMKHQAG
hypothetical protein	MQNFNRPVAAGRKRVYVAMFVLGCLVTGIPFFGVGFGLM<br>PPEPTYEIWGALIYIPLLVPLFTAWRDAPGENGTWTERAAEF<br>TMVWFPVTASSQITWETPWLIGDLTGVMHGAGPQDRWV<br>WLWWGYGAADTRYLTSDAGLYGMEVVAVIGGLALFTAW<br>FYLLRAKHTGDTMKRIKGLFVGIVACSMMLAVVTIYYVSETR<br>AGFRNLEQGFWEGFMFKFIFMNIWWVFAPLITIPFMIKQID<br>YLYKSMPRSIHQDVQPGAELQHAV
hypothetical protein	MPNRRSDALPGIRGARREMYRQQILTAAEYEFARTGFAETR<br>VATIAATADVSLATLYKNFAGKDEIWNALVEQRTNELVGLA<br>RAATVGVQSPLERIIVGARAQVDFFAQHPNFLRLNVKENW<br>SWATATEAARGGQRDAWRVGIAVMVREAEAAAASGELR<br>HLRPQIVAGLAVSALQVWLTDWVNSGVQRSPSQIADELQD<br>YLRRLLVVPRATDTTPLRD
hypothetical protein	MVRATGVIACSVFTIPMMIVFVTAWRDAPGENRTLLERAN<br>EFQTIWFFAAAAGSEITWETPWLIGDLMGWMHLTPKDTW<br>GFAWWYYGDVDQRYLTSDGGLWGLELAVVALAVILLVQW<br>FRLRSAATHDPKRINALWWAFFAMAGMLSLFATYFLMEVR<br>HGFSDFKLGFWEITVILGFENLPWLIAPVISLPFVAKQLTYLIR<br>SAPAATGTPLRSGAVSSSAAGL

						hypothetical protein	MNHINIVVEEFDAAVMHFQRVFGAQLVLDLPQPEWHACLLDIGDVLFEVFSPPQFLLNARYGPHYVGIEYQTGMDAARRAVTMRNIRLVRDIGHAIHTHPADTLGIAWEFFDGDFRVMPNLKWLEPLHSPERWRAHPIGYTGLRRLSVAVHDCDRAAQFLADFFGAMIVYKESRKSLSATIIGMLLADTTIELISPTGEGAISEHLHRYGDGIRSTVFEVADLERTHAHFKAIGIDLVDGDGPSTLAIEPRDNLGVMMEFCAS
						Transcriptional regulator, IclR family	MVNLLAGLRGETLSISEIAGQLRFNLSTCHSMLAELVKAGVLVRHPGTKRYALGPTLVTWGVASAFDSYRVLEFAEPEMSRLRHQLHVSCVARALVGPDTVVLARRDTDGPIAAFTPVGYRLAAVPPIGAEFIAWESDATIDEWLNRPQRALDTTERTRMHALLAEIRRDRYRFLWADPAEQTPLATELIQTSASEDEPGGDRLFELLQRRGYGRGPRTGTLSAITVPIFGADAKPVLALLLTFNPVPIDISEVEHYLRPLIHTCQRITTTIGGRAPN
GI5	14057	58	Contig 34	10279	24335	hypothetical protein	MPVAIGNPCVEEHVASENVESIETLRRNRVGRRARTSEHESDALE
						hypothetical protein	MQFSQRRLAVSRWGGCPTYWSTKFIASLEIRHKSPFEQ
						hypothetical protein	MFWQKVMINVACVEGQDPDVFDGGRVIAQSRSNRKGEALHHLVGVRLVLTERESD
						hypothetical protein	MILDLIQTVAAVSVPIVVAVIGYKLNHRLKLYEASQWRNQELIKARLQYFGQLAPMLNDLMCYLTFIGRWKELTPPDLIAIKRDTDRLFYSVAPLFSLAAVTAYQDFLGACFTTHTGWGTDARICSGFVRRREASREPWLAEWGQLFTHQDGDAIPESSMTAVRATYDKLLATLVEDVELLQPRDRYADSNLVANAR
						hypothetical protein	MPFHPEVSISRESAKFARNYGHLKEETVVINLPGVHRIW

hypothetical protein	MDALEGPSKISDSRQVALPKRLMDSLGWDKGDYVMFRLS<br>DDDPEVMKVVLESVVLRQLRRGEDAERTMREQRAETTTDE<br>SPQDRG
hypothetical protein	MTGLVRDPNVGFFDDQLTREAVVKLAACNRLTFFVGAGAS<br>LDLNLPTWSALVSDLLKDTISANFGPDSPVETICAELVNQLF<br>QVPAASVIDSLLFDEARASLSGRPARSTIHAKMLSTRNERLR<br>KSLYKRDRFGDITVGPTLVGKMLELAILLRRAGVDVHIVTTN<br>YDNAFEEAALHEPLFSIMSTERLRLVLFAESPPEAGDLDEGDI<br>PVVHIHGLLRAPSGYAAAGHNPIEGKIVFSEVDYIDWETGDF<br>RTYLTNRVQSGGLLTIGASLRDNNIVARLRDGTRSNNEPRYA<br>LMPSESEFKYLSDKNIAEKYWMPFVEMAARRGFIFGVSILRP<br>DFYGQVFQFLQELGVNVSSRINPGTAYVEYRARIRQWAVA<br>WSTVRESDEPGMRGNIETACRHLAVKFEALPQFDHCKVEV<br>WARCEPDDRTMRRWCSSQSTWRPDAWPHTTLIRPLSKQ<br>NAVMAFSSRGTVINKTPGSADRRWTHCLTVPVFLDGEPWL<br>GLPVGALEILLHESGAATSDECLNYLRANATPWVKEISKLGE<br>KILTPEPWVD

ATP-dependent DNA helicase UvrD/PcrA	MTFKLAGKAKDAYEQDGRVMVLGGPGSGKTTLALLKAKRL<br>LSSLEPEQDVLFLSFSRAAVRQVVLRCRDVLSAPERDRISVQT<br>YHAFCMGILRAHGRLLTGRPARILFPDREKVAKAGFEGDW<br>NTERDRLATEESIYVFDHFASGAARLIGDVAKVAELVADRFP<br>VIILDEFQDTSDSQWELVQALAVRSTVIILADADQRIFEYDD<br>NVDPNRLNQVREVLKPAEFDLVGENHRSPDAGILQFADAV<br>LRNRALPETDDVQVISVYPRALESTAHAAVRWLYSRLRKQGI<br>QRPSIAVLARANGLVSDVSDWLSTARTYNGSELKPVAHDVL<br>WDVELSAAAAQIVASILEWPLHDAGRAVAQTLNAVANFYD<br>VKNAINNTPIASAVRARDSYRANADSLTGGGTVRLKAAKHL<br>VEQYPAMSAYGGDPAADWKTARDILAGADALKEIVQAARF<br>IRLFGATDEIGGRLSEQWDRSGGYDNAVELVRRALDQGRLV<br>TELREPQGCVLMNIHKSKGKEFDGVLIVEAQYKGVLFDGRE<br>EPPHMSSRRLLRVGITRARHKVIIVRPKRAPPLASP
hypothetical protein	MKACYLHIQNFRGIEDGRVSFQPHTLLVGGNNVGKSTICEA<br>MDLVLGPERLYRRPVIDEHDFYHGKYLDEDDSPLDVRIDLVL<br>TDLTSEERRRFGDQHLRLWDNKTDSFIDEEAGGSELADAES<br>VEWGLPVCFIGRYDKEEDDFVGETFFCHPEPVQDELDVDQI<br>ASLGGGRAVFRRTHKRLAGYVYLRALRTGSRALSLQRGSLLD<br>TILQLGGEGSAEMWKDTLNHLSTLEPAIGDVPQLETVRELLR<br>KRLGAFVNLAPGEHSAAFFASDLTRQHLREVVRLFVATQPS<br>EHLVPYVRQGTGSINLLVFALLTIIADLKGSQSVIFAMEEPEIA<br>LPPHTQRRVTRYVLQQMGQSIVTSHSAPVIEQFEPESIVML<br>HRDGTKLAGAPIDLTKIKRKTYLTNRRQFAEAILARGVLVVE<br>GSTEAVVFPAISSVLERVRAGYTHLDFAGVSMFACSGDGDV<br>DRFGPIFTALGKKSYGACDKPNTAPSADVMANRAAFDHFW<br>ESAESGIEHVLVRGIPVEILRKFLDAVSARGDYPAAHPYNPA<br>MSDDDVPALAFKVLKARKGEAFAYAAVLIEQCETEADLPPE<br>LITILITIDDELRAEPEEPIEPESASESTEGE

						hypothetical protein	MVDWIRLTGGEHPDVAAQLSRAGLTAADAELRISFGRIDPT<br>RDTIIMRVIRGHLRVNDAVRQVQEFRRSQATGS
						hypothetical protein	MDDKAARLLSCLAEYELTLPEDVARQLISDRLDGIAKRMRIG<br>RQAARSYVTNDVIQSLADEVVGSRSGTDDEASNVVSLSDRR<br>RSRRSNRR
						putative ATPase	MSTRKLPSHISQLILTDYRVKPESVVYAAFNQSQLDGSQPYV<br>ARRIIRTSATLDVLALVPEHLQVSRRVSSRGTEVLLFSGDAET<br>VFLEYATEQRLEVYVSASDHNHATAIANQITSRIPEAAPPKD<br>TVGVTVWHSGARGASPSLKSIRVPSWADIRRNYPGAVAEPL<br>NDLFTTVRPHGYGKLILWHGEPGTGKTTALRALSREWKDW<br>CSIDYVADPERLFFDTGYLLEVIAKTSSDELQDSNTARWRLLI<br>AEDSDEFLRVSARREAGAALGRLLNLSDGILGQGSNTLILLTT<br>NERLDRLHPAVVRPGRCLAQVEFTRFPAAEANRWLPENHA<br>RVSEPKTLAELIEHRDATKQIATGIAPVTNIGAYL
GI6	12666	59.7	Contig 34	30105	42770	NADH oxidase (putative)	MTVRHIVAVGGSDAGISAALRARELDPTTEVTVVVADAYPN<br>FSICGIPYYVSGEVTHWTNLAHRTAADLAATGMRVFTDTRA<br>TGINVEDHTLDVLDPTGAPEQLSYDALIVGTGAISVRPPIAGI<br>SGPDALGTVDGVHLLHSMGDTFAVMDSLQQRDPATAVIV<br>GAGYIGLEMAEALTTRGIRVTQIEALPEVLPTVDPELGTLVH<br>AELERNGVEVLTNTAVSAITRTDTGALSVIATHDGQTISRTV<br>DFVLVVVGVRPDTELAADAGAELGVKGAVVVDEAMRTNLP<br>DIFAAGDCVHTHHRLLGITWLPLGTTAHKQGRVAGENALG<br>GQAHFAGSLGTQVVKVFDVVAARTGLREHEALAANRGWT<br>PVSSQSSPDDHKAYYPGATPVHIRITGDLHSGRLLGAQLVG<br>HRSAEIAKRVDTYATALYHEMTVGGLSELDLSYTPPLGSPW<br>DAIQMAAQTWARHHQPATATAASQPGTRRNLAAPN

dNTP triphosphohydrolase, broad substrate specificity, subgroup 2	MHAIIPNDPDQAAKRSTRPYAKPKSGGDARSEGQRDRDRV LYSTAWRRLGGVTQVVTPFSELPLLHNRLTHSEKVAQVARSI AEAKILSANETTQKLVQKLGGFDLDVCEAAALAHDLGHPPF GHIGEKILDDVARNKEILDLDDGFEGNAQSLRILVIGKIRSNK YEGNNPTYATIAAVSKYPWKRAGKRADHDEIIDTDSEYRKH WKKFSAYDPQYELLTQAREFLPPCISEKEIQSLEASVMDVAD DITYAVHDLEDFYLAGILDVSWIREDLEKYIRGEQKTGDVFFE LASRLGVDYSGWFNDDEMIRAAEWAERELKSGFNRRQVN YSEVEAQARKRGSDFIDTFINAIEVRDEPFWTGGPYVGLDER NWHRVQILKEITRSYVVQRPDIALLQRGQQEVLEKFVKLLYE WSENDRARLPARLKREIEIAEEQLDGGSTIGYGKDQHAPRG KNKNRAILDYICTLTDLECLQIYYKLSGIQVHRPGMVSF
hypothetical protein	MPEQERRGALINELKIVRKTGLHRLRERIDELPELSKLATATM GAGSADDIERMLRHAFTSYAEGAQGTAIGILFGLEIGRRGAR PSVLREVAAKRLGYDSVETFRKRPEYNAIAYFADLLHRLAVD ANSQKPADVNKIDHIMSLITELTIPEYWELTRRVRQLFAAGS QMS
hypothetical protein	MAQMDRQSIVRAREDFNRVREHLHQLELSRLAENAEADM REECTIHAMRNAREVDDAARHFEGGREGLAMTIREMQAA YNTGEIAHIAMRGCHR
hypothetical protein	MAALPCFEDLWNGVTCLAAAPPPVDPLIERLRQISQQLVEE KRAMDRRAQQVIRAMQAVASAAGQDGSTVQDDTSVER WVVTE
hypothetical protein	MNSSGPGHFVDRLIGWCFSILAAAIALYCAVKLLADILPALV VVVGALTLISLVVGAGIVVFRTIRNRW
hypothetical protein	MREAEADAEVKAAIADWAPDELQDEDNPSKGSDVEVGHD

hypothetical protein	MTDQLDVPLTADSLFWQQFFFQQPFQEAKAVGLLRHWAA<br>QDHAPQLILEARSDVTGVEYLVGSQLRHAQQVRRSVEDLVE<br>GSIVTSYEPGDREQISTARRLQLTTTTRPLEPSDPVAAGRSIL<br>HALTAVQRGERMVIQIVLGPRYQPLLGPPAPNRADQPVLSK<br>VLHGVLPEQRPDSRQALAHKLGQHGFRAALRIGVDAATAD<br>RRRTLLLGLAAAIRTAEAPGVHLKLRPEKPREINAPRSTWSM<br>FTRSQHLTVTEVARLTGWPVSDRDEPFPGQPPRHPKPVRPS<br>GAVQTGERVVATANAPGAEGPVGYSVTDALRHTWVIGPN<br>GVGKSTLLLNLIVQDLEAGRPIVVVEPKDLVRDILARVPEHRK<br>EDIVLVDPFDVAPVGINPLDSRYRDGRQPEVVADALFGTFK<br>AIHGDSLGPRSADILRNSLDVLARRDDASLVMLPLLLTNPGF<br>RRSLTQHTMRDDPYAAGPFWQWFDSLSPEAIATTVAPLSN<br>KLRPLLTKQLRAVLAQRSPKFNIRQVLTENKVLLVPLQKGVIG<br>PESAQLLGALVVAELWQAIRERAGTSEGTRTPLMVYIDEVQ<br>DYLRLPTDLGDALATARGLGAGFHLAHQYEKQLPPAMLDA<br>FRNNARSRICFQLRPGDAKEMVAGQSVLSVEDFGMLPAYH<br>VYASLVRDNAVQPWASGITSAPPPKTSDPDEIRRLSRERYG<br>QPLDDIETGFSELLSGAAKAEKSDEMGGPKRRRRQA
hypothetical protein	MRREDNHDHAGPIGDPIRLSNKPPLTSINDKSGLANDGAHT<br>GTTPPKQTKKRRTQSHDIEALRERLSERDLAILRSVAEHQFLT<br>VRQIEAFYFADHPSATGGRLARRALARMRNLRLLGATNRRV<br>GGVRSGSAGMVHYIDVVGDQLLDGRSGRGSRRFFNWSQR<br>FVHHRLAIADTHLALIEADRQAQLELVECLVEPASWRRFVGL<br>GGARLALKADLYIEIATTPGSDFVNPWFVELDLGTESIATVLK<br>KCRDYEAYRRTGTEQADGSGFPLVAWSMTHPDPTKAERRR<br>LALRDAIDNDRNLPAALFRIIAPDKLIPLLTRGGAV

DNA methylase N-4/N-6	MGDALDRLRELPDSSIDMALTSPPYFRLRNYGEDGQIGLEQ<br>HVDQWADRLAAISAEIHRVLVPTGTFWLNLGDTYATHPSQ<br>GADRKSLLMAPERLALRLQRAGWTVRNKVVWAKTNPMPT<br>SIKDRLNCTYEFVYVFAKQPSYFFDLDAVRQPHRSSLRKPYV<br>AERRDAELWRGPNSDTATGLDAIKAAGRVGHPLGKNPGD<br>VWLLPSSSFRGAHHATFPVRLASRAIQAGCPEARCTRCRLP<br>WRRRVIRAIGGTAIRAALVPTCECRTTSEPGLVLDPFIGSGTT<br>AIAAEELARDWLGIELNPDFAAMAERRILDARGRRQPEEKA<br>A
hypothetical protein	MSTPDESAKGVKTLGIRLQPDVHAQLSFIAQLREGTITDEIQI<br>AITGHIARSKEDPELRGRADAARAEIEREAAARQKAIASLFTT<br>EPPGSSGDSDDTAGQSRPARRGQKGDSPPEN
hypothetical protein	MSPKAKSLQAIGHVLGIPASELFAIAPWVTPQELPTIRPYLRT<br>KYRELPPAAVQEIETYFNDVARKHGISFDPNDGPLDGEDE

GI7	12727	59.9	Contig 35	75351	88077	Superfamily II DNA/RNA helicases, SNF2 family	MLLEELKPGLRIAGLIPGQVVTVIFAQSHGADTVELTFKTAS<br>GGLDQQLIFRKDENKLSVAQTGGRPFSAPASEFKLVAEAQRI<br>TLAGLYDPMLAVATSDVRPLPHQISAVYGELLPRTPLRFLLA<br>DDPGAGKTIMAGLYIKELILRDDIKRCLVVAPGGLVEQWQD<br>ELFFKFGLRFDLLTNQAIDAYINDNVFETHPLLIARMDQLSR<br>NQELQIQIQDTEWDLVVIDEAHRMAAHYFGGKLEKTKRFQ<br>LGELLGRLTRHLLLMTATPHSGKVEDYQLFLTLLDRDRFEGK<br>NSQNPETTGIMRRMIKEDLLTFDGKRLFPERKAETVPYELTQ<br>LEENLYEQVTDYVREGMNRADRVGGKRKNTVGFALTVLQR<br>RLASSPEAIYKSLVRRTQRLHRKKQEILNGTYRETEPTVDLEG<br>LDADDYNAEEVEQLEEELLDAATASQTVEELDAELAELADLT<br>ALAKQVRDSGTDRKWSELRTILEDHALITDNDGAPRKFIIFTE<br>HRDTLDYLTTRIRTLIGKPDAVKAIHGGVRRGERRLITEEFTK<br>NPETQILIATDAAGEGLNLQAAHLMVNYDLPWNPNRIEQR<br>FGRIHRIGQEEVCRLWNIVASNTREGDVYTRLLIKIEEQRKAY<br>GGKVFDVLGDAFHETPLRELLLKAIRYGDDPQVREKMHEVV<br>DATVSEGLPELLKERALAADHLAEADLAELRAAMDEARARR<br>LQPHYIELAFKAAFTRLGGRIAKRERGRYEIPHVPPQIRASKY<br>QPIATKYDRVTFDLEHVRPESQTRADLLAPGHPLHDAVMEE<br>TTKLLGGALNYGTVLVSSTLEEPHLLVGVIEEVCDATGASISR<br>RFGYAFVDSYGTVSHAGPAPYLDCVAAPDTPAVTAARQLP<br>WLAEAEDRAMSWIITNQLPGYLAEVQPRRAAELAKTRALVI<br>KRLEGERDRLLFDSAIAAEKEHAGDKPKESSESLNRKAVDLD<br>VRLRKRLELLDQQELMSTKPPRIITAALVLPISMLEHEIPASAP<br>VHAKETKDVERRGVDLVLAYEKALGRQPAEQTFTNKGFDIL<br>STTPNGDTYRIEVKARLEGATDFYVTHNEVLLGKNAVPRYRL<br>ALVKVDPRGPEHDQVRYLDHPFATTELGDFEATGIRGDWN

Adenine-specific DNA methylase containing a Zn-ribbon	MEGTLSDSAPKRKLIEVALPLEAINRESAREKSIRHGHPSTLH<br>LWWARRPLAAARAVLFAQLVDDPSARPDEFPTEELQRKER<br>NRLHKLIERLVVWESIRNEELFAEAHAEILKSTDGNPPPILDP<br>FAGGGTIPLEAQRLGMEAYASDLNPVAVLINKALIEIPPKFR<br>GQPPVFPGLADSEIRSWKGAEGLAADVRAYGRWARDEAE<br>KRIGHLYPKATLPDGSNATVIAWIWARTVTCPNPACGIDMP<br>LVRSWWLGKKKGKESYIVPTIVDDRTAPGGRRVRFEIGNDP<br>STGPMSSNDGTVARTTATCVACESPVPLPYVRAEGREGRLR<br>TELMAIVAEGQRRRVYLAPTALQSDAARIGRPASVPVGSLP<br>PHGLSPDSHQTIRIYGFYEWADLFTNRQLVALTTFSDLVAVA<br>HDRVLADGGPSDYADAVATYLGFAVSRLANKSSSTCSWDSS<br>TKMEAVRGVFARQALPMTWDFAEANPWGGSGGDYLEDV<br>EWVARAVERVPATRPGFAQQADARRVVMPANTLISTDPP<br>YYDNIGYSDLSDFFYVWLRRSLRGIYPDLLSTMLVPKADELV<br>ANPHRHDGREGARQFFEDGFKSVFASARATATADFPITVYY<br>AFKQSESDDDGQASTGWETLLEGMIQSGWEITSTWPMRS<br>ELSNRMLASGTNALASSIVLSLRPRRADAPTTDKRSFITALEA<br>ELPDALRKLQQGRIAPVDLPQATIGPGMAVFSRYAAVLEPD<br>GKHMSVRSALARINEILDRVLNEQEADFDPTSRFAIAWYRA<br>HGYNFGKYGDAELLANARGVVVQALDRAGVLISRAGRVNL<br>LKPNDLGPNYDVVADLHTSNWEALHHLVRVLDAEGIERAG<br>EFLRAALGRTDGIIDADLIKELAHLLFRVAETNGWTKDALSF<br>NGLVTSWPEIVDTAHTKTPRTSQGSFNFEEDAD

FIG00863870: hypothetical protein	MALSNRDRINKMFEIVAPPLDDYISLVIGQGDPAVGAAWP<br>KLVRAKDGTSSAKTYNAHDPQVQFRMLTESNITAGFKKGW<br>YPFNKTLGKAGESFAIELREVRNNWAHNGMFTDDDAYRAL<br>DTGERLLRLIGAANEADEVRSMRLNLRRVTADKEDKKVLRA<br>AVDNPEAAGLKAWRDVLPPHDDVATGNFAASEFAADLYK<br>VAFGGEQDSGYANPVEFFQRTYLTEGLSDLIGRAVRRLSGD<br>DNAPPVINLQTNFGGGKTHSMLALWHVAAGLPIGEFPQAT<br>QDLLSANKYSGGNVNRVAIVGNHFSPAGIERDGTHINTTW<br>GELAWQLGGPEAYAYVAKADADRTPPGDALAKLLAAYAPT<br>VILIDEWVAYARSLVGRDDLAGGTFDDQFTFAQLLTEAAKA<br>TSGVLVVISIPASESGDAADRVVAGNAEEVGGAHGLEALKR<br>LQNVVRRVADQWRPASSTEAYQIVKQRLFTQPDAVALASI<br>NATARAYVDMYRKYTDDFPRESRDTAYEDRIKRTYPIHPELF<br>DRLYEEWSSLERFQRTRGVLRLMSTVIHALWVGEDASPLIM<br>PGSVPLATGSVNSELTQYLQDSWKAIIDADVDGPNSEPARI<br>DKDKPLFGQRSLTKRLARTVFFGAAPTIGAAHKGLETQRVFL<br>GTAIPGDVPGNFHAALTQLADRATYFYTGSGKYWYDLQAN<br>ITRTARDQAERLHKEDVWAEIVRRLQSQARSRGDFTGVHV<br>CPETNGDIPDTDEARLVILHPKVAHKKGTDSPAKEFAHMAT<br>EHRGSANRTNRNTLVYLAADEARLEELDTAARDYLGWTHV<br>LNNAADLDLTENQKNQACQRQTTAEQTVTARLLQTFTWA<br>LVPAQPDPGAPFIIRETKVEGQSESLAERVSRRLGNDGDLSIR<br>QAAATIRLAINKVPQIWKDGHVSLGSLWALYCQYPYMPRL<br>RDRTVLNEGIVDMPMHWDVEAFALATAFDKSAHRYINLWI<br>SGDKNAAPAPTDAVLLVQPEVALKQREEEPPHGGGDKPPY<br>GGEPGSAKPPSIDVAFPPAKKRFYGVKTLSSDKIALDFKNIAD<br>EIISNLREPSTSLVVKIEIEATDASGFDESKIRTVSENARTLKFD
hypothetical protein	MVTPGACDTVIMPIGPAATALRADTTTSFEGKRHFPAPVW<br>AV

						FIG00828583: hypothetical protein	MSSHRTTFNHVGLCVSDRERSRRFYEGVLGFQFWWELDPPDGPTAQLVQLPEPLGVHATYLVRDGFVLELMDYSKRQVHAGSERVMDQIGLTHISFSVSDLPGALKKVEEFGGAVVKETVTEAMAMIRDPDGQLLELLSGGWLAALPLRS
GI8	37271	62.6	Contig 37	19212	56482	hypothetical protein	MRFTYFAAAVGIAIALLGASPAHADYSGFTRCVGGIKQLPLNEPDPQNFQRVGVIEQDLKSGVSPAAEAQKVAGMGFDQQTAATIVQCIIEENP
						hypothetical protein	MHGKQIAIAGAAIVLAGCGGSGKTATTVTSAVEITKTVTVTAPPPPFAPRTVIETDGTYRVGIDIVAGTYRSGGPSPEGGSDCYWARLSSLNSTHIIDSDIGTGPQVVMIAPTDKAFLTRSCQTWHKTD
						hypothetical protein	MMSGHYLKTTTDPGGGRAFTENWYFTPCGDGCADMSSPASGVSGRAMLVDGQWTLDSTEDIVCKGGVTEGNAANAHYTWDPNTLAGTVQVVQNRGVCDHPPQSYSLSFRLTKAP
						Integrase	MTIRRREAKSGTRWDVEWLLPDGTKRSKTFKTLRAAKLFDGEVRSAREDGEAVDPRGGKTELRLVYKSWLASRPDLSPKVRRGYEDNWRLRIEPKFGNWPIGRILREDVQEWINGMTADGLGPRTVRWTHSVLRMTLDHAVTDKKLRGKNPAANVRFPAMGETSHVYLTAVEVAKLAELCDTASGESSRAAKQGDVVLILAYTGLRFGELTGLNVEDVDLPARRIRVRRSITQLSGRLLEGPPKSRAGRRSVPIPERLIATLERRIAGRTASEPAIVSPKGARLGLENWKRAVGWRTRIGELGHPTMRVHDLRHTYASLARSAGADMKLLQVTMGHASIVVTAHTYADLYDSDLDRVADALDGLGD
						hypothetical protein	MEQALLQLLVRADPVPLSLLHFVITGTWPPEAMQPRFDVSFPKRSGSGAVPI

hypothetical protein	MPKKGRGRRPVWCSARCRRQASAERIAARNAGAAARVVE<br>VPRAHRPDPNARLPLPSMHTLQRLFLSSDYQCQTLLEDLAR<br>RYTSGAMGEQLRAAVQRFVAAIALEQALADDPVYRRARED<br>VERLRERLCREVERAERRDRELARLRRDAEELWRLRARVAEL<br>ESALAGVAHPLHQAGQHDQVPVSRQQRRAAQRAARKTC
Death on curing protein, Doc toxin	MRRGELWFAATPGGDRPVLILTRDPVADRIGAFVVAALTRT<br>RTGLVSELALTAPDDRLPSDCVVNFDKFIRCRAPRSDAGSLA<br>CLRRGYTRCVEHFERPPAAETSIRTRALISLVCLQER
Long-chain-fatty-acid--CoA ligase (EC 6.2.1.3)	MSISLLLEMAASTNPHRGAVVSGDARLTVDELSVLADGGA<br>GVITSCAARHVAYVGTGGALLPLLLFASARAGVPFTPLNYRL<br>SGESLRELIARLPEPLLIVDVAFAGQVAGVGALTMHSYDFITA<br>ARTAEPVSNFIDPDDVAVMLFTSGTTSRPKAVQITHNNLTS<br>YVTGTVEFDSAAAEDATLICVPPYHIAGIGAALSNLYAGRKM<br>VYLRNFDERKWLELIGAEGVTTATVVPTMLARIIAVLEDKPV<br>ELPTLRNLAYGGSKVALPLVRKALQLLPHVGFVNAYGLTETS<br>STVAVLGPEDHRAALCSPDPAVARRLGSVGKVVPGIEAQIR<br>GEDGSILGPGQRGELFVRGAQISGNYADIGSVLDADGWFA<br>TNDIAEIDEEGYLFIGGRSDDTIIRGGENIAPAEIEDVLIEHPH<br>VRDVAVVGTEDPQWGQTILAVVVPAEGTDPDPEELRAHVR<br>KHLRGSRTPDRVVFRNELPTNATGKVLRRQLIVEFSSVTDEF<br>A
hypothetical protein	MRINRDPAIDERLVPQLARLRGRDFFYAVDTIIAGLQARLAA<br>KSAS

Butyryl-CoA dehydrogenase (EC 1.3.99.2)	MVNAQSAEVADIIKAVRSFVRDHVVALEPKIEADDDIPPQL<br>RQACAEMGLFGTAIPEEYGGLGADVETEVLLAFELGWTTPA<br>LRAMFGTNNGIAGEVLISAGSDEQRSAWLPRLASGAAIASF<br>ALTESEAGSDPSTMKTSARRDGGDWIIDGAKRFITNAPYAD<br>IFVVFARTNPEVPAAQGISAFLVPKGTRGLQIGPRDAKMGQ<br>AGEVTAPVYMDGVRVPGEAMVGGEGQGYAAAMRSLGPG<br>RIRIAALCVGMAERLLEESLAYATTHHQSGRPIAEFQLIQAM<br>LADSQTDIYAGRALALEAARAFDAGTDRRTGPSVAKYFCSE<br>MVGRVADRAVQLHGGTGYMKGVTVERLYRDARVFRLYEG<br>TSQIQQLIIAGQSVRQYRNRAGIPS
Transcriptional regulator, GntR family	MPKRYGTKEKDQAVEYIIDRLLTGKLRTGDRVDRNEIARDL<br>GLSRVPVTEAIGQLEHDGIVTSRYHRGTFVERFDPATVLEHH<br>EVYGLLSGMASARAATYPTPRLIDKLNELIRVLRTATDSEIFE<br>QTTWEYRRTINHEYAGPRLQAAIRTSQSFIPKAFWVNFEDN<br>QRFMLPSYEREHAAILRHDSTAARAACEDRSMTTARVVVTE<br>LVRRGVFESTDAVAPVDGLLSP
hypothetical protein	MGHEEFRLLARDFIARNVTSYYDWITDGIFPAQSVPPTHQI<br>GVMGFRDS
putative cytochrome P450 hydroxylase	MNEWYYFTHRDDVLNALRTPEIYSSKKAFDMLGSPLPLVPIS<br>YDPPEHTRFRKVLQPFFSPHALGKMMPSLQQQVIDIIDNVA<br>AKGECDVVTDIAIPYPSQVFLTLYGLPLADRDKLVNWKDTVI<br>ALADLPGVPGEDKLGPALELFAYLTNAITERKANPGPDILSQ<br>VLTGEEPLDEAEAIGLSFLFVLAGLDTVTATIGFALEKLACDP<br>ALRGQLREDPKQTNVFVEEIVRLESAAPILPRITTEEVTVGGF<br>RLPPGSPIRLCIGAINRDGSDDISTDRMVLDGKVHRHWGFG<br>GGPHRCLGSHLARIELTLVVNEWLSRIPDFEVKPGFTPQIEW<br>PANTAALISLPVQWKS

Putative GntR-family transcriptional regulator	MTQFGVSRPTLREAFRILESEQLIEVLRGARGGARARRPDPG<br>AAGRYTGALLQSRGTTLDDVYQARAALEESAIGLASGRGLS<br>RQIRELEELSTRGAGLIEDPAAYAEFDVVFHHSIMALAGNTT<br>LSVLADQLYSIIEAHNRAHLADHPAGYDVPACRTHQRGHNK<br>LVRLLIEGDLEGAQRHWRRHLDGVQKYMIEDSRVAVVDVL<br>SAAL
L-carnitine dehydratase/bile acid-inducible protein F	MVDDLTGFEEAVALKKPMTGVRVIEVAEYTFVPSAGAVLA<br>DWGADVVKIENPVTGDAQRGLVSVLSRSASRQGVPFAPM<br>MEAPNRGKRSIGLSLALSESRPAFEELIRRSDVFLTSYLPRTRA<br>KLRIDVTDVRRVNPDIIYVAGSGFGHHGPDRDRGGYDATAF<br>WARGGSAQSVTQIDDDSPAPMPAGAYGDNIGGLTIAGAV<br>AAALFGRQNTGETSSIDVSLLSVGAWAMQANINLAMLYGC<br>PLPVIGGRAPAPGNPLTGTYRCKDGRFIQLSMLQPTRYWPE<br>FCSLFGLASAATDPRFASMEALAQHNDEAIRLVAEAIGSRPF<br>EECNRLLDQSSGPWAPLQDAWEVANDEALIANGRIADIIDS<br>QGHPQRLVANPAQFDGAPTRLNRAPLFAEHTDEVLRDLGV<br>DETELLQLKYAGAIT
Long-chain-fatty-acid--CoA ligase (EC 6.2.1.3)	MEPAPAPPRQFATWSQRILHHLLSDSDRDAIVSMNHERIES<br>WTRAELVARTAGASDYLDVHGVQAGDCVPALLTSQPHSVA<br>LLIAGALTRRPLAPLAPRVTHREVFASLKNISGPVLVTEPQYR<br>DFASELAKATGKRIAVVDEIGAGNVALGADARRPEDIACML<br>HTSGTTGLPKPVPVPEGPFERRAEILGQLCGFNGDARYVTA<br>ALFHHVAGLGNLMPALANGASIILIPPLSVDLWRNLASAAPT<br>HTTLVPSVIEMLLEEGVPAPPSLRVLAYGGSSIHPDTVRRMQ<br>KVMPAVDLINLFGQTEGSPVTVLNADDHRRAAAGQEGLLS<br>SVGRPAPGVELRLNEPDANGIGEIWARSDHSFVVDEQGW<br>QRTGDLGRVVDDFVYLVGRRGDKIIRGGENVFPLEVEQILET<br>HPDVVSAGVVGKPDRRLGETIVAFVVPVDVEEPPDSENLRV<br>HCRAQLAGFKVPVEWIFVDRLPRNPNGKLLRGELSRELARR<br>TSCGAKGHRVIWQ

Alpha-methylacyl-CoA racemase (EC 5.1.99.4)	MGPLRGTRIIELAGIGPAPFGCMLLADLGAEVVRVERAMSA<br>AALASIDDGGRSQADPRRYIPHRGRRSISLDLKSAEGRDALL<br>RLVETADVFVEGFRPGVTERLGVGPDDCLARNSRLVYARM<br>TGWGQSGPLASTAGHDINYIALAGVLDNFRRTGERPMPPL<br>NLVGDMGGGGMMLAFGITAALVHAARTGEGQVIDAAMV<br>DGAALQMAVVLGGRAQGRWPGEPGSNYSDTGAPQYEVY<br>ECADGKFVAVGALEGPFWSEFLRILDQPAGAVPSRDDPAL<br>WPQGKEALAKVFRTKTRDEWAAMFDGSDACVTPVLSIDEA<br>PAHPHAKARSAFITVGGIEQPAPAPRFSATPADVPSAPPDR<br>GEHTEEVLRELGLTDEEIVAATGSQRV
MaoC family protein	MTETGSVVVGYSPPPRTDKPLKVTDFVRYQGASGDMNPIH<br>HDPEYAARAGYDQVFAVGMLAAGRLATYVTDWLGPENVR<br>KFGVRFKEQAWPGDELTYTATVVDSRTDENERVVDLDLVC<br>TRHTGGVHLTGWATVALKTPTTDETDDGGPPSSD
hypothetical protein	MAEPFDIPIELGKSREFAKATGSKHPAHYSPGGVTPPTFLMT<br>AILWQGPDNNAWPKDRDMRRVLHASQEFAFPKGPPRIG<br>MQLTGQQRIEGSHTKEGKRGGTLTFTTLLTEFRDEKGDLVA<br>EVRNTFVETSKPTGGDH
Alcohol dehydrogenase (EC 1.1.1.1)	MMKATALVLETPNHLIERHIEIPAPAAGEAILRVLACGLCGS<br>DHELFTGAMPGSLPLIPGHEVVGVVERATDEFLSARGLEQG<br>EVVALEVFQRCEQCDACRRGAYPLCRTYGLARSYGNTSIEW<br>GSGLWGGFATHLLLGCDALVHRVPPGLDAAYATLFNPLGA<br>GVRWAHVLPQVQQGDVVAVLGPGLRGLSSVAVVSIAGAA<br>FTLLTGAGSRDRERMELGRTLGATETVDVTTTDARALLKERT<br>GGLADVVVDVTAAAPAAFLQAIDLARPGGTVVVAGTRGLH<br>ALKDFNPDRFVLKELTLLGARGVDGTAYARALELLATDDRFE<br>AIPRVTCALDAGSVADLLGDMAHGEAPPLHAVIVP

Putative uncharacterized protein BCG_1584c	MSEDEQRERHGKSLRFMFTKVPFNKVVGLELIDWSEPDVA<br>VVRLPFSEAIDNSGGTAHGGAIATLADVAGSAAAWNGHDY<br>EKGTRGATVSVTINFTGAARGEAVIATARCVRRARELNFTEV<br>SIVSESGRPVASGTVIYRIAT
3-ketoacyl-CoA thiolase (EC 2.3.1.16) @ Acetyl-CoA acetyltransferase (EC 2.3.1.9)	MPEAVIVEAVRTAIGKRNGALSGVHPTTLGAVVLGELVART<br>GVDPADVDDVQWGCVTQLGDQSSNVGRFTVLAAGWPET<br>VPAVTINRACGSSQQALDHAAYAVMSGQQEIVVAGGCES<br>MSRVPLGAGRATGMPYGPAAYDRFPGADFNQITGAQAIA<br>EKWGLSRRRLDEYSSRSHELSAEAADHGKFDEQIVAVETSD<br>GHFRVDEGLRRGTTPETLAKLSSVSGPDGVIHAGNSSQISDG<br>AAAMLVTTPEKAKSLNLRPLARYVRGVAVGADPRLVLTGPI<br>PATQQVLAKSGLTIGDVGAFEVNEAFAPVPLAWLAETGAD<br>PARLNPLGGAIAVGHPLGASGAILMTRLVHHMRDKGIRYGL<br>QTMCEGGGTANATIVELFGD
Enoyl-CoA hydratase (EC 4.2.1.17)	MPELLSDAEAGVLTLTLNRPQSRNAVSYSLLDNLIEAFEAAD<br>SDPSIRAIIVTGSGDSFSYGTDLAAGGGGLDVNAPGFKPLRG<br>TKRDVGGVLALRLFSCTKPVIAAVNGTAVGVGVTMILPMD<br>VRIAADSARFGLPFTRRGIVPESCATWFLPRIVGIATAIEWCV<br>TGRVFDAQEAAARGLVSELLPAEQVLPRAREIAAQIAEQASP<br>LSVALTRQMLWRQLGSPHPMSANRLESQAFLSLGGSADTK<br>EGIAAFKEKRKPHFTTAVPDDLPAFYPWFADEEFADKS

3-ketoacyl-(acyl-carrier-protein) reductase(EC:1.-)	MRLDGKVALVTGASRGIGLAIANCYAVNGAKVMLASRKAD<br>GLAAAAATLDGQVAVKVANTRNLDDIEGLVDAVLDRWGR<br>LDVLVNNAATCPYVGPVVDVEPGQRDRNFEVNLRGSLFLIQ<br>KAWRAWMVSNGGSIINISSVGAFRTHRTMGFYDIGKAALA<br>QLTRHLAGELGPRVRINAIVPGLVETRMAESIIKTRGVDIAAR<br>IPLQRNGIPEDIAGAALFPAGAAWSWMTLPRRHAKGAIEP<br>DRLAVDVGVLEQFARHAAELLRSSQPSRVRDVGCEGRTHFV<br>TESSEHRGIEDPWRDCVHPDALRRQIARQDECHSAHTGFR<br>GRIGRLTNLTLERSGRRRHHHGAPVAIGRRWIARDSFSREP<br>DDVVAADEIHSDDFFETVQRVCVSVTVEYTQPIAAPSRAMY<br>DRPQRSCINRGVERLTQTLGISDIRRDKGCADVLGGSRAVR<br>RREVGDNHAGATPRQRRRTRESQPRRSSGHQGSRVIQIHL<br>GLLYTSRQWIHINVKHLLARVDGTKSDRIVAGRRGVVLADS<br>NLWLNPTAGGRRGWIPRHELTGEAPEHAHA
putative acyl-CoA dehydrogenase(EC:1.3.99.-)	MGGWLSANAESLDEFRHHRAEAAEERWARDLRFIRVLYAA<br>GWNRHGWPAEYGGVGGPAVLRNVLYEELENAGYRVPDC<br>VIQLELQGEAFMKFAPEIAAARIPAALRGDEMWTQGFSEPE<br>AGSDLASLRCKAVQDGDEWVITGQKTWSSYALSARWMGV<br>LTRTGTPESRHRGITMFLVDLESPGVEVRPLRLANGVDECA<br>DVFFDNVRVSADNRIGEVGGGWDVAMYLLQFERANYGW<br>LRQAHLARRLRELVSNIDAPDRHTANVLGNCWLANLALRLR<br>CGDTVRRLFAGQRIGPEASIDKVLIAQAEQPLNDAFRDLFPA<br>QFLFDPGEESEVWRSEWYYSRAASIYGGAGEIQRGIIADRLL<br>GLPKEV
hypothetical protein	MVQTKSTELFEEDGRNSPVFVKHIFKANVTGHRSRVAAVSS

acyl-CoA dehydrogenase domain protein	MRGVDGDSVSSILLLAGEEAVLVPTSGLQCSPAKGFDIVDG<br>WTRWRGSVSADDSDVLSTDGARHVRAVVRAALASELNGIA<br>SKVNELAVDHVTSRHQFGQALGSFQSVRHRLAETHVAIHAA<br>MPVIELAWQATACGDAGSAELATAAKALAGRAFEIAAKNA<br>HQVCGGMGLSWEHPLHRWVRRGTVLNSLIGSPDELAADL<br>GGMLARGAALPVPNALCDDENQSTPA
Long-chain-fatty-acid--CoA ligase (EC 6.2.1.3)	MRRRESVHPCMTRQPGVEVDPRHDGVTPLGENVDSPSHH<br>YVTSLRHFAVKRPQDLAIVTPARSVTFAELSERANSLAGYLHS<br>RGVAAGTPVALMSANSAELIEAFYAALVLGAPPANVNPRYH<br>AHEVRYVLENCAAGAVLFDPTSATVVAEAVSAMEEPPFPLE<br>FGSSRWRAAISSTTDYVRPQSVDDRLLIYTGGTTGLPRAVE<br>WRVVDHFRMIWQMVKPTEPPPTPEALIDSGRRAPTASPCS<br>PILHGVGLSLTLNTLNGGGTVVVSDRLTFDAADTFDLVRRYD<br>VAVLGIVGDAFARPMLDELETGRWNGKLPSLRAISSAGAS<br>WTSSIRDRMANLLPGVKMVSNFGSTEALVARDISSDHSIDP<br>GDGLVVIGENDRPAQPGEVGVVATAGYLPLGYLGDPQKTA<br>ETFRTINGRRYAVTGDEARVEADGRITLLGRGSAVINTGGEK<br>VWPDEVEAVLRSVPGVLDAVVVGRPDERWGQRVAAVLRI<br>AGTAEISDQRLSQGCRERLAPYKCPRQYVRVDEIPRTPVGKP<br>DYRAIDSLLEES
hypothetical protein	MPKRYGTKEKEQESAAREERSTMMGRVAVTELVNRGGSE<br>TYRGRHARYRDLDTELVGGY

Butyryl-CoA dehydrogenase (EC 1.3.99.2)	MRRTLFDQEHEEFRVLVRDFIAKRVVPHFDEWVDEGIVPR<br>QLYRELGAIGAMGFAIPEQYGGGGVDDYRYNAVLQEEAAR<br>ANVTLGTMRTHLDVVLPYLLKYANDEQRERWFPGLVSGDL<br>FLAIAMTEPGTGSDLAGVKTTAKRDGSDFIVNGAKTFITGAA<br>HADLIIVLARTGEPIDGNRRSGLSLLVVEDGVAGFTKGRRLP<br>KLGLRVQDTVELSFEDVRVPAINLLGEVDQAFSYLGHNLAQ<br>ERLAIAVGSVAQSRAAMEMTIDYVKNRNLFGTTVSAMQNT<br>KFEIAAVATEIEAAQAMCDNGVRDLVDGELSGADAAKIKLF<br>CTEMQARTVDRCLQLHGGYGYILEYPISRLYADARVSRIYGG<br>TSEVLKSIISKSLGL
Enoyl-CoA hydratase (EC 4.2.1.17)	MSDVLIVRDEAGVRQITMNRPERRNAMDIDTRRRFADLIV<br>ESHDNPDVRSIVITGAGGHFCAGADVTRMARSVDEEAAYE<br>RVQTAQEIAQVMAEGAKPQIAAVTGSAVGLGMGIALACDY<br>IVAGPDARFAGGFVRVGLCADNGVLFTLPQRVGPARARTM<br>MLLSETVPADEALRIGLIDKLTASDDQVLPAAIEVAATLAAG<br>PPLALATISRAFTALPMTFMDALVREAELQAPLLCSDDHQE<br>AATAFREKRRPVFTGR
hypothetical protein	MARWSDEFVFGQVWTDSDIPWEQQILVAITALATLGHHT<br>QLRNYLHGALQGGVSEPALRHALSMLTVYAGFPVAIQALN<br>VLSEVVAREARTTTTHPENAPGSASGR
Acyl-CoA thioesterase II (EC 3.1.2.-)	MSDRLQERLQLDDVGNDRFSSSAPGAPVNIYGGELAAHAL<br>VAANMTVTDDRLPHSLHCTYLAAGDPAHGLEHQVTRIRDG<br>RSFSVRRVDVFNGGRLSVSATVSYQTKSAGVEHRRTAPRVP<br>CPDDLPTFHGASNAAWIPWAEENPELEMRVAPHDPGDSL<br>GRRTFWLKIRHDPAIGSADDLLEAAYAAYASDFTMIASIRLP<br>HEEPDVKTHVMTTLTHSLYFHKPFAASQWHLVDHWSPAA<br>AGGRGLSIAHAYNASGDLTMTAVQESLVRSASKKAG

	probable oxidoreductase/Short-chain dehydrogenase	MKIIITGASSGIGKATATALAARGHQVVIACRTLKKGEEAAAQMSGDVRVQHLDLADLASVRRFADAVDTVDVLVNNAGVLGLPYTRTVDGFEAHMGTNHLGHFALTCLLADRTTDRVVSLSSSAYKYATPHLDDLNWERRKYSKSQAYGESKLATMLFINELARRGVRAYAADPGIVFSDITRDGGAILRWSGKYMHPIIGQTTGNGARSTLLAIETTAPSGTCFAPGGLMHQWGKPKVVIPHRKARNPRTALRLWELSAELTGCDAPYEPQASE
	putative cytochrome P450 hydroxylase	MTETAVGKPDVRYDPYDFEIDNDPYPTFRRLRDEAPLYYNDEYDFYALSRFHDVKQASVEWETYPSGRGSVLELIKSGAAIPPGFILWEDPPRHDVHRGLLARVFTMRRIAQIEDKVRTVCTATLDKFVDTGRFDFVADLGSEIPMRTIGMMLGIPDSDQVSHRDRTNEGIKLADGQAPVDGSALLTLTDTQRIGDYINFRRRHPADDLMTDLLTATCTDSGGHERQLQDSTIQTYVGLLAAAGNETTVRLFSWAGKVLAEHPDQRRELANDPSLIPAAIEELLRFEAPSPVQARYVARDVEHHGRTMPQGSVVLLLTASANRDERAFEDPDRFDIHRKTKNHVSFGFGIHHCLGAHLARLEARVGLEEVFKRFPEWDVDWDNAVQSHTSTVRGWEKLPVVASPSLRDR
	hypothetical protein	MAFVTGAARGQGRAHRFPTSSPEEVANLAAGEARYITGQQIRIDGGALIEFPNGPTRI
	hypothetical protein	MKLPVEPELESLRAAIAARAKSTVRPLAAQVDREQKFSWELWAAVRELGLSRIHFPEDHGGDGGTFRAYTIASTEPAEYCAVASLYPGTSIQVAMALLQHGNPEHSRRFFAFWAAKRCGLGLYRAVDRCGFSAQRVAFIPGYMSARQAAAVDPEAPTRPTSRWLRSSSSLSWNFPAQTRRKIPARQLRPPMLWPSRR
