# Supplementary material for: Comparative Genomic Analysis Reveals a Possible Novel Non-Tuberculous Mycobacterium Species with High Pathogenic Potential
Source: PLoS One. 2016 Apr 1;11(4):e0150413. doi: 10.1371/journal.pone.0150413 (PMC4818103; doi:10.1371/journal.pone.0150413)
Supplement: S3 Table — (PDF) [file pone.0150413.s007.pdf]

Supplementary Table S3: A list of sporulation-related genes in UM_CSW genome.

Putative Ortholog in *Mycobacterium*	Annotation	NTM2 RAST_ORF_ID	NTM2 Contig No.	Start	End	NTM2 ID	NTM2 RAST Annotation	E-value
MMAR_0022	hypothetical protein	fig|6666666.34050.peg.468	contig_105	609	887	ID=M751500;	FIG025441: hypothetical protein	5.00E-54
MMAR_0523	urea amidolyase-related protein	fig|6666666.34050.peg.2543	contig_24	28274	27390	ID=M753601;	Allophanate hydrolase 2 subunit 2 (EC 3.5.1.54)	1.00E-157
MMAR_0524	hypothetical protein	fig|6666666.34050.peg.2544	contig_24	28963	28289	ID=M753602;	Allophanate hydrolase 2 subunit 1 (EC 3.5.1.54)	6.00E-108
MMAR_0541	hypothetical protein	fig|6666666.34050.peg.5719	contig_82	3038	4891	ID=M756788;	"AAA family ATPase, ESX-1 secretion system component Rv3868"	0
	hypothetical protein	fig|6666666.34050.peg.5824	contig_86	15287	13500	ID=M756894;	Type VII secretion AAA-ATPase EccA	7.00E-71
	hypothetical protein	fig|6666666.34050.peg.1653	contig_15	48723	49397	ID=M752705;	PROBABLE CONSERVED MEMBRANE PROTEIN	8.00E-30
MMAR_0606	alpha-D-glucose-1-phosphate thymidylyl-transferase, RmlA	fig|6666666.34050.peg.4337	contig_52	4643	5722	ID=M755404;	D-glycero-D-manno-heptose 1-phosphate guanosyltransferase	5.00E-20
MMAR_1081	dTDP-4-dehydrorhamnose 3,5-epimerase, RmlC	fig|6666666.34050.peg.1947	contig_18	3709	3089	ID=M753004;	"dTDP-4-dehydrorhamnose 3,5-epimerase (EC 5.1.3.13)"	3.00E-109
	dTDP-4-dehydrorhamnose 3,5-epimerase, RmlC	fig|6666666.34050.peg.483	contig_106	15242	14685	ID=M751515;	"dTDP-4-dehydrorhamnose 3,5-epimerase (EC 5.1.3.13)"	1.00E-20

MMAR_1082	TDP-glucose-4,6-dehydratase, RmlB	fig|6666666.34050.peg.1948	contig_18	4712	3711	ID=M753005;	"dTDP-glucose 4,6-dehydratase (EC 4.2.1.46)"	0
	TDP-glucose-4,6-dehydratase, RmlB	fig|6666666.34050.peg.4630	contig_58	3736	4731	ID=M755696;	"dTDP-glucose 4,6-dehydratase (EC 4.2.1.46)"	2.00E-26
	TDP-glucose-4,6-dehydratase, RmlB	fig|6666666.34050.peg.1327	contig_135	13053	13997	ID=M752377;	UDP-glucose 4-epimerase (EC 5.1.3.2)	9.00E-20
	TDP-glucose-4,6-dehydratase, RmlB	fig|6666666.34050.peg.5650	contig_80	1252	2301	ID=M756719;	"dTDP-glucose 4,6-dehydratase (EC 4.2.1.46)"	9.00E-17
	TDP-glucose-4,6-dehydratase, RmlB	fig|6666666.34050.peg.2450	contig_23	36072	35128	ID=M753508;	"dTDP-glucose 4,6-dehydratase (EC 4.2.1.46)"	5.00E-14
	TDP-glucose-4,6-dehydratase, RmlB	fig|6666666.34050.peg.4634	contig_58	11743	10670	ID=M755700;	NAD-dependent epimerase/dehydratase family protein	1.00E-13
	TDP-glucose-4,6-dehydratase, RmlB	fig|6666666.34050.peg.2448	contig_23	33113	34129	ID=M753506;	"GDP-mannose 4,6-dehydratase (EC 4.2.1.47)"	8.00E-13
MMAR_1248	alternate RNA polymerase sigma factor SigF	fig|6666666.34050.peg.5299	contig_70	17916	17128	ID=M756367;	RNA polymerase sigma factor SigB	4.00E-122
	alternate RNA polymerase sigma factor SigF	fig|6666666.34050.peg.1680	contig_153	6810	7619	ID=M752732;	RNA polymerase sigma factor SigB	2.00E-65
	alternate RNA polymerase sigma factor SigF	fig|6666666.34050.peg.6050	contig_98	10936	9413	ID=M757121;	RNA polymerase sigma factor RpoD	8.00E-10
	alternate RNA polymerase sigma factor SigF	fig|6666666.34050.peg.6074	contig_99	16607	15648	ID=M757145;	RNA polymerase sigma factor SigB	3.00E-09

MMAR_1275	dTDP-6-deoxy-L-lyxo-4-hexulose reductase RmlD	fig|6666666.34050.peg.4335	contig_52	2868	3755	ID=M755402;	dTDP-4-dehydrorhamnose reductase (EC 1.1.1.133)	3.00E-98
MMAR_1618	Multicopper oxidase, type 1, CueO	fig|6666666.34050.peg.3300	contig_35	44286	45827	ID=M754361;	Multicopper oxidase	1.00E-16
MMAR_1967	cell division transmembrane protein FtsK	fig|6666666.34050.peg.4404	contig_53	58648	60966	ID=M755471;	Cell division protein FtsK	0
	cell division transmembrane protein FtsK	fig|6666666.34050.peg.1966	contig_18	20480	23926	ID=M753023;	FtsK/SpoIIIE family protein	1.00E-16
	cell division transmembrane protein FtsK	fig|6666666.34050.peg.5968	contig_95	8836	13005	ID=M757039;	"Type VII secretion protein EccCa, FtsK/SpoIIIE family"	6.00E-09
MMAR_2098	Probable transcriptional regulatory protein	fig|6666666.34050.peg.420	contig_103	15265	14510	ID=M751452;	FIG000859: hypothetical protein	6.00E-153
MMAR_2129	site-specific integrase							
MMAR_2320	pyridoxal phosphate-dependent enzyme, WecE							
MMAR_3184	hypothetical protein	fig|6666666.34050.peg.1526	contig_14	131297	130935	ID=M752577;	Probable transmembrane protein	1.00E-12
MMAR_3194	FtsW-like protein FtsW	fig|6666666.34050.peg.4328	contig_51	32721	34406	ID=M755395;	Cell division protein FtsW	0
	FtsW-like protein FtsW	fig|6666666.34050.peg.471	contig_105	2985	4394	ID=M751503;	Cell division protein FtsW	3.00E-18

MMAR_3765	GTPase ObgE	fig|6666666.34050.peg.5229	contig_7	10571	12013	ID=M756297;	COG0536: GTP-binding protein Obg	0
	GTPase ObgE	fig|6666666.34050.peg.2556	contig_25	6205	5132	ID=M753614;	GTP-binding and nucleic acid-binding protein YchF	6.00E-12
MMAR_4208	proline and glycine rich transmembrane protein	fig|6666666.34050.peg.4011	contig_45	10221	9676	ID=M755077;	Basic proline-rich protein	2.00E-12
MMAR_4226	glycosyl transferase	fig|6666666.34050.peg.1292	contig_130	5848	4685	ID=M752340;	"Predicted glycogen synthase, ADP-glucose transglucosylase (EC 2.4.1.21), Actinobacterial type"	0
	glycosyl transferase	fig|6666666.34050.peg.3140	contig_33	39833	38604	ID=M754197;	Glycosyltransferase	4.00E-17
	glycosyl transferase	fig|6666666.34050.peg.5649	contig_80	27	1247	ID=M756718;	"Glycosyl transferase, group 1"	1.00E-16
	glycosyl transferase	fig|6666666.34050.peg.2880	contig_29	74155	75486	ID=M753937;	Glycosyltransferase MshA involved in mycothiol biosynthesis (EC 2.4.1.-)	1.00E-13
MMAR_4455	two-component transcriptional regulator TrcR	fig|6666666.34050.peg.4417	contig_54	6529	7302	ID=M755483;	DNA-binding response regulator TrcR	2.00E-148
	two-component transcriptional regulator TrcR	fig|6666666.34050.peg.2280	contig_21	26121	25417	ID=M753338;	putative two-component system response regulator	8.00E-86
	two-component transcriptional regulator TrcR	fig|6666666.34050.peg.5892	contig_91	20230	20922	ID=M756962;	Two-component response regulator	2.00E-59

	two-component transcriptional regulator TrcR	fig|6666666.34050.peg.5587	contig_79	44006	44677	ID=M756656;	DNA-binding response regulator	3.00E-57
MMAR_4473	peptidyl-tRNA hydrolase Pth	fig|6666666.34050.peg.503	contig_106	33574	32999	ID=M751535;	Peptidyl-tRNA hydrolase (EC 3.1.1.29)	3.00E-102
MMAR_4853	hypothetical protein	fig|6666666.34050.peg.1867	contig_17	105788	105991	ID=M752924;	zinc transporter Slc39a7	6.00E-04
MMAR_5181	anti-anti-sigma regulatory factor	fig|6666666.34050.peg.4150	contig_48	44227	43913	ID=M755217;	Anti-sigma F factor antagonist (spoIIAA-2); Anti-sigma B factor antagonist RsbV	5.00E-54
	anti-anti-sigma regulatory factor	fig|6666666.34050.peg.4218	contig_5	22087	22383	ID=M755285;	Anti-sigma F factor antagonist (spoIIAA-2); Anti-sigma B factor antagonist RsbV	5.00E-19
	anti-anti-sigma regulatory factor	fig|6666666.34050.peg.3491	contig_38	27862	28299	ID=M754557;	Anti-sigma F factor antagonist (spoIIAA-2); Anti-sigma B factor antagonist RsbV	2.00E-07
MMAR_5186	sensor-component of a two-component regulator	fig|6666666.34050.peg.4155	contig_48	46759	48975	ID=M755222;	"Serine phosphatase RsbU, regulator of sigma subunit"	0
	sensor-component of a two-component regulator	fig|6666666.34050.peg.5167	contig_69	11937	9406	ID=M756235;	"Serine phosphatase RsbU, regulator of sigma subunit"	7.00E-88
	sensor-component of a two-component regulator	fig|6666666.34050.peg.394	contig_102	7792	4001	ID=M751426;	FIG00829714: hypothetical protein	3.00E-47

	sensor-component of a two-component regulator	fig|6666666.34050.peg.840	contig_12	24997	26952	ID=M751875;	"Serine phosphatase RsbU, regulator of sigma subunit"	4.00E-29
	sensor-component of a two-component regulator	fig|6666666.34050.peg.4263	contig_5	67793	67443	ID=M755330;	"Serine phosphatase RsbU, regulator of sigma subunit"	2.00E-08
MMAR_5481	chromosome partitioning protein ParB	fig|6666666.34050.peg.1635	contig_15	28423	29406	ID=M752687;	Chromosome (plasmid) partitioning protein ParB / Stage 0 sporulation protein J	0
MMAR_5482	chromosome partitioning protein ParA	fig|6666666.34050.peg.1634	contig_15	27419	28399	ID=M752686;	Chromosome (plasmid) partitioning protein ParA / Sporulation initiation inhibitor protein Soj	0
	chromosome partitioning protein ParA	fig|6666666.34050.peg.4843	contig_61	8540	7620	ID=M755910;	Chromosome (plasmid) partitioning protein ParA / Sporulation initiation inhibitor protein Soj	1.00E-46
	chromosome partitioning protein ParA	fig|6666666.34050.peg.2996	contig_31	15415	14615	ID=M754053;	Chromosome (plasmid) partitioning protein ParA / Sporulation initiation inhibitor protein Soj	2.00E-44
MMAR_5484	hypothetical protein	fig|6666666.34050.peg.1632	contig_15	26031	26618	ID=M752684;	RNA-binding protein Jag	4.00E-64

MMAR_5485	putative inner membrane protein translocase component YidC	fig|6666666.34050.peg.1631	contig_15	24825	25949	ID=M752683;	"Inner membrane protein translocase component YidC, long form"	8.00E-163
MT0125	hypothetical protein	fig|6666666.34050.peg.3381	contig_36	42617	41862	ID=M754442;	Possible membrane protein	2.00E-101
MT0125	hypothetical protein	fig|6666666.34050.peg.738	contig_115	2314	1505	ID=M751773;	COG1376: Uncharacterized protein conserved in bacteria	1.00E-60
MT2818	acetyltransferase, GNAT family	fig|6666666.34050.peg.4405	contig_53	61521	61033	ID=M755472;	"Acetyltransferase, GNAT family"	1.00E-103
