# Supplementary material for: Comparative Genomic Analysis Reveals a Possible Novel Non-Tuberculous Mycobacterium Species with High Pathogenic Potential
Source: PLoS One. 2016 Apr 1;11(4):e0150413. doi: 10.1371/journal.pone.0150413 (PMC4818103; doi:10.1371/journal.pone.0150413)
Supplement: S4 Table — (PDF) [file pone.0150413.s008.pdf]

Supplementary Table S4: Prediction of human pathogen using PathogenFinder.

Organism	Sequences	Avg seq length	Probability of being human pathogen	Genome Coverage (%)	Pathogenic Families Matched	Non-Pathogenic Families Matched	The organism predicted as human pathogenic
Mycobacterium gilvum PYR-GCK	5368	323	0.222	1.02	1	54	No
Mycobacterium sp. JLS	5834	320	0.223	14.45	0	843	No
Mycobacterium sp. KMS	5527	321	0.231	17.01	0	940	No
Mycobacterium sp. MCS	5480	322	0.231	16.92	0	927	No
Mycobacterium rhodesiae NBB3	6277	316	0.245	0.81	2	49	No
Mycobacterium vaccae ATCC 25954	5914	325	0.417	0.24	4	10	No
Mycobacterium thermoresistibile ATCC19527	1695	327	0.449	0.53	3	6	No
Mycobacterium smegmatis JS623	6398	309	0.523	0.27	8	9	Yes
Mycobacterium kansasii ATCC12478	5633	340	0.833	0.67	33	5	Yes
Mycobacterium sp. UM CSW	6042	319	0.841	0.56	30	4	Yes
Mycobacterium avium 104	5192	322	0.883	13.52	702	0	Yes
Mycobacterium ulcerans Agy99	5477	306	0.895	12.93	708	0	Yes
Mycobacterium marinum M	5577	358	0.9	19.15	1068	0	Yes
Mycobacterium intracellulare ATCC13950	5036	327	0.906	0.85	42	1	Yes
Mycobacterium indicus pranii MTCC9506	5182	328	0.907	0.85	43	1	Yes
Mycobacterium tuberculosis H37Rv	4086	326	0.922	27.29	1115	0	Yes
Mycobacterium africanum GM041182	4041	327	0.924	22.17	896	0	Yes
Mycobacterium canettii CIPT140010059	4070	332	0.926	10.44	425	0	Yes
Mycobacterium cosmeticum strain DSM 44829	4232	197	0.931	3.07	130	0	Yes
Mycobacterium leprae TN	3995	187	0.931	3.25	130	0	Yes
