# Supplementary material for: Comparative Genomic Analysis Reveals a Possible Novel Non-Tuberculous Mycobacterium Species with High Pathogenic Potential
Source: PLoS One. 2016 Apr 1;11(4):e0150413. doi: 10.1371/journal.pone.0150413 (PMC4818103; doi:10.1371/journal.pone.0150413)
Supplement: S5 Table — (PDF) [file pone.0150413.s009.pdf]

Supplementary Table S5: A list of predicted virulence genes in the UM_CSW genome.

query_id	sub_id	virulence_gene	Sequence Identity (%)	Query length (aa)	Subject length (aa)
fig|6666666.28482.peg.9	R013489	(gi:118462718) nuoG (MAV 4039) - NADH-quinone oxidoreductase, chain g [Mycobacterium avium str. 104]	68.41	763	766
fig|6666666.28482.peg.9	R019200	(gi:183981492) nuoG (MMAR 1477) - NADH dehydrogenase subunit G [Mycobacterium marinum str. M]	69.21	764	766
fig|6666666.28482.peg.9	R013490	(gi:118617952) nuoG (MUL 2465) - NADH dehydrogenase I (chain G) NuoG (NADH-ubiquinone oxidoreductase chain G) [Mycobacterium ulcerans str. Agy99]	68.96	764	766
fig|6666666.28482.peg.9	R013485	(gi:15842727) nuoG (MT3239) - NADH dehydrogenase gamma subunit [Mycobacterium tuberculosis str. CDC1551]	69.46	764	766
fig|6666666.28482.peg.9	R019204	(gi:253800185) nuoG (TBMG 03194) - NADH dehydrogenase subunit G [Mycobacterium tuberculosis str. KZN 1435]	69.46	764	766
fig|6666666.28482.peg.9	R013482	(gi:148824345) nuoG (TBFG 13172) - NADH dehydrogenase I chain G nuoG (NADH-ubiquinone oxidoreductase chain G) [Mycobacterium tuberculosis str. F11]	69.46	764	766
fig|6666666.28482.peg.9	R019205	(gi:224991526) nuoG (JTY 3169) - NADH dehydrogenase subunit G [Mycobacterium bovis str. BCG str. Tokyo 172]	69.46	764	766
fig|6666666.28482.peg.9	R013487	(gi:121639034) nuoG (BCG 3174) - putative NADH dehydrogenase I (chain G) nuoG [Mycobacterium bovis str. BCG Pasteur 1173P2]	69.46	764	766
fig|6666666.28482.peg.9	R013486	(gi:31794327) nuoG (Mb3175) - NADH dehydrogenase gamma subunit [Mycobacterium bovis str. AF2122/97]	69.46	764	766
fig|6666666.28482.peg.9	R019202	(gi:339633158) nuoG (MAF 31590) - putative NADH dehydrogenase I (chain G) NUOG (NADH-ubiquinone oxidoreductase chain G) [Mycobacterium africanum str. GM041182]	69.46	764	766
fig|6666666.28482.peg.9	R019203	(gi:340628127) nuoG (MCAN 31651) - putative NADH dehydrogenase I subunit G NuoG [Mycobacterium canettii str. CIPT 140010059]	69.46	764	766
fig|6666666.28482.peg.9	R013483	(gi:15610287) nuoG (Rv3151) - NADH dehydrogenase gamma subunit [Mycobacterium tuberculosis str. H37Rv]	69.33	764	766
fig|6666666.28482.peg.9	R013484	(gi:148663006) nuoG (MRA 3184) - NADH dehydrogenase subunit gamma [Mycobacterium tuberculosis str. H37Ra]	69.33	764	766

fig|6666666.28482.peg.9	R013492	(gi:145225066) nuoG (Mflv 4487) - NADH-quinone oxidoreductase, chain G [Mycobacterium gilvum str. PYR-GCK]	68.03	763	766
fig|6666666.28482.peg.9	R013491	(gi:118467665) nuoG (MSMEG 2057) - NADH-quinone oxidoreductase, G subunit [Mycobacterium smegmatis str. MC2 155]	68.33	764	766
fig|6666666.28482.peg.9	R019206	(gi:333991505) nuoG (JDM601 2865) - NADH dehydrogenase I (chain G) NuoG (NADH- ubiquinone oxidoreductase chain G) [Mycobacterium sp. str. JDM601]	67.51	762	766
fig|6666666.28482.peg.9	R013496	(gi:108798544) nuoG (Mmcs 1574) - NADH-quinone oxidoreductase, chain G [Mycobacterium sp. str. MCS]	69.35	764	766
fig|6666666.28482.peg.9	R013495	(gi:119867644) nuoG (Mkms 1598) - NADH-quinone oxidoreductase, chain G [Mycobacterium sp. str. KMS]	69.35	764	766
fig|6666666.28482.peg.9	R013494	(gi:126434142) nuoG (Mjls 1544) - NADH-quinone oxidoreductase, chain G [Mycobacterium sp. str. JLS]	69.48	764	766
fig|6666666.28482.peg.9	R013493	(gi:120402877) nuoG (Mvan 1879) - NADH-quinone oxidoreductase, chain G [Mycobacterium vanbaalenii str. PYR-1]	70.28	763	766
fig|6666666.28482.peg.9	R013488	(gi:41409305) nuoG (MAP3207) - NADH dehydrogenase gamma subunit [Mycobacterium avium str. K-10 (subsp. paratuberculosis)]	68.39	734	766
fig|6666666.28482.peg.9	R019201	(gi:169629226) nuoG (MAB 2140) - NADH dehydrogenase subunit G [Mycobacterium abscessus str. ATCC 19977]	64.33	762	766
fig|6666666.28482.peg.35	R019302	(gi:253800166) devR/dosR (TBMG 03176) - two component system transcriptional regulator devR [Mycobacterium tuberculosis str. KZN 1435]	72.5	200	212
fig|6666666.28482.peg.35	R006291	(gi:15610269) devR/dosR (Rv3133c) - TWO COMPONENT TRANSCRIPTIONAL REGULATORY PROTEIN DEVR (PROBABLY LUXR/UHPA-FAMILY) [Mycobacterium tuberculosis str. H37Rv]	72.5	200	212
fig|6666666.28482.peg.35	R006292	(gi:148662987) devR/dosR (MRA 3165) - two component transcriptional regulatory protein DevR [Mycobacterium tuberculosis str. H37Ra]	72.5	200	212
fig|6666666.28482.peg.35	R013509	(gi:148824327) devR/dosR (TBFG 13154) - two component system transcriptional regulatory protein devR (luxR/uhpA-family) [Mycobacterium tuberculosis str. F11]	72.5	200	212
fig|6666666.28482.peg.35	R006293	(gi:15842706) devR/dosR (MT3219) - DNA-binding response regulator, LuxR family [Mycobacterium tuberculosis str. CDC1551]	72.5	200	212
fig|6666666.28482.peg.35	R019301	(gi:340628109) devR/dosR (MCAN 31471) - two component transcriptional regulatory protein [Mycobacterium canettii str. CIPT 140010059]	72.5	200	212

fig|6666666.28482.peg.35	R019303	(gi:224991508) devR/dosR (JTY 3151) - two component transcriptional regulatory protein [Mycobacterium bovis str. BCG str. Tokyo 172]	72.5	200	212
fig|6666666.28482.peg.35	R006295	(gi:121639016) devR/dosR (BCG 3156c) - Two component transcriptional regulatory protein devR (probably luxR/uhpA-family) [Mycobacterium bovis str. BCG Pasteur 1173P2]	72.5	200	212
fig|6666666.28482.peg.35	R006294	(gi:31794309) devR/dosR (Mb3157c) - TWO COMPONENT TRANSCRIPTIONAL REGULATORY PROTEIN DEVR (PROBABLY LUXR/UHPA-FAMILY) [Mycobacterium bovis str. AF2122/97]	72.5	200	212
fig|6666666.28482.peg.35	R019300	(gi:339633140) devR/dosR (MAF 31410) - two component transcriptional regulatory protein DEVR (probably LUXR/UHPA-family) [Mycobacterium africanum str. GM041182]	72.5	200	212
fig|6666666.28482.peg.35	R019304	(gi:333991618) devR/dosR (JDM601 2978) - two-component transcriptional regulatory protein DevR [Mycobacterium sp. str. JDM601]	72.73	198	212
fig|6666666.28482.peg.35	R006302	(gi:120402438) devR/dosR (Mvan 1427) - two component transcriptional regulator, LuxR family [Mycobacterium vanbaalenii str. PYR-1]	71.5	200	212
fig|6666666.28482.peg.35	R006298	(gi:118617914) devR/dosR (MUL 2423) - two component transcriptional regulatory protein DevR [Mycobacterium ulcerans str. Agy99]	72.22	198	212
fig|6666666.28482.peg.35	R019298	(gi:183981531) devR/dosR (MMAR 1516) - two-component transcriptional regulatory protein DevR [Mycobacterium marinum str. M]	72.22	198	212
fig|6666666.28482.peg.35	R006299	(gi:118472166) devR/dosR (MSMEG 5244) - two component transcriptional regulatory protein devr [Mycobacterium smegmatis str. MC2 155]	69.5	200	212
fig|6666666.28482.peg.35	R006307	(gi:108801090) devR/dosR (Mmcs 4126) - two component transcriptional regulator, LuxR family [Mycobacterium sp. str. MCS]	70	200	212
fig|6666666.28482.peg.35	R006305	(gi:119870231) devR/dosR (Mkms 4201) - two component transcriptional regulator, LuxR family [Mycobacterium sp. str. KMS]	70	200	212
fig|6666666.28482.peg.35	R006303	(gi:126436928) devR/dosR (Mjls 4357) - two component transcriptional regulator, LuxR family [Mycobacterium sp. str. JLS]	70	200	212
fig|6666666.28482.peg.35	R006300	(gi:145224429) devR/dosR (Mflv 3847) - two component transcriptional regulator, LuxR family [Mycobacterium gilvum str. PYR-GCK]	69	200	212
fig|6666666.28482.peg.35	R006296	(gi:41409369) devR/dosR (MAP3271c) - hypothetical protein [Mycobacterium avium str. K-10 (subsp. paratuberculosis)]	68.5	200	212
fig|6666666.28482.peg.35	R006297	(gi:118466851) devR/dosR (MAV 4109) - two component transcriptional regulatory protein devr [Mycobacterium avium str. 104]	68.5	200	212

fig|6666666.28482.peg.35	R006308	(gi:108798049) devR/dosR (Mmcs 1076) - two component transcriptional regulator, LuxR family [Mycobacterium sp. str. MCS]	67.17	198	212
fig|6666666.28482.peg.35	R006306	(gi:119867144) devR/dosR (Mkms 1092) - two component transcriptional regulator, LuxR family [Mycobacterium sp. str. KMS]	67.17	198	212
fig|6666666.28482.peg.35	R006304	(gi:126433709) devR/dosR (Mjls 1103) - two component transcriptional regulator, LuxR family [Mycobacterium sp. str. JLS]	67.17	198	212
fig|6666666.28482.peg.35	R006301	(gi:120403536) devR/dosR (Mvan 2548) - two component transcriptional regulator, LuxR family [Mycobacterium vanbaalenii str. PYR-1]	71.5	200	212
fig|6666666.28482.peg.35	R019299	(gi:169630970) devR/dosR (MAB 3891c) - LuxR family transcriptional regulator [Mycobacterium abscessus str. ATCC 19977]	61.11	198	212
fig|6666666.28482.peg.69	R019011	(gi:183983279) glnA1 (MMAR 3289) - glutamine synthetase GlnA1 [Mycobacterium marinum str. M]	87.24	478	478
fig|6666666.28482.peg.69	R005812	(gi:118617032) glnA1 (MUL 1340) - glutamine synthetase GlnA1 [Mycobacterium ulcerans str. Agy99]	86.4	478	478
fig|6666666.28482.peg.69	R005809	(gi:41408060) glnA1 (MAP1962) - GlnA1 [Mycobacterium avium str. K-10 (subsp. paratuberculosis)]	85.98	478	478
fig|6666666.28482.peg.69	R005810	(gi:118464473) glnA1 (MAV 2267) - glutamine synthetase, type I [Mycobacterium avium str. 104]	85.98	478	478
fig|6666666.28482.peg.69	R019015	(gi:253798714) glnA1 (TBMG 01760) - glutamine synthetase glnA1 [Mycobacterium tuberculosis str. KZN 1435]	85.56	478	478
fig|6666666.28482.peg.69	R005804	(gi:15609357) glnA1 (Rv2220) - GLUTAMINE SYNTHETASE GLNA1 (GLUTAMINE SYNTHASE) (GS-I) [Mycobacterium tuberculosis str. H37Rv]	85.56	478	478
fig|6666666.28482.peg.69	R005805	(gi:148662038) glnA1 (MRA 2237) - glutamine synthetase [Mycobacterium tuberculosis str. H37Ra]	85.56	478	478
fig|6666666.28482.peg.69	R013457	(gi:148823428) glnA1 (TBFG 12249) - glutamine synthetase glnA1 [Mycobacterium tuberculosis str. F11]	85.56	478	478
fig|6666666.28482.peg.69	R005806	(gi:15841712) glnA1 (MT2278) - glutamine synthetase [Mycobacterium tuberculosis str. CDC1551]	85.56	478	478
fig|6666666.28482.peg.69	R019014	(gi:340627226) glnA1 (MCAN 22431) - glutamine synthetase GLNA1 [Mycobacterium canettii str. CIPT 140010059]	85.56	478	478
fig|6666666.28482.peg.69	R019016	(gi:224990596) glnA1 (JTY 2231) - glutamine synthetase [Mycobacterium bovis str. BCG str. Tokyo 172]	85.56	478	478

fig|6666666.28482.peg.69	R005808	(gi:121638102) glnA1 (BCG 2237) - Glutamine synthetase glnA1 [Mycobacterium bovis str. BCG Pasteur 1173P2]	85.56	478	478
fig|6666666.28482.peg.69	R005807	(gi:31793400) glnA1 (Mb2244) - GLUTAMINE SYNTHETASE GLNA1 (GLUTAMINE SYNTHASE) (GS-I) [Mycobacterium bovis str. AF2122/97]	85.56	478	478
fig|6666666.28482.peg.69	R019013	(gi:339632248) glnA1 (MAF 22320) - Glutamine synthetase GLNA1 (Glutamine synthase) [Mycobacterium africanum str. GM041182]	85.56	478	478
fig|6666666.28482.peg.69	R005811	(gi:15827444) glnA1 (ML0925) - glutamine synthase class I . [Mycobacterium leprae str. TN]	85.56	478	478
fig|6666666.28482.peg.69	R019017	(gi:221229921) glnA1 (MLBr 00925) - glutamine synthase class I [Mycobacterium leprae str. Br4923]	85.56	478	478
fig|6666666.28482.peg.69	R005815	(gi:120404556) glnA1 (Mvan 3585) - glutamine synthetase, type I [Mycobacterium vanbaalenii str. PYR-1]	82.85	478	478
fig|6666666.28482.peg.69	R005813	(gi:118467956) glnA1 (MSMEG 4290) - glutamine synthetase, type I [Mycobacterium smegmatis str. MC2 155]	82.85	478	478
fig|6666666.28482.peg.69	R005818	(gi:108800286) glnA1 (Mmcs 3320) - glutamine synthetase, type I [Mycobacterium sp. str. MCS]	82.43	478	478
fig|6666666.28482.peg.69	R005817	(gi:119869414) glnA1 (Mkms 3382) - glutamine synthetase, type I [Mycobacterium sp. str. KMS]	82.43	478	478
fig|6666666.28482.peg.69	R005816	(gi:126435909) glnA1 (Mjls 3331) - glutamine synthetase, type I [Mycobacterium sp. str. JLS]	82.43	478	478
fig|6666666.28482.peg.69	R005814	(gi:145223515) glnA1 (Mflv 2928) - glutamine synthetase, type I [Mycobacterium gilvum str. PYR-GCK]	82.01	478	478
fig|6666666.28482.peg.69	R019018	(gi:333992818) glnA1 (JDM601 4178) - glutamine synthetase [Mycobacterium sp. str. JDM601]	81.53	471	478
fig|6666666.28482.peg.69	R019012	(gi:169629021) glnA1 (MAB 1933c) - glutamine synthetase, type I (GlnA1) [Mycobacterium abscessus str. ATCC 19977]	80.13	478	478
fig|6666666.28482.peg.69	R019019	(gi:333990322) glnA1 (JDM601 1682) - glutamine synthetase [Mycobacterium sp. str. JDM601]	78.87	478	478
fig|6666666.28482.peg.94	R018193	(gi:119867807) Mkms1768 - hypothetical protein [Mycobacterium sp. str. KMS]	70.71	198	209
fig|6666666.28482.peg.94	R018194	(gi:108798689) Mmcs1721 - hypothetical protein [Mycobacterium sp. str. MCS]	70.71	198	209

fig|6666666.28482.peg.94	R018192	(gi:126434290) Mjls1700 - hypothetical protein [Mycobacterium sp. str. JLS]	70.71	198	209
fig|6666666.28482.peg.94	R018189	(gi:118470319) MSMEG2128 - malonyl CoA decarboxylase [Mycobacterium smegmatis str. MC2 155]	68.16	201	209
fig|6666666.28482.peg.94	R018186	(gi:41409247) MAP3149c - hypothetical protein [Mycobacterium avium str. K-10 (subsp. paratuberculosis)]	63.16	209	209
fig|6666666.28482.peg.94	R019127	(gi:333990174) JDM6011533 - hypothetical protein [Mycobacterium sp. str. JDM601]	61.76	204	209
fig|6666666.28482.peg.94	R019121	(gi:183981603) MMAR1587 - hypothetical protein [Mycobacterium marinum str. M]	64.25	207	209
fig|6666666.28482.peg.94	R018188	(gi:118618753) MUL3447 - hypothetical protein [Mycobacterium ulcerans str. Agy99]	64.08	206	209
fig|6666666.28482.peg.94	R018190	(gi:145224991) Mflv4412 - hypothetical protein [Mycobacterium gilvum str. PYR-GCK]	62.12	198	209
fig|6666666.28482.peg.94	R018191	(gi:120402940) Mvan1943 - hypothetical protein [Mycobacterium vanbaalenii str. PYR-1]	63.13	198	209
fig|6666666.28482.peg.266	R005167	(gi:108797926) cmaA2 (Mmcs 0952) - Cyclopropane-fatty-acyl-phospholipid synthase [Mycobacterium sp. str. MCS]	80.21	283	287
fig|6666666.28482.peg.266	R018710	(gi:253797397) pcaA (TBMG 00472) - mycolic acid synthase PcaA [Mycobacterium tuberculosis str. KZN 1435]	74.56	287	287
fig|6666666.28482.peg.266	R005144	(gi:57116736) pcaA (Rv0470c) - MYCOLIC ACID SYNTHASE PCAA (CYCLOPROPANE SYNTHASE) [Mycobacterium tuberculosis str. H37Rv]	74.56	287	287
fig|6666666.28482.peg.266	R005145	(gi:148660236) pcaA (MRA 0476) - mycolic acid synthase PcaA [Mycobacterium tuberculosis str. H37Ra]	74.56	287	287
fig|6666666.28482.peg.266	R013397	(gi:148821668) pcaA (TBFG 10477) - mycolic acid synthase pcaA (cyclopropane synthase) [Mycobacterium tuberculosis str. F11]	74.56	287	287
fig|6666666.28482.peg.266	R005146	(gi:15839858) pcaA (MT0486) - mycolic acid synthase [Mycobacterium tuberculosis str. CDC1551]	74.56	287	287
fig|6666666.28482.peg.266	R018709	(gi:340625496) pcaA (MCAN 04691) - mycolic acid synthase PCAA [Mycobacterium canettii str. CIPT 140010059]	74.56	287	287
fig|6666666.28482.peg.266	R005148	(gi:121636385) pcaA (BCG 0510c) - Mycolic acid synthase pcaA [Mycobacterium bovis str. BCG Pasteur 1173P2]	74.56	287	287

fig|6666666.28482.peg.266	R005147	(gi:31791649) pcaA (Mb0479c) - MYCOLIC ACID SYNTHASE PCAA (CYCLOPROPANE SYNTHASE) [Mycobacterium bovis str. AF2122/97]	74.56	287	287
fig|6666666.28482.peg.266	R018708	(gi:339630539) pcaA (MAF 04730) - mycolic acid synthase PCAA (cyclopropane synthase) [Mycobacterium africanum str. GM041182]	74.56	287	287
fig|6666666.28482.peg.266	R018711	(gi:224988857) pcaA (JTY 0480) - mycolic acid synthase [Mycobacterium bovis str. BCG str. Tokyo 172]	74.56	287	287
fig|6666666.28482.peg.266	R005152	(gi:118619634) pcaA (MUL 4539) - mycolic acid synthase PcaA [Mycobacterium ulcerans str. Agy99]	72.47	287	287
fig|6666666.28482.peg.266	R018707	(gi:183980820) pcaA (MMAR 0796) - mycolic acid synthase PcaA [Mycobacterium marinum str. M]	72.47	287	287
fig|6666666.28482.peg.266	R005151	(gi:15828331) pcaA (ML2459) - Mycolic acid synthase [Mycobacterium leprae str. TN]	73.17	287	287
fig|6666666.28482.peg.266	R018712	(gi:221230808) pcaA (MLBr 02459) - Mycolic acid synthase [Mycobacterium leprae str. Br4923]	73.17	287	287
fig|6666666.28482.peg.266	R005149	(gi:41410062) pcaA (MAP3964c) - UmaA2 [Mycobacterium avium str. K-10 (subsp. paratuberculosis)]	73.52	287	287
fig|6666666.28482.peg.266	R005150	(gi:118465677) pcaA (MAV 4679) - cyclopropane-fatty-acyl-phospholipid synthase 1 [Mycobacterium avium str. 104]	74.56	283	287
fig|6666666.28482.peg.266	R005157	(gi:108799701) pcaA (Mmcs 2735) - Cyclopropane-fatty-acyl-phospholipid synthase [Mycobacterium sp. str. MCS]	72.73	286	287
fig|6666666.28482.peg.266	R005156	(gi:119868811) pcaA (Mkms 2779) - Cyclopropane-fatty-acyl-phospholipid synthase [Mycobacterium sp. str. KMS]	72.73	286	287
fig|6666666.28482.peg.266	R005155	(gi:126435345) pcaA (Mjls 2765) - Cyclopropane-fatty-acyl-phospholipid synthase [Mycobacterium sp. str. JLS]	72.73	286	287
fig|6666666.28482.peg.266	R005153	(gi:145223891) pcaA (Mflv 3304) - Cyclopropane-fatty-acyl-phospholipid synthase [Mycobacterium gilvum str. PYR-GCK]	71.68	286	287
fig|6666666.28482.peg.266	R005154	(gi:120404005) pcaA (Mvan 3025) - Cyclopropane-fatty-acyl-phospholipid synthase [Mycobacterium vanbaalenii str. PYR-1]	72.03	286	287
fig|6666666.28482.peg.266	R018720	(gi:333990669) cmaA2 (JDM601 2029) - methoxy mycolic acid synthase [Mycobacterium sp. str. JDM601]	62.98	284	287
fig|6666666.28482.peg.266	R018727	(gi:333989305) mma4 (JDM601 0665) - methoxy mycolic acid synthase [Mycobacterium sp. str. JDM601]	61.19	286	287

fig|6666666.28482.peg.266	R018713	(gi:333989092) pcaA (JDM601 0452) - mycolic acid synthase PcaA [Mycobacterium sp. str. JDM601]	62.09	276	287
fig|6666666.28482.peg.266	R005165	(gi:15828307) cmaA2 (ML2426) - cyclopropane mycolic acid synthase [Mycobacterium leprae str. TN]	60.34	285	287
fig|6666666.28482.peg.266	R018719	(gi:221230784) cmaA2 (MLBr 02426) - cyclopropane mycolic acid synthase [Mycobacterium leprae str. Br4923]	60.34	285	287
fig|6666666.28482.peg.266	R005175	(gi:15828017) mma4 (ML1903) - methyl mycolic acid synthase 4 [Mycobacterium leprae str. TN]	60.07	278	287
fig|6666666.28482.peg.266	R018726	(gi:221230494) mma4 (MLBr 01903) - methyl mycolic acid synthase 4 [Mycobacterium leprae str. Br4923]	60.07	278	287
fig|6666666.28482.peg.267	R005167	(gi:108797926) cmaA2 (Mmcs 0952) - Cyclopropane-fatty-acyl-phospholipid synthase [Mycobacterium sp. str. MCS]	66.9	284	293
fig|6666666.28482.peg.267	R005152	(gi:118619634) pcaA (MUL 4539) - mycolic acid synthase PcaA [Mycobacterium ulcerans str. Agy99]	65.71	280	293
fig|6666666.28482.peg.267	R018707	(gi:183980820) pcaA (MMAR 0796) - mycolic acid synthase PcaA [Mycobacterium marinum str. M]	65.71	280	293
fig|6666666.28482.peg.267	R005157	(gi:108799701) pcaA (Mmcs 2735) - Cyclopropane-fatty-acyl-phospholipid synthase [Mycobacterium sp. str. MCS]	61.77	293	293
fig|6666666.28482.peg.267	R005156	(gi:119868811) pcaA (Mkms 2779) - Cyclopropane-fatty-acyl-phospholipid synthase [Mycobacterium sp. str. KMS]	61.77	293	293
fig|6666666.28482.peg.267	R005155	(gi:126435345) pcaA (Mjls 2765) - Cyclopropane-fatty-acyl-phospholipid synthase [Mycobacterium sp. str. JLS]	61.77	293	293
fig|6666666.28482.peg.267	R018710	(gi:253797397) pcaA (TBMG 00472) - mycolic acid synthase PcaA [Mycobacterium tuberculosis str. KZN 1435]	64.29	280	293
fig|6666666.28482.peg.267	R005144	(gi:57116736) pcaA (Rv0470c) - MYCOLIC ACID SYNTHASE PCAA (CYCLOPROPANE SYNTHASE) [Mycobacterium tuberculosis str. H37Rv]	64.29	280	293
fig|6666666.28482.peg.267	R005145	(gi:148660236) pcaA (MRA 0476) - mycolic acid synthase PcaA [Mycobacterium tuberculosis str. H37Ra]	64.29	280	293
fig|6666666.28482.peg.267	R013397	(gi:148821668) pcaA (TBFG 10477) - mycolic acid synthase pcaA (cyclopropane synthase) [Mycobacterium tuberculosis str. F11]	64.29	280	293
fig|6666666.28482.peg.267	R005146	(gi:15839858) pcaA (MT0486) - mycolic acid synthase [Mycobacterium tuberculosis str. CDC1551]	64.29	280	293

fig|6666666.28482.peg.267	R018709	(gi:340625496) pcaA (MCAN 04691) - mycolic acid synthase PCAA [Mycobacterium canettii str. CIPT 140010059]	64.29	280	293
fig|6666666.28482.peg.267	R005148	(gi:121636385) pcaA (BCG 0510c) - Mycolic acid synthase pcaA [Mycobacterium bovis str. BCG Pasteur 1173P2]	64.29	280	293
fig|6666666.28482.peg.267	R005147	(gi:31791649) pcaA (Mb0479c) - MYCOLIC ACID SYNTHASE PCAA (CYCLOPROPANE SYNTHASE) [Mycobacterium bovis str. AF2122/97]	64.29	280	293
fig|6666666.28482.peg.267	R018708	(gi:339630539) pcaA (MAF 04730) - mycolic acid synthase PCAA (cyclopropane synthase) [Mycobacterium africanum str. GM041182]	64.29	280	293
fig|6666666.28482.peg.267	R018711	(gi:224988857) pcaA (JTY 0480) - mycolic acid synthase [Mycobacterium bovis str. BCG str. Tokyo 172]	64.29	280	293
fig|6666666.28482.peg.267	R005153	(gi:145223891) pcaA (Mflv 3304) - Cyclopropane-fatty-acyl-phospholipid synthase [Mycobacterium gilvum str. PYR-GCK]	60.75	293	293
fig|6666666.28482.peg.267	R005151	(gi:15828331) pcaA (ML2459) - Mycolic acid synthase [Mycobacterium leprae str. TN]	65.36	280	293
fig|6666666.28482.peg.267	R018712	(gi:221230808) pcaA (MLBr 02459) - Mycolic acid synthase [Mycobacterium leprae str. Br4923]	65.36	280	293
fig|6666666.28482.peg.267	R005150	(gi:118465677) pcaA (MAV 4679) - cyclopropane-fatty-acyl-phospholipid synthase 1 [Mycobacterium avium str. 104]	63.21	280	293
fig|6666666.28482.peg.267	R005149	(gi:41410062) pcaA (MAP3964c) - UmaA2 [Mycobacterium avium str. K-10 (subsp. paratuberculosis)]	62.86	280	293
fig|6666666.28482.peg.268	R018721	(gi:183980997) mma4 (MMAR 0977) - methoxy mycolic acid synthase 4, MmaA4 [Mycobacterium marinum str. M]	87.29	298	299
fig|6666666.28482.peg.268	R005168	(gi:15607782) mma4 (Rv0642c) - METHOXY MYCOLIC ACID SYNTHASE 4 MMAA4 (METHYL MYCOLIC ACID SYNTHASE 4) (MMA4) (HYDROXY MYCOLIC ACID SYNTHASE) [Mycobacterium tuberculosis str. H37Rv]	85.57	297	299
fig|6666666.28482.peg.268	R005169	(gi:148660416) mma4 (MRA 0653) - methoxy mycolic acid synthase 4 [Mycobacterium tuberculosis str. H37Ra]	85.57	297	299
fig|6666666.28482.peg.268	R018723	(gi:340625661) mma4 (MCAN 06411) - methoxy mycolic acid synthase 4 MMAA4 [Mycobacterium canettii str. CIPT 140010059]	85.57	297	299
fig|6666666.28482.peg.268	R013399	(gi:148821846) mma4 (TBFG 10655) - methoxy mycolic acid synthase 4 mmaA4 [Mycobacterium tuberculosis str. F11]	85.57	297	299
fig|6666666.28482.peg.268	R005170	(gi:15840045) mma4 (MT0670) - methoxy mycolic acid synthase 4 [Mycobacterium tuberculosis str. CDC1551]	85.57	297	299

fig|6666666.28482.peg.268	R018724	(gi:253797582) mma4 (TBMG 00653) - methoxy mycolic acid synthase 4 mmaA4 [Mycobacterium tuberculosis str. KZN 1435]	85.57	297	299
fig|6666666.28482.peg.268	R018725	(gi:224989035) mma4 (JTY 0661) - methoxy mycolic acid synthase 4 [Mycobacterium bovis str. BCG str. Tokyo 172]	85.57	297	299
fig|6666666.28482.peg.268	R005172	(gi:121636563) mma4 (BCG 0691c) - Methoxy mycolic acid synthase 4 mmaA4 [Mycobacterium bovis str. BCG Pasteur 1173P2]	85.57	297	299
fig|6666666.28482.peg.268	R005171	(gi:31791826) mma4 (Mb0661c) - METHOXY MYCOLIC ACID SYNTHASE 4 MMAA4 (METHYL MYCOLIC ACID SYNTHASE 4) (MMA4) (HYDROXY MYCOLIC ACID SYNTHASE) [Mycobacterium bovis str. AF2122/97]	85.57	297	299
fig|6666666.28482.peg.268	R018722	(gi:339630712) mma4 (MAF 06510) - methoxy mycolic acid synthase 4 MMAA4 (methyl mycolic acid synthase 4) [Mycobacterium africanum str. GM041182]	85.57	297	299
fig|6666666.28482.peg.268	R005176	(gi:118616517) mma4 (MUL 0729) - methoxy mycolic acid synthase 4, MmaA4 [Mycobacterium ulcerans str. Agy99]	86.29	298	299
fig|6666666.28482.peg.268	R005173	(gi:41410214) mma4 (MAP4116c) - MmaA4 [Mycobacterium avium str. K-10 (subsp. paratuberculosis)]	84.9	297	299
fig|6666666.28482.peg.268	R005174	(gi:118466420) mma4 (MAV 4517) - cyclopropane-fatty-acyl-phospholipid synthase 1 [Mycobacterium avium str. 104]	84.56	297	299
fig|6666666.28482.peg.268	R005175	(gi:15828017) mma4 (ML1903) - methyl mycolic acid synthase 4 [Mycobacterium leprae str. TN]	83.16	296	299
fig|6666666.28482.peg.268	R018726	(gi:221230494) mma4 (MLBr 01903) - methyl mycolic acid synthase 4 [Mycobacterium leprae str. Br4923]	83.16	296	299
fig|6666666.28482.peg.268	R018727	(gi:333989305) mma4 (JDM601 0665) - methoxy mycolic acid synthase [Mycobacterium sp. str. JDM601]	79.08	282	299
fig|6666666.28482.peg.268	R005178	(gi:120402251) mma4 (Mvan 1239) - Cyclopropane-fatty-acyl-phospholipid synthase [Mycobacterium vanbaalenii str. PYR-1]	70.81	297	299
fig|6666666.28482.peg.268	R005177	(gi:145225689) mma4 (Mflv 5113) - Cyclopropane-fatty-acyl-phospholipid synthase [Mycobacterium gilvum str. PYR-GCK]	69.9	298	299
fig|6666666.28482.peg.268	R005181	(gi:108797925) mma4 (Mmcs 0951) - Cyclopropane-fatty-acyl-phospholipid synthase [Mycobacterium sp. str. MCS]	69.97	293	299
fig|6666666.28482.peg.268	R005180	(gi:119867021) mma4 (Mkms 0969) - Cyclopropane-fatty-acyl-phospholipid synthase [Mycobacterium sp. str. KMS]	69.97	293	299

fig|6666666.28482.peg.268	R005179	(gi:126433581) mma4 (Mjls 0972) - Cyclopropane-fatty-acyl-phospholipid synthase [Mycobacterium sp. str. JLS]	69.97	293	299
fig|6666666.28482.peg.290	R006470	(gi:41410187) mce2F (MAP4089) - hypothetical protein [Mycobacterium avium str. K-10 (subsp. paratuberculosis)]	85.63	515	516
fig|6666666.28482.peg.290	R006471	(gi:118463921) mce2F (MAV 4546) - mce-family protein mce2f [Mycobacterium avium str. 104]	85.71	511	516
fig|6666666.28482.peg.290	R019385	(gi:253797529) mce2F (TBMG 00602) - MCE-family protein mce2F [Mycobacterium tuberculosis str. KZN 1435]	80.43	516	516
fig|6666666.28482.peg.290	R006465	(gi:15607734) mce2F (Rv0594) - MCE-FAMILY PROTEIN MCE2F [Mycobacterium tuberculosis str. H37Rv]	80.43	516	516
fig|6666666.28482.peg.290	R006466	(gi:148660364) mce2F (MRA 0602) - MCE-family protein Mce2F [Mycobacterium tuberculosis str. H37Ra]	80.43	516	516
fig|6666666.28482.peg.290	R013523	(gi:148821797) mce2F (TBFG 10606) - MCE-family protein mce2F [Mycobacterium tuberculosis str. F11]	80.43	516	516
fig|6666666.28482.peg.290	R006467	(gi:15839997) mce2F (MT0624) - virulence factor mce family protein [Mycobacterium tuberculosis str. CDC1551]	80.43	516	516
fig|6666666.28482.peg.290	R019386	(gi:224988984) mce2F (JTY 0609) - MCE-family protein [Mycobacterium bovis str. BCG str. Tokyo 172]	80.43	516	516
fig|6666666.28482.peg.290	R006469	(gi:121636512) mce2F (BCG 0640) - mce-family protein mce2F [Mycobacterium bovis str. BCG Pasteur 1173P2]	80.43	516	516
fig|6666666.28482.peg.290	R006468	(gi:31791776) mce2F (Mb0610) - MCE-FAMILY PROTEIN MCE2F [Mycobacterium bovis str. AF2122/97]	80.43	516	516
fig|6666666.28482.peg.290	R019383	(gi:339630662) mce2F (MAF 06010) - MCE-family protein MCE2F [Mycobacterium africanum str. GM041182]	80.43	516	516
fig|6666666.28482.peg.290	R019384	(gi:340625624) mce2F (MCAN 06001) - MCE-family protein MCE2F [Mycobacterium canettii str. CIPT 140010059]	80.23	516	516
fig|6666666.28482.peg.290	R006399	(gi:41409707) mce1F (MAP3609) - hypothetical protein [Mycobacterium avium str. K-10 (subsp. paratuberculosis)]	70.6	516	516
fig|6666666.28482.peg.290	R006401	(gi:15828398) mce1F (ML2594) - putative secreted protein [Mycobacterium leprae str. TN]	67.12	516	516
fig|6666666.28482.peg.290	R019360	(gi:221230875) mce1F (MLBr 02594) - putative secreted protein [Mycobacterium leprae str. Br4923]	67.12	516	516

fig|6666666.28482.peg.290	R019357	(gi:340625211) mce1F (MCAN 01801) - MCE-family protein MCE1F [Mycobacterium canettii str. CIPT 140010059]	70.29	515	516
fig|6666666.28482.peg.290	R019358	(gi:253797096) mce1F (TBMG 00175) - MCE-family protein mce1F [Mycobacterium tuberculosis str. KZN 1435]	70.1	515	516
fig|6666666.28482.peg.290	R013517	(gi:148821366) mce1F (TBFG 10175) - MCE-family protein mce1F [Mycobacterium tuberculosis str. F11]	70.1	515	516
fig|6666666.28482.peg.290	R006396	(gi:15839552) mce1F (MT0183) - virulence factor mce family protein [Mycobacterium tuberculosis str. CDC1551]	70.1	515	516
fig|6666666.28482.peg.290	R019359	(gi:224988559) mce1F (JTY 0180) - MCE-family protein [Mycobacterium bovis str. BCG str. Tokyo 172]	70.1	515	516
fig|6666666.28482.peg.290	R006398	(gi:121636086) mce1F (BCG 0211) - MCE-family protein mce1F [Mycobacterium bovis str. BCG Pasteur 1173P2]	70.1	515	516
fig|6666666.28482.peg.290	R006397	(gi:31791352) mce1F (Mb0180) - MCE-FAMILY PROTEIN MCE1F [Mycobacterium bovis str. AF2122/97]	70.1	515	516
fig|6666666.28482.peg.290	R019356	(gi:339630255) mce1F (MAF 01750) - MCE-family protein MCE1F [Mycobacterium africanum str. GM041182]	70.1	515	516
fig|6666666.28482.peg.290	R006394	(gi:15607315) mce1F (Rv0174) - MCE-FAMILY PROTEIN MCE1F [Mycobacterium tuberculosis str. H37Rv]	69.9	515	516
fig|6666666.28482.peg.290	R006395	(gi:148659938) mce1F (MRA 0182) - MCE-family protein Mce1F [Mycobacterium tuberculosis str. H37Ra]	69.9	515	516
fig|6666666.28482.peg.290	R019355	(gi:183980448) mce1F (MMAR 0417) - MCE-family protein Mce1F [Mycobacterium marinum str. M]	68.59	516	516
fig|6666666.28482.peg.290	R006402	(gi:118616800) mce1F (MUL 1067) - MCE-family protein Mce1F [Mycobacterium ulcerans str. Agy99]	68.4	516	516
fig|6666666.28482.peg.290	R006474	(gi:126432726) mce2F (Mjls 0113) - virulence factor Mce family protein [Mycobacterium sp. str. JLS]	67.12	515	516
fig|6666666.28482.peg.290	R006476	(gi:108797103) mce2F (Mmcs 0123) - Mammalian cell entry [Mycobacterium sp. str. MCS]	66.73	515	516
fig|6666666.28482.peg.290	R006475	(gi:119866188) mce2F (Mkms 0132) - virulence factor Mce family protein [Mycobacterium sp. str. KMS]	66.73	515	516
fig|6666666.28482.peg.290	R006403	(gi:118473635) mce1F (MSMEG 0139) - mce-family protein mce1f [Mycobacterium smegmatis str. MC2 155]	65.07	515	516

fig|6666666.28482.peg.290	R006472	(gi:145221301) mce2F (Mflv 0706) - virulence factor Mce family protein [Mycobacterium gilvum str. PYR-GCK]	64.69	516	516
fig|6666666.28482.peg.290	R006473	(gi:120401167) mce2F (Mvan 0139) - virulence factor Mce family protein [Mycobacterium vanbaalenii str. PYR-1]	64.5	516	516
fig|6666666.28482.peg.290	R019361	(gi:333988767) mce1F (JDM601 0127) - MCE-family protein Mce1F [Mycobacterium sp. str. JDM601]	61.44	514	516
fig|6666666.28482.peg.290	R006400	(gi:118462429) mce1F (MAV 5010) - mce-family protein mce1f [Mycobacterium avium str. 104]	72.61	313	516
fig|6666666.28482.peg.291	R019380	(gi:340625623) mce2E (MCAN 05991) - putative MCE-family lipoprotein LPRL [Mycobacterium canettii str. CIPT 140010059]	81.05	380	380
fig|6666666.28482.peg.291	R019381	(gi:253797528) mce2E (TBMG 00601) - MCE-family lipoprotein mce2E [Mycobacterium tuberculosis str. KZN 1435]	80.79	380	380
fig|6666666.28482.peg.291	R006453	(gi:15607733) mce2E (Rv0593) - POSSIBLE MCE-FAMILY LIPOPROTEIN LPRL (MCE-FAMILY LIPOPROTEIN MCE2E) [Mycobacterium tuberculosis str. H37Rv]	80.79	380	380
fig|6666666.28482.peg.291	R006454	(gi:148660363) mce2E (MRA 0601) - MCE-family lipoprotein LprL [Mycobacterium tuberculosis str. H37Ra]	80.79	380	380
fig|6666666.28482.peg.291	R013522	(gi:148821796) mce2E (TBFG 10605) - MCE-family lipoprotein lprL (MCE-family lipoprotein mce2e) [Mycobacterium tuberculosis str. F11]	80.79	380	380
fig|6666666.28482.peg.291	R006455	(gi:15839996) mce2E (MT0623) - virulence factor mce family protein [Mycobacterium tuberculosis str. CDC1551]	80.79	380	380
fig|6666666.28482.peg.291	R019382	(gi:224988983) mce2E (JTY 0608) - putative MCE-family lipoprotein [Mycobacterium bovis str. BCG str. Tokyo 172]	80.79	380	380
fig|6666666.28482.peg.291	R006457	(gi:121636511) mce2E (BCG 0639) - putative mce-family lipoprotein lprL [Mycobacterium bovis str. BCG Pasteur 1173P2]	80.79	380	380
fig|6666666.28482.peg.291	R019379	(gi:339630661) mce2E (MAF 06000) - putative MCE-family lipoprotein LPRL (MCE-family lipoprotein MCE2E) [Mycobacterium africanum str. GM041182]	80.79	380	380
fig|6666666.28482.peg.291	R006459	(gi:118462367) mce2E (MAV 4547) - virulence factor Mce family protein [Mycobacterium avium str. 104]	81.48	378	380
fig|6666666.28482.peg.291	R006458	(gi:41410186) mce2E (MAP4088) - LprL [Mycobacterium avium str. K-10 (subsp. paratuberculosis)]	80.69	378	380

fig|6666666.28482.peg.291	R006460	(gi:145221302) mce2E (Mflv 0707) - virulence factor Mce family protein [Mycobacterium gilvum str. PYR-GCK]	67.5	359	380
fig|6666666.28482.peg.291	R006461	(gi:120401166) mce2E (Mvan 0138) - virulence factor Mce family protein [Mycobacterium vanbaalenii str. PYR-1]	67.69	358	380
fig|6666666.28482.peg.291	R006393	(gi:118472695) mce1E (MSMEG 0138) - virulence factor Mce family protein [Mycobacterium smegmatis str. MC2 155]	67.5	359	380
fig|6666666.28482.peg.291	R006389	(gi:41409706) mce1E (MAP3608) - LprK [Mycobacterium avium str. K-10 (subsp. paratuberculosis)]	61.05	380	380
fig|6666666.28482.peg.291	R006464	(gi:108797102) mce2E (Mmcs 0122) - Mammalian cell entry [Mycobacterium sp. str. MCS]	68.06	359	380
fig|6666666.28482.peg.291	R006463	(gi:119866187) mce2E (Mkms 0131) - virulence factor Mce family protein [Mycobacterium sp. str. KMS]	68.06	359	380
fig|6666666.28482.peg.291	R006462	(gi:126432725) mce2E (Mjls 0112) - virulence factor Mce family protein [Mycobacterium sp. str. JLS]	68.33	359	380
fig|6666666.28482.peg.291	R006390	(gi:118466982) mce1E (MAV 5011) - virulence factor Mce family protein [Mycobacterium avium str. 104]	61.05	380	380
fig|6666666.28482.peg.291	R019350	(gi:340625210) mce1E (MCAN 01791) - putative MCE-family lipoprotein LPRK [Mycobacterium canettii str. CIPT 140010059]	64.53	358	380
fig|6666666.28482.peg.291	R019348	(gi:183980447) mce1E (MMAR 0416) - MCE family lipoprotein LprK [Mycobacterium marinum str. M]	62.4	359	380
fig|6666666.28482.peg.291	R006384	(gi:15607314) mce1E (Rv0173) - POSSIBLE MCE-FAMILY LIPOPROTEIN LPRK (MCE-FAMILY LIPOPROTEIN MCE1E) [Mycobacterium tuberculosis str. H37Rv]	64.53	358	380
fig|6666666.28482.peg.291	R006385	(gi:148659937) mce1E (MRA 0181) - MCE-family lipoprotein LprK [Mycobacterium tuberculosis str. H37Ra]	64.53	358	380
fig|6666666.28482.peg.291	R013516	(gi:148821365) mce1E (TBFG 10174) - MCE-family lipoprotein lprK (MCE-family lipoprotein mce1e) [Mycobacterium tuberculosis str. F11]	64.53	358	380
fig|6666666.28482.peg.291	R019352	(gi:224988558) mce1E (JTY 0179) - putative MCE-family lipoprotein mce1E [Mycobacterium bovis str. BCG str. Tokyo 172]	64.53	358	380
fig|6666666.28482.peg.291	R006388	(gi:121636085) mce1E (BCG 0210) - putative mce-family lipoprotein lprK (MCE-family lipoprotein mce1E) [Mycobacterium bovis str. BCG Pasteur 1173P2]	64.53	358	380

fig|6666666.28482.peg.291	R006387	(gi:31791351) mce1E (Mb0179) - POSSIBLE MCE-FAMILY LIPOPROTEIN LPRK (MCE-FAMILY LIPOPROTEIN MCE1E) [Mycobacterium bovis str. AF2122/97]	64.53	358	380
fig|6666666.28482.peg.291	R019349	(gi:339630254) mce1E (MAF 01740) - putative MCE-family lipoprotein LprK (MCE-family lipoprotein Mce1E) [Mycobacterium africanum str. GM041182]	64.53	358	380
fig|6666666.28482.peg.291	R019351	(gi:253797095) mce1E (TBMG 00174) - MCE-family lipoprotein mce1E [Mycobacterium tuberculosis str. KZN 1435]	64.53	358	380
fig|6666666.28482.peg.291	R006386	(gi:15839551) mce1E (MT0182) - virulence factor mce family protein [Mycobacterium tuberculosis str. CDC1551]	64.53	358	380
fig|6666666.28482.peg.291	R006392	(gi:118616799) mce1E (MUL 1066) - MCE-family lipoprotein LprK (MCE-family lipoprotein Mce1E) [Mycobacterium ulcerans str. Agy99]	62.12	359	380
fig|6666666.28482.peg.291	R006391	(gi:15828397) mce1E (ML2593) - putative lipoprotein [Mycobacterium leprae str. TN]	60.17	359	380
fig|6666666.28482.peg.291	R019353	(gi:221230874) mce1E (MLBr 02593) - putative lipoprotein [Mycobacterium leprae str. Br4923]	60.17	359	380
fig|6666666.28482.peg.292	R006446	(gi:41410185) mce2D (MAP4087) - hypothetical protein [Mycobacterium avium str. K-10 (subsp. paratuberculosis)]	87.73	481	485
fig|6666666.28482.peg.292	R006447	(gi:118464414) mce2D (MAV 4548) - virulence factor mce family protein [Mycobacterium avium str. 104]	87.32	481	485
fig|6666666.28482.peg.292	R019377	(gi:253797527) mce2D (TBMG 00600) - MCE-family protein mce2D [Mycobacterium tuberculosis str. KZN 1435]	84.2	481	485
fig|6666666.28482.peg.292	R006441	(gi:15607732) mce2D (Rv0592) - MCE-FAMILY PROTEIN MCE2D [Mycobacterium tuberculosis str. H37Rv]	84.2	481	485
fig|6666666.28482.peg.292	R006442	(gi:148660362) mce2D (MRA 0600) - MCE-family protein Mce2D [Mycobacterium tuberculosis str. H37Ra]	84.2	481	485
fig|6666666.28482.peg.292	R013521	(gi:148821795) mce2D (TBFG 10604) - MCE-family protein mce2D [Mycobacterium tuberculosis str. F11]	84.2	481	485
fig|6666666.28482.peg.292	R006443	(gi:15839995) mce2D (MT0622) - virulence factor mce family protein [Mycobacterium tuberculosis str. CDC1551]	84.2	481	485
fig|6666666.28482.peg.292	R019376	(gi:340625622) mce2D (MCAN 05981) - MCE-family protein MCE2D [Mycobacterium canettii str. CIPT 140010059]	84.2	481	485
fig|6666666.28482.peg.292	R019375	(gi:339630660) mce2D (MAF 05990) - MCE-family protein MCE2D [Mycobacterium africanum str. GM041182]	84.2	481	485

fig|6666666.28482.peg.292	R006444	(gi:31791774) mce2D (Mb0607) - MCE-FAMILY PROTEIN MCE2DA [FIRST PART] [Mycobacterium bovis str. AF2122/97]	83.01	471	485
fig|6666666.28482.peg.292	R019378	(gi:224988982) mce2D (JTY 0607) - MCE-family protein [Mycobacterium bovis str. BCG str. Tokyo 172]	83.66	465	485
fig|6666666.28482.peg.292	R006445	(gi:121636510) mce2D (BCG 0637) - mce-family protein mce2Da [Mycobacterium bovis str. BCG Pasteur 1173P2]	83.66	465	485
fig|6666666.28482.peg.292	R019343	(gi:340625209) mce1D (MCAN 01781) - MCE-family protein MCE1D [Mycobacterium canettii str. CIPT 140010059]	67.57	480	485
fig|6666666.28482.peg.292	R006374	(gi:15607313) mce1D (Rv0172) - MCE-FAMILY PROTEIN MCE1D [Mycobacterium tuberculosis str. H37Rv]	67.36	480	485
fig|6666666.28482.peg.292	R006375	(gi:148659936) mce1D (MRA 0180) - MCE-family protein Mce1D [Mycobacterium tuberculosis str. H37Ra]	67.36	480	485
fig|6666666.28482.peg.292	R006376	(gi:15839550) mce1D (MT0181) - virulence factor mce family protein [Mycobacterium tuberculosis str. CDC1551]	67.36	480	485
fig|6666666.28482.peg.292	R019345	(gi:224988557) mce1D (JTY 0178) - MCE-family protein [Mycobacterium bovis str. BCG str. Tokyo 172]	67.36	480	485
fig|6666666.28482.peg.292	R006378	(gi:121636084) mce1D (BCG 0209) - MCE-family protein mce1D [Mycobacterium bovis str. BCG Pasteur 1173P2]	67.36	480	485
fig|6666666.28482.peg.292	R006377	(gi:31791350) mce1D (Mb0178) - MCE-FAMILY PROTEIN MCE1D [Mycobacterium bovis str. AF2122/97]	67.36	480	485
fig|6666666.28482.peg.292	R019342	(gi:339630253) mce1D (MAF 01730) - MCE-family protein MCE1D [Mycobacterium africanum str. GM041182]	67.36	480	485
fig|6666666.28482.peg.292	R019344	(gi:253797094) mce1D (TBMG 00173) - MCE-family protein mce1D [Mycobacterium tuberculosis str. KZN 1435]	67.15	480	485
fig|6666666.28482.peg.292	R013515	(gi:148821364) mce1D (TBFG 10173) - MCE-family protein mce1D [Mycobacterium tuberculosis str. F11]	67.15	480	485
fig|6666666.28482.peg.292	R006452	(gi:108797101) mce2D (Mmcs 0121) - Mammalian cell entry [Mycobacterium sp. str. MCS]	66.6	480	485
fig|6666666.28482.peg.292	R006451	(gi:119866186) mce2D (Mkms 0130) - virulence factor Mce family protein [Mycobacterium sp. str. KMS]	66.6	480	485
fig|6666666.28482.peg.292	R006450	(gi:126432724) mce2D (Mjls 0111) - virulence factor Mce family protein [Mycobacterium sp. str. JLS]	66.6	480	485

fig|6666666.28482.peg.292	R019341	(gi:183980446) mce1D (MMAR 0415) - MCE-family protein Mce1D [Mycobacterium marinum str. M]	65.7	480	485
fig|6666666.28482.peg.292	R006379	(gi:41409705) mce1D (MAP3607) - hypothetical protein [Mycobacterium avium str. K-10 (subsp. paratuberculosis)]	66.53	481	485
fig|6666666.28482.peg.292	R006380	(gi:118466487) mce1D (MAV 5012) - mce-family protein mce1d [Mycobacterium avium str. 104]	66.32	481	485
fig|6666666.28482.peg.292	R006382	(gi:118616798) mce1D (MUL 1065) - MCE-family protein Mce1D [Mycobacterium ulcerans str. Agy99]	65.28	480	485
fig|6666666.28482.peg.292	R006449	(gi:120401165) mce2D (Mvan 0137) - virulence factor Mce family protein [Mycobacterium vanbaalenii str. PYR-1]	65.31	481	485
fig|6666666.28482.peg.292	R006448	(gi:145221303) mce2D (Mflv 0708) - virulence factor Mce family protein [Mycobacterium gilvum str. PYR-GCK]	65.44	481	485
fig|6666666.28482.peg.292	R006383	(gi:118471615) mce1D (MSMEG 0137) - virulence factor mce family protein [Mycobacterium smegmatis str. MC2 155]	63.21	481	485
fig|6666666.28482.peg.292	R006381	(gi:15828396) mce1D (ML2592) - putative secreted protein [Mycobacterium leprae str. TN]	64.6	480	485
fig|6666666.28482.peg.292	R019346	(gi:221230873) mce1D (MLBr 02592) - putative secreted protein [Mycobacterium leprae str. Br4923]	64.6	480	485
fig|6666666.28482.peg.292	R019347	(gi:333988765) mce1D (JDM601 0125) - MCE-family protein Mce1D [Mycobacterium sp. str. JDM601]	61	481	485
fig|6666666.28482.peg.293	R006435	(gi:118463703) mce2C (MAV 4549) - mce-family protein mce2c [Mycobacterium avium str. 104]	86.57	469	474
fig|6666666.28482.peg.293	R006434	(gi:41410184) mce2C (MAP4086) - hypothetical protein [Mycobacterium avium str. K-10 (subsp. paratuberculosis)]	85.93	469	474
fig|6666666.28482.peg.293	R019371	(gi:339630659) mce2C (MAF 05980) - MCE-family protein MCE2C [Mycobacterium africanum str. GM041182]	79.96	469	474
fig|6666666.28482.peg.293	R019374	(gi:224988981) mce2C (JTY 0606) - MCE-family protein [Mycobacterium bovis str. BCG str. Tokyo 172]	79.74	469	474
fig|6666666.28482.peg.293	R006433	(gi:121636509) mce2C (BCG 0636) - mce-family protein mce2C [Mycobacterium bovis str. BCG Pasteur 1173P2]	79.74	469	474
fig|6666666.28482.peg.293	R006432	(gi:31791773) mce2C (Mb0606) - MCE-FAMILY PROTEIN MCE2C [Mycobacterium bovis str. AF2122/97]	79.74	469	474

fig|6666666.28482.peg.293	R019373	(gi:253797526) mce2C (TBMG 00599) - MCE-family protein mce2C [Mycobacterium tuberculosis str. KZN 1435]	79.74	469	474
fig|6666666.28482.peg.293	R006429	(gi:15607731) mce2C (Rv0591) - MCE-FAMILY PROTEIN MCE2C [Mycobacterium tuberculosis str. H37Rv]	79.74	469	474
fig|6666666.28482.peg.293	R006430	(gi:148660361) mce2C (MRA 0599) - MCE-family protein Mce2C [Mycobacterium tuberculosis str. H37Ra]	79.74	469	474
fig|6666666.28482.peg.293	R013520	(gi:148821794) mce2C (TBFG 10603) - MCE-family protein mce2C [Mycobacterium tuberculosis str. F11]	79.74	469	474
fig|6666666.28482.peg.293	R019372	(gi:340625621) mce2C (MCAN 05971) - MCE-family protein MCE2C [Mycobacterium canettii str. CIPT 140010059]	80.17	469	474
fig|6666666.28482.peg.293	R006369	(gi:41409704) mce1C (MAP3606) - hypothetical protein [Mycobacterium avium str. K-10 (subsp. paratuberculosis)]	67.59	469	474
fig|6666666.28482.peg.293	R006370	(gi:118467050) mce1C (MAV 5013) - mce-family protein mce1c [Mycobacterium avium str. 104]	67.59	469	474
fig|6666666.28482.peg.293	R006364	(gi:15607312) mce1C (Rv0171) - MCE-FAMILY PROTEIN MCE1C [Mycobacterium tuberculosis str. H37Rv]	68.8	468	474
fig|6666666.28482.peg.293	R006365	(gi:148659935) mce1C (MRA 0179) - MCE-family protein Mce1C [Mycobacterium tuberculosis str. H37Ra]	68.8	468	474
fig|6666666.28482.peg.293	R013514	(gi:148821363) mce1C (TBFG 10172) - MCE-family protein mce1C [Mycobacterium tuberculosis str. F11]	68.8	468	474
fig|6666666.28482.peg.293	R019336	(gi:340625208) mce1C (MCAN 01771) - MCE-family protein MCE1C [Mycobacterium canettii str. CIPT 140010059]	69.02	468	474
fig|6666666.28482.peg.293	R019338	(gi:224988556) mce1C (JTY 0177) - MCE-family protein [Mycobacterium bovis str. BCG str. Tokyo 172]	68.8	468	474
fig|6666666.28482.peg.293	R006368	(gi:121636083) mce1C (BCG 0208) - MCE-family protein mce1C [Mycobacterium bovis str. BCG Pasteur 1173P2]	68.8	468	474
fig|6666666.28482.peg.293	R006367	(gi:31791349) mce1C (Mb0177) - MCE-FAMILY PROTEIN MCE1C [Mycobacterium bovis str. AF2122/97]	68.8	468	474
fig|6666666.28482.peg.293	R019335	(gi:339630252) mce1C (MAF 01720) - MCE-family protein MCE1C [Mycobacterium africanum str. GM041182]	68.8	468	474
fig|6666666.28482.peg.293	R006366	(gi:15839549) mce1C (MT0180) - virulence factor mce family protein [Mycobacterium tuberculosis str. CDC1551]	69	458	474

fig|6666666.28482.peg.293	R006373	(gi:118473407) mce1C (MSMEG 0136) - virulence factor Mce family protein, putative [Mycobacterium smegmatis str. MC2 155]	63.68	468	474
fig|6666666.28482.peg.293	R006431	(gi:15839994) mce2C (MT0621) - virulence factor mce family protein [Mycobacterium tuberculosis str. CDC1551]	82.02	356	474
fig|6666666.28482.peg.293	R019337	(gi:253797093) mce1C (TBMG 00172) - MCE-family protein mce1C [Mycobacterium tuberculosis str. KZN 1435]	68.94	454	474
fig|6666666.28482.peg.293	R006438	(gi:126432723) mce2C (Mjls 0110) - virulence factor Mce family protein [Mycobacterium sp. str. JLS]	64.27	454	474
fig|6666666.28482.peg.293	R019334	(gi:183980445) mce1C (MMAR 0414) - MCE-family protein Mce1C [Mycobacterium marinum str. M]	65.75	472	474
fig|6666666.28482.peg.293	R006440	(gi:108797100) mce2C (Mmcs 0120) - Mammalian cell entry [Mycobacterium sp. str. MCS]	64.71	454	474
fig|6666666.28482.peg.293	R006439	(gi:119866185) mce2C (Mkms 0129) - virulence factor Mce family protein [Mycobacterium sp. str. KMS]	64.71	454	474
fig|6666666.28482.peg.293	R006437	(gi:120401164) mce2C (Mvan 0136) - virulence factor Mce family protein [Mycobacterium vanbaalenii str. PYR-1]	63.64	451	474
fig|6666666.28482.peg.293	R006371	(gi:15828395) mce1C (ML2591) - putative secreted protein [Mycobacterium leprae str. TN]	62.63	469	474
fig|6666666.28482.peg.293	R019339	(gi:221230872) mce1C (MLBr 02591) - putative secreted protein [Mycobacterium leprae str. Br4923]	62.63	469	474
fig|6666666.28482.peg.293	R006372	(gi:118616797) mce1C (MUL 1064) - MCE-family protein Mce1C [Mycobacterium ulcerans str. Agy99]	65.12	472	474
fig|6666666.28482.peg.293	R006436	(gi:145221304) mce2C (Mflv 0709) - virulence factor Mce family protein [Mycobacterium gilvum str. PYR-GCK]	62.97	451	474
fig|6666666.28482.peg.294	R006422	(gi:41410183) mce2B (MAP4085) - hypothetical protein [Mycobacterium avium str. K-10 (subsp. paratuberculosis)]	87.46	343	343
fig|6666666.28482.peg.294	R019368	(gi:340625620) mce2B (MCAN 05961) - mce-family protein mce2b [Mycobacterium canettii str. CIPT 140010059]	82.51	343	343
fig|6666666.28482.peg.294	R019370	(gi:224988980) mce2B (JTY 0605) - MCE-family protein [Mycobacterium bovis str. BCG str. Tokyo 172]	82.51	343	343
fig|6666666.28482.peg.294	R006421	(gi:121636508) mce2B (BCG 0635) - mce-family protein mce2B [Mycobacterium bovis str. BCG Pasteur 1173P2]	82.51	343	343

fig|6666666.28482.peg.294	R006420	(gi:31791772) mce2B (Mb0605) - MCE-FAMILY PROTEIN MCE2B [Mycobacterium bovis str. AF2122/97]	82.51	343	343
fig|6666666.28482.peg.294	R019367	(gi:339630658) mce2B (MAF 05970) - MCE-family protein MCE2B [Mycobacterium africanum str. GM041182]	82.22	343	343
fig|6666666.28482.peg.294	R006423	(gi:118465867) mce2B (MAV 4550) - virulence factor Mce family protein [Mycobacterium avium str. 104]	87.17	343	343
fig|6666666.28482.peg.294	R006424	(gi:145221305) mce2B (Mflv 0710) - virulence factor Mce family protein [Mycobacterium gilvum str. PYR-GCK]	69.97	343	343
fig|6666666.28482.peg.294	R006428	(gi:108797099) mce2B (Mmcs 0119) - Mammalian cell entry [Mycobacterium sp. str. MCS]	70.55	343	343
fig|6666666.28482.peg.294	R006427	(gi:119866184) mce2B (Mkms 0128) - virulence factor Mce family protein [Mycobacterium sp. str. KMS]	70.55	343	343
fig|6666666.28482.peg.294	R006426	(gi:126432722) mce2B (Mjls 0109) - virulence factor Mce family protein [Mycobacterium sp. str. JLS]	70.55	343	343
fig|6666666.28482.peg.294	R006425	(gi:120401163) mce2B (Mvan 0135) - virulence factor Mce family protein [Mycobacterium vanbaalenii str. PYR-1]	69.68	343	343
fig|6666666.28482.peg.294	R006359	(gi:41409703) mce1B (MAP3605) - hypothetical protein [Mycobacterium avium str. K-10 (subsp. paratuberculosis)]	66.76	343	343
fig|6666666.28482.peg.294	R006360	(gi:118463171) mce1B (MAV 5014) - virulence factor Mce family protein [Mycobacterium avium str. 104]	66.76	343	343
fig|6666666.28482.peg.294	R019327	(gi:183980444) mce1B (MMAR 0413) - MCE-family protein Mce1B [Mycobacterium marinum str. M]	65.61	343	343
fig|6666666.28482.peg.294	R006362	(gi:118616796) mce1B (MUL 1063) - MCE-family protein Mce1B [Mycobacterium ulcerans str. Agy99]	65.61	343	343
fig|6666666.28482.peg.294	R006361	(gi:15828394) mce1B (ML2590) - putative secreted protein [Mycobacterium leprae str. TN]	65.32	343	343
fig|6666666.28482.peg.294	R019332	(gi:221230871) mce1B (MLBr 02590) - putative secreted protein [Mycobacterium leprae str. Br4923]	65.32	343	343
fig|6666666.28482.peg.294	R006363	(gi:118468987) mce1B (MSMEG 0135) - virulence factor Mce family protein [Mycobacterium smegmatis str. MC2 155]	64.72	343	343
fig|6666666.28482.peg.294	R019329	(gi:340625207) mce1B (MCAN 01761) - MCE-family protein MCE1B [Mycobacterium canettii str. CIPT 140010059]	63.29	343	343

fig|6666666.28482.peg.294	R019330	(gi:253797092) mce1B (TBMG 00171) - MCE-family protein mce1B [Mycobacterium tuberculosis str. KZN 1435]	63.29	343	343
fig|6666666.28482.peg.294	R006354	(gi:15607311) mce1B (Rv0170) - MCE-FAMILY PROTEIN MCE1B [Mycobacterium tuberculosis str. H37Rv]	63.29	343	343
fig|6666666.28482.peg.294	R006355	(gi:148659934) mce1B (MRA 0178) - MCE-family protein Mce1B [Mycobacterium tuberculosis str. H37Ra]	63.29	343	343
fig|6666666.28482.peg.294	R013513	(gi:148821362) mce1B (TBFG 10171) - MCE-family protein mce1B [Mycobacterium tuberculosis str. F11]	63.29	343	343
fig|6666666.28482.peg.294	R006356	(gi:15839548) mce1B (MT0179) - virulence factor mce family protein [Mycobacterium tuberculosis str. CDC1551]	63.29	343	343
fig|6666666.28482.peg.294	R019331	(gi:224988555) mce1B (JTY 0176) - MCE-family protein [Mycobacterium bovis str. BCG str. Tokyo 172]	63.01	343	343
fig|6666666.28482.peg.294	R006358	(gi:121636082) mce1B (BCG 0207) - MCE-family protein mce1B [Mycobacterium bovis str. BCG Pasteur 1173P2]	63.01	343	343
fig|6666666.28482.peg.294	R006357	(gi:31791348) mce1B (Mb0176) - MCE-FAMILY PROTEIN MCE1B [Mycobacterium bovis str. AF2122/97]	63.01	343	343
fig|6666666.28482.peg.294	R019328	(gi:339630251) mce1B (MAF 01710) - MCE-family protein MCE1B [Mycobacterium africanum str. GM041182]	63.01	343	343
fig|6666666.28482.peg.294	R019369	(gi:253797524) mce2B (TBMG 00597) - MCE-family protein mce2B [Mycobacterium tuberculosis str. KZN 1435]	83.59	256	343
fig|6666666.28482.peg.294	R006417	(gi:15607730) mce2B (Rv0590) - MCE-FAMILY PROTEIN MCE2B [Mycobacterium tuberculosis str. H37Rv]	83.59	256	343
fig|6666666.28482.peg.294	R006418	(gi:148660359) mce2B (MRA 0597) - MCE-family protein Mce2B [Mycobacterium tuberculosis str. H37Ra]	83.59	256	343
fig|6666666.28482.peg.294	R013519	(gi:148821792) mce2B (TBFG 10601) - MCE-family protein mce2B [Mycobacterium tuberculosis str. F11]	83.59	256	343
fig|6666666.28482.peg.294	R006419	(gi:15839992) mce2B (MT0619) - virulence factor mce family protein [Mycobacterium tuberculosis str. CDC1551]	83.2	256	343
fig|6666666.28482.peg.295	R006410	(gi:118465139) mce2A (MAV 4551) - virulence factor Mce family protein [Mycobacterium avium str. 104]	87.72	391	391
fig|6666666.28482.peg.295	R006409	(gi:41410182) mce2A (MAP4084) - Mce2 [Mycobacterium avium str. K-10 (subsp. paratuberculosis)]	87.03	370	391

fig|6666666.28482.peg.295	R019365	(gi:253797523) mce2A (TBMG 00596) - MCE-family protein mce2A [Mycobacterium tuberculosis str. KZN 1435]	82.86	391	391
fig|6666666.28482.peg.295	R013518	(gi:148821791) mce2A (TBFG 10600) - MCE-family protein mce2A [Mycobacterium tuberculosis str. F11]	82.86	391	391
fig|6666666.28482.peg.295	R006406	(gi:15839991) mce2A (MT0618) - virulence factor [Mycobacterium tuberculosis str. CDC1551]	82.86	391	391
fig|6666666.28482.peg.295	R019364	(gi:340625619) mce2A (MCAN 05951) - MCE-family protein MCE2A [Mycobacterium canettii str. CIPT 140010059]	82.61	391	391
fig|6666666.28482.peg.295	R006404	(gi:57116756) mce2A (Rv0589) - MCE-FAMILY PROTEIN MCE2A [Mycobacterium tuberculosis str. H37Rv]	82.61	391	391
fig|6666666.28482.peg.295	R006405	(gi:148660358) mce2A (MRA 0596) - MCE-family protein Mce2A [Mycobacterium tuberculosis str. H37Ra]	82.61	391	391
fig|6666666.28482.peg.295	R019363	(gi:339630657) mce2A (MAF 05960) - MCE-family protein MCE2A [Mycobacterium africanum str. GM041182]	82.61	391	391
fig|6666666.28482.peg.295	R019366	(gi:224988979) mce2A (JTY 0604) - MCE-family protein [Mycobacterium bovis str. BCG str. Tokyo 172]	82.61	391	391
fig|6666666.28482.peg.295	R006408	(gi:121636507) mce2A (BCG 0634) - mce-family protein mce2A [Mycobacterium bovis str. BCG Pasteur 1173P2]	82.61	391	391
fig|6666666.28482.peg.295	R006407	(gi:31791771) mce2A (Mb0604) - MCE-FAMILY PROTEIN MCE2A [Mycobacterium bovis str. AF2122/97]	82.61	391	391
fig|6666666.28482.peg.295	R006413	(gi:120401162) mce2A (Mvan 0134) - virulence factor Mce family protein [Mycobacterium vanbaalenii str. PYR-1]	67.35	387	391
fig|6666666.28482.peg.295	R006412	(gi:145221306) mce2A (Mflv 0711) - virulence factor Mce family protein [Mycobacterium gilvum str. PYR-GCK]	67.01	389	391
fig|6666666.28482.peg.295	R006416	(gi:108797098) mce2A (Mmcs 0118) - Mammalian cell entry [Mycobacterium sp. str. MCS]	66.93	387	391
fig|6666666.28482.peg.295	R006415	(gi:119866183) mce2A (Mkms 0127) - virulence factor Mce family protein [Mycobacterium sp. str. KMS]	66.93	387	391
fig|6666666.28482.peg.295	R006414	(gi:126432721) mce2A (Mjls 0108) - virulence factor Mce family protein [Mycobacterium sp. str. JLS]	66.32	389	391
fig|6666666.28482.peg.295	R006353	(gi:118472305) mce1A (MSMEG 0134) - virulence factor Mce family protein [Mycobacterium smegmatis str. MC2 155]	65.12	387	391

fig|6666666.28482.peg.295	R019323	(gi:253797091) mce1A (TBMG 00170) - MCE-family protein mce1A [Mycobacterium tuberculosis str. KZN 1435]	62.47	387	391
fig|6666666.28482.peg.295	R006341	(gi:57116701) mce1A (Rv0169) - MCE-FAMILY PROTEIN MCE1A [Mycobacterium tuberculosis str. H37Rv]	62.47	387	391
fig|6666666.28482.peg.295	R006342	(gi:148659933) mce1A (MRA 0177) - MCE-family protein Mce1A [Mycobacterium tuberculosis str. H37Ra]	62.47	387	391
fig|6666666.28482.peg.295	R013512	(gi:148821361) mce1A (TBFG 10170) - MCE-family protein mce1A [Mycobacterium tuberculosis str. F11]	62.47	387	391
fig|6666666.28482.peg.295	R006343	(gi:15839547) mce1A (MT0178) - virulence factor [Mycobacterium tuberculosis str. CDC1551]	62.47	387	391
fig|6666666.28482.peg.295	R019324	(gi:224988554) mce1A (JTY 0175) - MCE-family protein [Mycobacterium bovis str. BCG str. Tokyo 172]	61.24	387	391
fig|6666666.28482.peg.295	R006345	(gi:121636081) mce1A (BCG 0206) - MCE-family protein mce1A [Mycobacterium bovis str. BCG Pasteur 1173P2]	61.24	387	391
fig|6666666.28482.peg.295	R006344	(gi:31791347) mce1A (Mb0175) - MCE-FAMILY PROTEIN MCE1A [Mycobacterium bovis str. AF2122/97]	61.24	387	391
fig|6666666.28482.peg.295	R019322	(gi:340625206) mce1A (MCAN 01751) - MCE-family protein MCE1A [Mycobacterium canettii str. CIPT 140010059]	61.24	387	391
fig|6666666.28482.peg.295	R019321	(gi:339630250) mce1A (MAF 01700) - MCE-family protein MCE1A [Mycobacterium africanum str. GM041182]	61.24	387	391
fig|6666666.28482.peg.295	R006349	(gi:118464153) mce1A (MAV 4125) - virulence factor Mce family protein [Mycobacterium avium str. 104]	62.8	385	391
fig|6666666.28482.peg.295	R019362	(gi:183984851) mce2A (MMAR 4883) - MCE-family protein Mce2A [Mycobacterium marinum str. M]	65.78	375	391
fig|6666666.28482.peg.295	R019320	(gi:183980443) mce1A (MMAR 0412) - MCE-family protein Mce1A [Mycobacterium marinum str. M]	62.2	389	391
fig|6666666.28482.peg.295	R006348	(gi:118465982) mce1A (MAV 5015) - virulence factor Mce family protein [Mycobacterium avium str. 104]	61.48	389	391
fig|6666666.28482.peg.295	R006352	(gi:118616795) mce1A (MUL 1062) - MCE-family protein Mce1A [Mycobacterium ulcerans str. Agy99]	61.96	389	391
fig|6666666.28482.peg.295	R006346	(gi:41409702) mce1A (MAP3604) - Mce1 2 [Mycobacterium avium str. K-10 (subsp. paratuberculosis)]	62.2	389	391

fig|6666666.28482.peg.295	R006350	(gi:118464886) mce1A (MAV 4126) - virulence factor Mce family protein [Mycobacterium avium str. 104]	63.59	372	391
fig|6666666.28482.peg.295	R006411	(gi:118616268) mce2A (MUL 0415) - MCE-family protein Mce2A [Mycobacterium ulcerans str. Agy99]	65.25	375	391
fig|6666666.28482.peg.295	R006347	(gi:41409387) mce1A (MAP3289c) - Mce1 1 [Mycobacterium avium str. K-10 (subsp. paratuberculosis)]	63.59	372	391
fig|6666666.28482.peg.385	R018510	(gi:118466437) eccE3 (MAV 4860) - hypothetical protein [Mycobacterium avium str. 104]	67.68	328	332
fig|6666666.28482.peg.385	R018511	(gi:15828359) eccE3 (ML2527) - hypothetical protein [Mycobacterium leprae str. TN]	62.85	320	332
fig|6666666.28482.peg.385	R019744	(gi:221230836) eccE3 (MLBr 02527) - hypothetical protein [Mycobacterium leprae str. Br4923]	62.85	320	332
fig|6666666.28482.peg.385	R019738	(gi:183980579) eccE3 (MMAR 0551) - transmembrane protein [Mycobacterium marinum str. M]	61.08	327	332
fig|6666666.28482.peg.385	R019742	(gi:253797219) eccE3 (TBMG 00297) - hypothetical protein [Mycobacterium tuberculosis str. KZN 1435]	62.96	321	332
fig|6666666.28482.peg.385	R018503	(gi:148821490) eccE3 (TBFG 10299) - transmembrane protein [Mycobacterium tuberculosis str. F11]	62.96	321	332
fig|6666666.28482.peg.385	R018506	(gi:15839678) eccE3 (MT0305) - hypothetical protein [Mycobacterium tuberculosis str. CDC1551]	62.96	321	332
fig|6666666.28482.peg.385	R019741	(gi:340625330) eccE3 (MCAN 03011) - hypothetical protein [Mycobacterium canettii str. CIPT 140010059]	62.96	321	332
fig|6666666.28482.peg.385	R019743	(gi:224988680) eccE3 (JTY 0302) - putative transmembrane protein [Mycobacterium bovis str. BCG str. Tokyo 172]	62.96	321	332
fig|6666666.28482.peg.385	R018508	(gi:121636207) eccE3 (BCG 0332) - putative transmembrane protein [Mycobacterium bovis str. BCG Pasteur 1173P2]	62.96	321	332
fig|6666666.28482.peg.385	R018507	(gi:31791471) eccE3 (Mb0300) - transmembrane protein [Mycobacterium bovis str. AF2122/97]	62.96	321	332
fig|6666666.28482.peg.385	R019740	(gi:339630367) eccE3 (MAF 02940) - putative conserved transmembrane protein [Mycobacterium africanum str. GM041182]	62.96	321	332
fig|6666666.28482.peg.385	R018504	(gi:15607433) eccE3 (Rv0292) - transmembrane protein [Mycobacterium tuberculosis str. H37Rv]	62.65	321	332

fig|6666666.28482.peg.385	R018505	(gi:148660058) eccE3 (MRA 0301) - putative transmembrane protein [Mycobacterium tuberculosis str. H37Ra]	62.65	321	332
fig|6666666.28482.peg.385	R018509	(gi:41409886) eccE3 (MAP3788) - hypothetical protein [Mycobacterium avium str. K-10 (subsp. paratuberculosis)]	67.08	243	332
fig|6666666.28482.peg.386	R019734	(gi:253797218) mycP3 (TBMG 00296) - membrane-anchored mycosin mycP3 [Mycobacterium tuberculosis str. KZN 1435]	75	460	467
fig|6666666.28482.peg.386	R018488	(gi:15607432) mycP3 (Rv0291) - membrane-anchored mycosin [Mycobacterium tuberculosis str. H37Rv]	75	460	467
fig|6666666.28482.peg.386	R018489	(gi:148660057) mycP3 (MRA 0300) - putative membrane-anchored mycosin MycP3 [Mycobacterium tuberculosis str. H37Ra]	75	460	467
fig|6666666.28482.peg.386	R018487	(gi:148821489) mycP3 (TBFG 10298) - membrane-anchored mycosin mycP3 [Mycobacterium tuberculosis str. F11]	75	460	467
fig|6666666.28482.peg.386	R019733	(gi:340625329) mycP3 (MCAN 03001) - putative membrane-anchored mycosin MYCP3 [Mycobacterium canettii str. CIPT 140010059]	75	460	467
fig|6666666.28482.peg.386	R019735	(gi:224988679) mycP3 (JTY 0301) - putative protease precursor [Mycobacterium bovis str. BCG str. Tokyo 172]	75	460	467
fig|6666666.28482.peg.386	R018492	(gi:121636206) mycP3 (BCG 0331) - putative protease precursor [Mycobacterium bovis str. BCG Pasteur 1173P2]	75	460	467
fig|6666666.28482.peg.386	R018491	(gi:31791470) mycP3 (Mb0299) - protease precursor [Mycobacterium bovis str. AF2122/97]	75	460	467
fig|6666666.28482.peg.386	R019732	(gi:339630366) mycP3 (MAF 02930) - putative membrane-anchored mycosin mycp3 (serine protease) (subtilisin-like protease) (subtilase-like) (mycosin-3) [Mycobacterium africanum str. GM041182]	75	460	467
fig|6666666.28482.peg.386	R018495	(gi:15828360) mycP3 (ML2528) - protease [Mycobacterium leprae str. TN]	71.97	463	467
fig|6666666.28482.peg.386	R019736	(gi:221230837) mycP3 (MLBr 02528) - putative protease [Mycobacterium leprae str. Br4923]	71.97	463	467
fig|6666666.28482.peg.386	R019730	(gi:183980578) mycP3 (MMAR 0550) - membrane-anchored mycosin MycP3 [Mycobacterium marinum str. M]	72.9	459	467
fig|6666666.28482.peg.386	R018496	(gi:118616927) mycP3 (MUL 1213) - membrane-anchored mycosin MycP3 [Mycobacterium ulcerans str. Agy99]	72.47	459	467
fig|6666666.28482.peg.386	R018490	(gi:15839677) mycP3 (MT0304) - subtilase family protein [Mycobacterium tuberculosis str. CDC1551]	78.45	413	467

fig|6666666.28482.peg.386	R018494	(gi:118462463) mycP3 (MAV 4862) - subtilase [Mycobacterium avium str. 104]	80.52	420	467
fig|6666666.28482.peg.386	R018493	(gi:41409885) mycP3 (MAP3787) - hypothetical protein [Mycobacterium avium str. K-10 (subsp. paratuberculosis)]	80.09	421	467
fig|6666666.28482.peg.386	R018502	(gi:108797360) mycP3 (Mmcs 0380) - peptidase S8/S53 subtilisin kexin sedolisin [Mycobacterium sp. str. MCS]	66.82	437	467
fig|6666666.28482.peg.386	R018501	(gi:119866444) mycP3 (Mkms 0389) - peptidase S8/S53 subtilisin kexin sedolisin [Mycobacterium sp. str. KMS]	66.82	437	467
fig|6666666.28482.peg.386	R018500	(gi:126432981) mycP3 (Mjls 0368) - peptidase S8/S53 subtilisin kexin sedolisin [Mycobacterium sp. str. JLS]	66.82	437	467
fig|6666666.28482.peg.386	R018497	(gi:118469114) mycP3 (MSMEG 0624) - subtilase [Mycobacterium smegmatis str. MC2 155]	60.38	461	467
fig|6666666.28482.peg.386	R019737	(gi:333988939) mycP3 (JDM601 0299) - membrane-anchored mycosin MycP3 [Mycobacterium sp. str. JDM601]	70.31	417	467
fig|6666666.28482.peg.386	R018499	(gi:120401446) mycP3 (Mvan 0421) - peptidase S8/S53 subtilisin kexin sedolisin [Mycobacterium vanbaalenii str. PYR-1]	61.1	437	467
fig|6666666.28482.peg.386	R018498	(gi:145220924) mycP3 (Mflv 0320) - peptidase S8/S53 subtilisin kexin sedolisin [Mycobacterium gilvum str. PYR-GCK]	64.8	375	467
fig|6666666.28482.peg.387	R018478	(gi:118465427) eccD3 (MAV 4863) - hypothetical protein [Mycobacterium avium str. 104]	81.25	474	474
fig|6666666.28482.peg.387	R018477	(gi:41409884) eccD3 (MAP3786) - hypothetical protein [Mycobacterium avium str. K-10 (subsp. paratuberculosis)]	81.67	474	474
fig|6666666.28482.peg.387	R019726	(gi:253797217) eccD3 (TBMG 00295) - hypothetical protein [Mycobacterium tuberculosis str. KZN 1435]	80.72	472	474
fig|6666666.28482.peg.387	R018472	(gi:15607431) eccD3 (Rv0290) - transmembrane protein [Mycobacterium tuberculosis str. H37Rv]	80.72	472	474
fig|6666666.28482.peg.387	R018473	(gi:148660056) eccD3 (MRA 0299) - putative transmembrane protein [Mycobacterium tuberculosis str. H37Ra]	80.72	472	474
fig|6666666.28482.peg.387	R018471	(gi:148821488) eccD3 (TBFG 10297) - transmembrane protein [Mycobacterium tuberculosis str. F11]	80.72	472	474
fig|6666666.28482.peg.387	R018474	(gi:15839676) eccD3 (MT0303) - hypothetical protein [Mycobacterium tuberculosis str. CDC1551]	80.72	472	474

fig|6666666.28482.peg.387	R019724	(gi:339630365) eccD3 (MAF 02920) - putative conserved transmembrane protein [Mycobacterium africanum str. GM041182]	80.72	472	474
fig|6666666.28482.peg.387	R019725	(gi:340625328) eccD3 (MCAN 02991) - hypothetical protein [Mycobacterium canettii str. CIPT 140010059]	80.72	472	474
fig|6666666.28482.peg.387	R019727	(gi:224988678) eccD3 (JTY 0300) - putative transmembrane protein [Mycobacterium bovis str. BCG str. Tokyo 172]	80.51	472	474
fig|6666666.28482.peg.387	R018476	(gi:121636205) eccD3 (BCG 0330) - putative transmembrane protein [Mycobacterium bovis str. BCG Pasteur 1173P2]	80.51	472	474
fig|6666666.28482.peg.387	R018475	(gi:31791469) eccD3 (Mb0298) - transmembrane protein [Mycobacterium bovis str. AF2122/97]	80.51	472	474
fig|6666666.28482.peg.387	R018480	(gi:118616926) eccD3 (MUL 1212) - transmembrane protein [Mycobacterium ulcerans str. Agy99]	77.33	472	474
fig|6666666.28482.peg.387	R018479	(gi:15828361) eccD3 (ML2529) - hypothetical protein [Mycobacterium leprae str. TN]	76.37	474	474
fig|6666666.28482.peg.387	R019728	(gi:221230838) eccD3 (MLBr 02529) - hypothetical protein [Mycobacterium leprae str. Br4923]	76.37	474	474
fig|6666666.28482.peg.387	R019722	(gi:183980577) eccD3 (MMAR 0549) - transmembrane protein [Mycobacterium marinum str. M]	77.54	472	474
fig|6666666.28482.peg.387	R019729	(gi:333988938) eccD3 (JDM601 0298) - transmembrane protein [Mycobacterium sp. str. JDM601]	66.24	468	474
fig|6666666.28482.peg.387	R018481	(gi:118471932) eccD3 (MSMEG 0623) - secretion protein Snm4 [Mycobacterium smegmatis str. MC2 155]	60.88	474	474
fig|6666666.28482.peg.388	R019718	(gi:253797216) espG3 (TBMG 00294) - hypothetical protein [Mycobacterium tuberculosis str. KZN 1435]	77.63	289	293
fig|6666666.28482.peg.388	R018456	(gi:15607430) espG3 (Rv0289) - hypothetical protein [Mycobacterium tuberculosis str. H37Rv]	77.63	289	293
fig|6666666.28482.peg.388	R018457	(gi:148660055) espG3 (MRA 0298) - hypothetical protein [Mycobacterium tuberculosis str. H37Ra]	77.63	289	293
fig|6666666.28482.peg.388	R018455	(gi:148821487) espG3 (TBFG 10296) - hypothetical protein [Mycobacterium tuberculosis str. F11]	77.63	289	293
fig|6666666.28482.peg.388	R018458	(gi:15839675) espG3 (MT0302) - hypothetical protein [Mycobacterium tuberculosis str. CDC1551]	77.63	289	293

fig|6666666.28482.peg.388	R019717	(gi:340625327) espG3 (MCAN 02981) - hypothetical protein [Mycobacterium canettii str. CIPT 140010059]	77.63	289	293
fig|6666666.28482.peg.388	R019719	(gi:224988677) espG3 (JTY 0299) - hypothetical protein [Mycobacterium bovis str. BCG str. Tokyo 172]	77.63	289	293
fig|6666666.28482.peg.388	R018460	(gi:121636204) espG3 (BCG 0329) - hypothetical protein [Mycobacterium bovis str. BCG Pasteur 1173P2]	77.63	289	293
fig|6666666.28482.peg.388	R018459	(gi:31791468) espG3 (Mb0297) - hypothetical protein [Mycobacterium bovis str. AF2122/97]	77.63	289	293
fig|6666666.28482.peg.388	R019716	(gi:339630364) espG3 (MAF 02910) - hypothetical protein [Mycobacterium africanum str. GM041182]	77.63	289	293
fig|6666666.28482.peg.388	R018463	(gi:15828362) espG3 (ML2530) - DNA-binding protein [Mycobacterium leprae str. TN]	77.7	287	293
fig|6666666.28482.peg.388	R019720	(gi:221230839) espG3 (MLBr 02530) - putative DNA-binding protein [Mycobacterium leprae str. Br4923]	77.7	287	293
fig|6666666.28482.peg.388	R018462	(gi:118465963) espG3 (MAV 4864) - hypothetical protein [Mycobacterium avium str. 104]	77.13	288	293
fig|6666666.28482.peg.388	R019714	(gi:183980576) espG3 (MMAR 0548) - hypothetical protein [Mycobacterium marinum str. M]	74.32	287	293
fig|6666666.28482.peg.388	R018461	(gi:41409883) espG3 (MAP3785) - hypothetical protein [Mycobacterium avium str. K-10 (subsp. paratuberculosis)]	76.79	288	293
fig|6666666.28482.peg.388	R018464	(gi:118616925) espG3 (MUL 1211) - hypothetical protein [Mycobacterium ulcerans str. Agy99]	73.99	287	293
fig|6666666.28482.peg.388	R019721	(gi:333988937) espG3 (JDM601 0297) - hypothetical protein [Mycobacterium sp. str. JDM601]	71.33	283	293
fig|6666666.28482.peg.389	R018446	(gi:118465386) esxH (MAV 4865) - low molecular weight protein antigen 7 Cfp7 [Mycobacterium avium str. 104]	89.58	96	96
fig|6666666.28482.peg.389	R018445	(gi:41409882) esxH (MAP3784) - hypothetical protein [Mycobacterium avium str. K-10 (subsp. paratuberculosis)]	89.58	96	96
fig|6666666.28482.peg.389	R019706	(gi:183980575) esxH (MMAR 0547) - EsaT-6 like protein EsxH [Mycobacterium marinum str. M]	84.38	96	96
fig|6666666.28482.peg.389	R018448	(gi:118616924) esxH (MUL 1210) - EsaT-6 like protein EsxH [Mycobacterium ulcerans str. Agy99]	83.33	96	96

fig|6666666.28482.peg.389	R019710	(gi:253797215) esxH (TBMG 00293) - low molecular weight protein antigen 7 esxH [Mycobacterium tuberculosis str. KZN 1435]	86.46	96	96
fig|6666666.28482.peg.389	R018440	(gi:15607429) esxH (Rv0288) - low molecular weight protein antigen 7 ESXH (10 kDa antigen) (CFP-7) (protein TB10.4) [Mycobacterium tuberculosis str. H37Rv]	86.46	96	96
fig|6666666.28482.peg.389	R018441	(gi:148660054) esxH (MRA 0297) - antigen Esxh [Mycobacterium tuberculosis str. H37Ra]	86.46	96	96
fig|6666666.28482.peg.389	R018439	(gi:148821486) esxH (TBFG 10295) - putative antigen [Mycobacterium tuberculosis str. F11]	86.46	96	96
fig|6666666.28482.peg.389	R018442	(gi:15839674) esxH (MT0301) - secreted antigen, putative [Mycobacterium tuberculosis str. CDC1551]	86.46	96	96
fig|6666666.28482.peg.389	R019709	(gi:340625326) esxH (MCAN 02971) - low molecular weight protein antigen 7 ESXH [Mycobacterium canettii str. CIPT 140010059]	86.46	96	96
fig|6666666.28482.peg.389	R019711	(gi:224988676) esxH (JTY 0298) - low molecular weight protein antigen 7 [Mycobacterium bovis str. BCG str. Tokyo 172]	86.46	96	96
fig|6666666.28482.peg.389	R018444	(gi:121636203) esxH (BCG 0328) - low molecular weight protein antigen 7 cfp7 [Mycobacterium bovis str. BCG Pasteur 1173P2]	86.46	96	96
fig|6666666.28482.peg.389	R018443	(gi:31791467) esxH (Mb0296) - low molecular weight protein antigen 7 CFP7 (10 kDa antigen) (CFP-7) (protein TB10.4) [Mycobacterium bovis str. AF2122/97]	86.46	96	96
fig|6666666.28482.peg.389	R019708	(gi:339630363) esxH (MAF 02900) - low molecular weight protein antigen 7 EsxH (10 kDa antigen) (CFP-7) (protein TB10.4) [Mycobacterium africanum str. GM041182]	85.42	96	96
fig|6666666.28482.peg.389	R019713	(gi:333988928) esxH (JDM601 0288) - EsaT-6 like protein EsxH [Mycobacterium sp. str. JDM601]	84.21	95	96
fig|6666666.28482.peg.389	R018450	(gi:145220927) esxH (Mflv 0323) - hypothetical protein [Mycobacterium gilvum str. PYR-GCK]	76.84	95	96
fig|6666666.28482.peg.389	R018454	(gi:108797357) esxH (Mmcs 0377) - hypothetical protein [Mycobacterium sp. str. MCS]	75.79	95	96
fig|6666666.28482.peg.389	R018453	(gi:119866441) esxH (Mkms 0386) - hypothetical protein [Mycobacterium sp. str. KMS]	75.79	95	96
fig|6666666.28482.peg.389	R018452	(gi:126432978) esxH (Mjls 0365) - hypothetical protein [Mycobacterium sp. str. JLS]	75.79	95	96

fig|6666666.28482.peg.389	R018449	(gi:118470541) esxH (MSMEG 0621) - low molecular weight protein antigen 7 [Mycobacterium smegmatis str. MC2 155]	75.79	95	96
fig|6666666.28482.peg.389	R018451	(gi:120401443) esxH (Mvan 0418) - hypothetical protein [Mycobacterium vanbaalenii str. PYR-1]	74.74	95	96
fig|6666666.28482.peg.389	R018447	(gi:15828363) esxH (ML2531) - cell surface protein [Mycobacterium leprae str. TN]	70.83	96	96
fig|6666666.28482.peg.389	R019712	(gi:221230840) esxH (MLBr 02531) - putative cell surface protein [Mycobacterium leprae str. Br4923]	70.83	96	96
fig|6666666.28482.peg.389	R019707	(gi:169629314) esxH (MAB 2228c) - ESAT-6-like protein esxH [Mycobacterium abscessus str. ATCC 19977]	66.32	95	96
fig|6666666.28482.peg.390	R018687	(gi:41409881) esxG (MAP3783) - hypothetical protein [Mycobacterium avium str. K-10 (subsp. paratuberculosis)]	95.88	97	97
fig|6666666.28482.peg.390	R018688	(gi:118465920) esxG (MAV 4866) - PE family protein [Mycobacterium avium str. 104]	95.79	95	97
fig|6666666.28482.peg.390	R019904	(gi:126432977) esxG (Mjls 0364) - hypothetical protein [Mycobacterium sp. str. JLS]	78.35	97	97
fig|6666666.28482.peg.390	R019667	(gi:169629315) esxG (MAB 2229c) - PE family protein [Mycobacterium abscessus str. ATCC 19977]	76.29	97	97
fig|6666666.28482.peg.390	R018689	(gi:15828364) esxG (ML2532) - PE-family protein [Mycobacterium leprae str. TN]	76.04	96	97
fig|6666666.28482.peg.390	R019672	(gi:221230841) esxG (MLBr 02532) - PE-family protein [Mycobacterium leprae str. Br4923]	76.04	96	97
fig|6666666.28482.peg.390	R019906	(gi:108797356) esxG (Mmcs 0376) - hypothetical protein [Mycobacterium sp. str. MCS]	79.31	87	97
fig|6666666.28482.peg.390	R019905	(gi:119866440) esxG (Mkms 0385) - hypothetical protein [Mycobacterium sp. str. KMS]	79.31	87	97
fig|6666666.28482.peg.390	R019903	(gi:120401442) esxG (Mvan 0417) - hypothetical protein [Mycobacterium vanbaalenii str. PYR-1]	79.38	97	97
fig|6666666.28482.peg.390	R019673	(gi:333988927) esxG (JDM601 0287) - EsaT-6 like protein EsxG [Mycobacterium sp. str. JDM601]	77.89	95	97
fig|6666666.28482.peg.390	R019901	(gi:118469490) esxG (MSMEG 0620) - PE family protein [Mycobacterium smegmatis str. MC2 155]	76.29	97	97

fig|6666666.28482.peg.390	R019670	(gi:253797214) esxG (TBMG 00292) - esat-6 like protein esxG [Mycobacterium tuberculosis str. KZN 1435]	85.57	97	97
fig|6666666.28482.peg.390	R018682	(gi:15607428) esxG (Rv0287) - hypothetical protein [Mycobacterium tuberculosis str. H37Rv]	85.57	97	97
fig|6666666.28482.peg.390	R018683	(gi:148660053) esxG (MRA 0296) - PE family protein [Mycobacterium tuberculosis str. H37Ra]	85.57	97	97
fig|6666666.28482.peg.390	R018681	(gi:148821485) esxG (TBFG 10294) - Esat-6 like protein esxG [Mycobacterium tuberculosis str. F11]	85.57	97	97
fig|6666666.28482.peg.390	R018684	(gi:15839673) esxG (MT0300) - PE family protein [Mycobacterium tuberculosis str. CDC1551]	85.57	97	97
fig|6666666.28482.peg.390	R019669	(gi:340625325) esxG (MCAN 02961) - ESAT-6 like protein ESXG [Mycobacterium canettii str. CIPT 140010059]	85.57	97	97
fig|6666666.28482.peg.390	R019671	(gi:224988675) esxG (JTY 0297) - hypothetical protein TB9.8 [Mycobacterium bovis str. BCG str. Tokyo 172]	85.57	97	97
fig|6666666.28482.peg.390	R018686	(gi:121636202) esxG (BCG 0327) - hypothetical protein [Mycobacterium bovis str. BCG Pasteur 1173P2]	85.57	97	97
fig|6666666.28482.peg.390	R018685	(gi:31791466) esxG (Mb0295) - hypothetical protein [Mycobacterium bovis str. AF2122/97]	85.57	97	97
fig|6666666.28482.peg.390	R019668	(gi:339630362) esxG (MAF 02890) - ESAT-6 like protein ESXG (conserved hypothetical protein TB9.8) [Mycobacterium africanum str. GM041182]	85.57	97	97
fig|6666666.28482.peg.390	R018690	(gi:118616923) esxG (MUL 1209) - EsaT-6 like protein EsxG [Mycobacterium ulcerans str. Agy99]	84.54	97	97
fig|6666666.28482.peg.390	R019666	(gi:183980574) esxG (MMAR 0546) - EsaT-6 like protein EsxG [Mycobacterium marinum str. M]	84.54	97	97
fig|6666666.28482.peg.390	R019902	(gi:145220928) esxG (Mflv 0324) - hypothetical protein [Mycobacterium gilvum str. PYR-GCK]	74.23	97	97
fig|6666666.28482.peg.538	R006594	(gi:41408209) mce3F (MAP2111c) - hypothetical protein [Mycobacterium avium str. K-10 (subsp. paratuberculosis)]	87.2	492	492
fig|6666666.28482.peg.538	R006596	(gi:118466763) mce3F (MAV 2069) - virulence factor mce family protein [Mycobacterium avium str. 104]	86.3	489	492
fig|6666666.28482.peg.539	R006571	(gi:41408210) mce3E (MAP2112c) - LprM [Mycobacterium avium str. K-10 (subsp. paratuberculosis)]	88.76	356	369

fig|6666666.28482.peg.539	R006586	(gi:126436756) mce3E (Mjls 4184) - virulence factor Mce family protein [Mycobacterium sp. str. JLS]	62.6	361	369
fig|6666666.28482.peg.539	R006581	(gi:120401941) mce3E (Mvan 0926) - virulence factor Mce family protein [Mycobacterium vanbaalenii str. PYR-1]	61.33	362	369
fig|6666666.28482.peg.539	R019404	(gi:183984026) mce3E (MMAR 4055) - MCE family lipoprotein LprM [Mycobacterium marinum str. M]	63.28	353	369
fig|6666666.28482.peg.539	R006574	(gi:118619131) mce3E (MUL 3922) - MCE-family lipoprotein LprM 1 [Mycobacterium ulcerans str. Agy99]	62.99	353	369
fig|6666666.28482.peg.539	R006578	(gi:120401538) mce3E (Mvan 0517) - virulence factor Mce family protein [Mycobacterium vanbaalenii str. PYR-1]	62.3	364	369
fig|6666666.28482.peg.539	R006590	(gi:108798627) mce3E (Mmcs 1658) - Mammalian cell entry [Mycobacterium sp. str. MCS]	62.3	364	369
fig|6666666.28482.peg.539	R006588	(gi:119867723) mce3E (Mkms 1682) - virulence factor Mce family protein [Mycobacterium sp. str. KMS]	62.3	364	369
fig|6666666.28482.peg.539	R006585	(gi:126434224) mce3E (Mjls 1631) - virulence factor Mce family protein [Mycobacterium sp. str. JLS]	62.3	364	369
fig|6666666.28482.peg.539	R006577	(gi:145221247) mce3E (Mflv 0648) - virulence factor Mce family protein [Mycobacterium gilvum str. PYR-GCK]	62.3	364	369
fig|6666666.28482.peg.539	R006584	(gi:126436738) mce3E (Mjls 4166) - transcriptional regulator, Fis family [Mycobacterium sp. str. JLS]	61.37	362	369
fig|6666666.28482.peg.539	R019403	(gi:183982890) mce3E (MMAR 2886) - MCE family lipoprotein LprM [Mycobacterium marinum str. M]	60.87	362	369
fig|6666666.28482.peg.539	R019405	(gi:340626979) mce3E (MCAN 19861) - putative MCE-family lipoprotein LPRM [Mycobacterium canettii str. CIPT 140010059]	60.86	366	369
fig|6666666.28482.peg.539	R019406	(gi:253798982) mce3E (TBMG 02020) - MCE-family lipoprotein mce3E [Mycobacterium tuberculosis str. KZN 1435]	60.59	366	369
fig|6666666.28482.peg.539	R006568	(gi:15609107) mce3E (Rv1970) - POSSIBLE MCE-FAMILY LIPOPROTEIN LPRM (MCE-FAMILY LIPOPROTEIN MCE3E) [Mycobacterium tuberculosis str. H37Rv]	60.59	366	369
fig|6666666.28482.peg.539	R006569	(gi:148661779) mce3E (MRA 1981) - MCE-family lipoprotein LprM [Mycobacterium tuberculosis str. H37Ra]	60.59	366	369
fig|6666666.28482.peg.539	R013528	(gi:148823184) mce3E (TBFG 12001) - MCE-family lipoprotein lprM (MCE-family lipoprotein mce3e) [Mycobacterium tuberculosis str. F11]	60.59	366	369

fig|6666666.28482.peg.539	R006570	(gi:15841445) mce3E (MT2022) - virulence factor mce family protein [Mycobacterium tuberculosis str. CDC1551]	60.59	366	369
fig|6666666.28482.peg.539	R006575	(gi:118468700) mce3E (MSMEG 0350) - virulence factor Mce family protein [Mycobacterium smegmatis str. MC2 155]	60.89	357	369
fig|6666666.28482.peg.539	R006572	(gi:118463194) mce3E (MAV 2536) - virulence factor Mce family protein [Mycobacterium avium str. 104]	61.84	353	369
fig|6666666.28482.peg.539	R006583	(gi:120405552) mce3E (Mvan 4600) - virulence factor Mce family protein [Mycobacterium vanbaalenii str. PYR-1]	61.13	366	369
fig|6666666.28482.peg.539	R006576	(gi:145222693) mce3E (Mflv 2105) - virulence factor Mce family protein [Mycobacterium gilvum str. PYR-GCK]	61.83	366	369
fig|6666666.28482.peg.540	R006550	(gi:118465334) mce3D (MAV 2064) - virulence factor mce family protein [Mycobacterium avium str. 104]	78.7	446	446
fig|6666666.28482.peg.540	R006548	(gi:41408211) mce3D (MAP2113c) - hypothetical protein [Mycobacterium avium str. K-10 (subsp. paratuberculosis)]	78.7	446	446
fig|6666666.28482.peg.540	R006549	(gi:118466668) mce3D (MAV 2535) - virulence factor mce family protein [Mycobacterium avium str. 104]	61.6	362	446
fig|6666666.28482.peg.540	R019400	(gi:183984025) mce3D (MMAR 4054) - MCE-family protein Mce3D 1 [Mycobacterium marinum str. M]	61.17	358	446
fig|6666666.28482.peg.541	R006525	(gi:41408212) mce3C (MAP2114c) - hypothetical protein [Mycobacterium avium str. K-10 (subsp. paratuberculosis)]	83.33	360	390
fig|6666666.28482.peg.541	R006527	(gi:118466040) mce3C (MAV 2063) - virulence factor Mce family protein [Mycobacterium avium str. 104]	85.22	345	390
fig|6666666.28482.peg.542	R006503	(gi:41408213) mce3B (MAP2115c) - hypothetical protein [Mycobacterium avium str. K-10 (subsp. paratuberculosis)]	90.24	338	338
fig|6666666.28482.peg.542	R006505	(gi:118466491) mce3B (MAV 2062) - virulence factor Mce family protein [Mycobacterium avium str. 104]	89.94	338	338
fig|6666666.28482.peg.542	R006508	(gi:145222690) mce3B (Mflv 2102) - virulence factor Mce family protein [Mycobacterium gilvum str. PYR-GCK]	60.06	332	338
fig|6666666.28482.peg.543	R006480	(gi:41408214) mce3A (MAP2116c) - Mce3 [Mycobacterium avium str. K-10 (subsp. paratuberculosis)]	84.36	422	422
fig|6666666.28482.peg.543	R006482	(gi:118467175) mce3A (MAV 2061) - virulence factor Mce family protein [Mycobacterium avium str. 104]	84.36	422	422

fig|6666666.28482.peg.559	R019862	(gi:183982677) PE18 (MMAR 2670) - PE family protein, PE19 1 [Mycobacterium marinum str. M]	73.56	87	99
fig|6666666.28482.peg.559	R018659	(gi:118618471) PE18 (MUL 3088) - PE family protein [Mycobacterium ulcerans str. Agy99]	72.41	87	99
fig|6666666.28482.peg.559	R018680	(gi:41407605) PE19 (MAP1507) - hypothetical protein [Mycobacterium avium str. K-10 (subsp. paratuberculosis)]	73.56	87	99
fig|6666666.28482.peg.559	R019877	(gi:183982680) PE19 (MMAR 2673) - PE family protein, PE19 [Mycobacterium marinum str. M]	77.01	87	99
fig|6666666.28482.peg.559	R019865	(gi:253799172) PE18 (TBMG 02209) - PE family protein [Mycobacterium tuberculosis str. KZN 1435]	73.56	87	99
fig|6666666.28482.peg.559	R018654	(gi:57116910) PE18 (Rv1788) - PE family protein [Mycobacterium tuberculosis str. H37Rv]	73.56	87	99
fig|6666666.28482.peg.559	R018655	(gi:148661595) PE18 (MRA 1802) - PE family protein [Mycobacterium tuberculosis str. H37Ra]	73.56	87	99
fig|6666666.28482.peg.559	R018653	(gi:148823003) PE18 (TBFG 11818) - PE family protein [Mycobacterium tuberculosis str. F11]	73.56	87	99
fig|6666666.28482.peg.559	R018656	(gi:15841256) PE18 (MT1837) - PE family protein [Mycobacterium tuberculosis str. CDC1551]	73.56	87	99
fig|6666666.28482.peg.559	R019864	(gi:340626800) PE18 (MCAN 18061) - PE family protein [Mycobacterium canettii str. CIPT 140010059]	73.56	87	99
fig|6666666.28482.peg.559	R019866	(gi:224990173) PE18 (JTY 1804) - PE family protein [Mycobacterium bovis str. BCG str. Tokyo 172]	73.56	87	99
fig|6666666.28482.peg.559	R018658	(gi:121637689) PE18 (BCG 1820) - PE family protein [Mycobacterium bovis str. BCG Pasteur 1173P2]	73.56	87	99
fig|6666666.28482.peg.559	R018657	(gi:31792976) PE18 (Mb1816) - PE family protein [Mycobacterium bovis str. AF2122/97]	73.56	87	99
fig|6666666.28482.peg.559	R019863	(gi:339631842) PE18 (MAF 18100) - PE family protein [Mycobacterium africanum str. GM041182]	73.56	87	99
fig|6666666.28482.peg.559	R019880	(gi:253799168) PE19 (TBMG 02205) - PE family protein [Mycobacterium tuberculosis str. KZN 1435]	73.56	87	99
fig|6666666.28482.peg.559	R018674	(gi:148823006) PE19 (TBFG 11821) - PE family protein [Mycobacterium tuberculosis str. F11]	73.56	87	99

fig|6666666.28482.peg.559	R018677	(gi:15841261) PE19 (MT1840) - PE family protein [Mycobacterium tuberculosis str. CDC1551]	73.56	87	99
fig|6666666.28482.peg.559	R018675	(gi:57116913) PE19 (Rv1791) - PE family protein [Mycobacterium tuberculosis str. H37Rv]	73.56	87	99
fig|6666666.28482.peg.559	R018676	(gi:148661598) PE19 (MRA 1805) - PE family protein [Mycobacterium tuberculosis str. H37Ra]	73.56	87	99
fig|6666666.28482.peg.559	R019879	(gi:340626803) PE19 (MCAN 18091) - PE family protein [Mycobacterium canettii str. CIPT 140010059]	73.56	87	99
fig|6666666.28482.peg.559	R019881	(gi:224990176) PE19 (JTY 1807) - PE family protein [Mycobacterium bovis str. BCG str. Tokyo 172]	73.56	87	99
fig|6666666.28482.peg.559	R018679	(gi:121637692) PE19 (BCG 1823) - PE family protein [Mycobacterium bovis str. BCG Pasteur 1173P2]	73.56	87	99
fig|6666666.28482.peg.559	R018678	(gi:31792979) PE19 (Mb1819) - PE family protein [Mycobacterium bovis str. AF2122/97]	73.56	87	99
fig|6666666.28482.peg.559	R019878	(gi:339631845) PE19 (MAF 18130) - PE family protein [Mycobacterium africanum str. GM041182]	73.56	87	99
fig|6666666.28482.peg.559	R019867	(gi:333990534) PE18 (JDM601 1894) - PE-PGRS family protein [Mycobacterium sp. str. JDM601]	70.11	87	99
fig|6666666.28482.peg.694	R019488	(gi:183984673) mce7F (MMAR 4705) - MCE-family protein [Mycobacterium marinum str. M]	78.71	559	559
fig|6666666.28482.peg.694	R006783	(gi:118463631) mce7F (MAV 0953) - mce-family protein mce1f [Mycobacterium avium str. 104]	67.88	559	559
fig|6666666.28482.peg.694	R006782	(gi:41406863) mce7F (MAP0765) - hypothetical protein [Mycobacterium avium str. K-10 (subsp. paratuberculosis)]	67.76	559	559
fig|6666666.28482.peg.694	R006826	(gi:41408292) mce8F (MAP2194) - hypothetical protein [Mycobacterium avium str. K-10 (subsp. paratuberculosis)]	69.72	436	559
fig|6666666.28482.peg.694	R006828	(gi:145225111) mce8F (Mflv 4533) - virulence factor Mce family protein [Mycobacterium gilvum str. PYR-GCK]	65.98	434	559
fig|6666666.28482.peg.694	R006790	(gi:108800632) mce7F (Mmcs 3666) - Mammalian cell entry [Mycobacterium sp. str. MCS]	64.03	441	559
fig|6666666.28482.peg.694	R006789	(gi:119869771) mce7F (Mkms 3739) - virulence factor Mce family protein [Mycobacterium sp. str. KMS]	64.03	441	559

fig|6666666.28482.peg.694	R006788	(gi:126436256) mce7F (Mjls 3679) - virulence factor Mce family protein [Mycobacterium sp. str. JLS]	64.03	441	559
fig|6666666.28482.peg.706	R005289	(gi:41407336) ddrA (MAP1238c) - DrrA [Mycobacterium avium str. K-10 (subsp. paratuberculosis)]	67.54	305	309
fig|6666666.28482.peg.706	R005290	(gi:118466584) ddrA (MAV 3250) - daunorubicin resistance ATP-binding protein DrrA [Mycobacterium avium str. 104]	67.21	305	309
fig|6666666.28482.peg.770	R005764	(gi:41408029) trpD (MAP1931c) - anthranilate phosphoribosyltransferase [Mycobacterium avium str. K-10 (subsp. paratuberculosis)]	86.53	349	360
fig|6666666.28482.peg.770	R005765	(gi:118464588) trpD (MAV 2301) - anthranilate phosphoribosyltransferase [Mycobacterium avium str. 104]	86.82	349	360
fig|6666666.28482.peg.770	R005768	(gi:118469185) trpD (MSMEG 4258) - anthranilate phosphoribosyltransferase [Mycobacterium smegmatis str. MC2 155]	81.3	353	360
fig|6666666.28482.peg.770	R018988	(gi:183983227) trpD (MMAR 3236) - anthranilate phosphoribosyltransferase [Mycobacterium marinum str. M]	84.35	345	360
fig|6666666.28482.peg.770	R005767	(gi:118618834) trpD (MUL 3549) - anthranilate phosphoribosyltransferase TrpD [Mycobacterium ulcerans str. Agy99]	84.06	345	360
fig|6666666.28482.peg.770	R018992	(gi:253798743) trpD (TBMG 01789) - anthranilate phosphoribosyltransferase [Mycobacterium tuberculosis str. KZN 1435]	86.13	346	360
fig|6666666.28482.peg.770	R005759	(gi:15609329) trpD (Rv2192c) - anthranilate phosphoribosyltransferase [Mycobacterium tuberculosis str. H37Rv]	86.13	346	360
fig|6666666.28482.peg.770	R005760	(gi:148662009) trpD (MRA 2208) - anthranilate phosphoribosyltransferase [Mycobacterium tuberculosis str. H37Ra]	86.13	346	360
fig|6666666.28482.peg.770	R013454	(gi:148823399) trpD (TBFG 12220) - anthranilate phosphoribosyltransferase trpD [Mycobacterium tuberculosis str. F11]	86.13	346	360
fig|6666666.28482.peg.770	R018991	(gi:340627197) trpD (MCAN 22141) - putative anthranilate phosphoribosyltransferase TrpD [Mycobacterium canettii str. CIPT 140010059]	86.13	346	360
fig|6666666.28482.peg.770	R018993	(gi:224990567) trpD (JTY 2202) - anthranilate phosphoribosyltransferase [Mycobacterium bovis str. BCG str. Tokyo 172]	86.13	346	360
fig|6666666.28482.peg.770	R005763	(gi:121638073) trpD (BCG 2208c) - putative anthranilate phosphoribosyltransferase TrpD [Mycobacterium bovis str. BCG Pasteur 1173P2]	86.13	346	360

fig|6666666.28482.peg.770	R005762	(gi:31793371) trpD (Mb2215c) - anthranilate phosphoribosyltransferase [Mycobacterium bovis str. AF2122/97]	86.13	346	360
fig|6666666.28482.peg.770	R018990	(gi:339632220) trpD (MAF 22030) - putative anthranilate phosphoribosyltransferase TrpD [Mycobacterium africanum str. GM041182]	86.13	346	360
fig|6666666.28482.peg.770	R005761	(gi:15841683) trpD (MT2248) - anthranilate phosphoribosyltransferase [Mycobacterium tuberculosis str. CDC1551]	86.13	346	360
fig|6666666.28482.peg.770	R005766	(gi:15827406) trpD (ML0883) - anthranilate phosphoribosyltransferase [Mycobacterium leprae str. TN]	79.51	360	360
fig|6666666.28482.peg.770	R018994	(gi:221229883) trpD (MLBr 00883) - anthranilate phosphoribosyltransferase [Mycobacterium leprae str. Br4923]	79.51	360	360
fig|6666666.28482.peg.770	R005770	(gi:120404527) trpD (Mvan 3556) - anthranilate phosphoribosyltransferase [Mycobacterium vanbaalenii str. PYR-1]	79.72	359	360
fig|6666666.28482.peg.770	R005771	(gi:126435879) trpD (Mjls 3301) - anthranilate phosphoribosyltransferase [Mycobacterium sp. str. JLS]	79.89	348	360
fig|6666666.28482.peg.770	R005773	(gi:108800256) trpD (Mmcs 3290) - anthranilate phosphoribosyltransferase [Mycobacterium sp. str. MCS]	79.89	348	360
fig|6666666.28482.peg.770	R005772	(gi:119869384) trpD (Mkms 3352) - anthranilate phosphoribosyltransferase [Mycobacterium sp. str. KMS]	79.89	348	360
fig|6666666.28482.peg.770	R005769	(gi:145223544) trpD (Mflv 2957) - anthranilate phosphoribosyltransferase [Mycobacterium gilvum str. PYR-GCK]	79.14	350	360
fig|6666666.28482.peg.770	R018989	(gi:169629057) trpD (MAB 1970) - anthranilate phosphoribosyltransferase [Mycobacterium abscessus str. ATCC 19977]	76.79	345	360
fig|6666666.28482.peg.907	R005991	(gi:41408511) irtB (MAP2413c) - hypothetical protein [Mycobacterium avium str. K-10 (subsp. paratuberculosis)]	83.16	576	582
fig|6666666.28482.peg.907	R005992	(gi:118467076) irtB (MAV 1567) - ABC transporter, ATP-binding protein [Mycobacterium avium str. 104]	82.64	576	582
fig|6666666.28482.peg.907	R019135	(gi:183984007) irtB (MMAR 4036) - transmembrane ATP-binding protein ABC transporter [Mycobacterium marinum str. M]	78.3	576	582
fig|6666666.28482.peg.907	R005993	(gi:118619112) irtB (MUL 3901) - transmembrane ATP-binding protein ABC transporter [Mycobacterium ulcerans str. Agy99]	78.12	576	582

fig|6666666.28482.peg.907	R019139	(gi:253799603) irtB (TBMG 02629) - drugs-transport transmembrane ATP-binding protein ABC transporter [Mycobacterium tuberculosis str. KZN 1435]	78.3	576	582
fig|6666666.28482.peg.907	R005986	(gi:15608489) irtB (Rv1349) - PROBABLE DRUGS-TRANSPORT TRANSMEMBRANE ATP-BINDING PROTEIN ABC TRANSPORTER [Mycobacterium tuberculosis str. H37Rv]	78.3	576	582
fig|6666666.28482.peg.907	R013471	(gi:148822570) irtB (TBFG 11379) - hypothetical drugs-transport transmembrane ATP-binding protein ABC transporter [Mycobacterium tuberculosis str. F11]	78.3	576	582
fig|6666666.28482.peg.907	R005988	(gi:15840804) irtB (MT1392) - ABC transporter, ATP-binding protein [Mycobacterium tuberculosis str. CDC1551]	78.3	576	582
fig|6666666.28482.peg.907	R019138	(gi:340626365) irtB (MCAN 13671) - putative drugs-transport transmembrane ATP-binding protein ABC transporter [Mycobacterium canettii str. CIPT 140010059]	78.3	576	582
fig|6666666.28482.peg.907	R019140	(gi:224989754) irtB (JTY 1385) - putative drugs-transport transmembrane ATP-binding protein ABC transporter [Mycobacterium bovis str. BCG str. Tokyo 172]	78.3	576	582
fig|6666666.28482.peg.907	R005990	(gi:121637280) irtB (BCG 1411) - putative drugs-transport transmembrane ATP-binding protein ABC transporter [Mycobacterium bovis str. BCG Pasteur 1173P2]	78.3	576	582
fig|6666666.28482.peg.907	R005989	(gi:31792545) irtB (Mb1384) - PROBABLE DRUGS-TRANSPORT TRANSMEMBRANE ATP-BINDING PROTEIN ABC TRANSPORTER [Mycobacterium bovis str. AF2122/97]	78.3	576	582
fig|6666666.28482.peg.907	R019137	(gi:339631418) irtB (MAF 13730) - putative drugs-transport transmembrane ATP-binding protein ABC transporter [Mycobacterium africanum str. GM041182]	78.3	576	582
fig|6666666.28482.peg.907	R019141	(gi:333990176) irtB (JDM601 1536) - transmembrane ATP-binding protein ABC transporter [Mycobacterium sp. str. JDM601]	74.91	582	582
fig|6666666.28482.peg.907	R005994	(gi:118469265) irtB (MSMEG 6553) - ABC transporter ATP-binding protein [Mycobacterium smegmatis str. MC2 155]	71.53	576	582
fig|6666666.28482.peg.908	R005982	(gi:41408512) irtA (MAP2414c) - hypothetical protein [Mycobacterium avium str. K-10 (subsp. paratuberculosis)]	84.23	856	856

fig|6666666.28482.peg.908	R005983	(gi:118462514) irtA (MAV 1566) - putative ABC transporter ATP-binding protein [Mycobacterium avium str. 104]	84.47	849	856
fig|6666666.28482.peg.908	R005984	(gi:118619113) irtA (MUL 3902) - transmembrane ATP-binding protein ABC transporter [Mycobacterium ulcerans str. Agy99]	79.09	856	856
fig|6666666.28482.peg.908	R019128	(gi:183984008) irtA (MMAR 4037) - transmembrane ATP-binding protein ABC transporter [Mycobacterium marinum str. M]	79.09	856	856
fig|6666666.28482.peg.908	R019132	(gi:253799604) irtA (TBMG 02630) - drugs-transport transmembrane ATP-binding protein ABC transporter [Mycobacterium tuberculosis str. KZN 1435]	82.27	856	856
fig|6666666.28482.peg.908	R005977	(gi:15608488) irtA (Rv1348) - PROBABLE DRUGS-TRANSPORT TRANSMEMBRANE ATP-BINDING PROTEIN ABC TRANSPORTER [Mycobacterium tuberculosis str. H37Rv]	82.27	856	856
fig|6666666.28482.peg.908	R013470	(gi:148822569) irtA (TBFG 11378) - hypothetical drugs-transport transmembrane ATP-binding protein ABC transporter [Mycobacterium tuberculosis str. F11]	82.27	856	856
fig|6666666.28482.peg.908	R005979	(gi:15840803) irtA (MT1390) - ABC transporter, ATP-binding protein [Mycobacterium tuberculosis str. CDC1551]	82.27	856	856
fig|6666666.28482.peg.908	R019133	(gi:224989753) irtA (JTY 1384) - putative drugs-transport transmembrane ATP-binding protein ABC transporter [Mycobacterium bovis str. BCG str. Tokyo 172]	82.27	856	856
fig|6666666.28482.peg.908	R005981	(gi:121637279) irtA (BCG 1410) - putative drugs-transport transmembrane ATP-binding protein ABC transporter [Mycobacterium bovis str. BCG Pasteur 1173P2]	82.27	856	856
fig|6666666.28482.peg.908	R005980	(gi:31792544) irtA (Mb1383) - PROBABLE DRUGS-TRANSPORT TRANSMEMBRANE ATP-BINDING PROTEIN ABC TRANSPORTER [Mycobacterium bovis str. AF2122/97]	82.27	856	856
fig|6666666.28482.peg.908	R019131	(gi:340626364) irtA (MCAN 13661) - putative drugs-transport transmembrane ATP-binding protein ABC transporter [Mycobacterium canettii str. CIPT 140010059]	82.16	856	856
fig|6666666.28482.peg.908	R019130	(gi:339631417) irtA (MAF 13720) - putative drugs-transport transmembrane ATP-binding protein ABC transporter [Mycobacterium africanum str. GM041182]	82.16	856	856

fig|6666666.28482.peg.908	R005985	(gi:118470205) irtA (MSMEG 6554) - hypothetical ABC transporter ATP-binding protein [Mycobacterium smegmatis str. MC2 155]	74.01	856	856
fig|6666666.28482.peg.908	R019134	(gi:333990175) irtA (JDM601 1535) - transmembrane ATP-binding protein ABC transporter [Mycobacterium sp. str. JDM601]	78.05	841	856
fig|6666666.28482.peg.908	R019129	(gi:169629348) irtA (MAB 2262c) - ABC transporter ATP-binding protein [Mycobacterium abscessus str. ATCC 19977]	64.81	852	856
fig|6666666.28482.peg.966	R005795	(gi:118464128) lysA (MAV 1508) - diaminopimelate decarboxylase [Mycobacterium avium str. 104]	88.35	472	472
fig|6666666.28482.peg.966	R005794	(gi:41408567) lysA (MAP2469c) - LysA 2 [Mycobacterium avium str. K-10 (subsp. paratuberculosis)]	88.35	472	472
fig|6666666.28482.peg.966	R019003	(gi:183984075) lysA (MMAR 4104) - diaminopimelate decarboxylase LysA [Mycobacterium marinum str. M]	87.29	472	472
fig|6666666.28482.peg.966	R005797	(gi:118619175) lysA (MUL 3971) - diaminopimelate decarboxylase LysA [Mycobacterium ulcerans str. Agy99]	86.86	472	472
fig|6666666.28482.peg.966	R005796	(gi:15827559) lysA (ML1128) - diaminopimelate decarboxylase [Mycobacterium leprae str. TN]	83.05	472	472
fig|6666666.28482.peg.966	R019009	(gi:221230036) lysA (MLBr 01128) - diaminopimelate decarboxylase [Mycobacterium leprae str. Br4923]	83.05	472	472
fig|6666666.28482.peg.966	R019007	(gi:253799661) lysA (TBMG 02687) - diaminopimelate decarboxylase lysA [Mycobacterium tuberculosis str. KZN 1435]	85.2	446	472
fig|6666666.28482.peg.966	R005789	(gi:15608433) lysA (Rv1293) - PROBABLE DIAMINOPIMELATE DECARBOXYLASE LYSA (DAP DECARBOXYLASE) [Mycobacterium tuberculosis str. H37Rv]	85.2	446	472
fig|6666666.28482.peg.966	R005790	(gi:148661081) lysA (MRA 1301) - diaminopimelate decarboxylase [Mycobacterium tuberculosis str. H37Ra]	85.2	446	472
fig|6666666.28482.peg.966	R013456	(gi:148822511) lysA (TBFG 11320) - diaminopimelate decarboxylase lysA (dap decarboxylase) [Mycobacterium tuberculosis str. F11]	85.2	446	472
fig|6666666.28482.peg.966	R005791	(gi:15840743) lysA (MT1332) - diaminopimelate decarboxylase [Mycobacterium tuberculosis str. CDC1551]	85.2	446	472
fig|6666666.28482.peg.966	R019006	(gi:340626309) lysA (MCAN 13101) - putative diaminopimelate decarboxylase LYSA [Mycobacterium canettii str. CIPT 140010059]	85.2	446	472
fig|6666666.28482.peg.966	R019008	(gi:224989697) lysA (JTY 1328) - putative diaminopimelate decarboxylase [Mycobacterium bovis str. BCG str. Tokyo 172]	85.2	446	472

fig|6666666.28482.peg.966	R005793	(gi:121637222) lysA (BCG 1353) - putative diaminopimelate decarboxylase lysA [Mycobacterium bovis str. BCG Pasteur 1173P2]	85.2	446	472
fig|6666666.28482.peg.966	R005792	(gi:31792486) lysA (Mb1325) - PROBABLE DIAMINOPIMELATE DECARBOXYLASE LYSA (DAP DECARBOXYLASE) [Mycobacterium bovis str. AF2122/97]	85.2	446	472
fig|6666666.28482.peg.966	R019005	(gi:339631362) lysA (MAF 13150) - putative diaminopimelate decarboxylase LYSA (DAP decarboxylase) [Mycobacterium africanum str. GM041182]	85.2	446	472
fig|6666666.28482.peg.966	R005798	(gi:118467936) lysA (MSMEG 4958) - diaminopimelate decarboxylase [Mycobacterium smegmatis str. MC2 155]	80.3	472	472
fig|6666666.28482.peg.966	R005803	(gi:108800861) lysA (Mmcs 3897) - diaminopimelate decarboxylase [Mycobacterium sp. str. MCS]	78.13	471	472
fig|6666666.28482.peg.966	R005802	(gi:119870001) lysA (Mkms 3971) - diaminopimelate decarboxylase [Mycobacterium sp. str. KMS]	78.13	471	472
fig|6666666.28482.peg.966	R005801	(gi:126436459) lysA (Mjls 3883) - diaminopimelate decarboxylase [Mycobacterium sp. str. JLS]	77.92	471	472
fig|6666666.28482.peg.966	R019010	(gi:333989932) lysA (JDM601 1292) - diaminopimelate decarboxylase LysA [Mycobacterium sp. str. JDM601]	79.24	472	472
fig|6666666.28482.peg.966	R005800	(gi:120405303) lysA (Mvan 4349) - diaminopimelate decarboxylase [Mycobacterium vanbaalenii str. PYR-1]	77.28	471	472
fig|6666666.28482.peg.966	R005799	(gi:145222885) lysA (Mflv 2297) - diaminopimelate decarboxylase [Mycobacterium gilvum str. PYR-GCK]	78.18	472	472
fig|6666666.28482.peg.966	R019004	(gi:169628525) lysA (MAB 1434) - diaminopimelate decarboxylase [Mycobacterium abscessus str. ATCC 19977]	71.82	471	472
fig|6666666.28482.peg.1024	R018895	(gi:340625435) pknG (MCAN 04081) - serine/threonine-protein kinase PKNG [Mycobacterium canettii str. CIPT 140010059]	87.2	742	752
fig|6666666.28482.peg.1024	R018896	(gi:253797333) pknG (TBMG 00411) - serine/threonine-protein kinase pknG [Mycobacterium tuberculosis str. KZN 1435]	87.47	742	752
fig|6666666.28482.peg.1024	R005495	(gi:15607551) pknG (Rv0410c) - SERINE/THREONINE-PROTEIN KINASE PKNG (PROTEIN KINASE G) (STPK G) [Mycobacterium tuberculosis str. H37Rv]	87.47	742	752
fig|6666666.28482.peg.1024	R005496	(gi:148660175) pknG (MRA 0416) - serine/threonine protein kinase [Mycobacterium tuberculosis str. H37Ra]	87.47	742	752

fig|6666666.28482.peg.1024	R013430	(gi:148821606) pknG (TBFG 10415) - serine/threonine-protein kinase pknG [Mycobacterium tuberculosis str. F11]	87.47	742	752
fig|6666666.28482.peg.1024	R018897	(gi:224988796) pknG (JTY 0419) - serine/threonine-protein kinase [Mycobacterium bovis str. BCG str. Tokyo 172]	87.47	742	752
fig|6666666.28482.peg.1024	R005499	(gi:121636324) pknG (BCG 0449c) - Serine/threonine-protein kinase pknG [Mycobacterium bovis str. BCG Pasteur 1173P2]	87.47	742	752
fig|6666666.28482.peg.1024	R005498	(gi:31791588) pknG (Mb0418c) - SERINE/THREONINE-PROTEIN KINASE PKNG (PROTEIN KINASE G) (STPK G) [Mycobacterium bovis str. AF2122/97]	87.47	742	752
fig|6666666.28482.peg.1024	R018894	(gi:339630479) pknG (MAF 04120) - serine/threonine-protein kinase PknG (protein kinase G) (STPK G) [Mycobacterium africanum str. GM041182]	87.33	742	752
fig|6666666.28482.peg.1024	R005497	(gi:15839796) pknG (MT0423) - serine/threonine protein kinase [Mycobacterium tuberculosis str. CDC1551]	87.47	742	752
fig|6666666.28482.peg.1024	R005500	(gi:41409991) pknG (MAP3893c) - PknG [Mycobacterium avium str. K-10 (subsp. paratuberculosis)]	88.09	737	752
fig|6666666.28482.peg.1024	R005501	(gi:118463342) pknG (MAV 4751) - serine/threonine protein kinase PknG [Mycobacterium avium str. 104]	87.28	742	752
fig|6666666.28482.peg.1024	R005502	(gi:15827075) pknG (ML0304) - putative serine-threonine protein kinase [Mycobacterium leprae str. TN]	83.59	752	752
fig|6666666.28482.peg.1024	R018898	(gi:221229553) pknG (MLBr 00304) - putative serine-threonine protein kinase [Mycobacterium leprae str. Br4923]	83.59	752	752
fig|6666666.28482.peg.1024	R018892	(gi:183980736) pknG (MMAR 0713) - serine/threonine-protein kinase PknG [Mycobacterium marinum str. M]	85.58	742	752
fig|6666666.28482.peg.1024	R005503	(gi:118618244) pknG (MUL 2810) - serine/threonine-protein kinase PknG [Mycobacterium ulcerans str. Agy99]	85.46	736	752
fig|6666666.28482.peg.1024	R005506	(gi:120401718) pknG (Mvan 0703) - protein kinase [Mycobacterium vanbaalenii str. PYR-1]	78.44	752	752
fig|6666666.28482.peg.1024	R005504	(gi:118468912) pknG (MSMEG 0786) - serine/threonine protein kinase [Mycobacterium smegmatis str. MC2 155]	79.24	737	752
fig|6666666.28482.peg.1024	R005507	(gi:126433142) pknG (Mjls 0530) - protein kinase [Mycobacterium sp. str. JLS]	77.41	750	752
fig|6666666.28482.peg.1024	R005509	(gi:108797520) pknG (Mmcs 0540) - serine/threonine protein kinase [Mycobacterium sp. str. MCS]	77.28	750	752

fig|6666666.28482.peg.1024	R005508	(gi:119866606) pknG (Mkms 0552) - protein kinase [Mycobacterium sp. str. KMS]	77.28	750	752
fig|6666666.28482.peg.1024	R005505	(gi:145220807) pknG (Mflv 0202) - protein kinase [Mycobacterium gilvum str. PYR-GCK]	80.14	737	752
fig|6666666.28482.peg.1024	R018899	(gi:333989021) pknG (JDM601 0381) - serine/threonine-protein kinase [Mycobacterium sp. str. JDM601]	75.56	751	752
fig|6666666.28482.peg.1024	R018893	(gi:169631302) pknG (MAB 4224) - serine/threonine-protein kinase [Mycobacterium abscessus str. ATCC 19977]	69.18	748	752
fig|6666666.28482.peg.1222	R005170	(gi:15840045) mma4 (MT0670) - methoxy mycolic acid synthase 4 [Mycobacterium tuberculosis str. CDC1551]	78.95	304	318
fig|6666666.28482.peg.1222	R018721	(gi:183980997) mma4 (MMAR 0977) - methoxy mycolic acid synthase 4, MmaA4 [Mycobacterium marinum str. M]	80.87	298	318
fig|6666666.28482.peg.1222	R005168	(gi:15607782) mma4 (Rv0642c) - METHOXY MYCOLIC ACID SYNTHASE 4 MMAA4 (METHYL MYCOLIC ACID SYNTHASE 4) (MMA4) (HYDROXY MYCOLIC ACID SYNTHASE) [Mycobacterium tuberculosis str. H37Rv]	80	300	318
fig|6666666.28482.peg.1222	R005169	(gi:148660416) mma4 (MRA 0653) - methoxy mycolic acid synthase 4 [Mycobacterium tuberculosis str. H37Ra]	80	300	318
fig|6666666.28482.peg.1222	R018723	(gi:340625661) mma4 (MCAN 06411) - methoxy mycolic acid synthase 4 MMAA4 [Mycobacterium canettii str. CIPT 140010059]	80	300	318
fig|6666666.28482.peg.1222	R018724	(gi:253797582) mma4 (TBMG 00653) - methoxy mycolic acid synthase 4 mmaA4 [Mycobacterium tuberculosis str. KZN 1435]	78.95	304	318
fig|6666666.28482.peg.1222	R005173	(gi:41410214) mma4 (MAP4116c) - MmaA4 [Mycobacterium avium str. K-10 (subsp. paratuberculosis)]	80.47	297	318
fig|6666666.28482.peg.1222	R018725	(gi:224989035) mma4 (JTY 0661) - methoxy mycolic acid synthase 4 [Mycobacterium bovis str. BCG str. Tokyo 172]	79.67	300	318
fig|6666666.28482.peg.1222	R005172	(gi:121636563) mma4 (BCG 0691c) - Methoxy mycolic acid synthase 4 mmaA4 [Mycobacterium bovis str. BCG Pasteur 1173P2]	79.67	300	318
fig|6666666.28482.peg.1222	R005171	(gi:31791826) mma4 (Mb0661c) - METHOXY MYCOLIC ACID SYNTHASE 4 MMAA4 (METHYL MYCOLIC ACID SYNTHASE 4) (MMA4) (HYDROXY MYCOLIC ACID SYNTHASE) [Mycobacterium bovis str. AF2122/97]	79.67	300	318
fig|6666666.28482.peg.1222	R018722	(gi:339630712) mma4 (MAF 06510) - methoxy mycolic acid synthase 4 MMAA4 (methyl mycolic acid synthase 4) [Mycobacterium africanum str. GM041182]	79.67	300	318

fig|6666666.28482.peg.1222	R005174	(gi:118466420) mma4 (MAV 4517) - cyclopropane-fatty-acyl-phospholipid synthase 1 [Mycobacterium avium str. 104]	80.13	297	318
fig|6666666.28482.peg.1222	R013399	(gi:148821846) mma4 (TBFG 10655) - methoxy mycolic acid synthase 4 mmaA4 [Mycobacterium tuberculosis str. F11]	80.13	297	318
fig|6666666.28482.peg.1222	R005176	(gi:118616517) mma4 (MUL 0729) - methoxy mycolic acid synthase 4, MmaA4 [Mycobacterium ulcerans str. Agy99]	79.67	300	318
fig|6666666.28482.peg.1222	R005175	(gi:15828017) mma4 (ML1903) - methyl mycolic acid synthase 4 [Mycobacterium leprae str. TN]	78.45	297	318
fig|6666666.28482.peg.1222	R018726	(gi:221230494) mma4 (MLBr 01903) - methyl mycolic acid synthase 4 [Mycobacterium leprae str. Br4923]	78.45	297	318
fig|6666666.28482.peg.1222	R018727	(gi:333989305) mma4 (JDM601 0665) - methoxy mycolic acid synthase [Mycobacterium sp. str. JDM601]	73.05	282	318
fig|6666666.28482.peg.1222	R005181	(gi:108797925) mma4 (Mmcs 0951) - Cyclopropane-fatty-acyl-phospholipid synthase [Mycobacterium sp. str. MCS]	69.15	282	318
fig|6666666.28482.peg.1222	R005180	(gi:119867021) mma4 (Mkms 0969) - Cyclopropane-fatty-acyl-phospholipid synthase [Mycobacterium sp. str. KMS]	69.15	282	318
fig|6666666.28482.peg.1222	R005179	(gi:126433581) mma4 (Mjls 0972) - Cyclopropane-fatty-acyl-phospholipid synthase [Mycobacterium sp. str. JLS]	68.53	284	318
fig|6666666.28482.peg.1222	R005178	(gi:120402251) mma4 (Mvan 1239) - Cyclopropane-fatty-acyl-phospholipid synthase [Mycobacterium vanbaalenii str. PYR-1]	67.14	283	318
fig|6666666.28482.peg.1222	R005177	(gi:145225689) mma4 (Mflv 5113) - Cyclopropane-fatty-acyl-phospholipid synthase [Mycobacterium gilvum str. PYR-GCK]	65.72	283	318
fig|6666666.28482.peg.1300	R006150	(gi:41408655) sigE (MAP2557c) - RNA polymerase sigma-70 factor [Mycobacterium avium str. K-10 (subsp. paratuberculosis)]	82.26	248	260
fig|6666666.28482.peg.1300	R006152	(gi:15827526) sigE (ML1076) - RNA polymerase sigma-70 factor [Mycobacterium leprae str. TN]	81.47	255	260
fig|6666666.28482.peg.1300	R019228	(gi:221230003) sigE (MLBr 01076) - RNA polymerase sigma factor SigE [Mycobacterium leprae str. Br4923]	81.47	255	260
fig|6666666.28482.peg.1300	R019226	(gi:253799735) sigE (TBMG 02761) - RNA polymerase sigma factor SigE [Mycobacterium tuberculosis str. KZN 1435]	83.54	242	260
fig|6666666.28482.peg.1300	R006145	(gi:15608361) sigE (Rv1221) - RNA polymerase sigma-70 factor [Mycobacterium tuberculosis str. H37Rv]	83.54	242	260

fig|6666666.28482.peg.1300	R006146	(gi:148661008) sigE (MRA 1230) - RNA polymerase sigma-70 factor [Mycobacterium tuberculosis str. H37Ra]	83.54	242	260
fig|6666666.28482.peg.1300	R013499	(gi:148822436) sigE (TBFG 11245) - alternative RNA polymerase sigma factor sigE [Mycobacterium tuberculosis str. F11]	83.54	242	260
fig|6666666.28482.peg.1300	R006147	(gi:15840665) sigE (MT1259) - RNA polymerase sigma-70 factor [Mycobacterium tuberculosis str. CDC1551]	83.54	242	260
fig|6666666.28482.peg.1300	R019225	(gi:340626234) sigE (MCAN 12351) - alternative RNA polymerase sigma factor SIGE [Mycobacterium canettii str. CIPT 140010059]	83.54	242	260
fig|6666666.28482.peg.1300	R019227	(gi:224989625) sigE (JTY 1256) - RNA polymerase sigma factor SigE [Mycobacterium bovis str. BCG str. Tokyo 172]	83.54	242	260
fig|6666666.28482.peg.1300	R006149	(gi:121637150) sigE (BCG 1281) - Alternative RNA polymerase sigma factor sigE [Mycobacterium bovis str. BCG Pasteur 1173P2]	83.54	242	260
fig|6666666.28482.peg.1300	R006148	(gi:31792414) sigE (Mb1253) - RNA polymerase sigma-70 factor [Mycobacterium bovis str. AF2122/97]	83.54	242	260
fig|6666666.28482.peg.1300	R019224	(gi:339631288) sigE (MAF 12400) - alternative RNA polymerase sigma factor SIGE [Mycobacterium africanum str. GM041182]	83.54	242	260
fig|6666666.28482.peg.1300	R019222	(gi:183984188) sigE (MMAR 4216) - RNA polymerase sigma factor SigE [Mycobacterium marinum str. M]	84	250	260
fig|6666666.28482.peg.1300	R006153	(gi:118619621) sigE (MUL 4519) - alternative RNA polymerase sigma factor SigE [Mycobacterium ulcerans str. Agy99]	84	250	260
fig|6666666.28482.peg.1300	R006154	(gi:118468250) sigE (MSMEG 5072) - extracytoplasmic function alternative sigma factor [Mycobacterium smegmatis str. MC2 155]	77.73	235	260
fig|6666666.28482.peg.1300	R006159	(gi:108800958) sigE (Mmcs 3994) - RNA polymerase, sigma-24 subunit, ECF subfamily [Mycobacterium sp. str. MCS]	86.11	212	260
fig|6666666.28482.peg.1300	R006158	(gi:119870098) sigE (Mkms 4068) - RNA polymerase, sigma-24 subunit, ECF subfamily [Mycobacterium sp. str. KMS]	86.11	212	260
fig|6666666.28482.peg.1300	R006157	(gi:126436583) sigE (Mjls 4008) - RNA polymerase, sigma-24 subunit, ECF subfamily [Mycobacterium sp. str. JLS]	89.45	199	260
fig|6666666.28482.peg.1300	R006155	(gi:145222788) sigE (Mflv 2200) - RNA polymerase, sigma-24 subunit, ECF subfamily [Mycobacterium gilvum str. PYR-GCK]	75	239	260
fig|6666666.28482.peg.1300	R006156	(gi:120405448) sigE (Mvan 4496) - RNA polymerase, sigma-24 subunit, ECF subfamily [Mycobacterium vanbaalenii str. PYR-1]	89	200	260

fig|6666666.28482.peg.1300	R019229	(gi:333989835) sigE (JDM601 1195) - RNA polymerase sigma factor SigE [Mycobacterium sp. str. JDM601]	81.9	217	260
fig|6666666.28482.peg.1300	R019223	(gi:169628453) sigE (MAB 1362) - alternative RNA polymerase sigma factor [Mycobacterium abscessus str. ATCC 19977]	69.23	244	260
fig|6666666.28482.peg.1316	R014041	(gi:118464008) esxM (MAV 2922) - hypothetical protein [Mycobacterium avium str. 104]	97.96	98	98
fig|6666666.28482.peg.1316	R019810	(gi:340626804) esxM (MCAN 18101) - ESAT-6 like protein ESXM [Mycobacterium canettii str. CIPT 140010059]	87.76	98	98
fig|6666666.28482.peg.1316	R019812	(gi:224990177) esxM (JTY 1808) - hypothetical protein [Mycobacterium bovis str. BCG str. Tokyo 172]	87.76	98	98
fig|6666666.28482.peg.1316	R014040	(gi:121637693) esxM (BCG 1824) - hypothetical protein [Mycobacterium bovis str. BCG Pasteur 1173P2]	87.76	98	98
fig|6666666.28482.peg.1316	R014039	(gi:31792980) esxM (Mb1820) - hypothetical protein [Mycobacterium bovis str. AF2122/97]	87.76	98	98
fig|6666666.28482.peg.1316	R019809	(gi:339631846) esxM (MAF 18140) - ESAT-6 like protein ESXM [Mycobacterium africanum str. GM041182]	87.76	98	98
fig|6666666.28482.peg.1316	R019811	(gi:253799760) esxM (TBMG 02785) - esat-6 like protein esxK [Mycobacterium tuberculosis str. KZN 1435]	86.73	98	98
fig|6666666.28482.peg.1316	R019808	(gi:183982681) esxM (MMAR 2674) - EsaT-6 like protein EsxM [Mycobacterium marinum str. M]	86.46	96	98
fig|6666666.28482.peg.1316	R014042	(gi:118618466) esxM (MUL 3083) - EsaT-6 like protein EsxM [Mycobacterium ulcerans str. Agy99]	84.69	96	98
fig|6666666.28482.peg.1318	R014041	(gi:118464008) esxM (MAV 2922) - hypothetical protein [Mycobacterium avium str. 104]	84.95	93	93
fig|6666666.28482.peg.1318	R019811	(gi:253799760) esxM (TBMG 02785) - esat-6 like protein esxK [Mycobacterium tuberculosis str. KZN 1435]	86.02	93	93
fig|6666666.28482.peg.1318	R019810	(gi:340626804) esxM (MCAN 18101) - ESAT-6 like protein ESXM [Mycobacterium canettii str. CIPT 140010059]	86.02	93	93
fig|6666666.28482.peg.1318	R019812	(gi:224990177) esxM (JTY 1808) - hypothetical protein [Mycobacterium bovis str. BCG str. Tokyo 172]	86.02	93	93
fig|6666666.28482.peg.1318	R014040	(gi:121637693) esxM (BCG 1824) - hypothetical protein [Mycobacterium bovis str. BCG Pasteur 1173P2]	86.02	93	93

fig|6666666.28482.peg.1318	R014039	(gi:31792980) esxM (Mb1820) - hypothetical protein [Mycobacterium bovis str. AF2122/97]	86.02	93	93
fig|6666666.28482.peg.1318	R019809	(gi:339631846) esxM (MAF 18140) - ESAT-6 like protein ESXM [Mycobacterium africanum str. GM041182]	86.02	93	93
fig|6666666.28482.peg.1318	R019808	(gi:183982681) esxM (MMAR 2674) - EsaT-6 like protein EsxM [Mycobacterium marinum str. M]	82.42	91	93
fig|6666666.28482.peg.1318	R014042	(gi:118618466) esxM (MUL 3083) - EsaT-6 like protein EsxM [Mycobacterium ulcerans str. Agy99]	80.65	91	93
fig|6666666.28482.peg.1340	R019862	(gi:183982677) PE18 (MMAR 2670) - PE family protein, PE19 1 [Mycobacterium marinum str. M]	63.22	87	99
fig|6666666.28482.peg.1340	R018659	(gi:118618471) PE18 (MUL 3088) - PE family protein [Mycobacterium ulcerans str. Agy99]	62.07	87	99
fig|6666666.28482.peg.1340	R019865	(gi:253799172) PE18 (TBMG 02209) - PE family protein [Mycobacterium tuberculosis str. KZN 1435]	63.22	87	99
fig|6666666.28482.peg.1340	R018654	(gi:57116910) PE18 (Rv1788) - PE family protein [Mycobacterium tuberculosis str. H37Rv]	63.22	87	99
fig|6666666.28482.peg.1340	R018655	(gi:148661595) PE18 (MRA 1802) - PE family protein [Mycobacterium tuberculosis str. H37Ra]	63.22	87	99
fig|6666666.28482.peg.1340	R018653	(gi:148823003) PE18 (TBFG 11818) - PE family protein [Mycobacterium tuberculosis str. F11]	63.22	87	99
fig|6666666.28482.peg.1340	R018656	(gi:15841256) PE18 (MT1837) - PE family protein [Mycobacterium tuberculosis str. CDC1551]	63.22	87	99
fig|6666666.28482.peg.1340	R019864	(gi:340626800) PE18 (MCAN 18061) - PE family protein [Mycobacterium canettii str. CIPT 140010059]	63.22	87	99
fig|6666666.28482.peg.1340	R019866	(gi:224990173) PE18 (JTY 1804) - PE family protein [Mycobacterium bovis str. BCG str. Tokyo 172]	63.22	87	99
fig|6666666.28482.peg.1340	R018658	(gi:121637689) PE18 (BCG 1820) - PE family protein [Mycobacterium bovis str. BCG Pasteur 1173P2]	63.22	87	99
fig|6666666.28482.peg.1340	R018657	(gi:31792976) PE18 (Mb1816) - PE family protein [Mycobacterium bovis str. AF2122/97]	63.22	87	99
fig|6666666.28482.peg.1340	R019863	(gi:339631842) PE18 (MAF 18100) - PE family protein [Mycobacterium africanum str. GM041182]	63.22	87	99

fig|6666666.28482.peg.1340	R018680	(gi:41407605) PE19 (MAP1507) - hypothetical protein [Mycobacterium avium str. K-10 (subsp. paratuberculosis)]	63.22	87	99
fig|6666666.28482.peg.1340	R019877	(gi:183982680) PE19 (MMAR 2673) - PE family protein, PE19 [Mycobacterium marinum str. M]	64.37	87	99
fig|6666666.28482.peg.1340	R018675	(gi:57116913) PE19 (Rv1791) - PE family protein [Mycobacterium tuberculosis str. H37Rv]	64.37	87	99
fig|6666666.28482.peg.1340	R018676	(gi:148661598) PE19 (MRA 1805) - PE family protein [Mycobacterium tuberculosis str. H37Ra]	64.37	87	99
fig|6666666.28482.peg.1340	R019879	(gi:340626803) PE19 (MCAN 18091) - PE family protein [Mycobacterium canettii str. CIPT 140010059]	64.37	87	99
fig|6666666.28482.peg.1340	R019881	(gi:224990176) PE19 (JTY 1807) - PE family protein [Mycobacterium bovis str. BCG str. Tokyo 172]	64.37	87	99
fig|6666666.28482.peg.1340	R018679	(gi:121637692) PE19 (BCG 1823) - PE family protein [Mycobacterium bovis str. BCG Pasteur 1173P2]	64.37	87	99
fig|6666666.28482.peg.1340	R018678	(gi:31792979) PE19 (Mb1819) - PE family protein [Mycobacterium bovis str. AF2122/97]	64.37	87	99
fig|6666666.28482.peg.1340	R019878	(gi:339631845) PE19 (MAF 18130) - PE family protein [Mycobacterium africanum str. GM041182]	64.37	87	99
fig|6666666.28482.peg.1340	R019880	(gi:253799168) PE19 (TBMG 02205) - PE family protein [Mycobacterium tuberculosis str. KZN 1435]	64.37	87	99
fig|6666666.28482.peg.1340	R018674	(gi:148823006) PE19 (TBFG 11821) - PE family protein [Mycobacterium tuberculosis str. F11]	64.37	87	99
fig|6666666.28482.peg.1340	R018677	(gi:15841261) PE19 (MT1840) - PE family protein [Mycobacterium tuberculosis str. CDC1551]	64.37	87	99
fig|6666666.28482.peg.1340	R019867	(gi:333990534) PE18 (JDM601 1894) - PE-PGRS family protein [Mycobacterium sp. str. JDM601]	65.52	87	99
fig|6666666.28482.peg.1555	R005780	(gi:118465097) leuD (MAV 3837) - 3-isopropylmalate dehydratase, small subunit [Mycobacterium avium str. 104]	92.93	198	198
fig|6666666.28482.peg.1555	R005779	(gi:41409123) leuD (MAP3025c) - isopropylmalate isomerase small subunit [Mycobacterium avium str. K-10 (subsp. paratuberculosis)]	92.42	198	198
fig|6666666.28482.peg.1555	R005782	(gi:118617555) leuD (MUL 1969) - 3-isopropylmalate dehydratase (small subunit) LeuD [Mycobacterium ulcerans str. Agy99]	91.92	198	198

fig|6666666.28482.peg.1555	R018995	(gi:183981740) leuD (MMAR 1727) - isopropylmalate isomerase small subunit [Mycobacterium marinum str. M]	91.92	198	198
fig|6666666.28482.peg.1555	R018999	(gi:253797920) leuD (TBMG 00982) - isopropylmalate isomerase small subunit [Mycobacterium tuberculosis str. KZN 1435]	91.37	197	198
fig|6666666.28482.peg.1555	R005774	(gi:15610124) leuD (Rv2987c) - isopropylmalate isomerase small subunit [Mycobacterium tuberculosis str. H37Rv]	91.37	197	198
fig|6666666.28482.peg.1555	R005775	(gi:148662837) leuD (MRA 3016) - isopropylmalate isomerase small subunit [Mycobacterium tuberculosis str. H37Ra]	91.37	197	198
fig|6666666.28482.peg.1555	R013455	(gi:148824177) leuD (TBFG 13002) - 3-isopropylmalate dehydratase small subunit leuD [Mycobacterium tuberculosis str. F11]	91.37	197	198
fig|6666666.28482.peg.1555	R005776	(gi:15842543) leuD (MT3065) - isopropylmalate isomerase small subunit [Mycobacterium tuberculosis str. CDC1551]	91.37	197	198
fig|6666666.28482.peg.1555	R018998	(gi:340627976) leuD (MCAN 30091) - putative 3-isopropylmalate dehydratase [Mycobacterium canettii str. CIPT 140010059]	91.37	197	198
fig|6666666.28482.peg.1555	R019000	(gi:224991360) leuD (JTY 3003) - isopropylmalate isomerase small subunit [Mycobacterium bovis str. BCG str. Tokyo 172]	91.37	197	198
fig|6666666.28482.peg.1555	R005778	(gi:121638868) leuD (BCG 3008c) - putative 3-isopropylmalate dehydratase smallsubunit leuD [Mycobacterium bovis str. BCG Pasteur 1173P2]	91.37	197	198
fig|6666666.28482.peg.1555	R005777	(gi:31794163) leuD (Mb3011c) - isopropylmalate isomerase small subunit [Mycobacterium bovis str. AF2122/97]	91.37	197	198
fig|6666666.28482.peg.1555	R018997	(gi:339632993) leuD (MAF 29920) - putative 3-isopropylmalate dehydratase (small subunit) LEUD (isopropylmalate isomerase) [Mycobacterium africanum str. GM041182]	91.37	197	198
fig|6666666.28482.peg.1555	R005783	(gi:118470522) leuD (MSMEG 2388) - 3-isopropylmalate dehydratase, small subunit [Mycobacterium smegmatis str. MC2 155]	88.32	197	198
fig|6666666.28482.peg.1555	R005781	(gi:15827895) leuD (ML1684) - isopropylmalate isomerase small subunit [Mycobacterium leprae str. TN]	87.88	198	198
fig|6666666.28482.peg.1555	R019001	(gi:221230372) leuD (MLBr 01684) - isopropylmalate isomerase small subunit [Mycobacterium leprae str. Br4923]	87.88	198	198
fig|6666666.28482.peg.1555	R019002	(gi:333991330) leuD (JDM601 2690) - 3-isopropylmalate dehydratase (small subunit) LeuD [Mycobacterium sp. str. JDM601]	86.36	198	198
fig|6666666.28482.peg.1555	R005785	(gi:120403128) leuD (Mvan 2136) - 3-isopropylmalate dehydratase, small subunit [Mycobacterium vanbaalenii str. PYR-1]	87.31	197	198

fig|6666666.28482.peg.1555	R005788	(gi:108798890) leuD (Mmcs 1922) - 3-isopropylmalate dehydratase, small subunit [Mycobacterium sp. str. MCS]	86.8	197	198
fig|6666666.28482.peg.1555	R005787	(gi:119868005) leuD (Mkms 1968) - 3-isopropylmalate dehydratase, small subunit [Mycobacterium sp. str. KMS]	86.8	197	198
fig|6666666.28482.peg.1555	R005786	(gi:126434490) leuD (Mjls 1902) - 3-isopropylmalate dehydratase, small subunit [Mycobacterium sp. str. JLS]	86.29	197	198
fig|6666666.28482.peg.1555	R005784	(gi:145224805) leuD (Mflv 4226) - 3-isopropylmalate dehydratase, small subunit [Mycobacterium gilvum str. PYR-GCK]	85.79	197	198
fig|6666666.28482.peg.1555	R018996	(gi:169630374) leuD (MAB 3293c) - isopropylmalate isomerase small subunit [Mycobacterium abscessus str. ATCC 19977]	81.82	198	198
fig|6666666.28482.peg.1617	R019862	(gi:183982677) PE18 (MMAR 2670) - PE family protein, PE19 1 [Mycobacterium marinum str. M]	63.22	87	99
fig|6666666.28482.peg.1617	R018659	(gi:118618471) PE18 (MUL 3088) - PE family protein [Mycobacterium ulcerans str. Agy99]	62.07	87	99
fig|6666666.28482.peg.1617	R019877	(gi:183982680) PE19 (MMAR 2673) - PE family protein, PE19 [Mycobacterium marinum str. M]	68.97	87	99
fig|6666666.28482.peg.1617	R018680	(gi:41407605) PE19 (MAP1507) - hypothetical protein [Mycobacterium avium str. K-10 (subsp. paratuberculosis)]	65.52	87	99
fig|6666666.28482.peg.1617	R019880	(gi:253799168) PE19 (TBMG 02205) - PE family protein [Mycobacterium tuberculosis str. KZN 1435]	68.97	87	99
fig|6666666.28482.peg.1617	R018674	(gi:148823006) PE19 (TBFG 11821) - PE family protein [Mycobacterium tuberculosis str. F11]	68.97	87	99
fig|6666666.28482.peg.1617	R018677	(gi:15841261) PE19 (MT1840) - PE family protein [Mycobacterium tuberculosis str. CDC1551]	68.97	87	99
fig|6666666.28482.peg.1617	R019865	(gi:253799172) PE18 (TBMG 02209) - PE family protein [Mycobacterium tuberculosis str. KZN 1435]	67.82	87	99
fig|6666666.28482.peg.1617	R018675	(gi:57116913) PE19 (Rv1791) - PE family protein [Mycobacterium tuberculosis str. H37Rv]	68.97	87	99
fig|6666666.28482.peg.1617	R018654	(gi:57116910) PE18 (Rv1788) - PE family protein [Mycobacterium tuberculosis str. H37Rv]	67.82	87	99
fig|6666666.28482.peg.1617	R018676	(gi:148661598) PE19 (MRA 1805) - PE family protein [Mycobacterium tuberculosis str. H37Ra]	68.97	87	99

fig|6666666.28482.peg.1617	R018655	(gi:148661595) PE18 (MRA 1802) - PE family protein [Mycobacterium tuberculosis str. H37Ra]	67.82	87	99
fig|6666666.28482.peg.1617	R018653	(gi:148823003) PE18 (TBFG 11818) - PE family protein [Mycobacterium tuberculosis str. F11]	67.82	87	99
fig|6666666.28482.peg.1617	R018656	(gi:15841256) PE18 (MT1837) - PE family protein [Mycobacterium tuberculosis str. CDC1551]	67.82	87	99
fig|6666666.28482.peg.1617	R019879	(gi:340626803) PE19 (MCAN 18091) - PE family protein [Mycobacterium canettii str. CIPT 140010059]	68.97	87	99
fig|6666666.28482.peg.1617	R019864	(gi:340626800) PE18 (MCAN 18061) - PE family protein [Mycobacterium canettii str. CIPT 140010059]	67.82	87	99
fig|6666666.28482.peg.1617	R019881	(gi:224990176) PE19 (JTY 1807) - PE family protein [Mycobacterium bovis str. BCG str. Tokyo 172]	68.97	87	99
fig|6666666.28482.peg.1617	R019866	(gi:224990173) PE18 (JTY 1804) - PE family protein [Mycobacterium bovis str. BCG str. Tokyo 172]	67.82	87	99
fig|6666666.28482.peg.1617	R018679	(gi:121637692) PE19 (BCG 1823) - PE family protein [Mycobacterium bovis str. BCG Pasteur 1173P2]	68.97	87	99
fig|6666666.28482.peg.1617	R018658	(gi:121637689) PE18 (BCG 1820) - PE family protein [Mycobacterium bovis str. BCG Pasteur 1173P2]	67.82	87	99
fig|6666666.28482.peg.1617	R018678	(gi:31792979) PE19 (Mb1819) - PE family protein [Mycobacterium bovis str. AF2122/97]	68.97	87	99
fig|6666666.28482.peg.1617	R018657	(gi:31792976) PE18 (Mb1816) - PE family protein [Mycobacterium bovis str. AF2122/97]	67.82	87	99
fig|6666666.28482.peg.1617	R019878	(gi:339631845) PE19 (MAF 18130) - PE family protein [Mycobacterium africanum str. GM041182]	68.97	87	99
fig|6666666.28482.peg.1617	R019863	(gi:339631842) PE18 (MAF 18100) - PE family protein [Mycobacterium africanum str. GM041182]	67.82	87	99
fig|6666666.28482.peg.1617	R019867	(gi:333990534) PE18 (JDM601 1894) - PE-PGRS family protein [Mycobacterium sp. str. JDM601]	67.82	87	99
fig|6666666.28482.peg.1641	R006179	(gi:41410435) sigM (MAP4337) - RNA polymerase sigma-70 factor [Mycobacterium avium str. K-10 (subsp. paratuberculosis)]	86.74	181	203
fig|6666666.28482.peg.1641	R006180	(gi:118463700) sigM (MAV 5299) - RNA polymerase sigma-70 factor [Mycobacterium avium str. 104]	86.74	181	203

fig|6666666.28482.peg.1641	R019241	(gi:253800961) sigM (TBMG 03959) - RNA polymerase sigma factor SigM [Mycobacterium tuberculosis str. KZN 1435]	85.79	183	203
fig|6666666.28482.peg.1641	R006175	(gi:148663778) sigM (MRA 3950) - RNA polymerase sigma-70 factor [Mycobacterium tuberculosis str. H37Ra]	85.79	183	203
fig|6666666.28482.peg.1641	R013501	(gi:148825119) sigM (TBFG 13946) - alternative RNA polymerase sigma factor sigM [Mycobacterium tuberculosis str. F11]	85.79	183	203
fig|6666666.28482.peg.1641	R019242	(gi:224992317) sigM (JTY 3970) - RNA polymerase sigma factor SigM [Mycobacterium bovis str. BCG str. Tokyo 172]	85.79	183	203
fig|6666666.28482.peg.1641	R006178	(gi:121639822) sigM (BCG 3968) - putative alternative RNA polymerase sigma factor sigMa [Mycobacterium bovis str. BCG Pasteur 1173P2]	85.79	183	203
fig|6666666.28482.peg.1641	R006177	(gi:31795084) sigM (Mb3941) - RNA polymerase sigma-70 factor [Mycobacterium bovis str. AF2122/97]	85.25	183	203
fig|6666666.28482.peg.1641	R006176	(gi:15843544) sigM (MT4030) - RNA polymerase sigma-70 factor [Mycobacterium tuberculosis str. CDC1551]	85.25	183	203
fig|6666666.28482.peg.1641	R019240	(gi:340628881) sigM (MCAN 39311) - putative alternative RNA polymerase sigma factor SIGM [Mycobacterium canettii str. CIPT 140010059]	85.25	183	203
fig|6666666.28482.peg.1641	R019237	(gi:183985445) sigM (MMAR 5475) - RNA polymerase sigma factor SigM [Mycobacterium marinum str. M]	72.25	186	203
fig|6666666.28482.peg.1641	R006181	(gi:118620066) sigM (MUL 5064) - alternative RNA polymerase sigma factor SigM [Mycobacterium ulcerans str. Agy99]	70.16	186	203
fig|6666666.28482.peg.1641	R006174	(gi:15611047) sigM (Rv3911) - RNA polymerase sigma-70 factor [Mycobacterium tuberculosis str. H37Rv]	74.35	191	203
fig|6666666.28482.peg.1641	R006185	(gi:126438340) sigM (Mjls 5777) - transcriptional regulator, Fis family [Mycobacterium sp. str. JLS]	71.43	181	203
fig|6666666.28482.peg.1641	R006187	(gi:108802360) sigM (Mmcs 5401) - RNA polymerase, sigma-24 subunit, ECF subfamily [Mycobacterium sp. str. MCS]	70.33	181	203
fig|6666666.28482.peg.1641	R006186	(gi:119871513) sigM (Mkms 5490) - transcriptional regulator, Fis family [Mycobacterium sp. str. KMS]	70.33	181	203
fig|6666666.28482.peg.1641	R019243	(gi:333992975) sigM (JDM601 4335) - RNA polymerase sigma factor SigM [Mycobacterium sp. str. JDM601]	74.71	174	203
fig|6666666.28482.peg.1641	R006182	(gi:118469491) sigM (MSMEG 6931) - RNA polymerase sigma-70 factor [Mycobacterium smegmatis str. MC2 155]	65.36	171	203

fig|6666666.28482.peg.1641	R019238	(gi:169632011) sigM (MAB 4938) - alternative RNA polymerase sigma factor SigM [Mycobacterium abscessus str. ATCC 19977]	65.28	192	203
fig|6666666.28482.peg.1641	R006183	(gi:145221435) sigM (Mflv 0841) - transcriptional regulator, Fis family [Mycobacterium gilvum str. PYR-GCK]	67.58	177	203
fig|6666666.28482.peg.1659	R018301	(gi:31795034) Mb3890 - hypothetical protein [Mycobacterium bovis str. AF2122/97]	79.84	243	248
fig|6666666.28482.peg.1684	R006389	(gi:41409706) mce1E (MAP3608) - LprK [Mycobacterium avium str. K-10 (subsp. paratuberculosis)]	88.36	335	335
fig|6666666.28482.peg.1684	R006391	(gi:15828397) mce1E (ML2593) - putative lipoprotein [Mycobacterium leprae str. TN]	84.18	335	335
fig|6666666.28482.peg.1684	R019353	(gi:221230874) mce1E (MLBr 02593) - putative lipoprotein [Mycobacterium leprae str. Br4923]	84.18	335	335
fig|6666666.28482.peg.1684	R019348	(gi:183980447) mce1E (MMAR 0416) - MCE family lipoprotein LprK [Mycobacterium marinum str. M]	81.49	335	335
fig|6666666.28482.peg.1684	R006392	(gi:118616799) mce1E (MUL 1066) - MCE-family lipoprotein LprK (MCE-family lipoprotein Mce1E) [Mycobacterium ulcerans str. Agy99]	80.9	335	335
fig|6666666.28482.peg.1684	R019350	(gi:340625210) mce1E (MCAN 01791) - putative MCE-family lipoprotein LPRK [Mycobacterium canettii str. CIPT 140010059]	77.61	335	335
fig|6666666.28482.peg.1684	R006384	(gi:15607314) mce1E (Rv0173) - POSSIBLE MCE-FAMILY LIPOPROTEIN LPRK (MCE-FAMILY LIPOPROTEIN MCE1E) [Mycobacterium tuberculosis str. H37Rv]	77.31	335	335
fig|6666666.28482.peg.1684	R006385	(gi:148659937) mce1E (MRA 0181) - MCE-family lipoprotein LprK [Mycobacterium tuberculosis str. H37Ra]	77.31	335	335
fig|6666666.28482.peg.1684	R013516	(gi:148821365) mce1E (TBFG 10174) - MCE-family lipoprotein lprK (MCE-family lipoprotein mce1e) [Mycobacterium tuberculosis str. F11]	77.31	335	335
fig|6666666.28482.peg.1684	R019352	(gi:224988558) mce1E (JTY 0179) - putative MCE-family lipoprotein mce1E [Mycobacterium bovis str. BCG str. Tokyo 172]	77.31	335	335
fig|6666666.28482.peg.1684	R006388	(gi:121636085) mce1E (BCG 0210) - putative mce-family lipoprotein lprK (MCE-family lipoprotein mce1E) [Mycobacterium bovis str. BCG Pasteur 1173P2]	77.31	335	335
fig|6666666.28482.peg.1684	R006387	(gi:31791351) mce1E (Mb0179) - POSSIBLE MCE-FAMILY LIPOPROTEIN LPRK (MCE-FAMILY LIPOPROTEIN MCE1E) [Mycobacterium bovis str. AF2122/97]	77.31	335	335

fig|6666666.28482.peg.1684	R019349	(gi:339630254) mce1E (MAF 01740) - putative MCE-family lipoprotein LprK (MCE-family lipoprotein Mce1E) [Mycobacterium africanum str. GM041182]	77.31	335	335
fig|6666666.28482.peg.1684	R019351	(gi:253797095) mce1E (TBMG 00174) - MCE-family lipoprotein mce1E [Mycobacterium tuberculosis str. KZN 1435]	77.31	335	335
fig|6666666.28482.peg.1684	R006386	(gi:15839551) mce1E (MT0182) - virulence factor mce family protein [Mycobacterium tuberculosis str. CDC1551]	77.31	335	335
fig|6666666.28482.peg.1684	R006462	(gi:126432725) mce2E (Mjls 0112) - virulence factor Mce family protein [Mycobacterium sp. str. JLS]	67.47	332	335
fig|6666666.28482.peg.1684	R006464	(gi:108797102) mce2E (Mmcs 0122) - Mammalian cell entry [Mycobacterium sp. str. MCS]	66.87	332	335
fig|6666666.28482.peg.1684	R006463	(gi:119866187) mce2E (Mkms 0131) - virulence factor Mce family protein [Mycobacterium sp. str. KMS]	66.87	332	335
fig|6666666.28482.peg.1684	R019380	(gi:340625623) mce2E (MCAN 05991) - putative MCE-family lipoprotein LPRL [Mycobacterium canettii str. CIPT 140010059]	66.27	332	335
fig|6666666.28482.peg.1684	R019381	(gi:253797528) mce2E (TBMG 00601) - MCE-family lipoprotein mce2E [Mycobacterium tuberculosis str. KZN 1435]	65.66	332	335
fig|6666666.28482.peg.1684	R006453	(gi:15607733) mce2E (Rv0593) - POSSIBLE MCE-FAMILY LIPOPROTEIN LPRL (MCE-FAMILY LIPOPROTEIN MCE2E) [Mycobacterium tuberculosis str. H37Rv]	65.66	332	335
fig|6666666.28482.peg.1684	R006454	(gi:148660363) mce2E (MRA 0601) - MCE-family lipoprotein LprL [Mycobacterium tuberculosis str. H37Ra]	65.66	332	335
fig|6666666.28482.peg.1684	R013522	(gi:148821796) mce2E (TBFG 10605) - MCE-family lipoprotein lprL (MCE-family lipoprotein mce2e) [Mycobacterium tuberculosis str. F11]	65.66	332	335
fig|6666666.28482.peg.1684	R006455	(gi:15839996) mce2E (MT0623) - virulence factor mce family protein [Mycobacterium tuberculosis str. CDC1551]	65.66	332	335
fig|6666666.28482.peg.1684	R019382	(gi:224988983) mce2E (JTY 0608) - putative MCE-family lipoprotein [Mycobacterium bovis str. BCG str. Tokyo 172]	65.66	332	335
fig|6666666.28482.peg.1684	R006457	(gi:121636511) mce2E (BCG 0639) - putative mce-family lipoprotein lprL [Mycobacterium bovis str. BCG Pasteur 1173P2]	65.66	332	335
fig|6666666.28482.peg.1684	R019379	(gi:339630661) mce2E (MAF 06000) - putative MCE-family lipoprotein LPRL (MCE-family lipoprotein MCE2E) [Mycobacterium africanum str. GM041182]	65.66	332	335

fig|6666666.28482.peg.1684	R006393	(gi:118472695) mce1E (MSMEG 0138) - virulence factor Mce family protein [Mycobacterium smegmatis str. MC2 155]	65.36	332	335
fig|6666666.28482.peg.1684	R006458	(gi:41410186) mce2E (MAP4088) - LprL [Mycobacterium avium str. K-10 (subsp. paratuberculosis)]	64.16	332	335
fig|6666666.28482.peg.1684	R006461	(gi:120401166) mce2E (Mvan 0138) - virulence factor Mce family protein [Mycobacterium vanbaalenii str. PYR-1]	63.25	332	335
fig|6666666.28482.peg.1684	R006460	(gi:145221302) mce2E (Mflv 0707) - virulence factor Mce family protein [Mycobacterium gilvum str. PYR-GCK]	63.25	332	335
fig|6666666.28482.peg.1684	R006459	(gi:118462367) mce2E (MAV 4547) - virulence factor Mce family protein [Mycobacterium avium str. 104]	64.46	332	335
fig|6666666.28482.peg.1684	R019354	(gi:333988766) mce1E (JDM601 0126) - MCE family lipoprotein LprK [Mycobacterium sp. str. JDM601]	60.3	335	335
fig|6666666.28482.peg.1685	R006399	(gi:41409707) mce1F (MAP3609) - hypothetical protein [Mycobacterium avium str. K-10 (subsp. paratuberculosis)]	89.15	516	516
fig|6666666.28482.peg.1685	R006401	(gi:15828398) mce1F (ML2594) - putative secreted protein [Mycobacterium leprae str. TN]	81.01	516	516
fig|6666666.28482.peg.1685	R019360	(gi:221230875) mce1F (MLBr 02594) - putative secreted protein [Mycobacterium leprae str. Br4923]	81.01	516	516
fig|6666666.28482.peg.1685	R006402	(gi:118616800) mce1F (MUL 1067) - MCE-family protein Mce1F [Mycobacterium ulcerans str. Agy99]	83.01	516	516
fig|6666666.28482.peg.1685	R019355	(gi:183980448) mce1F (MMAR 0417) - MCE-family protein Mce1F [Mycobacterium marinum str. M]	83.01	516	516
fig|6666666.28482.peg.1685	R019358	(gi:253797096) mce1F (TBMG 00175) - MCE-family protein mce1F [Mycobacterium tuberculosis str. KZN 1435]	78.25	515	516
fig|6666666.28482.peg.1685	R013517	(gi:148821366) mce1F (TBFG 10175) - MCE-family protein mce1F [Mycobacterium tuberculosis str. F11]	78.25	515	516
fig|6666666.28482.peg.1685	R006396	(gi:15839552) mce1F (MT0183) - virulence factor mce family protein [Mycobacterium tuberculosis str. CDC1551]	78.25	515	516
fig|6666666.28482.peg.1685	R019359	(gi:224988559) mce1F (JTY 0180) - MCE-family protein [Mycobacterium bovis str. BCG str. Tokyo 172]	78.25	515	516
fig|6666666.28482.peg.1685	R006398	(gi:121636086) mce1F (BCG 0211) - MCE-family protein mce1F [Mycobacterium bovis str. BCG Pasteur 1173P2]	78.25	515	516

fig|6666666.28482.peg.1685	R006397	(gi:31791352) mce1F (Mb0180) - MCE-FAMILY PROTEIN MCE1F [Mycobacterium bovis str. AF2122/97]	78.25	515	516
fig|6666666.28482.peg.1685	R019356	(gi:339630255) mce1F (MAF 01750) - MCE-family protein MCE1F [Mycobacterium africanum str. GM041182]	78.25	515	516
fig|6666666.28482.peg.1685	R019357	(gi:340625211) mce1F (MCAN 01801) - MCE-family protein MCE1F [Mycobacterium canettii str. CIPT 140010059]	77.86	515	516
fig|6666666.28482.peg.1685	R006394	(gi:15607315) mce1F (Rv0174) - MCE-FAMILY PROTEIN MCE1F [Mycobacterium tuberculosis str. H37Rv]	78.06	515	516
fig|6666666.28482.peg.1685	R006395	(gi:148659938) mce1F (MRA 0182) - MCE-family protein Mce1F [Mycobacterium tuberculosis str. H37Ra]	78.06	515	516
fig|6666666.28482.peg.1685	R006474	(gi:126432726) mce2F (Mjls 0113) - virulence factor Mce family protein [Mycobacterium sp. str. JLS]	70.13	515	516
fig|6666666.28482.peg.1685	R006476	(gi:108797103) mce2F (Mmcs 0123) - Mammalian cell entry [Mycobacterium sp. str. MCS]	69.75	515	516
fig|6666666.28482.peg.1685	R006475	(gi:119866188) mce2F (Mkms 0132) - virulence factor Mce family protein [Mycobacterium sp. str. KMS]	69.75	515	516
fig|6666666.28482.peg.1685	R019385	(gi:253797529) mce2F (TBMG 00602) - MCE-family protein mce2F [Mycobacterium tuberculosis str. KZN 1435]	67.89	516	516
fig|6666666.28482.peg.1685	R006465	(gi:15607734) mce2F (Rv0594) - MCE-FAMILY PROTEIN MCE2F [Mycobacterium tuberculosis str. H37Rv]	67.89	516	516
fig|6666666.28482.peg.1685	R006466	(gi:148660364) mce2F (MRA 0602) - MCE-family protein Mce2F [Mycobacterium tuberculosis str. H37Ra]	67.89	516	516
fig|6666666.28482.peg.1685	R013523	(gi:148821797) mce2F (TBFG 10606) - MCE-family protein mce2F [Mycobacterium tuberculosis str. F11]	67.89	516	516
fig|6666666.28482.peg.1685	R019386	(gi:224988984) mce2F (JTY 0609) - MCE-family protein [Mycobacterium bovis str. BCG str. Tokyo 172]	67.89	516	516
fig|6666666.28482.peg.1685	R006469	(gi:121636512) mce2F (BCG 0640) - mce-family protein mce2F [Mycobacterium bovis str. BCG Pasteur 1173P2]	67.89	516	516
fig|6666666.28482.peg.1685	R006468	(gi:31791776) mce2F (Mb0610) - MCE-FAMILY PROTEIN MCE2F [Mycobacterium bovis str. AF2122/97]	67.89	516	516
fig|6666666.28482.peg.1685	R019383	(gi:339630662) mce2F (MAF 06010) - MCE-family protein MCE2F [Mycobacterium africanum str. GM041182]	67.89	516	516

fig|6666666.28482.peg.1685	R006467	(gi:15839997) mce2F (MT0624) - virulence factor mce family protein [Mycobacterium tuberculosis str. CDC1551]	67.89	516	516
fig|6666666.28482.peg.1685	R006472	(gi:145221301) mce2F (Mflv 0706) - virulence factor Mce family protein [Mycobacterium gilvum str. PYR-GCK]	67.5	516	516
fig|6666666.28482.peg.1685	R019384	(gi:340625624) mce2F (MCAN 06001) - MCE-family protein MCE2F [Mycobacterium canettii str. CIPT 140010059]	67.7	516	516
fig|6666666.28482.peg.1685	R006470	(gi:41410187) mce2F (MAP4089) - hypothetical protein [Mycobacterium avium str. K-10 (subsp. paratuberculosis)]	67.83	515	516
fig|6666666.28482.peg.1685	R006471	(gi:118463921) mce2F (MAV 4546) - mce-family protein mce2f [Mycobacterium avium str. 104]	69.72	491	516
fig|6666666.28482.peg.1685	R006473	(gi:120401167) mce2F (Mvan 0139) - virulence factor Mce family protein [Mycobacterium vanbaalenii str. PYR-1]	66.35	516	516
fig|6666666.28482.peg.1685	R006403	(gi:118473635) mce1F (MSMEG 0139) - mce-family protein mce1f [Mycobacterium smegmatis str. MC2 155]	65.96	515	516
fig|6666666.28482.peg.1685	R019361	(gi:333988767) mce1F (JDM601 0127) - MCE-family protein Mce1F [Mycobacterium sp. str. JDM601]	64.65	514	516
fig|6666666.28482.peg.1685	R006400	(gi:118462429) mce1F (MAV 5010) - mce-family protein mce1f [Mycobacterium avium str. 104]	89.46	313	516
fig|6666666.28482.peg.1928	R005734	(gi:41406712) purC (MAP0614) - phosphoribosylaminoimidazole-succinocarboxamide synthase [Mycobacterium avium str. K-10 (subsp. paratuberculosis)]	91.81	293	297
fig|6666666.28482.peg.1928	R005735	(gi:118466397) purC (MAV 0725) - phosphoribosylaminoimidazole-succinocarboxamide synthase [Mycobacterium avium str. 104]	91.47	293	297
fig|6666666.28482.peg.1928	R018972	(gi:183984877) purC (MMAR 4909) - phosphoribosylaminoimidazole-succinocarboxamide synthase [Mycobacterium marinum str. M]	90.57	297	297
fig|6666666.28482.peg.1928	R005737	(gi:118616338) purC (MUL 0497) - phosphoribosylaminoimidazole-succinocarboxamide synthase, PurC [Mycobacterium ulcerans str. Agy99]	89.56	297	297
fig|6666666.28482.peg.1928	R018976	(gi:253797726) purC (TBMG 00795) - phosphoribosylaminoimidazole-succinocarboxamide synthase [Mycobacterium tuberculosis str. KZN 1435]	88.55	297	297
fig|6666666.28482.peg.1928	R005729	(gi:15607920) purC (Rv0780) - phosphoribosylaminoimidazole-succinocarboxamide synthase [Mycobacterium tuberculosis str. H37Rv]	88.55	297	297
fig|6666666.28482.peg.1928	R005730	(gi:148660557) purC (MRA 0789) - phosphoribosylaminoimidazole-succinocarboxamide synthase [Mycobacterium tuberculosis str. H37Ra]	88.55	297	297

fig|6666666.28482.peg.1928	R013452	(gi:148821985) purC (TBFG 10795) - phosphoribosylaminoimidazole-succinocarboxamide synthase purC [Mycobacterium tuberculosis str. F11]	88.55	297	297
fig|6666666.28482.peg.1928	R005731	(gi:15840195) purC (MT0804) - phosphoribosylaminoimidazole-succinocarboxamide synthase [Mycobacterium tuberculosis str. CDC1551]	88.55	297	297
fig|6666666.28482.peg.1928	R018977	(gi:224989176) purC (JTY 0802) - phosphoribosylaminoimidazole-succinocarboxamide synthase [Mycobacterium bovis str. BCG str. Tokyo 172]	88.55	297	297
fig|6666666.28482.peg.1928	R005733	(gi:121636704) purC (BCG 0832) - Phosphoribosylaminoimidazole-succinocarboxamide synthase purC [Mycobacterium bovis str. BCG Pasteur 1173P2]	88.55	297	297
fig|6666666.28482.peg.1928	R005732	(gi:31791968) purC (Mb0803) - phosphoribosylaminoimidazole-succinocarboxamide synthase [Mycobacterium bovis str. AF2122/97]	88.55	297	297
fig|6666666.28482.peg.1928	R018974	(gi:339630850) purC (MAF 07920) - phosphoribosylaminoimidazole-succinocarboxamide synthase PURC (SAICAR synthetase) [Mycobacterium africanum str. GM041182]	88.55	297	297
fig|6666666.28482.peg.1928	R018975	(gi:340625799) purC (MCAN 07841) - phosphoribosylaminoimidazole-succinocarboxamide synthase PURC [Mycobacterium canettii str. CIPT 140010059]	88.22	297	297
fig|6666666.28482.peg.1928	R018979	(gi:333992221) purC (JDM601 3581) - phosphoribosylaminoimidazole-succinocarboxamide synthase PurC [Mycobacterium sp. str. JDM601]	82.77	296	297
fig|6666666.28482.peg.1928	R005736	(gi:15828193) purC (ML2227) - phosphoribosylaminoimidazole-succinocarboxamide synthase [Mycobacterium leprae str. TN]	83.84	297	297
fig|6666666.28482.peg.1928	R018978	(gi:221230670) purC (MLBr 02227) - phosphoribosylaminoimidazole-succinocarboxamide synthase [Mycobacterium leprae str. Br4923]	83.84	297	297
fig|6666666.28482.peg.1928	R005738	(gi:118471351) purC (MSMEG 5841) - phosphoribosylaminoimidazole-succinocarboxamide synthase [Mycobacterium smegmatis str. MC2 155]	80.54	294	297
fig|6666666.28482.peg.1928	R005741	(gi:126437508) purC (Mjls 4943) - phosphoribosylaminoimidazole-succinocarboxamide synthase [Mycobacterium sp. str. JLS]	79.59	294	297
fig|6666666.28482.peg.1928	R005743	(gi:108801523) purC (Mmcs 4560) - phosphoribosylaminoimidazole-succinocarboxamide synthase [Mycobacterium sp. str. MCS]	79.59	294	297
fig|6666666.28482.peg.1928	R005742	(gi:119870677) purC (Mkms 4648) - phosphoribosylaminoimidazole-succinocarboxamide synthase [Mycobacterium sp. str. KMS]	79.59	294	297

fig|6666666.28482.peg.1928	R005739	(gi:145222208) purC (Mflv 1616) - phosphoribosylaminoimidazole-succinocarboxamide synthase [Mycobacterium gilvum str. PYR-GCK]	78.57	294	297
fig|6666666.28482.peg.1928	R018973	(gi:169627791) purC (MAB 0689) - phosphoribosylaminoimidazole-succinocarboxamide synthase [Mycobacterium abscessus str. ATCC 19977]	77.1	297	297
fig|6666666.28482.peg.1928	R005740	(gi:120406086) purC (Mvan 5138) - phosphoribosylaminoimidazole-succinocarboxamide synthase [Mycobacterium vanbaalenii str. PYR-1]	75.5	294	297
fig|6666666.28482.peg.1948	R014657	(gi:157151431) rfbB-1 (SGO 1011) - dTDP-glucose 4,6-dehydratase [Streptococcus gordonii str. Challis (substr. CH1)]	60.3	325	333
fig|6666666.28482.peg.1963	R018607	(gi:15610586) eccB4 (Rv3450c) - hypothetical protein [Mycobacterium tuberculosis str. H37Rv]	63.4	458	462
fig|6666666.28482.peg.1963	R018608	(gi:148663315) eccB4 (MRA 3491) - hypothetical protein [Mycobacterium tuberculosis str. H37Ra]	63.4	458	462
fig|6666666.28482.peg.1963	R019791	(gi:224991867) eccB4 (JTY 3516) - hypothetical protein [Mycobacterium bovis str. BCG str. Tokyo 172]	63.4	458	462
fig|6666666.28482.peg.1963	R018611	(gi:121639371) eccB4 (BCG 3516c) - hypothetical protein [Mycobacterium bovis str. BCG Pasteur 1173P2]	63.4	458	462
fig|6666666.28482.peg.1963	R018610	(gi:31794627) eccB4 (Mb3480c) - hypothetical protein [Mycobacterium bovis str. AF2122/97]	63.4	458	462
fig|6666666.28482.peg.1963	R019788	(gi:339633455) eccB4 (MAF 34650) - putative conserved membrane protein [Mycobacterium africanum str. GM041182]	63.4	458	462
fig|6666666.28482.peg.1963	R019790	(gi:253800495) eccB4 (TBMG 03498) - hypothetical protein [Mycobacterium tuberculosis str. KZN 1435]	63.54	457	462
fig|6666666.28482.peg.1963	R018606	(gi:148824660) eccB4 (TBFG 13487) - hypothetical protein [Mycobacterium tuberculosis str. F11]	63.54	457	462
fig|6666666.28482.peg.1963	R018609	(gi:15843045) eccB4 (MT3556) - hypothetical protein [Mycobacterium tuberculosis str. CDC1551]	63.54	457	462
fig|6666666.28482.peg.1963	R019789	(gi:340628421) eccB4 (MCAN 34671) - hypothetical protein [Mycobacterium canettii str. CIPT 140010059]	62.98	458	462
fig|6666666.28482.peg.1963	R019786	(gi:183981119) eccB4 (MMAR 1099) - hypothetical protein [Mycobacterium marinum str. M]	60.81	457	462
fig|6666666.28482.peg.1963	R018614	(gi:118616632) eccB4 (MUL 0857) - hypothetical protein [Mycobacterium ulcerans str. Agy99]	60.39	457	462

fig|6666666.28482.peg.1963	R018613	(gi:118464971) eccB4 (MAV 4392) - hypothetical protein [Mycobacterium avium str. 104]	60.95	458	462
fig|6666666.28482.peg.1963	R018612	(gi:41410336) eccB4 (MAP4238) - hypothetical protein [Mycobacterium avium str. K-10 (subsp. paratuberculosis)]	61.22	457	462
fig|6666666.28482.peg.1964	R019783	(gi:253800494) mycP4 (TBMG 03497) - membrane-anchored mycosin mycP4 [Mycobacterium tuberculosis str. KZN 1435]	63.52	422	422
fig|6666666.28482.peg.1964	R018592	(gi:15610585) mycP4 (Rv3449) - membrane-anchored mycosin [Mycobacterium tuberculosis str. H37Rv]	63.52	422	422
fig|6666666.28482.peg.1964	R018593	(gi:148663314) mycP4 (MRA 3490) - membrane-anchored mycosin MycP4 [Mycobacterium tuberculosis str. H37Ra]	63.52	422	422
fig|6666666.28482.peg.1964	R018591	(gi:148824659) mycP4 (TBFG 13486) - membrane-anchored mycosin mycP4 [Mycobacterium tuberculosis str. F11]	63.52	422	422
fig|6666666.28482.peg.1964	R018594	(gi:15843044) mycP4 (MT3555) - serine protease [Mycobacterium tuberculosis str. CDC1551]	63.52	422	422
fig|6666666.28482.peg.1964	R019784	(gi:224991866) mycP4 (JTY 3515) - putative secreted serine protease [Mycobacterium bovis str. BCG str. Tokyo 172]	63.52	422	422
fig|6666666.28482.peg.1964	R018596	(gi:121639370) mycP4 (BCG 3515) - putative secreted serine protease [Mycobacterium bovis str. BCG Pasteur 1173P2]	63.52	422	422
fig|6666666.28482.peg.1964	R018595	(gi:31794626) mycP4 (Mb3479) - secreted serine protease [Mycobacterium bovis str. AF2122/97]	63.52	422	422
fig|6666666.28482.peg.1964	R019781	(gi:339633454) mycP4 (MAF 34640) - putative membrane-anchored mycosin MYCP4 (serine protease) [Mycobacterium africanum str. GM041182]	63.52	422	422
fig|6666666.28482.peg.1964	R019782	(gi:340628420) mycP4 (MCAN 34661) - putative membrane-anchored mycosin MYCP4 [Mycobacterium canettii str. CIPT 140010059]	62.86	422	422
fig|6666666.28482.peg.1964	R018598	(gi:118465498) mycP4 (MAV 4393) - peptidase [Mycobacterium avium str. 104]	68.98	392	422
fig|6666666.28482.peg.1964	R018597	(gi:41410337) mycP4 (MAP4239c) - hypothetical protein [Mycobacterium avium str. K-10 (subsp. paratuberculosis)]	68.97	395	422
fig|6666666.28482.peg.1964	R018599	(gi:118616633) mycP4 (MUL 0858) - membrane-anchored serine protease (mycosin), MycP4 [Mycobacterium ulcerans str. Agy99]	63.03	391	422
fig|6666666.28482.peg.1964	R019779	(gi:183981120) mycP4 (MMAR 1100) - membrane-anchored serine protease (mycosin), MycP4 [Mycobacterium marinum str. M]	63.03	391	422

fig|6666666.28482.peg.1966	R019768	(gi:340628418) eccC4 (MCAN 34641) - hypothetical protein [Mycobacterium canettii str. CIPT 140010059]	63.68	1148	1148
fig|6666666.28482.peg.1966	R018566	(gi:15610583) eccC4 (Rv3447c) - hypothetical protein [Mycobacterium tuberculosis str. H37Rv]	63.43	1148	1148
fig|6666666.28482.peg.1966	R018567	(gi:148663312) eccC4 (MRA 3488) - hypothetical protein [Mycobacterium tuberculosis str. H37Ra]	63.43	1148	1148
fig|6666666.28482.peg.1966	R019767	(gi:339633452) eccC4 (MAF 34620) - putative conserved membrane protein [Mycobacterium africanum str. GM041182]	63.43	1148	1148
fig|6666666.28482.peg.1966	R019769	(gi:253800492) eccC4 (TBMG 03495) - hypothetical protein [Mycobacterium tuberculosis str. KZN 1435]	63.43	1148	1148
fig|6666666.28482.peg.1966	R018565	(gi:148824657) eccC4 (TBFG 13484) - hypothetical protein [Mycobacterium tuberculosis str. F11]	63.43	1148	1148
fig|6666666.28482.peg.1966	R019770	(gi:224991864) eccC4 (JTY 3513) - hypothetical protein [Mycobacterium bovis str. BCG str. Tokyo 172]	63.43	1148	1148
fig|6666666.28482.peg.1966	R018570	(gi:121639368) eccC4 (BCG 3513c) - hypothetical protein [Mycobacterium bovis str. BCG Pasteur 1173P2]	63.43	1148	1148
fig|6666666.28482.peg.1966	R018569	(gi:31794624) eccC4 (Mb3477c) - hypothetical protein [Mycobacterium bovis str. AF2122/97]	63.35	1148	1148
fig|6666666.28482.peg.1966	R018568	(gi:15843042) eccC4 (MT3553) - FtsK/SpoIIIE family protein [Mycobacterium tuberculosis str. CDC1551]	63.28	1143	1148
fig|6666666.28482.peg.1966	R019765	(gi:183981122) eccC4 (MMAR 1102) - hypothetical protein [Mycobacterium marinum str. M]	60.22	1147	1148
fig|6666666.28482.peg.1966	R018572	(gi:118463646) eccC4 (MAV 4390) - ftsk/SpoIIIE family protein [Mycobacterium avium str. 104]	63.13	1147	1148
fig|6666666.28482.peg.1966	R018571	(gi:41410339) eccC4 (MAP4241) - hypothetical protein [Mycobacterium avium str. K-10 (subsp. paratuberculosis)]	63.3	1147	1148
fig|6666666.28482.peg.1968	R018538	(gi:15843040) esxU (MT3550) - hypothetical protein [Mycobacterium tuberculosis str. CDC1551]	79.35	92	92
fig|6666666.28482.peg.1968	R019756	(gi:253800490) esxU (TBMG 03493) - esat-6 like protein esxU [Mycobacterium tuberculosis str. KZN 1435]	79.35	92	92
fig|6666666.28482.peg.1968	R018536	(gi:15610581) esxU (Rv3445c) - ESAT-6 like protein ESXU [Mycobacterium tuberculosis str. H37Rv]	79.35	92	92

fig|6666666.28482.peg.1968	R018537	(gi:148663310) esxU (MRA 3486) - esat-6 like protein EsxU [Mycobacterium tuberculosis str. H37Ra]	79.35	92	92
fig|6666666.28482.peg.1968	R018535	(gi:148824655) esxU (TBFG 13482) - Esat-6 like protein esxU [Mycobacterium tuberculosis str. F11]	79.35	92	92
fig|6666666.28482.peg.1968	R019755	(gi:340628416) esxU (MCAN 34621) - ESAT-6 like protein ESXU [Mycobacterium canettii str. CIPT 140010059]	79.35	92	92
fig|6666666.28482.peg.1968	R019757	(gi:224991862) esxU (JTY 3511) - hypothetical protein [Mycobacterium bovis str. BCG str. Tokyo 172]	79.35	92	92
fig|6666666.28482.peg.1968	R018540	(gi:121639366) esxU (BCG 3511c) - hypothetical protein [Mycobacterium bovis str. BCG Pasteur 1173P2]	79.35	92	92
fig|6666666.28482.peg.1968	R018539	(gi:31794622) esxU (Mb3475c) - hypothetical protein [Mycobacterium bovis str. AF2122/97]	79.35	92	92
fig|6666666.28482.peg.1968	R019754	(gi:339633450) esxU (MAF 34600) - ESAT-6 like protein ESXU [Mycobacterium africanum str. GM041182]	79.35	92	92
fig|6666666.28482.peg.1968	R019753	(gi:183981124) esxU (MMAR 1104) - EsaT-6 like protein, EsxU [Mycobacterium marinum str. M]	75	92	92
fig|6666666.28482.peg.1968	R018543	(gi:118616637) esxU (MUL 0862) - EsaT-6 like protein, EsxU [Mycobacterium ulcerans str. Agy99]	74.73	91	92
fig|6666666.28482.peg.1968	R018542	(gi:118465412) esxU (MAV 4388) - hypothetical protein [Mycobacterium avium str. 104]	73.91	92	92
fig|6666666.28482.peg.1968	R018541	(gi:41410341) esxU (MAP4243) - hypothetical protein [Mycobacterium avium str. K-10 (subsp. paratuberculosis)]	70.65	92	92
fig|6666666.28482.peg.1968	R018546	(gi:120402473) esxU (Mvan 1464) - hypothetical protein [Mycobacterium vanbaalenii str. PYR-1]	65.22	92	92
fig|6666666.28482.peg.1968	R018545	(gi:145225533) esxU (Mflv 4957) - hypothetical protein [Mycobacterium gilvum str. PYR-GCK]	61.96	92	92
fig|6666666.28482.peg.1968	R019758	(gi:333991911) esxU (JDM601 3271) - hypothetical protein [Mycobacterium sp. str. JDM601]	64	75	92
fig|6666666.28482.peg.1969	R018525	(gi:41410342) esxT (MAP4244) - hypothetical protein [Mycobacterium avium str. K-10 (subsp. paratuberculosis)]	77.55	98	98
fig|6666666.28482.peg.1969	R018526	(gi:118467089) esxT (MAV 4387) - hypothetical protein [Mycobacterium avium str. 104]	77.55	98	98

fig|6666666.28482.peg.1969	R019748	(gi:340628415) esxT (MCAN 34611) - putative ESAT-6 like protein ESXT [Mycobacterium canettii str. CIPT 140010059]	79.38	97	98
fig|6666666.28482.peg.1969	R019749	(gi:253800489) esxT (TBMG 03492) - esat-6 like protein esxT [Mycobacterium tuberculosis str. KZN 1435]	79.38	97	98
fig|6666666.28482.peg.1969	R018520	(gi:15610580) esxT (Rv3444c) - putative ESAT-6 like protein ESXT [Mycobacterium tuberculosis str. H37Rv]	79.38	97	98
fig|6666666.28482.peg.1969	R018521	(gi:148663309) esxT (MRA 3485) - esat-6 like protein EsxT [Mycobacterium tuberculosis str. H37Ra]	79.38	97	98
fig|6666666.28482.peg.1969	R018519	(gi:148824654) esxT (TBFG 13481) - Esat-6 like protein esxT [Mycobacterium tuberculosis str. F11]	79.38	97	98
fig|6666666.28482.peg.1969	R018522	(gi:15843039) esxT (MT3549) - hypothetical protein [Mycobacterium tuberculosis str. CDC1551]	79.38	97	98
fig|6666666.28482.peg.1969	R019750	(gi:224991861) esxT (JTY 3510) - hypothetical protein [Mycobacterium bovis str. BCG str. Tokyo 172]	79.38	97	98
fig|6666666.28482.peg.1969	R018524	(gi:121639365) esxT (BCG 3510c) - hypothetical protein [Mycobacterium bovis str. BCG Pasteur 1173P2]	79.38	97	98
fig|6666666.28482.peg.1969	R018523	(gi:31794621) esxT (Mb3474c) - hypothetical protein [Mycobacterium bovis str. AF2122/97]	79.38	97	98
fig|6666666.28482.peg.1969	R019747	(gi:339633449) esxT (MAF 34590) - putative ESAT-6 like protein ESXT [Mycobacterium africanum str. GM041182]	79.38	97	98
fig|6666666.28482.peg.1969	R019746	(gi:183981125) esxT (MMAR 1105) - EsaT-6 like protein, EsxT [Mycobacterium marinum str. M]	73.47	98	98
fig|6666666.28482.peg.1969	R018528	(gi:118616638) esxT (MUL 0863) - EsaT-6 like protein, EsxT [Mycobacterium ulcerans str. Agy99]	71.43	98	98
fig|6666666.28482.peg.1969	R018529	(gi:118470444) esxT (MSMEG 1539) - hypothetical protein [Mycobacterium smegmatis str. MC2 155]	69.79	96	98
fig|6666666.28482.peg.1969	R018530	(gi:145225532) esxT (Mflv 4956) - hypothetical protein [Mycobacterium gilvum str. PYR-GCK]	65.62	96	98
fig|6666666.28482.peg.1969	R018531	(gi:120402474) esxT (Mvan 1465) - hypothetical protein [Mycobacterium vanbaalenii str. PYR-1]	65.62	96	98
fig|6666666.28482.peg.1969	R018534	(gi:108798101) esxT (Mmcs 1129) - hypothetical protein [Mycobacterium sp. str. MCS]	65.62	96	98

fig|6666666.28482.peg.1969	R018533	(gi:119867197) esxT (Mkms 1146) - hypothetical protein [Mycobacterium sp. str. KMS]	65.62	96	98
fig|6666666.28482.peg.1969	R018532	(gi:126433759) esxT (Mjls 1156) - hypothetical protein [Mycobacterium sp. str. JLS]	65.62	96	98
fig|6666666.28482.peg.1969	R019752	(gi:333991910) esxT (JDM601 3270) - EsaT-6 like protein EsxT [Mycobacterium sp. str. JDM601]	70.41	98	98
fig|6666666.28482.peg.1969	R018527	(gi:15827096) esxT (ML0363) - hypothetical protein [Mycobacterium leprae str. TN]	68.06	72	98
fig|6666666.28482.peg.1969	R019751	(gi:221229574) esxT (MLBr 00363) - putative secreted protein [Mycobacterium leprae str. Br4923]	68.06	72	98
fig|6666666.28482.peg.2006	R019865	(gi:253799172) PE18 (TBMG 02209) - PE family protein [Mycobacterium tuberculosis str. KZN 1435]	64.37	87	99
fig|6666666.28482.peg.2006	R018654	(gi:57116910) PE18 (Rv1788) - PE family protein [Mycobacterium tuberculosis str. H37Rv]	64.37	87	99
fig|6666666.28482.peg.2006	R018655	(gi:148661595) PE18 (MRA 1802) - PE family protein [Mycobacterium tuberculosis str. H37Ra]	64.37	87	99
fig|6666666.28482.peg.2006	R018653	(gi:148823003) PE18 (TBFG 11818) - PE family protein [Mycobacterium tuberculosis str. F11]	64.37	87	99
fig|6666666.28482.peg.2006	R018656	(gi:15841256) PE18 (MT1837) - PE family protein [Mycobacterium tuberculosis str. CDC1551]	64.37	87	99
fig|6666666.28482.peg.2006	R019864	(gi:340626800) PE18 (MCAN 18061) - PE family protein [Mycobacterium canettii str. CIPT 140010059]	64.37	87	99
fig|6666666.28482.peg.2006	R019866	(gi:224990173) PE18 (JTY 1804) - PE family protein [Mycobacterium bovis str. BCG str. Tokyo 172]	64.37	87	99
fig|6666666.28482.peg.2006	R018658	(gi:121637689) PE18 (BCG 1820) - PE family protein [Mycobacterium bovis str. BCG Pasteur 1173P2]	64.37	87	99
fig|6666666.28482.peg.2006	R018657	(gi:31792976) PE18 (Mb1816) - PE family protein [Mycobacterium bovis str. AF2122/97]	64.37	87	99
fig|6666666.28482.peg.2006	R019863	(gi:339631842) PE18 (MAF 18100) - PE family protein [Mycobacterium africanum str. GM041182]	64.37	87	99
fig|6666666.28482.peg.2006	R019877	(gi:183982680) PE19 (MMAR 2673) - PE family protein, PE19 [Mycobacterium marinum str. M]	60.92	87	99

fig|6666666.28482.peg.2006	R019867	(gi:333990534) PE18 (JDM601 1894) - PE-PGRS family protein [Mycobacterium sp. str. JDM601]	64.37	87	99
fig|6666666.28482.peg.2006	R018675	(gi:57116913) PE19 (Rv1791) - PE family protein [Mycobacterium tuberculosis str. H37Rv]	60.92	87	99
fig|6666666.28482.peg.2006	R018676	(gi:148661598) PE19 (MRA 1805) - PE family protein [Mycobacterium tuberculosis str. H37Ra]	60.92	87	99
fig|6666666.28482.peg.2006	R019879	(gi:340626803) PE19 (MCAN 18091) - PE family protein [Mycobacterium canettii str. CIPT 140010059]	60.92	87	99
fig|6666666.28482.peg.2006	R019881	(gi:224990176) PE19 (JTY 1807) - PE family protein [Mycobacterium bovis str. BCG str. Tokyo 172]	60.92	87	99
fig|6666666.28482.peg.2006	R018679	(gi:121637692) PE19 (BCG 1823) - PE family protein [Mycobacterium bovis str. BCG Pasteur 1173P2]	60.92	87	99
fig|6666666.28482.peg.2006	R018678	(gi:31792979) PE19 (Mb1819) - PE family protein [Mycobacterium bovis str. AF2122/97]	60.92	87	99
fig|6666666.28482.peg.2006	R019878	(gi:339631845) PE19 (MAF 18130) - PE family protein [Mycobacterium africanum str. GM041182]	60.92	87	99
fig|6666666.28482.peg.2006	R019880	(gi:253799168) PE19 (TBMG 02205) - PE family protein [Mycobacterium tuberculosis str. KZN 1435]	60.92	87	99
fig|6666666.28482.peg.2006	R018674	(gi:148823006) PE19 (TBFG 11821) - PE family protein [Mycobacterium tuberculosis str. F11]	60.92	87	99
fig|6666666.28482.peg.2006	R018677	(gi:15841261) PE19 (MT1840) - PE family protein [Mycobacterium tuberculosis str. CDC1551]	60.92	87	99
fig|6666666.28482.peg.2172	R019039	(gi:253798546) mbtH (TBMG 01598) - hypothetical protein [Mycobacterium tuberculosis str. KZN 1435]	78.87	71	71
fig|6666666.28482.peg.2172	R013460	(gi:148823579) mbtH (TBFG 12402) - hypotehtical mycobactin/exochelin synthesis protein mbtH [Mycobacterium tuberculosis str. F11]	78.87	71	71
fig|6666666.28482.peg.2172	R005850	(gi:15841889) mbtH (MT2445.1) - mbtH protein [Mycobacterium tuberculosis str. CDC1551]	78.87	71	71
fig|6666666.28482.peg.2172	R019038	(gi:340627388) mbtH (MCAN 24091) - hypothetical protein [Mycobacterium canettii str. CIPT 140010059]	78.87	71	71
fig|6666666.28482.peg.2172	R019040	(gi:224990750) mbtH (JTY 2385) - hypothetical protein [Mycobacterium bovis str. BCG str. Tokyo 172]	78.87	71	71

fig|6666666.28482.peg.2172	R005852	(gi:121638256) mbtH (BCG 2391c) - putative protein mbtH [Mycobacterium bovis str. BCG Pasteur 1173P2]	78.87	71	71
fig|6666666.28482.peg.2172	R005851	(gi:31793554) mbtH (Mb2398c) - PUTATIVE CONSERVED PROTEIN MBTH [Mycobacterium bovis str. AF2122/97]	78.87	71	71
fig|6666666.28482.peg.2172	R019037	(gi:339632403) mbtH (MAF 23910) - putative conserved protein MBTH [Mycobacterium africanum str. GM041182]	78.87	71	71
fig|6666666.28482.peg.2172	R005848	(gi:15609514) mbtH (Rv2377c) - PUTATIVE CONSERVED PROTEIN MBTH [Mycobacterium tuberculosis str. H37Rv]	77.46	71	71
fig|6666666.28482.peg.2172	R005849	(gi:148662209) mbtH (MRA 2400) - protein MbtH [Mycobacterium tuberculosis str. H37Ra]	77.46	71	71
fig|6666666.28482.peg.2172	R005859	(gi:126436052) mbtH (Mjls 3474) - MbtH domain protein [Mycobacterium sp. str. JLS]	71.83	71	71
fig|6666666.28482.peg.2172	R019035	(gi:183983167) mbtH (MMAR 3173) - protein MbtH [Mycobacterium marinum str. M]	71.83	71	71
fig|6666666.28482.peg.2172	R005861	(gi:108800429) mbtH (Mmcs 3463) - MbtH-like protein [Mycobacterium sp. str. MCS]	71.83	71	71
fig|6666666.28482.peg.2172	R005860	(gi:119869558) mbtH (Mkms 3526) - MbtH domain protein [Mycobacterium sp. str. KMS]	71.83	71	71
fig|6666666.28482.peg.2172	R019036	(gi:169631178) mbtH (MAB 4100c) - MbtH-like protein [Mycobacterium abscessus str. ATCC 19977]	71.01	69	71
fig|6666666.28482.peg.2172	R005856	(gi:118470732) mbtH (MSMEG 4508) - hypothetical protein [Mycobacterium smegmatis str. MC2 155]	72.06	68	71
fig|6666666.28482.peg.2172	R005857	(gi:145223286) mbtH (Mflv 2699) - MbtH-like protein [Mycobacterium gilvum str. PYR-GCK]	70	70	71
fig|6666666.28482.peg.2172	R005855	(gi:118618911) mbtH (MUL 3639) - conserved hypothetical protein MbtH 1 [Mycobacterium ulcerans str. Agy99]	66.2	71	71
fig|6666666.28482.peg.2172	R005858	(gi:120404800) mbtH (Mvan 3842) - MbtH domain protein [Mycobacterium vanbaalenii str. PYR-1]	66.67	71	71
fig|6666666.28482.peg.2172	R005853	(gi:41408267) mbtH (MAP2169c) - MbtH 3 [Mycobacterium avium str. K-10 (subsp. paratuberculosis)]	66.2	71	71
fig|6666666.28482.peg.2172	R005854	(gi:118464383) mbtH (MAV 2016) - hypothetical protein [Mycobacterium avium str. 104]	66.2	71	71

fig|6666666.28482.peg.2172	R019041	(gi:333990185) mbtH (JDM601 1545) - MbtH-like protein [Mycobacterium sp. str. JDM601]	63.51	71	71
fig|6666666.28482.peg.2172	R018198	(gi:118469728) MSMEG0016 - hypothetical protein [Mycobacterium smegmatis str. MC2 155]	64.29	67	71
fig|6666666.28482.peg.2173	R019046	(gi:253798545) mbtG (TBMG 01597) - lysine-N-oxygenase mbtG [Mycobacterium tuberculosis str. KZN 1435]	88.66	432	434
fig|6666666.28482.peg.2173	R005862	(gi:15609515) mbtG (Rv2378c) - LYSINE-N-OXYGENASE MBTG (L-LYSINE 6-MONOOXYGENASE) (LYSINE N6-HYDROXYLASE) [Mycobacterium tuberculosis str. H37Rv]	88.66	432	434
fig|6666666.28482.peg.2173	R005863	(gi:148662210) mbtG (MRA 2401) - lysine-N-oxygenase Mbtg [Mycobacterium tuberculosis str. H37Ra]	88.66	432	434
fig|6666666.28482.peg.2173	R013461	(gi:148823580) mbtG (TBFG 12403) - lysine-N-oxygenase mbtG [Mycobacterium tuberculosis str. F11]	88.66	432	434
fig|6666666.28482.peg.2173	R005864	(gi:15841890) mbtG (MT2446) - hypothetical protein [Mycobacterium tuberculosis str. CDC1551]	88.66	432	434
fig|6666666.28482.peg.2173	R019047	(gi:224990751) mbtG (JTY 2386) - lysine-N-oxygenase [Mycobacterium bovis str. BCG str. Tokyo 172]	88.66	432	434
fig|6666666.28482.peg.2173	R005866	(gi:121638257) mbtG (BCG 2392c) - Lysine-N-oxygenase mbtG [Mycobacterium bovis str. BCG Pasteur 1173P2]	88.66	432	434
fig|6666666.28482.peg.2173	R005865	(gi:31793555) mbtG (Mb2399c) - LYSINE-N-OXYGENASE MBTG (L-LYSINE 6-MONOOXYGENASE) (LYSINE N6-HYDROXYLASE) [Mycobacterium bovis str. AF2122/97]	88.66	432	434
fig|6666666.28482.peg.2173	R019044	(gi:339632404) mbtG (MAF 23920) - lysine-N-oxygenase MBTG (L-Lysine 6-monooxygenase) [Mycobacterium africanum str. GM041182]	88.66	432	434
fig|6666666.28482.peg.2173	R019045	(gi:340627389) mbtG (MCAN 24101) - lysine-N-oxygenase MBTG [Mycobacterium canettii str. CIPT 140010059]	88.43	432	434
fig|6666666.28482.peg.2173	R005868	(gi:118463821) mbtG (MAV 2015) - MbtG protein [Mycobacterium avium str. 104]	88.02	434	434
fig|6666666.28482.peg.2173	R005867	(gi:41408268) mbtG (MAP2170c) - MbtG [Mycobacterium avium str. K-10 (subsp. paratuberculosis)]	87.79	434	434
fig|6666666.28482.peg.2173	R005870	(gi:118467820) mbtG (MSMEG 4509) - MbtG protein [Mycobacterium smegmatis str. MC2 155]	76.33	429	434

fig|6666666.28482.peg.2173	R005871	(gi:145223285) mbtG (Mflv 2698) - FAD dependent oxidoreductase [Mycobacterium gilvum str. PYR-GCK]	76.39	432	434
fig|6666666.28482.peg.2173	R005872	(gi:120404801) mbtG (Mvan 3843) - FAD dependent oxidoreductase [Mycobacterium vanbaalenii str. PYR-1]	74.77	432	434
fig|6666666.28482.peg.2173	R019048	(gi:333990184) mbtG (JDM601 1544) - lysine-N-oxygenase MbtG [Mycobacterium sp. str. JDM601]	81.4	429	434
fig|6666666.28482.peg.2173	R005875	(gi:108800430) mbtG (Mmcs 3464) - FAD dependent oxidoreductase [Mycobacterium sp. str. MCS]	75.29	433	434
fig|6666666.28482.peg.2173	R005874	(gi:119869559) mbtG (Mkms 3527) - FAD dependent oxidoreductase [Mycobacterium sp. str. KMS]	75.29	433	434
fig|6666666.28482.peg.2173	R005873	(gi:126436053) mbtG (Mjls 3475) - FAD dependent oxidoreductase [Mycobacterium sp. str. JLS]	75.35	433	434
fig|6666666.28482.peg.2173	R019043	(gi:169629335) mbtG (MAB 2249) - lysine-N-oxygenase MbtG [Mycobacterium abscessus str. ATCC 19977]	68.13	433	434
fig|6666666.28482.peg.2173	R005869	(gi:118618912) mbtG (MUL 3640) - lysine-N-oxygenase MbtG [Mycobacterium ulcerans str. Agy99]	69.28	431	434
fig|6666666.28482.peg.2173	R019042	(gi:183983677) mbtG (MMAR 3697) - lysine-N-oxygenase MbtG [Mycobacterium marinum str. M]	69.52	431	434
fig|6666666.28482.peg.2174	R019052	(gi:340627390) mbtF (MCAN 24111) - peptide synthetase [Mycobacterium canettii str. CIPT 140010059]	74.88	1445	1447
fig|6666666.28482.peg.2174	R019053	(gi:253798544) mbtF (TBMG 01596) - peptide synthetase mbtF [Mycobacterium tuberculosis str. KZN 1435]	74.81	1445	1447
fig|6666666.28482.peg.2174	R005878	(gi:15841891) mbtF (MT2447) - peptide synthetase [Mycobacterium tuberculosis str. CDC1551]	74.81	1445	1447
fig|6666666.28482.peg.2174	R019051	(gi:339632405) mbtF (MAF 23930) - peptide synthetase MBTF (peptide synthase) [Mycobacterium africanum str. GM041182]	74.81	1445	1447
fig|6666666.28482.peg.2174	R005876	(gi:15609516) mbtF (Rv2379c) - PEPTIDE SYNTHETASE MBTF (PEPTIDE SYNTHASE) [Mycobacterium tuberculosis str. H37Rv]	74.83	1445	1447
fig|6666666.28482.peg.2174	R005877	(gi:148662211) mbtF (MRA 2402) - peptide synthetase [Mycobacterium tuberculosis str. H37Ra]	74.83	1445	1447
fig|6666666.28482.peg.2174	R013462	(gi:148823581) mbtF (TBFG 12404) - peptide synthetase mbtF [Mycobacterium tuberculosis str. F11]	74.74	1445	1447

fig|6666666.28482.peg.2174	R019054	(gi:224990752) mbtF (JTY 2387) - peptide synthetase [Mycobacterium bovis str. BCG str. Tokyo 172]	74.74	1445	1447
fig|6666666.28482.peg.2174	R005880	(gi:121638258) mbtF (BCG 2393c) - Peptide synthetase mbtF [Mycobacterium bovis str. BCG Pasteur 1173P2]	74.74	1445	1447
fig|6666666.28482.peg.2174	R005879	(gi:31793556) mbtF (Mb2400c) - PEPTIDE SYNTHETASE MBTF (PEPTIDE SYNTHASE) [Mycobacterium bovis str. AF2122/97]	74.74	1445	1447
fig|6666666.28482.peg.2174	R005889	(gi:108800431) mbtF (Mmcs 3465) - Amino acid adenylation [Mycobacterium sp. str. MCS]	65.66	1438	1447
fig|6666666.28482.peg.2174	R005888	(gi:119869560) mbtF (Mkms 3528) - amino acid adenylation domain [Mycobacterium sp. str. KMS]	65.66	1438	1447
fig|6666666.28482.peg.2174	R005887	(gi:126436054) mbtF (Mjls 3476) - amino acid adenylation domain [Mycobacterium sp. str. JLS]	65.45	1438	1447
fig|6666666.28482.peg.2174	R005885	(gi:145223284) mbtF (Mflv 2697) - amino acid adenylation domain [Mycobacterium gilvum str. PYR-GCK]	62.56	1437	1447
fig|6666666.28482.peg.2174	R005882	(gi:118462908) mbtF (MAV 2014) - putative sulfotransferase [Mycobacterium avium str. 104]	60.07	1441	1447
fig|6666666.28482.peg.2174	R005884	(gi:118470694) mbtF (MSMEG 4510) - peptide synthetase mbtf [Mycobacterium smegmatis str. MC2 155]	61	1447	1447
fig|6666666.28482.peg.2174	R005881	(gi:41408269) mbtF (MAP2171c) - MbtF [Mycobacterium avium str. K-10 (subsp. paratuberculosis)]	60.38	1203	1447
fig|6666666.28482.peg.2175	R019060	(gi:253798543) mbtE (TBMG 01595) - peptide synthetase mbtE [Mycobacterium tuberculosis str. KZN 1435]	78.9	1720	1720
fig|6666666.28482.peg.2175	R013463	(gi:148823582) mbtE (TBFG 12405) - peptide synthetase mbtE [Mycobacterium tuberculosis str. F11]	78.9	1720	1720
fig|6666666.28482.peg.2175	R005892	(gi:15841892) mbtE (MT2448) - peptide synthetase [Mycobacterium tuberculosis str. CDC1551]	78.85	1720	1720
fig|6666666.28482.peg.2175	R005890	(gi:15609517) mbtE (Rv2380c) - PEPTIDE SYNTHETASE MBTE (PEPTIDE SYNTHASE) [Mycobacterium tuberculosis str. H37Rv]	79.77	1676	1720
fig|6666666.28482.peg.2175	R005891	(gi:148662212) mbtE (MRA 2403) - peptide synthetase [Mycobacterium tuberculosis str. H37Ra]	79.77	1676	1720
fig|6666666.28482.peg.2175	R019061	(gi:224990753) mbtE (JTY 2388) - peptide synthetase [Mycobacterium bovis str. BCG str. Tokyo 172]	79.77	1676	1720

fig|6666666.28482.peg.2175	R005894	(gi:121638259) mbtE (BCG 2394c) - Peptide synthetase mbtE [Mycobacterium bovis str. BCG Pasteur 1173P2]	79.77	1676	1720
fig|6666666.28482.peg.2175	R005893	(gi:31793557) mbtE (Mb2401c) - PEPTIDE SYNTHETASE MBTE (PEPTIDE SYNTHASE) [Mycobacterium bovis str. AF2122/97]	79.77	1676	1720
fig|6666666.28482.peg.2175	R019058	(gi:339632406) mbtE (MAF 23940) - peptide synthetase MBTE (peptide synthase) [Mycobacterium africanum str. GM041182]	79.77	1676	1720
fig|6666666.28482.peg.2175	R019059	(gi:340627391) mbtE (MCAN 24121) - peptide synthetase [Mycobacterium canettii str. CIPT 140010059]	79.72	1676	1720
fig|6666666.28482.peg.2175	R005903	(gi:108800432) mbtE (Mmcs 3466) - Amino acid adenylation [Mycobacterium sp. str. MCS]	71.74	1684	1720
fig|6666666.28482.peg.2175	R005902	(gi:119869561) mbtE (Mkms 3529) - amino acid adenylation domain [Mycobacterium sp. str. KMS]	71.74	1684	1720
fig|6666666.28482.peg.2175	R005901	(gi:126436055) mbtE (Mjls 3477) - amino acid adenylation domain [Mycobacterium sp. str. JLS]	71.62	1684	1720
fig|6666666.28482.peg.2175	R005900	(gi:120404803) mbtE (Mvan 3845) - amino acid adenylation domain [Mycobacterium vanbaalenii str. PYR-1]	66.4	1717	1720
fig|6666666.28482.peg.2175	R005898	(gi:118470602) mbtE (MSMEG 4511) - linear gramicidin synthetase subunit B [Mycobacterium smegmatis str. MC2 155]	68.41	1592	1720
fig|6666666.28482.peg.2175	R005899	(gi:145223283) mbtE (Mflv 2696) - amino acid adenylation domain [Mycobacterium gilvum str. PYR-GCK]	66.6	1564	1720
fig|6666666.28482.peg.2175	R005896	(gi:118462221) mbtE (MAV 2013) - linear gramicidin synthetase subunit D [Mycobacterium avium str. 104]	66.21	1506	1720
fig|6666666.28482.peg.2175	R005895	(gi:41408271) mbtE (MAP2173c) - MbtE [Mycobacterium avium str. K-10 (subsp. paratuberculosis)]	66.02	1506	1720
fig|6666666.28482.peg.2175	R019062	(gi:333990182) mbtE (JDM601 1542) - non-ribosomal peptide synthetase MbtE [Mycobacterium sp. str. JDM601]	60.37	1491	1720
fig|6666666.28482.peg.2176	R019067	(gi:253798542) mbtD (TBMG 01594) - polyketide synthetase mbtD [Mycobacterium tuberculosis str. KZN 1435]	64.88	979	986
fig|6666666.28482.peg.2176	R005904	(gi:15609518) mbtD (Rv2381c) - POLYKETIDE SYNTHETASE MBTD (POLYKETIDE SYNTHASE) [Mycobacterium tuberculosis str. H37Rv]	64.88	979	986
fig|6666666.28482.peg.2176	R005905	(gi:148662213) mbtD (MRA 2404) - putative polyketide synthase MbtD [Mycobacterium tuberculosis str. H37Ra]	64.88	979	986

fig|6666666.28482.peg.2176	R013464	(gi:148823583) mbtD (TBFG 12406) - polyketide synthetase mbtD [Mycobacterium tuberculosis str. F11]	64.88	979	986
fig|6666666.28482.peg.2176	R019068	(gi:224990754) mbtD (JTY 2389) - polyketide synthetase [Mycobacterium bovis str. BCG str. Tokyo 172]	64.88	979	986
fig|6666666.28482.peg.2176	R005908	(gi:121638260) mbtD (BCG 2395c) - Polyketide synthetase mbtD [Mycobacterium bovis str. BCG Pasteur 1173P2]	64.88	979	986
fig|6666666.28482.peg.2176	R005907	(gi:31793558) mbtD (Mb2402c) - POLYKETIDE SYNTHETASE MBTD (POLYKETIDE SYNTHASE) [Mycobacterium bovis str. AF2122/97]	64.88	979	986
fig|6666666.28482.peg.2176	R019065	(gi:339632407) mbtD (MAF 23950) - polyketide synthetase MBTD (polyketide synthase) [Mycobacterium africanum str. GM041182]	64.88	979	986
fig|6666666.28482.peg.2176	R005906	(gi:15841893) mbtD (MT2449) - polyketide synthase, putative [Mycobacterium tuberculosis str. CDC1551]	64.88	979	986
fig|6666666.28482.peg.2176	R019066	(gi:340627392) mbtD (MCAN 24131) - polyketide synthetase MBTD [Mycobacterium canettii str. CIPT 140010059]	64.78	979	986
fig|6666666.28482.peg.2177	R005920	(gi:15841894) mbtC (MT2450) - polyketide synthase [Mycobacterium tuberculosis str. CDC1551]	89.15	433	437
fig|6666666.28482.peg.2177	R019074	(gi:253798541) mbtC (TBMG 01593) - polyketide synthetase mbtC [Mycobacterium tuberculosis str. KZN 1435]	89.15	433	437
fig|6666666.28482.peg.2177	R005918	(gi:15609519) mbtC (Rv2382c) - POLYKETIDE SYNTHETASE MBTC (POLYKETIDE SYNTHASE) [Mycobacterium tuberculosis str. H37Rv]	89.15	433	437
fig|6666666.28482.peg.2177	R005919	(gi:148662214) mbtC (MRA 2405) - Polyketide synthetase MbtC [Mycobacterium tuberculosis str. H37Ra]	89.15	433	437
fig|6666666.28482.peg.2177	R013465	(gi:148823584) mbtC (TBFG 12407) - polyketide synthetase mbtC [Mycobacterium tuberculosis str. F11]	89.15	433	437
fig|6666666.28482.peg.2177	R019073	(gi:340627393) mbtC (MCAN 24141) - polyketide synthetase MBTC [Mycobacterium canettii str. CIPT 140010059]	89.15	433	437
fig|6666666.28482.peg.2177	R019075	(gi:224990755) mbtC (JTY 2390) - polyketide synthetase [Mycobacterium bovis str. BCG str. Tokyo 172]	89.15	433	437
fig|6666666.28482.peg.2177	R005922	(gi:121638261) mbtC (BCG 2396c) - Polyketide synthetase mbtC [Mycobacterium bovis str. BCG Pasteur 1173P2]	89.15	433	437
fig|6666666.28482.peg.2177	R005921	(gi:31793559) mbtC (Mb2403c) - POLYKETIDE SYNTHETASE MBTC (POLYKETIDE SYNTHASE) [Mycobacterium bovis str. AF2122/97]	89.15	433	437

fig|6666666.28482.peg.2177	R019072	(gi:339632408) mbtC (MAF 23960) - polyketide synthetase MBTC (polyketide synthase) [Mycobacterium africanum str. GM041182]	89.15	433	437
fig|6666666.28482.peg.2177	R005929	(gi:126436057) mbtC (Mjls 3479) - beta-ketoacyl synthase [Mycobacterium sp. str. JLS]	77.86	429	437
fig|6666666.28482.peg.2177	R005931	(gi:108800434) mbtC (Mmcs 3468) - beta-ketoacyl synthase [Mycobacterium sp. str. MCS]	77.39	429	437
fig|6666666.28482.peg.2177	R005930	(gi:119869563) mbtC (Mkms 3531) - beta-ketoacyl synthase [Mycobacterium sp. str. KMS]	77.39	429	437
fig|6666666.28482.peg.2177	R005926	(gi:118468534) mbtC (MSMEG 4513) - polyketide synthase [Mycobacterium smegmatis str. MC2 155]	78.09	429	437
fig|6666666.28482.peg.2177	R005928	(gi:120404805) mbtC (Mvan 3847) - beta-ketoacyl synthase [Mycobacterium vanbaalenii str. PYR-1]	77.49	431	437
fig|6666666.28482.peg.2177	R005924	(gi:118467200) mbtC (MAV 2011) - erythronolide synthase, modules 1 and 2 [Mycobacterium avium str. 104]	72.08	430	437
fig|6666666.28482.peg.2177	R005923	(gi:41408273) mbtC (MAP2175c) - MbtC [Mycobacterium avium str. K-10 (subsp. paratuberculosis)]	72.08	430	437
fig|6666666.28482.peg.2177	R005927	(gi:145223281) mbtC (Mflv 2694) - beta-ketoacyl synthase [Mycobacterium gilvum str. PYR-GCK]	70.93	430	437
fig|6666666.28482.peg.2177	R019076	(gi:333990180) mbtC (JDM601 1540) - polyketide synthase MbtC [Mycobacterium sp. str. JDM601]	66.28	428	437
fig|6666666.28482.peg.2178	R019080	(gi:340627394) mbtB (MCAN 24151) - phenyloxazoline synthase [Mycobacterium canettii str. CIPT 140010059]	76.62	1407	1407
fig|6666666.28482.peg.2178	R019081	(gi:253798540) mbtB (TBMG 01592) - phenyloxazoline synthase mbtB [Mycobacterium tuberculosis str. KZN 1435]	76.62	1407	1407
fig|6666666.28482.peg.2178	R005932	(gi:15609520) mbtB (Rv2383c) - PHENYLOXAZOLINE SYNTHASE MBTB (PHENYLOXAZOLINE SYNTHETASE) [Mycobacterium tuberculosis str. H37Rv]	76.62	1407	1407
fig|6666666.28482.peg.2178	R005933	(gi:148662215) mbtB (MRA 2406) - dihydroaeruginoic acid synthetase [Mycobacterium tuberculosis str. H37Ra]	76.62	1407	1407
fig|6666666.28482.peg.2178	R013466	(gi:148823585) mbtB (TBFG 12408) - phenyloxazoline synthase mbtB [Mycobacterium tuberculosis str. F11]	76.62	1407	1407
fig|6666666.28482.peg.2178	R019079	(gi:339632409) mbtB (MAF 23970) - phenyloxazoline synthase MBTB (phenyloxazoline synthetase) [Mycobacterium africanum str. GM041182]	76.62	1407	1407

fig|6666666.28482.peg.2178	R005935	(gi:31793560) mbtB (Mb2404c) - PHENYLOXAZOLINE SYNTHASE MBTB (PHENYLOXAZOLINE SYNTHETASE) [Mycobacterium bovis str. AF2122/97]	76.55	1407	1407
fig|6666666.28482.peg.2178	R005934	(gi:15841895) mbtB (MT2451) - dihydroaeruginoic acid synthetase [Mycobacterium tuberculosis str. CDC1551]	76.55	1407	1407
fig|6666666.28482.peg.2178	R019082	(gi:224990756) mbtB (JTY 2391) - phenyloxazoline synthase [Mycobacterium bovis str. BCG str. Tokyo 172]	76.55	1407	1407
fig|6666666.28482.peg.2178	R005936	(gi:121638262) mbtB (BCG 2397c) - Phenyloxazoline synthase mbtB [Mycobacterium bovis str. BCG Pasteur 1173P2]	76.55	1407	1407
fig|6666666.28482.peg.2178	R005938	(gi:118464419) mbtB (MAV 2009) - MbtB protein [Mycobacterium avium str. 104]	75.71	1155	1407
fig|6666666.28482.peg.2178	R005937	(gi:41408275) mbtB (MAP2177c) - MbtB [Mycobacterium avium str. K-10 (subsp. paratuberculosis)]	75.28	1155	1407
fig|6666666.28482.peg.2178	R005940	(gi:118469771) mbtB (MSMEG 4515) - putative non-ribosomal peptide synthetase [Mycobacterium smegmatis str. MC2 155]	62.28	1157	1407
fig|6666666.28482.peg.2178	R019083	(gi:333990169) mbtB (JDM601 1529) - phenyloxazoline synthase MbtB [Mycobacterium sp. str. JDM601]	65.45	1161	1407
fig|6666666.28482.peg.2178	R005943	(gi:126436059) mbtB (Mjls 3481) - amino acid adenylation domain [Mycobacterium sp. str. JLS]	62.56	1160	1407
fig|6666666.28482.peg.2178	R005945	(gi:108800436) mbtB (Mmcs 3470) - Amino acid adenylation [Mycobacterium sp. str. MCS]	62.05	1160	1407
fig|6666666.28482.peg.2178	R005944	(gi:119869565) mbtB (Mkms 3533) - amino acid adenylation domain [Mycobacterium sp. str. KMS]	62.05	1160	1407
fig|6666666.28482.peg.2178	R005942	(gi:120404807) mbtB (Mvan 3849) - amino acid adenylation domain [Mycobacterium vanbaalenii str. PYR-1]	60.36	1156	1407
fig|6666666.28482.peg.2179	R019087	(gi:340627395) mbtA (MCAN 24161) - bifunctional salicyl-AMP ligase/salicyl-S-ArCP synthetase [Mycobacterium canettii str. CIPT 140010059]	78.89	537	547
fig|6666666.28482.peg.2179	R019088	(gi:253798539) mbtA (TBMG 01591) - bifunctional salicyl-AMP ligase/salicyl-S-arcp synthetase mbtA [Mycobacterium tuberculosis str. KZN 1435]	78.89	537	547
fig|6666666.28482.peg.2179	R005946	(gi:15609521) mbtA (Rv2384) - BIFUNCTIONAL ENZYME MBTA: SALICYL-AMP LIGASE (SAL-AMP LIGASE) + SALICYL-S-ArCP SYNTHETASE [Mycobacterium tuberculosis str. H37Rv]	78.89	537	547

fig|6666666.28482.peg.2179	R005947	(gi:148662216) mbtA (MRA 2407) - 2,3-dihydroxybenzoate-AMP ligase [Mycobacterium tuberculosis str. H37Ra]	78.89	537	547
fig|6666666.28482.peg.2179	R013467	(gi:148823586) mbtA (TBFG 12409) - bifunctional enzyme mbtA: salicyl-AMP ligase + salicyl-S-arcp synthetase [Mycobacterium tuberculosis str. F11]	78.89	537	547
fig|6666666.28482.peg.2179	R005948	(gi:15841896) mbtA (MT2452) - 2,3-dihydroxybenzoate-AMP ligase [Mycobacterium tuberculosis str. CDC1551]	78.89	537	547
fig|6666666.28482.peg.2179	R019089	(gi:224990757) mbtA (JTY 2392) - salicyl-AMP ligase [Mycobacterium bovis str. BCG str. Tokyo 172]	78.89	537	547
fig|6666666.28482.peg.2179	R005950	(gi:121638263) mbtA (BCG 2398) - Bifunctional enzyme mbtA: salicyl-AMP ligase + salicyl-S-ArCP synthetase [Mycobacterium bovis str. BCG Pasteur 1173P2]	78.89	537	547
fig|6666666.28482.peg.2179	R005949	(gi:31793561) mbtA (Mb2405) - BIFUNCTIONAL ENZYME MBTA: SALICYL-AMP LIGASE (SAL-AMP LIGASE) + SALICYL-S-ArCP SYNTHETASE [Mycobacterium bovis str. AF2122/97]	78.89	537	547
fig|6666666.28482.peg.2179	R019086	(gi:339632410) mbtA (MAF 23980) - bifunctional enzyme MBTA: salicyl-AMP ligase (SAL-AMP ligase) + salicyl-S-ArCP synthetase [Mycobacterium africanum str. GM041182]	78.89	537	547
fig|6666666.28482.peg.2179	R005952	(gi:118462251) mbtA (MAV 2008) - 2,3-dihydroxybenzoate-AMP ligase [Mycobacterium avium str. 104]	76.47	544	547
fig|6666666.28482.peg.2179	R019084	(gi:183983678) mbtA (MMAR 3698) - bifunctional enzyme MbtA: salicyl-AMP ligase (SAL-AMP ligase) + salicyl-S-ACP synthetase [Mycobacterium marinum str. M]	69.85	541	547
fig|6666666.28482.peg.2179	R005953	(gi:118618913) mbtA (MUL 3641) - bifunctional enzyme MbtA: salicyl-AMP ligase (SAL-AMP ligase) + salicyl-S-ACP synthetase [Mycobacterium ulcerans str. Agy99]	69.96	533	547
fig|6666666.28482.peg.2179	R005954	(gi:118468444) mbtA (MSMEG 4516) - 2,3-dihydroxybenzoate-AMP ligase [Mycobacterium smegmatis str. MC2 155]	68.33	536	547
fig|6666666.28482.peg.2179	R005957	(gi:126436060) mbtA (Mjls 3482) - AMP-dependent synthetase and ligase [Mycobacterium sp. str. JLS]	64.37	532	547
fig|6666666.28482.peg.2179	R005959	(gi:108800437) mbtA (Mmcs 3471) - AMP-dependent synthetase and ligase [Mycobacterium sp. str. MCS]	64.37	532	547

fig|6666666.28482.peg.2179	R005958	(gi:119869566) mbtA (Mkms 3534) - AMP-dependent synthetase and ligase [Mycobacterium sp. str. KMS]	64.37	532	547
fig|6666666.28482.peg.2179	R005955	(gi:145223278) mbtA (Mflv 2691) - AMP-dependent synthetase and ligase [Mycobacterium gilvum str. PYR-GCK]	63.06	532	547
fig|6666666.28482.peg.2179	R019090	(gi:333990168) mbtA (JDM601 1528) - bifunctional enzyme MbtA: salicyl-AMP ligase (SAL-AMP ligase)/salicyl-S-ACP synthetase [Mycobacterium sp. str. JDM601]	64.26	508	547
fig|6666666.28482.peg.2179	R019085	(gi:169629333) mbtA (MAB 2247c) - bifunctional salicyl-AMP ligase/salicyl-S-arcp synthetase [Mycobacterium abscessus str. ATCC 19977]	62.66	533	547
fig|6666666.28482.peg.2179	R005956	(gi:120404808) mbtA (Mvan 3850) - AMP-dependent synthetase and ligase [Mycobacterium vanbaalenii str. PYR-1]	62.9	527	547
fig|6666666.28482.peg.2179	R005951	(gi:41408276) mbtA (MAP2178) - MbtA [Mycobacterium avium str. K-10 (subsp. paratuberculosis)]	74.78	337	547
fig|6666666.28482.peg.2181	R005974	(gi:118466860) mbtI (MAV 1792) - anthranilate synthase component I [Mycobacterium avium str. 104]	81.11	450	450
fig|6666666.28482.peg.2181	R005973	(gi:41408303) mbtI (MAP2205c) - anthranilate synthase component I [Mycobacterium avium str. K-10 (subsp. paratuberculosis)]	80.89	450	450
fig|6666666.28482.peg.2181	R019098	(gi:183983686) mbtI (MMAR 3706) - salicylate synthase MbtI [Mycobacterium marinum str. M]	78.49	448	450
fig|6666666.28482.peg.2181	R005975	(gi:118618919) mbtI (MUL 3648) - isochorismate synthase MbtI [Mycobacterium ulcerans str. Agy99]	77.8	450	450
fig|6666666.28482.peg.2181	R019102	(gi:253798537) mbtI (TBMG 01589) - salicylate synthase MbtI [Mycobacterium tuberculosis str. KZN 1435]	77.33	450	450
fig|6666666.28482.peg.2181	R005968	(gi:57116981) mbtI (Rv2386c) - anthranilate synthase component I [Mycobacterium tuberculosis str. H37Rv]	77.33	450	450
fig|6666666.28482.peg.2181	R005969	(gi:148662218) mbtI (MRA 2409) - putative isochorismate synthase MbtI [Mycobacterium tuberculosis str. H37Ra]	77.33	450	450
fig|6666666.28482.peg.2181	R013469	(gi:148823588) mbtI (TBFG 12411) - isochorismate synthase mbtI [Mycobacterium tuberculosis str. F11]	77.33	450	450
fig|6666666.28482.peg.2181	R005970	(gi:15841898) mbtI (MT2454) - anthranilate synthase component I [Mycobacterium tuberculosis str. CDC1551]	77.33	450	450
fig|6666666.28482.peg.2181	R019103	(gi:224990759) mbtI (JTY 2394) - salicylate synthase MbtI [Mycobacterium bovis str. BCG str. Tokyo 172]	77.33	450	450

fig|6666666.28482.peg.2181	R005972	(gi:121638265) mbtI (BCG 2400c) - putative isochorismate synthase mbtI [Mycobacterium bovis str. BCG Pasteur 1173P2]	77.33	450	450
fig|6666666.28482.peg.2181	R005971	(gi:31793563) mbtI (Mb2407c) - anthranilate synthase component I [Mycobacterium bovis str. AF2122/97]	77.33	450	450
fig|6666666.28482.peg.2181	R019100	(gi:339632412) mbtI (MAF 24000) - putative isochorismate synthase MBTI [Mycobacterium africanum str. GM041182]	77.33	450	450
fig|6666666.28482.peg.2181	R019101	(gi:340627397) mbtI (MCAN 24181) - putative isochorismate synthase MBTI [Mycobacterium canettii str. CIPT 140010059]	77.33	450	450
fig|6666666.28482.peg.2181	R005976	(gi:118473231) mbtI (MSMEG 4524) - anthranilate synthase component I [Mycobacterium smegmatis str. MC2 155]	67.04	449	450
fig|6666666.28482.peg.2181	R019099	(gi:169629331) mbtI (MAB 2245) - salicylate synthase MbtI [Mycobacterium abscessus str. ATCC 19977]	64.68	449	450
fig|6666666.28482.peg.2226	R005372	(gi:41408216) stf0 (MAP2118) - hypothetical protein [Mycobacterium avium str. K-10 (subsp. paratuberculosis)]	89.89	267	267
fig|6666666.28482.peg.2226	R005373	(gi:118463655) stf0 (MAV 2058) - putative sulfotransferase [Mycobacterium avium str. 104]	89.92	258	267
fig|6666666.28482.peg.2226	R005377	(gi:108797369) stf0 (Mmcs 0389) - hypothetical protein [Mycobacterium sp. str. MCS]	85.02	267	267
fig|6666666.28482.peg.2226	R005376	(gi:119866453) stf0 (Mkms 0398) - hypothetical protein [Mycobacterium sp. str. KMS]	85.02	267	267
fig|6666666.28482.peg.2226	R005375	(gi:126432990) stf0 (Mjls 0377) - hypothetical protein [Mycobacterium sp. str. JLS]	85.02	267	267
fig|6666666.28482.peg.2226	R018843	(gi:169628636) stf0 (MAB 1546c) - hypothetical protein [Mycobacterium abscessus str. ATCC 19977]	83.46	266	267
fig|6666666.28482.peg.2226	R018842	(gi:183984227) stf0 (MMAR 4255) - hypothetical protein [Mycobacterium marinum str. M]	83.9	267	267
fig|6666666.28482.peg.2226	R005374	(gi:118467454) stf0 (MSMEG 0630) - hypothetical protein [Mycobacterium smegmatis str. MC2 155]	80.45	266	267
fig|6666666.28482.peg.2226	R018846	(gi:253797222) stf0 (TBMG 00300) - hypothetical protein [Mycobacterium tuberculosis str. KZN 1435]	73.41	267	267
fig|6666666.28482.peg.2226	R005367	(gi:15607436) stf0 (Rv0295c) - hypothetical protein [Mycobacterium tuberculosis str. H37Rv]	73.41	267	267

fig|6666666.28482.peg.2226	R005368	(gi:148660061) stf0 (MRA 0304) - hypothetical protein [Mycobacterium tuberculosis str. H37Ra]	73.41	267	267
fig|6666666.28482.peg.2226	R013419	(gi:148821493) stf0 (TBFG 10302) - hypothetical protein [Mycobacterium tuberculosis str. F11]	73.41	267	267
fig|6666666.28482.peg.2226	R005369	(gi:15839681) stf0 (MT0308) - hypothetical protein [Mycobacterium tuberculosis str. CDC1551]	73.41	267	267
fig|6666666.28482.peg.2226	R018845	(gi:340625333) stf0 (MCAN 03041) - hypothetical protein [Mycobacterium canettii str. CIPT 140010059]	73.41	267	267
fig|6666666.28482.peg.2226	R018847	(gi:224988683) stf0 (JTY 0305) - hypothetical protein [Mycobacterium bovis str. BCG str. Tokyo 172]	73.41	267	267
fig|6666666.28482.peg.2226	R005371	(gi:121636210) stf0 (BCG 0335c) - hypothetical protein [Mycobacterium bovis str. BCG Pasteur 1173P2]	73.41	267	267
fig|6666666.28482.peg.2226	R005370	(gi:31791474) stf0 (Mb0303c) - hypothetical protein [Mycobacterium bovis str. AF2122/97]	73.41	267	267
fig|6666666.28482.peg.2226	R018844	(gi:339630370) stf0 (MAF 02970) - hypothetical protein [Mycobacterium africanum str. GM041182]	73.41	267	267
fig|6666666.28482.peg.2276	R005515	(gi:41406359) lpqH (MAP0261c) - hypothetical protein [Mycobacterium avium str. K-10 (subsp. paratuberculosis)]	78.1	103	106
fig|6666666.28482.peg.2276	R005516	(gi:118462431) lpqH (MAV 0306) - lipoprotein LpqH [Mycobacterium avium str. 104]	78.1	103	106
fig|6666666.28482.peg.2276	R018905	(gi:253800810) lpqH (TBMG 03808) - 19 kDa lipoprotein antigen precursor lpqH [Mycobacterium tuberculosis str. KZN 1435]	75	106	106
fig|6666666.28482.peg.2276	R005510	(gi:15610899) lpqH (Rv3763) - 19 KDA LIPOPROTEIN ANTIGEN PRECURSOR LPQH [Mycobacterium tuberculosis str. H37Rv]	75	106	106
fig|6666666.28482.peg.2276	R005511	(gi:148663629) lpqH (MRA 3801) - lipoprotein LpqH [Mycobacterium tuberculosis str. H37Ra]	75	106	106
fig|6666666.28482.peg.2276	R013431	(gi:148824968) lpqH (TBFG 13795) - 19 kda lipoprotein antigen precursor lpqH [Mycobacterium tuberculosis str. F11]	75	106	106
fig|6666666.28482.peg.2276	R018904	(gi:340628734) lpqH (MCAN 37831) - 19 KDA lipoprotein antigen LPQH [Mycobacterium canettii str. CIPT 140010059]	75	106	106
fig|6666666.28482.peg.2276	R018906	(gi:224992173) lpqH (JTY 3824) - 19 kDa lipoprotein antigen precursor [Mycobacterium bovis str. BCG str. Tokyo 172]	75	106	106

fig|6666666.28482.peg.2276	R005514	(gi:121639677) lpqH (BCG 3822) - 19 kDa lipoprotein antigen precursor lpqH [Mycobacterium bovis str. BCG Pasteur 1173P2]	75	106	106
fig|6666666.28482.peg.2276	R005513	(gi:31794933) lpqH (Mb3789) - 19 KDA LIPOPROTEIN ANTIGEN PRECURSOR LPQH [Mycobacterium bovis str. AF2122/97]	75	106	106
fig|6666666.28482.peg.2276	R018903	(gi:339633757) lpqH (MAF 37760) - 19 KDa lipoprotein antigen precursor Lpq [Mycobacterium africanum str. GM041182]	75	106	106
fig|6666666.28482.peg.2276	R005518	(gi:118619526) lpqH (MUL 4389) - 19 kDa lipoprotein antigen precursor LpqH [Mycobacterium ulcerans str. Agy99]	75.24	103	106
fig|6666666.28482.peg.2276	R005512	(gi:15843383) lpqH (MT3870) - lipoprotein [Mycobacterium tuberculosis str. CDC1551]	74.07	106	106
fig|6666666.28482.peg.2276	R018900	(gi:183985283) lpqH (MMAR 5315) - 19 kDa lipoprotein antigen precursor LpqH [Mycobacterium marinum str. M]	75.51	96	106
fig|6666666.28482.peg.2276	R005520	(gi:145221818) lpqH (Mflv 1226) - lipoprotein antigen family protein [Mycobacterium gilvum str. PYR-GCK]	64.49	105	106
fig|6666666.28482.peg.2276	R005524	(gi:108801913) lpqH (Mmcs 4950) - Mycobacterium 19 kDa lipoprotein antigen [Mycobacterium sp. str. MCS]	60.75	105	106
fig|6666666.28482.peg.2276	R005523	(gi:119871065) lpqH (Mkms 5038) - Mycobacterium 19 kDa lipoprotein antigen family protein [Mycobacterium sp. str. KMS]	60.75	105	106
fig|6666666.28482.peg.2276	R005522	(gi:126437894) lpqH (Mjls 5331) - lipoprotein antigen family protein [Mycobacterium sp. str. JLS]	60.75	105	106
fig|6666666.28482.peg.2411	R006727	(gi:118466088) mce5F (MAV 2383) - mce related protein [Mycobacterium avium str. 104]	88.19	415	416
fig|6666666.28482.peg.2411	R006726	(gi:41407954) mce5F (MAP1856) - hypothetical protein [Mycobacterium avium str. K-10 (subsp. paratuberculosis)]	88.19	415	416
fig|6666666.28482.peg.2411	R019468	(gi:183983844) mce5F (MMAR 3869) - Mce family protein Mce5F [Mycobacterium marinum str. M]	82.45	416	416
fig|6666666.28482.peg.2411	R006728	(gi:118619040) mce5F (MUL 3801) - Mce family protein Mce5F [Mycobacterium ulcerans str. Agy99]	81.97	416	416
fig|6666666.28482.peg.2411	R006731	(gi:120401979) mce5F (Mvan 0964) - Mammalian cell entry related domain protein [Mycobacterium vanbaalenii str. PYR-1]	68.99	416	416
fig|6666666.28482.peg.2411	R019469	(gi:169628100) mce5F (MAB 1005c) - putative MCE family protein [Mycobacterium abscessus str. ATCC 19977]	68.59	416	416

fig|6666666.28482.peg.2411	R006730	(gi:145220611) mce5F (Mflv 0005) - virulence factor Mce family protein [Mycobacterium gilvum str. PYR-GCK]	69.71	416	416
fig|6666666.28482.peg.2411	R006843	(gi:118463870) mce9F (MAV 0107) - mce related protein [Mycobacterium avium str. 104]	64.18	416	416
fig|6666666.28482.peg.2411	R006842	(gi:41406211) mce9F (MAP0113) - hypothetical protein [Mycobacterium avium str. K-10 (subsp. paratuberculosis)]	63.94	416	416
fig|6666666.28482.peg.2411	R006729	(gi:118471128) mce5F (MSMEG 1148) - mce related protein [Mycobacterium smegmatis str. MC2 155]	66.27	416	416
fig|6666666.28482.peg.2411	R019470	(gi:333988775) mce5F (JDM601 0135) - Mce family protein Mce5F [Mycobacterium sp. str. JDM601]	63.7	416	416
fig|6666666.28482.peg.2411	R006737	(gi:118619940) mce6F (MUL 4914) - MCE-family protein Mce6F [Mycobacterium ulcerans str. Agy99]	66.58	398	416
fig|6666666.28482.peg.2411	R019481	(gi:183980216) mce6F (MMAR 0182) - MCE-family protein Mce6F [Mycobacterium marinum str. M]	66.33	398	416
fig|6666666.28482.peg.2411	R019482	(gi:169628790) mce6F (MAB 1700) - putative Mce family protein [Mycobacterium abscessus str. ATCC 19977]	64.82	398	416
fig|6666666.28482.peg.2412	R006720	(gi:41407953) mce5E (MAP1855) - hypothetical protein [Mycobacterium avium str. K-10 (subsp. paratuberculosis)]	84.86	370	402
fig|6666666.28482.peg.2412	R006721	(gi:118464806) mce5E (MAV 2384) - mce related protein [Mycobacterium avium str. 104]	84.59	370	402
fig|6666666.28482.peg.2412	R019465	(gi:183983843) mce5E (MMAR 3868) - Mce family protein, Mce5E [Mycobacterium marinum str. M]	76.04	384	402
fig|6666666.28482.peg.2412	R006722	(gi:118619039) mce5E (MUL 3800) - Mce family protein, Mce5E [Mycobacterium ulcerans str. Agy99]	75.26	384	402
fig|6666666.28482.peg.2412	R006724	(gi:145220612) mce5E (Mflv 0006) - Mammalian cell entry related domain protein [Mycobacterium gilvum str. PYR-GCK]	65.37	384	402
fig|6666666.28482.peg.2412	R006723	(gi:118472982) mce5E (MSMEG 1147) - mce related protein [Mycobacterium smegmatis str. MC2 155]	66.93	378	402
fig|6666666.28482.peg.2412	R006725	(gi:120401978) mce5E (Mvan 0963) - Mammalian cell entry related domain protein [Mycobacterium vanbaalenii str. PYR-1]	64.63	376	402
fig|6666666.28482.peg.2412	R019466	(gi:169628101) mce5E (MAB 1006c) - putative MCE family protein [Mycobacterium abscessus str. ATCC 19977]	64.77	369	402

fig|6666666.28482.peg.2412	R019467	(gi:333988776) mce5E (JDM601 0136) - Mce family protein Mce5E [Mycobacterium sp. str. JDM601]	64.97	354	402
fig|6666666.28482.peg.2412	R006840	(gi:41406210) mce9E (MAP0112) - hypothetical protein [Mycobacterium avium str. K-10 (subsp. paratuberculosis)]	63.2	367	402
fig|6666666.28482.peg.2412	R006841	(gi:118464466) mce9E (MAV 0106) - mce related protein [Mycobacterium avium str. 104]	62.93	367	402
fig|6666666.28482.peg.2412	R019480	(gi:169628789) mce6E (MAB 1699) - putative Mce family protein [Mycobacterium abscessus str. ATCC 19977]	61.34	357	402
fig|6666666.28482.peg.2413	R006716	(gi:118464427) mce5D (MAV 2385) - virulence factor Mce family protein [Mycobacterium avium str. 104]	81.25	368	373
fig|6666666.28482.peg.2413	R006715	(gi:41407952) mce5D (MAP1854) - hypothetical protein [Mycobacterium avium str. K-10 (subsp. paratuberculosis)]	80.98	368	373
fig|6666666.28482.peg.2413	R019462	(gi:183983842) mce5D (MMAR 3867) - MCE-family protein Mce5D [Mycobacterium marinum str. M]	80.06	346	373
fig|6666666.28482.peg.2413	R019463	(gi:169628102) mce5D (MAB 1007c) - putative MCE family protein [Mycobacterium abscessus str. ATCC 19977]	68.99	345	373
fig|6666666.28482.peg.2413	R006718	(gi:145220613) mce5D (Mflv 0007) - virulence factor Mce family protein [Mycobacterium gilvum str. PYR-GCK]	65.62	348	373
fig|6666666.28482.peg.2413	R019477	(gi:183980214) mce6D (MMAR 0180) - MCE-family protein Mce6D [Mycobacterium marinum str. M]	65.99	347	373
fig|6666666.28482.peg.2413	R006735	(gi:118619942) mce6D (MUL 4916) - MCE-family protein Mce6D [Mycobacterium ulcerans str. Agy99]	65.71	347	373
fig|6666666.28482.peg.2413	R019478	(gi:169628788) mce6D (MAB 1698) - putative Mce family protein [Mycobacterium abscessus str. ATCC 19977]	65.22	345	373
fig|6666666.28482.peg.2413	R006719	(gi:120401977) mce5D (Mvan 0962) - virulence factor Mce family protein [Mycobacterium vanbaalenii str. PYR-1]	64.66	347	373
fig|6666666.28482.peg.2413	R006838	(gi:41406209) mce9D (MAP0111) - hypothetical protein [Mycobacterium avium str. K-10 (subsp. paratuberculosis)]	62.93	348	373
fig|6666666.28482.peg.2413	R006839	(gi:118462753) mce9D (MAV 0105) - virulence factor Mce family protein [Mycobacterium avium str. 104]	62.93	348	373
fig|6666666.28482.peg.2413	R006717	(gi:118468242) mce5D (MSMEG 1146) - virulence factor Mce family protein [Mycobacterium smegmatis str. MC2 155]	63.19	344	373

fig|6666666.28482.peg.2413	R019464	(gi:333988777) mce5D (JDM601 0137) - MCE-family protein Mce5D [Mycobacterium sp. str. JDM601]	61.85	366	373
fig|6666666.28482.peg.2414	R006709	(gi:41407951) mce5C (MAP1853) - hypothetical protein [Mycobacterium avium str. K-10 (subsp. paratuberculosis)]	85.19	351	351
fig|6666666.28482.peg.2414	R006710	(gi:118463686) mce5C (MAV 2386) - virulence factor Mce family protein [Mycobacterium avium str. 104]	85.84	346	351
fig|6666666.28482.peg.2414	R019459	(gi:183983841) mce5C (MMAR 3866) - Mce family protein, Mce5C [Mycobacterium marinum str. M]	76.14	351	351
fig|6666666.28482.peg.2414	R006711	(gi:118619038) mce5C (MUL 3798) - Mce family protein, Mce5C [Mycobacterium ulcerans str. Agy99]	75.85	351	351
fig|6666666.28482.peg.2414	R019460	(gi:169628103) mce5C (MAB 1008c) - putative MCE family protein [Mycobacterium abscessus str. ATCC 19977]	67.06	343	351
fig|6666666.28482.peg.2414	R006712	(gi:118469321) mce5C (MSMEG 1145) - virulence factor Mce family protein [Mycobacterium smegmatis str. MC2 155]	63.99	336	351
fig|6666666.28482.peg.2414	R006714	(gi:120401976) mce5C (Mvan 0961) - virulence factor Mce family protein [Mycobacterium vanbaalenii str. PYR-1]	62.65	340	351
fig|6666666.28482.peg.2414	R006837	(gi:118464334) mce9C (MAV 0104) - virulence factor Mce family protein [Mycobacterium avium str. 104]	62.68	351	351
fig|6666666.28482.peg.2414	R006836	(gi:41406208) mce9C (MAP0110) - hypothetical protein [Mycobacterium avium str. K-10 (subsp. paratuberculosis)]	62.68	351	351
fig|6666666.28482.peg.2414	R019475	(gi:183980213) mce6C (MMAR 0179) - MCE-family protein Mce6C [Mycobacterium marinum str. M]	60.3	335	351
fig|6666666.28482.peg.2414	R006713	(gi:145220614) mce5C (Mflv 0008) - virulence factor Mce family protein [Mycobacterium gilvum str. PYR-GCK]	62.76	341	351
fig|6666666.28482.peg.2414	R019461	(gi:333988778) mce5C (JDM601 0138) - Mce family protein Mce5C [Mycobacterium sp. str. JDM601]	61.11	342	351
fig|6666666.28482.peg.2415	R006703	(gi:41407950) mce5B (MAP1852) - hypothetical protein [Mycobacterium avium str. K-10 (subsp. paratuberculosis)]	82.91	351	353
fig|6666666.28482.peg.2415	R006704	(gi:118463303) mce5B (MAV 2387) - virulence factor Mce family protein [Mycobacterium avium str. 104]	82.62	351	353
fig|6666666.28482.peg.2415	R019456	(gi:183983840) mce5B (MMAR 3865) - Mce family protein, Mce5B [Mycobacterium marinum str. M]	79.77	351	353

fig|6666666.28482.peg.2415	R006705	(gi:118619037) mce5B (MUL 3797) - Mce family protein, Mce5B [Mycobacterium ulcerans str. Agy99]	79.49	351	353
fig|6666666.28482.peg.2415	R019457	(gi:169628104) mce5B (MAB 1009c) - putative MCE family protein [Mycobacterium abscessus str. ATCC 19977]	69.6	351	353
fig|6666666.28482.peg.2415	R006707	(gi:145220615) mce5B (Mflv 0009) - virulence factor Mce family protein [Mycobacterium gilvum str. PYR-GCK]	67.81	351	353
fig|6666666.28482.peg.2415	R006706	(gi:118472058) mce5B (MSMEG 1144) - virulence factor Mce family protein [Mycobacterium smegmatis str. MC2 155]	66.67	351	353
fig|6666666.28482.peg.2415	R006708	(gi:120401975) mce5B (Mvan 0960) - virulence factor Mce family protein [Mycobacterium vanbaalenii str. PYR-1]	66.1	351	353
fig|6666666.28482.peg.2415	R006834	(gi:41406207) mce9B (MAP0109) - Mce1B [Mycobacterium avium str. K-10 (subsp. paratuberculosis)]	61.84	351	353
fig|6666666.28482.peg.2415	R006835	(gi:118463822) mce9B (MAV 0103) - virulence factor Mce family protein [Mycobacterium avium str. 104]	61.9	349	353
fig|6666666.28482.peg.2415	R019458	(gi:333988779) mce5B (JDM601 0139) - Mce family protein Mce5B [Mycobacterium sp. str. JDM601]	63.79	338	353
fig|6666666.28482.peg.2415	R019473	(gi:183980212) mce6B (MMAR 0178) - MCE-family protein Mce6B [Mycobacterium marinum str. M]	60.63	348	353
fig|6666666.28482.peg.2415	R019474	(gi:169628786) mce6B (MAB 1696) - putative Mce family protein [Mycobacterium abscessus str. ATCC 19977]	60.34	348	353
fig|6666666.28482.peg.2415	R006733	(gi:118619944) mce6B (MUL 4918) - MCE-family protein Mce6B [Mycobacterium ulcerans str. Agy99]	60.53	342	353
fig|6666666.28482.peg.2416	R006697	(gi:41407949) mce5A (MAP1851) - hypothetical protein [Mycobacterium avium str. K-10 (subsp. paratuberculosis)]	87.98	516	526
fig|6666666.28482.peg.2416	R006698	(gi:118466346) mce5A (MAV 2388) - mce related protein [Mycobacterium avium str. 104]	87.79	516	526
fig|6666666.28482.peg.2416	R019453	(gi:183983839) mce5A (MMAR 3864) - Mce protein, Mce5A [Mycobacterium marinum str. M]	71.43	514	526
fig|6666666.28482.peg.2416	R006699	(gi:118619036) mce5A (MUL 3796) - Mce protein, Mce5A [Mycobacterium ulcerans str. Agy99]	71.04	514	526
fig|6666666.28482.peg.2416	R006700	(gi:118472932) mce5A (MSMEG 1143) - mce related protein [Mycobacterium smegmatis str. MC2 155]	64.38	523	526

fig|6666666.28482.peg.2416	R006833	(gi:118465369) mce9A (MAV 0102) - mce related protein [Mycobacterium avium str. 104]	63.84	526	526
fig|6666666.28482.peg.2416	R006702	(gi:120401974) mce5A (Mvan 0959) - Mammalian cell entry related domain protein [Mycobacterium vanbaalenii str. PYR-1]	63.95	515	526
fig|6666666.28482.peg.2416	R019454	(gi:169631642) mce5A (MAB 4568c) - putative Mce family protein [Mycobacterium abscessus str. ATCC 19977]	62.91	510	526
fig|6666666.28482.peg.2416	R019472	(gi:169628785) mce6A (MAB 1695) - putative Mce family protein [Mycobacterium abscessus str. ATCC 19977]	61.11	522	526
fig|6666666.28482.peg.2416	R019455	(gi:333988780) mce5A (JDM601 0140) - Mce family protein Mce5A [Mycobacterium sp. str. JDM601]	62.79	514	526
fig|6666666.28482.peg.2416	R006832	(gi:41406206) mce9A (MAP0108) - hypothetical protein [Mycobacterium avium str. K-10 (subsp. paratuberculosis)]	63.81	509	526
fig|6666666.28482.peg.2416	R006701	(gi:145220616) mce5A (Mflv 0010) - Mammalian cell entry related domain protein [Mycobacterium gilvum str. PYR-GCK]	68.52	413	526
fig|6666666.28482.peg.2432	R018858	(gi:253800873) pks2 (TBMG 03872) - polyketide synthase pks2 [Mycobacterium tuberculosis str. KZN 1435]	68.96	2077	2088
fig|6666666.28482.peg.2432	R005394	(gi:15610961) pks2 (Rv3825c) - PROBABLE POLYKETIDE SYNTHASE PKS2 [Mycobacterium tuberculosis str. H37Rv]	68.96	2077	2088
fig|6666666.28482.peg.2432	R005395	(gi:148663693) pks2 (MRA 3865) - polyketide synthase Pks2 [Mycobacterium tuberculosis str. H37Ra]	68.96	2077	2088
fig|6666666.28482.peg.2432	R005396	(gi:15843449) pks2 (MT3933) - mycocerosic acid synthase [Mycobacterium tuberculosis str. CDC1551]	68.96	2077	2088
fig|6666666.28482.peg.2432	R005397	(gi:31794999) pks2 (Mb3855c) - POLYKETIDE SYNTHASE PKS2 [Mycobacterium bovis str. AF2122/97]	68.96	2077	2088
fig|6666666.28482.peg.2432	R018856	(gi:339633820) pks2 (MAF 38400) - putative polyketide synthase PKS2 [Mycobacterium africanum str. GM041182]	68.96	2077	2088
fig|6666666.28482.peg.2432	R013422	(gi:148825032) pks2 (TBFG 13859) - polyketide synthase pks2 [Mycobacterium tuberculosis str. F11]	68.96	2077	2088
fig|6666666.28482.peg.2432	R018859	(gi:224992238) pks2 (JTY 3890) - polyketide synthase [Mycobacterium bovis str. BCG str. Tokyo 172]	68.91	2077	2088
fig|6666666.28482.peg.2432	R005398	(gi:121639743) pks2 (BCG 3888c) - Polyketide synthase pks2 [Mycobacterium bovis str. BCG Pasteur 1173P2]	68.91	2077	2088

fig|6666666.28482.peg.2432	R018857	(gi:340628794) pks2 (MCAN 38441) - putative polyketide synthase PKS2 [Mycobacterium canettii str. CIPT 140010059]	68.96	2077	2088
fig|6666666.28482.peg.2432	R005400	(gi:118465538) pks2 (MAV 2370) - mycocerosic acid synthase [Mycobacterium avium str. 104]	68.56	2086	2088
fig|6666666.28482.peg.2432	R005399	(gi:41409862) pks2 (MAP3764c) - Pks2 [Mycobacterium avium str. K-10 (subsp. paratuberculosis)]	67.6	2078	2088
fig|6666666.28482.peg.2432	R005402	(gi:108800085) pks2 (Mmcs 3119) - acyl transferase region [Mycobacterium sp. str. MCS]	65.79	2079	2088
fig|6666666.28482.peg.2432	R005401	(gi:119869211) pks2 (Mkms 3179) - acyl transferase domain protein [Mycobacterium sp. str. KMS]	65.79	2079	2088
fig|6666666.28482.peg.2432	R018783	(gi:183981780) mas (MMAR 1767) - multifunctional mycocerosic acid synthase membrane-associated Mas [Mycobacterium marinum str. M]	66.54	2087	2088
fig|6666666.28482.peg.2432	R018785	(gi:340627931) mas (MCAN 29621) - putative multifunctional mycocerosic acid synthase membrane-associated MAS [Mycobacterium canettii str. CIPT 140010059]	65.19	2078	2088
fig|6666666.28482.peg.2432	R018786	(gi:253797972) mas (TBMG 01032) - multifunctional mycocerosic acid synthase membrane-associated mas [Mycobacterium tuberculosis str. KZN 1435]	65.14	2078	2088
fig|6666666.28482.peg.2432	R005270	(gi:15610077) mas (Rv2940c) - PROBABLE MULTIFUNCTIONAL MYCOCEROSIC ACID SYNTHASE MEMBRANE-ASSOCIATED MAS [Mycobacterium tuberculosis str. H37Rv]	65.14	2078	2088
fig|6666666.28482.peg.2432	R005271	(gi:148662786) mas (MRA 2966) - multifunctional mycocerosic acid synthase membrane-associated Mas [Mycobacterium tuberculosis str. H37Ra]	65.14	2078	2088
fig|6666666.28482.peg.2432	R013408	(gi:148824129) mas (TBFG 12954) - multi-functional membrane-associated mycocerosic acid synthase mas [Mycobacterium tuberculosis str. F11]	65.14	2078	2088
fig|6666666.28482.peg.2432	R005272	(gi:15842486) mas (MT3010) - mycocerosic acid synthase [Mycobacterium tuberculosis str. CDC1551]	65.14	2078	2088
fig|6666666.28482.peg.2432	R018784	(gi:339632947) mas (MAF 29450) - putative multifunctional mycocerosic acid synthase membrane-associated MAS [Mycobacterium africanum str. GM041182]	65.05	2078	2088

fig|6666666.28482.peg.2432	R018787	(gi:224991314) mas (JTY 2957) - putative multifunctional mycocerosic acid synthase membrane-associated [Mycobacterium bovis str. BCG str. Tokyo 172]	65.1	2078	2088
fig|6666666.28482.peg.2432	R005274	(gi:121638822) mas (BCG 2962c) - putative multifunctional mycocerosic acid synthase membrane-associated mas [Mycobacterium bovis str. BCG Pasteur 1173P2]	65.1	2078	2088
fig|6666666.28482.peg.2432	R005273	(gi:31794117) mas (Mb2965c) - PROBABLE MULTIFUNCTIONAL MYCOCEROSIC ACID SYNTHASE MEMBRANE-ASSOCIATED MAS [Mycobacterium bovis str. AF2122/97]	65.1	2078	2088
fig|6666666.28482.peg.2432	R005276	(gi:118617588) mas (MUL 2010) - multifunctional mycocerosic acid synthase membrane-associated Mas [Mycobacterium ulcerans str. Agy99]	66.44	2087	2088
fig|6666666.28482.peg.2432	R005275	(gi:15826970) mas (ML0139) - putative mycocerosic synthase [Mycobacterium leprae str. TN]	63.83	2088	2088
fig|6666666.28482.peg.2432	R018788	(gi:221229448) mas (MLBr 00139) - putative mycocerosic synthase [Mycobacterium leprae str. Br4923]	63.83	2088	2088
fig|6666666.28482.peg.2439	R018735	(gi:183981778) fadD28 (MMAR 1765) - acyl-CoA synthetase [Mycobacterium marinum str. M]	64.21	583	584
fig|6666666.28482.peg.2439	R005202	(gi:118617586) fadD28 (MUL 2008) - fatty-acyl AMP ligase FadD28 [Mycobacterium ulcerans str. Agy99]	63.87	583	584
fig|6666666.28482.peg.2439	R005199	(gi:41409850) fadD28 (MAP3752) - acyl-CoA synthase [Mycobacterium avium str. K-10 (subsp. paratuberculosis)]	63.14	583	584
fig|6666666.28482.peg.2439	R005197	(gi:31794118) fadD28 (Mb2966) - acyl-CoA synthase [Mycobacterium bovis str. AF2122/97]	63.93	584	584
fig|6666666.28482.peg.2439	R018738	(gi:253797971) fadD28 (TBMG 01031) - acyl-CoA synthetase [Mycobacterium tuberculosis str. KZN 1435]	63.93	584	584
fig|6666666.28482.peg.2439	R005194	(gi:15610078) fadD28 (Rv2941) - acyl-CoA synthase [Mycobacterium tuberculosis str. H37Rv]	63.93	584	584
fig|6666666.28482.peg.2439	R005195	(gi:148662787) fadD28 (MRA 2967) - fatty-acid-CoA ligase FadD28 [Mycobacterium tuberculosis str. H37Ra]	63.93	584	584
fig|6666666.28482.peg.2439	R013401	(gi:148824130) fadD28 (TBFG 12955) - fatty-acid-CoA ligase fadD28 [Mycobacterium tuberculosis str. F11]	63.93	584	584
fig|6666666.28482.peg.2439	R005196	(gi:15842487) fadD28 (MT3011) - acyl-CoA synthase [Mycobacterium tuberculosis str. CDC1551]	63.93	584	584

fig|6666666.28482.peg.2439	R018736	(gi:339632948) fadD28 (MAF 29460) - fatty-acid-CoA ligase FADD28 (fatty-acid-CoA synthetase) [Mycobacterium africanum str. GM041182]	63.93	584	584
fig|6666666.28482.peg.2439	R005201	(gi:15826969) fadD28 (ML0138) - acyl-CoA synthase [Mycobacterium leprae str. TN]	63.2	584	584
fig|6666666.28482.peg.2439	R018740	(gi:221229447) fadD28 (MLBr 00138) - acyl-CoA synthetase [Mycobacterium leprae str. Br4923]	63.2	584	584
fig|6666666.28482.peg.2439	R018737	(gi:340627932) fadD28 (MCAN 29631) - fatty-acid-CoA ligase FADD28 [Mycobacterium canettii str. CIPT 140010059]	63.76	584	584
fig|6666666.28482.peg.2439	R018739	(gi:224991315) fadD28 (JTY 2958) - acyl-CoA synthetase [Mycobacterium bovis str. BCG str. Tokyo 172]	63.93	584	584
fig|6666666.28482.peg.2439	R005198	(gi:121638823) fadD28 (BCG 2963) - fatty-acid-CoA ligase fadD28 [Mycobacterium bovis str. BCG Pasteur 1173P2]	63.93	584	584
fig|6666666.28482.peg.2453	R005338	(gi:41407332) MAP1234 - hypothetical protein [Mycobacterium avium str. K-10 (subsp. paratuberculosis)]	70.76	234	257
fig|6666666.28482.peg.2453	R018821	(gi:253797953) TBMG01014 - glycosyltransferase [Mycobacterium tuberculosis str. KZN 1435]	72.48	217	257
fig|6666666.28482.peg.2453	R005333	(gi:15610094) Rv2957 - POSSIBLE GLYCOSYL TRANSFERASE [Mycobacterium tuberculosis str. H37Rv]	72.48	217	257
fig|6666666.28482.peg.2453	R005334	(gi:148662804) MRA2984 - glycosyl transferase [Mycobacterium tuberculosis str. H37Ra]	72.48	217	257
fig|6666666.28482.peg.2453	R013414	(gi:148824146) TBFG12971 - hypothetical glycosyl transferase [Mycobacterium tuberculosis str. F11]	72.48	217	257
fig|6666666.28482.peg.2453	R018820	(gi:340627946) MCAN29771 - putative GLYCOSYL transferase [Mycobacterium canettii str. CIPT 140010059]	72.48	217	257
fig|6666666.28482.peg.2453	R018822	(gi:224991330) JTY2973 - putative glycosyl transferase [Mycobacterium bovis str. BCG str. Tokyo 172]	72.48	217	257
fig|6666666.28482.peg.2453	R005337	(gi:121638838) BCG2978 - putative glycosyl transferase [Mycobacterium bovis str. BCG Pasteur 1173P2]	72.48	217	257
fig|6666666.28482.peg.2453	R005336	(gi:31794133) Mb2981 - POSSIBLE GLYCOSYL TRANSFERASE [Mycobacterium bovis str. AF2122/97]	72.48	217	257
fig|6666666.28482.peg.2453	R018819	(gi:339632964) MAF29620 - putative glycosyl transferase [Mycobacterium africanum str. GM041182]	72.48	217	257

fig|6666666.28482.peg.2453	R005335	(gi:15842506) MT3031 - glycosyl transferase [Mycobacterium tuberculosis str. CDC1551]	72.48	217	257
fig|6666666.28482.peg.2453	R005339	(gi:118467159) MAV3253 - hypothetical protein [Mycobacterium avium str. 104]	69.62	156	257
fig|6666666.28482.peg.2533	R018887	(gi:169631477) hspX (MAB 4402) - heat shock protein Hsp20 [Mycobacterium abscessus str. ATCC 19977]	62.59	145	146
fig|6666666.28482.peg.2590	R005681	(gi:15843099) lipF (MT3591) - esterase [Mycobacterium tuberculosis str. CDC1551]	71.43	294	295
fig|6666666.28482.peg.2590	R018937	(gi:340628455) lipF (MCAN 35011) - putative esterase/lipase LIPF [Mycobacterium canettii str. CIPT 140010059]	73.6	250	295
fig|6666666.28482.peg.2590	R018938	(gi:253800530) lipF (TBMG 03533) - esterase/lipase lipF [Mycobacterium tuberculosis str. KZN 1435]	73.6	250	295
fig|6666666.28482.peg.2590	R005679	(gi:15610623) lipF (Rv3487c) - PROBABLE ESTERASE/LIPASE LIPF [Mycobacterium tuberculosis str. H37Rv]	73.6	250	295
fig|6666666.28482.peg.2590	R005680	(gi:148663351) lipF (MRA 3527) - esterase LipF [Mycobacterium tuberculosis str. H37Ra]	73.6	250	295
fig|6666666.28482.peg.2590	R013445	(gi:148824695) lipF (TBFG 13522) - esterase/lipase lipF [Mycobacterium tuberculosis str. F11]	73.6	250	295
fig|6666666.28482.peg.2590	R018939	(gi:224991902) lipF (JTY 3551) - putative esterase/lipase [Mycobacterium bovis str. BCG str. Tokyo 172]	73.6	250	295
fig|6666666.28482.peg.2590	R005683	(gi:121639406) lipF (BCG 3551c) - putative esterase/lipase lipF [Mycobacterium bovis str. BCG Pasteur 1173P2]	73.6	250	295
fig|6666666.28482.peg.2590	R005682	(gi:31794663) lipF (Mb3517c) - PROBABLE ESTERASE/LIPASE LIPF [Mycobacterium bovis str. AF2122/97]	73.6	250	295
fig|6666666.28482.peg.2590	R018936	(gi:339633490) lipF (MAF 35000) - putative esterase/lipase LIPF [Mycobacterium africanum str. GM041182]	73.6	250	295
fig|6666666.28482.peg.2628	R006193	(gi:41410371) whiB3 (MAP4273c) - WhiB3 [Mycobacterium avium str. K-10 (subsp. paratuberculosis)]	95.1	102	102
fig|6666666.28482.peg.2628	R019244	(gi:183981153) whiB3 (MMAR 1132) - WhiB-like regulatory protein, WhiB3 [Mycobacterium marinum str. M]	94.12	102	102
fig|6666666.28482.peg.2628	R006196	(gi:118616663) whiB3 (MUL 0895) - Whib-like regulatory protein, WhiB3 [Mycobacterium ulcerans str. Agy99]	93.14	102	102

fig|6666666.28482.peg.2628	R019248	(gi:253800463) whiB3 (TBMG 03467) - transcriptional regulator whib-like whiB3 [Mycobacterium tuberculosis str. KZN 1435]	91.18	102	102
fig|6666666.28482.peg.2628	R006188	(gi:15610552) whiB3 (Rv3416) - TRANSCRIPTIONAL REGULATORY PROTEIN WHIB-LIKE WHIB3 [Mycobacterium tuberculosis str. H37Rv]	91.18	102	102
fig|6666666.28482.peg.2628	R006189	(gi:148663280) whiB3 (MRA 3456) - transcriptional regulatory protein whib-like WhiB3 [Mycobacterium tuberculosis str. H37Ra]	91.18	102	102
fig|6666666.28482.peg.2628	R013502	(gi:148824623) whiB3 (TBFG 13450) - transcriptional regulatory protein whib-like whiB3 [Mycobacterium tuberculosis str. F11]	91.18	102	102
fig|6666666.28482.peg.2628	R006190	(gi:15843012) whiB3 (MT3525) - WhiB-related protein [Mycobacterium tuberculosis str. CDC1551]	91.18	102	102
fig|6666666.28482.peg.2628	R019247	(gi:340628394) whiB3 (MCAN 34401) - transcriptional regulatory protein WHIB-like WHIB3 [Mycobacterium canettii str. CIPT 140010059]	91.18	102	102
fig|6666666.28482.peg.2628	R019249	(gi:224991837) whiB3 (JTY 3486) - transcriptional regulatory protein whiB-like [Mycobacterium bovis str. BCG str. Tokyo 172]	91.18	102	102
fig|6666666.28482.peg.2628	R006192	(gi:121639341) whiB3 (BCG 3486) - Transcriptional regulatory protein whiB-like whib3 [Mycobacterium bovis str. BCG Pasteur 1173P2]	91.18	102	102
fig|6666666.28482.peg.2628	R006191	(gi:31794597) whiB3 (Mb3450) - TRANSCRIPTIONAL REGULATORY PROTEIN WHIB-LIKE WHIB3 [Mycobacterium bovis str. AF2122/97]	91.18	102	102
fig|6666666.28482.peg.2628	R019246	(gi:339633421) whiB3 (MAF 34300) - transcriptional regulatory protein WHIB-like WHIB3 [Mycobacterium africanum str. GM041182]	91.18	102	102
fig|6666666.28482.peg.2628	R006195	(gi:15827111) whiB3 (ML0382) - putative transcriptional regulator [Mycobacterium leprae str. TN]	89.22	102	102
fig|6666666.28482.peg.2628	R019250	(gi:221229589) whiB3 (MLBr 00382) - putative transcriptional regulator [Mycobacterium leprae str. Br4923]	89.22	102	102
fig|6666666.28482.peg.2628	R006199	(gi:120402515) whiB3 (Mvan 1506) - transcription factor WhiB [Mycobacterium vanbaalenii str. PYR-1]	82.35	102	102
fig|6666666.28482.peg.2628	R006202	(gi:108798133) whiB3 (Mmcs 1162) - transcription factor WhiB [Mycobacterium sp. str. MCS]	82.35	102	102
fig|6666666.28482.peg.2628	R006201	(gi:119867229) whiB3 (Mkms 1179) - transcription factor WhiB [Mycobacterium sp. str. KMS]	82.35	102	102
fig|6666666.28482.peg.2628	R006200	(gi:126433792) whiB3 (Mjls 1189) - transcription factor WhiB [Mycobacterium sp. str. JLS]	82.35	102	102

fig|6666666.28482.peg.2628	R006198	(gi:145225490) whiB3 (Mflv 4914) - transcription factor WhiB [Mycobacterium gilvum str. PYR-GCK]	80.39	102	102
fig|6666666.28482.peg.2628	R006197	(gi:118471283) whiB3 (MSMEG 1597) - Transcription factor WhiB [Mycobacterium smegmatis str. MC2 155]	81.37	102	102
fig|6666666.28482.peg.2628	R019245	(gi:169630805) whiB3 (MAB 3726) - WhiB family transcriptional regulator [Mycobacterium abscessus str. ATCC 19977]	71.57	102	102
fig|6666666.28482.peg.2628	R006194	(gi:118462777) whiB3 (MAV 4361) - Transcription factor WhiB [Mycobacterium avium str. 104]	92.86	84	102
fig|6666666.28482.peg.2793	R019164	(gi:169630604) narK2 (MAB 3523c) - integral membrane nitrite extrusion protein NarK3 [Mycobacterium abscessus str. ATCC 19977]	76.36	461	481
fig|6666666.28482.peg.2804	R006094	(gi:118466410) sodC (MAV 4722) - superoxide dismutase, Cu-Zn [Mycobacterium avium str. 104]	72.77	235	235
fig|6666666.28482.peg.2804	R006093	(gi:41410019) sodC (MAP3921) - SodC [Mycobacterium avium str. K-10 (subsp. paratuberculosis)]	72.77	235	235
fig|6666666.28482.peg.2804	R019187	(gi:340625457) sodC (MCAN 04301) - putative periplasmic superoxide dismutase [Cu-Zn] SODC [Mycobacterium canettii str. CIPT 140010059]	67.08	235	235
fig|6666666.28482.peg.2804	R019188	(gi:253797356) sodC (TBMG 00433) - periplasmic superoxide dismutase [Cu-Zn] sodC [Mycobacterium tuberculosis str. KZN 1435]	66.67	235	235
fig|6666666.28482.peg.2804	R006088	(gi:15607573) sodC (Rv0432) - PROBABLE PERIPLASMIC SUPEROXIDE DISMUTASE [CU-ZN] SODC [Mycobacterium tuberculosis str. H37Rv]	66.67	235	235
fig|6666666.28482.peg.2804	R006089	(gi:148660197) sodC (MRA 0437) - superoxide dismutase [Mycobacterium tuberculosis str. H37Ra]	66.67	235	235
fig|6666666.28482.peg.2804	R013480	(gi:148821628) sodC (TBFG 10437) - periplasmic superoxide dismutase [Cu-Zn] sodC [Mycobacterium tuberculosis str. F11]	66.67	235	235
fig|6666666.28482.peg.2804	R006090	(gi:15839819) sodC (MT0447) - superoxide dismutase [Mycobacterium tuberculosis str. CDC1551]	66.67	235	235
fig|6666666.28482.peg.2804	R019189	(gi:224988818) sodC (JTY 0441) - putative periplasmic Cu-Zn-superoxide dismutase [Mycobacterium bovis str. BCG str. Tokyo 172]	66.67	235	235
fig|6666666.28482.peg.2804	R006092	(gi:121636346) sodC (BCG 0471) - putative periplasmic superoxide dismutase [Cu-Zn] sodC [Mycobacterium bovis str. BCG Pasteur 1173P2]	66.67	235	235
fig|6666666.28482.peg.2804	R006091	(gi:31791610) sodC (Mb0440) - PROBABLE PERIPLASMIC SUPEROXIDE DISMUTASE [CU-ZN] SODC [Mycobacterium bovis str. AF2122/97]	66.67	235	235

fig|6666666.28482.peg.2804	R019186	(gi:339630501) sodC (MAF 04340) - putative periplasmic superoxide dismutase [Cu-Zn] SODC [Mycobacterium africanum str. GM041182]	66.67	235	235
fig|6666666.28482.peg.2804	R006095	(gi:15828035) sodC (ML1925) - superoxide dismutase precursor (Cu-Zn) [Mycobacterium leprae str. TN]	68.22	231	235
fig|6666666.28482.peg.2804	R019190	(gi:221230512) sodC (MLBr 01925) - superoxide dismutase precursor (Cu-Zn) [Mycobacterium leprae str. Br4923]	68.22	231	235
fig|6666666.28482.peg.2804	R006096	(gi:118617063) sodC (MUL 1379) - periplasmic superoxide dismutase [Cu-Zn] SodC [Mycobacterium ulcerans str. Agy99]	68.44	235	235
fig|6666666.28482.peg.2804	R019184	(gi:183980771) sodC (MMAR 0747) - periplasmic superoxide dismutase [Cu-Zn] SodC [Mycobacterium marinum str. M]	68.03	235	235
fig|6666666.28482.peg.2804	R006097	(gi:118473568) sodC (MSMEG 0835) - copper/zinc superoxide dismutase [Mycobacterium smegmatis str. MC2 155]	63.6	223	235
fig|6666666.28482.peg.2804	R019191	(gi:333989046) sodC (JDM601 0406) - periplasmic superoxide dismutase [Mycobacterium sp. str. JDM601]	62.56	215	235
fig|6666666.28482.peg.2804	R006102	(gi:108797552) sodC (Mmcs 0572) - superoxide dismutase, copper/zinc binding protein [Mycobacterium sp. str. MCS]	61.4	223	235
fig|6666666.28482.peg.2804	R006101	(gi:119866638) sodC (Mkms 0584) - superoxide dismutase, copper/zinc binding [Mycobacterium sp. str. KMS]	61.4	223	235
fig|6666666.28482.peg.2804	R006100	(gi:126433174) sodC (Mjls 0562) - superoxide dismutase, copper/zinc binding [Mycobacterium sp. str. JLS]	61.4	223	235
fig|6666666.28482.peg.2804	R006099	(gi:120401750) sodC (Mvan 0735) - superoxide dismutase, copper/zinc binding [Mycobacterium vanbaalenii str. PYR-1]	60.85	231	235
fig|6666666.28482.peg.2826	R008500	(gi:126697767) groEL (CD0194) - 60 kDa chaperonin [Clostridium difficile str. 630]	64.45	524	541
fig|6666666.28482.peg.2826	R008508	(gi:125975373) groEL (Cthe 2892) - chaperonin GroEL [Clostridium thermocellum str. ATCC 27405]	63.5	524	541
fig|6666666.28482.peg.2826	R008501	(gi:118444628) groEL (NT01CX 0462) - chaperonin GroEL [Clostridium novyi str. NT]	63.81	524	541
fig|6666666.28482.peg.2826	R008506	(gi:15895960) groEL (CAC2703) - chaperonin GroEL [Clostridium acetobutylicum str. ATCC 824]	64.38	524	541
fig|6666666.28482.peg.2826	R008502	(gi:18311271) groEL (CPE2289) - chaperonin GroEL [Clostridium perfringens str. 13]	61.21	540	541

fig|6666666.28482.peg.2826	R008503	(gi:110799227) groEL (CPF 2573) - chaperonin, 60 kDa [Clostridium perfringens str. ATCC 13124]	61.03	540	541
fig|6666666.28482.peg.2826	R015473	(gi:170760184) groEL (CLK 2714) - chaperonin GroEL [Clostridium botulinum str. Loch Maree (serotype A3)]	61.67	539	541
fig|6666666.28482.peg.2826	R008507	(gi:150015219) groEL (Cbei 0329) - chaperonin GroEL [Clostridium beijerinckii str. NCIMB 8052]	60.93	539	541
fig|6666666.28482.peg.2826	R008504	(gi:28212000) groEL (CTC02413) - chaperonin GroEL [Clostridium tetani str. E88]	60.93	539	541
fig|6666666.28482.peg.2826	R015474	(gi:170756380) groEL (CLD 1225) - chaperonin GroEL [Clostridium botulinum str. Okra (serotype B1)]	61.48	539	541
fig|6666666.28482.peg.2826	R015476	(gi:153941100) groEL (CLI 3468) - chaperonin GroEL [Clostridium botulinum str. Langeland (serotype F)]	61.48	539	541
fig|6666666.28482.peg.2826	R015472	(gi:153935823) groEL (CLC 3240) - chaperonin GroEL [Clostridium botulinum str. Hall (serotype A)]	61.48	539	541
fig|6666666.28482.peg.2826	R008505	(gi:148381241) groEL (CBO3298) - 60 kDa chaperonin GroEL [Clostridium botulinum str. ATCC 3502 (serotype A)]	61.48	539	541
fig|6666666.28482.peg.2826	R015471	(gi:153934242) groEL (CLB 3354) - chaperonin GroEL [Clostridium botulinum str. ATCC 19397 (serotype A)]	61.48	539	541
fig|6666666.28482.peg.2857	R018932	(gi:253797394) icl (TBMG 00469) - isocitrate lyase icl [Mycobacterium tuberculosis str. KZN 1435]	95.56	428	428
fig|6666666.28482.peg.2857	R005664	(gi:57116734) icl (Rv0467) - isocitrate lyase [Mycobacterium tuberculosis str. H37Rv]	95.56	428	428
fig|6666666.28482.peg.2857	R005665	(gi:148660233) icl (MRA 0473) - isocitrate lyase Icl [Mycobacterium tuberculosis str. H37Ra]	95.56	428	428
fig|6666666.28482.peg.2857	R013444	(gi:148821665) icl (TBFG 10474) - isocitrate lyase icl [Mycobacterium tuberculosis str. F11]	95.56	428	428
fig|6666666.28482.peg.2857	R005666	(gi:15839856) icl (MT0483) - isocitrate lyase [Mycobacterium tuberculosis str. CDC1551]	95.56	428	428
fig|6666666.28482.peg.2857	R018931	(gi:340625493) icl (MCAN 04661) - isocitrate lyase ICL [Mycobacterium canettii str. CIPT 140010059]	95.56	428	428
fig|6666666.28482.peg.2857	R018933	(gi:224988854) icl (JTY 0477) - isocitrate lyase [Mycobacterium bovis str. BCG str. Tokyo 172]	95.56	428	428

fig|6666666.28482.peg.2857	R005668	(gi:121636382) icl (BCG 0507) - Isocitrate lyase icl [Mycobacterium bovis str. BCG Pasteur 1173P2]	95.56	428	428
fig|6666666.28482.peg.2857	R005667	(gi:31791646) icl (Mb0476) - isocitrate lyase [Mycobacterium bovis str. AF2122/97]	95.56	428	428
fig|6666666.28482.peg.2857	R018930	(gi:339630536) icl (MAF 04700) - isocitrate lyase Icl [Mycobacterium africanum str. GM041182]	95.56	428	428
fig|6666666.28482.peg.2857	R005670	(gi:118462260) icl (MAV 4682) - isocitrate lyase [Mycobacterium avium str. 104]	93.69	428	428
fig|6666666.28482.peg.2857	R005669	(gi:41410059) icl (MAP3961) - isocitrate lyase [Mycobacterium avium str. K-10 (subsp. paratuberculosis)]	93.44	427	428
fig|6666666.28482.peg.2857	R018935	(gi:333989098) icl (JDM601 0458) - isocitrate lyase Icl [Mycobacterium sp. str. JDM601]	92.76	428	428
fig|6666666.28482.peg.2857	R005673	(gi:118471509) icl (MSMEG 0911) - isocitrate lyase [Mycobacterium smegmatis str. MC2 155]	92.52	428	428
fig|6666666.28482.peg.2857	R005676	(gi:126433240) icl (Mjls 0629) - isocitrate lyase [Mycobacterium sp. str. JLS]	92.52	428	428
fig|6666666.28482.peg.2857	R018928	(gi:183980816) icl (MMAR 0792) - isocitrate lyase Icl [Mycobacterium marinum str. M]	92.06	428	428
fig|6666666.28482.peg.2857	R005675	(gi:120401816) icl (Mvan 0801) - isocitrate lyase [Mycobacterium vanbaalenii str. PYR-1]	92.06	428	428
fig|6666666.28482.peg.2857	R005674	(gi:145220712) icl (Mflv 0107) - isocitrate lyase [Mycobacterium gilvum str. PYR-GCK]	91.59	428	428
fig|6666666.28482.peg.2857	R005678	(gi:108797616) icl (Mmcs 0636) - isocitrate lyase [Mycobacterium sp. str. MCS]	92.06	428	428
fig|6666666.28482.peg.2857	R005677	(gi:119866703) icl (Mkms 0649) - isocitrate lyase [Mycobacterium sp. str. KMS]	92.06	428	428
fig|6666666.28482.peg.2857	R005672	(gi:118619631) icl (MUL 4536) - isocitrate lyase Icl [Mycobacterium ulcerans str. Agy99]	91.59	428	428
fig|6666666.28482.peg.2857	R018929	(gi:169631173) icl (MAB 4095c) - isocitrate lyase (AceA) [Mycobacterium abscessus str. ATCC 19977]	83.49	428	428
fig|6666666.28482.peg.2859	R005153	(gi:145223891) pcaA (Mflv 3304) - Cyclopropane-fatty-acyl-phospholipid synthase [Mycobacterium gilvum str. PYR-GCK]	60.78	283	286

fig|6666666.28482.peg.2859	R005167	(gi:108797926) cmaA2 (Mmcs 0952) - Cyclopropane-fatty-acyl-phospholipid synthase [Mycobacterium sp. str. MCS]	61.13	283	286
fig|6666666.28482.peg.2860	R005152	(gi:118619634) pcaA (MUL 4539) - mycolic acid synthase PcaA [Mycobacterium ulcerans str. Agy99]	87.11	287	287
fig|6666666.28482.peg.2860	R018707	(gi:183980820) pcaA (MMAR 0796) - mycolic acid synthase PcaA [Mycobacterium marinum str. M]	87.11	287	287
fig|6666666.28482.peg.2860	R005151	(gi:15828331) pcaA (ML2459) - Mycolic acid synthase [Mycobacterium leprae str. TN]	83.97	287	287
fig|6666666.28482.peg.2860	R018712	(gi:221230808) pcaA (MLBr 02459) - Mycolic acid synthase [Mycobacterium leprae str. Br4923]	83.97	287	287
fig|6666666.28482.peg.2860	R018710	(gi:253797397) pcaA (TBMG 00472) - mycolic acid synthase PcaA [Mycobacterium tuberculosis str. KZN 1435]	82.93	287	287
fig|6666666.28482.peg.2860	R005144	(gi:57116736) pcaA (Rv0470c) - MYCOLIC ACID SYNTHASE PCAA (CYCLOPROPANE SYNTHASE) [Mycobacterium tuberculosis str. H37Rv]	82.93	287	287
fig|6666666.28482.peg.2860	R005145	(gi:148660236) pcaA (MRA 0476) - mycolic acid synthase PcaA [Mycobacterium tuberculosis str. H37Ra]	82.93	287	287
fig|6666666.28482.peg.2860	R013397	(gi:148821668) pcaA (TBFG 10477) - mycolic acid synthase pcaA (cyclopropane synthase) [Mycobacterium tuberculosis str. F11]	82.93	287	287
fig|6666666.28482.peg.2860	R005146	(gi:15839858) pcaA (MT0486) - mycolic acid synthase [Mycobacterium tuberculosis str. CDC1551]	82.93	287	287
fig|6666666.28482.peg.2860	R018709	(gi:340625496) pcaA (MCAN 04691) - mycolic acid synthase PCAA [Mycobacterium canettii str. CIPT 140010059]	82.93	287	287
fig|6666666.28482.peg.2860	R005148	(gi:121636385) pcaA (BCG 0510c) - Mycolic acid synthase pcaA [Mycobacterium bovis str. BCG Pasteur 1173P2]	82.93	287	287
fig|6666666.28482.peg.2860	R005147	(gi:31791649) pcaA (Mb0479c) - MYCOLIC ACID SYNTHASE PCAA (CYCLOPROPANE SYNTHASE) [Mycobacterium bovis str. AF2122/97]	82.93	287	287
fig|6666666.28482.peg.2860	R018708	(gi:339630539) pcaA (MAF 04730) - mycolic acid synthase PCAA (cyclopropane synthase) [Mycobacterium africanum str. GM041182]	82.93	287	287
fig|6666666.28482.peg.2860	R018711	(gi:224988857) pcaA (JTY 0480) - mycolic acid synthase [Mycobacterium bovis str. BCG str. Tokyo 172]	82.93	287	287
fig|6666666.28482.peg.2860	R005149	(gi:41410062) pcaA (MAP3964c) - UmaA2 [Mycobacterium avium str. K-10 (subsp. paratuberculosis)]	81.18	287	287

fig|6666666.28482.peg.2860	R005150	(gi:118465677) pcaA (MAV 4679) - cyclopropane-fatty-acyl-phospholipid synthase 1 [Mycobacterium avium str. 104]	81.05	285	287
fig|6666666.28482.peg.2860	R005167	(gi:108797926) cmaA2 (Mmcs 0952) - Cyclopropane-fatty-acyl-phospholipid synthase [Mycobacterium sp. str. MCS]	72.03	286	287
fig|6666666.28482.peg.2860	R005157	(gi:108799701) pcaA (Mmcs 2735) - Cyclopropane-fatty-acyl-phospholipid synthase [Mycobacterium sp. str. MCS]	71.73	283	287
fig|6666666.28482.peg.2860	R005156	(gi:119868811) pcaA (Mkms 2779) - Cyclopropane-fatty-acyl-phospholipid synthase [Mycobacterium sp. str. KMS]	71.73	283	287
fig|6666666.28482.peg.2860	R005153	(gi:145223891) pcaA (Mflv 3304) - Cyclopropane-fatty-acyl-phospholipid synthase [Mycobacterium gilvum str. PYR-GCK]	71.33	286	287
fig|6666666.28482.peg.2860	R005155	(gi:126435345) pcaA (Mjls 2765) - Cyclopropane-fatty-acyl-phospholipid synthase [Mycobacterium sp. str. JLS]	71.73	283	287
fig|6666666.28482.peg.2860	R005154	(gi:120404005) pcaA (Mvan 3025) - Cyclopropane-fatty-acyl-phospholipid synthase [Mycobacterium vanbaalenii str. PYR-1]	69.93	286	287
fig|6666666.28482.peg.2860	R018720	(gi:333990669) cmaA2 (JDM601 2029) - methoxy mycolic acid synthase [Mycobacterium sp. str. JDM601]	61.03	285	287
fig|6666666.28482.peg.2860	R018713	(gi:333989092) pcaA (JDM601 0452) - mycolic acid synthase PcaA [Mycobacterium sp. str. JDM601]	63.54	276	287
fig|6666666.28482.peg.2866	R005134	(gi:41410066) hbhA (MAP3968) - hypothetical protein [Mycobacterium avium str. K-10 (subsp. paratuberculosis)]	90.62	160	197
fig|6666666.28482.peg.2866	R005129	(gi:15607616) hbhA (Rv0475) - IRON-REGULATED HEPARIN BINDING HEMAGGLUTININ HBHA (ADHESIN) [Mycobacterium tuberculosis str. H37Rv]	89.31	159	197
fig|6666666.28482.peg.2866	R005130	(gi:148660242) hbhA (MRA 0482) - iron-regulated heparin binding hemagglutinin Hbha [Mycobacterium tuberculosis str. H37Ra]	89.31	159	197
fig|6666666.28482.peg.2866	R013396	(gi:148821674) hbhA (TBFG 10483) - iron-regulated heparin binding hemagglutinin hbhA (adhesin) [Mycobacterium tuberculosis str. F11]	89.31	159	197
fig|6666666.28482.peg.2866	R018702	(gi:340625501) hbhA (MCAN 04751) - iron-regulated heparin binding hemagglutinin HBHA (adhesin) [Mycobacterium canettii str. CIPT 140010059]	89.31	159	197
fig|6666666.28482.peg.2866	R018704	(gi:224988863) hbhA (JTY 0486) - heparin binding hemagglutinin [Mycobacterium bovis str. BCG str. Tokyo 172]	89.31	159	197

fig|6666666.28482.peg.2866	R005133	(gi:121636391) hbhA (BCG 0516) - Heparin binding hemagglutinin hbhA [Mycobacterium bovis str. BCG Pasteur 1173P2]	89.31	159	197
fig|6666666.28482.peg.2866	R005132	(gi:31791655) hbhA (Mb0485) - HEPARIN BINDING HEMAGGLUTININ HBHA (ADHESIN) [Mycobacterium bovis str. AF2122/97]	89.31	159	197
fig|6666666.28482.peg.2866	R018701	(gi:339630545) hbhA (MAF 04790) - iron-regulated heparin binding hemagglutinin HBHA (adhesin) [Mycobacterium africanum str. GM041182]	89.31	159	197
fig|6666666.28482.peg.2866	R005137	(gi:118619638) hbhA (MUL 4543) - iron-regulated heparin binding hemagglutinin HbhA [Mycobacterium ulcerans str. Agy99]	89.02	164	197
fig|6666666.28482.peg.2866	R018699	(gi:183980824) hbhA (MMAR 0800) - iron-regulated heparin binding hemagglutinin HbhA [Mycobacterium marinum str. M]	89.63	164	197
fig|6666666.28482.peg.2866	R018703	(gi:253797403) hbhA (TBMG 00478) - iron-regulated heparin binding hemagglutinin hbhA [Mycobacterium tuberculosis str. KZN 1435]	89.31	159	197
fig|6666666.28482.peg.2866	R005135	(gi:118463469) hbhA (MAV 4675) - heparin binding hemagglutinin hbha [Mycobacterium avium str. 104]	90	160	197
fig|6666666.28482.peg.2866	R005131	(gi:15839865) hbhA (MT0493) - hypothetical protein [Mycobacterium tuberculosis str. CDC1551]	88.68	159	197
fig|6666666.28482.peg.2866	R018706	(gi:333989103) hbhA (JDM601 0463) - iron-regulated heparin binding hemagglutinin HbhA [Mycobacterium sp. str. JDM601]	73.33	165	197
fig|6666666.28482.peg.2866	R005136	(gi:15828329) hbhA (ML2454) - possible hemagglutinin. [Mycobacterium leprae str. TN]	84.91	159	197
fig|6666666.28482.peg.2866	R018705	(gi:221230806) hbhA (MLBr 02454) - putative hemagglutinin [Mycobacterium leprae str. Br4923]	84.91	159	197
fig|6666666.28482.peg.2866	R005143	(gi:108797623) hbhA (Mmcs 0643) - hypothetical protein [Mycobacterium sp. str. MCS]	71.86	164	197
fig|6666666.28482.peg.2866	R005142	(gi:119866710) hbhA (Mkms 0656) - hypothetical protein [Mycobacterium sp. str. KMS]	71.86	164	197
fig|6666666.28482.peg.2866	R005141	(gi:126433247) hbhA (Mjls 0636) - hypothetical protein [Mycobacterium sp. str. JLS]	71.86	164	197
fig|6666666.28482.peg.2866	R005139	(gi:145220706) hbhA (Mflv 0100) - hypothetical protein [Mycobacterium gilvum str. PYR-GCK]	69.82	166	197
fig|6666666.28482.peg.2866	R005140	(gi:120401823) hbhA (Mvan 0808) - hypothetical protein [Mycobacterium vanbaalenii str. PYR-1]	69.68	155	197

fig|6666666.28482.peg.2866	R005138	(gi:118468465) hbhA (MSMEG 0919) - heparin-binding hemagglutinin [Mycobacterium smegmatis str. MC2 155]	66.67	159	197
fig|6666666.28482.peg.2866	R018700	(gi:169631161) hbhA (MAB 4083c) - Heparin-binding hemagglutinin (adhesin) [Mycobacterium abscessus str. ATCC 19977]	60.26	156	197
fig|6666666.28482.peg.2891	R005750	(gi:118463455) proC (MAV 4652) - pyrroline-5-carboxylate reductase [Mycobacterium avium str. 104]	86.6	290	290
fig|6666666.28482.peg.2891	R005749	(gi:41410089) proC (MAP3991) - pyrroline-5-carboxylate reductase [Mycobacterium avium str. K-10 (subsp. paratuberculosis)]	85.91	290	290
fig|6666666.28482.peg.2891	R005751	(gi:15828312) proC (ML2430) - pyrroline-5-carboxylate reductase [Mycobacterium leprae str. TN]	81.44	290	290
fig|6666666.28482.peg.2891	R018986	(gi:221230789) proC (MLBr 02430) - pyrroline-5-carboxylate reductase [Mycobacterium leprae str. Br4923]	81.44	290	290
fig|6666666.28482.peg.2891	R018980	(gi:183980850) proC (MMAR 0826) - pyrroline-5-carboxylate reductase [Mycobacterium marinum str. M]	81.23	277	290
fig|6666666.28482.peg.2891	R005752	(gi:118619662) proC (MUL 4570) - pyrroline-5-carboxylate reductase ProC [Mycobacterium ulcerans str. Agy99]	81.23	277	290
fig|6666666.28482.peg.2891	R018983	(gi:340625527) proC (MCAN 05011) - putative pyrroline-5-carboxylate reductase PROC [Mycobacterium canettii str. CIPT 140010059]	82.13	290	290
fig|6666666.28482.peg.2891	R018985	(gi:224988889) proC (JTY 0512) - pyrroline-5-carboxylate reductase [Mycobacterium bovis str. BCG str. Tokyo 172]	82.13	290	290
fig|6666666.28482.peg.2891	R005748	(gi:121636417) proC (BCG 0542) - putative pyrroline-5-carboxylate reductase proC [Mycobacterium bovis str. BCG Pasteur 1173P2]	82.13	290	290
fig|6666666.28482.peg.2891	R005747	(gi:31791681) proC (Mb0511) - pyrroline-5-carboxylate reductase [Mycobacterium bovis str. AF2122/97]	82.13	290	290
fig|6666666.28482.peg.2891	R018982	(gi:339630571) proC (MAF 05050) - putative pyrroline-5-carboxylate reductase PROC (P5CR) (P5C reductase) [Mycobacterium africanum str. GM041182]	82.13	290	290
fig|6666666.28482.peg.2891	R018984	(gi:253797430) proC (TBMG 00505) - pyrroline-5-carboxylate reductase [Mycobacterium tuberculosis str. KZN 1435]	81.79	290	290
fig|6666666.28482.peg.2891	R005744	(gi:15607641) proC (Rv0500) - pyrroline-5-carboxylate reductase [Mycobacterium tuberculosis str. H37Rv]	81.79	290	290
fig|6666666.28482.peg.2891	R005745	(gi:148660268) proC (MRA 0507) - pyrroline-5-carboxylate reductase [Mycobacterium tuberculosis str. H37Ra]	81.79	290	290

fig|6666666.28482.peg.2891	R013453	(gi:148821700) proC (TBFG 10509) - pyrroline-5-carboxylate reductase proC [Mycobacterium tuberculosis str. F11]	81.79	290	290
fig|6666666.28482.peg.2891	R005746	(gi:15839892) proC (MT0520) - pyrroline-5-carboxylate reductase [Mycobacterium tuberculosis str. CDC1551]	81.79	290	290
fig|6666666.28482.peg.2891	R005758	(gi:108797648) proC (Mmcs 0668) - pyrroline-5-carboxylate reductase [Mycobacterium sp. str. MCS]	72.76	290	290
fig|6666666.28482.peg.2891	R005757	(gi:119866735) proC (Mkms 0681) - pyrroline-5-carboxylate reductase [Mycobacterium sp. str. KMS]	72.76	290	290
fig|6666666.28482.peg.2891	R005756	(gi:126433272) proC (Mjls 0661) - pyrroline-5-carboxylate reductase [Mycobacterium sp. str. JLS]	72.76	290	290
fig|6666666.28482.peg.2891	R005753	(gi:118472220) proC (MSMEG 0943) - pyrroline-5-carboxylate reductase [Mycobacterium smegmatis str. MC2 155]	73.45	290	290
fig|6666666.28482.peg.2891	R005754	(gi:145220681) proC (Mflv 0075) - pyrroline-5-carboxylate reductase [Mycobacterium gilvum str. PYR-GCK]	72.41	290	290
fig|6666666.28482.peg.2891	R005755	(gi:120401852) proC (Mvan 0837) - transcriptional regulator, Fis family [Mycobacterium vanbaalenii str. PYR-1]	72.41	290	290
fig|6666666.28482.peg.2891	R018981	(gi:169631084) proC (MAB 4005c) - pyrroline-5-carboxylate reductase [Mycobacterium abscessus str. ATCC 19977]	66	290	290
fig|6666666.28482.peg.2891	R018987	(gi:333989135) proC (JDM601 0495) - pyrroline-5-carboxylate reductase ProC [Mycobacterium sp. str. JDM601]	65.87	290	290
fig|6666666.28482.peg.2895	R005158	(gi:15607644) cmaA2 (Rv0503c) - CYCLOPROPANE-FATTY-ACYL-PHOSPHOLIPID SYNTHASE 2 CMAA2 (CYCLOPROPANE FATTY ACID SYNTHASE) (CFA SYNTHASE) (CYCLOPROPANE MYCOLIC ACID SYNTHASE 2) (MYCOLIC ACID TRANS-CYCLOPROPANE SYNTHETASE) [Mycobacterium tuberculosis str. H37Rv]	89.77	303	306
fig|6666666.28482.peg.2895	R005159	(gi:148660272) cmaA2 (MRA 0511) - cyclopropane-fatty-acyl-phospholipid synthase 2 [Mycobacterium tuberculosis str. H37Ra]	89.77	303	306
fig|6666666.28482.peg.2895	R018718	(gi:224988893) cmaA2 (JTY 0516) - cyclopropane-fatty-acyl-phospholipid synthase 2 [Mycobacterium bovis str. BCG str. Tokyo 172]	89.77	303	306
fig|6666666.28482.peg.2895	R005162	(gi:121636421) cmaA2 (BCG 0546c) - Cyclopropane-fatty-acyl-phospholipid synthase 2 cmaA2 [Mycobacterium bovis str. BCG Pasteur 1173P2]	89.77	303	306

fig|6666666.28482.peg.2895	R005161	(gi:31791685) cmaA2 (Mb0515c) - CYCLOPROPANE-FATTY-ACYL-PHOSPHOLIPID SYNTHASE 2 CMAA2 (CYCLOPROPANE FATTY ACID SYNTHASE) (CFA SYNTHASE) (CYCLOPROPANE MYCOLIC ACID SYNTHASE 2) (MYCOLIC ACID TRANS-CYCLOPROPANE SYNTHETASE) [Mycobacterium bovis str. AF2122/97]	89.77	303	306
fig|6666666.28482.peg.2895	R018715	(gi:339630576) cmaA2 (MAF 05100) - cyclopropane-fatty-acyl-phospholipid synthase 2 CMAA2 (cyclopropane fatty acid synthase) [Mycobacterium africanum str. GM041182]	89.77	303	306
fig|6666666.28482.peg.2895	R018717	(gi:253797435) cmaA2 (TBMG 00510) - cyclopropane-fatty-acyl-phospholipid synthase 2 cmaA2 [Mycobacterium tuberculosis str. KZN 1435]	89.77	303	306
fig|6666666.28482.peg.2895	R013398	(gi:148821705) cmaA2 (TBFG 10514) - cyclopropane-fatty-acyl-phospholipid synthase 2 cmaA2 [Mycobacterium tuberculosis str. F11]	89.77	303	306
fig|6666666.28482.peg.2895	R005160	(gi:15839896) cmaA2 (MT0524) - cyclopropane-fatty-acyl-phospholipid synthase 2 [Mycobacterium tuberculosis str. CDC1551]	89.77	303	306
fig|6666666.28482.peg.2895	R018716	(gi:340625532) cmaA2 (MCAN 05061) - cyclopropane-fatty-acyl-phospholipid synthase 2 CMAA2 [Mycobacterium canettii str. CIPT 140010059]	89.44	303	306
fig|6666666.28482.peg.2895	R018714	(gi:183980855) cmaA2 (MMAR 0831) - cyclopropane-fatty-acyl-phospholipid synthase 2 CmaA2 [Mycobacterium marinum str. M]	82.15	297	306
fig|6666666.28482.peg.2895	R005166	(gi:118619667) cmaA2 (MUL 4575) - cyclopropane-fatty-acyl-phospholipid synthase 2 CmaA2 [Mycobacterium ulcerans str. Agy99]	81.82	297	306
fig|6666666.28482.peg.2895	R005164	(gi:118465133) cmaA2 (MAV 4647) - cyclopropane-fatty-acyl-phospholipid synthase 2 [Mycobacterium avium str. 104]	78.93	298	306
fig|6666666.28482.peg.2895	R005163	(gi:41410093) cmaA2 (MAP3995c) - CmaA2 [Mycobacterium avium str. K-10 (subsp. paratuberculosis)]	78.6	298	306
fig|6666666.28482.peg.2895	R005165	(gi:15828307) cmaA2 (ML2426) - cyclopropane mycolic acid synthase [Mycobacterium leprae str. TN]	78.67	297	306
fig|6666666.28482.peg.2895	R018719	(gi:221230784) cmaA2 (MLBr 02426) - cyclopropane mycolic acid synthase [Mycobacterium leprae str. Br4923]	78.67	297	306
fig|6666666.28482.peg.2895	R018720	(gi:333990669) cmaA2 (JDM601 2029) - methoxy mycolic acid synthase [Mycobacterium sp. str. JDM601]	65.1	298	306

fig|6666666.28482.peg.2930	R018221	(gi:121636448) espI (BCG 0573) - hypothetical protein [Mycobacterium bovis str. BCG Pasteur 1173P2]	68.75	423	428
fig|6666666.28482.peg.2946	R005631	(gi:41407632) secA2 (MAP1534) - translocase [Mycobacterium avium str. K-10 (subsp. paratuberculosis)]	93.18	774	774
fig|6666666.28482.peg.2946	R005632	(gi:118463295) secA2 (MAV 2894) - ATPase SecA2 [Mycobacterium avium str. 104]	93.44	774	774
fig|6666666.28482.peg.2946	R019897	(gi:253799135) secA2 (TBMG 02172) - preprotein translocase subunit SecA [Mycobacterium tuberculosis str. KZN 1435]	90.49	774	774
fig|6666666.28482.peg.2946	R005626	(gi:15608958) secA2 (Rv1821) - translocase [Mycobacterium tuberculosis str. H37Rv]	90.49	774	774
fig|6666666.28482.peg.2946	R005627	(gi:148661627) secA2 (MRA 1833) - translocase [Mycobacterium tuberculosis str. H37Ra]	90.49	774	774
fig|6666666.28482.peg.2946	R013441	(gi:148823034) secA2 (TBFG 11851) - preprotein translocase ATPase secA2 subunit [Mycobacterium tuberculosis str. F11]	90.49	774	774
fig|6666666.28482.peg.2946	R005628	(gi:15841291) secA2 (MT1869) - translocase [Mycobacterium tuberculosis str. CDC1551]	90.49	774	774
fig|6666666.28482.peg.2946	R019898	(gi:224990208) secA2 (JTY 1840) - preprotein translocase subunit SecA [Mycobacterium bovis str. BCG str. Tokyo 172]	90.49	774	774
fig|6666666.28482.peg.2946	R005630	(gi:121637724) secA2 (BCG 1856) - putative preprotein translocase secA2 [Mycobacterium bovis str. BCG Pasteur 1173P2]	90.49	774	774
fig|6666666.28482.peg.2946	R005629	(gi:31793011) secA2 (Mb1852) - translocase [Mycobacterium bovis str. AF2122/97]	90.49	774	774
fig|6666666.28482.peg.2946	R019895	(gi:339631874) secA2 (MAF 18430) - putative preprotein translocase ATPase SECA2 [Mycobacterium africanum str. GM041182]	90.49	774	774
fig|6666666.28482.peg.2946	R019896	(gi:340626829) secA2 (MCAN 18361) - putative preprotein translocase ATPase SECA2 [Mycobacterium canettii str. CIPT 140010059]	90.23	774	774
fig|6666666.28482.peg.2946	R019893	(gi:183982705) secA2 (MMAR 2698) - preprotein translocase subunit SecA [Mycobacterium marinum str. M]	90.36	774	774
fig|6666666.28482.peg.2946	R005634	(gi:118618429) secA2 (MUL 3040) - preprotein translocase ATPase SecA2 [Mycobacterium ulcerans str. Agy99]	90.36	774	774
fig|6666666.28482.peg.2946	R005633	(gi:15828125) secA2 (ML2082) - translocase [Mycobacterium leprae str. TN]	88.43	774	774

fig|6666666.28482.peg.2946	R019899	(gi:221230602) secA2 (MLBr 02082) - preprotein translocase subunit SecA [Mycobacterium leprae str. Br4923]	88.43	774	774
fig|6666666.28482.peg.2946	R005640	(gi:108799816) secA2 (Mmcs 2850) - SecA DEAD-like protein [Mycobacterium sp. str. MCS]	85.01	774	774
fig|6666666.28482.peg.2946	R005639	(gi:119868926) secA2 (Mkms 2894) - SecA DEAD domain protein [Mycobacterium sp. str. KMS]	85.01	774	774
fig|6666666.28482.peg.2946	R005638	(gi:126435460) secA2 (Mjls 2881) - SecA DEAD domain protein [Mycobacterium sp. str. JLS]	85.01	774	774
fig|6666666.28482.peg.2946	R005637	(gi:120404141) secA2 (Mvan 3162) - SecA DEAD domain protein [Mycobacterium vanbaalenii str. PYR-1]	83.44	772	774
fig|6666666.28482.peg.2946	R019900	(gi:333990556) secA2 (JDM601 1916) - preprotein translocase ATPase SecA2 [Mycobacterium sp. str. JDM601]	82.95	771	774
fig|6666666.28482.peg.2946	R005635	(gi:118468764) secA2 (MSMEG 3654) - ATPase SecA2 [Mycobacterium smegmatis str. MC2 155]	83.29	774	774
fig|6666666.28482.peg.2946	R005636	(gi:145223994) secA2 (Mflv 3408) - SecA DEAD domain protein [Mycobacterium gilvum str. PYR-GCK]	82.28	772	774
fig|6666666.28482.peg.2946	R019894	(gi:169629483) secA2 (MAB 2397) - preprotein translocase subunit SecA [Mycobacterium abscessus str. ATCC 19977]	79.82	763	774
fig|6666666.28482.peg.3003	R019234	(gi:253800263) sigH (TBMG 03269) - RNA polymerase sigma factor RpoE [Mycobacterium tuberculosis str. KZN 1435]	87.77	229	231
fig|6666666.28482.peg.3003	R013500	(gi:148824423) sigH (TBFG 13250) - alternative RNA polymerase sigma factor sigH (rpoE) [Mycobacterium tuberculosis str. F11]	87.77	229	231
fig|6666666.28482.peg.3003	R006162	(gi:15842811) sigH (MT3320) - RNA polymerase sigma-70 factor [Mycobacterium tuberculosis str. CDC1551]	87.77	229	231
fig|6666666.28482.peg.3003	R006166	(gi:118465818) sigH (MAV 4171) - RNA polymerase sigma-70 factor, family protein [Mycobacterium avium str. 104]	85.48	229	231
fig|6666666.28482.peg.3003	R006165	(gi:41409422) sigH (MAP3324c) - RNA polymerase sigma-70 factor [Mycobacterium avium str. K-10 (subsp. paratuberculosis)]	85.48	229	231
fig|6666666.28482.peg.3003	R019233	(gi:340628202) sigH (MCAN 32411) - alternative RNA polymerase sigma-E factor [Mycobacterium canettii str. CIPT 140010059]	89.81	216	231
fig|6666666.28482.peg.3003	R006160	(gi:15610359) sigH (Rv3223c) - RNA polymerase sigma-70 factor [Mycobacterium tuberculosis str. H37Rv]	89.35	216	231

fig|6666666.28482.peg.3003	R006161	(gi:148663085) sigH (MRA 3263) - RNA polymerase sigma-70 factor [Mycobacterium tuberculosis str. H37Ra]	89.35	216	231
fig|6666666.28482.peg.3003	R019235	(gi:224991603) sigH (JTY 3246) - RNA polymerase sigma factor RpoE [Mycobacterium bovis str. BCG str. Tokyo 172]	89.35	216	231
fig|6666666.28482.peg.3003	R006164	(gi:121639111) sigH (BCG 3251c) - Alternative rna polymerase sigma-E factor (sigma-24) sigH [Mycobacterium bovis str. BCG Pasteur 1173P2]	89.35	216	231
fig|6666666.28482.peg.3003	R006163	(gi:31794402) sigH (Mb3250c) - RNA polymerase sigma-70 factor [Mycobacterium bovis str. AF2122/97]	89.35	216	231
fig|6666666.28482.peg.3003	R019232	(gi:339633232) sigH (MAF 32330) - alternative RNA polymerase sigma-E factor (SIGMA-24) SIGH (RPOE) [Mycobacterium africanum str. GM041182]	88.89	216	231
fig|6666666.28482.peg.3003	R019230	(gi:183981357) sigH (MMAR 1334) - RNA polymerase sigma factor RpoE [Mycobacterium marinum str. M]	87.77	229	231
fig|6666666.28482.peg.3003	R006173	(gi:108798350) sigH (Mmcs 1379) - RNA polymerase, sigma-24 subunit, ECF subfamily [Mycobacterium sp. str. MCS]	84.89	225	231
fig|6666666.28482.peg.3003	R006172	(gi:119867447) sigH (Mkms 1397) - RNA polymerase, sigma-24 subunit, ECF subfamily [Mycobacterium sp. str. KMS]	84.89	225	231
fig|6666666.28482.peg.3003	R006171	(gi:126434015) sigH (Mjls 1413) - RNA polymerase, sigma-24 subunit, ECF subfamily [Mycobacterium sp. str. JLS]	84.89	225	231
fig|6666666.28482.peg.3003	R006167	(gi:118618027) sigH (MUL 2545) - alternative RNA polymerase sigma-E factor (sigma-24) SigH (RpoE) [Mycobacterium ulcerans str. Agy99]	89.81	216	231
fig|6666666.28482.peg.3003	R006170	(gi:120402777) sigH (Mvan 1778) - RNA polymerase, sigma-24 subunit, ECF subfamily [Mycobacterium vanbaalenii str. PYR-1]	82.76	228	231
fig|6666666.28482.peg.3003	R019236	(gi:333991577) sigH (JDM601 2937) - RNA polymerase sigma-E factor (sigma- 24) SigH (RpoE) [Mycobacterium sp. str. JDM601]	80.43	230	231
fig|6666666.28482.peg.3003	R006169	(gi:145225267) sigH (Mflv 4689) - RNA polymerase, sigma-24 subunit, ECF subfamily [Mycobacterium gilvum str. PYR-GCK]	81.78	228	231
fig|6666666.28482.peg.3003	R006168	(gi:118469630) sigH (MSMEG 1914) - RNA polymerase sigma-70 factor, family protein [Mycobacterium smegmatis str. MC2 155]	91.1	191	231
fig|6666666.28482.peg.3003	R019231	(gi:169630624) sigH (MAB 3543c) - RNA polymerase sigma-E factor [Mycobacterium abscessus str. ATCC 19977]	83.94	193	231
fig|6666666.28482.peg.3038	R016423	(gi:218891591) pvdY (PLES 28671) - hypothetical protein [Pseudomonas aeruginosa str. LESB58]	60.43	235	288

fig|6666666.28482.peg.3085	R019266	(gi:183984600) prrA (MMAR 4633) - two-component response transcriptional regulatory protein PrrA [Mycobacterium marinum str. M]	98.73	236	236
fig|6666666.28482.peg.3085	R019270	(gi:253800073) prrA (TBMG 03086) - two component system transcriptional regulator prrA [Mycobacterium tuberculosis str. KZN 1435]	98.31	236	236
fig|6666666.28482.peg.3085	R006231	(gi:15608043) prrA (Rv0903c) - TWO COMPONENT RESPONSE TRANSCRIPTIONAL REGULATORY PROTEIN PRRA [Mycobacterium tuberculosis str. H37Rv]	98.31	236	236
fig|6666666.28482.peg.3085	R006232	(gi:148660681) prrA (MRA 0910) - two component response transcriptional regulatory protein PrrA [Mycobacterium tuberculosis str. H37Ra]	98.31	236	236
fig|6666666.28482.peg.3085	R013505	(gi:148822112) prrA (TBFG 10921) - two component system response transcriptional regulatory protein prrA [Mycobacterium tuberculosis str. F11]	98.31	236	236
fig|6666666.28482.peg.3085	R006233	(gi:15840322) prrA (MT0926) - DNA-binding response regulator [Mycobacterium tuberculosis str. CDC1551]	98.31	236	236
fig|6666666.28482.peg.3085	R019269	(gi:340625914) prrA (MCAN 09031) - two component response transcriptional regulatory protein PRRA [Mycobacterium canettii str. CIPT 140010059]	98.31	236	236
fig|6666666.28482.peg.3085	R019271	(gi:224989297) prrA (JTY 0925) - two component response transcriptional regulatory protein [Mycobacterium bovis str. BCG str. Tokyo 172]	98.31	236	236
fig|6666666.28482.peg.3085	R006235	(gi:121636826) prrA (BCG 0955c) - Two component response transcriptional regulatory protein prrA [Mycobacterium bovis str. BCG Pasteur 1173P2]	98.31	236	236
fig|6666666.28482.peg.3085	R006234	(gi:31792091) prrA (Mb0927c) - TWO COMPONENT RESPONSE TRANSCRIPTIONAL REGULATORY PROTEIN PRRA [Mycobacterium bovis str. AF2122/97]	98.31	236	236
fig|6666666.28482.peg.3085	R019268	(gi:339630968) prrA (MAF 09120) - two component response transcriptional regulatory protein PRRA [Mycobacterium africanum str. GM041182]	98.31	236	236
fig|6666666.28482.peg.3085	R006239	(gi:118616126) prrA (MUL 0248) - two component response transcriptional regulatory protein PrrA [Mycobacterium ulcerans str. Agy99]	97.88	236	236
fig|6666666.28482.peg.3085	R006236	(gi:41406932) prrA (MAP0834c) - hypothetical protein [Mycobacterium avium str. K-10 (subsp. paratuberculosis)]	98.28	233	236

fig|6666666.28482.peg.3085	R006237	(gi:118465275) prrA (MAV 1022) - two component response transcriptional regulatory protein prra [Mycobacterium avium str. 104]	98.28	233	236
fig|6666666.28482.peg.3085	R006238	(gi:15828139) prrA (ML2123) - two-component response regulator [Mycobacterium leprae str. TN]	96.57	233	236
fig|6666666.28482.peg.3085	R019272	(gi:221230616) prrA (MLBr 02123) - two-component response regulator [Mycobacterium leprae str. Br4923]	96.57	233	236
fig|6666666.28482.peg.3085	R006242	(gi:120405958) prrA (Mvan 5009) - two component transcriptional regulator, winged helix family [Mycobacterium vanbaalenii str. PYR-1]	95.28	233	236
fig|6666666.28482.peg.3085	R006245	(gi:108801411) prrA (Mmcs 4448) - two component transcriptional regulator, winged helix family [Mycobacterium sp. str. MCS]	95.28	233	236
fig|6666666.28482.peg.3085	R006244	(gi:119870564) prrA (Mkms 4535) - two component transcriptional regulator, winged helix family [Mycobacterium sp. str. KMS]	95.28	233	236
fig|6666666.28482.peg.3085	R006241	(gi:145222334) prrA (Mflv 1744) - two component transcriptional regulator, winged helix family [Mycobacterium gilvum str. PYR-GCK]	95.71	233	236
fig|6666666.28482.peg.3085	R006243	(gi:126437395) prrA (Mjls 4830) - two component transcriptional regulator, winged helix family [Mycobacterium sp. str. JLS]	94.85	233	236
fig|6666666.28482.peg.3085	R006240	(gi:118468513) prrA (MSMEG 5662) - DNA-binding response regulator PrrA [Mycobacterium smegmatis str. MC2 155]	93.64	236	236
fig|6666666.28482.peg.3085	R019267	(gi:169628053) prrA (MAB 0956c) - transcriptional regulatory protein PrrA [Mycobacterium abscessus str. ATCC 19977]	91.42	233	236
fig|6666666.28482.peg.3085	R019273	(gi:333989463) prrA (JDM601 0823) - two-component response transcriptional regulatory protein PrrA [Mycobacterium sp. str. JDM601]	91.1	236	236
fig|6666666.28482.peg.3086	R019278	(gi:253800074) prrB (TBMG 03087) - two component system sensor histidine kinase prrB [Mycobacterium tuberculosis str. KZN 1435]	89.51	429	429
fig|6666666.28482.peg.3086	R006246	(gi:15608042) prrB (Rv0902c) - TWO COMPONENT SENSOR HISTIDINE KINASE PRRB [Mycobacterium tuberculosis str. H37Rv]	89.51	429	429
fig|6666666.28482.peg.3086	R006247	(gi:148660680) prrB (MRA 0909) - two component sensor histidine kinase PrrB [Mycobacterium tuberculosis str. H37Ra]	89.51	429	429
fig|6666666.28482.peg.3086	R013506	(gi:148822111) prrB (TBFG 10920) - two component system sensor histidine kinase prrB [Mycobacterium tuberculosis str. F11]	89.51	429	429
fig|6666666.28482.peg.3086	R019277	(gi:340625913) prrB (MCAN 09021) - two component sensor histidine kinase PRRB [Mycobacterium canettii str. CIPT 140010059]	89.51	429	429

fig|6666666.28482.peg.3086	R019279	(gi:224989296) prrB (JTY 0924) - two component sensor histidine kinase [Mycobacterium bovis str. BCG str. Tokyo 172]	89.51	429	429
fig|6666666.28482.peg.3086	R006250	(gi:121636825) prrB (BCG 0954c) - Two component sensor histidine kinase prrB [Mycobacterium bovis str. BCG Pasteur 1173P2]	89.51	429	429
fig|6666666.28482.peg.3086	R006249	(gi:31792090) prrB (Mb0926c) - TWO COMPONENT SENSOR HISTIDINE KINASE PRRB [Mycobacterium bovis str. AF2122/97]	89.51	429	429
fig|6666666.28482.peg.3086	R019276	(gi:339630967) prrB (MAF 09110) - two component sensor histidine kinase PRRB [Mycobacterium africanum str. GM041182]	89.51	429	429
fig|6666666.28482.peg.3086	R006248	(gi:15840321) prrB (MT0925) - sensor histidine kinase [Mycobacterium tuberculosis str. CDC1551]	89.51	429	429
fig|6666666.28482.peg.3086	R019274	(gi:183984601) prrB (MMAR 4634) - two-component sensor histidine kinase PrrB [Mycobacterium marinum str. M]	88.58	429	429
fig|6666666.28482.peg.3086	R006254	(gi:118616127) prrB (MUL 0249) - two component sensor histidine kinase PrrB [Mycobacterium ulcerans str. Agy99]	88.34	429	429
fig|6666666.28482.peg.3086	R006253	(gi:15828140) prrB (ML2124) - sensor histidine kinase [Mycobacterium leprae str. TN]	89.04	429	429
fig|6666666.28482.peg.3086	R019280	(gi:221230617) prrB (MLBr 02124) - sensor histidine kinase [Mycobacterium leprae str. Br4923]	89.04	429	429
fig|6666666.28482.peg.3086	R019281	(gi:333989462) prrB (JDM601 0822) - two-component sensor histidine kinase [Mycobacterium sp. str. JDM601]	80.37	428	429
fig|6666666.28482.peg.3086	R006252	(gi:118464817) prrB (MAV 1021) - sensor-type histidine kinase PrrB [Mycobacterium avium str. 104]	80.65	429	429
fig|6666666.28482.peg.3086	R006255	(gi:118472850) prrB (MSMEG 5663) - sensor-type histidine kinase PrrB [Mycobacterium smegmatis str. MC2 155]	78.74	428	429
fig|6666666.28482.peg.3086	R006260	(gi:108801412) prrB (Mmcs 4449) - periplasmic sensor signal transduction histidine kinase [Mycobacterium sp. str. MCS]	79.72	428	429
fig|6666666.28482.peg.3086	R006259	(gi:119870565) prrB (Mkms 4536) - integral membrane sensor signal transduction histidine kinase [Mycobacterium sp. str. KMS]	79.72	428	429
fig|6666666.28482.peg.3086	R006258	(gi:126437396) prrB (Mjls 4831) - integral membrane sensor signal transduction histidine kinase [Mycobacterium sp. str. JLS]	79.72	428	429
fig|6666666.28482.peg.3086	R006257	(gi:120405959) prrB (Mvan 5010) - integral membrane sensor signal transduction histidine kinase [Mycobacterium vanbaalenii str. PYR-1]	78.52	405	429

fig|6666666.28482.peg.3086	R006256	(gi:145222333) prrB (Mflv 1743) - integral membrane sensor signal transduction histidine kinase [Mycobacterium gilvum str. PYR-GCK]	75.29	428	429
fig|6666666.28482.peg.3086	R006251	(gi:41406931) prrB (MAP0833c) - hypothetical protein [Mycobacterium avium str. K-10 (subsp. paratuberculosis)]	80.15	388	429
fig|6666666.28482.peg.3142	R006069	(gi:118465627) katG (MAV 2753) - catalase/peroxidase HPI [Mycobacterium avium str. 104]	90.59	742	744
fig|6666666.28482.peg.3142	R006068	(gi:41407766) katG (MAP1668c) - KatG [Mycobacterium avium str. K-10 (subsp. paratuberculosis)]	90.32	742	744
fig|6666666.28482.peg.3142	R019170	(gi:183982917) katG (MMAR 2914) - catalase-peroxidase-peroxynitritase T KatG [Mycobacterium marinum str. M]	84.64	742	744
fig|6666666.28482.peg.3142	R006074	(gi:120404186) katG (Mvan 3208) - catalase/peroxidase HPI [Mycobacterium vanbaalenii str. PYR-1]	79.36	741	744
fig|6666666.28482.peg.3142	R006072	(gi:145224036) katG (Mflv 3451) - catalase/peroxidase HPI [Mycobacterium gilvum str. PYR-GCK]	77.78	742	744
fig|6666666.28482.peg.3142	R006077	(gi:108799649) katG (Mmcs 2682) - catalase/peroxidase HPI [Mycobacterium sp. str. MCS]	77.48	742	744
fig|6666666.28482.peg.3142	R006076	(gi:119868760) katG (Mkms 2727) - catalase/peroxidase HPI [Mycobacterium sp. str. KMS]	77.48	742	744
fig|6666666.28482.peg.3142	R006075	(gi:126435293) katG (Mjls 2713) - catalase/peroxidase HPI [Mycobacterium sp. str. JLS]	77.48	742	744
fig|6666666.28482.peg.3142	R006070	(gi:118617734) katG (MUL 2190) - catalase-peroxidase-peroxynitritase T KatG [Mycobacterium ulcerans str. Agy99]	84.89	708	744
fig|6666666.28482.peg.3142	R019173	(gi:340626917) katG (MCAN 19241) - catalase-peroxidase-peroxynitritase T KATG [Mycobacterium canettii str. CIPT 140010059]	70.76	706	744
fig|6666666.28482.peg.3142	R019175	(gi:224990298) katG (JTY 1931) - catalase-peroxidase-peroxynitritase T [Mycobacterium bovis str. BCG str. Tokyo 172]	70.76	706	744
fig|6666666.28482.peg.3142	R006067	(gi:121637814) katG (BCG 1947c) - Catalase-peroxidase-peroxynitritase T katG [Mycobacterium bovis str. BCG Pasteur 1173P2]	70.76	706	744
fig|6666666.28482.peg.3142	R006066	(gi:31793101) katG (Mb1943c) - CATALASE-PEROXIDASE-PEROXYNITRITASE T KATG [Mycobacterium bovis str. AF2122/97]	70.76	706	744
fig|6666666.28482.peg.3142	R019172	(gi:339631961) katG (MAF 19310) - catalase-peroxidase-peroxynitritase T KATG [Mycobacterium africanum str. GM041182]	70.76	706	744

fig|6666666.28482.peg.3142	R006063	(gi:15609045) katG (Rv1908c) - CATALASE-PEROXIDASE-PEROXYNITRITASE T KATG [Mycobacterium tuberculosis str. H37Rv]	70.62	706	744
fig|6666666.28482.peg.3142	R006064	(gi:148661716) katG (MRA 1919) - catalase-peroxidase [Mycobacterium tuberculosis str. H37Ra]	70.62	706	744
fig|6666666.28482.peg.3142	R006065	(gi:15841380) katG (MT1959) - catalase-peroxidase [Mycobacterium tuberculosis str. CDC1551]	70.62	706	744
fig|6666666.28482.peg.3142	R019174	(gi:253799045) katG (TBMG 02084) - catalase-peroxidase-peroxynitritase T katG [Mycobacterium tuberculosis str. KZN 1435]	70.48	706	744
fig|6666666.28482.peg.3142	R013478	(gi:148823120) katG (TBFG 11937) - catalase-peroxidase katG [Mycobacterium tuberculosis str. F11]	70.48	706	744
fig|6666666.28482.peg.3142	R019171	(gi:169629556) katG (MAB 2470c) - catalase/peroxidase (KatG) [Mycobacterium abscessus str. ATCC 19977]	67.23	706	744
fig|6666666.28482.peg.3142	R006071	(gi:118471761) katG (MSMEG 6384) - catalase/peroxidase HPI [Mycobacterium smegmatis str. MC2 155]	62.4	735	744
fig|6666666.28482.peg.3245	R005417	(gi:118467129) fbpA (MAV 0214) - antigen 85-A [Mycobacterium avium str. 104]	92.78	291	316
fig|6666666.28482.peg.3245	R005416	(gi:41406314) fbpA (MAP0216) - FbpA [Mycobacterium avium str. K-10 (subsp. paratuberculosis)]	92.44	291	316
fig|6666666.28482.peg.3245	R018864	(gi:183985336) fbpA (MMAR 5368) - secreted antigen 85-A FbpA [Mycobacterium marinum str. M]	87.92	298	316
fig|6666666.28482.peg.3245	R005419	(gi:118620004) fbpA (MUL 4987) - secreted antigen 85-A FbpA [Mycobacterium ulcerans str. Agy99]	86.91	298	316
fig|6666666.28482.peg.3245	R018868	(gi:253800852) fbpA (TBMG 03851) - secreted fibronectin-binding protein antigen fbpA [Mycobacterium tuberculosis str. KZN 1435]	80.59	304	316
fig|6666666.28482.peg.3245	R005411	(gi:15610940) fbpA (Rv3804c) - SECRETED ANTIGEN 85-A FBPA (MYCOLYL TRANSFERASE 85A) (FIBRONECTIN-BINDING PROTEIN A) (ANTIGEN 85 COMPLEX A) [Mycobacterium tuberculosis str. H37Rv]	80.59	304	316
fig|6666666.28482.peg.3245	R005412	(gi:148663672) fbpA (MRA 3844) - secreted antigen 85-A FbpA [Mycobacterium tuberculosis str. H37Ra]	80.59	304	316
fig|6666666.28482.peg.3245	R013424	(gi:148825011) fbpA (TBFG 13838) - secreted antigen 85-A fbpA (fibronectin-binding protein A) [Mycobacterium tuberculosis str. F11]	80.59	304	316
fig|6666666.28482.peg.3245	R018867	(gi:340628773) fbpA (MCAN 38231) - secreted antigen 85-A FBPA [Mycobacterium canettii str. CIPT 140010059]	80.59	304	316

fig|6666666.28482.peg.3245	R018869	(gi:224992216) fbpA (JTY 3868) - secreted antigen 85-A [Mycobacterium bovis str. BCG str. Tokyo 172]	80.59	304	316
fig|6666666.28482.peg.3245	R005415	(gi:121639721) fbpA (BCG 3866c) - Secreted antigen 85-A fbpA [Mycobacterium bovis str. BCG Pasteur 1173P2]	80.59	304	316
fig|6666666.28482.peg.3245	R005414	(gi:31794978) fbpA (Mb3834c) - SECRETED ANTIGEN 85-A FBPA (MYCOLYL TRANSFERASE 85A) (FIBRONECTIN-BINDING PROTEIN A) (ANTIGEN 85 COMPLEX A) [Mycobacterium bovis str. AF2122/97]	80.59	304	316
fig|6666666.28482.peg.3245	R005413	(gi:15843426) fbpA (MT3911) - esterase, putative, antigen 85-A [Mycobacterium tuberculosis str. CDC1551]	80.59	304	316
fig|6666666.28482.peg.3245	R018866	(gi:339633799) fbpA (MAF 38190) - secreted antigen 85-A FBPA (mycolyl transferase 85A) [Mycobacterium africanum str. GM041182]	80.26	304	316
fig|6666666.28482.peg.3245	R005426	(gi:41407707) fbpB (MAP1609c) - FbpB [Mycobacterium avium str. K-10 (subsp. paratuberculosis)]	80.13	297	316
fig|6666666.28482.peg.3245	R005427	(gi:118465697) fbpB (MAV 2816) - antigen 85-B [Mycobacterium avium str. 104]	80.13	297	316
fig|6666666.28482.peg.3245	R018872	(gi:183982783) fbpB (MMAR 2777) - secreted antigen 85-B FbpB [Mycobacterium marinum str. M]	80.82	292	316
fig|6666666.28482.peg.3245	R018875	(gi:253799070) fbpB (TBMG 02108) - secreted fibronectin-binding protein antigen 85-B fbpB [Mycobacterium tuberculosis str. KZN 1435]	82.39	284	316
fig|6666666.28482.peg.3245	R005421	(gi:15609023) fbpB (Rv1886c) - SECRETED ANTIGEN 85-B FBPB (85B) (ANTIGEN 85 COMPLEX B) (MYCOLYL TRANSFERASE 85B) (FIBRONECTIN-BINDING PROTEIN B) (EXTRACELLULAR ALPHA-ANTIGEN) [Mycobacterium tuberculosis str. H37Rv]	82.39	284	316
fig|6666666.28482.peg.3245	R005422	(gi:148661692) fbpB (MRA 1897) - secreted antigen 85-B FbpB [Mycobacterium tuberculosis str. H37Ra]	82.39	284	316
fig|6666666.28482.peg.3245	R013425	(gi:148823097) fbpB (TBFG 11914) - secreted antigen 85-B fbpB (fibronectin-binding protein B) [Mycobacterium tuberculosis str. F11]	82.39	284	316
fig|6666666.28482.peg.3245	R018874	(gi:340626894) fbpB (MCAN 19011) - secreted antigen 85-B FBPB [Mycobacterium canettii str. CIPT 140010059]	82.39	284	316
fig|6666666.28482.peg.3245	R005424	(gi:31793077) fbpB (Mb1918c) - secreted antigen 85-B fbpB (85B) (antigen 85 complex B) (Mycolyl transferase 85B) (Fibronectin-binding protein B) (Extracellular alpha-antigen) [Mycobacterium bovis str. AF2122/97]	82.39	284	316

fig|6666666.28482.peg.3245	R018873	(gi:339631939) fbpB (MAF 19080) - secreted antigen 85-B FBPB (85B) (antigen 85 complex B) [Mycobacterium africanum str. GM041182]	82.39	284	316
fig|6666666.28482.peg.3245	R005423	(gi:15841356) fbpB (MT1934) - esterase, putative, antigen 85-B [Mycobacterium tuberculosis str. CDC1551]	82.39	284	316
fig|6666666.28482.peg.3245	R018876	(gi:224990274) fbpB (JTY 1907) - secreted antigen 85-B [Mycobacterium bovis str. BCG str. Tokyo 172]	82.04	284	316
fig|6666666.28482.peg.3245	R005425	(gi:121637790) fbpB (BCG 1923c) - secreted antigen 85-B fbpB [Mycobacterium bovis str. BCG Pasteur 1173P2]	82.04	284	316
fig|6666666.28482.peg.3245	R005429	(gi:118618369) fbpB (MUL 2970) - secreted antigen 85-B FbpB [Mycobacterium ulcerans str. Agy99]	79.45	292	316
fig|6666666.28482.peg.3245	R005428	(gi:15828096) fbpB (ML2028) - antigen 85A, mycolyltransferase [Mycobacterium leprae str. TN]	79.8	297	316
fig|6666666.28482.peg.3245	R018877	(gi:221230573) fbpB (MLBr 02028) - antigen 85A, mycolyltransferase [Mycobacterium leprae str. Br4923]	79.8	297	316
fig|6666666.28482.peg.3245	R005418	(gi:15826932) fbpA (ML0097) - antigen 85A, mycolytransferase [Mycobacterium leprae str. TN]	78.31	295	316
fig|6666666.28482.peg.3245	R018870	(gi:221229410) fbpA (MLBr 00097) - antigen 85A, mycolytransferase [Mycobacterium leprae str. Br4923]	78.31	295	316
fig|6666666.28482.peg.3245	R018878	(gi:333990613) fbpB (JDM601 1973) - hypothetical protein [Mycobacterium sp. str. JDM601]	74.5	297	316
fig|6666666.28482.peg.3245	R018871	(gi:333992762) fbpA (JDM601 4122) - hypothetical protein [Mycobacterium sp. str. JDM601]	72.28	302	316
fig|6666666.28482.peg.3245	R005441	(gi:145221756) fbpC (Mflv 1164) - putative esterase [Mycobacterium gilvum str. PYR-GCK]	72.15	292	316
fig|6666666.28482.peg.3245	R005444	(gi:145225038) fbpC (Mflv 4459) - putative esterase [Mycobacterium gilvum str. PYR-GCK]	72.85	290	316
fig|6666666.28482.peg.3245	R005446	(gi:120402899) fbpC (Mvan 1902) - putative esterase [Mycobacterium vanbaalenii str. PYR-1]	71.13	290	316
fig|6666666.28482.peg.3245	R005430	(gi:118473709) fbpB (MSMEG 2078) - antigen 85-C [Mycobacterium smegmatis str. MC2 155]	70.41	292	316
fig|6666666.28482.peg.3245	R005466	(gi:108801978) fbpC (Mmcs 5015) - putative esterase [Mycobacterium sp. str. MCS]	69.02	291	316

fig|6666666.28482.peg.3245	R005461	(gi:119871130) fbpC (Mkms 5103) - putative esterase [Mycobacterium sp. str. KMS]	69.02	291	316
fig|6666666.28482.peg.3245	R005456	(gi:126437959) fbpC (Mjls 5396) - putative esterase [Mycobacterium sp. str. JLS]	69.02	291	316
fig|6666666.28482.peg.3245	R005420	(gi:118468744) fbpA (MSMEG 6398) - antigen 85-A [Mycobacterium smegmatis str. MC2 155]	69.77	297	316
fig|6666666.28482.peg.3245	R005455	(gi:126437080) fbpC (Mjls 4511) - putative esterase [Mycobacterium sp. str. JLS]	67.22	293	316
fig|6666666.28482.peg.3245	R018882	(gi:253797048) fbpC (TBMG 00130) - secreted fibronectin-binding protein C antigen 85-C fbpC [Mycobacterium tuberculosis str. KZN 1435]	70.1	291	316
fig|6666666.28482.peg.3245	R005431	(gi:57116693) fbpC (Rv0129c) - SECRETED ANTIGEN 85-C FBPC (85C) (ANTIGEN 85 COMPLEX C) (AG58C) (MYCOLYL TRANSFERASE 85C) (FIBRONECTIN-BINDING PROTEIN C) [Mycobacterium tuberculosis str. H37Rv]	70.1	291	316
fig|6666666.28482.peg.3245	R005432	(gi:148659893) fbpC (MRA 0136) - secreted antigen 86-C FbpC [Mycobacterium tuberculosis str. H37Ra]	70.1	291	316
fig|6666666.28482.peg.3245	R013426	(gi:148821321) fbpC (TBFG 10130) - secreted antigen 85-C fbpC (fibronectin-binding protein C) [Mycobacterium tuberculosis str. F11]	70.1	291	316
fig|6666666.28482.peg.3245	R018881	(gi:340625164) fbpC (MCAN 01321) - secreted antigen 85-C FBPC [Mycobacterium canettii str. CIPT 140010059]	70.1	291	316
fig|6666666.28482.peg.3245	R018883	(gi:224988515) fbpC (JTY 0133) - secreted antigen 85-C [Mycobacterium bovis str. BCG str. Tokyo 172]	70.1	291	316
fig|6666666.28482.peg.3245	R005435	(gi:121636042) fbpC (BCG 0163c) - Secreted antigen 85-c fbpC (85C) [Mycobacterium bovis str. BCG Pasteur 1173P2]	70.1	291	316
fig|6666666.28482.peg.3245	R005434	(gi:31791308) fbpC (Mb0134c) - SECRETED ANTIGEN 85-C FBPC (85C) (ANTIGEN 85 COMPLEX C) (AG58C) (MYCOLYL TRANSFERASE 85C) (FIBRONECTIN-BINDING PROTEIN C) [Mycobacterium bovis str. AF2122/97]	70.1	291	316
fig|6666666.28482.peg.3245	R018880	(gi:339630211) fbpC (MAF 01290) - secreted antigen 85-C FbpC [Mycobacterium africanum str. GM041182]	70.1	291	316
fig|6666666.28482.peg.3245	R005433	(gi:15839510) fbpC (MT0137) - esterase, putative, antigen 85-C [Mycobacterium tuberculosis str. CDC1551]	70.1	291	316

fig|6666666.28482.peg.3245	R005436	(gi:41409629) fbpC (MAP3531c) - FbpC2 [Mycobacterium avium str. K-10 (subsp. paratuberculosis)]	68.28	290	316
fig|6666666.28482.peg.3245	R005437	(gi:118465788) fbpC (MAV 5183) - antigen 85-C [Mycobacterium avium str. 104]	68.28	290	316
fig|6666666.28482.peg.3245	R005439	(gi:118619846) fbpC (MUL 4793) - secreted antigen 85-C FbpC [Mycobacterium ulcerans str. Agy99]	70.21	282	316
fig|6666666.28482.peg.3245	R018879	(gi:183980359) fbpC (MMAR 0328) - secreted antigen 85-C FbpC [Mycobacterium marinum str. M]	70.21	282	316
fig|6666666.28482.peg.3245	R018885	(gi:333992822) fbpC (JDM601 4182) - hypothetical protein [Mycobacterium sp. str. JDM601]	69.31	290	316
fig|6666666.28482.peg.3245	R005452	(gi:126434164) fbpC (Mjls 1566) - putative esterase [Mycobacterium sp. str. JLS]	69.31	290	316
fig|6666666.28482.peg.3245	R005438	(gi:15828432) fbpC (ML2655) - secreted antigen 85A, mycolyltransferase [Mycobacterium leprae str. TN]	65.64	291	316
fig|6666666.28482.peg.3245	R018884	(gi:221230909) fbpC (MLBr 02655) - secreted antigen 85A, mycolyltransferase [Mycobacterium leprae str. Br4923]	65.64	291	316
fig|6666666.28482.peg.3245	R005462	(gi:108798566) fbpC (Mmcs 1596) - putative esterase [Mycobacterium sp. str. MCS]	68.97	290	316
fig|6666666.28482.peg.3245	R005457	(gi:119867665) fbpC (Mkms 1620) - putative esterase [Mycobacterium sp. str. KMS]	68.97	290	316
fig|6666666.28482.peg.3245	R005440	(gi:118467737) fbpC (MSMEG 3580) - antigen 85-C [Mycobacterium smegmatis str. MC2 155]	65.32	292	316
fig|6666666.28482.peg.3245	R005448	(gi:120404040) fbpC (Mvan 3061) - putative esterase [Mycobacterium vanbaalenii str. PYR-1]	64.65	292	316
fig|6666666.28482.peg.3245	R005443	(gi:145223921) fbpC (Mflv 3335) - putative esterase [Mycobacterium gilvum str. PYR-GCK]	63.97	292	316
fig|6666666.28482.peg.3245	R005464	(gi:108799739) fbpC (Mmcs 2773) - putative esterase [Mycobacterium sp. str. MCS]	63.64	292	316
fig|6666666.28482.peg.3245	R005459	(gi:119868849) fbpC (Mkms 2817) - putative esterase [Mycobacterium sp. str. KMS]	63.64	292	316
fig|6666666.28482.peg.3245	R005454	(gi:126435380) fbpC (Mjls 2800) - putative esterase [Mycobacterium sp. str. JLS]	63.64	292	316

fig|6666666.28482.peg.3245	R005458	(gi:119867920) fbpC (Mkms 1882) - putative esterase [Mycobacterium sp. str. KMS]	63.48	292	316
fig|6666666.28482.peg.3245	R018865	(gi:169627281) fbpA (MAB 0176) - antigen 85-A precursor [Mycobacterium abscessus str. ATCC 19977]	61.87	291	316
fig|6666666.28482.peg.3245	R005453	(gi:126434404) fbpC (Mjls 1816) - putative esterase [Mycobacterium sp. str. JLS]	63.14	292	316
fig|6666666.28482.peg.3245	R005442	(gi:145223828) fbpC (Mflv 3241) - putative esterase [Mycobacterium gilvum str. PYR-GCK]	61.3	291	316
fig|6666666.28482.peg.3245	R005447	(gi:120403937) fbpC (Mvan 2954) - putative esterase [Mycobacterium vanbaalenii str. PYR-1]	61.43	292	316
fig|6666666.28482.peg.3275	R005651	(gi:145223363) kasB (Mflv 2776) - beta-ketoacyl synthase [Mycobacterium gilvum str. PYR-GCK]	65.55	416	416
fig|6666666.28482.peg.3275	R005652	(gi:120404716) kasB (Mvan 3757) - beta-ketoacyl synthase [Mycobacterium vanbaalenii str. PYR-1]	65.07	416	416
fig|6666666.28482.peg.3275	R005648	(gi:15827874) kasB (ML1656) - 3-oxoacyl-(acyl carrier protein) synthase [Mycobacterium leprae str. TN]	64.83	415	416
fig|6666666.28482.peg.3275	R018920	(gi:221230351) kasB (MLBr 01656) - 3-oxoacyl-(acyl carrier protein) synthase II [Mycobacterium leprae str. Br4923]	64.83	415	416
fig|6666666.28482.peg.3275	R005643	(gi:15841739) kasB (MT2306) - 3-oxoacyl-(acyl carrier protein) synthase [Mycobacterium tuberculosis str. CDC1551]	66.18	406	416
fig|6666666.28482.peg.3275	R018918	(gi:253798686) kasB (TBMG 01734) - 3-oxoacyl-(acyl carrier protein) synthase II [Mycobacterium tuberculosis str. KZN 1435]	66.18	406	416
fig|6666666.28482.peg.3275	R005641	(gi:15609383) kasB (Rv2246) - 3-oxoacyl-(acyl carrier protein) synthase [Mycobacterium tuberculosis str. H37Rv]	66.18	406	416
fig|6666666.28482.peg.3275	R005642	(gi:148662067) kasB (MRA 2266) - 3-oxoacyl-(acyl-carrier-protein) synthase 2 KasB [Mycobacterium tuberculosis str. H37Ra]	66.18	406	416
fig|6666666.28482.peg.3275	R013442	(gi:148823454) kasB (TBFG 12275) - 3-oxoacyl-[acyl-carrier protein] synthase 2 kasB [Mycobacterium tuberculosis str. F11]	66.18	406	416
fig|6666666.28482.peg.3275	R018917	(gi:340627250) kasB (MCAN 22681) - 3-oxoacyl-[acyl-carrier protein] synthase 2 KASB [Mycobacterium canettii str. CIPT 140010059]	66.18	406	416
fig|6666666.28482.peg.3275	R018919	(gi:224990622) kasB (JTY 2257) - 3-oxoacyl-(acyl carrier protein) synthase II [Mycobacterium bovis str. BCG str. Tokyo 172]	66.18	406	416

fig|6666666.28482.peg.3275	R005645	(gi:121638128) kasB (BCG 2263) - 3-oxoacyl-[acyl-carrier protein] synthase 2 kasB [Mycobacterium bovis str. BCG Pasteur 1173P2]	66.18	406	416
fig|6666666.28482.peg.3275	R005644	(gi:31793426) kasB (Mb2270) - 3-oxoacyl-(acyl carrier protein) synthase [Mycobacterium bovis str. AF2122/97]	66.18	406	416
fig|6666666.28482.peg.3275	R018916	(gi:339632272) kasB (MAF 22570) - 3-oxoacyl-[acyl-carrier protein] synthase 2 KASB (beta-ketoacyl-ACP synthase) [Mycobacterium africanum str. GM041182]	66.18	406	416
fig|6666666.28482.peg.3275	R005655	(gi:108800333) kasB (Mmcs 3367) - beta-ketoacyl synthase [Mycobacterium sp. str. MCS]	63.16	416	416
fig|6666666.28482.peg.3275	R005654	(gi:119869461) kasB (Mkms 3429) - beta-ketoacyl synthase [Mycobacterium sp. str. KMS]	63.16	416	416
fig|6666666.28482.peg.3275	R005653	(gi:126435956) kasB (Mjls 3378) - beta-ketoacyl synthase [Mycobacterium sp. str. JLS]	63.16	416	416
fig|6666666.28482.peg.3275	R005649	(gi:118617002) kasB (MUL 1303) - 3-oxoacyl-[acyl-carrier protein] synthase 2 KasB [Mycobacterium ulcerans str. Agy99]	64.22	406	416
fig|6666666.28482.peg.3275	R018915	(gi:183983329) kasB (MMAR 3339) - 3-oxoacyl-(acyl carrier protein) synthase II [Mycobacterium marinum str. M]	64.22	406	416
fig|6666666.28482.peg.3275	R005650	(gi:118467655) kasB (MSMEG 4328) - 3-oxoacyl-[acyl-carrier-protein] synthase 2 [Mycobacterium smegmatis str. MC2 155]	63.16	416	416
fig|6666666.28482.peg.3275	R005646	(gi:41408097) kasB (MAP1999) - 3-oxoacyl-(acyl carrier protein) synthase [Mycobacterium avium str. K-10 (subsp. paratuberculosis)]	64.3	405	416
fig|6666666.28482.peg.3275	R005647	(gi:118465046) kasB (MAV 2191) - 3-oxoacyl-[acyl-carrier-protein] synthase 2 [Mycobacterium avium str. 104]	64.13	403	416
fig|6666666.28482.peg.3275	R018921	(gi:333990667) kasB (JDM601 2027) - 3-oxoacyl-(acyl carrier protein) synthase II [Mycobacterium sp. str. JDM601]	61.72	416	416
fig|6666666.28482.peg.3275	R018922	(gi:333990289) kasB (JDM601 1649) - 3-oxoacyl-(acyl carrier protein) synthase II [Mycobacterium sp. str. JDM601]	62.69	395	416
fig|6666666.28482.peg.3276	R005643	(gi:15841739) kasB (MT2306) - 3-oxoacyl-(acyl carrier protein) synthase [Mycobacterium tuberculosis str. CDC1551]	92.57	404	404
fig|6666666.28482.peg.3276	R018918	(gi:253798686) kasB (TBMG 01734) - 3-oxoacyl-(acyl carrier protein) synthase II [Mycobacterium tuberculosis str. KZN 1435]	92.57	404	404
fig|6666666.28482.peg.3276	R005641	(gi:15609383) kasB (Rv2246) - 3-oxoacyl-(acyl carrier protein) synthase [Mycobacterium tuberculosis str. H37Rv]	92.57	404	404

fig|6666666.28482.peg.3276	R005642	(gi:148662067) kasB (MRA 2266) - 3-oxoacyl-(acyl-carrier-protein) synthase 2 KasB [Mycobacterium tuberculosis str. H37Ra]	92.57	404	404
fig|6666666.28482.peg.3276	R013442	(gi:148823454) kasB (TBFG 12275) - 3-oxoacyl-[acyl-carrier protein] synthase 2 kasB [Mycobacterium tuberculosis str. F11]	92.57	404	404
fig|6666666.28482.peg.3276	R018917	(gi:340627250) kasB (MCAN 22681) - 3-oxoacyl-[acyl-carrier protein] synthase 2 KASB [Mycobacterium canettii str. CIPT 140010059]	92.57	404	404
fig|6666666.28482.peg.3276	R018919	(gi:224990622) kasB (JTY 2257) - 3-oxoacyl-(acyl carrier protein) synthase II [Mycobacterium bovis str. BCG str. Tokyo 172]	92.57	404	404
fig|6666666.28482.peg.3276	R005645	(gi:121638128) kasB (BCG 2263) - 3-oxoacyl-[acyl-carrier protein] synthase 2 kasB [Mycobacterium bovis str. BCG Pasteur 1173P2]	92.57	404	404
fig|6666666.28482.peg.3276	R005644	(gi:31793426) kasB (Mb2270) - 3-oxoacyl-(acyl carrier protein) synthase [Mycobacterium bovis str. AF2122/97]	92.57	404	404
fig|6666666.28482.peg.3276	R018916	(gi:339632272) kasB (MAF 22570) - 3-oxoacyl-[acyl-carrier protein] synthase 2 KASB (beta-ketoacyl-ACP synthase) [Mycobacterium africanum str. GM041182]	92.57	404	404
fig|6666666.28482.peg.3276	R005649	(gi:118617002) kasB (MUL 1303) - 3-oxoacyl-[acyl-carrier protein] synthase 2 KasB [Mycobacterium ulcerans str. Agy99]	89.6	404	404
fig|6666666.28482.peg.3276	R018915	(gi:183983329) kasB (MMAR 3339) - 3-oxoacyl-(acyl carrier protein) synthase II [Mycobacterium marinum str. M]	89.6	404	404
fig|6666666.28482.peg.3276	R005648	(gi:15827874) kasB (ML1656) - 3-oxoacyl-(acyl carrier protein) synthase [Mycobacterium leprae str. TN]	88.37	404	404
fig|6666666.28482.peg.3276	R018920	(gi:221230351) kasB (MLBr 01656) - 3-oxoacyl-(acyl carrier protein) synthase II [Mycobacterium leprae str. Br4923]	88.37	404	404
fig|6666666.28482.peg.3276	R005647	(gi:118465046) kasB (MAV 2191) - 3-oxoacyl-[acyl-carrier-protein] synthase 2 [Mycobacterium avium str. 104]	86.95	404	404
fig|6666666.28482.peg.3276	R005646	(gi:41408097) kasB (MAP1999) - 3-oxoacyl-(acyl carrier protein) synthase [Mycobacterium avium str. K-10 (subsp. paratuberculosis)]	86.95	404	404
fig|6666666.28482.peg.3276	R005655	(gi:108800333) kasB (Mmcs 3367) - beta-ketoacyl synthase [Mycobacterium sp. str. MCS]	84.41	404	404
fig|6666666.28482.peg.3276	R005654	(gi:119869461) kasB (Mkms 3429) - beta-ketoacyl synthase [Mycobacterium sp. str. KMS]	84.41	404	404
fig|6666666.28482.peg.3276	R005653	(gi:126435956) kasB (Mjls 3378) - beta-ketoacyl synthase [Mycobacterium sp. str. JLS]	84.41	404	404

fig|6666666.28482.peg.3276	R005651	(gi:145223363) kasB (Mflv 2776) - beta-ketoacyl synthase [Mycobacterium gilvum str. PYR-GCK]	82.43	404	404
fig|6666666.28482.peg.3276	R005652	(gi:120404716) kasB (Mvan 3757) - beta-ketoacyl synthase [Mycobacterium vanbaalenii str. PYR-1]	82.18	404	404
fig|6666666.28482.peg.3276	R005650	(gi:118467655) kasB (MSMEG 4328) - 3-oxoacyl-[acyl-carrier-protein] synthase 2 [Mycobacterium smegmatis str. MC2 155]	81.44	404	404
fig|6666666.28482.peg.3276	R018922	(gi:333990289) kasB (JDM601 1649) - 3-oxoacyl-(acyl carrier protein) synthase II [Mycobacterium sp. str. JDM601]	79.16	398	404
fig|6666666.28482.peg.3276	R018921	(gi:333990667) kasB (JDM601 2027) - 3-oxoacyl-(acyl carrier protein) synthase II [Mycobacterium sp. str. JDM601]	77.48	404	404
fig|6666666.28482.peg.3423	R018631	(gi:126436732) Mjls4160 - cytochrome P450 [Mycobacterium sp. str. JLS]	69.43	350	352
fig|6666666.28482.peg.3423	R019850	(gi:253799175) TBMG02212 - cytochrome P450 143 cyp143 [Mycobacterium tuberculosis str. KZN 1435]	60.11	351	352
fig|6666666.28482.peg.3423	R018622	(gi:15608923) cyp143 (Rv1785c) - cytochrome P450 143 [Mycobacterium tuberculosis str. H37Rv]	60.11	351	352
fig|6666666.28482.peg.3423	R018623	(gi:148661592) cyp143 (MRA 1799) - putative cytochrome p450 143 CYP143 [Mycobacterium tuberculosis str. H37Ra]	60.11	351	352
fig|6666666.28482.peg.3423	R018621	(gi:148823000) TBFG11815 - cytochrome P450 143 cyp143 [Mycobacterium tuberculosis str. F11]	60.11	351	352
fig|6666666.28482.peg.3423	R019851	(gi:224990170) cyp143 (JTY 1801) - putative cytochrome P450 143 [Mycobacterium bovis str. BCG str. Tokyo 172]	60.11	351	352
fig|6666666.28482.peg.3423	R018626	(gi:121637686) cyp143 (BCG 1817c) - putative cytochrome P450 143 cyp143 [Mycobacterium bovis str. BCG Pasteur 1173P2]	60.11	351	352
fig|6666666.28482.peg.3423	R018625	(gi:31792973) cyp143 (Mb1813c) - cytochrome P450 143 [Mycobacterium bovis str. AF2122/97]	60.11	351	352
fig|6666666.28482.peg.3423	R019848	(gi:339631839) cyp143 (MAF 18070) - putative cytochrome P450 143 CYP143 [Mycobacterium africanum str. GM041182]	60.11	351	352
fig|6666666.28482.peg.3423	R019849	(gi:340626797) cyp143 (MCAN 18031) - putative cytochrome P450 143 CYP143 [Mycobacterium canettii str. CIPT 140010059]	60.11	351	352
fig|6666666.28482.peg.3423	R018624	(gi:15841253) MT1834 - P450 heme-thiolate protein [Mycobacterium tuberculosis str. CDC1551]	60.11	351	352

fig|6666666.28482.peg.3538	R006595	(gi:118464917) mce3F (MAV 2537) - virulence factor mce family protein [Mycobacterium avium str. 104]	80.45	491	492
fig|6666666.28482.peg.3538	R006607	(gi:126436737) mce3F (Mjls 4165) - virulence factor Mce family protein [Mycobacterium sp. str. JLS]	63.39	486	492
fig|6666666.28482.peg.3538	R006597	(gi:118619132) mce3F (MUL 3923) - MCE-family protein Mce3F 1 [Mycobacterium ulcerans str. Agy99]	63.66	487	492
fig|6666666.28482.peg.3538	R019408	(gi:183984027) mce3F (MMAR 4056) - MCE-family protein Mce3F 1 [Mycobacterium marinum str. M]	63.66	487	492
fig|6666666.28482.peg.3538	R006599	(gi:145222694) mce3F (Mflv 2106) - virulence factor Mce family protein [Mycobacterium gilvum str. PYR-GCK]	61.96	489	492
fig|6666666.28482.peg.3538	R019407	(gi:183982891) mce3F (MMAR 2887) - MCE-family protein Mce3F [Mycobacterium marinum str. M]	61.52	486	492
fig|6666666.28482.peg.3538	R006606	(gi:120405551) mce3F (Mvan 4599) - virulence factor Mce family protein [Mycobacterium vanbaalenii str. PYR-1]	61	486	492
fig|6666666.28482.peg.3538	R006603	(gi:120404931) mce3F (Mvan 3977) - virulence factor Mce family protein [Mycobacterium vanbaalenii str. PYR-1]	61.73	486	492
fig|6666666.28482.peg.3538	R006609	(gi:126436755) mce3F (Mjls 4183) - virulence factor Mce family protein [Mycobacterium sp. str. JLS]	61.93	486	492
fig|6666666.28482.peg.3538	R006593	(gi:15841446) mce3F (MT2023) - virulence factor mce family protein [Mycobacterium tuberculosis str. CDC1551]	61.5	422	492
fig|6666666.28482.peg.3538	R019409	(gi:340626980) mce3F (MCAN 19871) - MCE-family protein MCE3F [Mycobacterium canettii str. CIPT 140010059]	61.5	422	492
fig|6666666.28482.peg.3538	R006591	(gi:15609108) mce3F (Rv1971) - MCE-FAMILY PROTEIN MCE3F [Mycobacterium tuberculosis str. H37Rv]	61.5	422	492
fig|6666666.28482.peg.3538	R006592	(gi:148661780) mce3F (MRA 1982) - MCE-family protein Mce3F [Mycobacterium tuberculosis str. H37Ra]	61.5	422	492
fig|6666666.28482.peg.3538	R019410	(gi:253798981) mce3F (TBMG 02019) - MCE-family protein mce3F [Mycobacterium tuberculosis str. KZN 1435]	61.27	422	492
fig|6666666.28482.peg.3538	R013529	(gi:148823185) mce3F (TBFG 12002) - MCE-family protein mce3F [Mycobacterium tuberculosis str. F11]	61.27	422	492
fig|6666666.28482.peg.3539	R006572	(gi:118463194) mce3E (MAV 2536) - virulence factor Mce family protein [Mycobacterium avium str. 104]	80.66	362	385

fig|6666666.28482.peg.3539	R006584	(gi:126436738) mce3E (Mjls 4166) - transcriptional regulator, Fis family [Mycobacterium sp. str. JLS]	66.84	382	385
fig|6666666.28482.peg.3539	R019403	(gi:183982890) mce3E (MMAR 2886) - MCE family lipoprotein LprM [Mycobacterium marinum str. M]	65.36	383	385
fig|6666666.28482.peg.3539	R006574	(gi:118619131) mce3E (MUL 3922) - MCE-family lipoprotein LprM 1 [Mycobacterium ulcerans str. Agy99]	67.03	363	385
fig|6666666.28482.peg.3539	R019404	(gi:183984026) mce3E (MMAR 4055) - MCE family lipoprotein LprM [Mycobacterium marinum str. M]	66.76	363	385
fig|6666666.28482.peg.3539	R006586	(gi:126436756) mce3E (Mjls 4184) - virulence factor Mce family protein [Mycobacterium sp. str. JLS]	64.08	385	385
fig|6666666.28482.peg.3539	R006578	(gi:120401538) mce3E (Mvan 0517) - virulence factor Mce family protein [Mycobacterium vanbaalenii str. PYR-1]	63.61	381	385
fig|6666666.28482.peg.3539	R006590	(gi:108798627) mce3E (Mmcs 1658) - Mammalian cell entry [Mycobacterium sp. str. MCS]	63.61	381	385
fig|6666666.28482.peg.3539	R006588	(gi:119867723) mce3E (Mkms 1682) - virulence factor Mce family protein [Mycobacterium sp. str. KMS]	63.61	381	385
fig|6666666.28482.peg.3539	R006585	(gi:126434224) mce3E (Mjls 1631) - virulence factor Mce family protein [Mycobacterium sp. str. JLS]	63.61	381	385
fig|6666666.28482.peg.3539	R006577	(gi:145221247) mce3E (Mflv 0648) - virulence factor Mce family protein [Mycobacterium gilvum str. PYR-GCK]	63.35	381	385
fig|6666666.28482.peg.3539	R006583	(gi:120405552) mce3E (Mvan 4600) - virulence factor Mce family protein [Mycobacterium vanbaalenii str. PYR-1]	65.68	372	385
fig|6666666.28482.peg.3539	R019405	(gi:340626979) mce3E (MCAN 19861) - putative MCE-family lipoprotein LPRM [Mycobacterium canettii str. CIPT 140010059]	63.93	376	385
fig|6666666.28482.peg.3539	R019406	(gi:253798982) mce3E (TBMG 02020) - MCE-family lipoprotein mce3E [Mycobacterium tuberculosis str. KZN 1435]	63.66	376	385
fig|6666666.28482.peg.3539	R006568	(gi:15609107) mce3E (Rv1970) - POSSIBLE MCE-FAMILY LIPOPROTEIN LPRM (MCE-FAMILY LIPOPROTEIN MCE3E) [Mycobacterium tuberculosis str. H37Rv]	63.66	376	385
fig|6666666.28482.peg.3539	R006569	(gi:148661779) mce3E (MRA 1981) - MCE-family lipoprotein LprM [Mycobacterium tuberculosis str. H37Ra]	63.66	376	385
fig|6666666.28482.peg.3539	R013528	(gi:148823184) mce3E (TBFG 12001) - MCE-family lipoprotein lprM (MCE-family lipoprotein mce3e) [Mycobacterium tuberculosis str. F11]	63.66	376	385

fig|6666666.28482.peg.3539	R006570	(gi:15841445) mce3E (MT2022) - virulence factor mce family protein [Mycobacterium tuberculosis str. CDC1551]	63.66	376	385
fig|6666666.28482.peg.3539	R006576	(gi:145222693) mce3E (Mflv 2105) - virulence factor Mce family protein [Mycobacterium gilvum str. PYR-GCK]	63.8	384	385
fig|6666666.28482.peg.3539	R006581	(gi:120401941) mce3E (Mvan 0926) - virulence factor Mce family protein [Mycobacterium vanbaalenii str. PYR-1]	60.89	381	385
fig|6666666.28482.peg.3539	R006579	(gi:120401392) mce3E (Mvan 0367) - virulence factor Mce family protein [Mycobacterium vanbaalenii str. PYR-1]	61.01	376	385
fig|6666666.28482.peg.3539	R006575	(gi:118468700) mce3E (MSMEG 0350) - virulence factor Mce family protein [Mycobacterium smegmatis str. MC2 155]	61.68	367	385
fig|6666666.28482.peg.3540	R006549	(gi:118466668) mce3D (MAV 2535) - virulence factor mce family protein [Mycobacterium avium str. 104]	74.72	436	436
fig|6666666.28482.peg.3540	R006561	(gi:126436739) mce3D (Mjls 4167) - virulence factor Mce family protein [Mycobacterium sp. str. JLS]	65.07	435	436
fig|6666666.28482.peg.3540	R019399	(gi:183982889) mce3D (MMAR 2885) - MCE-family protein Mce3D [Mycobacterium marinum str. M]	61.84	435	436
fig|6666666.28482.peg.3540	R006545	(gi:15609106) mce3D (Rv1969) - MCE-FAMILY PROTEIN MCE3D [Mycobacterium tuberculosis str. H37Rv]	61.2	433	436
fig|6666666.28482.peg.3540	R006546	(gi:148661778) mce3D (MRA 1980) - MCE-family protein Mce3D [Mycobacterium tuberculosis str. H37Ra]	61.2	433	436
fig|6666666.28482.peg.3540	R013527	(gi:148823183) mce3D (TBFG 12000) - MCE-family protein mce3D [Mycobacterium tuberculosis str. F11]	61.2	433	436
fig|6666666.28482.peg.3540	R006547	(gi:15841444) mce3D (MT2021) - virulence factor mce family protein [Mycobacterium tuberculosis str. CDC1551]	61.2	433	436
fig|6666666.28482.peg.3540	R019402	(gi:253798983) mce3D (TBMG 02021) - MCE-family protein mce3D [Mycobacterium tuberculosis str. KZN 1435]	61.2	433	436
fig|6666666.28482.peg.3540	R006551	(gi:118619130) mce3D (MUL 3921) - MCE-family protein Mce3D 1 [Mycobacterium ulcerans str. Agy99]	64.23	411	436
fig|6666666.28482.peg.3540	R019401	(gi:340626978) mce3D (MCAN 19851) - MCE-family protein MCE3D [Mycobacterium canettii str. CIPT 140010059]	60.97	433	436
fig|6666666.28482.peg.3540	R006566	(gi:108798497) mce3D (Mmcs 1527) - Mammalian cell entry [Mycobacterium sp. str. MCS]	60.69	435	436

fig|6666666.28482.peg.3540	R006564	(gi:119867597) mce3D (Mkms 1550) - virulence factor Mce family protein [Mycobacterium sp. str. KMS]	60.69	435	436
fig|6666666.28482.peg.3540	R019400	(gi:183984025) mce3D (MMAR 4054) - MCE-family protein Mce3D 1 [Mycobacterium marinum str. M]	63.13	411	436
fig|6666666.28482.peg.3540	R006553	(gi:145222692) mce3D (Mflv 2104) - virulence factor Mce family protein [Mycobacterium gilvum str. PYR-GCK]	60.61	411	436
fig|6666666.28482.peg.3540	R006555	(gi:120401537) mce3D (Mvan 0516) - virulence factor Mce family protein [Mycobacterium vanbaalenii str. PYR-1]	60.45	435	436
fig|6666666.28482.peg.3540	R006567	(gi:108798626) mce3D (Mmcs 1657) - Mammalian cell entry [Mycobacterium sp. str. MCS]	60.45	435	436
fig|6666666.28482.peg.3540	R006565	(gi:119867722) mce3D (Mkms 1681) - virulence factor Mce family protein [Mycobacterium sp. str. KMS]	60.45	435	436
fig|6666666.28482.peg.3540	R006562	(gi:126434223) mce3D (Mjls 1630) - virulence factor Mce family protein [Mycobacterium sp. str. JLS]	60.45	435	436
fig|6666666.28482.peg.3540	R006554	(gi:145221248) mce3D (Mflv 0649) - virulence factor Mce family protein [Mycobacterium gilvum str. PYR-GCK]	60.45	435	436
fig|6666666.28482.peg.3540	R006560	(gi:120405553) mce3D (Mvan 4601) - virulence factor Mce family protein [Mycobacterium vanbaalenii str. PYR-1]	65.27	357	436
fig|6666666.28482.peg.3540	R006556	(gi:120401391) mce3D (Mvan 0366) - virulence factor Mce family protein [Mycobacterium vanbaalenii str. PYR-1]	62.02	413	436
fig|6666666.28482.peg.3541	R006526	(gi:118463925) mce3C (MAV 2534) - virulence factor Mce family protein [Mycobacterium avium str. 104]	79.3	401	469
fig|6666666.28482.peg.3541	R006538	(gi:126436740) mce3C (Mjls 4168) - virulence factor Mce family protein [Mycobacterium sp. str. JLS]	71.84	380	469
fig|6666666.28482.peg.3541	R019396	(gi:183984024) mce3C (MMAR 4053) - MCE-family protein, Mce3C 1 [Mycobacterium marinum str. M]	68.33	401	469
fig|6666666.28482.peg.3541	R006537	(gi:120405554) mce3C (Mvan 4602) - virulence factor Mce family protein [Mycobacterium vanbaalenii str. PYR-1]	67.72	441	469
fig|6666666.28482.peg.3541	R006530	(gi:145222691) mce3C (Mflv 2103) - virulence factor Mce family protein [Mycobacterium gilvum str. PYR-GCK]	68.33	400	469
fig|6666666.28482.peg.3541	R006540	(gi:126436758) mce3C (Mjls 4186) - virulence factor Mce family protein [Mycobacterium sp. str. JLS]	67.89	380	469

fig|6666666.28482.peg.3541	R006528	(gi:118619129) mce3C (MUL 3920) - MCE-family protein, Mce3C 1 [Mycobacterium ulcerans str. Agy99]	67.33	401	469
fig|6666666.28482.peg.3541	R006532	(gi:120401536) mce3C (Mvan 0515) - virulence factor Mce family protein [Mycobacterium vanbaalenii str. PYR-1]	65.25	400	469
fig|6666666.28482.peg.3541	R006544	(gi:108798625) mce3C (Mmcs 1656) - Mammalian cell entry [Mycobacterium sp. str. MCS]	65.25	400	469
fig|6666666.28482.peg.3541	R006542	(gi:119867721) mce3C (Mkms 1680) - virulence factor Mce family protein [Mycobacterium sp. str. KMS]	65.25	400	469
fig|6666666.28482.peg.3541	R006539	(gi:126434222) mce3C (Mjls 1629) - virulence factor Mce family protein [Mycobacterium sp. str. JLS]	65.25	400	469
fig|6666666.28482.peg.3541	R006531	(gi:145221249) mce3C (Mflv 0650) - virulence factor Mce family protein [Mycobacterium gilvum str. PYR-GCK]	65.25	400	469
fig|6666666.28482.peg.3541	R006543	(gi:108798496) mce3C (Mmcs 1526) - Mammalian cell entry [Mycobacterium sp. str. MCS]	65.19	450	469
fig|6666666.28482.peg.3541	R006541	(gi:119867596) mce3C (Mkms 1549) - virulence factor Mce family protein [Mycobacterium sp. str. KMS]	65.19	450	469
fig|6666666.28482.peg.3541	R006533	(gi:120401390) mce3C (Mvan 0365) - virulence factor Mce family protein [Mycobacterium vanbaalenii str. PYR-1]	65.09	401	469
fig|6666666.28482.peg.3541	R006534	(gi:120404934) mce3C (Mvan 3980) - virulence factor Mce family protein [Mycobacterium vanbaalenii str. PYR-1]	63.5	400	469
fig|6666666.28482.peg.3541	R006536	(gi:120405350) mce3C (Mvan 4397) - virulence factor Mce family protein [Mycobacterium vanbaalenii str. PYR-1]	61	400	469
fig|6666666.28482.peg.3541	R006535	(gi:120401939) mce3C (Mvan 0924) - virulence factor Mce family protein [Mycobacterium vanbaalenii str. PYR-1]	61.25	400	469
fig|6666666.28482.peg.3541	R019397	(gi:340626977) mce3C (MCAN 19841) - MCE-family protein MCE3C [Mycobacterium canettii str. CIPT 140010059]	65.26	380	469
fig|6666666.28482.peg.3541	R019398	(gi:253798984) mce3C (TBMG 02022) - MCE-family protein mce3C [Mycobacterium tuberculosis str. KZN 1435]	65.26	380	469
fig|6666666.28482.peg.3541	R006522	(gi:15609105) mce3C (Rv1968) - MCE-FAMILY PROTEIN MCE3C [Mycobacterium tuberculosis str. H37Rv]	65.26	380	469
fig|6666666.28482.peg.3541	R006523	(gi:148661777) mce3C (MRA 1979) - MCE-family protein Mce3C [Mycobacterium tuberculosis str. H37Ra]	65.26	380	469

fig|6666666.28482.peg.3541	R013526	(gi:148823182) mce3C (TBFG 11999) - MCE-family protein mce3C [Mycobacterium tuberculosis str. F11]	65.26	380	469
fig|6666666.28482.peg.3541	R006524	(gi:15841443) mce3C (MT2020) - virulence factor mce family protein [Mycobacterium tuberculosis str. CDC1551]	65.26	380	469
fig|6666666.28482.peg.3541	R006529	(gi:118470845) mce3C (MSMEG 0348) - mce-family protein mce3c [Mycobacterium smegmatis str. MC2 155]	61.32	380	469
fig|6666666.28482.peg.3541	R019395	(gi:183982888) mce3C (MMAR 2884) - MCE-family protein Mce3C [Mycobacterium marinum str. M]	64.74	380	469
fig|6666666.28482.peg.3542	R006504	(gi:118464539) mce3B (MAV 2533) - virulence factor Mce family protein [Mycobacterium avium str. 104]	80.99	342	342
fig|6666666.28482.peg.3542	R006516	(gi:126436741) mce3B (Mjls 4169) - virulence factor Mce family protein [Mycobacterium sp. str. JLS]	72.81	342	342
fig|6666666.28482.peg.3542	R006515	(gi:120405555) mce3B (Mvan 4603) - virulence factor Mce family protein [Mycobacterium vanbaalenii str. PYR-1]	71.64	333	342
fig|6666666.28482.peg.3542	R006521	(gi:108798624) mce3B (Mmcs 1655) - Mammalian cell entry [Mycobacterium sp. str. MCS]	69.59	342	342
fig|6666666.28482.peg.3542	R006519	(gi:119867720) mce3B (Mkms 1679) - virulence factor Mce family protein [Mycobacterium sp. str. KMS]	69.59	342	342
fig|6666666.28482.peg.3542	R006517	(gi:126434221) mce3B (Mjls 1628) - virulence factor Mce family protein [Mycobacterium sp. str. JLS]	69.59	342	342
fig|6666666.28482.peg.3542	R006509	(gi:145221250) mce3B (Mflv 0651) - virulence factor Mce family protein [Mycobacterium gilvum str. PYR-GCK]	69.59	342	342
fig|6666666.28482.peg.3542	R006508	(gi:145222690) mce3B (Mflv 2102) - virulence factor Mce family protein [Mycobacterium gilvum str. PYR-GCK]	70.06	332	342
fig|6666666.28482.peg.3542	R019392	(gi:183984023) mce3B (MMAR 4052) - MCE-family protein Mce3B 1 [Mycobacterium marinum str. M]	68.22	342	342
fig|6666666.28482.peg.3542	R006506	(gi:118619128) mce3B (MUL 3919) - MCE-family protein Mce3B 1 [Mycobacterium ulcerans str. Agy99]	68.22	342	342
fig|6666666.28482.peg.3542	R006511	(gi:120401389) mce3B (Mvan 0364) - virulence factor Mce family protein [Mycobacterium vanbaalenii str. PYR-1]	67.25	342	342
fig|6666666.28482.peg.3542	R019391	(gi:183982887) mce3B (MMAR 2883) - MCE-family protein Mce3B [Mycobacterium marinum str. M]	68.42	342	342

fig|6666666.28482.peg.3542	R006500	(gi:15609104) mce3B (Rv1967) - MCE-FAMILY PROTEIN MCE3B [Mycobacterium tuberculosis str. H37Rv]	66.96	342	342
fig|6666666.28482.peg.3542	R006501	(gi:148661776) mce3B (MRA 1978) - MCE-family protein Mce3B [Mycobacterium tuberculosis str. H37Ra]	66.96	342	342
fig|6666666.28482.peg.3542	R013525	(gi:148823181) mce3B (TBFG 11998) - MCE-family protein mce3B [Mycobacterium tuberculosis str. F11]	66.96	342	342
fig|6666666.28482.peg.3542	R006502	(gi:15841442) mce3B (MT2019) - virulence factor mce family protein [Mycobacterium tuberculosis str. CDC1551]	66.96	342	342
fig|6666666.28482.peg.3542	R006518	(gi:126436759) mce3B (Mjls 4187) - virulence factor Mce family protein [Mycobacterium sp. str. JLS]	67.54	342	342
fig|6666666.28482.peg.3542	R019394	(gi:253798985) mce3B (TBMG 02023) - MCE-family protein mce3B [Mycobacterium tuberculosis str. KZN 1435]	66.96	342	342
fig|6666666.28482.peg.3542	R019393	(gi:340626976) mce3B (MCAN 19831) - MCE-family protein MCE3B [Mycobacterium canettii str. CIPT 140010059]	66.67	342	342
fig|6666666.28482.peg.3542	R006512	(gi:120404935) mce3B (Mvan 3981) - virulence factor Mce family protein [Mycobacterium vanbaalenii str. PYR-1]	63.74	342	342
fig|6666666.28482.peg.3542	R006514	(gi:120405351) mce3B (Mvan 4398) - virulence factor Mce family protein [Mycobacterium vanbaalenii str. PYR-1]	61.99	342	342
fig|6666666.28482.peg.3542	R006510	(gi:120401523) mce3B (Mvan 0501) - virulence factor Mce family protein [Mycobacterium vanbaalenii str. PYR-1]	70.07	274	342
fig|6666666.28482.peg.3542	R006520	(gi:108798495) mce3B (Mmcs 1525) - Mammalian cell entry [Mycobacterium sp. str. MCS]	61.18	255	342
fig|6666666.28482.peg.3543	R006481	(gi:118465483) mce3A (MAV 2532) - virulence factor Mce family protein [Mycobacterium avium str. 104]	76.48	438	493
fig|6666666.28482.peg.3543	R006493	(gi:126436742) mce3A (Mjls 4170) - virulence factor Mce family protein [Mycobacterium sp. str. JLS]	61.96	437	493
fig|6666666.28482.peg.3543	R006498	(gi:108798494) mce3A (Mmcs 1524) - Mammalian cell entry [Mycobacterium sp. str. MCS]	60.05	397	493
fig|6666666.28482.peg.3543	R006496	(gi:119867595) mce3A (Mkms 1547) - virulence factor Mce family protein [Mycobacterium sp. str. KMS]	60.05	397	493
fig|6666666.28482.peg.3543	R006483	(gi:118619127) mce3A (MUL 3918) - MCE-family protein Mce3A 1 [Mycobacterium ulcerans str. Agy99]	60.47	463	493

fig|6666666.28482.peg.3543	R019388	(gi:183984022) mce3A (MMAR 4051) - MCE-family protein Mce3A 1 [Mycobacterium marinum str. M]	60.86	460	493
fig|6666666.28482.peg.3550	R018641	(gi:118465392) MAV2592 - hypothetical protein [Mycobacterium avium str. 104]	74.19	62	65
fig|6666666.28482.peg.3550	R018643	(gi:126436671) Mjls4098 - hypothetical protein [Mycobacterium sp. str. JLS]	68.75	64	65
fig|6666666.28482.peg.3550	R018640	(gi:41407602) MAP1504 - hypothetical protein [Mycobacterium avium str. K-10 (subsp. paratuberculosis)]	67.19	64	65
fig|6666666.28482.peg.3550	R018642	(gi:118618473) MUL3090 - ferredoxin [Mycobacterium ulcerans str. Agy99]	67.74	62	65
fig|6666666.28482.peg.3550	R019852	(gi:183982674) MMAR2667 - ferredoxin [Mycobacterium marinum str. M]	67.74	62	65
fig|6666666.28482.peg.3550	R019855	(gi:253799174) TBMG02211 - ferredoxin [Mycobacterium tuberculosis str. KZN 1435]	68.75	64	65
fig|6666666.28482.peg.3550	R018635	(gi:15608924) Rv1786 - ferredoxin [Mycobacterium tuberculosis str. H37Rv]	68.75	64	65
fig|6666666.28482.peg.3550	R018636	(gi:148661593) MRA1800 - putative ferredoxin [Mycobacterium tuberculosis str. H37Ra]	68.75	64	65
fig|6666666.28482.peg.3550	R018634	(gi:148823001) TBFG11816 - ferredoxin [Mycobacterium tuberculosis str. F11]	68.75	64	65
fig|6666666.28482.peg.3550	R018637	(gi:15841254) MT1835 - ferredoxin, putative [Mycobacterium tuberculosis str. CDC1551]	68.75	64	65
fig|6666666.28482.peg.3550	R019856	(gi:224990171) JTY1802 - putative ferredoxin [Mycobacterium bovis str. BCG str. Tokyo 172]	68.75	64	65
fig|6666666.28482.peg.3550	R018639	(gi:121637687) BCG1818 - putative ferredoxin [Mycobacterium bovis str. BCG Pasteur 1173P2]	68.75	64	65
fig|6666666.28482.peg.3550	R018638	(gi:31792974) Mb1814 - putative ferredoxin [Mycobacterium bovis str. AF2122/97]	68.75	64	65
fig|6666666.28482.peg.3550	R019853	(gi:339631840) MAF18080 - putative ferredoxin [Mycobacterium africanum str. GM041182]	68.75	64	65
fig|6666666.28482.peg.3550	R019854	(gi:340626798) MCAN18041 - putative ferredoxin [Mycobacterium canettii str. CIPT 140010059]	67.19	64	65

fig|6666666.28482.peg.3643	R005437	(gi:118465788) fbpC (MAV 5183) - antigen 85-C [Mycobacterium avium str. 104]	90.66	332	346
fig|6666666.28482.peg.3643	R005436	(gi:41409629) fbpC (MAP3531c) - FbpC2 [Mycobacterium avium str. K-10 (subsp. paratuberculosis)]	90.36	332	346
fig|6666666.28482.peg.3643	R005433	(gi:15839510) fbpC (MT0137) - esterase, putative, antigen 85-C [Mycobacterium tuberculosis str. CDC1551]	85.93	327	346
fig|6666666.28482.peg.3643	R018882	(gi:253797048) fbpC (TBMG 00130) - secreted fibronectin-binding protein C antigen 85-C fbpC [Mycobacterium tuberculosis str. KZN 1435]	85.93	327	346
fig|6666666.28482.peg.3643	R005431	(gi:57116693) fbpC (Rv0129c) - SECRETED ANTIGEN 85-C FBPC (85C) (ANTIGEN 85 COMPLEX C) (AG58C) (MYCOLYL TRANSFERASE 85C) (FIBRONECTIN-BINDING PROTEIN C) [Mycobacterium tuberculosis str. H37Rv]	85.93	327	346
fig|6666666.28482.peg.3643	R005432	(gi:148659893) fbpC (MRA 0136) - secreted antigen 86-C FbpC [Mycobacterium tuberculosis str. H37Ra]	85.93	327	346
fig|6666666.28482.peg.3643	R013426	(gi:148821321) fbpC (TBFG 10130) - secreted antigen 85-C fbpC (fibronectin-binding protein C) [Mycobacterium tuberculosis str. F11]	85.93	327	346
fig|6666666.28482.peg.3643	R018881	(gi:340625164) fbpC (MCAN 01321) - secreted antigen 85-C FBPC [Mycobacterium canettii str. CIPT 140010059]	85.93	327	346
fig|6666666.28482.peg.3643	R018883	(gi:224988515) fbpC (JTY 0133) - secreted antigen 85-C [Mycobacterium bovis str. BCG str. Tokyo 172]	85.93	327	346
fig|6666666.28482.peg.3643	R005435	(gi:121636042) fbpC (BCG 0163c) - Secreted antigen 85-c fbpC (85C) [Mycobacterium bovis str. BCG Pasteur 1173P2]	85.93	327	346
fig|6666666.28482.peg.3643	R005434	(gi:31791308) fbpC (Mb0134c) - SECRETED ANTIGEN 85-C FBPC (85C) (ANTIGEN 85 COMPLEX C) (AG58C) (MYCOLYL TRANSFERASE 85C) (FIBRONECTIN-BINDING PROTEIN C) [Mycobacterium bovis str. AF2122/97]	85.93	327	346
fig|6666666.28482.peg.3643	R018880	(gi:339630211) fbpC (MAF 01290) - secreted antigen 85-C FbpC [Mycobacterium africanum str. GM041182]	85.93	327	346
fig|6666666.28482.peg.3643	R005438	(gi:15828432) fbpC (ML2655) - secreted antigen 85A, mycolyltransferase [Mycobacterium leprae str. TN]	81.98	333	346
fig|6666666.28482.peg.3643	R018884	(gi:221230909) fbpC (MLBr 02655) - secreted antigen 85A, mycolyltransferase [Mycobacterium leprae str. Br4923]	81.98	333	346

fig|6666666.28482.peg.3643	R005439	(gi:118619846) fbpC (MUL 4793) - secreted antigen 85-C FbpC [Mycobacterium ulcerans str. Agy99]	86.85	327	346
fig|6666666.28482.peg.3643	R018879	(gi:183980359) fbpC (MMAR 0328) - secreted antigen 85-C FbpC [Mycobacterium marinum str. M]	86.85	327	346
fig|6666666.28482.peg.3643	R018885	(gi:333992822) fbpC (JDM601 4182) - hypothetical protein [Mycobacterium sp. str. JDM601]	84.21	323	346
fig|6666666.28482.peg.3643	R005464	(gi:108799739) fbpC (Mmcs 2773) - putative esterase [Mycobacterium sp. str. MCS]	72.73	325	346
fig|6666666.28482.peg.3643	R005459	(gi:119868849) fbpC (Mkms 2817) - putative esterase [Mycobacterium sp. str. KMS]	72.73	325	346
fig|6666666.28482.peg.3643	R005454	(gi:126435380) fbpC (Mjls 2800) - putative esterase [Mycobacterium sp. str. JLS]	72.73	325	346
fig|6666666.28482.peg.3643	R005440	(gi:118467737) fbpC (MSMEG 3580) - antigen 85-C [Mycobacterium smegmatis str. MC2 155]	72.04	323	346
fig|6666666.28482.peg.3643	R005443	(gi:145223921) fbpC (Mflv 3335) - putative esterase [Mycobacterium gilvum str. PYR-GCK]	73.25	324	346
fig|6666666.28482.peg.3643	R005430	(gi:118473709) fbpB (MSMEG 2078) - antigen 85-C [Mycobacterium smegmatis str. MC2 155]	70	326	346
fig|6666666.28482.peg.3643	R005448	(gi:120404040) fbpC (Mvan 3061) - putative esterase [Mycobacterium vanbaalenii str. PYR-1]	75.74	300	346
fig|6666666.28482.peg.3643	R018868	(gi:253800852) fbpA (TBMG 03851) - secreted fibronectin-binding protein antigen fbpA [Mycobacterium tuberculosis str. KZN 1435]	66.86	336	346
fig|6666666.28482.peg.3643	R005411	(gi:15610940) fbpA (Rv3804c) - SECRETED ANTIGEN 85-A FBPA (MYCOLYL TRANSFERASE 85A) (FIBRONECTIN-BINDING PROTEIN A) (ANTIGEN 85 COMPLEX A) [Mycobacterium tuberculosis str. H37Rv]	66.86	336	346
fig|6666666.28482.peg.3643	R005412	(gi:148663672) fbpA (MRA 3844) - secreted antigen 85-A FbpA [Mycobacterium tuberculosis str. H37Ra]	66.86	336	346
fig|6666666.28482.peg.3643	R013424	(gi:148825011) fbpA (TBFG 13838) - secreted antigen 85-A fbpA (fibronectin-binding protein A) [Mycobacterium tuberculosis str. F11]	66.86	336	346
fig|6666666.28482.peg.3643	R018867	(gi:340628773) fbpA (MCAN 38231) - secreted antigen 85-A FBPA [Mycobacterium canettii str. CIPT 140010059]	66.86	336	346
fig|6666666.28482.peg.3643	R018869	(gi:224992216) fbpA (JTY 3868) - secreted antigen 85-A [Mycobacterium bovis str. BCG str. Tokyo 172]	66.86	336	346

fig|6666666.28482.peg.3643	R005415	(gi:121639721) fbpA (BCG 3866c) - Secreted antigen 85-A fbpA [Mycobacterium bovis str. BCG Pasteur 1173P2]	66.86	336	346
fig|6666666.28482.peg.3643	R005414	(gi:31794978) fbpA (Mb3834c) - SECRETED ANTIGEN 85-A FBPA (MYCOLYL TRANSFERASE 85A) (FIBRONECTIN-BINDING PROTEIN A) (ANTIGEN 85 COMPLEX A) [Mycobacterium bovis str. AF2122/97]	66.86	336	346
fig|6666666.28482.peg.3643	R005413	(gi:15843426) fbpA (MT3911) - esterase, putative, antigen 85-A [Mycobacterium tuberculosis str. CDC1551]	66.86	336	346
fig|6666666.28482.peg.3643	R018866	(gi:339633799) fbpA (MAF 38190) - secreted antigen 85-A FBPA (mycolyl transferase 85A) [Mycobacterium africanum str. GM041182]	66.57	336	346
fig|6666666.28482.peg.3643	R005446	(gi:120402899) fbpC (Mvan 1902) - putative esterase [Mycobacterium vanbaalenii str. PYR-1]	68.63	319	346
fig|6666666.28482.peg.3643	R005444	(gi:145225038) fbpC (Mflv 4459) - putative esterase [Mycobacterium gilvum str. PYR-GCK]	68.01	319	346
fig|6666666.28482.peg.3643	R005417	(gi:118467129) fbpA (MAV 0214) - antigen 85-A [Mycobacterium avium str. 104]	68.63	320	346
fig|6666666.28482.peg.3643	R005466	(gi:108801978) fbpC (Mmcs 5015) - putative esterase [Mycobacterium sp. str. MCS]	67.46	326	346
fig|6666666.28482.peg.3643	R005461	(gi:119871130) fbpC (Mkms 5103) - putative esterase [Mycobacterium sp. str. KMS]	67.46	326	346
fig|6666666.28482.peg.3643	R005456	(gi:126437959) fbpC (Mjls 5396) - putative esterase [Mycobacterium sp. str. JLS]	67.46	326	346
fig|6666666.28482.peg.3643	R005416	(gi:41406314) fbpA (MAP0216) - FbpA [Mycobacterium avium str. K-10 (subsp. paratuberculosis)]	68.32	320	346
fig|6666666.28482.peg.3643	R018864	(gi:183985336) fbpA (MMAR 5368) - secreted antigen 85-A FbpA [Mycobacterium marinum str. M]	66.37	331	346
fig|6666666.28482.peg.3643	R005441	(gi:145221756) fbpC (Mflv 1164) - putative esterase [Mycobacterium gilvum str. PYR-GCK]	66.77	323	346
fig|6666666.28482.peg.3643	R005419	(gi:118620004) fbpA (MUL 4987) - secreted antigen 85-A FbpA [Mycobacterium ulcerans str. Agy99]	66.07	331	346
fig|6666666.28482.peg.3643	R018872	(gi:183982783) fbpB (MMAR 2777) - secreted antigen 85-B FbpB [Mycobacterium marinum str. M]	67.28	322	346
fig|6666666.28482.peg.3643	R005429	(gi:118618369) fbpB (MUL 2970) - secreted antigen 85-B FbpB [Mycobacterium ulcerans str. Agy99]	66.67	322	346

fig|6666666.28482.peg.3643	R005418	(gi:15826932) fbpA (ML0097) - antigen 85A, mycolytransferase [Mycobacterium leprae str. TN]	64.33	326	346
fig|6666666.28482.peg.3643	R018870	(gi:221229410) fbpA (MLBr 00097) - antigen 85A, mycolytransferase [Mycobacterium leprae str. Br4923]	64.33	326	346
fig|6666666.28482.peg.3643	R005426	(gi:41407707) fbpB (MAP1609c) - FbpB [Mycobacterium avium str. K-10 (subsp. paratuberculosis)]	68.59	310	346
fig|6666666.28482.peg.3643	R005427	(gi:118465697) fbpB (MAV 2816) - antigen 85-B [Mycobacterium avium str. 104]	68.27	310	346
fig|6666666.28482.peg.3643	R018875	(gi:253799070) fbpB (TBMG 02108) - secreted fibronectin-binding protein antigen 85-B fbpB [Mycobacterium tuberculosis str. KZN 1435]	68.42	321	346
fig|6666666.28482.peg.3643	R005421	(gi:15609023) fbpB (Rv1886c) - SECRETED ANTIGEN 85-B FBPB (85B) (ANTIGEN 85 COMPLEX B) (MYCOLYL TRANSFERASE 85B) (FIBRONECTIN-BINDING PROTEIN B) (EXTRACELLULAR ALPHA-ANTIGEN) [Mycobacterium tuberculosis str. H37Rv]	68.42	321	346
fig|6666666.28482.peg.3643	R005422	(gi:148661692) fbpB (MRA 1897) - secreted antigen 85-B FbpB [Mycobacterium tuberculosis str. H37Ra]	68.42	321	346
fig|6666666.28482.peg.3643	R013425	(gi:148823097) fbpB (TBFG 11914) - secreted antigen 85-B fbpB (fibronectin-binding protein B) [Mycobacterium tuberculosis str. F11]	68.42	321	346
fig|6666666.28482.peg.3643	R018874	(gi:340626894) fbpB (MCAN 19011) - secreted antigen 85-B FBPB [Mycobacterium canettii str. CIPT 140010059]	68.42	321	346
fig|6666666.28482.peg.3643	R005424	(gi:31793077) fbpB (Mb1918c) - secreted antigen 85-B fbpB (85B) (antigen 85 complex B) (Mycolyl transferase 85B) (Fibronectin-binding protein B) (Extracellular alpha-antigen) [Mycobacterium bovis str. AF2122/97]	68.42	321	346
fig|6666666.28482.peg.3643	R018873	(gi:339631939) fbpB (MAF 19080) - secreted antigen 85-B FBPB (85B) (antigen 85 complex B) [Mycobacterium africanum str. GM041182]	68.42	321	346
fig|6666666.28482.peg.3643	R005423	(gi:15841356) fbpB (MT1934) - esterase, putative, antigen 85-B [Mycobacterium tuberculosis str. CDC1551]	68.42	321	346
fig|6666666.28482.peg.3643	R005420	(gi:118468744) fbpA (MSMEG 6398) - antigen 85-A [Mycobacterium smegmatis str. MC2 155]	62.87	328	346
fig|6666666.28482.peg.3643	R018876	(gi:224990274) fbpB (JTY 1907) - secreted antigen 85-B [Mycobacterium bovis str. BCG str. Tokyo 172]	68.11	321	346
fig|6666666.28482.peg.3643	R005425	(gi:121637790) fbpB (BCG 1923c) - secreted antigen 85-B fbpB [Mycobacterium bovis str. BCG Pasteur 1173P2]	68.11	321	346

fig|6666666.28482.peg.3643	R005455	(gi:126437080) fbpC (Mjls 4511) - putative esterase [Mycobacterium sp. str. JLS]	64.92	316	346
fig|6666666.28482.peg.3643	R005452	(gi:126434164) fbpC (Mjls 1566) - putative esterase [Mycobacterium sp. str. JLS]	66.97	325	346
fig|6666666.28482.peg.3643	R005462	(gi:108798566) fbpC (Mmcs 1596) - putative esterase [Mycobacterium sp. str. MCS]	65.75	325	346
fig|6666666.28482.peg.3643	R005457	(gi:119867665) fbpC (Mkms 1620) - putative esterase [Mycobacterium sp. str. KMS]	65.75	325	346
fig|6666666.28482.peg.3643	R005428	(gi:15828096) fbpB (ML2028) - antigen 85A, mycolyltransferase [Mycobacterium leprae str. TN]	64.4	321	346
fig|6666666.28482.peg.3643	R018877	(gi:221230573) fbpB (MLBr 02028) - antigen 85A, mycolyltransferase [Mycobacterium leprae str. Br4923]	64.4	321	346
fig|6666666.28482.peg.3643	R018878	(gi:333990613) fbpB (JDM601 1973) - hypothetical protein [Mycobacterium sp. str. JDM601]	63.61	313	346
fig|6666666.28482.peg.3643	R005442	(gi:145223828) fbpC (Mflv 3241) - putative esterase [Mycobacterium gilvum str. PYR-GCK]	62.7	310	346
fig|6666666.28482.peg.3643	R005447	(gi:120403937) fbpC (Mvan 2954) - putative esterase [Mycobacterium vanbaalenii str. PYR-1]	61.34	312	346
fig|6666666.28482.peg.3643	R005465	(gi:108801146) fbpC (Mmcs 4182) - putative esterase [Mycobacterium sp. str. MCS]	61.89	301	346
fig|6666666.28482.peg.3643	R005460	(gi:119870278) fbpC (Mkms 4248) - putative esterase [Mycobacterium sp. str. KMS]	61.89	301	346
fig|6666666.28482.peg.3643	R005445	(gi:145225722) fbpC (Mflv 5146) - putative esterase [Mycobacterium gilvum str. PYR-GCK]	60.26	307	346
fig|6666666.28482.peg.3643	R018871	(gi:333992762) fbpA (JDM601 4122) - hypothetical protein [Mycobacterium sp. str. JDM601]	63.64	314	346
fig|6666666.28482.peg.3643	R005458	(gi:119867920) fbpC (Mkms 1882) - putative esterase [Mycobacterium sp. str. KMS]	61.47	326	346
fig|6666666.28482.peg.3643	R005453	(gi:126434404) fbpC (Mjls 1816) - putative esterase [Mycobacterium sp. str. JLS]	61.47	326	346
fig|6666666.28482.peg.3742	R006348	(gi:118465982) mce1A (MAV 5015) - virulence factor Mce family protein [Mycobacterium avium str. 104]	76.98	442	448

fig|6666666.28482.peg.3742	R006346	(gi:41409702) mce1A (MAP3604) - Mce1 2 [Mycobacterium avium str. K-10 (subsp. paratuberculosis)]	75.17	442	448
fig|6666666.28482.peg.3742	R006351	(gi:15828393) mce1A (ML2589) - putative cell invasion protein [Mycobacterium leprae str. TN]	75.91	440	448
fig|6666666.28482.peg.3742	R019325	(gi:221230870) mce1A (MLBr 02589) - putative cell invasion protein [Mycobacterium leprae str. Br4923]	75.91	440	448
fig|6666666.28482.peg.3742	R006350	(gi:118464886) mce1A (MAV 4126) - virulence factor Mce family protein [Mycobacterium avium str. 104]	75.51	440	448
fig|6666666.28482.peg.3742	R006349	(gi:118464153) mce1A (MAV 4125) - virulence factor Mce family protein [Mycobacterium avium str. 104]	72.81	444	448
fig|6666666.28482.peg.3742	R006347	(gi:41409387) mce1A (MAP3289c) - Mce1 1 [Mycobacterium avium str. K-10 (subsp. paratuberculosis)]	73.24	440	448
fig|6666666.28482.peg.3742	R019323	(gi:253797091) mce1A (TBMG 00170) - MCE-family protein mce1A [Mycobacterium tuberculosis str. KZN 1435]	70.07	442	448
fig|6666666.28482.peg.3742	R006341	(gi:57116701) mce1A (Rv0169) - MCE-FAMILY PROTEIN MCE1A [Mycobacterium tuberculosis str. H37Rv]	70.07	442	448
fig|6666666.28482.peg.3742	R006342	(gi:148659933) mce1A (MRA 0177) - MCE-family protein Mce1A [Mycobacterium tuberculosis str. H37Ra]	70.07	442	448
fig|6666666.28482.peg.3742	R013512	(gi:148821361) mce1A (TBFG 10170) - MCE-family protein mce1A [Mycobacterium tuberculosis str. F11]	70.07	442	448
fig|6666666.28482.peg.3742	R006343	(gi:15839547) mce1A (MT0178) - virulence factor [Mycobacterium tuberculosis str. CDC1551]	70.07	442	448
fig|6666666.28482.peg.3742	R019320	(gi:183980443) mce1A (MMAR 0412) - MCE-family protein Mce1A [Mycobacterium marinum str. M]	70.59	442	448
fig|6666666.28482.peg.3742	R006352	(gi:118616795) mce1A (MUL 1062) - MCE-family protein Mce1A [Mycobacterium ulcerans str. Agy99]	70.36	442	448
fig|6666666.28482.peg.3742	R019322	(gi:340625206) mce1A (MCAN 01751) - MCE-family protein MCE1A [Mycobacterium canettii str. CIPT 140010059]	69.84	442	448
fig|6666666.28482.peg.3742	R019324	(gi:224988554) mce1A (JTY 0175) - MCE-family protein [Mycobacterium bovis str. BCG str. Tokyo 172]	69.84	442	448
fig|6666666.28482.peg.3742	R006345	(gi:121636081) mce1A (BCG 0206) - MCE-family protein mce1A [Mycobacterium bovis str. BCG Pasteur 1173P2]	69.84	442	448

fig|6666666.28482.peg.3742	R006344	(gi:31791347) mce1A (Mb0175) - MCE-FAMILY PROTEIN MCE1A [Mycobacterium bovis str. AF2122/97]	69.84	442	448
fig|6666666.28482.peg.3742	R019321	(gi:339630250) mce1A (MAF 01700) - MCE-family protein MCE1A [Mycobacterium africanum str. GM041182]	69.84	442	448
fig|6666666.28482.peg.3742	R006410	(gi:118465139) mce2A (MAV 4551) - virulence factor Mce family protein [Mycobacterium avium str. 104]	61.65	412	448
fig|6666666.28482.peg.3742	R006409	(gi:41410182) mce2A (MAP4084) - Mce2 [Mycobacterium avium str. K-10 (subsp. paratuberculosis)]	60.64	409	448
fig|6666666.28482.peg.3743	R006359	(gi:41409703) mce1B (MAP3605) - hypothetical protein [Mycobacterium avium str. K-10 (subsp. paratuberculosis)]	88.73	346	346
fig|6666666.28482.peg.3743	R006360	(gi:118463171) mce1B (MAV 5014) - virulence factor Mce family protein [Mycobacterium avium str. 104]	88.73	346	346
fig|6666666.28482.peg.3743	R019327	(gi:183980444) mce1B (MMAR 0413) - MCE-family protein Mce1B [Mycobacterium marinum str. M]	81.5	346	346
fig|6666666.28482.peg.3743	R006362	(gi:118616796) mce1B (MUL 1063) - MCE-family protein Mce1B [Mycobacterium ulcerans str. Agy99]	81.21	346	346
fig|6666666.28482.peg.3743	R006361	(gi:15828394) mce1B (ML2590) - putative secreted protein [Mycobacterium leprae str. TN]	81.5	346	346
fig|6666666.28482.peg.3743	R019332	(gi:221230871) mce1B (MLBr 02590) - putative secreted protein [Mycobacterium leprae str. Br4923]	81.5	346	346
fig|6666666.28482.peg.3743	R019329	(gi:340625207) mce1B (MCAN 01761) - MCE-family protein MCE1B [Mycobacterium canettii str. CIPT 140010059]	78.03	346	346
fig|6666666.28482.peg.3743	R019330	(gi:253797092) mce1B (TBMG 00171) - MCE-family protein mce1B [Mycobacterium tuberculosis str. KZN 1435]	77.46	346	346
fig|6666666.28482.peg.3743	R006354	(gi:15607311) mce1B (Rv0170) - MCE-FAMILY PROTEIN MCE1B [Mycobacterium tuberculosis str. H37Rv]	77.46	346	346
fig|6666666.28482.peg.3743	R006355	(gi:148659934) mce1B (MRA 0178) - MCE-family protein Mce1B [Mycobacterium tuberculosis str. H37Ra]	77.46	346	346
fig|6666666.28482.peg.3743	R013513	(gi:148821362) mce1B (TBFG 10171) - MCE-family protein mce1B [Mycobacterium tuberculosis str. F11]	77.46	346	346
fig|6666666.28482.peg.3743	R006356	(gi:15839548) mce1B (MT0179) - virulence factor mce family protein [Mycobacterium tuberculosis str. CDC1551]	77.46	346	346

fig|6666666.28482.peg.3743	R019331	(gi:224988555) mce1B (JTY 0176) - MCE-family protein [Mycobacterium bovis str. BCG str. Tokyo 172]	77.17	346	346
fig|6666666.28482.peg.3743	R006358	(gi:121636082) mce1B (BCG 0207) - MCE-family protein mce1B [Mycobacterium bovis str. BCG Pasteur 1173P2]	77.17	346	346
fig|6666666.28482.peg.3743	R006357	(gi:31791348) mce1B (Mb0176) - MCE-FAMILY PROTEIN MCE1B [Mycobacterium bovis str. AF2122/97]	77.17	346	346
fig|6666666.28482.peg.3743	R019328	(gi:339630251) mce1B (MAF 01710) - MCE-family protein MCE1B [Mycobacterium africanum str. GM041182]	77.17	346	346
fig|6666666.28482.peg.3743	R006428	(gi:108797099) mce2B (Mmcs 0119) - Mammalian cell entry [Mycobacterium sp. str. MCS]	68.5	346	346
fig|6666666.28482.peg.3743	R006427	(gi:119866184) mce2B (Mkms 0128) - virulence factor Mce family protein [Mycobacterium sp. str. KMS]	68.5	346	346
fig|6666666.28482.peg.3743	R006426	(gi:126432722) mce2B (Mjls 0109) - virulence factor Mce family protein [Mycobacterium sp. str. JLS]	68.5	346	346
fig|6666666.28482.peg.3743	R006363	(gi:118468987) mce1B (MSMEG 0135) - virulence factor Mce family protein [Mycobacterium smegmatis str. MC2 155]	65.32	346	346
fig|6666666.28482.peg.3743	R006424	(gi:145221305) mce2B (Mflv 0710) - virulence factor Mce family protein [Mycobacterium gilvum str. PYR-GCK]	66.47	346	346
fig|6666666.28482.peg.3743	R006425	(gi:120401163) mce2B (Mvan 0135) - virulence factor Mce family protein [Mycobacterium vanbaalenii str. PYR-1]	65.9	346	346
fig|6666666.28482.peg.3743	R019333	(gi:333988763) mce1B (JDM601 0123) - MCE-family protein Mce1B [Mycobacterium sp. str. JDM601]	68.5	346	346
fig|6666666.28482.peg.3743	R019368	(gi:340625620) mce2B (MCAN 05961) - mce-family protein mce2b [Mycobacterium canettii str. CIPT 140010059]	64.45	346	346
fig|6666666.28482.peg.3743	R019370	(gi:224988980) mce2B (JTY 0605) - MCE-family protein [Mycobacterium bovis str. BCG str. Tokyo 172]	64.45	346	346
fig|6666666.28482.peg.3743	R006421	(gi:121636508) mce2B (BCG 0635) - mce-family protein mce2B [Mycobacterium bovis str. BCG Pasteur 1173P2]	64.45	346	346
fig|6666666.28482.peg.3743	R006420	(gi:31791772) mce2B (Mb0605) - MCE-FAMILY PROTEIN MCE2B [Mycobacterium bovis str. AF2122/97]	64.45	346	346
fig|6666666.28482.peg.3743	R019367	(gi:339630658) mce2B (MAF 05970) - MCE-family protein MCE2B [Mycobacterium africanum str. GM041182]	64.45	346	346

fig|6666666.28482.peg.3743	R006422	(gi:41410183) mce2B (MAP4085) - hypothetical protein [Mycobacterium avium str. K-10 (subsp. paratuberculosis)]	63.58	346	346
fig|6666666.28482.peg.3743	R006423	(gi:118465867) mce2B (MAV 4550) - virulence factor Mce family protein [Mycobacterium avium str. 104]	63.29	346	346
fig|6666666.28482.peg.3743	R019369	(gi:253797524) mce2B (TBMG 00597) - MCE-family protein mce2B [Mycobacterium tuberculosis str. KZN 1435]	65.76	257	346
fig|6666666.28482.peg.3743	R006417	(gi:15607730) mce2B (Rv0590) - MCE-FAMILY PROTEIN MCE2B [Mycobacterium tuberculosis str. H37Rv]	65.76	257	346
fig|6666666.28482.peg.3743	R006418	(gi:148660359) mce2B (MRA 0597) - MCE-family protein Mce2B [Mycobacterium tuberculosis str. H37Ra]	65.76	257	346
fig|6666666.28482.peg.3743	R013519	(gi:148821792) mce2B (TBFG 10601) - MCE-family protein mce2B [Mycobacterium tuberculosis str. F11]	65.76	257	346
fig|6666666.28482.peg.3743	R006419	(gi:15839992) mce2B (MT0619) - virulence factor mce family protein [Mycobacterium tuberculosis str. CDC1551]	65.37	257	346
fig|6666666.28482.peg.3744	R006370	(gi:118467050) mce1C (MAV 5013) - mce-family protein mce1c [Mycobacterium avium str. 104]	84.58	506	520
fig|6666666.28482.peg.3744	R006369	(gi:41409704) mce1C (MAP3606) - hypothetical protein [Mycobacterium avium str. K-10 (subsp. paratuberculosis)]	84.2	481	520
fig|6666666.28482.peg.3744	R006371	(gi:15828395) mce1C (ML2591) - putative secreted protein [Mycobacterium leprae str. TN]	76.53	506	520
fig|6666666.28482.peg.3744	R019339	(gi:221230872) mce1C (MLBr 02591) - putative secreted protein [Mycobacterium leprae str. Br4923]	76.53	506	520
fig|6666666.28482.peg.3744	R019334	(gi:183980445) mce1C (MMAR 0414) - MCE-family protein Mce1C [Mycobacterium marinum str. M]	77.39	481	520
fig|6666666.28482.peg.3744	R006372	(gi:118616797) mce1C (MUL 1064) - MCE-family protein Mce1C [Mycobacterium ulcerans str. Agy99]	76.97	481	520
fig|6666666.28482.peg.3744	R019338	(gi:224988556) mce1C (JTY 0177) - MCE-family protein [Mycobacterium bovis str. BCG str. Tokyo 172]	76.82	466	520
fig|6666666.28482.peg.3744	R006368	(gi:121636083) mce1C (BCG 0208) - MCE-family protein mce1C [Mycobacterium bovis str. BCG Pasteur 1173P2]	76.82	466	520
fig|6666666.28482.peg.3744	R006367	(gi:31791349) mce1C (Mb0177) - MCE-FAMILY PROTEIN MCE1C [Mycobacterium bovis str. AF2122/97]	76.82	466	520

fig|6666666.28482.peg.3744	R019335	(gi:339630252) mce1C (MAF 01720) - MCE-family protein MCE1C [Mycobacterium africanum str. GM041182]	76.82	466	520
fig|6666666.28482.peg.3744	R019336	(gi:340625208) mce1C (MCAN 01771) - MCE-family protein MCE1C [Mycobacterium canettii str. CIPT 140010059]	76.82	466	520
fig|6666666.28482.peg.3744	R006366	(gi:15839549) mce1C (MT0180) - virulence factor mce family protein [Mycobacterium tuberculosis str. CDC1551]	76.61	466	520
fig|6666666.28482.peg.3744	R006364	(gi:15607312) mce1C (Rv0171) - MCE-FAMILY PROTEIN MCE1C [Mycobacterium tuberculosis str. H37Rv]	76.61	466	520
fig|6666666.28482.peg.3744	R006365	(gi:148659935) mce1C (MRA 0179) - MCE-family protein Mce1C [Mycobacterium tuberculosis str. H37Ra]	76.61	466	520
fig|6666666.28482.peg.3744	R013514	(gi:148821363) mce1C (TBFG 10172) - MCE-family protein mce1C [Mycobacterium tuberculosis str. F11]	76.61	466	520
fig|6666666.28482.peg.3744	R019337	(gi:253797093) mce1C (TBMG 00172) - MCE-family protein mce1C [Mycobacterium tuberculosis str. KZN 1435]	76.57	461	520
fig|6666666.28482.peg.3744	R006373	(gi:118473407) mce1C (MSMEG 0136) - virulence factor Mce family protein, putative [Mycobacterium smegmatis str. MC2 155]	61.23	471	520
fig|6666666.28482.peg.3744	R019340	(gi:333988764) mce1C (JDM601 0124) - MCE-family protein Mce1C [Mycobacterium sp. str. JDM601]	61.95	467	520
fig|6666666.28482.peg.3744	R006438	(gi:126432723) mce2C (Mjls 0110) - virulence factor Mce family protein [Mycobacterium sp. str. JLS]	60.87	479	520
fig|6666666.28482.peg.3744	R006440	(gi:108797100) mce2C (Mmcs 0120) - Mammalian cell entry [Mycobacterium sp. str. MCS]	61.08	479	520
fig|6666666.28482.peg.3744	R006439	(gi:119866185) mce2C (Mkms 0129) - virulence factor Mce family protein [Mycobacterium sp. str. KMS]	61.08	479	520
fig|6666666.28482.peg.3744	R019371	(gi:339630659) mce2C (MAF 05980) - MCE-family protein MCE2C [Mycobacterium africanum str. GM041182]	66.81	470	520
fig|6666666.28482.peg.3744	R019374	(gi:224988981) mce2C (JTY 0606) - MCE-family protein [Mycobacterium bovis str. BCG str. Tokyo 172]	66.81	470	520
fig|6666666.28482.peg.3744	R006433	(gi:121636509) mce2C (BCG 0636) - mce-family protein mce2C [Mycobacterium bovis str. BCG Pasteur 1173P2]	66.81	470	520
fig|6666666.28482.peg.3744	R006432	(gi:31791773) mce2C (Mb0606) - MCE-FAMILY PROTEIN MCE2C [Mycobacterium bovis str. AF2122/97]	66.81	470	520

fig|6666666.28482.peg.3744	R019373	(gi:253797526) mce2C (TBMG 00599) - MCE-family protein mce2C [Mycobacterium tuberculosis str. KZN 1435]	66.6	470	520
fig|6666666.28482.peg.3744	R006429	(gi:15607731) mce2C (Rv0591) - MCE-FAMILY PROTEIN MCE2C [Mycobacterium tuberculosis str. H37Rv]	66.6	470	520
fig|6666666.28482.peg.3744	R006430	(gi:148660361) mce2C (MRA 0599) - MCE-family protein Mce2C [Mycobacterium tuberculosis str. H37Ra]	66.6	470	520
fig|6666666.28482.peg.3744	R013520	(gi:148821794) mce2C (TBFG 10603) - MCE-family protein mce2C [Mycobacterium tuberculosis str. F11]	66.6	470	520
fig|6666666.28482.peg.3744	R006435	(gi:118463703) mce2C (MAV 4549) - mce-family protein mce2c [Mycobacterium avium str. 104]	66.1	467	520
fig|6666666.28482.peg.3744	R006434	(gi:41410184) mce2C (MAP4086) - hypothetical protein [Mycobacterium avium str. K-10 (subsp. paratuberculosis)]	65.88	467	520
fig|6666666.28482.peg.3744	R019372	(gi:340625621) mce2C (MCAN 05971) - MCE-family protein MCE2C [Mycobacterium canettii str. CIPT 140010059]	66.81	470	520
fig|6666666.28482.peg.3744	R006437	(gi:120401164) mce2C (Mvan 0136) - virulence factor Mce family protein [Mycobacterium vanbaalenii str. PYR-1]	61.78	449	520
fig|6666666.28482.peg.3744	R006436	(gi:145221304) mce2C (Mflv 0709) - virulence factor Mce family protein [Mycobacterium gilvum str. PYR-GCK]	60.22	449	520
fig|6666666.28482.peg.3744	R006431	(gi:15839994) mce2C (MT0621) - virulence factor mce family protein [Mycobacterium tuberculosis str. CDC1551]	65.32	346	520
fig|6666666.28482.peg.3819	R005153	(gi:145223891) pcaA (Mflv 3304) - Cyclopropane-fatty-acyl-phospholipid synthase [Mycobacterium gilvum str. PYR-GCK]	68.06	288	291
fig|6666666.28482.peg.3819	R005157	(gi:108799701) pcaA (Mmcs 2735) - Cyclopropane-fatty-acyl-phospholipid synthase [Mycobacterium sp. str. MCS]	68.88	286	291
fig|6666666.28482.peg.3819	R005156	(gi:119868811) pcaA (Mkms 2779) - Cyclopropane-fatty-acyl-phospholipid synthase [Mycobacterium sp. str. KMS]	68.88	286	291
fig|6666666.28482.peg.3819	R005155	(gi:126435345) pcaA (Mjls 2765) - Cyclopropane-fatty-acyl-phospholipid synthase [Mycobacterium sp. str. JLS]	68.88	286	291
fig|6666666.28482.peg.3819	R005167	(gi:108797926) cmaA2 (Mmcs 0952) - Cyclopropane-fatty-acyl-phospholipid synthase [Mycobacterium sp. str. MCS]	68.9	283	291
fig|6666666.28482.peg.3819	R005154	(gi:120404005) pcaA (Mvan 3025) - Cyclopropane-fatty-acyl-phospholipid synthase [Mycobacterium vanbaalenii str. PYR-1]	67.25	287	291

fig|6666666.28482.peg.3819	R005151	(gi:15828331) pcaA (ML2459) - Mycolic acid synthase [Mycobacterium leprae str. TN]	67.61	284	291
fig|6666666.28482.peg.3819	R018712	(gi:221230808) pcaA (MLBr 02459) - Mycolic acid synthase [Mycobacterium leprae str. Br4923]	67.61	284	291
fig|6666666.28482.peg.3819	R005152	(gi:118619634) pcaA (MUL 4539) - mycolic acid synthase PcaA [Mycobacterium ulcerans str. Agy99]	67.49	283	291
fig|6666666.28482.peg.3819	R018707	(gi:183980820) pcaA (MMAR 0796) - mycolic acid synthase PcaA [Mycobacterium marinum str. M]	67.49	283	291
fig|6666666.28482.peg.3819	R018710	(gi:253797397) pcaA (TBMG 00472) - mycolic acid synthase PcaA [Mycobacterium tuberculosis str. KZN 1435]	66.43	283	291
fig|6666666.28482.peg.3819	R005144	(gi:57116736) pcaA (Rv0470c) - MYCOLIC ACID SYNTHASE PCAA (CYCLOPROPANE SYNTHASE) [Mycobacterium tuberculosis str. H37Rv]	66.43	283	291
fig|6666666.28482.peg.3819	R005145	(gi:148660236) pcaA (MRA 0476) - mycolic acid synthase PcaA [Mycobacterium tuberculosis str. H37Ra]	66.43	283	291
fig|6666666.28482.peg.3819	R013397	(gi:148821668) pcaA (TBFG 10477) - mycolic acid synthase pcaA (cyclopropane synthase) [Mycobacterium tuberculosis str. F11]	66.43	283	291
fig|6666666.28482.peg.3819	R005146	(gi:15839858) pcaA (MT0486) - mycolic acid synthase [Mycobacterium tuberculosis str. CDC1551]	66.43	283	291
fig|6666666.28482.peg.3819	R018709	(gi:340625496) pcaA (MCAN 04691) - mycolic acid synthase PCAA [Mycobacterium canettii str. CIPT 140010059]	66.43	283	291
fig|6666666.28482.peg.3819	R005148	(gi:121636385) pcaA (BCG 0510c) - Mycolic acid synthase pcaA [Mycobacterium bovis str. BCG Pasteur 1173P2]	66.43	283	291
fig|6666666.28482.peg.3819	R005147	(gi:31791649) pcaA (Mb0479c) - MYCOLIC ACID SYNTHASE PCAA (CYCLOPROPANE SYNTHASE) [Mycobacterium bovis str. AF2122/97]	66.43	283	291
fig|6666666.28482.peg.3819	R018708	(gi:339630539) pcaA (MAF 04730) - mycolic acid synthase PCAA (cyclopropane synthase) [Mycobacterium africanum str. GM041182]	66.43	283	291
fig|6666666.28482.peg.3819	R005150	(gi:118465677) pcaA (MAV 4679) - cyclopropane-fatty-acyl-phospholipid synthase 1 [Mycobacterium avium str. 104]	65.85	284	291
fig|6666666.28482.peg.3819	R018711	(gi:224988857) pcaA (JTY 0480) - mycolic acid synthase [Mycobacterium bovis str. BCG str. Tokyo 172]	66.43	283	291
fig|6666666.28482.peg.3819	R005149	(gi:41410062) pcaA (MAP3964c) - UmaA2 [Mycobacterium avium str. K-10 (subsp. paratuberculosis)]	65.49	284	291

fig|6666666.28482.peg.3819	R018720	(gi:333990669) cmaA2 (JDM601 2029) - methoxy mycolic acid synthase [Mycobacterium sp. str. JDM601]	60.76	283	291
fig|6666666.28482.peg.3972	R005809	(gi:41408060) glnA1 (MAP1962) - GlnA1 [Mycobacterium avium str. K-10 (subsp. paratuberculosis)]	94.98	478	478
fig|6666666.28482.peg.3972	R005810	(gi:118464473) glnA1 (MAV 2267) - glutamine synthetase, type I [Mycobacterium avium str. 104]	94.98	478	478
fig|6666666.28482.peg.3972	R019011	(gi:183983279) glnA1 (MMAR 3289) - glutamine synthetase GlnA1 [Mycobacterium marinum str. M]	91.84	478	478
fig|6666666.28482.peg.3972	R005812	(gi:118617032) glnA1 (MUL 1340) - glutamine synthetase GlnA1 [Mycobacterium ulcerans str. Agy99]	91	478	478
fig|6666666.28482.peg.3972	R019015	(gi:253798714) glnA1 (TBMG 01760) - glutamine synthetase glnA1 [Mycobacterium tuberculosis str. KZN 1435]	90.59	478	478
fig|6666666.28482.peg.3972	R005804	(gi:15609357) glnA1 (Rv2220) - GLUTAMINE SYNTHETASE GLNA1 (GLUTAMINE SYNTHASE) (GS-I) [Mycobacterium tuberculosis str. H37Rv]	90.59	478	478
fig|6666666.28482.peg.3972	R005805	(gi:148662038) glnA1 (MRA 2237) - glutamine synthetase [Mycobacterium tuberculosis str. H37Ra]	90.59	478	478
fig|6666666.28482.peg.3972	R013457	(gi:148823428) glnA1 (TBFG 12249) - glutamine synthetase glnA1 [Mycobacterium tuberculosis str. F11]	90.59	478	478
fig|6666666.28482.peg.3972	R005806	(gi:15841712) glnA1 (MT2278) - glutamine synthetase [Mycobacterium tuberculosis str. CDC1551]	90.59	478	478
fig|6666666.28482.peg.3972	R019014	(gi:340627226) glnA1 (MCAN 22431) - glutamine synthetase GLNA1 [Mycobacterium canettii str. CIPT 140010059]	90.59	478	478
fig|6666666.28482.peg.3972	R019016	(gi:224990596) glnA1 (JTY 2231) - glutamine synthetase [Mycobacterium bovis str. BCG str. Tokyo 172]	90.59	478	478
fig|6666666.28482.peg.3972	R005808	(gi:121638102) glnA1 (BCG 2237) - Glutamine synthetase glnA1 [Mycobacterium bovis str. BCG Pasteur 1173P2]	90.59	478	478
fig|6666666.28482.peg.3972	R005807	(gi:31793400) glnA1 (Mb2244) - GLUTAMINE SYNTHETASE GLNA1 (GLUTAMINE SYNTHASE) (GS-I) [Mycobacterium bovis str. AF2122/97]	90.59	478	478
fig|6666666.28482.peg.3972	R019013	(gi:339632248) glnA1 (MAF 22320) - Glutamine synthetase GLNA1 (Glutamine synthase) [Mycobacterium africanum str. GM041182]	90.59	478	478
fig|6666666.28482.peg.3972	R005811	(gi:15827444) glnA1 (ML0925) - glutamine synthase class I . [Mycobacterium leprae str. TN]	88.7	478	478

fig|6666666.28482.peg.3972	R019017	(gi:221229921) glnA1 (MLBr 00925) - glutamine synthase class I [Mycobacterium leprae str. Br4923]	88.7	478	478
fig|6666666.28482.peg.3972	R005815	(gi:120404556) glnA1 (Mvan 3585) - glutamine synthetase, type I [Mycobacterium vanbaalenii str. PYR-1]	85.15	478	478
fig|6666666.28482.peg.3972	R005814	(gi:145223515) glnA1 (Mflv 2928) - glutamine synthetase, type I [Mycobacterium gilvum str. PYR-GCK]	84.1	478	478
fig|6666666.28482.peg.3972	R005813	(gi:118467956) glnA1 (MSMEG 4290) - glutamine synthetase, type I [Mycobacterium smegmatis str. MC2 155]	84.1	478	478
fig|6666666.28482.peg.3972	R005818	(gi:108800286) glnA1 (Mmcs 3320) - glutamine synthetase, type I [Mycobacterium sp. str. MCS]	83.89	478	478
fig|6666666.28482.peg.3972	R005817	(gi:119869414) glnA1 (Mkms 3382) - glutamine synthetase, type I [Mycobacterium sp. str. KMS]	83.89	478	478
fig|6666666.28482.peg.3972	R005816	(gi:126435909) glnA1 (Mjls 3331) - glutamine synthetase, type I [Mycobacterium sp. str. JLS]	83.89	478	478
fig|6666666.28482.peg.3972	R019012	(gi:169629021) glnA1 (MAB 1933c) - glutamine synthetase, type I (GlnA1) [Mycobacterium abscessus str. ATCC 19977]	82.01	478	478
fig|6666666.28482.peg.3972	R019018	(gi:333992818) glnA1 (JDM601 4178) - glutamine synthetase [Mycobacterium sp. str. JDM601]	81.74	471	478
fig|6666666.28482.peg.3972	R019019	(gi:333990322) glnA1 (JDM601 1682) - glutamine synthetase [Mycobacterium sp. str. JDM601]	79.5	478	478
fig|6666666.28482.peg.4172	R019185	(gi:169631326) sodC (MAB 4248c) - hypothetical protein [Mycobacterium abscessus str. ATCC 19977]	60.8	199	212
fig|6666666.28482.peg.4230	R019275	(gi:169628052) prrB (MAB 0955c) - sensor histidine kinase PrrB [Mycobacterium abscessus str. ATCC 19977]	70.25	436	443
fig|6666666.28482.peg.4230	R019274	(gi:183984601) prrB (MMAR 4634) - two-component sensor histidine kinase PrrB [Mycobacterium marinum str. M]	61.01	432	443
fig|6666666.28482.peg.4230	R019281	(gi:333989462) prrB (JDM601 0822) - two-component sensor histidine kinase [Mycobacterium sp. str. JDM601]	60.37	430	443
fig|6666666.28482.peg.4230	R019278	(gi:253800074) prrB (TBMG 03087) - two component system sensor histidine kinase prrB [Mycobacterium tuberculosis str. KZN 1435]	60.78	432	443
fig|6666666.28482.peg.4230	R006246	(gi:15608042) prrB (Rv0902c) - TWO COMPONENT SENSOR HISTIDINE KINASE PRRB [Mycobacterium tuberculosis str. H37Rv]	60.78	432	443

fig|6666666.28482.peg.4230	R006247	(gi:148660680) prrB (MRA 0909) - two component sensor histidine kinase PrrB [Mycobacterium tuberculosis str. H37Ra]	60.78	432	443
fig|6666666.28482.peg.4230	R013506	(gi:148822111) prrB (TBFG 10920) - two component system sensor histidine kinase prrB [Mycobacterium tuberculosis str. F11]	60.78	432	443
fig|6666666.28482.peg.4230	R019277	(gi:340625913) prrB (MCAN 09021) - two component sensor histidine kinase PRRB [Mycobacterium canettii str. CIPT 140010059]	60.78	432	443
fig|6666666.28482.peg.4230	R019279	(gi:224989296) prrB (JTY 0924) - two component sensor histidine kinase [Mycobacterium bovis str. BCG str. Tokyo 172]	60.78	432	443
fig|6666666.28482.peg.4230	R006250	(gi:121636825) prrB (BCG 0954c) - Two component sensor histidine kinase prrB [Mycobacterium bovis str. BCG Pasteur 1173P2]	60.78	432	443
fig|6666666.28482.peg.4230	R006249	(gi:31792090) prrB (Mb0926c) - TWO COMPONENT SENSOR HISTIDINE KINASE PRRB [Mycobacterium bovis str. AF2122/97]	60.78	432	443
fig|6666666.28482.peg.4230	R019276	(gi:339630967) prrB (MAF 09110) - two component sensor histidine kinase PRRB [Mycobacterium africanum str. GM041182]	60.78	432	443
fig|6666666.28482.peg.4230	R006248	(gi:15840321) prrB (MT0925) - sensor histidine kinase [Mycobacterium tuberculosis str. CDC1551]	60.78	432	443
fig|6666666.28482.peg.4230	R006254	(gi:118616127) prrB (MUL 0249) - two component sensor histidine kinase PrrB [Mycobacterium ulcerans str. Agy99]	60.78	432	443
fig|6666666.28482.peg.4230	R006253	(gi:15828140) prrB (ML2124) - sensor histidine kinase [Mycobacterium leprae str. TN]	60.32	432	443
fig|6666666.28482.peg.4230	R019280	(gi:221230617) prrB (MLBr 02124) - sensor histidine kinase [Mycobacterium leprae str. Br4923]	60.32	432	443
fig|6666666.28482.peg.4230	R006252	(gi:118464817) prrB (MAV 1021) - sensor-type histidine kinase PrrB [Mycobacterium avium str. 104]	60.83	408	443
fig|6666666.28482.peg.4230	R006255	(gi:118472850) prrB (MSMEG 5663) - sensor-type histidine kinase PrrB [Mycobacterium smegmatis str. MC2 155]	61.71	394	443
fig|6666666.28482.peg.4258	R005155	(gi:126435345) pcaA (Mjls 2765) - Cyclopropane-fatty-acyl-phospholipid synthase [Mycobacterium sp. str. JLS]	84.01	294	294
fig|6666666.28482.peg.4258	R005157	(gi:108799701) pcaA (Mmcs 2735) - Cyclopropane-fatty-acyl-phospholipid synthase [Mycobacterium sp. str. MCS]	84.01	294	294
fig|6666666.28482.peg.4258	R005156	(gi:119868811) pcaA (Mkms 2779) - Cyclopropane-fatty-acyl-phospholipid synthase [Mycobacterium sp. str. KMS]	84.01	294	294

fig|6666666.28482.peg.4258	R005154	(gi:120404005) pcaA (Mvan 3025) - Cyclopropane-fatty-acyl-phospholipid synthase [Mycobacterium vanbaalenii str. PYR-1]	76.11	293	294
fig|6666666.28482.peg.4258	R005153	(gi:145223891) pcaA (Mflv 3304) - Cyclopropane-fatty-acyl-phospholipid synthase [Mycobacterium gilvum str. PYR-GCK]	75.43	293	294
fig|6666666.28482.peg.4258	R005152	(gi:118619634) pcaA (MUL 4539) - mycolic acid synthase PcaA [Mycobacterium ulcerans str. Agy99]	70.98	286	294
fig|6666666.28482.peg.4258	R018707	(gi:183980820) pcaA (MMAR 0796) - mycolic acid synthase PcaA [Mycobacterium marinum str. M]	70.98	286	294
fig|6666666.28482.peg.4258	R005151	(gi:15828331) pcaA (ML2459) - Mycolic acid synthase [Mycobacterium leprae str. TN]	69.58	286	294
fig|6666666.28482.peg.4258	R018712	(gi:221230808) pcaA (MLBr 02459) - Mycolic acid synthase [Mycobacterium leprae str. Br4923]	69.58	286	294
fig|6666666.28482.peg.4258	R005167	(gi:108797926) cmaA2 (Mmcs 0952) - Cyclopropane-fatty-acyl-phospholipid synthase [Mycobacterium sp. str. MCS]	70.67	283	294
fig|6666666.28482.peg.4258	R018710	(gi:253797397) pcaA (TBMG 00472) - mycolic acid synthase PcaA [Mycobacterium tuberculosis str. KZN 1435]	68.88	286	294
fig|6666666.28482.peg.4258	R005144	(gi:57116736) pcaA (Rv0470c) - MYCOLIC ACID SYNTHASE PCAA (CYCLOPROPANE SYNTHASE) [Mycobacterium tuberculosis str. H37Rv]	68.88	286	294
fig|6666666.28482.peg.4258	R005145	(gi:148660236) pcaA (MRA 0476) - mycolic acid synthase PcaA [Mycobacterium tuberculosis str. H37Ra]	68.88	286	294
fig|6666666.28482.peg.4258	R013397	(gi:148821668) pcaA (TBFG 10477) - mycolic acid synthase pcaA (cyclopropane synthase) [Mycobacterium tuberculosis str. F11]	68.88	286	294
fig|6666666.28482.peg.4258	R005146	(gi:15839858) pcaA (MT0486) - mycolic acid synthase [Mycobacterium tuberculosis str. CDC1551]	68.88	286	294
fig|6666666.28482.peg.4258	R018709	(gi:340625496) pcaA (MCAN 04691) - mycolic acid synthase PCAA [Mycobacterium canettii str. CIPT 140010059]	68.88	286	294
fig|6666666.28482.peg.4258	R005148	(gi:121636385) pcaA (BCG 0510c) - Mycolic acid synthase pcaA [Mycobacterium bovis str. BCG Pasteur 1173P2]	68.88	286	294
fig|6666666.28482.peg.4258	R005147	(gi:31791649) pcaA (Mb0479c) - MYCOLIC ACID SYNTHASE PCAA (CYCLOPROPANE SYNTHASE) [Mycobacterium bovis str. AF2122/97]	68.88	286	294
fig|6666666.28482.peg.4258	R018708	(gi:339630539) pcaA (MAF 04730) - mycolic acid synthase PCAA (cyclopropane synthase) [Mycobacterium africanum str. GM041182]	68.88	286	294

fig|6666666.28482.peg.4258	R018711	(gi:224988857) pcaA (JTY 0480) - mycolic acid synthase [Mycobacterium bovis str. BCG str. Tokyo 172]	68.88	286	294
fig|6666666.28482.peg.4258	R005149	(gi:41410062) pcaA (MAP3964c) - UmaA2 [Mycobacterium avium str. K-10 (subsp. paratuberculosis)]	67.48	286	294
fig|6666666.28482.peg.4258	R005150	(gi:118465677) pcaA (MAV 4679) - cyclopropane-fatty-acyl-phospholipid synthase 1 [Mycobacterium avium str. 104]	68.2	283	294
fig|6666666.28482.peg.4258	R018713	(gi:333989092) pcaA (JDM601 0452) - mycolic acid synthase PcaA [Mycobacterium sp. str. JDM601]	64.39	277	294
fig|6666666.28482.peg.4258	R018720	(gi:333990669) cmaA2 (JDM601 2029) - methoxy mycolic acid synthase [Mycobacterium sp. str. JDM601]	63.19	283	294
fig|6666666.28482.peg.4258	R005165	(gi:15828307) cmaA2 (ML2426) - cyclopropane mycolic acid synthase [Mycobacterium leprae str. TN]	60.75	283	294
fig|6666666.28482.peg.4258	R018719	(gi:221230784) cmaA2 (MLBr 02426) - cyclopropane mycolic acid synthase [Mycobacterium leprae str. Br4923]	60.75	283	294
fig|6666666.28482.peg.4409	R019862	(gi:183982677) PE18 (MMAR 2670) - PE family protein, PE19 1 [Mycobacterium marinum str. M]	90.8	87	99
fig|6666666.28482.peg.4409	R018659	(gi:118618471) PE18 (MUL 3088) - PE family protein [Mycobacterium ulcerans str. Agy99]	89.66	87	99
fig|6666666.28482.peg.4409	R018680	(gi:41407605) PE19 (MAP1507) - hypothetical protein [Mycobacterium avium str. K-10 (subsp. paratuberculosis)]	88.51	87	99
fig|6666666.28482.peg.4409	R019865	(gi:253799172) PE18 (TBMG 02209) - PE family protein [Mycobacterium tuberculosis str. KZN 1435]	91.95	87	99
fig|6666666.28482.peg.4409	R018654	(gi:57116910) PE18 (Rv1788) - PE family protein [Mycobacterium tuberculosis str. H37Rv]	91.95	87	99
fig|6666666.28482.peg.4409	R018655	(gi:148661595) PE18 (MRA 1802) - PE family protein [Mycobacterium tuberculosis str. H37Ra]	91.95	87	99
fig|6666666.28482.peg.4409	R018653	(gi:148823003) PE18 (TBFG 11818) - PE family protein [Mycobacterium tuberculosis str. F11]	91.95	87	99
fig|6666666.28482.peg.4409	R018656	(gi:15841256) PE18 (MT1837) - PE family protein [Mycobacterium tuberculosis str. CDC1551]	91.95	87	99
fig|6666666.28482.peg.4409	R019864	(gi:340626800) PE18 (MCAN 18061) - PE family protein [Mycobacterium canettii str. CIPT 140010059]	91.95	87	99

fig|6666666.28482.peg.4409	R019866	(gi:224990173) PE18 (JTY 1804) - PE family protein [Mycobacterium bovis str. BCG str. Tokyo 172]	91.95	87	99
fig|6666666.28482.peg.4409	R018658	(gi:121637689) PE18 (BCG 1820) - PE family protein [Mycobacterium bovis str. BCG Pasteur 1173P2]	91.95	87	99
fig|6666666.28482.peg.4409	R018657	(gi:31792976) PE18 (Mb1816) - PE family protein [Mycobacterium bovis str. AF2122/97]	91.95	87	99
fig|6666666.28482.peg.4409	R019863	(gi:339631842) PE18 (MAF 18100) - PE family protein [Mycobacterium africanum str. GM041182]	91.95	87	99
fig|6666666.28482.peg.4409	R019877	(gi:183982680) PE19 (MMAR 2673) - PE family protein, PE19 [Mycobacterium marinum str. M]	89.66	87	99
fig|6666666.28482.peg.4409	R018675	(gi:57116913) PE19 (Rv1791) - PE family protein [Mycobacterium tuberculosis str. H37Rv]	87.36	87	99
fig|6666666.28482.peg.4409	R018676	(gi:148661598) PE19 (MRA 1805) - PE family protein [Mycobacterium tuberculosis str. H37Ra]	87.36	87	99
fig|6666666.28482.peg.4409	R019879	(gi:340626803) PE19 (MCAN 18091) - PE family protein [Mycobacterium canettii str. CIPT 140010059]	87.36	87	99
fig|6666666.28482.peg.4409	R019881	(gi:224990176) PE19 (JTY 1807) - PE family protein [Mycobacterium bovis str. BCG str. Tokyo 172]	87.36	87	99
fig|6666666.28482.peg.4409	R018679	(gi:121637692) PE19 (BCG 1823) - PE family protein [Mycobacterium bovis str. BCG Pasteur 1173P2]	87.36	87	99
fig|6666666.28482.peg.4409	R018678	(gi:31792979) PE19 (Mb1819) - PE family protein [Mycobacterium bovis str. AF2122/97]	87.36	87	99
fig|6666666.28482.peg.4409	R019878	(gi:339631845) PE19 (MAF 18130) - PE family protein [Mycobacterium africanum str. GM041182]	87.36	87	99
fig|6666666.28482.peg.4409	R019880	(gi:253799168) PE19 (TBMG 02205) - PE family protein [Mycobacterium tuberculosis str. KZN 1435]	87.36	87	99
fig|6666666.28482.peg.4409	R018674	(gi:148823006) PE19 (TBFG 11821) - PE family protein [Mycobacterium tuberculosis str. F11]	87.36	87	99
fig|6666666.28482.peg.4409	R018677	(gi:15841261) PE19 (MT1840) - PE family protein [Mycobacterium tuberculosis str. CDC1551]	87.36	87	99
fig|6666666.28482.peg.4409	R019867	(gi:333990534) PE18 (JDM601 1894) - PE-PGRS family protein [Mycobacterium sp. str. JDM601]	87.36	87	99

fig|6666666.28482.peg.4410	R014041	(gi:118464008) esxM (MAV 2922) - hypothetical protein [Mycobacterium avium str. 104]	97.96	98	98
fig|6666666.28482.peg.4410	R019810	(gi:340626804) esxM (MCAN 18101) - ESAT-6 like protein ESXM [Mycobacterium canettii str. CIPT 140010059]	87.76	98	98
fig|6666666.28482.peg.4410	R019812	(gi:224990177) esxM (JTY 1808) - hypothetical protein [Mycobacterium bovis str. BCG str. Tokyo 172]	87.76	98	98
fig|6666666.28482.peg.4410	R014040	(gi:121637693) esxM (BCG 1824) - hypothetical protein [Mycobacterium bovis str. BCG Pasteur 1173P2]	87.76	98	98
fig|6666666.28482.peg.4410	R014039	(gi:31792980) esxM (Mb1820) - hypothetical protein [Mycobacterium bovis str. AF2122/97]	87.76	98	98
fig|6666666.28482.peg.4410	R019809	(gi:339631846) esxM (MAF 18140) - ESAT-6 like protein ESXM [Mycobacterium africanum str. GM041182]	87.76	98	98
fig|6666666.28482.peg.4410	R019811	(gi:253799760) esxM (TBMG 02785) - esat-6 like protein esxK [Mycobacterium tuberculosis str. KZN 1435]	86.73	98	98
fig|6666666.28482.peg.4410	R019808	(gi:183982681) esxM (MMAR 2674) - EsaT-6 like protein EsxM [Mycobacterium marinum str. M]	86.46	96	98
fig|6666666.28482.peg.4410	R014042	(gi:118618466) esxM (MUL 3083) - EsaT-6 like protein EsxM [Mycobacterium ulcerans str. Agy99]	84.69	96	98
fig|6666666.28482.peg.4411	R014035	(gi:118463289) esxN (MAV 2921) - hypothetical protein [Mycobacterium avium str. 104]	95.74	94	94
fig|6666666.28482.peg.4411	R019816	(gi:253799165) esxN (TBMG 02203) - esat-6 like protein esxN [Mycobacterium tuberculosis str. KZN 1435]	94.68	94	94
fig|6666666.28482.peg.4411	R014030	(gi:57116914) esxN (Rv1793) - PUTATIVE ESAT-6 LIKE PROTEIN ESXN (ESAT-6 LIKE PROTEIN 5) [Mycobacterium tuberculosis str. H37Rv]	94.68	94	94
fig|6666666.28482.peg.4411	R014031	(gi:148661599) esxN (MRA 1806) - putative esat-6 like protein EsxN [Mycobacterium tuberculosis str. H37Ra]	94.68	94	94
fig|6666666.28482.peg.4411	R014029	(gi:148823007) esxN (TBFG 11824) - Esat-6 like protein esxN (Esat-6 like protein 5) [Mycobacterium tuberculosis str. F11]	94.68	94	94
fig|6666666.28482.peg.4411	R014032	(gi:15841262) esxN (MT1842) - hypothetical protein [Mycobacterium tuberculosis str. CDC1551]	94.68	94	94
fig|6666666.28482.peg.4411	R019815	(gi:340626805) esxN (MCAN 18111) - putative ESAT-6 like protein ESXN [Mycobacterium canettii str. CIPT 140010059]	94.68	94	94

fig|6666666.28482.peg.4411	R019817	(gi:224990178) esxN (JTY 1809) - putative EsaT-6 like protein 5 [Mycobacterium bovis str. BCG str. Tokyo 172]	94.68	94	94
fig|6666666.28482.peg.4411	R014034	(gi:121637694) esxN (BCG 1825) - putative ESAT-6 like protein 5 [Mycobacterium bovis str. BCG Pasteur 1173P2]	94.68	94	94
fig|6666666.28482.peg.4411	R014033	(gi:31792981) esxN (Mb1821) - PUTATIVE ESAT-6 LIKE PROTEIN 5 [Mycobacterium bovis str. AF2122/97]	94.68	94	94
fig|6666666.28482.peg.4411	R019814	(gi:339631847) esxN (MAF 18150) - putative ESAT-6 like protein ESXN (ESAT-6 like protein 5) [Mycobacterium africanum str. GM041182]	94.68	94	94
fig|6666666.28482.peg.4411	R014036	(gi:118618465) esxN (MUL 3082) - EsaT-6 like protein EsxN [Mycobacterium ulcerans str. Agy99]	93.62	94	94
fig|6666666.28482.peg.4411	R019813	(gi:183982682) esxN (MMAR 2675) - EsaT-6 like protein EsxN [Mycobacterium marinum str. M]	93.62	94	94
fig|6666666.28482.peg.4411	R019818	(gi:333990536) esxN (JDM601 1896) - EsaT-6 like protein EsxN [Mycobacterium sp. str. JDM601]	87.23	94	94
fig|6666666.28482.peg.4423	R019446	(gi:183984950) mce4F (MMAR 4982) - MCE-family protein Mce4F [Mycobacterium marinum str. M]	81.85	573	573
fig|6666666.28482.peg.4423	R006690	(gi:118619247) mce4F (MUL 4057) - MCE-family protein Mce4F [Mycobacterium ulcerans str. Agy99]	81.15	573	573
fig|6666666.28482.peg.4423	R006689	(gi:41406667) mce4F (MAP0569) - hypothetical protein [Mycobacterium avium str. K-10 (subsp. paratuberculosis)]	91.52	506	573
fig|6666666.28482.peg.4423	R019450	(gi:253800536) mce4F (TBMG 03539) - MCE-family protein mce4F [Mycobacterium tuberculosis str. KZN 1435]	86.22	450	573
fig|6666666.28482.peg.4423	R006684	(gi:15610630) mce4F (Rv3494c) - MCE-FAMILY PROTEIN MCE4F [Mycobacterium tuberculosis str. H37Rv]	86.22	450	573
fig|6666666.28482.peg.4423	R006685	(gi:148663358) mce4F (MRA 3534) - MCE-family protein Mce4F [Mycobacterium tuberculosis str. H37Ra]	86.22	450	573
fig|6666666.28482.peg.4423	R013535	(gi:148824701) mce4F (TBFG 13528) - MCE-family protein mce4F [Mycobacterium tuberculosis str. F11]	86.22	450	573
fig|6666666.28482.peg.4423	R006686	(gi:15843106) mce4F (MT3598) - virulence factor mce family protein [Mycobacterium tuberculosis str. CDC1551]	86.22	450	573
fig|6666666.28482.peg.4423	R019449	(gi:340628462) mce4F (MCAN 35081) - MCE-family protein MCE4F [Mycobacterium canettii str. CIPT 140010059]	86.22	450	573

fig|6666666.28482.peg.4423	R019448	(gi:339633497) mce4F (MAF 35070) - MCE-family protein MCE4F [Mycobacterium africanum str. GM041182]	86.22	450	573
fig|6666666.28482.peg.4423	R019451	(gi:224991909) mce4F (JTY 3558) - MCE-family protein [Mycobacterium bovis str. BCG str. Tokyo 172]	86.22	450	573
fig|6666666.28482.peg.4423	R006688	(gi:121639413) mce4F (BCG 3558c) - Mce-family protein mce4F [Mycobacterium bovis str. BCG Pasteur 1173P2]	86.22	450	573
fig|6666666.28482.peg.4423	R006687	(gi:31794670) mce4F (Mb3524c) - MCE-FAMILY PROTEIN MCE4F [Mycobacterium bovis str. AF2122/97]	86.22	450	573
fig|6666666.28482.peg.4423	R006691	(gi:118467558) mce4F (MSMEG 5895) - virulence factor mce family protein [Mycobacterium smegmatis str. MC2 155]	71	438	573
fig|6666666.28482.peg.4423	R006692	(gi:145222153) mce4F (Mflv 1561) - virulence factor Mce family protein [Mycobacterium gilvum str. PYR-GCK]	68.26	438	573
fig|6666666.28482.peg.4423	R006693	(gi:120406145) mce4F (Mvan 5197) - virulence factor Mce family protein [Mycobacterium vanbaalenii str. PYR-1]	66.67	438	573
fig|6666666.28482.peg.4423	R006694	(gi:126437559) mce4F (Mjls 4995) - virulence factor Mce family protein [Mycobacterium sp. str. JLS]	66.21	438	573
fig|6666666.28482.peg.4423	R006696	(gi:108801575) mce4F (Mmcs 4612) - Mammalian cell entry [Mycobacterium sp. str. MCS]	65.53	438	573
fig|6666666.28482.peg.4423	R006695	(gi:119870729) mce4F (Mkms 4700) - virulence factor Mce family protein [Mycobacterium sp. str. KMS]	65.53	438	573
fig|6666666.28482.peg.4423	R019452	(gi:333992265) mce4F (JDM601 3625) - MCE-family protein Mce4F [Mycobacterium sp. str. JDM601]	65.38	438	573
fig|6666666.28482.peg.4423	R019447	(gi:169631226) mce4F (MAB 4148c) - MCE-family protein [Mycobacterium abscessus str. ATCC 19977]	65.97	429	573
fig|6666666.28482.peg.4424	R006675	(gi:41406666) mce4E (MAP0568) - LprN [Mycobacterium avium str. K-10 (subsp. paratuberculosis)]	87.5	384	388
fig|6666666.28482.peg.4424	R006676	(gi:118464340) mce4E (MAV 0661) - virulence factor Mce family protein [Mycobacterium avium str. 104]	87.66	381	388
fig|6666666.28482.peg.4424	R019439	(gi:183984951) mce4E (MMAR 4983) - MCE family lipoprotein LprN [Mycobacterium marinum str. M]	81.72	383	388
fig|6666666.28482.peg.4424	R006677	(gi:118619248) mce4E (MUL 4058) - MCE-family lipoprotein LprN [Mycobacterium ulcerans str. Agy99]	81.72	383	388

fig|6666666.28482.peg.4424	R019443	(gi:253800537) mce4E (TBMG 03540) - MCE-family lipoprotein mce4E [Mycobacterium tuberculosis str. KZN 1435]	81.25	384	388
fig|6666666.28482.peg.4424	R006670	(gi:15610631) mce4E (Rv3495c) - POSSIBLE MCE-FAMILY LIPOPROTEIN LPRN (MCE-FAMILY LIPOPROTEIN MCE4E) [Mycobacterium tuberculosis str. H37Rv]	81.25	384	388
fig|6666666.28482.peg.4424	R006671	(gi:148663359) mce4E (MRA 3535) - MCE-family lipoprotein LprN [Mycobacterium tuberculosis str. H37Ra]	81.25	384	388
fig|6666666.28482.peg.4424	R013534	(gi:148824702) mce4E (TBFG 13529) - MCE-family lipoprotein lprN (MCE-family lipoprotein mce4e) [Mycobacterium tuberculosis str. F11]	81.25	384	388
fig|6666666.28482.peg.4424	R006672	(gi:15843107) mce4E (MT3599) - virulence factor mce family protein [Mycobacterium tuberculosis str. CDC1551]	81.25	384	388
fig|6666666.28482.peg.4424	R019444	(gi:224991910) mce4E (JTY 3559) - putative MCE-family lipoprotein [Mycobacterium bovis str. BCG str. Tokyo 172]	81.25	384	388
fig|6666666.28482.peg.4424	R006674	(gi:121639414) mce4E (BCG 3559c) - putative mce-family lipoprotein lprN [Mycobacterium bovis str. BCG Pasteur 1173P2]	81.25	384	388
fig|6666666.28482.peg.4424	R006673	(gi:31794671) mce4E (Mb3525c) - POSSIBLE MCE-FAMILY LIPOPROTEIN LPRN (MCE-FAMILY LIPOPROTEIN MCE4E) [Mycobacterium bovis str. AF2122/97]	81.25	384	388
fig|6666666.28482.peg.4424	R019441	(gi:339633498) mce4E (MAF 35080) - putative MCE-family lipoprotein LPRN (MCE-family lipoprotein MCE4E) [Mycobacterium africanum str. GM041182]	81.25	384	388
fig|6666666.28482.peg.4424	R019442	(gi:340628463) mce4E (MCAN 35091) - putative MCE-family lipoprotein LPRN [Mycobacterium canettii str. CIPT 140010059]	80.99	384	388
fig|6666666.28482.peg.4424	R006683	(gi:108801576) mce4E (Mmcs 4613) - Mammalian cell entry [Mycobacterium sp. str. MCS]	69.95	386	388
fig|6666666.28482.peg.4424	R006682	(gi:119870730) mce4E (Mkms 4701) - virulence factor Mce family protein [Mycobacterium sp. str. KMS]	69.95	386	388
fig|6666666.28482.peg.4424	R006681	(gi:126437560) mce4E (Mjls 4996) - virulence factor Mce family protein [Mycobacterium sp. str. JLS]	69.43	386	388
fig|6666666.28482.peg.4424	R006680	(gi:120406146) mce4E (Mvan 5198) - virulence factor Mce family protein [Mycobacterium vanbaalenii str. PYR-1]	68.73	371	388
fig|6666666.28482.peg.4424	R006678	(gi:118470887) mce4E (MSMEG 5896) - virulence factor Mce family protein [Mycobacterium smegmatis str. MC2 155]	65.97	382	388

fig|6666666.28482.peg.4424	R006679	(gi:145222152) mce4E (Mflv 1560) - virulence factor Mce family protein [Mycobacterium gilvum str. PYR-GCK]	67.12	371	388
fig|6666666.28482.peg.4424	R019445	(gi:333992266) mce4E (JDM601 3626) - MCE family lipoprotein LprN [Mycobacterium sp. str. JDM601]	65.09	381	388
fig|6666666.28482.peg.4425	R006662	(gi:118462453) mce4D (MAV 0660) - virulence factor mce family protein [Mycobacterium avium str. 104]	86.97	422	465
fig|6666666.28482.peg.4425	R006661	(gi:41406665) mce4D (MAP0567) - hypothetical protein [Mycobacterium avium str. K-10 (subsp. paratuberculosis)]	86.73	422	465
fig|6666666.28482.peg.4425	R019435	(gi:340628464) mce4D (MCAN 35101) - MCE-family protein MCE4D [Mycobacterium canettii str. CIPT 140010059]	81.77	417	465
fig|6666666.28482.peg.4425	R019436	(gi:253800538) mce4D (TBMG 03541) - MCE-family protein mce4D [Mycobacterium tuberculosis str. KZN 1435]	81.77	417	465
fig|6666666.28482.peg.4425	R006656	(gi:15610632) mce4D (Rv3496c) - MCE-FAMILY PROTEIN MCE4D [Mycobacterium tuberculosis str. H37Rv]	81.77	417	465
fig|6666666.28482.peg.4425	R006657	(gi:148663360) mce4D (MRA 3536) - MCE-family protein Mce4D [Mycobacterium tuberculosis str. H37Ra]	81.77	417	465
fig|6666666.28482.peg.4425	R013533	(gi:148824703) mce4D (TBFG 13530) - MCE-family protein mce4D [Mycobacterium tuberculosis str. F11]	81.77	417	465
fig|6666666.28482.peg.4425	R019437	(gi:224991911) mce4D (JTY 3560) - MCE-family protein [Mycobacterium bovis str. BCG str. Tokyo 172]	81.77	417	465
fig|6666666.28482.peg.4425	R006660	(gi:121639415) mce4D (BCG 3560c) - Mce-family protein mce4D [Mycobacterium bovis str. BCG Pasteur 1173P2]	81.77	417	465
fig|6666666.28482.peg.4425	R006659	(gi:31794672) mce4D (Mb3526c) - MCE-FAMILY PROTEIN MCE4D [Mycobacterium bovis str. AF2122/97]	81.77	417	465
fig|6666666.28482.peg.4425	R019434	(gi:339633499) mce4D (MAF 35090) - MCE-family protein MCE4D [Mycobacterium africanum str. GM041182]	81.77	417	465
fig|6666666.28482.peg.4425	R006658	(gi:15843108) mce4D (MT3600) - virulence factor mce family protein [Mycobacterium tuberculosis str. CDC1551]	81.77	417	465
fig|6666666.28482.peg.4425	R019432	(gi:183984952) mce4D (MMAR 4984) - MCE-family protein Mce4D [Mycobacterium marinum str. M]	80.43	418	465
fig|6666666.28482.peg.4425	R006663	(gi:118619249) mce4D (MUL 4059) - MCE-family protein Mce4D [Mycobacterium ulcerans str. Agy99]	80.19	418	465

fig|6666666.28482.peg.4425	R006665	(gi:145222151) mce4D (Mflv 1559) - virulence factor Mce family protein [Mycobacterium gilvum str. PYR-GCK]	63.64	418	465
fig|6666666.28482.peg.4425	R006664	(gi:118470654) mce4D (MSMEG 5897) - virulence factor mce family protein [Mycobacterium smegmatis str. MC2 155]	66.02	415	465
fig|6666666.28482.peg.4425	R006666	(gi:120406147) mce4D (Mvan 5199) - virulence factor Mce family protein [Mycobacterium vanbaalenii str. PYR-1]	63.16	418	465
fig|6666666.28482.peg.4425	R006667	(gi:126437561) mce4D (Mjls 4997) - virulence factor Mce family protein [Mycobacterium sp. str. JLS]	63.07	417	465
fig|6666666.28482.peg.4425	R006669	(gi:108801577) mce4D (Mmcs 4614) - Mammalian cell entry [Mycobacterium sp. str. MCS]	62.11	417	465
fig|6666666.28482.peg.4425	R006668	(gi:119870731) mce4D (Mkms 4702) - virulence factor Mce family protein [Mycobacterium sp. str. KMS]	62.11	417	465
fig|6666666.28482.peg.4425	R019438	(gi:333992267) mce4D (JDM601 3627) - MCE-family protein Mce4D [Mycobacterium sp. str. JDM601]	61.84	411	465
fig|6666666.28482.peg.4426	R006647	(gi:41406664) mce4C (MAP0566) - hypothetical protein [Mycobacterium avium str. K-10 (subsp. paratuberculosis)]	92.37	354	354
fig|6666666.28482.peg.4426	R006648	(gi:118464170) mce4C (MAV 0659) - virulence factor Mce family protein [Mycobacterium avium str. 104]	91.81	354	354
fig|6666666.28482.peg.4426	R019429	(gi:253800539) mce4C (TBMG 03542) - MCE-family protein mce4C [Mycobacterium tuberculosis str. KZN 1435]	82.34	351	354
fig|6666666.28482.peg.4426	R006642	(gi:15610633) mce4C (Rv3497c) - MCE-FAMILY PROTEIN MCE4C [Mycobacterium tuberculosis str. H37Rv]	82.34	351	354
fig|6666666.28482.peg.4426	R006643	(gi:148663361) mce4C (MRA 3537) - MCE-family protein Mce4C [Mycobacterium tuberculosis str. H37Ra]	82.34	351	354
fig|6666666.28482.peg.4426	R013532	(gi:148824704) mce4C (TBFG 13531) - MCE-family protein mce4C [Mycobacterium tuberculosis str. F11]	82.34	351	354
fig|6666666.28482.peg.4426	R006644	(gi:15843109) mce4C (MT3601) - virulence factor mce family protein [Mycobacterium tuberculosis str. CDC1551]	82.34	351	354
fig|6666666.28482.peg.4426	R019428	(gi:340628465) mce4C (MCAN 35111) - MCE-family protein MCE4C [Mycobacterium canettii str. CIPT 140010059]	82.34	351	354
fig|6666666.28482.peg.4426	R006645	(gi:31794673) mce4C (Mb3527c) - MCE-FAMILY PROTEIN MCE4C [Mycobacterium bovis str. AF2122/97]	82.34	351	354

fig|6666666.28482.peg.4426	R019427	(gi:339633500) mce4C (MAF 35100) - MCE-family protein MCE4C [Mycobacterium africanum str. GM041182]	82.34	351	354
fig|6666666.28482.peg.4426	R019430	(gi:224991912) mce4C (JTY 3561) - MCE-family protein [Mycobacterium bovis str. BCG str. Tokyo 172]	82.34	351	354
fig|6666666.28482.peg.4426	R006646	(gi:121639416) mce4C (BCG 3561c) - Mce-family protein mce4C [Mycobacterium bovis str. BCG Pasteur 1173P2]	82.34	351	354
fig|6666666.28482.peg.4426	R019425	(gi:183984953) mce4C (MMAR 4985) - MCE-family protein Mce4C [Mycobacterium marinum str. M]	81.77	351	354
fig|6666666.28482.peg.4426	R006649	(gi:118619250) mce4C (MUL 4060) - MCE-family protein Mce4C [Mycobacterium ulcerans str. Agy99]	80.86	350	354
fig|6666666.28482.peg.4426	R006655	(gi:108801578) mce4C (Mmcs 4615) - Mammalian cell entry [Mycobacterium sp. str. MCS]	69.57	345	354
fig|6666666.28482.peg.4426	R006654	(gi:119870732) mce4C (Mkms 4703) - virulence factor Mce family protein [Mycobacterium sp. str. KMS]	69.57	345	354
fig|6666666.28482.peg.4426	R006652	(gi:120406148) mce4C (Mvan 5200) - virulence factor Mce family protein [Mycobacterium vanbaalenii str. PYR-1]	66.95	351	354
fig|6666666.28482.peg.4426	R006651	(gi:145222150) mce4C (Mflv 1558) - virulence factor Mce family protein [Mycobacterium gilvum str. PYR-GCK]	67.72	347	354
fig|6666666.28482.peg.4426	R006653	(gi:126437562) mce4C (Mjls 4998) - virulence factor Mce family protein [Mycobacterium sp. str. JLS]	68.12	345	354
fig|6666666.28482.peg.4426	R006650	(gi:118471840) mce4C (MSMEG 5898) - virulence factor Mce family protein [Mycobacterium smegmatis str. MC2 155]	66.38	348	354
fig|6666666.28482.peg.4426	R019426	(gi:169631229) mce4C (MAB 4151c) - MCE-family protein [Mycobacterium abscessus str. ATCC 19977]	65.23	348	354
fig|6666666.28482.peg.4426	R019431	(gi:333992268) mce4C (JDM601 3628) - MCE-family protein Mce4C [Mycobacterium sp. str. JDM601]	65.75	327	354
fig|6666666.28482.peg.4427	R006634	(gi:118463448) mce4B (MAV 0658) - virulence factor Mce family protein [Mycobacterium avium str. 104]	91.43	350	350
fig|6666666.28482.peg.4427	R006633	(gi:41406663) mce4B (MAP0565) - hypothetical protein [Mycobacterium avium str. K-10 (subsp. paratuberculosis)]	91.14	350	350
fig|6666666.28482.peg.4427	R019422	(gi:253800540) mce4B (TBMG 03543) - MCE-family protein mce4B [Mycobacterium tuberculosis str. KZN 1435]	82	350	350

fig|6666666.28482.peg.4427	R006628	(gi:15610634) mce4B (Rv3498c) - MCE-FAMILY PROTEIN MCE4B [Mycobacterium tuberculosis str. H37Rv]	82	350	350
fig|6666666.28482.peg.4427	R006629	(gi:148663362) mce4B (MRA 3538) - MCE-family protein Mce4B [Mycobacterium tuberculosis str. H37Ra]	82	350	350
fig|6666666.28482.peg.4427	R013531	(gi:148824705) mce4B (TBFG 13532) - MCE-family protein mce4B [Mycobacterium tuberculosis str. F11]	82	350	350
fig|6666666.28482.peg.4427	R006630	(gi:15843110) mce4B (MT3602) - virulence factor mce family protein [Mycobacterium tuberculosis str. CDC1551]	82	350	350
fig|6666666.28482.peg.4427	R019421	(gi:340628466) mce4B (MCAN 35121) - MCE-family protein MCE4B [Mycobacterium canettii str. CIPT 140010059]	82	350	350
fig|6666666.28482.peg.4427	R019423	(gi:224991913) mce4B (JTY 3562) - MCE-family protein [Mycobacterium bovis str. BCG str. Tokyo 172]	82	350	350
fig|6666666.28482.peg.4427	R006632	(gi:121639417) mce4B (BCG 3562c) - Mce-family protein mce4B [Mycobacterium bovis str. BCG Pasteur 1173P2]	82	350	350
fig|6666666.28482.peg.4427	R006631	(gi:31794674) mce4B (Mb3528c) - MCE-FAMILY PROTEIN MCE4B [Mycobacterium bovis str. AF2122/97]	82	350	350
fig|6666666.28482.peg.4427	R019420	(gi:339633501) mce4B (MAF 35110) - MCE-family protein MCE4B [Mycobacterium africanum str. GM041182]	82	350	350
fig|6666666.28482.peg.4427	R019418	(gi:183984954) mce4B (MMAR 4986) - MCE-family protein Mce4B [Mycobacterium marinum str. M]	83.43	350	350
fig|6666666.28482.peg.4427	R006635	(gi:118619251) mce4B (MUL 4061) - MCE-family protein Mce4B [Mycobacterium ulcerans str. Agy99]	82.86	350	350
fig|6666666.28482.peg.4427	R006641	(gi:108801579) mce4B (Mmcs 4616) - Mammalian cell entry [Mycobacterium sp. str. MCS]	72.54	335	350
fig|6666666.28482.peg.4427	R006640	(gi:119870733) mce4B (Mkms 4704) - virulence factor Mce family protein [Mycobacterium sp. str. KMS]	72.54	335	350
fig|6666666.28482.peg.4427	R019424	(gi:333992269) mce4B (JDM601 3629) - MCE-family protein Mce4B [Mycobacterium sp. str. JDM601]	67.16	341	350
fig|6666666.28482.peg.4427	R006636	(gi:118473663) mce4B (MSMEG 5899) - virulence factor Mce family protein [Mycobacterium smegmatis str. MC2 155]	69.88	342	350
fig|6666666.28482.peg.4427	R006637	(gi:145222149) mce4B (Mflv 1557) - virulence factor Mce family protein [Mycobacterium gilvum str. PYR-GCK]	68.42	342	350

fig|6666666.28482.peg.4427	R006638	(gi:120406149) mce4B (Mvan 5201) - virulence factor Mce family protein [Mycobacterium vanbaalenii str. PYR-1]	69.53	338	350
fig|6666666.28482.peg.4427	R006639	(gi:126437563) mce4B (Mjls 4999) - virulence factor Mce family protein [Mycobacterium sp. str. JLS]	72.54	335	350
fig|6666666.28482.peg.4427	R019419	(gi:169631230) mce4B (MAB 4152c) - MCE-family protein [Mycobacterium abscessus str. ATCC 19977]	66.67	339	350
fig|6666666.28482.peg.4428	R006620	(gi:118464721) mce4A (MAV 0657) - virulence factor Mce family protein [Mycobacterium avium str. 104]	85.94	384	400
fig|6666666.28482.peg.4428	R006619	(gi:41406662) mce4A (MAP0564) - Mce4 [Mycobacterium avium str. K-10 (subsp. paratuberculosis)]	86.54	379	400
fig|6666666.28482.peg.4428	R019415	(gi:253800541) mce4A (TBMG 03544) - MCE-family protein mce4A [Mycobacterium tuberculosis str. KZN 1435]	83	400	400
fig|6666666.28482.peg.4428	R006614	(gi:57117113) mce4A (Rv3499c) - MCE-FAMILY PROTEIN MCE4A [Mycobacterium tuberculosis str. H37Rv]	83	400	400
fig|6666666.28482.peg.4428	R006615	(gi:148663363) mce4A (MRA 3539) - MCE-family protein Mce4A [Mycobacterium tuberculosis str. H37Ra]	83	400	400
fig|6666666.28482.peg.4428	R013530	(gi:148824706) mce4A (TBFG 13533) - MCE-family protein mce4A [Mycobacterium tuberculosis str. F11]	83	400	400
fig|6666666.28482.peg.4428	R006616	(gi:15843111) mce4A (MT3603) - virulence factor mce family protein [Mycobacterium tuberculosis str. CDC1551]	83	400	400
fig|6666666.28482.peg.4428	R019414	(gi:340628467) mce4A (MCAN 35131) - MCE-family protein MCE4A [Mycobacterium canettii str. CIPT 140010059]	83	400	400
fig|6666666.28482.peg.4428	R019416	(gi:224991914) mce4A (JTY 3563) - MCE-family protein [Mycobacterium bovis str. BCG str. Tokyo 172]	83	400	400
fig|6666666.28482.peg.4428	R006618	(gi:121639418) mce4A (BCG 3563c) - Mce-family protein mce4A [Mycobacterium bovis str. BCG Pasteur 1173P2]	83	400	400
fig|6666666.28482.peg.4428	R006617	(gi:31794675) mce4A (Mb3529c) - MCE-FAMILY PROTEIN MCE4A [Mycobacterium bovis str. AF2122/97]	83	400	400
fig|6666666.28482.peg.4428	R019413	(gi:339633502) mce4A (MAF 35120) - MCE-family protein MCE4A [Mycobacterium africanum str. GM041182]	83	400	400
fig|6666666.28482.peg.4428	R019411	(gi:183984955) mce4A (MMAR 4987) - MCE-family protein Mce4A [Mycobacterium marinum str. M]	84.25	400	400

fig|6666666.28482.peg.4428	R006621	(gi:118619252) mce4A (MUL 4062) - MCE-family protein Mce4A [Mycobacterium ulcerans str. Agy99]	83.75	400	400
fig|6666666.28482.peg.4428	R006622	(gi:118470324) mce4A (MSMEG 5900) - virulence factor Mce family protein [Mycobacterium smegmatis str. MC2 155]	64.75	400	400
fig|6666666.28482.peg.4428	R006625	(gi:126437564) mce4A (Mjls 5000) - virulence factor Mce family protein [Mycobacterium sp. str. JLS]	65.5	400	400
fig|6666666.28482.peg.4428	R006627	(gi:108801580) mce4A (Mmcs 4617) - Mammalian cell entry [Mycobacterium sp. str. MCS]	65.25	400	400
fig|6666666.28482.peg.4428	R006626	(gi:119870734) mce4A (Mkms 4705) - virulence factor Mce family protein [Mycobacterium sp. str. KMS]	66.22	373	400
fig|6666666.28482.peg.4428	R019417	(gi:333992270) mce4A (JDM601 3630) - MCE-family protein Mce4A [Mycobacterium sp. str. JDM601]	60.51	390	400
fig|6666666.28482.peg.4428	R006623	(gi:145222148) mce4A (Mflv 1556) - virulence factor Mce family protein [Mycobacterium gilvum str. PYR-GCK]	64	400	400
fig|6666666.28482.peg.4428	R006624	(gi:120406150) mce4A (Mvan 5202) - virulence factor Mce family protein [Mycobacterium vanbaalenii str. PYR-1]	64.25	400	400
fig|6666666.28482.peg.4557	R005720	(gi:118466952) panD (MAV 0552) - aspartate 1-decarboxylase [Mycobacterium avium str. 104]	93.66	138	139
fig|6666666.28482.peg.4557	R005725	(gi:120406311) panD (Mvan 5363) - aspartate 1-decarboxylase [Mycobacterium vanbaalenii str. PYR-1]	91.11	135	139
fig|6666666.28482.peg.4557	R018971	(gi:333992530) panD (JDM601 3890) - aspartate 1-decarboxylase precursor PanD [Mycobacterium sp. str. JDM601]	90.58	138	139
fig|6666666.28482.peg.4557	R018964	(gi:183985072) panD (MMAR 5104) - aspartate alpha-decarboxylase [Mycobacterium marinum str. M]	90.85	138	139
fig|6666666.28482.peg.4557	R018968	(gi:253800641) panD (TBMG 03639) - aspartate alpha-decarboxylase [Mycobacterium tuberculosis str. KZN 1435]	90.44	136	139
fig|6666666.28482.peg.4557	R005714	(gi:15610737) panD (Rv3601c) - aspartate 1-decarboxylase precursor [Mycobacterium tuberculosis str. H37Rv]	90.44	136	139
fig|6666666.28482.peg.4557	R005715	(gi:148663465) panD (MRA 3640) - aspartate 1-decarboxylase precursor [Mycobacterium tuberculosis str. H37Ra]	90.44	136	139
fig|6666666.28482.peg.4557	R013451	(gi:148824806) panD (TBFG 13633) - aspartate 1-decarboxylase precursor panD [Mycobacterium tuberculosis str. F11]	90.44	136	139

fig|6666666.28482.peg.4557	R005716	(gi:15843213) panD (MT3706.1) - aspartate 1-decarboxylase precursor [Mycobacterium tuberculosis str. CDC1551]	90.44	136	139
fig|6666666.28482.peg.4557	R018969	(gi:224992017) panD (JTY 3666) - aspartate alpha-decarboxylase [Mycobacterium bovis str. BCG str. Tokyo 172]	90.44	136	139
fig|6666666.28482.peg.4557	R005718	(gi:121639520) panD (BCG 3665c) - putative aspartate 1-decarboxylase precursor panD [Mycobacterium bovis str. BCG Pasteur 1173P2]	90.44	136	139
fig|6666666.28482.peg.4557	R005717	(gi:31794777) panD (Mb3631c) - aspartate 1-decarboxylase precursor [Mycobacterium bovis str. AF2122/97]	90.44	136	139
fig|6666666.28482.peg.4557	R018966	(gi:339633600) panD (MAF 36140) - putative aspartate 1-decarboxylase precursor PAND (aspartate alpha-decarboxylase) [Mycobacterium africanum str. GM041182]	90.44	136	139
fig|6666666.28482.peg.4557	R018967	(gi:340628566) panD (MCAN 36131) - putative aspartate 1-decarboxylase PAND [Mycobacterium canettii str. CIPT 140010059]	90.44	136	139
fig|6666666.28482.peg.4557	R005722	(gi:118619357) panD (MUL 4183) - aspartate 1-decarboxylase precursor PanD [Mycobacterium ulcerans str. Agy99]	90.58	134	139
fig|6666666.28482.peg.4557	R005721	(gi:15827028) panD (ML0231) - aspartate 1-decarboxylase precursor [Mycobacterium leprae str. TN]	87.94	138	139
fig|6666666.28482.peg.4557	R018970	(gi:221229506) panD (MLBr 00231) - aspartate alpha-decarboxylase [Mycobacterium leprae str. Br4923]	87.94	138	139
fig|6666666.28482.peg.4557	R005728	(gi:108801724) panD (Mmcs 4761) - aspartate 1-decarboxylase [Mycobacterium sp. str. MCS]	86.67	135	139
fig|6666666.28482.peg.4557	R005727	(gi:119870875) panD (Mkms 4847) - aspartate 1-decarboxylase [Mycobacterium sp. str. KMS]	86.67	135	139
fig|6666666.28482.peg.4557	R005726	(gi:126437711) panD (Mjls 5147) - aspartate 1-decarboxylase [Mycobacterium sp. str. JLS]	86.67	135	139
fig|6666666.28482.peg.4557	R018965	(gi:169627646) panD (MAB 0542) - aspartate 1-decarboxylase precursor [Mycobacterium abscessus str. ATCC 19977]	78.99	135	139
fig|6666666.28482.peg.4557	R005723	(gi:118473676) panD (MSMEG 0021) - aspartate 1-decarboxylase [Mycobacterium smegmatis str. MC2 155]	63.91	132	139
fig|6666666.28482.peg.4558	R018956	(gi:183985073) panC (MMAR 5105) - pantoate--beta-alanine ligase [Mycobacterium marinum str. M]	77.42	307	309
fig|6666666.28482.peg.4558	R005707	(gi:118619358) panC (MUL 4184) - pantoate--beta-alanine ligase PanC [Mycobacterium ulcerans str. Agy99]	77.42	307	309

fig|6666666.28482.peg.4558	R005704	(gi:41406554) panC (MAP0456) - pantoate--beta-alanine ligase [Mycobacterium avium str. K-10 (subsp. paratuberculosis)]	79.29	309	309
fig|6666666.28482.peg.4558	R005705	(gi:118462570) panC (MAV 0551) - pantoate--beta-alanine ligase [Mycobacterium avium str. 104]	78.96	309	309
fig|6666666.28482.peg.4558	R018960	(gi:253800642) panC (TBMG 03640) - pantoate--beta-alanine ligase [Mycobacterium tuberculosis str. KZN 1435]	81.76	307	309
fig|6666666.28482.peg.4558	R005699	(gi:15610738) panC (Rv3602c) - pantoate--beta-alanine ligase [Mycobacterium tuberculosis str. H37Rv]	81.76	307	309
fig|6666666.28482.peg.4558	R005700	(gi:148663466) panC (MRA 3641) - pantoate--beta-alanine ligase [Mycobacterium tuberculosis str. H37Ra]	81.76	307	309
fig|6666666.28482.peg.4558	R013450	(gi:148824807) panC (TBFG 13634) - pantoate-beta-alanine ligase panC [Mycobacterium tuberculosis str. F11]	81.76	307	309
fig|6666666.28482.peg.4558	R005701	(gi:15843214) panC (MT3707) - pantoate--beta-alanine ligase [Mycobacterium tuberculosis str. CDC1551]	81.76	307	309
fig|6666666.28482.peg.4558	R018959	(gi:340628567) panC (MCAN 36141) - putative pantoate--beta-alanine ligase [Mycobacterium canettii str. CIPT 140010059]	81.43	307	309
fig|6666666.28482.peg.4558	R018961	(gi:224992018) panC (JTY 3667) - pantoate--beta-alanine ligase [Mycobacterium bovis str. BCG str. Tokyo 172]	81.76	307	309
fig|6666666.28482.peg.4558	R005703	(gi:121639521) panC (BCG 3666c) - putative pantoate--beta-alanine ligase panC [Mycobacterium bovis str. BCG Pasteur 1173P2]	81.76	307	309
fig|6666666.28482.peg.4558	R005702	(gi:31794778) panC (Mb3632c) - pantoate--beta-alanine ligase [Mycobacterium bovis str. AF2122/97]	81.76	307	309
fig|6666666.28482.peg.4558	R018958	(gi:339633601) panC (MAF 36150) - putative pantoate--beta-alanine ligase PANC (pantothenate synthetase) [Mycobacterium africanum str. GM041182]	81.76	307	309
fig|6666666.28482.peg.4558	R005709	(gi:145222016) panC (Mflv 1424) - pantoate--beta-alanine ligase [Mycobacterium gilvum str. PYR-GCK]	68.51	307	309
fig|6666666.28482.peg.4558	R005713	(gi:108801725) panC (Mmcs 4762) - pantoate--beta-alanine ligase [Mycobacterium sp. str. MCS]	71.48	305	309
fig|6666666.28482.peg.4558	R005712	(gi:119870876) panC (Mkms 4848) - pantoate--beta-alanine ligase [Mycobacterium sp. str. KMS]	71.48	305	309
fig|6666666.28482.peg.4558	R005711	(gi:126437712) panC (Mjls 5148) - pantoate--beta-alanine ligase [Mycobacterium sp. str. JLS]	71.15	305	309

fig|6666666.28482.peg.4558	R018957	(gi:169627645) panC (MAB 0541) - pantoate--beta-alanine ligase [Mycobacterium abscessus str. ATCC 19977]	69.87	302	309
fig|6666666.28482.peg.4558	R018963	(gi:333992531) panC (JDM601 3891) - pantoate-beta-alanine ligase PanC [Mycobacterium sp. str. JDM601]	75.44	285	309
fig|6666666.28482.peg.4558	R005706	(gi:15827027) panC (ML0230) - pantoate--beta-alanine ligase [Mycobacterium leprae str. TN]	76.72	305	309
fig|6666666.28482.peg.4558	R018962	(gi:221229505) panC (MLBr 00230) - pantoate--beta-alanine ligase [Mycobacterium leprae str. Br4923]	76.72	305	309
fig|6666666.28482.peg.4558	R005710	(gi:120406312) panC (Mvan 5364) - pantoate--beta-alanine ligase [Mycobacterium vanbaalenii str. PYR-1]	68.71	308	309
fig|6666666.28482.peg.4558	R005708	(gi:118467890) panC (MSMEG 6097) - pantoate--beta-alanine ligase [Mycobacterium smegmatis str. MC2 155]	68.61	308	309
fig|6666666.28482.peg.4571	R019816	(gi:253799165) esxN (TBMG 02203) - esat-6 like protein esxN [Mycobacterium tuberculosis str. KZN 1435]	91.49	94	94
fig|6666666.28482.peg.4571	R014030	(gi:57116914) esxN (Rv1793) - PUTATIVE ESAT-6 LIKE PROTEIN ESXN (ESAT-6 LIKE PROTEIN 5) [Mycobacterium tuberculosis str. H37Rv]	91.49	94	94
fig|6666666.28482.peg.4571	R014031	(gi:148661599) esxN (MRA 1806) - putative esat-6 like protein EsxN [Mycobacterium tuberculosis str. H37Ra]	91.49	94	94
fig|6666666.28482.peg.4571	R014029	(gi:148823007) esxN (TBFG 11824) - Esat-6 like protein esxN (Esat-6 like protein 5) [Mycobacterium tuberculosis str. F11]	91.49	94	94
fig|6666666.28482.peg.4571	R014032	(gi:15841262) esxN (MT1842) - hypothetical protein [Mycobacterium tuberculosis str. CDC1551]	91.49	94	94
fig|6666666.28482.peg.4571	R019815	(gi:340626805) esxN (MCAN 18111) - putative ESAT-6 like protein ESXN [Mycobacterium canettii str. CIPT 140010059]	91.49	94	94
fig|6666666.28482.peg.4571	R019817	(gi:224990178) esxN (JTY 1809) - putative EsaT-6 like protein 5 [Mycobacterium bovis str. BCG str. Tokyo 172]	91.49	94	94
fig|6666666.28482.peg.4571	R014034	(gi:121637694) esxN (BCG 1825) - putative ESAT-6 like protein 5 [Mycobacterium bovis str. BCG Pasteur 1173P2]	91.49	94	94
fig|6666666.28482.peg.4571	R014033	(gi:31792981) esxN (Mb1821) - PUTATIVE ESAT-6 LIKE PROTEIN 5 [Mycobacterium bovis str. AF2122/97]	91.49	94	94
fig|6666666.28482.peg.4571	R019814	(gi:339631847) esxN (MAF 18150) - putative ESAT-6 like protein ESXN (ESAT-6 like protein 5) [Mycobacterium africanum str. GM041182]	91.49	94	94

fig|6666666.28482.peg.4571	R014035	(gi:118463289) esxN (MAV 2921) - hypothetical protein [Mycobacterium avium str. 104]	90.43	94	94
fig|6666666.28482.peg.4571	R014036	(gi:118618465) esxN (MUL 3082) - EsaT-6 like protein EsxN [Mycobacterium ulcerans str. Agy99]	90.43	94	94
fig|6666666.28482.peg.4571	R019813	(gi:183982682) esxN (MMAR 2675) - EsaT-6 like protein EsxN [Mycobacterium marinum str. M]	90.43	94	94
fig|6666666.28482.peg.4571	R019818	(gi:333990536) esxN (JDM601 1896) - EsaT-6 like protein EsxN [Mycobacterium sp. str. JDM601]	85.11	94	94
fig|6666666.28482.peg.4572	R019810	(gi:340626804) esxM (MCAN 18101) - ESAT-6 like protein ESXM [Mycobacterium canettii str. CIPT 140010059]	91.84	98	98
fig|6666666.28482.peg.4572	R019812	(gi:224990177) esxM (JTY 1808) - hypothetical protein [Mycobacterium bovis str. BCG str. Tokyo 172]	91.84	98	98
fig|6666666.28482.peg.4572	R014040	(gi:121637693) esxM (BCG 1824) - hypothetical protein [Mycobacterium bovis str. BCG Pasteur 1173P2]	91.84	98	98
fig|6666666.28482.peg.4572	R014039	(gi:31792980) esxM (Mb1820) - hypothetical protein [Mycobacterium bovis str. AF2122/97]	91.84	98	98
fig|6666666.28482.peg.4572	R019809	(gi:339631846) esxM (MAF 18140) - ESAT-6 like protein ESXM [Mycobacterium africanum str. GM041182]	91.84	98	98
fig|6666666.28482.peg.4572	R019811	(gi:253799760) esxM (TBMG 02785) - esat-6 like protein esxK [Mycobacterium tuberculosis str. KZN 1435]	90.82	98	98
fig|6666666.28482.peg.4572	R014041	(gi:118464008) esxM (MAV 2922) - hypothetical protein [Mycobacterium avium str. 104]	87.76	98	98
fig|6666666.28482.peg.4572	R019808	(gi:183982681) esxM (MMAR 2674) - EsaT-6 like protein EsxM [Mycobacterium marinum str. M]	90.62	96	98
fig|6666666.28482.peg.4572	R014042	(gi:118618466) esxM (MUL 3083) - EsaT-6 like protein EsxM [Mycobacterium ulcerans str. Agy99]	88.78	96	98
fig|6666666.28482.peg.4589	R005167	(gi:108797926) cmaA2 (Mmcs 0952) - Cyclopropane-fatty-acyl-phospholipid synthase [Mycobacterium sp. str. MCS]	70.88	285	288
fig|6666666.28482.peg.4589	R005152	(gi:118619634) pcaA (MUL 4539) - mycolic acid synthase PcaA [Mycobacterium ulcerans str. Agy99]	67.71	288	288
fig|6666666.28482.peg.4589	R018707	(gi:183980820) pcaA (MMAR 0796) - mycolic acid synthase PcaA [Mycobacterium marinum str. M]	67.71	288	288

fig|6666666.28482.peg.4589	R005150	(gi:118465677) pcaA (MAV 4679) - cyclopropane-fatty-acyl-phospholipid synthase 1 [Mycobacterium avium str. 104]	67.02	285	288
fig|6666666.28482.peg.4589	R005149	(gi:41410062) pcaA (MAP3964c) - UmaA2 [Mycobacterium avium str. K-10 (subsp. paratuberculosis)]	66.32	288	288
fig|6666666.28482.peg.4589	R005151	(gi:15828331) pcaA (ML2459) - Mycolic acid synthase [Mycobacterium leprae str. TN]	65.28	288	288
fig|6666666.28482.peg.4589	R018712	(gi:221230808) pcaA (MLBr 02459) - Mycolic acid synthase [Mycobacterium leprae str. Br4923]	65.28	288	288
fig|6666666.28482.peg.4589	R018710	(gi:253797397) pcaA (TBMG 00472) - mycolic acid synthase PcaA [Mycobacterium tuberculosis str. KZN 1435]	64.58	288	288
fig|6666666.28482.peg.4589	R005144	(gi:57116736) pcaA (Rv0470c) - MYCOLIC ACID SYNTHASE PCAA (CYCLOPROPANE SYNTHASE) [Mycobacterium tuberculosis str. H37Rv]	64.58	288	288
fig|6666666.28482.peg.4589	R005145	(gi:148660236) pcaA (MRA 0476) - mycolic acid synthase PcaA [Mycobacterium tuberculosis str. H37Ra]	64.58	288	288
fig|6666666.28482.peg.4589	R013397	(gi:148821668) pcaA (TBFG 10477) - mycolic acid synthase pcaA (cyclopropane synthase) [Mycobacterium tuberculosis str. F11]	64.58	288	288
fig|6666666.28482.peg.4589	R005146	(gi:15839858) pcaA (MT0486) - mycolic acid synthase [Mycobacterium tuberculosis str. CDC1551]	64.58	288	288
fig|6666666.28482.peg.4589	R018709	(gi:340625496) pcaA (MCAN 04691) - mycolic acid synthase PCAA [Mycobacterium canettii str. CIPT 140010059]	64.58	288	288
fig|6666666.28482.peg.4589	R005148	(gi:121636385) pcaA (BCG 0510c) - Mycolic acid synthase pcaA [Mycobacterium bovis str. BCG Pasteur 1173P2]	64.58	288	288
fig|6666666.28482.peg.4589	R005147	(gi:31791649) pcaA (Mb0479c) - MYCOLIC ACID SYNTHASE PCAA (CYCLOPROPANE SYNTHASE) [Mycobacterium bovis str. AF2122/97]	64.58	288	288
fig|6666666.28482.peg.4589	R018708	(gi:339630539) pcaA (MAF 04730) - mycolic acid synthase PCAA (cyclopropane synthase) [Mycobacterium africanum str. GM041182]	64.58	288	288
fig|6666666.28482.peg.4589	R018711	(gi:224988857) pcaA (JTY 0480) - mycolic acid synthase [Mycobacterium bovis str. BCG str. Tokyo 172]	64.24	288	288
fig|6666666.28482.peg.4589	R005154	(gi:120404005) pcaA (Mvan 3025) - Cyclopropane-fatty-acyl-phospholipid synthase [Mycobacterium vanbaalenii str. PYR-1]	63.76	287	288
fig|6666666.28482.peg.4589	R005153	(gi:145223891) pcaA (Mflv 3304) - Cyclopropane-fatty-acyl-phospholipid synthase [Mycobacterium gilvum str. PYR-GCK]	63.07	287	288

fig|6666666.28482.peg.4589	R005155	(gi:126435345) pcaA (Mjls 2765) - Cyclopropane-fatty-acyl-phospholipid synthase [Mycobacterium sp. str. JLS]	65.16	287	288
fig|6666666.28482.peg.4589	R005157	(gi:108799701) pcaA (Mmcs 2735) - Cyclopropane-fatty-acyl-phospholipid synthase [Mycobacterium sp. str. MCS]	65.16	287	288
fig|6666666.28482.peg.4589	R005156	(gi:119868811) pcaA (Mkms 2779) - Cyclopropane-fatty-acyl-phospholipid synthase [Mycobacterium sp. str. KMS]	65.16	287	288
fig|6666666.28482.peg.4639	R019036	(gi:169631178) mbtH (MAB 4100c) - MbtH-like protein [Mycobacterium abscessus str. ATCC 19977]	86.84	76	76
fig|6666666.28482.peg.4639	R019035	(gi:183983167) mbtH (MMAR 3173) - protein MbtH [Mycobacterium marinum str. M]	85.51	69	76
fig|6666666.28482.peg.4639	R019039	(gi:253798546) mbtH (TBMG 01598) - hypothetical protein [Mycobacterium tuberculosis str. KZN 1435]	79.71	69	76
fig|6666666.28482.peg.4639	R013460	(gi:148823579) mbtH (TBFG 12402) - hypotehtical mycobactin/exochelin synthesis protein mbtH [Mycobacterium tuberculosis str. F11]	79.71	69	76
fig|6666666.28482.peg.4639	R005850	(gi:15841889) mbtH (MT2445.1) - mbtH protein [Mycobacterium tuberculosis str. CDC1551]	79.71	69	76
fig|6666666.28482.peg.4639	R019038	(gi:340627388) mbtH (MCAN 24091) - hypothetical protein [Mycobacterium canettii str. CIPT 140010059]	79.71	69	76
fig|6666666.28482.peg.4639	R019040	(gi:224990750) mbtH (JTY 2385) - hypothetical protein [Mycobacterium bovis str. BCG str. Tokyo 172]	79.71	69	76
fig|6666666.28482.peg.4639	R005852	(gi:121638256) mbtH (BCG 2391c) - putative protein mbtH [Mycobacterium bovis str. BCG Pasteur 1173P2]	79.71	69	76
fig|6666666.28482.peg.4639	R005851	(gi:31793554) mbtH (Mb2398c) - PUTATIVE CONSERVED PROTEIN MBTH [Mycobacterium bovis str. AF2122/97]	79.71	69	76
fig|6666666.28482.peg.4639	R019037	(gi:339632403) mbtH (MAF 23910) - putative conserved protein MBTH [Mycobacterium africanum str. GM041182]	79.71	69	76
fig|6666666.28482.peg.4639	R005848	(gi:15609514) mbtH (Rv2377c) - PUTATIVE CONSERVED PROTEIN MBTH [Mycobacterium tuberculosis str. H37Rv]	78.26	69	76
fig|6666666.28482.peg.4639	R005849	(gi:148662209) mbtH (MRA 2400) - protein MbtH [Mycobacterium tuberculosis str. H37Ra]	78.26	69	76
fig|6666666.28482.peg.4639	R005859	(gi:126436052) mbtH (Mjls 3474) - MbtH domain protein [Mycobacterium sp. str. JLS]	77.14	70	76

fig|6666666.28482.peg.4639	R005861	(gi:108800429) mbtH (Mmcs 3463) - MbtH-like protein [Mycobacterium sp. str. MCS]	75.71	70	76
fig|6666666.28482.peg.4639	R005860	(gi:119869558) mbtH (Mkms 3526) - MbtH domain protein [Mycobacterium sp. str. KMS]	75.71	70	76
fig|6666666.28482.peg.4639	R005857	(gi:145223286) mbtH (Mflv 2699) - MbtH-like protein [Mycobacterium gilvum str. PYR-GCK]	73.24	71	76
fig|6666666.28482.peg.4639	R005856	(gi:118470732) mbtH (MSMEG 4508) - hypothetical protein [Mycobacterium smegmatis str. MC2 155]	78.79	66	76
fig|6666666.28482.peg.4639	R019041	(gi:333990185) mbtH (JDM601 1545) - MbtH-like protein [Mycobacterium sp. str. JDM601]	72.37	73	76
fig|6666666.28482.peg.4639	R005855	(gi:118618911) mbtH (MUL 3639) - conserved hypothetical protein MbtH 1 [Mycobacterium ulcerans str. Agy99]	69.01	71	76
fig|6666666.28482.peg.4639	R005853	(gi:41408267) mbtH (MAP2169c) - MbtH 3 [Mycobacterium avium str. K-10 (subsp. paratuberculosis)]	70	70	76
fig|6666666.28482.peg.4639	R005854	(gi:118464383) mbtH (MAV 2016) - hypothetical protein [Mycobacterium avium str. 104]	70	70	76
fig|6666666.28482.peg.4639	R005858	(gi:120404800) mbtH (Mvan 3842) - MbtH domain protein [Mycobacterium vanbaalenii str. PYR-1]	68.49	69	76
fig|6666666.28482.peg.4639	R018198	(gi:118469728) MSMEG0016 - hypothetical protein [Mycobacterium smegmatis str. MC2 155]	62.32	66	76
fig|6666666.28482.peg.4729	R019176	(gi:183982761) ahpC (MMAR 2755) - alkyl hydroperoxide reductase C protein AhpC [Mycobacterium marinum str. M]	92.31	195	195
fig|6666666.28482.peg.4729	R019180	(gi:253798492) ahpC (TBMG 01544) - alkyl hydroperoxide reductase C protein ahpC [Mycobacterium tuberculosis str. KZN 1435]	92.31	195	195
fig|6666666.28482.peg.4729	R006078	(gi:15609565) ahpC (Rv2428) - ALKYL HYDROPEROXIDE REDUCTASE C PROTEIN AHPC (ALKYL HYDROPEROXIDASE C) [Mycobacterium tuberculosis str. H37Rv]	92.31	195	195
fig|6666666.28482.peg.4729	R006079	(gi:148662263) ahpC (MRA 2455) - AhpC [Mycobacterium tuberculosis str. H37Ra]	92.31	195	195
fig|6666666.28482.peg.4729	R013479	(gi:148823631) ahpC (TBFG 12456) - alkyl hydroperoxide reductase C protein ahpC [Mycobacterium tuberculosis str. F11]	92.31	195	195
fig|6666666.28482.peg.4729	R006080	(gi:15841949) ahpC (MT2503) - alkyl hydroperoxide reductase C [Mycobacterium tuberculosis str. CDC1551]	92.31	195	195

fig|6666666.28482.peg.4729	R019179	(gi:340627441) ahpC (MCAN 24661) - alkyl hydroperoxide reductase C protein [Mycobacterium canettii str. CIPT 140010059]	92.31	195	195
fig|6666666.28482.peg.4729	R019181	(gi:224990804) ahpC (JTY 2441) - alkyl hydroperoxide reductase C protein [Mycobacterium bovis str. BCG str. Tokyo 172]	92.31	195	195
fig|6666666.28482.peg.4729	R006082	(gi:121638310) ahpC (BCG 2447) - Alkyl hydroperoxide reductase C protein ahpC [Mycobacterium bovis str. BCG Pasteur 1173P2]	92.31	195	195
fig|6666666.28482.peg.4729	R006081	(gi:31793608) ahpC (Mb2454) - ALKYL HYDROPEROXIDE REDUCTASE C PROTEIN AHPC (ALKYL HYDROPEROXIDASE C) [Mycobacterium bovis str. AF2122/97]	92.31	195	195
fig|6666666.28482.peg.4729	R019178	(gi:339632455) ahpC (MAF 24440) - alkyl hydroperoxide reductase C protein AHPC (alkyl hydroperoxidase C) [Mycobacterium africanum str. GM041182]	92.31	195	195
fig|6666666.28482.peg.4729	R006084	(gi:118467085) ahpC (MAV 2839) - alkylhydroperoxide reductase [Mycobacterium avium str. 104]	91.79	195	195
fig|6666666.28482.peg.4729	R006083	(gi:41407687) ahpC (MAP1589c) - AhpC [Mycobacterium avium str. K-10 (subsp. paratuberculosis)]	91.28	195	195
fig|6666666.28482.peg.4729	R006086	(gi:118618391) ahpC (MUL 2999) - alkyl hydroperoxide reductase C protein AhpC [Mycobacterium ulcerans str. Agy99]	91.28	195	195
fig|6666666.28482.peg.4729	R006085	(gi:15828102) ahpC (ML2042) - alkyl hydroperoxide reductase [Mycobacterium leprae str. TN]	85.13	195	195
fig|6666666.28482.peg.4729	R019182	(gi:221230579) ahpC (MLBr 02042) - alkyl hydroperoxide reductase [Mycobacterium leprae str. Br4923]	85.13	195	195
fig|6666666.28482.peg.4729	R006087	(gi:118471486) ahpC (MSMEG 4891) - alkylhydroperoxide reductase [Mycobacterium smegmatis str. MC2 155]	84.02	194	195
fig|6666666.28482.peg.4729	R019183	(gi:333990603) ahpC (JDM601 1963) - alkyl hydroperoxide reductase C protein AhpC [Mycobacterium sp. str. JDM601]	85.05	194	195
fig|6666666.28482.peg.4729	R019177	(gi:169631483) ahpC (MAB 4408c) - putative alkylhydroperoxidase C [Mycobacterium abscessus str. ATCC 19977]	83.94	193	195
fig|6666666.28482.peg.4761	R005426	(gi:41407707) fbpB (MAP1609c) - FbpB [Mycobacterium avium str. K-10 (subsp. paratuberculosis)]	81.4	326	326
fig|6666666.28482.peg.4761	R005427	(gi:118465697) fbpB (MAV 2816) - antigen 85-B [Mycobacterium avium str. 104]	81.1	326	326

fig|6666666.28482.peg.4761	R018878	(gi:333990613) fbpB (JDM601 1973) - hypothetical protein [Mycobacterium sp. str. JDM601]	78.46	325	326
fig|6666666.28482.peg.4761	R018872	(gi:183982783) fbpB (MMAR 2777) - secreted antigen 85-B FbpB [Mycobacterium marinum str. M]	80.75	320	326
fig|6666666.28482.peg.4761	R005429	(gi:118618369) fbpB (MUL 2970) - secreted antigen 85-B FbpB [Mycobacterium ulcerans str. Agy99]	79.5	320	326
fig|6666666.28482.peg.4761	R018875	(gi:253799070) fbpB (TBMG 02108) - secreted fibronectin-binding protein antigen 85-B fbpB [Mycobacterium tuberculosis str. KZN 1435]	80.43	320	326
fig|6666666.28482.peg.4761	R005421	(gi:15609023) fbpB (Rv1886c) - SECRETED ANTIGEN 85-B FBPB (85B) (ANTIGEN 85 COMPLEX B) (MYCOLYL TRANSFERASE 85B) (FIBRONECTIN-BINDING PROTEIN B) (EXTRACELLULAR ALPHA-ANTIGEN) [Mycobacterium tuberculosis str. H37Rv]	80.43	320	326
fig|6666666.28482.peg.4761	R005422	(gi:148661692) fbpB (MRA 1897) - secreted antigen 85-B FbpB [Mycobacterium tuberculosis str. H37Ra]	80.43	320	326
fig|6666666.28482.peg.4761	R013425	(gi:148823097) fbpB (TBFG 11914) - secreted antigen 85-B fbpB (fibronectin-binding protein B) [Mycobacterium tuberculosis str. F11]	80.43	320	326
fig|6666666.28482.peg.4761	R018874	(gi:340626894) fbpB (MCAN 19011) - secreted antigen 85-B FBPB [Mycobacterium canettii str. CIPT 140010059]	80.43	320	326
fig|6666666.28482.peg.4761	R005424	(gi:31793077) fbpB (Mb1918c) - secreted antigen 85-B fbpB (85B) (antigen 85 complex B) (Mycolyl transferase 85B) (Fibronectin-binding protein B) (Extracellular alpha-antigen) [Mycobacterium bovis str. AF2122/97]	80.43	320	326
fig|6666666.28482.peg.4761	R018873	(gi:339631939) fbpB (MAF 19080) - secreted antigen 85-B FBPB (85B) (antigen 85 complex B) [Mycobacterium africanum str. GM041182]	80.43	320	326
fig|6666666.28482.peg.4761	R005423	(gi:15841356) fbpB (MT1934) - esterase, putative, antigen 85-B [Mycobacterium tuberculosis str. CDC1551]	80.43	320	326
fig|6666666.28482.peg.4761	R018876	(gi:224990274) fbpB (JTY 1907) - secreted antigen 85-B [Mycobacterium bovis str. BCG str. Tokyo 172]	80.12	320	326
fig|6666666.28482.peg.4761	R005425	(gi:121637790) fbpB (BCG 1923c) - secreted antigen 85-B fbpB [Mycobacterium bovis str. BCG Pasteur 1173P2]	80.12	320	326
fig|6666666.28482.peg.4761	R005428	(gi:15828096) fbpB (ML2028) - antigen 85A, mycolyltransferase [Mycobacterium leprae str. TN]	76.76	325	326
fig|6666666.28482.peg.4761	R018877	(gi:221230573) fbpB (MLBr 02028) - antigen 85A, mycolyltransferase [Mycobacterium leprae str. Br4923]	76.76	325	326

fig|6666666.28482.peg.4761	R018871	(gi:333992762) fbpA (JDM601 4122) - hypothetical protein [Mycobacterium sp. str. JDM601]	76.51	315	326
fig|6666666.28482.peg.4761	R005413	(gi:15843426) fbpA (MT3911) - esterase, putative, antigen 85-A [Mycobacterium tuberculosis str. CDC1551]	75.79	316	326
fig|6666666.28482.peg.4761	R018868	(gi:253800852) fbpA (TBMG 03851) - secreted fibronectin-binding protein antigen fbpA [Mycobacterium tuberculosis str. KZN 1435]	75.79	316	326
fig|6666666.28482.peg.4761	R005411	(gi:15610940) fbpA (Rv3804c) - SECRETED ANTIGEN 85-A FBPA (MYCOLYL TRANSFERASE 85A) (FIBRONECTIN-BINDING PROTEIN A) (ANTIGEN 85 COMPLEX A) [Mycobacterium tuberculosis str. H37Rv]	75.79	316	326
fig|6666666.28482.peg.4761	R005412	(gi:148663672) fbpA (MRA 3844) - secreted antigen 85-A FbpA [Mycobacterium tuberculosis str. H37Ra]	75.79	316	326
fig|6666666.28482.peg.4761	R013424	(gi:148825011) fbpA (TBFG 13838) - secreted antigen 85-A fbpA (fibronectin-binding protein A) [Mycobacterium tuberculosis str. F11]	75.79	316	326
fig|6666666.28482.peg.4761	R018867	(gi:340628773) fbpA (MCAN 38231) - secreted antigen 85-A FBPA [Mycobacterium canettii str. CIPT 140010059]	75.79	316	326
fig|6666666.28482.peg.4761	R018869	(gi:224992216) fbpA (JTY 3868) - secreted antigen 85-A [Mycobacterium bovis str. BCG str. Tokyo 172]	75.79	316	326
fig|6666666.28482.peg.4761	R005415	(gi:121639721) fbpA (BCG 3866c) - Secreted antigen 85-A fbpA [Mycobacterium bovis str. BCG Pasteur 1173P2]	75.79	316	326
fig|6666666.28482.peg.4761	R005414	(gi:31794978) fbpA (Mb3834c) - SECRETED ANTIGEN 85-A FBPA (MYCOLYL TRANSFERASE 85A) (FIBRONECTIN-BINDING PROTEIN A) (ANTIGEN 85 COMPLEX A) [Mycobacterium bovis str. AF2122/97]	75.79	316	326
fig|6666666.28482.peg.4761	R018866	(gi:339633799) fbpA (MAF 38190) - secreted antigen 85-A FBPA (mycolyl transferase 85A) [Mycobacterium africanum str. GM041182]	75.47	316	326
fig|6666666.28482.peg.4761	R018864	(gi:183985336) fbpA (MMAR 5368) - secreted antigen 85-A FbpA [Mycobacterium marinum str. M]	75.47	316	326
fig|6666666.28482.peg.4761	R005419	(gi:118620004) fbpA (MUL 4987) - secreted antigen 85-A FbpA [Mycobacterium ulcerans str. Agy99]	74.84	316	326
fig|6666666.28482.peg.4761	R005417	(gi:118467129) fbpA (MAV 0214) - antigen 85-A [Mycobacterium avium str. 104]	76.21	309	326
fig|6666666.28482.peg.4761	R005416	(gi:41406314) fbpA (MAP0216) - FbpA [Mycobacterium avium str. K-10 (subsp. paratuberculosis)]	75.88	309	326

fig|6666666.28482.peg.4761	R005418	(gi:15826932) fbpA (ML0097) - antigen 85A, mycolytransferase [Mycobacterium leprae str. TN]	73.42	314	326
fig|6666666.28482.peg.4761	R018870	(gi:221229410) fbpA (MLBr 00097) - antigen 85A, mycolytransferase [Mycobacterium leprae str. Br4923]	73.42	314	326
fig|6666666.28482.peg.4761	R005446	(gi:120402899) fbpC (Mvan 1902) - putative esterase [Mycobacterium vanbaalenii str. PYR-1]	68.94	318	326
fig|6666666.28482.peg.4761	R005430	(gi:118473709) fbpB (MSMEG 2078) - antigen 85-C [Mycobacterium smegmatis str. MC2 155]	69.14	317	326
fig|6666666.28482.peg.4761	R005444	(gi:145225038) fbpC (Mflv 4459) - putative esterase [Mycobacterium gilvum str. PYR-GCK]	68.01	318	326
fig|6666666.28482.peg.4761	R005466	(gi:108801978) fbpC (Mmcs 5015) - putative esterase [Mycobacterium sp. str. MCS]	65.17	319	326
fig|6666666.28482.peg.4761	R005461	(gi:119871130) fbpC (Mkms 5103) - putative esterase [Mycobacterium sp. str. KMS]	65.17	319	326
fig|6666666.28482.peg.4761	R005456	(gi:126437959) fbpC (Mjls 5396) - putative esterase [Mycobacterium sp. str. JLS]	65.17	319	326
fig|6666666.28482.peg.4761	R005441	(gi:145221756) fbpC (Mflv 1164) - putative esterase [Mycobacterium gilvum str. PYR-GCK]	65.95	316	326
fig|6666666.28482.peg.4761	R005420	(gi:118468744) fbpA (MSMEG 6398) - antigen 85-A [Mycobacterium smegmatis str. MC2 155]	66.67	313	326
fig|6666666.28482.peg.4761	R005462	(gi:108798566) fbpC (Mmcs 1596) - putative esterase [Mycobacterium sp. str. MCS]	68.42	318	326
fig|6666666.28482.peg.4761	R005457	(gi:119867665) fbpC (Mkms 1620) - putative esterase [Mycobacterium sp. str. KMS]	68.42	318	326
fig|6666666.28482.peg.4761	R005452	(gi:126434164) fbpC (Mjls 1566) - putative esterase [Mycobacterium sp. str. JLS]	68.73	318	326
fig|6666666.28482.peg.4761	R005440	(gi:118467737) fbpC (MSMEG 3580) - antigen 85-C [Mycobacterium smegmatis str. MC2 155]	65.75	316	326
fig|6666666.28482.peg.4761	R005464	(gi:108799739) fbpC (Mmcs 2773) - putative esterase [Mycobacterium sp. str. MCS]	65.12	315	326
fig|6666666.28482.peg.4761	R005459	(gi:119868849) fbpC (Mkms 2817) - putative esterase [Mycobacterium sp. str. KMS]	65.12	315	326

fig|6666666.28482.peg.4761	R005455	(gi:126437080) fbpC (Mjls 4511) - putative esterase [Mycobacterium sp. str. JLS]	62.54	313	326
fig|6666666.28482.peg.4761	R005454	(gi:126435380) fbpC (Mjls 2800) - putative esterase [Mycobacterium sp. str. JLS]	65.12	315	326
fig|6666666.28482.peg.4761	R005433	(gi:15839510) fbpC (MT0137) - esterase, putative, antigen 85-C [Mycobacterium tuberculosis str. CDC1551]	65.5	309	326
fig|6666666.28482.peg.4761	R018882	(gi:253797048) fbpC (TBMG 00130) - secreted fibronectin-binding protein C antigen 85-C fbpC [Mycobacterium tuberculosis str. KZN 1435]	65.5	309	326
fig|6666666.28482.peg.4761	R005431	(gi:57116693) fbpC (Rv0129c) - SECRETED ANTIGEN 85-C FBPC (85C) (ANTIGEN 85 COMPLEX C) (AG58C) (MYCOLYL TRANSFERASE 85C) (FIBRONECTIN-BINDING PROTEIN C) [Mycobacterium tuberculosis str. H37Rv]	65.5	309	326
fig|6666666.28482.peg.4761	R005432	(gi:148659893) fbpC (MRA 0136) - secreted antigen 86-C FbpC [Mycobacterium tuberculosis str. H37Ra]	65.5	309	326
fig|6666666.28482.peg.4761	R013426	(gi:148821321) fbpC (TBFG 10130) - secreted antigen 85-C fbpC (fibronectin-binding protein C) [Mycobacterium tuberculosis str. F11]	65.5	309	326
fig|6666666.28482.peg.4761	R018881	(gi:340625164) fbpC (MCAN 01321) - secreted antigen 85-C FBPC [Mycobacterium canettii str. CIPT 140010059]	65.5	309	326
fig|6666666.28482.peg.4761	R018883	(gi:224988515) fbpC (JTY 0133) - secreted antigen 85-C [Mycobacterium bovis str. BCG str. Tokyo 172]	65.5	309	326
fig|6666666.28482.peg.4761	R005435	(gi:121636042) fbpC (BCG 0163c) - Secreted antigen 85-c fbpC (85C) [Mycobacterium bovis str. BCG Pasteur 1173P2]	65.5	309	326
fig|6666666.28482.peg.4761	R005434	(gi:31791308) fbpC (Mb0134c) - SECRETED ANTIGEN 85-C FBPC (85C) (ANTIGEN 85 COMPLEX C) (AG58C) (MYCOLYL TRANSFERASE 85C) (FIBRONECTIN-BINDING PROTEIN C) [Mycobacterium bovis str. AF2122/97]	65.5	309	326
fig|6666666.28482.peg.4761	R018880	(gi:339630211) fbpC (MAF 01290) - secreted antigen 85-C FbpC [Mycobacterium africanum str. GM041182]	65.5	309	326
fig|6666666.28482.peg.4761	R005438	(gi:15828432) fbpC (ML2655) - secreted antigen 85A, mycolyltransferase [Mycobacterium leprae str. TN]	63.78	308	326
fig|6666666.28482.peg.4761	R018884	(gi:221230909) fbpC (MLBr 02655) - secreted antigen 85A, mycolyltransferase [Mycobacterium leprae str. Br4923]	63.78	308	326

fig|6666666.28482.peg.4761	R005436	(gi:41409629) fbpC (MAP3531c) - FbpC2 [Mycobacterium avium str. K-10 (subsp. paratuberculosis)]	63.58	309	326
fig|6666666.28482.peg.4761	R005437	(gi:118465788) fbpC (MAV 5183) - antigen 85-C [Mycobacterium avium str. 104]	63.58	309	326
fig|6666666.28482.peg.4761	R005448	(gi:120404040) fbpC (Mvan 3061) - putative esterase [Mycobacterium vanbaalenii str. PYR-1]	65.03	298	326
fig|6666666.28482.peg.4761	R005443	(gi:145223921) fbpC (Mflv 3335) - putative esterase [Mycobacterium gilvum str. PYR-GCK]	63.32	311	326
fig|6666666.28482.peg.4761	R018865	(gi:169627281) fbpA (MAB 0176) - antigen 85-A precursor [Mycobacterium abscessus str. ATCC 19977]	61.68	313	326
fig|6666666.28482.peg.4761	R005439	(gi:118619846) fbpC (MUL 4793) - secreted antigen 85-C FbpC [Mycobacterium ulcerans str. Agy99]	65.5	309	326
fig|6666666.28482.peg.4761	R018879	(gi:183980359) fbpC (MMAR 0328) - secreted antigen 85-C FbpC [Mycobacterium marinum str. M]	65.5	309	326
fig|6666666.28482.peg.4761	R018885	(gi:333992822) fbpC (JDM601 4182) - hypothetical protein [Mycobacterium sp. str. JDM601]	65.87	289	326
fig|6666666.28482.peg.4761	R005458	(gi:119867920) fbpC (Mkms 1882) - putative esterase [Mycobacterium sp. str. KMS]	60.5	316	326
fig|6666666.28482.peg.4761	R005453	(gi:126434404) fbpC (Mjls 1816) - putative esterase [Mycobacterium sp. str. JLS]	60.38	315	326
fig|6666666.28482.peg.4798	R006296	(gi:41409369) devR/dosR (MAP3271c) - hypothetical protein [Mycobacterium avium str. K-10 (subsp. paratuberculosis)]	87.78	221	223
fig|6666666.28482.peg.4798	R006297	(gi:118466851) devR/dosR (MAV 4109) - two component transcriptional regulatory protein devr [Mycobacterium avium str. 104]	87.78	221	223
fig|6666666.28482.peg.4798	R006299	(gi:118472166) devR/dosR (MSMEG 5244) - two component transcriptional regulatory protein devr [Mycobacterium smegmatis str. MC2 155]	85.31	211	223
fig|6666666.28482.peg.4798	R006300	(gi:145224429) devR/dosR (Mflv 3847) - two component transcriptional regulator, LuxR family [Mycobacterium gilvum str. PYR-GCK]	86.32	212	223
fig|6666666.28482.peg.4798	R019302	(gi:253800166) devR/dosR (TBMG 03176) - two component system transcriptional regulator devR [Mycobacterium tuberculosis str. KZN 1435]	84.58	214	223
fig|6666666.28482.peg.4798	R006291	(gi:15610269) devR/dosR (Rv3133c) - TWO COMPONENT TRANSCRIPTIONAL REGULATORY PROTEIN DEVR (PROBABLY LUXR/UHPA-FAMILY) [Mycobacterium tuberculosis str. H37Rv]	84.58	214	223

fig|6666666.28482.peg.4798	R006292	(gi:148662987) devR/dosR (MRA 3165) - two component transcriptional regulatory protein DevR [Mycobacterium tuberculosis str. H37Ra]	84.58	214	223
fig|6666666.28482.peg.4798	R013509	(gi:148824327) devR/dosR (TBFG 13154) - two component system transcriptional regulatory protein devR (luxR/uhpA-family) [Mycobacterium tuberculosis str. F11]	84.58	214	223
fig|6666666.28482.peg.4798	R006293	(gi:15842706) devR/dosR (MT3219) - DNA-binding response regulator, LuxR family [Mycobacterium tuberculosis str. CDC1551]	84.58	214	223
fig|6666666.28482.peg.4798	R019301	(gi:340628109) devR/dosR (MCAN 31471) - two component transcriptional regulatory protein [Mycobacterium canettii str. CIPT 140010059]	84.58	214	223
fig|6666666.28482.peg.4798	R019303	(gi:224991508) devR/dosR (JTY 3151) - two component transcriptional regulatory protein [Mycobacterium bovis str. BCG str. Tokyo 172]	84.58	214	223
fig|6666666.28482.peg.4798	R006295	(gi:121639016) devR/dosR (BCG 3156c) - Two component transcriptional regulatory protein devR (probably luxR/uhpA-family) [Mycobacterium bovis str. BCG Pasteur 1173P2]	84.58	214	223
fig|6666666.28482.peg.4798	R006294	(gi:31794309) devR/dosR (Mb3157c) - TWO COMPONENT TRANSCRIPTIONAL REGULATORY PROTEIN DEVR (PROBABLY LUXR/UHPA-FAMILY) [Mycobacterium bovis str. AF2122/97]	84.58	214	223
fig|6666666.28482.peg.4798	R019300	(gi:339633140) devR/dosR (MAF 31410) - two component transcriptional regulatory protein DEVR (probably LUXR/UHPA-family) [Mycobacterium africanum str. GM041182]	84.58	214	223
fig|6666666.28482.peg.4798	R019304	(gi:333991618) devR/dosR (JDM601 2978) - two-component transcriptional regulatory protein DevR [Mycobacterium sp. str. JDM601]	84.69	209	223
fig|6666666.28482.peg.4798	R006307	(gi:108801090) devR/dosR (Mmcs 4126) - two component transcriptional regulator, LuxR family [Mycobacterium sp. str. MCS]	85.17	209	223
fig|6666666.28482.peg.4798	R006305	(gi:119870231) devR/dosR (Mkms 4201) - two component transcriptional regulator, LuxR family [Mycobacterium sp. str. KMS]	84.04	213	223
fig|6666666.28482.peg.4798	R006303	(gi:126436928) devR/dosR (Mjls 4357) - two component transcriptional regulator, LuxR family [Mycobacterium sp. str. JLS]	84.04	213	223
fig|6666666.28482.peg.4798	R006298	(gi:118617914) devR/dosR (MUL 2423) - two component transcriptional regulatory protein DevR [Mycobacterium ulcerans str. Agy99]	82.79	215	223
fig|6666666.28482.peg.4798	R019298	(gi:183981531) devR/dosR (MMAR 1516) - two-component transcriptional regulatory protein DevR [Mycobacterium marinum str. M]	82.79	215	223

fig|6666666.28482.peg.4798	R006302	(gi:120402438) devR/dosR (Mvan 1427) - two component transcriptional regulator, LuxR family [Mycobacterium vanbaalenii str. PYR-1]	82.63	213	223
fig|6666666.28482.peg.4798	R006308	(gi:108798049) devR/dosR (Mmcs 1076) - two component transcriptional regulator, LuxR family [Mycobacterium sp. str. MCS]	82.46	211	223
fig|6666666.28482.peg.4798	R006306	(gi:119867144) devR/dosR (Mkms 1092) - two component transcriptional regulator, LuxR family [Mycobacterium sp. str. KMS]	82.46	211	223
fig|6666666.28482.peg.4798	R006304	(gi:126433709) devR/dosR (Mjls 1103) - two component transcriptional regulator, LuxR family [Mycobacterium sp. str. JLS]	82.46	211	223
fig|6666666.28482.peg.4798	R006301	(gi:120403536) devR/dosR (Mvan 2548) - two component transcriptional regulator, LuxR family [Mycobacterium vanbaalenii str. PYR-1]	87.85	214	223
fig|6666666.28482.peg.4798	R019299	(gi:169630970) devR/dosR (MAB 3891c) - LuxR family transcriptional regulator [Mycobacterium abscessus str. ATCC 19977]	69.77	215	223
fig|6666666.28482.peg.4799	R006314	(gi:41409368) devS (MAP3270c) - hypothetical protein [Mycobacterium avium str. K-10 (subsp. paratuberculosis)]	77.7	546	572
fig|6666666.28482.peg.4799	R006315	(gi:118467162) devS (MAV 4108) - GAF family protein [Mycobacterium avium str. 104]	77.7	546	572
fig|6666666.28482.peg.4868	R005662	(gi:118466775) sapM (MAV 4287) - secreted acid phosphatase [Mycobacterium avium str. 104]	81.02	270	283
fig|6666666.28482.peg.4868	R005661	(gi:41409530) sapM (MAP3432) - hypothetical protein [Mycobacterium avium str. K-10 (subsp. paratuberculosis)]	81.58	262	283
fig|6666666.28482.peg.4868	R018927	(gi:224991692) sapM (JTY 3335) - putative acid phosphatase [Mycobacterium bovis str. BCG str. Tokyo 172]	72.76	278	283
fig|6666666.28482.peg.4868	R005660	(gi:121639233) sapM (BCG 3375) - putative acid phosphatase [Mycobacterium bovis str. BCG Pasteur 1173P2]	72.76	278	283
fig|6666666.28482.peg.4868	R005659	(gi:31794490) sapM (Mb3338) - POSSIBLE ACID PHOSPHATASE (ACID PHOSPHOMONOESTERASE) (PHOSPHOMONOESTERASE) (GLYCEROPHOSPHATASE) [Mycobacterium bovis str. AF2122/97]	72.76	278	283
fig|6666666.28482.peg.4868	R018924	(gi:339633317) sapM (MAF 33210) - putative acid phosphatase (acid phosphomonoesterase) [Mycobacterium africanum str. GM041182]	72.76	278	283
fig|6666666.28482.peg.4868	R018925	(gi:340628292) sapM (MCAN 33331) - putative acid phosphatase [Mycobacterium canettii str. CIPT 140010059]	72.4	278	283
fig|6666666.28482.peg.4868	R018926	(gi:253800353) sapM (TBMG 03358) - acid phosphatase [Mycobacterium tuberculosis str. KZN 1435]	72.4	278	283

fig|6666666.28482.peg.4868	R005656	(gi:15610446) sapM (Rv3310) - POSSIBLE ACID PHOSPHATASE (ACID PHOSPHOMONOESTERASE) (PHOSPHOMONOESTERASE) (GLYCEROPHOSPHATASE) [Mycobacterium tuberculosis str. H37Rv]	72.4	278	283
fig|6666666.28482.peg.4868	R005657	(gi:148663173) sapM (MRA 3351) - putative acid phosphatase [Mycobacterium tuberculosis str. H37Ra]	72.4	278	283
fig|6666666.28482.peg.4868	R013443	(gi:148824512) sapM (TBFG 13339) - hypothetical acid phosphatase [Mycobacterium tuberculosis str. F11]	72.4	278	283
fig|6666666.28482.peg.4868	R005658	(gi:15842901) sapM (MT3409) - acid phosphatase, putative [Mycobacterium tuberculosis str. CDC1551]	72.4	278	283
fig|6666666.28482.peg.4868	R018923	(gi:183981238) sapM (MMAR 1216) - acid phosphatase [Mycobacterium marinum str. M]	70.42	280	283
fig|6666666.28482.peg.4868	R005663	(gi:118618136) sapM (MUL 2676) - acid phosphatase [Mycobacterium ulcerans str. Agy99]	71.22	275	283
fig|6666666.28482.peg.5149	R019483	(gi:183984678) mce7A (MMAR 4710) - MCE-family protein [Mycobacterium marinum str. M]	81.88	425	425
fig|6666666.28482.peg.5149	R006740	(gi:118472804) mce7A (MSMEG 4794) - virulence factor [Mycobacterium smegmatis str. MC2 155]	63.29	425	425
fig|6666666.28482.peg.5149	R006739	(gi:118467131) mce7A (MAV 0948) - virulence factor Mce family protein, putative [Mycobacterium avium str. 104]	67.94	393	425
fig|6666666.28482.peg.5149	R006738	(gi:41406857) mce7A (MAP0759) - hypothetical protein [Mycobacterium avium str. K-10 (subsp. paratuberculosis)]	66.58	407	425
fig|6666666.28482.peg.5149	R006744	(gi:126436261) mce7A (Mjls 3684) - virulence factor Mce family protein [Mycobacterium sp. str. JLS]	61.46	392	425
fig|6666666.28482.peg.5149	R006746	(gi:108800637) mce7A (Mmcs 3671) - Mammalian cell entry [Mycobacterium sp. str. MCS]	61.46	392	425
fig|6666666.28482.peg.5149	R006745	(gi:119869776) mce7A (Mkms 3744) - virulence factor Mce family protein [Mycobacterium sp. str. KMS]	61.46	392	425
fig|6666666.28482.peg.5149	R006791	(gi:41408287) mce8A (MAP2189) - hypothetical protein [Mycobacterium avium str. K-10 (subsp. paratuberculosis)]	62.15	391	425
fig|6666666.28482.peg.5149	R006793	(gi:145225116) mce8A (Mflv 4538) - virulence factor Mce family protein [Mycobacterium gilvum str. PYR-GCK]	61.28	390	425
fig|6666666.28482.peg.5149	R006794	(gi:120403835) mce8A (Mvan 2851) - virulence factor Mce family protein [Mycobacterium vanbaalenii str. PYR-1]	60.97	392	425

fig|6666666.28482.peg.5149	R006797	(gi:108797255) mce8A (Mmcs 0275) - Mammalian cell entry [Mycobacterium sp. str. MCS]	60.26	390	425
fig|6666666.28482.peg.5149	R006796	(gi:119866340) mce8A (Mkms 0285) - virulence factor Mce family protein [Mycobacterium sp. str. KMS]	60.26	390	425
fig|6666666.28482.peg.5150	R019484	(gi:183984677) mce7B (MMAR 4709) - MCE-family protein [Mycobacterium marinum str. M]	85.34	341	346
fig|6666666.28482.peg.5150	R006747	(gi:41406858) mce7B (MAP0760) - hypothetical protein [Mycobacterium avium str. K-10 (subsp. paratuberculosis)]	70.41	338	346
fig|6666666.28482.peg.5150	R006748	(gi:118465177) mce7B (MAV 0949) - virulence factor Mce family protein [Mycobacterium avium str. 104]	70.41	338	346
fig|6666666.28482.peg.5150	R006749	(gi:118473503) mce7B (MSMEG 4793) - virulence factor Mce family protein [Mycobacterium smegmatis str. MC2 155]	65.4	341	346
fig|6666666.28482.peg.5150	R006752	(gi:120405103) mce7B (Mvan 4149) - virulence factor Mce family protein [Mycobacterium vanbaalenii str. PYR-1]	65.59	340	346
fig|6666666.28482.peg.5150	R006750	(gi:145223096) mce7B (Mflv 2509) - virulence factor Mce family protein [Mycobacterium gilvum str. PYR-GCK]	64.12	340	346
fig|6666666.28482.peg.5150	R006753	(gi:126436260) mce7B (Mjls 3683) - virulence factor Mce family protein [Mycobacterium sp. str. JLS]	63.24	340	346
fig|6666666.28482.peg.5150	R006755	(gi:108800636) mce7B (Mmcs 3670) - Mammalian cell entry [Mycobacterium sp. str. MCS]	63.24	340	346
fig|6666666.28482.peg.5150	R006754	(gi:119869775) mce7B (Mkms 3743) - virulence factor Mce family protein [Mycobacterium sp. str. KMS]	63.24	340	346
fig|6666666.28482.peg.5150	R006804	(gi:108797256) mce8B (Mmcs 0276) - Mammalian cell entry [Mycobacterium sp. str. MCS]	63.77	334	346
fig|6666666.28482.peg.5150	R006803	(gi:119866341) mce8B (Mkms 0286) - virulence factor Mce family protein [Mycobacterium sp. str. KMS]	63.77	334	346
fig|6666666.28482.peg.5150	R006802	(gi:126432880) mce8B (Mjls 0267) - virulence factor Mce family protein [Mycobacterium sp. str. JLS]	63.77	334	346
fig|6666666.28482.peg.5150	R006799	(gi:118468491) mce8B (MSMEG 2856) - virulence factor Mce family protein [Mycobacterium smegmatis str. MC2 155]	62.97	341	346
fig|6666666.28482.peg.5150	R006798	(gi:41408288) mce8B (MAP2190) - hypothetical protein [Mycobacterium avium str. K-10 (subsp. paratuberculosis)]	63.93	341	346

fig|6666666.28482.peg.5150	R006800	(gi:145225115) mce8B (Mflv 4537) - virulence factor Mce family protein [Mycobacterium gilvum str. PYR-GCK]	63.17	332	346
fig|6666666.28482.peg.5150	R006801	(gi:120403836) mce8B (Mvan 2852) - virulence factor Mce family protein [Mycobacterium vanbaalenii str. PYR-1]	62.72	336	346
fig|6666666.28482.peg.5151	R019485	(gi:183984676) mce7C (MMAR 4708) - MCE-family protein [Mycobacterium marinum str. M]	83.24	358	360
fig|6666666.28482.peg.5151	R006756	(gi:41406859) mce7C (MAP0761) - hypothetical protein [Mycobacterium avium str. K-10 (subsp. paratuberculosis)]	64.35	356	360
fig|6666666.28482.peg.5151	R006757	(gi:118465942) mce7C (MAV 0950) - virulence factor Mce family protein [Mycobacterium avium str. 104]	64.35	356	360
fig|6666666.28482.peg.5151	R006761	(gi:120405102) mce7C (Mvan 4148) - virulence factor Mce family protein [Mycobacterium vanbaalenii str. PYR-1]	64.89	356	360
fig|6666666.28482.peg.5151	R006762	(gi:126436259) mce7C (Mjls 3682) - virulence factor Mce family protein [Mycobacterium sp. str. JLS]	61.52	356	360
fig|6666666.28482.peg.5151	R006764	(gi:108800635) mce7C (Mmcs 3669) - Mammalian cell entry [Mycobacterium sp. str. MCS]	61.24	356	360
fig|6666666.28482.peg.5151	R006763	(gi:119869774) mce7C (Mkms 3742) - virulence factor Mce family protein [Mycobacterium sp. str. KMS]	61.24	356	360
fig|6666666.28482.peg.5151	R006759	(gi:145223097) mce7C (Mflv 2510) - virulence factor Mce family protein [Mycobacterium gilvum str. PYR-GCK]	62.92	356	360
fig|6666666.28482.peg.5151	R006805	(gi:41408289) mce8C (MAP2191) - hypothetical protein [Mycobacterium avium str. K-10 (subsp. paratuberculosis)]	61.28	358	360
fig|6666666.28482.peg.5151	R006807	(gi:145225114) mce8C (Mflv 4536) - virulence factor Mce family protein [Mycobacterium gilvum str. PYR-GCK]	60.61	358	360
fig|6666666.28482.peg.5151	R006811	(gi:108797257) mce8C (Mmcs 0277) - Mammalian cell entry [Mycobacterium sp. str. MCS]	60.61	358	360
fig|6666666.28482.peg.5151	R006810	(gi:119866342) mce8C (Mkms 0287) - virulence factor Mce family protein [Mycobacterium sp. str. KMS]	60.61	358	360
fig|6666666.28482.peg.5151	R006809	(gi:126432881) mce8C (Mjls 0268) - virulence factor Mce family protein [Mycobacterium sp. str. JLS]	60.61	358	360
fig|6666666.28482.peg.5260	R018735	(gi:183981778) fadD28 (MMAR 1765) - acyl-CoA synthetase [Mycobacterium marinum str. M]	60.48	581	582

fig|6666666.28482.peg.5260	R005202	(gi:118617586) fadD28 (MUL 2008) - fatty-acyl AMP ligase FadD28 [Mycobacterium ulcerans str. Agy99]	60.14	581	582
fig|6666666.28482.peg.5260	R005200	(gi:118462725) fadD28 (MAV 1328) - acyl-CoA synthase [Mycobacterium avium str. 104]	62.03	582	582
fig|6666666.28482.peg.5260	R018739	(gi:224991315) fadD28 (JTY 2958) - acyl-CoA synthetase [Mycobacterium bovis str. BCG str. Tokyo 172]	60.48	581	582
fig|6666666.28482.peg.5260	R005198	(gi:121638823) fadD28 (BCG 2963) - fatty-acid-CoA ligase fadD28 [Mycobacterium bovis str. BCG Pasteur 1173P2]	60.48	581	582
fig|6666666.28482.peg.5260	R005197	(gi:31794118) fadD28 (Mb2966) - acyl-CoA synthase [Mycobacterium bovis str. AF2122/97]	60.48	581	582
fig|6666666.28482.peg.5260	R018737	(gi:340627932) fadD28 (MCAN 29631) - fatty-acid-CoA ligase FADD28 [Mycobacterium canettii str. CIPT 140010059]	60.31	581	582
fig|6666666.28482.peg.5260	R018738	(gi:253797971) fadD28 (TBMG 01031) - acyl-CoA synthetase [Mycobacterium tuberculosis str. KZN 1435]	60.31	581	582
fig|6666666.28482.peg.5260	R005194	(gi:15610078) fadD28 (Rv2941) - acyl-CoA synthase [Mycobacterium tuberculosis str. H37Rv]	60.31	581	582
fig|6666666.28482.peg.5260	R005195	(gi:148662787) fadD28 (MRA 2967) - fatty-acid-CoA ligase FadD28 [Mycobacterium tuberculosis str. H37Ra]	60.31	581	582
fig|6666666.28482.peg.5260	R013401	(gi:148824130) fadD28 (TBFG 12955) - fatty-acid-CoA ligase fadD28 [Mycobacterium tuberculosis str. F11]	60.31	581	582
fig|6666666.28482.peg.5260	R005196	(gi:15842487) fadD28 (MT3011) - acyl-CoA synthase [Mycobacterium tuberculosis str. CDC1551]	60.31	581	582
fig|6666666.28482.peg.5260	R018736	(gi:339632948) fadD28 (MAF 29460) - fatty-acid-CoA ligase FADD28 (fatty-acid-CoA synthetase) [Mycobacterium africanum str. GM041182]	60.31	581	582
fig|6666666.28482.peg.5263	R005384	(gi:118463607) papA2 (MAV 1762) - condensation domain protein [Mycobacterium avium str. 104]	83.51	473	480
fig|6666666.28482.peg.5295	R006136	(gi:41409504) sigF (MAP3406c) - sigma factor SigF [Mycobacterium avium str. K-10 (subsp. paratuberculosis)]	91.98	262	262
fig|6666666.28482.peg.5295	R006138	(gi:118618107) sigF (MUL 2640) - alternate RNA polymerase sigma factor SigF [Mycobacterium ulcerans str. Agy99]	89.31	262	262
fig|6666666.28482.peg.5295	R019215	(gi:183981270) sigF (MMAR 1248) - RNA polymerase sigma factor SigF [Mycobacterium marinum str. M]	89.31	262	262

fig|6666666.28482.peg.5295	R019219	(gi:253800329) sigF (TBMG 03334) - RNA polymerase sigma factor SigF [Mycobacterium tuberculosis str. KZN 1435]	88.55	262	262
fig|6666666.28482.peg.5295	R006131	(gi:15610422) sigF (Rv3286c) - sigma factor SigF [Mycobacterium tuberculosis str. H37Rv]	88.55	262	262
fig|6666666.28482.peg.5295	R006132	(gi:148663149) sigF (MRA 3327) - sigma factor SigF [Mycobacterium tuberculosis str. H37Ra]	88.55	262	262
fig|6666666.28482.peg.5295	R013498	(gi:148824488) sigF (TBFG 13315) - alternate RNA polymerase sigma factor sigF [Mycobacterium tuberculosis str. F11]	88.55	262	262
fig|6666666.28482.peg.5295	R006133	(gi:15842877) sigF (MT3385) - sigma factor SigF [Mycobacterium tuberculosis str. CDC1551]	88.55	262	262
fig|6666666.28482.peg.5295	R019218	(gi:340628268) sigF (MCAN 33091) - alternate RNA polymerase sigma factor SIGF [Mycobacterium canettii str. CIPT 140010059]	88.55	262	262
fig|6666666.28482.peg.5295	R019220	(gi:224991668) sigF (JTY 3311) - RNA polymerase sigma factor SigF [Mycobacterium bovis str. BCG str. Tokyo 172]	88.55	262	262
fig|6666666.28482.peg.5295	R006135	(gi:121639175) sigF (BCG 3315c) - putative rna polymerase sigma factor sigF [Mycobacterium bovis str. BCG Pasteur 1173P2]	88.55	262	262
fig|6666666.28482.peg.5295	R006134	(gi:31794466) sigF (Mb3314c) - sigma factor SigF [Mycobacterium bovis str. AF2122/97]	88.55	262	262
fig|6666666.28482.peg.5295	R019217	(gi:339633295) sigF (MAF 32970) - alternate RNA polymerase sigma factor SIGF [Mycobacterium africanum str. GM041182]	88.55	262	262
fig|6666666.28482.peg.5295	R006144	(gi:108798252) sigF (Mmcs 1281) - RNA polymerase, sigma 28 subunit [Mycobacterium sp. str. MCS]	84.41	262	262
fig|6666666.28482.peg.5295	R006143	(gi:119867348) sigF (Mkms 1298) - RNA polymerase, sigma 28 subunit, FliA/WhiG family [Mycobacterium sp. str. KMS]	84.41	262	262
fig|6666666.28482.peg.5295	R006142	(gi:126433912) sigF (Mjls 1310) - RNA polymerase, sigma 28 subunit, FliA/WhiG family [Mycobacterium sp. str. JLS]	84.41	262	262
fig|6666666.28482.peg.5295	R006137	(gi:118464372) sigF (MAV 4256) - RNA polymerase sigma-F factor [Mycobacterium avium str. 104]	90.87	241	262
fig|6666666.28482.peg.5295	R019221	(gi:333991745) sigF (JDM601 3105) - RNA polymerase sigma factor SigF [Mycobacterium sp. str. JDM601]	83.21	262	262
fig|6666666.28482.peg.5295	R006140	(gi:145225367) sigF (Mflv 4789) - RNA polymerase, sigma 28 subunit, FliA/WhiG family [Mycobacterium gilvum str. PYR-GCK]	87.1	248	262

fig|6666666.28482.peg.5295	R006141	(gi:120402666) sigF (Mvan 1660) - RNA polymerase, sigma 28 subunit, FliA/WhiG family [Mycobacterium vanbaalenii str. PYR-1]	85.48	248	262
fig|6666666.28482.peg.5295	R006139	(gi:118470482) sigF (MSMEG 1804) - RNA polymerase sigma-F factor [Mycobacterium smegmatis str. MC2 155]	83.94	249	262
fig|6666666.28482.peg.5402	R006348	(gi:118465982) mce1A (MAV 5015) - virulence factor Mce family protein [Mycobacterium avium str. 104]	69.2	431	441
fig|6666666.28482.peg.5402	R019323	(gi:253797091) mce1A (TBMG 00170) - MCE-family protein mce1A [Mycobacterium tuberculosis str. KZN 1435]	67.17	441	441
fig|6666666.28482.peg.5402	R006341	(gi:57116701) mce1A (Rv0169) - MCE-FAMILY PROTEIN MCE1A [Mycobacterium tuberculosis str. H37Rv]	67.17	441	441
fig|6666666.28482.peg.5402	R006342	(gi:148659933) mce1A (MRA 0177) - MCE-family protein Mce1A [Mycobacterium tuberculosis str. H37Ra]	67.17	441	441
fig|6666666.28482.peg.5402	R013512	(gi:148821361) mce1A (TBFG 10170) - MCE-family protein mce1A [Mycobacterium tuberculosis str. F11]	67.17	441	441
fig|6666666.28482.peg.5402	R006343	(gi:15839547) mce1A (MT0178) - virulence factor [Mycobacterium tuberculosis str. CDC1551]	67.17	441	441
fig|6666666.28482.peg.5402	R019322	(gi:340625206) mce1A (MCAN 01751) - MCE-family protein MCE1A [Mycobacterium canettii str. CIPT 140010059]	67.17	441	441
fig|6666666.28482.peg.5402	R019324	(gi:224988554) mce1A (JTY 0175) - MCE-family protein [Mycobacterium bovis str. BCG str. Tokyo 172]	67.17	441	441
fig|6666666.28482.peg.5402	R006345	(gi:121636081) mce1A (BCG 0206) - MCE-family protein mce1A [Mycobacterium bovis str. BCG Pasteur 1173P2]	67.17	441	441
fig|6666666.28482.peg.5402	R006344	(gi:31791347) mce1A (Mb0175) - MCE-FAMILY PROTEIN MCE1A [Mycobacterium bovis str. AF2122/97]	67.17	441	441
fig|6666666.28482.peg.5402	R019321	(gi:339630250) mce1A (MAF 01700) - MCE-family protein MCE1A [Mycobacterium africanum str. GM041182]	67.17	441	441
fig|6666666.28482.peg.5402	R006346	(gi:41409702) mce1A (MAP3604) - Mce1 2 [Mycobacterium avium str. K-10 (subsp. paratuberculosis)]	68.45	431	441
fig|6666666.28482.peg.5402	R006350	(gi:118464886) mce1A (MAV 4126) - virulence factor Mce family protein [Mycobacterium avium str. 104]	69.46	425	441
fig|6666666.28482.peg.5402	R006349	(gi:118464153) mce1A (MAV 4125) - virulence factor Mce family protein [Mycobacterium avium str. 104]	66.44	441	441

fig|6666666.28482.peg.5402	R006351	(gi:15828393) mce1A (ML2589) - putative cell invasion protein [Mycobacterium leprae str. TN]	65.9	430	441
fig|6666666.28482.peg.5402	R019325	(gi:221230870) mce1A (MLBr 02589) - putative cell invasion protein [Mycobacterium leprae str. Br4923]	65.9	430	441
fig|6666666.28482.peg.5402	R019320	(gi:183980443) mce1A (MMAR 0412) - MCE-family protein Mce1A [Mycobacterium marinum str. M]	66.67	423	441
fig|6666666.28482.peg.5402	R006347	(gi:41409387) mce1A (MAP3289c) - Mce1 1 [Mycobacterium avium str. K-10 (subsp. paratuberculosis)]	68	425	441
fig|6666666.28482.peg.5402	R006352	(gi:118616795) mce1A (MUL 1062) - MCE-family protein Mce1A [Mycobacterium ulcerans str. Agy99]	66.43	423	441
fig|6666666.28482.peg.5402	R006413	(gi:120401162) mce2A (Mvan 0134) - virulence factor Mce family protein [Mycobacterium vanbaalenii str. PYR-1]	60.23	435	441
fig|6666666.28482.peg.5402	R006410	(gi:118465139) mce2A (MAV 4551) - virulence factor Mce family protein [Mycobacterium avium str. 104]	65.67	402	441
fig|6666666.28482.peg.5402	R006414	(gi:126432721) mce2A (Mjls 0108) - virulence factor Mce family protein [Mycobacterium sp. str. JLS]	60.51	428	441
fig|6666666.28482.peg.5402	R006409	(gi:41410182) mce2A (MAP4084) - Mce2 [Mycobacterium avium str. K-10 (subsp. paratuberculosis)]	63.66	406	441
fig|6666666.28482.peg.5402	R019365	(gi:253797523) mce2A (TBMG 00596) - MCE-family protein mce2A [Mycobacterium tuberculosis str. KZN 1435]	63.18	402	441
fig|6666666.28482.peg.5402	R013518	(gi:148821791) mce2A (TBFG 10600) - MCE-family protein mce2A [Mycobacterium tuberculosis str. F11]	63.18	402	441
fig|6666666.28482.peg.5402	R006406	(gi:15839991) mce2A (MT0618) - virulence factor [Mycobacterium tuberculosis str. CDC1551]	63.18	402	441
fig|6666666.28482.peg.5402	R006404	(gi:57116756) mce2A (Rv0589) - MCE-FAMILY PROTEIN MCE2A [Mycobacterium tuberculosis str. H37Rv]	63.18	402	441
fig|6666666.28482.peg.5402	R006405	(gi:148660358) mce2A (MRA 0596) - MCE-family protein Mce2A [Mycobacterium tuberculosis str. H37Ra]	63.18	402	441
fig|6666666.28482.peg.5402	R019364	(gi:340625619) mce2A (MCAN 05951) - MCE-family protein MCE2A [Mycobacterium canettii str. CIPT 140010059]	62.94	402	441
fig|6666666.28482.peg.5402	R019366	(gi:224988979) mce2A (JTY 0604) - MCE-family protein [Mycobacterium bovis str. BCG str. Tokyo 172]	62.94	402	441

fig|6666666.28482.peg.5402	R006408	(gi:121636507) mce2A (BCG 0634) - mce-family protein mce2A [Mycobacterium bovis str. BCG Pasteur 1173P2]	62.94	402	441
fig|6666666.28482.peg.5402	R006407	(gi:31791771) mce2A (Mb0604) - MCE-FAMILY PROTEIN MCE2A [Mycobacterium bovis str. AF2122/97]	62.94	402	441
fig|6666666.28482.peg.5402	R019363	(gi:339630657) mce2A (MAF 05960) - MCE-family protein MCE2A [Mycobacterium africanum str. GM041182]	62.94	402	441
fig|6666666.28482.peg.5403	R006359	(gi:41409703) mce1B (MAP3605) - hypothetical protein [Mycobacterium avium str. K-10 (subsp. paratuberculosis)]	75.72	346	346
fig|6666666.28482.peg.5403	R006360	(gi:118463171) mce1B (MAV 5014) - virulence factor Mce family protein [Mycobacterium avium str. 104]	75.72	346	346
fig|6666666.28482.peg.5403	R019327	(gi:183980444) mce1B (MMAR 0413) - MCE-family protein Mce1B [Mycobacterium marinum str. M]	73.99	346	346
fig|6666666.28482.peg.5403	R006362	(gi:118616796) mce1B (MUL 1063) - MCE-family protein Mce1B [Mycobacterium ulcerans str. Agy99]	73.7	346	346
fig|6666666.28482.peg.5403	R019329	(gi:340625207) mce1B (MCAN 01761) - MCE-family protein MCE1B [Mycobacterium canettii str. CIPT 140010059]	73.7	346	346
fig|6666666.28482.peg.5403	R019330	(gi:253797092) mce1B (TBMG 00171) - MCE-family protein mce1B [Mycobacterium tuberculosis str. KZN 1435]	73.41	346	346
fig|6666666.28482.peg.5403	R006354	(gi:15607311) mce1B (Rv0170) - MCE-FAMILY PROTEIN MCE1B [Mycobacterium tuberculosis str. H37Rv]	73.41	346	346
fig|6666666.28482.peg.5403	R006355	(gi:148659934) mce1B (MRA 0178) - MCE-family protein Mce1B [Mycobacterium tuberculosis str. H37Ra]	73.41	346	346
fig|6666666.28482.peg.5403	R013513	(gi:148821362) mce1B (TBFG 10171) - MCE-family protein mce1B [Mycobacterium tuberculosis str. F11]	73.41	346	346
fig|6666666.28482.peg.5403	R006356	(gi:15839548) mce1B (MT0179) - virulence factor mce family protein [Mycobacterium tuberculosis str. CDC1551]	73.41	346	346
fig|6666666.28482.peg.5403	R019331	(gi:224988555) mce1B (JTY 0176) - MCE-family protein [Mycobacterium bovis str. BCG str. Tokyo 172]	73.12	346	346
fig|6666666.28482.peg.5403	R006358	(gi:121636082) mce1B (BCG 0207) - MCE-family protein mce1B [Mycobacterium bovis str. BCG Pasteur 1173P2]	73.12	346	346
fig|6666666.28482.peg.5403	R006357	(gi:31791348) mce1B (Mb0176) - MCE-FAMILY PROTEIN MCE1B [Mycobacterium bovis str. AF2122/97]	73.12	346	346

fig|6666666.28482.peg.5403	R019328	(gi:339630251) mce1B (MAF 01710) - MCE-family protein MCE1B [Mycobacterium africanum str. GM041182]	73.12	346	346
fig|6666666.28482.peg.5403	R006361	(gi:15828394) mce1B (ML2590) - putative secreted protein [Mycobacterium leprae str. TN]	73.41	346	346
fig|6666666.28482.peg.5403	R019332	(gi:221230871) mce1B (MLBr 02590) - putative secreted protein [Mycobacterium leprae str. Br4923]	73.41	346	346
fig|6666666.28482.peg.5403	R006428	(gi:108797099) mce2B (Mmcs 0119) - Mammalian cell entry [Mycobacterium sp. str. MCS]	66.76	346	346
fig|6666666.28482.peg.5403	R006427	(gi:119866184) mce2B (Mkms 0128) - virulence factor Mce family protein [Mycobacterium sp. str. KMS]	66.76	346	346
fig|6666666.28482.peg.5403	R006426	(gi:126432722) mce2B (Mjls 0109) - virulence factor Mce family protein [Mycobacterium sp. str. JLS]	66.76	346	346
fig|6666666.28482.peg.5403	R006363	(gi:118468987) mce1B (MSMEG 0135) - virulence factor Mce family protein [Mycobacterium smegmatis str. MC2 155]	65.32	346	346
fig|6666666.28482.peg.5403	R006424	(gi:145221305) mce2B (Mflv 0710) - virulence factor Mce family protein [Mycobacterium gilvum str. PYR-GCK]	67.34	346	346
fig|6666666.28482.peg.5403	R019333	(gi:333988763) mce1B (JDM601 0123) - MCE-family protein Mce1B [Mycobacterium sp. str. JDM601]	67.92	346	346
fig|6666666.28482.peg.5403	R006425	(gi:120401163) mce2B (Mvan 0135) - virulence factor Mce family protein [Mycobacterium vanbaalenii str. PYR-1]	66.18	346	346
fig|6666666.28482.peg.5403	R019368	(gi:340625620) mce2B (MCAN 05961) - mce-family protein mce2b [Mycobacterium canettii str. CIPT 140010059]	62.14	346	346
fig|6666666.28482.peg.5403	R019370	(gi:224988980) mce2B (JTY 0605) - MCE-family protein [Mycobacterium bovis str. BCG str. Tokyo 172]	62.14	346	346
fig|6666666.28482.peg.5403	R006421	(gi:121636508) mce2B (BCG 0635) - mce-family protein mce2B [Mycobacterium bovis str. BCG Pasteur 1173P2]	62.14	346	346
fig|6666666.28482.peg.5403	R006420	(gi:31791772) mce2B (Mb0605) - MCE-FAMILY PROTEIN MCE2B [Mycobacterium bovis str. AF2122/97]	62.14	346	346
fig|6666666.28482.peg.5403	R019367	(gi:339630658) mce2B (MAF 05970) - MCE-family protein MCE2B [Mycobacterium africanum str. GM041182]	62.14	346	346
fig|6666666.28482.peg.5403	R006422	(gi:41410183) mce2B (MAP4085) - hypothetical protein [Mycobacterium avium str. K-10 (subsp. paratuberculosis)]	60.98	346	346

fig|6666666.28482.peg.5403	R006423	(gi:118465867) mce2B (MAV 4550) - virulence factor Mce family protein [Mycobacterium avium str. 104]	60.69	346	346
fig|6666666.28482.peg.5403	R019369	(gi:253797524) mce2B (TBMG 00597) - MCE-family protein mce2B [Mycobacterium tuberculosis str. KZN 1435]	65.37	257	346
fig|6666666.28482.peg.5403	R006417	(gi:15607730) mce2B (Rv0590) - MCE-FAMILY PROTEIN MCE2B [Mycobacterium tuberculosis str. H37Rv]	65.37	257	346
fig|6666666.28482.peg.5403	R006418	(gi:148660359) mce2B (MRA 0597) - MCE-family protein Mce2B [Mycobacterium tuberculosis str. H37Ra]	65.37	257	346
fig|6666666.28482.peg.5403	R013519	(gi:148821792) mce2B (TBFG 10601) - MCE-family protein mce2B [Mycobacterium tuberculosis str. F11]	65.37	257	346
fig|6666666.28482.peg.5403	R006419	(gi:15839992) mce2B (MT0619) - virulence factor mce family protein [Mycobacterium tuberculosis str. CDC1551]	64.98	257	346
fig|6666666.28482.peg.5404	R006370	(gi:118467050) mce1C (MAV 5013) - mce-family protein mce1c [Mycobacterium avium str. 104]	76.88	505	519
fig|6666666.28482.peg.5404	R006369	(gi:41409704) mce1C (MAP3606) - hypothetical protein [Mycobacterium avium str. K-10 (subsp. paratuberculosis)]	75.88	480	519
fig|6666666.28482.peg.5404	R019334	(gi:183980445) mce1C (MMAR 0414) - MCE-family protein Mce1C [Mycobacterium marinum str. M]	74.69	480	519
fig|6666666.28482.peg.5404	R019336	(gi:340625208) mce1C (MCAN 01771) - MCE-family protein MCE1C [Mycobacterium canettii str. CIPT 140010059]	76.18	465	519
fig|6666666.28482.peg.5404	R006364	(gi:15607312) mce1C (Rv0171) - MCE-FAMILY PROTEIN MCE1C [Mycobacterium tuberculosis str. H37Rv]	75.54	465	519
fig|6666666.28482.peg.5404	R006365	(gi:148659935) mce1C (MRA 0179) - MCE-family protein Mce1C [Mycobacterium tuberculosis str. H37Ra]	75.54	465	519
fig|6666666.28482.peg.5404	R013514	(gi:148821363) mce1C (TBFG 10172) - MCE-family protein mce1C [Mycobacterium tuberculosis str. F11]	75.54	465	519
fig|6666666.28482.peg.5404	R019338	(gi:224988556) mce1C (JTY 0177) - MCE-family protein [Mycobacterium bovis str. BCG str. Tokyo 172]	75.32	465	519
fig|6666666.28482.peg.5404	R006368	(gi:121636083) mce1C (BCG 0208) - MCE-family protein mce1C [Mycobacterium bovis str. BCG Pasteur 1173P2]	75.32	465	519
fig|6666666.28482.peg.5404	R006367	(gi:31791349) mce1C (Mb0177) - MCE-FAMILY PROTEIN MCE1C [Mycobacterium bovis str. AF2122/97]	75.32	465	519

fig|6666666.28482.peg.5404	R019335	(gi:339630252) mce1C (MAF 01720) - MCE-family protein MCE1C [Mycobacterium africanum str. GM041182]	75.32	465	519
fig|6666666.28482.peg.5404	R006366	(gi:15839549) mce1C (MT0180) - virulence factor mce family protein [Mycobacterium tuberculosis str. CDC1551]	75.88	455	519
fig|6666666.28482.peg.5404	R006371	(gi:15828395) mce1C (ML2591) - putative secreted protein [Mycobacterium leprae str. TN]	68.99	505	519
fig|6666666.28482.peg.5404	R019339	(gi:221230872) mce1C (MLBr 02591) - putative secreted protein [Mycobacterium leprae str. Br4923]	68.99	505	519
fig|6666666.28482.peg.5404	R006372	(gi:118616797) mce1C (MUL 1064) - MCE-family protein Mce1C [Mycobacterium ulcerans str. Agy99]	74.07	480	519
fig|6666666.28482.peg.5404	R019337	(gi:253797093) mce1C (TBMG 00172) - MCE-family protein mce1C [Mycobacterium tuberculosis str. KZN 1435]	75.88	451	519
fig|6666666.28482.peg.5404	R006438	(gi:126432723) mce2C (Mjls 0110) - virulence factor Mce family protein [Mycobacterium sp. str. JLS]	62.32	478	519
fig|6666666.28482.peg.5404	R019371	(gi:339630659) mce2C (MAF 05980) - MCE-family protein MCE2C [Mycobacterium africanum str. GM041182]	68.58	470	519
fig|6666666.28482.peg.5404	R019374	(gi:224988981) mce2C (JTY 0606) - MCE-family protein [Mycobacterium bovis str. BCG str. Tokyo 172]	68.37	470	519
fig|6666666.28482.peg.5404	R006433	(gi:121636509) mce2C (BCG 0636) - mce-family protein mce2C [Mycobacterium bovis str. BCG Pasteur 1173P2]	68.37	470	519
fig|6666666.28482.peg.5404	R006432	(gi:31791773) mce2C (Mb0606) - MCE-FAMILY PROTEIN MCE2C [Mycobacterium bovis str. AF2122/97]	68.37	470	519
fig|6666666.28482.peg.5404	R019373	(gi:253797526) mce2C (TBMG 00599) - MCE-family protein mce2C [Mycobacterium tuberculosis str. KZN 1435]	68.37	470	519
fig|6666666.28482.peg.5404	R006429	(gi:15607731) mce2C (Rv0591) - MCE-FAMILY PROTEIN MCE2C [Mycobacterium tuberculosis str. H37Rv]	68.37	470	519
fig|6666666.28482.peg.5404	R006430	(gi:148660361) mce2C (MRA 0599) - MCE-family protein Mce2C [Mycobacterium tuberculosis str. H37Ra]	68.37	470	519
fig|6666666.28482.peg.5404	R013520	(gi:148821794) mce2C (TBFG 10603) - MCE-family protein mce2C [Mycobacterium tuberculosis str. F11]	68.37	470	519
fig|6666666.28482.peg.5404	R019372	(gi:340625621) mce2C (MCAN 05971) - MCE-family protein MCE2C [Mycobacterium canettii str. CIPT 140010059]	68.58	470	519

fig|6666666.28482.peg.5404	R006437	(gi:120401164) mce2C (Mvan 0136) - virulence factor Mce family protein [Mycobacterium vanbaalenii str. PYR-1]	64	448	519
fig|6666666.28482.peg.5404	R006440	(gi:108797100) mce2C (Mmcs 0120) - Mammalian cell entry [Mycobacterium sp. str. MCS]	62.73	478	519
fig|6666666.28482.peg.5404	R006439	(gi:119866185) mce2C (Mkms 0129) - virulence factor Mce family protein [Mycobacterium sp. str. KMS]	62.73	478	519
fig|6666666.28482.peg.5404	R006373	(gi:118473407) mce1C (MSMEG 0136) - virulence factor Mce family protein, putative [Mycobacterium smegmatis str. MC2 155]	61.43	475	519
fig|6666666.28482.peg.5404	R006435	(gi:118463703) mce2C (MAV 4549) - mce-family protein mce2c [Mycobacterium avium str. 104]	65.96	467	519
fig|6666666.28482.peg.5404	R006434	(gi:41410184) mce2C (MAP4086) - hypothetical protein [Mycobacterium avium str. K-10 (subsp. paratuberculosis)]	65.53	467	519
fig|6666666.28482.peg.5404	R006436	(gi:145221304) mce2C (Mflv 0709) - virulence factor Mce family protein [Mycobacterium gilvum str. PYR-GCK]	62.67	448	519
fig|6666666.28482.peg.5404	R006431	(gi:15839994) mce2C (MT0621) - virulence factor mce family protein [Mycobacterium tuberculosis str. CDC1551]	66.57	356	519
fig|6666666.28482.peg.5405	R006379	(gi:41409705) mce1D (MAP3607) - hypothetical protein [Mycobacterium avium str. K-10 (subsp. paratuberculosis)]	81.97	466	505
fig|6666666.28482.peg.5405	R006380	(gi:118466487) mce1D (MAV 5012) - mce-family protein mce1d [Mycobacterium avium str. 104]	81.97	466	505
fig|6666666.28482.peg.5405	R019344	(gi:253797094) mce1D (TBMG 00173) - MCE-family protein mce1D [Mycobacterium tuberculosis str. KZN 1435]	80.77	468	505
fig|6666666.28482.peg.5405	R013515	(gi:148821364) mce1D (TBFG 10173) - MCE-family protein mce1D [Mycobacterium tuberculosis str. F11]	80.77	468	505
fig|6666666.28482.peg.5405	R006374	(gi:15607313) mce1D (Rv0172) - MCE-FAMILY PROTEIN MCE1D [Mycobacterium tuberculosis str. H37Rv]	80.77	468	505
fig|6666666.28482.peg.5405	R006375	(gi:148659936) mce1D (MRA 0180) - MCE-family protein Mce1D [Mycobacterium tuberculosis str. H37Ra]	80.77	468	505
fig|6666666.28482.peg.5405	R006376	(gi:15839550) mce1D (MT0181) - virulence factor mce family protein [Mycobacterium tuberculosis str. CDC1551]	80.77	468	505
fig|6666666.28482.peg.5405	R019345	(gi:224988557) mce1D (JTY 0178) - MCE-family protein [Mycobacterium bovis str. BCG str. Tokyo 172]	80.77	468	505

fig|6666666.28482.peg.5405	R006378	(gi:121636084) mce1D (BCG 0209) - MCE-family protein mce1D [Mycobacterium bovis str. BCG Pasteur 1173P2]	80.77	468	505
fig|6666666.28482.peg.5405	R006377	(gi:31791350) mce1D (Mb0178) - MCE-FAMILY PROTEIN MCE1D [Mycobacterium bovis str. AF2122/97]	80.77	468	505
fig|6666666.28482.peg.5405	R019342	(gi:339630253) mce1D (MAF 01730) - MCE-family protein MCE1D [Mycobacterium africanum str. GM041182]	80.77	468	505
fig|6666666.28482.peg.5405	R019343	(gi:340625209) mce1D (MCAN 01781) - MCE-family protein MCE1D [Mycobacterium canettii str. CIPT 140010059]	80.77	468	505
fig|6666666.28482.peg.5405	R019341	(gi:183980446) mce1D (MMAR 0415) - MCE-family protein Mce1D [Mycobacterium marinum str. M]	79.91	468	505
fig|6666666.28482.peg.5405	R006382	(gi:118616798) mce1D (MUL 1065) - MCE-family protein Mce1D [Mycobacterium ulcerans str. Agy99]	79.49	468	505
fig|6666666.28482.peg.5405	R006381	(gi:15828396) mce1D (ML2592) - putative secreted protein [Mycobacterium leprae str. TN]	77.59	482	505
fig|6666666.28482.peg.5405	R019346	(gi:221230873) mce1D (MLBr 02592) - putative secreted protein [Mycobacterium leprae str. Br4923]	77.59	482	505
fig|6666666.28482.peg.5405	R019347	(gi:333988765) mce1D (JDM601 0125) - MCE-family protein Mce1D [Mycobacterium sp. str. JDM601]	68.24	465	505
fig|6666666.28482.peg.5405	R019377	(gi:253797527) mce2D (TBMG 00600) - MCE-family protein mce2D [Mycobacterium tuberculosis str. KZN 1435]	69.82	444	505
fig|6666666.28482.peg.5405	R006441	(gi:15607732) mce2D (Rv0592) - MCE-FAMILY PROTEIN MCE2D [Mycobacterium tuberculosis str. H37Rv]	69.82	444	505
fig|6666666.28482.peg.5405	R006442	(gi:148660362) mce2D (MRA 0600) - MCE-family protein Mce2D [Mycobacterium tuberculosis str. H37Ra]	69.82	444	505
fig|6666666.28482.peg.5405	R013521	(gi:148821795) mce2D (TBFG 10604) - MCE-family protein mce2D [Mycobacterium tuberculosis str. F11]	69.82	444	505
fig|6666666.28482.peg.5405	R006443	(gi:15839995) mce2D (MT0622) - virulence factor mce family protein [Mycobacterium tuberculosis str. CDC1551]	69.82	444	505
fig|6666666.28482.peg.5405	R019376	(gi:340625622) mce2D (MCAN 05981) - MCE-family protein MCE2D [Mycobacterium canettii str. CIPT 140010059]	69.82	444	505
fig|6666666.28482.peg.5405	R019375	(gi:339630660) mce2D (MAF 05990) - MCE-family protein MCE2D [Mycobacterium africanum str. GM041182]	69.82	444	505

fig|6666666.28482.peg.5405	R006444	(gi:31791774) mce2D (Mb0607) - MCE-FAMILY PROTEIN MCE2DA [FIRST PART] [Mycobacterium bovis str. AF2122/97]	69.91	432	505
fig|6666666.28482.peg.5405	R019378	(gi:224988982) mce2D (JTY 0607) - MCE-family protein [Mycobacterium bovis str. BCG str. Tokyo 172]	69.43	435	505
fig|6666666.28482.peg.5405	R006445	(gi:121636510) mce2D (BCG 0637) - mce-family protein mce2Da [Mycobacterium bovis str. BCG Pasteur 1173P2]	69.43	435	505
fig|6666666.28482.peg.5405	R006452	(gi:108797101) mce2D (Mmcs 0121) - Mammalian cell entry [Mycobacterium sp. str. MCS]	68.5	447	505
fig|6666666.28482.peg.5405	R006451	(gi:119866186) mce2D (Mkms 0130) - virulence factor Mce family protein [Mycobacterium sp. str. KMS]	68.5	447	505
fig|6666666.28482.peg.5405	R006450	(gi:126432724) mce2D (Mjls 0111) - virulence factor Mce family protein [Mycobacterium sp. str. JLS]	68.5	447	505
fig|6666666.28482.peg.5405	R006446	(gi:41410185) mce2D (MAP4087) - hypothetical protein [Mycobacterium avium str. K-10 (subsp. paratuberculosis)]	65.77	481	505
fig|6666666.28482.peg.5405	R006447	(gi:118464414) mce2D (MAV 4548) - virulence factor mce family protein [Mycobacterium avium str. 104]	65.77	481	505
fig|6666666.28482.peg.5405	R006383	(gi:118471615) mce1D (MSMEG 0137) - virulence factor mce family protein [Mycobacterium smegmatis str. MC2 155]	65.36	449	505
fig|6666666.28482.peg.5405	R006448	(gi:145221303) mce2D (Mflv 0708) - virulence factor Mce family protein [Mycobacterium gilvum str. PYR-GCK]	65.05	448	505
fig|6666666.28482.peg.5405	R006449	(gi:120401165) mce2D (Mvan 0137) - virulence factor Mce family protein [Mycobacterium vanbaalenii str. PYR-1]	64.67	443	505
fig|6666666.28482.peg.5406	R006389	(gi:41409706) mce1E (MAP3608) - LprK [Mycobacterium avium str. K-10 (subsp. paratuberculosis)]	75.99	379	392
fig|6666666.28482.peg.5406	R006390	(gi:118466982) mce1E (MAV 5011) - virulence factor Mce family protein [Mycobacterium avium str. 104]	75.99	379	392
fig|6666666.28482.peg.5406	R019350	(gi:340625210) mce1E (MCAN 01791) - putative MCE-family lipoprotein LPRK [Mycobacterium canettii str. CIPT 140010059]	76.7	382	392
fig|6666666.28482.peg.5406	R006391	(gi:15828397) mce1E (ML2593) - putative lipoprotein [Mycobacterium leprae str. TN]	73.16	380	392
fig|6666666.28482.peg.5406	R019353	(gi:221230874) mce1E (MLBr 02593) - putative lipoprotein [Mycobacterium leprae str. Br4923]	73.16	380	392

fig|6666666.28482.peg.5406	R019348	(gi:183980447) mce1E (MMAR 0416) - MCE family lipoprotein LprK [Mycobacterium marinum str. M]	72.51	382	392
fig|6666666.28482.peg.5406	R019351	(gi:253797095) mce1E (TBMG 00174) - MCE-family lipoprotein mce1E [Mycobacterium tuberculosis str. KZN 1435]	76.44	382	392
fig|6666666.28482.peg.5406	R006386	(gi:15839551) mce1E (MT0182) - virulence factor mce family protein [Mycobacterium tuberculosis str. CDC1551]	76.44	382	392
fig|6666666.28482.peg.5406	R006384	(gi:15607314) mce1E (Rv0173) - POSSIBLE MCE-FAMILY LIPOPROTEIN LPRK (MCE-FAMILY LIPOPROTEIN MCE1E) [Mycobacterium tuberculosis str. H37Rv]	76.44	382	392
fig|6666666.28482.peg.5406	R006385	(gi:148659937) mce1E (MRA 0181) - MCE-family lipoprotein LprK [Mycobacterium tuberculosis str. H37Ra]	76.44	382	392
fig|6666666.28482.peg.5406	R013516	(gi:148821365) mce1E (TBFG 10174) - MCE-family lipoprotein lprK (MCE-family lipoprotein mce1e) [Mycobacterium tuberculosis str. F11]	76.44	382	392
fig|6666666.28482.peg.5406	R019352	(gi:224988558) mce1E (JTY 0179) - putative MCE-family lipoprotein mce1E [Mycobacterium bovis str. BCG str. Tokyo 172]	76.44	382	392
fig|6666666.28482.peg.5406	R006388	(gi:121636085) mce1E (BCG 0210) - putative mce-family lipoprotein lprK (MCE-family lipoprotein mce1E) [Mycobacterium bovis str. BCG Pasteur 1173P2]	76.44	382	392
fig|6666666.28482.peg.5406	R006387	(gi:31791351) mce1E (Mb0179) - POSSIBLE MCE-FAMILY LIPOPROTEIN LPRK (MCE-FAMILY LIPOPROTEIN MCE1E) [Mycobacterium bovis str. AF2122/97]	76.44	382	392
fig|6666666.28482.peg.5406	R019349	(gi:339630254) mce1E (MAF 01740) - putative MCE-family lipoprotein LprK (MCE-family lipoprotein Mce1E) [Mycobacterium africanum str. GM041182]	76.44	382	392
fig|6666666.28482.peg.5406	R006392	(gi:118616799) mce1E (MUL 1066) - MCE-family lipoprotein LprK (MCE-family lipoprotein Mce1E) [Mycobacterium ulcerans str. Agy99]	71.99	382	392
fig|6666666.28482.peg.5406	R006393	(gi:118472695) mce1E (MSMEG 0138) - virulence factor Mce family protein [Mycobacterium smegmatis str. MC2 155]	66.94	360	392
fig|6666666.28482.peg.5406	R019380	(gi:340625623) mce2E (MCAN 05991) - putative MCE-family lipoprotein LPRL [Mycobacterium canettii str. CIPT 140010059]	65.14	370	392
fig|6666666.28482.peg.5406	R006461	(gi:120401166) mce2E (Mvan 0138) - virulence factor Mce family protein [Mycobacterium vanbaalenii str. PYR-1]	64.35	359	392
fig|6666666.28482.peg.5406	R006460	(gi:145221302) mce2E (Mflv 0707) - virulence factor Mce family protein [Mycobacterium gilvum str. PYR-GCK]	64.17	360	392

fig|6666666.28482.peg.5406	R019381	(gi:253797528) mce2E (TBMG 00601) - MCE-family lipoprotein mce2E [Mycobacterium tuberculosis str. KZN 1435]	64.59	370	392
fig|6666666.28482.peg.5406	R006453	(gi:15607733) mce2E (Rv0593) - POSSIBLE MCE-FAMILY LIPOPROTEIN LPRL (MCE-FAMILY LIPOPROTEIN MCE2E) [Mycobacterium tuberculosis str. H37Rv]	64.59	370	392
fig|6666666.28482.peg.5406	R006454	(gi:148660363) mce2E (MRA 0601) - MCE-family lipoprotein LprL [Mycobacterium tuberculosis str. H37Ra]	64.59	370	392
fig|6666666.28482.peg.5406	R013522	(gi:148821796) mce2E (TBFG 10605) - MCE-family lipoprotein lprL (MCE-family lipoprotein mce2e) [Mycobacterium tuberculosis str. F11]	64.59	370	392
fig|6666666.28482.peg.5406	R006455	(gi:15839996) mce2E (MT0623) - virulence factor mce family protein [Mycobacterium tuberculosis str. CDC1551]	64.59	370	392
fig|6666666.28482.peg.5406	R019382	(gi:224988983) mce2E (JTY 0608) - putative MCE-family lipoprotein [Mycobacterium bovis str. BCG str. Tokyo 172]	64.59	370	392
fig|6666666.28482.peg.5406	R006457	(gi:121636511) mce2E (BCG 0639) - putative mce-family lipoprotein lprL [Mycobacterium bovis str. BCG Pasteur 1173P2]	64.59	370	392
fig|6666666.28482.peg.5406	R019379	(gi:339630661) mce2E (MAF 06000) - putative MCE-family lipoprotein LPRL (MCE-family lipoprotein MCE2E) [Mycobacterium africanum str. GM041182]	64.59	370	392
fig|6666666.28482.peg.5406	R006464	(gi:108797102) mce2E (Mmcs 0122) - Mammalian cell entry [Mycobacterium sp. str. MCS]	65.96	379	392
fig|6666666.28482.peg.5406	R006463	(gi:119866187) mce2E (Mkms 0131) - virulence factor Mce family protein [Mycobacterium sp. str. KMS]	65.96	379	392
fig|6666666.28482.peg.5406	R006462	(gi:126432725) mce2E (Mjls 0112) - virulence factor Mce family protein [Mycobacterium sp. str. JLS]	65.7	379	392
fig|6666666.28482.peg.5406	R006459	(gi:118462367) mce2E (MAV 4547) - virulence factor Mce family protein [Mycobacterium avium str. 104]	63.96	369	392
fig|6666666.28482.peg.5406	R006458	(gi:41410186) mce2E (MAP4088) - LprL [Mycobacterium avium str. K-10 (subsp. paratuberculosis)]	63.69	369	392
fig|6666666.28482.peg.5406	R019354	(gi:333988766) mce1E (JDM601 0126) - MCE family lipoprotein LprK [Mycobacterium sp. str. JDM601]	61.74	379	392
fig|6666666.28482.peg.5407	R006399	(gi:41409707) mce1F (MAP3609) - hypothetical protein [Mycobacterium avium str. K-10 (subsp. paratuberculosis)]	76.65	514	516

fig|6666666.28482.peg.5407	R006401	(gi:15828398) mce1F (ML2594) - putative secreted protein [Mycobacterium leprae str. TN]	75.49	514	516
fig|6666666.28482.peg.5407	R019360	(gi:221230875) mce1F (MLBr 02594) - putative secreted protein [Mycobacterium leprae str. Br4923]	75.49	514	516
fig|6666666.28482.peg.5407	R019355	(gi:183980448) mce1F (MMAR 0417) - MCE-family protein Mce1F [Mycobacterium marinum str. M]	76.94	514	516
fig|6666666.28482.peg.5407	R006402	(gi:118616800) mce1F (MUL 1067) - MCE-family protein Mce1F [Mycobacterium ulcerans str. Agy99]	76.55	514	516
fig|6666666.28482.peg.5407	R019358	(gi:253797096) mce1F (TBMG 00175) - MCE-family protein mce1F [Mycobacterium tuberculosis str. KZN 1435]	78.21	514	516
fig|6666666.28482.peg.5407	R013517	(gi:148821366) mce1F (TBFG 10175) - MCE-family protein mce1F [Mycobacterium tuberculosis str. F11]	78.21	514	516
fig|6666666.28482.peg.5407	R006396	(gi:15839552) mce1F (MT0183) - virulence factor mce family protein [Mycobacterium tuberculosis str. CDC1551]	78.21	514	516
fig|6666666.28482.peg.5407	R019359	(gi:224988559) mce1F (JTY 0180) - MCE-family protein [Mycobacterium bovis str. BCG str. Tokyo 172]	78.21	514	516
fig|6666666.28482.peg.5407	R006398	(gi:121636086) mce1F (BCG 0211) - MCE-family protein mce1F [Mycobacterium bovis str. BCG Pasteur 1173P2]	78.21	514	516
fig|6666666.28482.peg.5407	R006397	(gi:31791352) mce1F (Mb0180) - MCE-FAMILY PROTEIN MCE1F [Mycobacterium bovis str. AF2122/97]	78.21	514	516
fig|6666666.28482.peg.5407	R019356	(gi:339630255) mce1F (MAF 01750) - MCE-family protein MCE1F [Mycobacterium africanum str. GM041182]	78.21	514	516
fig|6666666.28482.peg.5407	R019357	(gi:340625211) mce1F (MCAN 01801) - MCE-family protein MCE1F [Mycobacterium canettii str. CIPT 140010059]	78.02	514	516
fig|6666666.28482.peg.5407	R006394	(gi:15607315) mce1F (Rv0174) - MCE-FAMILY PROTEIN MCE1F [Mycobacterium tuberculosis str. H37Rv]	78.02	514	516
fig|6666666.28482.peg.5407	R006395	(gi:148659938) mce1F (MRA 0182) - MCE-family protein Mce1F [Mycobacterium tuberculosis str. H37Ra]	78.02	514	516
fig|6666666.28482.peg.5407	R006476	(gi:108797103) mce2F (Mmcs 0123) - Mammalian cell entry [Mycobacterium sp. str. MCS]	69.5	514	516
fig|6666666.28482.peg.5407	R006475	(gi:119866188) mce2F (Mkms 0132) - virulence factor Mce family protein [Mycobacterium sp. str. KMS]	69.5	514	516

fig|6666666.28482.peg.5407	R006474	(gi:126432726) mce2F (Mjls 0113) - virulence factor Mce family protein [Mycobacterium sp. str. JLS]	69.31	514	516
fig|6666666.28482.peg.5407	R019385	(gi:253797529) mce2F (TBMG 00602) - MCE-family protein mce2F [Mycobacterium tuberculosis str. KZN 1435]	69.13	514	516
fig|6666666.28482.peg.5407	R006465	(gi:15607734) mce2F (Rv0594) - MCE-FAMILY PROTEIN MCE2F [Mycobacterium tuberculosis str. H37Rv]	69.13	514	516
fig|6666666.28482.peg.5407	R006466	(gi:148660364) mce2F (MRA 0602) - MCE-family protein Mce2F [Mycobacterium tuberculosis str. H37Ra]	69.13	514	516
fig|6666666.28482.peg.5407	R013523	(gi:148821797) mce2F (TBFG 10606) - MCE-family protein mce2F [Mycobacterium tuberculosis str. F11]	69.13	514	516
fig|6666666.28482.peg.5407	R019386	(gi:224988984) mce2F (JTY 0609) - MCE-family protein [Mycobacterium bovis str. BCG str. Tokyo 172]	69.13	514	516
fig|6666666.28482.peg.5407	R006469	(gi:121636512) mce2F (BCG 0640) - mce-family protein mce2F [Mycobacterium bovis str. BCG Pasteur 1173P2]	69.13	514	516
fig|6666666.28482.peg.5407	R006468	(gi:31791776) mce2F (Mb0610) - MCE-FAMILY PROTEIN MCE2F [Mycobacterium bovis str. AF2122/97]	69.13	514	516
fig|6666666.28482.peg.5407	R019383	(gi:339630662) mce2F (MAF 06010) - MCE-family protein MCE2F [Mycobacterium africanum str. GM041182]	69.13	514	516
fig|6666666.28482.peg.5407	R006467	(gi:15839997) mce2F (MT0624) - virulence factor mce family protein [Mycobacterium tuberculosis str. CDC1551]	69.13	514	516
fig|6666666.28482.peg.5407	R019384	(gi:340625624) mce2F (MCAN 06001) - MCE-family protein MCE2F [Mycobacterium canettii str. CIPT 140010059]	68.93	514	516
fig|6666666.28482.peg.5407	R006403	(gi:118473635) mce1F (MSMEG 0139) - mce-family protein mce1f [Mycobacterium smegmatis str. MC2 155]	68.98	514	516
fig|6666666.28482.peg.5407	R006470	(gi:41410187) mce2F (MAP4089) - hypothetical protein [Mycobacterium avium str. K-10 (subsp. paratuberculosis)]	68.47	516	516
fig|6666666.28482.peg.5407	R006471	(gi:118463921) mce2F (MAV 4546) - mce-family protein mce2f [Mycobacterium avium str. 104]	68.42	512	516
fig|6666666.28482.peg.5407	R006472	(gi:145221301) mce2F (Mflv 0706) - virulence factor Mce family protein [Mycobacterium gilvum str. PYR-GCK]	65.26	514	516
fig|6666666.28482.peg.5407	R019361	(gi:333988767) mce1F (JDM601 0127) - MCE-family protein Mce1F [Mycobacterium sp. str. JDM601]	63.71	514	516

fig|6666666.28482.peg.5407	R006473	(gi:120401167) mce2F (Mvan 0139) - virulence factor Mce family protein [Mycobacterium vanbaalenii str. PYR-1]	65.83	514	516
fig|6666666.28482.peg.5407	R006400	(gi:118462429) mce1F (MAV 5010) - mce-family protein mce1f [Mycobacterium avium str. 104]	79.1	311	516
fig|6666666.28482.peg.5543	R006332	(gi:118467123) relA (MAV 3464) - GTP pyrophosphokinase [Mycobacterium avium str. 104]	94.69	789	797
fig|6666666.28482.peg.5543	R006331	(gi:41407145) relA (MAP1047) - RelA [Mycobacterium avium str. K-10 (subsp. paratuberculosis)]	94.8	786	797
fig|6666666.28482.peg.5543	R019316	(gi:253798336) relA (TBMG 01390) - GTP pyrophosphokinase relA [Mycobacterium tuberculosis str. KZN 1435]	94.45	789	797
fig|6666666.28482.peg.5543	R006328	(gi:15842122) relA (MT2660) - GTP pyrophosphokinase [Mycobacterium tuberculosis str. CDC1551]	94.45	789	797
fig|6666666.28482.peg.5543	R006326	(gi:15609720) relA (Rv2583c) - PROBABLE GTP PYROPHOSPHOKINASE RELA (ATP:GTP 3'-PYROPHOSPHOTRANSFERASE) (PPGPP SYNTHETASE I) ((P)PPGPP SYNTHETASE) (GTP DIPHOSPHOKINASE) [Mycobacterium tuberculosis str. H37Rv]	94.56	786	797
fig|6666666.28482.peg.5543	R006327	(gi:148662422) relA (MRA 2612) - GTP pyrophosphokinase RelA [Mycobacterium tuberculosis str. H37Ra]	94.56	786	797
fig|6666666.28482.peg.5543	R013511	(gi:148823778) relA (TBFG 12603) - GTP pyrophosphokinase relA [Mycobacterium tuberculosis str. F11]	94.56	786	797
fig|6666666.28482.peg.5543	R019315	(gi:340627600) relA (MCAN 26251) - putative GTP pyrophosphokinase RELA [Mycobacterium canettii str. CIPT 140010059]	94.56	786	797
fig|6666666.28482.peg.5543	R019317	(gi:224990962) relA (JTY 2600) - putative GTP pyrophosphokinase [Mycobacterium bovis str. BCG str. Tokyo 172]	94.56	786	797
fig|6666666.28482.peg.5543	R006330	(gi:121638468) relA (BCG 2606c) - putative GTP pyrophosphokinase relA [Mycobacterium bovis str. BCG Pasteur 1173P2]	94.56	786	797
fig|6666666.28482.peg.5543	R006329	(gi:31793767) relA (Mb2614c) - PROBABLE GTP PYROPHOSPHOKINASE RELA (ATP:GTP 3'-PYROPHOSPHOTRANSFERASE) (PPGPP SYNTHETASE I) ((P)PPGPP SYNTHETASE) (GTP DIPHOSPHOKINASE) [Mycobacterium bovis str. AF2122/97]	94.56	786	797
fig|6666666.28482.peg.5543	R019314	(gi:339632611) relA (MAF 26000) - putative GTP pyrophosphokinase RELA (ATP:GTP 3'-pyrophosphotransferase) [Mycobacterium africanum str. GM041182]	94.56	786	797

fig|6666666.28482.peg.5543	R019312	(gi:183982138) relA (MMAR 2124) - GTP pyrophosphokinase RelA [Mycobacterium marinum str. M]	94.28	786	797
fig|6666666.28482.peg.5543	R006334	(gi:118617353) relA (MUL 1729) - GTP pyrophosphokinase RelA [Mycobacterium ulcerans str. Agy99]	93.8	789	797
fig|6666666.28482.peg.5543	R006333	(gi:15827167) relA (ML0491) - putative GTP pyrophosphokinase [Mycobacterium leprae str. TN]	93.77	786	797
fig|6666666.28482.peg.5543	R019318	(gi:221229645) relA (MLBr 00491) - putative GTP pyrophosphokinase [Mycobacterium leprae str. Br4923]	93.77	786	797
fig|6666666.28482.peg.5543	R006340	(gi:108799247) relA (Mmcs 2280) - (p)ppGpp synthetase I, SpoT/RelA [Mycobacterium sp. str. MCS]	87.02	786	797
fig|6666666.28482.peg.5543	R006339	(gi:119868363) relA (Mkms 2327) - (p)ppGpp synthetase I, SpoT/RelA [Mycobacterium sp. str. KMS]	87.02	786	797
fig|6666666.28482.peg.5543	R006337	(gi:120403572) relA (Mvan 2587) - (p)ppGpp synthetase I, SpoT/RelA [Mycobacterium vanbaalenii str. PYR-1]	88.82	778	797
fig|6666666.28482.peg.5543	R006338	(gi:126434905) relA (Mjls 2319) - (p)ppGpp synthetase I, SpoT/RelA [Mycobacterium sp. str. JLS]	86.48	786	797
fig|6666666.28482.peg.5543	R019319	(gi:333991010) relA (JDM601 2370) - GTP pyrophosphokinase RelA [Mycobacterium sp. str. JDM601]	87.66	783	797
fig|6666666.28482.peg.5543	R006335	(gi:118471771) relA (MSMEG 2965) - GTP pyrophosphokinase [Mycobacterium smegmatis str. MC2 155]	91.6	738	797
fig|6666666.28482.peg.5543	R019313	(gi:169629960) relA (MAB 2876c) - GTP pyrophosphokinase [Mycobacterium abscessus str. ATCC 19977]	88.77	772	797
fig|6666666.28482.peg.5543	R006336	(gi:145224394) relA (Mflv 3812) - (p)ppGpp synthetase I, SpoT/RelA [Mycobacterium gilvum str. PYR-GCK]	87.42	786	797
fig|6666666.28482.peg.5582	R006209	(gi:118466812) phoP (MAV 0701) - DNA-binding response regulator PhoP [Mycobacterium avium str. 104]	96.86	223	223
fig|6666666.28482.peg.5582	R006208	(gi:41406689) phoP (MAP0591) - PhoP [Mycobacterium avium str. K-10 (subsp. paratuberculosis)]	96.41	223	223
fig|6666666.28482.peg.5582	R006205	(gi:15840172) phoP (MT0782) - DNA-binding response regulator [Mycobacterium tuberculosis str. CDC1551]	95.96	223	223
fig|6666666.28482.peg.5582	R019256	(gi:253797703) phoP (TBMG 00772) - two component system transcriptional regulator phoP [Mycobacterium tuberculosis str. KZN 1435]	95.96	223	223

fig|6666666.28482.peg.5582	R006203	(gi:15607897) phoP (Rv0757) - POSSIBLE TWO COMPONENT SYSTEM RESPONSE TRANSCRIPTIONAL POSITIVE REGULATOR PHOP [Mycobacterium tuberculosis str. H37Rv]	95.96	223	223
fig|6666666.28482.peg.5582	R013503	(gi:148821963) phoP (TBFG 10772) - two component system response transcriptional positive regulator phoP [Mycobacterium tuberculosis str. F11]	95.96	223	223
fig|6666666.28482.peg.5582	R019255	(gi:340625776) phoP (MCAN 07611) - putative two component system response transcriptional positive regulator PHOP [Mycobacterium canettii str. CIPT 140010059]	95.96	223	223
fig|6666666.28482.peg.5582	R019257	(gi:224989153) phoP (JTY 0779) - putative two component system response transcriptional positive regulator [Mycobacterium bovis str. BCG str. Tokyo 172]	95.96	223	223
fig|6666666.28482.peg.5582	R006207	(gi:121636681) phoP (BCG 0809) - putative two component system response transcriptional positive regulator phoP [Mycobacterium bovis str. BCG Pasteur 1173P2]	95.96	223	223
fig|6666666.28482.peg.5582	R006206	(gi:31791945) phoP (Mb0780) - POSSIBLE TWO COMPONENT SYSTEM RESPONSE TRANSCRIPTIONAL POSITIVE REGULATOR PHOP [Mycobacterium bovis str. AF2122/97]	95.96	223	223
fig|6666666.28482.peg.5582	R019254	(gi:339630827) phoP (MAF 07690) - putative two component system response transcriptional positive regulator PHOP [Mycobacterium africanum str. GM041182]	95.96	223	223
fig|6666666.28482.peg.5582	R019252	(gi:183984910) phoP (MMAR 4942) - two-component system response phosphate regulon transcriptional regulator, PhoP [Mycobacterium marinum str. M]	95.07	223	223
fig|6666666.28482.peg.5582	R006204	(gi:148660534) phoP (MRA 0767) - putative two component system response transcriptional positive regulator PhoP [Mycobacterium tuberculosis str. H37Ra]	95.52	223	223
fig|6666666.28482.peg.5582	R006210	(gi:118616306) phoP (MUL 0463) - two component system response phosphate regulon transcriptional regulator, PhoP [Mycobacterium ulcerans str. Agy99]	94.62	223	223
fig|6666666.28482.peg.5582	R019258	(gi:333992254) phoP (JDM601 3614) - two-component system response phosphate regulon transcriptional regulator PhoP [Mycobacterium sp. str. JDM601]	93.27	223	223

fig|6666666.28482.peg.5582	R006216	(gi:108801553) phoP (Mmcs 4590) - two component transcriptional regulator, winged helix family [Mycobacterium sp. str. MCS]	91.48	223	223
fig|6666666.28482.peg.5582	R006215	(gi:119870707) phoP (Mkms 4678) - two component transcriptional regulator, winged helix family [Mycobacterium sp. str. KMS]	91.48	223	223
fig|6666666.28482.peg.5582	R006214	(gi:126437538) phoP (Mjls 4973) - two component transcriptional regulator, winged helix family [Mycobacterium sp. str. JLS]	91.48	223	223
fig|6666666.28482.peg.5582	R006211	(gi:118471457) phoP (MSMEG 5872) - DNA-binding response regulator PhoP [Mycobacterium smegmatis str. MC2 155]	92.38	223	223
fig|6666666.28482.peg.5582	R006213	(gi:120406123) phoP (Mvan 5175) - two component transcriptional regulator, winged helix family [Mycobacterium vanbaalenii str. PYR-1]	90.58	223	223
fig|6666666.28482.peg.5582	R006212	(gi:145222173) phoP (Mflv 1581) - two component transcriptional regulator, winged helix family [Mycobacterium gilvum str. PYR-GCK]	90.13	223	223
fig|6666666.28482.peg.5582	R019253	(gi:169627776) phoP (MAB 0673) - putative DNA-binding response regulator PhoP [Mycobacterium abscessus str. ATCC 19977]	88.79	223	223
fig|6666666.28482.peg.5583	R006222	(gi:41406690) phoR (MAP0592) - PhoR [Mycobacterium avium str. K-10 (subsp. paratuberculosis)]	90.81	468	480
fig|6666666.28482.peg.5583	R006223	(gi:118464141) phoR (MAV 0703) - sensor histidine kinase PhoR [Mycobacterium avium str. 104]	90.6	468	480
fig|6666666.28482.peg.5583	R019259	(gi:183984909) phoR (MMAR 4941) - two-component system response phosphate sensor kinase, PhoR [Mycobacterium marinum str. M]	81.46	477	480
fig|6666666.28482.peg.5583	R006224	(gi:118616307) phoR (MUL 0464) - two component system response phosphate sensor kinase, PhoR [Mycobacterium ulcerans str. Agy99]	81.25	477	480
fig|6666666.28482.peg.5583	R006227	(gi:120406122) phoR (Mvan 5174) - integral membrane sensor signal transduction histidine kinase [Mycobacterium vanbaalenii str. PYR-1]	75.16	470	480
fig|6666666.28482.peg.5583	R006226	(gi:145222174) phoR (Mflv 1582) - integral membrane sensor signal transduction histidine kinase [Mycobacterium gilvum str. PYR-GCK]	75.59	466	480
fig|6666666.28482.peg.5583	R006228	(gi:126437537) phoR (Mjls 4972) - integral membrane sensor signal transduction histidine kinase [Mycobacterium sp. str. JLS]	76.12	468	480
fig|6666666.28482.peg.5583	R006230	(gi:108801552) phoR (Mmcs 4589) - periplasmic sensor signal transduction histidine kinase [Mycobacterium sp. str. MCS]	76.12	468	480
fig|6666666.28482.peg.5583	R006229	(gi:119870706) phoR (Mkms 4677) - integral membrane sensor signal transduction histidine kinase [Mycobacterium sp. str. KMS]	76.12	468	480

fig|6666666.28482.peg.5583	R019263	(gi:253797704) phoR (TBMG 00773) - two component system sensor kinase phoR [Mycobacterium tuberculosis str. KZN 1435]	75.21	471	480
fig|6666666.28482.peg.5583	R013504	(gi:148821964) phoR (TBFG 10773) - two component system response sensor kinase membrane associated phoR [Mycobacterium tuberculosis str. F11]	75.21	471	480
fig|6666666.28482.peg.5583	R006219	(gi:15840173) phoR (MT0783) - sensor histidine kinase [Mycobacterium tuberculosis str. CDC1551]	75.21	471	480
fig|6666666.28482.peg.5583	R006217	(gi:15607898) phoR (Rv0758) - POSSIBLE TWO COMPONENT SYSTEM RESPONSE SENSOR KINASE MEMBRANE ASSOCIATED PHOR [Mycobacterium tuberculosis str. H37Rv]	75.21	471	480
fig|6666666.28482.peg.5583	R006218	(gi:148660535) phoR (MRA 0768) - putative two component system response sensor kinase membrane associated PhoR [Mycobacterium tuberculosis str. H37Ra]	75.21	471	480
fig|6666666.28482.peg.5583	R019262	(gi:340625777) phoR (MCAN 07621) - putative two component system response sensor kinase membrane associated PHOR [Mycobacterium canettii str. CIPT 140010059]	75	471	480
fig|6666666.28482.peg.5583	R019264	(gi:224989154) phoR (JTY 0780) - putative two component system response sensor kinase membrane associated [Mycobacterium bovis str. BCG str. Tokyo 172]	75	471	480
fig|6666666.28482.peg.5583	R006221	(gi:121636682) phoR (BCG 0810) - putative two component system response sensor kinase membrane associated phoR [Mycobacterium bovis str. BCG Pasteur 1173P2]	75	471	480
fig|6666666.28482.peg.5583	R006220	(gi:31791946) phoR (Mb0781) - POSSIBLE TWO COMPONENT SYSTEM RESPONSE SENSOR KINASE MEMBRANE ASSOCIATED PHOR [Mycobacterium bovis str. AF2122/97]	75	471	480
fig|6666666.28482.peg.5583	R019261	(gi:339630828) phoR (MAF 07700) - putative two component system response sensor kinase membrane associated PHOR [Mycobacterium africanum str. GM041182]	74.79	471	480
fig|6666666.28482.peg.5583	R006225	(gi:118468443) phoR (MSMEG 5870) - sensor histidine kinase PhoR [Mycobacterium smegmatis str. MC2 155]	73.94	471	480
fig|6666666.28482.peg.5583	R019265	(gi:333992253) phoR (JDM601 3613) - two-component system response phosphate sensor kinase PhoR [Mycobacterium sp. str. JDM601]	70.66	465	480

fig|6666666.28482.peg.5583	R019260	(gi:169627777) phoR (MAB 0674) - putative sensor histidine kinase PhoR [Mycobacterium abscessus str. ATCC 19977]	64.09	463	480
fig|6666666.28482.peg.5649	R019811	(gi:253799760) esxM (TBMG 02785) - esat-6 like protein esxK [Mycobacterium tuberculosis str. KZN 1435]	83.87	93	93
fig|6666666.28482.peg.5649	R019810	(gi:340626804) esxM (MCAN 18101) - ESAT-6 like protein ESXM [Mycobacterium canettii str. CIPT 140010059]	83.87	93	93
fig|6666666.28482.peg.5649	R019812	(gi:224990177) esxM (JTY 1808) - hypothetical protein [Mycobacterium bovis str. BCG str. Tokyo 172]	83.87	93	93
fig|6666666.28482.peg.5649	R014040	(gi:121637693) esxM (BCG 1824) - hypothetical protein [Mycobacterium bovis str. BCG Pasteur 1173P2]	83.87	93	93
fig|6666666.28482.peg.5649	R014039	(gi:31792980) esxM (Mb1820) - hypothetical protein [Mycobacterium bovis str. AF2122/97]	83.87	93	93
fig|6666666.28482.peg.5649	R019809	(gi:339631846) esxM (MAF 18140) - ESAT-6 like protein ESXM [Mycobacterium africanum str. GM041182]	83.87	93	93
fig|6666666.28482.peg.5649	R014041	(gi:118464008) esxM (MAV 2922) - hypothetical protein [Mycobacterium avium str. 104]	81.72	93	93
fig|6666666.28482.peg.5649	R019808	(gi:183982681) esxM (MMAR 2674) - EsaT-6 like protein EsxM [Mycobacterium marinum str. M]	80.22	91	93
fig|6666666.28482.peg.5649	R014042	(gi:118618466) esxM (MUL 3083) - EsaT-6 like protein EsxM [Mycobacterium ulcerans str. Agy99]	78.49	91	93
fig|6666666.28482.peg.5650	R014035	(gi:118463289) esxN (MAV 2921) - hypothetical protein [Mycobacterium avium str. 104]	93.62	94	94
fig|6666666.28482.peg.5650	R019816	(gi:253799165) esxN (TBMG 02203) - esat-6 like protein esxN [Mycobacterium tuberculosis str. KZN 1435]	92.55	94	94
fig|6666666.28482.peg.5650	R014030	(gi:57116914) esxN (Rv1793) - PUTATIVE ESAT-6 LIKE PROTEIN ESXN (ESAT-6 LIKE PROTEIN 5) [Mycobacterium tuberculosis str. H37Rv]	92.55	94	94
fig|6666666.28482.peg.5650	R014031	(gi:148661599) esxN (MRA 1806) - putative esat-6 like protein EsxN [Mycobacterium tuberculosis str. H37Ra]	92.55	94	94
fig|6666666.28482.peg.5650	R014029	(gi:148823007) esxN (TBFG 11824) - Esat-6 like protein esxN (Esat-6 like protein 5) [Mycobacterium tuberculosis str. F11]	92.55	94	94
fig|6666666.28482.peg.5650	R014032	(gi:15841262) esxN (MT1842) - hypothetical protein [Mycobacterium tuberculosis str. CDC1551]	92.55	94	94

fig|6666666.28482.peg.5650	R019815	(gi:340626805) esxN (MCAN 18111) - putative ESAT-6 like protein ESXN [Mycobacterium canettii str. CIPT 140010059]	92.55	94	94
fig|6666666.28482.peg.5650	R019817	(gi:224990178) esxN (JTY 1809) - putative EsaT-6 like protein 5 [Mycobacterium bovis str. BCG str. Tokyo 172]	92.55	94	94
fig|6666666.28482.peg.5650	R014034	(gi:121637694) esxN (BCG 1825) - putative ESAT-6 like protein 5 [Mycobacterium bovis str. BCG Pasteur 1173P2]	92.55	94	94
fig|6666666.28482.peg.5650	R014033	(gi:31792981) esxN (Mb1821) - PUTATIVE ESAT-6 LIKE PROTEIN 5 [Mycobacterium bovis str. AF2122/97]	92.55	94	94
fig|6666666.28482.peg.5650	R019814	(gi:339631847) esxN (MAF 18150) - putative ESAT-6 like protein ESXN (ESAT-6 like protein 5) [Mycobacterium africanum str. GM041182]	92.55	94	94
fig|6666666.28482.peg.5650	R014036	(gi:118618465) esxN (MUL 3082) - EsaT-6 like protein EsxN [Mycobacterium ulcerans str. Agy99]	92.55	94	94
fig|6666666.28482.peg.5650	R019813	(gi:183982682) esxN (MMAR 2675) - EsaT-6 like protein EsxN [Mycobacterium marinum str. M]	92.55	94	94
fig|6666666.28482.peg.5650	R019818	(gi:333990536) esxN (JDM601 1896) - EsaT-6 like protein EsxN [Mycobacterium sp. str. JDM601]	85.11	94	94
fig|6666666.28482.peg.5714	R018366	(gi:41409876) eccA3 (MAP3778) - hypothetical protein [Mycobacterium avium str. K-10 (subsp. paratuberculosis)]	90.11	617	617
fig|6666666.28482.peg.5714	R018367	(gi:118466988) eccA3 (MAV 4871) - ATPase AAA [Mycobacterium avium str. 104]	90.28	617	617
fig|6666666.28482.peg.5714	R019661	(gi:340625320) eccA3 (MCAN 02911) - hypothetical protein [Mycobacterium canettii str. CIPT 140010059]	86.61	617	617
fig|6666666.28482.peg.5714	R019663	(gi:224988670) eccA3 (JTY 0292) - hypothetical protein [Mycobacterium bovis str. BCG str. Tokyo 172]	86.45	617	617
fig|6666666.28482.peg.5714	R018365	(gi:121636197) eccA3 (BCG 0322) - hypothetical protein [Mycobacterium bovis str. BCG Pasteur 1173P2]	86.45	617	617
fig|6666666.28482.peg.5714	R018364	(gi:31791461) eccA3 (Mb0290) - hypothetical protein [Mycobacterium bovis str. AF2122/97]	86.45	617	617
fig|6666666.28482.peg.5714	R019660	(gi:339630357) eccA3 (MAF 02840) - hypothetical protein [Mycobacterium africanum str. GM041182]	86.45	617	617
fig|6666666.28482.peg.5714	R019662	(gi:253797209) eccA3 (TBMG 00287) - hypothetical protein [Mycobacterium tuberculosis str. KZN 1435]	86.45	617	617

fig|6666666.28482.peg.5714	R018361	(gi:15607423) eccA3 (Rv0282) - hypothetical protein [Mycobacterium tuberculosis str. H37Rv]	86.45	617	617
fig|6666666.28482.peg.5714	R018362	(gi:148660048) eccA3 (MRA 0291) - hypothetical protein [Mycobacterium tuberculosis str. H37Ra]	86.45	617	617
fig|6666666.28482.peg.5714	R018360	(gi:148821480) eccA3 (TBFG 10289) - hypothetical protein [Mycobacterium tuberculosis str. F11]	86.45	617	617
fig|6666666.28482.peg.5714	R018363	(gi:15839668) eccA3 (MT0295) - AAA family ATPase [Mycobacterium tuberculosis str. CDC1551]	86.79	610	617
fig|6666666.28482.peg.5714	R019658	(gi:183980569) eccA3 (MMAR 0541) - hypothetical protein [Mycobacterium marinum str. M]	85.48	617	617
fig|6666666.28482.peg.5714	R018369	(gi:118616918) eccA3 (MUL 1204) - hypothetical protein [Mycobacterium ulcerans str. Agy99]	85.32	617	617
fig|6666666.28482.peg.5714	R018368	(gi:15828368) eccA3 (ML2537) - hypothetical protein [Mycobacterium leprae str. TN]	83.68	616	617
fig|6666666.28482.peg.5714	R019664	(gi:221230845) eccA3 (MLBr 02537) - hypothetical protein [Mycobacterium leprae str. Br4923]	83.68	616	617
fig|6666666.28482.peg.5714	R019665	(gi:333988930) eccA3 (JDM601 0290) - hypothetical protein [Mycobacterium sp. str. JDM601]	78.61	617	617
fig|6666666.28482.peg.5714	R018375	(gi:108797350) eccA3 (Mmcs 0370) - AAA ATPase, central region [Mycobacterium sp. str. MCS]	74.75	592	617
fig|6666666.28482.peg.5714	R018374	(gi:119866435) eccA3 (Mkms 0380) - ATPase central domain-containing protein [Mycobacterium sp. str. KMS]	74.75	592	617
fig|6666666.28482.peg.5714	R018373	(gi:126432972) eccA3 (Mjls 0359) - ATPase central domain-containing protein [Mycobacterium sp. str. JLS]	74.75	592	617
fig|6666666.28482.peg.5714	R018372	(gi:120401436) eccA3 (Mvan 0411) - ATPase central domain-containing protein [Mycobacterium vanbaalenii str. PYR-1]	74.33	596	617
fig|6666666.28482.peg.5714	R018371	(gi:145220933) eccA3 (Mflv 0329) - ATPase central domain-containing protein [Mycobacterium gilvum str. PYR-GCK]	73.2	596	617
fig|6666666.28482.peg.5714	R018370	(gi:118467798) eccA3 (MSMEG 0615) - ATPase AAA [Mycobacterium smegmatis str. MC2 155]	72.8	592	617
fig|6666666.28482.peg.5714	R019659	(gi:169629320) eccA3 (MAB 2234c) - hypothetical protein [Mycobacterium abscessus str. ATCC 19977]	70.54	594	617

fig|6666666.28482.peg.5715	R018382	(gi:41409877) eccB3 (MAP3779) - hypothetical protein [Mycobacterium avium str. K-10 (subsp. paratuberculosis)]	76.81	528	532
fig|6666666.28482.peg.5715	R019674	(gi:183980570) eccB3 (MMAR 0542) - hypothetical protein [Mycobacterium marinum str. M]	76.45	529	532
fig|6666666.28482.peg.5715	R019677	(gi:340625321) eccB3 (MCAN 02921) - hypothetical protein [Mycobacterium canettii str. CIPT 140010059]	74.44	532	532
fig|6666666.28482.peg.5715	R018385	(gi:118616919) eccB3 (MUL 1205) - hypothetical protein [Mycobacterium ulcerans str. Agy99]	75.89	529	532
fig|6666666.28482.peg.5715	R019678	(gi:253797210) eccB3 (TBMG 00288) - hypothetical protein [Mycobacterium tuberculosis str. KZN 1435]	74.26	532	532
fig|6666666.28482.peg.5715	R018377	(gi:15607424) eccB3 (Rv0283) - hypothetical protein [Mycobacterium tuberculosis str. H37Rv]	74.26	532	532
fig|6666666.28482.peg.5715	R018378	(gi:148660049) eccB3 (MRA 0292) - hypothetical protein [Mycobacterium tuberculosis str. H37Ra]	74.26	532	532
fig|6666666.28482.peg.5715	R018376	(gi:148821481) eccB3 (TBFG 10290) - hypothetical protein [Mycobacterium tuberculosis str. F11]	74.26	532	532
fig|6666666.28482.peg.5715	R018379	(gi:15839669) eccB3 (MT0296) - hypothetical protein [Mycobacterium tuberculosis str. CDC1551]	74.26	532	532
fig|6666666.28482.peg.5715	R019679	(gi:224988671) eccB3 (JTY 0293) - hypothetical protein [Mycobacterium bovis str. BCG str. Tokyo 172]	74.26	532	532
fig|6666666.28482.peg.5715	R018381	(gi:121636198) eccB3 (BCG 0323) - hypothetical protein [Mycobacterium bovis str. BCG Pasteur 1173P2]	74.26	532	532
fig|6666666.28482.peg.5715	R018380	(gi:31791462) eccB3 (Mb0291) - hypothetical protein [Mycobacterium bovis str. AF2122/97]	74.26	532	532
fig|6666666.28482.peg.5715	R019676	(gi:339630358) eccB3 (MAF 02850) - putative conserved membrane protein [Mycobacterium africanum str. GM041182]	74.26	532	532
fig|6666666.28482.peg.5715	R018383	(gi:118463197) eccB3 (MAV 4870) - hypothetical protein [Mycobacterium avium str. 104]	76.74	519	532
fig|6666666.28482.peg.5715	R018384	(gi:15828367) eccB3 (ML2536) - hypothetical protein [Mycobacterium leprae str. TN]	71.69	529	532
fig|6666666.28482.peg.5715	R019680	(gi:221230844) eccB3 (MLBr 02536) - hypothetical protein [Mycobacterium leprae str. Br4923]	71.69	529	532

fig|6666666.28482.peg.5715	R019681	(gi:333988931) eccB3 (JDM601 0291) - hypothetical protein [Mycobacterium sp. str. JDM601]	66.79	513	532
fig|6666666.28482.peg.5715	R018386	(gi:118473477) eccB3 (MSMEG 0616) - hypothetical protein [Mycobacterium smegmatis str. MC2 155]	66.93	508	532
fig|6666666.28482.peg.5715	R018388	(gi:120401437) eccB3 (Mvan 0412) - hypothetical protein [Mycobacterium vanbaalenii str. PYR-1]	65.95	508	532
fig|6666666.28482.peg.5715	R018389	(gi:126432973) eccB3 (Mjls 0360) - hypothetical protein [Mycobacterium sp. str. JLS]	68.04	508	532
fig|6666666.28482.peg.5715	R018391	(gi:108797351) eccB3 (Mmcs 0371) - hypothetical protein [Mycobacterium sp. str. MCS]	68.04	508	532
fig|6666666.28482.peg.5715	R018390	(gi:119866436) eccB3 (Mkms 0381) - hypothetical protein [Mycobacterium sp. str. KMS]	68.04	508	532
fig|6666666.28482.peg.5715	R018387	(gi:145220932) eccB3 (Mflv 0328) - hypothetical protein [Mycobacterium gilvum str. PYR-GCK]	61.6	510	532
fig|6666666.28482.peg.5716	R018398	(gi:41409878) eccC3 (MAP3780) - hypothetical protein [Mycobacterium avium str. K-10 (subsp. paratuberculosis)]	87.91	1324	1326
fig|6666666.28482.peg.5716	R019685	(gi:340625322) eccC3 (MCAN 02931) - hypothetical protein [Mycobacterium canettii str. CIPT 140010059]	86.41	1326	1326
fig|6666666.28482.peg.5716	R019687	(gi:224988672) eccC3 (JTY 0294) - hypothetical protein [Mycobacterium bovis str. BCG str. Tokyo 172]	86.34	1326	1326
fig|6666666.28482.peg.5716	R018397	(gi:121636199) eccC3 (BCG 0324) - hypothetical protein [Mycobacterium bovis str. BCG Pasteur 1173P2]	86.34	1326	1326
fig|6666666.28482.peg.5716	R019686	(gi:253797211) eccC3 (TBMG 00289) - hypothetical protein [Mycobacterium tuberculosis str. KZN 1435]	86.26	1326	1326
fig|6666666.28482.peg.5716	R018392	(gi:148821482) eccC3 (TBFG 10291) - hypothetical protein [Mycobacterium tuberculosis str. F11]	86.26	1326	1326
fig|6666666.28482.peg.5716	R018395	(gi:15839670) eccC3 (MT0297) - FtsK/SpoIIIE family protein [Mycobacterium tuberculosis str. CDC1551]	86.26	1326	1326
fig|6666666.28482.peg.5716	R018396	(gi:31791463) eccC3 (Mb0292) - hypothetical protein [Mycobacterium bovis str. AF2122/97]	86.26	1326	1326
fig|6666666.28482.peg.5716	R019684	(gi:339630359) eccC3 (MAF 02860) - putative conserved membrane protein [Mycobacterium africanum str. GM041182]	86.26	1326	1326

fig|6666666.28482.peg.5716	R018393	(gi:15607425) eccC3 (Rv0284) - hypothetical protein [Mycobacterium tuberculosis str. H37Rv]	86.19	1326	1326
fig|6666666.28482.peg.5716	R018394	(gi:148660050) eccC3 (MRA 0293) - hypothetical protein [Mycobacterium tuberculosis str. H37Ra]	86.19	1326	1326
fig|6666666.28482.peg.5716	R019682	(gi:183980571) eccC3 (MMAR 0543) - hypothetical protein [Mycobacterium marinum str. M]	85.04	1326	1326
fig|6666666.28482.peg.5716	R018399	(gi:15828366) eccC3 (ML2535) - hypothetical protein [Mycobacterium leprae str. TN]	85.2	1315	1326
fig|6666666.28482.peg.5716	R019688	(gi:221230843) eccC3 (MLBr 02535) - hypothetical protein [Mycobacterium leprae str. Br4923]	85.2	1315	1326
fig|6666666.28482.peg.5716	R018400	(gi:118616920) eccC3 (MUL 1206) - hypothetical protein [Mycobacterium ulcerans str. Agy99]	84.82	1326	1326
fig|6666666.28482.peg.5716	R019689	(gi:333988932) eccC3 (JDM601 0292) - hypothetical protein [Mycobacterium sp. str. JDM601]	80.06	1315	1326
fig|6666666.28482.peg.5716	R018406	(gi:108797352) eccC3 (Mmcs 0372) - cell divisionFtsK/SpoIIIE [Mycobacterium sp. str. MCS]	79.05	1326	1326
fig|6666666.28482.peg.5716	R018405	(gi:119866437) eccC3 (Mkms 0382) - cell divisionFtsK/SpoIIIE [Mycobacterium sp. str. KMS]	79.05	1326	1326
fig|6666666.28482.peg.5716	R018404	(gi:126432974) eccC3 (Mjls 0361) - cell divisionFtsK/SpoIIIE [Mycobacterium sp. str. JLS]	79.05	1326	1326
fig|6666666.28482.peg.5716	R018401	(gi:118468237) eccC3 (MSMEG 0617) - ftsk/SpoIIIE family protein [Mycobacterium smegmatis str. MC2 155]	78.13	1317	1326
fig|6666666.28482.peg.5716	R018402	(gi:145220931) eccC3 (Mflv 0327) - cell divisionFtsK/SpoIIIE [Mycobacterium gilvum str. PYR-GCK]	76.09	1315	1326
fig|6666666.28482.peg.5716	R019683	(gi:169629318) eccC3 (MAB 2232c) - putative FtsK/SpoIIIE family protein [Mycobacterium abscessus str. ATCC 19977]	73.59	1319	1326
fig|6666666.28482.peg.5716	R018403	(gi:120401438) eccC3 (Mvan 0413) - cell divisionFtsK/SpoIIIE [Mycobacterium vanbaalenii str. PYR-1]	75.51	1316	1326
fig|6666666.28482.peg.5717	R018414	(gi:118467190) PE5 (MAV 4868) - PE family protein [Mycobacterium avium str. 104]	92.08	101	102
fig|6666666.28482.peg.5717	R018415	(gi:15828365) PE5 (ML2534) - PE-family protein [Mycobacterium leprae str. TN]	76.77	99	102

fig|6666666.28482.peg.5717	R019696	(gi:221230842) PE5 (MLBr 02534) - PE-family protein [Mycobacterium leprae str. Br4923]	76.77	99	102
fig|6666666.28482.peg.5717	R018413	(gi:41409879) PE5 (MAP3781) - hypothetical protein [Mycobacterium avium str. K-10 (subsp. paratuberculosis)]	91.09	101	102
fig|6666666.28482.peg.5717	R019694	(gi:253797212) PE5 (TBMG 00290) - PE family protein [Mycobacterium tuberculosis str. KZN 1435]	90.2	102	102
fig|6666666.28482.peg.5717	R018408	(gi:57116715) PE5 (Rv0285) - PE family protein [Mycobacterium tuberculosis str. H37Rv]	90.2	102	102
fig|6666666.28482.peg.5717	R018409	(gi:148660051) PE5 (MRA 0294) - PE family protein [Mycobacterium tuberculosis str. H37Ra]	90.2	102	102
fig|6666666.28482.peg.5717	R018407	(gi:148821483) PE5 (TBFG 10292) - PE family protein [Mycobacterium tuberculosis str. F11]	90.2	102	102
fig|6666666.28482.peg.5717	R018410	(gi:15839671) PE5 (MT0298) - PE family protein [Mycobacterium tuberculosis str. CDC1551]	90.2	102	102
fig|6666666.28482.peg.5717	R019693	(gi:340625323) PE5 (MCAN 02941) - PE family protein [Mycobacterium canettii str. CIPT 140010059]	90.2	102	102
fig|6666666.28482.peg.5717	R019695	(gi:224988673) PE5 (JTY 0295) - PE family protein [Mycobacterium bovis str. BCG str. Tokyo 172]	90.2	102	102
fig|6666666.28482.peg.5717	R018412	(gi:121636200) PE5 (BCG 0325) - PE family protein [Mycobacterium bovis str. BCG Pasteur 1173P2]	90.2	102	102
fig|6666666.28482.peg.5717	R018411	(gi:31791464) PE5 (Mb0293) - PE family protein [Mycobacterium bovis str. AF2122/97]	90.2	102	102
fig|6666666.28482.peg.5717	R019692	(gi:339630360) PE5 (MAF 02870) - PE family protein [Mycobacterium africanum str. GM041182]	90.2	102	102
fig|6666666.28482.peg.5717	R018416	(gi:118616921) PE5 (MUL 1207) - PE family protein [Mycobacterium ulcerans str. Agy99]	86.14	101	102
fig|6666666.28482.peg.5717	R019690	(gi:183980572) PE5 (MMAR 0544) - PE family protein, PE5 [Mycobacterium marinum str. M]	86.14	101	102
fig|6666666.28482.peg.5717	R018418	(gi:145220930) PE5 (Mflv 0326) - PE domain-containing protein [Mycobacterium gilvum str. PYR-GCK]	74.51	102	102
fig|6666666.28482.peg.5717	R018422	(gi:108797353) PE5 (Mmcs 0373) - PE-like protein [Mycobacterium sp. str. MCS]	74.51	102	102

fig|6666666.28482.peg.5717	R018421	(gi:119866438) PE5 (Mkms 0383) - PE domain-containing protein [Mycobacterium sp. str. KMS]	74.51	102	102
fig|6666666.28482.peg.5717	R018420	(gi:126432975) PE5 (Mjls 0362) - PE domain-containing protein [Mycobacterium sp. str. JLS]	74.51	102	102
fig|6666666.28482.peg.5717	R018417	(gi:118470806) PE5 (MSMEG 0618) - PE family protein [Mycobacterium smegmatis str. MC2 155]	76.47	102	102
fig|6666666.28482.peg.5717	R019691	(gi:169629317) PE5 (MAB 2231c) - PE family protein [Mycobacterium abscessus str. ATCC 19977]	76.47	102	102
fig|6666666.28482.peg.5717	R019697	(gi:333988933) PE5 (JDM601 0293) - PE family protein PE5 [Mycobacterium sp. str. JDM601]	84.54	97	102
fig|6666666.28482.peg.5717	R018419	(gi:120401439) PE5 (Mvan 0414) - PE domain-containing protein [Mycobacterium vanbaalenii str. PYR-1]	71.57	102	102
fig|6666666.28482.peg.5812	R005827	(gi:41407622) mgtC (MAP1524) - MgtC [Mycobacterium avium str. K-10 (subsp. paratuberculosis)]	83.76	234	235
fig|6666666.28482.peg.5812	R005828	(gi:118464815) mgtC (MAV 2903) - putative Mg2+ transporter-C (MgtC) family protein [Mycobacterium avium str. 104]	83.76	234	235
fig|6666666.28482.peg.5812	R019020	(gi:183982694) mgtC (MMAR 2687) - Mg2+ transport p-type ATPase C MgtC [Mycobacterium marinum str. M]	81.55	233	235
fig|6666666.28482.peg.5812	R019024	(gi:253799145) mgtC (TBMG 02183) - Mg2+ transport ATPase C mgtC [Mycobacterium tuberculosis str. KZN 1435]	82.98	235	235
fig|6666666.28482.peg.5812	R005822	(gi:15608948) mgtC (Rv1811) - POSSIBLE Mg2+ TRANSPORT P-TYPE ATPASE C MGTC [Mycobacterium tuberculosis str. H37Rv]	82.98	235	235
fig|6666666.28482.peg.5812	R005823	(gi:148661617) mgtC (MRA 1823) - Mg2+ transport P-type ATPase C [Mycobacterium tuberculosis str. H37Ra]	82.98	235	235
fig|6666666.28482.peg.5812	R013458	(gi:148823024) mgtC (TBFG 11841) - Mg2+ transport P-type ATPase C mgtC [Mycobacterium tuberculosis str. F11]	82.98	235	235
fig|6666666.28482.peg.5812	R005824	(gi:15841281) mgtC (MT1859) - Mg2+ transport protein [Mycobacterium tuberculosis str. CDC1551]	82.98	235	235
fig|6666666.28482.peg.5812	R019023	(gi:340626820) mgtC (MCAN 18271) - putative Mg2+ transport P-type ATPase C MGTC [Mycobacterium canettii str. CIPT 140010059]	82.98	235	235
fig|6666666.28482.peg.5812	R019025	(gi:224990197) mgtC (JTY 1829) - putative Mg2+ transport P-type ATPase C [Mycobacterium bovis str. BCG str. Tokyo 172]	82.98	235	235

fig|6666666.28482.peg.5812	R005826	(gi:121637713) mgtC (BCG 1845) - putative Mg2+ transport P-type atpase C mgtC [Mycobacterium bovis str. BCG Pasteur 1173P2]	82.98	235	235
fig|6666666.28482.peg.5812	R005825	(gi:31793000) mgtC (Mb1841) - POSSIBLE Mg2+ TRANSPORT P-TYPE ATPASE C MGTC [Mycobacterium bovis str. AF2122/97]	82.98	235	235
fig|6666666.28482.peg.5812	R005829	(gi:118618454) mgtC (MUL 3069) - Mg2+ transport p-type ATPase C MgtC [Mycobacterium ulcerans str. Agy99]	80.69	233	235
fig|6666666.28482.peg.5812	R019022	(gi:339631864) mgtC (MAF 18330) - putative Mg2+ transport P-type ATPase C MGTC [Mycobacterium africanum str. GM041182]	82.55	235	235
fig|6666666.28482.peg.5812	R019026	(gi:333990549) mgtC (JDM601 1909) - Mg2+ transport p-type ATPase C MgtC [Mycobacterium sp. str. JDM601]	75.86	232	235
fig|6666666.28482.peg.5819	R019885	(gi:253799160) eccA5 (TBMG 02198) - hypothetical protein [Mycobacterium tuberculosis str. KZN 1435]	90.07	592	595
fig|6666666.28482.peg.5819	R013980	(gi:15608935) eccA5 (Rv1798) - hypothetical protein [Mycobacterium tuberculosis str. H37Rv]	90.07	592	595
fig|6666666.28482.peg.5819	R013981	(gi:148661604) eccA5 (MRA 1811) - hypothetical protein [Mycobacterium tuberculosis str. H37Ra]	90.07	592	595
fig|6666666.28482.peg.5819	R013979	(gi:148823012) eccA5 (TBFG 11829) - hypothetical protein [Mycobacterium tuberculosis str. F11]	90.07	592	595
fig|6666666.28482.peg.5819	R013982	(gi:15841267) eccA5 (MT1847) - hypothetical protein [Mycobacterium tuberculosis str. CDC1551]	90.07	592	595
fig|6666666.28482.peg.5819	R019884	(gi:340626810) eccA5 (MCAN 18161) - hypothetical protein [Mycobacterium canettii str. CIPT 140010059]	90.07	592	595
fig|6666666.28482.peg.5819	R019886	(gi:224990183) eccA5 (JTY 1814) - hypothetical protein [Mycobacterium bovis str. BCG str. Tokyo 172]	90.07	592	595
fig|6666666.28482.peg.5819	R013984	(gi:121637699) eccA5 (BCG 1830) - hypothetical protein [Mycobacterium bovis str. BCG Pasteur 1173P2]	90.07	592	595
fig|6666666.28482.peg.5819	R013983	(gi:31792986) eccA5 (Mb1826) - hypothetical protein [Mycobacterium bovis str. AF2122/97]	90.07	592	595
fig|6666666.28482.peg.5819	R019883	(gi:339631852) eccA5 (MAF 18200) - hypothetical protein [Mycobacterium africanum str. GM041182]	89.9	592	595
fig|6666666.28482.peg.5819	R013987	(gi:15827806) eccA5 (ML1536) - hypothetical protein [Mycobacterium leprae str. TN]	88.42	594	595

fig|6666666.28482.peg.5819	R019887	(gi:221230283) eccA5 (MLBr 01536) - hypothetical protein [Mycobacterium leprae str. Br4923]	88.42	594	595
fig|6666666.28482.peg.5819	R013986	(gi:118464185) eccA5 (MAV 2916) - ATPase AAA [Mycobacterium avium str. 104]	91.28	594	595
fig|6666666.28482.peg.5819	R019882	(gi:183982687) eccA5 (MMAR 2680) - hypothetical protein [Mycobacterium marinum str. M]	88.72	592	595
fig|6666666.28482.peg.5819	R013985	(gi:41407611) eccA5 (MAP1513) - hypothetical protein [Mycobacterium avium str. K-10 (subsp. paratuberculosis)]	91.11	594	595
fig|6666666.28482.peg.5819	R013988	(gi:118618460) eccA5 (MUL 3077) - hypothetical protein [Mycobacterium ulcerans str. Agy99]	88.22	592	595
fig|6666666.28482.peg.5819	R019888	(gi:333990541) eccA5 (JDM601 1901) - hypothetical protein [Mycobacterium sp. str. JDM601]	84.06	594	595
fig|6666666.28482.peg.5820	R013996	(gi:118465331) eccE5 (MAV 2917) - hypothetical protein [Mycobacterium avium str. 104]	76.37	401	401
fig|6666666.28482.peg.5820	R013995	(gi:41407610) eccE5 (MAP1512) - hypothetical protein [Mycobacterium avium str. K-10 (subsp. paratuberculosis)]	75.87	401	401
fig|6666666.28482.peg.5820	R019840	(gi:183982686) eccE5 (MMAR 2679) - hypothetical protein [Mycobacterium marinum str. M]	69.14	401	401
fig|6666666.28482.peg.5820	R013998	(gi:118618461) eccE5 (MUL 3078) - hypothetical protein [Mycobacterium ulcerans str. Agy99]	68.4	401	401
fig|6666666.28482.peg.5820	R019843	(gi:253799161) eccE5 (TBMG 02199) - hypothetical protein [Mycobacterium tuberculosis str. KZN 1435]	71.57	401	401
fig|6666666.28482.peg.5820	R013990	(gi:15608934) eccE5 (Rv1797) - hypothetical protein [Mycobacterium tuberculosis str. H37Rv]	71.57	401	401
fig|6666666.28482.peg.5820	R013991	(gi:148661603) eccE5 (MRA 1810) - hypothetical protein [Mycobacterium tuberculosis str. H37Ra]	71.57	401	401
fig|6666666.28482.peg.5820	R013989	(gi:148823011) eccE5 (TBFG 11828) - hypothetical protein [Mycobacterium tuberculosis str. F11]	71.57	401	401
fig|6666666.28482.peg.5820	R013992	(gi:15841266) eccE5 (MT1846) - hypothetical protein [Mycobacterium tuberculosis str. CDC1551]	71.57	401	401
fig|6666666.28482.peg.5820	R019842	(gi:340626809) eccE5 (MCAN 18151) - hypothetical protein [Mycobacterium canettii str. CIPT 140010059]	71.57	401	401

fig|6666666.28482.peg.5820	R019844	(gi:224990182) eccE5 (JTY 1813) - hypothetical protein [Mycobacterium bovis str. BCG str. Tokyo 172]	71.57	401	401
fig|6666666.28482.peg.5820	R013994	(gi:121637698) eccE5 (BCG 1829) - hypothetical protein [Mycobacterium bovis str. BCG Pasteur 1173P2]	71.57	401	401
fig|6666666.28482.peg.5820	R013993	(gi:31792985) eccE5 (Mb1825) - hypothetical protein [Mycobacterium bovis str. AF2122/97]	71.57	401	401
fig|6666666.28482.peg.5820	R019841	(gi:339631851) eccE5 (MAF 18190) - hypothetical protein [Mycobacterium africanum str. GM041182]	71.57	401	401
fig|6666666.28482.peg.5820	R013997	(gi:15827807) eccE5 (ML1537) - possible secreted protein [Mycobacterium leprae str. TN]	69.06	401	401
fig|6666666.28482.peg.5820	R019845	(gi:221230284) eccE5 (MLBr 01537) - putative secreted protein [Mycobacterium leprae str. Br4923]	69.06	401	401
fig|6666666.28482.peg.5821	R014005	(gi:41407609) mycP5 (MAP1511) - hypothetical protein [Mycobacterium avium str. K-10 (subsp. paratuberculosis)]	79.38	614	614
fig|6666666.28482.peg.5821	R014006	(gi:118465602) mycP5 (MAV 2918) - subtilase family protein [Mycobacterium avium str. 104]	79.22	614	614
fig|6666666.28482.peg.5821	R014007	(gi:15827808) mycP5 (ML1538) - possible protease [Mycobacterium leprae str. TN]	73.37	592	614
fig|6666666.28482.peg.5821	R019838	(gi:221230285) mycP5 (MLBr 01538) - putative protease [Mycobacterium leprae str. Br4923]	73.37	592	614
fig|6666666.28482.peg.5821	R014008	(gi:118618462) mycP5 (MUL 3079) - proline rich membrane-anchored mycosin MycP5 [Mycobacterium ulcerans str. Agy99]	74.15	614	614
fig|6666666.28482.peg.5821	R019833	(gi:183982685) mycP5 (MMAR 2678) - proline rich membrane-anchored mycosin MycP5 [Mycobacterium marinum str. M]	74.15	614	614
fig|6666666.28482.peg.5821	R019835	(gi:340626808) mycP5 (MCAN 18141) - putative proline rich membrane-anchored myosin MYCP5 [Mycobacterium canettii str. CIPT 140010059]	71.75	614	614
fig|6666666.28482.peg.5821	R019836	(gi:253799162) mycP5 (TBMG 02200) - hypothetical protein [Mycobacterium tuberculosis str. KZN 1435]	71.75	614	614
fig|6666666.28482.peg.5821	R014000	(gi:15608933) mycP5 (Rv1796) - PROBABLE PROLINE RICH MEMBRANE-ANCHORED MYCOSIN MYCP5 (SERINE PROTEASE) (SUBTILISIN-LIKE PROTEASE) (SUBTILASE-LIKE) (MYCOSIN-5) [Mycobacterium tuberculosis str. H37Rv]	71.75	614	614

fig|6666666.28482.peg.5821	R014001	(gi:148661602) mycP5 (MRA 1809) - proline rich membrane-anchored mycosin MycP5 [Mycobacterium tuberculosis str. H37Ra]	71.75	614	614
fig|6666666.28482.peg.5821	R014002	(gi:15841265) mycP5 (MT1845) - serine protease [Mycobacterium tuberculosis str. CDC1551]	71.75	614	614
fig|6666666.28482.peg.5821	R019837	(gi:224990181) mycP5 (JTY 1812) - hypothetical pro-rich protease [Mycobacterium bovis str. BCG str. Tokyo 172]	71.75	614	614
fig|6666666.28482.peg.5821	R014004	(gi:121637697) mycP5 (BCG 1828) - hypothetical pro-rich protease [Mycobacterium bovis str. BCG Pasteur 1173P2]	71.75	614	614
fig|6666666.28482.peg.5821	R014003	(gi:31792984) mycP5 (Mb1824) - CONSERVED HYPOTHETICAL PRO-RICH PROTEASE [Mycobacterium bovis str. AF2122/97]	71.75	614	614
fig|6666666.28482.peg.5821	R019834	(gi:339631850) mycP5 (MAF 18180) - putative proline rich membrane-anchored mycosin MYCP5 (Serine protease) [Mycobacterium africanum str. GM041182]	71.75	614	614
fig|6666666.28482.peg.5821	R013999	(gi:148823010) mycP5 (TBFG 11827) - proline rich membrane-anchored mycosin mycP5 [Mycobacterium tuberculosis str. F11]	71.59	614	614
fig|6666666.28482.peg.5822	R019826	(gi:183982684) eccD5 (MMAR 2677) - hypothetical protein [Mycobacterium marinum str. M]	80.96	478	478
fig|6666666.28482.peg.5822	R014018	(gi:118618463) eccD5 (MUL 3080) - hypothetical protein [Mycobacterium ulcerans str. Agy99]	80.75	478	478
fig|6666666.28482.peg.5822	R014016	(gi:118466119) eccD5 (MAV 2919) - secretion protein Snm4 [Mycobacterium avium str. 104]	83.68	478	478
fig|6666666.28482.peg.5822	R014015	(gi:41407608) eccD5 (MAP1510) - hypothetical protein [Mycobacterium avium str. K-10 (subsp. paratuberculosis)]	83.47	478	478
fig|6666666.28482.peg.5822	R019827	(gi:339631849) eccD5 (MAF 18170) - hypothetical protein [Mycobacterium africanum str. GM041182]	79.08	478	478
fig|6666666.28482.peg.5822	R014013	(gi:31792983) eccD5 (Mb1823) - hypothetical protein [Mycobacterium bovis str. AF2122/97]	79.5	478	478
fig|6666666.28482.peg.5822	R019830	(gi:224990180) eccD5 (JTY 1811) - hypothetical membrane protein [Mycobacterium bovis str. BCG str. Tokyo 172]	79.29	478	478
fig|6666666.28482.peg.5822	R014014	(gi:121637696) eccD5 (BCG 1827) - hypothetical membrane protein [Mycobacterium bovis str. BCG Pasteur 1173P2]	79.29	478	478
fig|6666666.28482.peg.5822	R019829	(gi:253799163) eccD5 (TBMG 02201) - hypothetical protein [Mycobacterium tuberculosis str. KZN 1435]	79.29	478	478

fig|6666666.28482.peg.5822	R014010	(gi:15608932) eccD5 (Rv1795) - hypothetical protein [Mycobacterium tuberculosis str. H37Rv]	79.29	478	478
fig|6666666.28482.peg.5822	R014011	(gi:148661601) eccD5 (MRA 1808) - hypothetical protein [Mycobacterium tuberculosis str. H37Ra]	79.29	478	478
fig|6666666.28482.peg.5822	R014009	(gi:148823009) eccD5 (TBFG 11826) - hypothetical protein [Mycobacterium tuberculosis str. F11]	79.29	478	478
fig|6666666.28482.peg.5822	R014012	(gi:15841264) eccD5 (MT1844) - hypothetical protein [Mycobacterium tuberculosis str. CDC1551]	79.29	478	478
fig|6666666.28482.peg.5822	R019828	(gi:340626807) eccD5 (MCAN 18131) - hypothetical protein [Mycobacterium canettii str. CIPT 140010059]	79.29	478	478
fig|6666666.28482.peg.5822	R014017	(gi:15827809) eccD5 (ML1539) - probable membrane protein [Mycobacterium leprae str. TN]	77.82	478	478
fig|6666666.28482.peg.5822	R019831	(gi:221230286) eccD5 (MLBr 01539) - hypothetical protein [Mycobacterium leprae str. Br4923]	77.82	478	478
fig|6666666.28482.peg.5822	R019832	(gi:333990538) eccD5 (JDM601 1898) - hypothetical protein [Mycobacterium sp. str. JDM601]	62.97	478	478
fig|6666666.28482.peg.5823	R019819	(gi:183982683) MMAR2676 - hypothetical protein [Mycobacterium marinum str. M]	95.67	300	300
fig|6666666.28482.peg.5823	R019822	(gi:253799164) TBMG02202 - hypothetical protein [Mycobacterium tuberculosis str. KZN 1435]	95	300	300
fig|6666666.28482.peg.5823	R014020	(gi:15608931) Rv1794 - hypothetical protein [Mycobacterium tuberculosis str. H37Rv]	95	300	300
fig|6666666.28482.peg.5823	R014021	(gi:148661600) MRA1807 - hypothetical protein [Mycobacterium tuberculosis str. H37Ra]	95	300	300
fig|6666666.28482.peg.5823	R014019	(gi:148823008) TBFG11825 - hypothetical protein [Mycobacterium tuberculosis str. F11]	95	300	300
fig|6666666.28482.peg.5823	R014022	(gi:15841263) MT1843 - hypothetical protein [Mycobacterium tuberculosis str. CDC1551]	95	300	300
fig|6666666.28482.peg.5823	R019821	(gi:340626806) MCAN18121 - hypothetical protein [Mycobacterium canettii str. CIPT 140010059]	95	300	300
fig|6666666.28482.peg.5823	R019823	(gi:224990179) JTY1810 - hypothetical protein [Mycobacterium bovis str. BCG str. Tokyo 172]	95	300	300

fig|6666666.28482.peg.5823	R014024	(gi:121637695) BCG1826 - hypothetical protein [Mycobacterium bovis str. BCG Pasteur 1173P2]	95	300	300
fig|6666666.28482.peg.5823	R014023	(gi:31792982) Mb1822 - hypothetical protein [Mycobacterium bovis str. AF2122/97]	95	300	300
fig|6666666.28482.peg.5823	R019820	(gi:339631848) MAF18160 - hypothetical protein [Mycobacterium africanum str. GM041182]	95	300	300
fig|6666666.28482.peg.5823	R014028	(gi:118618464) MUL3081 - hypothetical protein [Mycobacterium ulcerans str. Agy99]	95.33	300	300
fig|6666666.28482.peg.5823	R014025	(gi:41407607) MAP1509 - hypothetical protein [Mycobacterium avium str. K-10 (subsp. paratuberculosis)]	95	300	300
fig|6666666.28482.peg.5823	R014026	(gi:118462612) MAV2920 - hypothetical protein [Mycobacterium avium str. 104]	95	300	300
fig|6666666.28482.peg.5823	R014027	(gi:15827810) ML1540 - hypothetical protein [Mycobacterium leprae str. TN]	88.33	300	300
fig|6666666.28482.peg.5823	R019824	(gi:221230287) MLBr01540 - hypothetical protein [Mycobacterium leprae str. Br4923]	88.33	300	300
fig|6666666.28482.peg.5823	R019825	(gi:333990537) JDM6011897 - hypothetical protein [Mycobacterium sp. str. JDM601]	87.84	296	300
fig|6666666.28482.peg.5826	R006111	(gi:118620032) sodA (MUL 5018) - superoxide dismutase [fe] SodA [Mycobacterium ulcerans str. Agy99]	92.27	207	224
fig|6666666.28482.peg.5826	R019192	(gi:183985365) sodA (MMAR 5396) - superoxide dismutase [Fe] SodA [Mycobacterium marinum str. M]	92.27	207	224
fig|6666666.28482.peg.5826	R019196	(gi:253800897) sodA (TBMG 03895) - superoxide dismutase [Fe] sodA [Mycobacterium tuberculosis str. KZN 1435]	92.27	207	224
fig|6666666.28482.peg.5826	R006103	(gi:15610982) sodA (Rv3846) - SUPEROXIDE DISMUTASE [FE] SODA [Mycobacterium tuberculosis str. H37Rv]	92.27	207	224
fig|6666666.28482.peg.5826	R006104	(gi:148663714) sodA (MRA 3886) - superoxide dismutase [Mycobacterium tuberculosis str. H37Ra]	92.27	207	224
fig|6666666.28482.peg.5826	R013481	(gi:148825055) sodA (TBFG 13882) - superoxide dismutase [Fe] sodA [Mycobacterium tuberculosis str. F11]	92.27	207	224
fig|6666666.28482.peg.5826	R006105	(gi:15843476) sodA (MT3960) - superoxide dismutase [Mycobacterium tuberculosis str. CDC1551]	92.27	207	224

fig|6666666.28482.peg.5826	R019195	(gi:340628817) sodA (MCAN 38671) - superoxide dismutase [Fe] SODA [Mycobacterium canettii str. CIPT 140010059]	92.27	207	224
fig|6666666.28482.peg.5826	R019194	(gi:339633840) sodA (MAF 38610) - superoxide dismutase [FE] SODA [Mycobacterium africanum str. GM041182]	92.27	207	224
fig|6666666.28482.peg.5826	R019197	(gi:224992259) sodA (JTY 3911) - Fe-superoxide dismutase [Mycobacterium bovis str. BCG str. Tokyo 172]	91.79	207	224
fig|6666666.28482.peg.5826	R006107	(gi:121639764) sodA (BCG 3909) - Superoxide dismutase [fe] sodA [Mycobacterium bovis str. BCG Pasteur 1173P2]	91.79	207	224
fig|6666666.28482.peg.5826	R006106	(gi:31795020) sodA (Mb3876) - SUPEROXIDE DISMUTASE [FE] SODA [Mycobacterium bovis str. AF2122/97]	91.79	207	224
fig|6666666.28482.peg.5826	R006109	(gi:118464258) sodA (MAV 0182) - [Mn]-superoxide dismutase [Mycobacterium avium str. 104]	82.13	207	224
fig|6666666.28482.peg.5826	R006108	(gi:41406285) sodA (MAP0187c) - SodA [Mycobacterium avium str. K-10 (subsp. paratuberculosis)]	81.64	207	224
fig|6666666.28482.peg.5826	R019193	(gi:169627223) sodA (MAB 0118c) - superoxide dismutase (Mn) [Mycobacterium abscessus str. ATCC 19977]	81.16	207	224
fig|6666666.28482.peg.5826	R006112	(gi:118469767) sodA (MSMEG 6427) - [Mn] superoxide dismutase [Mycobacterium smegmatis str. MC2 155]	80.19	207	224
fig|6666666.28482.peg.5826	R019199	(gi:333992798) sodA (JDM601 4158) - superoxide dismutase [Mycobacterium sp. str. JDM601]	81.95	205	224
fig|6666666.28482.peg.5826	R006115	(gi:108802007) sodA (Mmcs 5044) - Superoxide dismutase [Mycobacterium sp. str. MCS]	79.23	207	224
fig|6666666.28482.peg.5826	R006114	(gi:119871159) sodA (Mkms 5132) - Superoxide dismutase [Mycobacterium sp. str. KMS]	79.23	207	224
fig|6666666.28482.peg.5826	R006113	(gi:126437986) sodA (Mjls 5423) - Superoxide dismutase [Mycobacterium sp. str. JLS]	79.23	207	224
fig|6666666.28482.peg.5826	R006110	(gi:15826917) sodA (ML0072) - superoxide dismutase [Mycobacterium leprae str. TN]	79.51	205	224
fig|6666666.28482.peg.5826	R019198	(gi:221229395) sodA (MLBr 00072) - superoxide dismutase [Mycobacterium leprae str. Br4923]	79.51	205	224
fig|6666666.28482.peg.5829	R018259	(gi:118473945) espR (MSMEG 6431) - hypothetical protein [Mycobacterium smegmatis str. MC2 155]	84.89	139	160

fig|6666666.28482.peg.5829	R018257	(gi:15826914) espR (ML0069) - hypothetical protein [Mycobacterium leprae str. TN]	89.31	131	160
fig|6666666.28482.peg.5829	R019596	(gi:221229392) espR (MLBr 00069) - hypothetical protein [Mycobacterium leprae str. Br4923]	89.31	131	160
fig|6666666.28482.peg.5829	R018258	(gi:118620035) espR (MUL 5021) - hypothetical protein [Mycobacterium ulcerans str. Agy99]	87.88	132	160
fig|6666666.28482.peg.5829	R019590	(gi:183985368) espR (MMAR 5399) - hypothetical protein [Mycobacterium marinum str. M]	87.88	132	160
fig|6666666.28482.peg.5829	R018255	(gi:41406283) espR (MAP0185c) - hypothetical protein [Mycobacterium avium str. K-10 (subsp. paratuberculosis)]	88.64	132	160
fig|6666666.28482.peg.5829	R018256	(gi:118463431) espR (MAV 0180) - hypothetical protein [Mycobacterium avium str. 104]	88.64	132	160
fig|6666666.28482.peg.5829	R018264	(gi:108802010) espR (Mmcs 5047) - hypothetical protein [Mycobacterium sp. str. MCS]	87.79	131	160
fig|6666666.28482.peg.5829	R018263	(gi:119871162) espR (Mkms 5135) - hypothetical protein [Mycobacterium sp. str. KMS]	87.79	131	160
fig|6666666.28482.peg.5829	R018262	(gi:126437989) espR (Mjls 5426) - hypothetical protein [Mycobacterium sp. str. JLS]	87.79	131	160
fig|6666666.28482.peg.5829	R019594	(gi:253800900) espR (TBMG 03898) - hypothetical protein [Mycobacterium tuberculosis str. KZN 1435]	87.12	132	160
fig|6666666.28482.peg.5829	R018250	(gi:15610985) espR (Rv3849) - hypothetical protein [Mycobacterium tuberculosis str. H37Rv]	87.12	132	160
fig|6666666.28482.peg.5829	R018251	(gi:148663717) espR (MRA 3889) - hypothetical protein [Mycobacterium tuberculosis str. H37Ra]	87.12	132	160
fig|6666666.28482.peg.5829	R018249	(gi:148825058) espR (TBFG 13885) - hypothetical protein [Mycobacterium tuberculosis str. F11]	87.12	132	160
fig|6666666.28482.peg.5829	R019593	(gi:340628821) espR (MCAN 38711) - hypothetical protein [Mycobacterium canettii str. CIPT 140010059]	87.12	132	160
fig|6666666.28482.peg.5829	R019595	(gi:224992262) espR (JTY 3914) - hypothetical protein [Mycobacterium bovis str. BCG str. Tokyo 172]	87.12	132	160
fig|6666666.28482.peg.5829	R018254	(gi:121639767) espR (BCG 3912) - hypothetical protein [Mycobacterium bovis str. BCG Pasteur 1173P2]	87.12	132	160

fig|6666666.28482.peg.5829	R018253	(gi:31795023) espR (Mb3879) - hypothetical protein [Mycobacterium bovis str. AF2122/97]	87.12	132	160
fig|6666666.28482.peg.5829	R019592	(gi:339633843) espR (MAF 38640) - hypothetical protein [Mycobacterium africanum str. GM041182]	87.12	132	160
fig|6666666.28482.peg.5829	R018260	(gi:145221725) espR (Mflv 1133) - hypothetical protein [Mycobacterium gilvum str. PYR-GCK]	85.5	131	160
fig|6666666.28482.peg.5829	R018252	(gi:15843480) espR (MT3964) - hypothetical protein [Mycobacterium tuberculosis str. CDC1551]	86.36	132	160
fig|6666666.28482.peg.5829	R019597	(gi:333992801) espR (JDM601 4161) - hypothetical protein [Mycobacterium sp. str. JDM601]	83.33	132	160
fig|6666666.28482.peg.5829	R019591	(gi:169627220) espR (MAB 0115c) - hypothetical protein [Mycobacterium abscessus str. ATCC 19977]	81.82	132	160
fig|6666666.28482.peg.5865	R012532	(gi:42560712) tuf (MSC 0160) - translation elongation factor Tu [Mycoplasma mycoides str. PG1]	70.53	396	396
fig|6666666.28482.peg.5865	R012537	(gi:83319675) tuf (MCAP 0154) - translation elongation factor Tu [Mycoplasma capricolum str. ATCC 27343]	70.53	396	396
fig|6666666.28482.peg.5865	R012535	(gi:71894677) tuf (MS53 0667) - Elongation factor Tu [Mycoplasma synoviae str. 53]	70.03	396	396
fig|6666666.28482.peg.5865	R012533	(gi:26553484) tuf (MYPE320) - elongation factor Tu [Mycoplasma penetrans str. HF-2]	68.26	396	396
fig|6666666.28482.peg.5865	R012534	(gi:15828876) tuf (MYPU 4050) - elongation factor Tu [Mycoplasma pulmonis str. UAB CTIP]	67.59	396	396
fig|6666666.28482.peg.5865	R012527	(gi:31544430) tuf (MGA 1033) - elongation factor Tu [Mycoplasma gallisepticum str. R]	67.76	396	396
fig|6666666.28482.peg.5865	R012525	(gi:13508404) tuf (MPN665) - elongation factor Tu [Mycoplasma pneumoniae str. M129]	65.49	396	396
fig|6666666.28482.peg.5865	R012536	(gi:148377586) tuf (MAG736) - Elongation factor Tu (EF-Tu) [Mycoplasma agalactiae str. PG2]	66.42	396	396
fig|6666666.28482.peg.5865	R012531	(gi:47459059) tuf (MMOB2240) - elongation factor Tu [Mycoplasma mobile str. 163K]	66.33	396	396
fig|6666666.28482.peg.5865	R012529	(gi:72080854) tuf (MHP7448 0523) - elongation factor Tu [Mycoplasma hyopneumoniae str. 7448]	65.99	395	396

fig|6666666.28482.peg.5865	R012528	(gi:54020567) tuf (mhp540) - elongation factor Tu [Mycoplasma hyopneumoniae str. 232]	65.99	395	396
fig|6666666.28482.peg.5865	R012530	(gi:71893875) tuf (MHJ 0524) - elongation factor Tu [Mycoplasma hyopneumoniae str. J]	65.74	395	396
fig|6666666.28482.peg.5865	R012526	(gi:12045310) tuf (MG 451) - elongation factor Tu [Mycoplasma genitalium str. G37]	64.48	396	396
fig|6666666.28482.peg.5887	R019285	(gi:340625993) mprA (MCAN 09861) - persistence regulator MRPA [Mycobacterium canettii str. CIPT 140010059]	96.96	230	230
fig|6666666.28482.peg.5887	R019287	(gi:224989380) mprA (JTY 1008) - persistence regulator MprA [Mycobacterium bovis str. BCG str. Tokyo 172]	96.96	230	230
fig|6666666.28482.peg.5887	R006265	(gi:121636908) mprA (BCG 1036) - Mycobacterial persistence regulator mrpA [Mycobacterium bovis str. BCG Pasteur 1173P2]	96.96	230	230
fig|6666666.28482.peg.5887	R006264	(gi:31792171) mprA (Mb1007) - MYCOBACTERIAL PERSISTENCE REGULATOR MRPA (TWO COMPONENT RESPONSE TRANSCRIPTIONAL REGULATORY PROTEIN) [Mycobacterium bovis str. AF2122/97]	96.96	230	230
fig|6666666.28482.peg.5887	R019284	(gi:339631044) mprA (MAF 09910) - mycobacterial persistence regulator MRPA (two component response transcriptional regulatory protein) [Mycobacterium africanum str. GM041182]	96.96	230	230
fig|6666666.28482.peg.5887	R006266	(gi:41407014) mprA (MAP0916) - hypothetical protein [Mycobacterium avium str. K-10 (subsp. paratuberculosis)]	97.81	228	230
fig|6666666.28482.peg.5887	R006267	(gi:118464125) mprA (MAV 1094) - mycobacterial persistence regulator mrpa [Mycobacterium avium str. 104]	97.81	228	230
fig|6666666.28482.peg.5887	R019286	(gi:253799991) mprA (TBMG 03008) - persistence regulator MprA [Mycobacterium tuberculosis str. KZN 1435]	96.52	230	230
fig|6666666.28482.peg.5887	R006261	(gi:15608121) mprA (Rv0981) - MYCOBACTERIAL PERSISTENCE REGULATOR MRPA (TWO COMPONENT RESPONSE TRANSCRIPTIONAL REGULATORY PROTEIN) [Mycobacterium tuberculosis str. H37Rv]	96.52	230	230
fig|6666666.28482.peg.5887	R006262	(gi:148660762) mprA (MRA 0988) - mycobacterial persistence regulator MrpA [Mycobacterium tuberculosis str. H37Ra]	96.52	230	230
fig|6666666.28482.peg.5887	R013507	(gi:148822190) mprA (TBFG 10999) - mycobacterial persistence regulator mprA (two component response transcriptional regulatory protein) [Mycobacterium tuberculosis str. F11]	96.52	230	230

fig|6666666.28482.peg.5887	R006263	(gi:15840406) mprA (MT1009) - DNA-binding response regulator [Mycobacterium tuberculosis str. CDC1551]	96.52	230	230
fig|6666666.28482.peg.5887	R019282	(gi:183984499) mprA (MMAR 4529) - two component response transcriptional regulatory protein MprA [Mycobacterium marinum str. M]	95.69	230	230
fig|6666666.28482.peg.5887	R006269	(gi:118619772) mprA (MUL 4701) - mycobacterial persistence regulator MprA [Mycobacterium ulcerans str. Agy99]	95.26	230	230
fig|6666666.28482.peg.5887	R006268	(gi:15826987) mprA (ML0174) - putative two-component response regulator [Mycobacterium leprae str. TN]	94.74	228	230
fig|6666666.28482.peg.5887	R019288	(gi:221229465) mprA (MLBr 00174) - putative two-component response regulator [Mycobacterium leprae str. Br4923]	94.74	228	230
fig|6666666.28482.peg.5887	R006272	(gi:120405794) mprA (Mvan 4844) - two component transcriptional regulator, winged helix family [Mycobacterium vanbaalenii str. PYR-1]	94.37	230	230
fig|6666666.28482.peg.5887	R006270	(gi:118473019) mprA (MSMEG 5488) - DNA-binding response regulator [Mycobacterium smegmatis str. MC2 155]	91.74	230	230
fig|6666666.28482.peg.5887	R006275	(gi:108801265) mprA (Mmcs 4301) - two component transcriptional regulator, winged helix family [Mycobacterium sp. str. MCS]	91.74	230	230
fig|6666666.28482.peg.5887	R006274	(gi:119870416) mprA (Mkms 4387) - two component transcriptional regulator, winged helix family [Mycobacterium sp. str. KMS]	91.74	230	230
fig|6666666.28482.peg.5887	R006273	(gi:126437246) mprA (Mjls 4681) - two component transcriptional regulator, winged helix family [Mycobacterium sp. str. JLS]	91.74	230	230
fig|6666666.28482.peg.5887	R006271	(gi:145222477) mprA (Mflv 1887) - two component transcriptional regulator, winged helix family [Mycobacterium gilvum str. PYR-GCK]	92.64	230	230
fig|6666666.28482.peg.5887	R019289	(gi:333989572) mprA (JDM601 0932) - two component response transcriptional regulatory protein MprA [Mycobacterium sp. str. JDM601]	89.57	230	230
fig|6666666.28482.peg.5887	R019283	(gi:169628171) mprA (MAB 1076) - two component response transcriptional regulatory protein MprA [Mycobacterium abscessus str. ATCC 19977]	88.26	230	230
fig|6666666.28482.peg.5888	R006283	(gi:15826988) mprB (ML0175) - putative two-component system sensor kinase [Mycobacterium leprae str. TN]	85.14	524	524
fig|6666666.28482.peg.5888	R019296	(gi:221229466) mprB (MLBr 00175) - putative two-component system sensor kinase [Mycobacterium leprae str. Br4923]	85.14	524	524
fig|6666666.28482.peg.5888	R006281	(gi:41407015) mprB (MAP0917) - hypothetical protein [Mycobacterium avium str. K-10 (subsp. paratuberculosis)]	85.33	516	524

fig|6666666.28482.peg.5888	R006284	(gi:118619771) mprB (MUL 4700) - two component sensor kinase MprB [Mycobacterium ulcerans str. Agy99]	84.73	524	524
fig|6666666.28482.peg.5888	R019290	(gi:183984498) mprB (MMAR 4528) - two-component sensor kinase MprB [Mycobacterium marinum str. M]	84.73	524	524
fig|6666666.28482.peg.5888	R006282	(gi:118464607) mprB (MAV 1095) - sensor histidine kinase [Mycobacterium avium str. 104]	85.49	501	524
fig|6666666.28482.peg.5888	R019293	(gi:340625994) mprB (MCAN 09871) - putative two component sensor kinase MPRB [Mycobacterium canettii str. CIPT 140010059]	83.17	523	524
fig|6666666.28482.peg.5888	R019295	(gi:224989381) mprB (JTY 1009) - putative two component sensor kinase [Mycobacterium bovis str. BCG str. Tokyo 172]	83.17	523	524
fig|6666666.28482.peg.5888	R006280	(gi:121636909) mprB (BCG 1037) - putative two component sensor kinase mprB [Mycobacterium bovis str. BCG Pasteur 1173P2]	83.17	523	524
fig|6666666.28482.peg.5888	R006279	(gi:31792172) mprB (Mb1008) - PROBABLE TWO COMPONENT SENSOR KINASE MPRB [Mycobacterium bovis str. AF2122/97]	83.17	523	524
fig|6666666.28482.peg.5888	R019292	(gi:339631045) mprB (MAF 09920) - putative two component sensor kinase MPRB [Mycobacterium africanum str. GM041182]	83.17	523	524
fig|6666666.28482.peg.5888	R019294	(gi:253799990) mprB (TBMG 03007) - two component system sensor kinase mprB [Mycobacterium tuberculosis str. KZN 1435]	83.17	523	524
fig|6666666.28482.peg.5888	R006276	(gi:15608122) mprB (Rv0982) - PROBABLE TWO COMPONENT SENSOR KINASE MPRB [Mycobacterium tuberculosis str. H37Rv]	83.17	523	524
fig|6666666.28482.peg.5888	R006277	(gi:148660763) mprB (MRA 0989) - putative two component sensor kinase MprB [Mycobacterium tuberculosis str. H37Ra]	83.17	523	524
fig|6666666.28482.peg.5888	R013508	(gi:148822191) mprB (TBFG 11000) - two component system sensor kinase mprB [Mycobacterium tuberculosis str. F11]	83.17	523	524
fig|6666666.28482.peg.5888	R006278	(gi:15840407) mprB (MT1010) - sensor histidine kinase [Mycobacterium tuberculosis str. CDC1551]	83.17	523	524
fig|6666666.28482.peg.5888	R006288	(gi:126437245) mprB (Mjls 4680) - integral membrane sensor signal transduction histidine kinase [Mycobacterium sp. str. JLS]	82.97	464	524
fig|6666666.28482.peg.5888	R006290	(gi:108801264) mprB (Mmcs 4300) - periplasmic sensor signal transduction histidine kinase [Mycobacterium sp. str. MCS]	83.59	457	524
fig|6666666.28482.peg.5888	R006289	(gi:119870415) mprB (Mkms 4386) - integral membrane sensor signal transduction histidine kinase [Mycobacterium sp. str. KMS]	83.59	457	524

fig|6666666.28482.peg.5888	R006287	(gi:120405793) mprB (Mvan 4843) - integral membrane sensor signal transduction histidine kinase [Mycobacterium vanbaalenii str. PYR-1]	75.94	453	524
fig|6666666.28482.peg.5888	R006286	(gi:145222478) mprB (Mflv 1888) - integral membrane sensor signal transduction histidine kinase [Mycobacterium gilvum str. PYR-GCK]	75.61	451	524
fig|6666666.28482.peg.5888	R019297	(gi:333989573) mprB (JDM601 0933) - two-component sensor kinase MprB [Mycobacterium sp. str. JDM601]	77.68	468	524
fig|6666666.28482.peg.5888	R006285	(gi:118473327) mprB (MSMEG 5487) - sensor histidine kinase [Mycobacterium smegmatis str. MC2 155]	78.49	465	524
fig|6666666.28482.peg.5888	R019291	(gi:169628172) mprB (MAB 1077) - two component sensor kinase MprB [Mycobacterium abscessus str. ATCC 19977]	65.3	522	524
fig|6666666.28482.peg.5910	R018659	(gi:118618471) PE18 (MUL 3088) - PE family protein [Mycobacterium ulcerans str. Agy99]	65.52	87	99
fig|6666666.28482.peg.5910	R019862	(gi:183982677) PE18 (MMAR 2670) - PE family protein, PE19 1 [Mycobacterium marinum str. M]	65.52	87	99
fig|6666666.28482.peg.5910	R019877	(gi:183982680) PE19 (MMAR 2673) - PE family protein, PE19 [Mycobacterium marinum str. M]	71.26	87	99
fig|6666666.28482.peg.5910	R019865	(gi:253799172) PE18 (TBMG 02209) - PE family protein [Mycobacterium tuberculosis str. KZN 1435]	68.97	87	99
fig|6666666.28482.peg.5910	R018654	(gi:57116910) PE18 (Rv1788) - PE family protein [Mycobacterium tuberculosis str. H37Rv]	68.97	87	99
fig|6666666.28482.peg.5910	R018655	(gi:148661595) PE18 (MRA 1802) - PE family protein [Mycobacterium tuberculosis str. H37Ra]	68.97	87	99
fig|6666666.28482.peg.5910	R018653	(gi:148823003) PE18 (TBFG 11818) - PE family protein [Mycobacterium tuberculosis str. F11]	68.97	87	99
fig|6666666.28482.peg.5910	R018656	(gi:15841256) PE18 (MT1837) - PE family protein [Mycobacterium tuberculosis str. CDC1551]	68.97	87	99
fig|6666666.28482.peg.5910	R019864	(gi:340626800) PE18 (MCAN 18061) - PE family protein [Mycobacterium canettii str. CIPT 140010059]	68.97	87	99
fig|6666666.28482.peg.5910	R019866	(gi:224990173) PE18 (JTY 1804) - PE family protein [Mycobacterium bovis str. BCG str. Tokyo 172]	68.97	87	99
fig|6666666.28482.peg.5910	R018658	(gi:121637689) PE18 (BCG 1820) - PE family protein [Mycobacterium bovis str. BCG Pasteur 1173P2]	68.97	87	99

fig|6666666.28482.peg.5910	R018657	(gi:31792976) PE18 (Mb1816) - PE family protein [Mycobacterium bovis str. AF2122/97]	68.97	87	99
fig|6666666.28482.peg.5910	R019863	(gi:339631842) PE18 (MAF 18100) - PE family protein [Mycobacterium africanum str. GM041182]	68.97	87	99
fig|6666666.28482.peg.5910	R018680	(gi:41407605) PE19 (MAP1507) - hypothetical protein [Mycobacterium avium str. K-10 (subsp. paratuberculosis)]	67.82	87	99
fig|6666666.28482.peg.5910	R018675	(gi:57116913) PE19 (Rv1791) - PE family protein [Mycobacterium tuberculosis str. H37Rv]	68.97	87	99
fig|6666666.28482.peg.5910	R018676	(gi:148661598) PE19 (MRA 1805) - PE family protein [Mycobacterium tuberculosis str. H37Ra]	68.97	87	99
fig|6666666.28482.peg.5910	R019879	(gi:340626803) PE19 (MCAN 18091) - PE family protein [Mycobacterium canettii str. CIPT 140010059]	68.97	87	99
fig|6666666.28482.peg.5910	R019881	(gi:224990176) PE19 (JTY 1807) - PE family protein [Mycobacterium bovis str. BCG str. Tokyo 172]	68.97	87	99
fig|6666666.28482.peg.5910	R018679	(gi:121637692) PE19 (BCG 1823) - PE family protein [Mycobacterium bovis str. BCG Pasteur 1173P2]	68.97	87	99
fig|6666666.28482.peg.5910	R018678	(gi:31792979) PE19 (Mb1819) - PE family protein [Mycobacterium bovis str. AF2122/97]	68.97	87	99
fig|6666666.28482.peg.5910	R019878	(gi:339631845) PE19 (MAF 18130) - PE family protein [Mycobacterium africanum str. GM041182]	68.97	87	99
fig|6666666.28482.peg.5910	R019880	(gi:253799168) PE19 (TBMG 02205) - PE family protein [Mycobacterium tuberculosis str. KZN 1435]	68.97	87	99
fig|6666666.28482.peg.5910	R018674	(gi:148823006) PE19 (TBFG 11821) - PE family protein [Mycobacterium tuberculosis str. F11]	68.97	87	99
fig|6666666.28482.peg.5910	R018677	(gi:15841261) PE19 (MT1840) - PE family protein [Mycobacterium tuberculosis str. CDC1551]	68.97	87	99
fig|6666666.28482.peg.5910	R019867	(gi:333990534) PE18 (JDM601 1894) - PE-PGRS family protein [Mycobacterium sp. str. JDM601]	67.82	87	99
fig|6666666.28482.peg.5912	R014041	(gi:118464008) esxM (MAV 2922) - hypothetical protein [Mycobacterium avium str. 104]	96.94	98	98
fig|6666666.28482.peg.5912	R019810	(gi:340626804) esxM (MCAN 18101) - ESAT-6 like protein ESXM [Mycobacterium canettii str. CIPT 140010059]	87.76	98	98

fig|6666666.28482.peg.5912	R019812	(gi:224990177) esxM (JTY 1808) - hypothetical protein [Mycobacterium bovis str. BCG str. Tokyo 172]	87.76	98	98
fig|6666666.28482.peg.5912	R014040	(gi:121637693) esxM (BCG 1824) - hypothetical protein [Mycobacterium bovis str. BCG Pasteur 1173P2]	87.76	98	98
fig|6666666.28482.peg.5912	R014039	(gi:31792980) esxM (Mb1820) - hypothetical protein [Mycobacterium bovis str. AF2122/97]	87.76	98	98
fig|6666666.28482.peg.5912	R019809	(gi:339631846) esxM (MAF 18140) - ESAT-6 like protein ESXM [Mycobacterium africanum str. GM041182]	87.76	98	98
fig|6666666.28482.peg.5912	R019811	(gi:253799760) esxM (TBMG 02785) - esat-6 like protein esxK [Mycobacterium tuberculosis str. KZN 1435]	86.73	98	98
fig|6666666.28482.peg.5912	R019808	(gi:183982681) esxM (MMAR 2674) - EsaT-6 like protein EsxM [Mycobacterium marinum str. M]	86.46	96	98
fig|6666666.28482.peg.5912	R014042	(gi:118618466) esxM (MUL 3083) - EsaT-6 like protein EsxM [Mycobacterium ulcerans str. Agy99]	84.69	96	98
fig|6666666.28482.peg.5962	R014070	(gi:118462835) eccB5 (MAV 2933) - hypothetical protein [Mycobacterium avium str. 104]	87.94	506	506
fig|6666666.28482.peg.5962	R014069	(gi:41407599) eccB5 (MAP1501) - hypothetical protein [Mycobacterium avium str. K-10 (subsp. paratuberculosis)]	87.75	506	506
fig|6666666.28482.peg.5962	R019795	(gi:253799177) eccB5 (TBMG 02214) - hypothetical protein [Mycobacterium tuberculosis str. KZN 1435]	85.18	506	506
fig|6666666.28482.peg.5962	R014064	(gi:15608920) eccB5 (Rv1782) - PROBABLE CONSERVED MEMBRANE PROTEIN [Mycobacterium tuberculosis str. H37Rv]	85.18	506	506
fig|6666666.28482.peg.5962	R014065	(gi:148661590) eccB5 (MRA 1797) - putative conserved membrane protein [Mycobacterium tuberculosis str. H37Ra]	85.18	506	506
fig|6666666.28482.peg.5962	R014063	(gi:148822998) eccB5 (TBFG 11813) - hypothetical protein [Mycobacterium tuberculosis str. F11]	85.18	506	506
fig|6666666.28482.peg.5962	R014066	(gi:15841251) eccB5 (MT1832) - hypothetical protein [Mycobacterium tuberculosis str. CDC1551]	85.18	506	506
fig|6666666.28482.peg.5962	R019796	(gi:224990168) eccB5 (JTY 1799) - hypothetical protein [Mycobacterium bovis str. BCG str. Tokyo 172]	84.98	506	506
fig|6666666.28482.peg.5962	R014068	(gi:121637684) eccB5 (BCG 1815) - hypothetical protein [Mycobacterium bovis str. BCG Pasteur 1173P2]	84.98	506	506

fig|6666666.28482.peg.5962	R014067	(gi:31792971) eccB5 (Mb1811) - PROBABLE CONSERVED MEMBRANE PROTEIN [Mycobacterium bovis str. AF2122/97]	84.98	506	506
fig|6666666.28482.peg.5962	R019793	(gi:339631836) eccB5 (MAF 18040) - putative conserved membrane protein [Mycobacterium africanum str. GM041182]	84.98	506	506
fig|6666666.28482.peg.5962	R019794	(gi:340626794) eccB5 (MCAN 18001) - hypothetical protein [Mycobacterium canettii str. CIPT 140010059]	84.98	506	506
fig|6666666.28482.peg.5962	R019792	(gi:183982671) eccB5 (MMAR 2664) - hypothetical protein [Mycobacterium marinum str. M]	83.63	506	506
fig|6666666.28482.peg.5962	R014072	(gi:118618476) eccB5 (MUL 3093) - hypothetical protein [Mycobacterium ulcerans str. Agy99]	83.04	506	506
fig|6666666.28482.peg.5962	R014071	(gi:15827812) eccB5 (ML1544) - hypothetical protein [Mycobacterium leprae str. TN]	81.23	506	506
fig|6666666.28482.peg.5962	R019797	(gi:221230289) eccB5 (MLBr 01544) - hypothetical protein [Mycobacterium leprae str. Br4923]	81.23	506	506
fig|6666666.28482.peg.5962	R019798	(gi:333990532) eccB5 (JDM601 1892) - hypothetical protein [Mycobacterium sp. str. JDM601]	71.13	484	506
fig|6666666.28482.peg.5963	R014059	(gi:41407600) eccCa5 (MAP1502) - hypothetical protein [Mycobacterium avium str. K-10 (subsp. paratuberculosis)]	91.75	1389	1389
fig|6666666.28482.peg.5963	R014049	(gi:41407600) eccCb5 (MAP1502) - hypothetical protein [Mycobacterium avium str. K-10 (subsp. paratuberculosis)]	91.75	1389	1389
fig|6666666.28482.peg.5963	R014060	(gi:118464740) eccCa5 (MAV 2932) - ftsk/spoiiie family protein [Mycobacterium avium str. 104]	91.59	1375	1389
fig|6666666.28482.peg.5963	R014050	(gi:118464740) eccCb5 (MAV 2932) - ftsk/spoiiie family protein [Mycobacterium avium str. 104]	91.59	1375	1389
fig|6666666.28482.peg.5963	R019804	(gi:253799176) eccCb5 (TBMG 02213) - FtsK/SpoIIIE family protein [Mycobacterium tuberculosis str. KZN 1435]	90.09	1389	1389
fig|6666666.28482.peg.5963	R014055	(gi:148661591) eccCa5 (MRA 1798) - FtsK/SpoIIIE family protein [Mycobacterium tuberculosis str. H37Ra]	90.09	1389	1389
fig|6666666.28482.peg.5963	R014045	(gi:148661591) eccCb5 (MRA 1798) - FtsK/SpoIIIE family protein [Mycobacterium tuberculosis str. H37Ra]	90.09	1389	1389
fig|6666666.28482.peg.5963	R014053	(gi:148822999) eccCa5 (TBFG 11814) - hypothetical protein [Mycobacterium tuberculosis str. F11]	90.09	1389	1389

fig|6666666.28482.peg.5963	R014043	(gi:148822999) eccCb5 (TBFG 11814) - hypothetical protein [Mycobacterium tuberculosis str. F11]	90.09	1389	1389
fig|6666666.28482.peg.5963	R014056	(gi:15841252) eccCa5 (MT1833) - FtsK/SpoIIIE family protein [Mycobacterium tuberculosis str. CDC1551]	90.09	1389	1389
fig|6666666.28482.peg.5963	R014046	(gi:15841252) eccCb5 (MT1833) - FtsK/SpoIIIE family protein [Mycobacterium tuberculosis str. CDC1551]	90.09	1389	1389
fig|6666666.28482.peg.5963	R014057	(gi:31792972) eccCa5 (Mb1812) - PROBABLE CONSERVED MEMBRANE PROTEIN [Mycobacterium bovis str. AF2122/97]	90.01	1389	1389
fig|6666666.28482.peg.5963	R014047	(gi:31792972) eccCb5 (Mb1812) - PROBABLE CONSERVED MEMBRANE PROTEIN [Mycobacterium bovis str. AF2122/97]	90.01	1389	1389
fig|6666666.28482.peg.5963	R014058	(gi:121637685) eccCa5 (BCG 1816) - hypothetical protein [Mycobacterium bovis str. BCG Pasteur 1173P2]	89.94	1389	1389
fig|6666666.28482.peg.5963	R014048	(gi:121637685) eccCb5 (BCG 1816) - hypothetical protein [Mycobacterium bovis str. BCG Pasteur 1173P2]	89.94	1389	1389
fig|6666666.28482.peg.5963	R019805	(gi:224990169) eccCb5 (JTY 1800) - hypothetical protein [Mycobacterium bovis str. BCG str. Tokyo 172]	89.94	1389	1389
fig|6666666.28482.peg.5963	R019801	(gi:183982672) eccCb5 (MMAR 2665) - FtsK/SpoIIIE family protein [Mycobacterium marinum str. M]	89.01	1389	1389
fig|6666666.28482.peg.5963	R014062	(gi:118618475) eccCa5 (MUL 3092) - conserved FtsK/SpoIIIE family protein [Mycobacterium ulcerans str. Agy99]	88.71	1389	1389
fig|6666666.28482.peg.5963	R014052	(gi:118618475) eccCb5 (MUL 3092) - conserved FtsK/SpoIIIE family protein [Mycobacterium ulcerans str. Agy99]	88.71	1389	1389
fig|6666666.28482.peg.5963	R014061	(gi:15827811) eccCa5 (ML1543) - possible SpoIIIE-family membrane protein [Mycobacterium leprae str. TN]	87.28	1337	1389
fig|6666666.28482.peg.5963	R014051	(gi:15827811) eccCb5 (ML1543) - possible SpoIIIE-family membrane protein [Mycobacterium leprae str. TN]	87.28	1337	1389
fig|6666666.28482.peg.5963	R019806	(gi:221230288) eccCb5 (MLBr 01543) - putative SpoIIIE-family membrane protein [Mycobacterium leprae str. Br4923]	87.28	1337	1389
fig|6666666.28482.peg.5963	R019807	(gi:333990533) eccCb5 (JDM601 1893) - FtsK/SpoIIIE family protein [Mycobacterium sp. str. JDM601]	80.6	1389	1389
fig|6666666.28482.peg.5963	R014044	(gi:15608922) eccCb5 (Rv1784) - hypothetical protein [Mycobacterium tuberculosis str. H37Rv]	90.57	930	1389

fig|6666666.28482.peg.5963	R019803	(gi:340626796) eccCb5 (MCAN 18021) - hypothetical protein [Mycobacterium canettii str. CIPT 140010059]	90.57	930	1389
fig|6666666.28482.peg.5963	R019802	(gi:339631838) eccCb5 (MAF 18060) - hypothetical protein [Mycobacterium africanum str. GM041182]	90.57	930	1389
fig|6666666.28482.peg.5964	R018629	(gi:118618474) cyp143A4 (MUL 3091) - cytochrome P450 143A4 Cyp143A4 [Mycobacterium ulcerans str. Agy99]	78.83	391	391
fig|6666666.28482.peg.5964	R019847	(gi:183982673) cyp143A4 (MMAR 2666) - cytochrome P450 143A4 Cyp143A4 [Mycobacterium marinum str. M]	78.57	391	391
fig|6666666.28482.peg.5964	R018628	(gi:118465559) MAV2931 - P450 heme-thiolate protein [Mycobacterium avium str. 104]	78.09	388	391
fig|6666666.28482.peg.5964	R018627	(gi:41407601) MAP1503c - hypothetical protein [Mycobacterium avium str. K-10 (subsp. paratuberculosis)]	77.25	378	391
fig|6666666.28482.peg.5964	R019849	(gi:340626797) cyp143 (MCAN 18031) - putative cytochrome P450 143 CYP143 [Mycobacterium canettii str. CIPT 140010059]	74.55	391	391
fig|6666666.28482.peg.5964	R019850	(gi:253799175) TBMG02212 - cytochrome P450 143 cyp143 [Mycobacterium tuberculosis str. KZN 1435]	74.55	391	391
fig|6666666.28482.peg.5964	R018622	(gi:15608923) cyp143 (Rv1785c) - cytochrome P450 143 [Mycobacterium tuberculosis str. H37Rv]	74.55	391	391
fig|6666666.28482.peg.5964	R018623	(gi:148661592) cyp143 (MRA 1799) - putative cytochrome p450 143 CYP143 [Mycobacterium tuberculosis str. H37Ra]	74.55	391	391
fig|6666666.28482.peg.5964	R018621	(gi:148823000) TBFG11815 - cytochrome P450 143 cyp143 [Mycobacterium tuberculosis str. F11]	74.55	391	391
fig|6666666.28482.peg.5964	R019851	(gi:224990170) cyp143 (JTY 1801) - putative cytochrome P450 143 [Mycobacterium bovis str. BCG str. Tokyo 172]	74.55	391	391
fig|6666666.28482.peg.5964	R018626	(gi:121637686) cyp143 (BCG 1817c) - putative cytochrome P450 143 cyp143 [Mycobacterium bovis str. BCG Pasteur 1173P2]	74.55	391	391
fig|6666666.28482.peg.5964	R018625	(gi:31792973) cyp143 (Mb1813c) - cytochrome P450 143 [Mycobacterium bovis str. AF2122/97]	74.55	391	391
fig|6666666.28482.peg.5964	R019848	(gi:339631839) cyp143 (MAF 18070) - putative cytochrome P450 143 CYP143 [Mycobacterium africanum str. GM041182]	74.55	391	391
fig|6666666.28482.peg.5964	R018624	(gi:15841253) MT1834 - P450 heme-thiolate protein [Mycobacterium tuberculosis str. CDC1551]	74.55	391	391

fig|6666666.28482.peg.5964	R018631	(gi:126436732) Mjls4160 - cytochrome P450 [Mycobacterium sp. str. JLS]	60.95	377	391
fig|6666666.28482.peg.5965	R018640	(gi:41407602) MAP1504 - hypothetical protein [Mycobacterium avium str. K-10 (subsp. paratuberculosis)]	89.13	46	46
fig|6666666.28482.peg.5965	R018642	(gi:118618473) MUL3090 - ferredoxin [Mycobacterium ulcerans str. Agy99]	86.96	46	46
fig|6666666.28482.peg.5965	R019852	(gi:183982674) MMAR2667 - ferredoxin [Mycobacterium marinum str. M]	86.96	46	46
fig|6666666.28482.peg.5965	R018643	(gi:126436671) Mjls4098 - hypothetical protein [Mycobacterium sp. str. JLS]	82.61	46	46
fig|6666666.28482.peg.5965	R019855	(gi:253799174) TBMG02211 - ferredoxin [Mycobacterium tuberculosis str. KZN 1435]	84.78	46	46
fig|6666666.28482.peg.5965	R018635	(gi:15608924) Rv1786 - ferredoxin [Mycobacterium tuberculosis str. H37Rv]	84.78	46	46
fig|6666666.28482.peg.5965	R018636	(gi:148661593) MRA1800 - putative ferredoxin [Mycobacterium tuberculosis str. H37Ra]	84.78	46	46
fig|6666666.28482.peg.5965	R018634	(gi:148823001) TBFG11816 - ferredoxin [Mycobacterium tuberculosis str. F11]	84.78	46	46
fig|6666666.28482.peg.5965	R018637	(gi:15841254) MT1835 - ferredoxin, putative [Mycobacterium tuberculosis str. CDC1551]	84.78	46	46
fig|6666666.28482.peg.5965	R019856	(gi:224990171) JTY1802 - putative ferredoxin [Mycobacterium bovis str. BCG str. Tokyo 172]	84.78	46	46
fig|6666666.28482.peg.5965	R018639	(gi:121637687) BCG1818 - putative ferredoxin [Mycobacterium bovis str. BCG Pasteur 1173P2]	84.78	46	46
fig|6666666.28482.peg.5965	R018638	(gi:31792974) Mb1814 - putative ferredoxin [Mycobacterium bovis str. AF2122/97]	84.78	46	46
fig|6666666.28482.peg.5965	R019853	(gi:339631840) MAF18080 - putative ferredoxin [Mycobacterium africanum str. GM041182]	84.78	46	46
fig|6666666.28482.peg.5965	R019854	(gi:340626798) MCAN18041 - putative ferredoxin [Mycobacterium canettii str. CIPT 140010059]	82.61	46	46
fig|6666666.28482.peg.5965	R018641	(gi:118465392) MAV2592 - hypothetical protein [Mycobacterium avium str. 104]	82.22	45	46

fig|6666666.28482.peg.5967	R019862	(gi:183982677) PE18 (MMAR 2670) - PE family protein, PE19 1 [Mycobacterium marinum str. M]	89.66	87	99
fig|6666666.28482.peg.5967	R018659	(gi:118618471) PE18 (MUL 3088) - PE family protein [Mycobacterium ulcerans str. Agy99]	88.51	87	99
fig|6666666.28482.peg.5967	R018680	(gi:41407605) PE19 (MAP1507) - hypothetical protein [Mycobacterium avium str. K-10 (subsp. paratuberculosis)]	87.36	87	99
fig|6666666.28482.peg.5967	R019865	(gi:253799172) PE18 (TBMG 02209) - PE family protein [Mycobacterium tuberculosis str. KZN 1435]	90.8	87	99
fig|6666666.28482.peg.5967	R018654	(gi:57116910) PE18 (Rv1788) - PE family protein [Mycobacterium tuberculosis str. H37Rv]	90.8	87	99
fig|6666666.28482.peg.5967	R018655	(gi:148661595) PE18 (MRA 1802) - PE family protein [Mycobacterium tuberculosis str. H37Ra]	90.8	87	99
fig|6666666.28482.peg.5967	R018653	(gi:148823003) PE18 (TBFG 11818) - PE family protein [Mycobacterium tuberculosis str. F11]	90.8	87	99
fig|6666666.28482.peg.5967	R018656	(gi:15841256) PE18 (MT1837) - PE family protein [Mycobacterium tuberculosis str. CDC1551]	90.8	87	99
fig|6666666.28482.peg.5967	R019864	(gi:340626800) PE18 (MCAN 18061) - PE family protein [Mycobacterium canettii str. CIPT 140010059]	90.8	87	99
fig|6666666.28482.peg.5967	R019866	(gi:224990173) PE18 (JTY 1804) - PE family protein [Mycobacterium bovis str. BCG str. Tokyo 172]	90.8	87	99
fig|6666666.28482.peg.5967	R018658	(gi:121637689) PE18 (BCG 1820) - PE family protein [Mycobacterium bovis str. BCG Pasteur 1173P2]	90.8	87	99
fig|6666666.28482.peg.5967	R018657	(gi:31792976) PE18 (Mb1816) - PE family protein [Mycobacterium bovis str. AF2122/97]	90.8	87	99
fig|6666666.28482.peg.5967	R019863	(gi:339631842) PE18 (MAF 18100) - PE family protein [Mycobacterium africanum str. GM041182]	90.8	87	99
fig|6666666.28482.peg.5967	R019877	(gi:183982680) PE19 (MMAR 2673) - PE family protein, PE19 [Mycobacterium marinum str. M]	88.51	87	99
fig|6666666.28482.peg.5967	R018675	(gi:57116913) PE19 (Rv1791) - PE family protein [Mycobacterium tuberculosis str. H37Rv]	86.21	87	99
fig|6666666.28482.peg.5967	R018676	(gi:148661598) PE19 (MRA 1805) - PE family protein [Mycobacterium tuberculosis str. H37Ra]	86.21	87	99

fig|6666666.28482.peg.5967	R019879	(gi:340626803) PE19 (MCAN 18091) - PE family protein [Mycobacterium canettii str. CIPT 140010059]	86.21	87	99
fig|6666666.28482.peg.5967	R019881	(gi:224990176) PE19 (JTY 1807) - PE family protein [Mycobacterium bovis str. BCG str. Tokyo 172]	86.21	87	99
fig|6666666.28482.peg.5967	R018679	(gi:121637692) PE19 (BCG 1823) - PE family protein [Mycobacterium bovis str. BCG Pasteur 1173P2]	86.21	87	99
fig|6666666.28482.peg.5967	R018678	(gi:31792979) PE19 (Mb1819) - PE family protein [Mycobacterium bovis str. AF2122/97]	86.21	87	99
fig|6666666.28482.peg.5967	R019878	(gi:339631845) PE19 (MAF 18130) - PE family protein [Mycobacterium africanum str. GM041182]	86.21	87	99
fig|6666666.28482.peg.5967	R019880	(gi:253799168) PE19 (TBMG 02205) - PE family protein [Mycobacterium tuberculosis str. KZN 1435]	86.21	87	99
fig|6666666.28482.peg.5967	R018674	(gi:148823006) PE19 (TBFG 11821) - PE family protein [Mycobacterium tuberculosis str. F11]	86.21	87	99
fig|6666666.28482.peg.5967	R018677	(gi:15841261) PE19 (MT1840) - PE family protein [Mycobacterium tuberculosis str. CDC1551]	86.21	87	99
fig|6666666.28482.peg.5967	R019867	(gi:333990534) PE18 (JDM601 1894) - PE-PGRS family protein [Mycobacterium sp. str. JDM601]	86.21	87	99
fig|6666666.28482.peg.6045	R006122	(gi:118465794) sigA/rpoV (MAV 3595) - sigma factor MysA [Mycobacterium avium str. 104]	87.82	507	507
fig|6666666.28482.peg.6045	R006121	(gi:41408918) sigA/rpoV (MAP2820) - RNA polymerase sigma factor [Mycobacterium avium str. K-10 (subsp. paratuberculosis)]	86.84	507	507
fig|6666666.28482.peg.6045	R006123	(gi:15827492) sigA/rpoV (ML1022) - RNA polymerase sigma factor [Mycobacterium leprae str. TN]	80.39	507	507
fig|6666666.28482.peg.6045	R019213	(gi:221229969) sigA/rpoV (MLBr 01022) - RNA polymerase sigma factor [Mycobacterium leprae str. Br4923]	80.39	507	507
fig|6666666.28482.peg.6045	R019211	(gi:253798215) sigA/rpoV (TBMG 01270) - RNA polymerase sigma factor [Mycobacterium tuberculosis str. KZN 1435]	78.27	507	507
fig|6666666.28482.peg.6045	R006116	(gi:15609840) sigA/rpoV (Rv2703) - RNA polymerase sigma factor [Mycobacterium tuberculosis str. H37Rv]	78.27	507	507
fig|6666666.28482.peg.6045	R006117	(gi:148662544) sigA/rpoV (MRA 2731) - RNA polymerase sigma factor [Mycobacterium tuberculosis str. H37Ra]	78.27	507	507

fig|6666666.28482.peg.6045	R013497	(gi:148823893) sigA/rpoV (TBFG 12718) - RNA polymerase sigma factor sigA [Mycobacterium tuberculosis str. F11]	78.27	507	507
fig|6666666.28482.peg.6045	R006118	(gi:15842241) sigA/rpoV (MT2777) - RNA polymerase sigma factor [Mycobacterium tuberculosis str. CDC1551]	78.27	507	507
fig|6666666.28482.peg.6045	R019210	(gi:340627704) sigA/rpoV (MCAN 27291) - RNA polymerase sigma factor SIGA [Mycobacterium canettii str. CIPT 140010059]	78.27	507	507
fig|6666666.28482.peg.6045	R019212	(gi:224991070) sigA/rpoV (JTY 2710) - RNA polymerase sigma factor [Mycobacterium bovis str. BCG str. Tokyo 172]	78.27	507	507
fig|6666666.28482.peg.6045	R006120	(gi:121638578) sigA/rpoV (BCG 2716) - RNA polymerase sigma factor sigA [Mycobacterium bovis str. BCG Pasteur 1173P2]	78.27	507	507
fig|6666666.28482.peg.6045	R006119	(gi:31793875) sigA/rpoV (Mb2722) - RNA polymerase sigma factor [Mycobacterium bovis str. AF2122/97]	78.27	507	507
fig|6666666.28482.peg.6045	R019209	(gi:339632715) sigA/rpoV (MAF 27070) - RNA polymerase sigma factor SIGA (SIGMA-A) [Mycobacterium africanum str. GM041182]	78.08	507	507
fig|6666666.28482.peg.6045	R006127	(gi:120403451) sigA/rpoV (Mvan 2461) - RNA polymerase, sigma 70 subunit, RpoD family [Mycobacterium vanbaalenii str. PYR-1]	83.19	448	507
fig|6666666.28482.peg.6045	R019207	(gi:183982025) sigA/rpoV (MMAR 2011) - RNA polymerase sigma factor [Mycobacterium marinum str. M]	97.97	345	507
fig|6666666.28482.peg.6045	R006124	(gi:118618666) sigA/rpoV (MUL 3341) - RNA polymerase sigma factor SigA [Mycobacterium ulcerans str. Agy99]	97.97	345	507
fig|6666666.28482.peg.6045	R006126	(gi:145224520) sigA/rpoV (Mflv 3939) - RNA polymerase, sigma 70 subunit, RpoD family [Mycobacterium gilvum str. PYR-GCK]	81.92	448	507
fig|6666666.28482.peg.6045	R006130	(gi:108799152) sigA/rpoV (Mmcs 2185) - RNA polymerase, sigma 28 subunit [Mycobacterium sp. str. MCS]	94.15	359	507
fig|6666666.28482.peg.6045	R006129	(gi:119868267) sigA/rpoV (Mkms 2231) - RNA polymerase, sigma 70 subunit, RpoD family [Mycobacterium sp. str. KMS]	94.15	359	507
fig|6666666.28482.peg.6045	R006128	(gi:126434760) sigA/rpoV (Mjls 2174) - RNA polymerase, sigma 70 subunit, RpoD family [Mycobacterium sp. str. JLS]	94.15	359	507
fig|6666666.28482.peg.6045	R019214	(gi:333991084) sigA/rpoV (JDM601 2444) - RNA polymerase sigma factor SigB [Mycobacterium sp. str. JDM601]	86.37	411	507
fig|6666666.28482.peg.6045	R006125	(gi:118469122) sigA/rpoV (MSMEG 2758) - sigma factor MysA [Mycobacterium smegmatis str. MC2 155]	95.69	348	507

fig|6666666.28482.peg.6045	R019208	(gi:169630092) sigA/rpoV (MAB 3009) - RNA polymerase sigma factor [Mycobacterium abscessus str. ATCC 19977]	97.63	338	507
fig|6666666.28482.peg.6068	R019031	(gi:253798207) ideR (TBMG 01263) - iron-dependent repressor and activator ideR [Mycobacterium tuberculosis str. KZN 1435]	93.51	231	231
fig|6666666.28482.peg.6068	R005833	(gi:15609848) ideR (Rv2711) - IRON-DEPENDENT REPRESSOR AND ACTIVATOR IDER [Mycobacterium tuberculosis str. H37Rv]	93.51	231	231
fig|6666666.28482.peg.6068	R005834	(gi:148662552) ideR (MRA 2739) - iron-dependent repressor IdeR [Mycobacterium tuberculosis str. H37Ra]	93.51	231	231
fig|6666666.28482.peg.6068	R013459	(gi:148823900) ideR (TBFG 12725) - iron-dependent repressor and activator ideR [Mycobacterium tuberculosis str. F11]	93.51	231	231
fig|6666666.28482.peg.6068	R005835	(gi:15842249) ideR (MT2784) - iron-dependent repressor IdeR [Mycobacterium tuberculosis str. CDC1551]	93.51	231	231
fig|6666666.28482.peg.6068	R019030	(gi:340627712) ideR (MCAN 27371) - iron-dependent repressor and activator IDER [Mycobacterium canettii str. CIPT 140010059]	93.51	231	231
fig|6666666.28482.peg.6068	R019032	(gi:224991078) ideR (JTY 2718) - iron-dependent repressor and activator [Mycobacterium bovis str. BCG str. Tokyo 172]	93.51	231	231
fig|6666666.28482.peg.6068	R005837	(gi:121638586) ideR (BCG 2724) - Iron-dependent repressor and activator ideR [Mycobacterium bovis str. BCG Pasteur 1173P2]	93.51	231	231
fig|6666666.28482.peg.6068	R005836	(gi:31793883) ideR (Mb2730) - IRON-DEPENDENT REPRESSOR AND ACTIVATOR IDER [Mycobacterium bovis str. AF2122/97]	93.51	231	231
fig|6666666.28482.peg.6068	R019029	(gi:339632723) ideR (MAF 27150) - iron-dependent repressor and activator IDER [Mycobacterium africanum str. GM041182]	93.51	231	231
fig|6666666.28482.peg.6068	R005839	(gi:118465346) ideR (MAV 3604) - iron-dependent repressor IdeR [Mycobacterium avium str. 104]	92.21	231	231
fig|6666666.28482.peg.6068	R019027	(gi:183982016) ideR (MMAR 2002) - iron-dependent repressor and activator IdeR [Mycobacterium marinum str. M]	92.64	231	231
fig|6666666.28482.peg.6068	R005841	(gi:118618674) ideR (MUL 3351) - iron-dependent repressor and activator IdeR [Mycobacterium ulcerans str. Agy99]	92.21	231	231
fig|6666666.28482.peg.6068	R005840	(gi:15827486) ideR (ML1013) - iron dependent repressor [Mycobacterium leprae str. TN]	89.61	231	231
fig|6666666.28482.peg.6068	R019033	(gi:221229963) ideR (MLBr 01013) - iron dependent repressor [Mycobacterium leprae str. Br4923]	89.61	231	231

fig|6666666.28482.peg.6068	R019034	(gi:333991093) ideR (JDM601 2453) - iron-dependent repressor and activator IdeR [Mycobacterium sp. str. JDM601]	86.58	231	231
fig|6666666.28482.peg.6068	R005847	(gi:108799143) ideR (Mmcs 2176) - iron (metal) dependent repressor, DtxR family [Mycobacterium sp. str. MCS]	86.15	231	231
fig|6666666.28482.peg.6068	R005846	(gi:119868258) ideR (Mkms 2222) - iron dependent repressor [Mycobacterium sp. str. KMS]	86.15	231	231
fig|6666666.28482.peg.6068	R005845	(gi:126434751) ideR (Mjls 2165) - iron dependent repressor [Mycobacterium sp. str. JLS]	86.15	231	231
fig|6666666.28482.peg.6068	R005843	(gi:145224530) ideR (Mflv 3949) - iron dependent repressor [Mycobacterium gilvum str. PYR-GCK]	85.71	231	231
fig|6666666.28482.peg.6068	R005842	(gi:118468273) ideR (MSMEG 2750) - iron-dependent repressor IdeR [Mycobacterium smegmatis str. MC2 155]	84.85	231	231
fig|6666666.28482.peg.6068	R005844	(gi:120403440) ideR (Mvan 2450) - iron dependent repressor [Mycobacterium vanbaalenii str. PYR-1]	85.28	231	231
fig|6666666.28482.peg.6068	R019028	(gi:169630112) ideR (MAB 3029) - Iron-dependent repressor IdeR [Mycobacterium abscessus str. ATCC 19977]	81.82	231	231
fig|6666666.28482.peg.6069	R006125	(gi:118469122) sigA/rpoV (MSMEG 2758) - sigma factor MysA [Mycobacterium smegmatis str. MC2 155]	62.86	311	319
fig|6666666.28482.peg.6069	R006128	(gi:126434760) sigA/rpoV (Mjls 2174) - RNA polymerase, sigma 70 subunit, RpoD family [Mycobacterium sp. str. JLS]	62.54	311	319
fig|6666666.28482.peg.6069	R006130	(gi:108799152) sigA/rpoV (Mmcs 2185) - RNA polymerase, sigma 28 subunit [Mycobacterium sp. str. MCS]	62.54	311	319
fig|6666666.28482.peg.6069	R006129	(gi:119868267) sigA/rpoV (Mkms 2231) - RNA polymerase, sigma 70 subunit, RpoD family [Mycobacterium sp. str. KMS]	62.54	311	319
fig|6666666.28482.peg.6069	R019208	(gi:169630092) sigA/rpoV (MAB 3009) - RNA polymerase sigma factor [Mycobacterium abscessus str. ATCC 19977]	62.54	311	319
fig|6666666.28482.peg.6069	R006122	(gi:118465794) sigA/rpoV (MAV 3595) - sigma factor MysA [Mycobacterium avium str. 104]	62.86	311	319
fig|6666666.28482.peg.6069	R006127	(gi:120403451) sigA/rpoV (Mvan 2461) - RNA polymerase, sigma 70 subunit, RpoD family [Mycobacterium vanbaalenii str. PYR-1]	62.86	311	319
fig|6666666.28482.peg.6069	R019207	(gi:183982025) sigA/rpoV (MMAR 2011) - RNA polymerase sigma factor [Mycobacterium marinum str. M]	62.86	311	319

fig|6666666.28482.peg.6069	R006126	(gi:145224520) sigA/rpoV (Mflv 3939) - RNA polymerase, sigma 70 subunit, RpoD family [Mycobacterium gilvum str. PYR-GCK]	62.54	311	319
fig|6666666.28482.peg.6069	R006124	(gi:118618666) sigA/rpoV (MUL 3341) - RNA polymerase sigma factor SigA [Mycobacterium ulcerans str. Agy99]	62.86	311	319
fig|6666666.28482.peg.6069	R006121	(gi:41408918) sigA/rpoV (MAP2820) - RNA polymerase sigma factor [Mycobacterium avium str. K-10 (subsp. paratuberculosis)]	62.54	311	319
fig|6666666.28482.peg.6069	R019214	(gi:333991084) sigA/rpoV (JDM601 2444) - RNA polymerase sigma factor SigB [Mycobacterium sp. str. JDM601]	62.22	311	319
